# Supplementary material for: Horseshoe crab genomes reveal the evolution of genes and microRNAs after three rounds of whole genome duplication
Source: Commun Biol. 2021 Jan 19;4:83. doi: 10.1038/s42003-020-01637-2 (PMC7815833; doi:10.1038/s42003-020-01637-2)
Supplement: Supplementary file 2 — Supplementary Information [file 42003_2020_1637_MOESM2_ESM.pdf]

Supplementary information for

Horseshoe crab genomes reveal the evolution of genes and microRNAs after three rounds of whole genome duplication

Wenyan Nong[1,^], Zhe Qu[1,^], Yiqian Li[1,^], Tom Barton-Owen[1,^], Annette Y.P. Wong[1,^], Ho Yin Yip[1], Hoi Ting Lee[1], Satya Narayana[1], Tobias Baril[2], Thomas Swale[3], Jianquan Cao[1], Ting Fung Chan[4], Hoi Shan Kwan[5], Ngai Sai Ming[4], Gianni Panagiotou[6,16], Pei-Yuan Qian[7], Jian-Wen Qiu[8], Kevin Y. Yip[9], Noraznawati Ismail[10], Siddhartha Pati[11, 17, 18], Akbar John[12], Stephen S. Tobe[13], William G. Bendena[14], Siu Gin Cheung[15], Alexander Hayward[2], Jerome H.L. Hui[1,*]

Supplementary Tables

Supplementary Table 1. *Carcinoscorpius rotundicauda* genome sequencing data information.

Platform	Read length(bp)	Library size	Reads	Bases(bp)	Coverage **
HiseqXten 150PE (Chromium WGS)	150	50 kb	799,339,678	120,700,291,378	70
HiSeq4000 100PE	100	200bp	207,051,562	20,705,156,200	12

****Assembled genome size = 1,725,596,044 bp**

Platform	Read length(bp)	Library size	Reads	Bases(bp)	Physical coverage
CHiCAGO 150PE	150	1-100kb	191,000,000	57,300,000,000	98.05
Hi-C 150PE		100-1000kb	232,000,000	69,600,000,000	1,472.08

Supplementary Table 2. *Tachypleus tridentatus* genome sequencing data information.

Platform	Read length(bp)	Library size	Reads	Bases(bp)	Coverage **
HiseqXten 150PE (Chromium WGS)	150	50 kb	2,653,443,020	400,669,896,020	233
HiSeq4000 100PE	100	200bp	195,252,954	19,525,295,400	11
HiSeq4000 151PE	151	550bp	1,112,540,752	167,993,653,552	98
PacBio RS II P6-C4	Average 6926	10kp	5,257,197	36,409,949,309	21

****Assembled genome size = 1,718,441,268 bp**

Platform	Read length(bp)	Library size	Reads	Bases(bp)	Physical coverage
CHiCAGO 150PE	150	1-100kb	405,000,000	121,500,000,000	75.25
Hi-C 150PE		100-1000kb	357,000,000	107,100,000,000	1,903.60

Supplementary Table 3. Longest scaffolds information of *C. rotundicauda* genome assembly.

#	Scaffold_length	Scaffold__ID	% of genome	sum% of genome
1	178168122	Sc28yqQ_31371	10.33%	10.33%
2	163974996	Sc28yqQ_31391	9.50%	19.83%
3	142590490	Sc28yqQ_31358	8.26%	28.09%
4	124683080	Sc28yqQ_29814	7.23%	35.32%
5	101415326	Sc28yqQ_31398	5.88%	41.19%
6	96212187	Sc28yqQ_10676	5.58%	46.77%
7	90264435	Sc28yqQ_4222	5.23%	52.00%
8	88715848	Sc28yqQ_31367	5.14%	57.14%
9	85949817	Sc28yqQ_29770	4.98%	62.12%
10	85410269	Sc28yqQ_6210	4.95%	67.07%
11	82098453	Sc28yqQ_31388	4.76%	71.83%
12	79870535	Sc28yqQ_31377	4.63%	76.46%
13	67249236	Sc28yqQ_31364	3.90%	80.36%
14	63649446	Sc28yqQ_9435	3.69%	84.04%
15	49882356	Sc28yqQ_3523	2.89%	86.93%
16	49417347	Sc28yqQ_31357	2.86%	89.80%

Supplementary Table 4. Longest scaffolds information of *T. tridentatus* genome assembly.

#	Scaffold_length	Scaffold__ID	% of genome	sum% of genome
1	161394085	Scaffold_44678	9.39%	9.39%
2	142096367	Scaffold_15738	8.27%	17.66%
3	135501044	Scaffold_11093	7.89%	25.55%
4	113327362	Scaffold_44553	6.59%	32.14%
5	109953960	Scaffold_44450	6.40%	38.54%
6	109941586	Scaffold_22712	6.40%	44.94%
7	109788719	Scaffold_20792	6.39%	51.33%
8	96326415	Scaffold_17368	5.61%	56.93%
9	92330412	Scaffold_15767	5.37%	62.30%
10	85635691	Scaffold_20684	4.98%	67.29%
11	80800453	Scaffold_44676	4.70%	71.99%
12	54053236	Scaffold_17076	3.15%	75.13%
13	52518514	Scaffold_38742	3.06%	78.19%
14	48775812	Scaffold_15334	2.84%	81.03%
15	47008499	Scaffold_44526	2.74%	83.76%
16	35123810	Scaffold_19944	2.04%	85.81%
17	32862481	Scaffold_44679	1.91%	87.72%
18	32581386	Scaffold_11607	1.90%	89.62%

Supplementary Table 5. Classification of repeat class/family in two horseshoe crab genomes.

Repeat Class/Family	*Carcinoscorpius roundiculata*			*Tachypleus tridentatus*		
	No. elements	Total length (bp)	Percentage (%)	No. elements	Total length (bp)	Percentage (%)
SINEs	345,070	57,677,200	3.34	364,667	61,354,604	3.57
LINEs	526,976	164,375,412	9.53	356,341	135,681,946	7.90
LTR elements	99,692	43,355,257	2.51	29,069	15,100,573	0.88
DNA elements	685,776	196,745,758	11.4	887,747	219,552,126	12.78
Unclassified	513,792	103,097,205	5.97	426,490	107,390,582	6.25
Total interspersed repeats	2,171,306	565,250,832	32.76	2,064,314	539,079,831	31.37
Other (Simple. Small RNA. Satellites. Low Complexity)	250,331	39,028,933	2.26	211,311	27,643,608	1.61
Total repeats	2,421,637	604,279,765	35.01	2,275,625	566,723,439	32.99

Supplementary Table 6. Gene copy numbers of homeobox genes in horseshoe crab genomes.

Classes	Families	*Cro*	*Ttr*	*Lpo*	Classes	Families	*Cro*	*Ttr*	*Lpo*
**ANTP**					**PRD**				
	Abox	**4**	**3**	**3**		Arx	**5**	**5**	**4**
	Barhl	**9**	**4**	**7**		CG11294	**1**	**1**	**1**
	Bari	**1**	**1**	**1**		Dmbx	**1**	**1**	**1**
	Barx	**1**	**1**	**1**		Drgx	**1**	**2**	**0**
	Bsx	**3**	**2**	**1**		Gsc	**2**	**2**	**2**
	Cdx	**5**	**5**	**5**		Hbn	**3**	**3**	**3**
	Dbx	**4**	**4**	**3**		Otp	**2**	**3**	**2**
	Dlx	**2**	**3**	**3**		Otx	**5**	**5**	**5**
	Emx	**8**	**8**	**9**		Pax3/7	**6**	**5**	**5**
	En	**7**	**13**	**7**		Pax4/6	**11**	**12**	**9**
	Evx	**2**	**2**	**2**		Phox	**3**	**3**	**2**
	Gbx	**7**	**6**	**6**		Pitx	**2**	**2**	**2**
	Gsx	**4**	**4**	**5**		Prop	**3**	**2**	**2**
	Hhex	**1**	**0**	**1**		Prrx	**1**	**3**	**3**
	Hlx	**3**	**3**	**3**		Rax	**2**	**3**	**3**
	Lab/Hox1	**6**	**5**	**5**		Repo	**1**	**2**	**3**
	Pb/Hox2	**5**	**4**	**4**		Shox	**3**	**3**	**4**
	Zen/Hox3	**5**	**3**	**3**		Uncx	**4**	**3**	**3**
	Dfd/Hox4	**7**	**5**	**5**		Vsx	**3**	**3**	**3**
	Scr/Hox5	**3**	**3**	**3**		Unknown	**1**	**1**	**1**
	Ftz/Hox6-8	**3**	**3**	**3**	**LIM**				
	Antp/Hox6-8	**3**	**3**	**3**		Isl	**2**	**2**	**2**
	Ubx/Hox6-8	**3**	**3**	**3**		Lhx1/5	**6**	**7**	**7**
	abdA/Hox6-8	**2**	**2**	**4**		Lhx2/9	**6**	**6**	**6**
	Hox6-8-like	**2**	**2**	**2**		Lhx3/4	**5**	**5**	**5**
	abdB/Hox9-13	**4**	**3**	**3**		Lhx6/8	**3**	**3**	**3**
	Lbx	**5**	**4**	**4**		Lmx	**2**	**2**	**2**
	Meox	**1**	**1**	**1**	**SINE**				
	Mnx	**3**	**3**	**1**		Six1/2	**6**	**6**	**6**
	Msx	**4**	**4**	**7**		Six3/6	**5**	**6**	**6**
	MsxL	**1**	**1**	**1**		Six4/5	**4**	**4**	**4**
	Nedx	**4**	**3**	**3**	**TALE**				
	Nk1	**5**	**4**	**4**		Irx	**12**	**11**	**11**
	Nk2.1	**4**	**4**	**4**		Meis	**3**	**3**	**4**
	Nk2.2	**4**	**4**	**4**		Mkx	**1**	**1**	**1**
	Nk3	**2**	**2**	**2**		Pbx	**5**	**5**	**6**
	Nk4	**2**	**1**	**1**		Pknox	**2**	**2**	**2**
	Nk5/Hmx	**6**	**5**	**5**		Tgif	**6**	**7**	**7**
	Nk6	**3**	**3**	**2**		Unknown	**1**	**1**	**1**
	Nk7	**2**	**3**	**2**	**ZF**				
	Noto	**3**	**3**	**2**		Zeb-like	**6**	**4**	**5**
	Ro	**3**	**2**	**2**		Zfhx	**5**	**4**	**4**
	Tlx	**3**	**3**	**3**	**CUT**				
	Unknown	**1**	**1**	**1**		Cmp	**1**	**1**	**2**
**POU**						Cux	**8**	**8**	**8**
	Pou2	**8**	**8**	**8**		Onecut	**8**	**8**	**8**
	Pou3	**8**	**8**	**7**					
	Pou4	**2**	**2**	**2**	**PROS**	Prox	**5**	**5**	**5**
	Pou6	**3**	**3**	**3**	**Cer**	Cers	**3**	**3**	**3**

Supplementary Table 7. *C. rotundicauda* transcriptome sequencing data information.

Type	Stages	Platform	Reads	Bases	Accession
mRNA	Blood	NovaSeq 6000	38,863,153	5,829,449,781	SAMN13738014
	Brain		31,575,827	4,736,353,472	SAMN13738015
	chelicerate		39,549,495	5,932,400,015	SAMN13738016
	Heart		27,783,626	4,167,527,190	SAMN13738017
	Leg1		35,135,528	5,270,307,850	SAMN13738018
	Leg5		40,202,979	6,030,422,836	SAMN13738019
	Tail		33,116,985	4,967,527,398	SAMN13738020
Small RNA	Brain	NovaSeq 50SE	20,410,408	1,020,520,400	SAMN13871197
	Blood		19,564,687	978,234,350	SAMN13871198
	Chelicerae		19,393,640	969,682,000	SAMN13871199
	Heart		20,309,098	1,015,454,900	SAMN13871200
	1st_pair_of_leg		19,043,622	952,181,100	SAMN13871201
	5th_pair_of_leg		29,345,855	1,467,292,750	SAMN13871202
	Telson		22,375,032	1,118,751,600	SAMN13871203

miRTrace outputs: http://137.189.43.6/miRTrace_output/cro/mirtrace-report.html

Supplementary Table 8. *T. tridentatus* transcriptome sequencing data information

Type	Stages	Platform	Reads	Bases	Accession
mRNA	fbl.blood.	NovaSeq 6000	37,977,195	5,696,301,567	SAMN13738021
	fbr.brain.		34,292,198	5,143,570,785	SAMN13738022
	fc.chelicerate.		32,805,893	4,920,650,285	SAMN13738023
	fh.heart.		35,230,816	5,284,362,030	SAMN13738024
	fl1.leg1.		36,576,210	5,486,164,882	SAMN13738025
	fl5.leg5.		33,927,286	5,088,844,698	SAMN13738026
	ft.telson.		39,699,452	5,954,617,801	SAMN13738027
	HSC.1st.Juv.		21,193,857	2,118,740,674	SAMN13738028
	HSC.2nd.Juv.		20,816,258	2,080,987,978	SAMN13738029
	HSC.3rd.Juv.		20,288,354	2,028,223,006	SAMN13738030
Small RNA	Adult_1st_pair_of_leg	HiSeq 2500	25,650,608	563,162,745	SAMN13871219
	Adult_5th_pair_of_leg		24,554,405	539,154,086	SAMN13871220
	Adult_blood		27,669,258	615,864,084	SAMN13871208
	Adult_brain		26,993,521	617,921,430	SAMN13871210
	Adult_chelicerae		31,948,259	703,790,441	SAMN13871211
	Adult_heart		26,082,982	575,132,790	SAMN13871217
	Adult_telson		26,857,159	605,349,870	SAMN13871223
	Juvenile_1st_pair_of_leg		14,965,599	332,545,166	SAMN13871221
	Juvenile_5th_pair_of_leg		17,705,657	387,406,260	SAMN13871222
	Juvenile_blood		17,054,020	405,397,024	SAMN13871209
	Juvenile_chelicerae		14,910,286	327,534,454	SAMN13871212
	Juvenile_heart		13,563,640	310,098,352	SAMN13871218
	Juvenile_telson		12,047,257	265,525,861	SAMN13871224
	1st_instar	NovaSeq 50SE	27,947,739	611,436,838	SAMN13871204
	2nd_instar		24,780,645	557,062,135	SAMN13871205
	3rd_instar		25,705,620	558,368,223	SAMN13871206
	Adult_blood		25,362,968	559,120,297	SAMN13871207
	Adult_heart		20,381,064	452,114,559	SAMN13871216
	Egg_1		20,641,958	494,478,133	SAMN13871213
	Egg_2		22,755,433	549,370,934	SAMN13871214
	Gonad		24,754,354	578,404,342	SAMN13871215

miRTrace outputs: http://137.189.43.6/miRTrace_output/ttr/mirtrace-report.html

Supplementary Table 9. Information of *C. rotundicauda* samples.

Batch	Sample ID	Date of Collection	Location	Juvenile/Adult Male/Female
HK	HK-LK1	October 2018	Luk Keung, Hong Kong	Juvenile
	HK-LK2	October 2018	Luk Keung, Hong Kong	Juvenile
	HK-LK3	October 2018	Luk Keung, Hong Kong	Juvenile
	HK-ST1	September 2018	San Tau, Hong Kong	Juvenile
	HK-STK1	October 2018	Sha Tau Kok, Hong Kong	Juvenile
	HK-STK2	October 2018	Sha Tau Kok, Hong Kong	Juvenile
	HK-STK3	October 2018	Sha Tau Kok, Hong Kong	Juvenile
	HK-TCB1	September 2018	Tung Chung Bay, Hong Kong	Juvenile
	HK-TCB2	September 2018	Tung Chung Bay, Hong Kong	Juvenile
	HK-TCB3	September 2018	Tung Chung Bay, Hong Kong	Juvenile
I	I-1	March 2018	Kharibil, Bichitrapur, Odisha, India	Adult Female
	I-2	February 2018	Kharibil, Bichitrapur, Odisha, India	Adult Male
	I-3	February 2018	Kharibil, Bichitrapur, Odisha, India	Adult Male
	I-4	February 2018	Kharibil, Bichitrapur, Odisha, India	Adult Male
	I-5	February 2018	Kharibil, Bichitrapur, Odisha, India	Adult Male
	I-6	February 2018	Kharibil, Bichitrapur, Odisha, India	Juvenile
M-1	M-SB1	February 2018	Tawau, Sabah, Malaysia	Adult Male
	M-SB2	February 2018	Tawau, Sabah, Malaysia	Adult Male
T-1	T-B1	August 2017	Phetchaburi, Bangkok, Thailand	Adult Female
	T-B2	August 2017	Phetchaburi, Bangkok, Thailand	Adult Male
	T-B3	August 2017	Phetchaburi, Bangkok, Thailand	Adult Male
	T-B4	August 2017	Phetchaburi, Bangkok, Thailand	Adult Male
	T-B5	August 2017	Phetchaburi, Bangkok, Thailand	Adult Male
	T-B6	August 2017	Phetchaburi, Bangkok, Thailand	Adult Female
T-2	T-PB1	January 2018	Pattani bay, Thailand	Adult Male
	T-PB2	January 2018	Pattani bay, Thailand	Adult Male
	T-PB3	January 2018	Pattani bay, Thailand	Adult Male
	T-PB4	January 2018	Pattani bay, Thailand	Adult Male
	T-PB5	January 2018	Pattani bay, Thailand	Adult Male
	T-PB6	January 2018	Pattani bay, Thailand	Adult Female
	T-PB7	January 2018	Pattani bay, Thailand	Adult Female
	T-PB8	January 2018	Pattani bay, Thailand	Adult Female
	T-PB9	January 2018	Pattani bay, Thailand	Adult Female
	T-PB10	January 2018	Pattani bay, Thailand	Adult Female

Supplementary Table 10. Information of *T. tridentatus* samples.

Batch	Sample ID	Date of Collection	Location	Juvenile/Adult Male/Female
HK	HK-HHW1	September 2018	Hau Hok Wan, Hong Kong	Juvenile
	HK-HHW2	September 2018	Hau Hok Wan, Hong Kong	Juvenile
	HK-HHW3	September 2018	Hau Hok Wan, Hong Kong	Juvenile
	HK-HPN1	August 2018	Ha Pak Nai, Hong Kong	Juvenile
	HK-HPN2	August 2018	Ha Pak Nai, Hong Kong	Juvenile
	HK-HPN3	August 2018	Ha Pak Nai, Hong Kong	Juvenile
	HK-PN1	August 2018	Pak Nai, Hong Kong	Juvenile
	HK-PN2	August 2018	Pak Nai, Hong Kong	Juvenile
	HK-PN3	August 2018	Pak Nai, Hong Kong	Juvenile
	HK-ST1	September 2018	San Tau, Hong Kong	Juvenile
	HK-ST2	September 2018	San Tau, Hong Kong	Juvenile
	HK-ST3	September 2018	San Tau, Hong Kong	Juvenile
	HK-ST4	September 2018	San Tau, Hong Kong	Juvenile
	HK-SH1	October 2018	Shui Hau, Hong Kong	Juvenile
	HK-SH2	October 2018	Shui Hau, Hong Kong	Juvenile
	HK-SH3	October 2018	Shui Hau, Hong Kong	Juvenile
	HK-TCB	September 2018	Tung Chung Bay, Hong Kong	Juvenile
	HK-YO1	October 2018	Yi O, Hong Kong	Juvenile
	HK-YO2	October 2018	Yi O, Hong Kong	Juvenile
	HK-YO3	October 2018	Yi O, Hong Kong	Juvenile
M-1	M-SB1	February 2018	Tawau, Sabah, Malaysia	Adult Male
	M-SB2	February 2018	Tawau, Sabah, Malaysia	Adult Male
	M-SB3	February 2018	Tawau, Sabah, Malaysia	Adult Male
	M-SB4	March 2018	Tawau, Sabah, Malaysia	Adult Female
	M-SB5	March 2018	Tawau, Sabah, Malaysia	Adult Female
	M-SB6	March 2018	Tawau, Sabah, Malaysia	Adult Female
	M-SB7	March 2018	Tawau, Sabah, Malaysia	Adult Female
	M-SB8	March 2018	Tawau, Sabah, Malaysia	Adult Female
	M-SB9	March 2018	Tawau, Sabah, Malaysia	Adult Female
	M-SB10	March 2018	Tawau, Sabah, Malaysia	Adult Female
	M-SB11	March 2018	Tawau, Sabah, Malaysia	Adult Female
	M-SB12	March 2018	Tawau, Sabah, Malaysia	Adult Female
	M-SB13	March 2018	Tawau, Sabah, Malaysia	Adult Female
M-2	M-K1	November 2016	Gading Gading, Kunak, Malaysia	Adult Male
	M-K2	November 2016	Gading Gading, Kunak, Malaysia	Adult Male
	M-K3	November 2016	Gading Gading, Kunak, Malaysia	Adult Male
	M-K4	November 2016	Gading Gading, Kunak, Malaysia	Adult Male
	M-K5	November 2016	Gading Gading, Kunak, Malaysia	Adult Male
	M-K6	November 2016	Gading Gading, Kunak, Malaysia	Adult Male
	M-K7	November 2016	Gading Gading, Kunak, Malaysia	Adult Male
	M-S1	November 2016	Pulau Bum Bum, Semporna, Malaysia	Adult Male
	M-S2	November 2016	Pulau Bum Bum, Semporna, Malaysia	Adult Male
	M-S3	November 2016	Pulau Bum Bum, Semporna, Malaysia	Adult Male
	M-S4	November 2016	Pulau Bum Bum, Semporna, Malaysia	Adult Male
	M-S5	November 2016	Pulau Bum Bum, Semporna, Malaysia	Adult Male
	M-S6	November 2016	Pulau Bum Bum, Semporna, Malaysia	Adult Male
	M-S7	November 2016	Pulau Bum Bum, Semporna, Malaysia	Adult Male

Supplementary Figures

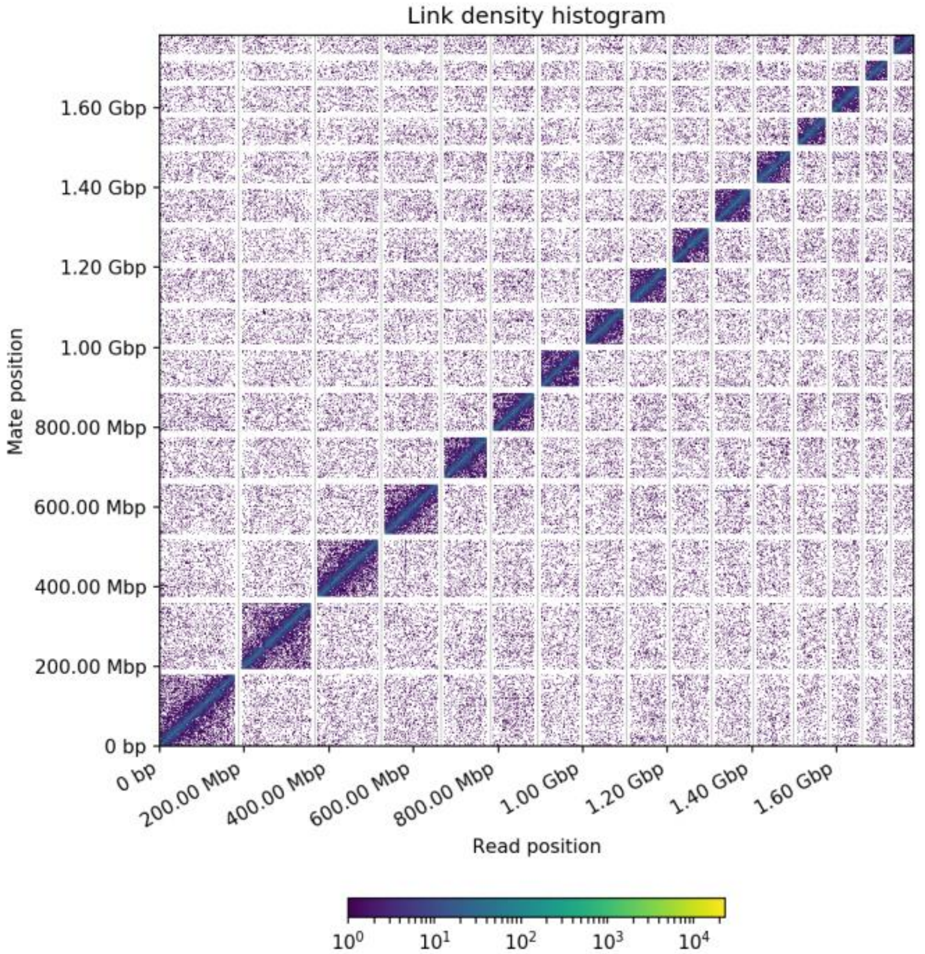

Supplementary Figure 1. ***C. rotundicauda*** **Hi-C information.** The x- and y- axes give the mapping positions of the first and second read in the read pair respectively, grouped into bins. The colour of each square gives the number of read pairs within that bin. White vertical and black horizontal lines have been added to show the borders between scaffolds. Scaffolds less than 1 Mb are excluded.

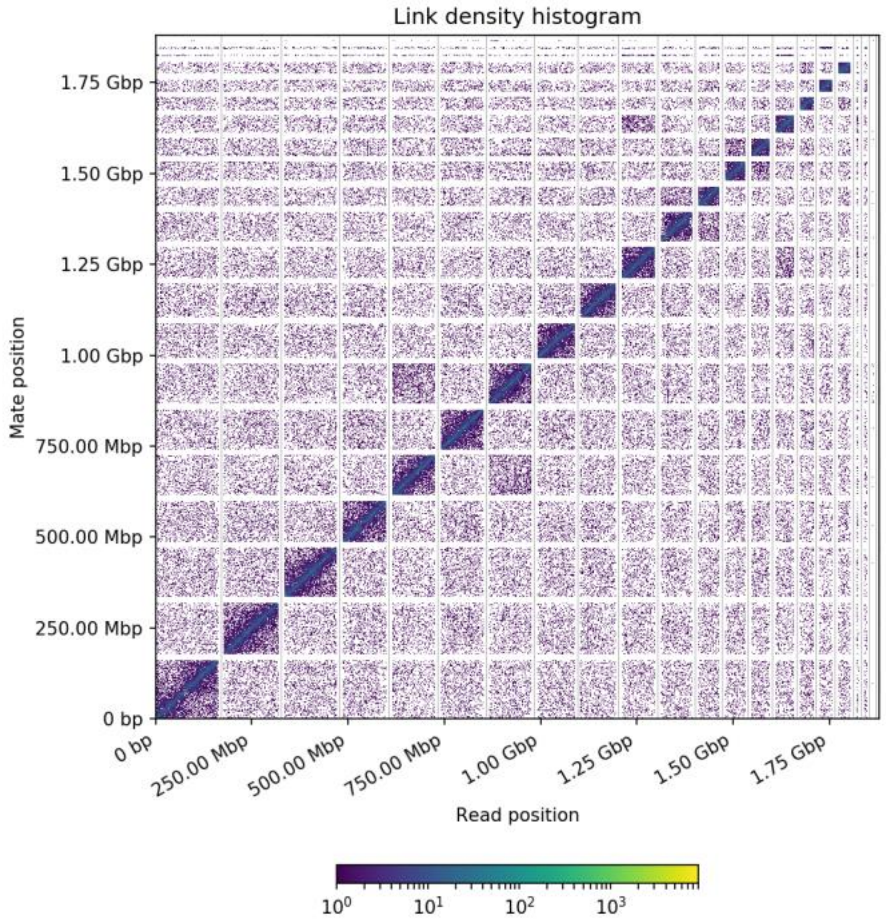

Supplementary Figure 2. *T. tridentatus* Hi-C information. The x- and y- axes give the mapping positions of the first and second read in the read pair respectively, grouped into bins. The colour of each square gives the number of read pairs within that bin. White vertical and black horizontal lines have been added to show the borders between scaffolds. Scaffolds less than 1 Mb are excluded.

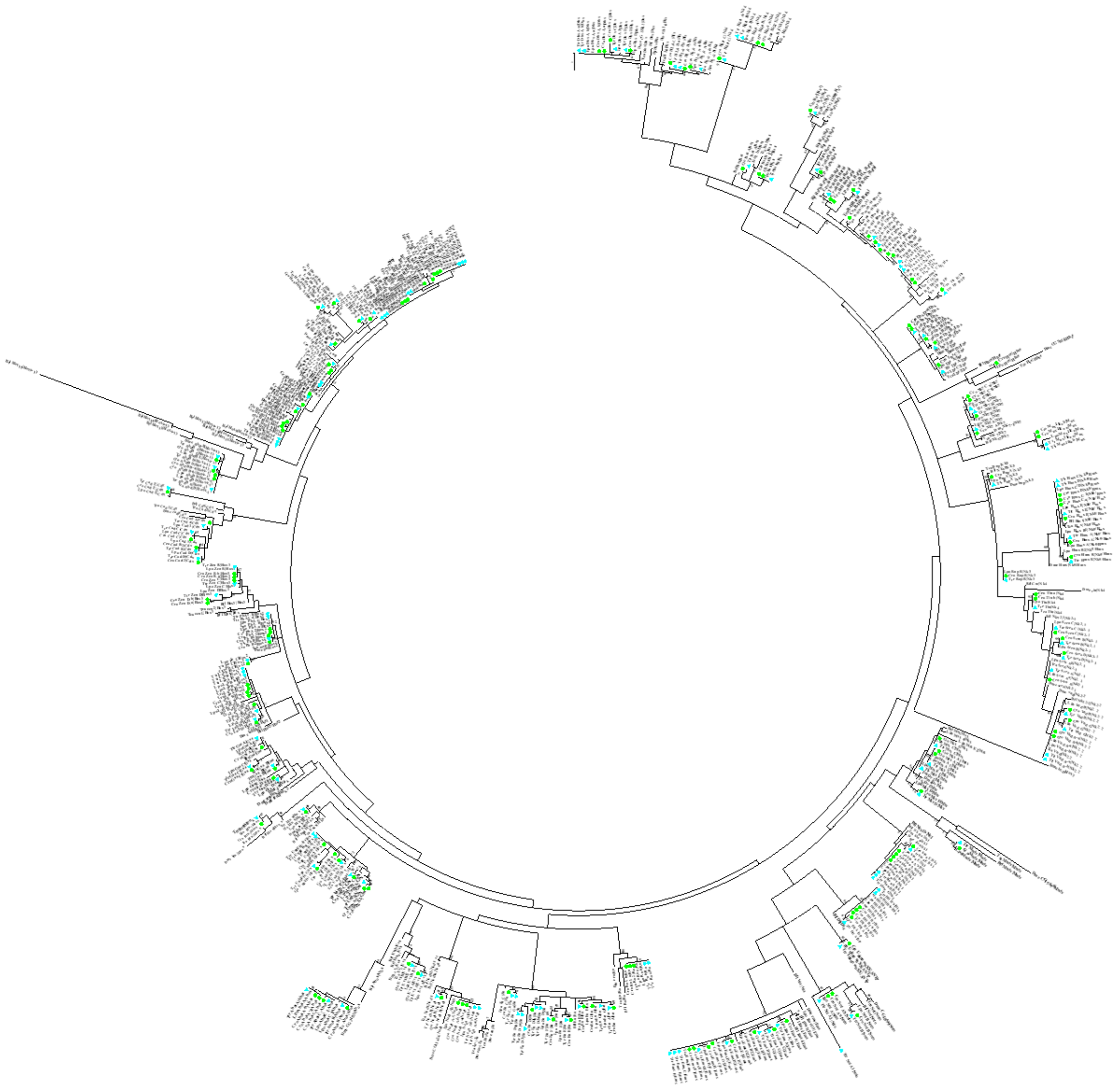

Supplementary Figure 3. Maximum-likelihood tree of ANTP-class homeobox genes (LG+G model).

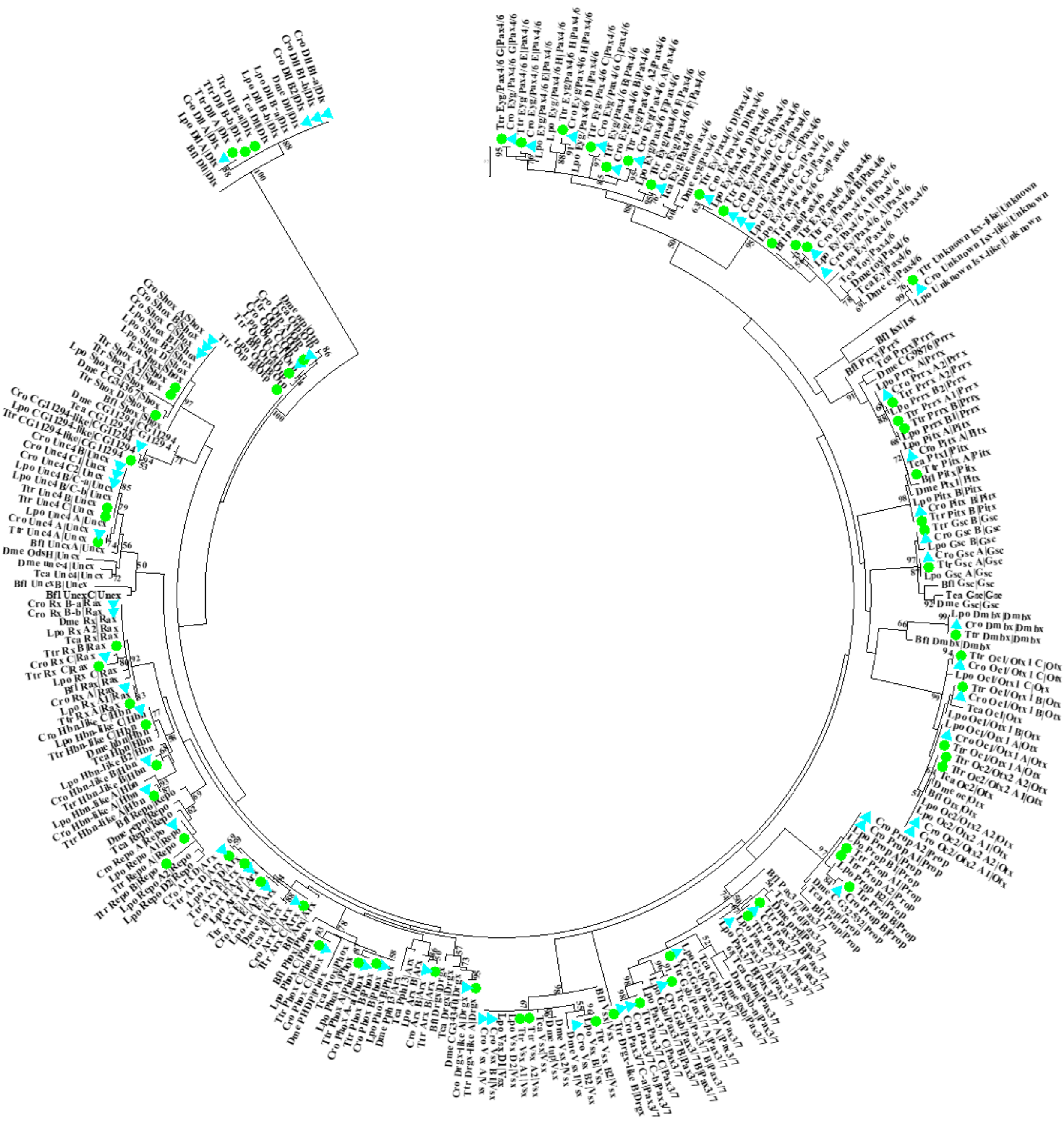

Supplementary Figure 4. Maximum-likelihood tree of PRD-class homeobox genes (LG+G model).

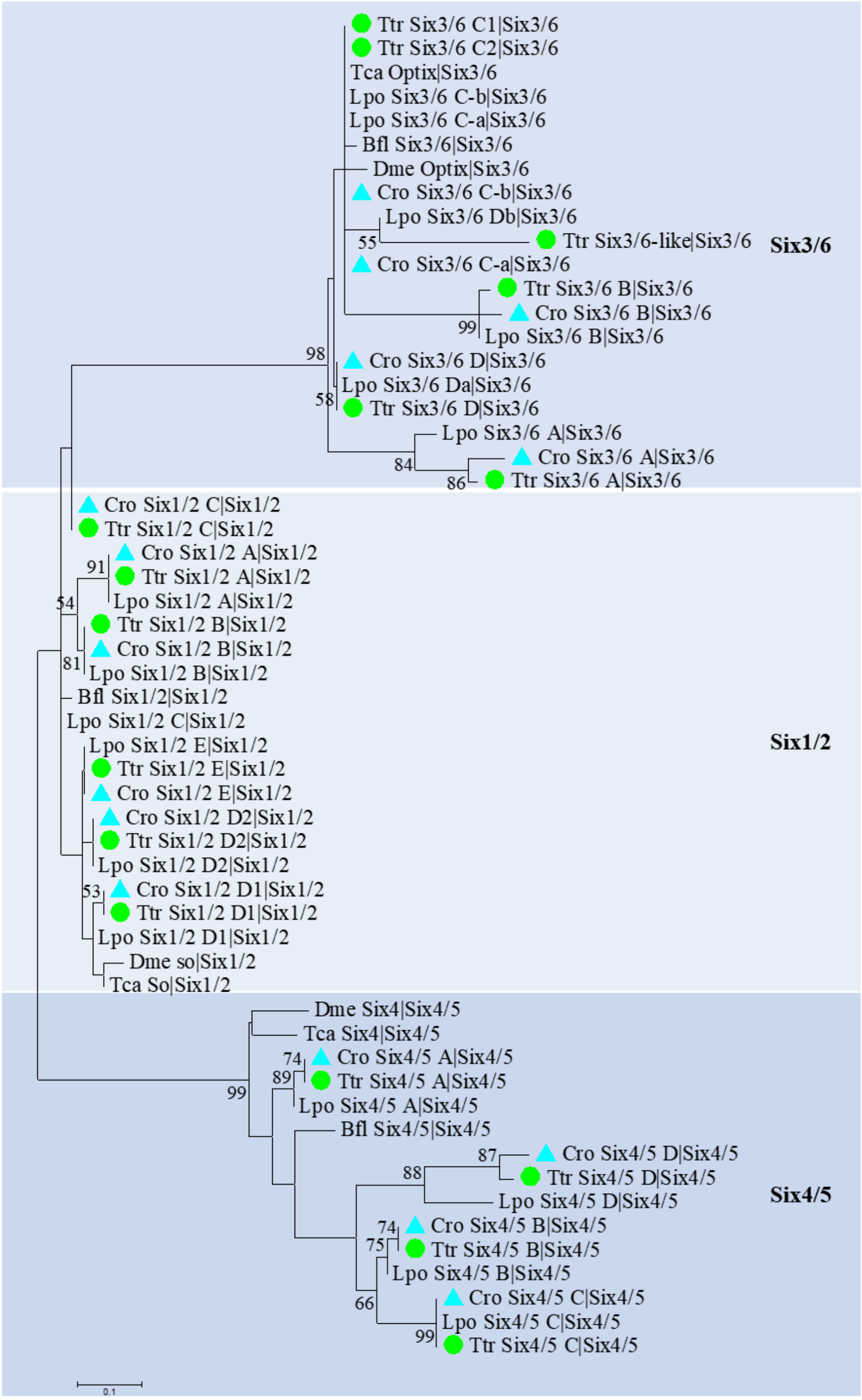

Supplementary Figure 5. Maximum-likelihood tree of SINE-class homeobox genes (JTT+G model).

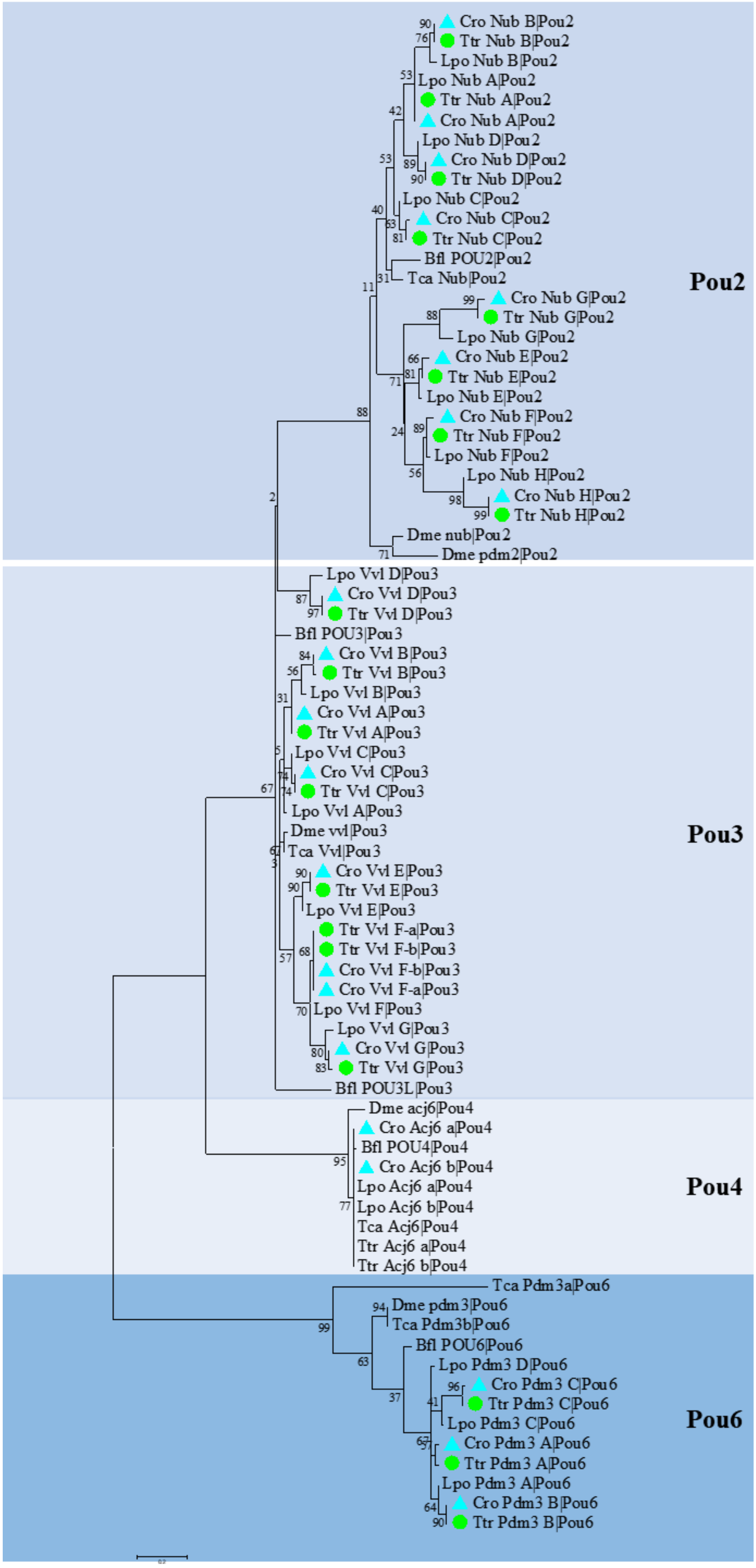

Supplementary Figure 6. Maximum-likelihood tree of POU-class homeobox genes (LG+G model).

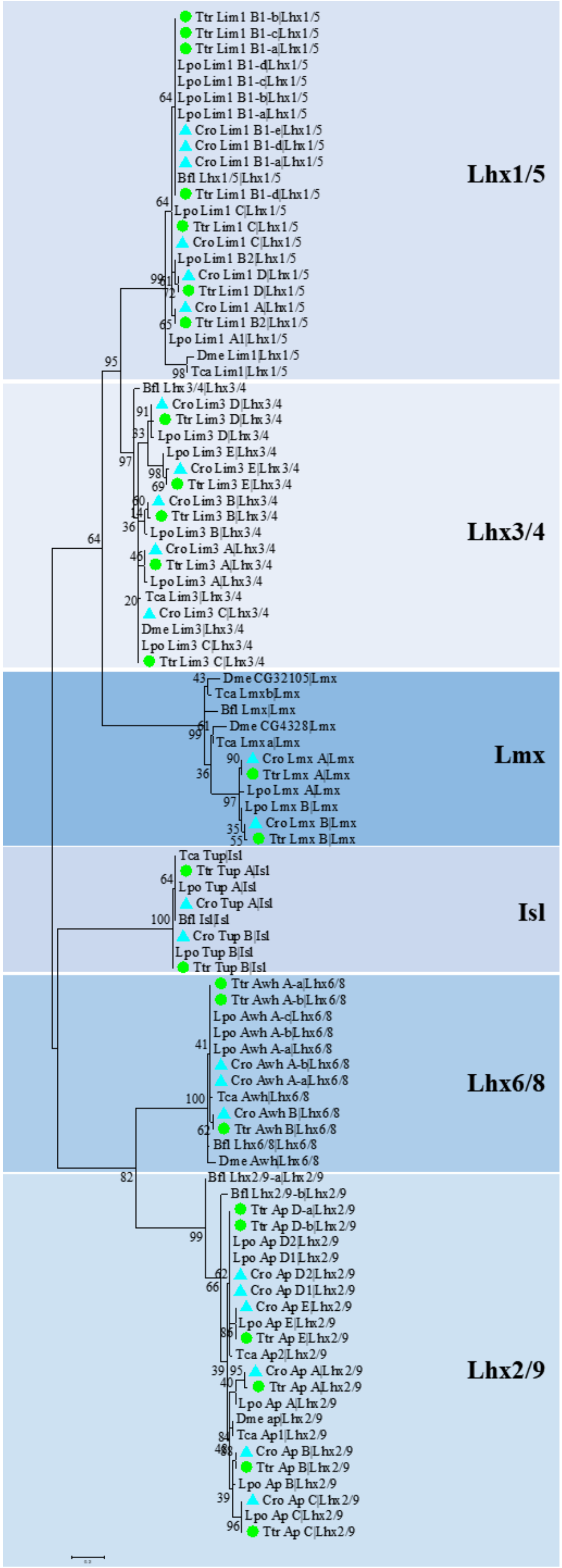

Supplementary Figure 7. Maximum-likelihood tree of LIM-class homeobox genes (LG+G+I model).

a

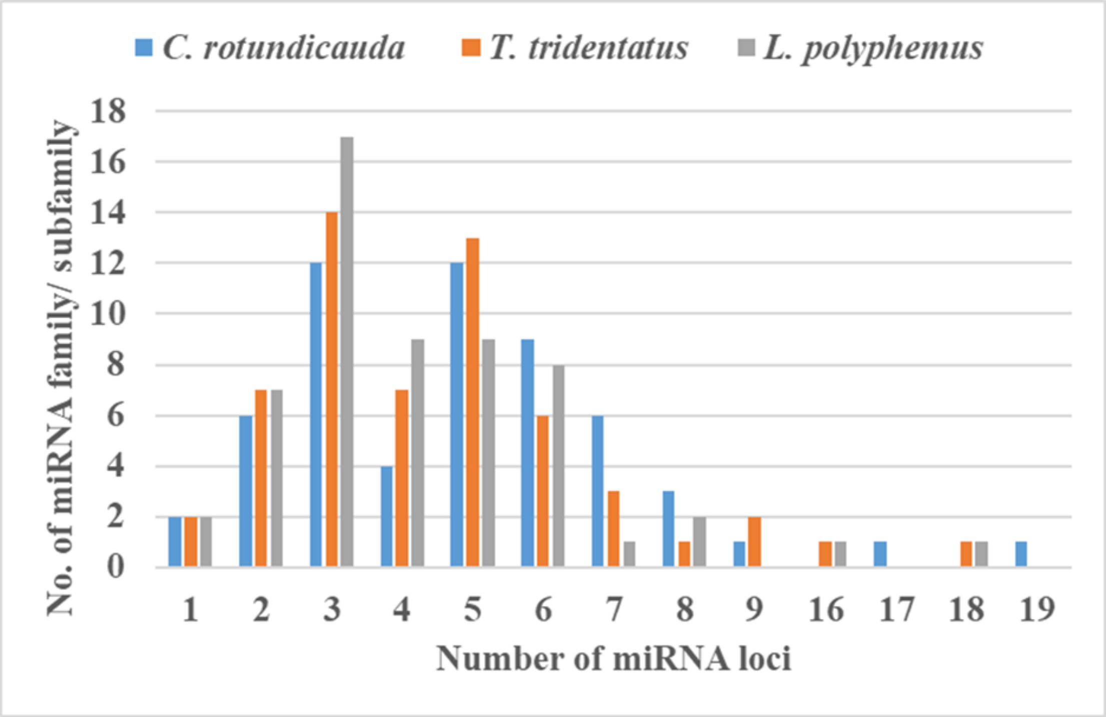

b

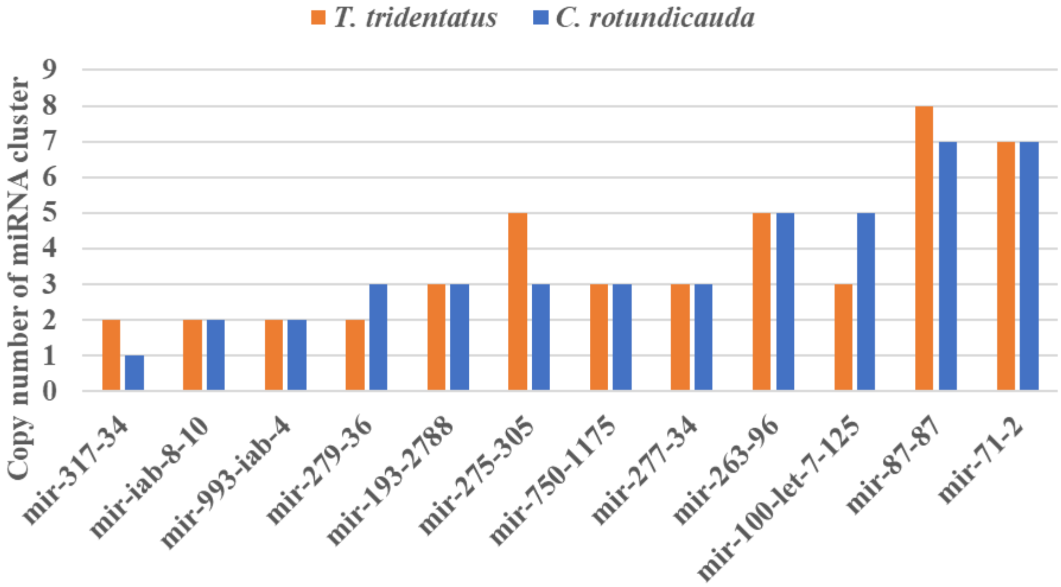

Supplementary Figure 8. Copy number distribution of conserved microRNAs (a) and microRNA clusters (b) in horseshoe crab genomes.

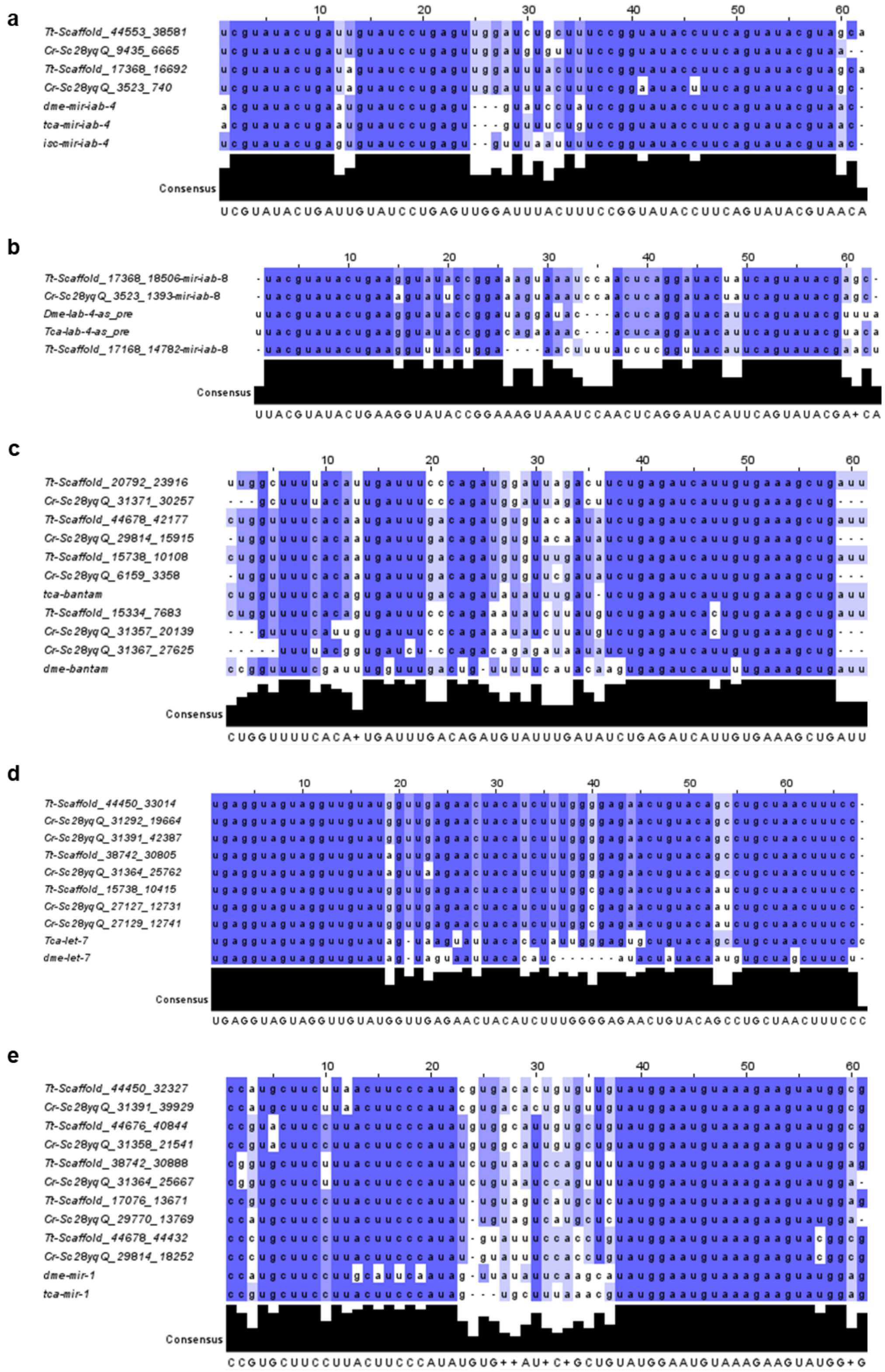

Supplementary Figure 9. Sequence alignments of mir-iab-4, bantam, let-7 and mir-1 families assigned for the two horseshoe crab genomes. Sequence alignment was performed using MAFFT (Katoh and Standley 2013) and displayed by Jalview (Waterhouse et al 2009). a) mir-iab-4; b) mir-iab-8; c) bantam; d) let-7; e) mir-1.

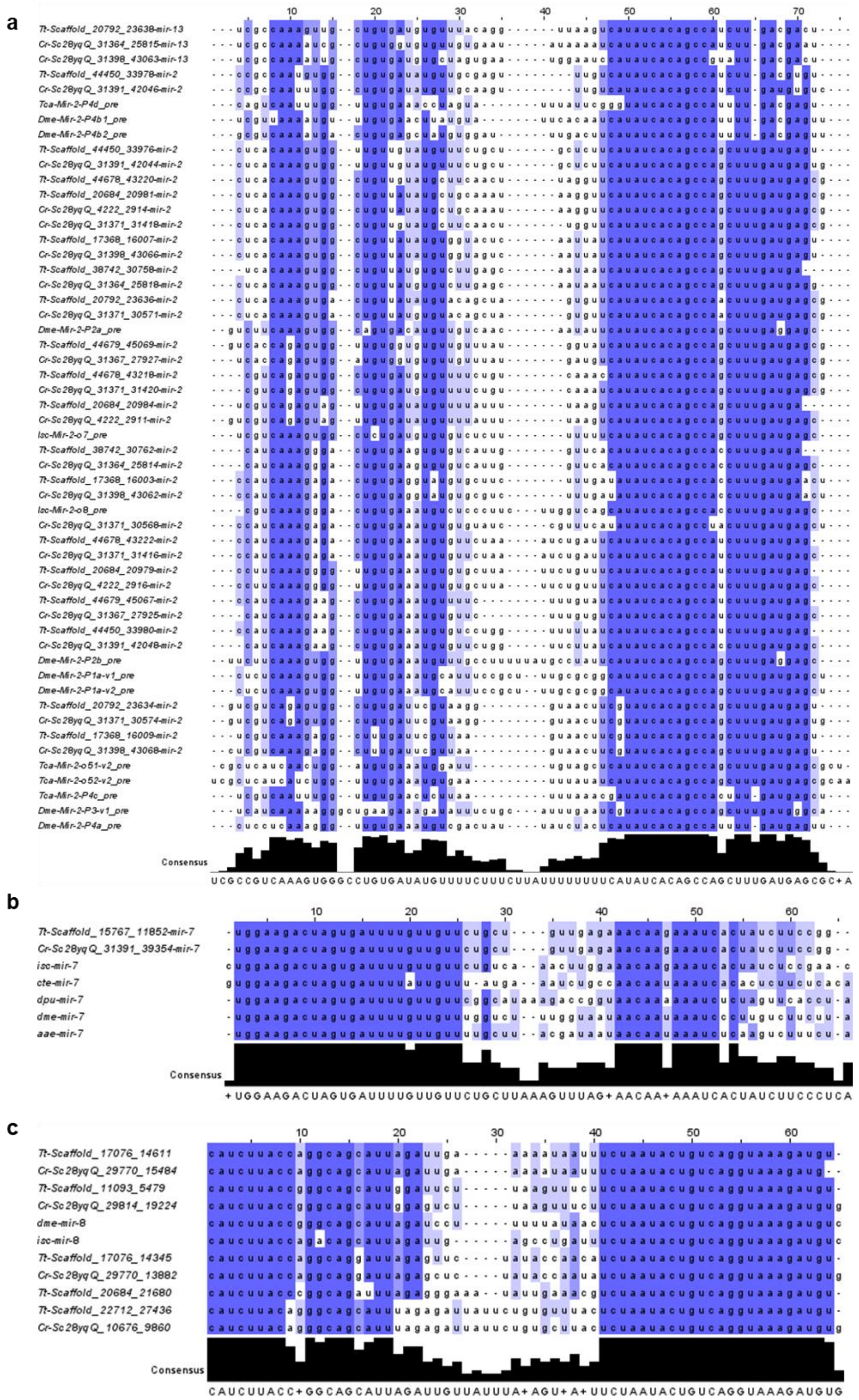

Supplementary Figure 10. Sequence alignments of mir-2, mir-7 and mir-8 families assigned for the two horseshoe crab genomes. Sequence alignment was performed using MAFFT (Katoh and Standley 2013) and displayed by Jalview (Waterhouse et al 2009). a) mir-2 and mir-13; b) mir-7; c) mir-8.

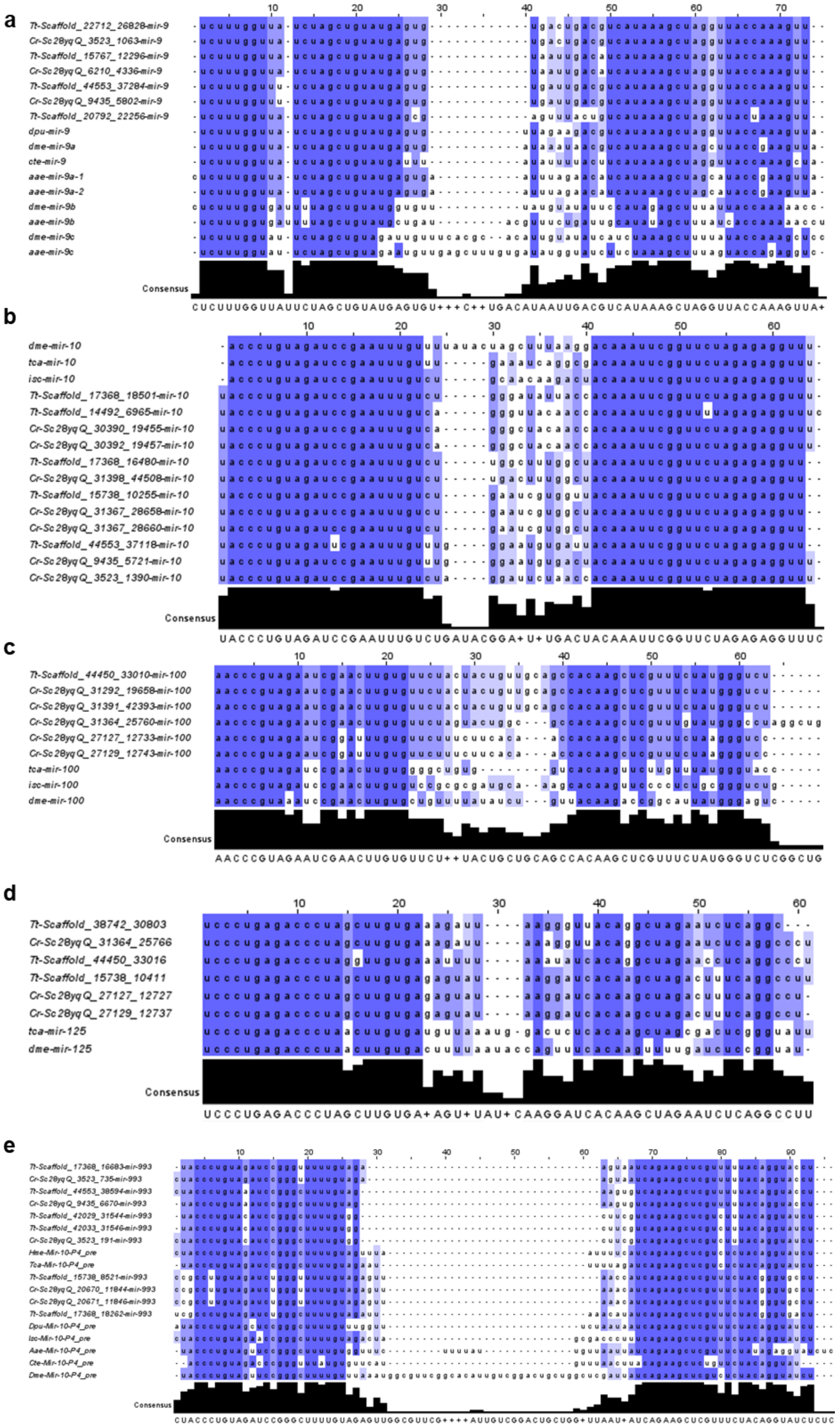

Supplementary Figure 11. Sequence alignments of mir-9, mir-10, mir-100, mir-125 and mir-993 assigned for the two horseshoe crab genomes. Sequence alignment was performed using MAFFT (Katoh and Standley 2013) and displayed by Jalview (Waterhouse et al 2009). a) mir-9; b) mir-10; c) mir-100; d) mir-125 and e) mir-993.

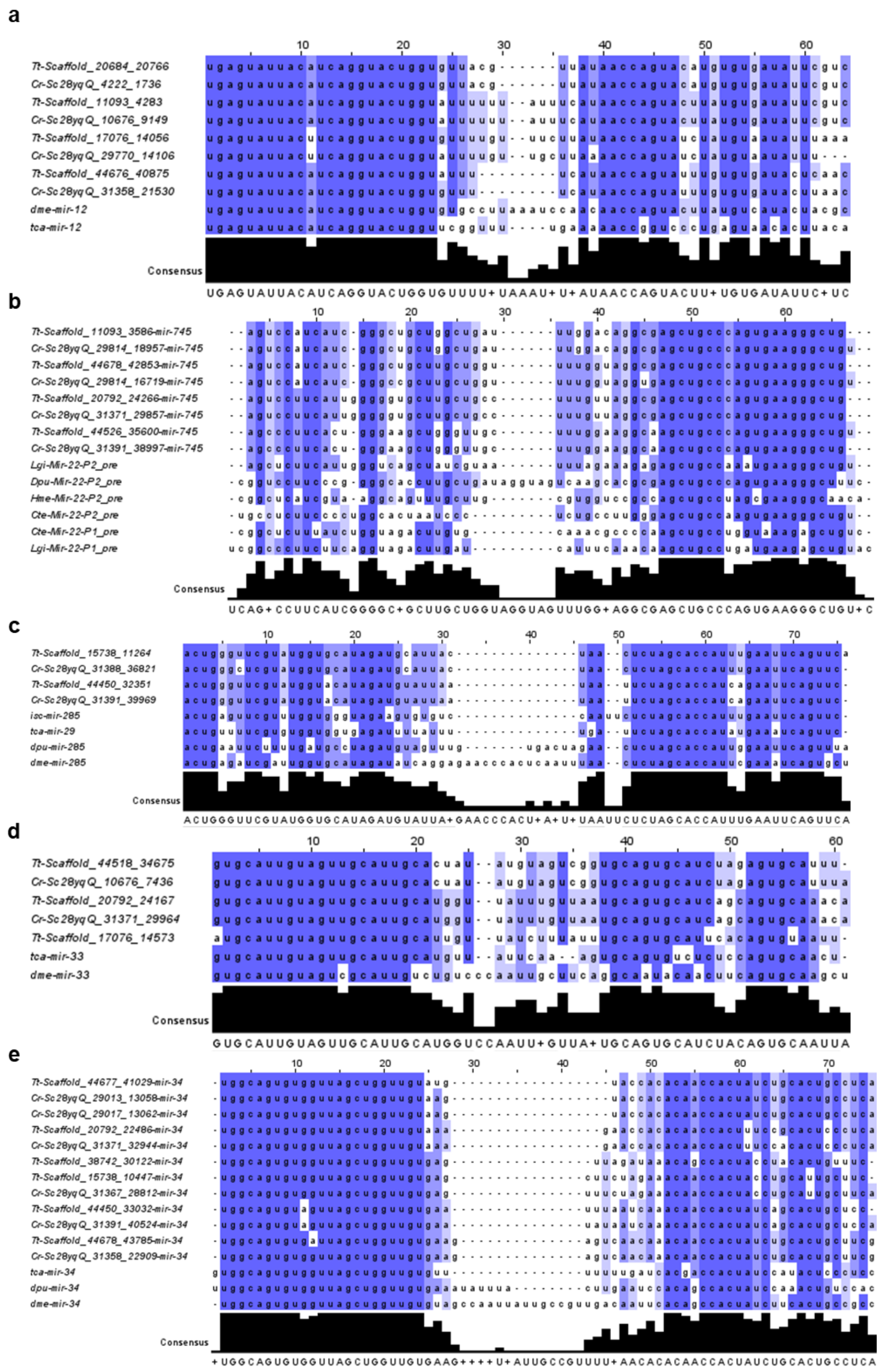

Supplementary Figure 12. Sequence alignments of mir-12, mir-22, mir-29, mir-33 and mir-34 assigned for the two horseshoe crab genomes. Sequence alignment was performed using MAFFT (Katoh and Standley 2013) and displayed by Jalview (Waterhouse et al 2009). a) mir-12; b) mir-22; c) mir-29; d) mir-33 and e) mir-34.

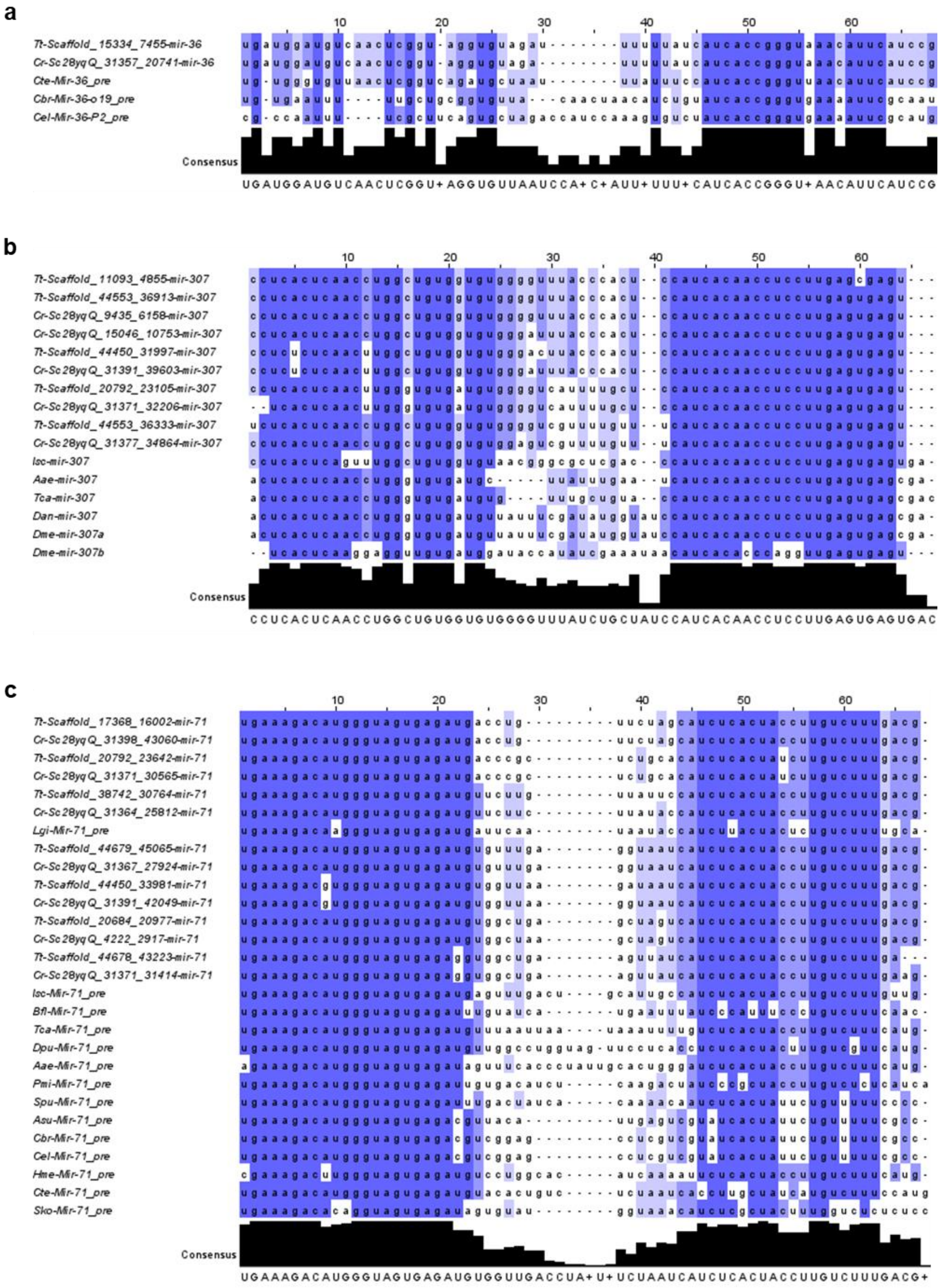

Supplementary Figure 13. Sequence alignments of mir-36, mir-307 and mir-71 assigned for the two horseshoe crab genomes. Sequence alignment was performed using MAFFT (Katoh and Standley 2013) and displayed by Jalview (Waterhouse et al 2009). a) mir-36; b) mir-307; c) mir-71.

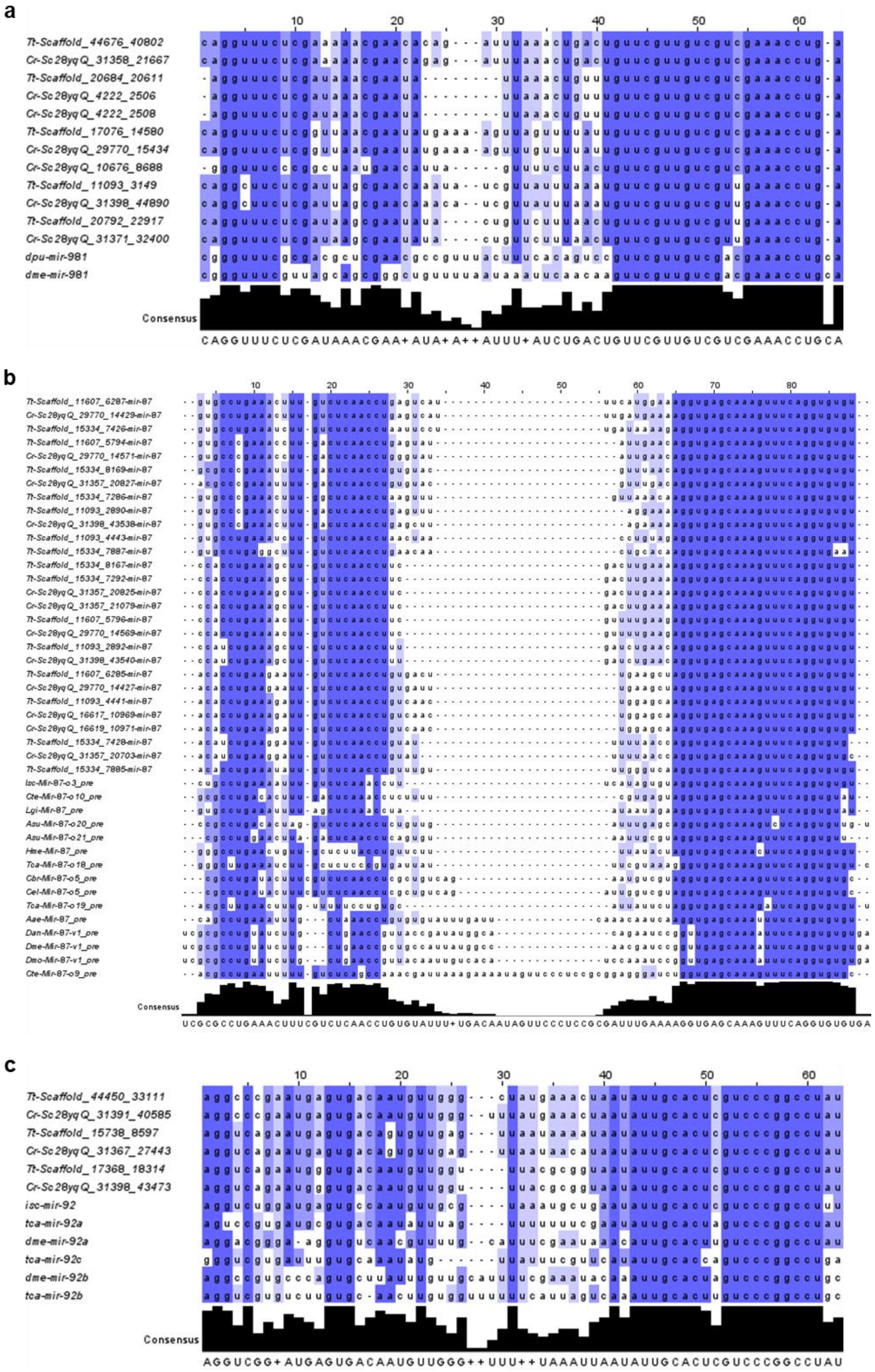

Supplementary Figure 14. Sequence alignments of mir-76, mir-87 and mir-92 families assigned for the two horseshoe crab genomes. Sequence alignment was performed using MAFFT (Katoh and Standley 2013) and displayed by Jalview (Waterhouse et al 2009). a) mir-981; b) mir-87; c) mir-92.

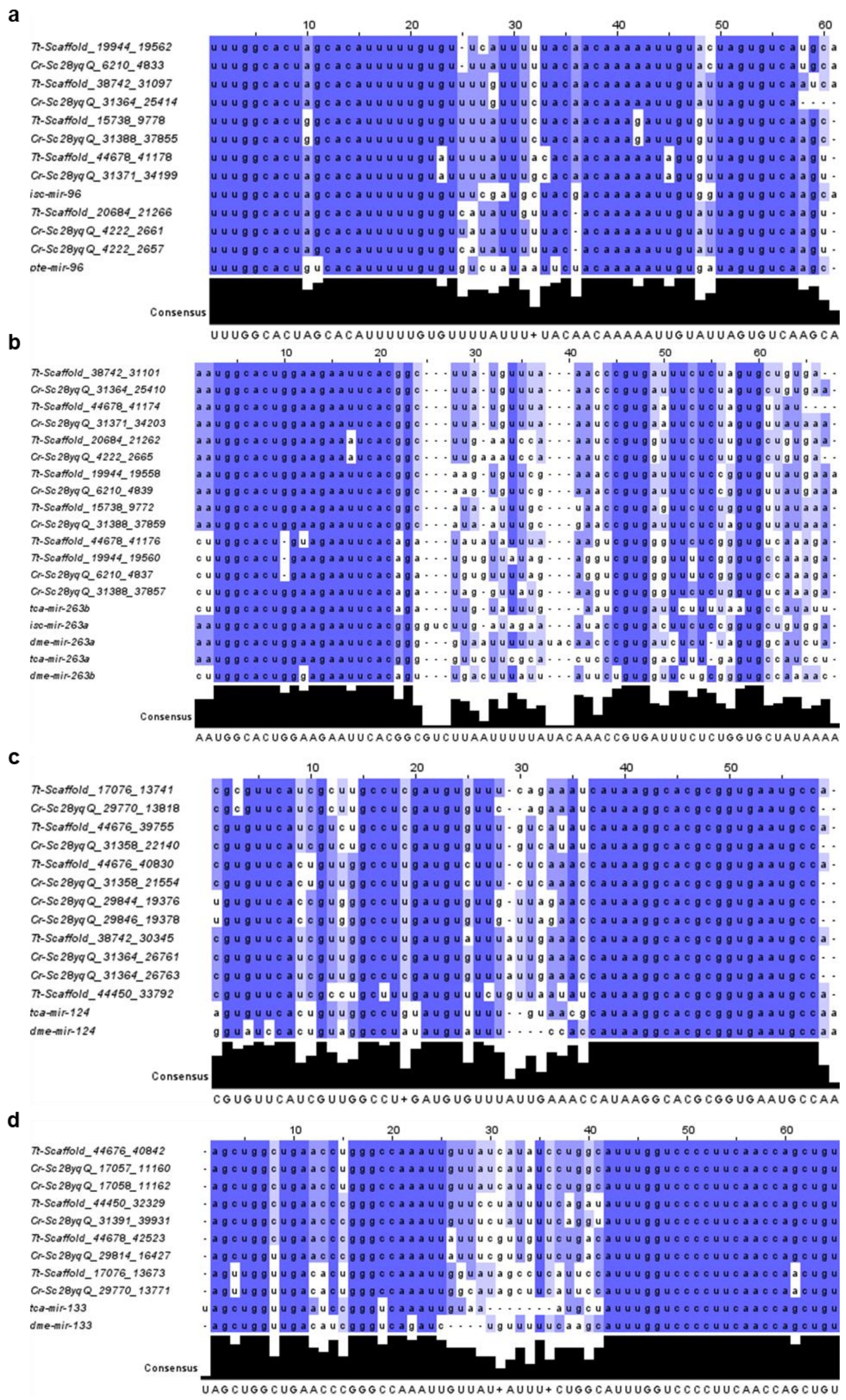

Supplementary Figure 15. Sequence alignments of mir-96, mir-124 and mir-133 families assigned for the two horseshoe crab genomes. Sequence alignment was performed using MAFFT (Katoh and Standley 2013) and displayed by Jalview (Waterhouse et al 2009). a) mir-96; b) mir-263; c) mir-124 and d) mir-133.

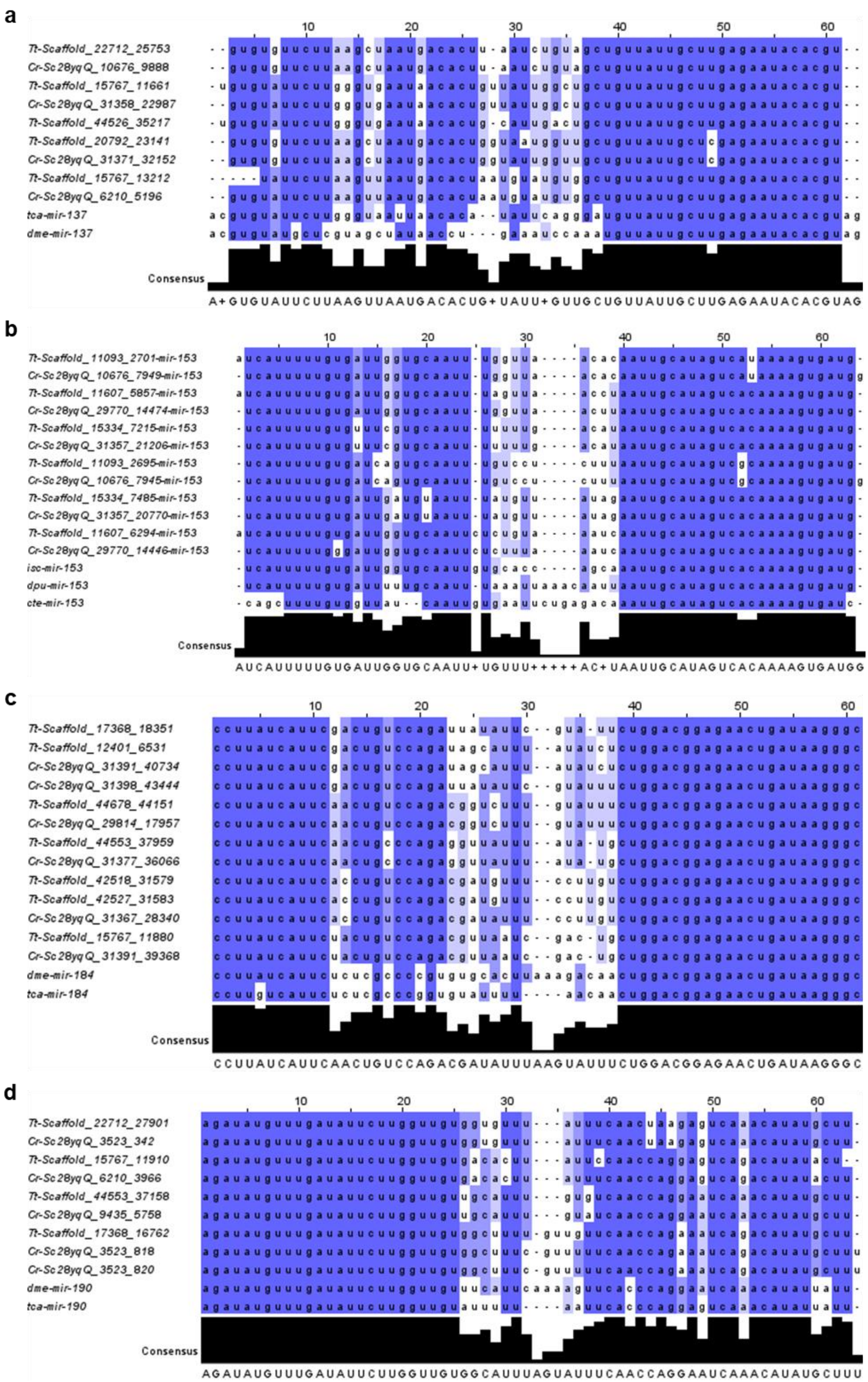

Supplementary Figure 16. Sequence alignments of mir-137, mir-153, mir-184 and mir-190 families assigned for the two horseshoe crab genomes. Sequence alignment was performed using MAFFT (Katoh and Standley 2013) and displayed by Jalview (Waterhouse et al 2009). a) mir-137; b) mir-153; c) mir-184 and d) mir-190.

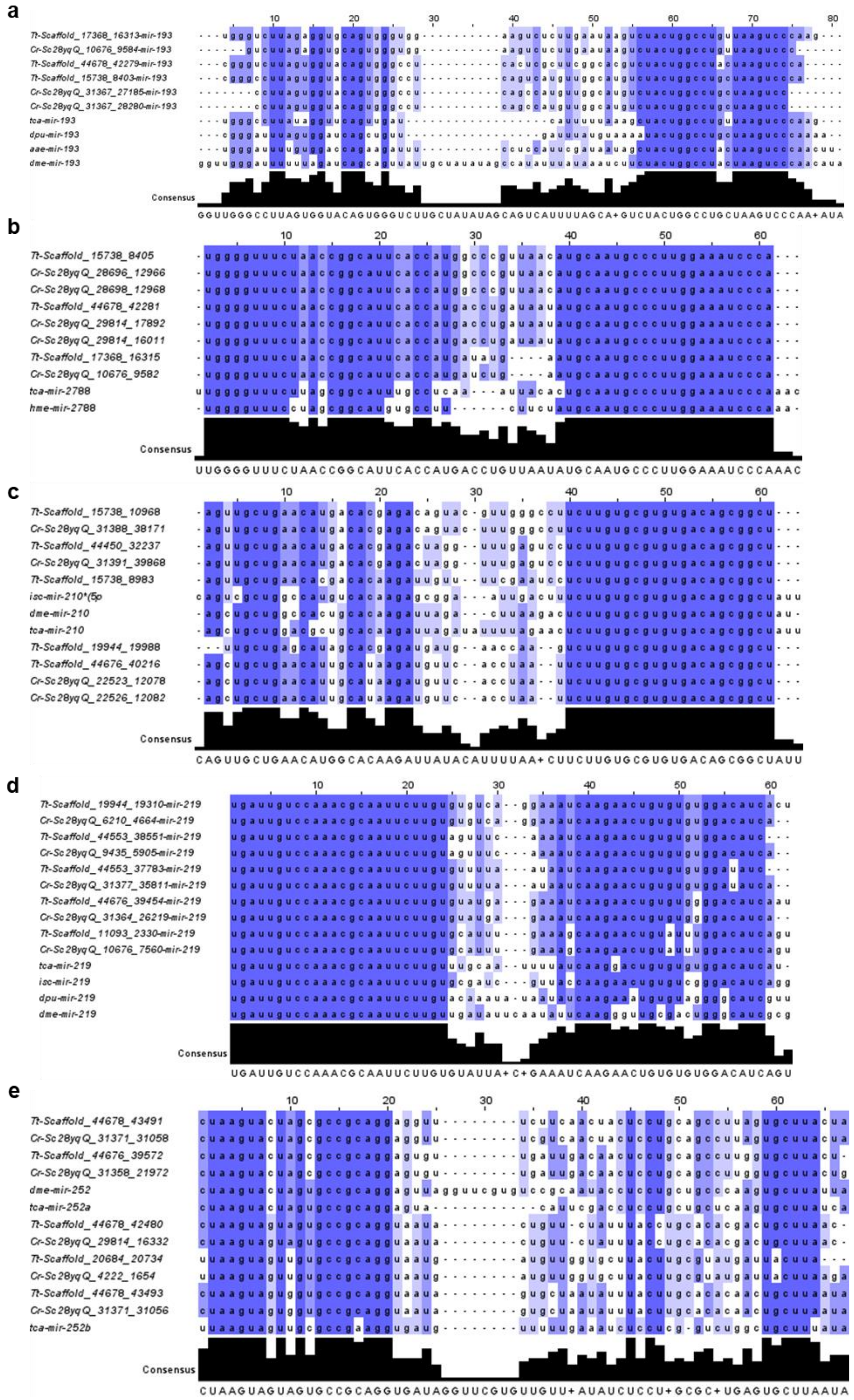

Supplementary Figure 17. Sequence alignments of mir-193, mir-210, mir-219 and mir-252 families assigned for the two horseshoe crab genomes. Sequence alignment was performed using MAFFT (Katoh and Standley 2013) and displayed by Jalview (Waterhouse et al 2009). a) mir-193; b) mir-2788; c) mir-210, d) mir-219 and e) mir-252.

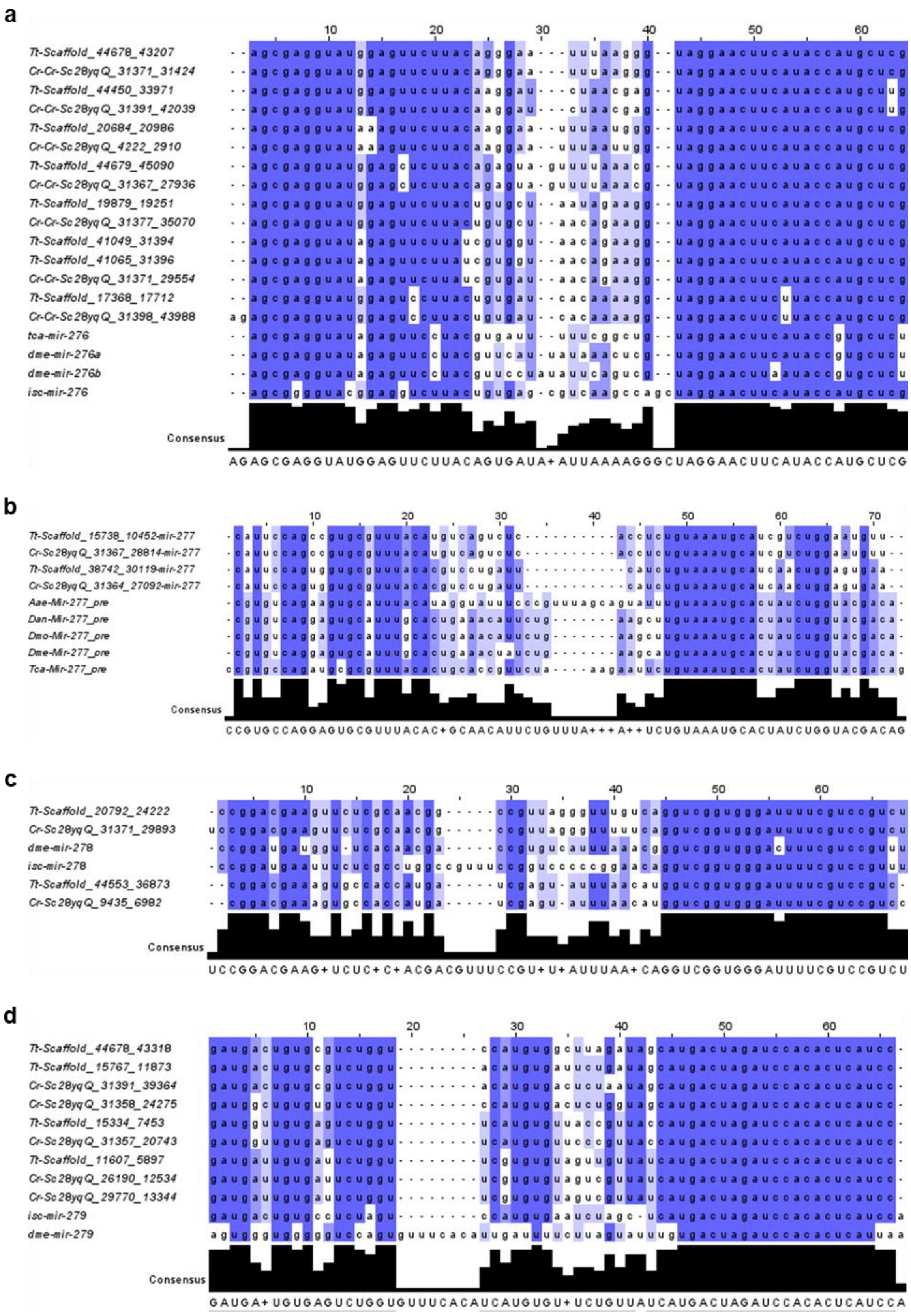

Supplementary Figure 18. Sequence alignments of mir-276, mir-277, mir-278 and mir-279 families assigned for the two horseshoe crab genomes. Sequence alignment was performed using MAFFT (Katoh and Standley 2013) and displayed by Jalview (Waterhouse et al 2009). a) mir-276; b) mir-277; c) mir-278 and d) mir-279.

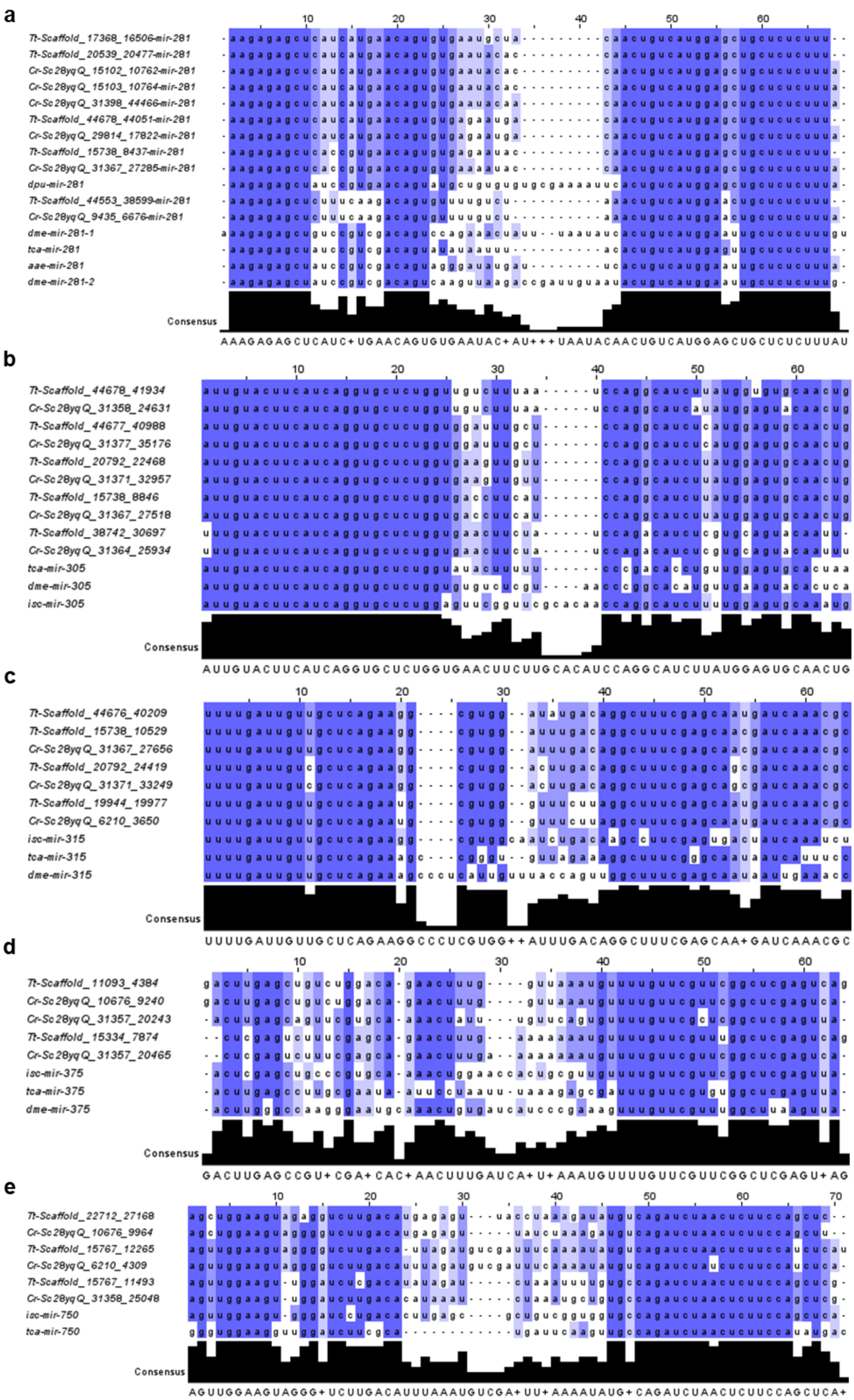

Supplementary Figure 19. Sequence alignments of mir-281, mir-305, mir-315, mir-375 and mir-750 families assigned for the two horseshoe crab genomes. Sequence alignment was performed using MAFFT (Katoh and Standley 2013) and displayed by Jalview (Waterhouse et al 2009). a) mir-281; b) mir-305; c) mir-315; d) mir-375 and e) mir-750.

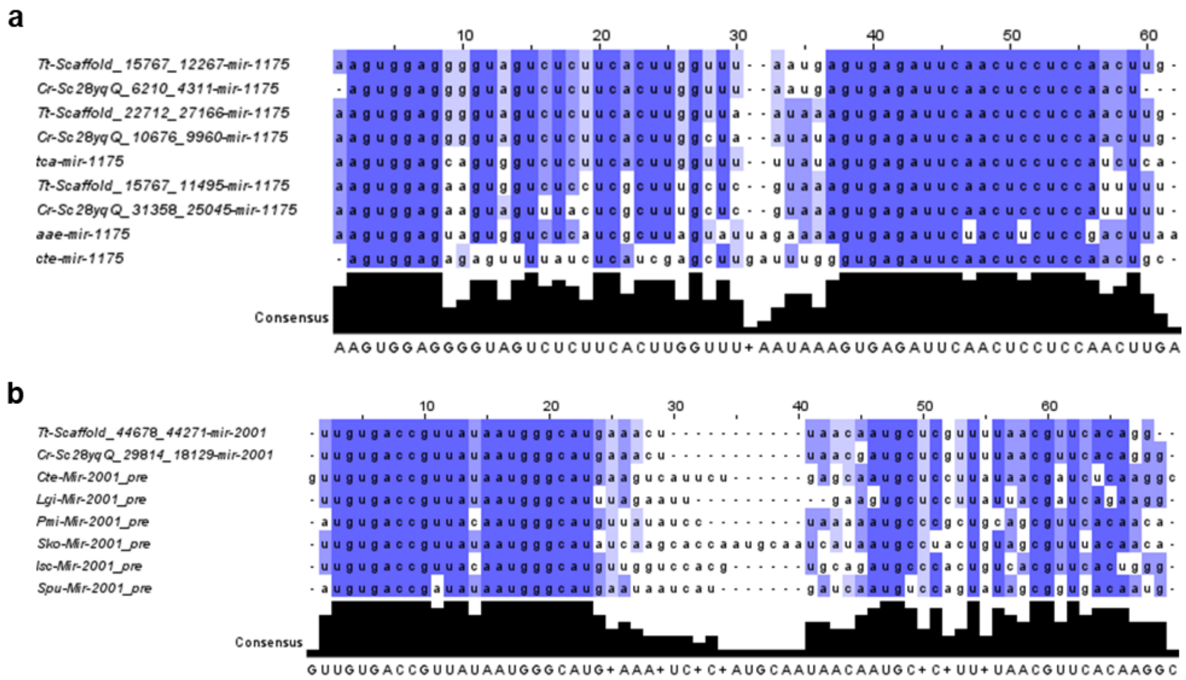

Supplementary Figure 20. Sequence alignments of mir-1175 and mir-2001 families assigned for the two horseshoe crab genomes. Sequence alignment was performed using MAFFT (Katoh and Standley 2013) and displayed by Jalview (Waterhouse et al 2009). a) mir-1175 and b) mir-2001.

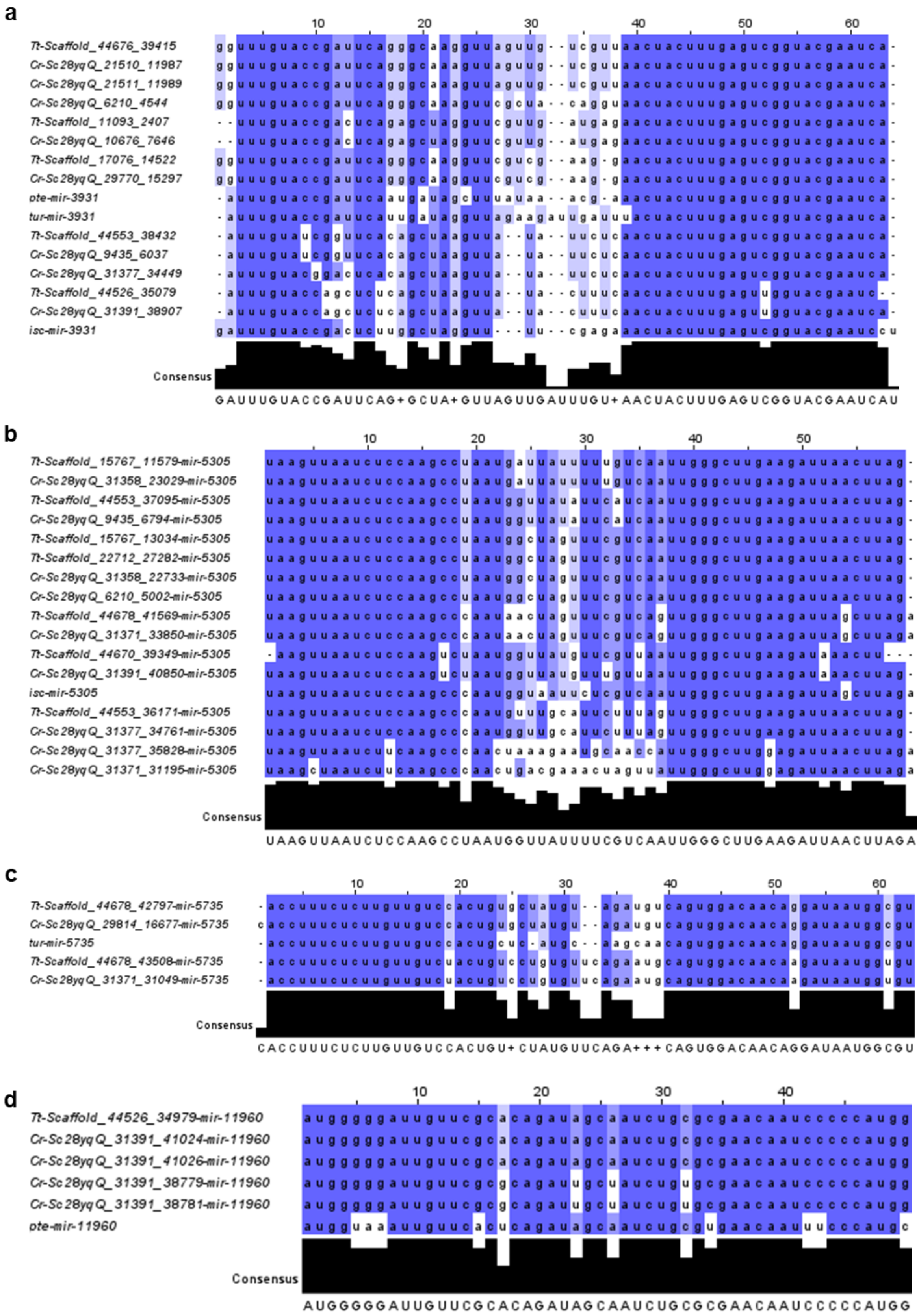

Supplementary Figure 21. Sequence alignments of Chelicerate specific miRNAs. Sequence alignment was performed using MAFFT (Katoh and Standley 2013) and displayed by Jalview (Waterhouse et al 2009). a) mir-3931; b) mir-5305; c) mir-5375 and d) mir-11960.

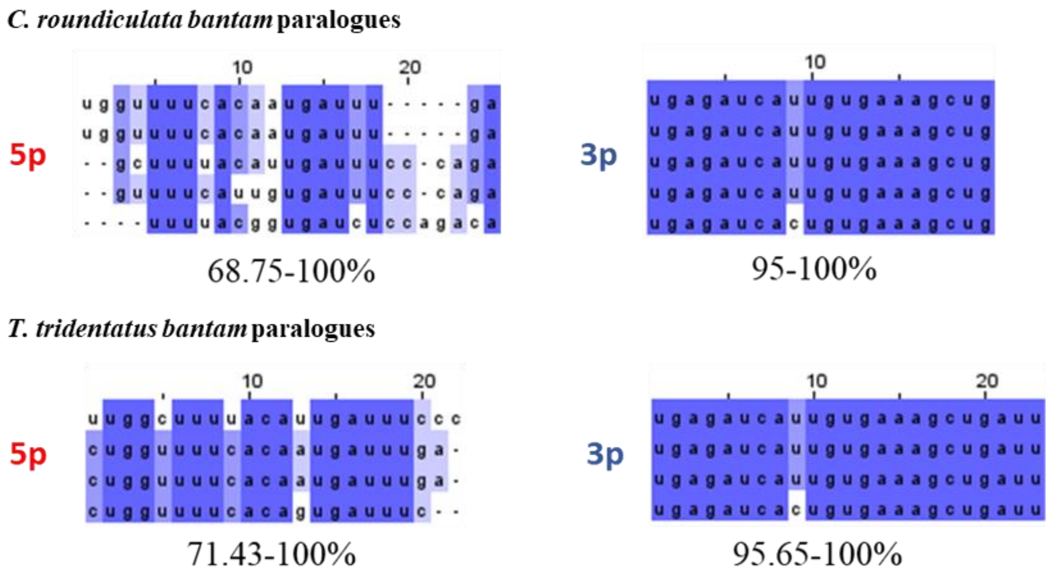

Supplementary Figure 22. Degree of sequence conservation between bantam paralogues.

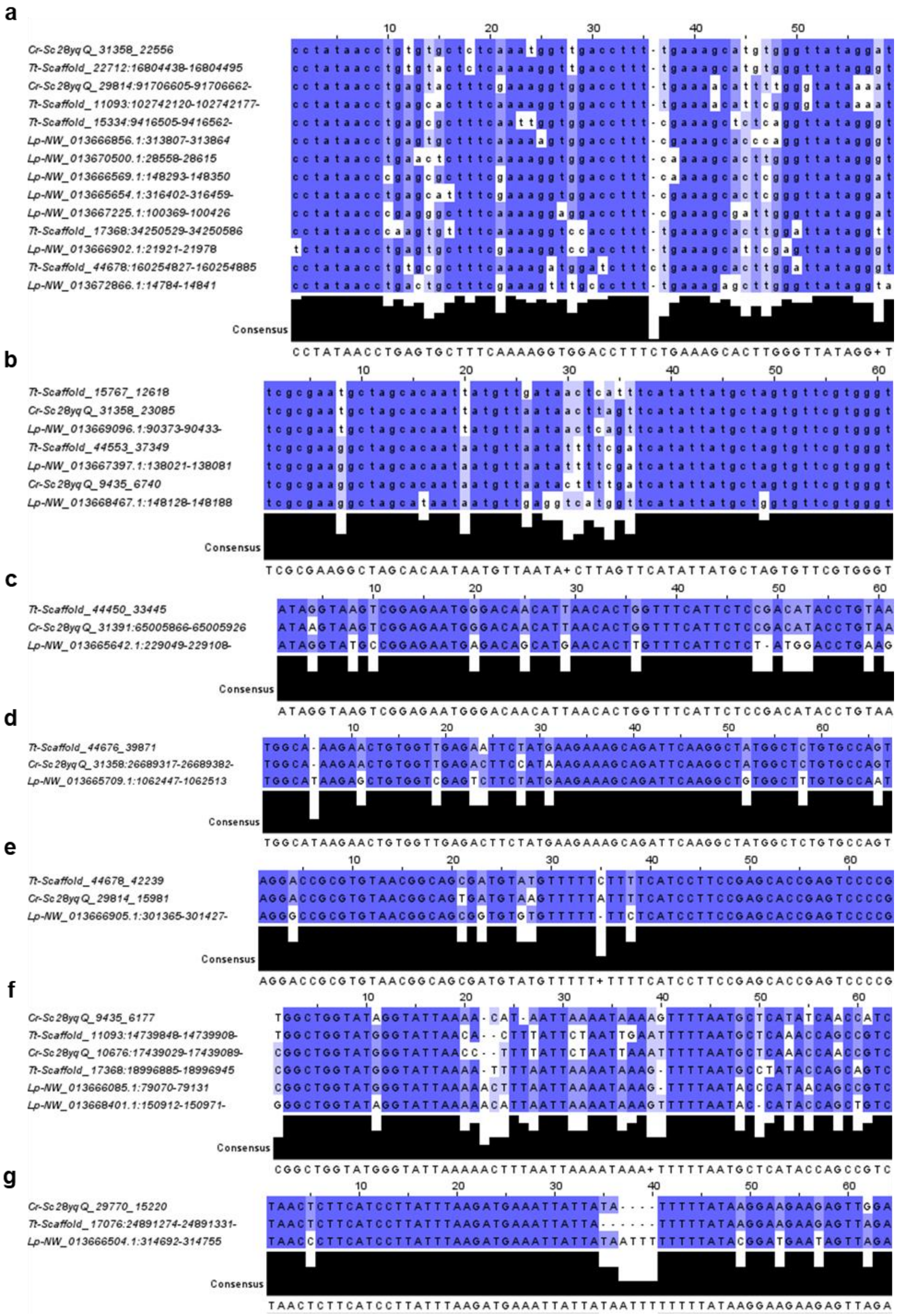

Supplementary Figure 23. Sequence alignments of 7 xiphosuran novel microRNAs conserved in three horseshoe crab genomes. Sequence alignment was performed using MAFFT (Katoh and Standley 2013) and displayed by Jalview (Waterhouse et al 2009). a-g: HC-N1-HC-N7.

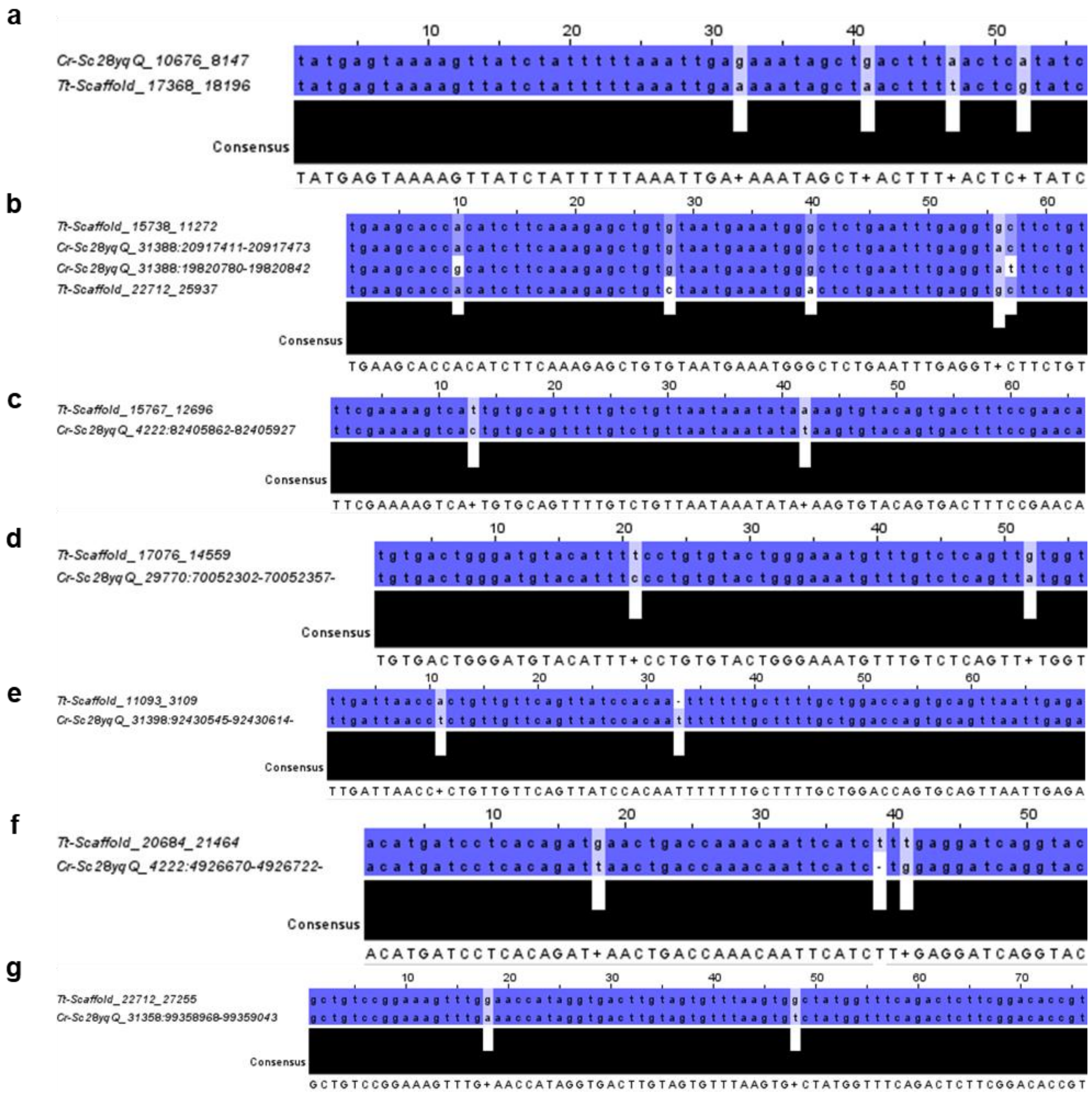

Supplementary Figure 24. Sequence alignments of 7 xiphosuran novel microRNAs conserved in both Tt and Cr. Sequence alignment was performed using MAFFT (Katoh and Standley 2013) and displayed by Jalview (Waterhouse et al 2009). a-g: CT-N1-CT-N7.

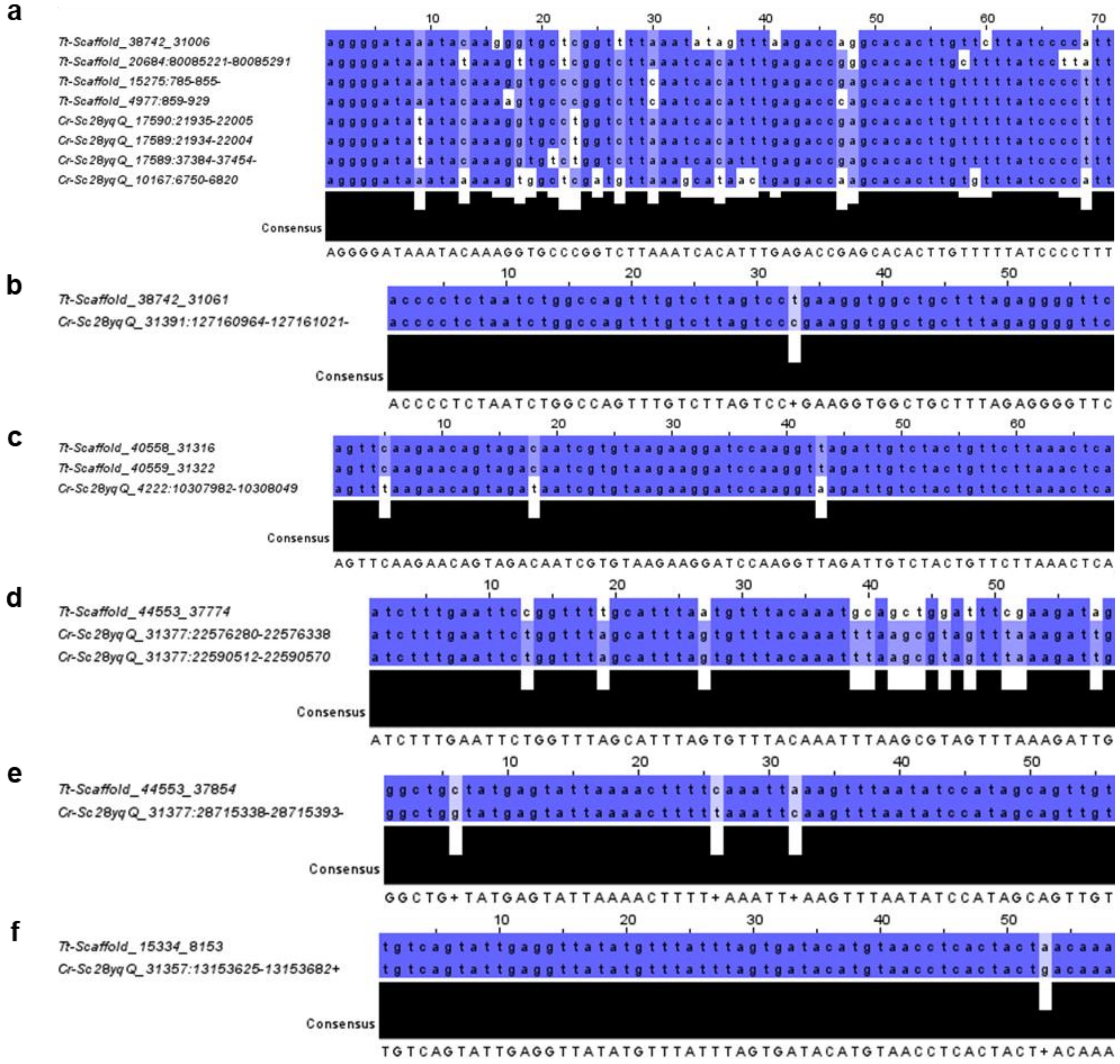

Supplementary Figure 25. Sequence alignments of other 6 xiphosuran novel microRNAs conserved in both Tt and Cr. Sequence alignment was performed using MAFFT (Katoh and Standley 2013) and displayed by Jalview (Waterhouse et al 2009). a-f: CT-N8-CT-N13.

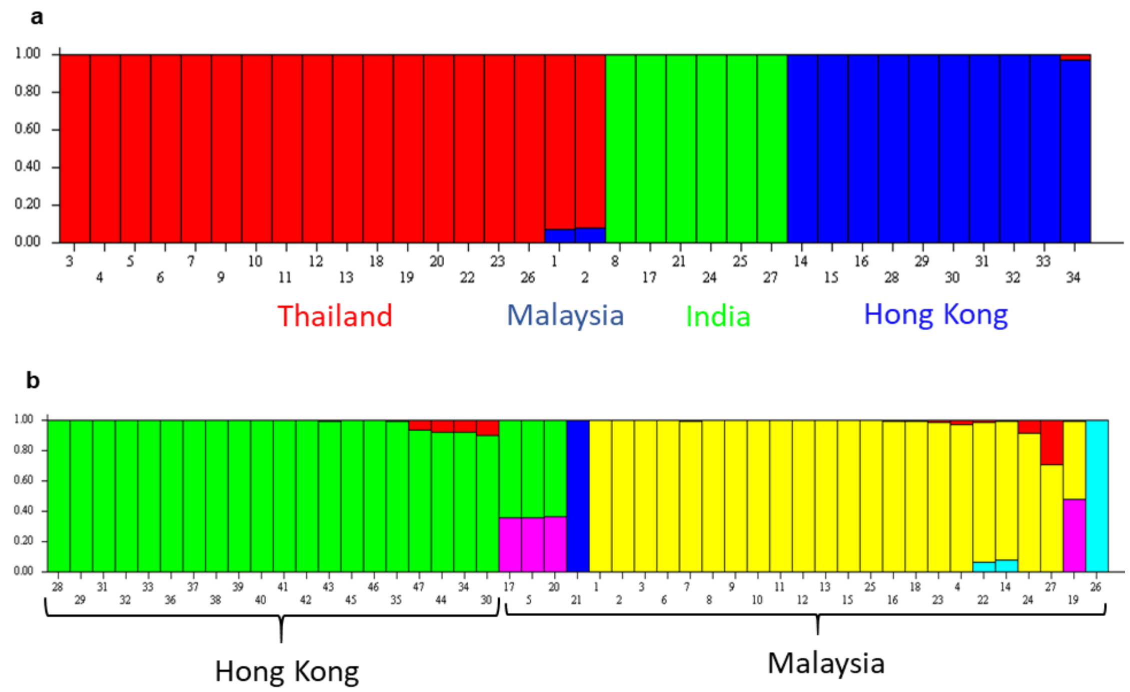

Supplementary Figure 26. Population structure of (a) *C. rotundicauda* (K=4, SNPs = 2249) and (b) *T. tridentatus* (K=2, SNPs = 2180).

a

Non-synonymous substitutions of *T. tridentatus* Six3/6-like in Malaysia population

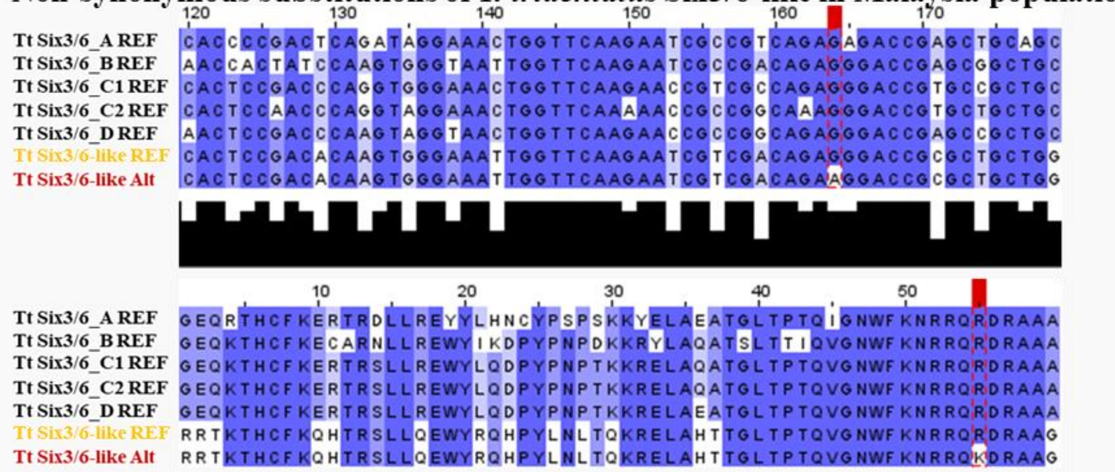

Six3/6-like	Hong Kong	Malaysia
ALT	0	15
REF	20	12

b

Non-synonymous substitutions of *C. roundiculata* En D in Thailand population

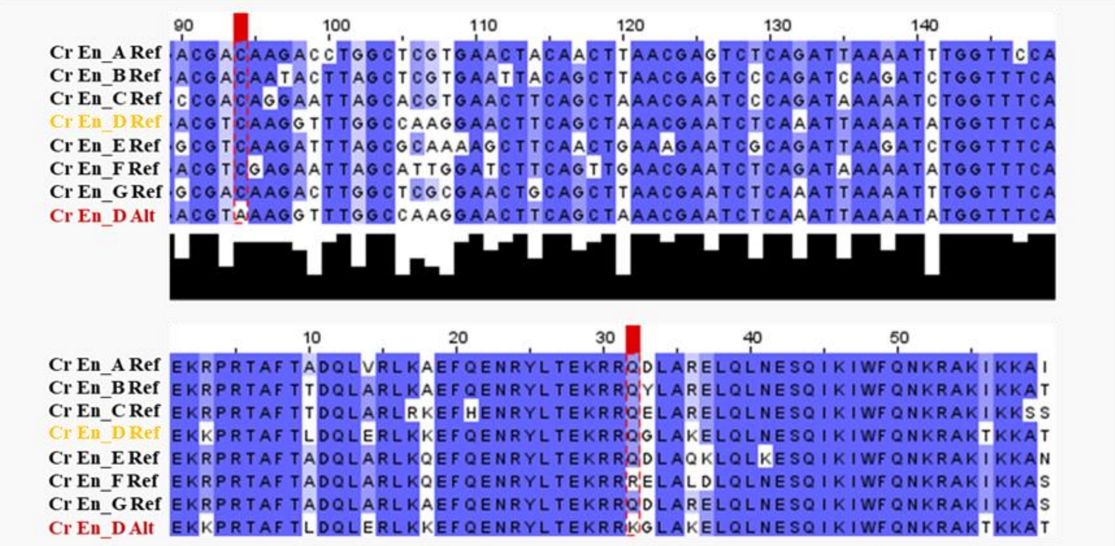

En-D	Hong Kong	Malaysia	Thailand	India
ALT	0	0	4	0
REF	10	2	12	6

Supplementary Figure 27. a) Non-synonymous substitutions of *T. tridentatus* Six3/6-like in Malaysia population; b) Non-synonymous substitutions of *C. roundiculata* En-D in Thailand population.

Supplementary Note

Transposable elements

The pattern of repeat content is highly similar for both species of horseshoe crab considered here, with the vast majority of repeats being transposable element (TE) rather than simple repeat, low complexity repeat, small RNA, or satellite (*C. roundiculata*: 32.76% TE vs 2.26% other repeats; *T. tridentatus*: 31.37% TE vs 1.61% other repeats, Supplementary Table 5). The proportion of each TE class present in each genome is also highly similar, with DNA elements accounting for the largest proportion of repeats, followed by LINEs and SINEs (Fig. 1c, Supplementary Table 7). LTR elements contribute a relatively small proportion of TEs in both genomes, with the contribution being several times smaller in *T. tridentatus* than in *C. roundiculata* (2.51% in *C. roundiculata* versus 0.88% in *T. tridentatus*, Supplementary Table 7). The described elements that contribute the greatest proportion of sequence in each TE class in both genomes are as follows: DNA elements - *Tc1*; LINEs - *I* elements; SINEs - tRNA-RTE elements; LTR elements - *Gypsy*-like elements. To our knowledge, only one study has considered repeat content in a horseshoe crab genome previously [(1)], however, the figures reported are contradictory, making a comparison with our results problematic (a TE content of 39.96% was reported for the *T. tridentatus* genome, but when summed together the figures reported in the accompanying TE table (i.e. their Supplementary Table 3) suggest a TE content of >47%).

In the *C. roundiculata* genome, repeats are evenly distributed across genic and intergenic regions (Fig. 1c). However, in the *T. tridentatus* genome, a greater proportion of repeats are found in genic regions, due to a higher density of DNA elements, LINEs, and unclassified elements (Fig. 1c). Repeat landscape plots (Fig. 1c) suggests a relatively similar pattern of historical TE activity for both species of horseshoe crab. However, recent activity appears to have tapered off more quickly in the *T. tridentatus* genome, particularly with respect to LTR elements, and certain DNA elements (Fig. 1c).

Supplementary Reference

1. Liao, Y. Y. *et al.* Draft genomic and transcriptome resources for marine chelicerate *Tachypleus tridentatus*. *Sci Data* 6, 190029, doi:10.1038/sdata.2019.29 (2019).
