# Supplementary material for: Horseshoe crab genomes reveal the evolution of genes and microRNAs after three rounds of whole genome duplication
Source: Commun Biol. 2021 Jan 19;4:83. doi: 10.1038/s42003-020-01637-2 (PMC7815833; doi:10.1038/s42003-020-01637-2)
Supplement: Supplementary file 3 — Description of Additional Supplementary Files [file 42003_2020_1637_MOESM3_ESM.pdf]

Description of Additional Supplementary Files

Supplementary Data 1. Information of homeobox gene sequence and genomic locations.

Supplementary Data 2. MicroRNA contents and arm usage of the two horseshoe crabs.

Supplementary Data 3. T. tridentatus microRNAs.

Supplementary Data 4. C. roundiculata microRNAs.

Supplementary Data 5. MicroRNA sequences of T. tridentatus and C. roundiculata

Supplementary Data 6. Horseshoe crab microRNA paralogues identity matrix.

Supplementary Data 7. SNPs at the homeodomains of the two horseshoe crabs.

Supplementary Data 8. Source data for Fig 1c and Fig 4a.
