# Supplementary material for: Horseshoe crab genomes reveal the evolution of genes and microRNAs after three rounds of whole genome duplication
Source: Commun Biol. 2021 Jan 19;4:83. doi: 10.1038/s42003-020-01637-2 (PMC7815833; doi:10.1038/s42003-020-01637-2)
Supplement: Supplementary file 6 — Supplementary Data 3 [file 42003_2020_1637_MOESM6_ESM.pdf]

Provisional ID	:	Scaffold_11093_2330
Score total	:	3881.5
Score for star read(s)	:	3.9
Score for read counts	:	3871.2
Score for mfe	:	1.8
Score for randfold	:	1.6
Score for cons. seed	:	3
Total read count	:	7605
Mature read count	:	7520
Loop read count	:	0
Star read count	:	85

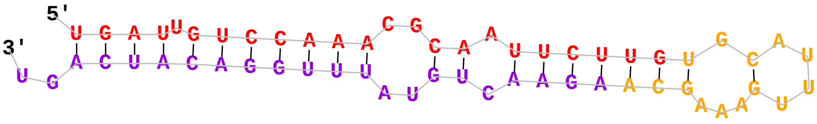

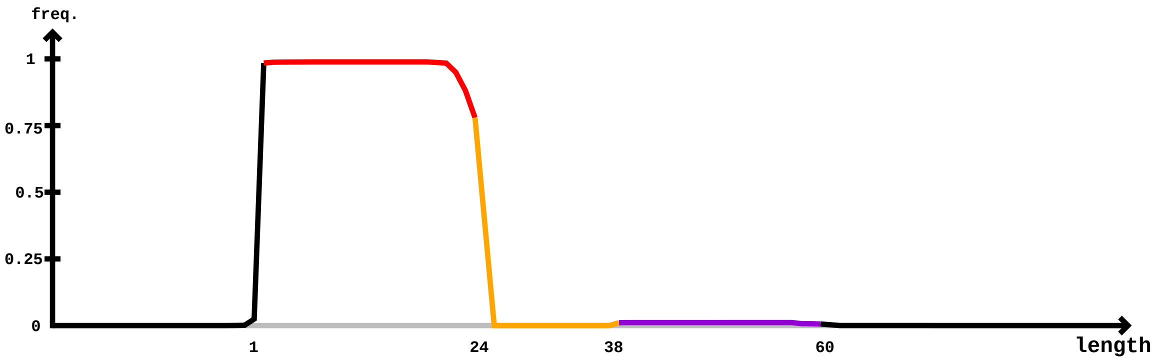

Mature Star

5'- ggaagcugaauuccaagcugugauuguccaaacgcaauucuugugcauuugaaagcaagaacuguauuuggacaucagugcuuggaaggcaauacaucugauacauuacc -3'	obs			
ggaagcugaauuccaagcugugauuguccaaacgcaauucuugugcauuugaaagcaagaacuguauuuggacaucagugcuuggaaggcaauacaucugauacauuacc	exp			
....(((...((((((((..((((.(((((((..((.(((((((.(....)...))))))).))..)))))))))))..))))))))))))....................		reads	mm	sample
..................ugugauuguccaaacgcaauuc..		3	0	1st
...................gugauuguccaaacgcaauu...		1	0	1st
...................gugauuguccaaacgcaauuc..		10	0	1st
...................gugauuguccaaacgcaauucu...		2	0	1st
...................gugauuguccaaacgcaauucuu..		21	0	1st
...................gugauuguccaaacgcaauucuug...		12	0	1st
....................ugauuguccaaacgcaau..		4	0	1st
....................ugauuguccaaacgcaauu...		3	0	1st
....................ugauuguccaaacgcaauuc..		67	0	1st
....................ugauuguccaaacgcaUuuc..		1	1	1st
....................ugauuguccaaacgcaauCcu...		1	1	1st
....................ugauuguccaaacgUaauucu...		1	1	1st
....................ugGuuguccaaacgcaauucu...		1	1	1st
....................ugauuguccaaacgcaauucu...		153	0	1st
....................ugauuguccaaacgcaauucuu..		219	0	1st
....................ugauuguccaaacgcaauuUuug...		1	1	1st
....................ugauugCccaaacgcaauucuug...		1	1	1st
....................ugauuguccaaacgcaauucuuU...		1	1	1st
....................Ggauuguccaaacgcaauucuug...		3	1	1st
....................ugauuguccaaacUcaauucuug...		1	1	1st
....................ugauuguccaaacgcaauucuug...		1438	0	1st
....................ugGuuguccaaacgcaauucuug...		1	1	1st
....................ugauuguccaaacgcaCuucuug...		1	1	1st
....................uAauuguccaaacgcaauucuug...		1	1	1st
....................ugauuguccaGacgcaauucuug...		1	1	1st
....................ugauuguccaaacAcaauucuug...		1	1	1st
....................Cgauuguccaaacgcaauucuug...		2	1	1st
....................ugauuguccaaacgUaauucuug...		2	1	1st
....................ugauuguccaaacgcaauGcuug...		3	1	1st
....................ugauuguccaaacgcaauCcuug...		1	1	1st
....................ugauuCuccaaacgcaauucuug...		1	1	1st
....................ugauuguccaaacgcaauucuugC..		1	1	1st
....................ugauuguccaaacgcaauucuugG..		3	1	1st

Mature **Star**

```
ggaagcugaauuccaagcugugauuguccaaacgcaauucuugugcauuugaaagcaagaacuguauuuggacaucagugcuuggaaggcaauacaucugauacauuacc
....................ugauuguccaaacgcaauucuugu..................................................................    4    0    1st
.....................gauuguccaaacgcaauucuu....................................................................    2    0    1st
.....................gauuguccaaacgcaauucuug...................................................................    2    0    1st
.......................uuguccaaacgcaauucuu....................................................................    1    0    1st
.........................guccaaacgcaauucuug...................................................................    1    0    1st
.........................................................agaacuguauuuggacaucag................................    1    0    1st
.........................................................agaacuguauuuggacaucagu...............................    2    0    1st

....................ugauuguccaaacgcaauucu.....................................................................    1    0    TJ1
....................ugauuguccaaacgcaauucuug...................................................................    1    0    TJ1
.........................................................agaacuguauuuggacauc..................................    15   0    TJ1
.........................................................agaacuguauuuggacauU..................................    1    1    TJ1
.........................................................agaacuguauuuggacaucaA................................    1    1    TJ1
.........................................................agaacuguauuuggacaucag................................    1    0    TJ1
.........................................................agaacuguauuuggacaucagA...............................    1    1    TJ1
.........................................................agaacuguauuuggacaucagC...............................    2    1    TJ1
.........................................................agaacuguauuuggacaucagu...............................    7    0    TJ1

....................ugauuguccaaacgcaauucuug...................................................................    1    0    B01

.................cuUgauuguccaaacgcaauucuug....................................................................    1    1    EGG
..................gugauuguccaaacgcaauu........................................................................    1    0    EGG
..................gugauuguccaaacgcaauucuu.....................................................................    6    0    EGG
..................gugauuguccaaacgcaauucuug....................................................................    2    0    EGG
....................ugauuguccaaacgcaauuc......................................................................    1    0    EGG
....................ugauuguccaaacgcaauucu.....................................................................    5    0    EGG
....................ugauuguccaaacgcaauucuu....................................................................    21   0    EGG
....................ugauuguccaaacgcaauucuGg...................................................................    1    1    EGG
....................ugauuguccaaacgcaauucuCg...................................................................    1    1    EGG
....................ugauuguccaaacgcaauucuug...................................................................    327  0    EGG
....................ugauuguccaaacgcaGuucuug...................................................................    1    1    EGG
....................ugauuguccaaacgcaauucuugA..................................................................    1    1    EGG
....................ugauuguccaaacgcaauucuugu..................................................................    1    0    EGG
......................auuguccaaacgcaauucuug...................................................................    1    0    EGG
.........................................................agaacuguauuuggacaucagu...............................    2    0    EGG

....................ugauuguccaaacgcaauucuug...................................................................    2    0    HA1

.........................................................agaacuguauuuggacauc..................................    3    0    BJ1

....................ugauuguccaaacgcaauucuug...................................................................    2    0    LJ1
.........................................................agaacuguauuuggacauc..................................    4    0    LJ1

..................gugauuguccaaacgcaauuc.......................................................................    1    0    E01
..................gugauuguccaaacgcaauucuu.....................................................................    1    0    E01
..................gugauuguccaaacgcaauucuug....................................................................    1    0    E01
....................ugauuguccaaacgcaauuc......................................................................    3    0    E01
....................ugauuguccaaacgcaauucu.....................................................................    2    0    E01
....................ugauuguccaaacgcaauucuu....................................................................    14   0    E01
....................ugauuguccaaacgcaauuUuug...................................................................    1    1    E01
....................ugauuguccaaacgUaauucuug...................................................................    1    1    E01
....................ugauuguccaaacgcaauucuug...................................................................    319  0    E01
....................ugauuguccaaacAcaauucuug...................................................................    1    1    E01
....................ugauuguccaaacgcaauucuugu..................................................................    2    0    E01
.....................gauuguccaaacgcaauucuug...................................................................    1    0    E01
........................uguccaaacgcaauucuug...................................................................    1    0    E01

....................ugauuguccaaacgcaauucu.....................................................................    1    0    TA1
......................auuguccaaacgcaauucu.....................................................................    1    0    TA1
.........................................................agaacuguauuuggacauc..................................    1    0    TA1
.........................................................agaacuguauuuggacaucagu...............................    2    0    TA1

....................ugauuguccaaacgcaauucuug...................................................................    1    0    LA5

.................ugugauuguccaaacgcaauuc.......................................................................    1    0    2nd
..................gugauuguccaaacgcaauu........................................................................    2    0    2nd
..................gugauuguccaaacgcaauuc.......................................................................    15   0    2nd
..................gugauuguccaaacgcaauucu......................................................................    4    0    2nd
..................gugauuguccaaacgcaauucuu.....................................................................    12   0    2nd
..................gugauuguccaaacgcaauucuug....................................................................    16   0    2nd
```

Mature **Star**

```
ggaagcugaauuccaagcugugauuguccaaacgcaauucuugugcauuugaaagcaagaacuguauuuggacaucagugcuuggaaggcaauacaucugauacauuacc
....................ugauuguccaaacgcaau........................................................................  1     0  2nd
....................ugauuguccaaacgcaauu.......................................................................  5     0  2nd
....................Cgauuguccaaacgcaauuc......................................................................  1     1  2nd
....................ugauuguccaaacgcaauuc......................................................................  64    0  2nd
....................ugauuguccaaacgcaGuuc......................................................................  1     1  2nd
....................ugaGuguccaaacgcaauucu.....................................................................  1     1  2nd
....................ugauugCccaaacgcaauucu.....................................................................  1     1  2nd
....................ugauuguccaaacgcaauucu.....................................................................  122   0  2nd
....................ugauuguccaaacgcaGuucu.....................................................................  1     1  2nd
....................ugauuguccaaacgcaauucuC....................................................................  1     1  2nd
....................ugauugCccaaacgcaauucuu....................................................................  1     1  2nd
....................ugauuguccaaacgcaauucuA....................................................................  2     1  2nd
....................ugauuguccaaacgcaauucuu....................................................................  168   0  2nd
....................uCauuguccaaacgcaauucuug...................................................................  1     1  2nd
....................ugauuguccaaacgcaaGucuug...................................................................  1     1  2nd
....................ugauuguccaaacgcaauuUuug...................................................................  3     1  2nd
....................ugauGguccaaacgcaauucuug...................................................................  1     1  2nd
....................ugauuCuccaaacgcaauucuug...................................................................  1     1  2nd
....................ugauuguccaaacUcaauucuug...................................................................  1     1  2nd
....................ugGuuguccaaacgcaauucuug...................................................................  3     1  2nd
....................ugauuUuccaaacgcaauucuug...................................................................  1     1  2nd
....................ugauuguccaaacgAaauucuug...................................................................  1     1  2nd
....................Ggauuguccaaacgcaauucuug...................................................................  2     1  2nd
....................Agauuguccaaacgcaauucuug...................................................................  1     1  2nd
....................ugauuguccaaacAcaauucuug...................................................................  2     1  2nd
....................ugauuguccaaacgcaauucuCg...................................................................  5     1  2nd
....................ugauuguccaaacgcaauucuug...................................................................  1368  0  2nd
....................ugauuguccaaaUgcaauucuug...................................................................  1     1  2nd
....................ugauuAuccaaacgcaauucuug...................................................................  1     1  2nd
....................ugauuguccaaacgUaauucuug...................................................................  1     1  2nd
....................ugauuguccaaacgcaauucuugu..................................................................  8     0  2nd
....................ugauuguccaaacgcaauucuugG..................................................................  1     1  2nd
....................ugauuguccaaacgcaauucuugC..................................................................  1     1  2nd
.....................gauuguccaaacgcaauucuu....................................................................  1     0  2nd
.....................gauuguccaaacgcaauucuug...................................................................  2     0  2nd
.......................uuguccaaacgcaauucu.....................................................................  1     0  2nd
.........................guccaaacgcaauucuug...................................................................  1     0  2nd
........................................................aagaacuguauuuggacaucag................................  1     0  2nd
.........................................................agaacuguauuuggacaucagu...............................  8     0  2nd

....................ugauuguccaaacgcaauucu.....................................................................  1     0  LJ5
....................ugauuguccaaacgUaauucuu....................................................................  1     1  LJ5
....................ugauuguccaaacgcaauucuug...................................................................  1     0  LJ5
.........................................................agaacuguauuuggacauc..................................  1     0  LJ5
..........................................................gaacuguauuuggacaucagu...............................  3     0  LJ5

...................gugauuguccaaacgcaauuc......................................................................  1     0  H01
...................gugauuguccaaacgcaauucu.....................................................................  1     0  H01
...................gugauuguccaaacgcaauucuug...................................................................  1     0  H01
....................ugauuguccaaacgUaau........................................................................  1     1  H01
....................ugauuguccaaacgcaau........................................................................  4     0  H01
....................ugauuguccaaacgcaauu.......................................................................  1     0  H01
....................ugauuguccaaacgcaauuc......................................................................  3     0  H01
....................ugauuguccaaacgcaauucu.....................................................................  4     0  H01
....................ugauuguccaaacgcaauucuu....................................................................  2     0  H01
....................ugauuguccaaacgcaauucuCg...................................................................  1     1  H01
....................ugauuguccaaacgcaauucuug...................................................................  49    0  H01

....................ugauuguccaaacgcaauucuug...................................................................  2     0  CA1

.................Uugugauuguccaaacgcaauuc......................................................................  1     1  3rd
..................ugugauuguccaaacgcaauuc......................................................................  1     0  3rd
...................gugauuguccaaacgcaau........................................................................  1     0  3rd
...................gugauuguccaaacgcaauuc......................................................................  13    0  3rd
...................gugauuguccaaacgcaauucu.....................................................................  6     0  3rd
...................gugauuguccaaacgcaauucuu....................................................................  27    0  3rd
...................gugauuguccaaacgcaauucuug...................................................................  20    0  3rd
....................ugauuguccaaacgcaau........................................................................  4     0  3rd
....................ugauuguccaaacgcaauu.......................................................................  5     0  3rd
....................ugauuguccaaacgcaauuc......................................................................  68    0  3rd
```

Mature **Star**

```
ggaagcugaauuccaagcugugauuguccaaacgcaauucuugugcauuugaaagcaagaacuguauuuggacaucagugcuuggaaggcaauacaucugauacauuacc
....................ugauuAuccaaacgcaauuc......................................................................  1     1  3rd
....................Ggauuguccaaacgcaauucu.....................................................................  1     1  3rd
....................ugauuguccaaacgcaGuucu.....................................................................  2     1  3rd
....................ugauuguccaaacgcaauuUu.....................................................................  2     1  3rd
....................ugauuguccaaacgcaauucu.....................................................................  196   0  3rd
....................ugauuguccaaacgcaauucuu....................................................................  256   0  3rd
....................ugauuCuccaaacgcaauucuu....................................................................  1     1  3rd
....................ugauuguccaaacgcaauucuC....................................................................  1     1  3rd
....................ugauuguccaaacgUaauucuu....................................................................  1     1  3rd
....................ugGuuguccaaacgcaauucuu....................................................................  1     1  3rd
....................Agauuguccaaacgcaauucuu....................................................................  1     1  3rd
....................Ggauuguccaaacgcaauucuug...................................................................  1     1  3rd
....................Agauuguccaaacgcaauucuug...................................................................  2     1  3rd
....................ugauuguccaaacgcaauucuCg...................................................................  1     1  3rd
....................ugauuguccaaacgcaaGucuug...................................................................  1     1  3rd
....................ugauugGccaaacgcaauucuug...................................................................  1     1  3rd
....................ugauuguccaCacgcaauucuug...................................................................  1     1  3rd
....................ugauuguccaGacgcaauucuug...................................................................  1     1  3rd
....................Cgauuguccaaacgcaauucuug...................................................................  1     1  3rd
....................ugaCuguccaaacgcaauucuug...................................................................  1     1  3rd
....................ugauCguccaaacgcaauucuug...................................................................  1     1  3rd
....................ugauuguccaaacgcaauucAug...................................................................  1     1  3rd
....................ugauuguAcaaacgcaauucuug...................................................................  1     1  3rd
....................ugauuguccaaacgcaauuUuug...................................................................  1     1  3rd
....................ugauuguccaaacUcaauucuug...................................................................  3     1  3rd
....................ugauuguccaaaUgcaauucuug...................................................................  2     1  3rd
....................ugauuguccaaacgcaaCucuug...................................................................  1     1  3rd
....................ugauuguccaaacAcaauucuug...................................................................  5     1  3rd
....................ugauuguccaaacgcaauucuuA...................................................................  2     1  3rd
....................ugauuguccaaacgcCauucuug...................................................................  1     1  3rd
....................ugauuguccaaacgcaauucuug...................................................................  2045  0  3rd
....................ugauuguccaaacgcUauucuug...................................................................  1     1  3rd
....................ugauugAccaaacgcaauucuug...................................................................  1     1  3rd
....................ugauuAuccaaacgcaauucuug...................................................................  3     1  3rd
....................ugGuuguccaaacgcaauucuug...................................................................  2     1  3rd
....................ugauuguccaaacgcaauuGuug...................................................................  1     1  3rd
....................ugauuguccaaacgcaauucuugA..................................................................  6     1  3rd
....................Agauuguccaaacgcaauucuugu..................................................................  1     1  3rd
....................ugauuguccaaacgcaauucuugu..................................................................  14    0  3rd
.....................gauuguccaaacgcaauuU......................................................................  1     1  3rd
.....................Cauuguccaaacgcaauucu.....................................................................  1     1  3rd
.....................gauuguccaaacgcaauucuug...................................................................  12    0  3rd
......................auuguccaaacgcaauucuug...................................................................  2     0  3rd
.........................................................agaacuguauuuggacauca.................................  2     0  3rd
.........................................................agaacuguauuuggacaucag................................  1     0  3rd
.........................................................agaacuguauuuggacaucagu...............................  7     0  3rd
....................................................................uggacaucagugcuuggaaggc....................  1     0  3rd

.........................................................agaacuguauuuggacauc..................................  1     0  BRA
.........................................................agaacuguauuuggacaucag................................  1     0  BRA
.........................................................agaacuguauuuggacaucagu...............................  1     0  BRA

....................ugauuguccaaacgcaauucuug...................................................................  1     0  LA1
.........................................................agaacuguauuuggacaucag................................  1     0  LA1

....................ugauuguccaaacgcaauucuug...................................................................  1     0  HJ1
....................ugauuguccaGacgcaauucuug...................................................................  1     1  HJ1
.........................................................agaacuguauuuggacauA..................................  1     1  HJ1
.........................................................agaacuguauuuggacauU..................................  2     1  HJ1
.........................................................agUacuguauuuggacaucag................................  1     1  HJ1
.........................................................agaacuguauuuggacaucagu...............................  8     0  HJ1
.........................................................agaacuguauuuggacaucagC...............................  1     1  HJ1

.........................................................agaacuguauuuggacauc..................................  1     0  CJ1
.........................................................agaacuguauuuggacauU..................................  1     1  CJ1

...................gugauuguccaaacgcaauucuug...................................................................  2     0  G01
....................ugauuguccaaacgcaau........................................................................  2     0  G01
....................ugauuguccaaacgcaauuc......................................................................  6     0  G01
....................ugauuguccaaacgcaauucu.....................................................................  9     0  G01
```

```
                    Mature                                 Star
ggaagcugaauuccaagcugugauuguccaaacgcaauucuugugcauuugaaagcaagaacuguauuuggacaucagugcuuggaaggcaauacaucugauacauuacc
....................ugauuguccaaacgcaauucuu....................................................................   13    0    G01
....................ugauuguccaaacgcaauucuuC...................................................................   1     1    G01
....................ugauuguccaaacAcaauucuug...................................................................   1     1    G01
....................ugauuguccaaacgcaauucuug...................................................................   155   0    G01
....................ugauugCccaaacgcaauucuug...................................................................   1     1    G01
....................ugauuguccaaacgcaauucuugu..................................................................   1     0    G01
```

Provisional ID	:	Scaffold_11093_2407
Score total	:	725512.5
Score for star read(s)	:	3.9
Score for read counts	:	725501.7
Score for mfe	:	2.3
Score for randfold	:	1.6
Score for cons. seed	:	3
Total read count	:	1423052
Mature read count	:	1422081
Loop read count	:	0
Star read count	:	971

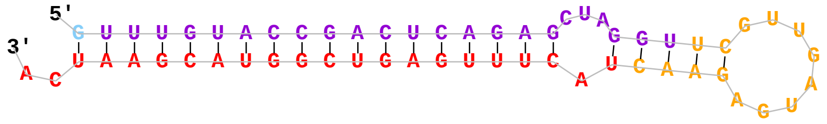

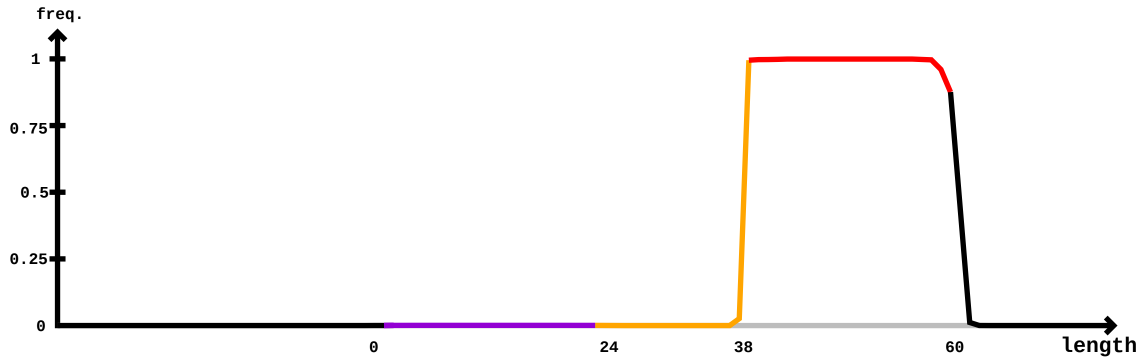


```
                                 Star                            Mature
5'- ggaucaaaauaacuuaacgugguucaggggcgguuuguaccgacucagagcuagguucguugaugagaacuacuuugagucgguacgaaucaccucagccaugaucca   -3'   obs
    ggaucaaaauaacuuaacgugguucaggggcgguuuguaccgacucagagcuagguucguugaugagaacuacuuugagucgguacgaaucaccucagccaugaucca         exp
    ((((((.............(((((..((((.(((((((((((((((((((...(((((........))))).))))))))))))))))))).))))))))))))))).   reads   mm   sample
    ...............................gguuuguaccgacucagagcu........................................................   1       0    EGG
    ...............................gguuuguaccgacucagagcua.......................................................   2       0    EGG
    ...............................gguuuguaccgacucagagcuU.......................................................   1       1    EGG
    ...............................gguuuguaccgacucagagcuag......................................................   1       0    EGG
    ...............................gguuuguaccgacucagagcuagg.....................................................   1       0    EGG
    ...............................gguuuguaccgacucagagcuaggu....................................................   6       0    EGG
    ...............................gguuuguaccgacucagagcuagguu...................................................   5       0    EGG
    .................................uuuguaccgacucagagcuaggu....................................................   3       0    EGG
    .................................uuuguaccgacucagagcuagguu...................................................   2       0    EGG
    ...................................uguaccgacucagagcuagguu...................................................   1       0    EGG
    ...................................uguaccgacucagagcuagguuc..................................................   1       0    EGG
    ...................................................................aacuacuuugagucgguacga....................   1       0    EGG
    .....................................................................cuacuuugagucgguacgaau..................   7       0    EGG
    .....................................................................cuacuuugagucgguacgaauc.................   10      0    EGG
    .....................................................................cuacuuugagucgguacgaUuca................   1       1    EGG
    .....................................................................Uuacuuugagucgguacgaauca................   1       1    EGG
    .....................................................................cuacuuugagucgguacgaauca................   217     0    EGG
    .....................................................................Guacuuugagucgguacgaauca................   1       1    EGG
    .....................................................................cuacuuugGgucgguacgaauca................   1       1    EGG
    .....................................................................cuacuuugagucgguacgGaucac...............   1       1    EGG
    .....................................................................cuacuuugagucgguacgaaucac...............   181     0    EGG
    .....................................................................Uuacuuugagucgguacgaaucac...............   4       1    EGG
    .....................................................................cuacuuugagucgAuacgaaucac...............   1       1    EGG
    .....................................................................cuacuuugagucgguacgaaucacA..............   2       1    EGG
    .....................................................................cuacuuugagucgguacgaaucacc..............   1       0    EGG
    ......................................................................uacuuugagucgguacga....................   3       0    EGG
    ......................................................................uacuuugagucgguacgaa...................   5       0    EGG
    ......................................................................uacuuugagucgguGcgaau..................   1       1    EGG
    ......................................................................uacCuugagucgguacgaau..................   1       1    EGG
    ......................................................................uacuuugagucgguacgaaA..................   2       1    EGG
    ......................................................................uacuuugagucgguacgaau..................   170     0    EGG
    ......................................................................uacCuugagucgguacgaauc.................   1       1    EGG
    ......................................................................uacuCugagucgguacgaauc.................   1       1    EGG
```

Star **Mature**

```
ggaucaaaauaacuuaacgugguucaggggcgguuuguaccgacucagagcuagguucguugaugagaacuacuuugagucgguacgaaucaccucagccaugaucca
......................................................................uacuuugagucgguacgaauG.................    2     1    EGG
......................................................................uacuuugagucgguacgaauA.................    1     1    EGG
......................................................................uacuuugagucgguGcgaauc.................    2     1    EGG
......................................................................uacuuugagucgguacgaauc.................    631   0    EGG
......................................................................uGcuuugagucgguacgaauca................    1     1    EGG
......................................................................uacuuugagucgguacAaauca................    1     1    EGG
......................................................................uacuuugagucggCacgaauca................    1     1    EGG
......................................................................uCcuuugagucgguacgaauca................    1     1    EGG
......................................................................uacuuugagucgguaUgaauca................    1     1    EGG
......................................................................uacuuugagucgguacgGauca................    1     1    EGG
......................................................................uacuuugagucgguacgaUuca................    1     1    EGG
......................................................................uacuuugagucgguacgaCuca................    1     1    EGG
......................................................................uacuuugagucgguaGgaauca................    2     1    EGG
......................................................................uacuuugagucgguacgCauca................    1     1    EGG
......................................................................uacuuugagucgguacgaaGca................    2     1    EGG
......................................................................uacuuugagucgguGcgaauca................    6     1    EGG
......................................................................uacuuugagucgguacgaauGa................    1     1    EGG
......................................................................uacuuCgagucgguacgaauca................    2     1    EGG
......................................................................uacuuugagucgguacgaaucU................    16    1    EGG
......................................................................uaUuuugagucgguacgaauca................    4     1    EGG
......................................................................uacCuugagucgguacgaauca................    5     1    EGG
......................................................................uacuuugUgucgguacgaauca................    3     1    EGG
......................................................................uacuuuUagucgguacgaauca................    1     1    EGG
......................................................................uacuuugagucgguUcgaauca................    1     1    EGG
......................................................................uacuuugagucgguacgaauUa................    1     1    EGG
......................................................................uacuuugagucgguacgaGuca................    2     1    EGG
......................................................................uacuuugGgucgguacgaauca................    3     1    EGG
......................................................................uacAuugagucgguacgaauca................    2     1    EGG
......................................................................uacuuugagucgAuacgaauca................    1     1    EGG
......................................................................uacuuugagucgguacgaauca................    6935  0    EGG
......................................................................uacuuGgagucgguacgaauca................    1     1    EGG
......................................................................uacuuugagucggAacgaaucac...............    1     1    EGG
......................................................................uacuuugagucgguacgaCucac...............    2     1    EGG
......................................................................uacuuGgagucgguacgaaucac...............    4     1    EGG
......................................................................uacuuugagucgUuacgaaucac...............    2     1    EGG
......................................................................uacuuugaCucgguacgaaucac...............    1     1    EGG
......................................................................uacuuugGgucgguacgaaucac...............    2     1    EGG
......................................................................uacuuugagucgguacAaaucac...............    4     1    EGG
......................................................................uacuuugagucgguacgUaucac...............    2     1    EGG
......................................................................uacuuugagucgguacgaUucac...............    1     1    EGG
......................................................................uacuuugUgucgguacgaaucac...............    3     1    EGG
......................................................................uacGuugagucgguacgaaucac...............    1     1    EGG
......................................................................Cacuuugagucgguacgaaucac...............    1     1    EGG
......................................................................uacuCugagucgguacgaaucac...............    1     1    EGG
......................................................................uacuuugagucgguacgaaAcac...............    2     1    EGG
......................................................................uacuuugagucgAuacgaaucac...............    1     1    EGG
......................................................................uacuuugCgucgguacgaaucac...............    2     1    EGG
......................................................................uacuuugagucgguaUgaaucac...............    2     1    EGG
......................................................................uacCuugagucgguacgaaucac...............    4     1    EGG
......................................................................uacuuugagucgguacgGaucac...............    1     1    EGG
......................................................................uacuuugagucgguacgaaucaA...............    66    1    EGG
......................................................................uacuuugagucgCuacgaaucac...............    1     1    EGG
......................................................................uacuuugagucgguacgaaucUc...............    1     1    EGG
......................................................................uacuuugagucgguacgaaucGc...............    2     1    EGG
......................................................................uacuuugagucgguGcgaaucac...............    13    1    EGG
......................................................................uacuuugagucgguaGgaaucac...............    1     1    EGG
......................................................................uacuuugagucgguacgCaucac...............    1     1    EGG
......................................................................uacuuugagucgguacgaaucac...............    6219  0    EGG
......................................................................uacuuugagucgguacgaGucac...............    4     1    EGG
......................................................................uacuuugagucgguacgaaucacA..............    125   1    EGG
......................................................................uacuuugagucgguacgaaucacc..............    9     0    EGG
......................................................................uacuuugagucgguacgaaucaccA.............    4     1    EGG
.......................................................................acuuugagucgguacgaau..................    2     0    EGG
.......................................................................acuuugagucgguacgaauca................    14    0    EGG
.......................................................................acuuugagCcgguacgaaucac...............    1     1    EGG
.......................................................................acuuugagucgguacgaaucac...............    44    0    EGG
........................................................................cuuugagucgguacgaauca................    1     0    EGG
........................................................................cuuugagucgguacgaaucac...............    2     0    EGG
........................................................................cuuugagucgguacgaaucacc..............    1     0    EGG
.........................................................................uuugagucgguacgaauca................    2     0    EGG
```

Star Mature

```
ggaucaaaauaacuuaacgugguucaggggcgguuuguaccgacucagagcuagguucguugaugagaacuacuuugagucgguacgaaucaccucagccaugaucca
.........................................................................uuugagucgguacgaaucac...............	3	0	EGG
.........................................................................uuugagucgguacgaaucacc..............	1	0	EGG
.........................................................................uuugagucgguacgaaucaccA.............	3	1	EGG
..........................................................................uugagucgguacgaaucac...............	3	0	EGG
...........................................................................ugagucgguacgaaucac...............	1	0	EGG

.........uaacuuaacgugguucaggggc.............................................................................	1	0	1st
...............................gguuugCaccgacucagagcu........................................................	1	1	1st
...............................gguuuguaccgacucagagcu........................................................	6	0	1st
...............................gguuuguaccgacucagagcua.......................................................	1	0	1st
...............................gguuuguaccgacucagagcuag......................................................	1	0	1st
...............................gguuuguaccgacucagagcuagg.....................................................	4	0	1st
...............................gguuuguaccgacucagagcuaggu....................................................	16	0	1st
...............................gguuuguaccgacucagagcuagguuU..................................................	2	1	1st
................................guuuguaccgacucaCagcua.......................................................	1	1	1st
................................guuuguaccgacucagagcuaggA....................................................	1	1	1st
................................guuuguaccgacucagagcuaggu....................................................	1	0	1st
................................guuuguaccgacucagagcuagguu...................................................	1	0	1st
.................................uuuguaccgacucagagc.........................................................	1	0	1st
.................................uuuguaccgacucagagcu........................................................	1	0	1st
.................................uuuguaccgacucagagcuU.......................................................	1	1	1st
.................................uuuguaccgacucagagcua.......................................................	7	0	1st
.................................uuuguaccgacucagagcuag......................................................	14	0	1st
.................................uuuguaccgacucagagcuagg.....................................................	9	0	1st
.................................uuuguaccgacucagagcAagg.....................................................	1	1	1st
.................................uuuguaccgacucagagcuaggu....................................................	42	0	1st
.................................uuuguaccgacucagagcuaggA....................................................	2	1	1st
.................................uuuguaccgacucagagcuagguu...................................................	7	0	1st
.................................uuuguaccgacucagagcuagguuU..................................................	2	1	1st
..................................uuguaccgacucagagcuagguu...................................................	5	0	1st
..................................uuguaccgacucagagcuagguuc..................................................	6	0	1st
...................................uguaccgacucagagcuaggu....................................................	1	0	1st
...................................uguaccgacucagagcuagguu...................................................	6	0	1st
...................................uguaccgacucagagcuagguuA..................................................	2	1	1st
...................................uguaccgacucagagcuagguuc..................................................	6	0	1st
...................................uguaccgacucagagcuagguucg.................................................	1	0	1st
.................................................................agaacuacuuugagucgguacg.....................	1	0	1st
..................................................................gaaGuacuuugagucgguacgaauca................	1	1	1st
...................................................................aacuacuuugagucgguacga....................	1	0	1st
....................................................................Ucuacuuugagucgguacgaaucac...............	1	1	1st
....................................................................acuacuuugagucgguacgaaucac...............	1	0	1st
.....................................................................cuacuuugagucgguacg.....................	14	0	1st
.....................................................................cuacuuugagucgguacga....................	2	0	1st
.....................................................................cuacuuugGgucgguacgaa...................	1	1	1st
.....................................................................Uuacuuugagucgguacgaa...................	1	1	1st
.....................................................................cuacuuugagucgguacgaa...................	10	0	1st
.....................................................................cAacuuugagucgguacgaau..................	1	1	1st
.....................................................................cuacuuugagucgguacgaau..................	204	0	1st
.....................................................................cuacuuugagucgguGcgaau..................	2	1	1st
.....................................................................cuacuuugagucggCacgaau..................	1	1	1st
.....................................................................cuacuuugagucgguacgaaA..................	1	1	1st
.....................................................................Uuacuuugagucgguacgaau..................	4	1	1st
.....................................................................cuacuuugagucgguacgaUu..................	1	1	1st
.....................................................................cuacuuugaCucgguacgaau..................	1	1	1st
.....................................................................cuacuuugagucgguacgaauA.................	1	1	1st
.....................................................................cuacuuugagucgguacgaauc.................	171	0	1st
.....................................................................cuacuuugagucgguacgaauU.................	1	1	1st
.....................................................................cuacuuugagucgUuacgaauc.................	1	1	1st
.....................................................................Guacuuugagucgguacgaauc.................	4	1	1st
.....................................................................cuacuuugUgucgguacgaauc.................	1	1	1st
.....................................................................cuacuuugagucgguacgaUuc.................	1	1	1st
.....................................................................cuacuuugagucgguGcgaauc.................	1	1	1st
.....................................................................cuacuuugagucgguacgaUuca................	13	1	1st
.....................................................................cuacuuugGgucgguacgaauca................	1	1	1st
.....................................................................cuacuuugagucggAacgaauca................	1	1	1st
.....................................................................cuacuuugagGcgguacgaauca................	1	1	1st
.....................................................................cuacuuugagucgguacCaauca................	1	1	1st
.....................................................................cuacuuugagucgguaUgaauca................	1	1	1st
.....................................................................cuUcuuugagucgguacgaauca................	1	1	1st
.....................................................................cuacuuugUgucgguacgaauca................	2	1	1st
```

	Star / Mature			
	ggaucaaaauaacuuaacgugguucaggggcgguuuguaccgacucagagcuagguucguugaugagaacuacuuugagucgguacgaaucaccucagccaugaucca			
	....cuacuuugagucgguacgaGuca................	1	1	1st
	....Auacuuugagucgguacgaauca................	1	1	1st
	....cuacuuugagucgUuacgaauca................	1	1	1st
	....Uuacuuugagucgguacgaauca................	26	1	1st
	....cuacuuugagucgguacgUauca................	1	1	1st
	....cuacuCugagucgguacgaauca................	1	1	1st
	....cuacuuugagucgguacgaauca................	3031	0	1st
	....Guacuuugagucgguacgaauca................	1	1	1st
	....cuacuuugagucgguGcgaauca................	3	1	1st
	....cuacuuugagucgguacgGauca................	1	1	1st
	....cuacuuugagucgAuacgaauca................	2	1	1st
	....cuacuuugagucgguacgaaucU................	5	1	1st
	....cuacuuugagCcgguacgaauca................	1	1	1st
	....cuaUuuugagucgguacgaauca................	1	1	1st
	....cuacuuugCgucgguacgaauca................	2	1	1st
	....cuacuuugagucgguacgUaucac...............	1	1	1st
	....cuacuuugagucgguacgaaCcac...............	1	1	1st
	....cAacuuugagucgguacgaaucac...............	1	1	1st
	....cuacuuugagucgAuacgaaucac...............	1	1	1st
	....cuacuuugagucgguacgGaucac...............	1	1	1st
	....cuacuuugagucgguacgaaucac...............	2900	0	1st
	....cuacuuugagucggAacgaaucac...............	1	1	1st
	....cuGcuuugagucgguacgaaucac...............	3	1	1st
	....cuacuuugagucUguacgaaucac...............	1	1	1st
	....cuacuuugagucgguacAaaucac...............	3	1	1st
	....cuacuuugagucgguacgaaucGc...............	2	1	1st
	....Uuacuuugagucgguacgaaucac...............	29	1	1st
	....cCacuuugagucgguacgaaucac...............	1	1	1st
	....cuacuuugagucgCuacgaaucac...............	1	1	1st
	....cuacuuugagucgguacgaUucac...............	9	1	1st
	....cuacuuugagucgguacgaauUac...............	2	1	1st
	....cuacuuugagucgguacCaaucac...............	2	1	1st
	....cuacuuCgagucgguacgaaucac...............	1	1	1st
	....cuacuuugUgucgguacgaaucac...............	6	1	1st
	....cuaUuuugagucgguacgaaucac...............	2	1	1st
	....cuacuuugagucAguacgaaucac...............	1	1	1st
	....Guacuuugagucgguacgaaucac...............	1	1	1st
	....cuacuuugagucgguGcgaaucac...............	8	1	1st
	....cuacuuugGgucgguacgaaucac...............	1	1	1st
	....cuacuuugagucgguacgaaucaA...............	24	1	1st
	....cuacuuugagucgguaUgaaucac...............	2	1	1st
	....cuacuuugagucgguacgaaucacA..............	9	1	1st
	....cuacuuugagucgguacgaaucacc..............	2	0	1st
	....uacuuugagucgguacga....................	447	0	1st
	....uacuuugaCucgguacga....................	1	1	1st
	....uacuuugagucgguGcga....................	2	1	1st
	....uacuuugagucgguacAa....................	1	1	1st
	....uacuuugagucgguacgaU...................	2	1	1st
	....uacuuugagucgguacgaa...................	354	0	1st
	....uacuuugagucgUuacgaa...................	1	1	1st
	....uacuGugagucgguacgaau..................	1	1	1st
	....uacuuugagucgguaGgaau..................	1	1	1st
	....uacuuugagAcgguacgaau..................	1	1	1st
	....uacuuugCgucgguacgaau..................	8	1	1st
	....uacuuugagucgguacAaau..................	2	1	1st
	....Gacuuugagucgguacgaau..................	4	1	1st
	....uacuuGgagucgguacgaau..................	1	1	1st
	....uacuuugGgucgguacgaau..................	3	1	1st
	....uacuuugagucgguacgaCu..................	2	1	1st
	....uacuuugaguAgguacgaau..................	4	1	1st
	....uacuuugagucgAuacgaau..................	1	1	1st
	....uacuuugaCucgguacgaau..................	1	1	1st
	....uacuCugagucgguacgaau..................	1	1	1st
	....uacuuugagCcgguacgaau..................	1	1	1st
	....uacuuCgagucgguacgaau..................	3	1	1st
	....uaGuuugagucgguacgaau..................	3	1	1st
	....uacCuugagucgguacgaau..................	1	1	1st
	....uacuuugagucggCacgaau..................	2	1	1st
	....uacuuugagucgguacgaau..................	11386	0	1st
	....uacuuugagucgguUcgaau..................	4	1	1st

Star   Mature

```
ggaucaaaauaacuuaacgugguucaggggcgguuuguaccgacucagagcuagguucguugaugagaacuacuuugagucgguacgaaucaccucagccaugaucca
......................................................................uacuuugagucgguacgUau..................   1       1    1st
......................................................................uacuuugUgucgguacgaau..................   9       1    1st
......................................................................uacuuugagucgUuacgaau..................   2       1    1st
......................................................................uacuuugagucgguacgaUu..................   1       1    1st
......................................................................uacuuugagucgguacgaGu..................   1       1    1st
......................................................................Cacuuugagucgguacgaau..................   3       1    1st
......................................................................uacuuugagucgguacgaaC..................   4       1    1st
......................................................................uaUuuugagucgguacgaau..................   1       1    1st
......................................................................uacuuuUagucgguacgaau..................   1       1    1st
......................................................................uacuuuCagucgguacgaau..................   1       1    1st
......................................................................uacuuugagucgguacgaaG..................   5       1    1st
......................................................................uacuuugagucgguaUgaau..................   4       1    1st
......................................................................uacuuugagucgguacCaau..................   1       1    1st
......................................................................uacuuugaAucgguacgaau..................   1       1    1st
......................................................................uacuuugagucgguCcgaau..................   1       1    1st
......................................................................uacuuugagucgguacgaaA..................   161     1    1st
......................................................................uacuuugagucgguGcgaau..................   23      1    1st
......................................................................uacuuugagucUguacgaau..................   3       1    1st
......................................................................uacuuugagucgguacAaauc.................   3       1    1st
......................................................................uacuuugagucgguacgCauc.................   2       1    1st
......................................................................uacGuugagucgguacgaauc.................   2       1    1st
......................................................................uacCuugagucgguacgaauc.................   10      1    1st
......................................................................uacuuugaAucgguacgaauc.................   1       1    1st
......................................................................uacuuugagucgguaAgaauc.................   4       1    1st
......................................................................uGcuuugagucgguacgaauc.................   1       1    1st
......................................................................uacuuugagucgUuacgaauc.................   4       1    1st
......................................................................uacuuugagucgguGcgaauc.................   26      1    1st
......................................................................uaUuuugagucgguacgaauc.................   4       1    1st
......................................................................uacuuugagCcgguacgaauc.................   1       1    1st
......................................................................uacuuugCgucgguacgaauc.................   9       1    1st
......................................................................uacuuugagucUguacgaauc.................   1       1    1st
......................................................................uacuuugagucgguCcgaauc.................   1       1    1st
......................................................................uacuuCgagucgguacgaauc.................   6       1    1st
......................................................................uacuuugagucggAacgaauc.................   3       1    1st
......................................................................uCcuuugagucgguacgaauc.................   1       1    1st
......................................................................uacuuugagucgguacgaauG.................   8       1    1st
......................................................................uacuuugagucCguacgaauc.................   1       1    1st
......................................................................uacuuugagucgguacgaauA.................   187     1    1st
......................................................................uacuuugagucgguacgaGuc.................   2       1    1st
......................................................................uacuuugagucgguacgaauc.................   23802   0    1st
......................................................................uacuuuCagucgguacgaauc.................   1       1    1st
......................................................................uacuuugGgucgguacgaauc.................   12      1    1st
......................................................................uacuuugagucgguacgaCuc.................   2       1    1st
......................................................................uacuuugaguGgguacgaauc.................   4       1    1st
......................................................................uUcuuugagucgguacgaauc.................   2       1    1st
......................................................................uacuuugagucgAuacgaauc.................   4       1    1st
......................................................................uacuuugagAcgguacgaauc.................   1       1    1st
......................................................................uaGuuugagucgguacgaauc.................   1       1    1st
......................................................................uaAuuugagucgguacgaauc.................   1       1    1st
......................................................................Gacuuugagucgguacgaauc.................   5       1    1st
......................................................................uacuuugagucgguacgUauc.................   6       1    1st
......................................................................uacuuugagucgguacgaauU.................   44      1    1st
......................................................................uacuuugagucgguaUgaauc.................   10      1    1st
......................................................................Aacuuugagucgguacgaauc.................   2       1    1st
......................................................................uacuGugagucgguacgaauc.................   1       1    1st
......................................................................uacuuugagucgguacCaauc.................   2       1    1st
......................................................................Cacuuugagucgguacgaauc.................   3       1    1st
......................................................................uacuuugaguAgguacgaauc.................   1       1    1st
......................................................................uacuuuUagucgguacgaauc.................   6       1    1st
......................................................................uacuuugagucgguaGgaauc.................   3       1    1st
......................................................................uacuuugagucgCuacgaauc.................   5       1    1st
......................................................................uacuuugagucgguUcgaauc.................   10      1    1st
......................................................................uacuuugagucAguacgaauc.................   1       1    1st
......................................................................uacuuugagucggCacgaauc.................   3       1    1st
......................................................................uacuuugagucgguacgaaCc.................   12      1    1st
......................................................................uacuuugagucgguacgaaAc.................   1       1    1st
......................................................................uacuuugaCucgguacgaauc.................   4       1    1st
......................................................................uacuuugUgucgguacgaauc.................   18      1    1st
......................................................................uacuuugagucgguacgaUuc.................   2       1    1st
......................................................................uacuuugagucgguacgGauc.................   7       1    1st
```

Star Mature

```
ggaucaaaauaacuuaacgugguucaggggcgguuuguaccgacucagagcuagguucguugaugagaacuacuuugagucgguacgaaucaccucagccaugaucca
......................................................................uacuuugagucgguacgaaGc.................   5       1    1st
......................................................................uGcuuugagucgguacgaauca................   22      1    1st
......................................................................uacGuugagucgguacgaauca................   2       1    1st
......................................................................uacuuugUgucgguacgaauca................   106     1    1st
......................................................................uacuGugagucgguacgaauca................   6       1    1st
......................................................................uacuuugaUucgguacgaauca................   6       1    1st
......................................................................uacuuugagucgguacgaCuca................   24      1    1st
......................................................................uacuuAgagucgguacgaauca................   9       1    1st
......................................................................uacuuugagucgguacgaaAca................   6       1    1st
......................................................................uUcuuugagucgguacgaauca................   3       1    1st
......................................................................uacuuugaguGgguacgaauca................   19      1    1st
......................................................................uacuuCgagucgguacgaauca................   23      1    1st
......................................................................uacuuugagucUguacgaauca................   7       1    1st
......................................................................uacuuugagucgguacgaaGca................   32      1    1st
......................................................................uaGuuugagucgguacgaauca................   19      1    1st
......................................................................uacuuugagucgguaGgaauca................   11      1    1st
......................................................................uacuuGgagucgguacgaauca................   9       1    1st
......................................................................uacuuugCgucgguacgaauca................   59      1    1st
......................................................................uacuuugagucgguacgaaucU................   573     1    1st
......................................................................uaAuuugagucgguacgaauca................   17      1    1st
......................................................................uacuuugagucgguacgaauca................   156855  0    1st
......................................................................uacuuugagucgguacgaauGa................   4       1    1st
......................................................................uacuuugagCcgguacgaauca................   4       1    1st
......................................................................uacuuugagAcgguacgaauca................   2       1    1st
......................................................................uacuCugagucgguacgaauca................   10      1    1st
......................................................................uacuuugagucgguacgaaucC................   7       1    1st
......................................................................uacCuugagucgguacgaauca................   68      1    1st
......................................................................uacuuugagucgCuacgaauca................   24      1    1st
......................................................................uacuuugagGcgguacgaauca................   2       1    1st
......................................................................uacuuugagucgguacgaUuca................   11      1    1st
......................................................................uacuuugagucCguacgaauca................   6       1    1st
......................................................................uaUuuugagucgguacgaauca................   40      1    1st
......................................................................uacuuugagucgguGcgaauca................   225     1    1st
......................................................................uacuuugagucgguaAgaauca................   21      1    1st
......................................................................uacuuugagucgAuacgaauca................   24      1    1st
......................................................................uacuuugagucgguacAaauca................   21      1    1st
......................................................................uacuuugagucggCacgaauca................   25      1    1st
......................................................................uacuuugagucgguacgaauUa................   18      1    1st
......................................................................uacuuugagucgguacgaauAa................   28      1    1st
......................................................................uacuuugagucgguacgaaucG................   60      1    1st
......................................................................uacuuugagucggGacgaauca................   5       1    1st
......................................................................uacuuugagucAguacgaauca................   17      1    1st
......................................................................uacuuugGgucgguacgaauca................   113     1    1st
......................................................................uCcuuugagucgguacgaauca................   1       1    1st
......................................................................uacAuugagucgguacgaauca................   1       1    1st
......................................................................uacuuugagucgguacgaGuca................   69      1    1st
......................................................................uacuAugagucgguacgaauca................   1       1    1st
......................................................................uacuuugagucgUuacgaauca................   16      1    1st
......................................................................uacuuugagucgguacgGauca................   37      1    1st
......................................................................uacuuuCagucgguacgaauca................   13      1    1st
......................................................................uacuuugagucgguacgCauca................   14      1    1st
......................................................................uacuuugagucgguacCaauca................   18      1    1st
......................................................................uacuuuUagucgguacgaauca................   40      1    1st
......................................................................uacuuugagucgguacgaaCca................   29      1    1st
......................................................................uacuuugagucgguaUgaauca................   51      1    1st
......................................................................uacuuugagucggAacgaauca................   5       1    1st
......................................................................uacuuugagucgguacUaauca................   8       1    1st
......................................................................Gacuuugagucgguacgaauca................   29      1    1st
......................................................................uacuuuAagucgguacgaauca................   2       1    1st
......................................................................uacuuugagucgguacgUauca................   17      1    1st
......................................................................uacuuugagucgguCcgaauca................   8       1    1st
......................................................................uacuuugaAucgguacgaauca................   3       1    1st
......................................................................Cacuuugagucgguacgaauca................   24      1    1st
......................................................................uacuuugaCucgguacgaauca................   10      1    1st
......................................................................uacuuugagucgguUcgaauca................   49      1    1st
......................................................................Aacuuugagucgguacgaauca................   13      1    1st
......................................................................uacuuugaguAgguacgaauca................   25      1    1st
......................................................................uacuuugagucgguacgaaucGc...............   8       1    1st
......................................................................uacuuugGgucgguacgaaucac..............   67      1    1st
......................................................................uacuuugagucgguacgaaucac..............   96893   0    1st
```

```
                                Star                                Mature
ggaucaaaauaacuuaacgugguucaggggcgguuuguaccgacucagagcuagguucguugaugagaacuacuuugagucgguacgaaucaccucagccaugaucca
......................................................................uacuuugagucgguacgaaucCc...............  2     1   1st
......................................................................Aacuuugagucgguacgaaucac...............  6     1   1st
......................................................................uacGuugagucgguacgaaucac...............  1     1   1st
......................................................................uacuuugagucggCacgaaucac...............  7     1   1st
......................................................................uacuuugagucgguacgaaucaG...............  28    1   1st
......................................................................uacuuugaAucgguacgaaucac...............  1     1   1st
......................................................................uacuuugaguAgguacgaaucac...............  8     1   1st
......................................................................uacuuugagucgguacgaauAac...............  4     1   1st
......................................................................uacuuugUgucgguacgaaucac...............  50    1   1st
......................................................................uacuuugagucgguaUgaaucac...............  22    1   1st
......................................................................uacuuugagucgUuacgaaucac...............  11    1   1st
......................................................................uaAuuugagucgguacgaaucac...............  5     1   1st
......................................................................uacuuugagucgguacgUaucac...............  8     1   1st
......................................................................uacuuugagucgguacgGaucac...............  22    1   1st
......................................................................uacuuugagAcgguacgaaucac...............  1     1   1st
......................................................................uacuuugagucgguUcgaaucac...............  30    1   1st
......................................................................uacuuugagucgguacgaCucac...............  19    1   1st
......................................................................uacuuugagucgguacCaaucac...............  17    1   1st
......................................................................uacuuugagucgguaGgaaucac...............  10    1   1st
......................................................................uacuuugagucgAuacgaaucac...............  9     1   1st
......................................................................uacuuugagucgguGcgaaucac...............  163   1   1st
......................................................................uacuuugagucCguacgaaucac...............  8     1   1st
......................................................................uacuuugagucggAacgaaucac...............  3     1   1st
......................................................................uacuuugagucgguacgCaucac...............  7     1   1st
......................................................................uacuuugagucgguacgaaCcac...............  16    1   1st
......................................................................uacuuugagucgguacgaaAcac...............  11    1   1st
......................................................................uacuuugagucgguacgaaGcac...............  18    1   1st
......................................................................uacuuugagucgguCcgaaucac...............  2     1   1st
......................................................................uaGuuugagucgguacgaaucac...............  17    1   1st
......................................................................uacuuugagucUguacgaaucac...............  7     1   1st
......................................................................uacuuugagucgguacgaaucaA...............  2996  1   1st
......................................................................uGcuuugagucgguacgaaucac...............  14    1   1st
......................................................................uacuuuCagucgguacgaaucac...............  9     1   1st
......................................................................Cacuuugagucgguacgaaucac...............  11    1   1st
......................................................................uacuuugagucgguaAgaaucac...............  20    1   1st
......................................................................uUcuuugagucgguacgaaucac...............  1     1   1st
......................................................................uacuuCgagucgguacgaaucac...............  15    1   1st
......................................................................uacuuugagucAguacgaaucac...............  8     1   1st
......................................................................uacuuugaCucgguacgaaucac...............  11    1   1st
......................................................................uacuuugagucgguacgaauUac...............  15    1   1st
......................................................................uacuuugagucgguacAaaucac...............  10    1   1st
......................................................................uacuuugagucggGacgaaucac...............  2     1   1st
......................................................................uacuuugagucgguacgaGucac...............  36    1   1st
......................................................................uacuuugagucgguacgaUucac...............  8     1   1st
......................................................................uacuGugagucgguacgaaucac...............  4     1   1st
......................................................................uacuuugagucgCuacgaaucac...............  11    1   1st
......................................................................uacuuugagGcgguacgaaucac...............  1     1   1st
......................................................................uacCuugagucgguacgaaucac...............  44    1   1st
......................................................................uacuuugaUucgguacgaaucac...............  6     1   1st
......................................................................uacuuugCgucgguacgaaucac...............  44    1   1st
......................................................................uacuuuAagucgguacgaaucac...............  3     1   1st
......................................................................uCcuuugagucgguacgaaucac...............  1     1   1st
......................................................................uacuuugagCcgguacgaaucac...............  5     1   1st
......................................................................uacuuAgagucgguacgaaucac...............  7     1   1st
......................................................................uacuuugaguGgguacgaaucac...............  8     1   1st
......................................................................uacuuugagucgguacgaaucUc...............  1     1   1st
......................................................................uacuuugagucgguacUaaucac...............  3     1   1st
......................................................................uaUuuugagucgguacgaaucac...............  19    1   1st
......................................................................uacuuGgagucgguacgaaucac...............  5     1   1st
......................................................................uacuCugagucgguacgaaucac...............  7     1   1st
......................................................................uacuuuUagucgguacgaaucac...............  22    1   1st
......................................................................Gacuuugagucgguacgaaucac...............  20    1   1st
......................................................................uacuuugagucgguacgaaucacG..............  1     1   1st
......................................................................uacuuugagucgguacgaaucacA..............  2697  1   1st
......................................................................uacuuugagucgguacgaaucaUc..............  3     1   1st
......................................................................uacuuugagucgguacgaaucacc..............  43    0   1st
......................................................................uacuuugagucgguacgaaucaAc..............  1     1   1st
......................................................................uacuuugagucgguacgaaucaccu.............  7     0   1st
......................................................................uacuuugagucgguacgaaucacAu.............  10    1   1st
......................................................................uacuuugagucgguacgaaucaccA.............  11    1   1st
```

Star Mature

```
ggaucaaaauaacuuaacgugguucaggggcgguuuguaccgacucagagcuagguucguugaugagaacuacuuugagucgguacgaaucaccucagccaugaucca
......................................................................uacuuugagucgguacgaaucacGu.............	1	1	1st
.......................................................................acuuugagucgguacgaau..................	18	0	1st
.......................................................................acuuugagucgguacgaauc.................	41	0	1st
.......................................................................acuuugGgucgguacgaauc.................	1	1	1st
.......................................................................acAuugagucgguacgaauc.................	1	1	1st
.......................................................................acuuugagucgguacgGauca................	1	1	1st
.......................................................................aUuuugagucgguacgaauca................	1	1	1st
.......................................................................acuuugagucgguacgaauca................	200	0	1st
.......................................................................acCuugagucgguacgaauca................	1	1	1st
.......................................................................acuuugUgucgguacgaauca................	1	1	1st
.......................................................................acuuugagucgguGcgaauca................	1	1	1st
.......................................................................acuuugagucgguacgaaucU................	1	1	1st
.......................................................................Ucuuugagucgguacgaauca................	3	1	1st
.......................................................................acuuugCgucgguacgaaucac...............	1	1	1st
.......................................................................acuuugGgucgguacgaaucac...............	1	1	1st
.......................................................................acuuugagucgguGcgaaucac...............	1	1	1st
.......................................................................acuuugagucgguacgaaucGc...............	2	1	1st
.......................................................................acuuugUgucgguacgaaucac...............	1	1	1st
.......................................................................acuuugagucgguacgaauUac...............	1	1	1st
.......................................................................acuuugagucgguacgaaucaA...............	1	1	1st
.......................................................................acuuugagucgguacgaaucac...............	359	0	1st
.......................................................................acuuugagucgguacgaaucacA..............	14	1	1st
........................................................................cuuugagucgguacgaau..................	3	0	1st
........................................................................cuuugagucgguacgaauc.................	3	0	1st
........................................................................Uuuugagucgguacgaauca................	2	1	1st
........................................................................cuuugagucgguacgaauca................	19	0	1st
........................................................................cuuugagucgguacgaaucac...............	19	0	1st
........................................................................cuuugagucgguacgaaucacc..............	1	0	1st
........................................................................cuuugagucgguacgaaucacA..............	8	1	1st
.........................................................................uuugagucgguacgaauc.................	6	0	1st
.........................................................................uuugagucgguacgaauca................	56	0	1st
.........................................................................uuuCagucgguacgaauca................	1	1	1st
.........................................................................uuugagucgAuacgaaucac...............	1	1	1st
.........................................................................uuugagucgguacgaaucac...............	123	0	1st
.........................................................................uuugagucgguacgUaucac...............	1	1	1st
.........................................................................uuugagucgguacgaaucaA...............	4	1	1st
.........................................................................uuugagucgguacgaaucacc..............	5	0	1st
.........................................................................uuugagucgguacgaaucacA..............	12	1	1st
.........................................................................uuugagucgguacgaaucaccA.............	7	1	1st
.........................................................................uuugagucgguacgaaucaccu.............	3	0	1st
..........................................................................uugagucgguacgaauca................	61	0	1st
..........................................................................uugagucgguacgaaucaA...............	1	1	1st
..........................................................................uugagucgguacgaaucac...............	296	0	1st
..........................................................................uugUgucgguacgaaucac...............	1	1	1st
..........................................................................uugagucgguacgaaucacA..............	2	1	1st
..........................................................................uugagucgguacgaaucaccA.............	1	1	1st
...........................................................................ugagucgguacgaaucac...............	8	0	1st

.....................................................................cuacuuugagucgguacgaauc.................	1	0	B01
.....................................................................cuacuuugagucgguacgaauca................	35	0	B01
.....................................................................cuacuuugagucgguacgaaucac...............	32	0	B01
.....................................................................cuacuuugagucgguacgaaucaA...............	1	1	B01
......................................................................uacuuugagucgguacga....................	1	0	B01
......................................................................uacuuugagucgguacgaa...................	1	0	B01
......................................................................uacuuugagucgguacgaau..................	30	0	B01
......................................................................uacuuugagucgguacgaauA.................	1	1	B01
......................................................................uacuuugagucgguacgaauc.................	96	0	B01
......................................................................uacuuugagucUguacgaauca................	1	1	B01
......................................................................uaUuuugagucgguacgaauca................	1	1	B01
......................................................................uacuuugagucgguacgaaucU................	2	1	B01
......................................................................uacuuugCgucgguacgaauca................	1	1	B01
......................................................................uacuuugUgucgguacgaauca................	1	1	B01
......................................................................uacuAugagucgguacgaauca................	1	1	B01
......................................................................uacuuugagucgguacgaauca................	807	0	B01
......................................................................uacuuugGgucgguacgaauca................	1	1	B01
......................................................................uacuuugagCcgguacgaauca................	1	1	B01
......................................................................uUcuuugagucgguacgaauca................	1	1	B01
......................................................................uacuuugagucgguacgUauca................	1	1	B01
......................................................................uacuuugagucgguacUaaucac...............	1	1	B01
......................................................................Gacuuugagucgguacgaaucac...............	1	1	B01
```

```
                                 Star                                 Mature
ggaucaaaauaacuuaacgugguucaggggcgguuuguaccgacucagagcuagguucguugaugagaacuacuuugagucgguacgaaucaccucagccaugaucca
......................................................................uaUuuugagucgguacgaaucac...............    1          1          B01
......................................................................uacuuuCagucgguacgaaucac...............    1          1          B01
......................................................................uacuuugagucgguacgUaucac...............    1          1          B01
......................................................................uacuuugagucgguGcgaaucac...............    3          1          B01
......................................................................uacCuugagucgguacgaaucac...............    1          1          B01
......................................................................uacuuugagucgAuacgaaucac...............    1          1          B01
......................................................................uacuuugagucgguacgaaucaA...............    10         1          B01
......................................................................uacuuugagucgguacgaauUac...............    1          1          B01
......................................................................uUcuuugagucgguacgaaucac...............    1          1          B01
......................................................................uacuAugagucgguacgaaucac...............    1          1          B01
......................................................................uacuuugagucgguacgaaucac...............    1465       0          B01
......................................................................uacuuugagucgguacgaaucacA..............    11         1          B01
.......................................................................acuuugagucgguacgaauca................    1          0          B01
.......................................................................acuuugagucgguacgaaucac...............    12         0          B01
........................................................................cuuugagucgguacgaau..................    1          0          B01
.........................................................................uuugagucgguacgaaucac...............    1          0          B01
.........................................................................uuugagucgguacgaaucacGu.............    2          1          B01
..........................................................................uugagucgguacgaaucacGu.............    1          1          B01

...............................gguuuguaccgacucagagcuaggu....................................................    1          0          TJ1
...............................gguuuguaccgacucagagcuagguu...................................................    1          0          TJ1
.................................uuuguaccgacucagagcu........................................................    1          0          TJ1
.................................uuuguaccgacucagagcuag......................................................    5          0          TJ1
.................................uuuguaccgacucagagcuagg.....................................................    5          0          TJ1
.................................uuuguaccgacucagagcuaggA....................................................    1          1          TJ1
.................................uuuguaccgacucagagcuaggC....................................................    4          1          TJ1
.................................uuuguacAgacucagagcuaggu....................................................    2          1          TJ1
.................................uuuguaccgacucagagcuaggu....................................................    22         0          TJ1
.................................uuuguaccgacucagagcuagguC...................................................    1          1          TJ1
.....................................................................cuacuuugagucgguacg.....................    2          0          TJ1
.....................................................................cuacuuugagucgguacgG....................    2          1          TJ1
.....................................................................cuacuuugagucgguacga....................    4          0          TJ1
.....................................................................cuacuuugGgucgguacgaau..................    1          1          TJ1
.....................................................................cuacuuugagucgguacgaauc.................    14         0          TJ1
.....................................................................cuacuuugagucgguacgaauU.................    1          1          TJ1
.....................................................................cuacuuugagucgguacgaauca................    17         0          TJ1
.....................................................................cuacuuugagucgguacgaaucG................    1          1          TJ1
.....................................................................cuUcuuugagucgguacgaauca................    1          1          TJ1
.....................................................................cuacuuAgagucgguacgaaucac...............    1          1          TJ1
.....................................................................cuacuuugagucgguacgaaucac...............    15         0          TJ1
.....................................................................cuacuuugagucgguacgaaucaA...............    3          1          TJ1
.....................................................................cuacuuugagucgguacgaaucacA..............    1          1          TJ1
.....................................................................cuacuuugagucgguacgaaucacc..............    5          0          TJ1
......................................................................uacuuugagucgguacga....................    30         0          TJ1
......................................................................uacuuugagucgguacgC....................    1          1          TJ1
......................................................................uaAuuugagucgguacga....................    1          1          TJ1
......................................................................uacuuugagucgguacgG....................    1          1          TJ1
......................................................................uacuuugGgucgguacgaa...................    1          1          TJ1
......................................................................uacuuugagucgguacgaa...................    9          0          TJ1
......................................................................uacuuugagucgguacgGa...................    1          1          TJ1
......................................................................Cacuuugagucgguacgaau..................    1          1          TJ1
......................................................................uacuuugagucgguacgaUu..................    2          1          TJ1
......................................................................uacuuugagucgguacgaau..................    156        0          TJ1
......................................................................uacuuugagucgguacgaGu..................    2          1          TJ1
......................................................................uacuuugagAcgguacgaau..................    2          1          TJ1
......................................................................uacuuugagucgguacgGau..................    2          1          TJ1
......................................................................uacuuugagucgguacgaaG..................    2          1          TJ1
......................................................................uacuuugagucgguacgaaA..................    8          1          TJ1
......................................................................uacCuugagucgguacgaau..................    1          1          TJ1
......................................................................uacuuugagucgguacgaaC..................    21         1          TJ1
......................................................................uGcuuugagucgguacgaauc.................    1          1          TJ1
......................................................................uacCuugagucgguacgaauc.................    3          1          TJ1
......................................................................uacuuugagucggCacgaauc.................    1          1          TJ1
......................................................................uacuuugagucgguacgaauc.................    399        0          TJ1
......................................................................uacuCugagucgguacgaauc.................    1          1          TJ1
......................................................................uacuuugagucgguacgaGuc.................    1          1          TJ1
......................................................................uacuuugGgucgguacgaauc.................    1          1          TJ1
......................................................................uacuuugagucgguacgaauU.................    31         1          TJ1
......................................................................uacuuugagucggAacgaauc.................    1          1          TJ1
......................................................................uacuuugagucgguacgGauc.................    1          1          TJ1
```

Star Mature

ggaucaaaauaacuuaacgugguucaggggcgguuuguaccgacucagagcuagguucguugaugagaacuacuuugagucgguacgaaucaccucagccaugaucca			
....Cacuuugagucgguacgaauc.................	1	1	TJ1
....uacuuugagucgguGcgaauc.................	1	1	TJ1
....uacuuugagucgguacgaauA.................	10	1	TJ1
....uacuuugagCcgguacgaauc.................	1	1	TJ1
....uacuuugagucgguacgaaCc.................	3	1	TJ1
....uacuuCgagucgguacgaauc.................	2	1	TJ1
....uacuuAgagucgguacgaauc.................	2	1	TJ1
....uaAuuugagucgguacgaauc.................	1	1	TJ1
....uacuuuAagucgguacgaauc.................	1	1	TJ1
....uacuuugagAcgguacgaauc.................	1	1	TJ1
....uacuuCgagucgguacgaauca................	2	1	TJ1
....uacuuugagucgguGcgaauca................	4	1	TJ1
....uacuuugagucgguacgaauca................	804	0	TJ1
....Cacuuugagucgguacgaauca................	3	1	TJ1
....uacuuugGgucgguacgaauca................	6	1	TJ1
....uacuuugagucgguacgaaucU................	14	1	TJ1
....uacuuugagucgguacgaaucG................	19	1	TJ1
....uacCuugagucgguacgaauca................	9	1	TJ1
....uacuuugagucgguacgaaucC................	2	1	TJ1
....uaAuuugagucgguacgaauca................	3	1	TJ1
....uacuuugagucUguacgaauca................	1	1	TJ1
....uacuuugagucAguacgaauca................	1	1	TJ1
....uacuuugagucgguacgGauca................	4	1	TJ1
....uacuuugagucgguacgaaGca................	1	1	TJ1
....uacuuuAagucgguacgaauca................	1	1	TJ1
....uacuuugagucgguacgaaCca................	5	1	TJ1
....uacuuugagCcgguacgaauca................	2	1	TJ1
....uacuuugagucgguacgaauUa................	2	1	TJ1
....uacuuAgagucgguacgaauca................	1	1	TJ1
....uacuuugagAcgguacgaauca................	3	1	TJ1
....uacuuugUgucgguacgaauca................	1	1	TJ1
....uacuuugagucgguacUaauca................	1	1	TJ1
....uacuuugagucgguacgaauAa................	2	1	TJ1
....Gacuuugagucgguacgaauca................	1	1	TJ1
....uacuCugagucgguacgaauca................	1	1	TJ1
....uacuuugagucgguacgaGuca................	1	1	TJ1
....uacuuugaAucgguacgaauca................	1	1	TJ1
....Aacuuugagucgguacgaauca................	1	1	TJ1
....uacuuugagucgguaAgaauca................	1	1	TJ1
....uacuuugagGcgguacgaauca................	1	1	TJ1
....uacuuugagucgguCcgaauca................	1	1	TJ1
....uacAuugagucgguacgaauca................	1	1	TJ1
....uacuuugagucggCacgaauca................	1	1	TJ1
....uacuuugagucgguaUgaauca................	2	1	TJ1
....uacuuugagucgguacgaaucac...............	97	0	TJ1
....uacuuugagucgguacgaGucac...............	1	1	TJ1
....uGcuuugagucgguacgaaucac...............	1	1	TJ1
....uacuuugagucgguacgaaucaA...............	35	1	TJ1
....uacuuugagucgguacgaaucGc...............	2	1	TJ1
....uacuuGgagucgguacgaaucac...............	1	1	TJ1
....uacuuCgagucgguacgaaucac...............	1	1	TJ1
....uacuuugagucgguacgaaucacG..............	1	1	TJ1
....uacuuugagucgguacgaaucacA..............	6	1	TJ1
....uacuuugagucgguacgaaucacc..............	11	0	TJ1
....uacuuugagAcgguacgaaucaccu.............	1	1	TJ1
....acuuugagucgguacgaauc.................	1	0	TJ1
....cuuugagucgguacgaaucac...............	1	0	TJ1
....uuugagucgguacgaaucacc..............	1	0	TJ1
....uuCgagucgguacgaaucaccu.............	1	1	TJ1
....uuugagucgguacgaaucaccu.............	2	0	TJ1
....uuugagucgguacgaaucaccA.............	3	1	TJ1
....uugagucgguacgaauca................	2	0	TJ1
....uugagucgguacgaaucaA...............	1	1	TJ1
.................................uuuguaccgacucagagcu..	2	0	BJ1
....gaacuacuuugagucgguacgUauca................	1	1	BJ1
....gaacuacuuugagucgguacgaauca................	3	0	BJ1
....cuacuuugagucgguGcgaau..................	1	1	BJ1
....cuacuuugagucgguacgaau..................	5	0	BJ1
....cuacuuugagucgguacgaauc.................	17	0	BJ1

Star Mature

```
ggaucaaaauaacuuaacgugguucaggggcgguuuguaccgacucagagcuagguucguugaugagaacuacuuugagucgguacgaaucaccucagccaugaucca
.....................................................................cuacuuugagCcgguacgaauc.................   1   1   BJ1
.....................................................................cuacuuugagucgguacgaauca................   5   0   BJ1
.....................................................................cCacuuugagucgguacgaauca................   1   1   BJ1
.....................................................................cuacuuugagucgguacgaaucac...............   1   0   BJ1
......................................................................uacuuugagucgguacga....................   6   0   BJ1
......................................................................uacuuugagucgguacgaa...................   1   0   BJ1
......................................................................uacuuugagucgguacgaaA..................   5   1   BJ1
......................................................................uacuuugagucgguacgaaC..................   1   1   BJ1
......................................................................uacuuugagucgguacgaUu..................   1   1   BJ1
......................................................................uacuuugagucgguacgaau..................   20  0   BJ1
......................................................................uacuuugagucgguacgaauA.................   1   1   BJ1
......................................................................uacuuugagucAguacgaauc.................   1   1   BJ1
......................................................................uacuuugagucggAacgaauc.................   1   1   BJ1
......................................................................uacuuugagucgguacgaauc.................   75  0   BJ1
......................................................................uacuuugagucgguacgaauU.................   3   1   BJ1
......................................................................uacuuugagucgguGcgaauc.................   1   1   BJ1
......................................................................uacuuugagucgAuacgaauca................   1   1   BJ1
......................................................................uacuuugagucgguacgaauca................   54  0   BJ1
......................................................................uacuAugagucgguacgaauca................   2   1   BJ1
......................................................................uacuuugagucgguacgaaucG................   1   1   BJ1
......................................................................uacuuugagucgguacgaGuca................   2   1   BJ1
......................................................................uacuuugagucgguacAaaucac...............   1   1   BJ1
......................................................................uacuuugagucgguacgaaCcac...............   1   1   BJ1
......................................................................uacuuugagucgguacgaaucac...............   23  0   BJ1
......................................................................uacuAugagucgguacgaaucac...............   1   1   BJ1
......................................................................uacuuugagucggAacgaaucac...............   1   1   BJ1
......................................................................uacuuugagucgguacgaaucGc...............   1   1   BJ1
.......................................................................acuuugagucgguacgaauc.................   1   0   BJ1
.......................................................................acuuugagucgguacgaauca................   1   0   BJ1
.......................................................................acuuugagucgguacgaaucac...............   2   0   BJ1

...............................gguuuguaccgacucagagcu........................................................   4   0   HA1
...............................gguuuguaccgacucagGgcu........................................................   1   1   HA1
...............................gguuuguaccgacucagagcua.......................................................   1   0   HA1
...............................Aguuuguaccgacucagagcuaggu....................................................   1   1   HA1
...............................gguuuguaccgacucagagcuaggu....................................................   11  0   HA1
...............................gguuuguaccgacucagagcuagguC...................................................   1   1   HA1
...............................gguuuguaccgacucagagcuagguu...................................................   3   0   HA1
.................................uuuguaccgacucagagcu........................................................   3   0   HA1
.................................uuuguaccgacucagagcuag......................................................   1   0   HA1
.................................uuuguaccgacucagagcuagg.....................................................   14  0   HA1
.................................uuuguaccgacucGgagcuagg.....................................................   1   1   HA1
.................................uuuguaccgacucagGgcuaggu....................................................   1   1   HA1
.................................uuuguaccgacucagagcuaggA....................................................   1   1   HA1
.................................uuuguaccgacucagagcuaggu....................................................   42  0   HA1
.................................uuuguaccgacucagagcuaggC....................................................   4   1   HA1
.................................uuuguaccgacucagagcuagguu...................................................   3   0   HA1
..................................uuguaccgacucagagcuagguuc..................................................   1   0   HA1
...................................uguaccgacucagagcuagguu...................................................   2   0   HA1
...................................uguaccgacucagagcuagguuc..................................................   1   0   HA1
...................................uguaccgacucagagcuagguuA..................................................   1   1   HA1
...................................uguaccgacucagagcuagguucC.................................................   1   1   HA1
.....................................................................cuacuuugagucgguacg.....................   2   0   HA1
.....................................................................cuacuuugagucgguacgG....................   2   1   HA1
.....................................................................cuacuuugagucgguacga....................   2   0   HA1
.....................................................................cuacuuugagucgguacgaa...................   6   0   HA1
.....................................................................cuacuuugagucgguacgaau..................   15  0   HA1
.....................................................................cuacuuugagucgguacgaaA..................   3   1   HA1
.....................................................................cuacuuugagucgguacgaaC..................   1   1   HA1
.....................................................................cuacuuugagucgguacgaauc.................   120 0   HA1
.....................................................................cuacuuugagucAguacgaauc.................   1   1   HA1
.....................................................................cuacuuugagucgguacgaaGc.................   1   1   HA1
.....................................................................cuacuuugagucgguacgaauU.................   2   1   HA1
.....................................................................cuacuuugagucgguacAaauc.................   1   1   HA1
.....................................................................cuacuuugagucgguGcgaauc.................   2   1   HA1
.....................................................................cuacuuugagucgguacgGauc.................   1   1   HA1
.....................................................................cuacuuugagucgguGcgaauca................   2   1   HA1
.....................................................................cuacuuugagucggCacgaauca................   2   1   HA1
.....................................................................cuacuuugagucgguacgaauca................   309 0   HA1
.....................................................................cuacuuugagCcgguacgaauca................   1   1   HA1
```

Star Mature

ggaucaaaauaacuuaacgugguucaggggcgguuuguaccgacucagagcuagguucguugaugagaacuacuuugagucgguacgaaucaccucagccaugaucca			
....cuacuuugagucgguacAaauca................	2	1	HA1
....cuacuuugGgucgguacgaauca................	1	1	HA1
....cCacuuugagucgguacgaauca................	1	1	HA1
....cuacuuugagucgguacgaaCca................	4	1	HA1
....cuacuuugaguAgguacgaauca................	1	1	HA1
....cuacuuugagucgguacgaaucC................	1	1	HA1
....cuacuuCgagucgguacgaauca................	2	1	HA1
....cuGcuuugagucgguacgaauca................	1	1	HA1
....cuacuuugagucAguacgaauca................	2	1	HA1
....cuacuuugagucgguacgaaucG................	10	1	HA1
....cuacuuuUagucgguacgaauca................	1	1	HA1
....cuacuCugagucgguacgaauca................	1	1	HA1
....cuacuuugagucgguacgaGuca................	2	1	HA1
....cuacuuugagucgguGcgaaucac...............	3	1	HA1
....cuacuuugagucgguacgaaucac...............	103	0	HA1
....cuacuuugagucgguacgaaucaA...............	10	1	HA1
....cuacuuugaAucgguacgaaucac...............	1	1	HA1
....cuacuuugagucggCacgaaucac...............	1	1	HA1
....cuacuuugagucgguacgaaucGc...............	1	1	HA1
....cuacuuugagucgguacgaaucUc...............	2	1	HA1
....cuacuuugagucgguacgaaucacc..............	5	0	HA1
....cuacuuugagucgguacgaaucacA..............	2	1	HA1
....cuacuuugagucgguacgaaucaccu.............	2	0	HA1
....uacuCugagucgguacga....................	2	1	HA1
....uacuuugagucgguacAa....................	3	1	HA1
....uacuuugagucgguacgG....................	3	1	HA1
....uacuuugagucgguacga....................	84	0	HA1
....uacuuugagucgguaUga....................	1	1	HA1
....uacuuugagucgguacgaa...................	126	0	HA1
....uacuuugagucgguacgaU...................	4	1	HA1
....uacuuugagucgAuacgaa...................	1	1	HA1
....uacuuugagucCguacgaa...................	1	1	HA1
....uacCuugagucgguacgaa...................	2	1	HA1
....uacuuCgagucgguacgaa...................	1	1	HA1
....Aacuuugagucgguacgaau..................	2	1	HA1
....uaAuuugagucgguacgaau..................	2	1	HA1
....uGcuuugagucgguacgaau..................	4	1	HA1
....uacuuugagCcgguacgaau..................	6	1	HA1
....uacuuugagucgguacgaaG..................	22	1	HA1
....uacuuugagucgguacgaaC..................	206	1	HA1
....uacuuGgagucgguacgaau..................	2	1	HA1
....uacuuugGgucgguacgaau..................	11	1	HA1
....uacuuAgagucgguacgaau..................	1	1	HA1
....Gacuuugagucgguacgaau..................	1	1	HA1
....uacuuugagucggCacgaau..................	2	1	HA1
....uacuuugagucgguaUgaau..................	6	1	HA1
....uacuuugagucgAuacgaau..................	3	1	HA1
....uacuuugagucgguaAgaau..................	1	1	HA1
....uacuuugagucgguacAaau..................	2	1	HA1
....uacuCugagucgguacgaau..................	3	1	HA1
....uacuuugagucgguaGgaau..................	3	1	HA1
....uacuuugagucgguacgUau..................	1	1	HA1
....uacuuugagucgguacgaCu..................	2	1	HA1
....uUcuuugagucgguacgaau..................	6	1	HA1
....uacuuugagucgguacgaau..................	2338	0	HA1
....uacuuugUgucgguacgaau..................	1	1	HA1
....uacuuugagucgguacgaaA..................	76	1	HA1
....uacAuugagucgguacgaau..................	1	1	HA1
....uacuuugagucgguGcgaau..................	4	1	HA1
....uacuuugagucgguacgGau..................	10	1	HA1
....uacuuugaAucgguacgaau..................	3	1	HA1
....uacuuugagucAguacgaau..................	6	1	HA1
....uacuuugagAcgguacgaau..................	3	1	HA1
....Cacuuugagucgguacgaau..................	5	1	HA1
....uacuuCgagucgguacgaau..................	4	1	HA1
....uacuuugagucgguacgaUu..................	5	1	HA1
....uacuuugaguGgguacgaau..................	1	1	HA1
....uacCuugagucgguacgaau..................	10	1	HA1
....uacuuugagucgguacgaGu..................	13	1	HA1
....uacuuugaguAgguacgaau..................	2	1	HA1

Star **Mature**

ggaucaaaauaacuuaacgugguucaggggcgguuuguaccgacucagagcuagguucguugaugagaacuacuuugagucgguacgaaucaccucagccaugaucca			
....uacCuugagucgguacgaauc.................	20	1	HA1
....uacuCugagucgguacgaauc.................	23	1	HA1
....uacuuugagucgguacgaauA.................	107	1	HA1
....uacuuugGgucgguacgaauc.................	20	1	HA1
....uacuuugagCcgguacgaauc.................	19	1	HA1
....uacuuugagucgguacgaaAc.................	3	1	HA1
....uacuuugagucgguacgGauc.................	18	1	HA1
....uacuuuAagucgguacgaauc.................	1	1	HA1
....uacuuugagucgguacgaUuc.................	2	1	HA1
....uacuuugagucgCuacgaauc.................	2	1	HA1
....uacuuugagucggCacgaauc.................	10	1	HA1
....uacuuugagucggAacgaauc.................	4	1	HA1
....uacuGugagucgguacgaauc.................	1	1	HA1
....uacuuugagGcgguacgaauc.................	1	1	HA1
....uacuuugaUucgguacgaauc.................	1	1	HA1
....uacuuugagucgguacgaGuc.................	19	1	HA1
....uacuuCgagucgguacgaauc.................	19	1	HA1
....uacuuugaguGgguacgaauc.................	1	1	HA1
....uUcuuugagucgguacgaauc.................	2	1	HA1
....uacuuugagucgguGcgaauc.................	24	1	HA1
....Cacuuugagucgguacgaauc.................	20	1	HA1
....uacuuugagucgguacgaaGc.................	17	1	HA1
....Aacuuugagucgguacgaauc.................	1	1	HA1
....uacuuugagucgguaUgaauc.................	5	1	HA1
....uacuuugagucgguacgaaCc.................	40	1	HA1
....uacuuugagucAguacgaauc.................	13	1	HA1
....uacuuugagucgguUcgaauc.................	1	1	HA1
....uacuuugagucgguacgaauc.................	6385	0	HA1
....uacuuugagucgguacgUauc.................	2	1	HA1
....uaUuuugagucgguacgaauc.................	4	1	HA1
....uacuuugagucgguacgaauG.................	5	1	HA1
....uacuuugagucgguacgaauU.................	329	1	HA1
....uacuuugaguAgguacgaauc.................	4	1	HA1
....uacuuugagAcgguacgaauc.................	1	1	HA1
....uacAuugagucgguacgaauc.................	5	1	HA1
....uacuuugagucgguacAaauc.................	3	1	HA1
....uacuuugagucgAuacgaauc.................	7	1	HA1
....uGcuuugagucgguacgaauc.................	22	1	HA1
....uacuuAgagucgguacgaauc.................	3	1	HA1
....uacuAugagucgguacgaauc.................	4	1	HA1
....uacGuugagucgguacgaauca................	4	1	HA1
....uacuuAgagucgguacgaauca................	7	1	HA1
....uacuuugagucgguacgaaucG................	372	1	HA1
....uacuuugagucgguacgaauAa................	13	1	HA1
....uacuuugagucgguaAgaauca................	2	1	HA1
....uacuuugagucgguacgaaAca................	4	1	HA1
....uacuuugaguGgguacgaauca................	2	1	HA1
....uacuuuAagucgguacgaauca................	6	1	HA1
....uacuuugagucgguacgaaGca................	13	1	HA1
....uGcuuugagucgguacgaauca................	35	1	HA1
....uaUuuugagucgguacgaauca................	20	1	HA1
....Gacuuugagucgguacgaauca................	1	1	HA1
....uacuuugagucgguacgGauca................	51	1	HA1
....uacuuugagucgguacUaauca................	5	1	HA1
....uacuuugagucgguacgaaucU................	281	1	HA1
....Aacuuugagucgguacgaauca................	12	1	HA1
....uUcuuugagucgguacgaauca................	7	1	HA1
....uacuuugagucgguUcgaauca................	1	1	HA1
....Cacuuugagucgguacgaauca................	31	1	HA1
....uacuuugagucgAuacgaauca................	9	1	HA1
....uacuuugaUucgguacgaauca................	2	1	HA1
....uacuuugCgucgguacgaauca................	2	1	HA1
....uacuuugagucgguaUgaauca................	33	1	HA1
....uacuuugagucgguacgaaCca................	40	1	HA1
....uacuuugagucgUuacgaauca................	1	1	HA1
....uacuuugagucggAacgaauca................	6	1	HA1
....uacuuugaAucgguacgaauca................	4	1	HA1
....uacuuugagucgguacCaauca................	5	1	HA1
....uacuuugUgucgguacgaauca................	6	1	HA1
....uacuuugagucgguacgaUuca................	3	1	HA1

```
                               Star                                  Mature
ggaucaaaauaacuuaacgugguucaggggcgguuuguaccgacucagagcuagguucguugaugagaacuacuuugagucgguacgaaucaccucagccaugaucca
......................................................................uaAuuugagucgguacgaauca................   1      1   HA1
......................................................................uacuuugagucUguacgaauca................   5      1   HA1
......................................................................uacuuugagucgguacgUauca................   6      1   HA1
......................................................................uacuAugagucgguacgaauca................   4      1   HA1
......................................................................uacuuCgagucgguacgaauca................   44     1   HA1
......................................................................uacuuugagucggCacgaauca................   24     1   HA1
......................................................................uacuuugagucAguacgaauca................   27     1   HA1
......................................................................uacAuugagucgguacgaauca................   5      1   HA1
......................................................................uacuuugagAcgguacgaauca................   4      1   HA1
......................................................................uacuuugagucgguacgCauca................   2      1   HA1
......................................................................uacuCugagucgguacgaauca................   45     1   HA1
......................................................................uacuuugaguAgguacgaauca................   6      1   HA1
......................................................................uacuuugagucgguacgaauUa................   11     1   HA1
......................................................................uacuuugagucgguacgaauca................   16352  0   HA1
......................................................................uacuuugagucgguacgaGuca................   35     1   HA1
......................................................................uacuuugGgucgguacgaauca................   59     1   HA1
......................................................................uacuuugagucgguacAaauca................   19     1   HA1
......................................................................uacuuugagucgguacgaCuca................   3      1   HA1
......................................................................uCcuuugagucgguacgaauca................   2      1   HA1
......................................................................uacuuugagCcgguacgaauca................   23     1   HA1
......................................................................uacCuugagucgguacgaauca................   57     1   HA1
......................................................................uacuuugagucgguacgaaucC................   35     1   HA1
......................................................................uacuuugagucgguGcgaauca................   30     1   HA1
......................................................................uacuuGgagucgguacgaauca................   4      1   HA1
......................................................................uacuuugagucgguaUgaaucac...............   4      1   HA1
......................................................................uacuuugagucgguacgaaucaA...............   448    1   HA1
......................................................................uCcuuugagucgguacgaaucac...............   1      1   HA1
......................................................................uUcuuugagucgguacgaaucac...............   2      1   HA1
......................................................................uacuuugagucgAuacgaaucac...............   3      1   HA1
......................................................................Cacuuugagucgguacgaaucac...............   3      1   HA1
......................................................................uGcuuugagucgguacgaaucac...............   4      1   HA1
......................................................................uacuuugagucgguacgaGucac...............   3      1   HA1
......................................................................uacuuugagucgguacgaCucac...............   1      1   HA1
......................................................................uacuuugagucgguacgaaucac...............   1357   0   HA1
......................................................................uacuuugagucgguacgaaucGc...............   9      1   HA1
......................................................................uacuuugagucgguaGgaaucac...............   1      1   HA1
......................................................................uacCuugagucgguacgaaucac...............   2      1   HA1
......................................................................uacuAugagucgguacgaaucac...............   1      1   HA1
......................................................................uacuuugagucgguacgGaucac...............   3      1   HA1
......................................................................uacuuugagucgguacgaaCcac...............   3      1   HA1
......................................................................uacuuugagucAguacgaaucac...............   1      1   HA1
......................................................................uacuuugagucgguGcgaaucac...............   8      1   HA1
......................................................................uacuuugagucgguacgaaucaG...............   16     1   HA1
......................................................................uacuuugagucgguacgaaAcac...............   2      1   HA1
......................................................................uacuCugagucgguacgaaucac...............   5      1   HA1
......................................................................uacuuugagucgguacgaaucCc...............   1      1   HA1
......................................................................uacuuugagucgguacAaaucac...............   1      1   HA1
......................................................................uacuuugagucUguacgaaucac...............   2      1   HA1
......................................................................uacuuCgagucgguacgaaucac...............   1      1   HA1
......................................................................uacuuugGgucgguacgaaucac...............   6      1   HA1
......................................................................uacuuugagucgguacgaauUac...............   1      1   HA1
......................................................................uacuuCgagucgguacgaaucacc..............   2      1   HA1
......................................................................uaUuuugagucgguacgaaucacc..............   1      1   HA1
......................................................................uacuuugagucgguacgaaucacA..............   82     1   HA1
......................................................................uacuuugagucgguacgaaucacc..............   78     0   HA1
......................................................................uacuuugagucgguacgaaucacG..............   15     1   HA1
......................................................................uacuuugagucgguGcgaaucacc..............   1      1   HA1
......................................................................uacuuugagucgguacgGaucacc..............   1      1   HA1
......................................................................uacuuugagucgguacgaaucaUc..............   3      1   HA1
......................................................................uacCuugagucgguacgaaucacc..............   1      1   HA1
......................................................................uacuuugaguUgguacgaaucacc..............   2      1   HA1
......................................................................uacuuugagucgguacgaaucaccC.............   1      1   HA1
......................................................................uacuuugagucgguacgaaucacGu.............   1      1   HA1
......................................................................uacuuugagucgguacgaaucaccu.............   3      0   HA1
......................................................................uacuuugagucgguacgaaucCccu.............   1      1   HA1
......................................................................uacuuugagucgguacgaaucaccuU............   1      1   HA1
.......................................................................acuuugagucgguacgaauc..................   2      0   HA1
.......................................................................acuuugagucgguacgaauca.................   7      0   HA1
.......................................................................acuuugagucgguacgaaucac...............   3      0   HA1
........................................................................cuugGgucgguacgaauca................   1      1   HA1
```

Star Mature

ggaucaaaauaacuuaacgugguucaggggcgguuuguaccgacucagagcuagguucguugaugagaacuacuuugagucgguacgaaucaccucagccaugaucca			
....cuuugagucgguacgaGuca................	1	1	HA1
....cuuugagucgguacgaauca................	1	0	HA1
....cuuugagucgguacgaaucacc..............	1	0	HA1
....cuuugagucgguacgaaucaccuU............	1	1	HA1
....uuugagucgguacgaauca................	1	0	HA1
....uuugagucgguacgaaucacc..............	3	0	HA1
....uuugagucgguacgaaucacA..............	1	1	HA1
....uuugagucgguacgaaucaccA.............	2	1	HA1
....uugagucgguacgaauca................	7	0	HA1
....uugagucUguacgaauca................	1	1	HA1
....uugagucgguacgaaucac...............	1	0	HA1
....uugagucgguacgaaucaA...............	1	1	HA1
....cuacuuugagucgguacgaau..................	1	0	LA5
....cuacuuugagucgguGcgaau..................	1	1	LA5
....cuacuuugagucgguacgaauc.................	2	0	LA5
....cuacuuugagucgguacgaauca................	14	0	LA5
....cuacuuugagucgguacgaaucac...............	8	0	LA5
....cuacuuugagucgguacgaaucacc..............	1	0	LA5
....uacuuugagucgguacgG....................	1	1	LA5
....uacuuugagucgguacga....................	5	0	LA5
....uacuuugagucgguacgaa...................	2	0	LA5
....uacuuugagucgguacgaau..................	73	0	LA5
....uacuuugagucgguacgGau..................	1	1	LA5
....uGcuuugagucgguacgaau..................	1	1	LA5
....uacCuugagucgguacgaau..................	5	1	LA5
....uacuuugagucgguacgaaA..................	3	1	LA5
....uacuuugagucgguacgaaC..................	5	1	LA5
....uacuuugagucgguaUgaau..................	3	1	LA5
....uacuuugagucgguacgaauA.................	4	1	LA5
....uacuuugagucgguacgaauc.................	305	0	LA5
....uacuCugagucgguacgaauc.................	1	1	LA5
....uacuuugagucgguacgaaCc.................	1	1	LA5
....uacuuugagucgguacgaauG.................	1	1	LA5
....uaUuuugagucgguacgaauc.................	1	1	LA5
....uacuuugagucgAuacgaauc.................	2	1	LA5
....uacuuugagucgguacgaauU.................	9	1	LA5
....uacuuugagucgguacgUauc.................	1	1	LA5
....uacuuugagCcgguacgaauc.................	1	1	LA5
....uacCuugagucgguacgaauca................	1	1	LA5
....uacuuugagucgguacgaaucG................	10	1	LA5
....uacuuugagucgguacgaauca................	752	0	LA5
....uacuuugagucgguacAaauca................	1	1	LA5
....uacuuugagucgguacgGauca................	5	1	LA5
....uacuuugaguGgguacgaauca................	1	1	LA5
....uacuuugagAcgguacgaauca................	1	1	LA5
....uacuuugagucgguGcgaauca................	4	1	LA5
....uacuuugagucgguacgaauUa................	1	1	LA5
....uacuuugagucggCacgaauca................	2	1	LA5
....uacuuugagucgguacgaaCca................	1	1	LA5
....uacuuugagucgguacgaaucC................	1	1	LA5
....uacuuugGgucgguacgaauca................	2	1	LA5
....uacuuugagucgguacgaaucU................	9	1	LA5
....uacuuugagucgguacgaGuca................	1	1	LA5
....uacuCugagucgguacgaauca................	1	1	LA5
....uacuuugagucgguGcgaaucac...............	2	1	LA5
....uacuuugagucgguacgaaucaA...............	14	1	LA5
....uacuCugagucgguacgaaucac...............	1	1	LA5
....uacuuugagucgguacgaGucac...............	1	1	LA5
....uacuuugagucggCacgaaucac...............	1	1	LA5
....uacuuugagucgguacgGaucac...............	2	1	LA5
....uacuuugUgucgguacgaaucac...............	1	1	LA5
....uacuuugagucgguacgaaucac...............	55	0	LA5
....uacuuugagucgguacgaaucacc..............	3	0	LA5
....uacuuugagucgguacgaaucacA..............	7	1	LA5
....uacuuugagucgguacgaaucaccu.............	1	0	LA5
....acuuugagucgguacgaaucac...............	3	0	LA5
....Ucuacuuugagucgguacgaaucac...............	1	1	H01
....cuacuuugagucgguacga....................	1	0	H01

Star Mature

```
ggaucaaaauaacuuaacgugguucaggggcgguuuguaccgacucagagcuagguucguugaugagaacuacuuugagucgguacgaaucaccucagccaugaucca
.....................................................................cuacuuugagucgguacgaau..................  2     0  H01
.....................................................................cuacuuugagucgguacgaauc.................  4     0  H01
.....................................................................cuacuuugagCcgguacgaauc.................  1     1  H01
.....................................................................cuacuuugagucgguacgaauca................  138   0  H01
.....................................................................cuacuuugUgucgguacgaauca................  1     1  H01
.....................................................................cuacuuugagucgguacCaauca................  1     1  H01
.....................................................................Uuacuuugagucgguacgaauca................  6     1  H01
.....................................................................Guacuuugagucgguacgaauca................  1     1  H01
.....................................................................cuacuuugagucgguacgaaucac...............  70    0  H01
.....................................................................cuacuuugagucgguacgaaucaA...............  3     1  H01
.....................................................................cuacuuugagucgguacgaGucac...............  1     1  H01
.....................................................................Uuacuuugagucgguacgaaucac...............  4     1  H01
......................................................................uacuuugagucgCuacga....................  1     1  H01
......................................................................uacuuugagucgguacga....................  12    0  H01
......................................................................uacuuugagucgguacgaa...................  5     0  H01
......................................................................uacuuugagucgguacgGau..................  1     1  H01
......................................................................uacuuugagucgguacgaaA..................  2     1  H01
......................................................................uacuuugagucgguacgaau..................  307   0  H01
......................................................................uacuuugagucgguacgUauc.................  1     1  H01
......................................................................uaUuuugagucgguacgaauc.................  1     1  H01
......................................................................uacuuugagucgguacgaauc.................  416   0  H01
......................................................................uacuuugagucgguacgaauA.................  4     1  H01
......................................................................uacuuugagucgguGcgaauca................  7     1  H01
......................................................................uacuuugagucgAuacgaauca................  1     1  H01
......................................................................uacuuugagucgguaGgaauca................  1     1  H01
......................................................................uacuuugagucggAacgaauca................  1     1  H01
......................................................................uacuuugUgucgguacgaauca................  5     1  H01
......................................................................uacuuugagucgUuacgaauca................  1     1  H01
......................................................................uacuuugCgucgguacgaauca................  1     1  H01
......................................................................uacuuugagucggCacgaauca................  1     1  H01
......................................................................uacuuugagucgCuacgaauca................  1     1  H01
......................................................................uacuuugagucgguacgaaucU................  28    1  H01
......................................................................uacuuugGgucgguacgaauca................  3     1  H01
......................................................................uacuuugagucgguacgaauAa................  3     1  H01
......................................................................uacuuugagucgguUcgaauca................  3     1  H01
......................................................................uacuuugagucgguacgaauGa................  4     1  H01
......................................................................uacuuugagucgguacgaauUa................  1     1  H01
......................................................................uGcuuugagucgguacgaauca................  1     1  H01
......................................................................uUcuuugagucgguacgaauca................  1     1  H01
......................................................................uacuuugagucgguacCaauca................  2     1  H01
......................................................................uacuuugagucgguacgaaCca................  1     1  H01
......................................................................uacCuugagucgguacgaauca................  2     1  H01
......................................................................uacuuugagucgguacgaGuca................  2     1  H01
......................................................................uacuuugagGcgguacgaauca................  1     1  H01
......................................................................uacuuugagucgguacgGauca................  5     1  H01
......................................................................uacuuugagucgguacgaauca................  4001  0  H01
......................................................................uacuuugagucgguUcgaaucac...............  1     1  H01
......................................................................uGcuuugagucgguacgaaucac...............  2     1  H01
......................................................................uacuuugagucgguacgaaucUc...............  1     1  H01
......................................................................uacuuugagucgguacgaauAac...............  1     1  H01
......................................................................uacuuugagucgguacgaaucaA...............  141   1  H01
......................................................................uacuuugagucgUuacgaaucac...............  2     1  H01
......................................................................uacuuugagucgCuacgaaucac...............  2     1  H01
......................................................................uacuuuCagucgguacgaaucac...............  1     1  H01
......................................................................uacuuugagucgguacgaGucac...............  1     1  H01
......................................................................Cacuuugagucgguacgaaucac...............  2     1  H01
......................................................................uacuuugagucgguacgaaCcac...............  1     1  H01
......................................................................uacuuugaguGgguacgaaucac...............  1     1  H01
......................................................................uacCuugagucgguacgaaucac...............  2     1  H01
......................................................................uacuuugagucgguGcgaaucac...............  5     1  H01
......................................................................uacuuugUgucgguacgaaucac...............  7     1  H01
......................................................................uUcuuugagucgguacgaaucac...............  3     1  H01
......................................................................uacuuugagucgguacgaaucac...............  2265  0  H01
......................................................................uacuuugagucgguacgaaucaG...............  2     1  H01
......................................................................uacuuugGgucgguacgaaucac...............  1     1  H01
......................................................................uacuuugagucgguaUgaaucac...............  1     1  H01
......................................................................uacuuAgagucgguacgaaucac...............  1     1  H01
......................................................................uacuuugagucgguacgCaucac...............  2     1  H01
......................................................................uacuuugagucgguacgaaucacc..............  1     0  H01
......................................................................uacuuugagucgguacgaaucacG..............  1     1  H01
```

Star Mature

```
ggaucaaaauaacuuaacgugguucaggggcgguuuguaccgacucagagcuagguucguugaugagaacuacuuugagucgguacgaaucaccucagccaugaucca
......................................................................uacuuugagucgguacgaaucacA..............   16    1    H01
......................................................................uacuuugagucgguacgaaucacAu.............   1     1    H01
.......................................................................acuuugagucgguacgaau..................   3     0    H01
.......................................................................acuuugagucgguacgaauc.................   1     0    H01
.......................................................................acuuugCgucgguacgaauc.................   1     1    H01
.......................................................................acuuugagucgguacgaauca................   6     0    H01
.......................................................................acuuugagucgguacgaaucac...............   11    0    H01
........................................................................cuuugagucgguacgaau..................   1     0    H01
........................................................................Uuuugagucgguacgaauca................   1     1    H01
........................................................................cuuugagucgguacgaauca................   2     0    H01
........................................................................cuuugagucgguacgaaucac...............   1     0    H01
.........................................................................uuugagucgguacgaauc.................   1     0    H01
.........................................................................uuugagucgguacgaauca................   10    0    H01
.........................................................................uuugagucgguacgaaucac...............   4     0    H01
..........................................................................uugagucgguacgaauca................   2     0    H01
..........................................................................uugagucgguacgaaucac...............   7     0    H01

...............................gguuuguaccgacucagagcuagguuc..................................................   1     0    LJ5
.................................uuuguaccgacucagagcuagU.....................................................   1     1    LJ5
.................................uuuguaccgacucagagcuaggu....................................................   3     0    LJ5
.....................................................................cuacuuugagucgguacgaau..................   1     0    LJ5
.....................................................................cuacuuugagucgguacgaauc.................   4     0    LJ5
.....................................................................cuacuuugagucgguacgaauU.................   1     1    LJ5
.....................................................................cuacuuugagucgguacgaauca................   12    0    LJ5
.....................................................................cuacCuugagucgguacgaaucac...............   1     1    LJ5
.....................................................................cuacuuugagucgguacgaaucGc...............   1     1    LJ5
.....................................................................cuacuuugagucgguacgaaucaA...............   2     1    LJ5
.....................................................................cuacuuugagucgguacgaaucac...............   15    0    LJ5
.....................................................................cuacuuugagucgguacgaaucacc..............   1     0    LJ5
......................................................................uacuuugagucgguacgU....................   1     1    LJ5
......................................................................uacuuugagucgguacgG....................   1     1    LJ5
......................................................................uacuuugagucgguacga....................   22    0    LJ5
......................................................................uacuuugagucgguacgaa...................   6     0    LJ5
......................................................................uacuuugagucgguacgaaC..................   5     1    LJ5
......................................................................uacuuugagucgguacgaaA..................   4     1    LJ5
......................................................................uacuuugagucgguacgaau..................   104   0    LJ5
......................................................................uacuuugagucggCacgaau..................   1     1    LJ5
......................................................................uacuuugagucgguacgGau..................   1     1    LJ5
......................................................................uacuuugagucAguacgaau..................   1     1    LJ5
......................................................................uacuuuCagucgguacgaau..................   1     1    LJ5
......................................................................uGcuuugagucgguacgaau..................   1     1    LJ5
......................................................................uaAuuugagucgguacgaauc.................   1     1    LJ5
......................................................................uacuuugagucgguacgaauU.................   22    1    LJ5
......................................................................uacuuuAagucgguacgaauc.................   1     1    LJ5
......................................................................uacuuugagucgguacgaauA.................   8     1    LJ5
......................................................................uacuuCgagucgguacgaauc.................   1     1    LJ5
......................................................................uacuuAgagucgguacgaauc.................   2     1    LJ5
......................................................................uacuuugagucgAuacgaauc.................   2     1    LJ5
......................................................................uacuuugagucgguacgUauc.................   2     1    LJ5
......................................................................uacuuugagucgguacCaauc.................   1     1    LJ5
......................................................................uaUuuugagucgguacgaauc.................   2     1    LJ5
......................................................................uacuuugagucgguacgaauc.................   416   0    LJ5
......................................................................uacuuugagucgguacgGauc.................   2     1    LJ5
......................................................................Aacuuugagucgguacgaauc.................   1     1    LJ5
......................................................................uacuuugGgucgguacgaauc.................   1     1    LJ5
......................................................................uacCuugagucgguacgaauc.................   1     1    LJ5
......................................................................uacuuGgagucgguacgaauc.................   2     1    LJ5
......................................................................uacuuugagucgguacAaauc.................   2     1    LJ5
......................................................................uacuuugagucgguacUaauc.................   1     1    LJ5
......................................................................uacuuugagucgguacgaGuc.................   3     1    LJ5
......................................................................uacuuugagucgguacgaGuca................   2     1    LJ5
......................................................................uacuuugaUucgguacgaauca................   1     1    LJ5
......................................................................uacuuugagucgguacgaaucC................   1     1    LJ5
......................................................................uacuuugagucgguacgaaCca................   5     1    LJ5
......................................................................uacuuugagucgguGcgaauca................   2     1    LJ5
......................................................................uacuuugagucgguacAaauca................   2     1    LJ5
......................................................................uacuuugagucgguacgaaGca................   1     1    LJ5
......................................................................uacuuugagucgguacgaaucG................   7     1    LJ5
......................................................................uacuuGgagucgguacgaauca................   2     1    LJ5
......................................................................uacuuugagucggCacgaauca................   1     1    LJ5
```

```
                                 Star                                 Mature
ggaucaaaauaacuuaacgugguucaggggcgguuuguaccgacucagagcuagguucguugaugagaacuacuuugagucgguacgaaucaccucagccaugaucca
......................................................................uaUuuugagucgguacgaauca................	1	1	LJ5
......................................................................uacuuugagucgguacgaaucU................	9	1	LJ5
......................................................................uacuuugagucgguaUgaauca................	1	1	LJ5
......................................................................uGcuuugagucgguacgaauca................	1	1	LJ5
......................................................................uacAuugagucgguacgaauca................	1	1	LJ5
......................................................................uacuuugagucgguacgaauca................	545	0	LJ5
......................................................................uUcuuugagucgguacgaauca................	1	1	LJ5
......................................................................uacuuugagucgguacgUauca................	1	1	LJ5
......................................................................uacCuugagucgguacgaauca................	1	1	LJ5
......................................................................uacuCugagucgguacgaauca................	1	1	LJ5
......................................................................uacuuugGgucgguacgaauca................	4	1	LJ5
......................................................................uacuuuAagucgguacgaauca................	2	1	LJ5
......................................................................uacuuugagucgguacgaaucaA...............	28	1	LJ5
......................................................................uacuuugagucgguacgaaucaG...............	1	1	LJ5
......................................................................uacuuugagucgguacgaaucac...............	84	0	LJ5
......................................................................uacuuugagucgguacUaaucac...............	1	1	LJ5
......................................................................uacuuugGgucgguacgaaucac...............	1	1	LJ5
......................................................................uacCuugagucgguacgaaucac...............	1	1	LJ5
......................................................................uacuuCgagucgguacgaaucac...............	1	1	LJ5
......................................................................uaAuuugagucgguacgaaucac...............	1	1	LJ5
......................................................................uacuuugCgucgguacgaaucac...............	2	1	LJ5
......................................................................uacuuugagucgguGcgaaucac...............	1	1	LJ5
......................................................................uacuuugagucgguacgaaucacG..............	2	1	LJ5
......................................................................uacuuugagucgguacgaaucacc..............	13	0	LJ5
......................................................................uacuuugagucgguacgaaucacA..............	5	1	LJ5
......................................................................uacuuugagucgguacgaaucaUc..............	1	1	LJ5
.......................................................................acuuugagucgguacgaau..................	1	0	LJ5
.......................................................................acuuugagucgguacgaaucac...............	1	0	LJ5
........................................................................cuuugagucgguacgaauca................	1	0	LJ5
........................................................................cuuugagucgguacgaaucacc..............	1	0	LJ5
.........................................................................uuugagucgguacgaauc.................	1	0	LJ5
.........................................................................uuugagucgguacgaaucacc..............	4	0	LJ5
.........................................................................uuugagucgguacgaaucaccC.............	1	1	LJ5
.........................................................................uuugagucgguacgaaucaccu.............	1	0	LJ5
..........................................................................uugagucgguacgaauca................	1	0	LJ5

...............................gguuuguaccgacucagagcu........................................................	8	0	2nd
...............................gguuuguaccgacucagagcua.......................................................	1	0	2nd
...............................gguuuguaccgacucagagcuagg.....................................................	1	0	2nd
...............................gguuuguaccgacucagagcuagA.....................................................	1	1	2nd
...............................gguuuguaccgacucagagcuaggu....................................................	23	0	2nd
...............................gguuuguaccgacucagagcuaggC....................................................	1	1	2nd
...............................gguuuguaccgacucagagcuagguu...................................................	8	0	2nd
.................................uuuguaccgacucagagcu........................................................	1	0	2nd
.................................uuuguaccgacucagagcua.......................................................	1	0	2nd
.................................uuuguaUcgacucagagcuag......................................................	1	1	2nd
.................................uuuguaccgacucagagcuag......................................................	14	0	2nd
.................................uuuguaccgacucagagcuagg.....................................................	13	0	2nd
.................................uuuguaccgacucagagcuaggu....................................................	61	0	2nd
.................................uuuguaccgacucagagcuaggA....................................................	4	1	2nd
.................................uuuguaccgGcucagagcuaggu....................................................	1	1	2nd
.................................uuuguaccgacucagagGuaggu....................................................	1	1	2nd
.................................uuuguaccgacucagagcuagguu...................................................	18	0	2nd
..................................uuguaccgacucagagcuagg.....................................................	1	0	2nd
..................................uuguaccgacucagagcuagguuc..................................................	1	0	2nd
...................................uguaccgacucagagcuagguu...................................................	3	0	2nd
...................................uguaccgacucagagcuagguuc..................................................	16	0	2nd
...................................uguaccgacucagagcuagguucU.................................................	1	1	2nd
.................................................................agaGcuacuuugagucgguacgaauca................	1	1	2nd
..................................................................gGacuacuuugagucgguacgaau..................	2	1	2nd
..................................................................gaaGuacuuugagucgguacgaauca................	1	1	2nd
..................................................................gGacuacuuugagucgguacgaauca................	2	1	2nd
..................................................................gaUcuacuuugagucgguacgaauca................	1	1	2nd
..................................................................Aaacuacuuugagucgguacgaaucac...............	1	1	2nd
...................................................................aacuacuuugagucgguacg.....................	1	0	2nd
...................................................................aacuacuuugagucgguacga....................	2	0	2nd
...................................................................aacuacuuugagucgguacgaa...................	1	0	2nd
...................................................................aaGuacuuugagucgguacgaauc.................	1	1	2nd
...................................................................aaGuacuuugagucgguacgaauca................	1	1	2nd
....................................................................acuacuuugagucgguacga....................	2	0	2nd
```

Star Mature

```
ggaucaaaauaacuuaacgugguucaggggcgguuuguaccgacucagagcuagguucguugaugagaacuacuuugagucgguacgaaucaccucagccaugaucca
....................................................................Ucuacuuugagucgguacgaauca................    2     1     2nd
....................................................................acuacuuugagucgguacgaaucac...............    1     0     2nd
....................................................................Ucuacuuugagucgguacgaaucac...............    2     1     2nd
.....................................................................cuacuuugagucgguacg.....................    10    0     2nd
.....................................................................cuacuuugagucgguacga....................    3     0     2nd
.....................................................................cuacuuugagucgguacgaa...................    16    0     2nd
.....................................................................cuacuuugagucgguacgaaA..................    1     1     2nd
.....................................................................Uuacuuugagucgguacgaau..................    5     1     2nd
.....................................................................cuacuuugagucgguacgaau..................    236   0     2nd
.....................................................................cuacuuugagucgguGcgaau..................    2     1     2nd
.....................................................................cuacuuugagucgguacgaUu..................    1     1     2nd
.....................................................................Guacuuugagucgguacgaau..................    1     1     2nd
.....................................................................cuacCuugagucgguacgaauc.................    1     1     2nd
.....................................................................cuacuuugagucgguacgaauc.................    322   0     2nd
.....................................................................Uuacuuugagucgguacgaauc.................    10    1     2nd
.....................................................................Guacuuugagucgguacgaauc.................    3     1     2nd
.....................................................................cuacuuugaguAgguacgaauc.................    1     1     2nd
.....................................................................cuacuuugagucgguacgaauU.................    2     1     2nd
.....................................................................cuacuuugagucgguacCaauc.................    1     1     2nd
.....................................................................cuacuuugUgucgguacgaauc.................    1     1     2nd
.....................................................................cuacuuugagucgguaGgaauca................    1     1     2nd
.....................................................................Uuacuuugagucgguacgaauca................    60    1     2nd
.....................................................................cuacuuugagucgAuacgaauca................    3     1     2nd
.....................................................................cuacuuugagucgguGcgaauca................    15    1     2nd
.....................................................................cuacuuuGGgucgguacgaauca................    7     1     2nd
.....................................................................cuacuuugagucgguacgaauca................    5728  0     2nd
.....................................................................cuacuuugagucgguacgaGuca................    5     1     2nd
.....................................................................cuacuuugagucgCuacgaauca................    1     1     2nd
.....................................................................cuacuuugagucgguCcgaauca................    1     1     2nd
.....................................................................cuacuGugagucgguacgaauca................    5     1     2nd
.....................................................................cuacuuugagucgguUcgaauca................    1     1     2nd
.....................................................................cuacuAugagucgguacgaauca................    9     1     2nd
.....................................................................cuacuuugagucgguacgGauca................    2     1     2nd
.....................................................................cuacuuugagAcgguacgaauca................    2     1     2nd
.....................................................................cuacuuugagucgguacgaaCca................    3     1     2nd
.....................................................................cuacuCugagucgguacgaauca................    2     1     2nd
.....................................................................cuGcuuugagucgguacgaauca................    1     1     2nd
.....................................................................cuacuuugagucgguaUgaauca................    1     1     2nd
.....................................................................cuacuuugagucgguacgaauAa................    1     1     2nd
.....................................................................cuacuuugaUucgguacgaauca................    1     1     2nd
.....................................................................cuacuuugagucgguacgaUuca................    11    1     2nd
.....................................................................cAacuuugagucgguacgaauca................    2     1     2nd
.....................................................................cuacuuugagucgguacgUauca................    2     1     2nd
.....................................................................cuacCuugagucgguacgaauca................    3     1     2nd
.....................................................................cuacuuugagucgguacgaaucU................    10    1     2nd
.....................................................................cuacuuugUgucgguacgaauca................    10    1     2nd
.....................................................................Guacuuugagucgguacgaauca................    2     1     2nd
.....................................................................cuacuuugagucgguacAaauca................    2     1     2nd
.....................................................................cuacuuAgagucgguacgaauca................    1     1     2nd
.....................................................................cuacuuugagucggCacgaauca................    1     1     2nd
.....................................................................cuacuuugagucgguacgaCuca................    3     1     2nd
.....................................................................cuacuuugagucggAacgaauca................    2     1     2nd
.....................................................................cuacuuugaguAgguacgaauca................    2     1     2nd
.....................................................................cuacuuuCagucgguacgaauca................    4     1     2nd
.....................................................................cuacuuugagCcgguacgaauca................    1     1     2nd
.....................................................................cuacuuugCgucgguacgaauca................    2     1     2nd
.....................................................................cuacuuugagucgAuacgaaucac...............    3     1     2nd
.....................................................................cuacuCugagucgguacgaaucac...............    2     1     2nd
.....................................................................cuaAuuugagucgguacgaaucac...............    1     1     2nd
.....................................................................cuacCuugagucgguacgaaucac...............    2     1     2nd
.....................................................................cuacuuugUgucgguacgaaucac...............    6     1     2nd
.....................................................................cuacuuugagucgguaUgaaucac...............    2     1     2nd
.....................................................................cuGcuuugagucgguacgaaucac...............    1     1     2nd
.....................................................................cuacuuugagucgguacgaaucUc...............    1     1     2nd
.....................................................................cAacuuugagucgguacgaaucac...............    3     1     2nd
.....................................................................cuacuAugagucgguacgaaucac...............    3     1     2nd
.....................................................................cuacuuuAagucgguacgaaucac...............    1     1     2nd
.....................................................................cuacuuAgagucgguacgaaucac...............    1     1     2nd
.....................................................................cuacuuugaAucgguacgaaucac...............    1     1     2nd
.....................................................................cuacuuGgagucgguacgaaucac...............    1     1     2nd
```

Star Mature

```
ggaucaaaauaacuuaacgugguucaggggcgguuuguaccgacucagagcuagguucguugaugagaacuacuuugagucgguacgaaucaccucagccaugaucca
.....................................................................cuacuuugagucgguacgaaucac...............	5576	0	2nd
.....................................................................cuacuuugagucgguacgaUucac...............	8	1	2nd
.....................................................................cuacuuugagucgguacCaaucac...............	4	1	2nd
.....................................................................cuacuuugagucgUuacgaaucac...............	3	1	2nd
.....................................................................cuacuuugGgucgguacgaaucac...............	6	1	2nd
.....................................................................Uuacuuugagucgguacgaaucac...............	37	1	2nd
.....................................................................cuacuuugagucgguacgaaCcac...............	1	1	2nd
.....................................................................cuacuuugagucgguacgaaucaA...............	72	1	2nd
.....................................................................cuacuuugagucgguacAaaucac...............	1	1	2nd
.....................................................................cuacuuugagucgguacgaauUac...............	1	1	2nd
.....................................................................cuacuuugagucUguacgaaucac...............	1	1	2nd
.....................................................................cuacuuugagucgguacgGaucac...............	4	1	2nd
.....................................................................cuacuuugagucggAacgaaucac...............	1	1	2nd
.....................................................................cuacuuugagAcgguacgaaucac...............	1	1	2nd
.....................................................................cuacuGugagucgguacgaaucac...............	7	1	2nd
.....................................................................cuacuuugagucgguacgaaucCc...............	1	1	2nd
.....................................................................cuacuuugagucgguGcgaaucac...............	22	1	2nd
.....................................................................cuacuuugagucgguacgaaucaG...............	1	1	2nd
.....................................................................cuaUuuugagucgguacgaaucac...............	3	1	2nd
.....................................................................cuacuuugCgucgguacgaaucac...............	3	1	2nd
.....................................................................cuacuuugagucgguacgaaucacc..............	6	0	2nd
.....................................................................cuacuuugagucgguacgaaucacA..............	34	1	2nd
......................................................................uacuuCgagucgguacga....................	1	1	2nd
......................................................................uacuuuCagucgguacga....................	1	1	2nd
......................................................................uacuuugagucgguacCa....................	1	1	2nd
......................................................................uacuuugagucgguacga....................	347	0	2nd
......................................................................uacuuAgagucgguacga....................	1	1	2nd
......................................................................uacuuugagucgguGcga....................	2	1	2nd
......................................................................uacuuugagucgguacgC....................	1	1	2nd
......................................................................uacuuugagucgguacUa....................	1	1	2nd
......................................................................uacuuugCgucgguacga....................	1	1	2nd
......................................................................uacuuAgagucgguacgaa...................	1	1	2nd
......................................................................uacuuugagucgguacgGa...................	2	1	2nd
......................................................................uacuuugagucgguacgaa...................	491	0	2nd
......................................................................uacuuugagucgguacgaU...................	1	1	2nd
......................................................................uacuuugagucgguGcgaa...................	1	1	2nd
......................................................................uacCuugagucgguacgaa...................	1	1	2nd
......................................................................uacuuugUgucgguacgaa...................	1	1	2nd
......................................................................Aacuuugagucgguacgaau..................	2	1	2nd
......................................................................uacuuugCgucgguacgaau..................	5	1	2nd
......................................................................uacuuugagucgguacgaaA..................	268	1	2nd
......................................................................uacuuugaguAgguacgaau..................	1	1	2nd
......................................................................uacuuugagucgguacgaCu..................	1	1	2nd
......................................................................uacuuugaCucgguacgaau..................	1	1	2nd
......................................................................uacuuGgagucgguacgaau..................	9	1	2nd
......................................................................uacuuuCagucgguacgaau..................	2	1	2nd
......................................................................uacuuugagucgguacgaGu..................	1	1	2nd
......................................................................uacuuugagCcgguacgaau..................	3	1	2nd
......................................................................uacuuAgagucgguacgaau..................	14	1	2nd
......................................................................uacuuugagGcgguacgaau..................	1	1	2nd
......................................................................uacuuugagucgguacgGau..................	5	1	2nd
......................................................................uacuuugaUucgguacgaau..................	2	1	2nd
......................................................................uacuuCgagucgguacgaau..................	17	1	2nd
......................................................................uacuuugagucggGacgaau..................	1	1	2nd
......................................................................uacuuugagucgguacgaau..................	13427	0	2nd
......................................................................uacuuugagucgguacCaau..................	1	1	2nd
......................................................................Cacuuugagucgguacgaau..................	3	1	2nd
......................................................................uacuuugagucggCacgaau..................	1	1	2nd
......................................................................uGcuuugagucgguacgaau..................	3	1	2nd
......................................................................uacuuugagucgguacgaaG..................	11	1	2nd
......................................................................uacuuugagucAguacgaau..................	6	1	2nd
......................................................................uUcuuugagucgguacgaau..................	1	1	2nd
......................................................................uacuGugagucgguacgaau..................	2	1	2nd
......................................................................uacuuugGgucgguacgaau..................	12	1	2nd
......................................................................uacuuugagucgguacgCau..................	4	1	2nd
......................................................................uacuuuAagucgguacgaau..................	2	1	2nd
......................................................................uacuuugagucgUuacgaau..................	3	1	2nd
......................................................................uacuuugagucggAacgaau..................	2	1	2nd
......................................................................uacuuugagucgCuacgaau..................	7	1	2nd
......................................................................Gacuuugagucgguacgaau..................	4	1	2nd
```

```
                                 Star                                Mature
ggaucaaaauaacuuaacgugguucaggggcgguuuguaccgacucagagcuagguucguugaugagaacuacuuugagucgguacgaaucaccucagccaugaucca
......................................................................uacuuugagucgguGcgaau..................  35     1   2nd
......................................................................uacuCugagucgguacgaau..................  1      1   2nd
......................................................................uacuuugagucgguacgUau..................  2      1   2nd
......................................................................uacuuugagucgguacAaau..................  1      1   2nd
......................................................................uacuuuUagucgguacgaau..................  5      1   2nd
......................................................................uaUuuugagucgguacgaau..................  1      1   2nd
......................................................................uacuuugagucUguacgaau..................  1      1   2nd
......................................................................uacCuugagucgguacgaau..................  8      1   2nd
......................................................................uacuuugagucgguacgaaC..................  17     1   2nd
......................................................................uacuuugagucgguacgaUu..................  1      1   2nd
......................................................................uacuuugagucgguUcgaau..................  7      1   2nd
......................................................................uacuuugUgucgguacgaau..................  13     1   2nd
......................................................................uCcuuugagucgguacgaau..................  1      1   2nd
......................................................................uacuuugagucgguacUaau..................  1      1   2nd
......................................................................uacuuugagucgguaUgaau..................  2      1   2nd
......................................................................uacuuugaUucgguacgaauc.................  1      1   2nd
......................................................................uacuuugaCucgguacgaauc.................  5      1   2nd
......................................................................uacuuugagucgguacgUauc.................  3      1   2nd
......................................................................uGcuuugagucgguacgaauc.................  6      1   2nd
......................................................................uaGuuugagucgguacgaauc.................  1      1   2nd
......................................................................uacuuugagucgguacUaauc.................  3      1   2nd
......................................................................uacuuugagucgguacgaauG.................  5      1   2nd
......................................................................Gacuuugagucgguacgaauc.................  5      1   2nd
......................................................................uacuuGgagucgguacgaauc.................  14     1   2nd
......................................................................uacuuugagCcgguacgaauc.................  2      1   2nd
......................................................................uacuuugagucgguacgaCuc.................  2      1   2nd
......................................................................uacuuugagucgCuacgaauc.................  8      1   2nd
......................................................................uacuuugagucgguacgaaCc.................  28     1   2nd
......................................................................uacuuugagucAguacgaauc.................  3      1   2nd
......................................................................uacuuugagucCguacgaauc.................  5      1   2nd
......................................................................uacuuugagucgguacgaauc.................  28208  0   2nd
......................................................................uacuuugagucggGacgaauc.................  2      1   2nd
......................................................................uacuuugagucgguacgaUuc.................  3      1   2nd
......................................................................uacuuugagucgguGcgaauc.................  56     1   2nd
......................................................................uacuuugaAucgguacgaauc.................  1      1   2nd
......................................................................uacuuugaguGgguacgaauc.................  3      1   2nd
......................................................................uacuuCgagucgguacgaauc.................  23     1   2nd
......................................................................uacuCugagucgguacgaauc.................  3      1   2nd
......................................................................uacuuugGgucgguacgaauc.................  40     1   2nd
......................................................................uacuGugagucgguacgaauc.................  3      1   2nd
......................................................................uacuuugagucgguacgaauA.................  294    1   2nd
......................................................................uacuuugUgucgguacgaauc.................  25     1   2nd
......................................................................uacuAugagucgguacgaauc.................  1      1   2nd
......................................................................uacuuugagucgguacCaauc.................  8      1   2nd
......................................................................uacuuugaguAgguacgaauc.................  5      1   2nd
......................................................................uaUuuugagucgguacgaauc.................  4      1   2nd
......................................................................uCcuuugagucgguacgaauc.................  3      1   2nd
......................................................................uacuuuAagucgguacgaauc.................  3      1   2nd
......................................................................uacuuugagucgguaAgaauc.................  9      1   2nd
......................................................................uacuuugagucgguCcgaauc.................  2      1   2nd
......................................................................uaAuuugagucgguacgaauc.................  1      1   2nd
......................................................................uacuuugagucgguUcgaauc.................  7      1   2nd
......................................................................uacuuugagucgguaGgaauc.................  1      1   2nd
......................................................................uUcuuugagucgguacgaauc.................  2      1   2nd
......................................................................uacuuugagGcgguacgaauc.................  1      1   2nd
......................................................................Aacuuugagucgguacgaauc.................  3      1   2nd
......................................................................uacuuAgagucgguacgaauc.................  15     1   2nd
......................................................................uacuuugagucgguacgaauU.................  44     1   2nd
......................................................................uacuuugagucgguacgaaGc.................  11     1   2nd
......................................................................uacuuuCagucgguacgaauc.................  6      1   2nd
......................................................................uacCuugagucgguacgaauc.................  8      1   2nd
......................................................................uacuuugagucgguacgCauc.................  2      1   2nd
......................................................................uacuuugagucgguacAaauc.................  3      1   2nd
......................................................................uacuuugagucgUuacgaauc.................  3      1   2nd
......................................................................uacuuugagucgguacgaaAc.................  5      1   2nd
......................................................................uacuuugagucgguaUgaauc.................  10     1   2nd
......................................................................uacuuugCgucgguacgaauc.................  9      1   2nd
......................................................................uacuuuUagucgguacgaauc.................  15     1   2nd
......................................................................uacuuugagucgAuacgaauc.................  6      1   2nd
......................................................................uacuuugagucgguacgGauc.................  19     1   2nd
```

Star Mature

```
ggaucaaaauaacuuaacgugguucaggggcgguuuguaccgacucagagcuagguucguugaugagaacuacuuugagucgguacgaaucaccucagccaugaucca
......................................................................Cacuuugagucgguacgaauc.................	1	1	2nd
......................................................................uacuuugagucggCacgaauc.................	5	1	2nd
......................................................................uacuuugagucgguacgaGuc.................	7	1	2nd
......................................................................uacuuugagucUguacgaauc.................	3	1	2nd
......................................................................uacuuugaguAgguacgaauca................	32	1	2nd
......................................................................uacuuugaUucgguacgaauca................	5	1	2nd
......................................................................uacuuuAagucgguacgaauca................	20	1	2nd
......................................................................uacuuugagucgguacgaaucU................	673	1	2nd
......................................................................uacuuugagucgguacgaaAca................	24	1	2nd
......................................................................uaAuuugagucgguacgaauca................	14	1	2nd
......................................................................uacuuugagucgguCcgaauca................	9	1	2nd
......................................................................uacuuugagucgguacCaauca................	49	1	2nd
......................................................................uacuuugagucgUuacgaauca................	13	1	2nd
......................................................................uacuuugagucgguaAgaauca................	30	1	2nd
......................................................................uacuuugagucgAuacgaauca................	37	1	2nd
......................................................................uacuuugagucgguacgUauca................	30	1	2nd
......................................................................uacuuugagucgguacgaCuca................	7	1	2nd
......................................................................uacuuugaAucgguacgaauca................	5	1	2nd
......................................................................Aacuuugagucgguacgaauca................	10	1	2nd
......................................................................uacuuugagucggCacgaauca................	39	1	2nd
......................................................................uacuuugagucgguacgaaucC................	15	1	2nd
......................................................................uacuuugagucCguacgaauca................	21	1	2nd
......................................................................uacCuugagucgguacgaauca................	120	1	2nd
......................................................................uacuuugagucgguacgaauUa................	38	1	2nd
......................................................................uacuuugagucgguacgaauAa................	96	1	2nd
......................................................................uacuuugagucgguacgaauca................	253207	0	2nd
......................................................................uacuuugagucgguGcgaauca................	523	1	2nd
......................................................................uacuuAgagucgguacgaauca................	191	1	2nd
......................................................................uacuuugagucgguacgaaCca................	110	1	2nd
......................................................................uacuuugagucgguacUaauca................	10	1	2nd
......................................................................uacuGugagucgguacgaauca................	9	1	2nd
......................................................................uGcuuugagucgguacgaauca................	33	1	2nd
......................................................................uacuuugagGcgguacgaauca................	12	1	2nd
......................................................................uacuuugagucgguacAaauca................	18	1	2nd
......................................................................uacuuugagucgguacgaaGca................	114	1	2nd
......................................................................Cacuuugagucgguacgaauca................	20	1	2nd
......................................................................uacuAugagucgguacgaauca................	4	1	2nd
......................................................................uacuuuCagucgguacgaauca................	44	1	2nd
......................................................................uacuuugagucgguacgaUuca................	5	1	2nd
......................................................................uacuuugagucgguacgaauGa................	23	1	2nd
......................................................................uUcuuugagucgguacgaauca................	23	1	2nd
......................................................................uacuuugagucAguacgaauca................	10	1	2nd
......................................................................uacuuugGgucgguacgaauca................	307	1	2nd
......................................................................uacuuCgagucgguacgaauca................	145	1	2nd
......................................................................uacuuGgagucgguacgaauca................	143	1	2nd
......................................................................uacuuugUgucgguacgaauca................	288	1	2nd
......................................................................uacuuugagucUguacgaauca................	9	1	2nd
......................................................................uaUuuugagucgguacgaauca................	52	1	2nd
......................................................................uacuuugagucgguUcgaauca................	79	1	2nd
......................................................................uacuuugagucgguacgaaucG................	111	1	2nd
......................................................................uCcuuugagucgguacgaauca................	7	1	2nd
......................................................................uacuCugagucgguacgaauca................	18	1	2nd
......................................................................uacuuugagucggGacgaauca................	36	1	2nd
......................................................................uacuuuUagucgguacgaauca................	152	1	2nd
......................................................................uaGuuugagucgguacgaauca................	21	1	2nd
......................................................................Gacuuugagucgguacgaauca................	37	1	2nd
......................................................................uacAuugagucgguacgaauca................	2	1	2nd
......................................................................uacuuugagucgCuacgaauca................	42	1	2nd
......................................................................uacGuugagucgguacgaauca................	4	1	2nd
......................................................................uacuuugagucgguacgaGuca................	24	1	2nd
......................................................................uacuuugagucggAacgaauca................	17	1	2nd
......................................................................uacuuugagucgguaUgaauca................	89	1	2nd
......................................................................uacuuugagCcgguacgaauca................	20	1	2nd
......................................................................uacuuugagucgguaGgaauca................	32	1	2nd
......................................................................uacuuugCgucgguacgaauca................	110	1	2nd
......................................................................uacuuugaguGgguacgaauca................	17	1	2nd
......................................................................uacuuugagucgguacgGauca................	125	1	2nd
......................................................................uacuuugagucgguacgCauca................	45	1	2nd
......................................................................uacuuugaCucgguacgaauca................	23	1	2nd
......................................................................uacuuugagucgguacgaGucac...............	35	1	2nd
```

Star Mature

```
ggaucaaaauaacuuaacgugguucaggggcgguuuguaccgacucagagcuaggucguugaugagaacuacuuugagucgguacgaaucaccucagccaugaucca
......................................................................uCcuuugagucgguacgaaucac...............	6	1	2nd
......................................................................uacuuugagucgguGcgaaucac...............	387	1	2nd
......................................................................uacuuugagucgCuacgaaucac...............	28	1	2nd
......................................................................uacuuugagucgguacgaaucac...............	139468	0	2nd
......................................................................uacuuugagucgguaGgaaucac...............	8	1	2nd
......................................................................uacuuugaguGgguacgaaucac...............	11	1	2nd
......................................................................uacuuugagucgguacgaaucCc...............	15	1	2nd
......................................................................uacCuugagucgguacgaaucac...............	60	1	2nd
......................................................................uacuuugagucgguacgaaCcac...............	25	1	2nd
......................................................................uacuCugagucgguacgaaucac...............	14	1	2nd
......................................................................uacuuugagucggAacgaaucac...............	6	1	2nd
......................................................................uacuuugagucgguacUaaucac...............	5	1	2nd
......................................................................uacuuugagucgguacgaaAcac...............	11	1	2nd
......................................................................uacuuugagucgguacgaaucUc...............	17	1	2nd
......................................................................uacuuugaUucgguacgaaucac...............	3	1	2nd
......................................................................uaGuuugagucgguacgaaucac...............	7	1	2nd
......................................................................uacuuugagucgguacgUaucac...............	10	1	2nd
......................................................................uacuuAgagucgguacgaaucac...............	104	1	2nd
......................................................................uacuuugagucgguacgaUucac...............	7	1	2nd
......................................................................Aacuuugagucgguacgaaucac...............	8	1	2nd
......................................................................Cacuuugagucgguacgaaucac...............	16	1	2nd
......................................................................uacuuugagucgguacgaaucaA...............	6586	1	2nd
......................................................................uacuuugagucgguacgGaucac...............	63	1	2nd
......................................................................uacuuuAagucgguacgaaucac...............	24	1	2nd
......................................................................uaAuuugagucgguacgaaucac...............	4	1	2nd
......................................................................uacuuugagucgguacgaaGcac...............	59	1	2nd
......................................................................uacuuugagucgguCcgaaucac...............	5	1	2nd
......................................................................uacuuugagucgguacgaauUac...............	32	1	2nd
......................................................................uacuuugaguAgguacgaaucac...............	15	1	2nd
......................................................................uacuuugagucUguacgaaucac...............	13	1	2nd
......................................................................uaUuuugagucgguacgaaucac...............	18	1	2nd
......................................................................uUcuuugagucgguacgaaucac...............	12	1	2nd
......................................................................uacuuugagucgguacgaaucaG...............	54	1	2nd
......................................................................uacuuugaAucgguacgaaucac...............	9	1	2nd
......................................................................uacGuugagucgguacgaaucac...............	6	1	2nd
......................................................................uacuuugagucgguaAgaaucac...............	29	1	2nd
......................................................................uacuuugagucgguacgaCucac...............	12	1	2nd
......................................................................uacuuugagucggGacgaaucac...............	13	1	2nd
......................................................................uacuuugagucgguacgaauGac...............	14	1	2nd
......................................................................uacuuGgagucgguacgaaucac...............	79	1	2nd
......................................................................uacuuugagucgguacgaauAac...............	16	1	2nd
......................................................................uacAuugagucgguacgaaucac...............	1	1	2nd
......................................................................uacuuuCagucgguacgaaucac...............	18	1	2nd
......................................................................uacuuugagAcgguacgaaucac...............	3	1	2nd
......................................................................uacuuugagucgUuacgaaucac...............	18	1	2nd
......................................................................uacuuugagCcgguacgaaucac...............	11	1	2nd
......................................................................uacuuugagucAguacgaaucac...............	9	1	2nd
......................................................................uacuuugagGcgguacgaaucac...............	7	1	2nd
......................................................................uacuuugGgucgguacgaaucac...............	180	1	2nd
......................................................................uacuuugagucgguacAaaucac...............	25	1	2nd
......................................................................uacuuuUagucgguacgaaucac...............	82	1	2nd
......................................................................uacuuugagucCguacgaaucac...............	13	1	2nd
......................................................................Gacuuugagucgguacgaaucac...............	19	1	2nd
......................................................................uacuGugagucgguacgaaucac...............	6	1	2nd
......................................................................uacuuugaCucgguacgaaucac...............	15	1	2nd
......................................................................uacuuugagucgguacgCaucac...............	12	1	2nd
......................................................................uacuuugagucggCacgaaucac...............	19	1	2nd
......................................................................uacuuCgagucgguacgaaucac...............	82	1	2nd
......................................................................uacuAugagucgguacgaaucac...............	3	1	2nd
......................................................................uacuuugagucgguacCaaucac...............	55	1	2nd
......................................................................uacuuugagucgguaUgaaucac...............	57	1	2nd
......................................................................uacuuugagucgguacgaaucGc...............	15	1	2nd
......................................................................uacuuugagucgguUcgaaucac...............	42	1	2nd
......................................................................uacuuugCgucgguacgaaucac...............	60	1	2nd
......................................................................uacuuugagucgAuacgaaucac...............	23	1	2nd
......................................................................uacuuugUgucgguacgaaucac...............	178	1	2nd
......................................................................uGcuuugagucgguacgaaucac...............	36	1	2nd
......................................................................uacuuugagucgguacgaaucaAc..............	1	1	2nd
......................................................................uacuuugagucgguacgaaucacA..............	6584	1	2nd
......................................................................uacuuugagucgguacgaaucaUc..............	4	1	2nd
```

Star Mature

```
ggaucaaaauaacuuaacgugguucaggggcgguuuguaccgacucagagcuagguucguugaugagaacuacuuugagucgguacgaaucaccucagccaugaucca
......................................................................uacuuugagucgguacgaaucacG..............	11	1	2nd
......................................................................uacuuugagucgguacgaaucacc..............	69	0	2nd
......................................................................uacuuugagucgguacgaaucacAu.............	16	1	2nd
......................................................................uacuuugagucgguacgaaucaccA.............	19	1	2nd
......................................................................uacuuugagucgguacgaaucaccu.............	8	0	2nd
......................................................................uacuuugagucgguacgaaucaUcu.............	2	1	2nd
......................................................................uacuuugagucgguacgaaucacGu.............	1	1	2nd
.......................................................................acuuugGgucgguacgaau..................	1	1	2nd
.......................................................................acuuugagucgguacgaau..................	18	0	2nd
.......................................................................acuuugagucCguacgaauc.................	1	1	2nd
.......................................................................acuuugagucgguacgaauc.................	42	0	2nd
.......................................................................acuuugGgucgguacgaauca................	3	1	2nd
.......................................................................acuuugCgucgguacgaauca................	4	1	2nd
.......................................................................acuuugagucgguGcgaauca................	2	1	2nd
.......................................................................acuuugagucgguacgaauca................	386	0	2nd
.......................................................................Gcuuugagucgguacgaauca................	1	1	2nd
.......................................................................acuuCgagucgguacgaauca................	1	1	2nd
.......................................................................acuuugUgucgguacgaauca................	2	1	2nd
.......................................................................acuuugagucgguacAaauca................	1	1	2nd
.......................................................................Ucuuugagucgguacgaaucac...............	2	1	2nd
.......................................................................acuuugUgucgguacgaaucac...............	3	1	2nd
.......................................................................acuuugaCucgguacgaaucac...............	1	1	2nd
.......................................................................acuuugGgucgguacgaaucac...............	2	1	2nd
.......................................................................acuuugagucgguacgaauGac...............	1	1	2nd
.......................................................................acuuugagucAguacgaaucac...............	1	1	2nd
.......................................................................acuuugagucgguacgaaucaA...............	7	1	2nd
.......................................................................acuuugagucgguacgaGucac...............	1	1	2nd
.......................................................................acuuugagucgguacgaauUac...............	2	1	2nd
.......................................................................acuuCgagucgguacgaaucac...............	1	1	2nd
.......................................................................acuuugagucgguacgaaucGc...............	1	1	2nd
.......................................................................acuuugagucgguacgaaucac...............	538	0	2nd
.......................................................................acuuugagucgguacgaaucacc..............	1	0	2nd
.......................................................................acuuugagucgguacgaaucacA..............	8	1	2nd
.......................................................................acuuugagucgguacgaaucacG..............	1	1	2nd
........................................................................cuuugagucgguacgaau..................	3	0	2nd
........................................................................cuuugagucgguacgaauc.................	5	0	2nd
........................................................................cuuugagucgguacgaauca................	21	0	2nd
........................................................................cuuGgagucgguacgaauca................	1	1	2nd
........................................................................cuuugagucgguGcgaauca................	1	1	2nd
........................................................................Uuuugagucgguacgaauca................	8	1	2nd
........................................................................Uuuugagucgguacgaaucac...............	1	1	2nd
........................................................................cuuugagucgguacgaaGcac...............	1	1	2nd
........................................................................cuuugagucgguacgaaucac...............	30	0	2nd
........................................................................cuuugagucgguGcgaaucac...............	2	1	2nd
........................................................................cuuugagucgguacgaaucacG..............	1	1	2nd
........................................................................cuuugagucgguacgaaucacA..............	11	1	2nd
.........................................................................uuugagucgguacgaauc.................	6	0	2nd
.........................................................................uuugagucgguacgaauGa................	1	1	2nd
.........................................................................uuugagucgguGcgaauca................	1	1	2nd
.........................................................................uuugagucgguacgaaucU................	1	1	2nd
.........................................................................uuugagucgguacgaauca................	89	0	2nd
.........................................................................Cuugagucgguacgaauca................	2	1	2nd
.........................................................................uuugGgucgguacgaauca................	1	1	2nd
.........................................................................uuugagucgguacgaaucaA...............	3	1	2nd
.........................................................................uuugagucgguacgaaucac...............	111	0	2nd
.........................................................................uuugUgucgguacgaaucac...............	1	1	2nd
.........................................................................uuugagucgguacgaaucacc..............	2	0	2nd
.........................................................................uuugagucgguacgaaucacA..............	10	1	2nd
.........................................................................uuugagucgguacgaaucaccA.............	3	1	2nd
.........................................................................uuugagucgguacgaaucaccu.............	1	0	2nd
..........................................................................uugagucgguacgaauAa................	1	1	2nd
..........................................................................uugagucgguacgaauca................	104	0	2nd
..........................................................................Gugagucgguacgaauca................	1	1	2nd
..........................................................................uugGgucgguacgaauca................	1	1	2nd
..........................................................................uugagucgguGcgaauca................	1	1	2nd
..........................................................................uugagucgguacgaaucU................	1	1	2nd
..........................................................................uugagucgguacgaaAcac...............	1	1	2nd
..........................................................................uugagucgguGcgaaucac...............	1	1	2nd
..........................................................................uugagucgguacgaaucac...............	303	0	2nd
..........................................................................uugagucgguacgaaucacA..............	6	1	2nd
```

Star **Mature**

```
ggaucaaaauaacuuaacgugguucaggggcgguuuguaccgacucagagcuagguucguugaugagaacuacuuugagucgguacgaaucaccucagccaugaucca
...........................................................................ugagucgguacgaaucac...............  15    0  2nd
...........................................................................ugagucgguacgaaucaA...............  1     1  2nd
...........................................................................Cgagucgguacgaaucac...............  1     1  2nd
...........................................................................ugagucgguacgaaucacA..............  1     1  2nd

...............................gguuuguaccgacucagagcuag......................................................  1     0  TA1
...............................gguuuguaccgacucagagcuagg.....................................................  1     0  TA1
...............................gguuuguaccgacucagagcuaggu....................................................  3     0  TA1
................................guuuguaccgacucagagcuagg.....................................................  2     0  TA1
................................guuuguaccgacucagagcuaggu....................................................  2     0  TA1
.................................uuuguaccgacucagagcA........................................................  1     1  TA1
.................................uuuguaccgacucagagcuag......................................................  3     0  TA1
.................................uuuguaccgacucagagcuagg.....................................................  9     0  TA1
.................................uuuguaccgacucagagcuaggu....................................................  16    0  TA1
.................................uuuguaccgacucagagcuaggC....................................................  1     1  TA1
.................................uuuguaccgacuUagagcuaggu....................................................  1     1  TA1
.................................uuuguaccgacucagagcuagguu...................................................  3     0  TA1
...................................uguaccgacucagagcuagguu...................................................  1     0  TA1
...................................uguaccgacucagagcuagguuU..................................................  1     1  TA1
.....................................................................cuacuuugagucgguacg.....................  4     0  TA1
.....................................................................cuacuuugagucgguacA.....................  1     1  TA1
.....................................................................cuacuuugagucgguacga....................  6     0  TA1
.....................................................................cuacuuugagucgguacgaa...................  6     0  TA1
.....................................................................cuacuuugagucgguacgaaC..................  2     1  TA1
.....................................................................cuacuuCgagucgguacgaau..................  2     1  TA1
.....................................................................cuacuuugagucgguacgaaA..................  5     1  TA1
.....................................................................cuacuuugagucgguacgaau..................  53    0  TA1
.....................................................................cuacuuugagucgguacgaaG..................  1     1  TA1
.....................................................................cuacuuugagucgguacgaauA.................  7     1  TA1
.....................................................................cuacuuCgagucgguacgaauc.................  3     1  TA1
.....................................................................cuacuuugGgucgguacgaauc.................  1     1  TA1
.....................................................................cuacuuugagucgguacgaauU.................  7     1  TA1
.....................................................................cuacuuugagucgguacgaaCc.................  2     1  TA1
.....................................................................cuacuuugagucgguacgaauc.................  318   0  TA1
.....................................................................cuacuuugagCcgguacgaauc.................  2     1  TA1
.....................................................................cuacuuugagucggCacgaauc.................  1     1  TA1
.....................................................................cuacGuugagucgguacgaauc.................  1     1  TA1
.....................................................................cuacuuugagucgAuacgaauc.................  1     1  TA1
.....................................................................cuacCuugagucgguacgaauc.................  1     1  TA1
.....................................................................cuGcuuugagucgguacgaauc.................  3     1  TA1
.....................................................................cuacuuugagucgguGcgaauc.................  1     1  TA1
.....................................................................cuacuuugUgucgguacgaauca................  1     1  TA1
.....................................................................cuacuuugCgucgguacgaauca................  1     1  TA1
.....................................................................cuacCuugagucgguacgaauca................  7     1  TA1
.....................................................................cuacuuugagucgguacgaaucG................  19    1  TA1
.....................................................................cuacuuugagCcgguacgaauca................  4     1  TA1
.....................................................................cAacuuugagucgguacgaauca................  1     1  TA1
.....................................................................cuacuuugagucgguGcgaauca................  3     1  TA1
.....................................................................cuacuuugagucgAuacgaauca................  1     1  TA1
.....................................................................cuacuuugagucggCacgaauca................  1     1  TA1
.....................................................................cuacuuugagucgguacgaauUa................  1     1  TA1
.....................................................................cuacuuugagucgguaUgaauca................  4     1  TA1
.....................................................................cuacuuugagucgguacAaauca................  5     1  TA1
.....................................................................cuacuuCgagucgguacgaauca................  5     1  TA1
.....................................................................cuacuuugagucgguacgaaCca................  5     1  TA1
.....................................................................cuacuuugagucgguacgaauca................  1451  0  TA1
.....................................................................Auacuuugagucgguacgaauca................  3     1  TA1
.....................................................................cuacuuugagucgguacgaGuca................  3     1  TA1
.....................................................................cuaUuuugagucgguacgaauca................  1     1  TA1
.....................................................................cuacuuugagucgguacgaaucC................  3     1  TA1
.....................................................................cuacuuugagAcgguacgaauca................  3     1  TA1
.....................................................................cuacuuugagucgguacgGauca................  9     1  TA1
.....................................................................cuacAuugagucgguacgaauca................  2     1  TA1
.....................................................................cCacuuugagucgguacgaauca................  2     1  TA1
.....................................................................cuacuuugGgucgguacgaauca................  11    1  TA1
.....................................................................cuGcuuugagucgguacgaauca................  2     1  TA1
.....................................................................cuacuuugagucgguacgaaucU................  13    1  TA1
.....................................................................Uuacuuugagucgguacgaauca................  5     1  TA1
.....................................................................cuacuuugGgucgguacgaaucac...............  1     1  TA1
.....................................................................cuacCuugagucgguacgaaucac...............  2     1  TA1
```

Star Mature

```
ggaucaaaauaacuuaacgugguucaggggcgguuuguaccgacucagagcuagguucguugaugagaacuacuuugagucgguacgaaucaccucagccaugaucca
.....................................................................cuacuuugagucgguacgaaucaA...............	34	1	TA1
.....................................................................cuacuuuAagucgguacgaaucac...............	1	1	TA1
.....................................................................cuacuuugagCcgguacgaaucac...............	1	1	TA1
.....................................................................cuacuuugagucgguacgaaucGc...............	1	1	TA1
.....................................................................cuacuuugagucggCacgaaucac...............	1	1	TA1
.....................................................................cuacuuugagucgguacgaaucaG...............	1	1	TA1
.....................................................................cuacuuugagucUguacgaaucac...............	1	1	TA1
.....................................................................cCacuuugagucgguacgaaucac...............	3	1	TA1
.....................................................................cuacuuugagucgguaUgaaucac...............	1	1	TA1
.....................................................................cuacuCugagucgguacgaaucac...............	1	1	TA1
.....................................................................cuacuuugagucgguacgaaucac...............	183	0	TA1
.....................................................................cuacuuugagucgguacgaaucacG..............	1	1	TA1
.....................................................................cuacCuugagucgguacgaaucacc..............	2	1	TA1
.....................................................................cuacuuugagucgguacgGaucacc..............	1	1	TA1
.....................................................................cuacuuugagucgguacgaGucacc..............	1	1	TA1
.....................................................................cuacuuugagucgguacgaaucacA..............	1	1	TA1
.....................................................................cuacuuugagucgguacgaaucacc..............	8	0	TA1
......................................................................uacuuugagucggCacga....................	1	1	TA1
......................................................................uacuuugagucgguacga....................	148	0	TA1
......................................................................uacuuugUgucgguacga....................	1	1	TA1
......................................................................uacuuugGgucgguacga....................	1	1	TA1
......................................................................uaAuuugagucgguacga....................	1	1	TA1
......................................................................uacuuugCgucgguacga....................	1	1	TA1
......................................................................uacuuugagucgguacgU....................	1	1	TA1
......................................................................uGcuuugagucgguacga....................	1	1	TA1
......................................................................uacuuugagucgguacgGa...................	2	1	TA1
......................................................................Cacuuugagucgguacgaa...................	1	1	TA1
......................................................................uacuuugagucgguacgaa...................	163	0	TA1
......................................................................uacuuugagucgguGcgaa...................	1	1	TA1
......................................................................uacuCugagucgguacgaa...................	1	1	TA1
......................................................................uacuuugagucgguacgaG...................	2	1	TA1
......................................................................uacCuugagucgguacgaa...................	2	1	TA1
......................................................................uacuuugagucgguaUgaa...................	1	1	TA1
......................................................................uUcuuugagucgguacgaau..................	1	1	TA1
......................................................................uacuuugagucgguacgaaA..................	57	1	TA1
......................................................................uacuuugaguAgguacgaau..................	1	1	TA1
......................................................................uacuuugagucgAuacgaau..................	1	1	TA1
......................................................................uacuuugagucgguacgaGu..................	20	1	TA1
......................................................................uacCuugagucgguacgaau..................	9	1	TA1
......................................................................uacuuugagucggCacgaau..................	6	1	TA1
......................................................................uacuuugagucAguacgaau..................	14	1	TA1
......................................................................uacuuugagucgguacgGau..................	15	1	TA1
......................................................................uacuuugagucgguacUaau..................	1	1	TA1
......................................................................Cacuuugagucgguacgaau..................	14	1	TA1
......................................................................uacuuugUgucgguacgaau..................	3	1	TA1
......................................................................uacuCugagucgguacgaau..................	13	1	TA1
......................................................................uacuuugagucgguaGgaau..................	1	1	TA1
......................................................................uacuuugagucgguGcgaau..................	3	1	TA1
......................................................................uGcuuugagucgguacgaau..................	6	1	TA1
......................................................................uacuuugCgucgguacgaau..................	1	1	TA1
......................................................................uacuuAgagucgguacgaau..................	1	1	TA1
......................................................................uacuuugagucgguacgaUu..................	2	1	TA1
......................................................................uacuuugagCcgguacgaau..................	9	1	TA1
......................................................................uacuuugagGcgguacgaau..................	2	1	TA1
......................................................................uacuuugagAcgguacgaau..................	1	1	TA1
......................................................................uacuuugagucgguacgaaG..................	41	1	TA1
......................................................................uacuuugagucgguacgaaC..................	258	1	TA1
......................................................................uacuuugagucgguacgaCu..................	1	1	TA1
......................................................................uacuuugagucgguaUgaau..................	2	1	TA1
......................................................................uacAuugagucgguacgaau..................	2	1	TA1
......................................................................uacuuugagucgguacAaau..................	3	1	TA1
......................................................................uacuuuAagucgguacgaau..................	1	1	TA1
......................................................................uacuuugagucgguacgCau..................	2	1	TA1
......................................................................uacuuugagucgguacgaau..................	3245	0	TA1
......................................................................uacuuugagucgguacgUau..................	5	1	TA1
......................................................................uaGuuugagucgguacgaau..................	1	1	TA1
......................................................................uacuuugagucggAacgaau..................	2	1	TA1
......................................................................uacuuugGgucgguacgaau..................	6	1	TA1
......................................................................uacuuCgagucgguacgaau..................	6	1	TA1
......................................................................uacuuugagucgguaUgaauc.................	18	1	TA1
```

```
                                 Star                                 Mature
ggaucaaaauaacuuaacgugguucaggggcgguuuguaccgacucagagcuagguucguugaugagaacuacuuugagucgguacgaaucaccucagccaugaucca
......................................................................uacuuugagCcgguacgaauc.................   52      1   TA1
......................................................................uacuuugagucgguacgaGuc.................   51      1   TA1
......................................................................uacuuugagucgguacgaauA.................   372     1   TA1
......................................................................uacuuugagucggAacgaauc.................   9       1   TA1
......................................................................uacuuuAagucgguacgaauc.................   15      1   TA1
......................................................................uacCuugagucgguacgaauc.................   91      1   TA1
......................................................................uacuuugagucgguGcgaauc.................   38      1   TA1
......................................................................uacuuugagucgguacgaUuc.................   4       1   TA1
......................................................................uacuuugagucgguUcgaauc.................   8       1   TA1
......................................................................uacuuAgagucgguacgaauc.................   19      1   TA1
......................................................................Aacuuugagucgguacgaauc.................   7       1   TA1
......................................................................uacuuugagucgguaAgaauc.................   1       1   TA1
......................................................................uCcuuugagucgguacgaauc.................   3       1   TA1
......................................................................uacuuugagucgUuacgaauc.................   2       1   TA1
......................................................................uacuuugagucgguacgaauc.................   18112   0   TA1
......................................................................uacuuugagucgguaGgaauc.................   3       1   TA1
......................................................................uaUuuugagucgguacgaauc.................   12      1   TA1
......................................................................uacuuugagucgguacgCauc.................   5       1   TA1
......................................................................uacuuugagucgguacgaaGc.................   30      1   TA1
......................................................................uacuuugagucgguacgaauU.................   1140    1   TA1
......................................................................uacuuugagucgguacgaaAc.................   3       1   TA1
......................................................................uacuuugagucgguacgaaCc.................   65      1   TA1
......................................................................uacuuugagucgguacgUauc.................   12      1   TA1
......................................................................uacuuugaguGgguacgaauc.................   2       1   TA1
......................................................................uacuGugagucgguacgaauc.................   1       1   TA1
......................................................................uacuAugagucgguacgaauc.................   11      1   TA1
......................................................................Cacuuugagucgguacgaauc.................   46      1   TA1
......................................................................uacuuGgagucgguacgaauc.................   3       1   TA1
......................................................................uacuuugUgucgguacgaauc.................   6       1   TA1
......................................................................uacuuugagucgguacgGauc.................   68      1   TA1
......................................................................uacAuugagucgguacgaauc.................   7       1   TA1
......................................................................uacuuugagucgguacAaauc.................   17      1   TA1
......................................................................uacuuCgagucgguacgaauc.................   43      1   TA1
......................................................................uacuuugagucgguacgaauG.................   6       1   TA1
......................................................................uacuuugGgucgguacgaauc.................   58      1   TA1
......................................................................Gacuuugagucgguacgaauc.................   5       1   TA1
......................................................................uacuuugagucAguacgaauc.................   20      1   TA1
......................................................................uGcuuugagucgguacgaauc.................   42      1   TA1
......................................................................uacuuugagucgAuacgaauc.................   15      1   TA1
......................................................................uacuuugagucggGacgaauc.................   1       1   TA1
......................................................................uacuuugaguCCguacgaauc.................   1       1   TA1
......................................................................uacuuugagucgguacUaauc.................   7       1   TA1
......................................................................uaAuuugagucgguacgaauc.................   1       1   TA1
......................................................................uacuuugagucgguCcgaauc.................   1       1   TA1
......................................................................uacuuugagAcgguacgaauc.................   9       1   TA1
......................................................................uacuuugagGcgguacgaauc.................   1       1   TA1
......................................................................uacuuuUagucgguacgaauc.................   3       1   TA1
......................................................................uacuCugagucgguacgaauc.................   46      1   TA1
......................................................................uacuuugagucgguacgaCuc.................   6       1   TA1
......................................................................uacuuugagucggCacgaauc.................   29      1   TA1
......................................................................uacuuugagucgCuacgaauc.................   1       1   TA1
......................................................................uacuuugagucUguacgaauc.................   1       1   TA1
......................................................................uacuuugaAucgguacgaauc.................   5       1   TA1
......................................................................uacGuugagucgguacgaauc.................   4       1   TA1
......................................................................uUcuuugagucgguacgaauc.................   13      1   TA1
......................................................................uacuuugaguAgguacgaauc.................   9       1   TA1
......................................................................uacuuugagucggCacgaauca................   97      1   TA1
......................................................................uacuuugagucAguacgaauca................   93      1   TA1
......................................................................uacuuugagucgCuacgaauca................   1       1   TA1
......................................................................uacuuugagAcgguacgaauca................   14      1   TA1
......................................................................uacuuugagucgguacgaauGa................   3       1   TA1
......................................................................uacuuugagucgguaGgaauca................   3       1   TA1
......................................................................uacuuugagucgguacgaGuca................   143     1   TA1
......................................................................uacuuugagucgguacgaaucU................   407     1   TA1
......................................................................uacuuugagucgguacgaUuca................   6       1   TA1
......................................................................uacuuugagucgguacAaauca................   59      1   TA1
......................................................................uacuuugaUucgguacgaauca................   11      1   TA1
......................................................................uacuuugagucgguCcgaauca................   1       1   TA1
......................................................................uacuAugagucgguacgaauca................   18      1   TA1
......................................................................uacuuugagucgguacgUauca................   37      1   TA1
```

Star **Mature**

ggaucaaaauaacuuaacgugguucaggggcgguuuguaccgacucagagcuagguucguugaugagaacuacuuugagucgguacgaaucaccucagccaugaucca			
....uacuuugagucgguacgaaCca................	109	1	TA1
....uacuuugagucgUuacgaauca................	5	1	TA1
....uacuuugagucgguacgGauca................	167	1	TA1
....uacuuugagucggGacgaauca................	3	1	TA1
....uacuuugagucgguaUgaauca................	74	1	TA1
....uacuuugagucgguacgaaucG................	964	1	TA1
....uGcuuugagucgguacgaauca................	96	1	TA1
....uacuuugaguAgguacgaauca................	9	1	TA1
....uacuuugagucgguacgaauUa................	29	1	TA1
....uacuuugagucgguacUaauca................	11	1	TA1
....uacuuGgagucgguacgaauca................	11	1	TA1
....uacuuugGgucgguacgaauca................	200	1	TA1
....uacuuugagucgguacgaaucC................	69	1	TA1
....uacuuugagCcgguacgaauca................	92	1	TA1
....uacuuCgagucgguacgaauca................	152	1	TA1
....uacuuugaCucgguacgaauca................	2	1	TA1
....uacuuugagucgguacgaaGca................	23	1	TA1
....uacuuugaAucgguacgaauca................	20	1	TA1
....uacuuugagucgguacgCauca................	2	1	TA1
....uacuuugCgucgguacgaauca................	5	1	TA1
....uacuuugagucgguacgaauAa................	8	1	TA1
....uacuuugagucUguacgaauca................	6	1	TA1
....uacuuugagucgguacgaaAca................	20	1	TA1
....uacuuugagucCguacgaauca................	3	1	TA1
....uacuuugagucgguUcgaauca................	7	1	TA1
....uacuuugagucgguacCaauca................	3	1	TA1
....uacuGugagucgguacgaauca................	4	1	TA1
....Gacuuugagucgguacgaauca................	7	1	TA1
....uacuuuCagucgguacgaauca................	3	1	TA1
....uacCuugagucgguacgaauca................	204	1	TA1
....uacuuugagucgguaAgaauca................	11	1	TA1
....uacuuugagGcgguacgaauca................	7	1	TA1
....uacuuuUagucgguacgaauca................	7	1	TA1
....uacuuugagucggAacgaauca................	18	1	TA1
....uaAuuugagucgguacgaauca................	8	1	TA1
....uCcuuugagucgguacgaauca................	2	1	TA1
....uacuuugagucgguacgaauca................	50801	0	TA1
....uacuuugaguGgguacgaauca................	2	1	TA1
....uacuuugUgucgguacgaauca................	29	1	TA1
....uacuuugagucgguGcgaauca................	114	1	TA1
....uaUuuugagucgguacgaauca................	21	1	TA1
....uacGuugagucgguacgaauca................	6	1	TA1
....uacuuugagucgAuacgaauca................	42	1	TA1
....uacuCugagucgguacgaauca................	168	1	TA1
....uacuuugagucgguacgaCuca................	4	1	TA1
....uUcuuugagucgguacgaauca................	13	1	TA1
....uacuuAgagucgguacgaauca................	27	1	TA1
....Aacuuugagucgguacgaauca................	45	1	TA1
....uacuuuAagucgguacgaauca................	15	1	TA1
....uacAuugagucgguacgaauca................	14	1	TA1
....Cacuuugagucgguacgaauca................	110	1	TA1
....uacuuugagucgguacgaaucac...............	1289	0	TA1
....uacuuugagucgguacgaaucGc...............	6	1	TA1
....uacuuugagucgguGcgaaucac...............	1	1	TA1
....uGcuuugagucgguacgaaucac...............	2	1	TA1
....uacuuugGgucgguacgaaucac...............	5	1	TA1
....uacuuugagucggCacgaaucac...............	4	1	TA1
....uaAuuugagucgguacgaaucac...............	1	1	TA1
....uacuuugagucgguacgaaucUc...............	8	1	TA1
....uacuCugagucgguacgaaucac...............	3	1	TA1
....uacuuugagucgguacgaGucac...............	5	1	TA1
....uacuuugagucgguacgGaucac...............	4	1	TA1
....uacuuugagucgguacUaaucac...............	6	1	TA1
....uacuuugagucgguacgaaucaG...............	27	1	TA1
....uacuuugagCcgguacgaaucac...............	3	1	TA1
....uacAuugagucgguacgaaucac...............	2	1	TA1
....uacuuugagucgAuacgaaucac...............	1	1	TA1
....Cacuuugagucgguacgaaucac...............	1	1	TA1
....uacuuugagucgguacgaaCcac...............	2	1	TA1
....uacuuAgagucgguacgaaucac...............	2	1	TA1

```
                                Star                                Mature
ggaucaaaauaacuuaacgugguucaggggcgguuuguaccgacucagagcuagguucguugaugagaacuacuuugagucgguacgaaucaccucagccaugaucca
......................................................................uacuuuCagucgguacgaaucac...............   1      1   TA1
......................................................................uacuuugagucgguaUgaaucac...............   1      1   TA1
......................................................................uacCuugagucgguacgaaucac...............   12     1   TA1
......................................................................uacuuugagucAguacgaaucac...............   1      1   TA1
......................................................................uacuuugagucgguacgaaucaA...............   1376   1   TA1
......................................................................uacuuCgagucgguacgaaucac...............   5      1   TA1
......................................................................uacGuugagucgguacgaaucac...............   1      1   TA1
......................................................................Gacuuugagucgguacgaaucac...............   1      1   TA1
......................................................................uacuuugUgucgguacgaaucac...............   1      1   TA1
......................................................................uacuuugagucgguacAaaucac...............   1      1   TA1
......................................................................uacuuugagucgguacgaaucacA..............   178    1   TA1
......................................................................uacuuugagCcgguacgaaucacc..............   1      1   TA1
......................................................................uacuuugagucgguacgaaucUcc..............   1      1   TA1
......................................................................uacuuugagucgguacgaaucacG..............   14     1   TA1
......................................................................uacuuugagucggCacgaaucacc..............   2      1   TA1
......................................................................Cacuuugagucgguacgaaucacc..............   2      1   TA1
......................................................................uacuuugagucgguacgaaucacc..............   63     0   TA1
......................................................................uacuuugagucgguacgaaucacAu.............   2      1   TA1
......................................................................uacuuugagucgguacgaaucaccC.............   3      1   TA1
......................................................................uacuuugagucgguacgaaucaccA.............   1      1   TA1
......................................................................uacuuugagucgguacgaaucaccu.............   1      0   TA1
.......................................................................acuuugagucgguacgaau..................   1      0   TA1
.......................................................................acuuugagucgguacgaauc.................   11     0   TA1
.......................................................................acuuugagucgguacgaaucU................   2      1   TA1
.......................................................................acuuugagucgguacgaaucG................   1      1   TA1
.......................................................................acuCugagucgguacgaauca................   1      1   TA1
.......................................................................acuuugagucgguacgaauca................   37     0   TA1
.......................................................................acuuugagucgguacgaaucaA...............   3      1   TA1
.......................................................................acuuugagucgguacgaaucac...............   12     0   TA1
........................................................................cuuugagucgguacgaauc.................   2      0   TA1
........................................................................cuuugagucgguacgaauA.................   1      1   TA1
........................................................................cuuugagucgguacgaauca................   11     0   TA1
........................................................................cuuugagucgguacgaaucU................   1      1   TA1
........................................................................cuuugagucgguacgaaucacA..............   1      1   TA1
.........................................................................uuugagucgguacgaauc.................   3      0   TA1
.........................................................................uuugagucgguacgaauca................   11     0   TA1
.........................................................................uuugagucgguacgaaucG................   1      1   TA1
.........................................................................uuugagucgguacgGauca................   2      1   TA1
.........................................................................uuugagucgguacgaaucac...............   1      0   TA1
.........................................................................uuugagucgguacgaaucacA..............   2      1   TA1
.........................................................................uuugagucgguacgaaucacc..............   8      0   TA1
.........................................................................uuugagucgguacgaaucaccu.............   2      0   TA1
..........................................................................uugagucgguacgaauca................   9      0   TA1
..........................................................................uugagucgguacgaaucaA...............   1      1   TA1

...............................gguuuguaccgacucagagcu........................................................   7      0   E01
...............................gguuuguaccgacucagagcua.......................................................   1      0   E01
...............................gguuuguaccgacucagagcuag......................................................   1      0   E01
...............................gguuuguaccgacucagagcuaggu....................................................   8      0   E01
...............................gguuuguaccgacucagagcuagguu...................................................   3      0   E01
................................guuuguaccgacucagagcuaggu....................................................   1      0   E01
.................................uuuguaccgacucagagcu........................................................   1      0   E01
.................................uuuguaccgacucagagcAagg.....................................................   1      1   E01
.................................uuuguaccgacucagagcuagg.....................................................   3      0   E01
.................................uuuguaccgacucagagcuaggu....................................................   5      0   E01
.................................uuuguaccgacucagagcuagguA...................................................   1      1   E01
..................................uuguaccgacucagagcuaggu....................................................   1      0   E01
..................................uuguaccgacucagagcuagguu...................................................   1      0   E01
..................................................................gaaAuacuuugagucgguacgaauca................   1      1   E01
....................................................................acuacuuugagucgguacgaauca................   1      0   E01
.....................................................................cuacuuugagucgguacgaau..................   3      0   E01
.....................................................................Uuacuuugagucgguacgaau..................   1      1   E01
.....................................................................cuacuuugagucgguacgaauc.................   4      0   E01
.....................................................................Auacuuugagucgguacgaauca................   2      1   E01
.....................................................................cuacuuugagucgguacgaauca................   203    0   E01
.....................................................................cuacuuugCgucgguacgaauca................   1      1   E01
.....................................................................Uuacuuugagucgguacgaauca................   3      1   E01
.....................................................................cuacuuugagucgguacgaaucaA...............   4      1   E01
.....................................................................cuacuuugagucgguacgaaucac...............   92     0   E01
.....................................................................Guacuuugagucgguacgaaucac...............   1      1   E01
```

```
                                 Star                                  Mature
ggaucaaaauaacuuaacgugguucaggggcgguuuguaccgacucagagcuagguucguugaugagaacuacuuugagucgguacgaaucaccucagccaugaucca
.....................................................................cuacuuugagucgguacgaaucaG...............    1      1      E01
.....................................................................Uuacuuugagucgguacgaaucac...............    5      1      E01
.....................................................................cuacuuugagucgguacgaaAcac...............    1      1      E01
.....................................................................cuacuuugagucgguacgaCucac...............    1      1      E01
.....................................................................cuacuuugagucgguacgaaucacc..............    1      0      E01
......................................................................uacuuugagucgguacga....................    7      0      E01
......................................................................uacuuugagucgguacgaa...................    9      0      E01
......................................................................uacuuugagucgguacgaau..................    266    0      E01
......................................................................uacuuugagucgguacgaaA..................    5      1      E01
......................................................................uacuuugagucgguacAaau..................    2      1      E01
......................................................................uacuuugagucgguGcgaauc.................    1      1      E01
......................................................................Cacuuugagucgguacgaauc.................    1      1      E01
......................................................................uacuuugagucgguacgaauG.................    1      1      E01
......................................................................uacuuugagucgguacgaauU.................    1      1      E01
......................................................................uacuuugagucgguacUaauc.................    1      1      E01
......................................................................uCcuuugagucgguacgaauc.................    1      1      E01
......................................................................uacuuugagucgguacgaauA.................    1      1      E01
......................................................................uacuGugagucgguacgaauc.................    1      1      E01
......................................................................uacuuugagucgguacgaauc.................    726    0      E01
......................................................................uaUuuugagucgguacgaauc.................    1      1      E01
......................................................................uacuuugagCcgguacgaauca................    2      1      E01
......................................................................uacuuugagucUguacgaauca................    1      1      E01
......................................................................uacuuugagucgguacgGauca................    1      1      E01
......................................................................uacuuugagucgguacgaUuca................    1      1      E01
......................................................................uacuuugagAcgguacgaauca................    1      1      E01
......................................................................uGcuuugagucgguacgaauca................    4      1      E01
......................................................................uacuuugCgucgguacgaauca................    3      1      E01
......................................................................uacuuugagucgguacgUauca................    2      1      E01
......................................................................uacuuugagucgguacCaauca................    1      1      E01
......................................................................uacCuugagucgguacgaauca................    3      1      E01
......................................................................uacuuugagucgCuacgaauca................    3      1      E01
......................................................................uacuuugagucgguaUgaauca................    2      1      E01
......................................................................uacuuugagucgguacgaaucU................    26     1      E01
......................................................................uacuuugagucgUuacgaauca................    1      1      E01
......................................................................uacuuugagucgguacgaaucG................    2      1      E01
......................................................................uacuuugGgucgguacgaauca................    6      1      E01
......................................................................uacuuugagucgguCcgaauca................    2      1      E01
......................................................................uacuuugagucggCacgaauca................    3      1      E01
......................................................................uacuuugagucgguacgaauca................    9219   0      E01
......................................................................uacuuugagucgguacgaauGa................    7      1      E01
......................................................................uacuuugagucgguacgCauca................    2      1      E01
......................................................................uacuuugagucgguacgaauUa................    2      1      E01
......................................................................uaUuuugagucgguacgaauca................    1      1      E01
......................................................................uacuuugagucgguacgaaCca................    1      1      E01
......................................................................uacuuugagucggAacgaauca................    1      1      E01
......................................................................uacuuugagucgguacUaauca................    1      1      E01
......................................................................uacuuugagucgguGcgaauca................    11     1      E01
......................................................................uacuuugagucgguUcgaauca................    1      1      E01
......................................................................uacuuugagucgguacgaaGca................    1      1      E01
......................................................................uacuuugagucgAuacgaauca................    1      1      E01
......................................................................uUcuuugagucgguacgaauca................    5      1      E01
......................................................................uacuuugUgucgguacgaauca................    10     1      E01
......................................................................uacuuugagucgguacgaCuca................    1      1      E01
......................................................................uacuuugaUucgguacgaaucac...............    1      1      E01
......................................................................uGcuuugagucgguacgaaucac...............    1      1      E01
......................................................................uCcuuugagucgguacgaaucac...............    2      1      E01
......................................................................uacuuugagucgguacgaauUac...............    2      1      E01
......................................................................uacuuugagucgguaUgaaucac...............    2      1      E01
......................................................................uacuuugagucgguacgaaGcac...............    1      1      E01
......................................................................uacuuugagucgguacgaUucac...............    1      1      E01
......................................................................uacCuugagucgguacgaaucac...............    1      1      E01
......................................................................uacuuugagucgguacgaaucaA...............    104    1      E01
......................................................................uacuuugagucgguacgaGucac...............    3      1      E01
......................................................................uacuuugagucgguCcgaaucac...............    1      1      E01
......................................................................uacuuugUgucgguacgaaucac...............    9      1      E01
......................................................................uacuuugGgucgguacgaaucac...............    2      1      E01
......................................................................uacuuugagucgguacgaaucac...............    5537   0      E01
......................................................................Aacuuugagucgguacgaaucac...............    1      1      E01
......................................................................uacuGugagucgguacgaaucac...............    1      1      E01
......................................................................uacuuugagucgguacgaauAac...............    2      1      E01
```

```
                                Star                                 Mature
ggaucaaaauaacuuaacgugguucaggggcgguuuguaccgacucagagcuagguucguugaugagaacuacuuugagucgguacgaaucaccucagccaugaucca
......................................................................uacuuugCgucgguacgaaucac...............	6	1	E01
......................................................................uacuuugagucgguUcgaaucac...............	3	1	E01
......................................................................uacuuugagucUguacgaaucac...............	3	1	E01
......................................................................uacuuugagucgguGcgaaucac...............	8	1	E01
......................................................................uacuuugagucgUuacgaaucac...............	2	1	E01
......................................................................uUcuuugagucgguacgaaucac...............	1	1	E01
......................................................................uacuuugagucgguacgaaCcac...............	1	1	E01
......................................................................uacuuugagucgguacgaaucacA..............	50	1	E01
......................................................................uacuuugagucgguacgaaucaccu.............	1	0	E01
......................................................................uacuuugagucgguacgaaucaccA.............	1	1	E01
.......................................................................acuuugagucgguacgaau..................	1	0	E01
.......................................................................acuuugagucgguacgaauc.................	2	0	E01
.......................................................................acuuugagucgguacgaaucU................	1	1	E01
.......................................................................acuuugagucgguacgaauca................	14	0	E01
.......................................................................acuuugagucgguacgaaucac...............	33	0	E01
........................................................................cuuugagucgguacgaauca................	4	0	E01
........................................................................cuuugagucgguacgaaucac...............	7	0	E01
.........................................................................uuugagucgguacgaauca................	5	0	E01
.........................................................................uuugagucgguacgaaucac...............	3	0	E01
.........................................................................uuugagucgguacgaaucacA..............	1	1	E01
.........................................................................uuugagucgguacgaaucaccA.............	4	1	E01
..........................................................................uugagucgguacgaauca................	3	0	E01
..........................................................................uugagucgguacgaaucac...............	7	0	E01
...........................................................................ugagucgguacgaaucac...............	2	0	E01

...............................gguuuguaccgacucagagcuagg.....................................................	1	0	LJ1
.................................uuuguaccgacucagagcG........................................................	1	1	LJ1
.................................uuuguaccgacucagagcuaggu....................................................	1	0	LJ1
....................................................................aAuacuuugagucgguacgaau..................	1	1	LJ1
.....................................................................cuacuuugagucgguacg.....................	1	0	LJ1
.....................................................................cuacuuugagucgguacgaau..................	1	0	LJ1
.....................................................................cuacuuugagucgguacgaauA.................	1	1	LJ1
.....................................................................cuacuuugagucgguacgaauc.................	10	0	LJ1
.....................................................................cuacuuugagucgguGcgaauc.................	1	1	LJ1
.....................................................................cCacuuugagucgguacgaauca................	1	1	LJ1
.....................................................................cuacuCugagucgguacgaauca................	1	1	LJ1
.....................................................................cuacuuugagucgguacgaauca................	19	0	LJ1
.....................................................................cuacuuugagucgguacgGauca................	1	1	LJ1
.....................................................................cuacuuugagucgguacgaaucac...............	12	0	LJ1
.....................................................................Uuacuuugagucgguacgaaucac...............	1	1	LJ1
.....................................................................cuacuuugagucgguacgaaucaA...............	2	1	LJ1
.....................................................................cuacuuugagucgguacgaaucacA..............	1	1	LJ1
......................................................................uacuuugagucgguacgU....................	1	1	LJ1
......................................................................uacuuugagucgguacga....................	28	0	LJ1
......................................................................uacCuugagucgguacga....................	1	1	LJ1
......................................................................uacuuugagucgguacCa....................	1	1	LJ1
......................................................................Cacuuugagucgguacga....................	1	1	LJ1
......................................................................uacuuugagucgguacgaa...................	8	0	LJ1
......................................................................uacuuugagucgguacgaU...................	2	1	LJ1
......................................................................uacuuugagucgguacgaaG..................	3	1	LJ1
......................................................................uacuuugagucgguacgaau..................	145	0	LJ1
......................................................................uacuuugagucgguacgaaA..................	2	1	LJ1
......................................................................uacuuCgagucgguacgaau..................	2	1	LJ1
......................................................................uacAuugagucgguacgaau..................	1	1	LJ1
......................................................................uacCuugagucgguacgaau..................	1	1	LJ1
......................................................................uacuuugagucgguacgaaC..................	8	1	LJ1
......................................................................uacuuugagCcgguacgaauc.................	1	1	LJ1
......................................................................uaUuuugagucgguacgaauc.................	2	1	LJ1
......................................................................uacuuugagucgguacgGauc.................	2	1	LJ1
......................................................................uacuuugagucgguacgCauc.................	1	1	LJ1
......................................................................uacuuugaUucgguacgaauc.................	1	1	LJ1
......................................................................uacCuugagucgguacgaauc.................	1	1	LJ1
......................................................................uacuuugagucgguacgaauA.................	16	1	LJ1
......................................................................uacuuugagucgguacgUauc.................	1	1	LJ1
......................................................................Cacuuugagucgguacgaauc.................	1	1	LJ1
......................................................................uacuuugagucgguacgaauc.................	537	0	LJ1
......................................................................uacuuugagucgguacUaauc.................	1	1	LJ1
......................................................................uacuuugagucgguaUgaauc.................	4	1	LJ1
......................................................................uacuuugagucAguacgaauc.................	3	1	LJ1
......................................................................uacuuAgagucgguacgaauc.................	3	1	LJ1
```

```
                                Star                               Mature
ggaucaaaauaacuuaacgugguucaggggcgguuuguaccgacucagagcuagguucguugaugagaacuacuuugagucgguacgaaucaccucagccaugaucca
......................................................................uaGuuugagucgguacgaauc.................   1     1     LJ1
......................................................................uacuuugagucggCacgaauc.................   2     1     LJ1
......................................................................uacuuCgagucgguacgaauc.................   2     1     LJ1
......................................................................uacuuugagucgguacgaauU.................   26    1     LJ1
......................................................................uacuuugagucgAuacgaauc.................   1     1     LJ1
......................................................................uacuuugagucgCuacgaauc.................   1     1     LJ1
......................................................................uacuuugagucgguacgaGuc.................   1     1     LJ1
......................................................................uaAuuugagucgguacgaauc.................   1     1     LJ1
......................................................................uacuuugGgucgguacgaauc.................   3     1     LJ1
......................................................................uacuuugGgucgguacgaauca................   4     1     LJ1
......................................................................uacuuugagucgguacgaaCca................   9     1     LJ1
......................................................................uacuuugaAucgguacgaauca................   1     1     LJ1
......................................................................uacuuugagucgguacAaauca................   3     1     LJ1
......................................................................uacuuugagucgCuacgaauca................   1     1     LJ1
......................................................................uaUuuugagucgguacgaauca................   2     1     LJ1
......................................................................uacuuAgagucgguacgaauca................   1     1     LJ1
......................................................................uacuuugagucgguacgaGuca................   2     1     LJ1
......................................................................uacuuugagucgUuacgaauca................   1     1     LJ1
......................................................................uUcuuugagucgguacgaauca................   1     1     LJ1
......................................................................uacuuugagucgAuacgaauca................   1     1     LJ1
......................................................................uacuuuCagucgguacgaauca................   1     1     LJ1
......................................................................uacuuugagucgguacgaaucU................   16    1     LJ1
......................................................................uacCuugagucgguacgaauca................   3     1     LJ1
......................................................................uacuuugagucggCacgaauca................   3     1     LJ1
......................................................................uacuuCgagucgguacgaauca................   3     1     LJ1
......................................................................uacuuugagucgguacgaaucC................   4     1     LJ1
......................................................................uaAuuugagucgguacgaauca................   2     1     LJ1
......................................................................uacuuugagucgguacgaauca................   897   0     LJ1
......................................................................uacuuugagucgguacgGauca................   2     1     LJ1
......................................................................uacuuugagucgguaUgaauca................   2     1     LJ1
......................................................................uacuuugagucgguGcgaauca................   6     1     LJ1
......................................................................uacuuugagCcgguacgaauca................   1     1     LJ1
......................................................................uGcuuugagucgguacgaauca................   1     1     LJ1
......................................................................uacuCugagucgguacgaauca................   2     1     LJ1
......................................................................uacuuugagucgguacgaaucG................   20    1     LJ1
......................................................................uacuuugagucggAacgaauca................   1     1     LJ1
......................................................................uacuuugUgucgguacgaaucac...............   1     1     LJ1
......................................................................uacuuugagucggCacgaaucac...............   1     1     LJ1
......................................................................uacuuugagucgguacgaaucaA...............   49    1     LJ1
......................................................................uacuuugagucUguacgaaucac...............   1     1     LJ1
......................................................................uacuuugagucgguacgaGucac...............   1     1     LJ1
......................................................................uacuuuAagucgguacgaaucac...............   1     1     LJ1
......................................................................uacuuugagCcgguacgaaucac...............   1     1     LJ1
......................................................................uacCuugagucgguacgaaucac...............   2     1     LJ1
......................................................................uacuuugagucgguacgaaucac...............   91    0     LJ1
......................................................................uacuuugagucgguacgaaucacA..............   12    1     LJ1
......................................................................uacuuugagucgguacgaaucacc..............   14    0     LJ1
......................................................................uacuuugagucgguacgaaucacG..............   1     1     LJ1
.......................................................................Ucuuugagucgguacgaauc.................   1     1     LJ1
.......................................................................acuuugagucgguacgaauca................   2     0     LJ1
.......................................................................acuuugagucgguacgaaucac...............   1     0     LJ1
.........................................................................uuugagucgguacgaaucacc..............   1     0     LJ1
.........................................................................uuugagucgguacgaaucacA..............   2     1     LJ1
.........................................................................uuugagucgguacgaaucaccA.............   1     1     LJ1
.........................................................................uuugagucgguacgaaucaccu.............   2     0     LJ1

.................................uuuguaccgacucagagcuagg.....................................................   1     0     CJ1
.................................uuuguaccgacucagagcuaggC....................................................   1     1     CJ1
.................................uuuguaccgacucagagcuaggu....................................................   7     0     CJ1
..................................uuguaccgacucagagcuagguuU..................................................   1     1     CJ1
.....................................................................cuacuuugagucgguacga....................   1     0     CJ1
.....................................................................cuacuuugagucgguacgaa...................   2     0     CJ1
.....................................................................cuacuuugagucgguacgaau..................   3     0     CJ1
.....................................................................cuacuuugagucgguacgaauU.................   2     1     CJ1
.....................................................................cuacuuugagucgguacgaauc.................   8     0     CJ1
.....................................................................cuacuuugagucgguacgaauca................   32    0     CJ1
.....................................................................cuacuCugagucgguacgaauca................   1     1     CJ1
.....................................................................cuacuuugagucgguacgaaucac...............   14    0     CJ1
.....................................................................cuacuuugagucgguGcgaaucac...............   2     1     CJ1
......................................................................uacuuugagucgguGcga....................   1     1     CJ1
```

Star **Mature**

```
ggaucaaaauaacuuaacgugguucaggggcgguuuguaccgacucagagcuagguucguugaugagaacuacuuugagucgguacgaaucaccucagccaugaucca
......................................................................uacuuugagucgguacgU....................  2     1  CJ1
......................................................................uacuuugagucgguacga....................  24    0  CJ1
......................................................................uacuuugagucgguacgaa...................  9     0  CJ1
......................................................................uacuuugagucgguacgaU...................  1     1  CJ1
......................................................................uacuuugagucgguacgaUu..................  1     1  CJ1
......................................................................uaAuuugagucgguacgaau..................  2     1  CJ1
......................................................................uacuuugagucgguGcgaau..................  3     1  CJ1
......................................................................uacuuugagucgguacgaaA..................  4     1  CJ1
......................................................................uacuuugagucgguacgaau..................  178   0  CJ1
......................................................................uacuuCgagucgguacgaau..................  1     1  CJ1
......................................................................uacuuugagucgguacgaaC..................  17    1  CJ1
......................................................................uacuuugagucgguacgGau..................  3     1  CJ1
......................................................................uacuuugGgucgguacgaau..................  1     1  CJ1
......................................................................uacuCugagucgguacgaau..................  1     1  CJ1
......................................................................uacAuugagucgguacgaau..................  1     1  CJ1
......................................................................uacuuugagAcgguacgaauc.................  1     1  CJ1
......................................................................uacuuugagucgguacgaGuc.................  3     1  CJ1
......................................................................uacuuugagucgguacgUauc.................  1     1  CJ1
......................................................................uGcuuugagucgguacgaauc.................  2     1  CJ1
......................................................................uacuuugagucgguaUgaauc.................  2     1  CJ1
......................................................................uacuuuUagucgguacgaauc.................  1     1  CJ1
......................................................................uacuuAgagucgguacgaauc.................  4     1  CJ1
......................................................................uacCuugagucgguacgaauc.................  2     1  CJ1
......................................................................uacuuugagucgguacgaaCc.................  3     1  CJ1
......................................................................uacuuuAagucgguacgaauc.................  1     1  CJ1
......................................................................uacuuugagucgguacgaauG.................  1     1  CJ1
......................................................................uacuuugagucgguacgGauc.................  3     1  CJ1
......................................................................uacuuugagucgguUcgaauc.................  1     1  CJ1
......................................................................uacuuugagucgguacgaauc.................  563   0  CJ1
......................................................................uacuuugGgucgguacgaauc.................  2     1  CJ1
......................................................................uacuuugaguAgguacgaauc.................  1     1  CJ1
......................................................................uacuuugagucgguacgaauA.................  18    1  CJ1
......................................................................uaAuuugagucgguacgaauc.................  2     1  CJ1
......................................................................uacuuugagucgguacgaaGc.................  1     1  CJ1
......................................................................uacuuugagucgguacgaauU.................  35    1  CJ1
......................................................................uacuuugagCcgguacgaauc.................  2     1  CJ1
......................................................................uUcuuugagucgguacgaauca................  1     1  CJ1
......................................................................uacuuugagucgAuacgaauca................  1     1  CJ1
......................................................................uacuuugagCcgguacgaauca................  3     1  CJ1
......................................................................uacuuCgagucgguacgaauca................  1     1  CJ1
......................................................................uacuuugaAucgguacgaauca................  1     1  CJ1
......................................................................uacuuugagucgguaUgaauca................  1     1  CJ1
......................................................................uacCuugagucgguacgaauca................  7     1  CJ1
......................................................................Cacuuugagucgguacgaauca................  3     1  CJ1
......................................................................uaUuuugagucgguacgaauca................  2     1  CJ1
......................................................................uacuuugaUucgguacgaauca................  2     1  CJ1
......................................................................uacuuugagucggCacgaauca................  2     1  CJ1
......................................................................uGcuuugagucgguacgaauca................  6     1  CJ1
......................................................................uacuuugagucgguacgaaucU................  18    1  CJ1
......................................................................uacuuugagucgguacgaaucG................  24    1  CJ1
......................................................................uacuuuUagucgguacgaauca................  1     1  CJ1
......................................................................uacuuugagucgguacAaauca................  1     1  CJ1
......................................................................uacuuugagucgguacgGauca................  2     1  CJ1
......................................................................uaGuuugagucgguacgaauca................  1     1  CJ1
......................................................................uacuCugagucgguacgaauca................  3     1  CJ1
......................................................................uCcuuugagucgguacgaauca................  1     1  CJ1
......................................................................uaAuuugagucgguacgaauca................  6     1  CJ1
......................................................................uacuuAgagucgguacgaauca................  4     1  CJ1
......................................................................uacuuugagucAguacgaauca................  1     1  CJ1
......................................................................uacuuugagucgguGcgaauca................  4     1  CJ1
......................................................................uacuuugUgucgguacgaauca................  1     1  CJ1
......................................................................uacuuugGgucgguacgaauca................  5     1  CJ1
......................................................................Gacuuugagucgguacgaauca................  1     1  CJ1
......................................................................uacuuugagucgguacgaauca................  1577  0  CJ1
......................................................................uacuAugagucgguacgaauca................  1     1  CJ1
......................................................................uacuuugagucgguacgaaCca................  5     1  CJ1
......................................................................uacuuuCagucgguacgaauca................  3     1  CJ1
......................................................................uacuuuAagucgguacgaauca................  2     1  CJ1
......................................................................uacuuugaguAgguacgaauca................  2     1  CJ1
......................................................................uacAuugagucgguacgaauca................  1     1  CJ1
```

```
                                  Star                                    Mature
ggaucaaaauaacuuaacgugguucaggggcgguuuguaccgacucagagcuagguucguugaugagaacuacuuugagucgguacgaaucaccucagccaugaucca
......................................................................uacuuugagucgguacUaauca................	1	1	CJ1
......................................................................uacuuugagucgguacgaGucac...............	1	1	CJ1
......................................................................uacuuugagucgguacgaaucac...............	87	0	CJ1
......................................................................uacuuugagucgguacgaaucaA...............	54	1	CJ1
......................................................................uacuuugGgucgguacgaaucac...............	2	1	CJ1
......................................................................uacuuugagucgguacgaaucGc...............	3	1	CJ1
......................................................................uaAuuugagucgguacgaaucac...............	1	1	CJ1
......................................................................uacuuugagucgguacgaaucaG...............	2	1	CJ1
......................................................................uacuuugagucgguacgaaCcac...............	1	1	CJ1
......................................................................uacCuugagucgguacgaaucacc..............	1	1	CJ1
......................................................................uacuuugagucgguacgaaucacc..............	19	0	CJ1
......................................................................uacuuugagucgguacgaaucaAc..............	1	1	CJ1
......................................................................uacuuugagucgguacgaaucacA..............	14	1	CJ1
......................................................................uacuuugagucgguacgaaucacAu.............	1	1	CJ1
......................................................................uacuuugagucgguacgaaucaccA.............	1	1	CJ1
.......................................................................acuuugagucgguacgaauca................	3	0	CJ1
.......................................................................acuuugagucgguacgaaucac...............	1	0	CJ1
.......................................................................acuuugagucgguacgaaucacA..............	1	1	CJ1
........................................................................cuuugagucgguacgaauca................	1	0	CJ1

...............................gguuuguaccgacucagagcu........................................................	1	0	G01
...............................gguuuguaccgacucagagcuag......................................................	1	0	G01
...............................gguuuguaccgacucagagcuagg.....................................................	1	0	G01
...............................gguuuguaccgacucagagcuaggu....................................................	6	0	G01
...............................gguuuguaccgacucagagcuagguu...................................................	1	0	G01
.................................uuuguaccgacucagagcu........................................................	1	0	G01
.................................uuuguaccgacucagagcuag......................................................	3	0	G01
.................................uuuguaccgacucagagcuagg.....................................................	3	0	G01
.................................uuuguaccgacucagagcuaCgu....................................................	1	1	G01
.................................uuuguaccgacucagagcuaggu....................................................	41	0	G01
.................................uuuguaccgacucagagcuaggA....................................................	3	1	G01
.................................uuuguaccgacucagagcuagguu...................................................	5	0	G01
..................................uuguaccgacucagagcuagguuc..................................................	1	0	G01
....................................................................Ccuacuuugagucgguacgaau..................	1	1	G01
....................................................................Ucuacuuugagucgguacgaauc.................	1	1	G01
....................................................................Ccuacuuugagucgguacgaauca................	1	1	G01
....................................................................aUuacuuugagucgguacgaauca................	1	1	G01
....................................................................acuacuuugagucgguacgaauca................	1	0	G01
.....................................................................cuacuuugagucgguacg.....................	7	0	G01
.....................................................................Uuacuuugagucgguacg.....................	2	1	G01
.....................................................................cuacuuugagucgguacga....................	2	0	G01
.....................................................................cuacuuugagucgguacgaa...................	5	0	G01
.....................................................................cuacuuugagucgguacgaau..................	41	0	G01
.....................................................................Uuacuuugagucgguacgaau..................	4	1	G01
.....................................................................cuacuuugUgucgguacgaau..................	1	1	G01
.....................................................................Uuacuuugagucgguacgaauc.................	4	1	G01
.....................................................................cuacuuugGgucgguacgaauc.................	1	1	G01
.....................................................................cuacuuugagucgguacgaGuc.................	1	1	G01
.....................................................................cuacuuugagucgguacgaauc.................	102	0	G01
.....................................................................cuacuuugagucgguacgaaGca................	1	1	G01
.....................................................................cuacuuugCgucgguacgaauca................	1	1	G01
.....................................................................cuacuuugagucggCacgaauca................	1	1	G01
.....................................................................cuacuuugaUucgguacgaauca................	1	1	G01
.....................................................................cuacuuugagucgguacgaaucG................	1	1	G01
.....................................................................cuacuuugagucgguGcgaauca................	5	1	G01
.....................................................................cuacuuugUgucgguacgaauca................	11	1	G01
.....................................................................cuacuuugagucgguacgaaucU................	8	1	G01
.....................................................................Uuacuuugagucgguacgaauca................	25	1	G01
.....................................................................cuacuuugaguGgguacgaauca................	2	1	G01
.....................................................................cuacuuuUagucgguacgaauca................	1	1	G01
.....................................................................cuacuuugagucUguacgaauca................	1	1	G01
.....................................................................cuacuuugGgucgguacgaauca................	4	1	G01
.....................................................................cuacCuugagucgguacgaauca................	3	1	G01
.....................................................................cuacuuugagucgguacgaauca................	1841	0	G01
.....................................................................cAacuuugagucgguacgaauca................	2	1	G01
.....................................................................cuacuuugagucgguacgCauca................	1	1	G01
.....................................................................cuacuuugUgucgguacgaaucac...............	1	1	G01
.....................................................................cuacuuugagucgguacgaauUac...............	1	1	G01
.....................................................................cuacuuugagucgguGcgaaucac...............	1	1	G01
.....................................................................cuacuuugagucgguacgaaucaG...............	3	1	G01
```

Star **Mature**

```
ggaucaaaauaacuuaacgugguucaggggcgguuuguaccgacucagagcuagguucguugaugagaacuacuuugagucgguacgaaucaccucagccaugaucca
.....................................................................cuacuuuUagucgguacgaaucac...............  1     1  G01
.....................................................................Auacuuugagucgguacgaaucac...............  2     1  G01
.....................................................................cuacuuugagucgguacgaaucaA...............  49    1  G01
.....................................................................cuacuuugagucgguacgaaucGc...............  1     1  G01
.....................................................................Uuacuuugagucgguacgaaucac...............  14    1  G01
.....................................................................cuacuuugaCucgguacgaaucac...............  1     1  G01
.....................................................................cuaUuuugagucgguacgaaucac...............  1     1  G01
.....................................................................cuacuuugagucgguacAaaucac...............  1     1  G01
.....................................................................cuUcuuugagucgguacgaaucac...............  1     1  G01
.....................................................................cuacuuugagucgguacgaaucac...............  592   0  G01
.....................................................................cuacuuugGgucgguacgaaucac...............  1     1  G01
.....................................................................cuacuuugagucgguacgaaucacA..............  2     1  G01
.....................................................................cuacuuugagucgguacgaaucacc..............  1     0  G01
.....................................................................cuacuuugagucgguacgaaucacGu.............  1     1  G01
......................................................................uacuuugagucgguGcga....................  2     1  G01
......................................................................uacuuugagucgguacga....................  86    0  G01
......................................................................uacuuugagucgguacgaU...................  1     1  G01
......................................................................uacuuugUgucgguacgaa...................  1     1  G01
......................................................................uacuuugagucgguacgaa...................  59    0  G01
......................................................................uacuuugUgucgguacgaau..................  4     1  G01
......................................................................uacuuugagCcgguacgaau..................  1     1  G01
......................................................................uacuuugagucgguacAaau..................  1     1  G01
......................................................................uGcuuugagucgguacgaau..................  1     1  G01
......................................................................uacuuugagAcgguacgaau..................  1     1  G01
......................................................................uacuuugagucgguaUgaau..................  1     1  G01
......................................................................uacuuugagucgguacgaaA..................  74    1  G01
......................................................................uacCuugagucgguacgaau..................  1     1  G01
......................................................................uacuuugagucgguacgaaC..................  2     1  G01
......................................................................uacuuugCgucgguacgaau..................  2     1  G01
......................................................................uacuuugagucgguGcgaau..................  5     1  G01
......................................................................uacuuugagucgguacgaUu..................  3     1  G01
......................................................................uacuuugagucgguacgaaG..................  2     1  G01
......................................................................uUcuuugagucgguacgaau..................  2     1  G01
......................................................................uacuuugagucUguacgaau..................  1     1  G01
......................................................................uacuuugagucgguacgaau..................  1972  0  G01
......................................................................uacuuugagucgUuacgaau..................  2     1  G01
......................................................................uacuuugagucgAuacgaau..................  1     1  G01
......................................................................uacuuugagucgguacgaaCc.................  4     1  G01
......................................................................uacuuugGgucgguacgaauc.................  3     1  G01
......................................................................uacuuugagucgguacgaauc.................  2775  0  G01
......................................................................uacuuugagucgguUcgaauc.................  1     1  G01
......................................................................uacuuugagucgguacgaauA.................  27    1  G01
......................................................................uacuuugUgucgguacgaauc.................  3     1  G01
......................................................................uacuuugagucgguacgGauc.................  4     1  G01
......................................................................uacCuugagucgguacgaauc.................  2     1  G01
......................................................................uacuuugagucgguacgaauU.................  19    1  G01
......................................................................uGcuuugagucgguacgaauc.................  1     1  G01
......................................................................uacuuugagucgguaUgaauc.................  1     1  G01
......................................................................uacuuugagucgguacgaauG.................  3     1  G01
......................................................................uUcuuugagucgguacgaauc.................  1     1  G01
......................................................................uacuuugagucgguCcgaauc.................  1     1  G01
......................................................................uacuuugagucgguacgaGuc.................  1     1  G01
......................................................................uacuuugagucgguGcgaauc.................  3     1  G01
......................................................................uacuCugagucgguacgaauc.................  1     1  G01
......................................................................uacuuugagucAguacgaauc.................  1     1  G01
......................................................................uacuuugagucgCuacgaauc.................  1     1  G01
......................................................................uacuuugagucgguacgaaAc.................  1     1  G01
......................................................................uacuuugagucgguacgaaGca................  4     1  G01
......................................................................uacuuugagucggAacgaauca................  6     1  G01
......................................................................uacuuugGgucgguacgaauca................  6     1  G01
......................................................................uGcuuugagucgguacgaauca................  7     1  G01
......................................................................uacuuugagucgguacgaGuca................  2     1  G01
......................................................................uacuuugagucgguacgaauAa................  12    1  G01
......................................................................uacuuugagucgguUcgaauca................  4     1  G01
......................................................................uacuuugagucUguacgaauca................  1     1  G01
......................................................................uacuuugagucgguCcgaauca................  6     1  G01
......................................................................uacuuugaCucgguacgaauca................  3     1  G01
......................................................................uacuuCgagucgguacgaauca................  1     1  G01
......................................................................uacuuugagucgguacUaauca................  1     1  G01
......................................................................uUcuuugagucgguacgaauca................  9     1  G01
```

```
                                 Star                              Mature
ggaucaaaauaacuuaacgugguucaggggcgguuuguaccgacucagagcuagguucguugaugagaacuacuuugagucgguacgaaucaccucagccaugaucca
......................................................................uacuuugagucgCuacgaauca................  5      1  G01
......................................................................uacuuGgagucgguacgaauca................  1      1  G01
......................................................................uacCuugagucgguacgaauca................  8      1  G01
......................................................................uacuuugagGcgguacgaauca................  1      1  G01
......................................................................uacuuuCagucgguacgaauca................  1      1  G01
......................................................................uacuuugaUucgguacgaauca................  2      1  G01
......................................................................uacuuAgagucgguacgaauca................  1      1  G01
......................................................................uacuuugagucgguGcgaauca................  46     1  G01
......................................................................uacuuuAagucgguacgaauca................  3      1  G01
......................................................................uacuuugagucgguacgaaucU................  330    1  G01
......................................................................uacuuugUgucgguacgaauca................  31     1  G01
......................................................................uacuuugagucgguacCaauca................  1      1  G01
......................................................................uacuuugagucgguacgaaCca................  13     1  G01
......................................................................uacuuugagucgguaUgaauca................  7      1  G01
......................................................................Cacuuugagucgguacgaauca................  1      1  G01
......................................................................uacuuugagucgUuacgaauca................  2      1  G01
......................................................................uacuuugagucggGacgaauca................  2      1  G01
......................................................................uacuuugagucggCacgaauca................  1      1  G01
......................................................................uacuuugagucgAuacgaauca................  3      1  G01
......................................................................uacuuugagucgguacgaaucG................  11     1  G01
......................................................................uacuuugagucgguacgaaucC................  5      1  G01
......................................................................uaUuuugagucgguacgaauca................  3      1  G01
......................................................................uacuuugaguGgguacgaauca................  4      1  G01
......................................................................uacuuugagucgguacgaauGa................  9      1  G01
......................................................................uacAuugagucgguacgaauca................  1      1  G01
......................................................................uacuuugagucgguacgUauca................  4      1  G01
......................................................................uacuuugagucgguacgGauca................  2      1  G01
......................................................................uacuuugagCcgguacgaauca................  2      1  G01
......................................................................uacuuugagAcgguacgaauca................  4      1  G01
......................................................................uCcuuugagucgguacgaauca................  3      1  G01
......................................................................uacuuugagucgguacgaaAca................  4      1  G01
......................................................................Gacuuugagucgguacgaauca................  4      1  G01
......................................................................uacuuugagucgguacgaauca................  26670  0  G01
......................................................................uacuuugCgucgguacgaauca................  9      1  G01
......................................................................uacuuugagucgguacgaauUa................  8      1  G01
......................................................................uacuCugagucgguacgaauca................  1      1  G01
......................................................................uacuAugagucgguacgaaucac...............  1      1  G01
......................................................................uacuuugagucgguacgaaucac...............  8061   0  G01
......................................................................uacuuugagucgCuacgaaucac...............  1      1  G01
......................................................................uacuuugaUucgguacgaaucac...............  3      1  G01
......................................................................uacuuugagucgguacgaUucac...............  1      1  G01
......................................................................uacuuugagucgguacgaaucaA...............  770    1  G01
......................................................................uacuuugagucgguacgaauUac...............  4      1  G01
......................................................................uacuuugagucgguacgCaucac...............  1      1  G01
......................................................................uacuuugagucgguacAaaucac...............  1      1  G01
......................................................................uacuuugagucgguacgaaucCc...............  2      1  G01
......................................................................uacuuugagucgguacgaaucUc...............  2      1  G01
......................................................................uUcuuugagucgguacgaaucac...............  2      1  G01
......................................................................uacuuugagucgguaUgaaucac...............  7      1  G01
......................................................................Gacuuugagucgguacgaaucac...............  1      1  G01
......................................................................uacuuugGgucgguacgaaucac...............  4      1  G01
......................................................................uacuuugagucgguacgaauGac...............  2      1  G01
......................................................................uacuuugUgucgguacgaaucac...............  9      1  G01
......................................................................uacuuugagucgguCcgaaucac...............  2      1  G01
......................................................................uacuuugCgucgguacgaaucac...............  3      1  G01
......................................................................uacuuugagucgguacgGaucac...............  1      1  G01
......................................................................uacCuugagucgguacgaaucac...............  2      1  G01
......................................................................uacuuGgagucgguacgaaucac...............  1      1  G01
......................................................................uacuuugagucgguacgaGucac...............  3      1  G01
......................................................................uacuuugagucgguacgaaucaG...............  4      1  G01
......................................................................uacuuugagucggCacgaaucac...............  1      1  G01
......................................................................uaUuuugagucgguacgaaucac...............  2      1  G01
......................................................................uacuuugagGcgguacgaaucac...............  1      1  G01
......................................................................uacuuugagucgguGcgaaucac...............  24     1  G01
......................................................................uacuuugaguGgguacgaaucac...............  2      1  G01
......................................................................Aacuuugagucgguacgaaucac...............  1      1  G01
......................................................................uacuuugagCcgguacgaaucac...............  1      1  G01
......................................................................uacuuugagucgUuacgaaucac...............  1      1  G01
......................................................................uGcuuugagucgguacgaaucac...............  2      1  G01
......................................................................uacuuugagucgguacgaaucacA..............  153    1  G01
```

Star **Mature**

```
ggaucaaaauaacuuaacgugguucaggggcgguuuguaccgacucagagcuagguucguugaugagaacuacuuugagucgguacgaaucaccucagccaugaucca
......................................................................uacuuugagucgguacgaaucacc..............	14	0	G01
......................................................................uacuuugagucgguacgaaucaccA.............	3	1	G01
......................................................................uacuuugagucgguacgaaucaccu.............	3	0	G01
......................................................................uacuuugagucgguacgaaucacAu.............	3	1	G01
.......................................................................acuuugagucgguacgaau..................	10	0	G01
.......................................................................Ucuuugagucgguacgaau..................	1	1	G01
.......................................................................acuuuUagucgguacgaau..................	1	1	G01
.......................................................................acuuugagucgguacgaauc.................	4	0	G01
.......................................................................acuuuUagucgguacgaauca................	1	1	G01
.......................................................................acuuugagucgguacgaauca................	50	0	G01
.......................................................................acuuugagucgguacgaaucac...............	59	0	G01
.......................................................................acuuugagucgguacgaaucaA...............	2	1	G01
.......................................................................acuuugagucgguaAgaaucac...............	1	1	G01
.......................................................................acuuugagucgguacgaaucacA..............	2	1	G01
........................................................................cuuugagucgguacgaau..................	1	0	G01
........................................................................cuuugagucgguacgaauca................	22	0	G01
........................................................................cuuugagucgguacgaaucac...............	4	0	G01
........................................................................cuuugagucgguacgaaucacA..............	1	1	G01
........................................................................cuuugagucgguacgaaucaccu.............	1	0	G01
........................................................................cAuugagucgguacgaaucaccu.............	1	1	G01
.........................................................................uuugagucgguacgaauc.................	3	0	G01
.........................................................................uuugagucgguacgaauca................	24	0	G01
.........................................................................uuugagucgguacgaaucaA...............	1	1	G01
.........................................................................uuugagucgguacgaaucac...............	16	0	G01
.........................................................................uuugagucgguacgaaucacc..............	1	0	G01
.........................................................................uuugagucgguacgaaucaccu.............	2	0	G01
..........................................................................uugagucgguacgaauca................	35	0	G01
..........................................................................uCgagucgguacgaauca................	1	1	G01
..........................................................................Gugagucgguacgaauca................	1	1	G01
..........................................................................uugagucgguacgaaucU................	1	1	G01
..........................................................................uugagucgguacgaaucac...............	20	0	G01
...........................................................................ugagucgguacgaaucac...............	2	0	G01

...............................gguuuguaccgacucagagc.........................................................	2	0	HJ1
...............................gguuuguaccgacucagagcu........................................................	3	0	HJ1
...............................gguuuguaccgacAcagagcu........................................................	1	1	HJ1
...............................gguuuguaccgacucagagcuaggu....................................................	1	0	HJ1
.................................uuuguaccgacucagagcA........................................................	1	1	HJ1
.................................uuuguaccgacucagagcuag......................................................	2	0	HJ1
.................................uuuguaccgacucagagcuagg.....................................................	4	0	HJ1
.................................uuuguaccgacCcagagcuagg.....................................................	1	1	HJ1
.................................uuuguaccgacucagagcuaggA....................................................	3	1	HJ1
.................................Cuuguaccgacucagagcuaggu....................................................	1	1	HJ1
.................................uuCguaccgacucagagcuaggu....................................................	1	1	HJ1
.................................uuuguaccgacucagagcuaggG....................................................	1	1	HJ1
.................................uuuguaccgacucagagcuaggu....................................................	38	0	HJ1
.................................uuuguaccgacucagagcuaggC....................................................	6	1	HJ1
.................................uuuguaccgacucagUgcuaggu....................................................	1	1	HJ1
.................................uuuguaccgacucagagcuagguu...................................................	2	0	HJ1
..................................uuguaccgacucagagcuaggu....................................................	1	0	HJ1
..................................uuguaccgacucagagcuagguu...................................................	1	0	HJ1
...................................uguaccgacucagagcuaggC....................................................	1	1	HJ1
...................................uguaccgacucagagcuaggu....................................................	1	0	HJ1
...................................uguaccgacucagagcuagguuc..................................................	1	0	HJ1
.....................................................................cuacuuugagucgguacg.....................	2	0	HJ1
.....................................................................cuacuuugagucgguacga....................	3	0	HJ1
.....................................................................cuacuuugagucgguacgaa...................	3	0	HJ1
.....................................................................cuacuuugagucgguacgaau..................	1	0	HJ1
.....................................................................cuacuuugagucgguacgaaC..................	2	1	HJ1
.....................................................................cuacuuugagucgguacgaaA..................	1	1	HJ1
.....................................................................cuacuuugagucgguacgGauc.................	1	1	HJ1
.....................................................................cuacuuugagucgguacgaauU.................	1	1	HJ1
.....................................................................cuacuuugagucgguacgaauc.................	12	0	HJ1
.....................................................................cuacuuugagucgguacgaauA.................	3	1	HJ1
.....................................................................cuacCuugagucgguacgaauc.................	1	1	HJ1
.....................................................................cuacuuugagucgguacgaaAca................	1	1	HJ1
.....................................................................cuacuuugagucgguacgaauca................	18	0	HJ1
.....................................................................cuacuuugGgucgguacgaauca................	2	1	HJ1
.....................................................................cuacuuugagucgguacgaaucG................	1	1	HJ1
.....................................................................cuacuuugagucgguacgaaucac...............	21	0	HJ1
```

Star **Mature**

```
ggaucaaaauaacuuaacgugguucaggggcgguuuguaccgacucagagcuagguucguugaugagaacuacuuugagucgguacgaaucaccucagccaugaucca
.....................................................................cuacuCugagucgguacgaaucac...............	1	1	HJ1
......................................................................uacuuugagucgguacgC....................	2	1	HJ1
......................................................................uacuuugagucgguacga....................	62	0	HJ1
......................................................................uacuuugagucgguacgU....................	4	1	HJ1
......................................................................uacuuugagucgguGcga....................	2	1	HJ1
......................................................................uacuuugagucgguacgaa...................	7	0	HJ1
......................................................................uacuCugagucgguacgaau..................	2	1	HJ1
......................................................................uacuuugagucggCacgaau..................	1	1	HJ1
......................................................................uacuuugagucgguacgaUu..................	1	1	HJ1
......................................................................uacuuugagucAguacgaau..................	1	1	HJ1
......................................................................Cacuuugagucgguacgaau..................	2	1	HJ1
......................................................................uUcuuugagucgguacgaau..................	1	1	HJ1
......................................................................uacCuugagucgguacgaau..................	1	1	HJ1
......................................................................uacuuugaAucgguacgaau..................	1	1	HJ1
......................................................................uacuuugagucgguacgaaG..................	1	1	HJ1
......................................................................uacuuugagucgguacgGau..................	1	1	HJ1
......................................................................Aacuuugagucgguacgaau..................	1	1	HJ1
......................................................................uaUuuugagucgguacgaau..................	1	1	HJ1
......................................................................uacuuugagucgguacgaaC..................	29	1	HJ1
......................................................................uacuuugagucgguGcgaau..................	2	1	HJ1
......................................................................uacuuugagucggAacgaau..................	2	1	HJ1
......................................................................uacuuugagucgguacgaGu..................	5	1	HJ1
......................................................................uacuuugagucgguacgaau..................	238	0	HJ1
......................................................................uacuuugagucgguacgaaA..................	4	1	HJ1
......................................................................uacCuugagucgguacgaauc.................	3	1	HJ1
......................................................................uacuuugagucggAacgaauc.................	2	1	HJ1
......................................................................uacuuugagucggCacgaauc.................	3	1	HJ1
......................................................................Cacuuugagucgguacgaauc.................	1	1	HJ1
......................................................................uacuuCgagucgguacgaauc.................	1	1	HJ1
......................................................................uacuuugagucgguacgaaGc.................	1	1	HJ1
......................................................................uacuuugagCcgguacgaauc.................	3	1	HJ1
......................................................................uacuuAgagucgguacgaauc.................	1	1	HJ1
......................................................................Aacuuugagucgguacgaauc.................	3	1	HJ1
......................................................................uacuuugaAucgguacgaauc.................	1	1	HJ1
......................................................................uacuuugUgucgguacgaauc.................	1	1	HJ1
......................................................................uacuuugagucgguacgaaCc.................	6	1	HJ1
......................................................................uacuuugagucgguacgaauc.................	671	0	HJ1
......................................................................uacuuugagucAguacgaauc.................	1	1	HJ1
......................................................................uacuuugGgucgguacgaauc.................	2	1	HJ1
......................................................................uaAuuugagucgguacgaauc.................	1	1	HJ1
......................................................................uacuAugagucgguacgaauc.................	2	1	HJ1
......................................................................uacuuugagucgguacgaauG.................	1	1	HJ1
......................................................................uacuuugagucgguaUgaauc.................	2	1	HJ1
......................................................................uacuuugagucgguacgaGuc.................	1	1	HJ1
......................................................................uacuuugagucgguGcgaauc.................	1	1	HJ1
......................................................................uacuuugagucgguacUaauc.................	3	1	HJ1
......................................................................uacuCugagucgguacgaauc.................	6	1	HJ1
......................................................................uacuuugagucgguacgaauA.................	16	1	HJ1
......................................................................uacuuugagucgguaGgaauc.................	1	1	HJ1
......................................................................uacuuugagucgguacgaauU.................	45	1	HJ1
......................................................................uacuuugagucgguacgGauc.................	4	1	HJ1
......................................................................uacuuugGgucgguacgaauca................	5	1	HJ1
......................................................................uacuuCgagucgguacgaauca................	2	1	HJ1
......................................................................uacuuAgagucgguacgaauca................	1	1	HJ1
......................................................................uUcuuugagucgguacgaauca................	2	1	HJ1
......................................................................uacCuugagucgguacgaauca................	3	1	HJ1
......................................................................uacuuugagucgguacgaaAca................	1	1	HJ1
......................................................................uacuuugagucgguacgaaucC................	5	1	HJ1
......................................................................uacuuugagucgguGcgaauca................	3	1	HJ1
......................................................................uacuGugagucgguacgaauca................	2	1	HJ1
......................................................................uacuuugagucgguaUgaauca................	3	1	HJ1
......................................................................uacuuugaAucgguacgaauca................	2	1	HJ1
......................................................................uacAuugagucgguacgaauca................	1	1	HJ1
......................................................................uacuuugagucgguacgaaucU................	37	1	HJ1
......................................................................Aacuuugagucgguacgaauca................	1	1	HJ1
......................................................................uacuuugagucgguacgaaucG................	23	1	HJ1
......................................................................uacuuugagucAguacgaauca................	2	1	HJ1
......................................................................uacuuugagucgguacgaGuca................	1	1	HJ1
......................................................................uacuuugagCcgguacgaauca................	1	1	HJ1
......................................................................uacuuugagucgguacgaaGca................	3	1	HJ1
```

```
                                 Star                            Mature
ggaucaaaauaacuuaacgugguucaggggcgguuuguaccgacucagagcuagguucguugaugagaacuacuuugagucgguacgaaucaccucagccaugaucca
......................................................................uacuuugagucgguaAgaauca................  2    1  HJ1
......................................................................uacuuugagucgguacgaauca................  861  0  HJ1
......................................................................uacuuugagucgguacgaauGa................  1    1  HJ1
......................................................................uacuuugagucgguacgaauAa................  1    1  HJ1
......................................................................Cacuuugagucgguacgaauca................  4    1  HJ1
......................................................................uacuCugagucgguacgaauca................  2    1  HJ1
......................................................................uacuuugagucgguacAaauca................  5    1  HJ1
......................................................................uacuuugagucggCacgaauca................  2    1  HJ1
......................................................................uacuuugagucggAacgaauca................  1    1  HJ1
......................................................................uacuuugagucgguacgaaCca................  5    1  HJ1
......................................................................uacuAugagucgguacgaauca................  5    1  HJ1
......................................................................uaAuuugagucgguacgaauca................  1    1  HJ1
......................................................................uacuuugagucgguaUgaaucac...............  2    1  HJ1
......................................................................uacuuAgagucgguacgaaucac...............  1    1  HJ1
......................................................................uacuuugagucgguacgaaucGc...............  1    1  HJ1
......................................................................uacuuugGgucgguacgaaucac...............  1    1  HJ1
......................................................................Cacuuugagucgguacgaaucac...............  1    1  HJ1
......................................................................uacuuugagucgguacgGaucac...............  1    1  HJ1
......................................................................uacuAugagucgguacgaaucac...............  2    1  HJ1
......................................................................uacuuugagucgguacgaaucaA...............  39   1  HJ1
......................................................................uacuuugagucgguacgaaucaG...............  3    1  HJ1
......................................................................uacCuugagucgguacgaaucac...............  1    1  HJ1
......................................................................uacuuugagucgguacgaaucac...............  159  0  HJ1
......................................................................uacuuugagucgguaAgaaucac...............  1    1  HJ1
......................................................................uacuuugagCcgguacgaaucac...............  1    1  HJ1
......................................................................uacuCugagucgguacgaaucac...............  1    1  HJ1
......................................................................uacuuugagucgguacgaaucacG..............  5    1  HJ1
......................................................................uGcuuugagucgguacgaaucacc..............  1    1  HJ1
......................................................................uacuuugagucgguacgaaucacA..............  8    1  HJ1
......................................................................uacuuugagucgguacgaaucacc..............  16   0  HJ1
......................................................................uacuuugagucgguacgaaucacAu.............  1    1  HJ1
......................................................................uacuuugagucgguacgaaucaccC.............  2    1  HJ1
......................................................................uacuuugagucgguacgaaucaccu.............  1    0  HJ1
.......................................................................acuuugagucgAuacgaauca................  1    1  HJ1
.......................................................................acuuugagucgguacgaaCca................  1    1  HJ1
.......................................................................acuuugagucgguacgaaucaA...............  1    1  HJ1
.........................................................................uuugagucgguacgaaucacc..............  2    0  HJ1
..........................................................................uugagucgguacgaauca................  3    0  HJ1
..........................................................................uugagucgguacgaaucG................  1    1  HJ1
..........................................................................uugagucgguacgaaucac...............  1    0  HJ1

.................................uuuguaccgacucagagcuagg.....................................................  1    0  LA1
.................................uuuguaccgacucagagcuaggu....................................................  2    0  LA1
...................................uguaccgacucagagcuagguuc..................................................  1    0  LA1
.....................................................................cuacuuugagucgguacgaa...................  2    0  LA1
.....................................................................cuacuuugagucgguacgaau..................  1    0  LA1
.....................................................................cuacuuugagucgguacgaauc.................  23   0  LA1
.....................................................................cuacuuugagCcgguacgaauca................  1    1  LA1
.....................................................................cuacuuugagucgguacgaauca................  100  0  LA1
.....................................................................cuacuuCgagucgguacgaauca................  1    1  LA1
.....................................................................cGacuuugagucgguacgaauca................  1    1  LA1
.....................................................................cuacuuugagucgguacgaaucG................  1    1  LA1
.....................................................................cuacuuugagucgguacgaaucac...............  34   0  LA1
.....................................................................cuacuuugagucgguacgaaucacc..............  1    0  LA1
......................................................................uacuuugagucgguacga....................  14   0  LA1
......................................................................uacuuugagucgguacgaa...................  13   0  LA1
......................................................................uacuCugagucgguacgaau..................  1    1  LA1
......................................................................uacuuugagCcgguacgaau..................  2    1  LA1
......................................................................uacuuugagucggCacgaau..................  1    1  LA1
......................................................................uacuuugagucgguacgaaC..................  15   1  LA1
......................................................................uacuuugagucgguacgaUu..................  1    1  LA1
......................................................................uacuuCgagucgguacgaau..................  1    1  LA1
......................................................................uGcuuugagucgguacgaau..................  1    1  LA1
......................................................................uacCuugagucgguacgaau..................  1    1  LA1
......................................................................uacuuugagucgguacgaaG..................  2    1  LA1
......................................................................uacuuugagucgguacgGau..................  1    1  LA1
......................................................................uacuuugagucgguacgaau..................  183  0  LA1
......................................................................uUcuuugagucgguacgaau..................  1    1  LA1
......................................................................uacuuugagucgguacgaaA..................  6    1  LA1
......................................................................Cacuuugagucgguacgaauc.................  2    1  LA1
```

Star Mature

```
ggaucaaaauaacuuaacgugguucaggggcgguuuguaccgacucagagcuagguucguugaugagaacuacuuugagucgguacgaaucaccucagccaugaucca
......................................................................uGcuuugagucgguacgaauc.................	3	1	LA1
......................................................................uacuGugagucgguacgaauc.................	1	1	LA1
......................................................................uacuuugagucgguacUaauc.................	1	1	LA1
......................................................................Aacuuugagucgguacgaauc.................	1	1	LA1
......................................................................uacuuugGgucgguacgaauc.................	2	1	LA1
......................................................................uacuuugagucgguacgaauU.................	19	1	LA1
......................................................................uacuuugaguGgguacgaauc.................	1	1	LA1
......................................................................uacuuugagucgguacgaGuc.................	1	1	LA1
......................................................................uacuuugagucgguacgGauc.................	3	1	LA1
......................................................................uacuuugagucgguacgaaAc.................	4	1	LA1
......................................................................uacuuugagucgguacgaaCc.................	2	1	LA1
......................................................................uacuuugagucgguacgaauc.................	1102	0	LA1
......................................................................uacuuugagucgguaUgaauc.................	1	1	LA1
......................................................................uacuuugagucgguacgaauA.................	6	1	LA1
......................................................................uacCuugagucgguacgaauc.................	1	1	LA1
......................................................................uacuuugagucAguacgaauc.................	1	1	LA1
......................................................................uacuuugagucgguacgaaGc.................	1	1	LA1
......................................................................uacuuugagucgguGcgaauc.................	2	1	LA1
......................................................................uacuuugagucggCacgaauc.................	6	1	LA1
......................................................................uacuuugaguAgguacgaauc.................	2	1	LA1
......................................................................uacuuugagucgguGcgaauca................	3	1	LA1
......................................................................uacuuugagucgguaGgaauca................	2	1	LA1
......................................................................uacuuugagucgguacAaauca................	2	1	LA1
......................................................................uacuuCgagucgguacgaauca................	12	1	LA1
......................................................................uacuuugagucgguacgaaucC................	3	1	LA1
......................................................................uacuuugCgucgguacgaauca................	1	1	LA1
......................................................................uacuuugagucUguacgaauca................	2	1	LA1
......................................................................uacuuugagCcgguacgaauca................	7	1	LA1
......................................................................uacuuugagucCguacgaauca................	1	1	LA1
......................................................................uacuuugagucgAuacgaauca................	1	1	LA1
......................................................................uacuuugagucgguaUgaauca................	2	1	LA1
......................................................................uCcuuugagucgguacgaauca................	1	1	LA1
......................................................................uacuuugagGcgguacgaauca................	1	1	LA1
......................................................................uacuCugagucgguacgaauca................	8	1	LA1
......................................................................uacuuuAagucgguacgaauca................	3	1	LA1
......................................................................uacuuugagucgguacgaauca................	3240	0	LA1
......................................................................uacuuAgagucgguacgaauca................	1	1	LA1
......................................................................uacuuugagucgguacgaaucU................	15	1	LA1
......................................................................uacuuugaAucgguacgaauca................	1	1	LA1
......................................................................uacCuugagucgguacgaauca................	10	1	LA1
......................................................................uacuuugGgucgguacgaauca................	7	1	LA1
......................................................................uacuuugagucgguacgaaCca................	12	1	LA1
......................................................................uacuuugagucggAacgaauca................	3	1	LA1
......................................................................uacuuugagucgguacUaauca................	1	1	LA1
......................................................................Cacuuugagucgguacgaauca................	11	1	LA1
......................................................................uGcuuugagucgguacgaauca................	13	1	LA1
......................................................................uacuuugagucAguacgaauca................	4	1	LA1
......................................................................uacuuugagucgguacgaaucG................	43	1	LA1
......................................................................uacuuugUgucgguacgaauca................	1	1	LA1
......................................................................uacuuugagucggCacgaauca................	3	1	LA1
......................................................................uacuuugagucgguacgaaGca................	2	1	LA1
......................................................................Aacuuugagucgguacgaauca................	1	1	LA1
......................................................................uacuuugagucgguCcgaauca................	2	1	LA1
......................................................................uaUuuugagucgguacgaauca................	1	1	LA1
......................................................................uacuuugagucgguacgaauUa................	1	1	LA1
......................................................................uacuuugagucgguacgGauca................	10	1	LA1
......................................................................uacuuugagucgguacgaGuca................	7	1	LA1
......................................................................Cacuuugagucgguacgaaucac...............	3	1	LA1
......................................................................uacuuugGgucgguacgaaucac...............	1	1	LA1
......................................................................uacuuugagucgguacgaaucaG...............	2	1	LA1
......................................................................uacuuugagucgguGcgaaucac...............	3	1	LA1
......................................................................uacuuugagucgguacgaaCcac...............	2	1	LA1
......................................................................uacuuugagucgguacgaaucaA...............	60	1	LA1
......................................................................uacCuugagucgguacgaaucac...............	2	1	LA1
......................................................................uacuuugagucgguacgaaucac...............	255	0	LA1
......................................................................uaUuuugagucgguacgaaucac...............	1	1	LA1
......................................................................uacuCugagucgguacgaaucac...............	1	1	LA1
......................................................................uacuuugagucgguacgaaucGc...............	1	1	LA1
......................................................................uacuuAgagucgguacgaaucac...............	1	1	LA1
......................................................................uacuuugagucgguacgaaucacc..............	10	0	LA1
```

```
                                Star                                Mature
ggaucaaaauaacuuaacgugguucaggggcgguuuguaccgacucagagcuagguucguugaugagaacuacuuugagucgguacgaaucaccucagccaugaucca
......................................................................uacuuugagucgguacgaaucacA..............  22    1    LA1
......................................................................uacuuugagucgguacgaaucaccu.............  1     0    LA1
.......................................................................acuuugagucggCacgaauc.................  2     1    LA1
.......................................................................acuuugagucgguacgaauc.................  2     0    LA1
.......................................................................acuuugagucgguacgaauca................  2     0    LA1
.......................................................................acuuugagucgguacgaaucac...............  2     0    LA1
........................................................................cuuugagucgguacgaauca................  2     0    LA1
.........................................................................uuugagucgguacgaaucacc..............  1     0    LA1
.........................................................................uuugagucgguacgaaucacA..............  1     1    LA1
..........................................................................uugagucgguacgaaucac...............  1     0    LA1

.................................uuuguaccgacucagagcuaggu....................................................  1     0    CA1
.....................................................................cuacuuugagucgguacgaau..................  1     0    CA1
.....................................................................cuacuuugagucgguacgaauc.................  10    0    CA1
.....................................................................cuacuuugagucgguacgaauU.................  1     1    CA1
.....................................................................cuacuuCgagucgguacgaauca................  1     1    CA1
.....................................................................cuacuuugagucgguacgaaucU................  1     1    CA1
.....................................................................cuacuuugagucgguacgaauca................  115   0    CA1
.....................................................................cuacuuugagucgguacgaaucUc...............  1     1    CA1
.....................................................................cuacuuugagucgguacgaaucCc...............  1     1    CA1
.....................................................................cuacuuugagucgguacgaaucac...............  44    0    CA1
.....................................................................cuacuuugagucgguacgaaucaA...............  2     1    CA1
.....................................................................cuacuuugagucgguacgaaucacA..............  2     1    CA1
.....................................................................cuacuuugagucgguacgaaucacc..............  1     0    CA1
......................................................................uacuuugagucgguacga....................  9     0    CA1
......................................................................uacuuugagucgguacgaa...................  13    0    CA1
......................................................................uacuuugagucgguGcgaau..................  1     1    CA1
......................................................................uacuuugGgucgguacgaau..................  1     1    CA1
......................................................................uacuuAgagucgguacgaau..................  1     1    CA1
......................................................................uacuuugagucgguacgaaA..................  5     1    CA1
......................................................................uUcuuugagucgguacgaau..................  1     1    CA1
......................................................................uacuuugagucgguacgaaG..................  3     1    CA1
......................................................................uacuuugagucgguacgaaC..................  15    1    CA1
......................................................................uacuuugagucgguacgaau..................  187   0    CA1
......................................................................uacuuugagucAguacgaau..................  1     1    CA1
......................................................................uacuuugagucgguacgaGu..................  1     1    CA1
......................................................................uacuuugagucgguacgGau..................  1     1    CA1
......................................................................uacGuugagucgguacgaau..................  1     1    CA1
......................................................................uacuuugaguAgguacgaauc.................  1     1    CA1
......................................................................uacuuugagucAguacgaauc.................  1     1    CA1
......................................................................uacuuugagucgguacgGauc.................  3     1    CA1
......................................................................uacuAugagucgguacgaauc.................  2     1    CA1
......................................................................uacuuugagucgguacgaauc.................  1056  0    CA1
......................................................................uacuuugagucgguacgaGuc.................  6     1    CA1
......................................................................uGcuuugagucgguacgaauc.................  3     1    CA1
......................................................................uacuuugagucgguGcgaauc.................  1     1    CA1
......................................................................uacuuugaAucgguacgaauc.................  1     1    CA1
......................................................................uacuuugagucgguacgUauc.................  1     1    CA1
......................................................................uacuuugGgucgguacgaauc.................  2     1    CA1
......................................................................uacuCugagucgguacgaauc.................  2     1    CA1
......................................................................uacuuugUgucgguacgaauc.................  1     1    CA1
......................................................................Cacuuugagucgguacgaauc.................  3     1    CA1
......................................................................uacuuugagucgguacgaauA.................  10    1    CA1
......................................................................uacuuCgagucgguacgaauc.................  2     1    CA1
......................................................................uacuuugagucggAacgaauc.................  2     1    CA1
......................................................................uacuuugagucgguaUgaauc.................  3     1    CA1
......................................................................uacCuugagucgguacgaauc.................  6     1    CA1
......................................................................uacuuugagucgguacgaauU.................  33    1    CA1
......................................................................uacuuugagucgguUcgaauc.................  1     1    CA1
......................................................................uacuuugagucgguacgaaCc.................  2     1    CA1
......................................................................Aacuuugagucgguacgaauc.................  1     1    CA1
......................................................................uacuAugagucgguacgaauca................  1     1    CA1
......................................................................uacuuugagucgguacgaaCca................  10    1    CA1
......................................................................uacuuugagucgguacgaaucU................  21    1    CA1
......................................................................uacGuugagucgguacgaauca................  1     1    CA1
......................................................................uacuuugagucgguacgUauca................  2     1    CA1
......................................................................uacuuuCagucgguacgaauca................  1     1    CA1
......................................................................uacuuAgagucgguacgaauca................  4     1    CA1
......................................................................uacuuugagucgguacgaauUa................  2     1    CA1
......................................................................Gacuuugagucgguacgaauca................  1     1    CA1
```

```
                                                 Star                                  Mature
ggaucaaaauaacuuaacgugguucaggggcgguuuguaccgacucagagcuagguucguugaugagaacuacuuugagucgguacgaaucaccucagccaugaucca
......................................................................uacuuugagucgguacgaauca................ 3338 0 CA1
......................................................................uacuuugGgucgguacgaauca................ 15 1 CA1
......................................................................Cacuuugagucgguacgaauca................ 12 1 CA1
......................................................................uacCuugagucgguacgaauca................ 19 1 CA1
......................................................................uacuuugagCcgguacgaauca................ 9 1 CA1
......................................................................uacuCugagucgguacgaauca................ 9 1 CA1
......................................................................uacuuugagucgguacgaaAca................ 2 1 CA1
......................................................................uacuuugagucggCacgaauca................ 6 1 CA1
......................................................................uacuuugagucgguGcgaauca................ 9 1 CA1
......................................................................uacuuugagucgguaUgaauca................ 5 1 CA1
......................................................................uacuuugagucgguacgaCuca................ 2 1 CA1
......................................................................uacuuugagucgguacgGauca................ 6 1 CA1
......................................................................uacuuugagucggAacgaauca................ 1 1 CA1
......................................................................uGcuuugagucgguacgaauca................ 11 1 CA1
......................................................................uacuuugagucgguacgaaucC................ 2 1 CA1
......................................................................uacuuugagucgguacgaaucG................ 43 1 CA1
......................................................................uacuuugagucgguacgaGuca................ 5 1 CA1
......................................................................uacuuugaguAgguacgaauca................ 2 1 CA1
......................................................................uacuuCgagucgguacgaauca................ 12 1 CA1
......................................................................uacuuugagucgguacAaauca................ 6 1 CA1
......................................................................uacuuugagucAguacgaauca................ 3 1 CA1
......................................................................uUcuuugagucgguacgaauca................ 1 1 CA1
......................................................................uacuuugagucgguUcgaauca................ 2 1 CA1
......................................................................uaUuuugagucgguacgaauca................ 2 1 CA1
......................................................................uacuuugagucgAuacgaauca................ 2 1 CA1
......................................................................Aacuuugagucgguacgaauca................ 1 1 CA1
......................................................................Cacuuugagucgguacgaaucac............... 2 1 CA1
......................................................................uacuuugagCcgguacgaaucac............... 2 1 CA1
......................................................................uacuuugagucgguacgaaucaA............... 79 1 CA1
......................................................................uacuuugGgucgguacgaaucac............... 3 1 CA1
......................................................................uGcuuugagucgguacgaaucac............... 2 1 CA1
......................................................................uacuuugagucgguaUgaaucac............... 1 1 CA1
......................................................................uacuCugagucgguacgaaucac............... 1 1 CA1
......................................................................uacuuugagucgguacgaaucac............... 286 0 CA1
......................................................................uacuuugagucgguacgGaucac............... 2 1 CA1
......................................................................uacuuugagucgguacgaaGcac............... 2 1 CA1
......................................................................uacuuugagucggCacgaaucac............... 1 1 CA1
......................................................................uacuuugagucgguacgaGucac............... 1 1 CA1
......................................................................uacuuugagucgguacgaaucGc............... 1 1 CA1
......................................................................uacuuugagucgguacgaaucacc.............. 7 0 CA1
......................................................................uacuuugagucgguacgaaucacG.............. 3 1 CA1
......................................................................uacuuugagucgguacgaaucacA.............. 11 1 CA1
.......................................................................acuuugagucgguacgaauc................. 2 0 CA1
.......................................................................acuuugagucgguacgaauca................ 3 0 CA1
.......................................................................acuuugagucgguacgaaucac............... 3 0 CA1
........................................................................cuuugagucgguacgaauca................ 1 0 CA1
........................................................................cuuugagucgguacgaaucacc.............. 1 0 CA1
........................................................................cuuugagucgguacgaaucacA.............. 1 1 CA1
.........................................................................uuugagucgguacgaaucacc.............. 2 0 CA1

...............................Aguuuguaccgacucagagc......................................................... 2 1 3rd
...............................gguuuguaccgacucagagcu........................................................ 16 0 3rd
...............................gguuuguaccgacucagagcua....................................................... 10 0 3rd
...............................gguuuguaccgacucagagcuU....................................................... 2 1 3rd
...............................gguuuguaccgacucagagcuag...................................................... 1 0 3rd
...............................gguuuguaccgacucagagcuagg..................................................... 4 0 3rd
...............................gguuuguaccgacucagagcuaggu.................................................... 34 0 3rd
...............................gguuuguaccgacucagagcuaggA.................................................... 2 1 3rd
...............................gguuuguaccgacucagagcuagguu................................................... 12 0 3rd
................................guuuguaccgacucagagcuag...................................................... 1 0 3rd
................................guuuguaccgacucagagcuagguu................................................... 1 0 3rd
.................................uuuguaccgacucagagcua....................................................... 3 0 3rd
.................................uuuguaccgacucagagcuag...................................................... 12 0 3rd
.................................uuuguaccgCcucagagcuag...................................................... 1 1 3rd
.................................uuuguaccgacucagagcuagg..................................................... 9 0 3rd
.................................uuuguaccgacucagagcuagA..................................................... 1 1 3rd
.................................uGuguaccgacucagagcuaggu.................................................... 1 1 3rd
.................................uuuguaccgacucagagcuaggu.................................................... 74 0 3rd
.................................uuGguaccgacucagagcuaggu.................................................... 1 1 3rd
.................................uuuguaccgacucagagcuaggG.................................................... 1 1 3rd
```

Star **Mature**

```
ggaucaaaauaacuuaacgugguucaggggcgguuuguaccgacucagagcuagguucguugaugagaacuacuuugagucgguacgaaucaccucagccaugaucca
.................................uuuguaccgacucagCgcuaggu....................................................	1	1	3rd
.................................uuuguaccgacucagagcuUggu....................................................	1	1	3rd
.................................uuuguaccgacucagagcuagguu...................................................	8	0	3rd
.................................uuuguaccgacucagagcuagguuU..................................................	1	1	3rd
..................................uuguaccgacucagagcuag......................................................	1	0	3rd
..................................uuguaccgacucagagcuaggA....................................................	1	1	3rd
..................................uuguaccgacucagagcuagguu...................................................	2	0	3rd
..................................uuguaccgacucagagcuagguuc..................................................	2	0	3rd
...................................uguaccgacucagagcuaggu....................................................	1	0	3rd
...................................uguaccgacucagagcuagguu...................................................	7	0	3rd
...................................uguaccgacucagagcuagguuU..................................................	1	1	3rd
...................................uguaccgacucagagcuagguuc..................................................	15	0	3rd
...................................uguaccgacucagagcuagguucg.................................................	3	0	3rd
...............................................................ugagaacuacuuugagucgg.........................	1	0	3rd
..................................................................gaGcuacuuugagucgguacgaau..................	1	1	3rd
..................................................................gGacuacuuugagucgguacgaauca................	1	1	3rd
..................................................................gGacuacuuugagucgguacgaaucac...............	1	1	3rd
...................................................................aacuacuuugagucgguacg.....................	1	0	3rd
...................................................................aacuacuuugagucgguacgaauca................	1	0	3rd
...................................................................aGcuacuuugagucgguacgaauca................	1	1	3rd
...................................................................Gacuacuuugagucgguacgaauca................	1	1	3rd
...................................................................Gacuacuuugagucgguacgaaucac...............	1	1	3rd
...................................................................aaAuacuuugagucgguacgaaucac...............	1	1	3rd
....................................................................acuacuuugagucgguacgaau..................	2	0	3rd
....................................................................aGuacuuugagucgguacgaauc.................	1	1	3rd
....................................................................acuacuuugagucgguacgaauca................	1	0	3rd
....................................................................Ucuacuuugagucgguacgaauca................	2	1	3rd
....................................................................Ucuacuuugagucgguacgaaucac...............	5	1	3rd
....................................................................acuacuuugagucgguacgaaucac...............	3	0	3rd
.....................................................................cuacuuugagucgguacg.....................	27	0	3rd
.....................................................................Uuacuuugagucgguacga....................	1	1	3rd
.....................................................................cuacuuugagucgguacAa....................	1	1	3rd
.....................................................................cuacuuugagucgguacga....................	2	0	3rd
.....................................................................cuacuuugagucgguacgaa...................	19	0	3rd
.....................................................................Uuacuuugagucgguacgaa...................	1	1	3rd
.....................................................................cuacuuugagucgguacgaau..................	222	0	3rd
.....................................................................Uuacuuugagucgguacgaau..................	20	1	3rd
.....................................................................cuacuuugagucgguacgaaA..................	4	1	3rd
.....................................................................cuGcuuugagucgguacgaau..................	1	1	3rd
.....................................................................cuacuuugUgucgguacgaau..................	1	1	3rd
.....................................................................Uuacuuugagucgguacgaauc.................	23	1	3rd
.....................................................................cuacuuugagucgguacgaauc.................	301	0	3rd
.....................................................................cuacuuugagucgguacgGauc.................	1	1	3rd
.....................................................................cuacuuugagucgguacgaUuc.................	1	1	3rd
.....................................................................cuUcuuugagucgguacgaauc.................	1	1	3rd
.....................................................................cuacuuugagucgguGcgaauc.................	1	1	3rd
.....................................................................cuacuuugagucgguacgaauU.................	1	1	3rd
.....................................................................cuacuuugCgucgguacgaauc.................	1	1	3rd
.....................................................................cuacuuugUgucgguacgaauca................	5	1	3rd
.....................................................................cuacuuugagCcgguacgaauca................	1	1	3rd
.....................................................................cuacuuugagucgguacAaauca................	2	1	3rd
.....................................................................cuacuGugagucgguacgaauca................	4	1	3rd
.....................................................................Auacuuugagucgguacgaauca................	2	1	3rd
.....................................................................cuacuuugagucgguacgCauca................	1	1	3rd
.....................................................................cuacuuugCgucgguacgaauca................	3	1	3rd
.....................................................................cuacuuugagucggGacgaauca................	1	1	3rd
.....................................................................cuacuuugagucgAuacgaauca................	2	1	3rd
.....................................................................cuacuuugagucgguacgaaucC................	2	1	3rd
.....................................................................cuacuuugagucgguacgaUuca................	10	1	3rd
.....................................................................cuacuuugagucgguacgaauca................	4905	0	3rd
.....................................................................Uuacuuugagucgguacgaauca................	125	1	3rd
.....................................................................cuacuAugagucgguacgaauca................	3	1	3rd
.....................................................................cuacuuugagucgguacgaaucU................	7	1	3rd
.....................................................................cGacuuugagucgguacgaauca................	1	1	3rd
.....................................................................cuacuuuUagucgguacgaauca................	1	1	3rd
.....................................................................cuacuuugagucgUuacgaauca................	1	1	3rd
.....................................................................cuacuuugagucgguGcgaauca................	2	1	3rd
.....................................................................cuUcuuugagucgguacgaauca................	3	1	3rd
.....................................................................cuacuuuCagucgguacgaauca................	1	1	3rd
.....................................................................cuacuuugagucgguaUgaauca................	3	1	3rd
```

```
                                Star                                  Mature
ggaucaaaauaacuuaacgugguucaggggcgguuuguaccgacucagagcuagguucguugaugagaacuacuuugagucgguacgaaucaccucagccaugaucca
.....................................................................Guacuuugagucgguacgaauca................	2	1	3rd
.....................................................................cuacuuugagucggCacgaauca................	2	1	3rd
.....................................................................cuacuuugagucgguacgaCuca................	1	1	3rd
.....................................................................cuacuuugGgucgguacgaauca................	4	1	3rd
.....................................................................cuacuuugagucgguacgGauca................	2	1	3rd
.....................................................................cuacuuugagucgguacgaauGa................	1	1	3rd
.....................................................................cuacuuugagucgguacgaauUa................	1	1	3rd
.....................................................................cAacuuugagucgguacgaauca................	2	1	3rd
.....................................................................cuacuuugagucgguacgaaucG................	1	1	3rd
.....................................................................cuacuuugagucggAacgaauca................	1	1	3rd
.....................................................................cuacuuugagGcgguacgaauca................	1	1	3rd
.....................................................................cuacuuugagucgguacgUauca................	1	1	3rd
.....................................................................cuacuuCgagucgguacgaauca................	2	1	3rd
.....................................................................cuacCuugagucgguacgaauca................	1	1	3rd
.....................................................................cuacuCugagucgguacgaauca................	6	1	3rd
.....................................................................cuacuuugagucgguacgaGuca................	4	1	3rd
.....................................................................cuacuuugagucgguacgaaucaA...............	61	1	3rd
.....................................................................cuacCuugagucgguacgaaucac...............	4	1	3rd
.....................................................................cuacuuugagucgguacCaaucac...............	2	1	3rd
.....................................................................cuacuuugagucggCacgaaucac...............	4	1	3rd
.....................................................................cAacuuugagucgguacgaaucac...............	3	1	3rd
.....................................................................cuGcuuugagucgguacgaaucac...............	1	1	3rd
.....................................................................cuacuuuCagucgguacgaaucac...............	1	1	3rd
.....................................................................cuacuuugagucgguaUgaaucac...............	3	1	3rd
.....................................................................cuacuuugagucgguacgaaucaG...............	2	1	3rd
.....................................................................cuacuuugagucUguacgaaucac...............	1	1	3rd
.....................................................................cuacuuugagucgguacAaaucac...............	1	1	3rd
.....................................................................cuacuuugagucgguacgGaucac...............	2	1	3rd
.....................................................................cuacuAugagucgguacgaaucac...............	4	1	3rd
.....................................................................cuacuuugUgucgguacgaaucac...............	5	1	3rd
.....................................................................cuacuuugagucgguacgaaGcac...............	1	1	3rd
.....................................................................cuaUuuugagucgguacgaaucac...............	1	1	3rd
.....................................................................cuacuuugagucgguacgaaucac...............	4660	0	3rd
.....................................................................cuacuuugaguGgguacgaaucac...............	1	1	3rd
.....................................................................cuacuuugagucgAuacgaaucac...............	3	1	3rd
.....................................................................cuacuuugGgucgguacgaaucac...............	1	1	3rd
.....................................................................cuacuuugagucgCuacgaaucac...............	1	1	3rd
.....................................................................cuacuuugagAcgguacgaaucac...............	1	1	3rd
.....................................................................cuacuuugCgucgguacgaaucac...............	1	1	3rd
.....................................................................cuacuuugagGcgguacgaaucac...............	1	1	3rd
.....................................................................cuacuuugagucgguacgaUucac...............	5	1	3rd
.....................................................................cuacuuugagucggAacgaaucac...............	1	1	3rd
.....................................................................cuacuGugagucgguacgaaucac...............	2	1	3rd
.....................................................................cuacuuugagCcgguacgaaucac...............	2	1	3rd
.....................................................................cuacuuugagucgUuacgaaucac...............	2	1	3rd
.....................................................................cuacuuugagucgguGcgaaucac...............	6	1	3rd
.....................................................................cuacuCugagucgguacgaaucac...............	4	1	3rd
.....................................................................cCacuuugagucgguacgaaucac...............	1	1	3rd
.....................................................................Uuacuuugagucgguacgaaucac...............	115	1	3rd
.....................................................................cuacuuugagucgguacgaCucac...............	1	1	3rd
.....................................................................cuacuuugagucgguacgaGucac...............	3	1	3rd
.....................................................................cuacuuugagucgguacgaaucacc..............	2	0	3rd
.....................................................................cuacuuugagucgguacgaaucacA..............	19	1	3rd
......................................................................uacuuugagucgAuacga....................	1	1	3rd
......................................................................uacuuugagucgguacga....................	659	0	3rd
......................................................................uacuuugagucgguGcga....................	2	1	3rd
......................................................................uacuuugagucgguacAa....................	1	1	3rd
......................................................................uacuuugCgucgguacga....................	1	1	3rd
......................................................................uacuuGgagucgguacga....................	2	1	3rd
......................................................................uacuuugUgucgguacga....................	1	1	3rd
......................................................................uacuuugagucgguaUga....................	1	1	3rd
......................................................................uacuuugagucUguacga....................	1	1	3rd
......................................................................uacuuugagucgguacgaG...................	1	1	3rd
......................................................................uacuuugagucgguacgGa...................	1	1	3rd
......................................................................uacuuugagucgguacgaU...................	1	1	3rd
......................................................................uacuuugagucggCacgaa...................	1	1	3rd
......................................................................uacuuugagucgguacgaa...................	536	0	3rd
......................................................................uacuuugagucgguUcgaa...................	1	1	3rd
......................................................................uacuuugagucgguaGgaa...................	1	1	3rd
......................................................................uacuuugGgucgguacgaa...................	1	1	3rd
```

```
                                 Star                                 Mature
ggaucaaaauaacuuaacgugguucaggggcgguuuguaccgacucagagcuagguucguugaugagaacuacuuugagucgguacgaaucaccucagccaugaucca
......................................................................uacuuugUgucgguacgaa...................  1      1   3rd
......................................................................Gacuuugagucgguacgaau..................  5      1   3rd
......................................................................uacuuugagucUguacgaau..................  2      1   3rd
......................................................................uacuuugagucgguacgaaA..................  267    1   3rd
......................................................................uacuuugagucgguCcgaau..................  1      1   3rd
......................................................................uacuuugagucgguUcgaau..................  5      1   3rd
......................................................................uacuuugagucgguacAaau..................  1      1   3rd
......................................................................uacuuugagucggGacgaau..................  2      1   3rd
......................................................................uacuuugagucgguacUaau..................  1      1   3rd
......................................................................uaGuuugagucgguacgaau..................  4      1   3rd
......................................................................uacuuugaguGgguacgaau..................  2      1   3rd
......................................................................uacuuugagucggAacgaau..................  1      1   3rd
......................................................................uacuuugagucgguaGgaau..................  1      1   3rd
......................................................................uacuuCgagucgguacgaau..................  7      1   3rd
......................................................................uacuuugUgucgguacgaau..................  8      1   3rd
......................................................................uacCuugagucgguacgaau..................  8      1   3rd
......................................................................uaUuuugagucgguacgaau..................  2      1   3rd
......................................................................uacuuugCgucgguacgaau..................  6      1   3rd
......................................................................uacuuugagucgguacgUau..................  1      1   3rd
......................................................................uacuuugaUucgguacgaau..................  1      1   3rd
......................................................................uaAuuugagucgguacgaau..................  1      1   3rd
......................................................................Aacuuugagucgguacgaau..................  1      1   3rd
......................................................................uacuuugagucgguacCaau..................  6      1   3rd
......................................................................uacuCugagucgguacgaau..................  1      1   3rd
......................................................................uacuuugagucAguacgaau..................  2      1   3rd
......................................................................uacuuGgagucgguacgaau..................  9      1   3rd
......................................................................uacuuugagucggCacgaau..................  3      1   3rd
......................................................................uacuuugagCcgguacgaau..................  1      1   3rd
......................................................................uacuuAgagucgguacgaau..................  14     1   3rd
......................................................................uacuuuAagucgguacgaau..................  2      1   3rd
......................................................................uacuuugagucgUuacgaau..................  5      1   3rd
......................................................................uacuuugGgucgguacgaau..................  11     1   3rd
......................................................................uacuuuUagucgguacgaau..................  4      1   3rd
......................................................................uacuuugagucgguacgaGu..................  2      1   3rd
......................................................................uacuuugagucgguaAgaau..................  2      1   3rd
......................................................................uacuuugagucgguacgGau..................  7      1   3rd
......................................................................uacuuugagucgCuacgaau..................  2      1   3rd
......................................................................uCcuuugagucgguacgaau..................  1      1   3rd
......................................................................uacuuugagucCguacgaau..................  1      1   3rd
......................................................................uacuuugaguAgguacgaau..................  3      1   3rd
......................................................................uacuuugagucgAuacgaau..................  1      1   3rd
......................................................................uGcuuugagucgguacgaau..................  3      1   3rd
......................................................................Cacuuugagucgguacgaau..................  2      1   3rd
......................................................................uacuuugagucgguaUgaau..................  8      1   3rd
......................................................................uacuuugaCucgguacgaau..................  2      1   3rd
......................................................................uacuuugagucgguacgaCu..................  2      1   3rd
......................................................................uacuuugagucgguacgaaC..................  7      1   3rd
......................................................................uacuuugagucgguacgaaG..................  12     1   3rd
......................................................................uacuuugagucgguacgaau..................  13049  0   3rd
......................................................................uacuuugagucgguGcgaau..................  14     1   3rd
......................................................................uacuuugagucgguacgaaCc.................  14     1   3rd
......................................................................uacuuugagucgguGcgaauc.................  16     1   3rd
......................................................................uacuuugagCcgguacgaauc.................  1      1   3rd
......................................................................uaAuuugagucgguacgaauc.................  2      1   3rd
......................................................................Aacuuugagucgguacgaauc.................  1      1   3rd
......................................................................uacuuugagAcgguacgaauc.................  2      1   3rd
......................................................................Gacuuugagucgguacgaauc.................  3      1   3rd
......................................................................uacuuugagucgUuacgaauc.................  6      1   3rd
......................................................................uacuuugagucgguacgaauU.................  47     1   3rd
......................................................................uacuuugagucgguacgaauc.................  25320  0   3rd
......................................................................uacuuugGgucgguacgaauc.................  18     1   3rd
......................................................................uacuuugagucgguacgaaGc.................  9      1   3rd
......................................................................uacuuAgagucgguacgaauc.................  27     1   3rd
......................................................................uacuuugagucggAacgaauc.................  1      1   3rd
......................................................................uacuuugCgucgguacgaauc.................  5      1   3rd
......................................................................uacuuugagucgguCcgaauc.................  2      1   3rd
......................................................................uacuuugagucgguacgaaAc.................  1      1   3rd
......................................................................uacuuugaCucgguacgaauc.................  3      1   3rd
......................................................................uacuuugagucgguacgaCuc.................  1      1   3rd
......................................................................uacuuugagucggGacgaauc.................  3      1   3rd
```

Star Mature

```
ggaucaaaauaacuuaacgugguucaggggcgguuuguaccgacucagagcuagguucguugaugagaacuacuuugagucgguacgaaucaccucagccaugaucca
......................................................................Cacuuugagucgguacgaauc.................	4	1	3rd
......................................................................uUcuuugagucgguacgaauc.................	1	1	3rd
......................................................................uacuuugUgucgguacgaauc.................	23	1	3rd
......................................................................uacuuugagucgguaAgaauc.................	2	1	3rd
......................................................................uacuuugagucgguacgUauc.................	4	1	3rd
......................................................................uacuuugagucgCuacgaauc.................	5	1	3rd
......................................................................uacuuGgagucgguacgaauc.................	23	1	3rd
......................................................................uacuuuUagucgguacgaauc.................	11	1	3rd
......................................................................uacuuugagucgguacgaauA.................	277	1	3rd
......................................................................uaGuuugagucgguacgaauc.................	5	1	3rd
......................................................................uCcuuugagucgguacgaauc.................	4	1	3rd
......................................................................uGcuuugagucgguacgaauc.................	5	1	3rd
......................................................................uacuuugagucgguacgaUuc.................	4	1	3rd
......................................................................uacuuugagucgguaUgaauc.................	9	1	3rd
......................................................................uacuuugagucgguacgaauG.................	10	1	3rd
......................................................................uacuuugagucgguUcgaauc.................	12	1	3rd
......................................................................uacuuuAagucgguacgaauc.................	4	1	3rd
......................................................................uacuuugaguAgguacgaauc.................	2	1	3rd
......................................................................uaUuuugagucgguacgaauc.................	7	1	3rd
......................................................................uacuuuCagucgguacgaauc.................	6	1	3rd
......................................................................uacuuugagucgAuacgaauc.................	2	1	3rd
......................................................................uacuuCgagucgguacgaauc.................	21	1	3rd
......................................................................uacuuugagucgguacgaGuc.................	8	1	3rd
......................................................................uacuuugagucgguacgGauc.................	13	1	3rd
......................................................................uacuuugagucgguacCaauc.................	7	1	3rd
......................................................................uacuGugagucgguacgaauc.................	4	1	3rd
......................................................................uacuuugagucgguacgCauc.................	4	1	3rd
......................................................................uacuuugagucgguaGgaauc.................	3	1	3rd
......................................................................uacuuugagucgguacAaauc.................	5	1	3rd
......................................................................uacCuugagucgguacgaauc.................	13	1	3rd
......................................................................uacuuugagucCguacgaauc.................	3	1	3rd
......................................................................uacuuugagucUguacgaauc.................	1	1	3rd
......................................................................uacuuugagucAguacgaauc.................	4	1	3rd
......................................................................uacuuugagucgCuacgaauca................	46	1	3rd
......................................................................uacuuugagucgguUcgaauca................	55	1	3rd
......................................................................uacuuugagucgguacgaauAa................	83	1	3rd
......................................................................uacuGugagucgguacgaauca................	6	1	3rd
......................................................................uacuuugCgucgguacgaauca................	72	1	3rd
......................................................................uacuuuUagucgguacgaauca................	74	1	3rd
......................................................................uacuuugagCcgguacgaauca................	12	1	3rd
......................................................................Cacuuugagucgguacgaauca................	35	1	3rd
......................................................................uacuuugagucgguacgaaCca................	87	1	3rd
......................................................................uacuuugagucUguacgaauca................	13	1	3rd
......................................................................uacuCugagucgguacgaauca................	22	1	3rd
......................................................................uaUuuugagucgguacgaauca................	46	1	3rd
......................................................................Gacuuugagucgguacgaauca................	50	1	3rd
......................................................................uacuuugagucgguacgUauca................	30	1	3rd
......................................................................uacuuugagucgguaGgaauca................	15	1	3rd
......................................................................uacuuugGgucgguacgaauca................	148	1	3rd
......................................................................uacuuugagAcgguacgaauca................	7	1	3rd
......................................................................uacuuugagucgguacgaCuca................	25	1	3rd
......................................................................uacGuugagucgguacgaauca................	5	1	3rd
......................................................................uacCuugagucgguacgaauca................	86	1	3rd
......................................................................Aacuuugagucgguacgaauca................	16	1	3rd
......................................................................uacuuugagucgguacgaauca................	213656	0	3rd
......................................................................uacuuugagucgguaAgaauca................	22	1	3rd
......................................................................uacuuugagucggCacgaauca................	37	1	3rd
......................................................................uCcuuugagucgguacgaauca................	11	1	3rd
......................................................................uacuuugaAucgguacgaauca................	6	1	3rd
......................................................................uacuuugaCucgguacgaauca................	21	1	3rd
......................................................................uacuuugagucAguacgaauca................	15	1	3rd
......................................................................uacuuugagucggAacgaauca................	21	1	3rd
......................................................................uacuuugagucgguGcgaauca................	140	1	3rd
......................................................................uacuuugagucgguacUaauca................	16	1	3rd
......................................................................uacuuGgagucgguacgaauca................	109	1	3rd
......................................................................uGcuuugagucgguacgaauca................	50	1	3rd
......................................................................uacuuCgagucgguacgaauca................	137	1	3rd
......................................................................uacuuugagGcgguacgaauca................	11	1	3rd
......................................................................uaGuuugagucgguacgaauca................	27	1	3rd
......................................................................uacuuugaguGgguacgaauca................	16	1	3rd
```

```
                                Star                                Mature
ggaucaaaauaacuuaacgugguucaggggcgguuuguaccgacucagagcuagguucguugaugagaacuacuuugagucgguacgaaucaccucagccaugaucca
......................................................................uacuuugagucgguacgaaucG................	80	1	3rd
......................................................................uacuuugUgucgguacgaauca................	190	1	3rd
......................................................................uacuuugagucgguacgaauUa................	52	1	3rd
......................................................................uacuuugaguAgguacgaauca................	23	1	3rd
......................................................................uacuuugagucgguacgaaGca................	83	1	3rd
......................................................................uacuuugagucgguacgaUuca................	4	1	3rd
......................................................................uacuuugagucgguacCaauca................	45	1	3rd
......................................................................uacuuugagucgguacgaaAca................	9	1	3rd
......................................................................uacuuuAagucgguacgaauca................	19	1	3rd
......................................................................uacuuugagucgguacgaauGa................	11	1	3rd
......................................................................uacuuugagucCguacgaauca................	16	1	3rd
......................................................................uacuuugagucgguacgaaucC................	13	1	3rd
......................................................................uacuuuCagucgguacgaauca................	44	1	3rd
......................................................................uacuuAgagucgguacgaauca................	155	1	3rd
......................................................................uacuuugagucgguaUgaauca................	69	1	3rd
......................................................................uacuuugagucgUuacgaauca................	27	1	3rd
......................................................................uacuuugaUucgguacgaauca................	8	1	3rd
......................................................................uacuuugagucggGacgaauca................	13	1	3rd
......................................................................uacuuugagucgguacgGauca................	99	1	3rd
......................................................................uUcuuugagucgguacgaauca................	11	1	3rd
......................................................................uacuAugagucgguacgaauca................	1	1	3rd
......................................................................uacuuugagucgguacAaauca................	31	1	3rd
......................................................................uacuuugagucgguacgCauca................	45	1	3rd
......................................................................uacuuugagucgguacgaaucU................	655	1	3rd
......................................................................uacuuugagucgAuacgaauca................	33	1	3rd
......................................................................uacuuugagucgguacgaGuca................	65	1	3rd
......................................................................uaAuuugagucgguacgaauca................	5	1	3rd
......................................................................uacuuugagucgguCcgaauca................	8	1	3rd
......................................................................uacuuugagucgAuacgaaucac...............	15	1	3rd
......................................................................uacuuugagGcgguacgaaucac...............	3	1	3rd
......................................................................uacuuugaAucgguacgaaucac...............	6	1	3rd
......................................................................uacuuugagucgguacgaaAcac...............	3	1	3rd
......................................................................uacuuCgagucgguacgaaucac...............	83	1	3rd
......................................................................uacuuugagucgguacgaauAac...............	10	1	3rd
......................................................................uacuuugagucgguacgaaucaA...............	5269	1	3rd
......................................................................uaUuuugagucgguacgaaucac...............	18	1	3rd
......................................................................uacuuugagucgguacgCaucac...............	17	1	3rd
......................................................................uacuuugCgucgguacgaaucac...............	58	1	3rd
......................................................................uacuuuUagucgguacgaaucac...............	75	1	3rd
......................................................................uacuuugagucgguacCaaucac...............	35	1	3rd
......................................................................uacuuuAagucgguacgaaucac...............	17	1	3rd
......................................................................Gacuuugagucgguacgaaucac...............	19	1	3rd
......................................................................uacuuugUgucgguacgaaucac...............	185	1	3rd
......................................................................uacuuugagucgguaAgaaucac...............	24	1	3rd
......................................................................uacuuugagucgguUcgaaucac...............	33	1	3rd
......................................................................uacuuugagCcgguacgaaucac...............	5	1	3rd
......................................................................uacuuugGgucgguacgaaucac...............	180	1	3rd
......................................................................uUcuuugagucgguacgaaucac...............	12	1	3rd
......................................................................uacuuuCagucgguacgaaucac...............	18	1	3rd
......................................................................uacuuugagucgguacgaaucGc...............	11	1	3rd
......................................................................uacuuugagucgguaGgaaucac...............	10	1	3rd
......................................................................uacGuugagucgguacgaaucac...............	7	1	3rd
......................................................................uacuuugaCucgguacgaaucac...............	12	1	3rd
......................................................................uacuCugagucgguacgaaucac...............	12	1	3rd
......................................................................uacuuGgagucgguacgaaucac...............	67	1	3rd
......................................................................uacuuAgagucgguacgaaucac...............	97	1	3rd
......................................................................uacuuugagucgguacAaaucac...............	17	1	3rd
......................................................................uacuuugagucgguacgaauUac...............	29	1	3rd
......................................................................uacuuugagucgguacgaaGcac...............	57	1	3rd
......................................................................uacuuugagucgguaUgaaucac...............	39	1	3rd
......................................................................uacuuugagucgguacgaaucCc...............	14	1	3rd
......................................................................uacuuugagucgguGcgaaucac...............	115	1	3rd
......................................................................uacuuugagucggCacgaaucac...............	14	1	3rd
......................................................................uacuuugagucggGacgaaucac...............	11	1	3rd
......................................................................Cacuuugagucgguacgaaucac...............	20	1	3rd
......................................................................uacuuugagucgguacgGaucac...............	72	1	3rd
......................................................................uaAuuugagucgguacgaaucac...............	8	1	3rd
......................................................................uacuuugagucgguacgUaucac...............	13	1	3rd
......................................................................uacuuugagucgCuacgaaucac...............	16	1	3rd
......................................................................uaGuuugagucgguacgaaucac...............	4	1	3rd
```

Star **Mature**

ggaucaaaauaacuuaacgugguucaggggcgguuuguaccgacucagagcuagguucguugaugagaacuacuuugagucgguacgaaucaccucagccaugaucca			
....uacuuugaUucgguacgaaucac...............	7	1	3rd
....uacuuugagucggAacgaaucac...............	12	1	3rd
....uacuuugagucCguacgaaucac...............	7	1	3rd
....uGcuuugagucgguacgaaucac...............	22	1	3rd
....uacuuugagucgUuacgaaucac...............	13	1	3rd
....uacuuugagucgguacUaaucac...............	4	1	3rd
....uacuuugagucgguacgaUucac...............	7	1	3rd
....uacuuugagucUguacgaaucac...............	9	1	3rd
....uCcuuugagucgguacgaaucac...............	4	1	3rd
....uacuuugagucgguCcgaaucac...............	4	1	3rd
....uacuuugagucAguacgaaucac...............	11	1	3rd
....uacuuugagAcgguacgaaucac...............	1	1	3rd
....uacuuugagucgguacgaCucac...............	13	1	3rd
....uacuuugagucgguacgaaucUc...............	13	1	3rd
....uacAuugagucgguacgaaucac...............	2	1	3rd
....uacuGugagucgguacgaaucac...............	7	1	3rd
....uacuuugaguGgguacgaaucac...............	13	1	3rd
....uacuuugagucgguacgaaucac...............	130667	0	3rd
....uacuuugaguAgguacgaaucac...............	16	1	3rd
....uacCuugagucgguacgaaucac...............	60	1	3rd
....uacuuugagucgguacgaaCcac...............	48	1	3rd
....uacuuugagucgguacgaaucaG...............	49	1	3rd
....uacuuugagucgguacgaGucac...............	16	1	3rd
....Aacuuugagucgguacgaaucac...............	8	1	3rd
....uacuuugagucgguacgaauGac...............	17	1	3rd
....uacuuugagucgguacgaaucaUc..............	3	1	3rd
....uacuuugagucgguacgaaucacA..............	5435	1	3rd
....Gacuuugagucgguacgaaucacc..............	1	1	3rd
....uacuuugagucgguacgaaucacG..............	3	1	3rd
....uacuuugagucgguacgaaucacc..............	48	0	3rd
....uacuuugagucgguacgaaucaAc..............	1	1	3rd
....uacuuugagucgguacgaaucacAu.............	27	1	3rd
....uacuuugagucgguacgaaucaccA.............	18	1	3rd
....uacuuugagucgguacgaaucaccu.............	6	0	3rd
....acuuugagucgguacgaa...................	1	0	3rd
....acuuugagucgguacgaau..................	23	0	3rd
....acuuugagucgguacgaaA..................	1	1	3rd
....Ucuuugagucgguacgaau..................	1	1	3rd
....acuuugagucgguacgaauc.................	52	0	3rd
....aAuuugagucgguacgaauc.................	1	1	3rd
....acuuugagucgguacgaauA.................	2	1	3rd
....Ucuuugagucgguacgaauc.................	1	1	3rd
....acuuugUgucgguacgaauca................	1	1	3rd
....acuuugGgucgguacgaauca................	1	1	3rd
....acuuugagucgguacgaauca................	371	0	3rd
....acuuugagucgguGcgaauca................	1	1	3rd
....Ucuuugagucgguacgaauca................	1	1	3rd
....aGuuugagucgguacgaauca................	1	1	3rd
....acuuugagucgguacgaaucU................	2	1	3rd
....acuuugagucgguacgaauUa................	1	1	3rd
....acuuugagucCguacgaauca................	1	1	3rd
....acuuugagucAguacgaauca................	1	1	3rd
....acuuuAagucgguacgaauca................	1	1	3rd
....acuuugUgucgguacgaaucac...............	2	1	3rd
....Ucuuugagucgguacgaaucac...............	1	1	3rd
....acuuugagucgguacgaaucaA...............	4	1	3rd
....acuuugagucCguacgaaucac...............	1	1	3rd
....aAuuugagucgguacgaaucac...............	1	1	3rd
....acuuuCagucgguacgaaucac...............	2	1	3rd
....acuuugagucgguGcgaaucac...............	1	1	3rd
....acuuugagucgguaUgaaucac...............	1	1	3rd
....acuuugagucgguacgaaucac...............	568	0	3rd
....acuuugagucgguacUaaucac...............	1	1	3rd
....acuuugagucgguacgaauUac...............	1	1	3rd
....acCuugagucgguacgaaucac...............	1	1	3rd
....aGuuugagucgguacgaaucac...............	2	1	3rd
....acuuugGgucgguacgaaucac...............	2	1	3rd
....acuuugagucgguacgaaucacA..............	19	1	3rd
....cuuugagucgguacgGau..................	1	1	3rd
....cuuugagucgguacgaau..................	9	0	3rd

```
                              Star                                Mature
ggaucaaaauaacuuaacgugguucaggggcgguuuguaccgacucagagcuagguucguugaugagaacuacuuugagucgguacgaaucaccucagccaugaucca
........................................................................cuuugagucgguacgaauc.................   17    0   3rd
........................................................................Uuuugagucgguacgaauc.................   1     1   3rd
........................................................................Uuuugagucgguacgaauca................   5     1   3rd
........................................................................cuuugagucgguacgaauca................   67    0   3rd
........................................................................cuuugagucgguacgaaucac...............   88    0   3rd
........................................................................Uuuugagucgguacgaaucac...............   1     1   3rd
........................................................................cuuugagucgguGcgaaucac...............   1     1   3rd
........................................................................cuuuUagucgguacgaaucac...............   1     1   3rd
........................................................................cuuugagucgguacgaaucacA..............   13    1   3rd
........................................................................cuuugagucgguacgaaucacc..............   2     0   3rd
........................................................................cuuugagucgguacgaaucacAu.............   1     1   3rd
.........................................................................uuugagucgguacgaauc.................   18    0   3rd
.........................................................................uuugagucgguacgaauca................   118   0   3rd
.........................................................................uuugagucgguacgaaucac...............   191   0   3rd
.........................................................................uuuCagucgguacgaaucac...............   1     1   3rd
.........................................................................uuugagucgguacgaGucac...............   1     1   3rd
.........................................................................uuugagucgguacgaaucaA...............   6     1   3rd
.........................................................................uuugagucgguacgaaucacA..............   12    1   3rd
.........................................................................uuugagucgguacgaaucacc..............   2     0   3rd
.........................................................................uuugagucgguacgaaucaccA.............   2     1   3rd
.........................................................................uuugagucgguacgaaucaccu.............   4     0   3rd
.........................................................................uuugagucgguacgaaucacAu.............   1     1   3rd
.........................................................................uuugagucgguacgaaucacGu.............   1     1   3rd
..........................................................................Gugagucgguacgaauca................   1     1   3rd
..........................................................................uuUagucgguacgaauca................   1     1   3rd
..........................................................................uugagucgguacgaauca................   145   0   3rd
..........................................................................uugGgucgguacgaaucac...............   1     1   3rd
..........................................................................uugCgucgguacgaaucac...............   1     1   3rd
..........................................................................uuCagucgguacgaaucac...............   1     1   3rd
..........................................................................uugaCucgguacgaaucac...............   1     1   3rd
..........................................................................uugagucgguacgaaucaA...............   3     1   3rd
..........................................................................uugagucgguCcgaaucac...............   1     1   3rd
..........................................................................uugUgucgguacgaaucac...............   1     1   3rd
..........................................................................uugagucgguacgaaucac...............   448   0   3rd
..........................................................................uugagCcgguacgaaucac...............   2     1   3rd
..........................................................................uugagucgguaUgaaucac...............   1     1   3rd
..........................................................................uugagucgguacgaaucacA..............   6     1   3rd
..........................................................................uugagucgguacgaaucaccA.............   1     1   3rd
...........................................................................uUagucgguacgaaucac...............   1     1   3rd
...........................................................................ugagucgguacgaaucac...............   19    0   3rd
...........................................................................ugagucgguacgaaucaA...............   1     1   3rd

.....................................................................cuacuuugagucgguacgaau..................   1     0   BA1
.....................................................................cuacuuugagucgguacgaauc.................   2     0   BA1
.....................................................................cuacuuugagucgguacgaauca................   7     0   BA1
.....................................................................cuacCuugagucgguacgaaucac...............   1     1   BA1
.....................................................................cuacuuugagucgguacgaaucac...............   8     0   BA1
......................................................................uacuuugagucgguacga....................   1     0   BA1
......................................................................uacuuugagucgguacgaa...................   6     0   BA1
......................................................................uacuuugagucgguacgaau..................   38    0   BA1
......................................................................uacuuugagucgguacgaaC..................   3     1   BA1
......................................................................uacuuugagucgguacgaaGc.................   1     1   BA1
......................................................................uacCuugagucgguacgaauc.................   3     1   BA1
......................................................................uacuCugagucgguacgaauc.................   1     1   BA1
......................................................................uacuuugagucgguacgaauU.................   8     1   BA1
......................................................................uacuuugagucgguacgaauc.................   132   0   BA1
......................................................................uacuuugagucgguaUgaauc.................   1     1   BA1
......................................................................uacuuugagucgguacgaauA.................   3     1   BA1
......................................................................uacuuugagucgguacgaGuc.................   1     1   BA1
......................................................................uacuuugagucgguGcgaauca................   1     1   BA1
......................................................................uacCuugagucgguacgaauca................   3     1   BA1
......................................................................uacuuugagucgguacgaaucG................   10    1   BA1
......................................................................uacuCugagucgguacgaauca................   2     1   BA1
......................................................................uacuuugagucgguacgaaucU................   5     1   BA1
......................................................................Aacuuugagucgguacgaauca................   1     1   BA1
......................................................................uacuuugagCcgguacgaauca................   1     1   BA1
......................................................................uacuuugagucgguacgaauca................   289   0   BA1
......................................................................uacuuugagucgguaUgaauca................   1     1   BA1
......................................................................uacuuCgagucgguacgaauca................   1     1   BA1
......................................................................uacuuugagucggCacgaauca................   1     1   BA1
```

Star Mature

```
ggaucaaaauaacuuaacgugguucaggggcgguuuguaccgacucagagcuagguucguugaugagaacuacuuugagucgguacgaaucaccucagccaugaucca
......................................................................uacuuugagucgguacgaaucGc...............    1     1     BA1
......................................................................Cacuuugagucgguacgaaucac...............    1     1     BA1
......................................................................uacuuugagucgguacgaaucaA...............    10    1     BA1
......................................................................uacuuugagucgguGcgaaucac...............    1     1     BA1
......................................................................uacGuugagucgguacgaaucac...............    1     1     BA1
......................................................................uacuuugagucgguacgaaucac...............    41    0     BA1
......................................................................uacuuugagucgguacgaaucacc..............    5     0     BA1
......................................................................uacuuugagucgguacgaaucacA..............    2     1     BA1
.........................................................................uuugagucgguacgaaucacc..............    1     0     BA1

.....................................................................cuacuuugagucgguacga....................    1     0     BRA
.....................................................................cuacuuugagucgguacgaau..................    1     0     BRA
.....................................................................cuacuuugagucgguacgaaG..................    1     1     BRA
.....................................................................cuacuuugagucgguGcgaauc.................    1     1     BRA
.....................................................................cuacuuugagucgguacgaauc.................    20    0     BRA
.....................................................................cuacuuugagucgguacgaauU.................    2     1     BRA
.....................................................................cuacuuugagucgguacgaauca................    88    0     BRA
.....................................................................cuGcuuugagucgguacgaauca................    1     1     BRA
.....................................................................cuacuuugagucggCacgaauca................    1     1     BRA
.....................................................................cuacuCugagucgguacgaauca................    1     1     BRA
.....................................................................cuacuuAgagucgguacgaauca................    1     1     BRA
.....................................................................cuacuuugagucgguacgaaucU................    1     1     BRA
.....................................................................cuacuuugagucgguacgaaucG................    1     1     BRA
.....................................................................cuacuCugagucgguacgaaucac...............    2     1     BRA
.....................................................................cuacuuugagucgguacgaaucac...............    49    0     BRA
.....................................................................cuacuuugagucgguacgaaucaA...............    3     1     BRA
.....................................................................cuGcuuugagucgguacgaaucac...............    1     1     BRA
.....................................................................cuacuuugagucgguacgaaucaUc..............    1     1     BRA
.....................................................................cuacuuugagucgguacgaaucacA..............    2     1     BRA
.....................................................................cuacuuugagucgguacgaaucacG..............    1     1     BRA
......................................................................uacuuugagucgguacga....................    8     0     BRA
......................................................................uacuuugagucgguGcgaa...................    1     1     BRA
......................................................................uacuuugagucgguaUgaa...................    1     1     BRA
......................................................................uacuuugagucgguacgaa...................    7     0     BRA
......................................................................uacuuugagucgguacgaau..................    337   0     BRA
......................................................................uacuuugagucgguacgGau..................    2     1     BRA
......................................................................uacuuugagucggCacgaau..................    1     1     BRA
......................................................................Aacuuugagucgguacgaau..................    1     1     BRA
......................................................................Cacuuugagucgguacgaau..................    2     1     BRA
......................................................................uacuuugagucgguacAaau..................    1     1     BRA
......................................................................uacuCugagucgguacgaau..................    1     1     BRA
......................................................................uacuuugaguAgguacgaau..................    1     1     BRA
......................................................................uacuuugagCcgguacgaau..................    2     1     BRA
......................................................................uacuuugagucgguacgaGu..................    1     1     BRA
......................................................................uacuuugGgucgguacgaau..................    1     1     BRA
......................................................................uacuuugagucgguacgaaG..................    5     1     BRA
......................................................................uacuuugagucgguGcgaau..................    2     1     BRA
......................................................................uacuuugagucgguacgaaA..................    9     1     BRA
......................................................................uacuuCgagucgguacgaau..................    1     1     BRA
......................................................................uacuuugagucgguaGgaau..................    1     1     BRA
......................................................................uGcuuugagucgguacgaau..................    1     1     BRA
......................................................................uacuuugagucAguacgaau..................    1     1     BRA
......................................................................uacuuugagucgguacgaaC..................    43    1     BRA
......................................................................uacuuugagucgguacgaaGc.................    3     1     BRA
......................................................................uacuuugGgucgguacgaauc.................    6     1     BRA
......................................................................uacCuugagucgguacgaauc.................    4     1     BRA
......................................................................uacuuugagucgguacgaGuc.................    7     1     BRA
......................................................................uacuuugagucggAacgaauc.................    1     1     BRA
......................................................................uacuuugagucgCuacgaauc.................    1     1     BRA
......................................................................uacuuAgagucgguacgaauc.................    2     1     BRA
......................................................................uGcuuugagucgguacgaauc.................    2     1     BRA
......................................................................uacuuugagucAguacgaauc.................    1     1     BRA
......................................................................uacuuugagucgguaUgaauc.................    3     1     BRA
......................................................................uacuuugagAcgguacgaauc.................    1     1     BRA
......................................................................uacuuugagCcgguacgaauc.................    3     1     BRA
......................................................................uacuuugagucgguacgaaCc.................    3     1     BRA
......................................................................uacuuugagucgguacgaauA.................    18    1     BRA
......................................................................uacuuugagucgguacgGauc.................    4     1     BRA
......................................................................uacuuugagucggCacgaauc.................    4     1     BRA
......................................................................uacuuCgagucgguacgaauc.................    2     1     BRA
```

Star Mature

ggaucaaaauaacuuaacgugguucaggggcgguuuguaccgacucagagcuagguucguugaugagaacuacuuugagucgguacgaaucaccucagccaugaucca			
....uacuuugagucgguacgaauU.................	75	1	BRA
....uacuuugagucgguGcgaauc.................	3	1	BRA
....uacuCugagucgguacgaauc.................	10	1	BRA
....uacuuugagucgguacAaauc.................	1	1	BRA
....uacuuugagucgguacgaauc.................	1143	0	BRA
....uacuuugagucggCacgaauca................	6	1	BRA
....uacuuugagucgAuacgaauca................	3	1	BRA
....uacuuugagucggAacgaauca................	1	1	BRA
....uGcuuugagucgguacgaauca................	5	1	BRA
....uaAuuugagucgguacgaauca................	2	1	BRA
....uacuuugCgucgguacgaauca................	1	1	BRA
....uacuuuAagucgguacgaauca................	1	1	BRA
....uacuuugagucgguacgaaucU................	41	1	BRA
....uacuuugUgucgguacgaauca................	2	1	BRA
....uacCuugagucgguacgaauca................	12	1	BRA
....uacuuugagucgguacgaaucG................	53	1	BRA
....uacuuugagucgguacgaGuca................	7	1	BRA
....uacuuugagucgguaUgaauca................	8	1	BRA
....uaUuuugagucgguacgaauca................	5	1	BRA
....uacuuugagucAguacgaauca................	2	1	BRA
....uaGuuugagucgguacgaauca................	1	1	BRA
....uacuuugagCcgguacgaauca................	6	1	BRA
....uacuuugagucgguacAaauca................	1	1	BRA
....uacuuugagucgguacgaauca................	2887	0	BRA
....uCcuuugagucgguacgaauca................	1	1	BRA
....uacuuugagucgguacgaauUa................	4	1	BRA
....uacuuugagucgguGcgaauca................	11	1	BRA
....uacuCugagucgguacgaauca................	7	1	BRA
....uacuuugagucgguacgGauca................	8	1	BRA
....Cacuuugagucgguacgaauca................	9	1	BRA
....uacuuugagucgguacgaaCca................	14	1	BRA
....uacuuugagAcgguacgaauca................	1	1	BRA
....Aacuuugagucgguacgaauca................	2	1	BRA
....uacuuAgagucgguacgaauca................	1	1	BRA
....uacuuugagucgguacgaaucC................	7	1	BRA
....uacuuCgagucgguacgaauca................	10	1	BRA
....uacAuugagucgguacgaauca................	1	1	BRA
....uacuuugGgucgguacgaauca................	5	1	BRA
....uacuuugaUucgguacgaauca................	1	1	BRA
....uacuuugaguAgguacgaauca................	1	1	BRA
....uacuAugagucgguacgaauca................	1	1	BRA
....uacuuugagucgguGcgaaucac...............	3	1	BRA
....uacuuugagucgguacgGaucac...............	1	1	BRA
....uacuuugGgucgguacgaaucac...............	1	1	BRA
....uacuuugagucgguacgaGucac...............	3	1	BRA
....uacuuugagucgguacgaaucaG...............	4	1	BRA
....uacuuugagucgguacgaaucac...............	205	0	BRA
....uacCuugagucgguacgaaucac...............	1	1	BRA
....uacuuugagucgguacgaaucUc...............	3	1	BRA
....uacuuugagucgguacgaaucaA...............	67	1	BRA
....uUcuuugagucgguacgaaucac...............	1	1	BRA
....uacuuugagucgguacgaaucacA..............	7	1	BRA
....uacuuugagucgguacgaaucacc..............	9	0	BRA
....uacuuugagucggCacgaaucacc..............	2	1	BRA
....uacuuugagucgguacgaaucacG..............	1	1	BRA
....uacuuugagucgguacgaaucaccA.............	1	1	BRA
....acuuugagucgguacgaauc.................	2	0	BRA
....acuuugagucgguacgaauca................	2	0	BRA
....acuuugagucgguacgaaucac...............	1	0	BRA
....uuugagucgguacgaaucac...............	1	0	BRA
....uuugagucgguacgaaucacc..............	2	0	BRA
....uuugagucgguGcgaaucacc..............	1	1	BRA
....uuugagucgguacgaaucaccA.............	1	1	BRA

Provisional ID	:	Scaffold_11093_2695
Score total	:	528.2
Score for star read(s)	:	3.9
Score for read counts	:	518.1
Score for mfe	:	1.6
Score for randfold	:	1.6
Score for cons. seed	:	3
Total read count	:	1028
Mature read count	:	875
Loop read count	:	0
Star read count	:	153

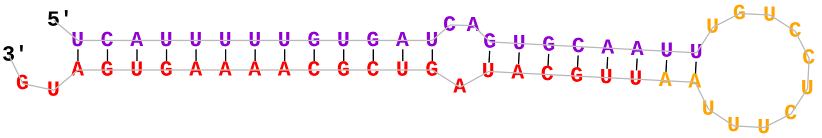

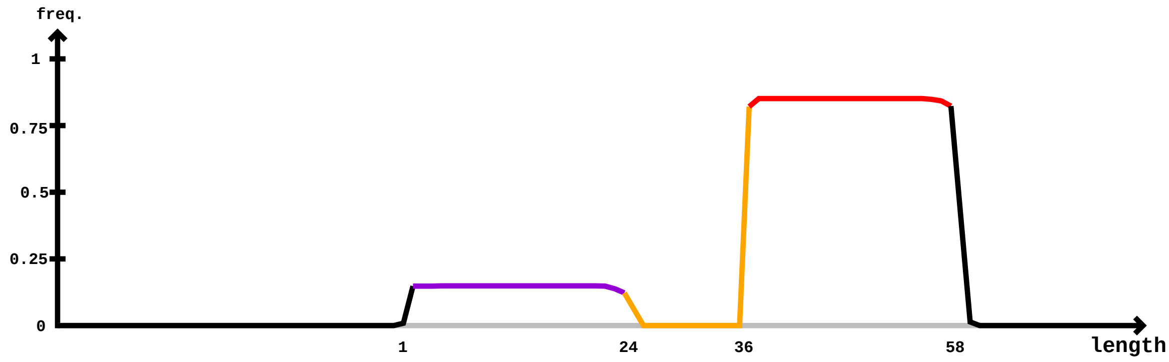

Star   Mature

```
5'- ggauucaauaaaaccucaucauccuuuuucgccaaucauuuuugugaucagugcaauuuguccucuuuaauugcauagucgcaaaagugaugggugg aguuggacaacaca   -3'   obs
    ggauucaauaaaaccucaucauccuuuuucgccaaucauuuuugugaucagugcaauuuguccucuuuaauugcauagucgcaaaagugaugggugg aguuggacaacaca         exp
    .....................(((..(((((((.((((((((((((((..((((((((..........)))))))).)))))))))))))).))))))))..))).......   reads   mm   sample
    ...................................ucauuuuugugaucagugcaauu.....................................................   4       0    G01
    ......................................uuuuugugaucagugcaauu.....................................................   1       0    G01
    ......................................................................uugcauagucgcaaaagugaGg...................   1       1    G01
    ......................................................................uugcauagucgcaaaagugaug...................   19      0    G01
    ......................................................................uugcauagucgcaaaagugauggU.................   1       1    G01

    ......................................................................uugcauagucgcaaaagugaug...................   1       0    HA1

    ..................................aucauuuuugugaucagugcaauu.....................................................   1       0    3rd
    ...................................ucauuuuugugaucagugcaa.......................................................   1       0    3rd
    ...................................ucauuuuugugaucagugcaau......................................................   4       0    3rd
    ...................................ucauuuuugugaucagugcaauu.....................................................   20      0    3rd
    ......................................................................uugcauagucgcaaaagug......................   2       0    3rd
    ......................................................................uugcauagucgcaaaagugau....................   3       0    3rd
    ......................................................................uugcauagucgcaaaagugaug...................   80      0    3rd
    ......................................................................uugcauagucgcaaaagugUug...................   1       1    3rd
    ......................................................................uugcauagucgcaaaagugaugg..................   31      0    3rd
    ......................................................................uugcauagucgcaaaagugauggC.................   1       1    3rd
    .......................................................................ugcauagucgcaaaagugaug...................   4       0    3rd

    ..................................aucauuuuugugaucagugcaauu.....................................................   2       0    EGG
    ...................................ucauuuuugugaucagugcaau......................................................   1       0    EGG
    ...................................ucauuuuugugaucagugcaauu.....................................................   25      0    EGG
    ......................................................................uugcauagucgcaaaagugau....................   2       0    EGG
    ......................................................................uugcauagucgcaaaagugaug...................   111     0    EGG
    ......................................................................uugcauagucgcaaaagugaugg..................   69      0    EGG
    ......................................................................uugcauagucgcaaaagugauggA.................   1       1    EGG
    .......................................................................ugcauagucgcaaaagugaug...................   3       0    EGG
    .......................................................................ugcauagucgcaaaagugaugg..................   5       0    EGG
    .......................................................................ugcauagucgcaaaagugauggA.................   1       1    EGG

    ..................................aucauuuuugugaucagugcaauu.....................................................   1       0    2nd
    ...................................ucauuuuugugaucagugcaa.......................................................   5       0    2nd
```

```
                                   Star                              Mature
ggauucaauaaaaccucaucauccuuuuucgccaaucauuuuugugaucagugcaauuuguccucuuuaauugcauagucgcaaaagugauggguggaguuggacaacaca
...................................ucauuuuugugaucagugcaU.......................................................    1    1    2nd
...................................ucauuuuugugaucagugcaau......................................................    2    0    2nd
...................................ucauuuuugugaucagugcaauu.....................................................    5    0    2nd
......................................................................uugcauagucgcaaaagug......................    1    0    2nd
......................................................................uugcauagucgcaaaaguga.....................    3    0    2nd
......................................................................uugcauagucgcaaaagugau....................    6    0    2nd
......................................................................uugcauagucgcaaUagugaug...................    1    1    2nd
......................................................................uugcauagucgcaaaagugaug...................    72   0    2nd
......................................................................uugcauagucgcaaaagugauA...................    2    1    2nd
......................................................................uugcauagucgcaaaagugaugg..................    38   0    2nd
......................................................................uugcauagucgcaaaagugauggC.................    1    1    2nd
......................................................................uugcauagucgcaaaagugauggA.................    1    1    2nd
.......................................................................ugcauagucgcaaaagugaugg..................    2    0    2nd

......................................................................uugcauagucgcaaaagugaug...................    2    0    H01
......................................................................uugcauagucgcaaaagugauggA.................    1    1    H01

...................................ucauuuuugugaucagugca........................................................    1    0    1st
...................................ucauuuuugugaucagugcaa.......................................................    2    0    1st
...................................ucauuuuugugaucagugcaau......................................................    4    0    1st
...................................ucauuuuugugaucagugcaauu.....................................................    32   0    1st
......................................................................uugcauagucgcaaaaguga.....................    3    0    1st
......................................................................uugcauagucgcaaaagugau....................    9    0    1st
......................................................................uugcauagucgUaaaagugaug...................    1    1    1st
......................................................................uugcauagucgcaaaagugaug...................    183  0    1st
......................................................................uugcauagucgcaaGagugaug...................    1    1    1st
......................................................................uugcauagucgcaaaagugauA...................    1    1    1st
......................................................................uugUauagucgcaaaagugaug...................    1    1    1st
......................................................................uugcauagucgcaaaagugUug...................    1    1    1st
......................................................................uugcauagucgcaaCagugaugg..................    1    1    1st
......................................................................uugcauagucgcaaaagugaugC..................    1    1    1st
......................................................................uugcauagucgcaaaagugaugg..................    90   0    1st
......................................................................uugcauagucgcaaaagugauggU.................    3    1    1st
......................................................................uugcauagucgcaaaagugauggA.................    1    1    1st
.......................................................................ugcauagucgcaaaagugaug...................    9    0    1st
.......................................................................ugcauagucgcaaaagugaugg..................    4    0    1st
.......................................................................ugcauagucgcaaaagugauggA.................    1    1    1st

..................................aucauuuuugugaucagugcaa.......................................................    1    0    E01
..................................aucauuuuugugaucagugcaauu.....................................................    4    0    E01
...................................ucauuuuugugaucagugcaau......................................................    4    0    E01
...................................Gcauuuuugugaucagugcaau......................................................    1    1    E01
...................................ucauuuuugugaucGgugcaauu.....................................................    1    1    E01
...................................ucauuuuugugaucagugcaauu.....................................................    30   0    E01
......................................................................uugcauagucgcaaaagugaug...................    64   0    E01
......................................................................uugcauagucgcaaaagugaugC..................    1    1    E01
......................................................................uugcauagucgcaaaagugaugg..................    24   0    E01
......................................................................uugcauagucgcaaaagugauggA.................    2    1    E01
.......................................................................ugcauagucgcaaaagugaug...................    1    0    E01
.......................................................................ugcauagucgcaaaagugaugg..................    1    0    E01

......................................................................uugcauagucgcaaaagugaug...................    1    0    TA1
......................................................................uugcauagucgcaaaagugaugg..................    5    0    TA1
```

Provisional ID : Scaffold_11093_2701
Score total : 9590.6
Score for star read(s) : 3.9
Score for read counts : 9580.3
Score for mfe : 1.8
Score for randfold : 1.6
Score for cons. seed : 3
Total read count : 18803
Mature read count : 18353
Loop read count : 0
Star read count : 450

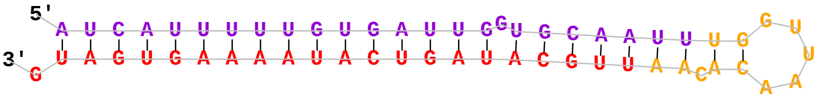

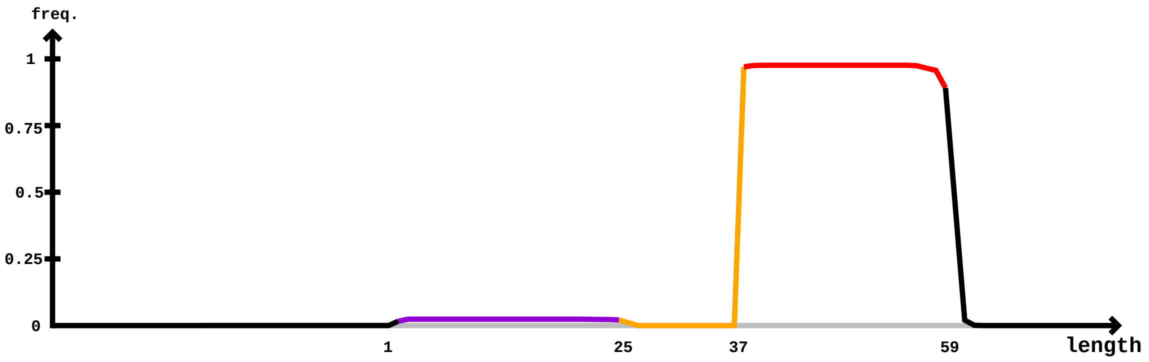

Star Mature

```
5'- cuggaacucucgaacuaacauuccuaggucuuccaucauuuuugugauuggugcaauuugguuaacacaauugcauagucauaaaagugauggguguccaagggacagc  -3'   obs
    cuggaacucucgaacuaacauuccuaggucuuccaucauuuuugugauuggugcaauuugguuaacacaauugcauagucauaaaagugauggguguccaagggacagc         exp
    ....................(((((.((.(.(((((((((((((((((((.(((((((((.....)).))))))))))))))))))))))))))).).)).)))))....    reads     mm     sample
    ...................................ucauuuuugugaCuggugcaauu...................................................    2         1      BRA

    ......................................................................uugcauagucauaaaagug....................    1         0      CA1

    ..................................aucauuuuugugauuggugc.......................................................    3         0      3rd
    ..................................aucauuuuugugauuggugca......................................................    2         0      3rd
    ..................................aucauuuuugugauuggugcaa.....................................................    6         0      3rd
    ..................................aucauuuuugugauuggugcaau....................................................    11        0      3rd
    ..................................aucauuuuugugauuggugcaauu...................................................    63        0      3rd
    ..................................aucauuuuugugauuggugcaauuu..................................................    1         0      3rd
    ...................................ucauuuuugugauuggugca......................................................    1         0      3rd
    ...................................ucauuuuugugauuggugcaU.....................................................    1         1      3rd
    ...................................ucauuuuugugauuggugcaa.....................................................    1         0      3rd
    ...................................ucauuuuugugauuggugcaau....................................................    1         0      3rd
    ...................................ucauuuuugugauuggugcaauu...................................................    24        0      3rd
    ....................................cauuuuugugauuggugcaau....................................................    1         0      3rd
    ....................................cauuuuugugauuggugcaauu...................................................    1         0      3rd
    .....................................................................Uuugcauagucauaaaagugaug.................    2         1      3rd
    .....................................................................Uuugcauagucauaaaagugaugg................    1         1      3rd
    ......................................................................uugcauagucauaaaagu.....................    17        0      3rd
    ......................................................................uugcauagucauaaaagug....................    49        0      3rd
    ......................................................................uugUauagucauaaaaguga...................    1         1      3rd
    ......................................................................uugcauagucauaaaaguga...................    54        0      3rd
    ......................................................................Augcauagucauaaaaguga...................    1         1      3rd
    ......................................................................Augcauagucauaaaagugau..................    1         1      3rd
    ......................................................................uugcauagucauaaaagugaA..................    2         1      3rd
    ......................................................................uugcaGagucauaaaagugau..................    1         1      3rd
    ......................................................................uugcauagucauaaaagugau..................    432       0      3rd
    ......................................................................uugcauaAucauaaaagugau..................    1         1      3rd
    ......................................................................uuUcauagucauaaaagugaug.................    1         1      3rd
    ......................................................................uugcaAagucauaaaagugaug.................    1         1      3rd
    ......................................................................uuAcauagucauaaaagugaug.................    3         1      3rd
    ......................................................................Augcauagucauaaaagugaug.................    6         1      3rd
```

Star Mature

```
cuggaacucucgaacuaacauuccuaggucuuccaucauuuuugugauuggugcaauuugguuaacacaauugcauagucauaaaagugauggguguccaagggacagc
......................................................................uugcauaCucauaaaagugaug.................	2	1	3rd
......................................................................uugcauagucauaaGagugaug.................	1	1	3rd
......................................................................Gugcauagucauaaaagugaug.................	2	1	3rd
......................................................................uugcauaguUauaaaagugaug.................	1	1	3rd
......................................................................uugcauagucauaCaagugaug.................	1	1	3rd
......................................................................uugcauagucauaaaaguAaug.................	1	1	3rd
......................................................................Cugcauagucauaaaagugaug.................	3	1	3rd
......................................................................uCgcauagucauaaaagugaug.................	1	1	3rd
......................................................................uugcauUgucauaaaagugaug.................	2	1	3rd
......................................................................uGgcauagucauaaaagugaug.................	1	1	3rd
......................................................................uugcauagucauaaaagGgaug.................	1	1	3rd
......................................................................uugcauagucauaaaagAgaug.................	1	1	3rd
......................................................................uugcauGgucauaaaagugaug.................	2	1	3rd
......................................................................uugcauagucauaaaagugCug.................	1	1	3rd
......................................................................uugcauagucauaaaaCugaug.................	3	1	3rd
......................................................................uugcauagucauaaCagugaug.................	2	1	3rd
......................................................................uugcauagucauaGaagugaug.................	1	1	3rd
......................................................................uugcauagucauaaaagugaug.................	3061	0	3rd
......................................................................uugUauagucauaaaagugaug.................	3	1	3rd
......................................................................uugcauagCcauaaaagugaug.................	1	1	3rd
......................................................................uugcauagucauaaaagugauU.................	4	1	3rd
......................................................................uugcauagucauaaaagugaGg.................	1	1	3rd
......................................................................uugcauagucauaaaagugUug.................	6	1	3rd
......................................................................uugcauagucauaaaagugauA.................	24	1	3rd
......................................................................uugcauagucauaaaagugGug.................	1	1	3rd
......................................................................uugcaCagucauaaaagugaug.................	1	1	3rd
......................................................................uAgcauagucauaaaagugaug.................	2	1	3rd
......................................................................uugcauagucCuaaaagugaug.................	1	1	3rd
......................................................................uugcaGagucauaaaagugaug.................	5	1	3rd
......................................................................uugcaCagucauaaaagugaugg................	2	1	3rd
......................................................................uugcauagucauaaUagugaugg................	1	1	3rd
......................................................................uugcauagucauaaGagugaugg................	1	1	3rd
......................................................................uugcaGagucauaaaagugaugg................	1	1	3rd
......................................................................uugcauagucauaaaaCugaugg................	2	1	3rd
......................................................................uugcauagucauaaaagAgaugg................	1	1	3rd
......................................................................uugUauagucauaaaagugaugg................	1	1	3rd
......................................................................uugcGuagucauaaaagugaugg................	1	1	3rd
......................................................................uugcauagucauaaaagugaugU................	6	1	3rd
......................................................................uugcauagucauaaaagugaugA................	8	1	3rd
......................................................................uugcauagucauaaaagugCugg................	1	1	3rd
......................................................................uugcauagucauaaaagugaugg................	1961	0	3rd
......................................................................uugcauagucauaaaagugauCg................	1	1	3rd
......................................................................uugcauagucauaaaagugGugg................	1	1	3rd
......................................................................uugcauaguUauaaaagugaugg................	3	1	3rd
......................................................................uugcauagucauaaaagugaugC................	2	1	3rd
......................................................................uugcauagucauaaaGgugaugg................	22	1	3rd
......................................................................uugcauagucauaGaagugaugg................	2	1	3rd
......................................................................uugcauagucauaaaagugUugg................	4	1	3rd
......................................................................Augcauagucauaaaagugaugg................	2	1	3rd
......................................................................uugcauagucauaUaagugaugg................	1	1	3rd
......................................................................uuAcauagucauaaaagugaugg................	1	1	3rd
......................................................................Gugcauagucauaaaagugaugg................	3	1	3rd
......................................................................uugcauagucauaaaagugauggg...............	3	0	3rd
......................................................................uugcauagucauaaaagugauggU...............	55	1	3rd
......................................................................uugcauagucauaaaagugauggA...............	53	1	3rd
......................................................................uugcauagucauaaaagugauggUu..............	1	1	3rd
......................................................................uugcauagucauaaaagugauggAu..............	3	1	3rd
.......................................................................ugcauagucauaaaagugau..................	1	0	3rd
.......................................................................ugcauagucauaaaagugaug.................	10	0	3rd
.......................................................................ugUauagucauaaaagugaug.................	1	1	3rd
.......................................................................ugcauagucauaaaagugaugg................	11	0	3rd
.......................................................................ugcauagucauaaaagugauggC...............	1	1	3rd
.......................................................................ugcauagucauaaaagugauggA...............	1	1	3rd
.......................................................................ugcauagucauaaaagugauggU...............	7	1	3rd
........................................................................gcauagucauaaaagugaug.................	3	0	3rd
........................................................................gcauagucauaaaagugaugg................	1	0	3rd
........................................................................gcauagucauaaaagugauggA...............	1	1	3rd
.........................................................................cauagucauaaaagugaugU................	1	1	3rd

......................................................................uugcauagucauaaaagugaugg................	1	0	HJ1
```

Star Mature

```
cuggaacucucgaacuaacauuccuaggucuuccaucauuuuugugauuggugcaauuugguuaacacaauugcauagucauaaaagugaugggugucaagggacagc

..................................aucauuuuugugauuggugcaau....................................................   1   0   LA1
..................................aucauuuuugugauuggugcaauC...................................................   1   1   LA1
..................................aucauuuuugugauuggugcaauu...................................................   1   0   LA1
...................................ucauuuuugugauuggugcaau....................................................   1   0   LA1
...................................ucauuuuugugauuggugcaauu...................................................   1   0   LA1
......................................................................uugcauagucauaaaagugaug.................   7   0   LA1

..................................aucauuuuugugauuggugcaauu...................................................   2   0   G01
...................................ucauuuuugugauuggugcaauu...................................................   1   0   G01
......................................................................uugcauagucauaaaagugaug.................   24  0   G01
......................................................................uugcauagucauaaaagugaugg................   7   0   G01
......................................................................uugcauagucauaaaGgugaugg................   1   1   G01
......................................................................uugcauagucauaaaagugauggU...............   3   1   G01
.......................................................................ugcauagucauaaaagugaug.................   1   0   G01

..................................aucauuuuugugauuggugcaauu...................................................   1   0   CJ1
......................................................................uugcauagucauaaaagugaug.................   1   0   CJ1

..................................Uucauuuuugugauuggugcaauu...................................................   1   1   LJ1
...................................ucauuuuugugauuggugcaauu...................................................   1   0   LJ1
......................................................................uugcauagucauaaaagugaug.................   1   0   LJ1
......................................................................uugcauagucauaaaagugauggA...............   1   1   LJ1

..................................aucauuuuugugauuggugcaau....................................................   4   0   TA1
..................................aucauuuuugugauuggugcaauG...................................................   1   1   TA1
..................................aucauuuuugugauuggugcaauu...................................................   2   0   TA1
..................................aucauuuuugugauuggugcaauC...................................................   2   1   TA1
...................................ucauuuuugugauuggugc.......................................................   1   0   TA1
...................................ucauuuuugugauuggugca......................................................   1   0   TA1
...................................ucauuuuugugauuggugcaau....................................................   4   0   TA1
...................................ucauuuuugugauuggugcaauu...................................................   33  0   TA1
...................................ucaCuuuugugauuggugcaauu...................................................   2   1   TA1
...................................ucauuuuugugauuggugcaauC...................................................   3   1   TA1

..................................aucauuuuugugauuggugcaauu...................................................   68  0   E01
..................................aucauuuuugugauuggCgcaauu...................................................   1   1   E01
..................................aucauuuuuCugauuggugcaauu...................................................   1   1   E01
...................................ucauuuuugugauuggugca......................................................   1   0   E01
...................................ucauuuuugugauCggugcaauu...................................................   1   1   E01
...................................ucauuuuugugauuggugcaauu...................................................   17  0   E01
.....................................auuuuugugauuggugcaauu...................................................   1   0   E01
......................................................................uugcauagucauaaaagugau..................   12  0   E01
......................................................................uugcauagucauaaaagugaug.................   140 0   E01
......................................................................uugcauagucauaaaagugaugg................   98  0   E01
......................................................................uuAcauagucauaaaagugaugg................   1   1   E01
......................................................................uugcauagucauaaGagugaugg................   1   1   E01
......................................................................uugcauagucauaaaagugaugU................   2   1   E01
......................................................................uugcauagucauaaaagugauggA...............   1   1   E01
......................................................................uugcauagucauaaaagugauggC...............   2   1   E01
......................................................................uugcauagucauaaaagugauggU...............   1   1   E01
......................................................................uugcauagucauaaaagugauggUu..............   2   1   E01
........................................................................gcauagucauaaaagugaug.................   1   0   E01
........................................................................gcauagucauaaaagugaugg................   1   0   E01

..................................aucauuuuugugauuggugc.......................................................   1   0   2nd
..................................aucauuuuugugauuggugca......................................................   1   0   2nd
..................................aucauuuuugugauuggugcaau....................................................   1   0   2nd
..................................aucauuuuugugauuggugcaauu...................................................   51  0   2nd
...................................ucauuuuugugauuggugc.......................................................   1   0   2nd
...................................ucauuuuugugauuggugcaauu...................................................   11  0   2nd
.....................................................................auugcauagucauaaaagugaug.................   1   0   2nd
......................................................................uugcauagucauaaaagu.....................   3   0   2nd
......................................................................uugcauagucauaaaaguA....................   2   1   2nd
......................................................................uugcauagucauaaaagug....................   62  0   2nd
......................................................................uugcauagucauaaaaguga...................   64  0   2nd
......................................................................uugcauagucauaaaagugau..................   415 0   2nd
......................................................................uAgcauagucauaaaagugau..................   1   1   2nd
......................................................................uugcauagucauaaaagugaA..................   2   1   2nd
......................................................................uugcauagucauaGaagugau..................   1   1   2nd
```

Star Mature

```
cuggaacucucgaacuaacauuccuaggucuuccaucauuuuugugauuggugcaauuugguuaacacaauugcauagucauaaaagugaugggguguccaagggacagc
......................................................................uugcauagucauaaaagugauA................. 27 1 2nd
......................................................................uugcauagucaGaaaagugaug................. 1 1 2nd
......................................................................uugcauagucauaaaagugUug................. 5 1 2nd
......................................................................uugcauagucauaaaagAgaug................. 1 1 2nd
......................................................................uAgcauagucauaaaagugaug................. 1 1 2nd
......................................................................uugcauUgucauaaaagugaug................. 6 1 2nd
......................................................................uugcauagucauaUaagugaug................. 1 1 2nd
......................................................................uuUcauagucauaaaagugaug................. 1 1 2nd
......................................................................uugcauagucauUaaagugaug................. 1 1 2nd
......................................................................uugcauagucauaaaagugGug................. 1 1 2nd
......................................................................uugcauagucauaGaagugaug................. 1 1 2nd
......................................................................uugUauagucauaaaagugaug................. 3 1 2nd
......................................................................uGgcauagucauaaaagugaug................. 1 1 2nd
......................................................................uugcauagucauaaaagugaug................. 2884 0 2nd
......................................................................uugcauagucauaaaaAugaug................. 1 1 2nd
......................................................................uugcauagucauaaaUgugaug................. 2 1 2nd
......................................................................uugcaCagucauaaaagugaug................. 1 1 2nd
......................................................................uugcGuagucauaaaagugaug................. 1 1 2nd
......................................................................uugcaAagucauaaaagugaug................. 1 1 2nd
......................................................................uuAcauagucauaaaagugaug................. 3 1 2nd
......................................................................uugcauagucauaaaagCgaug................. 1 1 2nd
......................................................................Augcauagucauaaaagugaug................. 2 1 2nd
......................................................................uugcauagucauaaaagugauC................. 1 1 2nd
......................................................................uugcauagucauaCaagugaug................. 1 1 2nd
......................................................................uugcaGagucauaaaagugaug................. 1 1 2nd
......................................................................uugcauagucauaaaagugCug................. 1 1 2nd
......................................................................uugcauagucauaaGagugaug................. 2 1 2nd
......................................................................uugcauagucauaaaagugauU................. 7 1 2nd
......................................................................uugcauagucauaaaagugaCg................. 2 1 2nd
......................................................................Gugcauagucauaaaagugaug................. 3 1 2nd
......................................................................uugcauagucauaaaagugaugC................ 4 1 2nd
......................................................................uugcauaguUauaaaagugaugg................ 4 1 2nd
......................................................................uugcUuagucauaaaagugaugg................ 1 1 2nd
......................................................................uugcaAagucauaaaagugaugg................ 1 1 2nd
......................................................................uugcauagucauaaaagugaCgg................ 1 1 2nd
......................................................................Gugcauagucauaaaagugaugg................ 2 1 2nd
......................................................................uugcauagucauaaaagugaugg................ 2154 0 2nd
......................................................................uugcauagucauaaaagugaAgg................ 1 1 2nd
......................................................................uugcaCagucauaaaagugaugg................ 1 1 2nd
......................................................................uugUauagucauaaaagugaugg................ 6 1 2nd
......................................................................uugcauagucauaaaGgugaugg................ 18 1 2nd
......................................................................uCgcauagucauaaaagugaugg................ 1 1 2nd
......................................................................uugcauagucauaaaagCgaugg................ 1 1 2nd
......................................................................uugcauagucauaaaagugaugA................ 7 1 2nd
......................................................................uugcauagucauaaaagugGugg................ 2 1 2nd
......................................................................uugcauagucauaaaagugauCg................ 1 1 2nd
......................................................................uugcauagucauaaaagugUugg................ 5 1 2nd
......................................................................uugcauagucauaaCagugaugg................ 3 1 2nd
......................................................................uugcauagucauaaaaguUaugg................ 1 1 2nd
......................................................................uugcauagucauaGaagugaugg................ 1 1 2nd
......................................................................uuAcauagucauaaaagugaugg................ 1 1 2nd
......................................................................Cugcauagucauaaaagugaugg................ 2 1 2nd
......................................................................uugcauagucauaaaagugaugU................ 5 1 2nd
......................................................................uAgcauagucauaaaagugaugg................ 3 1 2nd
......................................................................uugcaGagucauaaaagugaugg................ 3 1 2nd
......................................................................uugcauagucauaaaaAugaugg................ 1 1 2nd
......................................................................uugcauaCucauaaaagugaugg................ 1 1 2nd
......................................................................uugcauagucauaaaagGgaugg................ 1 1 2nd
......................................................................uugcauagucauaaaagugauggA............... 63 1 2nd
......................................................................uugcauagucauaaaagugauggg............... 1 0 2nd
......................................................................uugcauaCucauaaaagugauggg............... 1 1 2nd
......................................................................uugcauagucauaaaagugauggC............... 1 1 2nd
......................................................................uugcauagucauaaaagugauggU............... 56 1 2nd
......................................................................uugcauagucauaaaagugaugggA.............. 3 1 2nd
......................................................................uugcauagucauaaaagugauggUu.............. 1 1 2nd
......................................................................uugcauagucauaaaagugauggAu.............. 3 1 2nd
.......................................................................ugcauagucauaaaagugau.................. 1 0 2nd
.......................................................................ugcauagucauaaaagugaug................. 8 0 2nd
.......................................................................ugcauagucauaaaagugaugg................ 11 0 2nd
.......................................................................ugcauagucauaaaagugauggA............... 3 1 2nd
```

Star / Mature			
cuggaacucucgaacuaacauuccuaggucuuccaucauuuuugugauuggugcaauuugguuaacacaauugcauagucauaaaagugaugggugucaagggacagc			
....Ucauagucauaaaagugaug................	1	1	2nd
....gcauagucauaaaagugaug................	4	0	2nd
....gcauagucauaaaagugaugg...............	2	0	2nd
..................................aucauuuuugugauuggCgcaauu..	1	1	H01
...................................ucauuuuugugauuggugcaauu..	2	0	H01
...................................ucauuuuugugUuuggugcaauu..	1	1	H01
....uugcauagucauaaaagugaug................	2	0	H01
....ugcauagucauaaaagugaug................	1	0	H01
...................................ucauuuuugugauuggugcaauuu...	2	0	LA5
....uugcauagucauaaaagugaug................	1	0	HA1
..................................aucauuuuugugauuggugcaauu..	6	0	TJ1
...................................ucauuuuugugauuggug...	1	0	TJ1
...................................ucauuuuugugauuggugcaauC..	2	1	TJ1
...................................ucauuuuugugauuggugcaauu..	2	0	TJ1
..................................aucauuuuugugauuggugc..	4	0	1st
..................................aucauuuuugugUuuggugcaau...	1	1	1st
..................................aucauuuuugugauuggugcaau...	3	0	1st
..................................aucauuuuugugaCuggugcaauu..	1	1	1st
..................................aucauuuuugugauuggugcaauu..	33	0	1st
...................................ucauuuuugugauuggugc..	1	0	1st
...................................ucauuuuugugauuggugca...	1	0	1st
...................................ucauuuuugugauuggugcaauu..	9	0	1st
....Uuugcauagucauaaaagugau.................	1	1	1st
....Guugcauagucauaaaagugaug................	1	1	1st
....Uuugcauagucauaaaagugaug................	1	1	1st
....uugcauagucauaaaagu....................	10	0	1st
....uugcauagucauaGaagug...................	1	1	1st
....uugcauagucauaaaagug...................	52	0	1st
....uugcauagucauaaaaguA...................	4	1	1st
....uugcauagucauaaaagugU..................	2	1	1st
....uugcauagucauaaaaguga..................	36	0	1st
....uugcauagucauaaaagugaA.................	1	1	1st
....uugcauagucauaaaagugau.................	360	0	1st
....uugcauagucauaaGagugau.................	1	1	1st
....uugcauagucauaaUagugau.................	1	1	1st
....uugcauagucauaaaagugUu.................	1	1	1st
....uugcauCgucauaaaagugaug................	1	1	1st
....uugcauagucauaaaagugaug................	2690	0	1st
....uugcGuagucauaaaagugaug................	1	1	1st
....uugcauagucauaaGagugaug................	2	1	1st
....uugcauagucauaaaaUugaug................	1	1	1st
....uugcauUgucauaaaagugaug................	2	1	1st
....uugcauagucauaaaaAugaug................	1	1	1st
....uugUauagucauaaaagugaug................	2	1	1st
....Augcauagucauaaaagugaug................	3	1	1st
....uuAcauagucauaaaagugaug................	1	1	1st
....uugcauagucauaaaagugauC................	1	1	1st
....uugcauagucauaaaaguAaug................	1	1	1st
....uugcauagucauaGaagugaug................	1	1	1st
....uugcauagucGuaaaagugaug................	1	1	1st
....uugcauagucauaaaagugUug................	3	1	1st
....uugcaCagucauaaaagugaug................	1	1	1st
....uugcauagucauaCaagugaug................	1	1	1st
....uugcauagucUuaaaagugaug................	1	1	1st
....uuCcauagucauaaaagugaug................	1	1	1st
....Cugcauagucauaaaagugaug................	5	1	1st
....uugcauagucauaaaaguCaug................	1	1	1st
....Gugcauagucauaaaagugaug................	2	1	1st
....uugcauagucauaaaagugaCg................	1	1	1st
....uugcauagucauaaaagugauU................	1	1	1st
....uugcauagucauaaaagugauA................	30	1	1st
....uugcauagucauaaaagugCug................	1	1	1st
....Cugcauagucauaaaagugaugg...............	1	1	1st
....uugcauagucauaaCagugaugg...............	2	1	1st
....Augcauagucauaaaagugaugg...............	2	1	1st

Star Mature

```
cuggaacucucgaacuaacauuccuaggucuuccaucauuuuugugauuggugcaauuugguuaacacaauugcauagucauaaaagugauggguguccaagggacagc
......................................................................uugcauagucauaaaagugaugg................ 2388 0 1st
......................................................................uugcauagucauGaaagugaugg................ 1 1 1st
......................................................................Gugcauagucauaaaagugaugg................ 3 1 1st
......................................................................uugcaCagucauaaaagugaugg................ 1 1 1st
......................................................................uugcauagCcauaaaagugaugg................ 1 1 1st
......................................................................uugcauagucauaaaagCgaugg................ 1 1 1st
......................................................................uugcauagucauaaaagugaugU................ 9 1 1st
......................................................................uAgcauagucauaaaagugaugg................ 1 1 1st
......................................................................uugcauagucauaaGagugaugg................ 1 1 1st
......................................................................uugcauagucauaaaagugaugC................ 3 1 1st
......................................................................uugcauagucauaaaagugaAgg................ 15 1 1st
......................................................................uugcauaguUauaaaagugaugg................ 3 1 1st
......................................................................uugcauUgucauaaaagugaugg................ 1 1 1st
......................................................................uugcauagucauaaaGgugaugg................ 12 1 1st
......................................................................uugcauagucauaaaaguUaugg................ 1 1 1st
......................................................................uugcauagucauUaaagugaugg................ 1 1 1st
......................................................................uugcauagucauaaaagugGugg................ 1 1 1st
......................................................................uuAcauagucauaaaagugaugg................ 1 1 1st
......................................................................uugcauagucauaaaagugUugg................ 4 1 1st
......................................................................uugcauagucauaGaagugaugg................ 3 1 1st
......................................................................uugcauagucauaaaagugaugA................ 8 1 1st
......................................................................uugcauagucauaaaagugauggU............... 50 1 1st
......................................................................uugcauagucauaaaagugaugAg............... 1 1 1st
......................................................................uugcauagucauaaaagugauggA............... 46 1 1st
......................................................................uugcauagucauaaaagugauggUu.............. 1 1 1st
......................................................................uugcauagucauaaaagugauggAu.............. 3 1 1st
.......................................................................ugcauagucauaaaagug.................... 1 0 1st
.......................................................................ugcauagucauaaaaguga................... 1 0 1st
.......................................................................uUcauagucauaaaagugaug................. 1 1 1st
.......................................................................ugcauagucauaaaagugaug................. 14 0 1st
.......................................................................ugcauagucauaaaGgugaugg................ 2 1 1st
.......................................................................ugcauagucauaaaagugaugg................ 16 0 1st
.......................................................................ugcauagucauaaaagugauggU............... 3 1 1st
........................................................................gcauagucauaaaagugaugg................ 1 0 1st

..............cuaacauuccuaggucuucc........................................................................... 1 0 EGG
..................................aucauuuuugugauuggugcaau.................................................... 1 0 EGG
..................................aucauuuuugugauuggugcaauu................................................... 31 0 EGG
...................................ucauuuuugugauuggugcaau.................................................... 1 0 EGG
...................................ucauuuuugugauuggugcaauu................................................... 8 0 EGG
...................................ucauuuuugugauuggugcaauuu.................................................. 1 0 EGG
......................................................................uugcauagucauaaaaguga................... 1 0 EGG
......................................................................uugcauagucauaaaagugau.................. 10 0 EGG
......................................................................uugcauagucauaaaagugauU................. 1 1 EGG
......................................................................uugcaGagucauaaaagugaug................. 1 1 EGG
......................................................................uugcauagucauaaaagugaug................. 170 0 EGG
......................................................................uugcauagucauaaaaguUaug................. 1 1 EGG
......................................................................Augcauagucauaaaagugaugg................ 1 1 EGG
......................................................................uugcauagucauaaaagugaugA................ 1 1 EGG
......................................................................uugcauagucauaaaagugaugg................ 207 0 EGG
......................................................................uugcauagucauaaaagugauCg................ 1 1 EGG
......................................................................uugcauagucauaaGagugaugg................ 1 1 EGG
......................................................................uuAcauagucauaaaagugaugg................ 1 1 EGG
......................................................................uugcauagucauaGaagugaugg................ 1 1 EGG
......................................................................uugcauagucauaaaGgugaugg................ 1 1 EGG
......................................................................uugcauagucauaaaagugauggU............... 3 1 EGG
......................................................................uugcauagucauaaaagugauggA............... 7 1 EGG
.......................................................................ugcauagucauaaaagugaug................. 1 0 EGG
.......................................................................ugcauagucauaaaagugaugg................ 2 0 EGG
```

Provisional ID	:	Scaffold_11093_2890
Score total	:	1761693.4
Score for star read(s)	:	3.9
Score for read counts	:	1761683
Score for mfe	:	1.9
Score for randfold	:	1.6
Score for cons. seed	:	3
Total read count	:	3455477
Mature read count	:	3400686
Loop read count	:	0
Star read count	:	54791

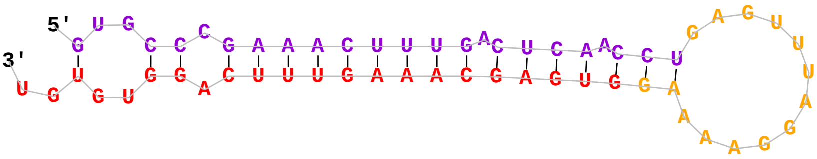

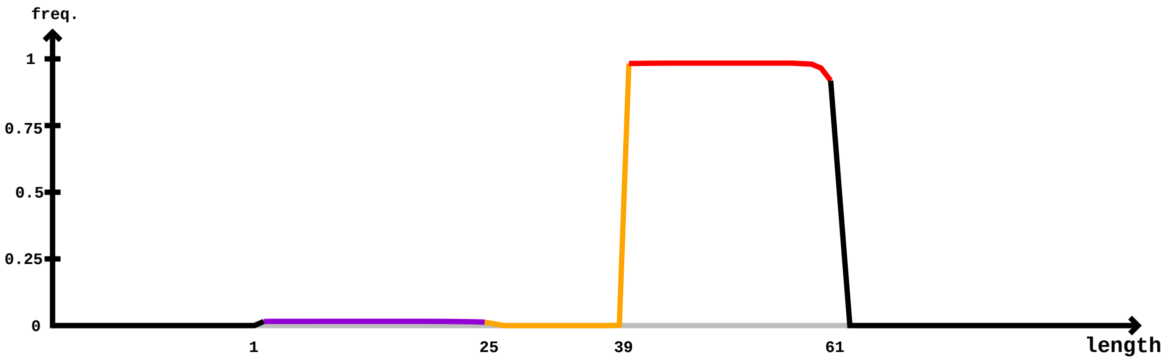


```
                            Star                                        Mature
5'- ugagagaaugucggccuugugugcccgaaacuuugacucaaccugaguuuaggaaaaggugagcaaaguuucaggugugucggguugacugaaaaaacugguuuaccagga  -3'  obs
    ugagagaaugucggccuugugugcccgaaacuuugacucaaccugaguuuaggaaaaggugagcaaaguuucaggugugucggguugacugaaaaaacugguuuaccagga       exp
    .........((((((((.(..((((.(((((((((.((((.(((............))))))))))))))))).)))).)..).))))))))........((((....))))..   reads   mm   sample
    ....................gugcccgaaacuuugacuc........................................................................   1       0    EGG
    ....................gugcccgaaacuuugacucaac.....................................................................   2       0    EGG
    ....................gugcccgaaacuuugacucaacc....................................................................   11      0    EGG
    ....................gugcccgaaacuuugacucaaccu...................................................................   107     0    EGG
    ....................gugcccgaaacuuugacucaaccuA..................................................................   1       1    EGG
    ....................gugcccgaaacuuugacucaaccuU..................................................................   2       1    EGG
    .....................ugcccgaaacuuugacucaa......................................................................   1       0    EGG
    .....................ugcccgaaacuuugacucaacc....................................................................   1       0    EGG
    .....................ugcccgaaacuuugacucaaccu...................................................................   14      0    EGG
    .....................................................aaaGggugagcaaaguuucaggugugu...............................   1       1    EGG
    ......................................................aaGggugagcaaaguuucaggugug................................   1       1    EGG
    ......................................................aaGggugagcaaaguuucaggugugu...............................   4       1    EGG
    .......................................................aGggugagcaaaguuucaggugugu...............................   5       1    EGG
    ........................................................Uggugagcaaaguuucaggugugu...............................   1       1    EGG
    ........................................................aggugagcaaaguuucaggugugu...............................   1       0    EGG
    .........................................................ggugagcaaaguuucaggugu.................................   9       0    EGG
    .........................................................Ugugagcaaaguuucaggugu.................................   1       1    EGG
    .........................................................ggugagcaaaguuucaggugug................................   75      0    EGG
    .........................................................ggugagcaaaguuucagguguU................................   4       1    EGG
    .........................................................ggugagcaaaguuucaggugugu...............................   70      0    EGG
    .........................................................ggugagcaaaguuucaggugugG...............................   1       1    EGG
    .........................................................Ugugagcaaaguuucaggugugu...............................   73      1    EGG
    .........................................................ggugagcaaaguuucaggugugA...............................   1       1    EGG
    .........................................................Cgugagcaaaguuucaggugugu...............................   1       1    EGG
    ..........................................................gugagcaaaguuucaggu...................................   54      0    EGG
    ..........................................................gugGgcaaaguuucaggug..................................   1       1    EGG
    ..........................................................gugagcaaaguuucagguU..................................   2       1    EGG
    ..........................................................gugagcaaaguCucaggug..................................   1       1    EGG
    ..........................................................gugUgcaaaguuucaggug..................................   1       1    EGG
    ..........................................................gugagcaaaguuucaggug..................................   102     0    EGG
    ..........................................................gugagcaaaguuucGggugu.................................   2       1    EGG
    ..........................................................gugaAcaaaguuucaggugu.................................   2       1    EGG
    ..........................................................gugagUaaaguuucaggugu.................................   1       1    EGG
```

```
                    Star                                      Mature
ugagagaaugucggccuugugugcccgaaacuugacucaaccugaguuuaggaaaaggugagcaaaguuucaggugugucggguugacugaaaaaacugguuuaccagga
.........................................................gugagcaaaguuucaggugu.................................    892     0     EGG
.........................................................gugagAaaaguuucaggugu.................................    1       1     EGG
.........................................................gugagcaaaguuucaggugC.................................    2       1     EGG
.........................................................gugagcaaaguuucGggugug................................    2       1     EGG
.........................................................gugagcaaaguAucaggugug................................    1       1     EGG
.........................................................gugagcaaaguuucagguguA................................    7       1     EGG
.........................................................gugagcaaaguuuUaggugug................................    1       1     EGG
.........................................................gugGgcaaaguuucaggugug................................    2       1     EGG
.........................................................gugagcaaaguuucagguguU................................    216     1     EGG
.........................................................gugagcaaaUuuucaggugug................................    1       1     EGG
.........................................................gugagcaaaguuucagguguC................................    1       1     EGG
.........................................................gugagcaaaguuucagAugug................................    3       1     EGG
.........................................................gugagcaaaguuucaggGgug................................    1       1     EGG
.........................................................gugagcaaaguuucagCugug................................    1       1     EGG
.........................................................gugagcaaaguuucagguUug................................    1       1     EGG
.........................................................gugCgcaaaguuucaggugug................................    4       1     EGG
.........................................................gugaAcaaaguuucaggugug................................    1       1     EGG
.........................................................gugagcaaagGuucaggugug................................    2       1     EGG
.........................................................gugagGaaaguuucaggugug................................    1       1     EGG
.........................................................gugagcaaaguuucagguAug................................    1       1     EGG
.........................................................gugagcaaaguuucCggugug................................    1       1     EGG
.........................................................gugagcaaaguuucUggugug................................    1       1     EGG
.........................................................gugagcaaaguuucaggugAg................................    1       1     EGG
.........................................................gugUgcaaaguuucaggugug................................    1       1     EGG
.........................................................gugagcaaaguuucaggugug................................    6868    0     EGG
.........................................................gugagAaaaguuucaggugug................................    1       1     EGG
.........................................................gCgagcaaaguuucaggugug................................    2       1     EGG
.........................................................gugagcaaagCuucaggugug................................    3       1     EGG
.........................................................gugagcaaGguuucaggugug................................    1       1     EGG
.........................................................gugagcaaaguuucaggugGg................................    1       1     EGG
.........................................................gugagUaaaguuucaggugug................................    3       1     EGG
.........................................................gugagcaaaCuuucaggugug................................    1       1     EGG
.........................................................gugagcaaaguuucagUugug................................    2       1     EGG
.........................................................gugaUcaaaguuucaggugugu...............................    2       1     EGG
.........................................................gugagcaaUguuucaggugugu...............................    18      1     EGG
.........................................................gugagcaaaguuucaggugugG...............................    48      1     EGG
.........................................................gugagUaaaguuucaggugugu...............................    71      1     EGG
.........................................................gugagcaaaguuucagguUugu...............................    12      1     EGG
.........................................................gugagcaaaguGucaggugugu...............................    14      1     EGG
.........................................................guUagcaaaguuucaggugugu...............................    2       1     EGG
.........................................................gugagcaaaguuucaggugGgu...............................    3       1     EGG
.........................................................gugagcaaaguuuUaggugugu...............................    50      1     EGG
.........................................................gAgagcaaaguuucaggugugu...............................    1       1     EGG
.........................................................gugagcaaaguuucaggGgugu...............................    9       1     EGG
.........................................................gugagcaaaguuAcaggugugu...............................    1       1     EGG
.........................................................gugagcaaGguuucaggugugu...............................    9       1     EGG
.........................................................gugagcaGaguuucaggugugu...............................    11      1     EGG
.........................................................gugagcaaagCuucaggugugu...............................    27      1     EGG
.........................................................gugagcaaaguuuAaggugugu...............................    3       1     EGG
.........................................................gugagcaaagGuucaggugugu...............................    15      1     EGG
.........................................................gugagcaaaUuuucaggugugu...............................    8       1     EGG
.........................................................gugagcaaaguuucaggugCgu...............................    12      1     EGG
.........................................................gugagcGaaguuucaggugugu...............................    7       1     EGG
.........................................................gugagcaaaguuucagUugugu...............................    20      1     EGG
.........................................................gugagcaaaguuucaggguCu...............................    6       1     EGG
.........................................................gugagcaaaguuucaggAgugu...............................    3       1     EGG
.........................................................gugagcaaaguuucagguguUu...............................    467     1     EGG
.........................................................gugagcaUaguuucaggugugu...............................    4       1     EGG
.........................................................gugagcaaaguuCcaggugugu...............................    8       1     EGG
.........................................................gGgagcaaaguuucaggugugu...............................    15      1     EGG
.........................................................gugCgcaaaguuucaggugugu...............................    55      1     EGG
.........................................................gugagcaaaguuucaCgugugu...............................    11      1     EGG
.........................................................gugagcaaCguuucaggugugu...............................    4       1     EGG
.........................................................gugagcaaaCuuucaggugugu...............................    14      1     EGG
.........................................................gugagcaaaguCucaggugugu...............................    11      1     EGG
.........................................................gugagcaaaguAucaggugugu...............................    4       1     EGG
.........................................................gugagcaaagAuucaggugugu...............................    6       1     EGG
.........................................................gugagcaaaguuucagguguAu...............................    9       1     EGG
.........................................................gugaAcaaaguuucaggugugu...............................    45      1     EGG
.........................................................gugUgcaaaguuucaggugugu...............................    15      1     EGG
```

```
                    Star                                  Mature
ugagagaaugucggccuugugugcccgaaacuuugacucaaccugaguuuaggaaaaggugagcaaaguuucaggugugucggguugacugaaaaaacugguuuaccagga
..........................................................gugagcaaaAuuucaggugugu............................... 4       1  EGG
..........................................................gugagcaaaguuucaAgugugu............................... 3       1  EGG
..........................................................gugagcaaaguuuGaggugugu............................... 6       1  EGG
..........................................................Cugagcaaaguuucaggugugu............................... 1       1  EGG
..........................................................gugagcaaaguuucaggugugA............................... 988     1  EGG
..........................................................gugagcaaaguuucaggCgugu............................... 18      1  EGG
..........................................................gugagcaaaguuucUggugugu............................... 40      1  EGG
..........................................................gugagcaCaguuucaggugugu............................... 5       1  EGG
..........................................................gugagcaaaguuucagAugugu............................... 96      1  EGG
..........................................................gCgagcaaaguuucaggugugu............................... 11      1  EGG
..........................................................Augagcaaaguuucaggugugu............................... 6       1  EGG
..........................................................gugagcaaaguuucagguCugu............................... 4       1  EGG
..........................................................gugagcaaaguuucaUgugugu............................... 9       1  EGG
..........................................................gugagcaaaguuuCggugugu................................ 11      1  EGG
..........................................................gugagcaaaguuucaggugugu............................... 168800  0  EGG
..........................................................gugagcaaaguuuCGgugugu................................ 101     1  EGG
..........................................................gugGgcaaaguuucaggugugu............................... 17      1  EGG
..........................................................gugaCcaaaguuucaggugugu............................... 3       1  EGG
..........................................................gugagcaaaguuucagguAugu............................... 14      1  EGG
..........................................................gugagAaaaguuucaggugugu............................... 21      1  EGG
..........................................................Uugagcaaaguuucaggugugu............................... 14      1  EGG
..........................................................gugagGaaaguuucaggugugu............................... 25      1  EGG
..........................................................gugagcaaaguuucagCugugu............................... 11      1  EGG
..........................................................guAagcaaaguuucaggugugu............................... 2       1  EGG
..........................................................guCagcaaaguuucaggugugu............................... 9       1  EGG
..........................................................gugagcaaaguuucaggugAgu............................... 3       1  EGG
..........................................................gugagcaaaguuucaggugugc.............................. 21      0  EGG
..........................................................gugagcaaaguuucaggugugugG.............................. 25      1  EGG
..........................................................gugagcaaaguuucaggugugCc.............................. 1       1  EGG
..........................................................gugagcaaaguuucaggugugucU............................. 2       1  EGG
..........................................................gugagcaaaguuucaggugugugGgg............................ 2       1  EGG
..........................................................gugagcaaaguuucaggugugugGggg........................... 2       1  EGG
..........................................................gugagcaaaguuucaggugugugGgggu.......................... 1       1  EGG
...........................................................ugagcaaaguuucaggugugu............................... 17      0  EGG
............................................................gagcaaaguuucaggugugu............................... 18      0  EGG
.............................................................agcaaaguuucaggugugu............................... 1       0  EGG
..............................................................gcaaaguuucaggugugu............................... 1       0  EGG

....................gugcccgaaacuuugacuc........................................................................ 1       0  1st
....................gugcccgaaacuuugacuca....................................................................... 9       0  1st
....................gugcccgaaacuuugacucaa...................................................................... 1       0  1st
....................gugcccgaaacuuugacucaac..................................................................... 7       0  1st
....................gugcccgaaacuuugUcucaacc.................................................................... 2       1  1st
....................gugcccgaaacuuugacucaacc.................................................................... 14      0  1st
....................gugcccgUaacuuugacucaaccu................................................................... 1       1  1st
....................gugcccgaaacuuugacucaaccA................................................................... 1       1  1st
....................gugcccgaaacuuugacucaaccu................................................................... 96      0  1st
....................gugcccgaaacuuugacucaaccuU.................................................................. 2       1  1st
.....................ugcccgaaacuuugacuca....................................................................... 1       0  1st
.....................ugcccgaaacuuugacucaacc.................................................................... 3       0  1st
.....................ugcccgaaacuuugacucaaccu................................................................... 10      0  1st
.....................ugcccgaaacuuugacucaaccuU.................................................................. 1       1  1st
........................ccgaaacuuugacucaaccu................................................................... 2       0  1st
.....................................................aaaGggugagcaaaguuucaggugug................................ 2       1  1st
.....................................................aaaGggugagcaaaguuucaggugugu............................... 3       1  1st
......................................................aaGggugagcaaaguuucaggugu................................. 1       1  1st
......................................................aaGggugagcaaaguuucaggugugu............................... 5       1  1st
.......................................................aGggugagcaaaguuucaggugug................................ 5       1  1st
.......................................................aaCgugagcaaaguuucaggugugu............................... 1       1  1st
.......................................................aGggugagcaaaguuucaggugugu............................... 11      1  1st
.......................................................aaggugagcaaaguuucaggugugu............................... 1       0  1st
........................................................Gggugagcaaaguuucaggu................................... 1       1  1st
........................................................aggugagcaaaguuucaggugu................................. 1       0  1st
........................................................aggugagcaaaguuucagguguU................................ 1       1  1st
........................................................Gggugagcaaaguuucaggugugu............................... 2       1  1st
........................................................aggugagcaaaguuucaggugugu............................... 1       0  1st
.........................................................Ugugagcaaaguuucaggugu................................. 5       1  1st
.........................................................ggugaAcaaaguuucaggugu................................. 1       1  1st
.........................................................ggugagcaaaguuucaggugu................................. 40      0  1st
.........................................................Ugugagcaaaguuucaggugug................................ 11      1  1st
```

Star Mature

ugagagaaugucggccuugugugcccgaaacuuugacucaaccugaguuuaggaaaaggugagcaaaguuucaggugugucggguugacugaaaaaacugguuuaccagga			
....ggugagcaaaguuucaggugug................................	58	0	1st
....ggugagcaaaguuucagguguU................................	5	1	1st
....ggugagcaaaguuucagguguC................................	1	1	1st
....Agugagcaaaguuucaggugugu...............................	6	1	1st
....ggugagcaaaguuCcaggugugu...............................	1	1	1st
....Cgugagcaaaguuucaggugugu...............................	5	1	1st
....ggugagcaaaguuucaggugugA...............................	4	1	1st
....ggugagcaaaguuucaggugugu...............................	176	0	1st
....Ugugagcaaaguuucaggugugu...............................	283	1	1st
....ggugagcaaaguuucagguguUu...............................	3	1	1st
....ggugagcaaaAuuucaggugugu...............................	1	1	1st
....ggGgagcaaaguuucaggugugu...............................	2	1	1st
....gugagcaaaguuucaggC...................................	4	1	1st
....Cugagcaaaguuucaggu...................................	1	1	1st
....gugagcUaaguuucaggu...................................	1	1	1st
....gugagcaaaguuucaggA...................................	6	1	1st
....gugaCcaaaguuucaggu...................................	1	1	1st
....gugagcaaaCuuucaggu...................................	1	1	1st
....gugagcaaaguuucCggu...................................	1	1	1st
....gugagcaaaguuucaggu...................................	780	0	1st
....gugaAcaaaguuucaggug..................................	1	1	1st
....gugagcaaaguuucaUgug..................................	1	1	1st
....gugagcaaaguuucagUug..................................	1	1	1st
....gugagcaaaguuucaggug..................................	865	0	1st
....gugagcaGaguuucaggug..................................	1	1	1st
....gugagcaaaguuucagguA..................................	1	1	1st
....gugagcaaaguuucGggug..................................	2	1	1st
....gugagcaaaguuucaggCg..................................	4	1	1st
....gugagcaaaguuucagguU..................................	6	1	1st
....gugagcUaaguuucaggug..................................	1	1	1st
....gugagcaaaguuucaggGg..................................	3	1	1st
....gugagcaaaguuucagguC..................................	5	1	1st
....gugagcaaaguuucaAgug..................................	1	1	1st
....gugagcaaaguuuUaggug..................................	2	1	1st
....gugagcGaaguuucaggugu.................................	2	1	1st
....gugagcUaaguuucaggugu.................................	16	1	1st
....gugagcaaaguuucaUgugu.................................	5	1	1st
....gugagcaaaguuucaggGgu.................................	25	1	1st
....gugaAcaaaguuucaggugu.................................	3	1	1st
....gugagcaaagGuucaggugu.................................	6	1	1st
....guAagcaaaguuucaggugu.................................	6	1	1st
....gugagcaaaguCucaggugu.................................	2	1	1st
....gugagcaaaguGucaggugu.................................	1	1	1st
....gugCgcaaaguuucaggugu.................................	1	1	1st
....gugagcaaaguuuUaggugu.................................	3	1	1st
....gugagUaaaguuucaggugu.................................	6	1	1st
....gugagcaaaguuuGaggugu.................................	2	1	1st
....gugagcaaaguuucagUugu.................................	6	1	1st
....gugagcaUaguuucaggugu.................................	2	1	1st
....gugagcaaaguuucaggAgu.................................	1	1	1st
....gCgagcaaaguuucaggugu.................................	3	1	1st
....Uugagcaaaguuucaggugu.................................	6	1	1st
....gGgagcaaaguuucaggugu.................................	2	1	1st
....gugagcaaagAuucaggugu.................................	4	1	1st
....gugGgcaaaguuucaggugu.................................	6	1	1st
....gugagcaaaguuucagCugu.................................	6	1	1st
....gugagcCaaguuucaggugu.................................	1	1	1st
....gugagcaaaguuucagguUu.................................	2	1	1st
....gugagcaaaguuucGggugu.................................	7	1	1st
....gugagcaaaguuucagAugu.................................	2	1	1st
....gugagcaaaguuucaggugu.................................	15861	0	1st
....gugagcaaagCuucaggugu.................................	7	1	1st
....gugagcaaaguuucaggugG.................................	9	1	1st
....guCagcaaaguuucaggugu.................................	4	1	1st
....gugagcaaaguuucaCgugu.................................	7	1	1st
....gugagcaaaguuucagguCu.................................	3	1	1st
....gAgagcaaaguuucaggugu.................................	6	1	1st
....gugaCcaaaguuucaggugu.................................	2	1	1st
....gugagAaaaguuucaggugu.................................	1	1	1st
....gugagcaaaguuucaggCgu.................................	1	1	1st

Star   Mature

ugagagaaugucggccuugugugcccgaaacuuugacucaaccugaguuuaggaaaaggugagcaaaguuucaggugugucggguugacugaaaaaacugguuuaccagga			
....gugagcaaaguuucUggugu.................................	10	1	1st
....gugagcaaaguuCcaggugu.................................	2	1	1st
....gugagcaaGguuucaggugu.................................	2	1	1st
....gugagcaaaCuuucaggugu.................................	1	1	1st
....gugagcaaCguuucaggugu.................................	2	1	1st
....gugagcaaaguuucaggugC.................................	19	1	1st
....gugagcaaaguuucaAgugu.................................	1	1	1st
....gugUgcaaaguuucaggugu.................................	3	1	1st
....gugagcaaaguuucagUugug................................	4	1	1st
....Augagcaaaguuucaggugug................................	2	1	1st
....guCagcaaaguuucaggugug................................	6	1	1st
....gugagcaaaCuuucaggugug................................	1	1	1st
....gugagcaaGguuucaggugug................................	2	1	1st
....gugagCaaaguuucaggugug................................	5	1	1st
....gugagcaaaguuucagguAug................................	3	1	1st
....gugUgcaaaguuucaggugug................................	9	1	1st
....gugagcaaaguuGcaggugug................................	2	1	1st
....gugagcaaaguuucaggugGg................................	3	1	1st
....gugagcaaaguuucaggugug................................	31001	0	1st
....gugagcaaaguCucaggugug................................	5	1	1st
....gugagcaaaguuucagCugug................................	9	1	1st
....gugagcaaagAuucaggugug................................	7	1	1st
....gCgagcaaaguuucaggugug................................	3	1	1st
....gugagcUaaguuucaggugug................................	28	1	1st
....gugagcaaaguuucagguguU................................	2076	1	1st
....gugaCcaaaguuucaggugug................................	1	1	1st
....gugagcaaaguuucagguguA................................	44	1	1st
....gugagcaaaUuuucaggugug................................	1	1	1st
....gGgagcaaaguuucaggugug................................	2	1	1st
....gugagcaaaguuucagguCug................................	4	1	1st
....gugagcaaaguuucaggugAg................................	3	1	1st
....gugagcaaaguuucGggugug................................	14	1	1st
....gugGgcaaaguuucaggugug................................	11	1	1st
....gugagAaaaguuucaggugug................................	2	1	1st
....gugagcaGaguuucaggugug................................	5	1	1st
....gugagcaaaguuucagguguC................................	10	1	1st
....gugaAcaaaguuucaggugug................................	9	1	1st
....Cugagcaaaguuucaggugug................................	1	1	1st
....gugCgcaaaguuucaggugug................................	3	1	1st
....gugagcaaaguuGaggugug.................................	5	1	1st
....gugagcaaaguuucaAgugug................................	12	1	1st
....gugagcaaaguAucaggugug................................	2	1	1st
....gugagcaaaguuucaCgugug................................	9	1	1st
....gugagcaaaguuCcaggugug................................	3	1	1st
....gugagcaaaguuucagAugug................................	11	1	1st
....gugaUcaaaguuucaggugug................................	1	1	1st
....Uugagcaaaguuucaggugug................................	3	1	1st
....gAgagcaaaguuucaggugug................................	17	1	1st
....gugagcaaaguuucaggugCg................................	20	1	1st
....guUagcaaaguuucaggugug................................	1	1	1st
....gugagUaaaguuucaggugug................................	19	1	1st
....gugagcaaaguuucaggCgug................................	8	1	1st
....guAagcaaaguuucaggugug................................	10	1	1st
....gugagcaaaguuuUaggugug................................	15	1	1st
....gugagcaaagCuucaggugug................................	9	1	1st
....gugagcaaUguuucaggugug................................	1	1	1st
....gugagcaaaAuucaggugug.................................	1	1	1st
....gugagcaaaguuucUggugug................................	18	1	1st
....gugagcaaagGuucaggugug................................	3	1	1st
....gugagcaaaguuucaggGgug................................	5	1	1st
....gugagcaaaguuucaUgugug................................	9	1	1st
....gugaUcaaaguuucaggugugu...............................	34	1	1st
....gugagcGaaguuucaggugugu...............................	80	1	1st
....gugagcaaaguuCcaggugugu...............................	48	1	1st
....gugagcaaaguuucagguguCu...............................	112	1	1st
....gugagcaaaguuucagCugugu...............................	281	1	1st
....gugagcaaaguuucaggAgugu...............................	25	1	1st
....gGgagcaaaguuucaggugugu...............................	96	1	1st
....gugagcaaaguuucaggugugG...............................	845	1	1st
....gugagcaaaguuucagguguAu...............................	69	1	1st

```
                    Star                                      Mature
ugagagaaugucggccuugugugcccgaaacuuugacucaaccugaguuuaggaaaaggugagcaaaguuucaggugugucggguugacugaaaaaacugguuuaccagga
..........................................................gugagcaaaguuucaggugugA...............................    4664     1     1st
..........................................................gugagcUaaguuucaggugugu...............................    1061     1     1st
..........................................................gugagcaaaguCucaggugugu...............................    81       1     1st
..........................................................gugagcaaaguuucaggGgugu...............................    109      1     1st
..........................................................gugagcaaaguuucCggugugu...............................    19       1     1st
..........................................................gugagcaaaguGucaggugugu...............................    61       1     1st
..........................................................gugagcaaaguuucUggugugu...............................    400      1     1st
..........................................................gugCgcaaaguuucaggugugu...............................    175      1     1st
..........................................................gugagcaaaCuuucaggugugu...............................    76       1     1st
..........................................................gugagcaUaguuucaggugugu...............................    80       1     1st
..........................................................gugagcaaaguuucaggugugu...............................    910254   0     1st
..........................................................gugagcaaUguuucaggugugu...............................    47       1     1st
..........................................................guCagcaaaguuucaggugugu...............................    150      1     1st
..........................................................gugagcaaaguuucagguAugu...............................    75       1     1st
..........................................................Augagcaaaguuucaggugugu...............................    55       1     1st
..........................................................gugagcaaagCuucaggugugu...............................    143      1     1st
..........................................................gugagcaaaguuucagUugugu...............................    197      1     1st
..........................................................gugagcaaagAuucaggugugu...............................    162      1     1st
..........................................................gugagcaaaguuucaggugGgu...............................    127      1     1st
..........................................................gugagcaaaguuucaggugCgu...............................    92       1     1st
..........................................................gugagcaaaguuucaggugAgu...............................    79       1     1st
..........................................................gugaCcaaaguuucaggugugu...............................    85       1     1st
..........................................................gugagcaaaguuucagguUugu...............................    85       1     1st
..........................................................Uugagcaaaguuucaggugugu...............................    97       1     1st
..........................................................gugagcaaaguuucaCgugugu...............................    232      1     1st
..........................................................gugagUaaaguuucaggugugu...............................    449      1     1st
..........................................................gugUgcaaaguuucaggugugu...............................    262      1     1st
..........................................................gCgagcaaaguuucaggugugu...............................    72       1     1st
..........................................................gugagcaGaguuucaggugugu...............................    55       1     1st
..........................................................gugagcaaaUuuucaggugugu...............................    17       1     1st
..........................................................gugagcaaaguuucGgugugu...............................    413      1     1st
..........................................................gugagcaaaguuAcaggugugu...............................    3        1     1st
..........................................................gugagGaaaguuucaggugugu...............................    40       1     1st
..........................................................gugagcaaaguuucaAgugugu...............................    289      1     1st
..........................................................gugagcaaGguuucaggugugu...............................    60       1     1st
..........................................................gugaAcaaaguuucaggugugu...............................    219      1     1st
..........................................................gugagcaaaAuuucaggugugu...............................    33       1     1st
..........................................................gugagcaaaguuuAaggugugu...............................    32       1     1st
..........................................................gugagcaaCguuucaggugugu...............................    21       1     1st
..........................................................gugagcaaaguuGcaggugugu...............................    15       1     1st
..........................................................gugagcaaaguuucaggCgugu...............................    91       1     1st
..........................................................gugagcaaaguAucaggugugu...............................    52       1     1st
..........................................................gugGgcaaaguuucaggugugu...............................    411      1     1st
..........................................................gugagcaCaguuucaggugugu...............................    35       1     1st
..........................................................Cugagcaaaguuucaggugugu...............................    39       1     1st
..........................................................gugagcaaaguuucagguguUu...............................    3714     1     1st
..........................................................gugagcCaaguuucaggugugu...............................    79       1     1st
..........................................................gugagcaaaguuUaggugugu...............................    442      1     1st
..........................................................gugagcaaaguuucaUgugugu...............................    153      1     1st
..........................................................gugagcaaagGuucaggugugu...............................    183      1     1st
..........................................................gugagcaaaguuuGaggugugu...............................    103      1     1st
..........................................................gugagAaaaguuucaggugugu...............................    61       1     1st
..........................................................gugagcaaaguuucagAugugu...............................    289      1     1st
..........................................................guAagcaaaguuucaggugugu...............................    198      1     1st
..........................................................guUagcaaaguuucaggugugu...............................    38       1     1st
..........................................................gAgagcaaaguuucaggugugu...............................    409      1     1st
..........................................................gugagcaaaguuucagguCugu...............................    111      1     1st
..........................................................gugagcaaaguuucaggugugCc..............................    6        1     1st
..........................................................gugagcaaaguuucaggugugucc..............................    44       0     1st
..........................................................gugagcaaaguuucaggugugGc..............................    1        1     1st
..........................................................gugagcaaaguuucagguguguG..............................    95       1     1st
..........................................................gugagcaaaguuucagguguUuc..............................    2        1     1st
..........................................................gugagcaaaguuucaggugugucg.............................    3        0     1st
..........................................................gugagcaaaguuucaggugugucU.............................    12       1     1st
..........................................................gugagcaaaguuucaggugugucC.............................    5        1     1st
..........................................................gugagcaaaguuucagguguguGg.............................    1        1     1st
..........................................................gugagcaaaguuucagguguguGgg............................    5        1     1st
..........................................................gugagcaaaguuucagguguguGggg...........................    1        1     1st
...........................................................ugagcaaaguuucaggugu.................................    2        0     1st
...........................................................ugagcUaaguuucaggugu.................................    1        1     1st
```

Star **Mature**

```
ugagagaaugucggccuugugugcccgaaacuuugacucaaccugaguuuaggaaaaggugagcaaaguuucaggugugucggguugacugaaaaaacugguuuaccagga
...........................................................ugagcaaaguuucaggugug................................ 3 0 1st
...........................................................ugagcaaaguuUaggugugu................................ 1 1 1st
...........................................................Ggagcaaaguuucaggugugu............................... 1 1 1st
...........................................................ugagcUaaguuucaggugugu............................... 2 1 1st
...........................................................ugagcCaaguuucaggugugu............................... 5 1 1st
...........................................................ugGgcaaaguuucaggugugu............................... 1 1 1st
...........................................................ugagcaaaguuucagguguUu............................... 1 1 1st
...........................................................ugagcaaaguuucGggugugu............................... 1 1 1st
...........................................................ugagcaaaguuucaggugugA............................... 1 1 1st
...........................................................ugagcaaaguuucaggugugu............................... 141 0 1st
...........................................................ugagcaGaguuucaggugugu............................... 1 1 1st
...........................................................ugagcaUaguuucaggugugu............................... 1 1 1st
...........................................................ugagcaaaguuucaggugugucc.............................. 1 0 1st
...........................................................ugagcaaaguuucaggugugucU............................. 1 1 1st
............................................................gagcaaaguuucaggugu................................. 2 0 1st
............................................................gagcaaaguuucagguguU................................ 1 1 1st
............................................................gagcaaaguuucaggugug................................ 3 0 1st
............................................................Uagcaaaguuucaggugugu............................... 1 1 1st
............................................................gagcaaGguuucaggugugu............................... 1 1 1st
............................................................gagcaaaAuuucaggugugu............................... 1 1 1st
............................................................gagcaaaguuucaggugugu............................... 213 0 1st
............................................................gGgcaaaguuucaggugugu............................... 1 1 1st
.............................................................agcaaaguuucaggugug................................ 3 0 1st
.............................................................agcaaaguuucaggugugu............................... 103 0 1st
.............................................................agcaaaguuucagguUugu............................... 1 1 1st
.............................................................agcaaaguuucaggugugA............................... 2 1 1st
..............................................................gcaaaguuucaggugugG............................... 1 1 1st
..............................................................gcaaaguuucaggugugu............................... 100 0 1st

..................gGgugcccgaaacuuugacucaacc.................................................................... 1 1 B01
..................Uugugcccgaaacuuugacucaaccu................................................................... 13 1 B01
...................Cgugcccgaaacuuugacucaaccu................................................................... 1 1 B01
...................ugugcccgaaacuuugacucaaccu................................................................... 6 0 B01
....................gugcccgaaacuuugacu......................................................................... 5 0 B01
....................gugcccgaaacuuugacuc........................................................................ 5 0 B01
....................gugcccgaaacuuugacuca....................................................................... 1 0 B01
....................gugcccgaaacuuugacucaa...................................................................... 15 0 B01
....................gugcccgaaacuuugacucaac..................................................................... 14 0 B01
....................gugUccgaaacuuugacucaac..................................................................... 1 1 B01
....................gugcccgaaacuuugUcucaacc.................................................................... 7 1 B01
....................gugcccgaaacuuugacucaacc.................................................................... 152 0 B01
....................gugcccgaaacuuugacucaaccG................................................................... 1 1 B01
....................gugcccgaUacuuugacucaaccu................................................................... 1 1 B01
....................gugccUgaaacuuugacucaaccu................................................................... 2 1 B01
....................gugcccgCaacuuugacucaaccu................................................................... 1 1 B01
....................gugcccgaaacuuugUcucaaccu................................................................... 46 1 B01
....................gGgcccgaaacuuugacucaaccu................................................................... 3 1 B01
....................gugcccgaaacuuugacucaaccA................................................................... 3 1 B01
....................gugcccgaaacuuugaUucaaccu................................................................... 6 1 B01
....................gugcccgaaacuuugacucaaccC................................................................... 7 1 B01
....................gugcccgaaacuuugacucaaccu................................................................... 2232 0 B01
....................gugcccgaaacuuugCcucaaccu................................................................... 2 1 B01
....................guUcccgaaacuuugacucaaccu................................................................... 1 1 B01
....................gugUccgaaacuuugacucaaccu................................................................... 1 1 B01
....................gugcccgaaacuuugacuUaaccu................................................................... 5 1 B01
....................gugcccgUaacuuugacucaaccu................................................................... 1 1 B01
....................gugcccgaaacuuugacucaaccuA.................................................................. 8 1 B01
....................gugcccgaaacuuugacucaaccuU.................................................................. 41 1 B01
.....................ugcccgaaacuuugacuc........................................................................ 1 0 B01
.....................ugcccgaaacuuugacuca....................................................................... 1 0 B01
.....................ugcccgaaacuuugacucaac..................................................................... 1 0 B01
.....................ugcccgaaacuuugacucaacc.................................................................... 4 0 B01
.....................ugcccgaaacuuugacuUaaccu................................................................... 1 1 B01
.....................Ggcccgaaacuuugacucaaccu................................................................... 1 1 B01
.....................ugcccgaaacuuugacucaaccu................................................................... 109 0 B01
.....................ugcccgaaacuuugacucaaccA................................................................... 1 1 B01
.....................Agcccgaaacuuugacucaaccu................................................................... 1 1 B01
.....................ugcccgaaacuuugacucaaccuU.................................................................. 27 1 B01
.....................ugcccgaaacuuugacucaaccuA.................................................................. 1 1 B01
......................gcccgaaacuuugacucaaccu................................................................... 1 0 B01
```

Star **Mature**

```
ugagagaaugucggccuugugugcccgaaacuuugacucaaccugaguuuaggaaaaggugagcaaaguuucaggugugucggguugacugaaaaacugguuuaccagga
........................ccgaaacuuugacucaaccu..................................................................  1     0  B01
..........................gaaacuuugacucaaccu..................................................................  2     0  B01
......................................................aaaGgugagcaaaguuucaggugug...............................  1     1  B01
.......................................................aaGgugagcaaaguuucaggugugu..............................  3     1  B01
........................................................aGgugagcaaaguuucaggugu................................  1     1  B01
.........................................................Ugugagcaaaguuucaggu..................................  1     1  B01
.........................................................Ugugagcaaaguuucaggug.................................  1     1  B01
.........................................................ggugagcaaaguuucaggugug...............................  1     0  B01
.........................................................Ugugagcaaaguuucaggugug...............................  4     1  B01
.........................................................Cgugagcaaaguuucaggugugu..............................  1     1  B01
.........................................................Ugugagcaaaguuucaggugugu..............................  46    1  B01
.........................................................ggugagcaaaguuucaggugugu..............................  1     0  B01
.........................................................Agugagcaaaguuucaggugugu..............................  2     1  B01
..........................................................gugagcaaaguuucaggu..................................  220   0  B01
..........................................................gugagcaaaguuucaggug.................................  76    0  B01
..........................................................gugagcaaaguuucagguA.................................  3     1  B01
..........................................................gugagcaaaguuucagUug.................................  1     1  B01
..........................................................gugGgcaaaguuucaggug.................................  1     1  B01
..........................................................gugagcaaaguuucagguU.................................  7     1  B01
..........................................................gugagcaaaguuucagguC.................................  2     1  B01
..........................................................gugagcaaaguuuUaggugu................................  2     1  B01
..........................................................gugagcaaaguuucaggugC................................  3     1  B01
..........................................................gugagcaaaguuucaAgugu................................  1     1  B01
..........................................................gugagcaaaguuucagUugu................................  1     1  B01
..........................................................gugagcaaaguuucaggugu................................  998   0  B01
..........................................................gugagcaaaguuucagAugu................................  1     1  B01
..........................................................gugagcaaaguuucaUgugu................................  2     1  B01
..........................................................gugagcaaaguuucagguUu................................  1     1  B01
..........................................................gugagcaaaguuucaggugCg...............................  1     1  B01
..........................................................gugagcaaaguuucaggGgug...............................  1     1  B01
..........................................................gugagcaaaCuuucaggugug...............................  1     1  B01
..........................................................gugagcaaaguuucagguUug...............................  1     1  B01
..........................................................gugagcaaaguuCcaggugug...............................  2     1  B01
..........................................................guCagcaaaguuucaggugug...............................  2     1  B01
..........................................................gugagcaaaguuucGggugug...............................  4     1  B01
..........................................................Augagcaaaguuucaggugug...............................  1     1  B01
..........................................................gugagcaaaguuucagguguA...............................  7     1  B01
..........................................................gugaAcaaaguuucaggugug...............................  1     1  B01
..........................................................Cugagcaaaguuucaggugug...............................  1     1  B01
..........................................................gugagcaaaguuucaggugug...............................  4216  0  B01
..........................................................gugaCcaaaguuucaggugug...............................  1     1  B01
..........................................................gugagcaaaguuucagguguC...............................  1     1  B01
..........................................................gugagcaaagCuucaggugug...............................  2     1  B01
..........................................................gugagcaaaguuucaggugGg...............................  2     1  B01
..........................................................gCgagcaaaguuucaggugug...............................  3     1  B01
..........................................................gugagcaaaguuuUaggugug...............................  5     1  B01
..........................................................gugGgcaaaguuucaggugug...............................  1     1  B01
..........................................................gugagUaaaguuucaggugug...............................  2     1  B01
..........................................................gugagcaaaguuucagguguU...............................  1074  1  B01
..........................................................gugagAaaaguuucaggugug...............................  1     1  B01
..........................................................gugCgcaaaguuucaggugugu..............................  12    1  B01
..........................................................guUagcaaaguuucaggugugu..............................  19    1  B01
..........................................................gugagcaaaguuucagguguAu..............................  10    1  B01
..........................................................gugagcaaaguGucaggugugu..............................  5     1  B01
..........................................................gugagcaaaguuCcaggugugu..............................  4     1  B01
..........................................................gugagcaaaguuucagCugugu..............................  6     1  B01
..........................................................gugagcaaaguuucagguCugu..............................  5     1  B01
..........................................................gugagcaaaguuucagguguUu..............................  1794  1  B01
..........................................................gugagcaaaguuucaCgugugu..............................  4     1  B01
..........................................................gugagUaaaguuucaggugugu..............................  25    1  B01
..........................................................gugaAcaaaguuucaggugugu..............................  17    1  B01
..........................................................gugagcaaaguuucaUgugugu..............................  7     1  B01
..........................................................gugagcaaaguuucCggugugu..............................  3     1  B01
..........................................................gugagcaaaguuucaggugugG..............................  77    1  B01
..........................................................gugagcaaaguuuAaggugugu..............................  1     1  B01
..........................................................gugagcaaUguuucaggugugu..............................  8     1  B01
..........................................................gugagcaaaguuucagUugugu..............................  12    1  B01
..........................................................gugUgcaaaguuucaggugugu..............................  6     1  B01
..........................................................gugagcaaagCuucaggugugu..............................  4     1  B01
..........................................................gugaCcaaaguuucaggugugu..............................  4     1  B01
```

Star **Mature**

```
ugagagaaugucggccuugugugcccgaaacuuugacucaaccugaguuuaggaaaaggugagcaaaguuucaggugugucggguugacugaaaaaacugguuuaccagga
..........................................................gugagcaaaguuucaggGgugu...............................  7      1   B01
..........................................................Cugagcaaaguuucaggugugu...............................  3      1   B01
..........................................................gugagcaaaguuucaggCgugu...............................  4      1   B01
..........................................................Uugagcaaaguuucaggugugu...............................  8      1   B01
..........................................................gugagAaaaguuucaggugugu...............................  1      1   B01
..........................................................gugagcaaagGuucaggugugu...............................  8      1   B01
..........................................................gugagcaaaguCucaggugugu...............................  7      1   B01
..........................................................gugagcaaaAuuucaggugugu...............................  2      1   B01
..........................................................gugagcaaaCuuucaggugugu...............................  2      1   B01
..........................................................gugagcaaaguuucUggugugu...............................  8      1   B01
..........................................................gugagcaaaguuucaAgugugu...............................  3      1   B01
..........................................................gugaUcaaaguuucaggugugu...............................  4      1   B01
..........................................................gugagcaaaguuucaggugGgu...............................  6      1   B01
..........................................................gugagcGaaguuucaggugugu...............................  4      1   B01
..........................................................gugagcaaaguuucagguguCu...............................  22     1   B01
..........................................................gGgagcaaaguuucaggugugu...............................  6      1   B01
..........................................................gCgagcaaaguuucaggugugu...............................  4      1   B01
..........................................................gugagcaaaguuucagguAugu...............................  1      1   B01
..........................................................gugagGaaaguuucaggugugu...............................  1      1   B01
..........................................................guCagcaaaguuucaggugugu...............................  9      1   B01
..........................................................gugagcaaaguuucagguUugu...............................  4      1   B01
..........................................................gugagcaaaguuucaggugCgu...............................  13     1   B01
..........................................................gugagcaaaguuuUaggugugu...............................  48     1   B01
..........................................................gugagcaaCguuucaggugugu...............................  2      1   B01
..........................................................gugagcaaaguuuGaggugugu...............................  2      1   B01
..........................................................guAagcaaaguuucaggugugu...............................  1      1   B01
..........................................................gugagcaaaguuucaggugugA...............................  3600   1   B01
..........................................................gugGgcaaaguuucaggugugu...............................  5      1   B01
..........................................................gugagcaGaguuucaggugugu...............................  7      1   B01
..........................................................gugagcaaaguuucagAugugu...............................  1      1   B01
..........................................................gugagcaaaguuucaggugugu...............................  42560  0   B01
..........................................................gugagcaaGguuucaggugugu...............................  1      1   B01
..........................................................gugagcaaaguuucGggugugu...............................  23     1   B01
..........................................................gAgagcaaaguuucaggugugu...............................  4      1   B01
..........................................................gugagcaaaguuucagguguUuc..............................  7      1   B01
..........................................................gugagcaaaguuucagguguguG..............................  13     1   B01
..........................................................gugagcaaaguuucaggugugucc..............................  8      0   B01
..........................................................gugagcaaaguuucaggugugCc..............................  1      1   B01
..........................................................gugagcaaaguuucaggugugAc..............................  5      1   B01
..........................................................gugagcaaaguuucaggugugucC.............................  1      1   B01
..........................................................gugagcaaaguuucagguguguGgg............................  1      1   B01
...........................................................ugagcaaaguuucaggugu.................................  1      0   B01
...........................................................ugagcaaaguuucaggugug................................  2      0   B01
...........................................................ugagcaaaguuucaggugugu...............................  11     0   B01
...........................................................ugagcaaaguuCcaggugugu...............................  1      1   B01
............................................................gagcaaaguuucaggugu.................................  2      0   B01
............................................................gagcaaaguuucaggugug................................  2      0   B01
............................................................gagcaaaguuucaggugugA...............................  3      1   B01
............................................................Uagcaaaguuucaggugugu...............................  1      1   B01
............................................................gagcaaaguuucaggugugu...............................  31     0   B01
.............................................................agcaaaguuucagguguU................................  1      1   B01
.............................................................agcaaaguuucaggugug................................  3      0   B01
.............................................................agcaaaguuuUaggugugu...............................  1      1   B01
.............................................................agcaaaguuucaggugugA...............................  1      1   B01
.............................................................agcaaaguuucaggugugu...............................  27     0   B01
..............................................................gcaaaguuucaggugugG...............................  1      1   B01
..............................................................gcaaaguuucaggugugA...............................  4      1   B01
..............................................................gcaaaguuucaggugugu...............................  38     0   B01

...................Cgugcccgaaacuuugacucaaccu...................................................................  1      1   TJ1
....................gugcccgaaacuCugacu.........................................................................  1      1   TJ1
....................gugcccgaaacuuugacA.........................................................................  2      1   TJ1
....................guAcccgaaacuuugacu.........................................................................  1      1   TJ1
....................gugcccgaaacuuugacG.........................................................................  1      1   TJ1
....................gugcccgaaacuuugacu.........................................................................  13     0   TJ1
....................gugcccgaaacuuugacC.........................................................................  2      1   TJ1
....................gugcccgaaacuuugacuc........................................................................  6      0   TJ1
....................gugcccgaaacuCugacuc........................................................................  1      1   TJ1
....................gugcccgaaacuuugUcuc........................................................................  2      1   TJ1
....................gugcccgaaacuuugacuU........................................................................  1      1   TJ1
```

Star   Mature

```
ugagagaaugucggccuugugugcccgaaacuuugacucaaccugaguuuaggaaaaggugagcaaaguuucaggugugucgguugacugaaaaaacugguuuaccagga
....................gugcccgaaacuuugacucC......................................................................  1    1  TJ1
....................gugcccgaaacuuugacucG......................................................................  1    1  TJ1
....................gugcccgGaacuuugacuca......................................................................  1    1  TJ1
....................gugcccgaaacuuugUcuca......................................................................  1    1  TJ1
....................gugcccgaaacuuugacuca......................................................................  19   0  TJ1
....................gugcccgaaacuuugUcucaa.....................................................................  1    1  TJ1
....................gugcccgaaGcuuugacucaa.....................................................................  1    1  TJ1
....................gugcccAaaacuuugacucaa.....................................................................  1    1  TJ1
....................gugcccgaaacuuugacucaa.....................................................................  44   0  TJ1
....................Cugcccgaaacuuugacucaa.....................................................................  1    1  TJ1
....................gugcccgaaacuuugaUucaac....................................................................  1    1  TJ1
....................gugcccgaaacuuugacucaac....................................................................  14   0  TJ1
....................gugcccgaaacuuugacucaacA...................................................................  8    1  TJ1
....................gugUccgaaacuuugacucaacc...................................................................  1    1  TJ1
....................gugcccgaaacuuugacucaacU...................................................................  13   1  TJ1
....................gugcccgaaacuuugUcucaacc...................................................................  2    1  TJ1
....................gugcccgaaacuuugacucaacc...................................................................  32   0  TJ1
....................gugcccgaaacuuugaUucaaccu..................................................................  1    1  TJ1
....................gugcccgaaacuuugacucCaccu..................................................................  2    1  TJ1
....................gugcccUaaacuuugacucaaccu..................................................................  2    1  TJ1
....................gugcccgGaacuuugacucaaccu..................................................................  1    1  TJ1
....................gugcccgaaacuuugacucaaccG..................................................................  12   1  TJ1
....................gugcccgaaacuuugacucaacUu..................................................................  2    1  TJ1
....................gugcccgaaacuuugacuUaaccu..................................................................  1    1  TJ1
....................gugcccgaaacuuugacucaaccC..................................................................  70   1  TJ1
....................gugcccgaaacuuuAacucaaccu..................................................................  1    1  TJ1
....................gugcccgaaGcuuugacucaaccu..................................................................  1    1  TJ1
....................gugcccgaaacuuugacCcaaccu..................................................................  2    1  TJ1
....................guAcccgaaacuuugacucaaccu..................................................................  1    1  TJ1
....................gugAccgaaacuuugacucaaccu..................................................................  1    1  TJ1
....................gugcccgaaacuuugacucaaGcu..................................................................  1    1  TJ1
....................gugcccgaaacuuugacucaaccu..................................................................  490  0  TJ1
....................gGgcccgaaacuuugacucaaccu..................................................................  1    1  TJ1
....................gugcccgaaacuuugacucGaccu..................................................................  2    1  TJ1
....................gugcUcgaaacuuugacucaaccu..................................................................  1    1  TJ1
....................gugcccgaaacuuugUcucaaccu..................................................................  4    1  TJ1
....................gugcccgaaacuuugacucaaUcu..................................................................  1    1  TJ1
....................gugccUgaaacuuugacucaaccu..................................................................  2    1  TJ1
....................gugUccgaaacuuugacucaaccu..................................................................  2    1  TJ1
....................gugcccAaaacuuugacucaaccu..................................................................  1    1  TJ1
....................gCgcccgaaacuuugacucaaccu..................................................................  2    1  TJ1
....................gugcccgaaacuuugacucaaccA..................................................................  48   1  TJ1
....................gugcccgaGacuuugacucaaccu..................................................................  3    1  TJ1
....................gugcccgaaacuuugacucaGccu..................................................................  2    1  TJ1
....................gugcccgaaacuuugacucaaccuA.................................................................  2    1  TJ1
....................gugcccgaaacuuugacucaaccuC.................................................................  1    1  TJ1
....................gugcccgaaacuuugacucaaccuU.................................................................  8    1  TJ1
.....................ugcccgaaacuuugacuca......................................................................  1    0  TJ1
.....................ugcccgaaacuuugacucaa.....................................................................  1    0  TJ1
.....................ugcccgaaacuuugacucaacU...................................................................  1    1  TJ1
.....................ugcccgaaacuuugacucaacc...................................................................  1    0  TJ1
.....................ugcccgaaacuuugacucaaccA..................................................................  3    1  TJ1
.....................ugcccgaaacuuugacucaaccG..................................................................  2    1  TJ1
.....................ugcccgaaacuuugacucaacGu..................................................................  1    1  TJ1
.....................ugcccgaaacuuugacucaaccu..................................................................  17   0  TJ1
.....................ugcccgaaacuuugacucGaccu..................................................................  1    1  TJ1
.....................ugcccgaaacuuugacucaaccC..................................................................  2    1  TJ1
.....................ugcccgaaacuuugacucaaccuA.................................................................  1    1  TJ1
.....................ugcccgaaacuuugacucaaccuU.................................................................  3    1  TJ1
........................ccgaaacuuugacucaaccu..................................................................  1    0  TJ1
........................................................aAgugagcaaaguuucaggugugu..............................  1    1  TJ1
........................................................ggugagcaaaguuucaggugug................................  2    0  TJ1
........................................................ggugagcaaaguuucaggugugu...............................  1    0  TJ1
.........................................................Cgugagcaaaguuucaggugugu..............................  8    1  TJ1
........................................................ggugagcaaaguuucaggugugA...............................  1    1  TJ1
..........................................................Augagcaaaguuucaggu..................................  1    1  TJ1
..........................................................gugagcaaaguuucaggC..................................  3    1  TJ1
..........................................................guUagcaaaguuucaggu..................................  1    1  TJ1
..........................................................gugagcaaaguuucaggA..................................  3    1  TJ1
..........................................................gugagcaaaguuucaggu..................................  36   0  TJ1
```

```
                 Star                                  Mature
ugagagaaugucggccuugugugcccgaaacuuugacucaaccugaguuuaggaaaaggugagcaaaguuucaggugugucggguugacugaaaaaacugguuuaccagga
..........................................................gugagcaaaguuucGggug..................................    1     1    TJ1
..........................................................gugGgcaaaguuucaggug..................................    2     1    TJ1
..........................................................gugagcaaaguuucagguU..................................    4     1    TJ1
..........................................................gugagcaaaguuucaggug..................................    125   0    TJ1
..........................................................gugagcaaaguuCcaggug..................................    1     1    TJ1
..........................................................gugagcaaaguuucagguC..................................    1     1    TJ1
..........................................................gugagcaaaCuuucaggug..................................    2     1    TJ1
..........................................................gugCgcaaaguuucaggug..................................    1     1    TJ1
..........................................................gugagcaaagCuucaggug..................................    1     1    TJ1
..........................................................gugagcaaaguuucaggCg..................................    1     1    TJ1
..........................................................gugagcaaaguuucagguA..................................    14    1    TJ1
..........................................................gugagcaaaguuuUaggugu.................................    2     1    TJ1
..........................................................gCgagcaaaguuucaggugu.................................    1     1    TJ1
..........................................................Augagcaaaguuucaggugu.................................    1     1    TJ1
..........................................................gugagcaaagCuucaggugu.................................    1     1    TJ1
..........................................................gugagUaaaguuucaggugu.................................    1     1    TJ1
..........................................................gAgagcaaaguuucaggugu.................................    1     1    TJ1
..........................................................guAagcaaaguuucaggugu.................................    2     1    TJ1
..........................................................gGgagcaaaguuucaggugu.................................    1     1    TJ1
..........................................................gugagcaaaguuucaggugG.................................    10    1    TJ1
..........................................................gugagcaaaguuucaggugu.................................    245   0    TJ1
..........................................................gugagcGaaguuucaggugu.................................    2     1    TJ1
..........................................................gugagcaaaguuucaggGgu.................................    4     1    TJ1
..........................................................gugagcaaGguuucaggugu.................................    1     1    TJ1
..........................................................gugagcaaaguuucGggugu.................................    1     1    TJ1
..........................................................gugagcaaaguuucUggugu.................................    1     1    TJ1
..........................................................gugagcaaaguuucaggugC.................................    48    1    TJ1
..........................................................gugagcaaaguuucaggugAg................................    1     1    TJ1
..........................................................gugagcaUaguuucaggugug................................    3     1    TJ1
..........................................................gugagcaaaguuucagguUug................................    2     1    TJ1
..........................................................gugagcaaaguuucagguguU................................    54    1    TJ1
..........................................................gugagcaGaguuucaggugug................................    6     1    TJ1
..........................................................gugagUaaaguuucaggugug................................    2     1    TJ1
..........................................................gugagcGaaguuucaggugug................................    9     1    TJ1
..........................................................gugagcaaaguuucagguguA................................    242   1    TJ1
..........................................................gugagcaaaguuCcaggugug................................    10    1    TJ1
..........................................................gugagcaaaguuucagguCug................................    1     1    TJ1
..........................................................gugagcaaaguuucagguAug................................    8     1    TJ1
..........................................................gugagcaaaguuucagguguC................................    11    1    TJ1
..........................................................gugagcaaaguCucaggugug................................    6     1    TJ1
..........................................................gugagcaaGguuucaggugug................................    10    1    TJ1
..........................................................guUagcaaaguuucaggugug................................    1     1    TJ1
..........................................................gugagcaaaAuuucaggugug................................    1     1    TJ1
..........................................................gugagcaaaCuuucaggugug................................    1     1    TJ1
..........................................................Augagcaaaguuucaggugug................................    2     1    TJ1
..........................................................gugaAcaaaguuucaggugug................................    3     1    TJ1
..........................................................gugagcaaaguuucaggGgug................................    1     1    TJ1
..........................................................gugagcaaaguuucUggugug................................    2     1    TJ1
..........................................................gugagcaaaguuucaggugug................................    1998  0    TJ1
..........................................................gugagcaaagCuucaggugug................................    6     1    TJ1
..........................................................gugagcaCaguuucaggugug................................    1     1    TJ1
..........................................................gugagcaaaguuucaggugGg................................    5     1    TJ1
..........................................................gugagcaaaguuucaggugCg................................    11    1    TJ1
..........................................................gugagAaaaguuucaggugug................................    5     1    TJ1
..........................................................gugagcUaaguuucaggugug................................    1     1    TJ1
..........................................................guAagcaaaguuucaggugug................................    2     1    TJ1
..........................................................gugGgcaaaguuucaggugug................................    12    1    TJ1
..........................................................gGgagcaaaguuucaggugug................................    1     1    TJ1
..........................................................gugagcaaaguuucaCgugug................................    2     1    TJ1
..........................................................gugagcaaaguuucaggCgug................................    5     1    TJ1
..........................................................gugagcaaaguuGcaggugug................................    1     1    TJ1
..........................................................gugagcaaaguuucaUgugug................................    1     1    TJ1
..........................................................gCgagcaaaguuucaggugug................................    6     1    TJ1
..........................................................gugagcaaaguuuUaggugug................................    3     1    TJ1
..........................................................gugUgcaaaguuucaggugug................................    1     1    TJ1
..........................................................gugagcaaaguuucaAgugug................................    1     1    TJ1
..........................................................gugagcaaaguuucGggugug................................    4     1    TJ1
..........................................................gugUgcaaaguuucaggugugu...............................    1     1    TJ1
..........................................................gugagcaaaguuucCggugugu...............................    2     1    TJ1
..........................................................gugagcaaGguuucaggugugu...............................    27    1    TJ1
```

Star Mature

```
ugagagaaugucggccuugugugcccgaaacuuugacucaaccugaguuuaggaaaaggugagcaaaguuucaggugugucggguugacugaaaaaacugguuuaccagga
..........................................................gugagcaaaCuuucaggugugu...............................  1     1  TJ1
..........................................................gugagcaaaguuucaggAgugu...............................  4     1  TJ1
..........................................................gGgagcaaaguuucaggugugu...............................  2     1  TJ1
..........................................................gugagcaGaguuucaggugugu...............................  23    1  TJ1
..........................................................gugagcaaagGuucaggugugu...............................  2     1  TJ1
..........................................................gugagcaaaguuucaggugCgu...............................  22    1  TJ1
..........................................................gugagcaaagAuucaggugugu...............................  2     1  TJ1
..........................................................gugagUaaaguuucaggugugu...............................  12    1  TJ1
..........................................................gugagcaaaguuucaggugugA...............................  372   1  TJ1
..........................................................gugagcaaaguuucagguAugu...............................  19    1  TJ1
..........................................................gugagcaaaUuuucaggugugu...............................  8     1  TJ1
..........................................................gugagcaaaguuAcaggugugu...............................  1     1  TJ1
..........................................................Augagcaaaguuucaggugugu...............................  9     1  TJ1
..........................................................gugaAcaaaguuucaggugugu...............................  7     1  TJ1
..........................................................gugGgcaaaguuucaggugugu...............................  29    1  TJ1
..........................................................gugagcaaagCuucaggugugu...............................  15    1  TJ1
..........................................................gugagcaaaguuucUggugugu...............................  11    1  TJ1
..........................................................gugagcaaaguuucaggugGgu...............................  8     1  TJ1
..........................................................guUagcaaaguuucaggugugu...............................  5     1  TJ1
..........................................................gugagcaaaguuuAaggugugu...............................  1     1  TJ1
..........................................................gugagcaaUguuucaggugugu...............................  3     1  TJ1
..........................................................gugagcaaaguuucagAugugu...............................  7     1  TJ1
..........................................................guAagcaaaguuucaggugugu...............................  30    1  TJ1
..........................................................gugagcaaaguuucaggugugu...............................  7220  0  TJ1
..........................................................gugagcaaaguuucGgugugu................................  34    1  TJ1
..........................................................gugagcaaaguuucagUugugu...............................  1     1  TJ1
..........................................................gugagcaUaguuucaggugugu...............................  3     1  TJ1
..........................................................gugagcaaaguuucagguguUu...............................  18    1  TJ1
..........................................................gugagcaaaguuucaUgugugu...............................  2     1  TJ1
..........................................................gugagcaaaguuucaggCgugu...............................  21    1  TJ1
..........................................................gugagcaaaguuucagguguAu...............................  18    1  TJ1
..........................................................gugagcaaaguuCcaggugugu...............................  20    1  TJ1
..........................................................gugagcaaaguuucagguguCu...............................  1     1  TJ1
..........................................................Uugagcaaaguuucaggugugu...............................  5     1  TJ1
..........................................................gugagcaaaguuucaggGgugu...............................  5     1  TJ1
..........................................................gugagcaaaguuUaggugugu................................  7     1  TJ1
..........................................................gugagcaaaguuucaggugugG...............................  202   1  TJ1
..........................................................guCagcaaaguuucaggugugu...............................  2     1  TJ1
..........................................................gugagcGaaguuucaggugugu...............................  37    1  TJ1
..........................................................gugagcaaaAuucaggugugu................................  9     1  TJ1
..........................................................gAgagcaaaguuucaggugugu...............................  5     1  TJ1
..........................................................gugagcaaaguuucaAgugugu...............................  4     1  TJ1
..........................................................gugagcCaaguuucaggugugu...............................  2     1  TJ1
..........................................................gugagAaaaguuucaggugugu...............................  7     1  TJ1
..........................................................gCgagcaaaguuucaggugugu...............................  11    1  TJ1
..........................................................gugagcUaaguuucaggugugu...............................  5     1  TJ1
..........................................................gugagcaaaguuGcaggugugu...............................  1     1  TJ1
..........................................................gugCgcaaaguuucaggugugu...............................  1     1  TJ1
..........................................................gugagcaaaguCucaggugugu...............................  10    1  TJ1
..........................................................gugagcaaaguuucaggugAgu...............................  4     1  TJ1
..........................................................gugagGaaaguuucaggugugu...............................  4     1  TJ1
..........................................................gugagcaaaguuuGaggugugu...............................  2     1  TJ1
..........................................................gugagcaaaguuucaggugugugG..............................  62    1  TJ1
..........................................................gugagcaaaguuucagguguguc..............................  27    0  TJ1
..........................................................gugagcaaaguuucaggugugCc..............................  1     1  TJ1
..........................................................gugagcaaaguuucaggugugCcg.............................  1     1  TJ1
..........................................................gugagcaaaguuucaggugugucU.............................  1     1  TJ1
..........................................................gugagcaaaguuucaggugugucggA...........................  1     1  TJ1
..........................................................gugagcaaaguuucaggugugucggg...........................  1     0  TJ1
...........................................................ugagcaaaguuucagguguA................................  1     1  TJ1
...........................................................ugagcaaaguuucaggugug................................  2     0  TJ1
...........................................................ugagcaaaguuucaggugugu...............................  8     0  TJ1
............................................................gagcaaaguuucaggugC.................................  1     1  TJ1
............................................................gagcaaaguuucaggugugu...............................  3     0  TJ1

...................Cgugcccgaaacuuugacucaaccu...................................................................  4     1  BJ1
....................gugcccgaaacuuugUcu.........................................................................  1     1  BJ1
....................gugcUcgaaacuuugacu.........................................................................  2     1  BJ1
....................gugcccgaaacuuugacu.........................................................................  17    0  BJ1
....................gugcAcgaaacuuugacu.........................................................................  1     1  BJ1
```

Star Mature

```
ugagagaaugucggccuugugugcccgaaacuuugacucaaccugaguuuaggaaaaggugagcaaaguuucaggugugucggguugacugaaaaaacugguuuaccagga
....................gugcccgaaacuuugacuU........................................................................ 1 1 BJ1
....................gugcccgaaacuuugacuA........................................................................ 2 1 BJ1
....................gugccUgaaacuuugacuc........................................................................ 1 1 BJ1
....................gugcccgaaacuuugacuc........................................................................ 22 0 BJ1
....................gugcccgaaacuuugUcuc........................................................................ 4 1 BJ1
....................gugccAgaaacuuugacuc........................................................................ 1 1 BJ1
....................gugcGcgaaacuuugacuc........................................................................ 1 1 BJ1
....................gugcccgaaacuuCgacuca....................................................................... 1 1 BJ1
....................gugcccgaaacuuugacucG....................................................................... 2 1 BJ1
....................gugcccgaaacuuugUcuca....................................................................... 3 1 BJ1
....................gCgcccgaaacuuugacuca....................................................................... 2 1 BJ1
....................gugUccgaaacuuugacuca....................................................................... 1 1 BJ1
....................gugcccgaaacuuugacuca....................................................................... 95 0 BJ1
....................gugcccgaUacuuugacucaa...................................................................... 1 1 BJ1
....................gugcccgaaacuuugUcucaa...................................................................... 4 1 BJ1
....................gugUccgaaacuuugacucaa...................................................................... 1 1 BJ1
....................gugcAcgaaacuuugacucaa...................................................................... 1 1 BJ1
....................gugcccgaaacuuugacucaG...................................................................... 3 1 BJ1
....................gugccUgaaacuuugacucaa...................................................................... 1 1 BJ1
....................gugcccgaaacuuCgacucaa...................................................................... 1 1 BJ1
....................gugcccgaaacuuugacucaa...................................................................... 100 0 BJ1
....................gugcccgaaacuuugUcucaac..................................................................... 3 1 BJ1
....................gugcAcgaaacuuugacucaac..................................................................... 1 1 BJ1
....................gugccUgaaacuuugacucaac..................................................................... 2 1 BJ1
....................gugcccgaaacuCugacucaac..................................................................... 1 1 BJ1
....................gugccAgaaacuuugacucaac..................................................................... 1 1 BJ1
....................gugcccgaaacuuugacucaac..................................................................... 70 0 BJ1
....................gugcccgaaacuuugacucaaU..................................................................... 3 1 BJ1
....................gugcccgGaacuuugacucaac..................................................................... 2 1 BJ1
....................gugcccgaaacuuugacucaacA.................................................................... 15 1 BJ1
....................gugcccgaaacuAugacucaacc.................................................................... 1 1 BJ1
....................gugcccgGaacuuugacucaacc.................................................................... 1 1 BJ1
....................gugcccgaaacuuAgacucaacc.................................................................... 2 1 BJ1
....................gugcccgaaacuuugacucaGcc.................................................................... 1 1 BJ1
....................gugcccgaGacuuugacucaacc.................................................................... 2 1 BJ1
....................gugcccgaaacuuugacucaacG.................................................................... 1 1 BJ1
....................gugcccgaaacuCugacucaacc.................................................................... 1 1 BJ1
....................guAcccgaaacuuugacucaacc.................................................................... 1 1 BJ1
....................gugcccgaaCcuuugacucaacc.................................................................... 1 1 BJ1
....................gugccUgaaacuuugacucaacc.................................................................... 1 1 BJ1
....................gugcccgaaacuuugacucaCcc.................................................................... 1 1 BJ1
....................gugcccgaaacuuugacucaacU.................................................................... 55 1 BJ1
....................gugcccgaaacuuugacucaacc.................................................................... 327 0 BJ1
....................gugcccgaaGcuuugacucaacc.................................................................... 1 1 BJ1
....................gugcccgaaacuuCgacucaacc.................................................................... 3 1 BJ1
....................gugcAcgaaacuuugacucaacc.................................................................... 2 1 BJ1
....................Augcccgaaacuuugacucaacc.................................................................... 2 1 BJ1
....................gugcccgaaacuuugUcucaacc.................................................................... 5 1 BJ1
....................gugcccgaaaGuuugacucaacc.................................................................... 1 1 BJ1
....................gugccAgaaacuuugacucaacc.................................................................... 1 1 BJ1
....................gugcGcgaaacuuugacucaacc.................................................................... 1 1 BJ1
....................gugcccgaaacuuugacCcaacc.................................................................... 2 1 BJ1
....................gugcccgaaacuuuUacucaaccu................................................................... 1 1 BJ1
....................gugcccgaGacuuugacucaaccu................................................................... 14 1 BJ1
....................gugcccgaaacuuugacAcaaccu................................................................... 3 1 BJ1
....................gugcccgaaaUuuugacucaaccu................................................................... 3 1 BJ1
....................gAgcccgaaacuuugacucaaccu................................................................... 8 1 BJ1
....................gugcccgaaacuuugacucGaccu................................................................... 14 1 BJ1
....................gugcccgaaGcuuugacucaaccu................................................................... 13 1 BJ1
....................gugcccgaCacuuugacucaaccu................................................................... 4 1 BJ1
....................gugcccgaaacuuugacuUaaccu................................................................... 6 1 BJ1
....................gugcccgaaacuuGgacucaaccu................................................................... 3 1 BJ1
....................gugcccgaaacuuugacucaaccA................................................................... 207 1 BJ1
....................gugcccgaaacuuCgacucaaccu................................................................... 22 1 BJ1
....................gugcGcgaaacuuugacucaaccu................................................................... 6 1 BJ1
....................gCgcccgaaacuuugacucaaccu................................................................... 11 1 BJ1
....................gugcccgaaacuuugacucaCccu................................................................... 3 1 BJ1
....................guAcccgaaacuuugacucaaccu................................................................... 4 1 BJ1
....................gugcccgaUacuuugacucaaccu................................................................... 3 1 BJ1
....................Uugcccgaaacuuugacucaaccu................................................................... 6 1 BJ1
```

Star Mature

ugagagaaugucggccuugugugcccgaaacuuugacucaaccugaguuuaggaaaaggugagcaaaguuucaggugugucgguugacugaaaaaacugguuuaccagga			
....................gGgcccgaaacuuugacucaaccu..	2	1	BJ1
....................gugcccgaaacuuugacucaaccu..	3666	0	BJ1
....................gugcccgaaacuuugUcucaaccu..	100	1	BJ1
....................gugcccgaaacuuugacuAaaccu..	1	1	BJ1
....................Augcccgaaacuuugacucaaccu..	18	1	BJ1
....................gugcccAaaacuuugacucaaccu..	3	1	BJ1
....................gugcAcgaaacuuugacucaaccu..	23	1	BJ1
....................gugcccgGaacuuugacucaaccu..	11	1	BJ1
....................gugcccgaaacuGugacucaaccu..	1	1	BJ1
....................gugcccgaaacuuugacCcaaccu..	14	1	BJ1
....................gugcccgaaacuCugacucaaccu..	16	1	BJ1
....................gugcccgaaacuuugacucaacUu..	11	1	BJ1
....................gugcccgaaacuuugacucaGccu..	15	1	BJ1
....................gugccUgaaacuuugacucaaccu..	30	1	BJ1
....................gugcccgaaacuuugacucaaccC..	363	1	BJ1
....................gugccAgaaacuuugacucaaccu..	10	1	BJ1
....................guUccgaaacuuugacucaaccu..	2	1	BJ1
....................gugcccgaaacuuugacucaaccG..	57	1	BJ1
....................gugcccgaaacuuugCcucaaccu..	1	1	BJ1
....................gugcccgaaacuuugacucUaccu..	1	1	BJ1
....................gugcccgaaacuAugacucaaccu..	1	1	BJ1
....................gugcccgaaacuuuAacucaaccu..	5	1	BJ1
....................gugcccgaaacuuugaUucaaccu..	4	1	BJ1
....................gugcUcgaaacuuugacucaaccu..	14	1	BJ1
....................gugAccgaaacuuugacucaaccu..	1	1	BJ1
....................Cugcccgaaacuuugacucaaccu..	2	1	BJ1
....................gugcccgaaacuuAgacucaaccu..	10	1	BJ1
....................gugUccgaaacuuugacucaaccu..	4	1	BJ1
....................gugcccgaaacuuugacucaaUcu..	8	1	BJ1
....................gugcccgaaacuuugacucaaccuA...	7	1	BJ1
....................gugcccgaaacuuugacucaaccuC...	7	1	BJ1
....................gugcccgaaacuuugacucaaccuU...	66	1	BJ1
....................gugcccgaaacuuugacucaaccAga..	1	1	BJ1
.....................ugcccgaaacuuugacuc...	1	0	BJ1
.....................ugcccgaaacuuugUcuc...	1	1	BJ1
.....................uAcccgaaacuuugacuca..	1	1	BJ1
.....................ugcccgaaacuuugacuca..	1	0	BJ1
.....................ugcccgaaacuuugacucaa...	15	0	BJ1
.....................ugcccgaaacuuugacucaac..	2	0	BJ1
.....................ugcccgaaacuuugacucaacc...	5	0	BJ1
.....................ugcccgaaacuCugacucaaccu..	1	1	BJ1
.....................ugcccgaaacuuugacucaaccA..	6	1	BJ1
.....................ugcccgaaacuuugacucaaccG..	1	1	BJ1
.....................Ggcccgaaacuuugacucaaccu..	1	1	BJ1
.....................ugcccgaaacuuugacucaacUu..	1	1	BJ1
.....................Cgcccgaaacuuugacucaaccu..	5	1	BJ1
.....................ugcccgaaacuuugacuAaaccu..	1	1	BJ1
.....................Agcccgaaacuuugacucaaccu..	1	1	BJ1
.....................ugcccgaaacuuugacucaaccC..	13	1	BJ1
.....................ugcccgaaacuuugacucaaccu..	96	0	BJ1
.....................ugcccgaaacuuugacucaaccuA...	1	1	BJ1
.....................ugcccgaaacuuugacucaaccuC...	5	1	BJ1
.....................ugcccgaaacuuugacucaaccuU...	37	1	BJ1
......................gcccgaaacuuugacuca..	1	0	BJ1
........................ccgaaacuuugacucaaccu..	3	0	BJ1
....Cgugagcaaaguuucaggugugu..............................	1	1	BJ1
....gugagcaaaguuucaggugC................................	2	1	BJ1
....gugagcaaaguuucaggugu................................	5	0	BJ1
....gugagcaaagCuucaggugug...............................	1	1	BJ1
....gugagcaaaguuucaggugug...............................	23	0	BJ1
....gugagcaaaguuucaggugCg...............................	1	1	BJ1
....gugagcaaaguuucagguguA...............................	1	1	BJ1
....gAgagcaaaguuucaggugugu..............................	2	1	BJ1
....gugagcaaCguuucaggugugu..............................	2	1	BJ1
....gugagcaaaguuucaggugCgu..............................	1	1	BJ1
....gugagcaaaguuuAaggugugu..............................	2	1	BJ1
....gugagcaaagCuucaggugugu..............................	4	1	BJ1
....gugGgcaaaguuucaggugugu..............................	2	1	BJ1
....gugaUcaaaguuucaggugugu..............................	2	1	BJ1
....gugagcaaaguuCcaggugugu..............................	1	1	BJ1

```
                    Star                                  Mature
ugagagaaugucggccuugugugcccgaaacuuugacucaaccugaguuuaggaaaaggugagcaaaguuucaggugugucggguugacugaaaaaacugguuuaccagga
..........................................................gugagGaaaguuucaggugugu...............................    1     1     BJ1
..........................................................gugagcaaGguuucaggugugu...............................    1     1     BJ1
..........................................................gugagcGaaguuucaggugugu...............................    1     1     BJ1
..........................................................guAagcaaaguuucaggugugu...............................    1     1     BJ1
..........................................................gugagcaaaguuucaggugugu...............................    250   0     BJ1
..........................................................gugagcUaaguuucaggugugu...............................    1     1     BJ1
..........................................................Augagcaaaguuucaggugugu...............................    1     1     BJ1
..........................................................gugagcaaaguuucGggugugu...............................    2     1     BJ1
..........................................................gugagcaaaguuucagAugugu...............................    1     1     BJ1
..........................................................gugagcaaaguuucaCgugugu...............................    1     1     BJ1
..........................................................gCgagcaaaguuucaggugugu...............................    3     1     BJ1
..........................................................gugagcaaaguuucaggAgugu...............................    1     1     BJ1
..........................................................gugaAcaaaguuucaggugugu...............................    1     1     BJ1
..........................................................gugagcaaaguCucaggugugu...............................    1     1     BJ1
..........................................................gugagcaaaguuucaggugugA...............................    7     1     BJ1
...........................................................ugagcaaaguuucGggugugu...............................    1     1     BJ1
...........................................................Cgagcaaaguuucaggugugu...............................    1     1     BJ1

....................gugcccgaaacuuugacu.........................................................................    4     0     HA1
....................gugcccgaaacuuugacuA........................................................................    4     1     HA1
....................gugcccgaaacuuugaUuc........................................................................    1     1     HA1
....................gugcccgaaacuuugacuU........................................................................    3     1     HA1
....................gugccUgaaacuuugacuc........................................................................    1     1     HA1
....................gugcccgaaacuuugacCc........................................................................    1     1     HA1
....................gugcccgaaacuuugacuc........................................................................    38    0     HA1
....................gugcccgaaacuuugacucU.......................................................................    5     1     HA1
....................gugcccgaaacuuugacuca.......................................................................    108   0     HA1
....................gugcccgaaaUuuugacuca.......................................................................    1     1     HA1
....................gugcccgaaacuuugacucG.......................................................................    5     1     HA1
....................gugcccgaaacuuugacCca.......................................................................    2     1     HA1
....................gugcccgaaacuuugUcuca.......................................................................    3     1     HA1
....................gugcccgaaacuCugacuca.......................................................................    1     1     HA1
....................Augcccgaaacuuugacucaa......................................................................    1     1     HA1
....................gugcccgaGacuuugacucaa......................................................................    1     1     HA1
....................gugcccgaaacuuugacCcaa......................................................................    1     1     HA1
....................gugcccgaaacuuugUcucaa......................................................................    1     1     HA1
....................gugcccgaaacuuugacucaa......................................................................    134   0     HA1
....................gugcccgaaacuuugacucaG......................................................................    3     1     HA1
....................gugcccgaaacuuugacucaU......................................................................    1     1     HA1
....................gugcccgaaacuuugUcucaac.....................................................................    7     1     HA1
....................gugcccgaaacuuugacucaaA.....................................................................    1     1     HA1
....................gugcccgaaacuuugacucGac.....................................................................    1     1     HA1
....................gugcccgaaacuuCgacucaac.....................................................................    1     1     HA1
....................gCgcccgaaacuuugacucaac.....................................................................    2     1     HA1
....................gugcccgaaacuuugacucaaU.....................................................................    15    1     HA1
....................gugcccgGaacuuugacucaac.....................................................................    2     1     HA1
....................gugcccgaaacuuugacCcaac.....................................................................    1     1     HA1
....................gugcccgaaacuuugacucaGc.....................................................................    1     1     HA1
....................gugcccAaaacuuugacucaac.....................................................................    1     1     HA1
....................gugcccgaaacuuugacucaac.....................................................................    161   0     HA1
....................gugcccgaaacuuugacucaacU....................................................................    33    1     HA1
....................gugcccgaCacuuugacucaacc....................................................................    1     1     HA1
....................gugcccgaaacuCugacucaacc....................................................................    1     1     HA1
....................gugcccgaaacuuugUcucaacc....................................................................    1     1     HA1
....................gugcccgaaacuuugacucaacA....................................................................    7     1     HA1
....................gugcccgaaacuuugacucGacc....................................................................    2     1     HA1
....................gugccUgaaacuuugacucaacc....................................................................    1     1     HA1
....................gugcccgaaacuuugacuUaacc....................................................................    1     1     HA1
....................gugcccgaaacuuugacucaGcc....................................................................    1     1     HA1
....................Augcccgaaacuuugacucaacc....................................................................    1     1     HA1
....................gugcccgaaacuuugacucaCcc....................................................................    2     1     HA1
....................gugcccgaaacuuugacucaacc....................................................................    145   0     HA1
....................gugcccgaaGcuuugacucaacc....................................................................    1     1     HA1
....................gugcccgaaacuuugacCcaacc....................................................................    1     1     HA1
....................gugcUcgaaacuuugacucaaccu...................................................................    1     1     HA1
....................gugcccgaaacuuugacucaaccu...................................................................    1227  0     HA1
....................gugcccgaaacuuugacuUaaccu...................................................................    1     1     HA1
....................gugcccgaaacuuugacucaaccC...................................................................    167   1     HA1
....................gCgcccgaaacuuugacucaaccu...................................................................    2     1     HA1
....................gugcccgaaacuCugacucaaccu...................................................................    4     1     HA1
```

```
                    Star                                  Mature
ugagagaaugucggccuugugugcccgaaacuuugacucaaccugaguuuaggaaaaggugagcaaaguuucaggugugucgguugacugaaaaaacugguuuaccagga
....................gugcccgaUacuuugacucaaccu..................................................................  1    1  HA1
....................gugcccgaaacuuugacucUaccu..................................................................  1    1  HA1
....................gugcccgaaaUuuugacucaaccu..................................................................  2    1  HA1
....................gugcccgaaacuuugacucaaccG..................................................................  42   1  HA1
....................gugcAcgaaacuuugacucaaccu..................................................................  2    1  HA1
....................gugcccgaaacuuugacucGaccu..................................................................  4    1  HA1
....................gugcccgUaacuuugacucaaccu..................................................................  3    1  HA1
....................gugcccgaaacuuugacucaGccu..................................................................  4    1  HA1
....................Augcccgaaacuuugacucaaccu..................................................................  1    1  HA1
....................gugcccgaGacuuugacucaaccu..................................................................  6    1  HA1
....................gugccUgaaacuuugacucaaccu..................................................................  7    1  HA1
....................gugcccgaaacuuugacucaaAcu..................................................................  2    1  HA1
....................gugcccgaaacuuugacucaacGu..................................................................  1    1  HA1
....................gugUccgaaacuuugacucaaccu..................................................................  2    1  HA1
....................gugcccgaaacuuugacAcaaccu..................................................................  2    1  HA1
....................guAcccgaaacuuugacucaaccu..................................................................  1    1  HA1
....................gugcccgaaGcuuugacucaaccu..................................................................  4    1  HA1
....................gugcccgaaacuuugacCcaaccu..................................................................  3    1  HA1
....................gugcccgaaacGuugacucaaccu..................................................................  1    1  HA1
....................gugcccCaaacuuugacucaaccu..................................................................  1    1  HA1
....................gugcccgGaacuuugacucaaccu..................................................................  2    1  HA1
....................gugcccgaaacuuugaUucaaccu..................................................................  3    1  HA1
....................gugcccgaaacuuCgacucaaccu..................................................................  2    1  HA1
....................gugcccgaaacuuugUcucaaccu..................................................................  33   1  HA1
....................gugcccgaaacuuugacucaaccA..................................................................  108  1  HA1
....................gugcccgaaacuuAgacucaaccu..................................................................  2    1  HA1
....................gugcccgaaacuuugacucaaccuU.................................................................  13   1  HA1
....................gugcccgaaacuuugacucaaccuA.................................................................  6    1  HA1
.....................ugcccgaaacuuugacucaac....................................................................  3    0  HA1
.....................ugcccgaaacuuugacAcaac....................................................................  1    1  HA1
.....................ugcccgaaacuuugacucaacc...................................................................  3    0  HA1
.....................ugcccgaaacuuugacucaacA...................................................................  2    1  HA1
.....................ugcccgaaacuuugacucaacU...................................................................  6    1  HA1
.....................ugcccgGaacuuugacucaacc...................................................................  1    1  HA1
.....................ugcccgaaacuuugacucaaccu..................................................................  42   0  HA1
.....................ugcccgaaacuuugacucGaccu..................................................................  1    1  HA1
.....................ugcccgaaacuuugacucaaccC..................................................................  5    1  HA1
.....................ugcccgaaacuuugacucaaccA..................................................................  5    1  HA1
.....................ugcccgaaacuuugacucaaccG..................................................................  3    1  HA1
.....................ugcccgaaacuuugacucaaccuU.................................................................  2    1  HA1
.....................ugcccgaaacuuugacucaaccuC.................................................................  2    1  HA1
.....................ugcccgaaacuuugacucaaccugC................................................................  2    1  HA1
........................ccgaaacuuugacucaaccu..................................................................  1    0  HA1
..........................gaaacuuugacucaaccu..................................................................  1    0  HA1
........................................................aAgugagcaaaguuucaggugugu..............................  3    1  HA1
.........................................................ggugagcaaaguuucaggugu................................  9    0  HA1
.........................................................Cgugagcaaaguuucaggugu................................  2    1  HA1
.........................................................ggugagcaaaguuucagguguA...............................  1    1  HA1
.........................................................ggugagcaaaguuucaggugug...............................  3    0  HA1
.........................................................Cgugagcaaaguuucaggugug...............................  1    1  HA1
.........................................................Ugugagcaaaguuucaggugugu..............................  4    1  HA1
.........................................................ggugGgcaaaguuucaggugugu..............................  2    1  HA1
.........................................................Cgugagcaaaguuucaggugugu..............................  22   1  HA1
.........................................................ggugagcaaaguuucaggugugu..............................  4    0  HA1
.........................................................Agugagcaaaguuucaggugugu..............................  2    1  HA1
.........................................................ggugagcaaGguuucaggugugu..............................  1    1  HA1
.........................................................ggugagcaaaguuucagguguUu..............................  1    1  HA1
..........................................................gugagcaaaguuucaggC..................................  2    1  HA1
..........................................................gugagcaaaguuucaggu..................................  39   0  HA1
..........................................................gugagcaaaguuucaggA..................................  2    1  HA1
..........................................................gugagcaaGguuucaggug.................................  2    1  HA1
..........................................................gugagcaaaguuucaggCg.................................  2    1  HA1
..........................................................gugagcaaaguuCcaggug.................................  2    1  HA1
..........................................................gugagcaaaguuucagguU.................................  6    1  HA1
..........................................................gugagcaaaguuucagguA.................................  14   1  HA1
..........................................................gugagcaaaguuucaggug.................................  144  0  HA1
..........................................................gugagcaaaguuuUaggug.................................  1    1  HA1
..........................................................gugagcaaaguuucCggug.................................  1    1  HA1
..........................................................gugagcaaaguuucGggugu................................  1    1  HA1
..........................................................gugagcaaaguuucagguUu................................  1    1  HA1
```

Star — Mature

ugagagaaugucggccuugugugcccgaaacuugacucaaccugaguuuaggaaaaggugagcaaaguuucaggugugucggguugacugaaaaaacugguuuaccagga			
....gugagcGaaguuucaggugu.................................	2	1	HA1
....gugagcaaaguuucaggCgu.................................	2	1	HA1
....gAgagcaaaguuucaggugu.................................	1	1	HA1
....gCgagcaaaguuucaggugu.................................	1	1	HA1
....gugagcaGaguuucaggugu.................................	2	1	HA1
....gugagcaaaguuucaggGgu.................................	2	1	HA1
....gugagcaaaguuucaggugC.................................	39	1	HA1
....gugagcaaagGuucaggugu.................................	1	1	HA1
....gugagAaaaguuucaggugu.................................	1	1	HA1
....gugagcaaGguuucaggugu.................................	1	1	HA1
....gugagcaaagCuucaggugu.................................	2	1	HA1
....gugagcaaaguuucaggugu.................................	312	0	HA1
....gugagcaaaguuucaggugG.................................	6	1	HA1
....gugagUaaaguuucaggugu.................................	1	1	HA1
....gugagcaaUguuucaggugu.................................	1	1	HA1
....Augagcaaaguuucaggugu.................................	1	1	HA1
....gugagcaaaUuuucaggugu.................................	1	1	HA1
....gugagcaaaguuucaggCgug................................	6	1	HA1
....gugagcaaaguuucaUgugug................................	1	1	HA1
....gugagcaaaguuucaggAgug................................	1	1	HA1
....gugUgcaaaguuucaggugug................................	1	1	HA1
....gugagcGaaguuucaggugug................................	2	1	HA1
....gugagcaCaguuucaggugug................................	1	1	HA1
....gugagcaaaguuCcaggugug................................	5	1	HA1
....gugagcaaaguuucaggugCg................................	14	1	HA1
....gugagcaaaguuucaggugGg................................	3	1	HA1
....gugGgcaaaguuucaggugug................................	4	1	HA1
....gugagcaaaguuucaggGgug................................	4	1	HA1
....gugagcaaaguCucaggugug................................	4	1	HA1
....gugagcaaaguuucagguCug................................	1	1	HA1
....gugagcaaaguuucGggugug................................	13	1	HA1
....gugagcaaaguuucaggugug................................	1224	0	HA1
....gugagcaaaguuucagguguU................................	35	1	HA1
....gugagcaaaguuucagguguC................................	1	1	HA1
....gAgagcaaaguuucaggugug................................	1	1	HA1
....gugagcaGaguuucaggugug................................	4	1	HA1
....gugagcaaagCuucaggugug................................	3	1	HA1
....gugagAaaaguuucaggugug................................	1	1	HA1
....gCgagcaaaguuucaggugug................................	3	1	HA1
....Uugagcaaaguuucaggugug................................	2	1	HA1
....guAagcaaaguuucaggugug................................	1	1	HA1
....gugagcaaGguuucaggugug................................	6	1	HA1
....gugagcaaaguuuUaggugug................................	5	1	HA1
....gugagcaUaguuucaggugug................................	2	1	HA1
....gugagcaaaguuucaggugAg................................	2	1	HA1
....gugagcaaaguuucagguguA................................	122	1	HA1
....gugagcaaCguuucaggugugu...............................	1	1	HA1
....gugagcaaaguuucaggugugG...............................	583	1	HA1
....gugagcaaaguuucagCugugu...............................	1	1	HA1
....gugagcaaagCuucaggugugu...............................	47	1	HA1
....gugagcUaaguuucaggugugu...............................	3	1	HA1
....Augagcaaaguuucaggugugu...............................	26	1	HA1
....gugagcaaaguuAcaggugugu...............................	8	1	HA1
....gugagcaaaguuucagguAugu...............................	10	1	HA1
....gugagcaaagGuucaggugugu...............................	13	1	HA1
....gugagcaaaguuucCggugugu...............................	4	1	HA1
....guAagcaaaguuucaggugugu...............................	12	1	HA1
....gugagcaaaguCucaggugugu...............................	41	1	HA1
....gugagcaaaguuucGggugugu...............................	96	1	HA1
....Uugagcaaaguuucaggugugu...............................	13	1	HA1
....gugagcaaaguuucaggugugA...............................	695	1	HA1
....gugagcaaaguuucaggGgugu...............................	21	1	HA1
....gugagcaaaguuucUggugugu...............................	19	1	HA1
....gugGgcaaaguuucaggugugu...............................	94	1	HA1
....gugagcaaaguuucagguCugu...............................	2	1	HA1
....gugagAaaaguuucaggugugu...............................	6	1	HA1
....gugagcaaGguuucaggugugu...............................	87	1	HA1
....gugagcaaaguuucagguguUu...............................	15	1	HA1
....gugagcaaaguuucaggugGgu...............................	65	1	HA1
....gugagcCaaguuucaggugugu...............................	2	1	HA1

```
                    Star                                 Mature
ugagagaaugucggccuugugugcccgaaacuuugacucaaccugaguuuaggaaaaggugagcaaaguuucaggugugucggguugacugaaaaaacugguuuaccagga
..........................................................gugagcaaaAuuucaggugugu...............................	6	1	HA1
..........................................................guUagcaaaguuucaggugugu...............................	1	1	HA1
..........................................................gugaUcaaaguuucaggugugu...............................	7	1	HA1
..........................................................gugagcaaaguAucaggugugu...............................	5	1	HA1
..........................................................gugagcaaaUuuucaggugugu...............................	2	1	HA1
..........................................................gugaAcaaaguuucaggugugu...............................	11	1	HA1
..........................................................gugagcaaaguuucaggugAgu...............................	14	1	HA1
..........................................................gugagcaaUguuucaggugugu...............................	7	1	HA1
..........................................................gugagcaaaguuGcaggugugu...............................	2	1	HA1
..........................................................gugagcaaaguuucagguguCu...............................	8	1	HA1
..........................................................gugagcaUaguuucaggugugu...............................	16	1	HA1
..........................................................gugUgcaaaguuucaggugugu...............................	7	1	HA1
..........................................................gugCgcaaaguuucaggugugu...............................	4	1	HA1
..........................................................gugagcaaaguuucagAugugu...............................	6	1	HA1
..........................................................gugagcaaaguuucaggCgugu...............................	84	1	HA1
..........................................................gugagcaaaguuuGaggugugu...............................	1	1	HA1
..........................................................gugagcaaaguuCcaggugugu...............................	59	1	HA1
..........................................................gAgagcaaaguuucaggugugu...............................	13	1	HA1
..........................................................gugagcaCaguuucaggugugu...............................	4	1	HA1
..........................................................gugagcaaaguuucagguUugu...............................	2	1	HA1
..........................................................gugagcGaaguuucaggugugu...............................	57	1	HA1
..........................................................gugagcaaaguuucaggAgugu...............................	16	1	HA1
..........................................................gugagcaGaguuucaggugugu...............................	77	1	HA1
..........................................................gugagcaaaguuucaggugugu...............................	19632	0	HA1
..........................................................gugagcaaaguuucaggugCgu...............................	63	1	HA1
..........................................................gugagcaaaguuucaAgugugu...............................	17	1	HA1
..........................................................gugagcaaaguuuUaggugugu...............................	20	1	HA1
..........................................................gugagcaaaCuuucaggugugu...............................	2	1	HA1
..........................................................gCgagcaaaguuucaggugugu...............................	50	1	HA1
..........................................................gugagcaaaguuucagUugugu...............................	1	1	HA1
..........................................................gugagUaaaguuucaggugugu...............................	25	1	HA1
..........................................................gugagGaaaguuucaggugugu...............................	4	1	HA1
..........................................................gugagcaaaguuucagguguAu...............................	22	1	HA1
..........................................................gugagcaaaguuucaCgugugu...............................	1	1	HA1
..........................................................gGgagcaaaguuucaggugugu...............................	2	1	HA1
..........................................................gugagcaaaguuucaUgugugu...............................	5	1	HA1
..........................................................gugagcaaaguuucaggugugAc..............................	1	1	HA1
..........................................................gugagcaaaguuucagguguguc..............................	53	0	HA1
..........................................................gugagcaaaguuCcagguguguc..............................	2	1	HA1
..........................................................gugagcGaaguuucagguguguc..............................	1	1	HA1
..........................................................gugagcaaaguuucaggugCguc..............................	1	1	HA1
..........................................................gugagcaaaguuucagguguguG..............................	25	1	HA1
..........................................................gCgagcaaaguuucagguguguc..............................	1	1	HA1
..........................................................gugagcaaaguuucagguguguGg.............................	1	1	HA1
...........................................................ugagcaaaguuucaggugu.................................	1	0	HA1
...........................................................ugagcaaaguuucaggugug................................	1	0	HA1
...........................................................ugagcaaaguuucaggugugu...............................	10	0	HA1
...........................................................ugagcaaaguuucaggugugA...............................	1	1	HA1
...........................................................Cgagcaaaguuucaggugugu...............................	1	1	HA1
............................................................gagcaaaguuucGggugugu...............................	1	1	HA1
............................................................gagcaaaguuucaggugugu...............................	13	0	HA1
.............................................................agcaaaguuucaggugugu...............................	1	0	HA1
..............................................................gcaaaguuucaggugugu...............................	3	0	HA1

...................Cgugcccgaaacuuugacucaa......................................................................	1	1	LA5
...................Cgugcccgaaacuuugacucaacc....................................................................	1	1	LA5
...................Cgugcccgaaacuuugacucaaccu...................................................................	2	1	LA5
....................gugcccgGaacuuugacu.........................................................................	1	1	LA5
....................gugcccgaaacuAugacu.........................................................................	1	1	LA5
....................gugcccgaaacuuugacu.........................................................................	107	0	LA5
....................gugcccgaaacuuugacC.........................................................................	17	1	LA5
....................gugcccgaaacuuugacA.........................................................................	11	1	LA5
....................gugcccgaaacuuugacG.........................................................................	2	1	LA5
....................gugcccgaaacuuugacuU........................................................................	18	1	LA5
....................gugcccgaaacuuugacuA........................................................................	7	1	LA5
....................gugcccgaaacuuugacuc........................................................................	132	0	LA5
....................gugcccgaaacuuCgacuca.......................................................................	4	1	LA5
....................gugcccgaaaUuuugacuca.......................................................................	1	1	LA5
....................gugcccgaaacuuugacuca.......................................................................	172	0	LA5
....................gugcccgaaacuCugacuca.......................................................................	1	1	LA5
```

Star **Mature**

ugagagaaugucggccuugugugcccgaaacuuugacucaaccugaguuuaggaaaaggugagcaaaguuucaggugugucgguugacugaaaaaacugguuuaccagga			
....................gugcccgaaacuuugacucG..	4	1	LA5
....................gugcccgUaacuuugacuca..	1	1	LA5
....................Augcccgaaacuuugacuca..	1	1	LA5
....................gugcccgaaacuuugacucU..	3	1	LA5
....................gugcccgaaacuuugacCca..	1	1	LA5
....................guAcccgaaacuuugacuca..	2	1	LA5
....................gugcccgaGacuuugacuca..	1	1	LA5
....................gugcccgGaacuuugacuca..	1	1	LA5
....................gugcUcgaaacuuugacucaa...	1	1	LA5
....................gugcccgaaacuuugacucGa...	2	1	LA5
....................gugcccgaaacuuugacGcaa...	1	1	LA5
....................gugcccgaaacuuugacucaG...	5	1	LA5
....................gugcccgaaacuuugacucaU...	2	1	LA5
....................gugcccgaaacuuugacCcaa...	2	1	LA5
....................gugcccgaaacuuugacucaa...	266	0	LA5
....................gugcccgaaacuuCgacucaa...	2	1	LA5
....................gugUccgaaacuuugacucaa...	3	1	LA5
....................gugcccgaaacuuugacuAaac..	1	1	LA5
....................gugcccgaaacuuugacucaaU..	27	1	LA5
....................gugcccgaaacuuugacucaac..	312	0	LA5
....................gugcccgaaacuuugacucaaA..	1	1	LA5
....................gugcccgaaacuuugacucGac..	2	1	LA5
....................Uugcccgaaacuuugacucaac..	1	1	LA5
....................gugcccgaGacuuugacucaac..	1	1	LA5
....................gugcccgaaacuCugacucaac..	1	1	LA5
....................gugcccgaaacuuuAacucaac..	2	1	LA5
....................gugcccgGaacuuugacucaac..	1	1	LA5
....................gCgcccgaaacuuugacucaac..	3	1	LA5
....................gugcccgaaacuuugacucaGc..	3	1	LA5
....................gugcccgaaacuuugacCcaacc...	1	1	LA5
....................gAgcccgaaacuuugacucaacc...	2	1	LA5
....................gugcccgaaGcuuugacucaacc...	1	1	LA5
....................gugcccgaaacuuugacucGacc...	5	1	LA5
....................gugcccgaaacuuugacucaUcc...	3	1	LA5
....................gugcccgaaacuuugacucaacA...	49	1	LA5
....................guUcccgaaacuuugacucaacc...	1	1	LA5
....................gugcccgaaacuuugacucaacc...	451	0	LA5
....................gugcccgaaacuuugacAcaacc...	2	1	LA5
....................gugcccgaaacuuugUcucaacc...	1	1	LA5
....................gugcccgaaacuuugacucaacU...	110	1	LA5
....................gugcccgaaacuuugaUucaacc...	1	1	LA5
....................gugcccgGaacuuugacucaacc...	1	1	LA5
....................gugccUgaaacuuugacucaacc...	1	1	LA5
....................gugcccgaaacuuugacucaGcc...	1	1	LA5
....................gugcccgaaacuuCgacucaacc...	1	1	LA5
....................gugcccgaaacuCugacucaacc...	1	1	LA5
....................gugcccgaaacuuugacucaaccC..	633	1	LA5
....................gugcccgaaacuCugacucaaccu..	11	1	LA5
....................Uugcccgaaacuuugacucaaccu..	2	1	LA5
....................gugcccgUaacuuugacucaaccu..	2	1	LA5
....................gugcccgaaacuuugacCcaaccu..	20	1	LA5
....................gugcccgaaacuuugacucaaccA..	347	1	LA5
....................gugcccgaaacuuugaAucaaccu..	1	1	LA5
....................gugcccgaaacuuugacucaaccG..	122	1	LA5
....................gugccGgaaacuuugacucaaccu..	1	1	LA5
....................gCgcccgaaacuuugacucaaccu..	7	1	LA5
....................gugcccAaaacuuugacucaaccu..	4	1	LA5
....................gugcccgaaacuAugacucaaccu..	1	1	LA5
....................gugcccgGaacuuugacucaaccu..	18	1	LA5
....................gugAccgaaacuuugacucaaccu..	1	1	LA5
....................gugccAgaaacuuugacucaaccu..	1	1	LA5
....................gugcccgaGacuuugacucaaccu..	11	1	LA5
....................gugcccgaaacuuCgacucaaccu..	13	1	LA5
....................gugcccgaaacuuugacucaaUcu..	6	1	LA5
....................gugcccgaaacuuugaUucaaccu..	4	1	LA5
....................gugcccgCaacuuugacucaaccu..	1	1	LA5
....................gugUccgaaacuuugacucaaccu..	5	1	LA5
....................gugcccgaaUcuuugacucaaccu..	1	1	LA5
....................gugcccgaaacGuugacucaaccu..	1	1	LA5
....................gugcccgaaacuuugacAcaaccu..	7	1	LA5

```
                    Star                                  Mature
ugagagaaugucggccuugugugcccgaaacuuugacucaaccugaguuuaggaaaaggugagcaaaguuucaggugugucggguugacugaaaaaacugguuuaccagga
....................gugcccgaaacuuugacucaaccu...................................................................  5020   0   LA5
....................gAgcccgaaacuuugacucaaccu...................................................................  2      1   LA5
....................gugcccgaaacuuugacucaacUu...................................................................  6      1   LA5
....................gugcccgaaacuuugacucGaccu...................................................................  11     1   LA5
....................gugcccgaaacuuugUcucaaccu...................................................................  14     1   LA5
....................gugcUcgaaacuuugacucaaccu...................................................................  8      1   LA5
....................gugcccgaaacuuugacucaaAcu...................................................................  1      1   LA5
....................gugcccgaaaUuuugacucaaccu...................................................................  2      1   LA5
....................gugcccgaaacAuugacucaaccu...................................................................  3      1   LA5
....................Augcccgaaacuuugacucaaccu...................................................................  5      1   LA5
....................gugcccgaaacuuugacucaCccu...................................................................  1      1   LA5
....................gugcccgaaacuuugacuUaaccu...................................................................  2      1   LA5
....................guAcccgaaacuuugacucaaccu...................................................................  1      1   LA5
....................gugcccgaaacuuugacucCaccu...................................................................  1      1   LA5
....................gugcccgaaacuuugacucaaGcu...................................................................  1      1   LA5
....................gugcccgaaacuuugacucUaccu...................................................................  1      1   LA5
....................gugccUgaaacuuugacucaaccu...................................................................  8      1   LA5
....................gugcccgaaacuuugacucaGccu...................................................................  8      1   LA5
....................gugcccgaCacuuugacucaaccu...................................................................  1      1   LA5
....................gugcccgaaGcuuugacucaaccu...................................................................  20     1   LA5
....................gugcccgaaacuuuAacucaaccu...................................................................  1      1   LA5
....................gugcccgaaacuuugCcucaaccu...................................................................  1      1   LA5
....................gugcccgaaacuuugacucaaccuU..................................................................  81     1   LA5
....................gugcccgaaacuuCgacucaaccug..................................................................  1      1   LA5
....................gugcccgaaacuuugacucaaccuC..................................................................  9      1   LA5
....................gugcccgaaacuuugacucaaccuA..................................................................  6      1   LA5
....................gugcccgaaacuuugacucaaccug..................................................................  1      0   LA5
....................gugcccgaaacuuugacucaaccuAa.................................................................  2      1   LA5
.....................ugcccgaaacuuugacuc........................................................................  1      0   LA5
.....................ugcccgaaacuuugacuca.......................................................................  4      0   LA5
.....................ugcccgaaacuuugacucaa......................................................................  4      0   LA5
.....................ugccAgaaacuuugacucaa......................................................................  1      1   LA5
.....................ugcccgaaacuuugacucaac.....................................................................  3      0   LA5
.....................ugcccgaaacuuugacucaacU....................................................................  1      1   LA5
.....................ugcccgaaacuuugacucaacA....................................................................  2      1   LA5
.....................ugcccgGaacuuugacucaacc....................................................................  1      1   LA5
.....................ugcccgaaaUuuugacucaacc....................................................................  1      1   LA5
.....................ugcccgaaacuuugacucaacc....................................................................  9      0   LA5
.....................ugcccgaaacuuugacucaaccu...................................................................  126    0   LA5
.....................ugcccgaaacuuugacucaaccC...................................................................  17     1   LA5
.....................ugcccgaaacuuugacucaaccA...................................................................  8      1   LA5
.....................ugcccgGaacuuugacucaaccu...................................................................  1      1   LA5
.....................ugcccgaGacuuugacucaaccu...................................................................  1      1   LA5
.....................ugcAcgaaacuuugacucaaccu...................................................................  1      1   LA5
.....................Cgcccgaaacuuugacucaaccu...................................................................  1      1   LA5
.....................ugcccgaaacuuugacucaaccuU..................................................................  6      1   LA5
......................gcccgaaacuuugacucaaccu...................................................................  2      0   LA5
.......................cccgaaacuuugacucaaccC...................................................................  1      1   LA5
........................ccgaaacuuugacucaacc....................................................................  1      0   LA5
........................ccgaaacuuugacucaaccu...................................................................  5      0   LA5
..........................gaaacuuugacucaaccA...................................................................  2      1   LA5
..........................gaaacuuugacucaaccu...................................................................  3      0   LA5
.........................................................ggugagcaaaguuucaggug..................................  1      0   LA5
.........................................................ggugagcaaaguuucaggugC.................................  1      1   LA5
.........................................................Cgugagcaaaguuucaggugugu...............................  15     1   LA5
..........................................................gugagcaaaguuucaggu...................................  10     0   LA5
..........................................................gugagcaaaguuucaggug..................................  17     0   LA5
..........................................................gugagcaaaguuucagguA..................................  1      1   LA5
..........................................................gugagcGaaguuucaggugu.................................  1      1   LA5
..........................................................gugagcaaaguuucaggAgu.................................  1      1   LA5
..........................................................gugagcaaaguuucaggugC.................................  8      1   LA5
..........................................................gugagcaGaguuucaggugu.................................  2      1   LA5
..........................................................gugagcaaaguuucaggugG.................................  1      1   LA5
..........................................................Uugagcaaaguuucaggugu.................................  3      1   LA5
..........................................................gugagcaaaguuucaggugu.................................  77     0   LA5
..........................................................gugagUaaaguuucaggugu.................................  1      1   LA5
..........................................................gugagcaaaguuucagguAug................................  1      1   LA5
..........................................................Augagcaaaguuucaggugug................................  2      1   LA5
..........................................................gugagcaaaguuucagguguA................................  23     1   LA5
..........................................................gugagcaaaguuucaggCgug................................  2      1   LA5
```

```
                    Star                                      Mature
ugagagaaugucggccuugugugcccgaaacuuugacucaaccugaguuuaggaaaaggugagcaaaguuucaggugugucggguugacugaaaaaacugguuuaccagga
..........................................................gugagcaaaguuucagguguU................................   7      1    LA5
..........................................................gugagcaaaguuCcaggugug................................   2      1    LA5
..........................................................gugGgcaaaguuucaggugug................................   1      1    LA5
..........................................................gugagcGaaguuucaggugug................................   1      1    LA5
..........................................................gugagcaaaguuucagguguC................................   1      1    LA5
..........................................................gugagcaaaguuucGggugug................................   1      1    LA5
..........................................................gugagcaaaguuucaggugug................................   251    0    LA5
..........................................................gugagcaaGguuucaggugug................................   2      1    LA5
..........................................................gugagcaaaguuucaggugGg................................   1      1    LA5
..........................................................gugagcaaUguuucaggugug................................   1      1    LA5
..........................................................gugagcaGaguuucaggugug................................   1      1    LA5
..........................................................gugagcaaaguuuAaggugug................................   1      1    LA5
..........................................................gugagcaaagAuucaggugug................................   1      1    LA5
..........................................................gugagcaaaguuucaggugugG...............................   105    1    LA5
..........................................................gugagcaCaguuucaggugugu...............................   1      1    LA5
..........................................................gugagcaaaguuucagAugugu...............................   7      1    LA5
..........................................................gugagcaaaguuucaggugugA...............................   238    1    LA5
..........................................................gugagcaaaguuuAaggugugu...............................   1      1    LA5
..........................................................gugagcaaaguuucaggugCgu...............................   12     1    LA5
..........................................................gugagcaaaguuucaAgugugu...............................   4      1    LA5
..........................................................gugagcUaaguuucaggugugu...............................   1      1    LA5
..........................................................gugagcaaaguuucaggugGgu...............................   8      1    LA5
..........................................................gugagcaaaguuucagguguCu...............................   1      1    LA5
..........................................................gugaAcaaaguuucaggugugu...............................   10     1    LA5
..........................................................gugagcaaaguCucaggugugu...............................   19     1    LA5
..........................................................guAagcaaaguuucaggugugu...............................   6      1    LA5
..........................................................gugagUaaaguuucaggugugu...............................   9      1    LA5
..........................................................gugagcGaaguuucaggugugu...............................   17     1    LA5
..........................................................gugagcaaaguuCcaggugugu...............................   11     1    LA5
..........................................................gugagcaaaguuucaggugugu...............................   5980   0    LA5
..........................................................gugagcaaUguuucaggugugu...............................   1      1    LA5
..........................................................gugagcaaaguuucaggGgugu...............................   5      1    LA5
..........................................................gugUgcaaaguuucaggugugu...............................   5      1    LA5
..........................................................gugagcaaaguuucagguguUu...............................   3      1    LA5
..........................................................gugagcaaaguuucaggugAgu...............................   3      1    LA5
..........................................................Augagcaaaguuucaggugugu...............................   6      1    LA5
..........................................................gugagcaaaguuucaggAgugu...............................   4      1    LA5
..........................................................gugagcaaaguuucaggCgugu...............................   20     1    LA5
..........................................................gCgagcaaaguuucaggugugu...............................   15     1    LA5
..........................................................Cugagcaaaguuucaggugugu...............................   1      1    LA5
..........................................................gugagcaaGguuucaggugugu...............................   21     1    LA5
..........................................................gugagcaaaguuuUaggugugu...............................   6      1    LA5
..........................................................gugagAaaaguuucaggugugu...............................   1      1    LA5
..........................................................gugagcaaagAuucaggugugu...............................   1      1    LA5
..........................................................gugagcCaaguuucaggugugu...............................   1      1    LA5
..........................................................gugagcaaaguAucaggugugu...............................   2      1    LA5
..........................................................gugagcaaagGuucaggugugu...............................   2      1    LA5
..........................................................gugagcaaaguuucagguguAu...............................   6      1    LA5
..........................................................gugagcaaaguuucagguCugu...............................   2      1    LA5
..........................................................gugagcaaaguuucGggugugu...............................   30     1    LA5
..........................................................gugagcaGaguuucaggugugu...............................   22     1    LA5
..........................................................gugagcaaagCuucaggugugu...............................   19     1    LA5
..........................................................gugagcaaaAuuucaggugugu...............................   1      1    LA5
..........................................................gAgagcaaaguuucaggugugu...............................   1      1    LA5
..........................................................gugagcaaaguuucagguAugu...............................   2      1    LA5
..........................................................Uugagcaaaguuucaggugugu...............................   4      1    LA5
..........................................................gugagcaaaguuAcaggugugu...............................   2      1    LA5
..........................................................gugGgcaaaguuucaggugugu...............................   23     1    LA5
..........................................................gugagcaaaguuucUggugugu...............................   4      1    LA5
..........................................................gugagcaaaguuGcaggugugu...............................   2      1    LA5
..........................................................gugagcaaaguuucagguguguG..............................   7      1    LA5
..........................................................gugagcaaaguuucagguguguc..............................   20     0    LA5
..........................................................gugagcaaagCuucagguguguc..............................   1      1    LA5
...........................................................ugagcaaaguuucaggugC.................................   1      1    LA5
...........................................................ugagcaaaguuucaggugugu...............................   5      0    LA5
............................................................gagcaaaguuucaggugugu...............................   3      0    LA5
.............................................................agcaaaguuucaggugugA...............................   1      1    LA5

..................Uugugcccgaaacuuugacucaaccu...................................................................   1      1    H01
...................ugugcccgaaacuuugacucaaccu...................................................................   1      0    H01
```

```
                    Star                                      Mature
ugagagaaugucggccuugugugcccgaaacuuugacucaaccugaguuuaggaaaaggugagcaaaguuucaggugugucggguugacugaaaaaacugguuuaccagga
....................gugcccgaaacuuugacu.........................................................................	1	0	H01
....................gugcccgaaacuuugacuc........................................................................	3	0	H01
....................gugcccgaaacuuugacuca.......................................................................	9	0	H01
....................gugcccgaaacuuugacucaC......................................................................	1	1	H01
....................gugcccgaaacuuugacuAaa......................................................................	1	1	H01
....................gugcccgaaacuuugacucaU......................................................................	1	1	H01
....................gugcccgaaacuuugacucaa......................................................................	14	0	H01
....................gugcccgaaacuuugacucaac.....................................................................	21	0	H01
....................gugcccgaaacuuugacuAaacc....................................................................	1	1	H01
....................gugcccgaaacuuugacucaacU....................................................................	1	1	H01
....................gugcccgaaacuuugacucaacc....................................................................	58	0	H01
....................gugcccgaaacuuugacucaacA....................................................................	1	1	H01
....................gugcccgaaCcuuugacucaacc....................................................................	1	1	H01
....................gugcccgaaacuuugaUucaaccu...................................................................	3	1	H01
....................gugccUgaaacuuugacucaaccu...................................................................	1	1	H01
....................gugcccgaaGcuuugacucaaccu...................................................................	2	1	H01
....................gCgcccgaaacuuugacucaaccu...................................................................	1	1	H01
....................gugcccgaaacuuugacucaaUcu...................................................................	1	1	H01
....................gugcccgaaacuuugUcucaaccu...................................................................	4	1	H01
....................gugcccgaaacuuugacucaaccC...................................................................	5	1	H01
....................gugUccgaaacuuugacucaaccu...................................................................	1	1	H01
....................gugcccgaaacuuugacucaaccu...................................................................	628	0	H01
....................gugcccgaaacuuugacuUaaccu...................................................................	1	1	H01
....................gugcccgaaaUuuugacucaaccu...................................................................	1	1	H01
....................gugcccgaaacuuAgacucaaccu...................................................................	1	1	H01
....................gGgcccgaaacuuugacucaaccu...................................................................	1	1	H01
....................gugcccgaaacuuugacucaaccA...................................................................	2	1	H01
....................gugcccgaUacuuugacucaaccu...................................................................	1	1	H01
....................gugcccgaaacuuugacucaaccuA..................................................................	1	1	H01
....................gugcccgaaacuuugacucaaccuU..................................................................	6	1	H01
....................gugcccgaaacAuugacucaaccug..................................................................	1	1	H01
.....................ugcccgaaacuuugacuc........................................................................	1	0	H01
.....................ugcccgaaacuuugacucaa......................................................................	1	0	H01
.....................ugcccgaaacuuugacucaacc....................................................................	3	0	H01
.....................ugcccgaaacuuugacucaaccu...................................................................	35	0	H01
.....................ugcccgaaacAuugacucaaccu...................................................................	1	1	H01
.....................ugcccgaaacuuugacucaaccuU..................................................................	1	1	H01
.....................ugcccgaaaUuuugacucaaccug..................................................................	1	1	H01
......................gcccgaaacuuugacucaaccu...................................................................	1	0	H01
........................ccgaaacuuugacucaaccu...................................................................	1	0	H01
.........................cgaaacuuugacucaaccu...................................................................	2	0	H01
....................................................gaaaaggugagcaaaguuucaggugug................................	1	0	H01
.....................................................aaaGggugagcaaaguuucaggugugu...............................	1	1	H01
......................................................aaGggugagcaaaguuucaggugugu...............................	7	1	H01
.......................................................aGggugagcaaaguuucaggu...................................	1	1	H01
.......................................................aGggugagcaaaguuucaggugu.................................	1	1	H01
.......................................................aaUgugagcaaaguuucaggugugu...............................	1	1	H01
.......................................................aaCgugagcaaaguuucaggugugu...............................	2	1	H01
.......................................................aGggugagcaaaguuucaggugugu...............................	17	1	H01
........................................................aggugagcaaaguuucaggugu.................................	1	0	H01
........................................................Uggugagcaaaguuucaggugug................................	1	1	H01
........................................................aAgugagcaaaguuucaggugugu...............................	3	1	H01
........................................................Uggugagcaaaguuucaggugugu...............................	4	1	H01
........................................................Gggugagcaaaguuucaggugugu...............................	2	1	H01
........................................................aCgugagcaaaguuucaggugugu...............................	3	1	H01
........................................................aUgugagcaaaguuucaggugugu...............................	7	1	H01
........................................................aggugagcaaaguuucaggugugu...............................	3	0	H01
.........................................................ggugagcaaaguuucagg....................................	3	0	H01
.........................................................ggugagcaaaguuucagA....................................	1	1	H01
.........................................................ggugagcaaaguuucaggu...................................	5	0	H01
.........................................................Ugugagcaaaguuucaggu...................................	3	1	H01
.........................................................Cgugagcaaaguuucaggu...................................	1	1	H01
.........................................................Ugugagcaaaguuucaggug..................................	1	1	H01
.........................................................ggugagcaaaguuucaggug..................................	3	0	H01
.........................................................Ugugagcaaaguuucaggugu.................................	4	1	H01
.........................................................ggugagcaaaguuucaggugu.................................	76	0	H01
.........................................................ggugagcaaaguuucagguUu.................................	1	1	H01
.........................................................ggugagcaaaguuuUaggugu.................................	1	1	H01
.........................................................Ugugagcaaaguuucaggugug................................	18	1	H01
.........................................................ggugagcaaaguuucaggugug................................	152	0	H01
```

Star Mature

ugagagaaugucggccuugugugcccgaaacuuugacucaaccugaguuuaggaaaaggugagcaaaguuucaggugugucggguugacugaaaaaacugguuuaccagga			
....ggugagcaaaguuucaggugCg.................................	1	1	H01
....ggugagcaaaguuucagguguU................................	27	1	H01
....Cgugagcaaaguuucaggugug................................	1	1	H01
....Agugagcaaaguuucaggugug................................	1	1	H01
....ggugagcaaaguuucGggugug................................	1	1	H01
....ggugagcaaaguuucagguguA................................	3	1	H01
....Ugugagcaaaguuucaggugugu...............................	402	1	H01
....ggugagcaaaguuucaggugugu...............................	133	0	H01
....ggugagcaaaguuucagguguAu...............................	1	1	H01
....ggugagcaaaguuucaggugugA...............................	12	1	H01
....Agugagcaaaguuucaggugugu...............................	15	1	H01
....ggugGgcaaaguuucaggugugu...............................	1	1	H01
....ggugagcaaaguuucagguguUu...............................	6	1	H01
....ggugagcaaaguuucagguguCu...............................	1	1	H01
....Cgugagcaaaguuucaggugugu...............................	23	1	H01
....gugagcaaaguuucCggu...................................	16	1	H01
....gugagcaaaguuGcaggu...................................	1	1	H01
....gugagcaGaguuucaggu...................................	1	1	H01
....gugagcaaaCuuucaggu...................................	1	1	H01
....gugGgcaaaguuucaggu...................................	1	1	H01
....gugagcaaaguuucaggC...................................	8	1	H01
....gugagcaaagCuucaggu...................................	1	1	H01
....gAgagcaaaguuucaggu...................................	2	1	H01
....gugagcaaaguuucGggu...................................	8	1	H01
....gugagcaaaguuucaggA...................................	50	1	H01
....gCgagcaaaguuucaggu...................................	1	1	H01
....gugagcaaaguuucUggu...................................	3	1	H01
....gugagcaaaguuucaggu...................................	3657	0	H01
....gugagcaaaguuuUaggu...................................	1	1	H01
....gugaCcaaaguuucaggu...................................	1	1	H01
....gugaAcaaaguuucaggu...................................	2	1	H01
....gugagcaaaguuCcaggu...................................	1	1	H01
....gugagcaaaguuucagguC..................................	4	1	H01
....gugagcaaaguuucaggug..................................	939	0	H01
....gugagcaaaguuuUaggug..................................	6	1	H01
....gugaAcaaaguuucaggug..................................	1	1	H01
....gugagcaaaguuucaggCg..................................	2	1	H01
....gugagcaaaguuucaUgug..................................	1	1	H01
....gugagcaaaguuucCggug..................................	3	1	H01
....gugagcaaaguuucUggug..................................	3	1	H01
....gugagcaaaguuucGggug..................................	4	1	H01
....gugagcaaaguuucaAgug..................................	1	1	H01
....gugagcaaaguuucagguA..................................	27	1	H01
....gugagcaaaguuucagguU..................................	32	1	H01
....gugagAaaaguuucaggugu.................................	1	1	H01
....gAgagcaaaguuucaggugu.................................	5	1	H01
....gugagcaaaguuuGaggugu.................................	2	1	H01
....Uugagcaaaguuucaggugu.................................	1	1	H01
....gugagcGaaguuucaggugu.................................	1	1	H01
....gugagcaaaguuucaggugu.................................	6877	0	H01
....gugagUaaaguuucaggugu.................................	5	1	H01
....gugagcaaaguuucaggGgu.................................	2	1	H01
....gugagcaaagCuucaggugu.................................	1	1	H01
....gugagcaaaguuuUaggugu.................................	15	1	H01
....gugagcaaaAuuucaggugu.................................	1	1	H01
....gGgagcaaaguuucaggugu.................................	1	1	H01
....gugagcaaaguuucUggugu.................................	6	1	H01
....guCagcaaaguuucaggugu.................................	1	1	H01
....gugagcaaaguuucaUgugu.................................	2	1	H01
....gugagcaGaguuucaggugu.................................	2	1	H01
....gugagcaaaguuCcaggugu.................................	4	1	H01
....gugagcaaaguuucagguUu.................................	2	1	H01
....gugagcaaaguuucCggugu.................................	1	1	H01
....gugagcaaaguuucaggugC.................................	5	1	H01
....gugagcaaagAuucaggugu.................................	1	1	H01
....gugagcaaaguuucagAugu.................................	1	1	H01
....gugagcaaaguuucaAgugu.................................	1	1	H01
....gugagcaUaguuucaggugu.................................	2	1	H01
....gugagcaaaguuucagguCu.................................	1	1	H01
....gugagcaaaUuuucaggugu.................................	1	1	H01

```
                    Star                                  Mature
ugagagaaugucggccuugugugcccgaaacuuugacucaaccugaguuuaggaaaaggugagcaaaguuucaggugugucggguugacugaaaaaacugguuuaccagga
..........................................................gugagcaaaguuucaggugG.................................    3      1    H01
..........................................................gugagcaaagGuucaggugu.................................    6      1    H01
..........................................................gugagcaaaguCucaggugu.................................    4      1    H01
..........................................................Augagcaaaguuucaggugu.................................    1      1    H01
..........................................................gugaAcaaaguuucaggugu.................................    2      1    H01
..........................................................gugagcaaaguuucGggugu.................................    14     1    H01
..........................................................gugagcaaaCuuucaggugu.................................    4      1    H01
..........................................................gugagcaaaguuucaggAgu.................................    1      1    H01
..........................................................gugagcaGaguuucaggugug................................    3      1    H01
..........................................................gugagcaaaguuCcaggugug................................    7      1    H01
..........................................................gugagcaaaUuuucaggugug................................    1      1    H01
..........................................................gGgagcaaaguuucaggugug................................    7      1    H01
..........................................................gugagcaaGguuucaggugug................................    1      1    H01
..........................................................gugagcaaaguuucaggGgug................................    10     1    H01
..........................................................gugagcaaagGuucaggugug................................    18     1    H01
..........................................................gugagcaaaguuGcaggugug................................    2      1    H01
..........................................................guUagcaaaguuucaggugug................................    1      1    H01
..........................................................gugagcaaaguuuGaggugug................................    4      1    H01
..........................................................gugagcUaaguuucaggugug................................    1      1    H01
..........................................................gugagcaaaguuucaAgugug................................    3      1    H01
..........................................................gugagcaaaguuucaCgugug................................    2      1    H01
..........................................................gugagcaCaguuucaggugug................................    2      1    H01
..........................................................gugagcaaaguuucCggugug................................    2      1    H01
..........................................................gugagcaaaguuucagguAug................................    2      1    H01
..........................................................gugagcaaaguuucagCugug................................    1      1    H01
..........................................................gugagcaaaguuucaggugU................................    2223   1    H01
..........................................................gugagcaaaguuAcaggugug................................    2      1    H01
..........................................................Cugagcaaaguuucaggugug................................    1      1    H01
..........................................................gugagcaaaguCucaggugug................................    3      1    H01
..........................................................gugagcaaagCuucaggugug................................    2      1    H01
..........................................................gugagcaaaguuucUggugug................................    7      1    H01
..........................................................gugagcaaaguuucaggugC................................    14     1    H01
..........................................................guCagcaaaguuucaggugug................................    1      1    H01
..........................................................gugagcaaaguuuAaggugug................................    1      1    H01
..........................................................guAagcaaaguuucaggugug................................    2      1    H01
..........................................................gugagUaaaguuucaggugug................................    15     1    H01
..........................................................gugagcaaaguuucagAugug................................    2      1    H01
..........................................................gugagcaaaguuucagguguA................................    162    1    H01
..........................................................gugaCcaaaguuucaggugug................................    1      1    H01
..........................................................gugagcaaaCuuucaggugug................................    11     1    H01
..........................................................gugagcaaaguuucagguCug................................    6      1    H01
..........................................................gugUgcaaaguuucaggugug................................    2      1    H01
..........................................................gugagcaUaguuucaggugug................................    1      1    H01
..........................................................gCgagcaaaguuucaggugug................................    1      1    H01
..........................................................gugagcaaaguuuUaggugug................................    14     1    H01
..........................................................gugagcaaaguuucaggugAg................................    8      1    H01
..........................................................gugagcaaaguuucaggugug................................    22180  0    H01
..........................................................gugagcaaaguuucaggCgug................................    2      1    H01
..........................................................gugagcaaaguuucagUugug................................    5      1    H01
..........................................................gugaAcaaaguuucaggugug................................    10     1    H01
..........................................................gugagcaaaguuucagguUug................................    3      1    H01
..........................................................Uugagcaaaguuucaggugug................................    6      1    H01
..........................................................gugagcaaaguuucGggugug................................    37     1    H01
..........................................................gugGgcaaaguuucaggugug................................    3      1    H01
..........................................................gugagcaaaguuucaUgugug................................    3      1    H01
..........................................................gugagAaaaguuucaggugug................................    1      1    H01
..........................................................gugagcaaaguuucaggugCg................................    14     1    H01
..........................................................gAgagcaaaguuucaggugug................................    12     1    H01
..........................................................gugaUcaaaguuucaggugug................................    2      1    H01
..........................................................gugaCcaaaguuucaggugugu...............................    29     1    H01
..........................................................gugagcaaaguuucagguCugu...............................    25     1    H01
..........................................................gugagcaaaCuuucaggugugu...............................    133    1    H01
..........................................................gugagcaaGguuucaggugugu...............................    18     1    H01
..........................................................gugagcaaaguuucaggugugG...............................    141    1    H01
..........................................................gugagcaaaguuucaggugugA...............................    3760   1    H01
..........................................................gugagcaaaguuucagguUugu...............................    48     1    H01
..........................................................gugagcaaaUuuucaggugugu...............................    21     1    H01
..........................................................gugagcaaaguuucaUgugugu...............................    61     1    H01
..........................................................gugagcaaaguuuAggugugu...............................    29     1    H01
..........................................................guCagcaaaguuucaggugugu...............................    34     1    H01
```

Star **Mature**

ugagagaaugucggccuugugugcccgaaacuuugacucaaccugaguuuaggaaaaggugagcaaaguuucaggugugucggguugacugaaaaaacugguuuaccagga			
....gugagcaaCguuucaggugugu...............................	7	1	H01
....gugagcCaaguuucaggugugu...............................	2	1	H01
....Uugagcaaaguuucaggugugu...............................	90	1	H01
....gugagcaaaguuucaggAgugu...............................	8	1	H01
....gugagcaaaguuGcaggugugu...............................	33	1	H01
....gugagcaaagGuucaggugugu...............................	170	1	H01
....gugagcaaaguuucagCugugu...............................	72	1	H01
....gugagcaaaguuucagguAugu...............................	28	1	H01
....gugagcaaaguuucaggGgugu...............................	63	1	H01
....gugagcaaaguuucaggugGgu...............................	26	1	H01
....guUagcaaaguuucaggugugu...............................	34	1	H01
....gugagcaaaguAucaggugugu...............................	3	1	H01
....gugagcaaaguuucUggugugu...............................	276	1	H01
....gugaAcaaaguuucaggugugu...............................	89	1	H01
....gugagcaaaguuucaggugAgu...............................	10	1	H01
....gugagcaaaguuucagUugugu...............................	68	1	H01
....Cugagcaaaguuucaggugugu...............................	8	1	H01
....gugagcaaaguuucaggugCgu...............................	24	1	H01
....gugagAaaaguuucaggugugu...............................	23	1	H01
....gugUgcaaaguuucaggugugu...............................	48	1	H01
....gGgagcaaaguuucaggugugu...............................	94	1	H01
....gugagcaUaguuucaggugugu...............................	74	1	H01
....gugagUaaaguuucaggugugu...............................	231	1	H01
....gugCgcaaaguuucaggugugu...............................	21	1	H01
....gugagcaaaguuucCggugugu...............................	49	1	H01
....gugagcaaaAuuucaggugugu...............................	11	1	H01
....gugaUcaaaguuucaggugugu...............................	30	1	H01
....gugagcaaaguCucaggugugu...............................	31	1	H01
....gugagcaGaguuucaggugugu...............................	53	1	H01
....gugagcaaaguuucagguguCu...............................	155	1	H01
....gugagcaaUguuucaggugugu...............................	19	1	H01
....gugagcaaaguuAcaggugugu...............................	14	1	H01
....Augagcaaaguuucaggugugu...............................	7	1	H01
....gugagcaaaguuucGggugugu...............................	696	1	H01
....gugagcaaagCuucaggugugu...............................	32	1	H01
....gugagcaaaguuucaggugugu...............................	391286	0	H01
....gugagcGaaguuucaggugugu...............................	24	1	H01
....gugGgcaaaguuucaggugugu...............................	40	1	H01
....gugagcaaaguuucaggCgugu...............................	27	1	H01
....gugagcaaaguuucagguguAu...............................	175	1	H01
....gugagcaaaguuucaCgugugu...............................	51	1	H01
....guAagcaaaguuucaggugugu...............................	12	1	H01
....gAgagcaaaguuucaggugugu...............................	229	1	H01
....gugagcaaaguuucaAgugugu...............................	51	1	H01
....gugagcaaaguuucagAugugu...............................	29	1	H01
....gugagcUaaguuucaggugugu...............................	9	1	H01
....gugagcaaaguuuUaggugugu...............................	524	1	H01
....gugagcaaaguuuGaggugugu...............................	87	1	H01
....gugagcaaaguuucagguguUu...............................	3183	1	H01
....gugagGaaaguuucaggugugu...............................	6	1	H01
....gugagcaaaguGucaggugugu...............................	13	1	H01
....gugagcaCaguuucaggugugu...............................	8	1	H01
....gugagcaaagAuucaggugugu...............................	11	1	H01
....gugagcaaaguuCcaggugugu...............................	112	1	H01
....gCgagcaaaguuucaggugugu...............................	48	1	H01
....gugagcaaaguuucaggugugGc..............................	1	1	H01
....gugagcaaaguuucaggugugAc..............................	2	1	H01
....gugagUaaaguuucagguguguc..............................	1	1	H01
....gugagcaaaguuucagguguUuc..............................	1	1	H01
....gugagcaaaguuucaUguguguc..............................	1	1	H01
....gugagcaaaguuucagguguguc..............................	85	0	H01
....gugagcaaaguuucagguguguG..............................	111	1	H01
....gugagcaaaguuucGgguguguc..............................	1	1	H01
....gugagcaaaguuucaggugugCc..............................	1	1	H01
....gugagcaaaguuucagguguCuc..............................	1	1	H01
....gugagcaaaguuucagguguguGg.............................	6	1	H01
....gugagcaaaguuucaggugugucC.............................	71	1	H01
....gugagcaaaguuucaggugugCcg.............................	1	1	H01
....gugagcaaaguuucaggugugucg.............................	14	0	H01
....gugagcaaaguuucaggugugucU.............................	18	1	H01

Star **Mature**

```
ugagagaaugucggccuugugugcccgaaacuuugacucaaccugaguuuaggaaaaggugagcaaaguuucaggugugucggguugacugaaaaaacugguuuaccagga
..........................................................gugagcaaaguuucagguguguGgg............................ 3 1 H01
..........................................................gugagcaaaguuucagguguguAgg............................ 1 1 H01
..........................................................gugagcaaaguuucaggugugucgU............................ 2 1 H01
..........................................................gugagcaaaguuucaggugugucCg............................ 3 1 H01
..........................................................gugagcaaaguuucaggugugucgAg........................... 2 1 H01
..........................................................gugagcaaaguuucagguguguGggg........................... 2 1 H01
..........................................................gugagcaaaguuucaggugugucggU........................... 1 1 H01
..........................................................gugagcaaaguuucaggugugucggC........................... 1 1 H01
..........................................................gugagcaaaguuucaggugugucggUu.......................... 1 1 H01
..........................................................gugagcaaaguuucaggugugucgggu.......................... 2 0 H01
...........................................................ugagcaaaguuucaggugu................................. 2 0 H01
...........................................................ugagcaaaguuucaggugug................................ 4 0 H01
...........................................................Cgagcaaaguuucaggugugu............................... 1 1 H01
...........................................................Ggagcaaaguuucaggugugu............................... 5 1 H01
...........................................................ugagcaaaguuucaggugugu............................... 133 0 H01
...........................................................ugagcaaaguuucaggugugc.............................. 1 0 H01
............................................................gCgcaaaguuucaggugu................................. 1 1 H01
............................................................gagcaaaguuucaggugu................................. 6 0 H01
............................................................gagcaaaguuucagguguU................................ 5 1 H01
............................................................gagcaaaguuucaggugug................................ 15 0 H01
............................................................gagcaaaguuucagguguUu............................... 4 1 H01
............................................................Cagcaaaguuucaggugugu............................... 4 1 H01
............................................................gagcaaaguuucUggugugu............................... 1 1 H01
............................................................gagcaaaguuucaggugugu............................... 401 0 H01
............................................................gagcaaaguuucGggugugu............................... 1 1 H01
............................................................gUgcaaaguuucaggugugu............................... 2 1 H01
............................................................Uagcaaaguuucaggugugu............................... 3 1 H01
............................................................gagcaaaguuucaggugugA............................... 2 1 H01
.............................................................agcaaaguuucagguguU................................ 1 1 H01
.............................................................agcaaaguuucagUugug................................ 1 1 H01
.............................................................agcaaaguuucaggugug................................ 23 0 H01
.............................................................agcaaaguuucagguCugu............................... 1 1 H01
.............................................................agUaaaguuucaggugugu............................... 2 1 H01
.............................................................aUcaaaguuucaggugugu............................... 1 1 H01
.............................................................agcaaaguuucagguguUu............................... 5 1 H01
.............................................................agcaaaguuucaggugugu............................... 455 0 H01
.............................................................agcaaaguuucagAugugu............................... 1 1 H01
.............................................................agcaaaguuucaCgugugu............................... 1 1 H01
..............................................................gcaaaguuucaggugugA............................... 2 1 H01
..............................................................gcaaaguuucaggugugu............................... 346 0 H01
..............................................................Ucaaaguuucaggugugu............................... 1 1 H01
..............................................................gUaaaguuucaggugugu............................... 2 1 H01
..............................................................gcaaaguuuUaggugugu............................... 1 1 H01
..............................................................gcaaagCuucaggugugu............................... 2 1 H01

...................Cgugcccgaaacuuugacucaac..................................................................... 1 1 LJ5
....................gugcccgaaacuuugacA......................................................................... 1 1 LJ5
....................gugcccgaaacuuugacC......................................................................... 4 1 LJ5
....................gugcccgaaacuuugacu......................................................................... 39 0 LJ5
....................gugcccgaaacuuugacG......................................................................... 1 1 LJ5
....................gugcccgaaacuuugacuU........................................................................ 3 1 LJ5
....................gugcccgaaacuuugacuA........................................................................ 3 1 LJ5
....................gugcccgaaacuuugacuc........................................................................ 27 0 LJ5
....................gugcccgaaacuuCgacuc........................................................................ 1 1 LJ5
....................gugcccAaaacuuugacuc........................................................................ 1 1 LJ5
....................gugcccgaaacuuugacuca....................................................................... 26 0 LJ5
....................gugcccgaaacuuugacuAa....................................................................... 1 1 LJ5
....................gugcccgaaacuuugacucU....................................................................... 1 1 LJ5
....................gugcccgaaacuuugacucG....................................................................... 1 1 LJ5
....................gugcccgaaacuuugaUucaa...................................................................... 1 1 LJ5
....................guCcccgaaacuuugacucaa...................................................................... 1 1 LJ5
....................gugcccgaaacuuCgacucaa...................................................................... 1 1 LJ5
....................gugcccgaaacuCugacucaa...................................................................... 1 1 LJ5
....................gugcccgaaacuuugacucGa...................................................................... 1 1 LJ5
....................gugcccgaaacuuugacCcaa...................................................................... 1 1 LJ5
....................gugcccUaaacuuugacucaa...................................................................... 1 1 LJ5
....................Uugcccgaaacuuugacucaa...................................................................... 1 1 LJ5
....................gugcccgaaacuuugacucaa...................................................................... 94 0 LJ5
....................gugcccgaGacuuugacucaac..................................................................... 1 1 LJ5
....................gugcccgaaacuuugacucaac..................................................................... 89 0 LJ5
```

Star Mature

ugagagaaugucggccuugugugcccgaaacuuugacucaaccugaguuuaggaaaaggugagcaaaguuucaggugugucgguugacugaaaaaacugguuuaccagga			
....................gugcccgaaacuuugacucaaU..	9	1	LJ5
....................gugcccgaaacuuugacucaaA..	2	1	LJ5
....................gugcccgaaaUuuugacucaac..	1	1	LJ5
....................gugcccgaaacuuuAacucaacc...	1	1	LJ5
....................gugcccgaaacuuCgacucaacc...	2	1	LJ5
....................gugcccgaaacuuugacucaacU...	26	1	LJ5
....................gugcccgaaacuuugacucaacc...	117	0	LJ5
....................gugcccgaaGcuuugacucaacc...	1	1	LJ5
....................guAcccgaaacuuugacucaacc...	2	1	LJ5
....................gCgcccgaaacuuugacucaacc...	2	1	LJ5
....................gugcccgGaacuuugacucaacc...	1	1	LJ5
....................gugcccgaaacuuugacucaacA...	8	1	LJ5
....................guUcccgaaacuuugacucaacc...	2	1	LJ5
....................guAcccgaaacuuugacucaaccu..	7	1	LJ5
....................gugcccgaaGcuuugacucaaccu..	5	1	LJ5
....................gugcccgaaacuuugacuAaaccu..	2	1	LJ5
....................gugcccgaaUcuuugacucaaccu..	1	1	LJ5
....................gugcccgaaacuuugUcucaaccu..	6	1	LJ5
....................gugcccgaaacuuCgacucaaccu..	1	1	LJ5
....................guUcccgaaacuuugacucaaccu..	3	1	LJ5
....................gugcccgaaacuuugacucaaccu..	1232	0	LJ5
....................gugcccgaaacuuugacucaGccu..	1	1	LJ5
....................gugccAgaaacuuugacucaaccu..	1	1	LJ5
....................gugcccgaaacuuugacucaaccC..	173	1	LJ5
....................gugcccgaaacuuugacucaaccA..	129	1	LJ5
....................gugcccgaaAuuugacucaaccu..	1	1	LJ5
....................gugcccUaaacuuugacucaaccu..	1	1	LJ5
....................gugcccgaaacuuugacucaacAu..	1	1	LJ5
....................gugcccgaaacuuugacCcaaccu..	4	1	LJ5
....................gugcccgGaacuuugacucaaccu..	6	1	LJ5
....................gugcccgaaacuuugacucGaccu..	9	1	LJ5
....................guCcccgaaacuuugacucaaccu..	1	1	LJ5
....................gugcccgaaacuuugacucaaccG..	39	1	LJ5
....................gugcccgaaacuuugacuUaaccu..	4	1	LJ5
....................gugcccgaaacuuugacucUaccu..	1	1	LJ5
....................gCgcccgaaacuuugacucaaccu..	2	1	LJ5
....................gugcUcgaaacuuugacucaaccu..	2	1	LJ5
....................gugcccgaaacuuugaUucaaccu..	1	1	LJ5
....................Augcccgaaacuuugacucaaccu..	3	1	LJ5
....................gugcccgaGacuuugacucaaccu..	2	1	LJ5
....................gugccUgaaacuuugacucaaccu..	4	1	LJ5
....................gugUccgaaacuuugacucaaccu..	4	1	LJ5
....................gugcccgaaacuuugacucaaUcu..	1	1	LJ5
....................gugcccgaaacuCugacucaaccu..	3	1	LJ5
....................gugcccCaaacuuugacucaaccu..	2	1	LJ5
....................gugcccgaaacuuugacucaacUu..	7	1	LJ5
....................gugcccgaaacuuugacucaaccuC...	1	1	LJ5
....................gugcccgaaacuuugacucaaccuA...	2	1	LJ5
....................gugcccgaaacuuugacucaaccuU...	20	1	LJ5
.....................ugcccgaaacuuugacuca..	1	0	LJ5
.....................ugcccgaaacuuugacucaa...	1	0	LJ5
.....................ugcccgaaacuuugacucaac..	1	0	LJ5
.....................ugcccgaaacuuugacucaacU...	1	1	LJ5
.....................ugcccgaaacuuugacucaacA...	1	1	LJ5
.....................ugcccgaaacuuugacucaaccA..	2	1	LJ5
.....................ugcccgaaacuuugacucaaccC..	7	1	LJ5
.....................ugcccgaaacuuugacucaaccG..	1	1	LJ5
.....................ugcccgaaacuuugacucaaccu..	29	0	LJ5
.....................ugcccgaaacuuugacucaaccug...	2	0	LJ5
.....................ugcccgaaacuuugacucaaccuU...	8	1	LJ5
.......................cccgaaacuuugacucaaccC..	1	1	LJ5
........................ccgaaacuuugacucaaccC..	1	1	LJ5
....Cgugagcaaaguuucaggugug...............................	1	1	LJ5
....Cgugagcaaaguuucaggugugu..............................	6	1	LJ5
....gAugagcaaaguuucaggugugu..............................	1	1	LJ5
....gugagcaaaguuucaggu..................................	21	0	LJ5
....gugagcaaaguuucaggC..................................	3	1	LJ5
....gugagcaaaguuucagguU.................................	2	1	LJ5
....gugGgcaaaguuucaggug.................................	1	1	LJ5
....gugagcaaaguuucagguA.................................	3	1	LJ5

Star **Mature**

```
ugagagaaugucggccuugugugcccgaaacuuugacucaaccugaguuuaggaaaaggugagcaaaguuucaggugugucggguugacugaaaaaacugguuuaccagga
..........................................................gugagcaGaguuucaggug..................................  1    1  LJ5
..........................................................gugagcaaaguuucaggug..................................  62   0  LJ5
..........................................................gugagcaaaguuucaggGg..................................  1    1  LJ5
..........................................................gugagcaaagGuucaggugu.................................  1    1  LJ5
..........................................................gugagcaaaguuucaggugG.................................  4    1  LJ5
..........................................................gugagcaaaguuucGggugu.................................  1    1  LJ5
..........................................................gugagcaaaguuucaggugu.................................  140  0  LJ5
..........................................................gugagcaaaguuucaggugC.................................  20   1  LJ5
..........................................................guUagcaaaguuucaggugu.................................  1    1  LJ5
..........................................................gCgagcaaaguuucaggugu.................................  1    1  LJ5
..........................................................gugagcaaaguuucaggGgu.................................  1    1  LJ5
..........................................................gugagcaaaguuucagguAug................................  1    1  LJ5
..........................................................gugagcaGaguuucaggugug................................  3    1  LJ5
..........................................................gugagcaaaguuucGggugug................................  1    1  LJ5
..........................................................gugagcaaaguuucaAgugug................................  1    1  LJ5
..........................................................guUagcaaaguuucaggugug................................  4    1  LJ5
..........................................................gugagcGaaguuucaggugug................................  3    1  LJ5
..........................................................gugaAcaaaguuucaggugug................................  1    1  LJ5
..........................................................gugagcaaagCuucaggugug................................  3    1  LJ5
..........................................................gugagcaaaguuCcaggugug................................  3    1  LJ5
..........................................................gugagUaaaguuucaggugug................................  1    1  LJ5
..........................................................gugagcaaaguuucagguguA................................  76   1  LJ5
..........................................................gAgagcaaaguuucaggugug................................  3    1  LJ5
..........................................................gugagcaaaguuucaUgugug................................  4    1  LJ5
..........................................................gugagcaaaguuucaggCgug................................  1    1  LJ5
..........................................................gugagcaaaguuucagguguU................................  23   1  LJ5
..........................................................gCgagcaaaguuucaggugug................................  3    1  LJ5
..........................................................gugagcCaaguuucaggugug................................  1    1  LJ5
..........................................................guAagcaaaguuucaggugug................................  2    1  LJ5
..........................................................gugagcaaaguCucaggugug................................  1    1  LJ5
..........................................................gugagcaaaguuucagguguC................................  4    1  LJ5
..........................................................gugagcaaaguuucaggugCg................................  9    1  LJ5
..........................................................Augagcaaaguuucaggugug................................  1    1  LJ5
..........................................................guCagcaaaguuucaggugug................................  2    1  LJ5
..........................................................gugagcaaaguuucaggugug................................  818  0  LJ5
..........................................................gugGgcaaaguuucaggugug................................  2    1  LJ5
..........................................................gugUgcaaaguuucaggugug................................  3    1  LJ5
..........................................................gugagcaaaguuucUggugug................................  2    1  LJ5
..........................................................gugagcaaGguuucaggugug................................  4    1  LJ5
..........................................................gugagcaaaguuucagguguUu...............................  11   1  LJ5
..........................................................gugagcaaaguuucagUugugu...............................  1    1  LJ5
..........................................................gugagAaaaguuucaggugugu...............................  2    1  LJ5
..........................................................gugagcaaaguuucagguguCu...............................  4    1  LJ5
..........................................................gugagcaCaguuucaggugugu...............................  4    1  LJ5
..........................................................gugGgcaaaguuucaggugugu...............................  22   1  LJ5
..........................................................gugagcaaaguuucUggugugu...............................  7    1  LJ5
..........................................................gugagcaaaUuuucaggugugu...............................  3    1  LJ5
..........................................................guUagcaaaguuucaggugugu...............................  14   1  LJ5
..........................................................gugagcaaaguuucaggugugA...............................  176  1  LJ5
..........................................................gugagcaaaguuCcaggugugu...............................  15   1  LJ5
..........................................................guAagcaaaguuucaggugugu...............................  21   1  LJ5
..........................................................gugaAcaaaguuucaggugugu...............................  5    1  LJ5
..........................................................gugagcaaaguuucGggugugu...............................  25   1  LJ5
..........................................................gugagcaaaguuucaUgugugu...............................  1    1  LJ5
..........................................................gugagcaaaguuucaggAgugu...............................  1    1  LJ5
..........................................................gugagcaaaguCucaggugugu...............................  9    1  LJ5
..........................................................gugagcaaaguuucagguCugu...............................  1    1  LJ5
..........................................................gugagcaaaguuucaggugCgu...............................  28   1  LJ5
..........................................................gugagcaaaguuucagCugugu...............................  1    1  LJ5
..........................................................gugagcaaaguuucagguAugu...............................  3    1  LJ5
..........................................................guCagcaaaguuucaggugugu...............................  9    1  LJ5
..........................................................gugagcaaaguGucaggugugu...............................  1    1  LJ5
..........................................................gugagUaaaguuucaggugugu...............................  7    1  LJ5
..........................................................gugagcaaaguuucaggugAgu...............................  2    1  LJ5
..........................................................gugagcaGaguuucaggugugu...............................  22   1  LJ5
..........................................................gugagcaUaguuucaggugugu...............................  4    1  LJ5
..........................................................gugagcaaaguuucaggCgugu...............................  17   1  LJ5
..........................................................Uugagcaaaguuucaggugugu...............................  1    1  LJ5
..........................................................gugagcUaaguuucaggugugu...............................  4    1  LJ5
..........................................................gugagcaaaguuucagAugugu...............................  4    1  LJ5
```

Star   Mature

ugagagaaugucggccuugugugcccgaaacuuugacucaaccugaguuuaggaaaaggugagcaaaguuucaggugugucggguugacugaaaaaacugguuuaccagga			
....gugaCcaaaguuucaggugugu...............................	2	1	LJ5
....gugagcaaaguuucCggugugu...............................	1	1	LJ5
....gugagcaaagCuucaggugugu...............................	9	1	LJ5
....gugagcaaaguuGcaggugugu...............................	1	1	LJ5
....gugaUcaaaguuucaggugugu...............................	2	1	LJ5
....gugagcaaaguuucaggugugu...............................	5517	0	LJ5
....gugagcGaaguuucaggugugu...............................	12	1	LJ5
....gugagcaaagGuucaggugugu...............................	10	1	LJ5
....gugagcaaaguuuAaggugugu...............................	1	1	LJ5
....gugagcaaaguuucaAgugugu...............................	2	1	LJ5
....gugagcaaaguuucaggGgugu...............................	3	1	LJ5
....gugagcaaaguuuUaggugugu...............................	11	1	LJ5
....gugagcaaaguuAcaggugugu...............................	2	1	LJ5
....gugagcaaGguuucaggugugu...............................	27	1	LJ5
....gugagcaaaguuuGaggugugu...............................	1	1	LJ5
....Augagcaaaguuucaggugugu...............................	4	1	LJ5
....gugagcaaaguuucaggugugG...............................	93	1	LJ5
....gugagcCaaguuucaggugugu...............................	1	1	LJ5
....gCgagcaaaguuucaggugugu...............................	17	1	LJ5
....gugagcaaaguuucaggugGgu...............................	7	1	LJ5
....gGgagcaaaguuucaggugugu...............................	1	1	LJ5
....gugagcaaUguuucaggugugu...............................	1	1	LJ5
....gugagcaaaguuucagguguAu...............................	5	1	LJ5
....gugagcaaaguuucagguguguc..............................	11	0	LJ5
....gugagcaaaguuucaggCguguc..............................	2	1	LJ5
....gugagcaaaguuucagguguguG..............................	42	1	LJ5
....gugagcaaaguuucaggGguguc..............................	1	1	LJ5
....gugagcaaaguuucagguguguggC...........................	1	1	LJ5
....ugagcaaaguuucaggugug................................	1	0	LJ5
....ugagcaaaguuucaggugugu...............................	7	0	LJ5
....ugagcaaaguuCcaggugugu...............................	1	1	LJ5
....gagcaaaguuucaggugug................................	2	0	LJ5
....gagcaaaguuucaggugugu...............................	2	0	LJ5
....................gugcccgGaacuuugacuca...	1	1	2nd
....................gugcccgaaacuuugacuca...	2	0	2nd
....................gugcccgaaacuuugacucaa..	7	0	2nd
....................gugcccAaaacuuugacucaac...	1	1	2nd
....................gugcccgaaacuuugacucaac...	3	0	2nd
....................gugcccgaaacuuugacuUaacc..	1	1	2nd
....................gugcccgaaacuuugacucaacc..	22	0	2nd
....................gugcccgUaacuuugacucaaccu...	1	1	2nd
....................gugcccgaaacuuugacucaaccu...	79	0	2nd
....................gugcccgaaacuuugUcucaaccu...	1	1	2nd
....................gugccAgaaacuuugacucaaccu...	1	1	2nd
.....................ugcccgaaacuuugacucaaccu...	4	0	2nd
....aaaaggugagcaaaguuucaggugugu...............................	1	0	2nd
....aaGggugagcaaaguuucaggugugu...............................	2	1	2nd
....aaggugagcaaaguuucaggugu.................................	1	0	2nd
....aaCgugagcaaaguuucaggugug................................	2	1	2nd
....aGggugagcaaaguuucaggugugu...............................	9	1	2nd
....Uggugagcaaaguuucagg....................................	1	1	2nd
....Uggugagcaaaguuucaggugug................................	2	1	2nd
....aCgugagcaaaguuucaggugugu...............................	1	1	2nd
....Gggugagcaaaguuucaggugugu...............................	2	1	2nd
....Ugugagcaaaguuucaggu...................................	1	1	2nd
....ggugagcaaaguuucaggu...................................	2	0	2nd
....ggugagcaaaguuucaggug..................................	1	0	2nd
....Ugugagcaaaguuucaggugu.................................	5	1	2nd
....ggugagcaaaguuucaggugu.................................	23	0	2nd
....ggugagcaaaguuucaggugug................................	57	0	2nd
....ggugagcaaaguuucagguguC................................	3	1	2nd
....ggugagcaaaguuucagguguA................................	1	1	2nd
....Ugugagcaaaguuucaggugug................................	13	1	2nd
....Cgugagcaaaguuucaggugug................................	1	1	2nd
....ggugagcaaaguuucGggugug................................	1	1	2nd
....ggugagcaaaguuucagguguU................................	2	1	2nd
....ggugagcaaaAuuucaggugug................................	1	1	2nd
....Ugugagcaaaguuucaggugugu...............................	171	1	2nd
....ggugagcaaaguuucagguguUu...............................	5	1	2nd

Star **Mature**

```
ugagagaaugucggccuugugugcccgaaacuuugacucaaccugaguuuaggaaaaggugagcaaaguuucaggugugucggguugacugaaaaaacugguuuaccagga
.........................................................Cgugagcaaaguuucaggugugu...............................   4      1   2nd
.........................................................ggugagcaaaguuucaggugugu...............................   125    0   2nd
.........................................................ggGgagcaaaguuucaggugugu...............................   6      1   2nd
.........................................................ggugagcaaaguuucaggugugA...............................   6      1   2nd
.........................................................Agugagcaaaguuucaggugugu...............................   2      1   2nd
.........................................................ggugagUaaaguuucaggugugu...............................   1      1   2nd
..........................................................gugagGaaaguuucaggu...................................   2      1   2nd
..........................................................gugagcaaaguCucaggu...................................   1      1   2nd
..........................................................gugagcaaaguuucCggu...................................   3      1   2nd
..........................................................gugagcaaaguuucaggC...................................   1      1   2nd
..........................................................gugagcaaaguuucaggu...................................   567    0   2nd
..........................................................gAgagcaaaguuucaggu...................................   2      1   2nd
..........................................................Uugagcaaaguuucaggu...................................   1      1   2nd
..........................................................gugagcaaaguuucaggA...................................   12     1   2nd
..........................................................gugagcUaaguuucaggu...................................   1      1   2nd
..........................................................gugCgcaaaguuucaggug..................................   1      1   2nd
..........................................................gugagcaaaguuucaggug..................................   797    0   2nd
..........................................................gugagGaaaguuucaggug..................................   1      1   2nd
..........................................................gugagcaaaguGucaggug..................................   1      1   2nd
..........................................................gugagUaaaguuucaggug..................................   1      1   2nd
..........................................................guCagcaaaguuucaggug..................................   1      1   2nd
..........................................................gugagcaaagAuucaggug..................................   1      1   2nd
..........................................................gugagcaaaguuucagguC..................................   6      1   2nd
..........................................................gugagcaaaguuucaggCg..................................   3      1   2nd
..........................................................gugagcaaaguuucUggug..................................   2      1   2nd
..........................................................gugagcaaaguuuUaggug..................................   2      1   2nd
..........................................................gugagcaaaguuucagguU..................................   8      1   2nd
..........................................................gugagcaaaguuucCggug..................................   3      1   2nd
..........................................................gugGgcaaaguuucaggug..................................   1      1   2nd
..........................................................gugagcaaaguuucaggGg..................................   1      1   2nd
..........................................................gugagcaaaguuucagguA..................................   7      1   2nd
..........................................................gugagcaaaguuucagguUu.................................   4      1   2nd
..........................................................gugagcCaaguuucaggugu.................................   2      1   2nd
..........................................................gugGgcaaaguuucaggugu.................................   4      1   2nd
..........................................................gugagGaaaguuucaggugu.................................   4      1   2nd
..........................................................gugagcaaaguuuGaggugu.................................   1      1   2nd
..........................................................gugagcaaaguuucaUgugu.................................   3      1   2nd
..........................................................gugagcaGaguuucaggugu.................................   1      1   2nd
..........................................................gugagcaaaguuucaggugG.................................   4      1   2nd
..........................................................gugagcaaaCuuucaggugu.................................   1      1   2nd
..........................................................gGgagcaaaguuucaggugu.................................   4      1   2nd
..........................................................gugagcaaaguuucagguAu.................................   1      1   2nd
..........................................................gAgagcaaaguuucaggugu.................................   5      1   2nd
..........................................................gCgagcaaaguuucaggugu.................................   1      1   2nd
..........................................................gugCgcaaaguuucaggugu.................................   2      1   2nd
..........................................................Augagcaaaguuucaggugu.................................   1      1   2nd
..........................................................gugagcaaaguuucaCgugu.................................   2      1   2nd
..........................................................gugagcaaaguuucaggugC.................................   18     1   2nd
..........................................................gugagcaaaguuucagUugu.................................   2      1   2nd
..........................................................gugagcaaaguuucagguCu.................................   3      1   2nd
..........................................................gugagUaaaguuucaggugu.................................   3      1   2nd
..........................................................gugagcUaaguuucaggugu.................................   7      1   2nd
..........................................................gugagcaaaguuucaAgugu.................................   3      1   2nd
..........................................................gugagcaaaguuucUggugu.................................   3      1   2nd
..........................................................gugagcaaaguuucaggugu.................................   7905   0   2nd
..........................................................gugagcaaaguuucaggCgu.................................   3      1   2nd
..........................................................gugUgcaaaguuucaggugu.................................   2      1   2nd
..........................................................gugagcGaaguuucaggugu.................................   1      1   2nd
..........................................................gugagAaaaguuucaggugu.................................   7      1   2nd
..........................................................gugagcaaaguuuUaggugu.................................   4      1   2nd
..........................................................guUagcaaaguuucaggugu.................................   1      1   2nd
..........................................................gugagcaaagCuucaggugu.................................   2      1   2nd
..........................................................Cugagcaaaguuucaggugu.................................   1      1   2nd
..........................................................gugagcaaaguuucagAugu.................................   5      1   2nd
..........................................................gugagcaaaguuGcaggugu.................................   2      1   2nd
..........................................................gugagcaaaguuucaggGgu.................................   8      1   2nd
..........................................................gugagcaaaguCucaggugu.................................   4      1   2nd
..........................................................gugaAcaaaguuucaggugu.................................   3      1   2nd
..........................................................gugagcaaagAuucaggugu.................................   1      1   2nd
..........................................................gugagcaaagGuucaggugu.................................   3      1   2nd
```

Star / Mature			
ugagagaaugucggccuugugugcccgaaacuuugacucaaccugaguuuaggaaaaggugagcaaaguuucaggugugucggguugacugaaaaaacugguuuaccagga			
....gugUgcaaaguuucaggugug................................	5	1	2nd
....gugagcaaagGuucaggugug................................	3	1	2nd
....gCgagcaaaguuucaggugug................................	3	1	2nd
....gugagcaaaguCucaggugug................................	4	1	2nd
....gugagcaaaguuucaggugug................................	22296	0	2nd
....gugagcaaaguuucagUugug................................	2	1	2nd
....Uugagcaaaguuucaggugug................................	6	1	2nd
....gugagcaaaguuucaggugGg................................	11	1	2nd
....gugagcaaaguuucaCgugug................................	10	1	2nd
....gugagcaaaguuucagguguC................................	9	1	2nd
....gugagcUaaguuucaggugug................................	20	1	2nd
....gugagcaaUguuucaggugug................................	1	1	2nd
....gugagcaaGguuucaggugug................................	2	1	2nd
....gugagcaaagCuucaggugug................................	6	1	2nd
....gugagcaaaguuucaggCgug................................	9	1	2nd
....gugagcaaaguuuUaggugug................................	8	1	2nd
....gugagUaaaguuucaggugug................................	17	1	2nd
....gugagcaaaguuucaUgugug................................	4	1	2nd
....gugagcaaaguuucagAugug................................	6	1	2nd
....guAagcaaaguuucaggugug................................	7	1	2nd
....gugagGaaaguuucaggugug................................	11	1	2nd
....gAgagcaaaguuucaggugug................................	17	1	2nd
....gugagcaaaguuucaggugu A................................	50	1	2nd
....gugagcaaaguuucaggAgug................................	3	1	2nd
....gugagcaaaguAucaggugug................................	2	1	2nd
....gGgagcaaaguuucaggugug................................	2	1	2nd
....guCagcaaaguuucaggugug................................	4	1	2nd
....gugagcaaaAuuucaggugug................................	2	1	2nd
....gugagcaaaguuucagCugug................................	4	1	2nd
....gugagcaaaCuuucaggugug................................	4	1	2nd
....gugGgcaaaguuucaggugug................................	8	1	2nd
....gugagcaaaguuucaggugCg................................	22	1	2nd
....gugaAcaaaguuucaggugug................................	5	1	2nd
....gugCgcaaaguuucaggugug................................	4	1	2nd
....gugaCcaaaguuucaggugug................................	4	1	2nd
....gugagcaaaguuCcaggugug................................	4	1	2nd
....gugagcGaaguuucaggugug................................	2	1	2nd
....gugagcaaagAuucaggugug................................	1	1	2nd
....gugagcaaaguuucagguCug................................	2	1	2nd
....gugagcaaaguuucaAgugug................................	7	1	2nd
....gugagcaaaguuucGgugug................................	12	1	2nd
....gugagcaaaguuucagguguU................................	865	1	2nd
....gugagcaaaguuucCggugug................................	1	1	2nd
....gugagcaCaguuucaggugug................................	4	1	2nd
....gugagcaaaguuucaggGgug................................	12	1	2nd
....gugagAaaaguuucaggugug................................	16	1	2nd
....gugagcaaaguuucaggugAg................................	5	1	2nd
....gugaUcaaaguuucaggugug................................	1	1	2nd
....gugagcaUaguuucaggugug................................	2	1	2nd
....gugagcaaaguuucagguAug................................	3	1	2nd
....gugagcaaaguuuGaggugug................................	1	1	2nd
....gugagcaaaguuucUggugug................................	8	1	2nd
....gugagcaaaguuucGgugugu...............................	222	1	2nd
....gugCgcaaaguuucaggugugu...............................	66	1	2nd
....gugagcaaaguuucagguUugu...............................	50	1	2nd
....gugagcaaaguuucaggugGgu...............................	188	1	2nd
....gugagcaaagCuucaggugugu...............................	75	1	2nd
....gugagcaaaguuGcaggugugu...............................	3	1	2nd
....gugaAcaaaguuucaggugugu...............................	119	1	2nd
....gugagcaaaguuucaggugugu...............................	449436	0	2nd
....gugagcaaaguGucaggugugu...............................	29	1	2nd
....gugagcaaaguCucaggugugu...............................	45	1	2nd
....guAagcaaaguuucaggugugu...............................	71	1	2nd
....gAgagcaaaguuucaggugugu...............................	287	1	2nd
....guCagcaaaguuucaggugugu...............................	94	1	2nd
....gugagcaaaguuucaggGugu...............................	171	1	2nd
....gugGgcaaaguuucaggugugu...............................	195	1	2nd
....gGgagcaaaguuucaggugugu...............................	93	1	2nd
....gugagcaaaguuucaggugAgu...............................	52	1	2nd
....Uugagcaaaguuucaggugugu...............................	50	1	2nd

Star **Mature**

```
ugagagaaugucggccuugugugcccgaaacuuugacucaaccugaguuuaggaaaaggugagcaaaguuucaggugugucgguugacugaaaaaacugguuuaccagga
..........................................................gugagUaaaguuucaggugugu..............................  379   1   2nd
..........................................................gugagcaUaguuucaggugugu..............................  95    1   2nd
..........................................................gugagcaaaguuucagguguCu..............................  68    1   2nd
..........................................................gugagcaaaguuucaCgugugu..............................  178   1   2nd
..........................................................gugagcaaaguuucCggugugu..............................  12    1   2nd
..........................................................Augagcaaaguuucaggugugu..............................  34    1   2nd
..........................................................gugagcaaaguuucagCugugu..............................  142   1   2nd
..........................................................Cugagcaaaguuucaggugugu..............................  35    1   2nd
..........................................................gugagAaaaguuucaggugugu..............................  306   1   2nd
..........................................................gugagcaaaguuAcaggugugu..............................  8     1   2nd
..........................................................gugagcaaaCuuucaggugugu..............................  47    1   2nd
..........................................................gugagcaaagAuucaggugugu..............................  71    1   2nd
..........................................................gugagcaaaguuucagguAugu..............................  29    1   2nd
..........................................................gugagcaaaguuucaggAgugu..............................  32    1   2nd
..........................................................gugagcaaaguuucaggugugA..............................  3374  1   2nd
..........................................................gugagcaCaguuucaggugugu..............................  59    1   2nd
..........................................................gugagcaaaguuucagguguAu..............................  109   1   2nd
..........................................................gugUgcaaaguuucaggugugu..............................  97    1   2nd
..........................................................gugagGaaaguuucaggugugu..............................  178   1   2nd
..........................................................gugagcaaGguuucaggugugu..............................  20    1   2nd
..........................................................gugagcaaaguuucUggugugu..............................  201   1   2nd
..........................................................gugaCcaaaguuucaggugugu..............................  73    1   2nd
..........................................................gugagcaaaguuucaAgugugu..............................  117   1   2nd
..........................................................gugagcaaaguuucagAugugu..............................  131   1   2nd
..........................................................gugagcaaaUuuucaggugugu..............................  14    1   2nd
..........................................................gugagcUaaguuucaggugugu..............................  484   1   2nd
..........................................................gugagcaaagGuucaggugugu..............................  120   1   2nd
..........................................................gugagcaaaAuuucaggugugu..............................  43    1   2nd
..........................................................gugagcaaUguuucaggugugu..............................  23    1   2nd
..........................................................gugagcaaaguuucaggCgugu..............................  92    1   2nd
..........................................................gugagcaaaguuucagUugugu..............................  129   1   2nd
..........................................................gugagcaaaguuucaggugugG..............................  242   1   2nd
..........................................................gugagcaaaguuuGaggugugu..............................  41    1   2nd
..........................................................gCgagcaaaguuucaggugugu..............................  48    1   2nd
..........................................................gugagcaaaguuCcaggugugu..............................  46    1   2nd
..........................................................gugagcaaaguuucagguCugu..............................  59    1   2nd
..........................................................gugagcaaaguuuUaggugugu..............................  265   1   2nd
..........................................................gugagcCaaguuucaggugugu..............................  19    1   2nd
..........................................................gugagcaaaguAucaggugugu..............................  23    1   2nd
..........................................................gugagcaaaguuuAaggugugu..............................  29    1   2nd
..........................................................gugagcaaaguuucaUgugugu..............................  111   1   2nd
..........................................................guUagcaaaguuucaggugugu..............................  33    1   2nd
..........................................................gugagcaaaguuucaggugCgu..............................  118   1   2nd
..........................................................gugaUcaaaguuucaggugugu..............................  20    1   2nd
..........................................................gugagcaaaguuucagguguUu..............................  2028  1   2nd
..........................................................gugagcGaaguuucaggugugu..............................  69    1   2nd
..........................................................gugagcaaCguuucaggugugu..............................  10    1   2nd
..........................................................gugagcaGaguuucaggugugu..............................  35    1   2nd
..........................................................gugagcaaaguuucaggugGguc.............................  1     1   2nd
..........................................................gugagcaaaguuucagguguguG.............................  78    1   2nd
..........................................................gugagcaaaguuucaggugugCc.............................  6     1   2nd
..........................................................gugagcaaaguuucaggugugAc.............................  1     1   2nd
..........................................................gugagcaaaguuucaggugugGc.............................  3     1   2nd
..........................................................gugagcaaaguuucagguguguc.............................  48    0   2nd
..........................................................gugagcaaaguuucagguguUuc.............................  1     1   2nd
..........................................................gugagcaaaguuucaggugugucg............................  11    0   2nd
..........................................................gugagcaaaguuucaggugugucU............................  13    1   2nd
..........................................................gugagcaaaguuucagguguguGg............................  1     1   2nd
..........................................................gugagcaaaguuucaggugugucC............................  9     1   2nd
..........................................................gugagcaaaguuucaggugugucgA...........................  1     1   2nd
..........................................................gugagcaaaguuucaggugugucgU...........................  2     1   2nd
..........................................................gugagcaaaguuucagguguguGgg...........................  1     1   2nd
..........................................................gugagcaaaguuucagguguguAgg...........................  1     1   2nd
..........................................................gugagcaaaguuucaggugugucgC...........................  5     1   2nd
..........................................................gugagcaaaguuucaggugugucggU..........................  1     1   2nd
..........................................................gugagcaaaguuucaggugugucCgg..........................  1     1   2nd
..........................................................gugagcaaaguuucaggugugucgUg..........................  3     1   2nd
..........................................................gugagcaaaguuucaggugugucgCg..........................  1     1   2nd
..........................................................gugagcaaaguuucagguguguGggg..........................  1     1   2nd
..........................................................gugagcaaaguuucaggugugucgggu.........................  1     0   2nd
```

```
                    Star                                Mature
ugagagaaugucggccuugugugcccgaaacuuugacucaaccugaguuuaggaaaaggugagcaaaguuucaggugugucggguugacugaaaaacugguuuaccagga
..........................................................gugagcaaaguuucagguguguGgggu.........................  1    1    2nd
...........................................................Ggagcaaaguuucaggugug...............................  1    1    2nd
...........................................................ugagcaaaguuucaggugug...............................  6    0    2nd
...........................................................ugagcaaaguuucagguguUu..............................  1    1    2nd
...........................................................ugagGaaaguuucaggugugu..............................  1    1    2nd
...........................................................uCagcaaaguuucaggugugu..............................  1    1    2nd
...........................................................ugagcCaaguuucaggugugu..............................  4    1    2nd
...........................................................Ggagcaaaguuucaggugugu..............................  1    1    2nd
...........................................................ugagcaaaguuucaggugugu..............................  52   0    2nd
............................................................gagcaaaguuucaggugu................................  4    0    2nd
............................................................gagcaaaguuucaggugug...............................  8    0    2nd
............................................................gagcaaaguuucagguguUu..............................  1    1    2nd
............................................................gagcaaaguuucaggugugu..............................  152  0    2nd
............................................................gagcaaaguuucGggugugu..............................  1    1    2nd
............................................................gagcaaaguuucaggugAgu..............................  1    1    2nd
............................................................gagcaaaguuucaggugugG..............................  1    1    2nd
............................................................gagcaaaguuucaggAgugu..............................  1    1    2nd
.............................................................agcaaaguuucaggugug...............................  4    0    2nd
.............................................................agcaaaguuucagguguUu..............................  1    1    2nd
.............................................................agcaaGguuucaggugugu..............................  1    1    2nd
.............................................................agcaaaguuucaggugugA..............................  1    1    2nd
.............................................................agcaaaguuucaggugugu..............................  125  0    2nd
..............................................................gcaaaguuucaggCgugu..............................  1    1    2nd
..............................................................gcaaaguuucaggugugu..............................  105  0    2nd
..............................................................gcaaaguuucaggugugA..............................  1    1    2nd
..............................................................gcaaaguuucagguguguc.............................  1    0    2nd

..................Uugugcccgaaacuuugacucaaccu..................................................................  2    1    E01
...................uUugcccgaaacuuugacucaaccu..................................................................  2    1    E01
....................gugcccgaaacuuugacu........................................................................  1    0    E01
....................gugcccgaaacuuugacucaa.....................................................................  6    0    E01
....................gugcccgaaacuuugacucaac....................................................................  5    0    E01
....................gugcccgaaacuuugacucaacc...................................................................  36   0    E01
....................gugcGcgaaacuuugacucaaccu..................................................................  1    1    E01
....................gugcccgaaacuuugUcucaaccu..................................................................  4    1    E01
....................gugcccgaaacuCugacucaaccu..................................................................  1    1    E01
....................gugcccgaaacuuugCcucaaccu..................................................................  1    1    E01
....................gugcccgaUacuuugacucaaccu..................................................................  1    1    E01
....................gugcccgaaacuuugacucaaccu..................................................................  345  0    E01
....................gugcccgaaacuuugacuUaaccu..................................................................  1    1    E01
....................gugcccgaCacuuugacucaaccu..................................................................  1    1    E01
....................gugcccgaaacuuCgacucaaccu..................................................................  1    1    E01
....................gugcccgaaacuuugacucaaccuga................................................................  1    0    E01
.....................ugcccgaaacuuugacucaac....................................................................  1    0    E01
.....................ugcccgaaacuuugacucaacc...................................................................  3    0    E01
.....................ugcccgaaacuuugacucaaccu..................................................................  20   0    E01
.....................ugcccgaaacuuugacuUaaccu..................................................................  1    1    E01
.....................ugcccgaaacuuugacucaaccuU.................................................................  3    1    E01
.......................cccgaaacuuugacucaaccu..................................................................  1    0    E01
..........................gaaacuuugacucaaccu..................................................................  1    0    E01
......................................................aaGggugagcaaaguuucaggugugu..............................  3    1    E01
.......................................................aGggugagcaaaguuucaggugugu..............................  6    1    E01
........................................................aggugagcaaaguuucaggugu................................  1    0    E01
........................................................aggugagcaaaguuucaggugugu..............................  1    0    E01
........................................................Gggugagcaaaguuucaggugugu..............................  1    1    E01
.........................................................ggugagcaaaguuucagg...................................  1    0    E01
.........................................................Ugugagcaaaguuucaggu..................................  1    1    E01
.........................................................ggugagcaaaguuucaggugu................................  15   0    E01
.........................................................ggugagcaaaguuucagguguU...............................  14   1    E01
.........................................................Ugugagcaaaguuucaggugug...............................  2    1    E01
.........................................................ggugagcaaaguuucaggugug...............................  93   0    E01
.........................................................ggugaAcaaaguuucaggugugu..............................  1    1    E01
.........................................................ggugagcaaaguuucagguguUu..............................  3    1    E01
.........................................................Cgugagcaaaguuucaggugugu..............................  4    1    E01
.........................................................ggugagcaaaguuucaggugugA..............................  8    1    E01
.........................................................Ugugagcaaaguuucaggugugu..............................  119  1    E01
.........................................................gUugagcaaaguuucaggugugu..............................  2    1    E01
.........................................................Agugagcaaaguuucaggugugu..............................  2    1    E01
.........................................................ggugagcaaaguuucaggugugu..............................  237  0    E01
..........................................................gugagcaaaguuucUggu..................................  1    1    E01
```

Star **Mature**

ugagagaaugucggccuugugugcccgaaacuuugacucaaccugaguuuaggaaaaggugagcaaaguuucaggugugucggguugacugaaaaaacugguuuaccagga			
....gugagcaaaguuucaggu...................................	265	0	E01
....gugagcaaaguuucagguC..................................	2	1	E01
....gugagcaaaguuucCggug..................................	1	1	E01
....gugagcaaaguuucaggug..................................	120	0	E01
....gugagcaaaguuucaggugu.................................	949	0	E01
....gAgagcaaaguuucaggugu.................................	1	1	E01
....gugagcaaaguuucaUgugu.................................	2	1	E01
....gugagcaaaguuucGggugu.................................	1	1	E01
....gugagcaaaguuucaggGgu.................................	1	1	E01
....guCagcaaaguuucaggugu.................................	1	1	E01
....gugagcaaaguuucaggCgu.................................	1	1	E01
....gugagcaaaguuucUggugu.................................	2	1	E01
....gugagcaaaguuucagguUu.................................	1	1	E01
....gugagcaaaguuAcaggugu.................................	1	1	E01
....gugagcaaagGuucaggugug................................	1	1	E01
....gugagcaaaguuucUggugug................................	2	1	E01
....gugagcaaaguuucaggugAg................................	3	1	E01
....gugUgcaaaguuucaggugug................................	1	1	E01
....gugagcaaaguuucagguguU................................	255	1	E01
....gugagcUaaguuucaggugug................................	1	1	E01
....gCgagcaaaguuucaggugug................................	1	1	E01
....gugCgcaaaguuucaggugug................................	1	1	E01
....gugaAcaaaguuucaggugug................................	1	1	E01
....guCagcaaaguuucaggugug................................	1	1	E01
....gAgagcaaaguuucaggugug................................	4	1	E01
....gugagcaaaguuucaggugCg................................	2	1	E01
....gugagcaaaguuucaAgugug................................	1	1	E01
....gugagcaaaguuucaUgugug................................	1	1	E01
....gugagcaaaguuuUaggugug................................	4	1	E01
....gugagcaaaguuucagguguC................................	3	1	E01
....gGgagcaaaguuucaggugug................................	1	1	E01
....gugagcaUaguuucaggugug................................	1	1	E01
....gugagcaaaguuucaggGgug................................	1	1	E01
....gugagcaaaguuucGggugug................................	3	1	E01
....gugagcaaaCuuucaggugug................................	2	1	E01
....gugagcaaaguuuGaggugug................................	1	1	E01
....gugagcaaaguuucagguguA................................	9	1	E01
....gugagcaaaguuucaggugug................................	6737	0	E01
....gugagcaaaguuCcaggugug................................	3	1	E01
....gugagcaaGguuucaggugug................................	1	1	E01
....guUagcaaaguuucaggugug................................	1	1	E01
....gugagUaaaguuucaggugug................................	4	1	E01
....gugagcaaaUuuucaggugug................................	1	1	E01
....gugagcaaCguuucaggugug................................	1	1	E01
....gugagcaaaguuucagCugug................................	1	1	E01
....gugGgcaaaguuucaggugug................................	2	1	E01
....gugagcaaaguuucaggugugG...............................	90	1	E01
....gugagcaaaguuAcaggugugu...............................	8	1	E01
....gugaAcaaaguuucaggugugu...............................	56	1	E01
....gugagcGaaguuucaggugugu...............................	12	1	E01
....gugagcaaaguuucaggCgugu...............................	17	1	E01
....gugagcaaaguuucaggugAgu...............................	11	1	E01
....gugagcaaaguuucagguAugu...............................	17	1	E01
....gugagcaaaguuucaCgugugu...............................	11	1	E01
....gAgagcaaaguuucaggugugu...............................	101	1	E01
....gugagcaaaguuucaggAgugu...............................	5	1	E01
....gugaUcaaaguuucaggugugu...............................	15	1	E01
....gugagcaaaguuucagguguUu...............................	584	1	E01
....gugGgcaaaguuucaggugugu...............................	39	1	E01
....gugUgcaaaguuucaggugugu...............................	38	1	E01
....gugagcaaaguuucagguUugu...............................	15	1	E01
....gugagcaaaguuucaggGgugu...............................	23	1	E01
....gugagcaaaguuucagguCugu...............................	4	1	E01
....gugagcaaaguuucaAgugugu...............................	13	1	E01
....gugagcaaaguCucaggugugu...............................	8	1	E01
....gugagcaaGguuucaggugugu...............................	7	1	E01
....gugagcaaUguuucaggugugu...............................	5	1	E01
....gugagcUaaguuucaggugugu...............................	8	1	E01
....gugagcaaaguuuAaggugugu...............................	14	1	E01
....gugagcaaaguuucaggugGgu...............................	10	1	E01

```
                    Star                                    Mature
ugagagaaugucggccuugugugcccgaaacuuugacucaaccugaguuuaggaaaaggugagcaaaguuucaggugugucggguugacugaaaaaacugguuuaccagga
..........................................................guAagcaaaguuucaggugugu...............................   10      1   E01
..........................................................Augagcaaaguuucaggugugu...............................   2       1   E01
..........................................................gugagcaaaguuucagAugugu...............................   16      1   E01
..........................................................gugagAaaaguuucaggugugu...............................   9       1   E01
..........................................................gugagcaCaguuucaggugugu...............................   2       1   E01
..........................................................gGgagcaaaguuucaggugugu...............................   52      1   E01
..........................................................guCagcaaaguuucaggugugu...............................   15      1   E01
..........................................................gugagcaaaguAucaggugugu...............................   2       1   E01
..........................................................gugagcaaagAuucaggugugu...............................   5       1   E01
..........................................................gugagcaaaguuucagCugugu...............................   29      1   E01
..........................................................gugagcaaaCuuucaggugugu...............................   38      1   E01
..........................................................gugCgcaaaguuucaggugugu...............................   10      1   E01
..........................................................gugagcaaaguuucaggugugCu...............................   88      1   E01
..........................................................gugagcaUaguuucaggugugu...............................   46      1   E01
..........................................................gugagcaaaguuucaggugCgu...............................   18      1   E01
..........................................................gugagcaaaguuucaggugugu...............................   202186  0   E01
..........................................................gugagcaaagGuucaggugugu...............................   11      1   E01
..........................................................gugaCcaaaguuucaggugugu...............................   6       1   E01
..........................................................gugagcaaaguuucCggugugu...............................   18      1   E01
..........................................................gugagcaaaguuucagUugugu...............................   28      1   E01
..........................................................gugagcaaaguuuUaggugugu...............................   104     1   E01
..........................................................gugagcaaCguuucaggugugu...............................   1       1   E01
..........................................................gugagcaaaguuucaggugugA...............................   1256    1   E01
..........................................................gugagcaaaguuucUggugugu...............................   174     1   E01
..........................................................gugagcaaaAuuucaggugugu...............................   2       1   E01
..........................................................gugagcaaaguuuGaggugugu...............................   17      1   E01
..........................................................gugagGaaaguuucaggugugu...............................   5       1   E01
..........................................................gugagcaaaguuucaggugugAu...............................   46      1   E01
..........................................................Cugagcaaaguuucaggugugu...............................   4       1   E01
..........................................................gugagcaaaguGucaggugugu...............................   2       1   E01
..........................................................gugagcaGaguuucaggugugu...............................   18      1   E01
..........................................................gugagcaaaguuucGggugugu...............................   257     1   E01
..........................................................gugagUaaaguuucaggugugu...............................   79      1   E01
..........................................................Uugagcaaaguuucaggugugu...............................   36      1   E01
..........................................................gugagcaaagCuucaggugugu...............................   19      1   E01
..........................................................gugagcaaaguuucaUgugugu...............................   31      1   E01
..........................................................gugagcaaaguuGcaggugugu...............................   15      1   E01
..........................................................gugagcaaaUuuucaggugugu...............................   10      1   E01
..........................................................gugagcaaaguuCcaggugugu...............................   46      1   E01
..........................................................gCgagcaaaguuucaggugugu...............................   27      1   E01
..........................................................guUagcaaaguuucaggugugu...............................   20      1   E01
..........................................................gugagUaaaguuucaggugugUc..............................   2       1   E01
..........................................................gugagcaaaguuucaggugugCc..............................   1       1   E01
..........................................................gugagcaaaguuucagguguguG..............................   41      1   E01
..........................................................gugagcaaaguuucaggugugAc..............................   1       1   E01
..........................................................gugagcaaaguuucaggugugucc..............................   20      0   E01
..........................................................gugagcaaaguuucaggugugucg.............................   8       0   E01
..........................................................gugagcaaaguuucaggugugucC.............................   5       1   E01
..........................................................gugagcaaaguuucaggugugucU.............................   3       1   E01
..........................................................gugagcaaaguuucagguguguGgg............................   1       1   E01
..........................................................gugagcaaaguuucagguguguAgg............................   1       1   E01
..........................................................gugagcaaaguuucagguguguGggg...........................   2       1   E01
...........................................................ugagcaaaguuucaggugu.................................   1       0   E01
...........................................................ugagcaaaguuucaggugugu...............................   46      0   E01
...........................................................Ggagcaaaguuucaggugugu...............................   1       1   E01
...........................................................ugagcaaaguuucagguguguG..............................   1       1   E01
............................................................gagcaaaguuucaggugug................................   4       0   E01
............................................................gagcaaaguuucaggugugA...............................   2       1   E01
............................................................gUgcaaaguuucaggugugu...............................   1       1   E01
............................................................gagAaaaguuucaggugugu...............................   1       1   E01
............................................................gagcUaaguuucaggugugu...............................   1       1   E01
............................................................gGgcaaaguuucaggugugu...............................   1       1   E01
............................................................gagcaaaguuucaggugugu...............................   152     0   E01
............................................................Uagcaaaguuucaggugugu...............................   1       1   E01
.............................................................agcaaaguuucaggugU.................................   2       1   E01
.............................................................agcaaaguuucaggugug................................   2       0   E01
.............................................................agcaaaguuucaggugugu...............................   220     0   E01
.............................................................agcaaaguuucaggugugA...............................   1       1   E01
..............................................................gcaaaguuucaggugugA...............................   2       1   E01
..............................................................gcaaaguuucaggugugu...............................   203     0   E01
```

Star Mature

ugagagaaugucggccuugugugcccgaaacuuugacucaaccugaguuuaggaaaaggugagcaaaguuucaggugugucggguugacugaaaaaacugguuuaccagga

...................Cgugcccgaaacuuugacucaa..	2	1	TA1
....................gugcccgaaacuuugacu...	8	0	TA1
....................gugcccgaaacuuugacuU..	1	1	TA1
....................gugcccgaaacuuugacuc..	28	0	TA1
....................gugcccgaaacuuugacCc..	1	1	TA1
....................gugcccgaaacuuugacuA..	1	1	TA1
....................gugcccgaaacuuugacuca...	84	0	TA1
....................gugcccgaaacuuugacucU...	2	1	TA1
....................Augcccgaaacuuugacuca...	1	1	TA1
....................gugcccgaaacuuugUcuca...	2	1	TA1
....................gugccUgaaacuuugacuca...	1	1	TA1
....................gugcccgaaacuuugacAca...	1	1	TA1
....................gugcccgaaacuuugUcucaa..	4	1	TA1
....................gugcccgaaacuuugacucaa..	55	0	TA1
....................gugcccgaaacuuugacucaU..	1	1	TA1
....................gugcccgaaacuuugacucaG..	2	1	TA1
....................gugcccgaaacuuugacucaaU...	8	1	TA1
....................Augcccgaaacuuugacucaac...	1	1	TA1
....................gugcccgaaacuuugacucaac...	53	0	TA1
....................gugcccgaaacuuugacucGac...	2	1	TA1
....................gugcccgaaacuuugacucaaA...	1	1	TA1
....................gugcccgaaacuuugacucaacc..	114	0	TA1
....................gugcccgaaacuuugUcucaacc..	2	1	TA1
....................gugcccgaaaUuuugacucaacc..	1	1	TA1
....................gugcccgaaacuuuAacucaacc..	1	1	TA1
....................gCgcccgaaacuuugacucaacc..	3	1	TA1
....................gugcccgaaacuCugacucaacc..	1	1	TA1
....................gugcccgGaacuuugacucaacc..	1	1	TA1
....................gugcccgaaacuuugacucaacA..	15	1	TA1
....................gugcccgaaacuuCgacucaacc..	2	1	TA1
....................gugcccgaaacuuugacucaacU..	19	1	TA1
....................gugcccgaaacuuugacucaGcc..	4	1	TA1
....................gugccAaaacuuugacucaaccu...	2	1	TA1
....................Augcccgaaacuuugacucaaccu...	4	1	TA1
....................gugcccgaaacuuugacucGaccu...	6	1	TA1
....................gugcccgaaacuuugacucaaccA...	50	1	TA1
....................gugcccgaGacuuugacucaaccu...	3	1	TA1
....................gugcccgaaacuuugUcucaaccu...	32	1	TA1
....................gugcccgaaacuuugacucaaccG...	16	1	TA1
....................gugcccgaaacAuugacucaaccu...	3	1	TA1
....................gugUccgaaacuuugacucaaccu...	1	1	TA1
....................gugcccgaaacuuugacucaacUu...	1	1	TA1
....................gugcccgGaacuuugacucaaccu...	3	1	TA1
....................gugcUcgaaacuuugacucaaccu...	1	1	TA1
....................gCgcccgaaacuuugacucaaccu...	5	1	TA1
....................gugccUgaaacuuugacucaaccu...	4	1	TA1
....................gAgcccgaaacuuugacucaaccu...	1	1	TA1
....................gugcccgaaacuCugacucaaccu...	2	1	TA1
....................gugcccgaaacuuugacucaGccu...	1	1	TA1
....................gugcccgaaacuuugacucaaccu...	881	0	TA1
....................gugcccgaaacuuCgacucaaccu...	3	1	TA1
....................gugcccgaaaUuuugacucaaccu...	2	1	TA1
....................gugcccgaaGcuuugacucaaccu...	1	1	TA1
....................gugcccgaaacuuugacCcaaccu...	3	1	TA1
....................gugcccgaaacuuugacucaaAcu...	1	1	TA1
....................gugcccgaaacuuugacucaaccC...	104	1	TA1
....................gugcccgaaacuuugacucaaccuC..	2	1	TA1
....................gugcccgaaacuuugacucaaccuA..	1	1	TA1
....................gugcccgaaacuuugacucaaccuU..	4	1	TA1
.....................ugcccgaaacuuugacuc..	1	0	TA1
.....................ugcccgaaacuuugacuca...	3	0	TA1
.....................ugcccgaaacuuugacucaa..	1	0	TA1
.....................ugcccgaaacuuugacucaac...	2	0	TA1
.....................ugcccgaaacuuugacucaacA..	1	1	TA1
.....................ugcccgaaacuuugacucaacc..	3	0	TA1
.....................ugcccgaaacuuugacCcaacc..	2	1	TA1
.....................ugcccgaaacuuugacucaaccA...	8	1	TA1
.....................ugcccgaaacuCugacucaaccu...	1	1	TA1
.....................ugcccgaaacuuugacCcaaccu...	1	1	TA1

```
                    Star                                   Mature
ugagagaaugucggccuugugugcccgaaacuuugacucaaccugaguuuaggaaaaggugagcaaaguuucaggugugucggguugacugaaaaaacugguuuaccagga
.....................ugcccgaaacuuugacucaaccC...................................................................   8      1   TA1
.....................ugcccgaaacuuugacucaaccu...................................................................   36     0   TA1
.....................ugcccgGaacuuugacucaaccu...................................................................   1      1   TA1
.....................ugcccgaaGcuuugacucaaccu...................................................................   1      1   TA1
.....................ugcccgaaacuuugacucaaccuU..................................................................   5      1   TA1
.......................cccgaaacuuugacucaaccu...................................................................   1      0   TA1
........................ccgaaacuuugacucaaccA...................................................................   1      1   TA1
.........................cgaaacuuugacucaaccu...................................................................   1      0   TA1
..........................gaaacuuugacucaaccu...................................................................   3      0   TA1
.........................................................Cgugagcaaaguuucaggugug................................   4      1   TA1
.........................................................ggugagcaaaguuucaggugug................................   1      0   TA1
.........................................................ggugagcaaaguuucagguguA................................   1      1   TA1
.........................................................ggugagcaaaguuucaggugugu...............................   2      0   TA1
.........................................................ggugagcaaaguuCcaggugugu...............................   1      1   TA1
.........................................................Cgugagcaaaguuucaggugugu...............................   8      1   TA1
..........................................................gugagcaaaguuucaggu...................................   23     0   TA1
..........................................................gugagcaaGguuucaggug..................................   2      1   TA1
..........................................................gugagcaaaguuucagguA..................................   4      1   TA1
..........................................................gugagcaaaguCucaggug..................................   2      1   TA1
..........................................................gugagcaaaguuCcaggug..................................   1      1   TA1
..........................................................gugagcaaCguuucaggug..................................   1      1   TA1
..........................................................gugagcaaaguuucaggGg..................................   1      1   TA1
..........................................................gugagcaaaguuucaggug..................................   64     0   TA1
..........................................................Cugagcaaaguuucaggugu.................................   1      1   TA1
..........................................................gugagcaaaguuucaggugu.................................   117    0   TA1
..........................................................gugagcaaaguCucaggugu.................................   1      1   TA1
..........................................................gugagcaaaguuucaggugC.................................   14     1   TA1
..........................................................gugagcaaaguuucaggGgu.................................   2      1   TA1
..........................................................gugagcaaaguuucaggugG.................................   1      1   TA1
..........................................................gugagcaaaguuucGggugu.................................   2      1   TA1
..........................................................gugGgcaaaguuucaggugu.................................   1      1   TA1
..........................................................gugagcaaaguuucaggugug................................   1006   0   TA1
..........................................................gugagcaaaguGucaggugug................................   1      1   TA1
..........................................................gugagcaUaguuucaggugug................................   2      1   TA1
..........................................................gugagcaaaguuucagguguU................................   11     1   TA1
..........................................................gugagcaaaguuucaggugCg................................   5      1   TA1
..........................................................gugagcaCaguuucaggugug................................   1      1   TA1
..........................................................gugagcGaaguuucaggugug................................   3      1   TA1
..........................................................gugagcaaUguuucaggugug................................   1      1   TA1
..........................................................gugagcaaaguuucagAugug................................   2      1   TA1
..........................................................gugagAaaaguuucaggugug................................   2      1   TA1
..........................................................gugagcaGaguuucaggugug................................   4      1   TA1
..........................................................gugagcaaagCuucaggugug................................   3      1   TA1
..........................................................gugagcaaaguuucaggugGg................................   2      1   TA1
..........................................................gugCgcaaaguuucaggugug................................   1      1   TA1
..........................................................gCgagcaaaguuucaggugug................................   4      1   TA1
..........................................................gugagcaaaguuucagguUug................................   1      1   TA1
..........................................................gugagcaaaguuucaUgugug................................   1      1   TA1
..........................................................gGgagcaaaguuucaggugug................................   1      1   TA1
..........................................................gugagcaaaguCucaggugug................................   7      1   TA1
..........................................................gugagcaaaguuucaggCgug................................   4      1   TA1
..........................................................gugagUaaaguuucaggugug................................   1      1   TA1
..........................................................gugagcaaaguuucagguguC................................   3      1   TA1
..........................................................gugagcaaagGuucaggugug................................   1      1   TA1
..........................................................gugagcaaagAuucaggugug................................   1      1   TA1
..........................................................gugGgcaaaguuucaggugug................................   4      1   TA1
..........................................................gugagcaaaguuucaggugAg................................   3      1   TA1
..........................................................Augagcaaaguuucaggugug................................   1      1   TA1
..........................................................gugagcaaaguuucagguguA................................   78     1   TA1
..........................................................gugagcaaGguuucaggugug................................   5      1   TA1
..........................................................gugagcaaaguuucGggugug................................   4      1   TA1
..........................................................gugagcaaaguuucUggugug................................   4      1   TA1
..........................................................gugagcaaaguuucagCugugu...............................   1      1   TA1
..........................................................Augagcaaaguuucaggugugu...............................   20     1   TA1
..........................................................gugCgcaaaguuucaggugugu...............................   2      1   TA1
..........................................................gugagcaaaguuucagguguUu...............................   7      1   TA1
..........................................................gugagcaaaguuucCggugugu...............................   3      1   TA1
..........................................................gugagcaaaguAucaggugugu...............................   5      1   TA1
..........................................................gugagcCaaguuucaggugugu...............................   2      1   TA1
..........................................................gugagcaaaguuucaCgugugu...............................   1      1   TA1
```

```
                    Star                                  Mature
ugagagaaugucggccuugugugcccgaaacuuugacucaaccugaguuuaggaaaaggugagcaaaguuucaggugugucggguugacugaaaaaacugguuuaccagga
..........................................................gugagcaaaguuucagguCugu...............................   2      1    TA1
..........................................................Cugagcaaaguuucaggugugu...............................   1      1    TA1
..........................................................gugagcaaaguuuUaggugugu...............................   12     1    TA1
..........................................................gugagcaaaAuuucaggugugu...............................   10     1    TA1
..........................................................gugagcaaCguuucaggugugu...............................   4      1    TA1
..........................................................gugagcaaaguuucaAgugugu...............................   10     1    TA1
..........................................................gGgagcaaaguuucaggugugu...............................   6      1    TA1
..........................................................guUagcaaaguuucaggugugu...............................   1      1    TA1
..........................................................gugagcaaaguuucaggugugA...............................   490    1    TA1
..........................................................gugagUaaaguuucaggugugu...............................   21     1    TA1
..........................................................guAagcaaaguuucaggugugu...............................   5      1    TA1
..........................................................gugagcaUaguuucaggugugu...............................   13     1    TA1
..........................................................gugagcaaaguuucaggCgugu...............................   54     1    TA1
..........................................................gugagcaaaguuCcaggugugu...............................   76     1    TA1
..........................................................gugagcaCaguuucaggugugu...............................   2      1    TA1
..........................................................gugagcaaaguuucaggugAgu...............................   15     1    TA1
..........................................................gugagcUaaguuucaggugugu...............................   3      1    TA1
..........................................................gugagcaaaguuucagAugugu...............................   13     1    TA1
..........................................................gugaAcaaaguuucaggugugu...............................   12     1    TA1
..........................................................gugagcaaaguuucaggGgugu...............................   19     1    TA1
..........................................................gAgagcaaaguuucaggugugu...............................   12     1    TA1
..........................................................gugagcaaaguuucGggugugu...............................   68     1    TA1
..........................................................gugagcaaaguuAcaggugugu...............................   6      1    TA1
..........................................................gugagcaaaguuucagguAugu...............................   15     1    TA1
..........................................................gugagcaaaguCucaggugugu...............................   57     1    TA1
..........................................................gugUgcaaaguuucaggugugu...............................   15     1    TA1
..........................................................gugagcaaaguuucaggugCgu...............................   40     1    TA1
..........................................................gugagAaaaguuucaggugugu...............................   3      1    TA1
..........................................................gugagcaaagCuucaggugugu...............................   44     1    TA1
..........................................................gugagcaaaguuucaggugugG...............................   258    1    TA1
..........................................................gugagcaaaguuuAaggugugu...............................   1      1    TA1
..........................................................gugagcaaaguuucaggugGgu...............................   25     1    TA1
..........................................................gugagcaaaguuucaggugugu...............................   14761  0    TA1
..........................................................gugagcaGaguuucaggugugu...............................   62     1    TA1
..........................................................gugagcaaaguuucaggAgugu...............................   13     1    TA1
..........................................................gugagcaaUguuucaggugugu...............................   3      1    TA1
..........................................................gugagcaaaguGucaggugugu...............................   1      1    TA1
..........................................................gugagcaaaguuGcaggugugu...............................   1      1    TA1
..........................................................gugagcaaagAuucaggugugu...............................   4      1    TA1
..........................................................Uugagcaaaguuucaggugugu...............................   2      1    TA1
..........................................................gugagcaaGguuucaggugugu...............................   62     1    TA1
..........................................................gugagcaaaUuuucaggugugu...............................   1      1    TA1
..........................................................gugaCcaaaguuucaggugugu...............................   1      1    TA1
..........................................................gugaUcaaaguuucaggugugu...............................   8      1    TA1
..........................................................gCgagcaaaguuucaggugugu...............................   44     1    TA1
..........................................................gugagcaaaguuucagguguAu...............................   14     1    TA1
..........................................................gugGgcaaaguuucaggugugu...............................   49     1    TA1
..........................................................gugagcGaaguuucaggugugu...............................   44     1    TA1
..........................................................gugagcaaaguuucagguguCu...............................   2      1    TA1
..........................................................gugagcaaaguuuGaggugugu...............................   1      1    TA1
..........................................................gugagcaaaguuucUggugugu...............................   14     1    TA1
..........................................................gugagcaaagGuucaggugugu...............................   6      1    TA1
..........................................................gugagcaaaguuucaggugCguc..............................   2      1    TA1
..........................................................gugGgcaaaguuucagguguguc..............................   1      1    TA1
..........................................................gugagcaaaguCucagguguguc..............................   1      1    TA1
..........................................................gugagcaaaguuucagguguguG..............................   29     1    TA1
..........................................................gugagcaaaguuucagguguguc..............................   24     0    TA1
..........................................................gugagcaaaguuucaggugugucggC...........................   1      1    TA1
...........................................................ugagcGaaguuucaggugugu...............................   1      1    TA1
...........................................................ugagcaaaguuucaggugugu...............................   3      0    TA1
............................................................gagcaaaguuucaggugugu...............................   4      0    TA1
..............................................................gcaaaguuucaggugugu...............................   1      0    TA1

....................gugcccgaaacuuCgacu.........................................................................   1      1    LJ1
....................gugcccgaaacuuugacu.........................................................................   12     0    LJ1
....................gugcccgaaacuuugacC.........................................................................   6      1    LJ1
....................gugcccgaaacuuugacA.........................................................................   4      1    LJ1
....................gugcccgaaacuuugacuA........................................................................   3      1    LJ1
....................gugcccgaaacuuugacuU........................................................................   5      1    LJ1
....................gugcccgaaacuuugacAc........................................................................   1      1    LJ1
```

```
                    Star                                    Mature
ugagagaaugucggccuugugugcccgaaacuuugacucaaccugaguuuaggaaaaggugagcaaaguuucaggugugucggguugacugaaaaaacugguuuaccagga
....................gugcccgaGacuuugacuc........................................................................ 1     1   LJ1
....................gugcccgaaacuuugUcuc........................................................................ 3     1   LJ1
....................gugcccgaaacuuGgacuc........................................................................ 1     1   LJ1
....................gugcccgaaacuuugacuc........................................................................ 26    0   LJ1
....................gugcccgaaacuuAgacuca....................................................................... 1     1   LJ1
....................gugcccgaaacuuugacuca....................................................................... 28    0   LJ1
....................gugcccgaaacuuugUcuca....................................................................... 1     1   LJ1
....................gugcccgaaacuuugacCcaa...................................................................... 1     1   LJ1
....................guUcccgaaacuuugacucaa...................................................................... 1     1   LJ1
....................gugcccgaaacuuugacucGa...................................................................... 1     1   LJ1
....................gugcccgaaacuuugacucaa...................................................................... 65    0   LJ1
....................Augcccgaaacuuugacucaac..................................................................... 1     1   LJ1
....................guUcccgaaacuuugacucaac..................................................................... 1     1   LJ1
....................gugcccgaaacuuugacucaac..................................................................... 54    0   LJ1
....................gugcccgaaacuuugacucaaU..................................................................... 4     1   LJ1
....................gugcccgaaacuuugacucaacU.................................................................... 36    1   LJ1
....................gugUccgaaacuuugacucaacc.................................................................... 1     1   LJ1
....................gugcccgaaacuuugacucaacc.................................................................... 95    0   LJ1
....................gugcccgaaGcuuugacucaacc.................................................................... 1     1   LJ1
....................gugccAgaaacuuugacucaacc.................................................................... 1     1   LJ1
....................gugcccgaaacuuugacucaacA.................................................................... 12    1   LJ1
....................gugcUcgaaacuuugacucaaccu................................................................... 3     1   LJ1
....................gugcccgaGacuuugacucaaccu................................................................... 1     1   LJ1
....................gugcccgaaacuuugacucaaccA................................................................... 123   1   LJ1
....................guCcccgaaacuuugacucaaccu................................................................... 2     1   LJ1
....................gugcccgaaacuuAgacucaaccu................................................................... 1     1   LJ1
....................gugccAgaaacuuugacucaaccu................................................................... 1     1   LJ1
....................gugcccgaaacuuugacCcaaccu................................................................... 2     1   LJ1
....................gugcAcgaaacuuugacucaaccu................................................................... 1     1   LJ1
....................gugccUgaaacuuugacucaaccu................................................................... 3     1   LJ1
....................gCgcccgaaacuuugacucaaccu................................................................... 1     1   LJ1
....................gugcccgaaacuuugacucaaccC................................................................... 170   1   LJ1
....................gugUccgaaacuuugacucaaccu................................................................... 5     1   LJ1
....................gugcccgaaacuuugacuUaaccu................................................................... 4     1   LJ1
....................gugcccgaUacuuugacucaaccu................................................................... 1     1   LJ1
....................gugcccgaaacuuugacucCaccu................................................................... 1     1   LJ1
....................gugcccgaaacuuugacucaGccu................................................................... 2     1   LJ1
....................Augcccgaaacuuugacucaaccu................................................................... 2     1   LJ1
....................gugcccCaaacuuugacucaaccu................................................................... 2     1   LJ1
....................gugcccgaaacuuugacucaacUu................................................................... 2     1   LJ1
....................guAcccgaaacuuugacucaaccu................................................................... 1     1   LJ1
....................gugcccgaaacuuugUcucaaccu................................................................... 5     1   LJ1
....................gugcccgaaacAuugacucaaccu................................................................... 1     1   LJ1
....................gugcccgaaacuuugacucaaccG................................................................... 35    1   LJ1
....................gugcccgaaacuCugacucaaccu................................................................... 2     1   LJ1
....................gugcccgaaacuuugacucGaccu................................................................... 5     1   LJ1
....................gugcccgaaacuuugacucaaccu................................................................... 1139  0   LJ1
....................guUcccgaaacuuugacucaaccu................................................................... 2     1   LJ1
....................gugcccgaaacuuCgacucaaccu................................................................... 1     1   LJ1
....................gugcccgaaacuuugacucaacAu................................................................... 1     1   LJ1
....................gugcccgaaacuGugacucaaccu................................................................... 2     1   LJ1
....................gugcccgaaacuuugacucaaUcu................................................................... 1     1   LJ1
....................gugcccgaaGcuuugacucaaccu................................................................... 3     1   LJ1
....................gugcccgUaacuuugacucaaccu................................................................... 1     1   LJ1
....................gugcccgaaacuuugacucaaccuA.................................................................. 2     1   LJ1
....................gugcccgaaacuuugacucaaccuC.................................................................. 3     1   LJ1
....................gugcccgaaacuuugacucaaccuU.................................................................. 16    1   LJ1
....................gugcccgaaacuuugacucaaccug.................................................................. 1     0   LJ1
....................gugcccgaaacuuugacucaaccuAa................................................................. 1     1   LJ1
.....................ugcccgaaacuuugacucaac..................................................................... 1     0   LJ1
.....................ugcccgaaacuuugacucaaU..................................................................... 1     1   LJ1
.....................ugcccgaaacuuugacucaacc.................................................................... 1     0   LJ1
.....................ugcccgaaacuuugacucaacA.................................................................... 1     1   LJ1
.....................ugcccgaaacuuugacucaacU.................................................................... 1     1   LJ1
.....................ugUccgaaacuuugacucaacc.................................................................... 1     1   LJ1
.....................ugcccgaaacuuugacucaaccC................................................................... 3     1   LJ1
.....................ugcccgaaacuuugacucaaccu................................................................... 19    0   LJ1
.....................ugcccgaaacuuugacucaaccA................................................................... 2     1   LJ1
.....................ugcccgaaacuuugacucaaccuU.................................................................. 2     1   LJ1
........................ccgaaacuuugacucaaccu................................................................... 3     0   LJ1
```

```
                    Star                                  Mature
ugagagaaugucggccuugugugcccgaaacuuugacucaaccugaguuuaggaaaaggugagcaaaguuucaggugugucggguugacugaaaaaacugguuuaccagga
..........................gaaacuuugacucaaccu...................................................................  1     0  LJ1
........................................................aAgugagcaaaguuucaggugugu...............................  1     1  LJ1
.........................................................ggugagcaaaguuucagguA..................................  1     1  LJ1
.........................................................Cgugagcaaaguuucaggugug................................  3     1  LJ1
.........................................................ggugagcaaaguuucaggugug................................  1     0  LJ1
.........................................................ggugagcaaaguuucaggugugA...............................  1     1  LJ1
.........................................................Cgugagcaaaguuucaggugugu...............................  6     1  LJ1
.........................................................ggugagcaaaguuucaggugugu...............................  2     0  LJ1
..........................................................gugagcaaaguuucaggC...................................  5     1  LJ1
..........................................................gugagcaaaguuucaggA...................................  1     1  LJ1
..........................................................gugagcaaaguuucaggu...................................  33    0  LJ1
..........................................................gugagcaaaguuucaAgu...................................  1     1  LJ1
..........................................................gugGgcaaaguuucaggu...................................  1     1  LJ1
..........................................................gugagcaaaguuucaggug..................................  144   0  LJ1
..........................................................gugagcaaaguuucagguU..................................  7     1  LJ1
..........................................................gugagcaaGguuucaggug..................................  2     1  LJ1
..........................................................gugagAaaaguuucaggug..................................  1     1  LJ1
..........................................................gugagcaaaguuCcaggug..................................  1     1  LJ1
..........................................................gugagcaaaguuucagguA..................................  16    1  LJ1
..........................................................gugagcaaaguuucaggCg..................................  1     1  LJ1
..........................................................gugagcCaaguuucaggug..................................  1     1  LJ1
..........................................................guUagcaaaguuucaggug..................................  1     1  LJ1
..........................................................gugagcaaaguuucaggAg..................................  1     1  LJ1
..........................................................gugagcGaaguuucaggugu.................................  1     1  LJ1
..........................................................gugagcaaaguuucaggugC.................................  30    1  LJ1
..........................................................gugagcaGaguuucaggugu.................................  2     1  LJ1
..........................................................gugagcaUaguuucaggugu.................................  1     1  LJ1
..........................................................gugagcaaaguuucaggugu.................................  249   0  LJ1
..........................................................gugagcaaaguuucGggugu.................................  1     1  LJ1
..........................................................gugagcaaGguuucaggugu.................................  1     1  LJ1
..........................................................gCgagcaaaguuucaggugu.................................  1     1  LJ1
..........................................................gugGgcaaaguuucaggugu.................................  3     1  LJ1
..........................................................gugagcaaaguCucaggugu.................................  2     1  LJ1
..........................................................gugagcaaaguuCcaggugu.................................  1     1  LJ1
..........................................................gugagcaaaguuucaggugG.................................  12    1  LJ1
..........................................................gugagcaaaguuucaggGgu.................................  2     1  LJ1
..........................................................gugagcaaagCuucaggugu.................................  1     1  LJ1
..........................................................gugagcaaCguuucaggugug................................  1     1  LJ1
..........................................................gugagcaaaguuucaggugGg................................  4     1  LJ1
..........................................................gugagcaaaguuucaAgugug................................  1     1  LJ1
..........................................................gugagcaaaguuucaggGgug................................  4     1  LJ1
..........................................................guCagcaaaguuucaggugug................................  3     1  LJ1
..........................................................gugagcaaaguuucaggugAg................................  3     1  LJ1
..........................................................gugagcaGaguuucaggugug................................  4     1  LJ1
..........................................................gugagcaaaAuuucaggugug................................  1     1  LJ1
..........................................................gCgagcaaaguuucaggugug................................  3     1  LJ1
..........................................................gGgagcaaaguuucaggugug................................  1     1  LJ1
..........................................................gugagUaaaguuucaggugug................................  2     1  LJ1
..........................................................gugagcaaaguuucagguguA................................  200   1  LJ1
..........................................................gugagcaaaguuucaggugCg................................  6     1  LJ1
..........................................................gugagcaaagGuucaggugug................................  4     1  LJ1
..........................................................Uugagcaaaguuucaggugug................................  3     1  LJ1
..........................................................gugagcaaaguuucagguAug................................  2     1  LJ1
..........................................................gugagcaaaguuucGggugug................................  7     1  LJ1
..........................................................gugagcaaaguCucaggugug................................  3     1  LJ1
..........................................................gugagcaaaguuucaUgugug................................  6     1  LJ1
..........................................................gugagcCaaguuucaggugug................................  1     1  LJ1
..........................................................gugagcaaaguuucaggugug................................  1724  0  LJ1
..........................................................Augagcaaaguuucaggugug................................  8     1  LJ1
..........................................................gugagcaaagCuucaggugug................................  3     1  LJ1
..........................................................gugaAcaaaguuucaggugug................................  1     1  LJ1
..........................................................gugagcaaaguuucaggugugU...............................  49    1  LJ1
..........................................................gugagcaaUguuucaggugug................................  2     1  LJ1
..........................................................gugagcaaaguuucUggugug................................  1     1  LJ1
..........................................................gugagcaaaguuucaggCgug................................  3     1  LJ1
..........................................................guAagcaaaguuucaggugug................................  5     1  LJ1
..........................................................gugagcaaaguuucaggugugC...............................  11    1  LJ1
..........................................................gugagcaaaguuCcaggugug................................  5     1  LJ1
..........................................................gugagcUaaguuucaggugug................................  2     1  LJ1
..........................................................gugagcaaaguuAcaggugug................................  1     1  LJ1
```

Star Mature

```
ugagagaaugucggccuugugugcccgaaacuuugacucaaccugaguuuaggaaaaggugagcaaaguuucaggugugucgguugacugaaaaaacugguuuaccagga
..........................................................gugagcGaaguuucaggugug...............................    7      1    LJ1
..........................................................gugagcaCaguuucaggugug...............................    1      1    LJ1
..........................................................gugagcaaaguuuUaggugug...............................    2      1    LJ1
..........................................................gugagcaaaguuucagAugug...............................    1      1    LJ1
..........................................................guUagcaaaguuucaggugug...............................    6      1    LJ1
..........................................................gugGgcaaaguuucaggugug...............................    7      1    LJ1
..........................................................gugagcaaGguuucaggugug...............................    5      1    LJ1
..........................................................gugagcaUaguuucaggugugu..............................    4      1    LJ1
..........................................................guUagcaaaguuucaggugugu..............................    22     1    LJ1
..........................................................gugagcaaaAuuucaggugugu..............................    3      1    LJ1
..........................................................gugagcaaagAuucaggugugu..............................    2      1    LJ1
..........................................................gugagcaaaguuucGggugugu..............................    26     1    LJ1
..........................................................gugagcaaaguuucagguCugu..............................    1      1    LJ1
..........................................................gAgagcaaaguuucaggugugu..............................    4      1    LJ1
..........................................................gugagcaaaguuGcaggugugu..............................    2      1    LJ1
..........................................................gugagcaaaguuucagguguAu..............................    6      1    LJ1
..........................................................guCagcaaaguuucaggugugu..............................    12     1    LJ1
..........................................................gugagcaaaguuucaggAgugu..............................    3      1    LJ1
..........................................................gugagcaaaguuucagguguCu..............................    6      1    LJ1
..........................................................gugagcaGaguuucaggugugu..............................    31     1    LJ1
..........................................................gugagcaaaUuuucaggugugu..............................    2      1    LJ1
..........................................................gugagcCaaguuucaggugugu..............................    4      1    LJ1
..........................................................gugagcaaaguuucagAugugu..............................    8      1    LJ1
..........................................................gugagcaaaguuucaggugGgu..............................    7      1    LJ1
..........................................................gugagcaaCguuucaggugugu..............................    1      1    LJ1
..........................................................gugCgcaaaguuucaggugugu..............................    1      1    LJ1
..........................................................gugagGaaaguuucaggugugu..............................    2      1    LJ1
..........................................................gugagcaaaguuucaAgugugu..............................    7      1    LJ1
..........................................................gugagcaaaguuucaggugCgu..............................    32     1    LJ1
..........................................................gugagcaaaguuucagguguUu..............................    15     1    LJ1
..........................................................gugaAcaaaguuucaggugugu..............................    4      1    LJ1
..........................................................gugUgcaaaguuucaggugugu..............................    2      1    LJ1
..........................................................gugagcaaaguuucaggugugA..............................    311    1    LJ1
..........................................................guAagcaaaguuucaggugugu..............................    37     1    LJ1
..........................................................gugagcaaaguuuAaggugugu..............................    3      1    LJ1
..........................................................gugagcUaaguuucaggugugu..............................    1      1    LJ1
..........................................................gugagUaaaguuucaggugugu..............................    16     1    LJ1
..........................................................gugagcaaaguuucUggugugu..............................    5      1    LJ1
..........................................................gugagcaaaguuucaggugugG..............................    137    1    LJ1
..........................................................gugagcaaGguuucaggugugu..............................    41     1    LJ1
..........................................................gugagcaaaguuAcaggugugu..............................    3      1    LJ1
..........................................................gugagcaaaguuucaggCgugu..............................    28     1    LJ1
..........................................................gugagcaaaguuCcaggugugu..............................    15     1    LJ1
..........................................................gugaUcaaaguuucaggugugu..............................    2      1    LJ1
..........................................................gugagcaaaguuucaggGgugu..............................    5      1    LJ1
..........................................................gugagcaaaguuucagguUugu..............................    1      1    LJ1
..........................................................gugagcaaaguCucaggugugu..............................    21     1    LJ1
..........................................................gugagcaaaCuuucaggugugu..............................    1      1    LJ1
..........................................................gugagcaaaguuucaggugugu..............................    7615   0    LJ1
..........................................................gugagcGaaguuucaggugugu..............................    31     1    LJ1
..........................................................gugagcaaaguuuUaggugugu..............................    4      1    LJ1
..........................................................gugagcaaaguuucCggugugu..............................    1      1    LJ1
..........................................................gugagcaaagCuucaggugugu..............................    16     1    LJ1
..........................................................gugagcaaaguuuGaggugugu..............................    1      1    LJ1
..........................................................Uugagcaaaguuucaggugugu..............................    3      1    LJ1
..........................................................gCgagcaaaguuucaggugugu..............................    16     1    LJ1
..........................................................gugagAaaaguuucaggugugu..............................    5      1    LJ1
..........................................................gugagcaaaguuucagguAugu..............................    1      1    LJ1
..........................................................gugGgcaaaguuucaggugugu..............................    29     1    LJ1
..........................................................gugagcaaaguuucaggugAgu..............................    4      1    LJ1
..........................................................gugagcaaaguAucaggugugu..............................    1      1    LJ1
..........................................................Augagcaaaguuucaggugugu..............................    8      1    LJ1
..........................................................gugagcaaaguuucagguguguc.............................    30     0    LJ1
..........................................................gugagcaaaguuucagguguguG.............................    76     1    LJ1
..........................................................gugagcaaaguuucagguguguUg............................    1      1    LJ1
...........................................................ugagcaaaguuucaggugugu..............................    4      0    LJ1
...........................................................ugagcaaaguuucagguguguc.............................    1      0    LJ1

....................gugcccgaaacuuugacu........................................................................    1      0    G01
....................gugcccgaaacuuugacuca......................................................................    11     0    G01
```

```
                    Star                                  Mature
ugagagaaugucggccuuguGugcccgaaacuuugacucaaccugaguuuaggaaaaggugagcaaaguuucaggugugucggguugacugaaaaaacugguuuaccagga
....................gugcccgaaacuuugacucaa......................................................................    9     0    G01
....................gugcccgaaacuuugacucaac.....................................................................    9     0    G01
....................gugcccgaaacuCugacucaacc....................................................................    1     1    G01
....................gugcccgaaacuuugacuUaacc....................................................................    1     1    G01
....................gugcccgaaacuuugacucaacc....................................................................    35    0    G01
....................gugcccgaaacuuugUcucaaccu...................................................................    5     1    G01
....................gugcccgaaacuuugaUucaaccu...................................................................    2     1    G01
....................gugcccgaaacuuugacucaaccC...................................................................    1     1    G01
....................gugcccgaaacuuugacucaaccA...................................................................    2     1    G01
....................gugcccgaaacuuugacucaaccu...................................................................    375   0    G01
....................gugcccgaaacuuCgacucaaccu...................................................................    1     1    G01
....................gugcccgaaacuuugacucaaccuU..................................................................    10    1    G01
....................gugcccgaaacuuugacucaaccuA..................................................................    3     1    G01
.....................ugcccgaaacuuugacucaacc....................................................................    2     0    G01
.....................ugcccgaaacuuugUcucaaccu...................................................................    1     1    G01
.....................ugcccgaaacuuugacucaaccu...................................................................    24    0    G01
.....................ugcccgaaacuuugacucaaccuU..................................................................    3     1    G01
........................ccgaaacuuugacucaaccu...................................................................    1     0    G01
......................................................aaGggugagcaaaguuucaggugugu...............................    4     1    G01
.......................................................aaUgugagcaaaguuucaggugugu...............................    1     1    G01
.......................................................aGggugagcaaaguuucaggugugu...............................    3     1    G01
........................................................aggugagcaaaguuucaggugug................................    2     0    G01
........................................................aAgugagcaaaguuucaggugugu...............................    1     1    G01
........................................................Cggugagcaaaguuucaggugugu...............................    1     1    G01
........................................................Gggugagcaaaguuucaggugugu...............................    1     1    G01
........................................................aUgugagcaaaguuucaggugugu...............................    2     1    G01
........................................................aggugagcaaaguuucaggugugu...............................    1     0    G01
.........................................................ggugagcaaaguuucaggu...................................    2     0    G01
.........................................................Ugugagcaaaguuucaggug..................................    1     1    G01
.........................................................Ugugagcaaaguuucaggugu.................................    4     1    G01
.........................................................ggugagcaaaguuucaggugu.................................    6     0    G01
.........................................................ggugagcaaaguuucaggugug................................    13    0    G01
.........................................................ggugagcaaaguuucagguguU................................    2     1    G01
.........................................................Ugugagcaaaguuucaggugug................................    2     1    G01
.........................................................Agugagcaaaguuucaggugug................................    1     1    G01
.........................................................ggugagcaaaguuucagguguAu...............................    2     1    G01
.........................................................ggGgagcaaaguuucaggugugu...............................    1     1    G01
.........................................................ggugagcaaaguuucaggugugu...............................    27    0    G01
.........................................................ggugagcaaaguuucaggugugA...............................    1     1    G01
.........................................................ggugagcaaaguuucagguguUu...............................    1     1    G01
.........................................................Cgugagcaaaguuucaggugugu...............................    5     1    G01
.........................................................Agugagcaaaguuucaggugugu...............................    1     1    G01
.........................................................Ugugagcaaaguuucaggugugu...............................    127   1    G01
..........................................................gugagcaaaguuucGggu...................................    2     1    G01
..........................................................gugagcaaaguuucaggu...................................    576   0    G01
..........................................................gugagcaaaguuucCggu...................................    3     1    G01
..........................................................gugagcaaaguuucaggA...................................    11    1    G01
..........................................................gugaAcaaaguuucaggu...................................    1     1    G01
..........................................................gugagcaaaguuucaggC...................................    1     1    G01
..........................................................gugagcaUaguuucaggu...................................    1     1    G01
..........................................................gugagcaaaCuuucaggu...................................    1     1    G01
..........................................................gugagcaaaguuucaggug..................................    197   0    G01
..........................................................gugagcaaaguuucagguA..................................    6     1    G01
..........................................................gugagcaaaguuuUaggug..................................    1     1    G01
..........................................................gugagcaaaguuucCggug..................................    5     1    G01
..........................................................gugagcaaaguuucagguU..................................    3     1    G01
..........................................................gugagcaaagGuucaggug..................................    1     1    G01
..........................................................gugagcaaagCuucaggugu.................................    2     1    G01
..........................................................gugagcaaaguuGcaggugu.................................    1     1    G01
..........................................................gugUgcaaaguuucaggugu.................................    1     1    G01
..........................................................gugagcaaaguuucagguAu.................................    1     1    G01
..........................................................gugaAcaaaguuucaggugu.................................    1     1    G01
..........................................................gugagcaaagGuucaggugu.................................    2     1    G01
..........................................................gugagcaaaguuucagguUu.................................    1     1    G01
..........................................................gugagcaaaguuucaggGgu.................................    2     1    G01
..........................................................gugagcaaaguuCcaggugu.................................    2     1    G01
..........................................................gugagcaaaguuuUaggugu.................................    2     1    G01
..........................................................gAgagcaaaguuucaggugu.................................    2     1    G01
..........................................................gugagcaaaguuucGggugu.................................    6     1    G01
..........................................................gugagcaaaguuucaggugC.................................    2     1    G01
```

```
                    Star                                   Mature
ugagagaaugucggccuugugugcccgaaacuuugacucaaccugaguuuaggaaaaggugagcaaaguuucaggugugucggguugacugaaaaaacugguuuaccagga
..........................................................gugagcaaGguuucaggugu.................................   1        1    G01
..........................................................gugagcaaaguuuGaggugu.................................   1        1    G01
..........................................................gugagcaaaguuucUggugu.................................   4        1    G01
..........................................................gugagcaaCguuucaggugu.................................   1        1    G01
..........................................................gugagcaaaguuucaggugu.................................   1944     0    G01
..........................................................gugagcaaaguuucagguAug................................   1        1    G01
..........................................................gugagcaaaguuucagguUug................................   1        1    G01
..........................................................gugagcaaaCuuucaggugug................................   3        1    G01
..........................................................gugaUcaaaguuucaggugug................................   1        1    G01
..........................................................gAgagcaaaguuucaggugug................................   6        1    G01
..........................................................gugagcaUaguuucaggugug................................   3        1    G01
..........................................................gugGgcaaaguuucaggugug................................   2        1    G01
..........................................................Uugagcaaaguuucaggugug................................   2        1    G01
..........................................................gugagcaaaguuuAaggugug................................   1        1    G01
..........................................................gugagcaaaguuCcaggugug................................   3        1    G01
..........................................................gugaAcaaaguuucaggugug................................   1        1    G01
..........................................................gugagcaaaguuucaggugCg................................   2        1    G01
..........................................................gugagcaaaguuucaggugugA................................   20       1    G01
..........................................................gCgagcaaaguuucaggugug................................   1        1    G01
..........................................................gugagcaaaguuucaggAgug................................   1        1    G01
..........................................................gugagcaaaguuuUaggugug................................   7        1    G01
..........................................................gGgagcaaaguuucaggugug................................   1        1    G01
..........................................................gugagcaGaguuucaggugug................................   3        1    G01
..........................................................gugagcaaaguuucaggGgug................................   2        1    G01
..........................................................gugUgcaaaguuucaggugug................................   1        1    G01
..........................................................gugagcaaaguuucaggugugU................................   651      1    G01
..........................................................gugagcaaaguuucGgugug................................   8        1    G01
..........................................................gugagcaaaguuucUggugug................................   5        1    G01
..........................................................gugagcaaaguuucCggugug................................   1        1    G01
..........................................................gugagcaaaguuucaggugug................................   6919     0    G01
..........................................................gugagcaaagGuucaggugug................................   1        1    G01
..........................................................gugagcaaaguuucagCugug................................   2        1    G01
..........................................................gugagUaaaguuucaggugug................................   4        1    G01
..........................................................gugagcaaaguuucaggugGg................................   1        1    G01
..........................................................gugagcaaaguuucagAugug................................   1        1    G01
..........................................................gugagAaaaguuucaggugug................................   1        1    G01
..........................................................gugagcaaaguuucaggugugu...............................   127925   0    G01
..........................................................gugagcaaaguuucaggugGgu...............................   7        1    G01
..........................................................gugagcaaaguuucaggAgugu...............................   2        1    G01
..........................................................gugagcaaaguuucagguguUu...............................   1214     1    G01
..........................................................gugagcaaGguuucaggugugu...............................   3        1    G01
..........................................................gugagcaaaguuucUggugugu...............................   77       1    G01
..........................................................gugagcaaaguuucagCugugu...............................   21       1    G01
..........................................................gugagcaaagCuucaggugugu...............................   10       1    G01
..........................................................Cugagcaaaguuucaggugugu...............................   1        1    G01
..........................................................guCagcaaaguuucaggugugu...............................   5        1    G01
..........................................................gugaAcaaaguuucaggugugu...............................   30       1    G01
..........................................................gugagcaaaguuucagAugugu...............................   8        1    G01
..........................................................gugagcaaagGuucaggugugu...............................   38       1    G01
..........................................................gugagcUaaguuucaggugugu...............................   3        1    G01
..........................................................gugaCcaaaguuucaggugugu...............................   3        1    G01
..........................................................gugagcaGaguuucaggugugu...............................   11       1    G01
..........................................................gugagcaaUguuucaggugugu...............................   2        1    G01
..........................................................gugagcaUaguuucaggugugu...............................   24       1    G01
..........................................................gugagAaaaguuucaggugugu...............................   6        1    G01
..........................................................gugagcaaaUuuucaggugugu...............................   2        1    G01
..........................................................gugagcaaaguuucaCgugugu...............................   10       1    G01
..........................................................gugUgcaaaguuucaggugugu...............................   28       1    G01
..........................................................gugagcaaaguGucaggugugu...............................   9        1    G01
..........................................................gugagcaaaguuucGggugugu...............................   190      1    G01
..........................................................guAagcaaaguuucaggugugu...............................   5        1    G01
..........................................................gugagcaaCguuucaggugugu...............................   2        1    G01
..........................................................gugagcaaaguuucaggugCgu...............................   13       1    G01
..........................................................gugagcaaaguuucagUugugu...............................   18       1    G01
..........................................................gugagcaaaguuucaggugugA...............................   1236     1    G01
..........................................................gugagUaaaguuucaggugugu...............................   77       1    G01
..........................................................gugGgcaaaguuucaggugugu...............................   28       1    G01
..........................................................gugagcaaaguuucaUgugugu...............................   17       1    G01
..........................................................gugagcaaaguuucagguAugu...............................   12       1    G01
..........................................................gugagcaaaguuCcaggugugu...............................   40       1    G01
```

```
                    Star                                Mature
ugagagaaugucggccuugugugcccgaaacuuugacucaaccugaguuuaggaaaaggugagcaaaguuucaggugugucggguugacugaaaaaacugguuuaccagga
..........................................................gugagcaaaguuuAaggugugu...............................  5    1  G01
..........................................................gugaUcaaaguuucaggugugu...............................  9    1  G01
..........................................................gugagcaaaguuGcaggugugu...............................  6    1  G01
..........................................................gugagcaaaguuucagguguAu...............................  40   1  G01
..........................................................gugagcaaaguuAcaggugugu...............................  3    1  G01
..........................................................gugagcaaaguuucaggCgugu...............................  3    1  G01
..........................................................gugagcaaagAuucaggugugu...............................  2    1  G01
..........................................................guUagcaaaguuucaggugugu...............................  7    1  G01
..........................................................gugagcaaaguuucagguCugu...............................  9    1  G01
..........................................................gugagcaaaguuuUaggugugu...............................  139  1  G01
..........................................................gugagcCaaguuucaggugugu...............................  1    1  G01
..........................................................gugagcaaaguuucaggugugG...............................  68   1  G01
..........................................................Uugagcaaaguuucaggugugu...............................  30   1  G01
..........................................................gugagcaaaguuucaAgugugu...............................  14   1  G01
..........................................................gugagcaaaguuucaggugAgu...............................  6    1  G01
..........................................................gCgagcaaaguuucaggugugu...............................  24   1  G01
..........................................................gugagcaaaguuucCggugugu...............................  10   1  G01
..........................................................gugagcGaaguuucaggugugu...............................  8    1  G01
..........................................................gAgagcaaaguuucaggugugu...............................  50   1  G01
..........................................................gugagGaaaguuucaggugugu...............................  2    1  G01
..........................................................gugagcaaaguuuGaggugugu...............................  24   1  G01
..........................................................gGgagcaaaguuucaggugugu...............................  30   1  G01
..........................................................gugagcaaaguuucagguUugu...............................  9    1  G01
..........................................................gugCgcaaaguuucaggugugu...............................  6    1  G01
..........................................................gugagcaaaguCucaggugugu...............................  10   1  G01
..........................................................Augagcaaaguuucaggugugu...............................  3    1  G01
..........................................................gugagcaaaCuuucaggugugu...............................  34   1  G01
..........................................................gugagcaaaguuucagguguCu...............................  26   1  G01
..........................................................gugagcaaaguuucaggGgugu...............................  15   1  G01
..........................................................gugagcaaaguuucaggugugucc..............................  25   0  G01
..........................................................gugagcaaaguuucaggugugugG..............................  28   1  G01
..........................................................gugagcaaaguuucaggugugucC.............................  16   1  G01
..........................................................gugagcaaaguuucaggugugugGg.............................  1    1  G01
..........................................................gugagcaaaguuucaggugugucg.............................  7    0  G01
..........................................................gugagcaaaguuucaggugugucU.............................  3    1  G01
..........................................................gugagcaaaguuucaggugugucUg............................  1    1  G01
..........................................................gugagcaaaguuucaggugugugGgg............................  1    1  G01
..........................................................gugagcaaaguuucaggugugucgUg...........................  1    1  G01
..........................................................gugagcaaaguuucaggugugucgggu..........................  2    0  G01
...........................................................ugagcaaaguuucaggugug................................  1    0  G01
...........................................................ugagcaaaguuucaggugugu...............................  35   0  G01
...........................................................Ggagcaaaguuucaggugugu...............................  2    1  G01
...........................................................ugagcaaaguuucaggugugucc..............................  2    0  G01
............................................................Cagcaaaguuucaggugu.................................  1    1  G01
............................................................gagcaaaguuucaggugug................................  4    0  G01
............................................................Cagcaaaguuucaggugugu...............................  1    1  G01
............................................................gagcaaaguuucaggugugA...............................  1    1  G01
............................................................gagcaaaguuucaggugugu...............................  83   0  G01
............................................................Uagcaaaguuucaggugugu...............................  3    1  G01
.............................................................agcaaaguuucaggugug................................  6    0  G01
.............................................................agcaaaguuucaggugugA...............................  2    1  G01
.............................................................agcaaaguuucaggugugu...............................  116  0  G01
.............................................................Ggcaaaguuucaggugugu...............................  1    1  G01
..............................................................gcaaaguuucaggugugA...............................  1    1  G01
..............................................................gcaaaguuuUaggugugu...............................  1    1  G01
..............................................................gcaaaguuucaggugugu...............................  95   0  G01
..............................................................gcaaaguuucaggugugugG..............................  1    1  G01
................................................................aaaguuucaggugugugGggg...........................  1    1  G01

...................Cgugcccgaaacuuugacucaaccu...................................................................  1    1  CJ1
....................guAcccgaaacuuugacu.........................................................................  1    1  CJ1
....................gCgcccgaaacuuugacu.........................................................................  1    1  CJ1
....................Augcccgaaacuuugacu.........................................................................  1    1  CJ1
....................gugcccgaaacuuugaUu.........................................................................  1    1  CJ1
....................gugcccgaaacuuugacC.........................................................................  4    1  CJ1
....................gugcccgaaacuuugacA.........................................................................  4    1  CJ1
....................gugcccgaaacuuugacu.........................................................................  32   0  CJ1
....................gugcccgaaacuuugacuc........................................................................  25   0  CJ1
....................gugcccgaaacuuugacuA........................................................................  3    1  CJ1
....................gCgcccgaaacuuugacuc........................................................................  1    1  CJ1
```

Star **Mature**

ugagagaaugucggccuuguguggcccgaaacuuugacucaaccugaguuuaggaaaaggugagcaaaguuucaggugugucggguugacugaaaaacugguuuaccagga			
....................gugcccgaaacuuugacuU...	3	1	CJ1
....................gugcccgaaacuuugacucG..	2	1	CJ1
....................gugcccAaaacuuugacuca..	1	1	CJ1
....................gugcccgaaacuuugUcuca..	1	1	CJ1
....................gugcccgaaacuuugacuAa..	2	1	CJ1
....................gugcccgaaacuuugacuca..	54	0	CJ1
....................gugcccgaaacuuCgacucaa...	2	1	CJ1
....................gCgcccgaaacuuugacucaa...	1	1	CJ1
....................gugcccgaaacuuugaUucaa...	1	1	CJ1
....................gugcccgaaacuuugacucaa...	118	0	CJ1
....................gugcccAaaacuuugacucaa...	1	1	CJ1
....................guAcccgaaacuuugacucaa...	1	1	CJ1
....................gugGccgaaacuuugacucaa...	1	1	CJ1
....................gugcccgaaacuuugUcucaa...	3	1	CJ1
....................gugcccgaaGcuuugacucaa...	2	1	CJ1
....................gugcccgaaacuuugUcucaac..	1	1	CJ1
....................guUcccgaaacuuugacucaac..	1	1	CJ1
....................gugcccgaaacuuugacucaaA..	1	1	CJ1
....................gugcccgaaacuuCgacucaac..	1	1	CJ1
....................guCcccgaaacuuugacucaac..	1	1	CJ1
....................gugcccgaaacuuugacucaac..	86	0	CJ1
....................gugcccgaaacuuugacucaaU..	2	1	CJ1
....................guAcccgaaacuuugacucaacc...	1	1	CJ1
....................gugcccgaaacuuugacucaacU...	34	1	CJ1
....................gugcccgGaacuuugacucaacc...	1	1	CJ1
....................gugcccgaaacuuugacucGacc...	1	1	CJ1
....................gugcccgaaacuuugacucaacG...	1	1	CJ1
....................gugcccgaaacuuugacucaacc...	139	0	CJ1
....................gugcccgaaacuuugacucaacA...	15	1	CJ1
....................gugcccgaaaUuuugacucaacc...	1	1	CJ1
....................gugcccAaaacuuugacucaacc...	1	1	CJ1
....................gugcccgaaGcuuugacucaacc...	2	1	CJ1
....................gugcccgaaacuuugUcucaacc...	1	1	CJ1
....................gugcccgaaacuAugacucaaccu..	1	1	CJ1
....................gugcccgaaaUuuugacucaaccu..	1	1	CJ1
....................gugcccgaaacuuugUcucaaccu..	9	1	CJ1
....................gCgcccgaaacuuugacucaaccu..	3	1	CJ1
....................gugcccgaaacuuugacCcaaccu..	3	1	CJ1
....................gugUccgaaacuuugacucaaccu..	3	1	CJ1
....................gugcccgaaacuuugacAcaaccu..	1	1	CJ1
....................gugccUgaaacuuugacucaaccu..	6	1	CJ1
....................gugcccgaaacuuugacuUaaccu..	3	1	CJ1
....................gugcccgaaGcuuugacucaaccu..	2	1	CJ1
....................gugcccgaaacuuugacucaaccG..	41	1	CJ1
....................gugcccAaaacuuugacucaaccu..	3	1	CJ1
....................gugcccgaaacuuCgacucaaccu..	3	1	CJ1
....................gugcccgaaacuuugacucaGccu..	6	1	CJ1
....................gugcccCaaacuuugacucaaccu..	1	1	CJ1
....................gugcccgaaacuuugacucaaccu..	1796	0	CJ1
....................gugcUcgaaacuuugacucaaccu..	2	1	CJ1
....................gugcccgaaacuuugacucaaccC..	261	1	CJ1
....................gugccAgaaacuuugacucaaccu..	2	1	CJ1
....................gugcccgaaacuuugacucGaccu..	10	1	CJ1
....................Augcccgaaacuuugacucaaccu..	1	1	CJ1
....................gugcccgaaacuuugacucaaAcu..	1	1	CJ1
....................gugcccgaaacuGugacucaaccu..	1	1	CJ1
....................gugcccgaaacuuugaUucaaccu..	3	1	CJ1
....................gugcccgaaacuuAgacucaaccu..	1	1	CJ1
....................gugcccgaGacuuugacucaaccu..	7	1	CJ1
....................gugcccgaaacuuugacucaaUcu..	2	1	CJ1
....................gugcccgaaacuuugacucCaccu..	1	1	CJ1
....................guUcccgaaacuuugacucaaccu..	6	1	CJ1
....................gugcccgaCacuuugacucaaccu..	1	1	CJ1
....................gugcccgaaacuuugacucaaccA..	144	1	CJ1
....................gugcccgGaacuuugacucaaccu..	5	1	CJ1
....................guCcccgaaacuuugacucaaccu..	3	1	CJ1
....................guAcccgaaacuuugacucaaccu..	9	1	CJ1
....................gugcccgaaacuCugacucaaccu..	1	1	CJ1
....................gugcccgaaacuuugacucaaccuU...	27	1	CJ1
....................gugcccgaaacuuugacucaaccuA...	8	1	CJ1

```
                    Star                                  Mature
ugagagaaugucggccuugugugcccgaaacuuugacucaaccugaguuuaggaaaaggugagcaaaguuucaggugugucggguugacugaaaaaacugguuuaccagga
....................gugcccgaaacuuugacucaaccuC..................................................................    4    1    CJ1
.....................ugcccgaaacuuugacuca.......................................................................    1    0    CJ1
.....................ugcccgaaacuuugacucaa......................................................................    3    0    CJ1
.....................ugcccgaaacuuugacucaac.....................................................................    3    0    CJ1
.....................ugcccgaaacuuugacucaacc....................................................................    3    0    CJ1
.....................Agcccgaaacuuugacucaacc....................................................................    1    1    CJ1
.....................Cgcccgaaacuuugacucaaccu...................................................................    1    1    CJ1
.....................ugcccgaaacuuugacucaaccA...................................................................    6    1    CJ1
.....................ugcccgaaacuuugacucaaccC...................................................................    4    1    CJ1
.....................ugcccgaaacuuugacucaaccu...................................................................    43   0    CJ1
.....................ugcccgaaacuuugacucaaccuU..................................................................    3    1    CJ1
......................gcccgaaacuuugacucaacA....................................................................    1    1    CJ1
......................gcccgaaacuuugacucaaccC...................................................................    1    1    CJ1
........................ccgaaacuuugacucaaccC...................................................................    1    1    CJ1
.........................cgaaacuuugacucaacc....................................................................    1    0    CJ1
..........................gaaacuuugacucaaccC...................................................................    1    1    CJ1
........................................................aAgugagcaaaguuucaggugugu...............................    2    1    CJ1
.........................................................ggugagcaaaguuucaggug..................................    1    0    CJ1
.........................................................ggugagcaaaguuucaggugu.................................    1    0    CJ1
.........................................................ggugagcaaaguuucaggugug................................    2    0    CJ1
.........................................................ggugagcaaaguuucagguguU................................    1    1    CJ1
.........................................................ggugagcaaaguuucaggugugu...............................    6    0    CJ1
.........................................................Cgugagcaaaguuucaggugugu...............................    16   1    CJ1
.........................................................ggugagAaaaguuucaggugugu...............................    1    1    CJ1
..........................................................gugagcaaaguuucaggu...................................    14   0    CJ1
..........................................................gugagcaaaguuucaggC...................................    1    1    CJ1
..........................................................gugagcaaGguuucaggug..................................    1    1    CJ1
..........................................................gugagcaaaguuucagguC..................................    1    1    CJ1
..........................................................gugagcCaaguuucaggug..................................    1    1    CJ1
..........................................................gugagcaaaguuucGggug..................................    1    1    CJ1
..........................................................gugagcaaaguuucaggug..................................    57   0    CJ1
..........................................................gugagcaaaguuucagguU..................................    2    1    CJ1
..........................................................gugagcaaaguuucagguA..................................    8    1    CJ1
..........................................................gugagcaaaguuucagguUu.................................    1    1    CJ1
..........................................................gugUgcaaaguuucaggugu.................................    1    1    CJ1
..........................................................guCagcaaaguuucaggugu.................................    1    1    CJ1
..........................................................Augagcaaaguuucaggugu.................................    1    1    CJ1
..........................................................gugagcaGaguuucaggugu.................................    1    1    CJ1
..........................................................gugagcaaaguuucaggugG.................................    2    1    CJ1
..........................................................gugagcCaaguuucaggugu.................................    1    1    CJ1
..........................................................gugagcaaaguuucaggugC.................................    18   1    CJ1
..........................................................gugagcaaaguuucaggCgu.................................    1    1    CJ1
..........................................................gugagcaaaguuucGggugu.................................    1    1    CJ1
..........................................................gugagcaaaguuucaggGgu.................................    1    1    CJ1
..........................................................gugGgcaaaguuucaggugu.................................    1    1    CJ1
..........................................................gugagcaaaguuuUaggugu.................................    1    1    CJ1
..........................................................gugagcaaaguuucaggugu.................................    161  0    CJ1
..........................................................gugagcaaaguCucaggugu.................................    1    1    CJ1
..........................................................gugagcaaagCuucaggugug................................    1    1    CJ1
..........................................................gugUgcaaaguuucaggugug................................    1    1    CJ1
..........................................................gugagcaaaguuCcaggugug................................    3    1    CJ1
..........................................................gugagUaaaguuucaggugug................................    1    1    CJ1
..........................................................gugagcaaaguuucaUgugug................................    4    1    CJ1
..........................................................gugagcaaaguuucaAgugug................................    1    1    CJ1
..........................................................gugagcaaaguuucaggCgug................................    1    1    CJ1
..........................................................gugagcaaaguuucagguguC................................    3    1    CJ1
..........................................................gugagcaaaguCucaggugug................................    2    1    CJ1
..........................................................gugagcaaaguuucagguAug................................    1    1    CJ1
..........................................................gugagcGaaguuucaggugug................................    1    1    CJ1
..........................................................gugagcaaaguuucUggugug................................    2    1    CJ1
..........................................................gugagcaaaguuucGggugug................................    6    1    CJ1
..........................................................gugagcaaaguuucaggugAg................................    2    1    CJ1
..........................................................gugagcaaaguuucagguguU................................    18   1    CJ1
..........................................................gugagcaaagGuucaggugug................................    1    1    CJ1
..........................................................guUagcaaaguuucaggugug................................    4    1    CJ1
..........................................................gCgagcaaaguuucaggugug................................    4    1    CJ1
..........................................................gugagcaaaguuucaggugug................................    793  0    CJ1
..........................................................Uugagcaaaguuucaggugug................................    1    1    CJ1
..........................................................gugagcaaUguuucaggugug................................    1    1    CJ1
..........................................................gugagcaaaguuucaggGgug................................    1    1    CJ1
```

```
                    Star                                Mature
ugagagaaugucggccuugugugcccgaaacuuugacucaaccugaguuuaggaaaaggugagcaaaguuucaggugugucggguugacugaaaaaacugguuuaccagga
..........................................................gugagcaaaguuucaggugCg................................ 3     1  CJ1
..........................................................gugaAcaaaguuucaggugug................................ 1     1  CJ1
..........................................................gugGgcaaaguuucaggugug................................ 4     1  CJ1
..........................................................gugagcaaaguuucaggugGg................................ 1     1  CJ1
..........................................................gugagcaaaguuucagguguA................................ 84    1  CJ1
..........................................................gugagcaaGguuucaggugug................................ 3     1  CJ1
..........................................................guAagcaaaguuucaggugugu............................... 26    1  CJ1
..........................................................gugagcaaaguuucaggugAgu............................... 4     1  CJ1
..........................................................Cugagcaaaguuucaggugugu............................... 1     1  CJ1
..........................................................gugagcaaaguuucaAgugugu............................... 7     1  CJ1
..........................................................gugagcaaaguuucUggugugu............................... 2     1  CJ1
..........................................................gugagcaaagAuucaggugugu............................... 1     1  CJ1
..........................................................gugagcaaaguuucaggGgugu............................... 6     1  CJ1
..........................................................gugaUcaaaguuucaggugugu............................... 3     1  CJ1
..........................................................gugGgcaaaguuucaggugugu............................... 31    1  CJ1
..........................................................gugagcaaaguuuUaggugugu............................... 14    1  CJ1
..........................................................gugaAcaaaguuucaggugugu............................... 8     1  CJ1
..........................................................gugagcaaaguuucCggugugu............................... 4     1  CJ1
..........................................................guCagcaaaguuucaggugugu............................... 13    1  CJ1
..........................................................Augagcaaaguuucaggugugu............................... 12    1  CJ1
..........................................................gAgagcaaaguuucaggugugu............................... 1     1  CJ1
..........................................................gugagcaaaguuucagguguAu............................... 1     1  CJ1
..........................................................gugagcaaaguCucaggugugu............................... 12    1  CJ1
..........................................................gugagcaaaguuucaggCgugu............................... 35    1  CJ1
..........................................................gugagcaaUguuucaggugugu............................... 6     1  CJ1
..........................................................gugagcaaaguuCcaggugugu............................... 36    1  CJ1
..........................................................gugagcaaaguuAcaggugugu............................... 2     1  CJ1
..........................................................gugagcaaaguuucagAugugu............................... 6     1  CJ1
..........................................................Uugagcaaaguuucaggugugu............................... 6     1  CJ1
..........................................................gugagcUaaguuucaggugugu............................... 12    1  CJ1
..........................................................gugagcaaaCuuucaggugugu............................... 3     1  CJ1
..........................................................gugagcaUaguuucaggugugu............................... 6     1  CJ1
..........................................................gugagcaaaguuucaggAgugu............................... 12    1  CJ1
..........................................................gugagcaGaguuucaggugugu............................... 28    1  CJ1
..........................................................gugagcaaaguuucagguCugu............................... 3     1  CJ1
..........................................................gugagcaaaguuucagguUugu............................... 6     1  CJ1
..........................................................gugagcaaaguuucaUgugugu............................... 2     1  CJ1
..........................................................gugagcaaaguuGcaggugugu............................... 3     1  CJ1
..........................................................gugagcaaaguuucaggugugu............................... 8095  0  CJ1
..........................................................gugagcaaCguuucaggugugu............................... 3     1  CJ1
..........................................................gugagcaaaguuucagguAugu............................... 5     1  CJ1
..........................................................gugagcaaaAuuucaggugugu............................... 6     1  CJ1
..........................................................gugagcaaagCuucaggugugu............................... 19    1  CJ1
..........................................................gugagcaaaguuucaggugugA............................... 324   1  CJ1
..........................................................gugagAaaaguuucaggugugu............................... 5     1  CJ1
..........................................................gugagcCaaguuucaggugugu............................... 1     1  CJ1
..........................................................gugagcaaaguuucagguguCu............................... 4     1  CJ1
..........................................................gugUgcaaaguuucaggugugu............................... 3     1  CJ1
..........................................................gugagcaaaguuucaggugGgu............................... 6     1  CJ1
..........................................................gugagcaaaguAucaggugugu............................... 1     1  CJ1
..........................................................gugagcaaaguuucGggugugu............................... 46    1  CJ1
..........................................................guUagcaaaguuucaggugugu............................... 22    1  CJ1
..........................................................gugagcaCaguuucaggugugu............................... 3     1  CJ1
..........................................................gugagcaaagGuucaggugugu............................... 4     1  CJ1
..........................................................gCgagcaaaguuucaggugugu............................... 23    1  CJ1
..........................................................gugagcGaaguuucaggugugu............................... 24    1  CJ1
..........................................................gugagcaaaguuucaggugugG............................... 142   1  CJ1
..........................................................gugagcaaaguuucagguguUu............................... 9     1  CJ1
..........................................................gugagUaaaguuucaggugugu............................... 16    1  CJ1
..........................................................gugagcaaGguuucaggugugu............................... 40    1  CJ1
..........................................................gugagcaaaguuucaggugCgu............................... 24    1  CJ1
..........................................................gugagcaaaguuucagguguguG.............................. 62    1  CJ1
..........................................................gugagcaaaguuucagguguguc.............................. 21    0  CJ1
..........................................................gugagcGaaguuucagguguguc.............................. 1     1  CJ1
...........................................................ugagcaaaguuucaggugug................................ 1     0  CJ1
...........................................................ugagcaaaguuucaggugugu............................... 3     0  CJ1
............................................................gagcaaaguuucaggugugu............................... 4     0  CJ1
.............................................................agcaaaguuucaggugugu............................... 1     0  CJ1
..............................................................gcaaaguuucaggugugu............................... 1     0  CJ1

....................gugccUgaaacuuugacu......................................................................... 1     1  HJ1
```

Star Mature

ugagagaaugucggccuugugugcccgaaacuuugacucaaccugaguuuaggaaaaggugagcaaaguuucaggugugucggguugacugaaaaaacugguuuaccagga			
....................gugcccgaaacuuugacA...	2	1	HJ1
....................gugcccgaaacuuugUcu...	1	1	HJ1
....................gugcccgaaacuuugacu...	12	0	HJ1
....................gugcccgaaacuuugacC...	1	1	HJ1
....................gugccUgaaacuuugacuc..	2	1	HJ1
....................gugcccgaaacuuugacuc..	5	0	HJ1
....................gugcccgaaacuuugacuU..	1	1	HJ1
....................gugcccgaaacuuugUcuc..	7	1	HJ1
....................gugcccgaaacuuugUcuca...	2	1	HJ1
....................gugcccgaaacuuugacuca...	5	0	HJ1
....................gugccUgaaacuuugacucaa..	1	1	HJ1
....................gugcccgaaacuuugacucaa..	6	0	HJ1
....................gugcccgaaacuuugUcucaac...	1	1	HJ1
....................gugcccgaaacuuugacucaac...	9	0	HJ1
....................gugccUgaaacuuugacucaacc..	1	1	HJ1
....................gugccGgaaacuuugacucaacc..	1	1	HJ1
....................gugcccgaaacuuugacucaacA..	1	1	HJ1
....................gugcccgaaacuuugUcucaacc..	1	1	HJ1
....................gugcccgaaacuuugacucaacU..	8	1	HJ1
....................gugcccgaaacuuugacucaacc..	14	0	HJ1
....................gugcccgaaacuuugUcucaaccu...	3	1	HJ1
....................gugcccgaaGcuuugacucaaccu...	2	1	HJ1
....................gugccUgaaacuuugacucaaccu...	1	1	HJ1
....................gugcccgaaacuuugacucaaccG...	2	1	HJ1
....................gugcccgaaacuuugacucGaccu...	1	1	HJ1
....................gugcccgaaacuuugacucaGccu...	1	1	HJ1
....................gugcccgaaacuuugacucUaccu...	1	1	HJ1
....................gugcccgaaacuuugacucaaccC...	14	1	HJ1
....................gugcccgaaacuuugacCcaaccu...	1	1	HJ1
....................gugcccgaaacuuugacucaUccu...	1	1	HJ1
....................gugcccgaaacuuugacucaaccA...	17	1	HJ1
....................gugcccgaaacuuugacucaaccu...	92	0	HJ1
....................gugcccgaaacuuugacucaaAcu...	1	1	HJ1
....................gugcccgaaacuuugacucaaccuU..	1	1	HJ1
.....................ugcccgaaacuuugacucaac...	5	0	HJ1
.....................ugcccgaaacuuugacucaaccA...	1	1	HJ1
.....................ugcccgaaacuuugacucaaccu...	9	0	HJ1
.....................uUcccgaaacuuugacucaaccu...	1	1	HJ1
.....................ugcccgaaacuuugacucaaccuU..	1	1	HJ1
....aaggugagcaaaguuucaggugug................................	1	0	HJ1
....aAgugagcaaaguuucaggugugu...............................	1	1	HJ1
....ggugagcaaaguuucaggugu.................................	1	0	HJ1
....ggugagcaaaguuucagguguC................................	1	1	HJ1
....Cgugagcaaaguuucaggugug................................	1	1	HJ1
....ggugagcaaaguuucaggugug................................	5	0	HJ1
....ggugagcaaaguuucGggugugu...............................	1	1	HJ1
....Cgugagcaaaguuucaggugugu...............................	9	1	HJ1
....ggugagcaaaguuucaggugugu...............................	2	0	HJ1
....Agugagcaaaguuucaggugugu...............................	2	1	HJ1
....gugagcaaaguuucaUgu...................................	2	1	HJ1
....gugagcaaaguuucaggA...................................	6	1	HJ1
....Augagcaaaguuucaggu...................................	1	1	HJ1
....gugagcaaaguuucaggu...................................	51	0	HJ1
....gAgagcaaaguuucaggu...................................	1	1	HJ1
....gugagcaaaguuucaggC...................................	14	1	HJ1
....gugaUcaaaguuucaggu...................................	1	1	HJ1
....gugagcaaaguuCcaggu...................................	1	1	HJ1
....gugagcaaaguuucGggu...................................	1	1	HJ1
....gugagcGaaguuucaggug..................................	1	1	HJ1
....gugagcaaaguuucaggAg..................................	1	1	HJ1
....gugagcaaaguuucaggGg..................................	1	1	HJ1
....gugagcaaaguuucaggug..................................	181	0	HJ1
....gugagcaaaguuucagCug..................................	2	1	HJ1
....gugagcaaaguuucaggCg..................................	1	1	HJ1
....gugagcaaaguuucagguU..................................	3	1	HJ1
....gugagcaaagCuucaggug..................................	2	1	HJ1
....gugagcaaagGuucaggug..................................	1	1	HJ1
....gugagcaaaguuucGggug..................................	1	1	HJ1
....gugagcaaaguuucagguC..................................	1	1	HJ1
....gugagcaaaguuucagguA..................................	25	1	HJ1

```
                    Star                                         Mature
ugagagaaugucggccuugugugcccgaaacuugacucaaccugaguuuaggaaaaggugagcaaaguuucaggugugucggguugacugaaaaaacugguuuaccagga
.........................................................gugGgcaaaguuucaggug..................................    1     1    HJ1
.........................................................gugaAcaaaguuucaggug..................................    1     1    HJ1
.........................................................Augagcaaaguuucaggug..................................    1     1    HJ1
.........................................................gAgagcaaaguuucaggug..................................    1     1    HJ1
.........................................................gugagcaGaguuucaggug..................................    1     1    HJ1
.........................................................gugGgcaaaguuucaggugu.................................    2     1    HJ1
.........................................................gugagcaaaguuucagguAu.................................    1     1    HJ1
.........................................................Uugagcaaaguuucaggugu.................................    1     1    HJ1
.........................................................gugagcGaaguuucaggugu.................................    2     1    HJ1
.........................................................gugagcaaaguuucaggugC.................................    72    1    HJ1
.........................................................gugagcaaaguuucaggGgu.................................    1     1    HJ1
.........................................................gugagcaaaguuucaUgugu.................................    1     1    HJ1
.........................................................gugagcaaaguuucaggCgu.................................    2     1    HJ1
.........................................................gugagcaaaguuuCUggugu.................................    1     1    HJ1
.........................................................gugagcaaaguuucGggugu.................................    1     1    HJ1
.........................................................gugagcaaaguuCcaggugu.................................    3     1    HJ1
.........................................................gugagcaaaguuucagAugu.................................    1     1    HJ1
.........................................................gugagUaaaguuucaggugu.................................    1     1    HJ1
.........................................................gGgagcaaaguuucaggugu.................................    1     1    HJ1
.........................................................gugagcaaaguCucaggugu.................................    2     1    HJ1
.........................................................gCgagcaaaguuucaggugu.................................    1     1    HJ1
.........................................................gugagcaGaguuucaggugu.................................    1     1    HJ1
.........................................................gugaAcaaaguuucaggugu.................................    1     1    HJ1
.........................................................gAgagcaaaguuucaggugu.................................    3     1    HJ1
.........................................................gugagcaaaguuucaggugu.................................    455   0    HJ1
.........................................................gugagcaaaguuucaggugG.................................    12    1    HJ1
.........................................................Augagcaaaguuucaggugu.................................    3     1    HJ1
.........................................................gugaCcaaaguuucaggugu.................................    1     1    HJ1
.........................................................gugCgcaaaguuucaggugug................................    1     1    HJ1
.........................................................guAagcaaaguuucaggugug................................    1     1    HJ1
.........................................................gugagUaaaguuucaggugug................................    1     1    HJ1
.........................................................gugagcaaaguuucagguguU................................    61    1    HJ1
.........................................................gugagcaaaguuucaggCgug................................    2     1    HJ1
.........................................................gugagcaaaguuucaUgugug................................    3     1    HJ1
.........................................................gugagcaaaguuucagguAug................................    2     1    HJ1
.........................................................gugagcaaaguuucagguguA................................    200   1    HJ1
.........................................................gugagcaaaguuucaggugCg................................    8     1    HJ1
.........................................................gugagcaaaguuucaggGgug................................    3     1    HJ1
.........................................................gugagcaaaguuucaggugGg................................    14    1    HJ1
.........................................................gugagcaaaguuucagguguC................................    15    1    HJ1
.........................................................gugGgcaaaguuucaggugug................................    6     1    HJ1
.........................................................gugagcaaaguCucaggugug................................    3     1    HJ1
.........................................................gugaCcaaaguuucaggugug................................    1     1    HJ1
.........................................................Cugagcaaaguuucaggugug................................    1     1    HJ1
.........................................................gugagcaaaguuucGggugug................................    7     1    HJ1
.........................................................gugagcaaaguuAaggugug................................    9     1    HJ1
.........................................................gugagcaaaguuucaAgugug................................    4     1    HJ1
.........................................................gugUgcaaaguuucaggugug................................    1     1    HJ1
.........................................................gugagcaGaguuucaggugug................................    4     1    HJ1
.........................................................gugagcaaaguuucaggugug................................    1724  0    HJ1
.........................................................gugagcaaaguuucUggugug................................    1     1    HJ1
.........................................................gugagcaaUguuucaggugug................................    3     1    HJ1
.........................................................gugagcaaaguuucaggugAg................................    1     1    HJ1
.........................................................gugagcaaagCuucaggugug................................    3     1    HJ1
.........................................................gugagcUaaguuucaggugug................................    2     1    HJ1
.........................................................gugagcaaaguuucagguCug................................    1     1    HJ1
.........................................................gugaUcaaaguuucaggugug................................    2     1    HJ1
.........................................................gugagcaaaguuCcaggugug................................    5     1    HJ1
.........................................................gugagcGaaguuucaggugug................................    3     1    HJ1
.........................................................Uugagcaaaguuucaggugug................................    2     1    HJ1
.........................................................gugagcaaaguuuGaggugug................................    1     1    HJ1
.........................................................gugagcaaagAuucaggugug................................    1     1    HJ1
.........................................................gugagcaaaguuucagAugug................................    2     1    HJ1
.........................................................gAgagcaaaguuucaggugug................................    8     1    HJ1
.........................................................gugagcaaaguuAcaggugug................................    1     1    HJ1
.........................................................gugagcaUaguuucaggugug................................    2     1    HJ1
.........................................................gugagcaaaguuUaggugug................................    2     1    HJ1
.........................................................gugagAaaaguuucaggugug................................    2     1    HJ1
.........................................................gugaAcaaaguuucaggugug................................    7     1    HJ1
.........................................................Augagcaaaguuucaggugug................................    5     1    HJ1
```

Star Mature

```
ugagagaaugucggccuugugugcccgaaacuuugacucaaccugaguuuaggaaaaggugagcaaaguuucaggugugucgguugacugaaaaaacugguuuaccagga
.........................................................gCgagcaaaguuucaggugug................................  1      1   HJ1
.........................................................gugagcaaCguuucaggugug................................  1      1   HJ1
.........................................................gugagcaaGguuucaggugug................................  11     1   HJ1
.........................................................gGgagcaaaguuucaggugug................................  1      1   HJ1
.........................................................gugagcaaagGuucaggugug................................  1      1   HJ1
.........................................................guUagcaaaguuucaggugugu...............................  3      1   HJ1
.........................................................gugagGaaaguuucaggugugu...............................  2      1   HJ1
.........................................................guCagcaaaguuucaggugugu...............................  1      1   HJ1
.........................................................gugagcaaaguCucaggugugu...............................  38     1   HJ1
.........................................................gugaUcaaaguuucaggugugu...............................  14     1   HJ1
.........................................................gAgagcaaaguuucaggugugu...............................  29     1   HJ1
.........................................................gugagcaaaguuucUggugugu...............................  7      1   HJ1
.........................................................gGgagcaaaguuucaggugugu...............................  6      1   HJ1
.........................................................gugagcaaaguuucaCgugugu...............................  2      1   HJ1
.........................................................gugagcaaaguuucaUgugugu...............................  4      1   HJ1
.........................................................gugagcUaaguuucaggugugu...............................  7      1   HJ1
.........................................................gugagcaaaguuucagAugugu...............................  13     1   HJ1
.........................................................gugagcaaGguuucaggugugu...............................  64     1   HJ1
.........................................................Cugagcaaaguuucaggugugu...............................  8      1   HJ1
.........................................................gugagcaaaguuucagguCugu...............................  1      1   HJ1
.........................................................gugagcaaaguuAcaggugugu...............................  4      1   HJ1
.........................................................gugagcaaaguuuUaggugugu...............................  15     1   HJ1
.........................................................gugagcaaaguuucaggugugA...............................  633    1   HJ1
.........................................................gugagcaGaguuucaggugugu...............................  49     1   HJ1
.........................................................gugagcaaaguuucagUugugu...............................  1      1   HJ1
.........................................................gugagcaaaguuucaggugugG...............................  443    1   HJ1
.........................................................gugagcCaaguuucaggugugu...............................  1      1   HJ1
.........................................................guAagcaaaguuucaggugugu...............................  9      1   HJ1
.........................................................gugagcaaaAuuucaggugugu...............................  3      1   HJ1
.........................................................gugagcaaaguuucaggAgugu...............................  6      1   HJ1
.........................................................gugagcaaaguuuAaggugugu...............................  18     1   HJ1
.........................................................gugUgcaaaguuucaggugugu...............................  9      1   HJ1
.........................................................gugagcaaaguuucCggugugu...............................  1      1   HJ1
.........................................................gugagcaaaguuGcaggugugu...............................  1      1   HJ1
.........................................................gugagcaaaguuucagguguCu...............................  23     1   HJ1
.........................................................gugaCcaaaguuucaggugugu...............................  11     1   HJ1
.........................................................gugagcaaaguuCcaggugugu...............................  58     1   HJ1
.........................................................Uugagcaaaguuucaggugugu...............................  17     1   HJ1
.........................................................gugagcaaaCuuucaggugugu...............................  1      1   HJ1
.........................................................gugagcaaagGuucaggugugu...............................  4      1   HJ1
.........................................................gugagUaaaguuucaggugugu...............................  27     1   HJ1
.........................................................gugaAcaaaguuucaggugugu...............................  61     1   HJ1
.........................................................gugagcaaaguuucaggugCgu...............................  47     1   HJ1
.........................................................gugagcaaaguuucagguUugu...............................  12     1   HJ1
.........................................................gugagcaaaguuucaAgugugu...............................  20     1   HJ1
.........................................................gugagcaaaguuucaggugAgu...............................  11     1   HJ1
.........................................................gugagcaaaguuucaggCgugu...............................  40     1   HJ1
.........................................................gCgagcaaaguuucaggugugu...............................  42     1   HJ1
.........................................................gugagcaaUguuucaggugugu...............................  15     1   HJ1
.........................................................gugagcaUaguuucaggugugu...............................  8      1   HJ1
.........................................................gugagcaaagCuucaggugugu...............................  41     1   HJ1
.........................................................gugagcaaCguuucaggugugu...............................  7      1   HJ1
.........................................................gugGgcaaaguuucaggugugu...............................  60     1   HJ1
.........................................................gugagAaaaguuucaggugugu...............................  25     1   HJ1
.........................................................gugagcaaaguuucGggugugu...............................  60     1   HJ1
.........................................................gugagcaaaguuucagguAugu...............................  8      1   HJ1
.........................................................gugagcaaaguuucaggugGgu...............................  42     1   HJ1
.........................................................gugagcaaaUuuucaggugugu...............................  1      1   HJ1
.........................................................gugagcaaaguuucaggGgugu...............................  10     1   HJ1
.........................................................gugagcaaaguGucaggugugu...............................  3      1   HJ1
.........................................................gugagcaaaguuucaggugugAu...............................  39     1   HJ1
.........................................................gugCgcaaaguuucaggugugu...............................  6      1   HJ1
.........................................................gugagcaaagAuucaggugugu...............................  1      1   HJ1
.........................................................gugagcGaaguuucaggugugu...............................  45     1   HJ1
.........................................................Augagcaaaguuucaggugugu...............................  42     1   HJ1
.........................................................gugagcaaaguuuGaggugugu...............................  8      1   HJ1
.........................................................gugagcaaaguuucagguguUu...............................  37     1   HJ1
.........................................................gugagcaaaguuucaggugugu...............................  13486  0   HJ1
.........................................................gugagcaaaguuucaggugAguc..............................  1      1   HJ1
.........................................................gugagcaaaguuucGgguguguc..............................  1      1   HJ1
```

Star **Mature**

```
ugagagaaugucggccuugugugcccgaaacuuugacucaaccugaguuuaggaaaaggugagcaaaguuucaggugugucggguugacugaaaaaacugguuuaccagga
..........................................................gugagcaaaguuucaggugugCc..............................   1    1  HJ1
..........................................................gugagcaaaguuucagguguguG..............................   63   1  HJ1
..........................................................gugagcaaaguuucagguguguc..............................   46   0  HJ1
..........................................................gugagcaaaguuucaggCguguc..............................   1    1  HJ1
..........................................................gugagcaaaguuucagguguguucggU...........................   1    1  HJ1
...........................................................ugagcaaaguuucaggugugA...............................   2    1  HJ1
...........................................................ugagcaGaguuucaggugugu...............................   1    1  HJ1
...........................................................ugagcaaaguuucaggugugG...............................   2    1  HJ1
...........................................................Cgagcaaaguuucaggugugu...............................   1    1  HJ1
...........................................................ugagcaaaguuucaggugugu...............................   7    0  HJ1
............................................................gagcaaaguuucaggugG.................................   1    1  HJ1
............................................................gagcaaaguuucaggugugu...............................   7    0  HJ1
.............................................................agcaaaguuucagguguA................................   1    1  HJ1
.............................................................agcaaaguuucaggugugu...............................   3    0  HJ1
..............................................................gcaaaguuucaggugugG...............................   1    1  HJ1
..............................................................gcaaaguuucaggugugu...............................   1    0  HJ1

...................Cgugcccgaaacuuugacu.........................................................................   2    1  LA1
...................Cgugcccgaaacuuugacucaa......................................................................   1    1  LA1
...................Cgugcccgaaacuuugacucaacc....................................................................   3    1  LA1
...................Cgugcccgaaacuuugacucaaccu...................................................................   1    1  LA1
....................gugcccgaaacuuugacA.........................................................................   1    1  LA1
....................gugcccgaaacuuugacu.........................................................................   69   0  LA1
....................gugcccgaaacuuugacC.........................................................................   8    1  LA1
....................gugAccgaaacuuugacu.........................................................................   1    1  LA1
....................gugcccgaaGcuuugacu.........................................................................   1    1  LA1
....................gugcccgaaacuCugacu.........................................................................   1    1  LA1
....................gugcccgaaacuuugacuA........................................................................   6    1  LA1
....................gugcccgaaacuuugacCc........................................................................   1    1  LA1
....................gugcccgaaacuuugacuU........................................................................   7    1  LA1
....................gugcccgaaacuuugacuc........................................................................   83   0  LA1
....................gugcccgaaacuuugacucU.......................................................................   1    1  LA1
....................gugcUcgaaacuuugacuca.......................................................................   1    1  LA1
....................gugcccgaaacuuugacuca.......................................................................   210  0  LA1
....................gugcccgaaacuuugacuUa.......................................................................   1    1  LA1
....................gugcccgaaacuuugacCca.......................................................................   1    1  LA1
....................gugcccgaaacuCugacuca.......................................................................   1    1  LA1
....................gugcccgaaacuuugacucG.......................................................................   2    1  LA1
....................gCgcccgaaacuuugacuca.......................................................................   3    1  LA1
....................gugcccgaaacuuugacuUaa......................................................................   1    1  LA1
....................gugcccgaaacuCugacucaa......................................................................   1    1  LA1
....................Augcccgaaacuuugacucaa......................................................................   2    1  LA1
....................gugccUgaaacuuugacucaa......................................................................   1    1  LA1
....................gugcUcgaaacuuugacucaa......................................................................   1    1  LA1
....................gugcccgaaacuuugacAcaa......................................................................   1    1  LA1
....................Cugcccgaaacuuugacucaa......................................................................   1    1  LA1
....................gugcccgaaacuuugacucaa......................................................................   215  0  LA1
....................gugcccgaaacuCugacucaac.....................................................................   1    1  LA1
....................gugcccgaaacuuugacucaaU.....................................................................   15   1  LA1
....................gugcccgaaacuuugacucaac.....................................................................   231  0  LA1
....................gugcccgaaacuuugacucGac.....................................................................   1    1  LA1
....................gugcccgaaacuuugacCcaac.....................................................................   1    1  LA1
....................gGgcccgaaacuuugacucaac.....................................................................   1    1  LA1
....................gugcccgaaacuuugUcucaac.....................................................................   2    1  LA1
....................gugcccgGaacuuugacucaac.....................................................................   2    1  LA1
....................gugcccAaaacuuugacucaac.....................................................................   1    1  LA1
....................gugcccgaaacuuugacucaCc.....................................................................   1    1  LA1
....................gugcccgaGacuuugacucaac.....................................................................   1    1  LA1
....................Augcccgaaacuuugacucaac.....................................................................   1    1  LA1
....................gugcccgaaacuuugacCcaacc....................................................................   10   1  LA1
....................gugcccgaaacuuCgacucaacc....................................................................   1    1  LA1
....................gugcccgaaacuuugacucaaUc....................................................................   1    1  LA1
....................gugcccgaaacuuugacucaacc....................................................................   371  0  LA1
....................gugccUgaaacuuugacucaacc....................................................................   3    1  LA1
....................gugcccgaaacuuugacucaacA....................................................................   22   1  LA1
....................gAgcccgaaacuuugacucaacc....................................................................   2    1  LA1
....................gugcccgaaacuuugacucaacU....................................................................   50   1  LA1
....................Augcccgaaacuuugacucaacc....................................................................   1    1  LA1
....................gugcccgaaaUuuugacucaacc....................................................................   1    1  LA1
....................gugcccgaaacuuuAacucaacc....................................................................   1    1  LA1
```

Star **Mature**

```
ugagagaaugucggccuugugugcccgaaacuuugacucaaccugaguuuaggaaaaggugagcaaaguuucaggugugucggguugacugaaaaaacugguuuaccagga
....................gugcccgGaacuuugacucaacc....................................................................  1     1  LA1
....................gugcccgaaacuuugacucGacc....................................................................  3     1  LA1
....................gugcccgaaacuuugacuUaaccu...................................................................  2     1  LA1
....................gugUccgaaacuuugacucaaccu...................................................................  1     1  LA1
....................gugcccgaaacuuugacucGaccu...................................................................  7     1  LA1
....................gugcccgaaacuuugaUucaaccu...................................................................  13    1  LA1
....................gugcccgaaacuuuAacucaaccu...................................................................  4     1  LA1
....................gCgcccgaaacuuugacucaaccu...................................................................  4     1  LA1
....................gugcUcgaaacuuugacucaaccu...................................................................  9     1  LA1
....................gugcccUaaacuuugacucaaccu...................................................................  1     1  LA1
....................gugccGgaaacuuugacucaaccu...................................................................  2     1  LA1
....................gugcccgGaacuuugacucaaccu...................................................................  7     1  LA1
....................gugcccgaaacuAugacucaaccu...................................................................  1     1  LA1
....................gugcccgaaacuuugacucaaccC...................................................................  382   1  LA1
....................gugcccgaaacGuugacucaaccu...................................................................  1     1  LA1
....................gugcccgaaacuuugacucaaccG...................................................................  83    1  LA1
....................gugcccgaaacuuugacucCaccu...................................................................  1     1  LA1
....................gugcccgaaacuCugacucaaccu...................................................................  6     1  LA1
....................gugcccgaaacuuugacucaacUu...................................................................  2     1  LA1
....................gugcccAaaacuuugacucaaccu...................................................................  5     1  LA1
....................Augcccgaaacuuugacucaaccu...................................................................  4     1  LA1
....................gugcccgaaacuuugUcucaaccu...................................................................  34    1  LA1
....................gAgcccgaaacuuugacucaaccu...................................................................  1     1  LA1
....................guAcccgaaacuuugacucaaccu...................................................................  2     1  LA1
....................gugcccgaGacuuugacucaaccu...................................................................  4     1  LA1
....................gugcccgaaacuuugacucaaUcu...................................................................  6     1  LA1
....................Uugcccgaaacuuugacucaaccu...................................................................  2     1  LA1
....................gugcccgaaacuuugacucaaccu...................................................................  3323  0  LA1
....................gugcccgaaacuuugCcucaaccu...................................................................  1     1  LA1
....................gugcccgaaaUuuugacucaaccu...................................................................  2     1  LA1
....................guUcccgaaacuuugacucaaccu...................................................................  2     1  LA1
....................gugcccgaaacuGugacucaaccu...................................................................  2     1  LA1
....................gugcccgaaacuuGgacucaaccu...................................................................  1     1  LA1
....................gugcccgaaacuuCgacucaaccu...................................................................  9     1  LA1
....................gugcccgaaacuuugacucUaccu...................................................................  1     1  LA1
....................gugcccgaaacuuugacucaGccu...................................................................  7     1  LA1
....................gugccUgaaacuuugacucaaccu...................................................................  6     1  LA1
....................gugcccgaaacuuugacucaacAu...................................................................  1     1  LA1
....................gugcccgaaacuuugacucaaccA...................................................................  207   1  LA1
....................gugcccgaaGcuuugacucaaccu...................................................................  20    1  LA1
....................gugcccgaaacuuugacCcaaccu...................................................................  11    1  LA1
....................gugcccgaaacuuugacucaaccuC..................................................................  3     1  LA1
....................gugcccgaaacuuugacucaaccuU..................................................................  48    1  LA1
....................gugcccgaaacuuugacucaaccuA..................................................................  9     1  LA1
.....................ugcccgaaacuuugacuca.......................................................................  2     0  LA1
.....................ugcccgaaacuuugacucaa......................................................................  6     0  LA1
.....................ugcccgaaacuuugacucaaA.....................................................................  1     1  LA1
.....................ugcccgaaacuuugacucaacU....................................................................  2     1  LA1
.....................ugcccgaaacuuugacucaacc....................................................................  9     0  LA1
.....................ugcUcgaaacuuugacucaaccu...................................................................  1     1  LA1
.....................ugcccgaaacuuugacucaaccC...................................................................  2     1  LA1
.....................ugcccgaaacuuugacucaaccu...................................................................  79    0  LA1
.....................ugcccgaaacuuugacucaaccA...................................................................  9     1  LA1
.....................ugcccgaaacuuugacucaaccuU..................................................................  10    1  LA1
......................gcccgaaacuuugacucaaccC...................................................................  1     1  LA1
........................ccgaaacuuugacucaaccu...................................................................  1     0  LA1
.........................................................ggugagcaaaguuucaggugugu...............................  2     0  LA1
.........................................................Cgugagcaaaguuucaggugugu...............................  7     1  LA1
..........................................................gugagcaaaguuucaggC...................................  2     1  LA1
..........................................................gugagcaaaguuucaggu...................................  19    0  LA1
..........................................................gugagcaaaguuucagguA..................................  5     1  LA1
..........................................................gugagcaaaguuGcaggug..................................  1     1  LA1
..........................................................gugagcaaaguuucaggug..................................  48    0  LA1
..........................................................gugUgcaaaguuucaggugu.................................  1     1  LA1
..........................................................gugagcaaaguuucaggugu.................................  118   0  LA1
..........................................................gugagcaaaguuucUggugu.................................  1     1  LA1
..........................................................gugagcaaaguuucaggugG.................................  1     1  LA1
..........................................................gugagcaaaguCucaggugu.................................  1     1  LA1
..........................................................gugagcaaaguuucaggGgu.................................  1     1  LA1
..........................................................gugagcaaaguuucaggugC.................................  4     1  LA1
```

Star **Mature**

```
ugagagaaugucggccuugugugcccgaaacuuugacucaaccugaguuuaggaaaaggugagcaaaguuucaggugugucggguugacugaaaaaacugguuuaccagga
..........................................................gugagcaaaguuuUaggugu.................................   1      1   LA1
..........................................................gAgagcaaaguuucaggugug................................   1      1   LA1
..........................................................gugagcUaaguuucaggugug................................   1      1   LA1
..........................................................gugGgcaaaguuucaggugug................................   3      1   LA1
..........................................................gugagcaaaguuAcaggugug................................   2      1   LA1
..........................................................gugagcaaaguuucagguguU................................   15     1   LA1
..........................................................gugagcaaaguuucGggugug................................   3      1   LA1
..........................................................gCgagcaaaguuucaggugug................................   1      1   LA1
..........................................................gugagcaaaguuucaggugAg................................   2      1   LA1
..........................................................gugagcaaaguuucaggugug................................   879    0   LA1
..........................................................gugagcaaaguuucagguguA................................   56     1   LA1
..........................................................gugagcaaaguuucUggugug................................   3      1   LA1
..........................................................gugagcaaGguuucaggugug................................   7      1   LA1
..........................................................gugagcaaaguAucaggugug................................   1      1   LA1
..........................................................gugagcaaaguuuUaggugug................................   2      1   LA1
..........................................................gugagcaaaguuucCggugug................................   2      1   LA1
..........................................................gugagcaaaguuucaggugCg................................   12     1   LA1
..........................................................gugagcaaaguCucaggugug................................   1      1   LA1
..........................................................gugagcaaaguuucagguguC................................   1      1   LA1
..........................................................gugagcaaagGuucaggugug................................   2      1   LA1
..........................................................gugagcaaaguuucaggGgug................................   1      1   LA1
..........................................................gugagcGaaguuucaggugug................................   3      1   LA1
..........................................................gugagcaGaguuucaggugug................................   2      1   LA1
..........................................................gugagcaaaguuucaggugGg................................   4      1   LA1
..........................................................gugagcaaCguuucaggugug................................   1      1   LA1
..........................................................gugagcaaaguuCcaggugug................................   1      1   LA1
..........................................................gugagcaaagAuucaggugugu...............................   2      1   LA1
..........................................................gugagcaaaguuucagguCugu...............................   1      1   LA1
..........................................................gugaCcaaaguuucaggugugu...............................   2      1   LA1
..........................................................gugagcaaaguuucaggugugA...............................   171    1   LA1
..........................................................gugagcaaaguuucaggAgugu...............................   9      1   LA1
..........................................................guCagcaaaguuucaggugugu...............................   1      1   LA1
..........................................................gAgagcaaaguuucaggugugu...............................   2      1   LA1
..........................................................gugagcaaaguuucagguguAu...............................   7      1   LA1
..........................................................gugagcaaaguuucUggugugu...............................   4      1   LA1
..........................................................gugagcaaaguuucaggugCgu...............................   13     1   LA1
..........................................................gugagcaaaguuucagAugugu...............................   3      1   LA1
..........................................................gugagcCaaguuucaggugugu...............................   2      1   LA1
..........................................................Uugagcaaaguuucaggugugu...............................   5      1   LA1
..........................................................gugagcaaaguuucaggugugG...............................   96     1   LA1
..........................................................gugagcaaagGuucaggugugu...............................   1      1   LA1
..........................................................gugagcaaaguuucaAgugugu...............................   5      1   LA1
..........................................................gCgagcaaaguuucaggugugu...............................   25     1   LA1
..........................................................gugagcaaaguAucaggugugu...............................   2      1   LA1
..........................................................gugagcUaaguuucaggugugu...............................   1      1   LA1
..........................................................gugagcaaaguuucagguAugu...............................   1      1   LA1
..........................................................gugGgcaaaguuucaggugugu...............................   32     1   LA1
..........................................................gugagcaGaguuucaggugugu...............................   19     1   LA1
..........................................................gugagAaaaguuucaggugugu...............................   1      1   LA1
..........................................................gugagcaaaguuuAaggugugu...............................   3      1   LA1
..........................................................Augagcaaaguuucaggugugu...............................   10     1   LA1
..........................................................gugagcaaGguuucaggugugu...............................   21     1   LA1
..........................................................gugUgcaaaguuucaggugugu...............................   2      1   LA1
..........................................................gugagcaaaguuucaggGgugu...............................   5      1   LA1
..........................................................gugagcaaaguuucagguguUu...............................   5      1   LA1
..........................................................gugCgcaaaguuucaggugugu...............................   3      1   LA1
..........................................................gugagUaaaguuucaggugugu...............................   10     1   LA1
..........................................................gugagcaaaguuuUaggugugu...............................   9      1   LA1
..........................................................gugagcaaagCuucaggugugu...............................   9      1   LA1
..........................................................gugagcaaaguuAcaggugugu...............................   4      1   LA1
..........................................................gugagcaaaguuucaggugugu...............................   6414   0   LA1
..........................................................gugagcaaaguuucaggugAgu...............................   13     1   LA1
..........................................................gugagcaaaguCucaggugugu...............................   15     1   LA1
..........................................................gugagcaaaguuCcaggugugu...............................   18     1   LA1
..........................................................gGgagcaaaguuucaggugugu...............................   1      1   LA1
..........................................................gugagcaaaguuucGggugugu...............................   33     1   LA1
..........................................................gugagcaaaguGucaggugugu...............................   1      1   LA1
..........................................................gugaAcaaaguuucaggugugu...............................   5      1   LA1
..........................................................gugagcaaaguuucCggugugu...............................   2      1   LA1
..........................................................gugagcaaaAuuucaggugugu...............................   1      1   LA1
```

```
                    Star                                  Mature
ugagagaaugucggccuugugugcccgaaacuuugacucaaccugaguuuaggaaaaggugagcaaaguuucaggugugucggguugacugaaaaaacugguuuaccagga
..........................................................gugagcaaaguuucaUgugugu...............................  1    1  LA1
..........................................................guAagcaaaguuucaggugugu...............................  5    1  LA1
..........................................................gugagcaUaguuucaggugugu...............................  2    1  LA1
..........................................................gugagcaaUguuucaggugugu...............................  2    1  LA1
..........................................................gugagcGaaguuucaggugugu...............................  13   1  LA1
..........................................................gugagcaaaguuucaggCgugu...............................  19   1  LA1
..........................................................gugagcaaaguuucagguguCu...............................  1    1  LA1
..........................................................gugagcaaaguuucaggugGgu...............................  10   1  LA1
..........................................................gugagcaaCguuucaggugugu...............................  3    1  LA1
..........................................................gugaUcaaaguuucaggugugu...............................  1    1  LA1
..........................................................gugagcaaaguuucagguguguc..............................  28   0  LA1
..........................................................gugagcaaaguuucagguguguG..............................  20   1  LA1
..........................................................Augagcaaaguuucagguguguc..............................  1    1  LA1
...........................................................ugagcaaaguuucaggugugu...............................  4    0  LA1
...........................................................ugagcaaaguuucaggugugG...............................  1    1  LA1
............................................................gagcaaaguuucagguguA................................  1    1  LA1

...................Cgugcccgaaacuuugacucaa......................................................................  1    1  CA1
...................Cgugcccgaaacuuugacucaacc....................................................................  1    1  CA1
...................Cgugcccgaaacuuugacucaaccu...................................................................  3    1  CA1
....................gugcccgaaacuuugacu.........................................................................  29   0  CA1
....................gugcccgaaacuuugacC.........................................................................  3    1  CA1
....................gugcccgaaacuuugacG.........................................................................  1    1  CA1
....................gugcAcgaaacuuugacu.........................................................................  1    1  CA1
....................gugcccgaaacuuCgacuc........................................................................  3    1  CA1
....................gugcccgaaacuuugacuU........................................................................  13   1  CA1
....................gugcccgaaacuuugacuc........................................................................  100  0  CA1
....................gugcccgaaacuuugacAc........................................................................  1    1  CA1
....................gugcccgaGacuuugacuc........................................................................  2    1  CA1
....................gugcccgaaacuCugacuc........................................................................  1    1  CA1
....................gugcccgaaacuuugacuA........................................................................  13   1  CA1
....................Augcccgaaacuuugacuc........................................................................  1    1  CA1
....................gugcccgaaacuuugacuca.......................................................................  178  0  CA1
....................gugcccgaaacuuugacucG.......................................................................  3    1  CA1
....................gugcccgaaacuuCgacuca.......................................................................  1    1  CA1
....................gugcccgaaacuuugacucU.......................................................................  1    1  CA1
....................gugcccgaaacuuuAacuca.......................................................................  1    1  CA1
....................gugcccgaGacuuugacuca.......................................................................  1    1  CA1
....................gugcccgaaacuuugacuUa.......................................................................  3    1  CA1
....................gugcccgaaGcuuugacuca.......................................................................  1    1  CA1
....................gugcccgaaacuuugacCca.......................................................................  1    1  CA1
....................gugcccgaaacuuugacucC.......................................................................  1    1  CA1
....................gugcccgaaGcuuugacucaa......................................................................  1    1  CA1
....................gugcccgaaacuuugacucaa......................................................................  231  0  CA1
....................gugcccgGaacuuugacucaa......................................................................  1    1  CA1
....................gugcccgaaacuuugacucaG......................................................................  1    1  CA1
....................gCgcccgaaacuuugacucaa......................................................................  2    1  CA1
....................gugcccgaaacuuugacucGa......................................................................  1    1  CA1
....................gugcccgaaacuuugaUucaa......................................................................  1    1  CA1
....................gugcccgaaacuuCgacucaa......................................................................  1    1  CA1
....................gugcccgaaaUuuugacucaa......................................................................  1    1  CA1
....................gugcccgGaacuuugacucaac.....................................................................  2    1  CA1
....................gugcccgaaacuuugacucaGc.....................................................................  2    1  CA1
....................gugcccgaaacuuugacucaac.....................................................................  275  0  CA1
....................gCgcccgaaacuuugacucaac.....................................................................  1    1  CA1
....................gugcccgaaacuuugUcucaac.....................................................................  2    1  CA1
....................gugcccgaaacuuCgacucaac.....................................................................  1    1  CA1
....................gugcccgaaacuAugacucaac.....................................................................  1    1  CA1
....................gugcccgaaGcuuugacucaac.....................................................................  1    1  CA1
....................gugcccgaaacuuugacucaaU.....................................................................  11   1  CA1
....................gugccUgaaacuuugacucaac.....................................................................  1    1  CA1
....................gugcccgaaacuuugacCcaac.....................................................................  2    1  CA1
....................gugcccgaaaUuuugacucaac.....................................................................  1    1  CA1
....................gugcccgaaacuuugacucaacG....................................................................  1    1  CA1
....................gugcccgaaacuuugUcucaacc....................................................................  2    1  CA1
....................gugcccgaaacuCugacucaacc....................................................................  1    1  CA1
....................gugcccgaaacuuugacucaGcc....................................................................  2    1  CA1
....................gugcccgaaacuuugacucaacU....................................................................  62   1  CA1
....................gugcccgaaacuuugacucaacc....................................................................  449  0  CA1
....................guAcccgaaacuuugacucaacc....................................................................  1    1  CA1
```

Star Mature

```
ugagagaaugucggccuugugugcccgaaacuuugacucaaccugaguuuaggaaaaggugagcaaaguuucaggugugucggguugacugaaaaaacugguuuaccagga
....................gugcUcgaaacuuugacucaacc....................................................................  1     1  CA1
....................gugcccgaaacuGugacucaacc....................................................................  1     1  CA1
....................gugcccgaaGcuuugacucaacc....................................................................  1     1  CA1
....................Augcccgaaacuuugacucaacc....................................................................  1     1  CA1
....................gugccAgaaacuuugacucaacc....................................................................  1     1  CA1
....................gugcccgaaacuuugacucaacA....................................................................  24    1  CA1
....................gugccUgaaacuuugacucaacc....................................................................  4     1  CA1
....................gugcccgaaacuuugacCcaacc....................................................................  2     1  CA1
....................gugcccgaaacuuugacucaaUc....................................................................  1     1  CA1
....................gCgcccgaaacuuugacucaacc....................................................................  1     1  CA1
....................gugcccgGaacuuugacucaacc....................................................................  2     1  CA1
....................gugcccgaaacuuCgacucaacc....................................................................  2     1  CA1
....................gugcccgaaacAuugacucaaccu...................................................................  4     1  CA1
....................gugcccgaaacuuugacCcaaccu...................................................................  9     1  CA1
....................gugcccgaaacuuugacucaacAu...................................................................  3     1  CA1
....................gugUccgaaacuuugacucaaccu...................................................................  4     1  CA1
....................guCcccgaaacuuugacucaaccu...................................................................  1     1  CA1
....................gugcccgaaacuuugacuUaaccu...................................................................  4     1  CA1
....................gugccUgaaacuuugacucaaccu...................................................................  14    1  CA1
....................Augcccgaaacuuugacucaaccu...................................................................  7     1  CA1
....................gugcccgaaacuuugacucaaccG...................................................................  90    1  CA1
....................gugcccgaaacGuugacucaaccu...................................................................  2     1  CA1
....................gugcccgaaacuuugacucaacUu...................................................................  8     1  CA1
....................gugcccgaaacuuuAacucaaccu...................................................................  2     1  CA1
....................gugcccgaaacuuugaUucaaccu...................................................................  5     1  CA1
....................gugcccgaaacuuugacucaaccC...................................................................  478   1  CA1
....................gugGccgaaacuuugacucaaccu...................................................................  1     1  CA1
....................gugcccgaaacuuAgacucaaccu...................................................................  1     1  CA1
....................gugcccgaaacuuugUcucaaccu...................................................................  19    1  CA1
....................gGgcccgaaacuuugacucaaccu...................................................................  1     1  CA1
....................gugcccgaaacuAugacucaaccu...................................................................  1     1  CA1
....................gugcccgaGacuuugacucaaccu...................................................................  7     1  CA1
....................gugcccAaaacuuugacucaaccu...................................................................  6     1  CA1
....................gugcccgaaacuuugacucaaUcu...................................................................  5     1  CA1
....................gugcccgaaacuuCgacucaaccu...................................................................  3     1  CA1
....................gAgcccgaaacuuugacucaaccu...................................................................  1     1  CA1
....................gugcccgaaGcuuugacucaaccu...................................................................  9     1  CA1
....................gugcccgaaCcuuugacucaaccu...................................................................  1     1  CA1
....................gugcccgaaacuuugacAcaaccu...................................................................  3     1  CA1
....................gugcccgaaacuuugacucaaccA...................................................................  243   1  CA1
....................gugcccgaaacuuugacucaGccu...................................................................  14    1  CA1
....................gugcccgaaacuuugacucGaccu...................................................................  11    1  CA1
....................gCgcccgaaacuuugacucaaccu...................................................................  10    1  CA1
....................guAcccgaaacuuugacucaaccu...................................................................  1     1  CA1
....................gugcccgaUacuuugacucaaccu...................................................................  1     1  CA1
....................gugAccgaaacuuugacucaaccu...................................................................  1     1  CA1
....................gugcUcgaaacuuugacucaaccu...................................................................  6     1  CA1
....................gugcccgGaacuuugacucaaccu...................................................................  10    1  CA1
....................gugcccgaaacuCugacucaaccu...................................................................  10    1  CA1
....................gugcccgUaacuuugacucaaccu...................................................................  4     1  CA1
....................gugcccgaaacuuugacucaaccu...................................................................  4090  0  CA1
....................gugccAgaaacuuugacucaaccu...................................................................  1     1  CA1
....................gugcccgaaacuGugacucaaccu...................................................................  1     1  CA1
....................gugcccgaaacuuugacucaaccuA..................................................................  18    1  CA1
....................gugcccgaaacuuugacucaaccuU..................................................................  64    1  CA1
....................gugcccgaaacuuugacucaaccug..................................................................  1     0  CA1
....................gugcccgaaacuuugacucaaccuC..................................................................  11    1  CA1
.....................ugcccgaaacuuugacuca.......................................................................  3     0  CA1
.....................ugcccgaaacuuugacucaa......................................................................  4     0  CA1
.....................ugcccgaaacuuugacucaac.....................................................................  11    0  CA1
.....................ugcccgaaacuuugacucaacA....................................................................  2     1  CA1
.....................ugcccgaaacuuugacucaacc....................................................................  14    0  CA1
.....................ugcccgaaacuuCgacucaacc....................................................................  1     1  CA1
.....................ugccUgaaacuuugacucaacc....................................................................  1     1  CA1
.....................ugcccgaaacuuugacucaaccA...................................................................  4     1  CA1
.....................ugcccgaaacuuugacucaaccu...................................................................  129   0  CA1
.....................ugcccgaaacuuugacucaaccG...................................................................  2     1  CA1
.....................ugcccgaaacuuugacucaaccC...................................................................  22    1  CA1
.....................ugcccgaaacuCugacucaaccu...................................................................  3     1  CA1
.....................ugcccgaaacuuugacucaaccuU..................................................................  8     1  CA1
```

```
                    Star                                   Mature
ugagagaaugucggccuugugugcccgaaacuuugacucaaccugaguuuaggaaaaggugagcaaaguuucaggugugucggguugacugaaaaaacugguuuaccagga
.....................ugcccgaaacuuugacucaaccug..................................................................  1    0  CA1
......................gcccgaaacuuugacucaaccu...................................................................  3    0  CA1
........................ccgaaacuuugacucaaccu...................................................................  3    0  CA1
..........................gaaacuuugacucaaccu...................................................................  3    0  CA1
.........................................................Cgugagcaaaguuucaggugug................................  2    1  CA1
.........................................................ggugagcaaaguuucaggugug................................  2    0  CA1
.........................................................ggugagcaaaguuucaggugugA...............................  2    1  CA1
.........................................................ggugagcaaaguuucaggugugu...............................  3    0  CA1
.........................................................Cgugagcaaaguuucaggugugu...............................  21   1  CA1
..........................................................gugagcaaaguuucaggu...................................  10   0  CA1
..........................................................gugagcaaaguuucaggC...................................  1    1  CA1
..........................................................gugagcaaaguuucagguU..................................  2    1  CA1
..........................................................gugagcaaaguuucaggCg..................................  1    1  CA1
..........................................................gugagcaaaguuucagguA..................................  5    1  CA1
..........................................................gugagcaaaguuucaggug..................................  55   0  CA1
..........................................................gugagcaaaguuucaggugu.................................  135  0  CA1
..........................................................gugagcaaaguuucaggugC.................................  16   1  CA1
..........................................................gugagcaaaguuucaggugG.................................  3    1  CA1
..........................................................gugagcaaaguuucaggGgu.................................  1    1  CA1
..........................................................gugagcaaGguuucaggugu.................................  1    1  CA1
..........................................................gugagcaGaguuucaggugu.................................  1    1  CA1
..........................................................gugagcaaaguuucaggugGg................................  1    1  CA1
..........................................................gCgagcaaaguuucaggugug................................  1    1  CA1
..........................................................gugagcaaaguuucagguguA................................  43   1  CA1
..........................................................gugagcaaGguuucaggugug................................  3    1  CA1
..........................................................guAagcaaaguuucaggugug................................  1    1  CA1
..........................................................gugagcaaaguuucaggugCg................................  3    1  CA1
..........................................................gugagcaaaguuucaggugug................................  580  0  CA1
..........................................................gugagcaUaguuucaggugug................................  1    1  CA1
..........................................................gugagcaaaguuucagAugug................................  2    1  CA1
..........................................................Augagcaaaguuucaggugug................................  1    1  CA1
..........................................................gugGgcaaaguuucaggugug................................  5    1  CA1
..........................................................gugagcaaaguuucUggugug................................  1    1  CA1
..........................................................gugagcaaaguuucagguguC................................  7    1  CA1
..........................................................gugagcaaaguCucaggugug................................  2    1  CA1
..........................................................gugagcaaagGuucaggugug................................  1    1  CA1
..........................................................gugagcaaaguuucCggugug................................  1    1  CA1
..........................................................gugagcaaaguuucagguguU................................  12   1  CA1
..........................................................gugagcUaaguuucaggugug................................  1    1  CA1
..........................................................gugagcaaaguuucGggugug................................  6    1  CA1
..........................................................gugagcaaaguuucaggCgug................................  4    1  CA1
..........................................................gugagcaGaguuucaggugug................................  1    1  CA1
..........................................................gugagcaaaguuucaggugAg................................  1    1  CA1
..........................................................gugagcaaaguuUaggugug.................................  3    1  CA1
..........................................................gugagAaaaguuucaggugugu...............................  3    1  CA1
..........................................................gugagcaaaguGucaggugugu...............................  1    1  CA1
..........................................................gugagcaaaguuucaggAgugu...............................  11   1  CA1
..........................................................gGgagcaaaguuucaggugugu...............................  3    1  CA1
..........................................................gugagcaaaguuAcaggugugu...............................  2    1  CA1
..........................................................gugagcaUaguuucaggugugu...............................  9    1  CA1
..........................................................gugGgcaaaguuucaggugugu...............................  46   1  CA1
..........................................................gugagcaaaguuucGggugugu...............................  49   1  CA1
..........................................................gugagcaaaguuucCggugugu...............................  3    1  CA1
..........................................................gugagcaaaguuucaggGgugu...............................  5    1  CA1
..........................................................gCgagcaaaguuucaggugugu...............................  20   1  CA1
..........................................................gugaUcaaaguuucaggugugu...............................  1    1  CA1
..........................................................gugagcaaaguuucaggugGgu...............................  14   1  CA1
..........................................................gugagcaaaguuucagguguCu...............................  3    1  CA1
..........................................................gugagcaaaguuCcaggugugu...............................  30   1  CA1
..........................................................gugagcCaaguuucaggugugu...............................  4    1  CA1
..........................................................gugagcaaaguuucaAgugugu...............................  5    1  CA1
..........................................................gugagcaaaguuucaggugugG...............................  190  1  CA1
..........................................................gugagcaCaguuucaggugugu...............................  1    1  CA1
..........................................................gugagcaaagCuucaggugugu...............................  38   1  CA1
..........................................................gugagcaaaguuucaggugCgu...............................  31   1  CA1
..........................................................gugagcaaaguuucaggCgugu...............................  23   1  CA1
..........................................................gugagcaaaguuucagguguUu...............................  4    1  CA1
..........................................................gAgagcaaaguuucaggugugu...............................  2    1  CA1
..........................................................gugagcaaagGuucaggugugu...............................  3    1  CA1
..........................................................gugagcaaaguAucaggugugu...............................  1    1  CA1
```

Star **Mature**

```
ugagagaaugucggccuugugugcccgaaacuuugacucaaccugaguuuaggaaaaggugagcaaaguuucaggugugucggguugacugaaaaaacugguuuaccagga
..........................................................gugagcaaaAuuucaggugugu............................... 3      1   CA1
..........................................................gugagcaaaguuuUaggugugu............................... 20     1   CA1
..........................................................gugagcaaaguuucUggugugu............................... 11     1   CA1
..........................................................gugagcaaGguuucaggugugu............................... 45     1   CA1
..........................................................Augagcaaaguuucaggugugu............................... 12     1   CA1
..........................................................gugagcGaaguuucaggugugu............................... 28     1   CA1
..........................................................gugagcaaaguuucaggugugAu............................... 8      1   CA1
..........................................................gugagcaaaguuucagguAugu............................... 8      1   CA1
..........................................................Cugagcaaaguuucaggugugu............................... 3      1   CA1
..........................................................gugaAcaaaguuucaggugugu............................... 18     1   CA1
..........................................................gugagcaGaguuucaggugugu............................... 33     1   CA1
..........................................................gugUgcaaaguuucaggugugu............................... 5      1   CA1
..........................................................gugagcaaaguuucaUgugugu............................... 3      1   CA1
..........................................................guUagcaaaguuucaggugugu............................... 1      1   CA1
..........................................................gugagcaaaguuGcaggugugu............................... 4      1   CA1
..........................................................gugagcaaUguuucaggugugu............................... 5      1   CA1
..........................................................gugCgcaaaguuucaggugugu............................... 3      1   CA1
..........................................................gugagcaaaguuucaggugugA............................... 303    1   CA1
..........................................................gugagUaaaguuucaggugugu............................... 17     1   CA1
..........................................................gugagcaaaguuucaggugugu............................... 11198  0   CA1
..........................................................gugagcaaaguCucaggugugu............................... 20     1   CA1
..........................................................gugagcaaaguuucaggugAgu............................... 14     1   CA1
..........................................................Uugagcaaaguuucaggugugu............................... 4      1   CA1
..........................................................guAagcaaaguuucaggugugu............................... 7      1   CA1
..........................................................gugagcaaaguuucagAugugu............................... 2      1   CA1
..........................................................gugagcUaaguuucaggugugu............................... 1      1   CA1
..........................................................gugagcaaaguuucagguUugu............................... 6      1   CA1
..........................................................gugagGaaaguuucaggugugu............................... 2      1   CA1
..........................................................gugagcaaaguuucaggugugucu.............................. 33     0   CA1
..........................................................gugagcaaaguuucaggugugG.............................. 21     1   CA1
..........................................................gugagcaaaguuucGgguguguc.............................. 2      1   CA1
...........................................................ugagcaaaguuucaggugugu............................... 9      0   CA1
...........................................................ugagcaaaguuucaggGgugu............................... 1      1   CA1
...........................................................ugGgcaaaguuucaggugugu............................... 1      1   CA1
............................................................gagcaaaguuucaggugug................................ 1      0   CA1
............................................................gagcaaaguuucaggugugu............................... 5      0   CA1
.............................................................agcaaaguuucaggugugu............................... 2      0   CA1

...................uUugcccgaaacuuugacucaacc.................................................................... 1      1   3rd
....................gugcccgaaacuuugacu......................................................................... 1      0   3rd
....................gugcccgaaacuuugacuca....................................................................... 5      0   3rd
....................gugcccgaaacuuugacucaa...................................................................... 8      0   3rd
....................gugcccgaaacuuugacucaac..................................................................... 7      0   3rd
....................gugcccgaaacuuugacucaacA.................................................................... 1      1   3rd
....................gugcccgaaacuuugacucaacc.................................................................... 39     0   3rd
....................gugcccgGaacuuugacucaaccu................................................................... 1      1   3rd
....................gugcccgaaacuuugacucaUccu................................................................... 1      1   3rd
....................gugcccgaaacuuugacucaaccu................................................................... 234    0   3rd
....................gugcccgaaGcuuugacucaaccu................................................................... 1      1   3rd
....................gugcccgaaacuuugUcucaaccu................................................................... 3      1   3rd
....................gugcccgaaacuuugacuUaaccu................................................................... 1      1   3rd
....................gugcccgaaacuuugacucaaccuU.................................................................. 1      1   3rd
....................gugcccgaaacuuugacucaaccuA.................................................................. 2      1   3rd
.....................ugcccgaaacuuugacucaa...................................................................... 1      0   3rd
.....................ugcccgaaacuuugacucaac..................................................................... 1      0   3rd
.....................ugcccgaaacuuugacucaacc.................................................................... 4      0   3rd
.....................ugcccgaaacuuugacucaaccu................................................................... 25     0   3rd
.....................ugcccgaaacuuugacucaaccuA.................................................................. 1      1   3rd
.....................ugcccgaaacuuugacucaaccug.................................................................. 1      0   3rd
.....................ugcccgaaacuuugacucaaccuU.................................................................. 1      1   3rd
........................ccgaaacuuugacucaaccuU.................................................................. 1      1   3rd
.....................................................aaaGggugagcaaaguuucaggugugu............................... 1      1   3rd
......................................................aaGggugagcaaaguuucaggugug................................ 2      1   3rd
......................................................aaaUgugagcaaaguuucaggugugu............................... 1      1   3rd
......................................................aaGggugagcaaaguuucaggugugu............................... 14     1   3rd
......................................................aaUggugagcaaaguuucaggugugu............................... 1      1   3rd
......................................................aaaAgugagcaaaguuucaggugugu............................... 1      1   3rd
......................................................Gaaggugagcaaaguuucaggugugu............................... 2      1   3rd
......................................................aaaCgugagcaaaguuucaggugugu............................... 2      1   3rd
.......................................................aGggugagcaaaguuucaggugu................................. 3      1   3rd
```

```
                    Star                                    Mature
ugagagaaugucggccuugugugcccgaaacuuugacucaaccugaguuuaggaaaaggugagcaaaguuucaggugugucggguugacugaaaaaacugguuuaccagga
.......................................................aaCgugagcaaaguuucaggugugu...............................  1    1  3rd
.......................................................aGggugagcaaaguuucaggugugu...............................  23   1  3rd
.......................................................aaggugagcaaaguuucaggugugU..............................  1    1  3rd
........................................................aggugagcaaaguuucaggugu.................................  2    0  3rd
........................................................aggugagcaaaguuucaggugug................................  1    0  3rd
........................................................Gggugagcaaaguuucaggugug................................  1    1  3rd
........................................................aUgugagcaaaguuucaggugugu...............................  1    1  3rd
........................................................aggugagcaaaguuucagguguUu...............................  1    1  3rd
........................................................aCgugagcaaaguuucaggugugu...............................  1    1  3rd
........................................................Uggugagcaaaguuucaggugugu...............................  1    1  3rd
........................................................Gggugagcaaaguuucaggugugu...............................  2    1  3rd
.........................................................ggugagcaaaguuucagg....................................  1    0  3rd
.........................................................ggugagcaaaguuucaggug..................................  5    0  3rd
.........................................................ggugagcaaaguuucCggug..................................  1    1  3rd
.........................................................ggugagcaaaguuucGggugu.................................  1    1  3rd
.........................................................Ugugagcaaaguuucaggugu.................................  10   1  3rd
.........................................................ggugagcaaaguuucaggugu.................................  59   0  3rd
.........................................................gguCagcaaaguuucaggugug................................  1    1  3rd
.........................................................ggugagcaaaguuucagguguU................................  3    1  3rd
.........................................................Cgugagcaaaguuucaggugug................................  1    1  3rd
.........................................................Ugugagcaaaguuucaggugug................................  30   1  3rd
.........................................................ggugagcaaaguuucaggugug................................  168  0  3rd
.........................................................Agugagcaaaguuucaggugug................................  1    1  3rd
.........................................................ggGgagcaaaguuucaggugug................................  1    1  3rd
.........................................................ggugagcaaagAuucaggugug................................  1    1  3rd
.........................................................ggugagcaaaguuucaggugugA...............................  17   1  3rd
.........................................................ggugGgcaaaguuucaggugugu...............................  2    1  3rd
.........................................................ggugCgcaaaguuucaggugugu...............................  1    1  3rd
.........................................................ggugagUaaaguuucaggugugu...............................  1    1  3rd
.........................................................ggugagcaaaguuucagguguUu...............................  12   1  3rd
.........................................................ggAgagcaaaguuucaggugugu...............................  1    1  3rd
.........................................................Agugagcaaaguuucaggugugu...............................  9    1  3rd
.........................................................ggugaUcaaaguuucaggugugu...............................  1    1  3rd
.........................................................ggugagcaaaguuucagCugugu...............................  2    1  3rd
.........................................................ggGgagcaaaguuucaggugugu...............................  12   1  3rd
.........................................................gAugagcaaaguuucaggugugu...............................  1    1  3rd
.........................................................Cgugagcaaaguuucaggugugu...............................  4    1  3rd
.........................................................gCugagcaaaguuucaggugugu...............................  1    1  3rd
.........................................................ggugagcaaaguuucaggugugu...............................  292  0  3rd
.........................................................Ugugagcaaaguuucaggugugu...............................  386  1  3rd
..........................................................Cugagcaaaguuucaggu...................................  1    1  3rd
..........................................................gugagcaaaguuucaggu...................................  729  0  3rd
..........................................................gugagcaaaguuucaggA...................................  3    1  3rd
..........................................................gugagcaaaguuucaggC...................................  3    1  3rd
..........................................................gugagcaaaguuucGggu...................................  1    1  3rd
..........................................................guCagcaaaguuucaggu...................................  1    1  3rd
..........................................................gugagAaaaguuucaggu...................................  1    1  3rd
..........................................................gugagcaaaguuucCggu...................................  3    1  3rd
..........................................................gugagcaaaguuucaggGg..................................  1    1  3rd
..........................................................gugagcaaaguuucagguA..................................  9    1  3rd
..........................................................gugagcaaagAuucaggug..................................  1    1  3rd
..........................................................gugagcaaaguuuGaggug..................................  1    1  3rd
..........................................................gAgagcaaaguuucaggug..................................  1    1  3rd
..........................................................gugagcaaaguuucaggAg..................................  1    1  3rd
..........................................................gugagcaaaguuucaggug..................................  979  0  3rd
..........................................................gugagcaaaguuucagguU..................................  10   1  3rd
..........................................................gugagcaaaguuucagAug..................................  2    1  3rd
..........................................................gugagcUaaguuucaggug..................................  1    1  3rd
..........................................................gugagcaaaguuucagguC..................................  9    1  3rd
..........................................................gugagAaaaguuucaggug..................................  3    1  3rd
..........................................................gugagcaaaguAucaggugu.................................  2    1  3rd
..........................................................gugagcaaagGuucaggugu.................................  5    1  3rd
..........................................................gugaAcaaaguuucaggugu.................................  1    1  3rd
..........................................................guCagcaaaguuucaggugu.................................  4    1  3rd
..........................................................gugagcaaagCuucaggugu.................................  3    1  3rd
..........................................................gugagcaaaguuucagCugu.................................  2    1  3rd
..........................................................gugagcaaaguuucaggugC.................................  15   1  3rd
..........................................................gugagcaaaguuucagUugu.................................  3    1  3rd
..........................................................gugagcaaaUuuucaggugu.................................  2    1  3rd
..........................................................gugagcaaGguuucaggugu.................................  3    1  3rd
```

```
                    Star                                 Mature
ugagagaaugucggccuugugugcccgaaacuuugacucaaccugaguuuaggaaaaggugagcaaaguuucaggugugucgguugacugaaaaaacugguuuaccagga
..........................................................gugagcaaaguGucaggugu................................  1      1    3rd
..........................................................gugagAaaaguuucaggugu................................  9      1    3rd
..........................................................guUagcaaaguuucaggugu................................  1      1    3rd
..........................................................gugagcaCaguuucaggugu................................  3      1    3rd
..........................................................gugagcaaaguuucaUgugu................................  2      1    3rd
..........................................................gugagcaaaguuuGaggugu................................  2      1    3rd
..........................................................gugagcaaaguuucaggugG................................  9      1    3rd
..........................................................gugagcaaaguuucGggugu................................  5      1    3rd
..........................................................gAgagcaaaguuucaggugu................................  8      1    3rd
..........................................................gugagcaaaguuucaCgugu................................  3      1    3rd
..........................................................gugagcaaaCuuucaggugu................................  5      1    3rd
..........................................................gugagcaUaguuucaggugu................................  3      1    3rd
..........................................................Uugagcaaaguuucaggugu................................  1      1    3rd
..........................................................gugagcaaaguuucaggGgu................................  21     1    3rd
..........................................................gugagcaaCguuucaggugu................................  1      1    3rd
..........................................................gugUgcaaaguuucaggugu................................  1      1    3rd
..........................................................gugagcaaaguuucaggugu................................  11747  0    3rd
..........................................................gugagcaaaguuuUaggugu................................  3      1    3rd
..........................................................gugagUaaaguuucaggugu................................  8      1    3rd
..........................................................gugagcaaaguuucaggAgu................................  2      1    3rd
..........................................................gugagcaaaguuuAaggugu................................  1      1    3rd
..........................................................gugaUcaaaguuucaggugu................................  3      1    3rd
..........................................................gugagcaaaguuucagguCu................................  2      1    3rd
..........................................................gugagcaaaguCucaggugu................................  1      1    3rd
..........................................................gugagcaaaguuucaAgugu................................  5      1    3rd
..........................................................gugCgcaaaguuucaggugu................................  2      1    3rd
..........................................................gGgagcaaaguuucaggugu................................  5      1    3rd
..........................................................gugagcaaaguuucagAugu................................  2      1    3rd
..........................................................gugagcaaaguuucaggCgu................................  5      1    3rd
..........................................................gugagcaaaguuucUggugu................................  5      1    3rd
..........................................................gugagGaaaguuucaggugu................................  5      1    3rd
..........................................................gugagcUaaguuucaggugu................................  6      1    3rd
..........................................................gCgagcaaaguuucaggugu................................  2      1    3rd
..........................................................gugGgcaaaguuucaggugu................................  11     1    3rd
..........................................................gugagcaaagAuucaggugu................................  1      1    3rd
..........................................................gugagcCaaguuucaggugu................................  1      1    3rd
..........................................................guCagcaaaguuucaggugug...............................  10     1    3rd
..........................................................gugagcaaaguuAcaggugug...............................  1      1    3rd
..........................................................gugagcaaagCuucaggugug...............................  6      1    3rd
..........................................................gugagcaaaguuucagAugug...............................  10     1    3rd
..........................................................guAagcaaaguuucaggugug...............................  4      1    3rd
..........................................................gugagcaaaguuucaCgugug...............................  22     1    3rd
..........................................................gugUgcaaaguuucaggugug...............................  5      1    3rd
..........................................................gugagcaaaguuucagguguU...............................  1000   1    3rd
..........................................................gugagcaaaguuuAaggugug...............................  6      1    3rd
..........................................................gugagcaaaguuucaggugGg...............................  11     1    3rd
..........................................................gugagcaaaguuucaAgugug...............................  9      1    3rd
..........................................................gugagcaaaguuucaggugug...............................  35459  0    3rd
..........................................................gugagcGaaguuucaggugug...............................  6      1    3rd
..........................................................gugagcaUaguuucaggugug...............................  8      1    3rd
..........................................................gugagcaaaguuucagguguC...............................  12     1    3rd
..........................................................gugCgcaaaguuucaggugug...............................  7      1    3rd
..........................................................gugagcaaaguuucagCugug...............................  11     1    3rd
..........................................................gugagcaaaguuuGaggugug...............................  1      1    3rd
..........................................................gugagcaaaguuucaggCgug...............................  8      1    3rd
..........................................................gugagcaaagGuucaggugug...............................  11     1    3rd
..........................................................gugagcaaaguuucaggugAg...............................  8      1    3rd
..........................................................guUagcaaaguuucaggugug...............................  1      1    3rd
..........................................................gugagcaaaguGucaggugug...............................  5      1    3rd
..........................................................gugagcaaaguuucaUgugug...............................  4      1    3rd
..........................................................gugaCcaaaguuucaggugug...............................  3      1    3rd
..........................................................gGgagcaaaguuucaggugug...............................  6      1    3rd
..........................................................gugagcaaUguuucaggugug...............................  2      1    3rd
..........................................................gugagAaaaguuucaggugug...............................  21     1    3rd
..........................................................gugagcaaaguuucagguUug...............................  6      1    3rd
..........................................................gugagcaaaguuucagguCug...............................  5      1    3rd
..........................................................gugagcaaCguuucaggugug...............................  2      1    3rd
..........................................................gugaAcaaaguuucaggugug...............................  12     1    3rd
..........................................................gugaUcaaaguuucaggugug...............................  6      1    3rd
..........................................................gugagcUaaguuucaggugug...............................  43     1    3rd
```

Star Mature

```
ugagagaaugucggccuugugugcccgaaacuuugacucaaccugaguuuaggaaaaggugagcaaaguuucaggugugucggguugacugaaaaacugguuuaccagga
..........................................................gugagcaaaguuucaggGgug...............................	32	1	3rd
..........................................................gAgagcaaaguuucaggugug...............................	25	1	3rd
..........................................................gugagcaaaguuucagguAug...............................	3	1	3rd
..........................................................gugagcaaaguuucagUugug...............................	8	1	3rd
..........................................................Augagcaaaguuucaggugug...............................	4	1	3rd
..........................................................gugagcaaaguuucGggugug...............................	19	1	3rd
..........................................................gCgagcaaaguuucaggugug...............................	3	1	3rd
..........................................................gugagcaGaguuucaggugug...............................	3	1	3rd
..........................................................gugagcaaaguCucaggugug...............................	2	1	3rd
..........................................................gugagcaaGguuucaggugug...............................	2	1	3rd
..........................................................gugGgcaaaguuucaggugug...............................	24	1	3rd
..........................................................gugagcaaaguuuUaggugug...............................	16	1	3rd
..........................................................gugagcaaaguuucaggAgug...............................	3	1	3rd
..........................................................gugagcaCaguuucaggugug...............................	5	1	3rd
..........................................................gugagcaaaguuucCggugug...............................	2	1	3rd
..........................................................gugagcaaaAuuucaggugug...............................	1	1	3rd
..........................................................gugagcaaagAuucaggugug...............................	4	1	3rd
..........................................................Cugagcaaaguuucaggugug...............................	2	1	3rd
..........................................................gugagUaaaguuucaggugug...............................	27	1	3rd
..........................................................gugagcaaaguuCcaggugug...............................	4	1	3rd
..........................................................gugagcaaaguuucagguguA...............................	87	1	3rd
..........................................................gugagcaaaguuucaggugCg...............................	22	1	3rd
..........................................................gugagGaaaguuucaggugug...............................	17	1	3rd
..........................................................gugagcaaaCuuucaggugug...............................	5	1	3rd
..........................................................gugagcaaaguuGcaggugug...............................	1	1	3rd
..........................................................gugagcaaaguuucUggugug...............................	13	1	3rd
..........................................................gugagcCaaguuucaggugug...............................	2	1	3rd
..........................................................gugagcaaaguuucagguUugu..............................	72	1	3rd
..........................................................gugagcaaaguuucagAugugu..............................	171	1	3rd
..........................................................gugagcaaaguuucGggugugu..............................	363	1	3rd
..........................................................gugagcUaaguuucaggugugu..............................	797	1	3rd
..........................................................gugGgcaaaguuucaggugugu..............................	271	1	3rd
..........................................................guAagcaaaguuucaggugugu..............................	117	1	3rd
..........................................................gugaUcaaaguuucaggugugu..............................	54	1	3rd
..........................................................gugagcaaGguuucaggugugu..............................	37	1	3rd
..........................................................gugaCcaaaguuucaggugugu..............................	107	1	3rd
..........................................................gugagcaaaguuucaAgugugu..............................	158	1	3rd
..........................................................guUagcaaaguuucaggugugu..............................	59	1	3rd
..........................................................gugagcaaaguuAcaggugugu..............................	2	1	3rd
..........................................................gugagcaaaguGucaggugugu..............................	37	1	3rd
..........................................................gugagcaaaguuucaggugAgu..............................	75	1	3rd
..........................................................gugagcaaCguuucaggugugu..............................	8	1	3rd
..........................................................gugagcaaaguuuUaggugugu..............................	309	1	3rd
..........................................................gugagcaaaguuCcaggugugu..............................	73	1	3rd
..........................................................gugaAcaaaguuucaggugugu..............................	183	1	3rd
..........................................................gugagUaaaguuucaggugugu..............................	578	1	3rd
..........................................................gugagcaaaguuuGaggugugu..............................	47	1	3rd
..........................................................Uugagcaaaguuucaggugugu..............................	87	1	3rd
..........................................................gCgagcaaaguuucaggugugu..............................	92	1	3rd
..........................................................gugagcGaaguuucaggugugu..............................	81	1	3rd
..........................................................gugagcaaagCuucaggugugu..............................	123	1	3rd
..........................................................gAgagcaaaguuucaggugugu..............................	490	1	3rd
..........................................................gugagcaaaguuucagguAugu..............................	54	1	3rd
..........................................................gugCgcaaaguuucaggugugu..............................	118	1	3rd
..........................................................gugagcaGaguuucaggugugu..............................	80	1	3rd
..........................................................gugagcaaaguuucaggugGgu..............................	233	1	3rd
..........................................................gugagcaaaguuucagguguAu..............................	146	1	3rd
..........................................................gGgagcaaaguuucaggugugu..............................	238	1	3rd
..........................................................gugagcaaaguuucagguguCu..............................	98	1	3rd
..........................................................Cugagcaaaguuucaggugugu..............................	48	1	3rd
..........................................................gugagcaaaAuuucaggugugu..............................	46	1	3rd
..........................................................gugagcaaaguuucagguCugu..............................	78	1	3rd
..........................................................gugagcaCaguuucaggugugu..............................	110	1	3rd
..........................................................gugagcaaaguuucaggugugu..............................	668121	0	3rd
..........................................................gugagcaaaguuucagCugugu..............................	198	1	3rd
..........................................................gugagcaaaguCucaggugugu..............................	80	1	3rd
..........................................................gugagcaaaguuucaggugCgu..............................	143	1	3rd
..........................................................gugagcaaaguuucCggugugu..............................	24	1	3rd
..........................................................gugagcaaUguuucaggugugu..............................	32	1	3rd
..........................................................gugagcaaagGuucaggugugu..............................	199	1	3rd
```

Star **Mature**

ugagagaaugucggcuugugugcccgaaacuuugacucaaccugaguuuaggaaaaggugagcaaaguuucaggugugucggguugacugaaaaacugguuuaccagga			
....gugagcaaaguuucaggAgugu..............................	63	1	3rd
....gugagcaaaguuucagguguUu..............................	1758	1	3rd
....gugagcaaaguuucaCgugugu..............................	199	1	3rd
....gugagcaaaguuucagUugugu..............................	172	1	3rd
....Augagcaaaguuucaggugugu..............................	58	1	3rd
....gugagcaaaguAucaggugugu..............................	25	1	3rd
....gugagcaUaguuucaggugugu..............................	123	1	3rd
....gugagcaaaguuucaggugugA..............................	3691	1	3rd
....gugagcaaaguuucUggugugu..............................	268	1	3rd
....gugagcaaaguuucaggGgugu..............................	296	1	3rd
....gugagcaaaguuGcaggugugu..............................	12	1	3rd
....gugagcaaaUuuucaggugugu..............................	38	1	3rd
....gugagGaaaguuucaggugugu..............................	268	1	3rd
....gugagcaaagAuucaggugugu..............................	90	1	3rd
....gugagcaaaCuuucaggugugu..............................	92	1	3rd
....gugagcaaaguuucaUgugugu..............................	143	1	3rd
....gugagcCaaguuucaggugugu..............................	34	1	3rd
....gugagcaaaguuuAaggugugu..............................	46	1	3rd
....gugagcaaaguuucaggCgugu..............................	125	1	3rd
....guCagcaaaguuucaggugugu..............................	140	1	3rd
....gugagcaaaguuucaggugugG..............................	170	1	3rd
....gugagAaaaguuucaggugugu..............................	484	1	3rd
....gugUgcaaaguuucaggugugu..............................	149	1	3rd
....gugagcaaaguuucagguguguG.............................	89	1	3rd
....gugagcaaaguuucaggugugCc.............................	4	1	3rd
....gugagcaaaguuucagguguguc.............................	61	0	3rd
....gugagcaaaguuucaggugugucU............................	3	1	3rd
....gugagcaaaguuucaggugugucC............................	2	1	3rd
....gugagcaaaguuucaggugugucg............................	1	0	3rd
....gugagcaaaguuucaggugugucgC...........................	1	1	3rd
....gugagcaaaguuucagguguguGgg...........................	5	1	3rd
....gugagcaaaguuucaggugugucCg...........................	2	1	3rd
....gugagcaaaguuucagguguguGggg..........................	2	1	3rd
....gugagcaaaguuucagguguguGgggu.........................	1	1	3rd
....ugagcaaaguuucaggugu................................	3	0	3rd
....ugagcaaaguuucagguguU...............................	1	1	3rd
....ugagcaaaguuucaggugug...............................	6	0	3rd
....Agagcaaaguuucaggugugu..............................	1	1	3rd
....uUagcaaaguuucaggugugu..............................	1	1	3rd
....uCagcaaaguuucaggugugu..............................	2	1	3rd
....ugagcaaaguuucagguUugu..............................	1	1	3rd
....uAagcaaaguuucaggugugu..............................	1	1	3rd
....ugagcaaaguuucaggugugu..............................	109	0	3rd
....Ggagcaaaguuucaggugugu..............................	7	1	3rd
....ugagcaaaguuucagguguguG.............................	1	1	3rd
....Uagcaaaguuucaggugu................................	1	1	3rd
....gagcaaaguuucaggugu................................	8	0	3rd
....gagcaaaguuucaggugug...............................	24	0	3rd
....Uagcaaaguuucaggugugu..............................	1	1	3rd
....gagcaaaguuucaggugugu..............................	302	0	3rd
....gCgcaaaguuucaggugugu..............................	1	1	3rd
....gagcaGaguuucaggugugu..............................	1	1	3rd
....Cagcaaaguuucaggugugu..............................	1	1	3rd
....gagcaCaguuucaggugugu..............................	1	1	3rd
....agcaaaguuucaggugug...............................	2	0	3rd
....agcaaaguuucaggugugu..............................	108	0	3rd
....agcaaaguuucagUugugu..............................	1	1	3rd
....agcaaaguuucagguguUu..............................	1	1	3rd
....agcaaaguuucaggugugA..............................	2	1	3rd
....agcaaaguuucagguguAu..............................	1	1	3rd
....Ucaaaguuucaggugugu..............................	2	1	3rd
....gcaaaguuucaggugugu..............................	119	0	3rd
..................Cgugcccgaaacuuugacuca..	1	1	BA1
..................Cgugcccgaaacuuugacucaaccu..	1	1	BA1
...................gugcccgaaacuuugacA..	1	1	BA1
...................gugcccgaaacuuugacu..	5	0	BA1
...................gugcccgaaacGuugacuc...	1	1	BA1
...................gugcccgaaacuuugUcuc...	5	1	BA1
...................gugcccgaaacuuugacuU...	7	1	BA1

```
                    Star                            Mature
ugagagaaugucggccuugugugcccgaaacuuugacucaaccugaguuuaggaaaaggugagcaaaguuucaggugugucggguugacugaaaaaacugguuuaccagga
....................gugcccgaaacuuugacuA........................................................................ 1     1  BA1
....................gugcccgaaacuuugacuc........................................................................ 33    0  BA1
....................gugcccgaaacuuugacCc........................................................................ 1     1  BA1
....................gugcUcgaaacuuugacuca....................................................................... 1     1  BA1
....................gugcccgGaacuuugacuca....................................................................... 1     1  BA1
....................gugcccgaaacuuugacCca....................................................................... 3     1  BA1
....................gugcccgaaacuuugacucG....................................................................... 3     1  BA1
....................gugcccgaaacuCugacuca....................................................................... 2     1  BA1
....................gugcccgaaacuuugUcuca....................................................................... 2     1  BA1
....................gugcccgaaacuuugacucU....................................................................... 5     1  BA1
....................gugcccgaaacuuugacuca....................................................................... 133   0  BA1
....................gugcccgaaacuuCgacuca....................................................................... 1     1  BA1
....................gugcccgaaacuuugacucaG...................................................................... 2     1  BA1
....................gugcccgaaacuuugacucaa...................................................................... 84    0  BA1
....................gAgcccgaaacuuugacucaa...................................................................... 1     1  BA1
....................gugcccgaaacuuugCcucaa...................................................................... 1     1  BA1
....................gugcccgaaacuuugacucGa...................................................................... 2     1  BA1
....................gugcccgaaacuuugUcucaa...................................................................... 1     1  BA1
....................gugcccgaaGcuuugacucaa...................................................................... 1     1  BA1
....................gugcccgaaacuuugacucaaU..................................................................... 8     1  BA1
....................gugcccgaaacuuugacucaac..................................................................... 99    0  BA1
....................gugcccgaaacuCugacucaac..................................................................... 1     1  BA1
....................gugcccgaaacuuugUcucaac..................................................................... 7     1  BA1
....................gCgcccgaaacuuugacucaac..................................................................... 2     1  BA1
....................gugcccgaGacuuugacucaac..................................................................... 1     1  BA1
....................gugcccgaaacuuugCcucaac..................................................................... 1     1  BA1
....................gugcccgGaacuuugacucaacc.................................................................... 4     1  BA1
....................gugcccgaaacuuugacucGacc.................................................................... 4     1  BA1
....................gugcccgaaacuuugUcucaacc.................................................................... 21    1  BA1
....................gugcccgaaacuuugacCcaacc.................................................................... 2     1  BA1
....................gugcccgaaacuuugacucaacc.................................................................... 370   0  BA1
....................gugcccgCaacuuugacucaacc.................................................................... 1     1  BA1
....................gugccUgaaacuuugacucaacc.................................................................... 1     1  BA1
....................gugcccgaaGcuuugacucaacc.................................................................... 3     1  BA1
....................gugcUcgaaacuuugacucaacc.................................................................... 2     1  BA1
....................gugcccgaaacuuugacucaacA.................................................................... 20    1  BA1
....................gugcccgaaUcuuugacucaacc.................................................................... 2     1  BA1
....................gugcccgaaacuuugacuAaacc.................................................................... 1     1  BA1
....................gugcccgaaacuuCgacucaacc.................................................................... 1     1  BA1
....................gugcccgaaacuuugacucaacU.................................................................... 127   1  BA1
....................gAgcccgaaacuuugacucaacc.................................................................... 1     1  BA1
....................gugcccgaaacuuugacucaacG.................................................................... 3     1  BA1
....................gugcccgaaacuuugacucaaccG................................................................... 110   1  BA1
....................gugcccgaaacuuugUcucaaccu................................................................... 133   1  BA1
....................gugcUcgaaacuuugacucaaccu................................................................... 2     1  BA1
....................gugcccgaaacuuCgacucaaccu................................................................... 13    1  BA1
....................gugcccAaaacuuugacucaaccu................................................................... 3     1  BA1
....................gugcccgaaacuuugacucaaUcu................................................................... 1     1  BA1
....................guAcccgaaacuuugacucaaccu................................................................... 2     1  BA1
....................gugcccgaaacuuugacucGaccu................................................................... 17    1  BA1
....................gugcccgaaacuuugacuUaaccu................................................................... 6     1  BA1
....................gugcccgaaacuuugacucaaccu................................................................... 3893  0  BA1
....................gugcccgGaacuuugacucaaccu................................................................... 21    1  BA1
....................gugcccgaaacuuugCcucaaccu................................................................... 6     1  BA1
....................gugcccgaaacuuGgacucaaccu................................................................... 1     1  BA1
....................gugcccgaaacuGugacucaaccu................................................................... 2     1  BA1
....................gugcccgaaGcuuugacucaaccu................................................................... 18    1  BA1
....................gugcccgaaacuuugacucCaccu................................................................... 1     1  BA1
....................gugcccgaaCcuuugacucaaccu................................................................... 1     1  BA1
....................gugcccgaaacuCugacucaaccu................................................................... 8     1  BA1
....................gugcccgaaacuuugacucaGccu................................................................... 8     1  BA1
....................gugcccgaaaUuuugacucaaccu................................................................... 1     1  BA1
....................gugcccgaaacAuugacucaaccu................................................................... 2     1  BA1
....................gugccUgaaacuuugacucaaccu................................................................... 26    1  BA1
....................Augcccgaaacuuugacucaaccu................................................................... 8     1  BA1
....................gugcccgUaacuuugacucaaccu................................................................... 2     1  BA1
....................gugcccgaaacuuugaUucaaccu................................................................... 7     1  BA1
....................gAgcccgaaacuuugacucaaccu................................................................... 3     1  BA1
....................gugcccUaaacuuugacucaaccu................................................................... 2     1  BA1
....................gugcccgaaacuuuAacucaaccu................................................................... 1     1  BA1
```

```
                    Star                                  Mature
ugagagaaugucggccuuguGUGCCCGAAACUUUGACUCAACCUgaguuuaggaaaagGUGAGCAAAGUUUCAGGUGUGUcggguugacugaaaaaacugguuuaccagga
```

```
ugagagaaugucggccuugugugcccgaaacuuugacucaaccugaguuuaggaaaaggugagcaaaguuucaggugugucggguugacugaaaaaacugguuuaccagga
....................gugcccgaaacuuugacucaaccA...................................................................   455   1   BA1
....................gugcccgaaacuuugacCcaaccu...................................................................   19    1   BA1
....................gCgcccgaaacuuugacucaaccu...................................................................   6     1   BA1
....................gugcccgaaacuuugacucaacUu...................................................................   2     1   BA1
....................gugcccgaGacuuugacucaaccu...................................................................   15    1   BA1
....................gugcccgaaacuuugacucaaccC...................................................................   676   1   BA1
....................gugcccgaaacuuugacAcaaccu...................................................................   3     1   BA1
....................gugUccgaaacuuugacucaaccu...................................................................   3     1   BA1
....................gugcccgaaacuuugacucaaccuC..................................................................   9     1   BA1
....................gugcccgaaacuuugacucaaccuA..................................................................   7     1   BA1
....................gugcccgaaacuuugacucaaccuU..................................................................   121   1   BA1
.....................ugcccgaaacuuugacuca.......................................................................   1     0   BA1
.....................ugcccgaaacuuugacucaa......................................................................   10    0   BA1
.....................ugcccgaaacuuugacucaac.....................................................................   8     0   BA1
.....................ugcccgaaacuuugacucaacc....................................................................   10    0   BA1
.....................ugcccgaaacuuugacucaacA....................................................................   3     1   BA1
.....................ugcccgaaacuuugacucaacU....................................................................   4     1   BA1
.....................ugcccAaaacuuugacucaaccu...................................................................   1     1   BA1
.....................ugcccgaaacuuugacucaaccu...................................................................   282   0   BA1
.....................ugcccgaaacuuugacucaaccA...................................................................   25    1   BA1
.....................Agcccgaaacuuugacucaaccu...................................................................   1     1   BA1
.....................ugcccgaaacuuugacucaaccC...................................................................   27    1   BA1
.....................ugcccgaaacuuugacucaacUu...................................................................   1     1   BA1
.....................ugcccgGaacuuugacucaaccu...................................................................   3     1   BA1
.....................ugcccgaaacuuugacCcaaccu...................................................................   3     1   BA1
.....................ugcccgaaacuuugacucaaccG...................................................................   6     1   BA1
.....................ugcccgaaacuuCgacucaaccu...................................................................   2     1   BA1
.....................ugcccgaaacuuugUcucaaccu...................................................................   1     1   BA1
.....................ugcccgaaacuuugacucaaccuA..................................................................   8     1   BA1
.....................ugcccgaaacuuugacucaaccuC..................................................................   11    1   BA1
.....................ugcccgaaacuuugacucaaccuU..................................................................   58    1   BA1
..........................gaaacuuugacucaaccu...................................................................   3     0   BA1
.........................................................Cgugagcaaaguuucaggugugu...............................   12    1   BA1
.........................................................Ugugagcaaaguuucaggugugu...............................   1     1   BA1
.........................................................Agugagcaaaguuucaggugugu...............................   1     1   BA1
..........................................................gugagcaaaguuucaggu...................................   4     0   BA1
..........................................................gugagcaaaguuucaggC...................................   2     1   BA1
..........................................................gugagcaaaguuucaggug..................................   31    0   BA1
..........................................................gugagcaaaguuucagguA..................................   6     1   BA1
..........................................................gugagUaaaguuucaggug..................................   1     1   BA1
..........................................................gugagcaaaguuucUggugu.................................   2     1   BA1
..........................................................gugagcaaaguuucaggugC.................................   11    1   BA1
..........................................................gugagcaaaguuucaggugu.................................   76    0   BA1
..........................................................gAgagcaaaguuucaggugu.................................   1     1   BA1
..........................................................gugagcaaaguuucagguguU................................   17    1   BA1
..........................................................gugagcaaUguuucaggugug................................   1     1   BA1
..........................................................gugagcaaGguuucaggugug................................   5     1   BA1
..........................................................gugagcaaaguuucaggugug................................   274   0   BA1
..........................................................gugagcaaaguuucagguguA................................   31    1   BA1
..........................................................gugagcaaaguuucaggCgug................................   1     1   BA1
..........................................................gugagcGaaguuucaggugug................................   1     1   BA1
..........................................................gugagcaaaguuCcaggugug................................   1     1   BA1
..........................................................gugagcaaaguuucagguguC................................   1     1   BA1
..........................................................gugagcaaaguuucUggugugu...............................   4     1   BA1
..........................................................gugagcaGaguuucaggugugu...............................   14    1   BA1
..........................................................gugagcGaaguuucaggugugu...............................   12    1   BA1
..........................................................gugagcaaaguuucagAugugu...............................   2     1   BA1
..........................................................gugagcaaaguuucaAgugugu...............................   3     1   BA1
..........................................................gugaAcaaaguuucaggugugu...............................   2     1   BA1
..........................................................gugagcaaaguuAcaggugugu...............................   3     1   BA1
..........................................................gugGgcaaaguuucaggugugu...............................   26    1   BA1
..........................................................gugagcaaaguuucaggugCgu...............................   9     1   BA1
..........................................................gugagcaaaAuuucaggugugu...............................   1     1   BA1
..........................................................gugagcaaagCuucaggugugu...............................   12    1   BA1
..........................................................gugagcaaCguuucaggugugu...............................   1     1   BA1
..........................................................Augagcaaaguuucaggugugu...............................   7     1   BA1
..........................................................gugaUcaaaguuucaggugugu...............................   2     1   BA1
..........................................................gugagcaaaguuucGggugugu...............................   18    1   BA1
..........................................................gugagcaaaguuuUaggugugu...............................   4     1   BA1
..........................................................gugagcaaaguuCcaggugugu...............................   16    1   BA1
```

Star Mature

```
ugagagaaugucggccuugugugcccgaaacuuugacucaaccugaguuuaggaaaaggugagcaaaguuucaggugugucggguugacugaaaaaacugguuuaccagga
..........................................................gugagcaUaguuucaggugugu...............................  1     1   BA1
..........................................................gAgagcaaaguuucaggugugu...............................  3     1   BA1
..........................................................gugagcaaaguuucaggAgugu...............................  2     1   BA1
..........................................................gugagcaaaguuucaggCgugu...............................  16    1   BA1
..........................................................gugagcaaaguCucaggugugu...............................  17    1   BA1
..........................................................gugagUaaaguuucaggugugu...............................  5     1   BA1
..........................................................gugaCcaaaguuucaggugugu...............................  1     1   BA1
..........................................................gugagcaaUguuucaggugugu...............................  1     1   BA1
..........................................................gugagcaCaguuucaggugugu...............................  2     1   BA1
..........................................................gugagcaaaguuucaggugugu...............................  4211  0   BA1
..........................................................gugagcaaaguuucaUgugugu...............................  2     1   BA1
..........................................................gugagcaaaguuucaggGgugu...............................  2     1   BA1
..........................................................gugagcaaagAuucaggugugu...............................  1     1   BA1
..........................................................guUagcaaaguuucaggugugu...............................  1     1   BA1
..........................................................gugagcaaaguuucaggugugA...............................  299   1   BA1
..........................................................gugagcaaaguuucagguguAu...............................  3     1   BA1
..........................................................gugagcaaaguuucaCgugugu...............................  3     1   BA1
..........................................................gugagcaaaguuucagguAugu...............................  1     1   BA1
..........................................................gugagcaaaguuucaggugGgu...............................  5     1   BA1
..........................................................gugagcaaaguuucagguUugu...............................  1     1   BA1
..........................................................gCgagcaaaguuucaggugugu...............................  11    1   BA1
..........................................................gugagcaaaguuucaggugugG...............................  103   1   BA1
..........................................................gugagcUaaguuucaggugugu...............................  1     1   BA1
..........................................................gugUgcaaaguuucaggugugu...............................  7     1   BA1
..........................................................gugagcaaagGuucaggugugu...............................  4     1   BA1
..........................................................guAagcaaaguuucaggugugu...............................  2     1   BA1
..........................................................gugagcaaaguuucagguguUu...............................  13    1   BA1
..........................................................gugagcaaGguuucaggugugu...............................  16    1   BA1
..........................................................gugagcaaGguuucaggugugugc..............................  1     1   BA1
..........................................................gugagcaaaguuucaggugugugc..............................  15    0   BA1
..........................................................gugagcaaaguuucagguguguG..............................  10    1   BA1
..........................................................gugagcaaaguuucaggugugucggU...........................  1     1   BA1
...........................................................ugagcaaaguuucaggugugu...............................  3     0   BA1
...........................................................ugagcaaaguuucaggugugA...............................  1     1   BA1
............................................................gagcaaaguuucaggugugu...............................  2     0   BA1
............................................................gagcaaGguuucaggugugu...............................  1     1   BA1

....................Augcccgaaacuuugacu.........................................................................  1     1   BRA
....................gugcccgaaacuuugaUuc........................................................................  1     1   BRA
....................gugcccgaaacuuugacuA........................................................................  4     1   BRA
....................gugcccgaaacuuugacuc........................................................................  53    0   BRA
....................gugcccgaGacuuugacuc........................................................................  1     1   BRA
....................gugcccgaaacuuugacuU........................................................................  8     1   BRA
....................gugcccgaaacuuugacuca.......................................................................  180   0   BRA
....................gugcccgaaacuuugacucU.......................................................................  5     1   BRA
....................gugcccgaaacuuugacGca.......................................................................  1     1   BRA
....................gugcccgaaacuuugacucG.......................................................................  3     1   BRA
....................gugcccgGaacuuugacuca.......................................................................  1     1   BRA
....................gugcccgaGacuuugacuca.......................................................................  1     1   BRA
....................gugcccgaaacuuugacCca.......................................................................  2     1   BRA
....................gugcccgaaacuuugacuUa.......................................................................  1     1   BRA
....................gCgcccgaaacuuugacuca.......................................................................  1     1   BRA
....................Uugcccgaaacuuugacuca.......................................................................  1     1   BRA
....................gugcccgaaGcuuugacuca.......................................................................  1     1   BRA
....................gugcccgaaacuCugacucaa......................................................................  1     1   BRA
....................gugcccgaaacuuugacucaa......................................................................  152   0   BRA
....................gugcccgaaacuuugacucGa......................................................................  1     1   BRA
....................gugcccgaaacuuugacCcaa......................................................................  1     1   BRA
....................gugcccgaaacuuugacucaU......................................................................  3     1   BRA
....................gugcccgaaacuuugaUucaa......................................................................  1     1   BRA
....................gugcccgaaacuuugUcucaa......................................................................  1     1   BRA
....................gugcccgaaacuuugacCcaac.....................................................................  1     1   BRA
....................gugcccgaaacuuugacucaaU.....................................................................  17    1   BRA
....................gugcccgaaacuuugacucaGc.....................................................................  4     1   BRA
....................gugcccgaaacuuugaAucaac.....................................................................  2     1   BRA
....................gugcccgGaacuuugacucaac.....................................................................  4     1   BRA
....................gugcccgaaacuuugacuUaac.....................................................................  1     1   BRA
....................gugcccgaaacuuugacucaaA.....................................................................  1     1   BRA
....................gugcccgaaacuuugacucaac.....................................................................  130   0   BRA
....................gugcccgaaacuuugacucaacA....................................................................  22    1   BRA
```

```
                    Star                                  Mature
ugagagaaugucggccuugugugcccgaaacuuugacucaaccugaguuuaggaaaaggugagcaaaguuucaggugugucggguugacugaaaaaacugguuuaccagga
....................gugcccgaaacuCugacucaacc....................................................................   1      1   BRA
....................gugcccgaaacuuugacucaacc....................................................................   237    0   BRA
....................gugcccgaaacuuugUcucaacc....................................................................   5      1   BRA
....................gugcccgaaacuuCgacucaacc....................................................................   1      1   BRA
....................gugcccgaaacuuugacucaGcc....................................................................   1      1   BRA
....................gugccAgaaacuuugacucaacc....................................................................   1      1   BRA
....................gugcccgaaacuuugacucaacU....................................................................   67     1   BRA
....................gugcccgaaacuuugacucaaUc....................................................................   2      1   BRA
....................gugcccgaaacuuugacucaaccu...................................................................   1696   0   BRA
....................gugcUcgaaacuuugacucaaccu...................................................................   7      1   BRA
....................gugcccgaaacuuugacucGaccu...................................................................   13     1   BRA
....................gugcccgaaacuuugacCcaaccu...................................................................   6      1   BRA
....................gugccUgaaacuuugacucaaccu...................................................................   12     1   BRA
....................gCgcccgaaacuuugacucaaccu...................................................................   2      1   BRA
....................gugcccgaaacuuugacucaacAu...................................................................   2      1   BRA
....................Uugcccgaaacuuugacucaaccu...................................................................   1      1   BRA
....................gugcccgaaacuuCgacucaaccu...................................................................   13     1   BRA
....................gugcccgaaacuuugacuUaaccu...................................................................   1      1   BRA
....................gugcccgaaacuuugCcucaaccu...................................................................   3      1   BRA
....................Augcccgaaacuuugacucaaccu...................................................................   5      1   BRA
....................gugcccAaaacuuugacucaaccu...................................................................   5      1   BRA
....................gugcccgaaaUuuugacucaaccu...................................................................   2      1   BRA
....................gugccAgaaacuuugacucaaccu...................................................................   1      1   BRA
....................gugcccgaaacuuugacucaacUu...................................................................   1      1   BRA
....................gugcccgaaacAuugacucaaccu...................................................................   3      1   BRA
....................gugcccgaaacuuugacucaaccC...................................................................   163    1   BRA
....................gugcccgaaacuuugacucaGccu...................................................................   4      1   BRA
....................gAgcccgaaacuuugacucaaccu...................................................................   4      1   BRA
....................gugcccgaaacuuugacucaaccA...................................................................   130    1   BRA
....................gugcccgGaacuuugacucaaccu...................................................................   3      1   BRA
....................gugcccgaaacuuugUcucaaccu...................................................................   49     1   BRA
....................guAcccgaaacuuugacucaaccu...................................................................   1      1   BRA
....................gugcccgaaGcuuugacucaaccu...................................................................   6      1   BRA
....................gugcccgaaacuuugacucaaccG...................................................................   43     1   BRA
....................gugcccgaGacuuugacucaaccu...................................................................   6      1   BRA
....................gugccGgaaacuuugacucaaccu...................................................................   1      1   BRA
....................gugcccgaaacuuugacAcaaccu...................................................................   4      1   BRA
....................gugcccgaaacuCugacucaaccu...................................................................   5      1   BRA
....................gugcccgaaacuuugacucUaccu...................................................................   2      1   BRA
....................gugcccgaaacuuugacucaaccuU..................................................................   30     1   BRA
....................gugcccgaaacuuugacucaaccug..................................................................   2      0   BRA
....................gugcccgaaacuuugacucaaccuA..................................................................   6      1   BRA
....................gugcccgaaacuuugacucaaccuC..................................................................   2      1   BRA
.....................ugcccgaaacuuugacuca.......................................................................   1      0   BRA
.....................ugcccgaaacuuugacucaa......................................................................   3      0   BRA
.....................ugcccgaaacuuugacucaac.....................................................................   1      0   BRA
.....................ugcccgaaacuuugacucaacU....................................................................   2      1   BRA
.....................ugcccgaaacuuugacucaacc....................................................................   6      0   BRA
.....................ugcccgaaacuuugacucaaccC...................................................................   16     1   BRA
.....................ugcccgaaacuAugacucaaccu...................................................................   2      1   BRA
.....................ugcccgaaacuuugacucaaccG...................................................................   3      1   BRA
.....................ugcccgaaacuuugacucaaccA...................................................................   7      1   BRA
.....................ugcccgaaacuuCgacucaaccu...................................................................   1      1   BRA
.....................ugUccgaaacuuugacucaaccu...................................................................   1      1   BRA
.....................ugcccgaaacuuugacucaaccu...................................................................   105    0   BRA
.....................ugcccgaaacuuugacucaacUu...................................................................   1      1   BRA
.....................ugcccgaaacuuugacucaaccuC..................................................................   1      1   BRA
.....................ugcccgaaacuuugacucaaccuU..................................................................   20     1   BRA
......................gcccgaaacuuugacuca.......................................................................   1      0   BRA
......................gcccgaaacuuugacucaaccu...................................................................   2      0   BRA
......................gcccgaaacuuugacucaaccC...................................................................   1      1   BRA
..........................gaaacuuugacucaaccu...................................................................   1      0   BRA
.........................................................Cgugagcaaaguuucaggugu.................................   1      1   BRA
.........................................................Cgugagcaaaguuucaggugugu...............................   18     1   BRA
.........................................................ggugagcaaaguuucaggugugu...............................   3      0   BRA
..........................................................gugagcaaaguuucaggA...................................   1      1   BRA
..........................................................gugagcaaaguuucaggu...................................   24     0   BRA
..........................................................gugagcaaaguuucGggu...................................   1      1   BRA
..........................................................gugagcaaaguuucaggC...................................   7      1   BRA
..........................................................gugagcaaaguuucagguA..................................   10     1   BRA
```

```
                    Star                                  Mature
ugagagaaugucggccuugugugcccgaaacuuugacucaaccugaguuuaggaaaaggugagcaaaguuucaggugugucgguugacugaaaaaacugguuuaccagga
..........................................................gugagcaaGguuucaggug.................................    1      1      BRA
..........................................................gugagcaaaguuucaggug.................................    97     0      BRA
..........................................................gugaAcaaaguuucaggug.................................    1      1      BRA
..........................................................gugGgcaaaguuucaggug.................................    1      1      BRA
..........................................................gugagcaaaguuucagguU.................................    1      1      BRA
..........................................................gugagcaaaguCucaggugu................................    1      1      BRA
..........................................................gugagcaaaguuucaggugG................................    3      1      BRA
..........................................................gugagcaaaguuucUggugu................................    1      1      BRA
..........................................................gugagcaaaguuucGggugu................................    1      1      BRA
..........................................................gugagcaaaguuucaggugC................................    20     1      BRA
..........................................................gugagcaaaguuuUaggugu................................    1      1      BRA
..........................................................gugagcaaaguuucaggugu................................    261    0      BRA
..........................................................gugagcaaaguuucagguAu................................    1      1      BRA
..........................................................gugagcaaGguuucaggugu................................    1      1      BRA
..........................................................gugagcaaCguuucaggugug...............................    1      1      BRA
..........................................................gugaUcaaaguuucaggugug...............................    1      1      BRA
..........................................................gugagcaaagAuucaggugug...............................    1      1      BRA
..........................................................gugagcaaaguuucagguguU...............................    39     1      BRA
..........................................................gugagcCaaguuucaggugug...............................    2      1      BRA
..........................................................gAgagcaaaguuucaggugug...............................    4      1      BRA
..........................................................gugagcaaaguuucaggugAg...............................    3      1      BRA
..........................................................gugagcaaaguCucaggugug...............................    1      1      BRA
..........................................................gugagcaaaguuucagAugug...............................    1      1      BRA
..........................................................gugagcaaaguuucGggugug...............................    15     1      BRA
..........................................................gugagcaaaguuucaggugug...............................    1847   0      BRA
..........................................................gugagcaaGguuucaggugug...............................    6      1      BRA
..........................................................guAagcaaaguuucaggugug...............................    1      1      BRA
..........................................................gugagcGaaguuucaggugug...............................    13     1      BRA
..........................................................gugaAcaaaguuucaggugug...............................    1      1      BRA
..........................................................gugagcaaagCuucaggugug...............................    4      1      BRA
..........................................................gugagcUaaguuucaggugug...............................    1      1      BRA
..........................................................gugagcaaaguuucagguguC...............................    6      1      BRA
..........................................................gugagcaUaguuucaggugug...............................    1      1      BRA
..........................................................gugagcaGaguuucaggugug...............................    11     1      BRA
..........................................................gugagcaaUguuucaggugug...............................    2      1      BRA
..........................................................gugGgcaaaguuucaggugug...............................    9      1      BRA
..........................................................gugagcaaaguuuAaggugug...............................    1      1      BRA
..........................................................gugagcaaaguuCcaggugug...............................    4      1      BRA
..........................................................gugagcaaagGuucaggugug...............................    1      1      BRA
..........................................................Uugagcaaaguuucaggugug...............................    1      1      BRA
..........................................................gugagcaaaguuucaggugCg...............................    24     1      BRA
..........................................................gCgagcaaaguuucaggugug...............................    4      1      BRA
..........................................................gugagcaaaguuucaAgugug...............................    2      1      BRA
..........................................................gugagcaaaguuAcaggugug...............................    1      1      BRA
..........................................................gugagcaaaguuucagguguA...............................    154    1      BRA
..........................................................gugagcaaaguuucaggugGg...............................    3      1      BRA
..........................................................gugagcaaaguuucagguUug...............................    3      1      BRA
..........................................................gugagUaaaguuucaggugug...............................    1      1      BRA
..........................................................gugagcaaaguuucaUgugug...............................    8      1      BRA
..........................................................Augagcaaaguuucaggugug...............................    2      1      BRA
..........................................................gugagcaaaguuuUaggugug...............................    3      1      BRA
..........................................................gugagcaaaguuucaggCgug...............................    3      1      BRA
..........................................................gugagcaaaguuucagguAugu..............................    3      1      BRA
..........................................................gugagcaaaguuucaggAgugu..............................    22     1      BRA
..........................................................gugagcaaaguuucagguUugu..............................    1      1      BRA
..........................................................gGgagcaaaguuucaggugugu..............................    3      1      BRA
..........................................................gugagcaaaguuucaggugugA.............................    503    1      BRA
..........................................................gugCgcaaaguuucaggugugu..............................    1      1      BRA
..........................................................gugagcaaaguuucaggugugG.............................    273    1      BRA
..........................................................gugagcaaaguuucCggugugu..............................    3      1      BRA
..........................................................gugagcaaaguuuAaggugugu..............................    5      1      BRA
..........................................................gugagcaaaguuucagguguUu.............................    11     1      BRA
..........................................................gCgagcaaaguuucaggugugu..............................    21     1      BRA
..........................................................gugagcaaaUuuucaggugugu..............................    1      1      BRA
..........................................................Uugagcaaaguuucaggugugu..............................    4      1      BRA
..........................................................gugagcaaaguuucGgugugu..............................    62     1      BRA
..........................................................Augagcaaaguuucaggugugu..............................    17     1      BRA
..........................................................gugagcaaGguuucaggugugu..............................    53     1      BRA
..........................................................gugagcaaaguuucaAgugugu..............................    11     1      BRA
..........................................................gugagcaGaguuucaggugugu..............................    40     1      BRA
```

Star Mature

ugagagaaugucggccuugugugcccgaaacuuugacucaaccugaguuuaggaaaaggugagcaaaguuucaggugugucggguugacugaaaaaacugguuuaccagga			
....guUagcaaaguuucaggugugu...............................	2	1	BRA
....gugagcaaaguAucaggugugu...............................	3	1	BRA
....gugagcaaaguuucaggugAgu...............................	10	1	BRA
....gugagcaaaguuucagguguAu...............................	8	1	BRA
....gugagcaaCguuucaggugugu...............................	1	1	BRA
....gugagcaCaguuucaggugugu...............................	1	1	BRA
....gugagcaaagGuucaggugugu...............................	7	1	BRA
....gugagcaaaguuucaggugCgu...............................	31	1	BRA
....gugagcaaaguuucagAugugu...............................	11	1	BRA
....gugagcaaaAuuucaggugugu...............................	3	1	BRA
....gugagcaaaguuucaggCgugu...............................	56	1	BRA
....gugagcaaaguuAcaggugugu...............................	3	1	BRA
....gugagcaaaguuuGaggugugu...............................	2	1	BRA
....gugagcUaaguuucaggugugu...............................	4	1	BRA
....gugagcaaagCuucaggugugu...............................	41	1	BRA
....gugagcaaaguuuUaggugugu...............................	9	1	BRA
....gugagcaaaguCucaggugugu...............................	46	1	BRA
....guAagcaaaguuucaggugugu...............................	7	1	BRA
....gugaUcaaaguuucaggugugu...............................	1	1	BRA
....gugagcaaaguuCcaggugugu...............................	39	1	BRA
....gugagGaaaguuucaggugugu...............................	3	1	BRA
....gugagcaaaguuucaggugGgu...............................	9	1	BRA
....gugagcaaaguuucagCugugu...............................	1	1	BRA
....gugagcGaaguuucaggugugu...............................	48	1	BRA
....gugagcaaaguuucaggGgugu...............................	8	1	BRA
....gugagcaaaguuucaggugugu...............................	12802	0	BRA
....gugagcaaagAuucaggugugu...............................	4	1	BRA
....gugaCcaaaguuucaggugugu...............................	1	1	BRA
....gugagcaUaguuucaggugugu...............................	6	1	BRA
....gAgagcaaaguuucaggugugu...............................	9	1	BRA
....gugGgcaaaguuucaggugugu...............................	50	1	BRA
....gugagUaaaguuucaggugugu...............................	7	1	BRA
....gugaAcaaaguuucaggugugu...............................	5	1	BRA
....gugagcaaaguuucaUgugugu...............................	6	1	BRA
....guCagcaaaguuucaggugugu...............................	2	1	BRA
....gugagcaaaguuucUggugugu...............................	14	1	BRA
....gugUgcaaaguuucaggugugu...............................	4	1	BRA
....gugagAaaaguuucaggugugu...............................	3	1	BRA
....gugagcaaUguuucaggugugu...............................	7	1	BRA
....gugagcaaaguGucaggugugu...............................	2	1	BRA
....gugagcaaaguuucaggugugugG..............................	58	1	BRA
....gugagcaaaguuucaggugAguc..............................	1	1	BRA
....gugagcaaGguuucaggugugc..............................	1	1	BRA
....gugagcaaaguuuUaggugugc..............................	1	1	BRA
....gugGgcaaaguuucaggugugc..............................	1	1	BRA
....gugagcaaaguuucaggugugc..............................	43	0	BRA
....ugagcaaaguuucagguA..................................	1	1	BRA
....ugagcaaaguuucaggugugu...............................	1	0	BRA
....gagcaaaguuucaggugugu...............................	3	0	BRA
....Ccaaaguuucaggugugu...............................	1	1	BRA

Provisional ID : Scaffold_11093_2892
Score total : 1737210
Score for star read(s) : 3.9
Score for read counts : 1737200.2
Score for mfe : 1.3
Score for randfold : 1.6
Score for cons. seed : 3
Total read count : 3407455
Mature read count : 3401503
Loop read count : 0
Star read count : 5952

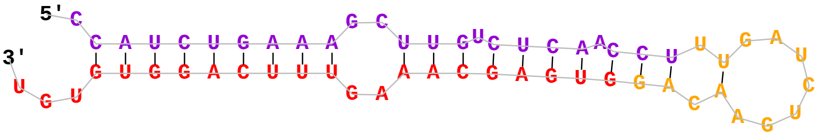

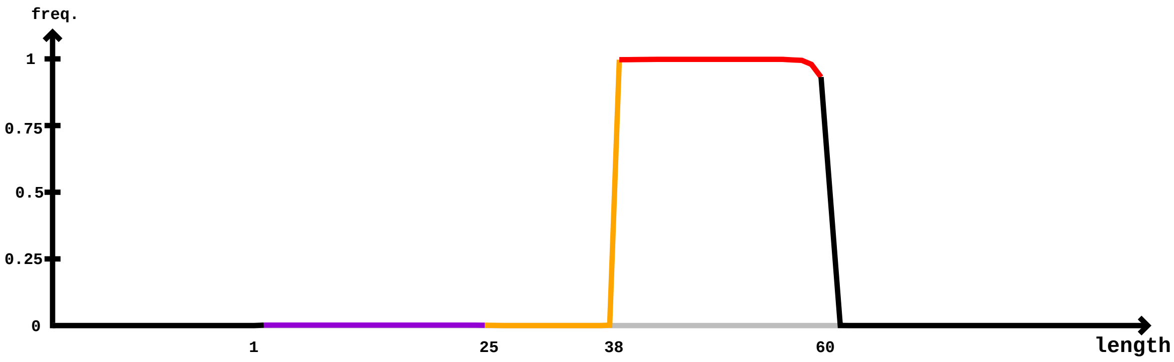

Star Mature

```
5'- ugauaauuugcuucauugauccaucugaaagcuugucucaaccuuugaucugaacaggugagcaaaguuucaggugugucagagaaguuuauuuucaucuuauuaucaacua  -3'   obs
    ugauaauuugcuucauugauccaucugaaagcuugucucaaccuuugaucugaacaggugagcaaaguuucaggugugucagagaaguuuauuuucaucuuauuaucaacua         exp
    (((.(((..(((((.((((..(((((((((..(((.((((.(((.(.......).))))))))))..)))))))))..)))).)))))..))).)))..............    reads   mm   sample
    ....................ccaucugaaagcuugucuc.........................................................................    5       0    BRA
    ....................ccaucugaaagcuugucuU.........................................................................    2       1    BRA
    ....................ccaucugaaagcuugucuca........................................................................    2       0    BRA
    ....................ccaucugaaagcuugucucaa.......................................................................    3       0    BRA
    ....................ccaucugaaagcuugucucaaA......................................................................    1       1    BRA
    ....................cUaucugaaagcuugucucaac......................................................................    1       1    BRA
    ....................ccaucugGaagcuugucucaac......................................................................    1       1    BRA
    ....................ccaucugaaagcuugucucaaU......................................................................    5       1    BRA
    ....................ccaucugaaagcuugucucaac......................................................................    40      0    BRA
    ....................ccaucugaaagcuugucucaacc.....................................................................    48      0    BRA
    ....................ccaucugaaagcuugucucaacU.....................................................................    20      1    BRA
    ....................ccaucugGaagcuugucucaacc.....................................................................    1       1    BRA
    ....................ccaucugaaagcuugucucaacA.....................................................................    3       1    BRA
    ....................ccauUugaaagcuugucucaacc.....................................................................    2       1    BRA
    ....................ccaucugaaagcuugucucaacAu....................................................................    2       1    BRA
    ....................ccaucugaGagcuugucucaaccu....................................................................    1       1    BRA
    ....................ccaucugaaagcuugucucaaccC....................................................................    5       1    BRA
    ....................ccaucugaaagcuugucucaaccG....................................................................    1       1    BRA
    ....................ccaucugaaagcuugucucaaccA....................................................................    8       1    BRA
    ....................ccaucugaaagcuugucucaaccu....................................................................    109     0    BRA
    ....................ccGucugaaagcuugucucaaccu....................................................................    2       1    BRA
    ....................ccaucugaaagcuugucucaaccuu...................................................................    2       0    BRA
    ....................ccaucugaaagcuugucucaaccuuu..................................................................    5       0    BRA
    ........................................................Cgugagcaaaguuucaggugu...................................    1       1    BRA
    ........................................................Cgugagcaaaguuucaggugugu.................................    18      1    BRA
    ........................................................ggugagcaaaguuucaggugugu.................................    3       0    BRA
    .........................................................gugagcaaaguuucaggu.....................................    24      0    BRA
    .........................................................gugagcaaaguuucGggu.....................................    1       1    BRA
    .........................................................gugagcaaaguuucaggC.....................................    7       1    BRA
    .........................................................gugagcaaaguuucaggA.....................................    1       1    BRA
    .........................................................gugagcaaaguuucaggug....................................    97      0    BRA
    .........................................................gugagcaaaguuucagguA....................................    10      1    BRA
    .........................................................gugagcaaaguuucagguU....................................    1       1    BRA
```

Star Mature

```
ugauaauuugcuucauugauccaucugaaagcuugucucaaccuuugaucugaacaggugagcaaaguuucaggugugucagagaaguuuauuuucaucuuauuaucaacua
.........................................................gugagcaaGguuucaggug....................................	1	1	BRA
.........................................................gugGgcaaaguuucaggug....................................	1	1	BRA
.........................................................gugaAcaaaguuucaggug....................................	1	1	BRA
.........................................................gugagcaaaguuucGggugu...................................	1	1	BRA
.........................................................gugagcaaaguuucUggugu...................................	1	1	BRA
.........................................................gugagcaaaguuucaggugu...................................	261	0	BRA
.........................................................gugagcaaGguuucaggugu...................................	1	1	BRA
.........................................................gugagcaaaguCucaggugu...................................	1	1	BRA
.........................................................gugagcaaaguuucaggugG...................................	3	1	BRA
.........................................................gugagcaaaguuuUaggugu...................................	1	1	BRA
.........................................................gugagcaaaguuucagguAu...................................	1	1	BRA
.........................................................gugagcaaaguuucaggugC...................................	20	1	BRA
.........................................................gugagcaaaguuucaUgugug..................................	8	1	BRA
.........................................................gAgagcaaaguuucaggugug..................................	4	1	BRA
.........................................................gugagcaaagCuucaggugug..................................	4	1	BRA
.........................................................gugagcaaaguuucaggugGg..................................	3	1	BRA
.........................................................gugagcaaagAuucaggugug..................................	1	1	BRA
.........................................................gCgagcaaaguuucaggugug..................................	4	1	BRA
.........................................................gugagcCaaguuucaggugug..................................	2	1	BRA
.........................................................gugagcaaaguuucaggCgug..................................	3	1	BRA
.........................................................gugagcaaaguuucaAgugug..................................	2	1	BRA
.........................................................gugagcaaaguuucaggugug..................................	1847	0	BRA
.........................................................guAagcaaaguuucaggugug..................................	1	1	BRA
.........................................................gugagcaaGguuucaggugug..................................	6	1	BRA
.........................................................gugagcaUaguuucaggugug..................................	1	1	BRA
.........................................................gugagcaaaguuucGggugug..................................	15	1	BRA
.........................................................gugagcaGaguuucaggugug..................................	11	1	BRA
.........................................................gugagcaaaguuuAaggugug..................................	1	1	BRA
.........................................................gugaAcaaaguuucaggugug..................................	1	1	BRA
.........................................................Uugagcaaaguuucaggugug..................................	1	1	BRA
.........................................................gugagcaaaguuucagguguA..................................	154	1	BRA
.........................................................gugagcaaaguuCcaggugug..................................	4	1	BRA
.........................................................gugagcaaUguuucaggugug..................................	2	1	BRA
.........................................................gugGgcaaaguuucaggugug..................................	9	1	BRA
.........................................................gugagUaaaguuucaggugug..................................	1	1	BRA
.........................................................gugagcaaaguuuUaggugug..................................	3	1	BRA
.........................................................gugagcGaaguuucaggugug..................................	13	1	BRA
.........................................................gugagcaaaguuucagAugug..................................	1	1	BRA
.........................................................gugagcaaaguuucaggugCg..................................	24	1	BRA
.........................................................gugagcaaCguuucaggugug..................................	1	1	BRA
.........................................................gugagcaaaguCucaggugug..................................	1	1	BRA
.........................................................gugagcaaaguuucaggugAg..................................	3	1	BRA
.........................................................gugagcaaaguuucagguguC..................................	6	1	BRA
.........................................................gugagcaaaguuucagguguU..................................	39	1	BRA
.........................................................gugagcaaaguuucagguUug..................................	3	1	BRA
.........................................................gugaUcaaaguuucaggugug..................................	1	1	BRA
.........................................................gugagcaaaguuAcaggugug..................................	1	1	BRA
.........................................................gugagcaaagGuucaggugug..................................	1	1	BRA
.........................................................Augagcaaaguuucaggugug..................................	2	1	BRA
.........................................................gugagcUaaguuucaggugug..................................	1	1	BRA
.........................................................gugagcaaaguuCcaggugugu.................................	39	1	BRA
.........................................................gugagcaaaguuuUaggugugu.................................	9	1	BRA
.........................................................gugagcaaaguuucaggugGgu.................................	9	1	BRA
.........................................................gugaAcaaaguuucaggugugu.................................	5	1	BRA
.........................................................gugagcaaaguuucUggugugu.................................	14	1	BRA
.........................................................gugagcaaaguuucagguguUu.................................	11	1	BRA
.........................................................gugGgcaaaguuucaggugugu.................................	50	1	BRA
.........................................................gugagcaaaguuucagAugugu.................................	11	1	BRA
.........................................................gugagUaaaguuucaggugugu.................................	7	1	BRA
.........................................................gugagcaaUguuucaggugugu.................................	7	1	BRA
.........................................................gCgagcaaaguuucaggugugu.................................	21	1	BRA
.........................................................gugagcaaaguuucagguguAu.................................	8	1	BRA
.........................................................gugagAaaaguuucaggugugu.................................	3	1	BRA
.........................................................gGgagcaaaguuucaggugugu.................................	3	1	BRA
.........................................................gugagcaaaguGucaggugugu.................................	2	1	BRA
.........................................................gugagcaaaguuucagCugugu.................................	1	1	BRA
.........................................................guUagcaaaguuucaggugugu.................................	2	1	BRA
.........................................................gugagcaaaguuucaggugCgu.................................	31	1	BRA
.........................................................gugagcUaaguuucaggugugu.................................	4	1	BRA
.........................................................gugagcaaaguuucaUgugugu.................................	6	1	BRA
```

Star Mature

ugauaauuugcuucauugauccaucugaaagcuugucucaaccuuugaucugaacaggugagcaaaguuucaggugugucagagaaguuuauuuucaucuuauuaucaacua			
....gugagcaaaguuuGaggugugu.................................	2	1	BRA
....gugagcGaaguuucaggugugu.................................	48	1	BRA
....gugagcaaGguuucaggugugu.................................	53	1	BRA
....gugagcaaaguCucaggugugu.................................	46	1	BRA
....gugagcaaaguuucaAgugugu.................................	11	1	BRA
....gugagcaUaguuucaggugugu.................................	6	1	BRA
....Uugagcaaaguuucaggugugu.................................	4	1	BRA
....gugagcaaaguuuAaggugugu.................................	5	1	BRA
....gugaCcaaaguuucaggugugu.................................	1	1	BRA
....gugagcaaaguuucGggugugu.................................	62	1	BRA
....gugagcaaaguuucCggugugu.................................	3	1	BRA
....gugagcaCaguuucaggugugu.................................	1	1	BRA
....Augagcaaaguuucaggugugu.................................	17	1	BRA
....guAagcaaaguuucaggugugu.................................	7	1	BRA
....gugagcaaaAuuucaggugugu.................................	3	1	BRA
....gugaUcaaaguuucaggugugu.................................	1	1	BRA
....gugagcaaaguuucaggugugG.................................	273	1	BRA
....gugCgcaaaguuucaggugugu.................................	1	1	BRA
....gugagcaaaguuAcaggugugu.................................	3	1	BRA
....gugagcaaagGuucaggugugu.................................	7	1	BRA
....gugagcaaCguuucaggugugu.................................	1	1	BRA
....gugUgcaaaguuucaggugugu.................................	4	1	BRA
....gugagcaGaguuucaggugugu.................................	40	1	BRA
....gugagcaaaguuucaggugugu.................................	12802	0	BRA
....gugagcaaaguuucaggAgugu.................................	22	1	BRA
....gugagcaaaguAucaggugugu.................................	3	1	BRA
....gugagcaaaguuucaggCgugu.................................	56	1	BRA
....gugagcaaagAuucaggugugu.................................	4	1	BRA
....gugagcaaaguuucaggGgugu.................................	8	1	BRA
....gugagcaaaguuucagguAugu.................................	3	1	BRA
....gugagcaaaguuucagguUugu.................................	1	1	BRA
....gugagGaaaguuucaggugugu.................................	3	1	BRA
....gugagcaaaguuucaggugAgu.................................	10	1	BRA
....gugagcaaaguuucaggugugA.................................	503	1	BRA
....gugagcaaagCuucaggugugu.................................	41	1	BRA
....guCagcaaaguuucaggugugu.................................	2	1	BRA
....gugagcaaaUuuucaggugugu.................................	1	1	BRA
....gAgagcaaaguuucaggugugu.................................	9	1	BRA
....gugagcaaGguuucagguguguc................................	1	1	BRA
....gugagcaaaguuucaggugAguc................................	1	1	BRA
....gugagcaaaguuucagguguguG................................	58	1	BRA
....gugagcaaaguuucagguguguc................................	43	0	BRA
....gugagcaaaguuuUagguguguc................................	1	1	BRA
....gugGgcaaaguuucagguguguc................................	1	1	BRA
....gugagcaaaguuucagguguguUa...............................	3	1	BRA
....ugagcaaaguuucagguA....................................	1	1	BRA
....ugagcaaaguuucaggugugu.................................	1	0	BRA
....gagcaaaguuucaggugugu.................................	3	0	BRA
....Ccaaaguuucaggugugu.................................	1	1	BRA
....................ccaucugaaagcuugucuca..	2	0	BA1
....................ccaucugaaagcuugGcucaac..	1	1	BA1
....................ccaucugaaagcuugucucaaU..	1	1	BA1
....................ccaucugaaagcuugucucaac..	13	0	BA1
....................ccaucugaaGgcuugucucaacc...	1	1	BA1
....................ccGucugaaagcuugucucaacc...	1	1	BA1
....................ccaucCgaaagcuugucucaacc...	1	1	BA1
....................ccaucugaaagcuugucucaacc...	31	0	BA1
....................ccaucugaaagcuugucucaacU...	8	1	BA1
....................ccaucugaaagcuugucucaacA...	1	1	BA1
....................ccaucCgaaagcuugucucaaccu..	1	1	BA1
....................ccaucugaaGgcuugucucaaccu..	1	1	BA1
....................ccaucugaaagcuCgucucaaccu..	1	1	BA1
....................ccaucugaaagcuugucucGaccu..	1	1	BA1
....................ccaucugaaagcuugucucaaccA..	10	1	BA1
....................ccaucugaaagcuugucucaaccC..	15	1	BA1
....................ccaucugaaagcuugucucaaccu..	102	0	BA1
....................ccaucugaaagcuugGcucaaccu..	1	1	BA1
....................ccaucugaGagcuugucucaaccu..	1	1	BA1
....................ccaucugaaagcuugucucaaccuu...	2	0	BA1

```
                        Star                                   Mature
ugauaauuugcuucauugauccaucugaaagcuugucucaaccuuugaucugaacaggugagcaaaguuucaggugugucagagaaguuuauuuucaucuuauuaucaacua
....................ccaucugaaagcuugucucaaccuA...................................................................	3	1	BA1
....................ccaucugaaagcuugucucaaccuuu..................................................................	4	0	BA1
....................ccaCcugaaagcuugucucaaccuuu..................................................................	2	1	BA1
.....................caucugaaagcuugucucaaccu....................................................................	1	0	BA1
........................................................Cgugagcaaaguuucaggugugu.................................	12	1	BA1
........................................................Ugugagcaaaguuucaggugugu.................................	1	1	BA1
........................................................Agugagcaaaguuucaggugugu.................................	1	1	BA1
.........................................................gugagcaaaguuucaggC.....................................	2	1	BA1
.........................................................gugagcaaaguuucaggu.....................................	4	0	BA1
.........................................................gugagUaaaguuucaggug....................................	1	1	BA1
.........................................................gugagcaaaguuucagguA....................................	6	1	BA1
.........................................................gugagcaaaguuucaggug....................................	31	0	BA1
.........................................................gugagcaaaguuucUggugu...................................	2	1	BA1
.........................................................gAgagcaaaguuucaggugu...................................	1	1	BA1
.........................................................gugagcaaaguuucaggugC...................................	11	1	BA1
.........................................................gugagcaaaguuucaggugu...................................	76	0	BA1
.........................................................gugagcaaaguuucaggugug..................................	274	0	BA1
.........................................................gugagcaaaguuucagguguC..................................	1	1	BA1
.........................................................gugagcaaaguuucagguguU..................................	17	1	BA1
.........................................................gugagcaaGguuucaggugug..................................	5	1	BA1
.........................................................gugagcaaUguuucaggugug..................................	1	1	BA1
.........................................................gugagcaaaguuCcaggugug..................................	1	1	BA1
.........................................................gugagcaaaguuucagguguA..................................	31	1	BA1
.........................................................gugagcaaaguuucaggCgug..................................	1	1	BA1
.........................................................gugagcGaaguuucaggugug..................................	1	1	BA1
.........................................................gugaCcaaaguuucaggugugu.................................	1	1	BA1
.........................................................gAgagcaaaguuucaggugugu.................................	3	1	BA1
.........................................................gugagcaaaguuucaggugGgu.................................	5	1	BA1
.........................................................gugagcaaaguuucagguAugu.................................	1	1	BA1
.........................................................gugagUaaaguuucaggugugu.................................	5	1	BA1
.........................................................gugGgcaaaguuucaggugugu.................................	26	1	BA1
.........................................................gugagcaaaguuucagAugugu.................................	2	1	BA1
.........................................................gugagcaaaguuucagguUugu.................................	1	1	BA1
.........................................................gugagcaaaguuucaggugugA.................................	299	1	BA1
.........................................................Augagcaaaguuucaggugugu.................................	7	1	BA1
.........................................................gugagcaaaguuucaggugCgu.................................	9	1	BA1
.........................................................gugagcaaaguuucaAgugugu.................................	3	1	BA1
.........................................................gugagcaaaguuucGggugugu.................................	18	1	BA1
.........................................................gugagcaaaguuucaggCgugu.................................	16	1	BA1
.........................................................gugagcaaaguuucaggugugu.................................	4211	0	BA1
.........................................................gugagcaaaguuucagguguAu.................................	3	1	BA1
.........................................................guUagcaaaguuucaggugugu.................................	1	1	BA1
.........................................................gugUgcaaaguuucaggugugu.................................	7	1	BA1
.........................................................guAagcaaaguuucaggugugu.................................	2	1	BA1
.........................................................gugagcaaagCuucaggugugu.................................	12	1	BA1
.........................................................gugagcaCaguuucaggugugu.................................	2	1	BA1
.........................................................gugagcaaaguuucaUgugugu.................................	2	1	BA1
.........................................................gCgagcaaaguuucaggugugu.................................	11	1	BA1
.........................................................gugagcaUaguuucaggugugu.................................	1	1	BA1
.........................................................gugagcUaaguuucaggugugu.................................	1	1	BA1
.........................................................gugaUcaaaguuucaggugugu.................................	2	1	BA1
.........................................................gugagcaaagGuucaggugugu.................................	4	1	BA1
.........................................................gugagcaGaguuucaggugugu.................................	14	1	BA1
.........................................................gugagcaaGguuucaggugugu.................................	16	1	BA1
.........................................................gugagcaaCguuucaggugugu.................................	1	1	BA1
.........................................................gugagcaaaguuucUggugugu.................................	4	1	BA1
.........................................................gugagcaaaguuCcaggugugu.................................	16	1	BA1
.........................................................gugagcaaaAuuucaggugugu.................................	1	1	BA1
.........................................................gugagcaaaguuucaggugugG.................................	103	1	BA1
.........................................................gugaAcaaaguuucaggugugu.................................	2	1	BA1
.........................................................gugagcGaaguuucaggugugu.................................	12	1	BA1
.........................................................gugagcaaUguuucaggugugu.................................	1	1	BA1
.........................................................gugagcaaaguuucagguguUu.................................	13	1	BA1
.........................................................gugagcaaagAuucaggugugu.................................	1	1	BA1
.........................................................gugagcaaaguuucaggGgugu.................................	2	1	BA1
.........................................................gugagcaaaguuAcaggugugu.................................	3	1	BA1
.........................................................gugagcaaaguuUaggugugu..................................	4	1	BA1
.........................................................gugagcaaaguCucaggugugu.................................	17	1	BA1
.........................................................gugagcaaaguuucaggAgugu.................................	2	1	BA1
.........................................................gugagcaaaguuucaCgugugu.................................	3	1	BA1
```

```
                    Star                                 Mature
ugauaauuugcuucauugauccaucugaaagcuugucucaaccuuugaucugaacaggugagcaaaguuucaggugugucagagaaguuuauuuucaucuuauuaucaacua
.........................................................gugagcaaaguuucagguguguG................................   10   1   BA1
.........................................................gugagcaaGguuucagguguguc................................   1    1   BA1
.........................................................gugagcaaaguuucagguguguc................................   15   0   BA1
..........................................................ugagcaaaguuucaggugugu.................................   3    0   BA1
..........................................................ugagcaaaguuucaggugugA.................................   1    1   BA1
...........................................................gagcaaaguuucaggugugu.................................   2    0   BA1
...........................................................gagcaaGguuucaggugugu.................................   1    1   BA1

...................uccaCcugaaagcuugucucaaccu....................................................................   1    1   3rd
....................ccaucugaaagcuugucu..........................................................................   1    0   3rd
....................ccaucugaaagcuugucuc.........................................................................   5    0   3rd
....................ccaucugaaagcuugucuca........................................................................   12   0   3rd
....................ccaucugaaagcuugucucaa.......................................................................   6    0   3rd
....................ccaucugaaagcuugucucaac......................................................................   22   0   3rd
....................ccaucugaaagUuugucucaacc.....................................................................   1    1   3rd
....................ccaucugaaagcuugucucaacA.....................................................................   2    1   3rd
....................ccaucugaaagcuugucucaacc.....................................................................   72   0   3rd
....................ccaucugaaagcuugucucaCcc.....................................................................   1    1   3rd
....................ccaucugaaagcuugucucUaccu....................................................................   1    1   3rd
....................ccaucugaaaUcuugucucaaccu....................................................................   2    1   3rd
....................ccaucugaaagcuCgucucaaccu....................................................................   1    1   3rd
....................ccaucugaaagcuugucCcaaccu....................................................................   1    1   3rd
....................ccaucugaaagcuugucucaaccu....................................................................   318  0   3rd
....................ccaucugaGagcuugucucaaccu....................................................................   1    1   3rd
....................ccaucugaaCgcuugucucaaccu....................................................................   1    1   3rd
....................ccaucugCaagcuugucucaaccu....................................................................   1    1   3rd
....................ccaucugaaagcuugucucaaccA....................................................................   4    1   3rd
....................ccaucugUaagcuugucucaaccu....................................................................   1    1   3rd
....................ccaucugaaagcuugucuUaaccu....................................................................   1    1   3rd
....................Ucaucugaaagcuugucucaaccu....................................................................   1    1   3rd
....................ccaucugGaagcuugucucaaccu....................................................................   1    1   3rd
....................ccaucugaaagcuugucucaaccuu...................................................................   2    0   3rd
....................ccaCcugaaagcuugucucaaccuuu..................................................................   11   1   3rd
.....................caCcugaaagcuugucucaaccuuu..................................................................   5    1   3rd
........................cugaaagcuugucucaacc.....................................................................   1    0   3rd
.........................ugaaagcuugucucaacc.....................................................................   1    0   3rd
.........................ugaaagcuugucucaaccu....................................................................   1    0   3rd
..........................gaaagcuugucucaaccu....................................................................   1    0   3rd
.....................................................acGgugagcaaaguuucaggugugu.................................   1    1   3rd
......................................................cGgugagcaaaguuucaggugugu.................................   1    1   3rd
.......................................................aggugagcaaaguuucaggugu...................................   2    0   3rd
.......................................................aggugagcaaaguuucaggugug..................................   1    0   3rd
.......................................................Gggugagcaaaguuucaggugug..................................   1    1   3rd
.......................................................Gggugagcaaaguuucaggugugu.................................   2    1   3rd
.......................................................Uggugagcaaaguuucaggugugu.................................   1    1   3rd
.......................................................aggugagcaaaguuucagguguUu.................................   1    1   3rd
.......................................................aUgugagcaaaguuucaggugugu.................................   1    1   3rd
.......................................................aCgugagcaaaguuucaggugugu.................................   1    1   3rd
........................................................ggugagcaaaguuucagg......................................   1    0   3rd
........................................................ggugagcaaaguuucaggug....................................   5    0   3rd
........................................................ggugagcaaaguuucCggug....................................   1    1   3rd
........................................................Ugugagcaaaguuucaggugu...................................   10   1   3rd
........................................................ggugagcaaaguuucaggugu...................................   59   0   3rd
........................................................ggugagcaaaguuucGggugu...................................   1    1   3rd
........................................................ggugagcaaaguuucagguguU..................................   3    1   3rd
........................................................Agugagcaaaguuucaggugug..................................   1    1   3rd
........................................................ggugagcaaagAuucaggugug..................................   1    1   3rd
........................................................gguCagcaaaguuucaggugug..................................   1    1   3rd
........................................................Ugugagcaaaguuucaggugug..................................   30   1   3rd
........................................................Cgugagcaaaguuucaggugug..................................   1    1   3rd
........................................................ggGgagcaaaguuucaggugug..................................   1    1   3rd
........................................................ggugagcaaaguuucaggugug..................................   168  0   3rd
........................................................gCugagcaaaguuucaggugugu.................................   1    1   3rd
........................................................Cgugagcaaaguuucaggugugu.................................   4    1   3rd
........................................................ggugCgcaaaguuucaggugugu.................................   1    1   3rd
........................................................ggugaUcaaaguuucaggugugu.................................   1    1   3rd
........................................................ggugagcaaaguuucaggugugA.................................   17   1   3rd
........................................................ggugGgcaaaguuucaggugugu.................................   2    1   3rd
........................................................ggugagcaaaguuucagCugugu.................................   2    1   3rd
........................................................ggugagcaaaguuucaggugugu.................................   292  0   3rd
```

Star Mature

ugauaauuugcuucauugauccaucugaaagcuugucucaaccuuugaucugaacaggugagcaaaguuucaggugugucagagaaguuuauuuucaucuuauuaucaacua

....Agugagcaaaguuucaggugugu.................................	9	1	3rd
....ggAgagcaaaguuucaggugugu.................................	1	1	3rd
....ggGgagcaaaguuucaggugugu.................................	12	1	3rd
....gAugagcaaaguuucaggugugu.................................	1	1	3rd
....Ugugagcaaaguuucaggugugu.................................	386	1	3rd
....ggugagcaaaguuucagguguUu.................................	12	1	3rd
....ggugagUaaaguuucaggugugu.................................	1	1	3rd
....gugagcaaaguuucGggu.....................................	1	1	3rd
....gugagcaaaguuucCggu.....................................	3	1	3rd
....gugagcaaaguuucaggu.....................................	729	0	3rd
....gugagAaaaguuucaggu.....................................	1	1	3rd
....Cugagcaaaguuucaggu.....................................	1	1	3rd
....gugagcaaaguuucaggA.....................................	3	1	3rd
....gugagcaaaguuucaggC.....................................	3	1	3rd
....guCagcaaaguuucaggu.....................................	1	1	3rd
....gugagAaaaguuucaggug....................................	3	1	3rd
....gugagcaaagAuucaggug....................................	1	1	3rd
....gugagcaaaguuucagguC....................................	9	1	3rd
....gugagcaaaguuucagguA....................................	9	1	3rd
....gAgagcaaaguuucaggug....................................	1	1	3rd
....gugagcaaaguuucaggGg....................................	1	1	3rd
....gugagcUaaguuucaggug....................................	1	1	3rd
....gugagcaaaguuucagguU....................................	10	1	3rd
....gugagcaaaguuucaggug....................................	979	0	3rd
....gugagcaaaguuucagAug....................................	2	1	3rd
....gugagcaaaguuuGaggug....................................	1	1	3rd
....gugagcaaaguuucaggAg....................................	1	1	3rd
....guCagcaaaguuucaggugu...................................	4	1	3rd
....guUagcaaaguuucaggugu...................................	1	1	3rd
....gugagcaaaguuucaAgugu...................................	5	1	3rd
....gAgagcaaaguuucaggugu...................................	8	1	3rd
....gugagcaaaguAucaggugu...................................	2	1	3rd
....gugagcaaagCuucaggugu...................................	3	1	3rd
....gugagcaaagAuucaggugu...................................	1	1	3rd
....gugagcCaaguuucaggugu...................................	1	1	3rd
....gugagcaaagGuucaggugu...................................	5	1	3rd
....gugagcaaaguuucaggCgu...................................	5	1	3rd
....gugaAcaaaguuucaggugu...................................	1	1	3rd
....gugagcaUaguuucaggugu...................................	3	1	3rd
....gugagcaaaguGucaggugu...................................	1	1	3rd
....gugaUcaaaguuucaggugu...................................	3	1	3rd
....gugagcaaaguCucaggugu...................................	1	1	3rd
....gugagcaaaguuucagAugu...................................	2	1	3rd
....gugGgcaaaguuucaggugu...................................	11	1	3rd
....gCgagcaaaguuucaggugu...................................	2	1	3rd
....gugagcaaaguuucaggugu...................................	11747	0	3rd
....gugagcaaaguuucGggugu...................................	5	1	3rd
....gugagcaaaguuucaggugC...................................	15	1	3rd
....gugUgcaaaguuucaggugu...................................	1	1	3rd
....gugagcaaaguuuAaggugu...................................	1	1	3rd
....gugagcaaaguuucUggugu...................................	5	1	3rd
....gugagcaaaguuucaggAgu...................................	2	1	3rd
....gugagcaaaguuucaggGgu...................................	21	1	3rd
....gugagcaCaguuucaggugu...................................	3	1	3rd
....gugCgcaaaguuucaggugu...................................	2	1	3rd
....gugagcaaaguuucaUgugu...................................	2	1	3rd
....gugagcaaaguuucaggugG...................................	9	1	3rd
....gugagGaaaguuucaggugu...................................	5	1	3rd
....gugagcaaaUuuucaggugu...................................	2	1	3rd
....gugagcaaaguuucaCgugu...................................	3	1	3rd
....gugagcaaaguuuUaggugu...................................	3	1	3rd
....gugagcaaGguuucaggugu...................................	3	1	3rd
....Uugagcaaaguuucaggugu...................................	1	1	3rd
....gugagcaaaguuuGaggugu...................................	2	1	3rd
....gGgagcaaaguuucaggugu...................................	5	1	3rd
....gugagcaaCguuucaggugu...................................	1	1	3rd
....gugagcaaaCuuucaggugu...................................	5	1	3rd
....gugagUaaaguuucaggugu...................................	8	1	3rd
....gugagcUaaguuucaggugu...................................	6	1	3rd
....gugagAaaaguuucaggugu...................................	9	1	3rd

Star Mature

```
ugauaauuugcuucauugauccaucugaaagcuugucucaaccuuugaucugaacaggugagcaaaguuucaggugugucagagaaguuuauuuucaucuuauuaucaacua
.........................................................gugagcaaaguuucagguCu...................................    2      1    3rd
.........................................................gugagcaaaguuucagCugu...................................    2      1    3rd
.........................................................gugagcaaaguuucagUugu...................................    3      1    3rd
.........................................................gugagcaaaguuucagguUug..................................    6      1    3rd
.........................................................gugagcaCaguuucaggugug..................................    5      1    3rd
.........................................................gugagcaaaguuucaggGgug..................................    32     1    3rd
.........................................................Cugagcaaaguuucaggugug..................................    2      1    3rd
.........................................................gugagcaaaguuucGggugug..................................    19     1    3rd
.........................................................gugaUcaaaguuucaggugug..................................    6      1    3rd
.........................................................gugagcaaaguuucCggugug..................................    2      1    3rd
.........................................................gugagAaaaguuucaggugug..................................    21     1    3rd
.........................................................gugagcaaaguuucaggugug..................................    35459  0    3rd
.........................................................gugagcaGaguuucaggugug..................................    3      1    3rd
.........................................................guCagcaaaguuucaggugug..................................    10     1    3rd
.........................................................gugagcaaCguuucaggugug..................................    2      1    3rd
.........................................................gugagcUaaguuucaggugug..................................    43     1    3rd
.........................................................gugagcaaaguuucaggugC..................................    12     1    3rd
.........................................................gugagcaaaguCucaggugug..................................    2      1    3rd
.........................................................gugagcaaaguuucaggugAg..................................    8      1    3rd
.........................................................gugagcaaaguuuGaggugug..................................    1      1    3rd
.........................................................gCgagcaaaguuucaggugug..................................    3      1    3rd
.........................................................gugagcaaaguuucagguCug..................................    5      1    3rd
.........................................................gugagcCaaguuucaggugug..................................    2      1    3rd
.........................................................gugUgcaaaguuucaggugug..................................    5      1    3rd
.........................................................gugagcaaaguGucaggugug..................................    5      1    3rd
.........................................................gugCgcaaaguuucaggugug..................................    7      1    3rd
.........................................................gugagcaUaguuucaggugug..................................    8      1    3rd
.........................................................gugagcaaaguuUaggugug..................................    16     1    3rd
.........................................................gugagcaaaguuucagguguA..................................    87     1    3rd
.........................................................gugagcaaUguuucaggugug..................................    2      1    3rd
.........................................................gugGgcaaaguuucaggugug..................................    24     1    3rd
.........................................................gugaCcaaaguuucaggugug..................................    3      1    3rd
.........................................................gugagcaaaguuCcaggugug..................................    4      1    3rd
.........................................................gugagcaaaguuucaggAgug..................................    3      1    3rd
.........................................................gugagcaaagAuucaggugug..................................    4      1    3rd
.........................................................gugagcaaagGuucaggugug..................................    11     1    3rd
.........................................................gugagcaaaguuuAaggugug..................................    6      1    3rd
.........................................................gugagcGaaguuucaggugug..................................    6      1    3rd
.........................................................guUagcaaaguuucaggugug..................................    1      1    3rd
.........................................................gugagcaaaguuucaUgugug..................................    4      1    3rd
.........................................................gugagcaaaguuucagCugug..................................    11     1    3rd
.........................................................gugagGaaaguuucaggugug..................................    17     1    3rd
.........................................................gugagcaaaguuucUggugug..................................    13     1    3rd
.........................................................gugagcaaaguuAcaggugug..................................    1      1    3rd
.........................................................gugagcaaaguuucaggCgug..................................    8      1    3rd
.........................................................gugagUaaaguuucaggugug..................................    27     1    3rd
.........................................................gugagcaaagCuucaggugug..................................    6      1    3rd
.........................................................gugagcaaaCuuucaggugug..................................    5      1    3rd
.........................................................gAgagcaaaguuucaggugug..................................    25     1    3rd
.........................................................gugagcaaaguuucagAugug..................................    10     1    3rd
.........................................................gugaAcaaaguuucaggugug..................................    12     1    3rd
.........................................................gugagcaaaguuGcaggugug..................................    1      1    3rd
.........................................................gugagcaaaguuucaAgugug..................................    9      1    3rd
.........................................................gugagcaaaguuucaggugCg..................................    22     1    3rd
.........................................................gugagcaaaguuucagguguU..................................    1000   1    3rd
.........................................................gugagcaaaguuucaggugGg..................................    11     1    3rd
.........................................................gugagcaaaguuucagUugug..................................    8      1    3rd
.........................................................gugagcaaaguuucaCgugug..................................    22     1    3rd
.........................................................Augagcaaaguuucaggugug..................................    4      1    3rd
.........................................................gGgagcaaaguuucaggugug..................................    6      1    3rd
.........................................................gugagcaaaguuucagguAug..................................    3      1    3rd
.........................................................gugagcaaaAuuucaggugug..................................    1      1    3rd
.........................................................guAagcaaaguuucaggugug..................................    4      1    3rd
.........................................................gugagcaaGguuucaggugug..................................    2      1    3rd
.........................................................gugagcaaaguuucaggugCgu.................................    143    1    3rd
.........................................................gugaCcaaaguuucaggugugu.................................    107    1    3rd
.........................................................gugagcaaaguuUaggugugu.................................    309    1    3rd
.........................................................gugCgcaaaguuucaggugugu.................................    118    1    3rd
.........................................................Uugagcaaaguuucaggugugu.................................    87     1    3rd
.........................................................gugagUaaaguuucaggugugu.................................    578    1    3rd
```

```
                    Star                                     Mature
ugauaauuugcuucauugauccaucugaaagcuugucucaaccuuugaucugaacaggugagcaaaguuucaggugugucagagaaguuuauuuucaucuuauuaucaacua
.........................................................gugagcaaaguuucaAgugugu.................................   158     1   3rd
.........................................................gugagcaaaguuucaggAgugu.................................   63      1   3rd
.........................................................gugagcaGaguuucaggugugu.................................   80      1   3rd
.........................................................gugagcaUaguuucaggugugu.................................   123     1   3rd
.........................................................gugagcaaaguuuAaggugugu.................................   46      1   3rd
.........................................................gugagcaaaguuucaggCgugu.................................   125     1   3rd
.........................................................gugagcaaaguuucagguguAu.................................   146     1   3rd
.........................................................gugagcaaaguuucaggGgugu.................................   296     1   3rd
.........................................................gugagcaaaguuGcaggugugu.................................   12      1   3rd
.........................................................gugagcaaaguuAcaggugugu.................................   2       1   3rd
.........................................................gugagcaaaguuucaggugugG.................................   170     1   3rd
.........................................................gugagcaaUguuucaggugugu.................................   32      1   3rd
.........................................................gugagcaaaguuucagguCugu.................................   78      1   3rd
.........................................................gugagcUaaguuucaggugugu.................................   797     1   3rd
.........................................................gugGgcaaaguuucaggugugu.................................   271     1   3rd
.........................................................gugagcaaaguCucaggugugu.................................   80      1   3rd
.........................................................Cugagcaaaguuucaggugugu.................................   48      1   3rd
.........................................................gugagcaaaguuucagAugugu.................................   171     1   3rd
.........................................................gugagcGaaguuucaggugugu.................................   81      1   3rd
.........................................................gugagGaaaguuucaggugugu.................................   268     1   3rd
.........................................................gugagcaaaguuucaggugAgu.................................   75      1   3rd
.........................................................gugagcaaaguuucagguUugu.................................   72      1   3rd
.........................................................Augagcaaaguuucaggugugu.................................   58      1   3rd
.........................................................gugaUcaaaguuucaggugugu.................................   54      1   3rd
.........................................................gugagcaaaUuuucaggugugu.................................   38      1   3rd
.........................................................gugagcaaGguuucaggugugu.................................   37      1   3rd
.........................................................gugagcaaaguuCcaggugugu.................................   73      1   3rd
.........................................................gCgagcaaaguuucaggugugu.................................   92      1   3rd
.........................................................gugagcaaaguuucaUgugugu.................................   143     1   3rd
.........................................................gugagcaaagCuucaggugugu.................................   123     1   3rd
.........................................................gugagcaaaguGucaggugugu.................................   37      1   3rd
.........................................................gugagAaaaguuucaggugugu.................................   484     1   3rd
.........................................................gugagcaaaguuucaggugugUu.................................   1758    1   3rd
.........................................................gugagcaaaguuuGaggugugu.................................   47      1   3rd
.........................................................gugagcaaaAuuucaggugugu.................................   46      1   3rd
.........................................................gugaAcaaaguuucaggugugu.................................   183     1   3rd
.........................................................gGgagcaaaguuucaggugugu.................................   238     1   3rd
.........................................................guCagcaaaguuucaggugugu.................................   140     1   3rd
.........................................................gugagcaaaguuucaggugGgu.................................   233     1   3rd
.........................................................gugagcaaaCuuucaggugugu.................................   92      1   3rd
.........................................................gugagcaaagGuucaggugugu.................................   199     1   3rd
.........................................................gugagcaaaguuucaCgugugu.................................   199     1   3rd
.........................................................gAgagcaaaguuucaggugugu.................................   490     1   3rd
.........................................................gugagcaaaguuucUggugugu.................................   268     1   3rd
.........................................................gugagcaaCguuucaggugugu.................................   8       1   3rd
.........................................................guAagcaaaguuucaggugugu.................................   117     1   3rd
.........................................................gugagcaaaguuuCggugugu.................................   24      1   3rd
.........................................................gugagcaaaguuucagCugugu.................................   198     1   3rd
.........................................................gugUgcaaaguuucaggugugu.................................   149     1   3rd
.........................................................gugagcaaaguuucaggugugu.................................   668121  0   3rd
.........................................................guUagcaaaguuucaggugugu.................................   59      1   3rd
.........................................................gugagcaaaguAucaggugugu.................................   25      1   3rd
.........................................................gugagcaCaguuucaggugugu.................................   110     1   3rd
.........................................................gugagcaaagAuucaggugugu.................................   90      1   3rd
.........................................................gugagcaaaguuucaggugugA.................................   3691    1   3rd
.........................................................gugagcaaaguuucagguAugu.................................   54      1   3rd
.........................................................gugagcaaaguuucaggugugCu.................................   98      1   3rd
.........................................................gugagcaaaguuucagUugugu.................................   172     1   3rd
.........................................................gugagcCaaguuucaggugugu.................................   34      1   3rd
.........................................................gugagcaaaguuucGggugugu.................................   363     1   3rd
.........................................................gugagcaaaguuucaggugugUc................................   61      0   3rd
.........................................................gugagcaaaguuucaggugugUG................................   89      1   3rd
.........................................................gugagcaaaguuucaggugugCc................................   4       1   3rd
.........................................................gugagcaaaguuucaggugugucC...............................   2       1   3rd
.........................................................gugagcaaaguuucagguguguUa...............................   138     1   3rd
.........................................................gugagcaaaguuucaggugugucU...............................   3       1   3rd
.........................................................gugagcaaaguuucagguguguGa...............................   3       1   3rd
.........................................................gugagcaaaguuucaggugugCca...............................   2       1   3rd
.........................................................gugagcaaaguuucaggugugucа...............................   5       0   3rd
.........................................................gugagcaaaguuucagguguguGag..............................   1       1   3rd
```

```
                    Star                                   Mature
ugauaauuugcuucauugauccaucugaaagcuugucucaaccuuugaucugaacaggugagcaaaguuucaggugugucagagaaguuuauuuucaucuuauuaucaacua
.........................................................gugagcaaaguuucaggugugucCg.............................    2      1      3rd
.........................................................gugagcaaaguuucaggugugucagC............................    1      1      3rd
.........................................................gugagcaaaguuucagguguguGagag...........................    3      1      3rd
..........................................................ugagcaaaguuucaggugu..................................    3      0      3rd
..........................................................ugagcaaaguuucagguguU.................................    1      1      3rd
..........................................................ugagcaaaguuucaggugug.................................    6      0      3rd
..........................................................ugagcaaaguuucaggugugu................................    109    0      3rd
..........................................................ugagcaaaguuucagguUugu................................    1      1      3rd
..........................................................Agagcaaaguuucaggugugu................................    1      1      3rd
..........................................................uUagcaaaguuucaggugugu................................    1      1      3rd
..........................................................uAagcaaaguuucaggugugu................................    1      1      3rd
..........................................................uCagcaaaguuucaggugugu................................    2      1      3rd
..........................................................Ggagcaaaguuucaggugugu................................    7      1      3rd
..........................................................ugagcaaaguuucagguguguG...............................    1      1      3rd
...........................................................gagcaaaguuucaggugu..................................    8      0      3rd
...........................................................Uagcaaaguuucaggugu..................................    1      1      3rd
...........................................................gagcaaaguuucaggugug.................................    24     0      3rd
...........................................................gagcaCaguuucaggugugu................................    1      1      3rd
...........................................................Cagcaaaguuucaggugugu................................    1      1      3rd
...........................................................gagcaGaguuucaggugugu................................    1      1      3rd
...........................................................gagcaaaguuucaggugugu................................    302    0      3rd
...........................................................Uagcaaaguuucaggugugu................................    1      1      3rd
...........................................................gCgcaaaguuucaggugugu................................    1      1      3rd
............................................................agcaaaguuucaggugug.................................    2      0      3rd
............................................................agcaaaguuucaggugugu................................    108    0      3rd
............................................................agcaaaguuucagguguAu................................    1      1      3rd
............................................................agcaaaguuucagguguUu................................    1      1      3rd
............................................................agcaaaguuucagUugugu................................    1      1      3rd
............................................................agcaaaguuucaggugugA................................    2      1      3rd
.............................................................Ucaaaguuucaggugugu................................    2      1      3rd
.............................................................gcaaaguuucaggugugu................................    119    0      3rd

....................ccaucugaaagcuugucuc........................................................................    4      0      CA1
....................ccGucugaaagcuugucuc........................................................................    1      1      CA1
....................ccaucugaaagcuugucuca.......................................................................    3      0      CA1
....................ccaucugaaagcuugucucaa......................................................................    5      0      CA1
....................ccaucugaaagcuugucucaac.....................................................................    45     0      CA1
....................ccaucugaaagcuugucucaaU.....................................................................    3      1      CA1
....................ccaucugaaagcuugucucaacA....................................................................    5      1      CA1
....................ccaucugaaagcuugucucaacU....................................................................    9      1      CA1
....................ccaucCgaaagcuugucucaacc....................................................................    2      1      CA1
....................ccaucugaaagcuugucucaacc....................................................................    58     0      CA1
....................ccaucugaaaAcuugucucaaccu...................................................................    3      1      CA1
....................ccaucugaaagcuugucucaaccA...................................................................    18     1      CA1
....................Ucaucugaaagcuugucucaaccu...................................................................    1      1      CA1
....................ccaucGgaaagcuugucucaaccu...................................................................    1      1      CA1
....................ccaucugGaagcuugucucaaccu...................................................................    1      1      CA1
....................ccaucCgaaagcuugucucaaccu...................................................................    1      1      CA1
....................ccaucugaaagcuugucucaaccC...................................................................    18     1      CA1
....................ccaucugaaagcuugCcucaaccu...................................................................    2      1      CA1
....................ccaucugaaagcuugucucaaccG...................................................................    10     1      CA1
....................ccaucugaaagcuugucucGaccu...................................................................    1      1      CA1
....................ccaucugaaagcuugucCcaaccu...................................................................    1      1      CA1
....................ccaucugaaagcuugucucaaccu...................................................................    136    0      CA1
....................ccaucugaaagcuugucucaaccuA..................................................................    3      1      CA1
....................ccaucugaaagcuugucucaaccuu..................................................................    4      0      CA1
....................ccaCcugaaagcuugucucaaccuuu.................................................................    1      1      CA1
.....................caucugaaagcuugucuca.......................................................................    1      0      CA1
.....................caucugaaagcuCgucucaaccu...................................................................    1      1      CA1
.....................caCcugaaagcuugucucaaccuuu.................................................................    3      1      CA1
........................................................Cgugagcaaaguuucaggugug.................................    2      1      CA1
........................................................ggugagcaaaguuucaggugug.................................    2      0      CA1
........................................................Cgugagcaaaguuucaggugugu................................    21     1      CA1
........................................................ggugagcaaaguuucaggugugA................................    2      1      CA1
........................................................ggugagcaaaguuucaggugugu................................    3      0      CA1
.........................................................gugagcaaaguuucaggC....................................    1      1      CA1
.........................................................gugagcaaaguuucaggu....................................    10     0      CA1
.........................................................gugagcaaaguuucaggug...................................    55     0      CA1
.........................................................gugagcaaaguuucagguU...................................    2      1      CA1
.........................................................gugagcaaaguuucagguA...................................    5      1      CA1
```

```
                                            Star                                    Mature
ugauaauuugcuucauugauccaucugaaagcuugucucaaccuuugaucugaacaggugagcaaaguuucaggugugucagagaaguuuauuuucaucuuauuaucaacua
........................................................gugagcaaaguuucaggCg.....................................    1      1    CA1
........................................................gugagcaaaguuucaggugG....................................    3      1    CA1
........................................................gugagcaGaguuucaggugu....................................    1      1    CA1
........................................................gugagcaaaguuucaggugu....................................    135    0    CA1
........................................................gugagcaaGguuucaggugu....................................    1      1    CA1
........................................................gugagcaaaguuucaggGgu....................................    1      1    CA1
........................................................gugagcaaaguuucaggugC....................................    16     1    CA1
........................................................gugagcaaagGuucaggugug...................................    1      1    CA1
........................................................gugagcaUaguuucaggugug...................................    1      1    CA1
........................................................gugagcaaaguuucUggugug...................................    1      1    CA1
........................................................gugagcaaaguuucaggugAg...................................    1      1    CA1
........................................................gugagcaaaguuuUaggugug...................................    3      1    CA1
........................................................gugagcaGaguuucaggugug...................................    1      1    CA1
........................................................gugagcaaaguuucGggugug...................................    6      1    CA1
........................................................gugagcaaaguuucaggCgug...................................    4      1    CA1
........................................................guAagcaaaguuucaggugug...................................    1      1    CA1
........................................................gugagcaaaguCucaggugug...................................    2      1    CA1
........................................................gugGgcaaaguuucaggugug...................................    5      1    CA1
........................................................gugagcaaaguuucaggugCg...................................    3      1    CA1
........................................................gCgagcaaaguuucaggugug...................................    1      1    CA1
........................................................gugagcaaaguuucagguguU...................................    12     1    CA1
........................................................gugagcaaaguuucagguguC...................................    7      1    CA1
........................................................gugagcaaGguuucaggugug...................................    3      1    CA1
........................................................gugagcUaaguuucaggugug...................................    1      1    CA1
........................................................gugagcaaaguuucaggugug...................................    580    0    CA1
........................................................gugagcaaaguuucagguguA...................................    43     1    CA1
........................................................gugagcaaaguuucagAugug...................................    2      1    CA1
........................................................gugagcaaaguuucaggugGg...................................    1      1    CA1
........................................................gugagcaaaguuucCggugug...................................    1      1    CA1
........................................................Augagcaaaguuucaggugug...................................    1      1    CA1
........................................................gugagcaaaguuucaggCgugu..................................    23     1    CA1
........................................................gugagcaaaguuucagAugugu..................................    2      1    CA1
........................................................gugagcaaaguuucaggugugG..................................    190    1    CA1
........................................................gugagcGaaguuucaggugugu..................................    28     1    CA1
........................................................gugagcaaaguuucaggugGgu..................................    14     1    CA1
........................................................gCgagcaaaguuucaggugugu..................................    20     1    CA1
........................................................gugagcaaaguuucaggugCgu..................................    31     1    CA1
........................................................gugagcaaaguuucagguguAu..................................    8      1    CA1
........................................................gugagcaaaguAucaggugugu..................................    1      1    CA1
........................................................gugagcUaaguuucaggugugu..................................    1      1    CA1
........................................................gugagcaaaguuAcaggugugu..................................    2      1    CA1
........................................................gGgagcaaaguuucaggugugu..................................    3      1    CA1
........................................................gugagcCaaguuucaggugugu..................................    4      1    CA1
........................................................gugagcaCaguuucaggugugu..................................    1      1    CA1
........................................................gugagcaaaguuucCggugugu..................................    3      1    CA1
........................................................gugagcaaagCuucaggugugu..................................    38     1    CA1
........................................................gugaAcaaaguuucaggugugu..................................    18     1    CA1
........................................................gugagcaaaguuCcaggugugu..................................    30     1    CA1
........................................................gugagcaaaguuucGggugugu..................................    49     1    CA1
........................................................gugagcaUaguuucaggugugu..................................    9      1    CA1
........................................................gugagcaaaguuucagguAugu..................................    8      1    CA1
........................................................gAgagcaaaguuucaggugugu..................................    2      1    CA1
........................................................Uugagcaaaguuucaggugugu..................................    4      1    CA1
........................................................gugagUaaaguuucaggugugu..................................    17     1    CA1
........................................................gugUgcaaaguuucaggugugu..................................    5      1    CA1
........................................................gugagcaaaguuGcaggugugu..................................    4      1    CA1
........................................................gugaUcaaaguuucaggugugu..................................    1      1    CA1
........................................................gugagcaaaguuucaAgugugu..................................    5      1    CA1
........................................................gugagcaaaguuuUaggugugu..................................    20     1    CA1
........................................................gugagcaaUguuucaggugugu..................................    5      1    CA1
........................................................gugagcaaaguuucUggugugu..................................    11     1    CA1
........................................................gugagcaaaguuucaggGgugu..................................    5      1    CA1
........................................................Cugagcaaaguuucaggugugu..................................    3      1    CA1
........................................................gugagAaaaguuucaggugugu..................................    3      1    CA1
........................................................gugagcaaaguuucaggAgugu..................................    11     1    CA1
........................................................Augagcaaaguuucaggugugu..................................    12     1    CA1
........................................................gugagcaaaguuucaUgugugu..................................    3      1    CA1
........................................................guUagcaaaguuucaggugugu..................................    1      1    CA1
........................................................gugagcaaGguuucaggugugu..................................    45     1    CA1
........................................................gugagcaaaguuucaggugAgu..................................    14     1    CA1
```

```
                    Star                                    Mature
ugauaauuugcuucauugauccaucugaaagcuugucucaaccuuugaucugaacaggugagcaaaguuucaggugugucagagaaguuuauuuucaucuuauuaucaacua
.........................................................gugagcaaaAuuucaggugugu.................................      3      1      CA1
.........................................................gugagcaaaguuucagguguCu.................................      3      1      CA1
.........................................................gugagcaaagGuucaggugugu.................................      3      1      CA1
.........................................................gugagcaaaguuucaggugugu.................................      11198  0      CA1
.........................................................gugagcaaaguuucagguUugu.................................      6      1      CA1
.........................................................gugCgcaaaguuucaggugugu.................................      3      1      CA1
.........................................................gugagGaaaguuucaggugugu.................................      2      1      CA1
.........................................................gugagcaaaguuucaggugugA.................................      303    1      CA1
.........................................................gugagcaaaguGucaggugugu.................................      1      1      CA1
.........................................................gugGgcaaaguuucaggugugu.................................      46     1      CA1
.........................................................gugagcaGaguuucaggugugu.................................      33     1      CA1
.........................................................gugagcaaaguuucagguguUu.................................      4      1      CA1
.........................................................gugagcaaaguCucaggugugu.................................      20     1      CA1
.........................................................guAagcaaaguuucaggugugu.................................      7      1      CA1
.........................................................gugagcaaaguuucaggugugUc................................      33     0      CA1
.........................................................gugagcaaaguuucaggugugugG................................      21     1      CA1
.........................................................gugagcaaaguuucGgguguguc................................      2      1      CA1
.........................................................gugagcaaaguuucaggugugugUa...............................      1      1      CA1
..........................................................ugagcaaaguuucaggugugu.................................      9      0      CA1
..........................................................ugGgcaaaguuucaggugugu.................................      1      1      CA1
..........................................................ugagcaaaguuucaggGgugu.................................      1      1      CA1
...........................................................gagcaaaguuucaggugug..................................      1      0      CA1
...........................................................gagcaaaguuucaggugugu.................................      5      0      CA1
............................................................agcaaaguuucaggugugu.................................      2      0      CA1

....................ccaucugaaagcuugucucaacc.....................................................................      4      0      HJ1
....................ccaucugaaagcuugucucaacA.....................................................................      2      1      HJ1
....................ccaucugaaagcuugucucaacU.....................................................................      1      1      HJ1
....................ccaucugaaagcuugucucaaccC....................................................................      1      1      HJ1
....................ccaucugaaagcuugucucaaccu....................................................................      3      0      HJ1
.....................caucugaaagcuugucucaacc.....................................................................      1      0      HJ1
........................cugaaagcuugucucaaccu....................................................................      1      0      HJ1
............................................uugaucugaacaggugagcaaaguuuc.........................................      1      0      HJ1
.......................................................aAgugagcaaaguuucaggugugu.................................      1      1      HJ1
........................................................ggugagcaaaguuucaggugu...................................      1      0      HJ1
........................................................Cgugagcaaaguuucaggugug..................................      1      1      HJ1
........................................................ggugagcaaaguuucagguguC..................................      1      1      HJ1
........................................................ggugagcaaaguuucaggugug..................................      5      0      HJ1
........................................................ggugagcaaaguuucaggugugu.................................      2      0      HJ1
........................................................Agugagcaaaguuucaggugugu.................................      2      1      HJ1
........................................................ggugagcaaaguuucGggugugu.................................      1      1      HJ1
........................................................Cgugagcaaaguuucaggugugu.................................      9      1      HJ1
.........................................................gugagcaaaguuucaggu.....................................      51     0      HJ1
.........................................................gugagcaaaguuucaUgu.....................................      2      1      HJ1
.........................................................gugagcaaaguuucaggA.....................................      6      1      HJ1
.........................................................gAgagcaaaguuucaggu.....................................      1      1      HJ1
.........................................................gugaUcaaaguuucaggu.....................................      1      1      HJ1
.........................................................gugagcaaaguuucGggu.....................................      1      1      HJ1
.........................................................Augagcaaaguuucaggu.....................................      1      1      HJ1
.........................................................gugagcaaaguuCcaggu.....................................      1      1      HJ1
.........................................................gugagcaaaguuucaggC.....................................      14     1      HJ1
.........................................................gugGgcaaaguuucaggug....................................      1      1      HJ1
.........................................................gugagcaaaguuucagguU....................................      3      1      HJ1
.........................................................gAgagcaaaguuucaggug....................................      1      1      HJ1
.........................................................gugagcaaaguuucagCug....................................      2      1      HJ1
.........................................................gugagcaaaguuucaggGg....................................      1      1      HJ1
.........................................................gugagcaaagCuucaggug....................................      2      1      HJ1
.........................................................gugaAcaaaguuucaggug....................................      1      1      HJ1
.........................................................gugagcaaaguuucaggug....................................      181    0      HJ1
.........................................................gugagcaaaguuucGggug....................................      1      1      HJ1
.........................................................gugagcaaaguuucagguA....................................      25     1      HJ1
.........................................................gugagcGaaguuucaggug....................................      1      1      HJ1
.........................................................gugagcaaaguuucagguC....................................      1      1      HJ1
.........................................................gugagcaaaguuucaggAg....................................      1      1      HJ1
.........................................................Augagcaaaguuucaggug....................................      1      1      HJ1
.........................................................gugagcaaagGuucaggug....................................      1      1      HJ1
.........................................................gugagcaGaguuucaggug....................................      1      1      HJ1
.........................................................gugagcaaaguuucaggCg....................................      1      1      HJ1
.........................................................gGgagcaaaguuucaggugu...................................      1      1      HJ1
.........................................................gAgagcaaaguuucaggugu...................................      3      1      HJ1
```

Star Mature

```
ugauaauuugcuucauugauccaucugaaagcuugucucaaccuuugaucugaacaggugagcaaaguuucaggugugucagagaaguuuauuuucaucuuauuaucaacua
.........................................................gugGgcaaaguuucaggugu...................................   2      1     HJ1
.........................................................gugagcGaaguuucaggugu...................................   2      1     HJ1
.........................................................gugagcaaaguuCcaggugu...................................   3      1     HJ1
.........................................................gugagcaaaguuucaggugG...................................   12     1     HJ1
.........................................................gugagcaaaguuucagguAu...................................   1      1     HJ1
.........................................................gugagUaaaguuucaggugu...................................   1      1     HJ1
.........................................................gugagcaaaguuucGggugu...................................   1      1     HJ1
.........................................................gugaCcaaaguuucaggugu...................................   1      1     HJ1
.........................................................gugagcaaaguCucaggugu...................................   2      1     HJ1
.........................................................gugagcaaaguuucaggugC...................................   72     1     HJ1
.........................................................gCgagcaaaguuucaggugu...................................   1      1     HJ1
.........................................................Augagcaaaguuucaggugu...................................   3      1     HJ1
.........................................................gugagcaaaguuucaggGgu...................................   1      1     HJ1
.........................................................gugagcaaaguuucUggugu...................................   1      1     HJ1
.........................................................gugagcaGaguuucaggugu...................................   1      1     HJ1
.........................................................gugaAcaaaguuucaggugu...................................   1      1     HJ1
.........................................................gugagcaaaguuucaggCgu...................................   2      1     HJ1
.........................................................gugagcaaaguuucaUgugu...................................   1      1     HJ1
.........................................................gugagcaaaguuucaggugu...................................   455    0     HJ1
.........................................................Uugagcaaaguuucaggugu...................................   1      1     HJ1
.........................................................gugagcaaaguuucagAugu...................................   1      1     HJ1
.........................................................gugagAaaaguuucaggugug..................................   2      1     HJ1
.........................................................gugagcaaaguuCcaggugug..................................   5      1     HJ1
.........................................................gugaAcaaaguuucaggugug..................................   7      1     HJ1
.........................................................Augagcaaaguuucaggugug..................................   5      1     HJ1
.........................................................gugagcaaaguuAcaggugug..................................   1      1     HJ1
.........................................................gugagcaaaguuucagAugug..................................   2      1     HJ1
.........................................................gugagcaaaguuucagguAug..................................   2      1     HJ1
.........................................................gugUgcaaaguuucaggugug..................................   1      1     HJ1
.........................................................gugagcaaaguuucaggugCg..................................   8      1     HJ1
.........................................................gugagcaaCguuucaggugug..................................   1      1     HJ1
.........................................................gugagcaaagCuucaggugug..................................   3      1     HJ1
.........................................................gGgagcaaaguuucaggugug..................................   1      1     HJ1
.........................................................gugagcaaUguuucaggugug..................................   3      1     HJ1
.........................................................gugagcaaaguuucaggCgug..................................   2      1     HJ1
.........................................................gCgagcaaaguuucaggugug..................................   1      1     HJ1
.........................................................gugaCcaaaguuucaggugug..................................   1      1     HJ1
.........................................................gugagcaaaguuucagguguC..................................   15     1     HJ1
.........................................................gugagcaaagGuucaggugug..................................   1      1     HJ1
.........................................................gugagcaaaguuucGggugug..................................   7      1     HJ1
.........................................................gugagcaaaguuucagguCug..................................   1      1     HJ1
.........................................................Cugagcaaaguuucaggugug..................................   1      1     HJ1
.........................................................gAgagcaaaguuucaggugug..................................   8      1     HJ1
.........................................................gugagcaaaguuucagguguA..................................   200    1     HJ1
.........................................................gugagUaaaguuucaggugug..................................   1      1     HJ1
.........................................................gugagcaaaguuuGaggugug..................................   1      1     HJ1
.........................................................gugagcaaaguuuAaggugug..................................   9      1     HJ1
.........................................................gugagcaaaguuucagguguU..................................   61     1     HJ1
.........................................................gugagcGaaguuucaggugug..................................   3      1     HJ1
.........................................................gugagcaaaguuucaAgugug..................................   4      1     HJ1
.........................................................gugCgcaaaguuucaggugug..................................   1      1     HJ1
.........................................................gugagcaaaguuucaggugGg..................................   14     1     HJ1
.........................................................gugagcaaaguuucaggugAg..................................   1      1     HJ1
.........................................................gugagcaaaguuucaggugug..................................   1724   0     HJ1
.........................................................gugagcaaaguCucaggugug..................................   3      1     HJ1
.........................................................gugagcUaaguuucaggugug..................................   2      1     HJ1
.........................................................gugagcaaagAuucaggugug..................................   1      1     HJ1
.........................................................gugagcaaaguuucaggGgug..................................   3      1     HJ1
.........................................................gugGgcaaaguuucaggugug..................................   6      1     HJ1
.........................................................gugagcaaaguuucaUgugug..................................   3      1     HJ1
.........................................................Uugagcaaaguuucaggugug..................................   2      1     HJ1
.........................................................gugagcaUaguuucaggugug..................................   2      1     HJ1
.........................................................gugagcaGaguuucaggugug..................................   4      1     HJ1
.........................................................gugagcaaaguuucUggugug..................................   1      1     HJ1
.........................................................gugagcaaaguuuUaggugug..................................   2      1     HJ1
.........................................................guAagcaaaguuucaggugug..................................   1      1     HJ1
.........................................................gugaUcaaaguuucaggugug..................................   2      1     HJ1
.........................................................gugagcaaGguuucaggugug..................................   11     1     HJ1
.........................................................gGgagcaaaguuucaggugugu.................................   6      1     HJ1
.........................................................gugagcUaaguuucaggugugu.................................   7      1     HJ1
```

Star Mature

ugauaauuugcuucauugauccaucugaaagcuugucucaaccuuugaucugaacaggugagcaaaguuucaggugugucagagaaguuuauuuucaucuuauuaucaacua			
....gugagcaaaguuCcaggugugu.................................	58	1	HJ1
....gugagcaaaguuucaUgugugu.................................	4	1	HJ1
....gugagcaaaguuucCggugugu.................................	1	1	HJ1
....gugagcaaaguuucUggugugu.................................	7	1	HJ1
....gugagUaaaguuucaggugugu.................................	27	1	HJ1
....gugagcaaagCuucaggugugu.................................	41	1	HJ1
....gugagcaaaguuucaggAgugu.................................	6	1	HJ1
....gugagcaaaguuucaggugAgu.................................	11	1	HJ1
....gugagcaaaguuuAaggugugu.................................	18	1	HJ1
....gugagcCaaguuucaggugugu.................................	1	1	HJ1
....gugagcaaaguuucagguguUu.................................	37	1	HJ1
....gugUgcaaaguuucaggugugu.................................	9	1	HJ1
....Uugagcaaaguuucaggugugu.................................	17	1	HJ1
....gugaUcaaaguuucaggugugu.................................	14	1	HJ1
....gugagcaaaguuucaggCgugu.................................	40	1	HJ1
....gugagcaaaguuuUaggugugu.................................	15	1	HJ1
....gugagcaaaguuucGggugugu.................................	60	1	HJ1
....gCgagcaaaguuucaggugugu.................................	42	1	HJ1
....gugagcaUaguuucaggugugu.................................	8	1	HJ1
....guUagcaaaguuucaggugugu.................................	3	1	HJ1
....gugagcaaaguuAcaggugugu.................................	4	1	HJ1
....gugagcaaaguuucaCgugugu.................................	2	1	HJ1
....gugagcaaagGuucaggugugu.................................	4	1	HJ1
....gugagcaaaUuuucaggugugu.................................	1	1	HJ1
....gugagGaaaguuucaggugugu.................................	2	1	HJ1
....gugagcaaaguuucagguguCu.................................	23	1	HJ1
....gugagAaaaguuucaggugugu.................................	25	1	HJ1
....gugagcGaaguuucaggugugu.................................	45	1	HJ1
....gugagcaaaguuucaggugugA.................................	633	1	HJ1
....gugagcaaaguuucaggugugu.................................	13486	0	HJ1
....gugagcaaaguuucaggugugG.................................	443	1	HJ1
....guCagcaaaguuucaggugugu.................................	1	1	HJ1
....gugagcaaaguuuGaggugugu.................................	8	1	HJ1
....gugagcaaaguuucagUugugu.................................	1	1	HJ1
....Cugagcaaaguuucaggugugu.................................	8	1	HJ1
....gugagcaaUguuucaggugugu.................................	15	1	HJ1
....gugagcaaaAuuucaggugugu.................................	3	1	HJ1
....gugagcaaaguuucagAugugu.................................	13	1	HJ1
....gugagcaaGguuucaggugugu.................................	64	1	HJ1
....gugagcaaaguuGcaggugugu.................................	1	1	HJ1
....gugagcaaaguuucagguguAu.................................	39	1	HJ1
....gugagcaaaguuucaggugGgu.................................	42	1	HJ1
....gugCgcaaaguuucaggugugu.................................	6	1	HJ1
....gugagcaaCguuucaggugugu.................................	7	1	HJ1
....gugGgcaaaguuucaggugugu.................................	60	1	HJ1
....gugagcaaaguuucagguCugu.................................	1	1	HJ1
....Augagcaaaguuucaggugugu.................................	42	1	HJ1
....gugagcaaaCuuucaggugugu.................................	1	1	HJ1
....guAagcaaaguuucaggugugu.................................	9	1	HJ1
....gugagcaaaguuucaggGgugu.................................	10	1	HJ1
....gugagcaGaguuucaggugugu.................................	49	1	HJ1
....gugagcaaaguuucaAgugugu.................................	20	1	HJ1
....gugagcaaaguuucaggugCgu.................................	47	1	HJ1
....gugaAcaaaguuucaggugugu.................................	61	1	HJ1
....gugagcaaagAuucaggugugu.................................	1	1	HJ1
....gugagcaaaguuucagguUugu.................................	12	1	HJ1
....gAgagcaaaguuucaggugugu.................................	29	1	HJ1
....gugaCcaaaguuucaggugugu.................................	11	1	HJ1
....gugagcaaaguCucaggugugu.................................	38	1	HJ1
....gugagcaaaguuucagguAugu.................................	8	1	HJ1
....gugagcaaaguGucaggugugu.................................	3	1	HJ1
....gugagcaaaguuucaggugugCc................................	1	1	HJ1
....gugagcaaaguuucagguguguG................................	63	1	HJ1
....gugagcaaaguuucaggugAguc................................	1	1	HJ1
....gugagcaaaguuucagguguguc................................	46	0	HJ1
....gugagcaaaguuucGgguguguc................................	1	1	HJ1
....gugagcaaaguuucaggCguguc................................	1	1	HJ1
....ugagcaaaguuucaggugugu.................................	7	0	HJ1
....ugagcaaaguuucaggugugA.................................	2	1	HJ1
....ugagcaGaguuucaggugugu.................................	1	1	HJ1

Star **Mature**

```
ugauaauuugcuucauugauccaucugaaagcuugucucaaccuuugaucugaacaggugagcaaaguuucaggugugucagagaaguuuauuuucaucuuauuaucaacua
..........................................................Cgagcaaaguuucaggugugu.................................   1     1   HJ1
..........................................................ugagcaaaguuucaggugugG.................................   2     1   HJ1
...........................................................gagcaaaguuucaggugG...................................   1     1   HJ1
...........................................................gagcaaaguuucaggugugu.................................   7     0   HJ1
............................................................agcaaaguuucagguguA..................................   1     1   HJ1
............................................................agcaaaguuucaggugugu.................................   3     0   HJ1
.............................................................gcaaaguuucaggugugu.................................   1     0   HJ1
.............................................................gcaaaguuucaggugugG.................................   1     1   HJ1

....................ccaucugaaagcuugucuU.........................................................................   1     1   LA1
....................ccaucugaaagcuugucuc.........................................................................   11    0   LA1
....................ccaucugaaagcuugucuca........................................................................   2     0   LA1
....................ccaucugaaagcuugucucaG.......................................................................   1     1   LA1
....................ccaucugaaagcuugucucaa.......................................................................   7     0   LA1
....................ccaucugaaagcuugCcucaac......................................................................   1     1   LA1
....................ccaucugaaagcuugucucaac......................................................................   34    0   LA1
....................ccaucugaaGgcuugucucaac......................................................................   1     1   LA1
....................ccauUugaaagcuugucucaacc.....................................................................   1     1   LA1
....................ccaucugaaagcuugucucaacc.....................................................................   60    0   LA1
....................ccaucugaaagcuugucucaacU.....................................................................   11    1   LA1
....................ccaucugaaagcuugucucaacA.....................................................................   1     1   LA1
....................ccaucuAaaagcuugucucaaccu....................................................................   1     1   LA1
....................ccaucugaGagcuugucucaaccu....................................................................   1     1   LA1
....................ccaucCgaaagcuugucucaaccu....................................................................   2     1   LA1
....................ccaucugaaagcuugucucaaccu....................................................................   132   0   LA1
....................ccaucugaaagcuugucucaaccC....................................................................   10    1   LA1
....................ccaucugaaagcuugucucaaccG....................................................................   3     1   LA1
....................ccaucugaaagcuugucucaaccA....................................................................   14    1   LA1
....................ccaucugaaagcCugucucaaccu....................................................................   2     1   LA1
....................ccaucugaaagcuugucucaaccuu...................................................................   1     0   LA1
....................ccaucugaaagcuugucucaaccuA...................................................................   1     1   LA1
.....................caCcugaaagcuugucucaaccuuu..................................................................   2     1   LA1
.......................ucugaaagcuugucucaacc.....................................................................   2     0   LA1
............................................uugaucugaacaggugagcaaaguuuc.........................................   1     0   LA1
........................................................Cgugagcaaaguuucaggugugu.................................   7     1   LA1
........................................................ggugagcaaaguuucaggugugu.................................   2     0   LA1
.........................................................gugagcaaaguuucaggu.....................................   19    0   LA1
.........................................................gugagcaaaguuucaggC.....................................   2     1   LA1
.........................................................gugagcaaaguuucaggug....................................   48    0   LA1
.........................................................gugagcaaaguuucagguA....................................   5     1   LA1
.........................................................gugagcaaaguuGcaggug....................................   1     1   LA1
.........................................................gugagcaaaguuucUggugu...................................   1     1   LA1
.........................................................gugagcaaaguCucaggugu...................................   1     1   LA1
.........................................................gugagcaaaguuucaggugu...................................   118   0   LA1
.........................................................gugUgcaaaguuucaggugu...................................   1     1   LA1
.........................................................gugagcaaaguuucaggGgu...................................   1     1   LA1
.........................................................gugagcaaaguuucaggugG...................................   1     1   LA1
.........................................................gugagcaaaguuucaggugC...................................   4     1   LA1
.........................................................gugagcaaaguuuUaggugu...................................   1     1   LA1
.........................................................gugagcaaaguuucUggugug..................................   3     1   LA1
.........................................................gugagcaaaguuuUaggugug..................................   2     1   LA1
.........................................................gugagcaaCguuucaggugug..................................   1     1   LA1
.........................................................gugagcaaaguuucaggugAg..................................   2     1   LA1
.........................................................gugagcaGaguuucaggugug..................................   2     1   LA1
.........................................................gugagcaaaguAucaggugug..................................   1     1   LA1
.........................................................gugagcUaaguuucaggugug..................................   1     1   LA1
.........................................................gCgagcaaaguuucaggugug..................................   1     1   LA1
.........................................................gugagcaaaguuucaggugGg..................................   4     1   LA1
.........................................................gugagcaaaguuAcaggugug..................................   2     1   LA1
.........................................................gugagcaaaguuucagguguU..................................   15    1   LA1
.........................................................gugagcaaaguuucaggugug..................................   879   0   LA1
.........................................................gugagcaaaguuucCggugug..................................   2     1   LA1
.........................................................gugagcaaGguuucaggugug..................................   7     1   LA1
.........................................................gugagcaaaguuucaggGgug..................................   1     1   LA1
.........................................................gugagcaaaguuucaggugCg..................................   12    1   LA1
.........................................................gugagcaaaguuucagguguA..................................   56    1   LA1
.........................................................gugagcaaaguuucGggugug..................................   3     1   LA1
.........................................................gAgagcaaaguuucaggugug..................................   1     1   LA1
.........................................................gugagcaaaguCucaggugug..................................   1     1   LA1
.........................................................gugagcGaaguuucaggugug..................................   3     1   LA1
```

Star **Mature**

```
ugauaauuugcuucauugauccaucugaaagcuugucucaaccuuugaucugaacaggugagcaaaguuucaggugugucagagaaguuuauuuucaucuuauuaucaacua
........................................................gugagcaaagGuucaggugug...................................  2     1  LA1
........................................................gugagcaaaguuCcaggugug...................................  1     1  LA1
........................................................gugagcaaaguuucagguguC...................................  1     1  LA1
........................................................gugGgcaaaguuucaggugug...................................  3     1  LA1
........................................................gugagcaaaguuucaAgugugu..................................  5     1  LA1
........................................................gugagcaaGguuucaggugugu..................................  21    1  LA1
........................................................gugagcaaaguuucaggugugG..................................  96    1  LA1
........................................................gugagcaaaguuuAaggugugu..................................  3     1  LA1
........................................................gGgagcaaaguuucaggugugu..................................  1     1  LA1
........................................................gugagcaaaguuAcaggugugu..................................  4     1  LA1
........................................................gCgagcaaaguuucaggugugu..................................  25    1  LA1
........................................................gugagcaaaguuucUggugugu..................................  4     1  LA1
........................................................gugagcaUaguuucaggugugu..................................  2     1  LA1
........................................................gugagcaaaguuucagguguCu..................................  1     1  LA1
........................................................gugagcaaaguuucaUgugugu..................................  1     1  LA1
........................................................gugagcaaaguuucaggugugA..................................  171   1  LA1
........................................................gugagcaaaguuucGggugugu..................................  33    1  LA1
........................................................gugagcaaaguuucaggugGgu..................................  10    1  LA1
........................................................gugagcaaUguuucaggugugu..................................  2     1  LA1
........................................................gugGgcaaaguuucaggugugu..................................  32    1  LA1
........................................................gugagcaaaguuucaggugAgu..................................  13    1  LA1
........................................................gugagcaaaguuucagguguUu..................................  5     1  LA1
........................................................gugagcaaaguuuUaggugugu..................................  9     1  LA1
........................................................gugagcaaagGuucaggugugu..................................  1     1  LA1
........................................................gugagcaaaguuucagAugugu..................................  3     1  LA1
........................................................gugagcaaagCuucaggugugu..................................  9     1  LA1
........................................................gugagcaaaguuucaggugCgu..................................  13    1  LA1
........................................................gugagcaaaguAucaggugugu..................................  2     1  LA1
........................................................gugaAcaaaguuucaggugugu..................................  5     1  LA1
........................................................gugagcaaaguuucagguAugu..................................  1     1  LA1
........................................................Uugagcaaaguuucaggugugu..................................  5     1  LA1
........................................................gugagcaaaAuuucaggugugu..................................  1     1  LA1
........................................................gugaUcaaaguuucaggugugu..................................  1     1  LA1
........................................................gugagcaaaguuucagguCugu..................................  1     1  LA1
........................................................Augagcaaaguuucaggugugu..................................  10    1  LA1
........................................................gugagcaaaguuucaggugugu..................................  6414  0  LA1
........................................................gugagcaaaguuucagguguAu..................................  7     1  LA1
........................................................gugagcaaaguuucaggCgugu..................................  19    1  LA1
........................................................gugagcaaagAuucaggugugu..................................  2     1  LA1
........................................................gugagcCaaguuucaggugugu..................................  2     1  LA1
........................................................guAagcaaaguuucaggugugu..................................  5     1  LA1
........................................................gugCgcaaaguuucaggugugu..................................  3     1  LA1
........................................................gugagAaaaguuucaggugugu..................................  1     1  LA1
........................................................gugagcaaaguCucaggugugu..................................  15    1  LA1
........................................................gugagcGaaguuucaggugugu..................................  13    1  LA1
........................................................gugagcaaaguuucaggAgugu..................................  9     1  LA1
........................................................gugagcaaaguuucCggugugu..................................  2     1  LA1
........................................................gugagUaaaguuucaggugugu..................................  10    1  LA1
........................................................gugagcUaaguuucaggugugu..................................  1     1  LA1
........................................................guCagcaaaguuucaggugugu..................................  1     1  LA1
........................................................gugagcaaaguuCcaggugugu..................................  18    1  LA1
........................................................gugaCcaaaguuucaggugugu..................................  2     1  LA1
........................................................gugagcaGaguuucaggugugu..................................  19    1  LA1
........................................................gAgagcaaaguuucaggugugu..................................  2     1  LA1
........................................................gugUgcaaaguuucaggugugu..................................  2     1  LA1
........................................................gugagcaaaguGucaggugugu..................................  1     1  LA1
........................................................gugagcaaCguuucaggugugu..................................  3     1  LA1
........................................................gugagcaaaguuucaggGgugu..................................  5     1  LA1
........................................................gugagcaaaguuucaggugugucc.................................  28    0  LA1
........................................................gugagcaaaguuucaggugugug G................................  20    1  LA1
........................................................Augagcaaaguuucaggugugucc.................................  1     1  LA1
.........................................................ugagcaaaguuucaggugugu..................................  4     0  LA1
.........................................................ugagcaaaguuucaggugugG..................................  1     1  LA1
..........................................................gagcaaaguuucagguguA..................................  1     1  LA1

....................ccaucugaaagcuugucuc.........................................................................  2     0  CJ1
....................ccaucugaaagcuugucuca........................................................................  1     0  CJ1
....................ccaucugaaagcuugucucaa.......................................................................  1     0  CJ1
....................ccaucugaaagcuugucucaaU......................................................................  1     1  CJ1
....................ccaucugaaagcuugucucaac......................................................................  8     0  CJ1
```

```
                    Star                                 Mature
ugauaauuugcuucauugauccaucugaaagcuugucucaaccuuugaucugaacaggugagcaaaguuucaggugugucagagaaguuuauuuucaucuuauuaucaacua
....................ccaucugaaagcuugucucaacU.....................................................................    8     1    CJ1
....................ccaucugaaagcuugucucaacc.....................................................................    7     0    CJ1
....................ccaucugaaagcuugucucaaccC....................................................................    1     1    CJ1
....................ccaucugaaagcuugucucaaccA....................................................................    2     1    CJ1
....................ccaucugaaagcuugucucaaccu....................................................................    19    0    CJ1
.......................................................aAgugagcaaaguuucaggugugu.................................    2     1    CJ1
........................................................ggugagcaaaguuucaggug....................................    1     0    CJ1
........................................................ggugagcaaaguuucaggugu...................................    1     0    CJ1
........................................................ggugagcaaaguuucaggugU...................................    1     1    CJ1
........................................................ggugagcaaaguuucaggugug..................................    2     0    CJ1
........................................................ggugagAaaaguuucaggugugu.................................    1     1    CJ1
........................................................Cgugagcaaaguuucaggugugu.................................    16    1    CJ1
........................................................ggugagcaaaguuucaggugugu.................................    6     0    CJ1
.........................................................gugagcaaaguuucaggC.....................................    1     1    CJ1
.........................................................gugagcaaaguuucaggu.....................................    14    0    CJ1
.........................................................gugagcaaaguuucagguC....................................    1     1    CJ1
.........................................................gugagcCaaguuucaggug....................................    1     1    CJ1
.........................................................gugagcaaaguuucagguU....................................    2     1    CJ1
.........................................................gugagcaaaguuucaggug....................................    57    0    CJ1
.........................................................gugagcaaGguuucaggug....................................    1     1    CJ1
.........................................................gugagcaaaguuucGggug....................................    1     1    CJ1
.........................................................gugagcaaaguuucagguA....................................    8     1    CJ1
.........................................................gugagcaaaguuucaggugG...................................    2     1    CJ1
.........................................................gugagcaaaguuucGgggu...................................    1     1    CJ1
.........................................................gugagcaaaguuucaggCgu...................................    1     1    CJ1
.........................................................gugagcaaaguuucaggugC...................................    18    1    CJ1
.........................................................gugagcaaaguuucaggugu...................................    161   0    CJ1
.........................................................guCagcaaaguuucaggugu...................................    1     1    CJ1
.........................................................gugagcaaaguuuUaggugu...................................    1     1    CJ1
.........................................................Augagcaaaguuucaggugu...................................    1     1    CJ1
.........................................................gugGgcaaaguuucaggugu...................................    1     1    CJ1
.........................................................gugagcaaaguuucaggGgu...................................    1     1    CJ1
.........................................................gugagcaaaguuucagguUu...................................    1     1    CJ1
.........................................................gugagcaGaguuucaggugu...................................    1     1    CJ1
.........................................................gugagcCaaguuucaggugu...................................    1     1    CJ1
.........................................................gugUgcaaaguuucaggugu...................................    1     1    CJ1
.........................................................gugagcaaaguCucaggugu...................................    1     1    CJ1
.........................................................gugagcaaaguuucaggugAg..................................    2     1    CJ1
.........................................................gugagcaaGguuucaggugug..................................    3     1    CJ1
.........................................................gugagcaaaguuucagguguU..................................    18    1    CJ1
.........................................................gugagUaaaguuucaggugug..................................    1     1    CJ1
.........................................................gugagcaaaguuucagguguA..................................    84    1    CJ1
.........................................................gugagcaaUguuucaggugug..................................    1     1    CJ1
.........................................................gugagcaaaguuucGggugug..................................    6     1    CJ1
.........................................................gugUgcaaaguuucaggugug..................................    1     1    CJ1
.........................................................gugagcaaaguuucaUgugug..................................    4     1    CJ1
.........................................................gugagcaaaguuucUggugug..................................    2     1    CJ1
.........................................................guUagcaaaguuucaggugug..................................    4     1    CJ1
.........................................................gugagcaaaguuucaggugGg..................................    1     1    CJ1
.........................................................gugagcaaaguuucaggCgug..................................    1     1    CJ1
.........................................................gCgagcaaaguuucaggugug..................................    4     1    CJ1
.........................................................gugGgcaaaguuucaggugug..................................    4     1    CJ1
.........................................................gugagcaaagGuucaggugug..................................    1     1    CJ1
.........................................................gugagcaaaguuCcaggugug..................................    3     1    CJ1
.........................................................gugagcaaaguuucaggugug..................................    793   0    CJ1
.........................................................gugagcaaaguCucaggugug..................................    2     1    CJ1
.........................................................gugagcaaaguuucaggugCg..................................    3     1    CJ1
.........................................................gugaAcaaaguuucaggugug..................................    1     1    CJ1
.........................................................gugagcaaaguuucagguAug..................................    1     1    CJ1
.........................................................Uugagcaaaguuucaggugug..................................    1     1    CJ1
.........................................................gugagcaaaguuucaggGgug..................................    1     1    CJ1
.........................................................gugagcaaagCuucaggugug..................................    1     1    CJ1
.........................................................gugagcaaaguuucaAgugug..................................    1     1    CJ1
.........................................................gugagcaaaguuucagguguC..................................    3     1    CJ1
.........................................................gugagcGaaguuucaggugug..................................    1     1    CJ1
.........................................................guAagcaaaguuucaggugugu.................................    26    1    CJ1
.........................................................gugagcaaaguuucUggugugu.................................    2     1    CJ1
.........................................................gugagcaaaguuucagguguCu.................................    4     1    CJ1
.........................................................gugagcaaaguuucaggugAgu.................................    4     1    CJ1
.........................................................Augagcaaaguuucaggugugu.................................    12    1    CJ1
```

Star Mature

```
ugauaauuugcuucauugauccaucugaaagcuugucucaaccuuugaucugaacaggugagcaaaguuucaggugugucagagaaguuuauuuucaucuuauuaucaacua
.........................................................gugagcaaaguuuUaggugugu.................................   14     1   CJ1
.........................................................gugagcaCaguuucaggugugu.................................   3      1   CJ1
.........................................................gugagcaGaguuucaggugugu.................................   28     1   CJ1
.........................................................gugagcaaaguuucaAgugugu.................................   7      1   CJ1
.........................................................gugagcaaGguuucaggugugu.................................   40     1   CJ1
.........................................................Cugagcaaaguuucaggugugu.................................   1      1   CJ1
.........................................................gugagcCaaguuucaggugugu.................................   1      1   CJ1
.........................................................gugagcaaaguuAcaggugugu.................................   2      1   CJ1
.........................................................gAgagcaaaguuucaggugugu.................................   1      1   CJ1
.........................................................gugaAcaaaguuucaggugugu.................................   8      1   CJ1
.........................................................gugagcaaaguuucaggugugu.................................   8095   0   CJ1
.........................................................gugagcaaaguuucCggugugu.................................   4      1   CJ1
.........................................................gugagcaaaguuucaggAgugu.................................   12     1   CJ1
.........................................................gugagcaaaAuuucaggugugu.................................   6      1   CJ1
.........................................................gugagcaaaguuucaggugugG.................................   142    1   CJ1
.........................................................gugaUcaaaguuucaggugugu.................................   3      1   CJ1
.........................................................gugagcaaUguuucaggugugu.................................   6      1   CJ1
.........................................................gugagcaaaguuucagAugugu.................................   6      1   CJ1
.........................................................gugagcaaaguuucaggugCgu.................................   24     1   CJ1
.........................................................gCgagcaaaguuucaggugugu.................................   23     1   CJ1
.........................................................gugGgcaaaguuucaggugugu.................................   31     1   CJ1
.........................................................gugagcaaaguuucagguguUu.................................   9      1   CJ1
.........................................................gugagUaaaguuucaggugugu.................................   16     1   CJ1
.........................................................gugagcaaaguuucGgggugugu.................................   46     1   CJ1
.........................................................gugagcaaaguAucaggugugu.................................   1      1   CJ1
.........................................................gugagcaaCguuucaggugugu.................................   3      1   CJ1
.........................................................gugagcaUaguuucaggugugu.................................   6      1   CJ1
.........................................................gugagcUaaguuucaggugugu.................................   12     1   CJ1
.........................................................Uugagcaaaguuucaggugugu.................................   6      1   CJ1
.........................................................gugagcaaagCuucaggugugu.................................   19     1   CJ1
.........................................................gugagcaaaguuucaggGgugu.................................   6      1   CJ1
.........................................................guCagcaaaguuucaggugugu.................................   13     1   CJ1
.........................................................gugagcaaaguuucaggCgugu.................................   35     1   CJ1
.........................................................gugagcaaaguuucagguUugu.................................   6      1   CJ1
.........................................................gugagcaaaguuGcaggugugu.................................   3      1   CJ1
.........................................................gugagcaaaguuucagguCugu.................................   3      1   CJ1
.........................................................gugUgcaaaguuucaggugugu.................................   3      1   CJ1
.........................................................gugagcaaaguuucagguAugu.................................   5      1   CJ1
.........................................................gugagcaaaCuuucaggugugu.................................   3      1   CJ1
.........................................................gugagcaaaguuucaggugugA.................................   324    1   CJ1
.........................................................gugagcGaaguuucaggugugu.................................   24     1   CJ1
.........................................................guUagcaaaguuucaggugugu.................................   22     1   CJ1
.........................................................gugagAaaaguuucaggugugu.................................   5      1   CJ1
.........................................................gugagcaaaguuCcaggugugu.................................   36     1   CJ1
.........................................................gugagcaaaguuucaggugGgu.................................   6      1   CJ1
.........................................................gugagcaaaguCucaggugugu.................................   12     1   CJ1
.........................................................gugagcaaagAuucaggugugu.................................   1      1   CJ1
.........................................................gugagcaaaguuucaUgugugu.................................   2      1   CJ1
.........................................................gugagcaaagGuucaggugugu.................................   4      1   CJ1
.........................................................gugagcaaaguuucagguguAu.................................   1      1   CJ1
.........................................................gugagcaaaguuucaggugugugc................................   21     0   CJ1
.........................................................gugagcaaaguuucaggugugugG................................   62     1   CJ1
.........................................................gugagcGaaguuucaggugugugc................................   1      1   CJ1
.........................................................gugagcaaaguuucagguguguGa...............................   1      1   CJ1
..........................................................ugagcaaaguuucaggugug..................................   1      0   CJ1
..........................................................ugagcaaaguuucaggugugu.................................   3      0   CJ1
...........................................................gagcaaaguuucaggugugu.................................   4      0   CJ1
............................................................agcaaaguuucaggugugu.................................   1      0   CJ1
.............................................................gcaaaguuucaggugugu.................................   1      0   CJ1

...................uccaCcugaaagcuugucucaacc.....................................................................   1      1   G01
....................ccaucugaaagcuugucu..........................................................................   1      0   G01
....................ccaucugaaagcuugucuc.........................................................................   5      0   G01
....................ccaucugaaagcuugucuca........................................................................   2      0   G01
....................ccaucugaaagcuugucucaac......................................................................   16     0   G01
....................ccaucugaaUgcuugucucaacc.....................................................................   1      1   G01
....................ccaucugaaagcuugucucaacG.....................................................................   1      1   G01
....................ccaucugaaagcuugucucaCcc.....................................................................   1      1   G01
....................ccaucugaaagcuugucucaacU.....................................................................   1      1   G01
....................ccaucugaaagcuugucucaacc.....................................................................   83     0   G01
```

```
                    Star                                    Mature
ugauaauuugcuucauugauccaucugaaagcuugucucaaccuuugaucugaacaggugagcaaaguuucaggugugucagagaaguuuauuuucaucuuauuaucaacua
....................ccaucugCaagcuugucucaacc.....................................................................  1     1   G01
....................ccaucugUaagcuugucucaaccu....................................................................  1     1   G01
....................ccaucCgaaagcuugucucaaccu....................................................................  1     1   G01
....................ccaucugaaagcuugucucaGccu....................................................................  1     1   G01
....................Ucaucugaaagcuugucucaaccu....................................................................  1     1   G01
....................ccaucugaaagcuugucucaaccu....................................................................  220   0   G01
....................ccaucugaaagcuCgucucaaccu....................................................................  1     1   G01
....................ccaucugaaagcuugucucaaUcu....................................................................  1     1   G01
....................ccaucugaaagcuugucucaaccA....................................................................  3     1   G01
....................ccaucugaaagcuugucucaaccC....................................................................  1     1   G01
....................ccaucugaaagcuugucucaaccuu...................................................................  1     0   G01
....................ccaucugaaagcuugucucaaccuA...................................................................  4     1   G01
....................ccaCcugaaagcuugucucaaccuuu..................................................................  3     1   G01
.....................cCucugaaagcuugucucaaccu....................................................................  1     1   G01
.....................caCcugaaagcuugucucaaccuuu..................................................................  2     1   G01
......................aucugaaagcuugucucaaccu....................................................................  1     0   G01
.......................ucugaaagcuugucucaaccu....................................................................  1     0   G01
.......................ucugaaagcuugucucaaccuA...................................................................  1     1   G01
........................cugaaagcuugucucaacc.....................................................................  1     0   G01
........................cugaaagcuugucucaaccu....................................................................  3     0   G01
.........................ugaaagcuugucucaacc.....................................................................  2     0   G01
.........................ugaaagcuugucucaaccu....................................................................  15    0   G01
.........................ugaaagcuugucucaaccuu...................................................................  2     0   G01
..........................gaaagcuugucucaaccu....................................................................  2     0   G01
.....................................................acCggugagcaaaguuucaggugugu.................................  1     1   G01
......................................................cGgugagcaaaguuucaggugugu.................................  2     1   G01
.......................................................aggugagcaaaguuucaggugug..................................  2     0   G01
.......................................................Cggugagcaaaguuucaggugugu.................................  1     1   G01
.......................................................Gggugagcaaaguuucaggugugu.................................  1     1   G01
.......................................................aggugagcaaaguuucaggugugu.................................  1     0   G01
.......................................................aAgugagcaaaguuucaggugugu.................................  1     1   G01
.......................................................aUgugagcaaaguuucaggugugu.................................  2     1   G01
........................................................ggugagcaaaguuucaggu.....................................  2     0   G01
........................................................Ugugagcaaaguuucaggug....................................  1     1   G01
........................................................ggugagcaaaguuucaggugu...................................  6     0   G01
........................................................Ugugagcaaaguuucaggugu...................................  4     1   G01
........................................................ggugagcaaaguuucaggugug..................................  13    0   G01
........................................................Agugagcaaaguuucaggugug..................................  1     1   G01
........................................................Ugugagcaaaguuucaggugug..................................  2     1   G01
........................................................ggugagcaaaguuucagguguU..................................  2     1   G01
........................................................Cgugagcaaaguuucaggugugu.................................  5     1   G01
........................................................ggugagcaaaguuucagguguAu.................................  2     1   G01
........................................................Ugugagcaaaguuucaggugugu.................................  127   1   G01
........................................................Agugagcaaaguuucaggugugu.................................  1     1   G01
........................................................ggGgagcaaaguuucaggugugu.................................  1     1   G01
........................................................ggugagcaaaguuucaggugugA.................................  1     1   G01
........................................................ggugagcaaaguuucaggugugu.................................  27    0   G01
........................................................ggugagcaaaguuucagguguUu.................................  1     1   G01
.........................................................gugagcaaaguuucGggu.....................................  2     1   G01
.........................................................gugagcaaaguuucCggu.....................................  3     1   G01
.........................................................gugagcaaaguuucaggA.....................................  11    1   G01
.........................................................gugagcaaaCuuucaggu.....................................  1     1   G01
.........................................................gugagcaUaguuucaggu.....................................  1     1   G01
.........................................................gugagcaaaguuucaggC.....................................  1     1   G01
.........................................................gugagcaaaguuucaggu.....................................  576   0   G01
.........................................................gugaAcaaaguuucaggu.....................................  1     1   G01
.........................................................gugagcaaaguuucagguA....................................  6     1   G01
.........................................................gugagcaaaguuucagguU....................................  3     1   G01
.........................................................gugagcaaagGuucaggug....................................  1     1   G01
.........................................................gugagcaaaguuucCggug....................................  5     1   G01
.........................................................gugagcaaaguuuUaggug....................................  1     1   G01
.........................................................gugagcaaaguuucaggug....................................  197   0   G01
.........................................................gugagcaaaguuucaggugu...................................  1944  0   G01
.........................................................gugaAcaaaguuucaggugu...................................  1     1   G01
.........................................................gugagcaaaguuucagguUu...................................  1     1   G01
.........................................................gugagcaaagCuucaggugu...................................  2     1   G01
.........................................................gugagcaaaguuucagguAu...................................  1     1   G01
.........................................................gugagcaaagGuucaggugu...................................  2     1   G01
.........................................................gugagcaaaguuuUaggugu...................................  2     1   G01
.........................................................gugagcaaaguuucGggugu...................................  6     1   G01
```

```
                    Star                                  Mature
ugauaauuugcuucauugauccaucugaaagcuugucucaaccuuugaucugaacaggugagcaaaguuucaggugugucagagaaguuuauuuucaucuuauuaucaacua
.........................................................gugUgcaaaguuucaggugu...................................  1     1  G01
.........................................................gAgagcaaaguuucaggugu...................................  2     1  G01
.........................................................gugagcaaCguuucaggugu...................................  1     1  G01
.........................................................gugagcaaaguuuGaggugu...................................  1     1  G01
.........................................................gugagcaaaguuGcaggugu...................................  1     1  G01
.........................................................gugagcaaaguuucUggugu...................................  4     1  G01
.........................................................gugagcaaaguuucaggugC...................................  2     1  G01
.........................................................gugagcaaGguuucaggugu...................................  1     1  G01
.........................................................gugagcaaaguuCcaggugu...................................  2     1  G01
.........................................................gugagcaaaguuucaggGgu...................................  2     1  G01
.........................................................gugagcaUaguuucaggugug..................................  3     1  G01
.........................................................gugagcaaaguuucagguUug..................................  1     1  G01
.........................................................gugagcaaaguuucagguAug..................................  1     1  G01
.........................................................gugagcaaaguuucagCugug..................................  2     1  G01
.........................................................gugagcaaaguuucagguguA..................................  20    1  G01
.........................................................gugagcaaaCuuucaggugug..................................  3     1  G01
.........................................................gugagcaaaguuuAaggugug..................................  1     1  G01
.........................................................gGgagcaaaguuucaggugug..................................  1     1  G01
.........................................................Uugagcaaaguuucaggugug..................................  2     1  G01
.........................................................gugagcaaaguuCcaggugug..................................  3     1  G01
.........................................................gugagcaaaguuucaggGgug..................................  2     1  G01
.........................................................gAgagcaaaguuucaggugug..................................  6     1  G01
.........................................................gugagUaaaguuucaggugug..................................  4     1  G01
.........................................................gugagcaaaguuucGggugug..................................  8     1  G01
.........................................................gugagcaaaguuucCggugug..................................  1     1  G01
.........................................................gugagcaaaguuucaggAgug..................................  1     1  G01
.........................................................gugagcaaaguuuUaggugug..................................  7     1  G01
.........................................................gugUgcaaaguuucaggugug..................................  1     1  G01
.........................................................gugagcaaaguuucagguguU..................................  651   1  G01
.........................................................gugagcaaagGuucaggugug..................................  1     1  G01
.........................................................gugagcaaaguuucaggugCg..................................  2     1  G01
.........................................................gugagcaaaguuucUggugug..................................  5     1  G01
.........................................................gugagcaaaguuucaggugug..................................  6919  0  G01
.........................................................gugagcaGaguuucaggugug..................................  3     1  G01
.........................................................gCgagcaaaguuucaggugug..................................  1     1  G01
.........................................................gugaUcaaaguuucaggugug..................................  1     1  G01
.........................................................gugagAaaaguuucaggugug..................................  1     1  G01
.........................................................gugagcaaaguuucagAugug..................................  1     1  G01
.........................................................gugGgcaaaguuucaggugug..................................  2     1  G01
.........................................................gugaAcaaaguuucaggugug..................................  1     1  G01
.........................................................gugagcaaaguuucaggugGg..................................  1     1  G01
.........................................................gugaCcaaaguuucaggugugu.................................  3     1  G01
.........................................................gugagcaaGguuucaggugugu.................................  3     1  G01
.........................................................gugagcaaaguuucaggAgugu.................................  2     1  G01
.........................................................gugagcaaaguuucagguguAu.................................  40    1  G01
.........................................................gugagcaaaguuucGggugugu.................................  190   1  G01
.........................................................gugGgcaaaguuucaggugugu.................................  28    1  G01
.........................................................gugagcaGaguuucaggugugu.................................  11    1  G01
.........................................................guCagcaaaguuucaggugugu.................................  5     1  G01
.........................................................gugagGaaaguuucaggugugu.................................  2     1  G01
.........................................................gugagcaaaguGucaggugugu.................................  9     1  G01
.........................................................gugagcaaaguuucaggugAgu.................................  6     1  G01
.........................................................gugagcaaaguuCcaggugugu.................................  40    1  G01
.........................................................gugagcUaaguuucaggugugu.................................  3     1  G01
.........................................................gGgagcaaaguuucaggugugu.................................  30    1  G01
.........................................................gugUgcaaaguuucaggugugu.................................  28    1  G01
.........................................................gugagcaaaguuucaggugGgu.................................  7     1  G01
.........................................................gugagcaaaguuucUggugugu.................................  77    1  G01
.........................................................gugagcaaaguuucagguguCu.................................  26    1  G01
.........................................................gugagcaaaguuucCggugugu.................................  10    1  G01
.........................................................gugagcaaaguuuAaggugugu.................................  5     1  G01
.........................................................gugagcaaaguuucagguguUu.................................  1214  1  G01
.........................................................gugagcaaUguuucaggugugu.................................  2     1  G01
.........................................................gugagcaaaguuucagguUugu.................................  9     1  G01
.........................................................gugagcaaaguuucagAugugu.................................  8     1  G01
.........................................................gugagcaaaguuAcaggugugu.................................  3     1  G01
.........................................................guAagcaaaguuucaggugugu.................................  5     1  G01
.........................................................Cugagcaaaguuucaggugugu.................................  1     1  G01
.........................................................gugagcaaCguuucaggugugu.................................  2     1  G01
.........................................................gugagcaaaguuuUaggugugu.................................  139   1  G01
```

```
                    Star                                Mature
ugauaauuugcuucauugauccaucugaaagcuugucucaaccuuugaucugaacaggugagcaaaguuucaggugugucagagaaguuuauuuucaucuuauuaucaacua
.........................................................gugagcaaaguCucaggugugu.................................   10      1   G01
.........................................................gCgagcaaaguuucaggugugu.................................   24      1   G01
.........................................................gugagcaaaCuuucaggugugu.................................   34      1   G01
.........................................................gugagcaUaguuucaggugugu.................................   24      1   G01
.........................................................gugaAcaaaguuucaggugugu.................................   30      1   G01
.........................................................gugagcaaagCuucaggugugu.................................   10      1   G01
.........................................................gugagcaaaguuucaggCgugu.................................   3       1   G01
.........................................................gugagcaaagGuucaggugugu.................................   38      1   G01
.........................................................gugagcaaaguuucaggugugG.................................   68      1   G01
.........................................................gugagAaaaguuucaggugugu.................................   6       1   G01
.........................................................gugagcaaaguuucaggugugu.................................   127925  0   G01
.........................................................gugagcaaaguuucaggugCgu.................................   13      1   G01
.........................................................gugagcaaaguuucaggGgugu.................................   15      1   G01
.........................................................gugagcaaaguuucaggugugA.................................   1236    1   G01
.........................................................gugagcaaaguuucagguAugu.................................   12      1   G01
.........................................................Augagcaaaguuucaggugugu.................................   3       1   G01
.........................................................gugagcCaaguuucaggugugu.................................   1       1   G01
.........................................................gugaUcaaaguuucaggugugu.................................   9       1   G01
.........................................................gugagcaaaguuGcaggugugu.................................   6       1   G01
.........................................................gugCgcaaaguuucaggugugu.................................   6       1   G01
.........................................................gugagcaaaguuucagUugugu.................................   18      1   G01
.........................................................gugagcaaaguuucaUgugugu.................................   17      1   G01
.........................................................gugagcaaaguuucagCugugu.................................   21      1   G01
.........................................................gugagUaaaguuucaggugugu.................................   77      1   G01
.........................................................gugagcGaaguuucaggugugu.................................   8       1   G01
.........................................................gAgagcaaaguuucaggugugu.................................   50      1   G01
.........................................................gugagcaaagAuucaggugugu.................................   2       1   G01
.........................................................gugagcaaaUuuucaggugugu.................................   2       1   G01
.........................................................Uugagcaaaguuucaggugugu.................................   30      1   G01
.........................................................gugagcaaaguuucaAgugugu.................................   14      1   G01
.........................................................guUagcaaaguuucaggugugu.................................   7       1   G01
.........................................................gugagcaaaguuucaCgugugu.................................   10      1   G01
.........................................................gugagcaaaguuucagguCugu.................................   9       1   G01
.........................................................gugagcaaaguuuGaggugugu.................................   24      1   G01
.........................................................gugagcaaaguuucaggugugucu................................   25      0   G01
.........................................................gugagcaaaguuucagguguguG................................   28      1   G01
.........................................................gugagcaaaguuucagguguguUa...............................   68      1   G01
.........................................................gugagcaaaguuucaggugugucC...............................   16      1   G01
.........................................................gugagcaaaguuucaggugugucа...............................   3       0   G01
.........................................................gugagcaaaguuucagguguguGa...............................   3       1   G01
.........................................................gugagcaaaguuucaggugugucU...............................   3       1   G01
.........................................................gugagcaaaguuucagguguguUag..............................   1       1   G01
.........................................................gugagcaaaguuucaggugugucUg..............................   1       1   G01
..........................................................ugagcaaaguuucaggugug..................................   1       0   G01
..........................................................Ggagcaaaguuucaggugugu.................................   2       1   G01
..........................................................ugagcaaaguuucaggugugu.................................   35      0   G01
..........................................................ugagcaaaguuucaggugugu c...............................   2       0   G01
...........................................................Cagcaaaguuucaggugu...................................   1       1   G01
...........................................................gagcaaaguuucaggugug..................................   4       0   G01
...........................................................gagcaaaguuucaggugugu.................................   83      0   G01
...........................................................gagcaaaguuucaggugugA.................................   1       1   G01
...........................................................Uagcaaaguuucaggugugu.................................   3       1   G01
...........................................................Cagcaaaguuucaggugugu.................................   1       1   G01
............................................................agcaaaguuucaggugug..................................   6       0   G01
............................................................agcaaaguuucaggugugu.................................   116     0   G01
............................................................Ggcaaaguuucaggugugu.................................   1       1   G01
............................................................agcaaaguuucaggugugA.................................   2       1   G01
.............................................................gcaaaguuuUaggugugu.................................   1       1   G01
.............................................................gcaaaguuucaggugugu.................................   95      0   G01
.............................................................gcaaaguuucaggugugA.................................   1       1   G01
.............................................................gcaaaguuucagguguguG................................   1       1   G01
..............................................................caaaguuucagguguguGag..............................   1       1   G01

....................ccaucugaaagcuugucuc.........................................................................   4       0   LJ1
....................ccaucugaaagcuugucucaaU......................................................................   2       1   LJ1
....................ccaucugaaagcuugucucaac......................................................................   9       0   LJ1
....................ccaucugaaagcuugucucaacA.....................................................................   2       1   LJ1
....................ccaucugaaagcuugucucaacc.....................................................................   6       0   LJ1
....................ccaucugaaagcuugucucaaccu....................................................................   14      0   LJ1
....................ccaucugaaagcuCgucucaaccu....................................................................   1       1   LJ1
```

Star **Mature**

```
ugauaauuugcuucauugauccaucugaaagcuugucucaaccuuugaucugaacaggugagcaaaguuucaggugugucagagaaguuuauuuucaucuuauuaucaacua
....................ccaCcugaaagcuugucucaaccuuu..................................................................   1   1   LJ1
.......................................................aAgugagcaaaguuucaggugugu.................................   1   1   LJ1
........................................................ggugagcaaaguuucagguA....................................   1   1   LJ1
........................................................ggugagcaaaguuucaggugug..................................   1   0   LJ1
........................................................Cgugagcaaaguuucaggugug..................................   3   1   LJ1
........................................................ggugagcaaaguuucaggugugu.................................   2   0   LJ1
........................................................ggugagcaaaguuucaggugugA.................................   1   1   LJ1
........................................................Cgugagcaaaguuucaggugugu.................................   6   1   LJ1
.........................................................gugagcaaaguuucaggC.....................................   5   1   LJ1
.........................................................gugGgcaaaguuucaggu.....................................   1   1   LJ1
.........................................................gugagcaaaguuucaggA.....................................   1   1   LJ1
.........................................................gugagcaaaguuucaAgu.....................................   1   1   LJ1
.........................................................gugagcaaaguuucaggu.....................................   33  0   LJ1
.........................................................gugagcaaaguuucaggAg....................................   1   1   LJ1
.........................................................gugagcaaGguuucaggug....................................   2   1   LJ1
.........................................................gugagcaaaguuucaggug....................................   144 0   LJ1
.........................................................guUagcaaaguuucaggug....................................   1   1   LJ1
.........................................................gugagcCaaguuucaggug....................................   1   1   LJ1
.........................................................gugagAaaaguuucaggug....................................   1   1   LJ1
.........................................................gugagcaaaguuucagguU....................................   7   1   LJ1
.........................................................gugagcaaaguuCcaggug....................................   1   1   LJ1
.........................................................gugagcaaaguuucaggCg....................................   1   1   LJ1
.........................................................gugagcaaaguuucagguA....................................   16  1   LJ1
.........................................................gugagcaaaguuucGggugu...................................   1   1   LJ1
.........................................................gugagcaaGguuucaggugu...................................   1   1   LJ1
.........................................................gugagcGaaguuucaggugu...................................   1   1   LJ1
.........................................................gugagcaGaguuucaggugu...................................   2   1   LJ1
.........................................................gugagcaaagCuucaggugu...................................   1   1   LJ1
.........................................................gugagcaaaguuCcaggugu...................................   1   1   LJ1
.........................................................gugGgcaaaguuucaggugu...................................   3   1   LJ1
.........................................................gCgagcaaaguuucaggugu...................................   1   1   LJ1
.........................................................gugagcaaaguuucaggugC...................................   30  1   LJ1
.........................................................gugagcaaaguuucaggugG...................................   12  1   LJ1
.........................................................gugagcaaaguCucaggugu...................................   2   1   LJ1
.........................................................gugagcaaaguuucaggGgu...................................   2   1   LJ1
.........................................................gugagcaaaguuucaggugu...................................   249 0   LJ1
.........................................................gugagcaUaguuucaggugu...................................   1   1   LJ1
.........................................................Uugagcaaaguuucaggugug..................................   3   1   LJ1
.........................................................gugagcaaagGuucaggugug..................................   4   1   LJ1
.........................................................gugagcaaaguuCcaggugug..................................   5   1   LJ1
.........................................................gugagcaaaguCucaggugug..................................   3   1   LJ1
.........................................................Augagcaaaguuucaggugug..................................   8   1   LJ1
.........................................................gugagcaaagCuucaggugug..................................   3   1   LJ1
.........................................................gGgagcaaaguuucaggugug..................................   1   1   LJ1
.........................................................gugagcCaaguuucaggugug..................................   1   1   LJ1
.........................................................gugagcaaaguuucaUgugug..................................   6   1   LJ1
.........................................................gugagcaaaguuucaggugGg..................................   4   1   LJ1
.........................................................gugagcaaaguuucagguguU..................................   49  1   LJ1
.........................................................gugagcaaaguuucagguguA..................................   200 1   LJ1
.........................................................gugagcaaaguuucaggugAg..................................   3   1   LJ1
.........................................................gugagcaaUguuucaggugug..................................   2   1   LJ1
.........................................................gugagcaaaguuuUaggugug..................................   2   1   LJ1
.........................................................gugagcaaGguuucaggugug..................................   5   1   LJ1
.........................................................gugaAcaaaguuucaggugug..................................   1   1   LJ1
.........................................................gugGgcaaaguuucaggugug..................................   7   1   LJ1
.........................................................gugagcaaaguuucaggCgug..................................   3   1   LJ1
.........................................................gugagcaaaAuuucaggugug..................................   1   1   LJ1
.........................................................gugagcaaaguuucaAgugug..................................   1   1   LJ1
.........................................................gugagUaaaguuucaggugug..................................   2   1   LJ1
.........................................................gugagcaaaguuucagguguC..................................   11  1   LJ1
.........................................................gugagcaaaguuucaggugug..................................   1724 0  LJ1
.........................................................gugagcaGaguuucaggugug..................................   4   1   LJ1
.........................................................gugagcaaaguuucGggugug..................................   7   1   LJ1
.........................................................gugagcGaaguuucaggugug..................................   7   1   LJ1
.........................................................gugagcaCaguuucaggugug..................................   1   1   LJ1
.........................................................gCgagcaaaguuucaggugug..................................   3   1   LJ1
.........................................................gugagcaaaguuucagguAug..................................   2   1   LJ1
.........................................................gugagcUaaguuucaggugug..................................   2   1   LJ1
.........................................................gugagcaaaguuucaggugCg..................................   6   1   LJ1
.........................................................guAagcaaaguuucaggugug..................................   5   1   LJ1
```

```
                    Star                                    Mature
ugauaauuugcuucauugauccaucugaaagcuugucucaaccuuugaucugaacaggugagcaaaguuucaggugugucagagaaguuuauuuucaucuuauuaucaacua
.........................................................guUagcaaaguuucaggugug..................................    6     1    LJ1
.........................................................gugagcaaaguuucagAugug..................................    1     1    LJ1
.........................................................gugagcaaaguuucaggGgug..................................    4     1    LJ1
.........................................................gugagcaaaguuucUggugug..................................    1     1    LJ1
.........................................................guCagcaaaguuucaggugug..................................    3     1    LJ1
.........................................................gugagcaaaguuAcaggugug..................................    1     1    LJ1
.........................................................gugagcaaCguuucaggugug..................................    1     1    LJ1
.........................................................gugagcaaaguuucaggugCgu.................................    32    1    LJ1
.........................................................gugagcaaaguuucagAugugu.................................    8     1    LJ1
.........................................................gugaAcaaaguuucaggugugu.................................    4     1    LJ1
.........................................................gugagcaaaguuucaggGgugu.................................    5     1    LJ1
.........................................................gugagcaaaguuucagguguUu.................................    15    1    LJ1
.........................................................gAgagcaaaguuucaggugugu.................................    4     1    LJ1
.........................................................gugagcaaaCuuucaggugugu.................................    1     1    LJ1
.........................................................Uugagcaaaguuucaggugugu.................................    3     1    LJ1
.........................................................gugGgcaaaguuucaggugugu.................................    29    1    LJ1
.........................................................gugagGaaaguuucaggugugu.................................    2     1    LJ1
.........................................................gugagcaaCguuucaggugugu.................................    1     1    LJ1
.........................................................guCagcaaaguuucaggugugu.................................    12    1    LJ1
.........................................................gugagcaaaguuucagguUugu.................................    1     1    LJ1
.........................................................gugagcUaaguuucaggugugu.................................    1     1    LJ1
.........................................................gugagcaaaguuuGaggugugu.................................    1     1    LJ1
.........................................................gugagcaaaguuucaggugugA.................................    311   1    LJ1
.........................................................gugagcaaaguuucagguCugu.................................    1     1    LJ1
.........................................................gugagcCaaguuucaggugugu.................................    4     1    LJ1
.........................................................gugagcaaaguuucaggugGgu.................................    7     1    LJ1
.........................................................gugagcaaaAuuucaggugugu.................................    3     1    LJ1
.........................................................gugagcaaaguuucaggugugG.................................    137   1    LJ1
.........................................................gugUgcaaaguuucaggugugu.................................    2     1    LJ1
.........................................................gugagcaaaguuucGggugugu.................................    26    1    LJ1
.........................................................gugagcaaaguuCcaggugugu.................................    15    1    LJ1
.........................................................Augagcaaaguuucaggugugu.................................    8     1    LJ1
.........................................................gugagcaaaguuAcaggugugu.................................    3     1    LJ1
.........................................................gugagcaaaguCucaggugugu.................................    21    1    LJ1
.........................................................guAagcaaaguuucaggugugu.................................    37    1    LJ1
.........................................................gugCgcaaaguuucaggugugu.................................    1     1    LJ1
.........................................................gugagcaaaguuuAaggugugu.................................    3     1    LJ1
.........................................................gugagcaaaguuGcaggugugu.................................    2     1    LJ1
.........................................................gugagUaaaguuucaggugugu.................................    16    1    LJ1
.........................................................gugagcaGaguuucaggugugu.................................    31    1    LJ1
.........................................................gugagcaaaguuucagguguAu.................................    6     1    LJ1
.........................................................gugagcaaaguuuUaggugugu.................................    4     1    LJ1
.........................................................gugagcaaaguAucaggugugu.................................    1     1    LJ1
.........................................................guUagcaaaguuucaggugugu.................................    22    1    LJ1
.........................................................gugagcaaaguuucaggCgugu.................................    28    1    LJ1
.........................................................gugagcaaaguuucaggAgugu.................................    3     1    LJ1
.........................................................gugagcaaaguuucaggugAgu.................................    4     1    LJ1
.........................................................gugagcaaGguuucaggugugu.................................    41    1    LJ1
.........................................................gugagcaaaguuucCggugugu.................................    1     1    LJ1
.........................................................gugagAaaaguuucaggugugu.................................    5     1    LJ1
.........................................................gugagcaaaUuuucaggugugu.................................    2     1    LJ1
.........................................................gCgagcaaaguuucaggugugu.................................    16    1    LJ1
.........................................................gugagcaaaguuucUggugugu.................................    5     1    LJ1
.........................................................gugagcaaagAuucaggugugu.................................    2     1    LJ1
.........................................................gugagcaaaguuucaAgugugu.................................    7     1    LJ1
.........................................................gugagcaaaguuucaggugugu.................................    7615  0    LJ1
.........................................................gugaUcaaaguuucaggugugu.................................    2     1    LJ1
.........................................................gugagcaUaguuucaggugugu.................................    4     1    LJ1
.........................................................gugagcaaaguuucagguguCu.................................    6     1    LJ1
.........................................................gugagcGaaguuucaggugugu.................................    31    1    LJ1
.........................................................gugagcaaagCuucaggugugu.................................    16    1    LJ1
.........................................................gugagcaaaguuucagguAugu.................................    1     1    LJ1
.........................................................gugagcaaaguuucagguguguc................................    30    0    LJ1
.........................................................gugagcaaaguuucagguguguG................................    76    1    LJ1
.........................................................gugagcaaaguuucagguguguUa...............................    1     1    LJ1
.........................................................gugagcaaaguuucaggugugucUg..............................    1     1    LJ1
..........................................................ugagcaaaguuucaggugugu.................................    4     0    LJ1
..........................................................ugagcaaaguuucagguguguc................................    1     0    LJ1

....................ccaucugaaagcuugucuca........................................................................    7     0    TA1
```

Star / Mature

```
                    Star                              Mature
ugauaauuugcuucauugauccaucugaaagcuugucucaaccuuugaucugaacaggugagcaaaguuucaggugugucagagaaguuuauuuucaucuuauuaucaacua
....................ccaucugaaagcuugucucaa.......................................................................   3     0     TA1
....................ccaucAgaaagcuugucucaac......................................................................   1     1     TA1
....................ccaucugaaagcuugucucaac......................................................................   26    0     TA1
....................ccaucugaaagcuugucucaaU......................................................................   2     1     TA1
....................ccaucugaaagcuugCcucaac......................................................................   1     1     TA1
....................ccaucugaaagcuugucucaaA......................................................................   1     1     TA1
....................ccaucugaaagcuugucucaacU.....................................................................   6     1     TA1
....................ccaucugaaagcuugucucaacA.....................................................................   3     1     TA1
....................ccaucCgaaagcuugucucaacc.....................................................................   1     1     TA1
....................ccaucugaaagcuugCcucaacc.....................................................................   1     1     TA1
....................ccaucugaaagcuugucucaacc.....................................................................   23    0     TA1
....................ccaGcugaaagcuugucucaacc.....................................................................   3     1     TA1
....................ccaucugaaagcuugucucaaccC....................................................................   6     1     TA1
....................ccaucugaGagcuugucucaaccu....................................................................   1     1     TA1
....................ccaucugaaagcuugucCcaaccu....................................................................   1     1     TA1
....................ccaucugaaagcuugucucaGccu....................................................................   4     1     TA1
....................ccaucuAaaagcuugucucaaccu....................................................................   1     1     TA1
....................ccaucugaaagcuugucucaaccu....................................................................   78    0     TA1
....................ccaucugaaagcuugCcucaaccu....................................................................   1     1     TA1
....................ccaucugaaUgcuugucucaaccu....................................................................   1     1     TA1
....................ccaucugaaagcuugucucaaccG....................................................................   2     1     TA1
....................ccaucugaaagcuugucucaaccA....................................................................   6     1     TA1
....................ccaucugaaagUuugucucaaccu....................................................................   1     1     TA1
....................ccaucugaaaAcuugucucaaccu....................................................................   1     1     TA1
....................ccaCcugaaagcuugucucaaccuuu..................................................................   1     1     TA1
........................................................ggugagcaaaguuucagguguA..................................   1     1     TA1
........................................................ggugagcaaaguuucaggugug..................................   1     0     TA1
........................................................Cgugagcaaaguuucaggugug..................................   4     1     TA1
........................................................ggugagcaaaguuCcaggugugu.................................   1     1     TA1
........................................................Cgugagcaaaguuucaggugugu.................................   8     1     TA1
........................................................ggugagcaaaguuucaggugugu.................................   2     0     TA1
.........................................................gugagcaaaguuucaggu.....................................   23    0     TA1
.........................................................gugagcaaaguuucaggGg....................................   1     1     TA1
.........................................................gugagcaaaguCucaggug....................................   2     1     TA1
.........................................................gugagcaaGguuucaggug....................................   2     1     TA1
.........................................................gugagcaaaguuCcaggug....................................   1     1     TA1
.........................................................gugagcaaCguuucaggug....................................   1     1     TA1
.........................................................gugagcaaaguuucaggug....................................   64    0     TA1
.........................................................gugagcaaaguuucagguA....................................   4     1     TA1
.........................................................gugagcaaaguuucGggugu...................................   2     1     TA1
.........................................................gugagcaaaguuucaggugG...................................   1     1     TA1
.........................................................gugGgcaaaguuucaggugu...................................   1     1     TA1
.........................................................gugagcaaaguuucaggGgu...................................   2     1     TA1
.........................................................gugagcaaaguuucaggugC...................................   14    1     TA1
.........................................................Cugagcaaaguuucaggugu...................................   1     1     TA1
.........................................................gugagcaaaguCucaggugu...................................   1     1     TA1
.........................................................gugagcaaaguuucaggugu...................................   117   0     TA1
.........................................................gCgagcaaaguuucaggugug..................................   4     1     TA1
.........................................................gugagcaUaguuucaggugug..................................   2     1     TA1
.........................................................gugagcaaaguuucaggCgug..................................   4     1     TA1
.........................................................Augagcaaaguuucaggugug..................................   1     1     TA1
.........................................................gugagcaaaguuucaggugCg..................................   5     1     TA1
.........................................................gugagcaaaguuucagguguU..................................   11    1     TA1
.........................................................gugagcaaaguuucagAugug..................................   2     1     TA1
.........................................................gugagAaaaguuucaggugug..................................   2     1     TA1
.........................................................gugagcaaaguuucaUgugug..................................   1     1     TA1
.........................................................gugagcaGaguuucaggugug..................................   4     1     TA1
.........................................................gugagUaaaguuucaggugug..................................   1     1     TA1
.........................................................gugagcaaGguuucaggugug..................................   5     1     TA1
.........................................................gugagcaaaguuucaggugAg..................................   3     1     TA1
.........................................................gugCgcaaaguuucaggugug..................................   1     1     TA1
.........................................................gugagcaaaguuucUggugug..................................   4     1     TA1
.........................................................gugGgcaaaguuucaggugug..................................   4     1     TA1
.........................................................gugagcaaaguuucGggugug..................................   4     1     TA1
.........................................................gugagcaaaguuucagguguC..................................   3     1     TA1
.........................................................gugagcaaaguuucagguUug..................................   1     1     TA1
.........................................................gugagcaaaguuucagguguA..................................   78    1     TA1
.........................................................gugagcaaagGuucaggugug..................................   1     1     TA1
.........................................................gugagcaaaguCucaggugug..................................   7     1     TA1
.........................................................gugagcaaUguuucaggugug..................................   1     1     TA1
```

Star Mature

```
ugauaauuugcuucauugauccaucugaaagcuugucucaaccuuugaucugaacaggugagcaaaguuucaggugugucagagaaguuuauuuucaucuuauuaucaacua
.........................................................gGgagcaaaguuucaggugug..................................  1      1  TA1
.........................................................gugagcaaaguGucaggugug..................................  1      1  TA1
.........................................................gugagcGaaguuucaggugug..................................  3      1  TA1
.........................................................gugagcaaagCuucaggugug..................................  3      1  TA1
.........................................................gugagcaCaguuucaggugug..................................  1      1  TA1
.........................................................gugagcaaaguuucaggugug..................................  1006   0  TA1
.........................................................gugagcaaaguuucaggugGg..................................  2      1  TA1
.........................................................gugagcaaagAuucaggugug..................................  1      1  TA1
.........................................................gugagcaUaguuucaggugugu.................................  13     1  TA1
.........................................................gugagcaaaguuucaggugGgu.................................  25     1  TA1
.........................................................gugagcaaaguuucagguguAu.................................  14     1  TA1
.........................................................gugagcaaaguuGcaggugugu.................................  1      1  TA1
.........................................................gGgagcaaaguuucaggugugu.................................  6      1  TA1
.........................................................gugGgcaaaguuucaggugugu.................................  49     1  TA1
.........................................................gugagcaaaguuucagCugugu.................................  1      1  TA1
.........................................................gugagcaaaguuucaggugugG.................................  258    1  TA1
.........................................................gugagcaaaguuucagguCugu.................................  2      1  TA1
.........................................................gugagcaaaguuucagAugugu.................................  13     1  TA1
.........................................................Augagcaaaguuucaggugugu.................................  20     1  TA1
.........................................................gugagcaGaguuucaggugugu.................................  62     1  TA1
.........................................................gugagcaaaUuuucaggugugu.................................  1      1  TA1
.........................................................gugagcaaaguGucaggugugu.................................  1      1  TA1
.........................................................gugagAaaaguuucaggugugu.................................  3      1  TA1
.........................................................Cugagcaaaguuucaggugugu.................................  1      1  TA1
.........................................................gugagcaaaguuucCggugugu.................................  3      1  TA1
.........................................................gugCgcaaaguuucaggugugu.................................  2      1  TA1
.........................................................gugaUcaaaguuucaggugugu.................................  8      1  TA1
.........................................................gugagcaaaguuucaggugugA.................................  490    1  TA1
.........................................................gugaAcaaaguuucaggugugu.................................  12     1  TA1
.........................................................gugagcaaaguuCcaggugugu.................................  76     1  TA1
.........................................................gugagcaaaAuuucaggugugu.................................  10     1  TA1
.........................................................gugagcaaaguuucaggugAgu.................................  15     1  TA1
.........................................................gugagcaaaguuucUggugugu.................................  14     1  TA1
.........................................................gugagcaaaguuucaggCgugu.................................  54     1  TA1
.........................................................gugagcaaUguuucaggugugu.................................  3      1  TA1
.........................................................Uugagcaaaguuucaggugugu.................................  2      1  TA1
.........................................................gugagcaaaguuuUaggugugu.................................  12     1  TA1
.........................................................gugagcaaagCuucaggugugu.................................  44     1  TA1
.........................................................gAgagcaaaguuucaggugugu.................................  12     1  TA1
.........................................................gugUgcaaaguuucaggugugu.................................  15     1  TA1
.........................................................gugagcaaaguuAcaggugugu.................................  6      1  TA1
.........................................................gugagcaaagGuucaggugugu.................................  6      1  TA1
.........................................................gugagcaaaguuucagguguUu.................................  7      1  TA1
.........................................................gugagcaCaguuucaggugugu.................................  2      1  TA1
.........................................................gugagcaaaguuucagguguCu.................................  2      1  TA1
.........................................................gugagcaaaguuuGaggugugu.................................  1      1  TA1
.........................................................gugagcaaaguuucaCgugugu.................................  1      1  TA1
.........................................................gugagcaaaguuucaggugugu.................................  14761  0  TA1
.........................................................gugaCcaaaguuucaggugugu.................................  1      1  TA1
.........................................................gugagcaaaguuucaggAgugu.................................  13     1  TA1
.........................................................gugagcaaaguuucaAgugugu.................................  10     1  TA1
.........................................................gugagcaaagAuucaggugugu.................................  4      1  TA1
.........................................................gugagcaaaguuucaggGgugu.................................  19     1  TA1
.........................................................guUagcaaaguuucaggugugu.................................  1      1  TA1
.........................................................gugagcaaaguCucaggugugu.................................  57     1  TA1
.........................................................gugagcaaaguAucaggugugu.................................  5      1  TA1
.........................................................gugagcaaaguuucaggugCgu.................................  40     1  TA1
.........................................................gCgagcaaaguuucaggugugu.................................  44     1  TA1
.........................................................gugagcCaaguuucaggugugu.................................  2      1  TA1
.........................................................gugagcUaaguuucaggugugu.................................  3      1  TA1
.........................................................gugagcaaaguuucGggugugu.................................  68     1  TA1
.........................................................guAagcaaaguuucaggugugu.................................  5      1  TA1
.........................................................gugagcaaGguuucaggugugu.................................  62     1  TA1
.........................................................gugagcaaCguuucaggugugu.................................  4      1  TA1
.........................................................gugagcaaaguuuAaggugugu.................................  1      1  TA1
.........................................................gugagcGaaguuucaggugugu.................................  44     1  TA1
.........................................................gugagUaaaguuucaggugugu.................................  21     1  TA1
.........................................................gugagcaaaguuucagguAugu.................................  15     1  TA1
.........................................................gugagcaaaguCucagguguguc................................  1      1  TA1
.........................................................gugagcaaaguuucagguguguc................................  24     0  TA1
```

```
                    Star                               Mature
ugauaauuugcuucauugauccaucugaaagcuugucucaaccuuugaucugaacaggugagcaaaguuucaggugugucagagaaguuuauuuucaucuuauuaucaacua
.........................................................gugagcaaaguuucagguguguG................................  29   1   TA1
.........................................................gugagcaaaguuucaggugCguc................................  2    1   TA1
.........................................................gugGgcaaaguuucagguguguc................................  1    1   TA1
.........................................................gugagcaaaguuucagguguguGa...............................  2    1   TA1
.........................................................gugagcaaaguuucagguguguUa...............................  6    1   TA1
..........................................................ugagcaaaguuucaggugugu.................................  3    0   TA1
..........................................................ugagcGaaguuucaggugugu.................................  1    1   TA1
...........................................................gagcaaaguuucaggugugu.................................  4    0   TA1
.............................................................gcaaaguuucaggugugu.................................  1    0   TA1

....................ccaucugaaagcuugucuc.........................................................................  1    0   E01
....................ccaucugaaagcuugucuca........................................................................  2    0   E01
....................ccaucugaaagcuugucucaac......................................................................  7    0   E01
....................ccaucugaaagcuuAucucaac......................................................................  1    1   E01
....................ccaucugaaagcuugucucaacc.....................................................................  40   0   E01
....................Gcaucugaaagcuugucucaacc.....................................................................  1    1   E01
....................ccaucugaaUgcuugucucaaccu....................................................................  1    1   E01
....................ccaucugGaagcuugucucaaccu....................................................................  1    1   E01
....................ccaucugaaCgcuugucucaaccu....................................................................  1    1   E01
....................ccaucugaaagcuugucucaaccu....................................................................  387  0   E01
....................ccaucugaGagcuugucucaaccu....................................................................  1    1   E01
....................ccaucugUaagcuugucucaaccu....................................................................  3    1   E01
....................ccaucugaUagcuugucucaaccu....................................................................  2    1   E01
....................ccaucugaaagcuugucucaaccC....................................................................  1    1   E01
....................ccaucugaaagcuugucucaaccuu...................................................................  4    0   E01
....................ccaCcugaaagcuugucucaaccuuu..................................................................  8    1   E01
....................ccaucugaaagcuugucucaaccuuug.................................................................  1    0   E01
.....................caucugaaagcuugucucaaccu....................................................................  2    0   E01
.....................caCcugaaagcuugucucaaccuuu..................................................................  8    1   E01
.......................ucugaaagcuugucucaaccuu...................................................................  1    0   E01
........................cugaaagcuugucucaaccu....................................................................  2    0   E01
.........................ugaaagcuugucucaacc.....................................................................  1    0   E01
.........................ugaaagcuugucucaaccu....................................................................  9    0   E01
.........................ugaaagcuugucucaaccuu...................................................................  1    0   E01
..........................gaaagcuugucucaaccu....................................................................  2    0   E01
......................................................cGggugagcaaaguuucaggugugu.................................  1    1   E01
.......................................................aggugagcaaaguuucaggugu...................................  1    0   E01
.......................................................aggugagcaaaguuucaggugugu.................................  1    0   E01
.......................................................Gggugagcaaaguuucaggugugu.................................  1    1   E01
........................................................ggugagcaaaguuucagg......................................  1    0   E01
........................................................Ugugagcaaaguuucaggu.....................................  1    1   E01
........................................................ggugagcaaaguuucaggugu...................................  15   0   E01
........................................................Ugugagcaaaguuucaggugug..................................  2    1   E01
........................................................ggugagcaaaguuucaggugug..................................  93   0   E01
........................................................ggugagcaaaguuucagguguU..................................  14   1   E01
........................................................Cgugagcaaaguuucaggugugu.................................  4    1   E01
........................................................ggugagcaaaguuucaggugugu.................................  237  0   E01
........................................................gUugagcaaaguuucaggugugu.................................  2    1   E01
........................................................Agugagcaaaguuucaggugugu.................................  2    1   E01
........................................................ggugaAcaaaguuucaggugugu.................................  1    1   E01
........................................................ggugagcaaaguuucaggugugA.................................  8    1   E01
........................................................Ugugagcaaaguuucaggugugu.................................  119  1   E01
........................................................ggugagcaaaguuucaggugugUu................................  3    1   E01
.........................................................gugagcaaaguuucUggu.....................................  1    1   E01
.........................................................gugagcaaaguuucaggu.....................................  265  0   E01
.........................................................gugagcaaaguuucCggug....................................  1    1   E01
.........................................................gugagcaaaguuucagguC....................................  2    1   E01
.........................................................gugagcaaaguuucaggug....................................  120  0   E01
.........................................................gugagcaaaguuucUggugu...................................  2    1   E01
.........................................................gugagcaaaguuAcaggugu...................................  1    1   E01
.........................................................gugagcaaaguuucaggGgu...................................  1    1   E01
.........................................................guCagcaaaguuucaggugu...................................  1    1   E01
.........................................................gugagcaaaguuucaggCgu...................................  1    1   E01
.........................................................gugagcaaaguuucaggugu...................................  949  0   E01
.........................................................gugagcaaaguuucaUgugu...................................  2    1   E01
.........................................................gugagcaaaguuucGggugu...................................  1    1   E01
.........................................................gugagcaaaguuucagguUu...................................  1    1   E01
.........................................................gAgagcaaaguuucaggugu...................................  1    1   E01
.........................................................gugagcaaaguuucagCugug..................................  1    1   E01
.........................................................gugagcaaaguuucaggugAg..................................  3    1   E01
```

```
                    Star                                 Mature
ugauaauuugcuucauugauccaucugaaagcuugucucaaccuuugaucugaacaggugagcaaaguuucaggugugucagagaaguuuauuuucaucuuauuaucaacua
.........................................................gugagcaaaCuuucaggugug..................................	2	1	E01
.........................................................gugagcaaaguuucaUgugug..................................	1	1	E01
.........................................................gugagcUaaguuucaggugug..................................	1	1	E01
.........................................................gugagcaaaguuucUggugug..................................	2	1	E01
.........................................................gugUgcaaaguuucaggugug..................................	1	1	E01
.........................................................gAgagcaaaguuucaggugug..................................	4	1	E01
.........................................................guUagcaaaguuucaggugug..................................	1	1	E01
.........................................................gugagcaaaguuucaggugCg..................................	2	1	E01
.........................................................guCagcaaaguuucaggugug..................................	1	1	E01
.........................................................gugagcaaaguuCcaggugug..................................	3	1	E01
.........................................................gugCgcaaaguuucaggugug..................................	1	1	E01
.........................................................gugagcaaaUuuucaggugug..................................	1	1	E01
.........................................................gugagcaaGguuucaggugug..................................	1	1	E01
.........................................................gugagcaaaguuucagguguU..................................	255	1	E01
.........................................................gugagcaaaguuucaAgugug..................................	1	1	E01
.........................................................gugagcaaaguuuGaggugug..................................	1	1	E01
.........................................................gugagcaaaguuucagguguC..................................	3	1	E01
.........................................................gugagcaaaguuuUaggugug..................................	4	1	E01
.........................................................gugaAcaaaguuucaggugug..................................	1	1	E01
.........................................................gugGgcaaaguuucaggugug..................................	2	1	E01
.........................................................gugagcaaaguuucagguguA..................................	9	1	E01
.........................................................gugagcaaaguuucaggugug..................................	6737	0	E01
.........................................................gCgagcaaaguuucaggugug..................................	1	1	E01
.........................................................gugagcaaagGuucaggugug..................................	1	1	E01
.........................................................gugagcaaaguuucaggGgug..................................	1	1	E01
.........................................................gugagcaaCguuucaggugug..................................	1	1	E01
.........................................................gugagcaUaguuucaggugug..................................	1	1	E01
.........................................................gGgagcaaaguuucaggugug..................................	1	1	E01
.........................................................gugagUaaaguuucaggugug..................................	4	1	E01
.........................................................gugagcaaaguuucGggugug..................................	3	1	E01
.........................................................Uugagcaaaguuucaggugugu.................................	36	1	E01
.........................................................gugagcaaaguuucaUgugugu.................................	31	1	E01
.........................................................gugagGaaaguuucaggugugu.................................	5	1	E01
.........................................................gugagAaaaguuucaggugugu.................................	9	1	E01
.........................................................gugagcaaaguuucaCgugugu.................................	11	1	E01
.........................................................gugaAcaaaguuucaggugugu.................................	56	1	E01
.........................................................gugagcaaaUuuucaggugugu.................................	10	1	E01
.........................................................gugagcaUaguuucaggugugu.................................	46	1	E01
.........................................................gugagcaaaCuuucaggugugu.................................	38	1	E01
.........................................................gugagcaaaguuucaggugugA.................................	1256	1	E01
.........................................................gugagcaaaguuucaggugugG.................................	90	1	E01
.........................................................gCgagcaaaguuucaggugugu.................................	27	1	E01
.........................................................gugagcaaaguuuUaggugugu.................................	104	1	E01
.........................................................gugagcaCaguuucaggugugu.................................	2	1	E01
.........................................................gugagcaaaguuucaggugCgu.................................	18	1	E01
.........................................................guCagcaaaguuucaggugugu.................................	15	1	E01
.........................................................gugagcaaaguuucaggugGgu.................................	10	1	E01
.........................................................gugagcaaaguuucagguguUu.................................	584	1	E01
.........................................................gugagcaaaguuucaggCgugu.................................	17	1	E01
.........................................................gugagcaaaguuucaAgugugu.................................	13	1	E01
.........................................................gugagcaaaguuucagguCugu.................................	4	1	E01
.........................................................gugagcaaaguuucaggugugu.................................	202186	0	E01
.........................................................gugagcaGaguuucaggugugu.................................	18	1	E01
.........................................................gugagcaaaguuucagguAugu.................................	17	1	E01
.........................................................gAgagcaaaguuucaggugugu.................................	101	1	E01
.........................................................gugagcaaaguuuAaggugugu.................................	14	1	E01
.........................................................guUagcaaaguuucaggugugu.................................	20	1	E01
.........................................................Cugagcaaaguuucaggugugu.................................	4	1	E01
.........................................................guAagcaaaguuucaggugugu.................................	10	1	E01
.........................................................gugagcaaaguuucUggugugu.................................	174	1	E01
.........................................................gugagcaaaguuCcaggugugu.................................	46	1	E01
.........................................................gugagcaaaguuGcaggugugu.................................	15	1	E01
.........................................................gugagcaaaguuucaggGgugu.................................	23	1	E01
.........................................................gugagcaaaguuucagguguCu.................................	88	1	E01
.........................................................gugagcaaCguuucaggugugu.................................	1	1	E01
.........................................................gugagcaaagCuucaggugugu.................................	19	1	E01
.........................................................gugagcaaaguuucaggAgugu.................................	5	1	E01
.........................................................gugagcaaaguuucagguguAu.................................	46	1	E01
.........................................................gugagcaaagAuucaggugugu.................................	5	1	E01
.........................................................gGgagcaaaguuucaggugugu.................................	52	1	E01
```

```
                    Star                                Mature
ugauaauuugcuucauugauccaucugaaagcuugucucaaccuuugaucugaacaggugagcaaaguuucaggugugucagagaaguuuauuuucaucuuauuaucaacua
.........................................................gugaCcaaaguuucaggugugu.................................	6	1	E01
.........................................................gugagcaaaguuucagAugugu.................................	16	1	E01
.........................................................gugagUaaaguuucaggugugu.................................	79	1	E01
.........................................................gugagcaaaAuuucaggugugu.................................	2	1	E01
.........................................................Augagcaaaguuucaggugugu.................................	2	1	E01
.........................................................gugagcGaaguuucaggugugu.................................	12	1	E01
.........................................................gugagcaaUguuucaggugugu.................................	5	1	E01
.........................................................gugagcaaaguuucagguUugu.................................	15	1	E01
.........................................................gugUgcaaaguuucaggugugu.................................	38	1	E01
.........................................................gugagcaaaguuucGggugugu.................................	257	1	E01
.........................................................gugagcaaGguuucaggugugu.................................	7	1	E01
.........................................................gugGgcaaaguuucaggugugu.................................	39	1	E01
.........................................................gugagcaaaguCucaggugugu.................................	8	1	E01
.........................................................gugagcaaaguAucaggugugu.................................	2	1	E01
.........................................................gugagcaaagGuucaggugugu.................................	11	1	E01
.........................................................gugagcaaaguuucaggugAgu.................................	11	1	E01
.........................................................gugagcaaaguuucagUugugu.................................	28	1	E01
.........................................................gugagcaaaguGucaggugugu.................................	2	1	E01
.........................................................gugagcaaaguuAcaggugugu.................................	8	1	E01
.........................................................gugCgcaaaguuucaggugugu.................................	10	1	E01
.........................................................gugagcUaaguuucaggugugu.................................	8	1	E01
.........................................................gugagcaaaguuuGaggugugu.................................	17	1	E01
.........................................................gugagcaaaguuucagCugugu.................................	29	1	E01
.........................................................gugaUcaaaguuucaggugugu.................................	15	1	E01
.........................................................gugagcaaaguuucCggugugu.................................	18	1	E01
.........................................................gugagcaaaguuucaggugugAc................................	1	1	E01
.........................................................gugagcaaaguuucagguguguc................................	20	0	E01
.........................................................gugagcaaaguuucaggugugguG................................	41	1	E01
.........................................................gugagcaaaguuucaggugugCc................................	1	1	E01
.........................................................gugagUaaaguuucagguguguc................................	2	1	E01
.........................................................gugagcaaaguuucaggugugucC...............................	5	1	E01
.........................................................gugagcaaaguuucaggugugucA...............................	3	0	E01
.........................................................gugagcaaaguuucagguguguGa...............................	4	1	E01
.........................................................gugagcaaaguuucaggugugucU...............................	3	1	E01
.........................................................gugagcaaaguuucagguguguUa...............................	23	1	E01
.........................................................gugagcaaaguuucagguguguGag..............................	1	1	E01
..........................................................ugagcaaaguuucaggugu...................................	1	0	E01
..........................................................ugagcaaaguuucaggugugu.................................	46	0	E01
..........................................................Ggagcaaaguuucaggugugu.................................	1	1	E01
..........................................................ugagcaaaguuucagguguguG................................	1	1	E01
...........................................................gagcaaaguuucaggugug..................................	4	0	E01
...........................................................gagcaaaguuucaggugugA.................................	2	1	E01
...........................................................Uagcaaaguuucaggugugu.................................	1	1	E01
...........................................................gUgcaaaguuucaggugugu.................................	1	1	E01
...........................................................gagcUaaguuucaggugugu.................................	1	1	E01
...........................................................gagcaaaguuucaggugugu.................................	152	0	E01
...........................................................gagAaaaguuucaggugugu.................................	1	1	E01
...........................................................gGgcaaaguuucaggugugu.................................	1	1	E01
............................................................agcaaaguuucagguguU..................................	2	1	E01
............................................................agcaaaguuucaggugug..................................	2	0	E01
............................................................agcaaaguuucaggugugu.................................	220	0	E01
............................................................agcaaaguuucaggugugA.................................	1	1	E01
.............................................................gcaaaguuucaggugugu.................................	203	0	E01
.............................................................gcaaaguuucaggugugA.................................	2	1	E01

...................uccaCcugaaagcuugucucaaccu....................................................................	1	1	2nd
....................ccaucugaaagcuugucuc.........................................................................	2	0	2nd
....................ccaucugaaagcuugucuca........................................................................	8	0	2nd
....................ccaucugaaagcuugucucaa.......................................................................	1	0	2nd
....................ccaucugaaagcuugucucaac......................................................................	5	0	2nd
....................ccaucugaaagcuugucucaacA.....................................................................	1	1	2nd
....................ccaucugaaagcuugucucaacc.....................................................................	33	0	2nd
....................ccaucugaaagcuugucucaaccC....................................................................	1	1	2nd
....................ccaucugaaagcuugucucUaccu....................................................................	2	1	2nd
....................ccaucuAaaagcuugucucaaccu....................................................................	1	1	2nd
....................ccaucugaaagcuugucucaaccu....................................................................	124	0	2nd
....................ccaucugaaagcuugGcucaaccu....................................................................	1	1	2nd
....................ccaucugaaagcuugucucaaccA....................................................................	1	1	2nd
....................ccaucugaaagcuugucucaaccuu...................................................................	1	0	2nd
....................ccaCcugaaagcuugucucaaccuuu..................................................................	9	1	2nd
```

Star Mature

ugauaauuugcuucauugauccaucugaaagcuugucucaaccuuugaucugaacaggugagcaaaguuucaggugugucagagaaguuuauuuucaucuuauuaucaacua			
.....................caCcugaaagcuugucucaaccuuu..	5	1	2nd
........................cugaaagcuugucucaaccu..	1	0	2nd
.........................ugaaagcuugucucaacc...	1	0	2nd
.........................ugaaagcuugucucaaccu..	8	0	2nd
..........................gaaagcuugucucaaccu..	1	0	2nd
....cCggugagcaaaguuucaggugugu.................................	1	1	2nd
....cUggugagcaaaguuucaggugugu.................................	2	1	2nd
....caAgugagcaaaguuucaggugugu.................................	1	1	2nd
....caCgugagcaaaguuucaggugugu.................................	1	1	2nd
....Uggugagcaaaguuucagg......................................	1	1	2nd
....Uggugagcaaaguuucaggugug..................................	2	1	2nd
....Gggugagcaaaguuucaggugugu.................................	2	1	2nd
....aCgugagcaaaguuucaggugugu.................................	1	1	2nd
....ggugagcaaaguuucaggu.....................................	2	0	2nd
....Ugugagcaaaguuucaggu.....................................	1	1	2nd
....ggugagcaaaguuucaggug....................................	1	0	2nd
....ggugagcaaaguuucaggugu...................................	23	0	2nd
....Ugugagcaaaguuucaggugu...................................	5	1	2nd
....ggugagcaaaguuucGggugug..................................	1	1	2nd
....ggugagcaaaguuucagguguC..................................	3	1	2nd
....Cgugagcaaaguuucaggugug..................................	1	1	2nd
....ggugagcaaaAuuucaggugug..................................	1	1	2nd
....ggugagcaaaguuucagguguA..................................	1	1	2nd
....ggugagcaaaguuucagguguU..................................	2	1	2nd
....ggugagcaaaguuucaggugug..................................	57	0	2nd
....Ugugagcaaaguuucaggugug..................................	13	1	2nd
....Agugagcaaaguuucaggugugu.................................	2	1	2nd
....ggugagUaaaguuucaggugugu.................................	1	1	2nd
....ggugagcaaaguuucaggugugu.................................	125	0	2nd
....ggugagcaaaguuucagguguUu.................................	5	1	2nd
....Cgugagcaaaguuucaggugugu.................................	4	1	2nd
....ggugagcaaaguuucaggugugA.................................	6	1	2nd
....ggGgagcaaaguuucaggugugu.................................	6	1	2nd
....Ugugagcaaaguuucaggugugu.................................	171	1	2nd
....gugagcaaaguuucCggu.....................................	3	1	2nd
....gugagGaaaguuucaggu.....................................	2	1	2nd
....gAgagcaaaguuucaggu.....................................	2	1	2nd
....gugagcaaaguuucaggC.....................................	1	1	2nd
....gugagcaaaguCucaggu.....................................	1	1	2nd
....gugagcaaaguuucaggu.....................................	567	0	2nd
....Uugagcaaaguuucaggu.....................................	1	1	2nd
....gugagcUaaguuucaggu.....................................	1	1	2nd
....gugagcaaaguuucaggA.....................................	12	1	2nd
....guCagcaaaguuucaggug....................................	1	1	2nd
....gugagcaaaguuucagguC....................................	6	1	2nd
....gugagcaaaguuucCggug....................................	3	1	2nd
....gugCgcaaaguuucaggug....................................	1	1	2nd
....gugagcaaaguuucaggCg....................................	3	1	2nd
....gugagcaaaguuucaggGg....................................	1	1	2nd
....gugGgcaaaguuucaggug....................................	1	1	2nd
....gugagcaaagAuucaggug....................................	1	1	2nd
....gugagcaaaguuucUggug....................................	2	1	2nd
....gugagcaaaguuucagguU....................................	8	1	2nd
....gugagcaaaguuucaggug....................................	797	0	2nd
....gugagUaaaguuucaggug....................................	1	1	2nd
....gugagGaaaguuucaggug....................................	1	1	2nd
....gugagcaaaguGucaggug....................................	1	1	2nd
....gugagcaaaguuuUaggug....................................	2	1	2nd
....gugagcaaaguuucagguA....................................	7	1	2nd
....gugaAcaaaguuucaggugu...................................	3	1	2nd
....gugagcaaaguuGcaggugu...................................	2	1	2nd
....Cugagcaaaguuucaggugu...................................	1	1	2nd
....gugCgcaaaguuucaggugu...................................	2	1	2nd
....guUagcaaaguuucaggugu...................................	1	1	2nd
....gGgagcaaaguuucaggugu...................................	4	1	2nd
....gugagcGaaguuucaggugu...................................	1	1	2nd
....gAgagcaaaguuucaggugu...................................	5	1	2nd
....gugagcaaaguuucaggugG...................................	4	1	2nd
....gugUgcaaaguuucaggugu...................................	2	1	2nd
....gugagcaaaguuucaAgugu...................................	3	1	2nd

```
                    Star                                  Mature
ugauaauuugcuucauugauccaucugaaagcuugucucaaccuuugaucugaacaggugagcaaaguuucaggugugucagagaaguuuauuuucaucuuauuaucaacua
.........................................................gugagcaaaguuucagguUu...................................    4      1    2nd
.........................................................Augagcaaaguuucaggugu...................................    1      1    2nd
.........................................................gugagcUaaguuucaggugu...................................    7      1    2nd
.........................................................gugagcaaaguuucagguAu...................................    1      1    2nd
.........................................................gugagcaaaguuucUggugu...................................    3      1    2nd
.........................................................gugagAaaaguuucaggugu...................................    7      1    2nd
.........................................................gugagcaaagCuucaggugu...................................    2      1    2nd
.........................................................gugagcaaaguuucagguCu...................................    3      1    2nd
.........................................................gugagcaaaguuucaggCgu...................................    3      1    2nd
.........................................................gugagcaaaguuuGaggugu...................................    1      1    2nd
.........................................................gugagUaaaguuucaggugu...................................    3      1    2nd
.........................................................gugagcaaaguCucaggugu...................................    4      1    2nd
.........................................................gugagcCaaguuucaggugu...................................    2      1    2nd
.........................................................gugagcaaaguuuUaggugu...................................    4      1    2nd
.........................................................gCgagcaaaguuucaggugu...................................    1      1    2nd
.........................................................gugagcaaaguuucaCgugu...................................    2      1    2nd
.........................................................gugagcaaaguuucagAugu...................................    5      1    2nd
.........................................................gugagcaaagAuucaggugu...................................    1      1    2nd
.........................................................gugagcaaagGuucaggugu...................................    3      1    2nd
.........................................................gugagcaGaguuucaggugu...................................    1      1    2nd
.........................................................gugagcaaaguuucaggugu...................................    7905   0    2nd
.........................................................gugagcaaaguuucaUgugu...................................    3      1    2nd
.........................................................gugagcaaaguuucagUugu...................................    2      1    2nd
.........................................................gugagcaaaCuuucaggugu...................................    1      1    2nd
.........................................................gugagcaaaguuucaggGgu...................................    8      1    2nd
.........................................................gugagcaaaguuucaggugC...................................    18     1    2nd
.........................................................gugagGaaaguuucaggugu...................................    4      1    2nd
.........................................................gugGgcaaaguuucaggugu...................................    4      1    2nd
.........................................................gugagcaaaguuucagguAug..................................    3      1    2nd
.........................................................gugagcaaaguuucaggugCg..................................    22     1    2nd
.........................................................gugagcaUaguuucaggugug..................................    2      1    2nd
.........................................................gugagUaaaguuucaggugug..................................    17     1    2nd
.........................................................gAgagcaaaguuucaggugug..................................    17     1    2nd
.........................................................gugagcaaaguuucaggugAg..................................    5      1    2nd
.........................................................gugagcaaaguuucaggCgug..................................    9      1    2nd
.........................................................gGgagcaaaguuucaggugug..................................    2      1    2nd
.........................................................guAagcaaaguuucaggugug..................................    7      1    2nd
.........................................................gugagcaaaguuucaCgugug..................................    10     1    2nd
.........................................................gugagcaaaguuuUaggugug..................................    8      1    2nd
.........................................................gugagcaCaguuucaggugug..................................    4      1    2nd
.........................................................gugagcaaaAuuucaggugug..................................    2      1    2nd
.........................................................gugagcUaaguuucaggugug..................................    20     1    2nd
.........................................................gugaAcaaaguuucaggugug..................................    5      1    2nd
.........................................................gugaCcaaaguuucaggugug..................................    4      1    2nd
.........................................................gugagcaaaguuucagguguA..................................    50     1    2nd
.........................................................gugagcaaaguuucCggugug..................................    1      1    2nd
.........................................................gugGgcaaaguuucaggugug..................................    8      1    2nd
.........................................................gugagcaaaguuucaAgugug..................................    7      1    2nd
.........................................................gCgagcaaaguuucaggugug..................................    3      1    2nd
.........................................................gugagcaaaguuucagAugug..................................    6      1    2nd
.........................................................gugagcaaagAuucaggugug..................................    1      1    2nd
.........................................................gugagcaaaguuucaggugGg..................................    11     1    2nd
.........................................................gugagcaaaguuucaggAgug..................................    3      1    2nd
.........................................................gugagcaaaguuucGggugug..................................    12     1    2nd
.........................................................gugagcaaaguuucagguguU..................................    865    1    2nd
.........................................................gugagAaaaguuucaggugug..................................    16     1    2nd
.........................................................gugagcaaaguuucUggugug..................................    8      1    2nd
.........................................................gugagcaaaguuuGaggugug..................................    1      1    2nd
.........................................................gugagcaaaCuuucaggugug..................................    4      1    2nd
.........................................................gugCgcaaaguuucaggugug..................................    4      1    2nd
.........................................................gugagGaaaguuucaggugug..................................    11     1    2nd
.........................................................guCagcaaaguuucaggugug..................................    4      1    2nd
.........................................................gugUgcaaaguuucaggugug..................................    5      1    2nd
.........................................................gugagcaaUguuucaggugug..................................    1      1    2nd
.........................................................gugagcaaaguuCcaggugug..................................    4      1    2nd
.........................................................gugagcaaaguAucaggugug..................................    2      1    2nd
.........................................................gugagcaaagGuucaggugug..................................    3      1    2nd
.........................................................gugagcaaaguuucagCugug..................................    4      1    2nd
.........................................................gugagcGaaguuucaggugug..................................    2      1    2nd
.........................................................gugagcaaaguuucaggugug..................................    22296  0    2nd
```

Star Mature

```
ugauaauuugcuucauugauccaucugaaagcuugucucaaccuuugaucugaacaggugagcaaaguuucaggugugucagagaaguuuauuuucaucuuauuaucaacua
.........................................................Uugagcaaaguuucaggugug...................................	6	1	2nd
.........................................................gugagcaaaguCucaggugug...................................	4	1	2nd
.........................................................gugagcaaaguuucaggGgug...................................	12	1	2nd
.........................................................gugagcaaaguuucagguguC...................................	9	1	2nd
.........................................................gugagcaaaguuucagguCug...................................	2	1	2nd
.........................................................gugagcaaGguuucaggugug...................................	2	1	2nd
.........................................................gugagcaaagCuucaggugug...................................	6	1	2nd
.........................................................gugagcaaaguuucaUgugug...................................	4	1	2nd
.........................................................gugagcaaaguuucagUugug...................................	2	1	2nd
.........................................................gugaUcaaaguuucaggugug...................................	1	1	2nd
.........................................................gGgagcaaaguuucaggugugu.................................	93	1	2nd
.........................................................Uugagcaaaguuucaggugugu.................................	50	1	2nd
.........................................................gugagcaaaguuucagguCugu.................................	59	1	2nd
.........................................................guCagcaaaguuucaggugugu.................................	94	1	2nd
.........................................................gugGgcaaaguuucaggugugu.................................	195	1	2nd
.........................................................gugagcaaaguuuGaggugugu.................................	41	1	2nd
.........................................................gugagcaaaguuucaggugugA.................................	3374	1	2nd
.........................................................guAagcaaaguuucaggugugu.................................	71	1	2nd
.........................................................gugagcaaaguuuUaggugugu.................................	265	1	2nd
.........................................................gugagcaaaguuucaggGgugu.................................	171	1	2nd
.........................................................gugagcGaaguuucaggugugu.................................	69	1	2nd
.........................................................gugagUaaaguuucaggugugu.................................	379	1	2nd
.........................................................gugagcaaaguuucagguguAu.................................	109	1	2nd
.........................................................gugagcaaaguuGcaggugugu.................................	3	1	2nd
.........................................................gugagcaaaguuucagguUugu.................................	50	1	2nd
.........................................................gugagcaaaguuucaggugugu.................................	449436	0	2nd
.........................................................gugagcaaaguuucagAugugu.................................	131	1	2nd
.........................................................gugagcaaUguuucaggugugu.................................	23	1	2nd
.........................................................gugagcaaaguuucaggugugUu.................................	2028	1	2nd
.........................................................gugagcaaaAuuucaggugugu.................................	43	1	2nd
.........................................................Augagcaaaguuucaggugugu.................................	34	1	2nd
.........................................................Cugagcaaaguuucaggugugu.................................	35	1	2nd
.........................................................gugagcaaaguuAcaggugugu.................................	8	1	2nd
.........................................................gugagcaaaCuuucaggugugu.................................	47	1	2nd
.........................................................gugagcaaaguuuAaggugugu.................................	29	1	2nd
.........................................................gugagcaaagGuucaggugugu.................................	120	1	2nd
.........................................................gCgagcaaaguuucaggugugu.................................	48	1	2nd
.........................................................gugagcaaaguuCcaggugugu.................................	46	1	2nd
.........................................................gugagcaaaguuucaggugGgu.................................	188	1	2nd
.........................................................gugaCcaaaguuucaggugugu.................................	73	1	2nd
.........................................................gugagcUaaguuucaggugugu.................................	484	1	2nd
.........................................................gugagcaaCguuucaggugugu.................................	10	1	2nd
.........................................................gugagcaaaguGucaggugugu.................................	29	1	2nd
.........................................................gugagcaaaguuucagUugugu.................................	129	1	2nd
.........................................................gugagcaaagAuucaggugugu.................................	71	1	2nd
.........................................................gugagcaaGguuucaggugugu.................................	20	1	2nd
.........................................................gugagcaaaguuucagguAugu.................................	29	1	2nd
.........................................................gugagcaaaguuucGggugugu.................................	222	1	2nd
.........................................................gugagcaaaguuucCggugugu.................................	12	1	2nd
.........................................................gugagcaaaguuucaAgugugu.................................	117	1	2nd
.........................................................gugagGaaaguuucaggugugu.................................	178	1	2nd
.........................................................gugagcaaaguuucaggugugCu.................................	68	1	2nd
.........................................................gugagcaGaguuucaggugugu.................................	35	1	2nd
.........................................................gugagcaaaguuucUggugugu.................................	201	1	2nd
.........................................................gugUgcaaaguuucaggugugu.................................	97	1	2nd
.........................................................gugagcaaaguCucaggugugu.................................	45	1	2nd
.........................................................gugagcaUaguuucaggugugu.................................	95	1	2nd
.........................................................gugagcaaaguuucaUgugugu.................................	111	1	2nd
.........................................................gugagcaaaUuuucaggugugu.................................	14	1	2nd
.........................................................gugaUcaaaguuucaggugugu.................................	20	1	2nd
.........................................................gugagcaaaguuucaggAgugu.................................	32	1	2nd
.........................................................gugagcaaaguuucagCugugu.................................	142	1	2nd
.........................................................gugagcaaaguAucaggugugu.................................	23	1	2nd
.........................................................gugagAaaaguuucaggugugu.................................	306	1	2nd
.........................................................gugaAcaaaguuucaggugugu.................................	119	1	2nd
.........................................................guUagcaaaguuucaggugugu.................................	33	1	2nd
.........................................................gugagcaaaguuucaggugugG.................................	242	1	2nd
.........................................................gugagcaaaguuucaggCgugu.................................	92	1	2nd
.........................................................gAgagcaaaguuucaggugugu.................................	287	1	2nd
.........................................................gugagcaaaguuucaCgugugu.................................	178	1	2nd
```

```
                    Star                                Mature
ugauaauuugcuucauugauccaucugaaagcuugucucaaccuuugaucugaacaggugagcaaaguuucaggugugucagagaaguuuauuuucaucuuauuaucaacua
.........................................................gugagcaaaguuucaggugCgu.................................    118    1    2nd
.........................................................gugCgcaaaguuucaggugugu.................................    66    1    2nd
.........................................................gugagcaCaguuucaggugugu.................................    59    1    2nd
.........................................................gugagcaaagCuucaggugugu.................................    75    1    2nd
.........................................................gugagcCaaguuucaggugugu.................................    19    1    2nd
.........................................................gugagcaaaguuucaggugAgu.................................    52    1    2nd
.........................................................gugagcaaaguuucagguguguG................................    78    1    2nd
.........................................................gugagcaaaguuucaggugugGc................................    3    1    2nd
.........................................................gugagcaaaguuucaggugugAc................................    1    1    2nd
.........................................................gugagcaaaguuucagguguUuc................................    1    1    2nd
.........................................................gugagcaaaguuucagguguguc................................    48    0    2nd
.........................................................gugagcaaaguuucaggugGguc................................    1    1    2nd
.........................................................gugagcaaaguuucaggugugCc................................    6    1    2nd
.........................................................gugagcaaaguuucaggugugucC...............................    9    1    2nd
.........................................................gugagcaaaguuucagguguguca...............................    4    0    2nd
.........................................................gugagcaaaguuucagguguguUa...............................    164    1    2nd
.........................................................gugagAaaaguuucagguguguca...............................    1    1    2nd
.........................................................gugagcaaaguuucagguguguсU...............................    13    1    2nd
.........................................................gugagcaaaguuucagguguguGaga.............................    1    1    2nd
.........................................................gugagcaaaguuucagguguguGagag............................    1    1    2nd
..........................................................Ggagcaaaguuucaggugug..................................    1    1    2nd
..........................................................ugagcaaaguuucaggugug..................................    6    0    2nd
..........................................................Ggagcaaaguuucaggugugu.................................    1    1    2nd
..........................................................uCagcaaaguuucaggugugu.................................    1    1    2nd
..........................................................ugagGaaaguuucaggugugu.................................    1    1    2nd
..........................................................ugagcaaaguuucagguguUu.................................    1    1    2nd
..........................................................ugagcCaaguuucaggugugu.................................    4    1    2nd
..........................................................ugagcaaaguuucaggugugu.................................    52    0    2nd
...........................................................gagcaaaguuucaggugu...................................    4    0    2nd
...........................................................gagcaaaguuucaggugug..................................    8    0    2nd
...........................................................gagcaaaguuucaggugugu.................................    152    0    2nd
...........................................................gagcaaaguuucaggugAgu.................................    1    1    2nd
...........................................................gagcaaaguuucagguguUu.................................    1    1    2nd
...........................................................gagcaaaguuucGggugugu.................................    1    1    2nd
...........................................................gagcaaaguuucaggAgugu.................................    1    1    2nd
...........................................................gagcaaaguuucaggugugG.................................    1    1    2nd
............................................................agcaaaguuucaggugug..................................    4    0    2nd
............................................................agcaaaguuucagguguUu.................................    1    1    2nd
............................................................agcaaaguuucaggugugu.................................    125    0    2nd
............................................................agcaaGguuucaggugugu.................................    1    1    2nd
............................................................agcaaaguuucaggugugA.................................    1    1    2nd
.............................................................gcaaaguuucaggugugu.................................    105    0    2nd
.............................................................gcaaaguuucaggugugA.................................    1    1    2nd
.............................................................gcaaaguuucaggCgugu.................................    1    1    2nd
.............................................................gcaaaguuucagguguguc................................    1    0    2nd

....................ccaucugaaagcuugucuc.........................................................................    4    0    LJ5
....................ccaucugaaagcuugucucaac......................................................................    3    0    LJ5
....................ccaucugaaagcuugucuUaac......................................................................    2    1    LJ5
....................ccaucugaaagcuugucucaacA.....................................................................    1    1    LJ5
....................ccaucCgaaagcuugucucaacc.....................................................................    1    1    LJ5
....................ccUucugaaagcuugucucaacc.....................................................................    1    1    LJ5
....................ccaucugaaagcuugucucaacU.....................................................................    1    1    LJ5
....................ccaucugaaagcuugucucaacc.....................................................................    5    0    LJ5
....................ccaucugaaagcuugucucaaccA....................................................................    1    1    LJ5
....................ccaucugaaagcuugucucaaccu....................................................................    10    0    LJ5
....................ccaucugaaagcuugucucaaUcu....................................................................    1    1    LJ5
.....................caCcugaaagcuugucucaaccuuu..................................................................    1    1    LJ5
............................................uugaucugaacaggugagcaaaguuuc.........................................    1    0    LJ5
........................................................Cgugagcaaaguuucaggugug..................................    1    1    LJ5
........................................................Cgugagcaaaguuucaggugugu.................................    6    1    LJ5
........................................................gAugagcaaaguuucaggugugu.................................    1    1    LJ5
.........................................................gugagcaaaguuucaggC.....................................    3    1    LJ5
.........................................................gugagcaaaguuucaggu.....................................    21    0    LJ5
.........................................................gugagcaaaguuucagguU....................................    2    1    LJ5
.........................................................gugGgcaaaguuucaggug....................................    1    1    LJ5
.........................................................gugagcaaaguuucagguA....................................    3    1    LJ5
.........................................................gugagcaaaguuucaggGg....................................    1    1    LJ5
.........................................................gugagcaGaguuucaggug....................................    1    1    LJ5
.........................................................gugagcaaaguuucaggug....................................    62    0    LJ5
```

```
                     Star                                Mature
ugauaauuugcuucauugauccaucugaaagcuugucucaaccuuugaucugaacaggugagcaaaguuucaggugugucagagaaguuuauuuucaucuuauuaucaacua
.........................................................gugagcaaaguuucaggGgu...................................    1     1    LJ5
.........................................................gugagcaaaguuucaggugG...................................    4     1    LJ5
.........................................................gugagcaaaguuucGggugu...................................    1     1    LJ5
.........................................................gCgagcaaaguuucaggugu...................................    1     1    LJ5
.........................................................gugagcaaaguuucaggugu...................................    140   0    LJ5
.........................................................gugagcaaaguuucaggugC...................................    20    1    LJ5
.........................................................gugagcaaagGuucaggugu...................................    1     1    LJ5
.........................................................guUagcaaaguuucaggugu...................................    1     1    LJ5
.........................................................gugagcaGaguuucaggugug..................................    3     1    LJ5
.........................................................gugagcaaaguCucaggugug..................................    1     1    LJ5
.........................................................gugagcaaaguuucaggCgug..................................    1     1    LJ5
.........................................................gugagUaaaguuucaggugug..................................    1     1    LJ5
.........................................................guCagcaaaguuucaggugug..................................    2     1    LJ5
.........................................................gugagcaaaguuCcaggugug..................................    3     1    LJ5
.........................................................gugagcaaaguuucaggugCg..................................    9     1    LJ5
.........................................................gAgagcaaaguuucaggugug..................................    3     1    LJ5
.........................................................Augagcaaaguuucaggugug..................................    1     1    LJ5
.........................................................gugagcaaaguuucagguguA..................................    76    1    LJ5
.........................................................gCgagcaaaguuucaggugug..................................    3     1    LJ5
.........................................................gugagcaaGguuucaggugug..................................    4     1    LJ5
.........................................................gugaAcaaaguuucaggugug..................................    1     1    LJ5
.........................................................gugagcaaaguuucaggugug..................................    818   0    LJ5
.........................................................gugUgcaaaguuucaggugug..................................    3     1    LJ5
.........................................................gugGgcaaaguuucaggugug..................................    2     1    LJ5
.........................................................gugagcGaaguuucaggugug..................................    3     1    LJ5
.........................................................gugagcaaaguuucaAgugug..................................    1     1    LJ5
.........................................................gugagcaaaguuucagguguC..................................    4     1    LJ5
.........................................................gugagcaaaguuucUggugug..................................    2     1    LJ5
.........................................................guAagcaaaguuucaggugug..................................    2     1    LJ5
.........................................................gugagcaaaguuucGggugug..................................    1     1    LJ5
.........................................................gugagcaaagCuucaggugug..................................    3     1    LJ5
.........................................................gugagcaaaguuucagguguU..................................    23    1    LJ5
.........................................................guUagcaaaguuucaggugug..................................    4     1    LJ5
.........................................................gugagcCaaguuucaggugug..................................    1     1    LJ5
.........................................................gugagcaaaguuucaUgugug..................................    4     1    LJ5
.........................................................gugagcaaaguuucagguAug..................................    1     1    LJ5
.........................................................Augagcaaaguuucaggugugu.................................    4     1    LJ5
.........................................................gugagcaaaguuucagguguCu.................................    4     1    LJ5
.........................................................gugagcaaaguuucagguguAu.................................    5     1    LJ5
.........................................................gugagcGaaguuucaggugugu.................................    12    1    LJ5
.........................................................gugagcaaaguuucGggugugu.................................    25    1    LJ5
.........................................................gugagcaaaguuucaggugugG.................................    93    1    LJ5
.........................................................gugagcaaaguuucagAugugu.................................    4     1    LJ5
.........................................................gugagcaaaguuucagguguUu.................................    11    1    LJ5
.........................................................gGgagcaaaguuucaggugugu.................................    1     1    LJ5
.........................................................guAagcaaaguuucaggugugu.................................    21    1    LJ5
.........................................................gugagcaaaguuGcaggugugu.................................    1     1    LJ5
.........................................................gugagcaaaguuucagUugugu.................................    1     1    LJ5
.........................................................guCagcaaaguuucaggugugu.................................    9     1    LJ5
.........................................................gugagcaaaguuucaAgugugu.................................    2     1    LJ5
.........................................................gugagcaaaguCucaggugugu.................................    9     1    LJ5
.........................................................gugagcaaaguuucaggugAgu.................................    2     1    LJ5
.........................................................gugagcaaaguuuAaggugugu.................................    1     1    LJ5
.........................................................gugGgcaaaguuucaggugugu.................................    22    1    LJ5
.........................................................gugagUaaaguuucaggugugu.................................    7     1    LJ5
.........................................................gugagcaaaguuucaggugugA.................................    176   1    LJ5
.........................................................gugagcUaaguuucaggugugu.................................    4     1    LJ5
.........................................................gCgagcaaaguuucaggugugu.................................    17    1    LJ5
.........................................................gugagcaaaguuAcaggugugu.................................    2     1    LJ5
.........................................................gugagcaaaguuucUggugugu.................................    7     1    LJ5
.........................................................gugagcCaaguuucaggugugu.................................    1     1    LJ5
.........................................................gugagcaaUguuucaggugugu.................................    1     1    LJ5
.........................................................gugagcaaagGuucaggugugu.................................    10    1    LJ5
.........................................................gugagcaaGguuucaggugugu.................................    27    1    LJ5
.........................................................gugagcaaaguGucaggugugu.................................    1     1    LJ5
.........................................................gugagcaaaguuucaggCgugu.................................    17    1    LJ5
.........................................................gugagcaaaguuCcaggugugu.................................    15    1    LJ5
.........................................................gugagAaaaguuucaggugugu.................................    2     1    LJ5
.........................................................gugagcaaaguuucaggGgugu.................................    3     1    LJ5
.........................................................gugagcaaaguuucaggugugu.................................    5517  0    LJ5
```

```
                    Star                                    Mature
ugauaauuugcuucauugauccaucugaaagcuugucucaaccuuugaucugaacaggugagcaaaguuucaggugugucagagaaguuuauuuucaucuuauuaucaacua
.........................................................gugagcaCaguuucaggugugu.................................    4     1     LJ5
.........................................................gugagcaaaguuucaUgugugu.................................    1     1     LJ5
.........................................................gugagcaGaguuucaggugugu.................................    22    1     LJ5
.........................................................gugagcaaaguuucagCugugu.................................    1     1     LJ5
.........................................................gugagcaaaguuucagguAugu.................................    3     1     LJ5
.........................................................gugaAcaaaguuucaggugugu.................................    5     1     LJ5
.........................................................guUagcaaaguuucaggugugu.................................    14    1     LJ5
.........................................................gugagcaaaguuucCggugugu.................................    1     1     LJ5
.........................................................gugaCcaaaguuucaggugugu.................................    2     1     LJ5
.........................................................gugagcaaaUuuucaggugugu.................................    3     1     LJ5
.........................................................gugaUcaaaguuucaggugugu.................................    2     1     LJ5
.........................................................gugagcaaaguuucaggAgugu.................................    1     1     LJ5
.........................................................gugagcaaaguuuUaggugugu.................................    11    1     LJ5
.........................................................gugagcaaaguuucaggugCgu.................................    28    1     LJ5
.........................................................gugagcaaaguuuGaggugugu.................................    1     1     LJ5
.........................................................gugagcaaaguuucaggugGgu.................................    7     1     LJ5
.........................................................Uugagcaaaguuucaggugugu.................................    1     1     LJ5
.........................................................gugagcaaagCuucaggugugu.................................    9     1     LJ5
.........................................................gugagcaUaguuucaggugugu.................................    4     1     LJ5
.........................................................gugagcaaaguuucagguCugu.................................    1     1     LJ5
.........................................................gugagcaaaguuucaggugugc................................    11    0     LJ5
.........................................................gugagcaaaguuucaggCguguc................................    2     1     LJ5
.........................................................gugagcaaaguuucaggugugugG................................    42    1     LJ5
.........................................................gugagcaaaguuucaggGguguc................................    1     1     LJ5
..........................................................ugagcaaaguuucaggugug..................................    1     0     LJ5
..........................................................ugagcaaaguuCcaggugugu.................................    1     1     LJ5
..........................................................ugagcaaaguuucaggugugu.................................    7     0     LJ5
...........................................................gagcaaaguuucaggugug..................................    2     0     LJ5
...........................................................gagcaaaguuucaggugugu.................................    2     0     LJ5

...................uccaCcugaaagcuugucucaacc.....................................................................    5     1     H01
...................Accaucugaaagcuugucucaaccu....................................................................    1     1     H01
...................uccaCcugaaagcuugucucaaccu....................................................................    4     1     H01
....................ccaucugaaagcuugucu..........................................................................    2     0     H01
....................ccaucugaaagcuugucuc.........................................................................    11    0     H01
....................ccaAcugaaagcuugucuca........................................................................    1     1     H01
....................ccaucugaaagcuugucuca........................................................................    6     0     H01
....................ccaucugaaagcuugucucaa.......................................................................    2     0     H01
....................ccaucugaaagcuugucucaac......................................................................    20    0     H01
....................ccaucugUaagcuugucucaacc.....................................................................    1     1     H01
....................ccaucugaaagcuugucucaacc.....................................................................    90    0     H01
....................ccaucugaaagcCugucucaacc.....................................................................    1     1     H01
....................ccaucugaaagcuugucucaaccA....................................................................    6     1     H01
....................ccaucugaaagcuugucucaaccu....................................................................    375   0     H01
....................ccaucugaaaAcuugucucaaccu....................................................................    2     1     H01
....................ccaucugaaagcAugucucaaccu....................................................................    1     1     H01
....................ccaucugaaaCcuugucucaaccu....................................................................    1     1     H01
....................ccaucugaaagcuugucucaUccu....................................................................    1     1     H01
....................ccaucugUaagcuugucucaaccu....................................................................    2     1     H01
....................cUaucugaaagcuugucucaaccu....................................................................    1     1     H01
....................ccaucugaaagcuugucucaaccG....................................................................    1     1     H01
....................ccaucugaaagcuugucucaaccuA...................................................................    2     1     H01
....................ccaucugaaagcuugucucaaccuu...................................................................    5     0     H01
....................ccaCcugaaagcuugucucaaccuuu..................................................................    4     1     H01
....................ccaucugaaagcuugucucaaccuuA..................................................................    1     1     H01
....................ccaucugaaagcuugucucaaccuuuU.................................................................    2     1     H01
.....................cCucugaaagcuugucucaaccu....................................................................    2     1     H01
.....................caucugaaagcuugucucaaccuu...................................................................    1     0     H01
.....................caCcugaaagcuugucucaaccuuu..................................................................    20    1     H01
......................aucugaaagcuugucucaaccu....................................................................    1     0     H01
......................Cucugaaagcuugucucaaccuuu..................................................................    1     1     H01
........................cugaaagcuugucucaac......................................................................    1     0     H01
........................cugaaagcuugucucaacc.....................................................................    5     0     H01
........................cugaaagcuugucucaaccu....................................................................    14    0     H01
.........................ugaaagcuugucucaacc.....................................................................    38    0     H01
.........................ugaaagcuugucucaaccu....................................................................    103   0     H01
.........................ugaaagcuugucucaaccG....................................................................    1     1     H01
.........................ugaaagcuugucucaaccC....................................................................    1     1     H01
.........................ugaaagcuugucucaaccuu...................................................................    14    0     H01
..........................gGaagcuugucucaaccu....................................................................    1     1     H01
```

Star **Mature**

```
ugauaauuugcuucauugauccaucugaaagcuugucucaaccuuugaucugaacaggugagcaaaguuucaggugugucagagaaguuuauuuucaucuuauuaucaacua
..........................gaaagcuugucucaaccu....................................................................	35	0	H01
..........................gaaagcuugucucaaccuu...................................................................	3	0	H01
..........................gGaagcuugucucaaccuu...................................................................	1	1	H01
...........................aaagcuugucucaaccuu...................................................................	2	0	H01
...........................aaaCcuugucucaaccuu...................................................................	1	1	H01
.....................................................acaCgugagcaaaguuucaggugugu.................................	1	1	H01
.....................................................acGggugagcaaaguuucaggugugu.................................	1	1	H01
......................................................caAgugagcaaaguuucaggugugu.................................	2	1	H01
......................................................cUggugagcaaaguuucaggugugu.................................	1	1	H01
.......................................................aggugagcaaaguuucaggugu...................................	1	0	H01
.......................................................Uggugagcaaaguuucaggugug..................................	1	1	H01
.......................................................aUgugagcaaaguuucaggugugu.................................	7	1	H01
.......................................................Uggugagcaaaguuucaggugugu.................................	4	1	H01
.......................................................aggugagcaaaguuucaggugugu.................................	3	0	H01
.......................................................aCgugagcaaaguuucaggugugu.................................	3	1	H01
.......................................................Gggugagcaaaguuucaggugugu.................................	2	1	H01
.......................................................aAgugagcaaaguuucaggugugu.................................	3	1	H01
........................................................ggugagcaaaguuucagA......................................	1	1	H01
........................................................ggugagcaaaguuucagg......................................	3	0	H01
........................................................Ugugagcaaaguuucaggu.....................................	3	1	H01
........................................................ggugagcaaaguuucaggu.....................................	5	0	H01
........................................................Cgugagcaaaguuucaggu.....................................	1	1	H01
........................................................Ugugagcaaaguuucaggug....................................	1	1	H01
........................................................ggugagcaaaguuucaggug....................................	3	0	H01
........................................................ggugagcaaaguuucagguUu...................................	1	1	H01
........................................................ggugagcaaaguuucaggugu...................................	76	0	H01
........................................................ggugagcaaaguuuUaggugu...................................	1	1	H01
........................................................Ugugagcaaaguuucaggugu...................................	4	1	H01
........................................................ggugagcaaaguuucGggugug..................................	1	1	H01
........................................................Cgugagcaaaguuucaggugug..................................	1	1	H01
........................................................ggugagcaaaguuucaggugCg..................................	1	1	H01
........................................................ggugagcaaaguuucaggugug..................................	152	0	H01
........................................................Agugagcaaaguuucaggugug..................................	1	1	H01
........................................................ggugagcaaaguuucagguguU..................................	27	1	H01
........................................................Ugugagcaaaguuucaggugug..................................	18	1	H01
........................................................ggugagcaaaguuucagguguA..................................	3	1	H01
........................................................ggugGgcaaaguuucaggugugu.................................	1	1	H01
........................................................Cgugagcaaaguuucaggugugu.................................	23	1	H01
........................................................Ugugagcaaaguuucaggugugu.................................	402	1	H01
........................................................Agugagcaaaguuucaggugugu.................................	15	1	H01
........................................................ggugagcaaaguuucagguguAu.................................	1	1	H01
........................................................ggugagcaaaguuucaggugugu.................................	133	0	H01
........................................................ggugagcaaaguuucagguguUu.................................	6	1	H01
........................................................ggugagcaaaguuucagguguCu.................................	1	1	H01
........................................................ggugagcaaaguuucaggugugA.................................	12	1	H01
.........................................................gugagcaaaguuucaggC.....................................	8	1	H01
.........................................................gugagcaaaCuuucaggu.....................................	1	1	H01
.........................................................gugagcaaaguuucaggA.....................................	50	1	H01
.........................................................gCgagcaaaguuucaggu.....................................	1	1	H01
.........................................................gugagcaGaguuucaggu.....................................	1	1	H01
.........................................................gugagcaaaguuucGggu.....................................	8	1	H01
.........................................................gugagcaaaguuucaggu.....................................	3657	0	H01
.........................................................gugagcaaaguuucCggu.....................................	16	1	H01
.........................................................gugagcaaaguuucUggu.....................................	3	1	H01
.........................................................gugaAcaaaguuucaggu.....................................	2	1	H01
.........................................................gugagcaaagCuucaggu.....................................	1	1	H01
.........................................................gugagcaaaguuuUaggu.....................................	1	1	H01
.........................................................gugagcaaaguuCcaggu.....................................	1	1	H01
.........................................................gugagcaaaguuGcaggu.....................................	1	1	H01
.........................................................gugGgcaaaguuucaggu.....................................	1	1	H01
.........................................................gAgagcaaaguuucaggu.....................................	2	1	H01
.........................................................gugaCcaaaguuucaggu.....................................	1	1	H01
.........................................................gugagcaaaguuucagguC....................................	4	1	H01
.........................................................gugagcaaaguuucaggCg....................................	2	1	H01
.........................................................gugagcaaaguuucagguU....................................	32	1	H01
.........................................................gugagcaaaguuucCggug....................................	3	1	H01
.........................................................gugagcaaaguuucaggug....................................	939	0	H01
.........................................................gugaAcaaaguuucaggug....................................	1	1	H01
.........................................................gugagcaaaguuucGggug....................................	4	1	H01
.........................................................gugagcaaaguuucUggug....................................	3	1	H01
```

Star **Mature**

ugauaauuugcuucauugauccaucugaaagcuugucucaaccuuugaucugaacaggugagcaaaguuucaggugugucagagaaguuuauuuucaucuuauuaucaacua			
....gugagcaaaguuucagguA....................................	27	1	H01
....gugagcaaaguuucaAgug....................................	1	1	H01
....gugagcaaaguuuUaggug....................................	6	1	H01
....gugagcaaaguuucaUgug....................................	1	1	H01
....gugagUaaaguuucaggugu...................................	5	1	H01
....gugagcaaaAuuucaggugu...................................	1	1	H01
....gugagcaaaguuucagguCu...................................	1	1	H01
....Augagcaaaguuucaggugu...................................	1	1	H01
....gugagcaaaguuuGaggugu...................................	2	1	H01
....gugagcaaagAuucaggugu...................................	1	1	H01
....gugagcaaaguuucagguUu...................................	2	1	H01
....gugagcaaaguuucGggugu...................................	14	1	H01
....gugagcaaaguuucaUgugu...................................	2	1	H01
....gugagcaaaguuucaggugC...................................	5	1	H01
....gugagcaaaUuuucaggugu...................................	1	1	H01
....gGgagcaaaguuucaggugu...................................	1	1	H01
....gugagcaaaguuucCggugu...................................	1	1	H01
....gugagcaaaguCucaggugu...................................	4	1	H01
....gugagcaaagCuucaggugu...................................	1	1	H01
....gugagcaaaguuuUaggugu...................................	15	1	H01
....gugagcaaaCuuucaggugu...................................	4	1	H01
....Uugagcaaaguuucaggugu...................................	1	1	H01
....guCagcaaaguuucaggugu...................................	1	1	H01
....gugagcaaaguuucaAgugu...................................	1	1	H01
....gugaAcaaaguuucaggugu...................................	2	1	H01
....gugagcGaaguuucaggugu...................................	1	1	H01
....gAgagcaaaguuucaggugu...................................	5	1	H01
....gugagcaaaguuucUggugu...................................	6	1	H01
....gugagAaaaguuucaggugu...................................	1	1	H01
....gugagcaaaguuucaggGgu...................................	2	1	H01
....gugagcaUaguuucaggugu...................................	2	1	H01
....gugagcaaaguuucagAugu...................................	1	1	H01
....gugagcaaaguuucaggAgu...................................	1	1	H01
....gugagcaGaguuucaggugu...................................	2	1	H01
....gugagcaaagGuucaggugu...................................	6	1	H01
....gugagcaaaguuCcaggugu...................................	4	1	H01
....gugagcaaaguuucaggugu...................................	6877	0	H01
....gugagcaaaguuucaggugG...................................	3	1	H01
....gugagcaaaguuucUggugug..................................	7	1	H01
....gugagcaaaguuucaUgugug..................................	3	1	H01
....gugagcaaaUuuucaggugug..................................	1	1	H01
....gugagcaaaguCucaggugug..................................	3	1	H01
....gugagcaaaguuucagUugug..................................	5	1	H01
....gugagcaaGguuucaggugug..................................	1	1	H01
....gugagcaaaguuucaggCgug..................................	2	1	H01
....gugagcaaaguuucagguCug..................................	6	1	H01
....gugagcaaaguuucaCgugug..................................	2	1	H01
....gugGgcaaaguuucaggugug..................................	3	1	H01
....gugagUaaaguuucaggugug..................................	15	1	H01
....Uugagcaaaguuucaggugug..................................	6	1	H01
....gCgagcaaaguuucaggugug..................................	1	1	H01
....Cugagcaaaguuucaggugug..................................	1	1	H01
....gugagcaaaguuAcaggugug..................................	2	1	H01
....guCagcaaaguuucaggugug..................................	1	1	H01
....gugagcaaaguuucaggugugU.................................	2223	1	H01
....gugagcaaagCuucaggugug..................................	2	1	H01
....gugagcaaaguuGcaggugug..................................	2	1	H01
....gugagcaaaguuucaggugCg..................................	14	1	H01
....gugagcaaaguuucagCugug..................................	1	1	H01
....gugagcaaaguuCcaggugug..................................	7	1	H01
....gugaUcaaaguuucaggugug..................................	2	1	H01
....gugagcaaaguuucagguguC..................................	14	1	H01
....gugagcaaaguuucaggugAg..................................	8	1	H01
....gugagcaaaguuucagAugug..................................	2	1	H01
....gGgagcaaaguuucaggugug..................................	7	1	H01
....gugagAaaaguuucaggugug..................................	1	1	H01
....gugagcaUaguuucaggugug..................................	1	1	H01
....gugagcaaaguuucaggGgug..................................	10	1	H01
....gugagcaaaguuuGaggugug..................................	4	1	H01
....guAagcaaaguuucaggugug..................................	2	1	H01

Star **Mature**

ugauaauuugcuucauugauccaucugaaagcuugucucaaccuuugaucugaacaggugagcaaaguuucaggugugucagagaaguuuauuuucaucuuauuaucaacua			
....gugagcaaaguuucaAgugug..................................	3	1	H01
....gugagcaGaguuucaggugug..................................	3	1	H01
....gugagcaaaguuuUaggugug..................................	14	1	H01
....gugaCcaaaguuucaggugug..................................	1	1	H01
....gugaAcaaaguuucaggugug..................................	10	1	H01
....guUagcaaaguuucaggugug..................................	1	1	H01
....gugagcaaaguuucagguguA..................................	162	1	H01
....gugagcaaaguuucCggugug..................................	2	1	H01
....gugUgcaaaguuucaggugug..................................	2	1	H01
....gugagcaaaguuucagguAug..................................	2	1	H01
....gugagcaaaguuuAaggugug..................................	1	1	H01
....gugagcUaaguuucaggugug..................................	1	1	H01
....gugagcaaaCuuucaggugug..................................	11	1	H01
....gugagcaaaguuucGggugug..................................	37	1	H01
....gugagcaCaguuucaggugug..................................	2	1	H01
....gugagcaaagGuucaggugug..................................	18	1	H01
....gAgagcaaaguuucaggugug..................................	12	1	H01
....gugagcaaaguuucaggugug..................................	22180	0	H01
....gugagcaaaguuucagguUug..................................	3	1	H01
....gugagcaaCguuucaggugugu.................................	7	1	H01
....gugagcaaaguuuAaggugugu.................................	29	1	H01
....gGgagcaaaguuucaggugugu.................................	94	1	H01
....gugagcaaaUuuucaggugugu.................................	21	1	H01
....gugUgcaaaguuucaggugugu.................................	48	1	H01
....gugagcUaaguuucaggugugu.................................	9	1	H01
....gugagcaaaguuucagguCugu.................................	25	1	H01
....gugagcaaaguCucaggugugu.................................	31	1	H01
....gugagcaaagGuucaggugugu.................................	170	1	H01
....gugGgcaaaguuucaggugugu.................................	40	1	H01
....gugagcaaaguuucaggugCgu.................................	24	1	H01
....gugagcaaaguuGcaggugugu.................................	33	1	H01
....gugagcaaaguuucUggugugu.................................	276	1	H01
....gugagcaaaguuucaggugGgu.................................	26	1	H01
....gugaCcaaaguuucaggugugu.................................	29	1	H01
....gugagcaaagAuucaggugugu.................................	11	1	H01
....gugagcaaaguuAcaggugugu.................................	14	1	H01
....Augagcaaaguuucaggugugu.................................	7	1	H01
....gugagcaaaguuucaggAgugu.................................	8	1	H01
....gugagcaaaguuucaUgugugu.................................	61	1	H01
....gugagcaaaguuucaCgugugu.................................	51	1	H01
....gugagcaGaguuucaggugugu.................................	53	1	H01
....gugagcaaaguuucaggGgugu.................................	63	1	H01
....guCagcaaaguuucaggugugu.................................	34	1	H01
....gugagcaaaAuuucaggugugu.................................	11	1	H01
....gugagcaaaguuucaggugAgu.................................	10	1	H01
....gugaAcaaaguuucaggugugu.................................	89	1	H01
....guUagcaaaguuucaggugugu.................................	34	1	H01
....gugagcaaaguuucaggugugu.................................	391286	0	H01
....gugagcaaaguuucaggugugG.................................	141	1	H01
....Cugagcaaaguuucaggugugu.................................	8	1	H01
....gugagcGaaguuucaggugugu.................................	24	1	H01
....Uugagcaaaguuucaggugugu.................................	90	1	H01
....gugagcaaUguuucaggugugu.................................	19	1	H01
....gugagGaaaguuucaggugugu.................................	6	1	H01
....gugagcaaaguuuUaggugugu.................................	524	1	H01
....gugagUaaaguuucaggugugu.................................	231	1	H01
....gugagcaaagCuucaggugugu.................................	32	1	H01
....gugaUcaaaguuucaggugugu.................................	30	1	H01
....gugCgcaaaguuucaggugugu.................................	21	1	H01
....gugagcaUaguuucaggugugu.................................	74	1	H01
....gugagcaaaguuCcaggugugu.................................	112	1	H01
....gugagcaaaguuucaggugugA.................................	3760	1	H01
....gugagcCaaguuucaggugugu.................................	2	1	H01
....gugagAaaaguuucaggugugu.................................	23	1	H01
....gugagcaaaguuucagCugugu.................................	72	1	H01
....gugagcaaGguuucaggugugu.................................	18	1	H01
....gugagcaaaguGucaggugugu.................................	13	1	H01
....gugagcaaaguAucaggugugu.................................	3	1	H01
....gugagcaaaguuucagguguAu.................................	175	1	H01
....gugagcaaaguuucaAgugugu.................................	51	1	H01

Star Mature

ugauaauuugcuucauugauccaucugaaagcuugucucaaccuuugaucugaacaggugagcaaaguuucaggugugucagagaaguuuauuuucaucuuauuaucaacua

....gugagcaaaguuucagUugugu.................................	68	1	H01
....gugagcaaaguuucagguguUu.................................	3183	1	H01
....gugagcaaaguuucagAugugu.................................	29	1	H01
....gugagcaaaguuucGggugugu.................................	696	1	H01
....gugagcaaaguuucagguAugu.................................	28	1	H01
....gAgagcaaaguuucaggugugu.................................	229	1	H01
....gugagcaaaguuucagguUugu.................................	48	1	H01
....gugagcaaaguuucaggCgugu.................................	27	1	H01
....guAagcaaaguuucaggugugu.................................	12	1	H01
....gugagcaaaguuucagguguCu.................................	155	1	H01
....gugagcaCaguuucaggugugu.................................	8	1	H01
....gugagcaaaguuucCggugugu.................................	49	1	H01
....gCgagcaaaguuucaggugugu.................................	48	1	H01
....gugagcaaaCuuucaggugugu.................................	133	1	H01
....gugagcaaaguuuGaggugugu.................................	87	1	H01
....gugagUaaaguuucagguguguc................................	1	1	H01
....gugagcaaaguuucagguguguc................................	85	0	H01
....gugagcaaaguuucaggugugCc................................	1	1	H01
....gugagcaaaguuucaggugugGc................................	1	1	H01
....gugagcaaaguuucagguguguG................................	111	1	H01
....gugagcaaaguuucagguguUuc................................	1	1	H01
....gugagcaaaguuucaggugugAc................................	2	1	H01
....gugagcaaaguuucagguguCuc................................	1	1	H01
....gugagcaaaguuucGgguguguc................................	1	1	H01
....gugagcaaaguuucaUguguguc................................	1	1	H01
....gugagcaaaguuucaggugugucC...............................	71	1	H01
....gugagcaaaguuucagguguguUa...............................	157	1	H01
....gugagcaaaguuucagguguguGa...............................	4	1	H01
....gugagcaaaguuucagguguguca...............................	11	0	H01
....gugagcaaaguuucaggugugucU...............................	18	1	H01
....gugagcaaaguuucagguguguGag..............................	2	1	H01
....gugagcaaaguuucaggugugucCg..............................	3	1	H01
....ugagcaaaguuucaggugu...................................	2	0	H01
....ugagcaaaguuucaggugug..................................	4	0	H01
....Cgagcaaaguuucaggugugu.................................	1	1	H01
....ugagcaaaguuucaggugugu.................................	133	0	H01
....Ggagcaaaguuucaggugugu.................................	5	1	H01
....ugagcaaaguuucagguguguc................................	1	0	H01
....gCgcaaaguuucaggugu...................................	1	1	H01
....gagcaaaguuucaggugu...................................	6	0	H01
....gagcaaaguuucaggugug..................................	15	0	H01
....gagcaaaguuucagguguU..................................	5	1	H01
....gagcaaaguuucaggugugu.................................	401	0	H01
....gagcaaaguuucaggugugA.................................	2	1	H01
....gagcaaaguuucUggugugu.................................	1	1	H01
....gagcaaaguuucGggugugu.................................	1	1	H01
....gagcaaaguuucagguguUu.................................	4	1	H01
....gUgcaaaguuucaggugugu.................................	2	1	H01
....Uagcaaaguuucaggugugu.................................	3	1	H01
....Cagcaaaguuucaggugugu.................................	4	1	H01
....agcaaaguuucagguguU..................................	1	1	H01
....agcaaaguuucaggugug..................................	23	0	H01
....agcaaaguuucagUugug..................................	1	1	H01
....agcaaaguuucagguguUu.................................	5	1	H01
....aUcaaaguuucaggugugu.................................	1	1	H01
....agcaaaguuucagAugugu.................................	1	1	H01
....agcaaaguuucaggugugu.................................	455	0	H01
....agcaaaguuucagguCugu.................................	1	1	H01
....agUaaaguuucaggugugu.................................	2	1	H01
....agcaaaguuucaCgugugu.................................	1	1	H01
....gcaaaguuucaggugugu.................................	346	0	H01
....gcaaaguuucaggugugA.................................	2	1	H01
....gcaaagCuucaggugugu.................................	2	1	H01
....gUaaaguuucaggugugu.................................	2	1	H01
....gcaaaguuuUaggugugu.................................	1	1	H01
....Ucaaaguuucaggugugu.................................	1	1	H01
....................ccaucugaaagcuugucu..	2	0	LA5
....................ccaucugaaagcuugucuU...	1	1	LA5
....................ccaucugaaagcuugucuc...	8	0	LA5

```
                    Star                                    Mature
ugauaauuugcuucauugauccaucugaaagcuugucucaaccuuugaucugaacaggugagcaaaguuucaggugugucagagaaguuuauuuucaucuuauuaucaacua
....................ccaucugaGagcuugucuc.........................................................................   1    1   LA5
....................ccaucugaaagcuugucuca........................................................................   2    0   LA5
....................ccaucugaaagcuugucucaa.......................................................................   9    0   LA5
....................ccaucugaaagcuugucucaac......................................................................   44   0   LA5
....................ccaucugaaagcuugucucaaU......................................................................   4    1   LA5
....................ccaucugaaagcuugucucaacU.....................................................................   6    1   LA5
....................ccaucugaaagcuugCcucaacc.....................................................................   2    1   LA5
....................ccaucugaaagcCugucucaacc.....................................................................   5    1   LA5
....................ccaucugaaagcuugucucaacc.....................................................................   63   0   LA5
....................ccaucugaaagcuugucAcaacc.....................................................................   1    1   LA5
....................ccaucugaaaAcuugucucaacc.....................................................................   1    1   LA5
....................ccaucugaaagcuugucucaacA.....................................................................   4    1   LA5
....................ccaucugGaagcuugucucaaccu....................................................................   1    1   LA5
....................ccaucugaaagcuugucucaaccA....................................................................   14   1   LA5
....................ccaucuAaaagcuugucucaaccu....................................................................   1    1   LA5
....................ccaucugaaUgcuugucucaaccu....................................................................   2    1   LA5
....................ccaucugaaagcuCgucucaaccu....................................................................   2    1   LA5
....................ccaucugaaagcuugucucaaccG....................................................................   3    1   LA5
....................ccaucCgaaagcuugucucaaccu....................................................................   1    1   LA5
....................ccaucugaaagcuugCcucaaccu....................................................................   2    1   LA5
....................ccaucugaaagcuugucucaaUcu....................................................................   1    1   LA5
....................ccaucugaaGgcuugucucaaccu....................................................................   1    1   LA5
....................ccaucugaGagcuugucucaaccu....................................................................   1    1   LA5
....................ccaucugaaagcuugucucaaccu....................................................................   179  0   LA5
....................ccaucugaaagcuugucucaaccC....................................................................   21   1   LA5
....................ccaGcugaaagcuugucucaaccu....................................................................   3    1   LA5
....................ccaucugaaagcCugucucaaccu....................................................................   1    1   LA5
....................ccaucugUaagcuugucucaaccu....................................................................   1    1   LA5
....................ccaucugaaagcuugucucaaccuu...................................................................   3    0   LA5
....................ccaCcugaaagcuugucucaaccuuu..................................................................   2    1   LA5
.....................caucugaaagcuugucucaacc.....................................................................   1    0   LA5
.....................caCcugaaagcuugucucaaccuuu..................................................................   1    1   LA5
............................................uugaucugaacaggugagcaaaguuuc.........................................   1    0   LA5
........................................................ggugagcaaaguuucaggug....................................   1    0   LA5
........................................................ggugagcaaaguuucaggugC...................................   1    1   LA5
........................................................Cgugagcaaaguuucaggugugu.................................   15   1   LA5
.........................................................gugagcaaaguuucaggu.....................................   10   0   LA5
.........................................................gugagcaaaguuucagguA....................................   1    1   LA5
.........................................................gugagcaaaguuucaggug....................................   17   0   LA5
.........................................................gugagcaaaguuucaggugC...................................   8    1   LA5
.........................................................gugagcaaaguuucaggAgu...................................   1    1   LA5
.........................................................gugagcaaaguuucaggugG...................................   1    1   LA5
.........................................................Uugagcaaaguuucaggugu...................................   3    1   LA5
.........................................................gugagcGaaguuucaggugu...................................   1    1   LA5
.........................................................gugagcaGaguuucaggugu...................................   2    1   LA5
.........................................................gugagUaaaguuucaggugu...................................   1    1   LA5
.........................................................gugagcaaaguuucaggugu...................................   77   0   LA5
.........................................................gugagcaaUguuucaggugug..................................   1    1   LA5
.........................................................gugagcaaGguuucaggugug..................................   2    1   LA5
.........................................................gugagcaaaguuucagguguC..................................   1    1   LA5
.........................................................gugGgcaaaguuucaggugug..................................   1    1   LA5
.........................................................gugagcaaaguuucaggCgug..................................   2    1   LA5
.........................................................gugagcaaaguuucagguAug..................................   1    1   LA5
.........................................................gugagcaaaguuuAaggugug..................................   1    1   LA5
.........................................................gugagcaGaguuucaggugug..................................   1    1   LA5
.........................................................gugagcGaaguuucaggugug..................................   1    1   LA5
.........................................................gugagcaaaguuucaggugug..................................   251  0   LA5
.........................................................gugagcaaagAuucaggugug..................................   1    1   LA5
.........................................................gugagcaaaguuucagguguA..................................   23   1   LA5
.........................................................Augagcaaaguuucaggugug..................................   2    1   LA5
.........................................................gugagcaaaguuucaggugGg..................................   1    1   LA5
.........................................................gugagcaaaguuucagguguU..................................   7    1   LA5
.........................................................gugagcaaaguuCcaggugug..................................   2    1   LA5
.........................................................gugagcaaaguuucGggugug..................................   1    1   LA5
.........................................................gugagcaaaAuuucaggugugu.................................   1    1   LA5
.........................................................gugagcaaaguuucGggugugu.................................   30   1   LA5
.........................................................gugagcaaaguuucagguguAu.................................   6    1   LA5
.........................................................Augagcaaaguuucaggugugu.................................   6    1   LA5
.........................................................gugagcaaaguAucaggugugu.................................   2    1   LA5
.........................................................gugagcaGaguuucaggugugu.................................   22   1   LA5
```

```
                    Star                                 Mature
ugauaauuugcuucauugauccaucugaaagcuugucucaaccuuugaucugaacaggugagcaaaguuucaggugugucagagaaguuuauuuucaucuuauuaucaacua
.........................................................gugagcaaaguuucaggugugA.................................  238    1    LA5
.........................................................Cugagcaaaguuucaggugugu.................................  1      1    LA5
.........................................................gugagcaaagGuucaggugugu.................................  2      1    LA5
.........................................................guAagcaaaguuucaggugugu.................................  6      1    LA5
.........................................................Uugagcaaaguuucaggugugu.................................  4      1    LA5
.........................................................gugagcaaaguuucaggugugG.................................  105    1    LA5
.........................................................gugagcaaaguuucagAugugu.................................  7      1    LA5
.........................................................gugagUaaaguuucaggugugu.................................  9      1    LA5
.........................................................gugagcaaaguuucUggugugu.................................  4      1    LA5
.........................................................gugagcaaaguuucagguCugu.................................  2      1    LA5
.........................................................gugagcaaaguuucagguguUu.................................  3      1    LA5
.........................................................gugagcaaaguCucaggugugu.................................  19     1    LA5
.........................................................gugagcaaGguuucaggugugu.................................  21     1    LA5
.........................................................gAgagcaaaguuucaggugugu.................................  1      1    LA5
.........................................................gugagcaaaguuucaggugGgu.................................  8      1    LA5
.........................................................gugagcaaaguuucagguAugu.................................  2      1    LA5
.........................................................gugagAaaaguuucaggugugu.................................  1      1    LA5
.........................................................gugagcUaaguuucaggugugu.................................  1      1    LA5
.........................................................gugagcaaaguuCcaggugugu.................................  11     1    LA5
.........................................................gugagcaaaguuucaggugugu.................................  5980   0    LA5
.........................................................gCgagcaaaguuucaggugugu.................................  15     1    LA5
.........................................................gugagcaaaguuucaggugCgu.................................  12     1    LA5
.........................................................gugaAcaaaguuucaggugugu.................................  10     1    LA5
.........................................................gugagcaaaguuucaggCgugu.................................  20     1    LA5
.........................................................gugagcaaaguuucaggGgugu.................................  5      1    LA5
.........................................................gugagcaaaguuAcaggugugu.................................  2      1    LA5
.........................................................gugUgcaaaguuucaggugugu.................................  5      1    LA5
.........................................................gugagcGaaguuucaggugugu.................................  17     1    LA5
.........................................................gugagcaaagAuucaggugugu.................................  1      1    LA5
.........................................................gugagcaaUguuucaggugugu.................................  1      1    LA5
.........................................................gugagcaaaguuGcaggugugu.................................  2      1    LA5
.........................................................gugagcaCaguuucaggugugu.................................  1      1    LA5
.........................................................gugagcCaaguuucaggugugu.................................  1      1    LA5
.........................................................gugagcaaaguuucagguguCu.................................  1      1    LA5
.........................................................gugGgcaaaguuucaggugugu.................................  23     1    LA5
.........................................................gugagcaaaguuucaggAgugu.................................  4      1    LA5
.........................................................gugagcaaagCuucaggugugu.................................  19     1    LA5
.........................................................gugagcaaaguuuAaggugugu.................................  1      1    LA5
.........................................................gugagcaaaguuucaggugAgu.................................  3      1    LA5
.........................................................gugagcaaaguuucaAgugugu.................................  4      1    LA5
.........................................................gugagcaaaguuuUaggugugu.................................  6      1    LA5
.........................................................gugagcaaagCuucaggugugugc................................  1      1    LA5
.........................................................gugagcaaaguuucaggugugugG................................  7      1    LA5
.........................................................gugagcaaaguuucaggugugugc................................  20     0    LA5
..........................................................ugagcaaaguuucaggugC...................................  1      1    LA5
..........................................................ugagcaaaguuucaggugugu.................................  5      0    LA5
...........................................................gagcaaaguuucaggugugu.................................  3      0    LA5
............................................................agcaaaguuucaggugugA.................................  1      1    LA5

....................ccaucugaaagcuugucCc.........................................................................  1      1    HA1
....................cUaucugaaagcuugucuc.........................................................................  2      1    HA1
....................ccaucugaaagcuugucuc.........................................................................  5      0    HA1
....................ccaucugaaagcuAgucuc.........................................................................  1      1    HA1
....................ccaucugaaagcuugucuca........................................................................  7      0    HA1
....................ccaucugGaagcuugucucaa.......................................................................  1      1    HA1
....................ccaucugaaagcuugucucaa.......................................................................  5      0    HA1
....................ccaucugaaagcuugucucaaU......................................................................  2      1    HA1
....................ccaucugaaagcCugucucaac......................................................................  1      1    HA1
....................ccaucugaaagcuugucucaac......................................................................  34     0    HA1
....................ccaucugaaagcuugucucaacc.....................................................................  44     0    HA1
....................ccaucugaaagcuugucucaacA.....................................................................  2      1    HA1
....................ccaucugaaagcuugucucaacU.....................................................................  10     1    HA1
....................ccaucugaaagcuugucCcaaccu....................................................................  1      1    HA1
....................ccaucugaaagcuugCcucaaccu....................................................................  1      1    HA1
....................ccaucugaaagcuugucucaaccA....................................................................  3      1    HA1
....................ccaucugaaagcuugucucaaccG....................................................................  1      1    HA1
....................ccaucugaaagcuugucucaGccu....................................................................  2      1    HA1
....................ccaucugaaagcuugucucaaUcu....................................................................  1      1    HA1
....................ccaucugaaagcuugucucaaccu....................................................................  116    0    HA1
....................ccaucugaaagcuugucucaaccC....................................................................  17     1    HA1
```

```
                    Star                                 Mature
ugauaauuugcuucauugauccaucugaaagcuugucucaaccuuugaucugaacaggugagcaaaguuucaggugugucagagaaguuuauuuucaucuuauuaucaacua
....................ccaucugaaGgcuugucucaaccu....................................................................    1     1    HA1
....................ccaucugaaagcuugucucaaccuu...................................................................    8     0    HA1
....................ccaucugaaagcuugucucaaccuG...................................................................    1     1    HA1
....................ccaCcugaaagcuugucucaaccuuu..................................................................    3     1    HA1
....................ccaucugaaagcuugucucaaccuuA..................................................................    1     1    HA1
.....................caucugaaagcuugucucaa.......................................................................    1     0    HA1
.....................caCcugaaagcuugucucaaccuuu..................................................................    2     1    HA1
.......................................................aAgugagcaaaguuucaggugugu.................................    3     1    HA1
........................................................ggugagcaaaguuucaggugu...................................    9     0    HA1
........................................................Cgugagcaaaguuucaggugu...................................    2     1    HA1
........................................................ggugagcaaaguuucagguguA..................................    1     1    HA1
........................................................Cgugagcaaaguuucaggugug..................................    1     1    HA1
........................................................ggugagcaaaguuucaggugug..................................    3     0    HA1
........................................................ggugagcaaGguuucaggugugu.................................    1     1    HA1
........................................................ggugGgcaaaguuucaggugugu.................................    2     1    HA1
........................................................ggugagcaaaguuucaggugugu.................................    4     0    HA1
........................................................Agugagcaaaguuucaggugugu.................................    2     1    HA1
........................................................Cgugagcaaaguuucaggugugu.................................    22    1    HA1
........................................................Ugugagcaaaguuucaggugugu.................................    4     1    HA1
........................................................ggugagcaaaguuucagguguUu.................................    1     1    HA1
.........................................................gugagcaaaguuucaggA.....................................    2     1    HA1
.........................................................gugagcaaaguuucaggC.....................................    2     1    HA1
.........................................................gugagcaaaguuucaggu.....................................    39    0    HA1
.........................................................gugagcaaaguuuUaggug....................................    1     1    HA1
.........................................................gugagcaaaguuucagguU....................................    6     1    HA1
.........................................................gugagcaaaguuucagguA....................................    14    1    HA1
.........................................................gugagcaaaguuCcaggug....................................    2     1    HA1
.........................................................gugagcaaaguuucaggCg....................................    2     1    HA1
.........................................................gugagcaaaguuucCggug....................................    1     1    HA1
.........................................................gugagcaaaguuucaggug....................................    144   0    HA1
.........................................................gugagcaaGguuucaggug....................................    2     1    HA1
.........................................................gugagcaaaguuucaggugu...................................    312   0    HA1
.........................................................gugagcaaUguuucaggugu...................................    1     1    HA1
.........................................................gugagcaaaguuucaggugC...................................    39    1    HA1
.........................................................gugagcaaagGuucaggugu...................................    1     1    HA1
.........................................................Augagcaaaguuucaggugu...................................    1     1    HA1
.........................................................gugagUaaaguuucaggugu...................................    1     1    HA1
.........................................................gugagcaaagCuucaggugu...................................    2     1    HA1
.........................................................gCgagcaaaguuucaggugu...................................    1     1    HA1
.........................................................gugagcGaaguuucaggugu...................................    2     1    HA1
.........................................................gAgagcaaaguuucaggugu...................................    1     1    HA1
.........................................................gugagcaaaguuucGggugu...................................    1     1    HA1
.........................................................gugagcaaaguuucaggugG...................................    6     1    HA1
.........................................................gugagcaaaUuuucaggugu...................................    1     1    HA1
.........................................................gugagcaaaguuucagguUu...................................    1     1    HA1
.........................................................gugagAaaaguuucaggugu...................................    1     1    HA1
.........................................................gugagcaGaguuucaggugu...................................    2     1    HA1
.........................................................gugagcaaGguuucaggugu...................................    1     1    HA1
.........................................................gugagcaaaguuucaggCgu...................................    2     1    HA1
.........................................................gugagcaaaguuucaggGgu...................................    2     1    HA1
.........................................................gugagcaGaguuucaggugug..................................    4     1    HA1
.........................................................Uugagcaaaguuucaggugug..................................    2     1    HA1
.........................................................gugagcaaaguCucaggugug..................................    4     1    HA1
.........................................................gugagcGaaguuucaggugug..................................    2     1    HA1
.........................................................gugGgcaaaguuucaggugug..................................    4     1    HA1
.........................................................gugagcaaaguuucaggGgug..................................    4     1    HA1
.........................................................gugagcaaaguuucagguguC..................................    1     1    HA1
.........................................................gugagcaaaguuucGggugug..................................    13    1    HA1
.........................................................gugagcaaaguuCcaggugug..................................    5     1    HA1
.........................................................gugagcaUaguuucaggugug..................................    2     1    HA1
.........................................................gugagcaCaguuucaggugug..................................    1     1    HA1
.........................................................gugagcaaaguuucaggugug..................................    1224  0    HA1
.........................................................gugagcaaaguuucaggCgug..................................    6     1    HA1
.........................................................gAgagcaaaguuucaggugug..................................    1     1    HA1
.........................................................gugagcaaaguuucaggugCg..................................    14    1    HA1
.........................................................gCgagcaaaguuucaggugug..................................    3     1    HA1
.........................................................gugagcaaaguuucagguguU..................................    35    1    HA1
.........................................................gugagcaaaguuucaggugGg..................................    3     1    HA1
.........................................................gugUgcaaaguuucaggugug..................................    1     1    HA1
.........................................................gugagcaaaguuucagguguA..................................    122   1    HA1
```

Star Mature

```
ugauaauuugcuucauugauccaucugaaagcuugucucaaccuuugaucugaacaggugagcaaaguuucaggugugucagagaaguuuauuuucaucuuauuaucaacua
.........................................................gugagAaaaguuucaggugug.................................. 1 1 HA1
.........................................................gugagcaaaguuucaggugAg.................................. 2 1 HA1
.........................................................guAagcaaaguuucaggugug.................................. 1 1 HA1
.........................................................gugagcaaaguuuUaggugug.................................. 5 1 HA1
.........................................................gugagcaaGguuucaggugug.................................. 6 1 HA1
.........................................................gugagcaaaguuucaUgugug.................................. 1 1 HA1
.........................................................gugagcaaaguuucaggAgug.................................. 1 1 HA1
.........................................................gugagcaaaguuucagguCug.................................. 1 1 HA1
.........................................................gugagcaaagCuucaggugug.................................. 3 1 HA1
.........................................................gugagcaaaguuucGggugugu................................. 96 1 HA1
.........................................................gugagcaaaguuucagUugugu................................. 1 1 HA1
.........................................................gugagcaaaguuucagguUugu................................. 2 1 HA1
.........................................................gugagcaaaguuucagguCugu................................. 2 1 HA1
.........................................................gugagcaaaguuucagguAugu................................. 10 1 HA1
.........................................................guAagcaaaguuucaggugugu................................. 12 1 HA1
.........................................................gugagcaaUguuucaggugugu................................. 7 1 HA1
.........................................................gugCgcaaaguuucaggugugu................................. 4 1 HA1
.........................................................gugagcaaaguuucCggugugu................................. 4 1 HA1
.........................................................gugagcaaaguuGcaggugugu................................. 2 1 HA1
.........................................................gugagcaaaguuucaggugugA................................. 695 1 HA1
.........................................................gugagcaaaguuucaAgugugu................................. 17 1 HA1
.........................................................gugagcaaaguuucagCugugu................................. 1 1 HA1
.........................................................gugagcaaaguuucaCgugugu................................. 1 1 HA1
.........................................................gugagcUaaguuucaggugugu................................. 3 1 HA1
.........................................................gugagcCaaguuucaggugugu................................. 2 1 HA1
.........................................................gugagcaaaguCucaggugugu................................. 41 1 HA1
.........................................................gugagcaaaguuucaggugGgu................................. 65 1 HA1
.........................................................gugagcaaCguuucaggugugu................................. 1 1 HA1
.........................................................gugagcaaaguuucaggugugu................................. 19632 0 HA1
.........................................................gugagcaaaguuucaggugAgu................................. 14 1 HA1
.........................................................gugagAaaaguuucaggugugu................................. 6 1 HA1
.........................................................gugagcaaaCuuucaggugugu................................. 2 1 HA1
.........................................................gGgagcaaaguuucaggugugu................................. 2 1 HA1
.........................................................gugUgcaaaguuucaggugugu................................. 7 1 HA1
.........................................................gugagcaaaguuucagguguAu................................. 22 1 HA1
.........................................................gugagcGaaguuucaggugugu................................. 57 1 HA1
.........................................................gugagcaaaguuucaggugugG................................. 583 1 HA1
.........................................................gugagcaaaguuuGaggugugu................................. 1 1 HA1
.........................................................gugagcaaaguuucagguguUu................................. 15 1 HA1
.........................................................gugagcaaagCuucaggugugu................................. 47 1 HA1
.........................................................gugaUcaaaguuucaggugugu................................. 7 1 HA1
.........................................................gugagcaaaguuucagAugugu................................. 6 1 HA1
.........................................................gugaAcaaaguuucaggugugu................................. 11 1 HA1
.........................................................gugagcaaaguuAcaggugugu................................. 8 1 HA1
.........................................................gAgagcaaaguuucaggugugu................................. 13 1 HA1
.........................................................gugagcaaGguuucaggugugu................................. 87 1 HA1
.........................................................gugagcaaaguuucaggCgugu................................. 84 1 HA1
.........................................................gugagcaaaguuucUggugugu................................. 19 1 HA1
.........................................................gugagcaaaUuuucaggugugu................................. 2 1 HA1
.........................................................gugagcaaaguuucaUgugugu................................. 5 1 HA1
.........................................................gugagGaaaguuucaggugugu................................. 4 1 HA1
.........................................................gugagcaaaguAucaggugugu................................. 5 1 HA1
.........................................................gCgagcaaaguuucaggugugu................................. 50 1 HA1
.........................................................gugagUaaaguuucaggugugu................................. 25 1 HA1
.........................................................gugagcaaaAuuucaggugugu................................. 6 1 HA1
.........................................................gugagcaaagGuucaggugugu................................. 13 1 HA1
.........................................................gugagcaaaguuucagguguCu................................. 8 1 HA1
.........................................................gugagcaaaguuucaggAgugu................................. 16 1 HA1
.........................................................gugGgcaaaguuucaggugugu................................. 94 1 HA1
.........................................................guUagcaaaguuucaggugugu................................. 1 1 HA1
.........................................................gugagcaCaguuucaggugugu................................. 4 1 HA1
.........................................................gugagcaaaguuuUaggugugu................................. 20 1 HA1
.........................................................gugagcaaaguuCcaggugugu................................. 59 1 HA1
.........................................................gugagcaaaguuucaggGgugu................................. 21 1 HA1
.........................................................Uugagcaaaguuucaggugugu................................. 13 1 HA1
.........................................................gugagcaUaguuucaggugugu................................. 16 1 HA1
.........................................................gugagcaaaguuucaggugCgu................................. 63 1 HA1
.........................................................Augagcaaaguuucaggugugu................................. 26 1 HA1
.........................................................gugagcaGaguuucaggugugu................................. 77 1 HA1
.........................................................gugagcGaaguuucagguguguc................................ 1 1 HA1
```

Star **Mature**

ugauaauuugcuucauugauccaucugaaagcuugucucaaccuuugaucugaacaggugagcaaaguuucaggugugucagagaaguuuauuucaucuuauuaucaacua			
....gugagcaaaguuucaggugugAc................................	1	1	HA1
....gugagcaaaguuucaggugguguG...............................	25	1	HA1
....gugagcaaaguuCcagguguguc...............................	2	1	HA1
....gCgagcaaaguuucagguguguc...............................	1	1	HA1
....gugagcaaaguuucagguguguc...............................	53	0	HA1
....gugagcaaaguuucaggugCguc...............................	1	1	HA1
....gugagcaaaguuucaggugugUa..............................	3	1	HA1
....ugagcaaaguuucaggugu...................................	1	0	HA1
....ugagcaaaguuucaggugug..................................	1	0	HA1
....ugagcaaaguuucaggugugu................................	10	0	HA1
....Cgagcaaaguuucaggugugu................................	1	1	HA1
....ugagcaaaguuucaggugugA................................	1	1	HA1
....gagcaaaguuucaggugugu................................	13	0	HA1
....gagcaaaguuucGggugugu................................	1	1	HA1
....agcaaaguuucaggugugu................................	1	0	HA1
....gcaaaguuucaggugugu................................	3	0	HA1
....................ccaucugaaagcuugucuc..	3	0	BJ1
....................ccaucugaaagcuugucucaac...	8	0	BJ1
....................ccaucugaaagcuugucucaacc..	17	0	BJ1
....................ccaucugaaagcuugucucaacA..	2	1	BJ1
....................Acaucugaaagcuugucucaacc..	1	1	BJ1
....................ccaucugaaagcuugucucaaccu...	3	0	BJ1
....................ccaucugaaagcuugCcucaaccu...	1	1	BJ1
....................ccaucugaaagcuugucucaaccuu..	1	0	BJ1
....Cgugagcaaaguuucaggugugu................................	1	1	BJ1
....gugagcaaaguuucaggugu..................................	5	0	BJ1
....gugagcaaaguuucaggugC..................................	2	1	BJ1
....gugagcaaaguuucaggugug.................................	23	0	BJ1
....gugagcaaaguuucagguguA.................................	1	1	BJ1
....gugagcaaaguuucaggugCg.................................	1	1	BJ1
....gugagcaaagCuucaggugug.................................	1	1	BJ1
....gugGgcaaaguuucaggugugu................................	2	1	BJ1
....gugagcaaaguuucagAugugu................................	1	1	BJ1
....gugagcaaaguuucaggAgugu................................	1	1	BJ1
....gugagcaaGguuucaggugugu................................	1	1	BJ1
....gugagcaaaguuCcaggugugu................................	1	1	BJ1
....gugagcaaaguuucGggugugu................................	2	1	BJ1
....gugaUcaaaguuucaggugugu................................	2	1	BJ1
....gugagcaaagCuucaggugugu................................	4	1	BJ1
....gugagcaaaguuucaggugugA................................	7	1	BJ1
....gCgagcaaaguuucaggugugu................................	3	1	BJ1
....gugagcaaaguuuAaggugugu................................	2	1	BJ1
....gugagcaaCguuucaggugugu................................	2	1	BJ1
....gugagGaaaguuucaggugugu................................	1	1	BJ1
....gugaAcaaaguuucaggugugu................................	1	1	BJ1
....gugagcUaaguuucaggugugu................................	1	1	BJ1
....gugagcaaaguCucaggugugu................................	1	1	BJ1
....gugagcGaaguuucaggugugu................................	1	1	BJ1
....gugagcaaaguuucaggugugu................................	250	0	BJ1
....Augagcaaaguuucaggugugu................................	1	1	BJ1
....gAgagcaaaguuucaggugugu................................	2	1	BJ1
....guAagcaaaguuucaggugugu................................	1	1	BJ1
....gugagcaaaguuucaggugCgu................................	1	1	BJ1
....gugagcaaaguuucaCgugugu................................	1	1	BJ1
....Cgagcaaaguuucaggugugu................................	1	1	BJ1
....ugagcaaaguuucGggugugu................................	1	1	BJ1
....................ccaucuAaaagcuugucuc..	1	1	TJ1
....................ccaucugaaagcuugucuA..	1	1	TJ1
....................ccaucugaaagcuugucuca...	1	0	TJ1
....................ccaucugaaagcuugucucaac...	2	0	TJ1
....................ccaucugaaagcuugucucaacc..	7	0	TJ1
....................ccaucugaaagcuugucucaacU..	3	1	TJ1
....................ccaucugaaagcuugucucaaccC...	1	1	TJ1
....................ccaucugaaagcuugucCcaaccu...	1	1	TJ1
....................ccaucugaaagcuugucucaaccu...	11	0	TJ1
....................ccaucugaaagcuugucucaaccA...	1	1	TJ1
....................ccaucugaaagcuugucucaaccG...	1	1	TJ1
....aAgugagcaaaguuucaggugugu................................	1	1	TJ1

```
                    Star                                  Mature
ugauaauuugcuucauugauccaucugaaagcuugucucaaccuuugaucugaacaggugagcaaaguuucaggugugucagagaaguuuauuuucaucuuauuaucaacua
........................................................ggugagcaaaguuucaggugug..................................    2      0      TJ1
........................................................Cgugagcaaaguuucaggugugu.................................    8      1      TJ1
........................................................ggugagcaaaguuucaggugugA.................................    1      1      TJ1
........................................................ggugagcaaaguuucaggugugu.................................    1      0      TJ1
.........................................................Augagcaaaguuucaggu.....................................    1      1      TJ1
.........................................................guUagcaaaguuucaggu.....................................    1      1      TJ1
.........................................................gugagcaaaguuucaggA.....................................    3      1      TJ1
.........................................................gugagcaaaguuucaggC.....................................    3      1      TJ1
.........................................................gugagcaaaguuucaggu.....................................    36     0      TJ1
.........................................................gugagcaaaguuucagguC....................................    1      1      TJ1
.........................................................gugCgcaaaguuucaggug....................................    1      1      TJ1
.........................................................gugagcaaaguuCcaggug....................................    1      1      TJ1
.........................................................gugagcaaaguuucaggug....................................    125    0      TJ1
.........................................................gugagcaaaguuucaggCg....................................    1      1      TJ1
.........................................................gugagcaaaguuucGggug....................................    1      1      TJ1
.........................................................gugGgcaaaguuucaggug....................................    2      1      TJ1
.........................................................gugagcaaagCuucaggug....................................    1      1      TJ1
.........................................................gugagcaaaguuucagguU....................................    4      1      TJ1
.........................................................gugagcaaaguuucagguA....................................    14     1      TJ1
.........................................................gugagcaaaCuuucaggug....................................    2      1      TJ1
.........................................................gugagUaaaguuucaggugu...................................    1      1      TJ1
.........................................................gugagcaaaguuucaggGgu...................................    4      1      TJ1
.........................................................gugagcaaaguuuUaggugu...................................    2      1      TJ1
.........................................................gugagcaaaguuucaggugG...................................    10     1      TJ1
.........................................................gAgagcaaaguuucaggugu...................................    1      1      TJ1
.........................................................guAagcaaaguuucaggugu...................................    2      1      TJ1
.........................................................gugagcaaGguuucaggugu...................................    1      1      TJ1
.........................................................gGgagcaaaguuucaggugu...................................    1      1      TJ1
.........................................................gugagcaaagCuucaggugu...................................    1      1      TJ1
.........................................................gugagcaaaguuucGggugu...................................    1      1      TJ1
.........................................................gugagcGaaguuucaggugu...................................    2      1      TJ1
.........................................................gCgagcaaaguuucaggugu...................................    1      1      TJ1
.........................................................Augagcaaaguuucaggugu...................................    1      1      TJ1
.........................................................gugagcaaaguuucaggugC...................................    48     1      TJ1
.........................................................gugagcaaaguuucUggugu...................................    1      1      TJ1
.........................................................gugagcaaaguuucaggugu...................................    245    0      TJ1
.........................................................gugagcaaaguuGcaggugug..................................    1      1      TJ1
.........................................................gugagcaaaguuCcaggugug..................................    10     1      TJ1
.........................................................gugagcUaaguuucaggugug..................................    1      1      TJ1
.........................................................guUagcaaaguuucaggugug..................................    1      1      TJ1
.........................................................gGgagcaaaguuucaggugug..................................    1      1      TJ1
.........................................................gugagcaaaCuuucaggugug..................................    1      1      TJ1
.........................................................Augagcaaaguuucaggugug..................................    2      1      TJ1
.........................................................gugagcaaaguuucagguguA..................................    242    1      TJ1
.........................................................gugUgcaaaguuucaggugug..................................    1      1      TJ1
.........................................................gugagcaaaguuucaggugCg..................................    11     1      TJ1
.........................................................gugagcaaaguuucagguguC..................................    11     1      TJ1
.........................................................gugaAcaaaguuucaggugug..................................    3      1      TJ1
.........................................................gugagcaaaguuucagguguU..................................    54     1      TJ1
.........................................................gugagcaaaguuucaggGgug..................................    1      1      TJ1
.........................................................gugagcaaaguuuUaggugug..................................    3      1      TJ1
.........................................................gugagcaGaguuucaggugug..................................    6      1      TJ1
.........................................................gugagcaaaguuucaUgugug..................................    1      1      TJ1
.........................................................gugagcaaaguuucaggugAg..................................    1      1      TJ1
.........................................................gugGgcaaaguuucaggugug..................................    12     1      TJ1
.........................................................gugagcaaaAuuucaggugug..................................    1      1      TJ1
.........................................................gugagcGaaguuucaggugug..................................    9      1      TJ1
.........................................................gugagcaaaguuucagguAug..................................    8      1      TJ1
.........................................................gugagcaaaguuucagguUug..................................    2      1      TJ1
.........................................................gugagcaaaguuucaAgugug..................................    1      1      TJ1
.........................................................gugagcaCaguuucaggugug..................................    1      1      TJ1
.........................................................gugagcaaagCuucaggugug..................................    6      1      TJ1
.........................................................gugagcaaaguuucUggugug..................................    2      1      TJ1
.........................................................gugagcaaaguuucaCgugug..................................    2      1      TJ1
.........................................................gugagUaaaguuucaggugug..................................    2      1      TJ1
.........................................................gugagcaaaguuucaggCgug..................................    5      1      TJ1
.........................................................gugagcaaaguCucaggugug..................................    6      1      TJ1
.........................................................gugagcaaaguuucaggugGg..................................    5      1      TJ1
.........................................................guAagcaaaguuucaggugug..................................    2      1      TJ1
.........................................................gCgagcaaaguuucaggugug..................................    6      1      TJ1
```

Star **Mature**

```
ugauaauuugcuucauugauccaucugaaagcuugucucaaccuuugaucugaacaggugagcaaaguuucaggugugucagagaaguuuauuuucaucuuauuaucaacua
.........................................................gugagcaaaguuucaggugug...................................      1998    0    TJ1
.........................................................gugagcaaGguuucaggugug...................................      10      1    TJ1
.........................................................gugagAaaaguuucaggugug...................................      5       1    TJ1
.........................................................gugagcaUaguuucaggugug...................................      3       1    TJ1
.........................................................gugagcaaaguuucagguCug...................................      1       1    TJ1
.........................................................gugagcaaaguuucGggugug...................................      4       1    TJ1
.........................................................gugagGaaaguuucaggugugu.................................      4       1    TJ1
.........................................................gugagcaaaguuuGaggugugu.................................      2       1    TJ1
.........................................................gugagcaaaAuuucaggugugu.................................      9       1    TJ1
.........................................................gugagcaaGguuucaggugugu.................................      27      1    TJ1
.........................................................gugagcaaaguuucaggAgugu.................................      4       1    TJ1
.........................................................gugagcaUaguuucaggugugu.................................      3       1    TJ1
.........................................................gugagcaaaUuuucaggugugu.................................      8       1    TJ1
.........................................................gugagcaaaguuucCggugugu.................................      2       1    TJ1
.........................................................gugagcaaagAuucaggugugu.................................      2       1    TJ1
.........................................................gugagcaaaguuucagguguUu.................................      18      1    TJ1
.........................................................gugagcaaaguuuAaggugugu.................................      1       1    TJ1
.........................................................gugagcaaaguuucaggugAgu.................................      4       1    TJ1
.........................................................gugagcUaaguuucaggugugu.................................      5       1    TJ1
.........................................................gugagAaaaguuucaggugugu.................................      7       1    TJ1
.........................................................gugagcaaaguuucagUugugu.................................      1       1    TJ1
.........................................................guAagcaaaguuucaggugugu.................................      30      1    TJ1
.........................................................gugagcaaaguuucaAgugugu.................................      4       1    TJ1
.........................................................gugagcCaaguuucaggugugu.................................      2       1    TJ1
.........................................................gugagcaaaguuucaggugugG.................................      202     1    TJ1
.........................................................gugagcaaaguuucagguguCu.................................      1       1    TJ1
.........................................................gugagcaaaguuCcaggugugu.................................      20      1    TJ1
.........................................................gAgagcaaaguuucaggugugu.................................      5       1    TJ1
.........................................................gCgagcaaaguuucaggugugu.................................      11      1    TJ1
.........................................................gugCgcaaaguuucaggugugu.................................      1       1    TJ1
.........................................................gugagcaaaguuucaggugCgu.................................      22      1    TJ1
.........................................................gugagcaaaguuGcaggugugu.................................      1       1    TJ1
.........................................................gugagcaaaguuucagguguAu.................................      18      1    TJ1
.........................................................gugagcaaaguCucaggugugu.................................      10      1    TJ1
.........................................................gugagcaaaguuucaggugGgu.................................      8       1    TJ1
.........................................................gugagUaaaguuucaggugugu.................................      12      1    TJ1
.........................................................gugagcaaaguuucaggCgugu.................................      21      1    TJ1
.........................................................gugagcaaaCuuucaggugugu.................................      1       1    TJ1
.........................................................gugagcaaaguuuUaggugugu.................................      7       1    TJ1
.........................................................Augagcaaaguuucaggugugu.................................      9       1    TJ1
.........................................................gugagcGaaguuucaggugugu.................................      37      1    TJ1
.........................................................gugaAcaaaguuucaggugugu.................................      7       1    TJ1
.........................................................gugagcaaaguuucUggugugu.................................      11      1    TJ1
.........................................................gugagcaGaguuucaggugugu.................................      23      1    TJ1
.........................................................gugagcaaaguuAcaggugugu.................................      1       1    TJ1
.........................................................guCagcaaaguuucaggugugu.................................      2       1    TJ1
.........................................................gugagcaaaguuucaggugugu.................................      7220    0    TJ1
.........................................................gugagcaaagGuucaggugugu.................................      2       1    TJ1
.........................................................gugagcaaaguuucaggGgugu.................................      5       1    TJ1
.........................................................gGgagcaaaguuucaggugugu.................................      2       1    TJ1
.........................................................Uugagcaaaguuucaggugugu.................................      5       1    TJ1
.........................................................gugagcaaaguuucaggugugA.................................      372     1    TJ1
.........................................................gugagcaaaguuucaUgugugu.................................      2       1    TJ1
.........................................................gugGgcaaaguuucaggugugu.................................      29      1    TJ1
.........................................................gugagcaaaguuucagguAugu.................................      19      1    TJ1
.........................................................gugagcaaagCuucaggugugu.................................      15      1    TJ1
.........................................................gugUgcaaaguuucaggugugu.................................      1       1    TJ1
.........................................................guUagcaaaguuucaggugugu.................................      5       1    TJ1
.........................................................gugagcaaaguuucagAugugu.................................      7       1    TJ1
.........................................................gugagcaaaguuucGggugugu.................................      34      1    TJ1
.........................................................gugagcaaUguuucaggugugu.................................      3       1    TJ1
.........................................................gugagcaaaguuucagguguguG................................      62      1    TJ1
.........................................................gugagcaaaguuucaggugugugc...............................      27      0    TJ1
.........................................................gugagcaaaguuucaggugugCc................................      1       1    TJ1
.........................................................gugagcaaaguuucagguguguGa...............................      1       1    TJ1
.........................................................gugagcaaaguuucaggugugucU...............................      1       1    TJ1
.........................................................gugagcaaaguuucaggugugucGga.............................      1       1    TJ1
..........................................................ugagcaaaguuucaggugug...................................      2       0    TJ1
..........................................................ugagcaaaguuucagguguA..................................      1       1    TJ1
..........................................................ugagcaaaguuucaggugugu.................................      8       0    TJ1
```

```
                    Star                                Mature
ugauaauuugcuucauugauccaucugaaagcuugucucaaccuuugaucugaacaggugagcaaaguuucaggugugucagagaaguuuauuuucaucuuauuaucaacua
...........................................................gagcaaaguuucaggugC...................................    1    1    TJ1
...........................................................gagcaaaguuucaggugugu.................................    3    0    TJ1

...................uccaCcugaaagcuugucucaaccu....................................................................    1    1    B01
....................ccaucugaaagcuugucuca........................................................................    3    0    B01
....................cUaucugaaagcuugucucaac......................................................................    1    1    B01
....................ccaucugaaagcuugucucaac......................................................................    8    0    B01
....................ccaucugaaagcuugucucaacc.....................................................................    101  0    B01
....................ccaucugaaagcuugucucaaccG....................................................................    1    1    B01
....................ccaucugaaagcuugucucaaccu....................................................................    476  0    B01
....................ccUucugaaagcuugucucaaccu....................................................................    1    1    B01
....................ccaucugaaaAcuugucucaaccu....................................................................    1    1    B01
....................ccaucugaaagUuugucucaaccu....................................................................    1    1    B01
....................ccaucugaaagcuugucucaaccuu...................................................................    6    0    B01
....................Ucaucugaaagcuugucucaaccuu...................................................................    1    1    B01
....................ccaucugaaagcuugucucaaccuA...................................................................    6    1    B01
....................ccaCcugaaagcuugucucaaccuuug.................................................................    1    1    B01
....................ccaucugaaagcuugucucaaccuuug.................................................................    1    0    B01
....................ccaucugaaagcuugucucaaccuuuU.................................................................    4    1    B01
.....................caucugGaagcuugucucaaccu....................................................................    1    1    B01
......................aucugaaagcuugucucaaccu....................................................................    1    0    B01
.......................uUugaaagcuugucucaaccu....................................................................    1    1    B01
.........................ugaaagcuugucucaaccu....................................................................    1    0    B01
..........................gaaagcuugucucaaccu....................................................................    2    0    B01
..........................................cuuugaucugaacaggugagc.................................................    1    0    B01
........................................................Ugugagcaaaguuucaggu.....................................    1    1    B01
........................................................Ugugagcaaaguuucaggug....................................    1    1    B01
........................................................Ugugagcaaaguuucaggugug..................................    4    1    B01
........................................................ggugagcaaaguuucaggugug..................................    1    0    B01
........................................................Ugugagcaaaguuucaggugugu.................................    46   1    B01
........................................................Agugagcaaaguuucaggugugu.................................    2    1    B01
........................................................ggugagcaaaguuucaggugugu.................................    1    0    B01
........................................................Cgugagcaaaguuucaggugugu.................................    1    1    B01
.........................................................gugagcaaaguuucaggu.....................................    220  0    B01
.........................................................gugagcaaaguuucagguU....................................    7    1    B01
.........................................................gugagcaaaguuucagguC....................................    2    1    B01
.........................................................gugagcaaaguuucagUug....................................    1    1    B01
.........................................................gugagcaaaguuucagguA....................................    3    1    B01
.........................................................gugGgcaaaguuucaggug....................................    1    1    B01
.........................................................gugagcaaaguuucaggug....................................    76   0    B01
.........................................................gugagcaaaguuucaUgugu...................................    2    1    B01
.........................................................gugagcaaaguuucagAugu...................................    1    1    B01
.........................................................gugagcaaaguuucagguUu...................................    1    1    B01
.........................................................gugagcaaaguuucaggugC...................................    3    1    B01
.........................................................gugagcaaaguuuUaggugu...................................    2    1    B01
.........................................................gugagcaaaguuucagUugu...................................    1    1    B01
.........................................................gugagcaaaguuucaggugu...................................    998  0    B01
.........................................................gugagcaaaguuucaAgugu...................................    1    1    B01
.........................................................gCgagcaaaguuucaggugug..................................    3    1    B01
.........................................................Cugagcaaaguuucaggugug..................................    1    1    B01
.........................................................gugGgcaaaguuucaggugug..................................    1    1    B01
.........................................................gugagcaaaguuuUaggugug..................................    5    1    B01
.........................................................gugagcaaaguuucagguguA..................................    7    1    B01
.........................................................gugagUaaaguuucaggugug..................................    2    1    B01
.........................................................gugagcaaaguuucagguguC..................................    1    1    B01
.........................................................gugagcaaaguuCcaggugug..................................    2    1    B01
.........................................................gugagcaaaguuucaggugCg..................................    1    1    B01
.........................................................gugagcaaaguuucagguUug..................................    1    1    B01
.........................................................gugagcaaaCuuucaggugug..................................    1    1    B01
.........................................................gugagAaaaguuucaggugug..................................    1    1    B01
.........................................................gugaAcaaaguuucaggugug..................................    1    1    B01
.........................................................Augagcaaaguuucaggugug..................................    1    1    B01
.........................................................gugagcaaaguuucagguguU..................................    1074 1    B01
.........................................................gugagcaaagCuucaggugug..................................    2    1    B01
.........................................................gugagcaaaguuucaggGgug..................................    1    1    B01
.........................................................guCagcaaaguuucaggugug..................................    2    1    B01
.........................................................gugagcaaaguuucaggugGg..................................    2    1    B01
.........................................................gugagcaaaguuucGggugug..................................    4    1    B01
.........................................................gugaCcaaaguuucaggugug..................................    1    1    B01
.........................................................gugagcaaaguuucaggugug..................................    4216 0    B01
```

Star **Mature**

```
ugauaauuugcuucauugauccaucugaaagcuugucucaaccuuugaucugaacaggugagcaaaguuucaggugugucagagaaguuuauuuucaucuuauuaucaacua
.........................................................guCagcaaaguuucaggugugu.................................	9	1	B01
.........................................................gugagUaaaguuucaggugugu.................................	25	1	B01
.........................................................gugagcGaaguuucaggugugu.................................	4	1	B01
.........................................................gugagcaaaguuucaggugCgu.................................	13	1	B01
.........................................................gugagcaaaguGucaggugugu.................................	5	1	B01
.........................................................gugagcaaaguuucaggugugu.................................	42560	0	B01
.........................................................gugUgcaaaguuucaggugugu.................................	6	1	B01
.........................................................gugagcaaaguuucagguguAu.................................	10	1	B01
.........................................................gugCgcaaaguuucaggugugu.................................	12	1	B01
.........................................................gugagcaaagGuucaggugugu.................................	8	1	B01
.........................................................gugagcaaaguuucaggGgugu.................................	7	1	B01
.........................................................gugagcaaaguuuAaggugugu.................................	1	1	B01
.........................................................Uugagcaaaguuucaggugugu.................................	8	1	B01
.........................................................gugagcaGaguuucaggugugu.................................	7	1	B01
.........................................................gugagcaaaguuucagguguUu.................................	1794	1	B01
.........................................................gugagcaaaguCucaggugugu.................................	7	1	B01
.........................................................gugagcaaaguuucaggugugG.................................	77	1	B01
.........................................................gGgagcaaaguuucaggugugu.................................	6	1	B01
.........................................................gugagcaaaCuuucaggugugu.................................	2	1	B01
.........................................................gCgagcaaaguuucaggugugu.................................	4	1	B01
.........................................................gugagcaaaguuucCggugugu.................................	3	1	B01
.........................................................gugagcaaaguuucagguguCu.................................	22	1	B01
.........................................................gugagcaaCguuucaggugugu.................................	2	1	B01
.........................................................gugagcaaaguuucagguAugu.................................	1	1	B01
.........................................................gugagcaaaguuucGggugugu.................................	23	1	B01
.........................................................gugagcaaUguuucaggugugu.................................	8	1	B01
.........................................................gugagcaaaguuucaUgugugu.................................	7	1	B01
.........................................................gugagcaaGguuucaggugugu.................................	1	1	B01
.........................................................gugagcaaaguuucaggCgugu.................................	4	1	B01
.........................................................gAgagcaaaguuucaggugugu.................................	4	1	B01
.........................................................gugagcaaaguuucaggugugA.................................	3600	1	B01
.........................................................gugagcaaaguuucagCugugu.................................	6	1	B01
.........................................................gugagcaaagCuucaggugugu.................................	4	1	B01
.........................................................gugagAaaaguuucaggugugu.................................	1	1	B01
.........................................................gugagcaaaguuucagUugugu.................................	12	1	B01
.........................................................gugagcaaaguuuUaggugugu.................................	48	1	B01
.........................................................guUagcaaaguuucaggugugu.................................	19	1	B01
.........................................................guAagcaaaguuucaggugugu.................................	1	1	B01
.........................................................gugagGaaaguuucaggugugu.................................	1	1	B01
.........................................................gugagcaaaguuucagAugugu.................................	1	1	B01
.........................................................gugagcaaaguuuGaggugugu.................................	2	1	B01
.........................................................gugGgcaaaguuucaggugugu.................................	5	1	B01
.........................................................Cugagcaaaguuucaggugugu.................................	3	1	B01
.........................................................gugaAcaaaguuucaggugugu.................................	17	1	B01
.........................................................gugaUcaaaguuucaggugugu.................................	4	1	B01
.........................................................gugagcaaaguuucaAgugugu.................................	3	1	B01
.........................................................gugagcaaaguuucUggugugu.................................	8	1	B01
.........................................................gugagcaaaguuucagguCugu.................................	5	1	B01
.........................................................gugagcaaaAuuucaggugugu.................................	2	1	B01
.........................................................gugagcaaaguuucaCgugugu.................................	4	1	B01
.........................................................gugaCcaaaguuucaggugugu.................................	4	1	B01
.........................................................gugagcaaaguuCcaggugugu.................................	4	1	B01
.........................................................gugagcaaaguuucaggugGgu.................................	6	1	B01
.........................................................gugagcaaaguuucagguUugu.................................	4	1	B01
.........................................................gugagcaaaguuucaggugugCc................................	1	1	B01
.........................................................gugagcaaaguuucagguguUuc................................	7	1	B01
.........................................................gugagcaaaguuucaggugugAc................................	5	1	B01
.........................................................gugagcaaaguuucagguguguc................................	8	0	B01
.........................................................gugagcaaaguuucagguguguG................................	13	1	B01
.........................................................gugagcaaaguuucagguguguUa...............................	21	1	B01
.........................................................gugagcaaaguuucaggugugucC...............................	1	1	B01
.........................................................gugagcaaaguuucagguguguGa...............................	1	1	B01
..........................................................ugagcaaaguuucaggugu...................................	1	0	B01
..........................................................ugagcaaaguuucaggugug..................................	2	0	B01
..........................................................ugagcaaaguuucaggugugu.................................	11	0	B01
..........................................................ugagcaaaguuCcaggugugu.................................	1	1	B01
...........................................................gagcaaaguuucaggugu...................................	2	0	B01
...........................................................gagcaaaguuucaggugug..................................	2	0	B01
...........................................................gagcaaaguuucaggugugu.................................	31	0	B01
...........................................................gagcaaaguuucaggugugA.................................	3	1	B01
```

Star		Mature		

```
ugauaauuugcuucauugauccaucugaaagcuugucucaaccuuugaucugaacaggugagcaaaguuucaggugugucagagaaguuuauuuucaucuuauuaucaacua
...........................................................Uagcaaaguuucaggugugu.................................   1     1   B01
............................................................agcaaaguuucaggugug..................................   3     0   B01
............................................................agcaaaguuucaggugugU..................................   1     1   B01
............................................................agcaaaguuucaggugugA.................................   1     1   B01
............................................................agcaaaguuucaggugugu.................................   27    0   B01
............................................................agcaaaguuuUaggugugu.................................   1     1   B01
.............................................................gcaaaguuucaggugugu.................................   38    0   B01
.............................................................gcaaaguuucaggugugA.................................   4     1   B01
.............................................................gcaaaguuucaggugugG.................................   1     1   B01

...................uccaCcugaaagcuugucucaaccu....................................................................   1     1   1st
...................Gccaucugaaagcuugucucaaccu....................................................................   1     1   1st
....................ccaucugaaagcuugucu..........................................................................   1     0   1st
....................ccaucugaaagcuugucuc.........................................................................   3     0   1st
....................ccaucugaaagcuugucuca........................................................................   23    0   1st
....................ccaucugaaagcuugucucaa.......................................................................   7     0   1st
....................ccaucugaaagcuugucucaac......................................................................   22    0   1st
....................ccaucugaaagcuugucucUac......................................................................   1     1   1st
....................ccaGcugaaagcuugucucaacc.....................................................................   1     1   1st
....................ccaucugaaagcuugucucaacc.....................................................................   70    0   1st
....................ccaucugaaagcuugucucaacA.....................................................................   1     1   1st
....................ccaucugaaagcuugucucUacc.....................................................................   1     1   1st
....................ccaucugaaagcuugucucaaccG....................................................................   1     1   1st
....................ccaucugaaagcuugucucaaccA....................................................................   1     1   1st
....................ccaucugaaagcuugucucaaUcu....................................................................   1     1   1st
....................ccaucugUaagcuugucucaaccu....................................................................   1     1   1st
....................Gcaucugaaagcuugucucaaccu....................................................................   1     1   1st
....................ccaucugaaagcuugucucaaccu....................................................................   294   0   1st
....................ccaucugaaagcuugucucaaccuu...................................................................   3     0   1st
....................ccaCcugaaagcuugucucaaccuuu..................................................................   9     1   1st
.....................caCcugaaagcuugucucaaccuuu..................................................................   7     1   1st
........................cugaaagcuugucucaaccu....................................................................   1     0   1st
.........................ugaaagcuugucucaaccu....................................................................   2     0   1st
.........................ugaaagcuugucucaaccuuuU.................................................................   1     1   1st
..........................gaaagcuugucucaaccu....................................................................   3     0   1st
.......................................................Gggugagcaaaguuucaggu.....................................   1     1   1st
.......................................................aggugagcaaaguuucaggugu...................................   1     0   1st
.......................................................aggugagcaaaguuucagguguU..................................   1     1   1st
.......................................................aggugagcaaaguuucaggugugu.................................   1     0   1st
.......................................................Gggugagcaaaguuucaggugugu.................................   2     1   1st
........................................................ggugagcaaaguuucaggugu...................................   40    0   1st
........................................................ggugaAcaaaguuucaggugu...................................   1     1   1st
........................................................Ugugagcaaaguuucaggugu...................................   5     1   1st
........................................................ggugagcaaaguuucaggugug..................................   58    0   1st
........................................................Ugugagcaaaguuucaggugug..................................   11    1   1st
........................................................ggugagcaaaguuucagguguU..................................   5     1   1st
........................................................ggugagcaaaguuucagguguC..................................   1     1   1st
........................................................Agugagcaaaguuucaggugugu.................................   6     1   1st
........................................................Cgugagcaaaguuucaggugugu.................................   5     1   1st
........................................................Ugugagcaaaguuucaggugugu.................................   283   1   1st
........................................................ggugagcaaaguuucaggugugA.................................   4     1   1st
........................................................ggugagcaaaAuuucaggugugu.................................   1     1   1st
........................................................ggugagcaaaguuCcaggugugu.................................   1     1   1st
........................................................ggugagcaaaguuucaggugugu.................................   176   0   1st
........................................................ggugagcaaaguuucagguguUu.................................   3     1   1st
........................................................ggGgagcaaaguuucaggugugu.................................   2     1   1st
.........................................................Cugagcaaaguuucaggu.....................................   1     1   1st
.........................................................gugagcUaaguuucaggu.....................................   1     1   1st
.........................................................gugagcaaaguuucaggC.....................................   4     1   1st
.........................................................gugagcaaaCuuucaggu.....................................   1     1   1st
.........................................................gugagcaaaguuucaggu.....................................   780   0   1st
.........................................................gugaCcaaaguuucaggu.....................................   1     1   1st
.........................................................gugagcaaaguuucCggu.....................................   1     1   1st
.........................................................gugagcaaaguuucaggA.....................................   6     1   1st
.........................................................gugagcaGaguuucaggug....................................   1     1   1st
.........................................................gugagcaaaguuuUaggug....................................   2     1   1st
.........................................................gugagcaaaguuucagguA....................................   1     1   1st
.........................................................gugagcaaaguuucaggGg....................................   3     1   1st
.........................................................gugagcaaaguuucagguU....................................   6     1   1st
.........................................................gugagcaaaguuucaAgug....................................   1     1   1st
```

Star Mature

```
ugauaauuugcuucauugauccaucugaaagcuugucucaaccuuugaucugaacaggugagcaaaguuucaggugugucagagaaguuuauuuucaucuuauuaucaacua
.........................................................gugagcaaaguuucGggug....................................   2      1   1st
.........................................................gugagcUaaguuucaggug....................................   1      1   1st
.........................................................gugagcaaaguuucagguC....................................   5      1   1st
.........................................................gugagcaaaguuucaUgug....................................   1      1   1st
.........................................................gugaAcaaaguuucaggug....................................   1      1   1st
.........................................................gugagcaaaguuucaggug....................................   865    0   1st
.........................................................gugagcaaaguuucagUug....................................   1      1   1st
.........................................................gugagcaaaguuucaggCg....................................   4      1   1st
.........................................................gugagcaaaguuucagCugu...................................   6      1   1st
.........................................................gugagcaaGguuucaggugu...................................   2      1   1st
.........................................................gugCgcaaaguuucaggugu...................................   1      1   1st
.........................................................gugaCcaaaguuucaggugu...................................   2      1   1st
.........................................................gugagcaaaguuucUggugu...................................   10     1   1st
.........................................................gugagcUaaguuucaggugu...................................   16     1   1st
.........................................................gugagcaaaguuucaggGgu...................................   25     1   1st
.........................................................gugagcaaaguuucagguCu...................................   3      1   1st
.........................................................guCagcaaaguuucaggugu...................................   4      1   1st
.........................................................gugagcaaaguuucaggCgu...................................   1      1   1st
.........................................................guAagcaaaguuucaggugu...................................   6      1   1st
.........................................................gugUgcaaaguuucaggugu...................................   3      1   1st
.........................................................gugagcaaaguuucagguUu...................................   2      1   1st
.........................................................gugagcaaaguuucaggugC...................................   19     1   1st
.........................................................gugagcaaaguuuUaggugu...................................   3      1   1st
.........................................................gugagcaaaguuucaggAgu...................................   1      1   1st
.........................................................gugagAaaaguuucaggugu...................................   1      1   1st
.........................................................gugaAcaaaguuucaggugu...................................   3      1   1st
.........................................................gugagcCaaguuucaggugu...................................   1      1   1st
.........................................................gugagcaaaguGucaggugu...................................   1      1   1st
.........................................................gugagcaaaguuucaggugu...................................   15861  0   1st
.........................................................gAgagcaaaguuucaggugu...................................   6      1   1st
.........................................................Uugagcaaaguuucaggugu...................................   6      1   1st
.........................................................gugagcaUaguuucaggugu...................................   2      1   1st
.........................................................gugagcaaagGuucaggugu...................................   6      1   1st
.........................................................gugagcGaaguuucaggugu...................................   2      1   1st
.........................................................gugagcaaaguuucagAugu...................................   2      1   1st
.........................................................gugagcaaaguuucGggugu...................................   7      1   1st
.........................................................gugagcaaagCuucaggugu...................................   7      1   1st
.........................................................gugagcaaaguuuGaggugu...................................   2      1   1st
.........................................................gugagcaaaguuucaggugG...................................   9      1   1st
.........................................................gugagcaaaguuucaCgugu...................................   7      1   1st
.........................................................gugagcaaCguuucaggugu...................................   2      1   1st
.........................................................gugagcaaaguuucaUgugu...................................   5      1   1st
.........................................................gGgagcaaaguuucaggugu...................................   2      1   1st
.........................................................gugGgcaaaguuucaggugu...................................   6      1   1st
.........................................................gugagcaaaguCucaggugu...................................   2      1   1st
.........................................................gugagcaaaguuCcaggugu...................................   2      1   1st
.........................................................gugagcaaaCuuucaggugu...................................   1      1   1st
.........................................................gugagcaaaguuucagUugu...................................   6      1   1st
.........................................................gugagUaaaguuucaggugu...................................   6      1   1st
.........................................................gugagcaaagAuucaggugu...................................   4      1   1st
.........................................................gugagcaaaguuucaAgugu...................................   1      1   1st
.........................................................gCgagcaaaguuucaggugu...................................   3      1   1st
.........................................................gugagcaaaguuucaggugGg..................................   3      1   1st
.........................................................gugagcaaaguuucagguguA..................................   44     1   1st
.........................................................gugagcaaaguuucaAgugug..................................   12     1   1st
.........................................................Augagcaaaguuucaggugug..................................   2      1   1st
.........................................................gugagcaaaguuuGaggugug..................................   5      1   1st
.........................................................gugagcaaaguuuUaggugug..................................   15     1   1st
.........................................................gugUgcaaaguuucaggugug..................................   9      1   1st
.........................................................gugagcaaGguuucaggugug..................................   2      1   1st
.........................................................gugagcaaagAuucaggugug..................................   7      1   1st
.........................................................gugagcaaaguuucaCgugug..................................   9      1   1st
.........................................................gugagcaaaguCucaggugug..................................   5      1   1st
.........................................................gugagcaaaguuucaggugug..................................   31001  0   1st
.........................................................gugagcaaaguuucaggCgug..................................   8      1   1st
.........................................................gugagcaaaguuucagguCug..................................   4      1   1st
.........................................................gugagcaaaguuucUggugug..................................   18     1   1st
.........................................................gugagcaaaguuucagCugug..................................   9      1   1st
.........................................................gugagcaaaguuGcaggugug..................................   2      1   1st
.........................................................gugCgcaaaguuucaggugug..................................   3      1   1st
```

```
                    Star                                    Mature
ugauaauuugcuucauugauccaucugaaagcuugucucaaccuuugaucugaacaggugagcaaaguuucaggugugucagagaaguuuauuuucaucuuauuaucaacua
.........................................................gugagcUaaguuucaggugug..................................    28      1    1st
.........................................................gugagcaaaguAucaggugug..................................    2       1    1st
.........................................................gugagcaaaguuucGgugug..................................    14      1    1st
.........................................................gugagcaaaguuucaggugAg..................................    3       1    1st
.........................................................gugagcaGaguuucaggugug..................................    5       1    1st
.........................................................Cugagcaaaguuucaggugug..................................    1       1    1st
.........................................................gugaAcaaaguuucaggugug..................................    9       1    1st
.........................................................gugagcaaaguuucagguguU..................................    2076    1    1st
.........................................................gugagcaaaUuuucaggugug..................................    1       1    1st
.........................................................gugagcaaaguuucaggGgug..................................    5       1    1st
.........................................................gugagcaaagCuucaggugug..................................    9       1    1st
.........................................................Uugagcaaaguuucaggugug..................................    3       1    1st
.........................................................guCagcaaaguuucaggugug..................................    6       1    1st
.........................................................gugagcCaaguuucaggugug..................................    5       1    1st
.........................................................gugagcaaaguuucaUgugug..................................    9       1    1st
.........................................................guUagcaaaguuucaggugug..................................    1       1    1st
.........................................................gugaCcaaaguuucaggugug..................................    1       1    1st
.........................................................gugGgcaaaguuucaggugug..................................    11      1    1st
.........................................................gugaUcaaaguuucaggugug..................................    1       1    1st
.........................................................gugagcaaaguuucagguguC..................................    10      1    1st
.........................................................gCgagcaaaguuucaggugug..................................    3       1    1st
.........................................................gugagAaaaguuucaggugug..................................    2       1    1st
.........................................................gugagcaaaguuCcaggugug..................................    3       1    1st
.........................................................gugagcaaaguuucagguAug..................................    3       1    1st
.........................................................gugagcaaaguuucagUugug..................................    4       1    1st
.........................................................gugagcaaaCuuucaggugug..................................    1       1    1st
.........................................................guAagcaaaguuucaggugug..................................    10      1    1st
.........................................................gugagcaaaguuucaggugCg..................................    20      1    1st
.........................................................gugagcaaaAuuucaggugug..................................    1       1    1st
.........................................................gGgagcaaaguuucaggugug..................................    2       1    1st
.........................................................gugagcaaUguuucaggugug..................................    1       1    1st
.........................................................gugagcaaagGuucaggugug..................................    3       1    1st
.........................................................gugagcaaaguuucagAugug..................................    11      1    1st
.........................................................gAgagcaaaguuucaggugug..................................    17      1    1st
.........................................................gugagUaaaguuucaggugug..................................    19      1    1st
.........................................................guCagcaaaguuucaggugugu.................................    150     1    1st
.........................................................gugagcaaaguCucaggugugu.................................    81      1    1st
.........................................................gugagcaaaguuucagguCugu.................................    111     1    1st
.........................................................gugagcaaaguuucaggGgugu.................................    109     1    1st
.........................................................gCgagcaaaguuucaggugugu.................................    72      1    1st
.........................................................gugagcaaaguuucGgguguguu................................    413     1    1st
.........................................................gugagcaaaguuAcaggugugu.................................    3       1    1st
.........................................................gugagcaaaguuucagCugugu.................................    281     1    1st
.........................................................gugagcaaaCuuucaggugugu.................................    76      1    1st
.........................................................gugagcaUaguuucaggugugu.................................    80      1    1st
.........................................................gugagGaaaguuucaggugugu.................................    40      1    1st
.........................................................gGgagcaaaguuucaggugugu.................................    96      1    1st
.........................................................gugagcaaaguuucaCgugugu.................................    232     1    1st
.........................................................gugagcaaaguuucaggAgugu.................................    25      1    1st
.........................................................gugagcaaaguuucagguguAu.................................    69      1    1st
.........................................................gugagcaaaguuucagguAugu.................................    75      1    1st
.........................................................gugagcaaaguuucagAugugu.................................    289     1    1st
.........................................................Uugagcaaaguuucaggugugu.................................    97      1    1st
.........................................................gugagcaaaguuuAaggugugu.................................    32      1    1st
.........................................................gugagcaaaguuucaAgugugu.................................    289     1    1st
.........................................................gugagAaaaguuucaggugugu.................................    61      1    1st
.........................................................gugagcaaaguuucaggugugA.................................    4664    1    1st
.........................................................gugagcaaaguuCcaggugugu.................................    48      1    1st
.........................................................gugagcCaaguuucaggugugu.................................    79      1    1st
.........................................................gugagcaaaguuucCgguguguu................................    19      1    1st
.........................................................gugagcaaaguuucagUugugu.................................    197     1    1st
.........................................................gugagcaCaguuucaggugugu.................................    35      1    1st
.........................................................gugaCcaaaguuucaggugugu.................................    85      1    1st
.........................................................gugagcaaagCuucaggugugu.................................    143     1    1st
.........................................................gAgagcaaaguuucaggugugu.................................    409     1    1st
.........................................................gugagcaaaAuuucaggugugu.................................    33      1    1st
.........................................................gugagcaaaguuuUaggugugu.................................    442     1    1st
.........................................................gugagcaaaguuucaggugAgu.................................    79      1    1st
.........................................................gugagcaaaguuucagguUugu.................................    85      1    1st
.........................................................gugagcaaaguuGcaggugugu.................................    15      1    1st
```

```
                     Star                                   Mature
ugauaauuugcuucauugauccaucugaaagcuugucucaaccuuugaucugaacaggugagcaaaguuucaggugugucagagaaguuuauuuucaucuuauuaucaacua
........................................................gugagcaaaguuucaUgugugu.................................   153      1   1st
........................................................gugagcaaaguuucaggugugu.................................   910254   0   1st
........................................................Cugagcaaaguuucaggugugu.................................   39       1   1st
........................................................gugagcaaaguAucaggugugu.................................   52       1   1st
........................................................gugagcaaaguuucUggugugu.................................   400      1   1st
........................................................gugagcaaaguuucaggugCgu.................................   92       1   1st
........................................................gugagcaaaUuuucaggugugu.................................   17       1   1st
........................................................gugagcaaaguuucaggugGgu.................................   127      1   1st
........................................................gugGgcaaaguuucaggugugu.................................   411      1   1st
........................................................gugagcUaaguuucaggugugu.................................   1061     1   1st
........................................................gugagcaaUguuucaggugugu.................................   47       1   1st
........................................................gugagUaaaguuucaggugugu.................................   449      1   1st
........................................................guAagcaaaguuucaggugugu.................................   198      1   1st
........................................................gugCgcaaaguuucaggugugu.................................   175      1   1st
........................................................gugagcaaGguuucaggugugu.................................   60       1   1st
........................................................gugUgcaaaguuucaggugugu.................................   262      1   1st
........................................................gugagcaaaguuucagguguCu.................................   112      1   1st
........................................................gugagcaaaguGucaggugugu.................................   61       1   1st
........................................................gugagcaGaguuucaggugugu.................................   55       1   1st
........................................................guUagcaaaguuucaggugugu.................................   38       1   1st
........................................................gugagcaaaguuuGaggugugu.................................   103      1   1st
........................................................gugagcaaaguuucaggCgugu.................................   91       1   1st
........................................................gugagcaaagAuucaggugugu.................................   162      1   1st
........................................................gugagcaaaguuucaggugugG.................................   845      1   1st
........................................................gugaAcaaaguuucaggugugu.................................   219      1   1st
........................................................gugagcaaaguuucagguguUu.................................   3714     1   1st
........................................................gugagcaaagGuucaggugugu.................................   183      1   1st
........................................................gugagcGaaguuucaggugugu.................................   80       1   1st
........................................................Augagcaaaguuucaggugugu.................................   55       1   1st
........................................................gugaUcaaaguuucaggugugu.................................   34       1   1st
........................................................gugagcaaCguuucaggugugu.................................   21       1   1st
........................................................gugagcaaaguuucaggugugugc................................   44       0   1st
........................................................gugagcaaaguuucagguguguG................................   95       1   1st
........................................................gugagcaaaguuucagguguUuc................................   2        1   1st
........................................................gugagcaaaguuucaggugugGc................................   1        1   1st
........................................................gugagcaaaguuucaggugugCc................................   6        1   1st
........................................................gugagcaaaguuucaggugugugca...............................   5        0   1st
........................................................gugagcaaaguuucagguguguGa...............................   7        1   1st
........................................................gugagcaaaguuucaggugugucU...............................   12       1   1st
........................................................gugagcaaaguuucagguguguUa...............................   218      1   1st
........................................................gugagcaaaguuucaggugugucC...............................   5        1   1st
........................................................gugagcaaaguuucagguguguGag..............................   4        1   1st
........................................................gugagcaaaguuucagguguguGaga.............................   2        1   1st
........................................................gugagcaaaguuucagguguguGagag............................   2        1   1st
.........................................................ugagcUaaguuucaggugu...................................   1        1   1st
.........................................................ugagcaaaguuucaggugu...................................   2        0   1st
.........................................................ugagcaaaguuucaggugug..................................   3        0   1st
.........................................................Ggagcaaaguuucaggugugu.................................   1        1   1st
.........................................................ugagcaaaguuucaggugugA.................................   1        1   1st
.........................................................ugagcaaaguuuUaggugugu.................................   1        1   1st
.........................................................ugagcaaaguuucGggugugu.................................   1        1   1st
.........................................................ugagcaUaguuucaggugugu.................................   1        1   1st
.........................................................ugGgcaaaguuucaggugugu.................................   1        1   1st
.........................................................ugagcaaaguuucaggugugu.................................   141      0   1st
.........................................................ugagcaaaguuucagguguUu.................................   1        1   1st
.........................................................ugagcaGaguuucaggugugu.................................   1        1   1st
.........................................................ugagcCaaguuucaggugugu.................................   5        1   1st
.........................................................ugagcUaaguuucaggugugu.................................   2        1   1st
.........................................................ugagcaaaguuucaggugugugc................................   1        0   1st
.........................................................ugagcaaaguuucaggugugucU...............................   1        1   1st
..........................................................gagcaaaguuucaggugu...................................   2        0   1st
..........................................................gagcaaaguuucagguguU..................................   1        1   1st
..........................................................gagcaaaguuucaggugug..................................   3        0   1st
..........................................................gagcaaGguuucaggugugu.................................   1        1   1st
..........................................................gagcaaaAuuucaggugugu.................................   1        1   1st
..........................................................gGgcaaaguuucaggugugu.................................   1        1   1st
..........................................................gagcaaaguuucaggugugu.................................   213      0   1st
..........................................................Uagcaaaguuucaggugugu.................................   1        1   1st
...........................................................agcaaaguuucaggugug..................................   3        0   1st
...........................................................agcaaaguuucaggugugA.................................   2        1   1st
```

Star **Mature**

```
ugauaauuugcuucauugauccaucugaaagcuugucucaaccuuugaucugaacaggugagcaaaguuucaggugugucagagaaguuuauuuucaucuuauuaucaacua
............................................................agcaaaguuucagguUugu.................................  1     1  1st
............................................................agcaaaguuucaggugugu.................................  103   0  1st
.............................................................gcaaaguuucaggugugu.................................  100   0  1st
.............................................................gcaaaguuucaggugugG.................................  1     1  1st

....................ccaucugaaagcuugucuca........................................................................  1     0  EGG
....................ccaucugaaagcuugucucaa.......................................................................  1     0  EGG
....................ccaucugaaagcuugucucaac......................................................................  8     0  EGG
....................ccaucugaaagcuugucucaacc.....................................................................  33    0  EGG
....................ccaucugaaagcuugucucaacU.....................................................................  1     1  EGG
....................ccaGcugaaagcuugucucaacc.....................................................................  1     1  EGG
....................ccaGcugaaagcuugucucaaccu....................................................................  1     1  EGG
....................ccaucugGaagcuugucucaaccu....................................................................  1     1  EGG
....................ccaucugaaagcuugucucaaccu....................................................................  284   0  EGG
....................ccaucugaaagcuugucucaaccuA...................................................................  2     1  EGG
....................ccaucugaaagcuugucucaaccuu...................................................................  1     0  EGG
....................ccaucugaaagcuugucucaaccuuu..................................................................  1     0  EGG
....................ccaCcugaaagcuugucucaaccuuu..................................................................  12    1  EGG
.....................caucugaaagcuugucucaaccuuu..................................................................  1     0  EGG
.....................caCcugaaagcuugucucaaccuuu..................................................................  12    1  EGG
..........................gaaagcuugucucaaccuu...................................................................  1     0  EGG
......................................................acGgugagcaaaguuucaggugugu.................................  1     1  EGG
.......................................................Uggugagcaaaguuucaggugugu.................................  1     1  EGG
.......................................................aggugagcaaaguuucaggugugu.................................  1     0  EGG
........................................................Ugugagcaaaguuucaggugu...................................  1     1  EGG
........................................................ggugagcaaaguuucaggugu...................................  9     0  EGG
........................................................ggugagcaaaguuucagguguU..................................  4     1  EGG
........................................................ggugagcaaaguuucaggugug..................................  75    0  EGG
........................................................Ugugagcaaaguuucaggugugu.................................  73    1  EGG
........................................................Cgugagcaaaguuucaggugugu.................................  1     1  EGG
........................................................ggugagcaaaguuucaggugugG.................................  1     1  EGG
........................................................ggugagcaaaguuucaggugugu.................................  70    0  EGG
........................................................ggugagcaaaguuucaggugugA.................................  1     1  EGG
.........................................................gugagcaaaguuucaggu.....................................  54    0  EGG
.........................................................gugagcaaaguCucaggug....................................  1     1  EGG
.........................................................gugagcaaaguuucaggug....................................  102   0  EGG
.........................................................gugGgcaaaguuucaggug....................................  1     1  EGG
.........................................................gugUgcaaaguuucaggug....................................  1     1  EGG
.........................................................gugagcaaaguuucagguU....................................  2     1  EGG
.........................................................gugagcaaaguuucaggugu...................................  892   0  EGG
.........................................................gugagUaaaguuucaggugu...................................  1     1  EGG
.........................................................gugaAcaaaguuucaggugu...................................  2     1  EGG
.........................................................gugagcaaaguuucaggugC...................................  2     1  EGG
.........................................................gugagcaaaguuucGggugu...................................  2     1  EGG
.........................................................gugagAaaaguuucaggugu...................................  1     1  EGG
.........................................................gugagcaaaguuucagguguC..................................  1     1  EGG
.........................................................gugagcaaaguuucaggugug..................................  6868  0  EGG
.........................................................gugagcaaaguuucagguAug..................................  1     1  EGG
.........................................................gugagAaaaguuucaggugug..................................  1     1  EGG
.........................................................gugagcaaaguuucCggugug..................................  1     1  EGG
.........................................................gugagcaaagCuucaggugug..................................  3     1  EGG
.........................................................gugagcaaagGuucaggugug..................................  2     1  EGG
.........................................................gCgagcaaaguuucaggugug..................................  2     1  EGG
.........................................................gugGgcaaaguuucaggugug..................................  2     1  EGG
.........................................................gugagcaaaguuucagguguU..................................  216   1  EGG
.........................................................gugagcaaaguuucagguguA..................................  7     1  EGG
.........................................................gugagcaaaguuucagCugug..................................  1     1  EGG
.........................................................gugagcaaaUuuucaggugug..................................  1     1  EGG
.........................................................gugUgcaaaguuucaggugug..................................  1     1  EGG
.........................................................gugagcaaaCuuucaggugug..................................  1     1  EGG
.........................................................gugagGaaaguuucaggugug..................................  1     1  EGG
.........................................................gugagcaaaguuucaggugGg..................................  1     1  EGG
.........................................................gugagcaaaguuucagguUug..................................  1     1  EGG
.........................................................gugCgcaaaguuucaggugug..................................  4     1  EGG
.........................................................gugagcaaaguuucagAugug..................................  3     1  EGG
.........................................................gugagcaaaguuucGggugug..................................  2     1  EGG
.........................................................gugagcaaaguAucaggugug..................................  1     1  EGG
.........................................................gugagcaaaguuucUggugug..................................  1     1  EGG
.........................................................gugagUaaaguuucaggugug..................................  3     1  EGG
.........................................................gugagcaaaguuucaggugAg..................................  1     1  EGG
```

Star Mature

ugauaauuugcuucauugauccaucugaaagcuugucucaaccuuugaucugaacaggugagcaaaguuucaggugugucagagaaguuuauuuucaucuuauuaucaacua			
....gugagcaaGguuucaggugug..................................	1	1	EGG
....gugagcaaaguuucaggGgug..................................	1	1	EGG
....gugaAcaaaguuucaggugug..................................	1	1	EGG
....gugagcaaaguuuUaggugug..................................	1	1	EGG
....gugagcaaaguuucagUugug..................................	2	1	EGG
....Augagcaaaguuucaggugugu.................................	6	1	EGG
....gugagcaaaguuucaAgugugu.................................	3	1	EGG
....gugagcaaaguuucagguCugu.................................	4	1	EGG
....gugagcaaaAuuucaggugugu.................................	4	1	EGG
....gugagcaaaguuucaggAgugu.................................	3	1	EGG
....gugagcaaaguuucaggGgugu.................................	9	1	EGG
....gugagcaaaguuucagguguUu.................................	467	1	EGG
....gugagGaaaguuucaggugugu.................................	25	1	EGG
....gugagcaaagAuucaggugugu.................................	6	1	EGG
....guAagcaaaguuucaggugugu.................................	2	1	EGG
....gugagcaaagCuucaggugugu.................................	27	1	EGG
....gugagcaaaguuucagAugugu.................................	96	1	EGG
....gugagAaaaguuucaggugugu.................................	21	1	EGG
....gugagcaaaguGucaggugugu.................................	14	1	EGG
....gugagcGaaguuucaggugugu.................................	7	1	EGG
....gugagcaaaguuAcaggugugu.................................	1	1	EGG
....gCgagcaaaguuucaggugugu.................................	11	1	EGG
....gugagcaaaguuCcaggugugu.................................	8	1	EGG
....gugagcaaCguuucaggugugu.................................	4	1	EGG
....gugagcaGaguuucaggugugu.................................	11	1	EGG
....gugagcaaaguuucGggugugu.................................	101	1	EGG
....gGgagcaaaguuucaggugugu.................................	15	1	EGG
....gugagcaaaguuucagguAugu.................................	14	1	EGG
....gugagcaUaguuucaggugugu.................................	4	1	EGG
....gugagcaaaguuucaggugAgu.................................	3	1	EGG
....gAgagcaaaguuucaggugugu.................................	1	1	EGG
....gugagcaaaguuucaggCgugu.................................	18	1	EGG
....gugagcaaaguuucaggugugu.................................	168800	0	EGG
....gugagcaaaCuuucaggugugu.................................	14	1	EGG
....gugagcaaaguuucagUugugu.................................	20	1	EGG
....gugaCcaaaguuucaggugugu.................................	3	1	EGG
....gugaAcaaaguuucaggugugu.................................	45	1	EGG
....gugagcaaaguuucaggugGgu.................................	3	1	EGG
....gugagcaaUguuucaggugugu.................................	18	1	EGG
....gugagcaaaguuucagguguAu.................................	9	1	EGG
....gugUgcaaaguuucaggugugu.................................	15	1	EGG
....Uugagcaaaguuucaggugugu.................................	14	1	EGG
....gugagcaaaguCucaggugugu.................................	11	1	EGG
....gugagcaaaguAucaggugugu.................................	4	1	EGG
....gugagcaaaguuucagCugugu.................................	11	1	EGG
....gugagcaaaguuucaggugugA.................................	988	1	EGG
....gugaUcaaaguuucaggugugu.................................	2	1	EGG
....gugagcaaaguuuAaggugugu.................................	3	1	EGG
....gugagcaaagGuucaggugugu.................................	15	1	EGG
....Cugagcaaaguuucaggugugu.................................	1	1	EGG
....gugagUaaaguuucaggugugu.................................	71	1	EGG
....gugagcaaGguuucaggugugu.................................	9	1	EGG
....gugagcaCaguuucaggugugu.................................	5	1	EGG
....gugagcaaaguuuGaggugugu.................................	6	1	EGG
....gugagcaaaguuucCggugugu.................................	11	1	EGG
....gugagcaaaguuucaCgugugu.................................	11	1	EGG
....gugagcaaaguuucaUgugugu.................................	9	1	EGG
....guCagcaaaguuucaggugugu.................................	9	1	EGG
....gugagcaaaguuuUaggugugu.................................	50	1	EGG
....gugagcaaaguuucaggugugG.................................	48	1	EGG
....gugGgcaaaguuucaggugugu.................................	17	1	EGG
....gugCgcaaaguuucaggugugu.................................	55	1	EGG
....gugagcaaaguuucUggugugu.................................	40	1	EGG
....gugagcaaaguuucagguguCu.................................	6	1	EGG
....gugagcaaaguuucaggugCgu.................................	12	1	EGG
....gugagcaaaUuuucaggugugu.................................	8	1	EGG
....guUagcaaaguuucaggugugu.................................	2	1	EGG
....gugagcaaaguuucagguUugu.................................	12	1	EGG
....gugagcaaaguuucaggugugu c................................	21	0	EGG
....gugagcaaaguuucaggugugCc................................	1	1	EGG

Star Mature

ugauaauuugcuucauugauccaucugaaagcuugucucaaccuuugaucugaacaggugagcaaaguuucaggugugucagagaaguuuauuucaucuuauuaucaacua			
....gugagcaaaguuucaggugugu G...............................	25	1	EGG
....gugagcaaaguuucaggugugu ca..............................	2	0	EGG
....gugagcaaaguuucaggugugu Ua..............................	55	1	EGG
....gugagcaaaguuucaggugugu Ga..............................	2	1	EGG
....gugagcaaaguuucaggugugucU..............................	2	1	EGG
....ugagcaaaguuucaggugugu................................	17	0	EGG
....gagcaaaguuucaggugugu................................	18	0	EGG
....agcaaaguuucaggugugu................................	1	0	EGG
....gcaaaguuucaggugugu................................	1	0	EGG

Provisional ID	: Scaffold_11093_2931
Score total	: 126.6
Score for star read(s)	: 3.9
Score for read counts	: 119.4
Score for mfe	: -1.2
Score for randfold	: 1.6
Score for cons. seed	: 3
Total read count	: 246
Mature read count	: 221
Loop read count	: 0
Star read count	: 25

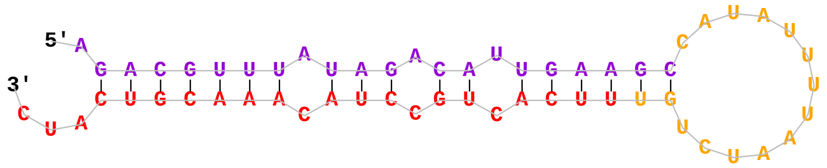

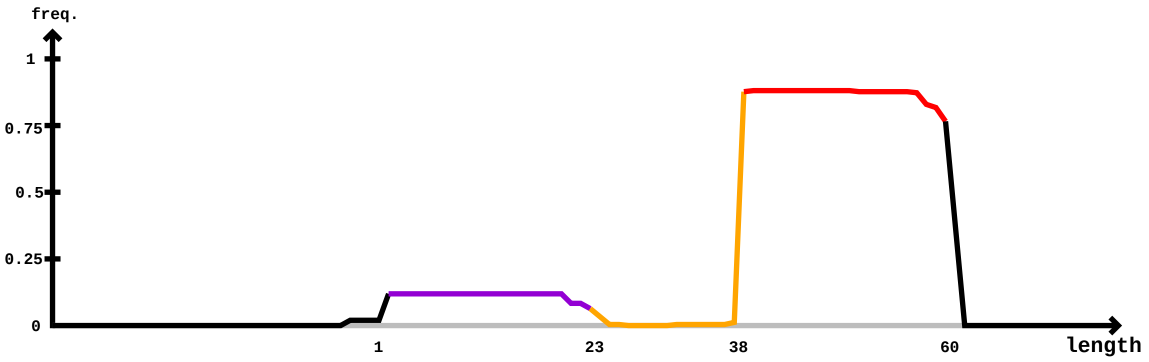


```
                                     Star                                  Mature
5'- caauacuuuuuguugcaacaauuugucaugaaaagacguuuauagacauugaagccauauuuuaaucuguuucacugccuacaaacgucaucucacgauauaaaauugug  -3'   obs
    caauacuuuuuguugcaacaauuugucaugaaaagacguuuauagacauugaagccauauuuuaaucuguuucacugccuacaaacgucaucucacgauauaaaauugug         exp
    (((((.....)))))..((((((((((.(((...(((((((.(((.((.((((((..............)))))).)).))).)))))))...))).))))..)))))).   reads   mm   sample
    .....................................................................uuucacugccuacaaacgucau...................   1       0    EGG
    ......................................................................uucacugccuacaaGcguca....................   1       1    EGG
    ......................................................................uucacugccuacaaacgucau...................   1       0    EGG
    ......................................................................uucacugccuacaaacgucauc..................   10      0    EGG

    .................................agacguuuaGagacauuga..........................................................   1       1    TJ1
    .................................agacguuuauagacauuga..........................................................   2       0    TJ1
    .................................agacguuuauagacauugaagU.......................................................   1       1    TJ1
    ......................................................................uucacugccuacaaacgucauc..................   4       0    TJ1

    ...............................................................aaucuguuucacugccuac............................   1       0    B01
    ......................................................................uucacugccuacaaacgucauc..................   33      0    B01
    .......................................................................ucacugccuacaaacgucauc..................   1       0    B01

    .....................................................................uuucacugccuacaaacgucU...................   1       1    1st
    ......................................................................uucacugccuacaaacgucauc..................   11      0    1st

    .............................gaaaGgacguuuauagacauuga..........................................................   1       1    BJ1
    .............................gaaaagacguuuauagacauuga..........................................................   2       0    BJ1
    .............................gaaaagacguuuauagacauugaag........................................................   1       0    BJ1
    .............................gaaaagacguuuauaCacauugaag........................................................   1       1    BJ1
    ......................................................................Cucacugccuacaaacgucauc..................   1       1    BJ1
    ......................................................................uucacugccuacaaacgucauc..................   5       0    BJ1

    .................................agacguuuauagacauugaag........................................................   1       0    HA1
    .................................agacguuuauagacauugaagc.......................................................   1       0    HA1
    .................................agacguuuauGgacauugaagc.......................................................   1       1    HA1
    ......................................................................uucacugccuacaaacguc.....................   3       0    HA1
    ......................................................................uucacugccuacaaacgucau...................   3       0    HA1
    ......................................................................uucacugccuacaaacgucauc..................   21      0    HA1
    ......................................................................uucacugccuacaaacgucaCc..................   1       1    HA1
    ......................................................................uucacugccuacaaacgucaucu.................   3       0    HA1

    ......................................................................uucacugccuacaaacgucauc..................   8       0    2nd
```

```
                                  Star                        Mature
caauacuuuuuguugcaacaauuugucaugaaaagacguuuauagacauugaagccauauuuuaaucuguuucacugccuacaaacgucaucucacgauauaaaauugug

.................................agacguuuauagacauugaagc.......................................................	2	0	H01
......................................................................uucacugccuacaaacgucauc..................	4	0	H01

......................................................................uucacugccuacaaacgucaCc..................	1	1	LJ5
......................................................................uucacugccuacaaacgucauU..................	1	1	LJ5

.................................agacguuuauagacauugaag........................................................	1	0	LA5
......................................................................uucacugccuacaaacguc.....................	1	0	LA5
......................................................................uucacugccuacaaacgucau...................	1	0	LA5
......................................................................uucacugccuacaaacgucaA...................	1	1	LA5
......................................................................uucacugccuacGaacgucauc..................	2	1	LA5
......................................................................uucacugccuacaaacgucauc..................	2	0	LA5
......................................................................uucacugccuacaaacgucaucu.................	1	0	LA5

.................................agacguuuauagacauugaag........................................................	1	0	LJ1
......................................................................uucacugccuacaaacgucauc..................	1	0	LJ1

.................................agacguuuauagacauugaagc.......................................................	1	0	TA1
......................................................................uucacugccuacaaacguc.....................	5	0	TA1
......................................................................uucacugccuacaaacgucaC...................	1	1	TA1
......................................................................uucacugccuacaaacgucau...................	4	0	TA1
......................................................................uucacugccuacaaacgucauU..................	1	1	TA1
......................................................................uucacugccuacaaacgucauc..................	11	0	TA1
......................................................................uucacugccuacaaacgucaucA.................	1	1	TA1

.................................agacguuuauagacauugaagc.......................................................	1	0	E01
......................................................................uucacugccuacaaacgucauc..................	10	0	E01

.................................agacguuuauagacauuga..........................................................	1	0	LA1
.................................agacguuuauagacauugaagc.......................................................	2	0	LA1
......................................................................uucacugccuacaaacguc.....................	1	0	LA1
......................................................................uucacugccuacaaacgucauc..................	1	0	LA1
......................................................................uucacugccuacaaacgucaucu.................	1	0	LA1

.................................agacguuuauagacauugaGgc.......................................................	1	1	HJ1
......................................................................uucacugccuacaaacgu......................	1	0	HJ1

......................................................................uucacugccuacaaacguc.....................	1	0	G01
......................................................................uucacugccuacaaacgucauc..................	14	0	G01
......................................................................uGcacugccuacaaacgucauc..................	1	1	G01

.................................agacguuuauagacauugaagU.......................................................	1	1	CJ1

.................................agacguuuauagacauugaagc.......................................................	3	0	BRA
......................................................................uucacugccuacaaacgucauc..................	6	0	BRA
......................................................................uucacCgccuacaaacgucauc..................	1	1	BRA

.................................agacguuuauagacauuga..........................................................	2	0	BA1
.................................agacguuuauagacauugaagc.......................................................	1	0	BA1
.................................agacguuuauagacauugaagccau....................................................	1	0	BA1
......................................................................uucacugccuacaaacgucauU..................	2	1	BA1
......................................................................uucacugccuacaaacgucauc..................	14	0	BA1
......................................................................uucacugccuacaaacgucaucu.................	3	0	BA1

......................................................................uucacugcGuacaaacgucauc..................	2	1	CA1
......................................................................uucacugccuacaaacgucauc..................	3	0	CA1

......................................................................uucacugccuacaaacguca....................	1	0	3rd
......................................................................uucacugccuacaaacgucau...................	1	0	3rd
......................................................................uucacugccuacaaacgucauc..................	12	0	3rd
```

Provisional ID : Scaffold_11093_3109
Score total : 672.2
Score for star read(s) : 3.9
Score for read counts : 666.5
Score for mfe : 0.8
Score for randfold : 1.6
Score for cons. seed : -0.6
Total read count : 1319
Mature read count : 498
Loop read count : 0
Star read count : 821

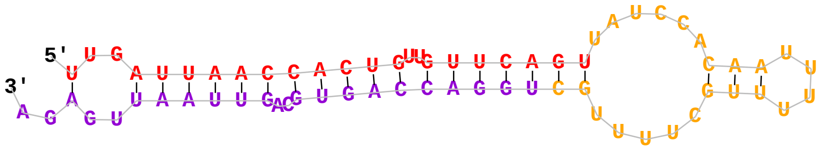

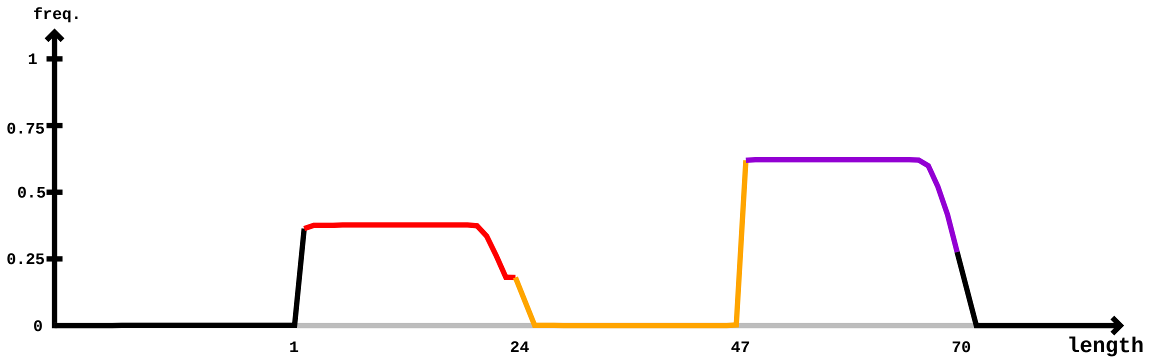


```
                        Mature                                        Star
5'- aaacuaguaacauacacuagugccuugauuaaccacuguuguucaguuauccacaauuuuuugcuuuugcuggaccagugcaguuaauugagacaccagugcauauuca  -3'  obs
    aaacuaguaacauacacuagugccuugauuaaccacuguuguucaguuauccacaauuuuuugcuuuugcuggaccagugcaguuaauugagacaccagugcauauuca       exp
    ..............((((.(((.((..(((((((((((..(((((((......(((....))).....))))))))))))..)))))).)).))).))))........  reads  mm  sample
    ........................uugauuaaccacuguuguuc.................................................................  1      0   HA1
    ......................................................................uggaccagugcaguuaauug...................  5      0   HA1
    ......................................................................uggaccagugcaguuaauuga..................  3      0   HA1
    ......................................................................uggaccagugcaguuaauugag.................  10     0   HA1
    ......................................................................uggaccagugcaguuaauugaga................  10     0   HA1
    ......................................................................uggaccagugcaguuaaAugaga................  1      1   HA1
    ......................................................................uggaccagugcaguuaauugagU................  2      1   HA1
    ......................................................................uggaccagugcaguuaauugagC................  1      1   HA1

    ........................uugauuaaccacuguuguuc.................................................................  2      0   TJ1
    ......................................................................uggaccagugcaguuaauu....................  2      0   TJ1
    ......................................................................uggaccagugcaguuaauuga..................  3      0   TJ1
    ......................................................................uggaccagugcaguuaauugag.................  3      0   TJ1
    ......................................................................uggaccagugcaguuaauugagU................  1      1   TJ1
    ......................................................................uggaccagugcaguuaaCugaga................  1      1   TJ1

    ........................uugauuaaccacuguuguu..................................................................  1      0   B01
    ........................uugauuaaccacuguuguucagu..............................................................  2      0   B01
    ......................................................................uggaccagugUaguuaauug...................  1      1   B01

    ........................uugauuaaccacuguuguu..................................................................  5      0   1st
    ........................uugauuaaccacuguuguuc.................................................................  11     0   1st
    ........................uugauuaaccacuguuguuca................................................................  9      0   1st
    ........................uugauuaaccacuguuguucagu..............................................................  21     0   1st
    ......................................................................uggaccagugcaguuaauu....................  2      0   1st
    ......................................................................uggaccagugcaguuaauug...................  6      0   1st
    ......................................................................uggaccagugcaguuaauuga..................  22     0   1st
    ......................................................................uggaccagugcaguuaauugag.................  3      0   1st
    ......................................................................uggaccagugcaguuaauugagC................  1      1   1st
    ......................................................................uggaccagugcaguuaauugaga................  10     0   1st

    .....aguaacauacacuagugcc.....................................................................................  1      0   EGG
    ........................uugauuaaccacuguuguuc.................................................................  2      0   EGG
```

Mature Star

```
aaacuaguaacauacacuagugccuugauuaaccacuguuguucaguuauccacaauuuuuugcuuuugcuggaccagugcaguuaauugagacaccagugcauauuca
........................uugauuaaccacuguuguuca................................................................  15  0  EGG
........................uugauuaaccacuguuguucagu..............................................................  17  0  EGG
......................................................................uggaccagugcaguuaauug...................  2  0  EGG
......................................................................uggaccagugcaguuaauuA...................  1  1  EGG
......................................................................uggaccagugcaguuaauuga..................  9  0  EGG
......................................................................uggaccagugcaguuaauugag.................  11  0  EGG
......................................................................uggaccagugcaguuaauugagU................  1  1  EGG
......................................................................uggaccagugcaguuaauugaga................  4  0  EGG
......................................................................uggaccagugcaguuaauugagaU...............  1  1  EGG

........................uugauuaaccacuguugu...................................................................  2  0  BA1
......................................................................uggaccagugcaguuaGuu....................  1  1  BA1
......................................................................uggaccagugcaguuaauug...................  1  0  BA1
......................................................................uggaccagugcaguuaauugag.................  1  0  BA1

........................uugauuaaccacuguuguuc.................................................................  1  0  BRA
......................................................................uggaccagugcaguuaauuA...................  1  1  BRA
......................................................................uggaccagugcaguuaauug...................  2  0  BRA
......................................................................uggaccagugcaguuaauuga..................  4  0  BRA
......................................................................uggaccagugcaguuaauugaA.................  1  1  BRA
......................................................................uggaccagugcaguuaauugaga................  5  0  BRA
......................................................................uggaccagugcaguuaauugagU................  2  1  BRA

........................uugauuaaccacuguuguu..................................................................  3  0  3rd
........................uugauuaaccacuguuguuc.................................................................  7  0  3rd
........................uugauuaaccacuguuguuca................................................................  14  0  3rd
........................uugauuaaccacuguuguucGgu..............................................................  1  1  3rd
........................uugauuaaccacuguuguucagu..............................................................  23  0  3rd
........................uugauuaaccacuguuguuUagu..............................................................  1  1  3rd
........................uugauuaaccacuguuguucaguA.............................................................  1  1  3rd
.........................ugauuaaccacuguuguucagu..............................................................  2  0  3rd
......................................................................uggaccagugcaguuaauu....................  1  0  3rd
......................................................................uggaccagugcaguuaauug...................  15  0  3rd
......................................................................uggaccagugcaguuaauuga..................  12  0  3rd
......................................................................uggaccagugcaguuaauugag.................  13  0  3rd
......................................................................uggaccagugcaguuaauugaga................  13  0  3rd
......................................................................uggaccagugcaguuaauugagU................  1  1  3rd
.......................................................................ggaccagugcaguuaauugaga................  1  0  3rd

........................uugauuaacUacuguuguuc.................................................................  2  1  CA1
........................uugauuaaccacuguuguuc.................................................................  2  0  CA1
......................................................................uggaccagugcaguuaauC....................  1  1  CA1
......................................................................uggaccagugcaguuaauu....................  3  0  CA1
......................................................................uggaccagugcaguuaauug...................  10  0  CA1
......................................................................uggGccagugcaguuaauug...................  1  1  CA1
......................................................................uggaccagugcaguuaauuga..................  3  0  CA1
......................................................................uggaccagugcaguuaauugag.................  21  0  CA1
......................................................................uggaccagugcaguuaauugCg.................  1  1  CA1
......................................................................uggaccagugcaguuaauugaga................  34  0  CA1
......................................................................uggaccagugcaguuaauugagU................  3  1  CA1
......................................................................uggaccagugcaguuaauugagaA...............  1  1  CA1

........................uugauuaaccacuguuguu..................................................................  1  0  HJ1
........................uugauuaaccacuguuguucagu..............................................................  1  0  HJ1
......................................................................uggaccagugcaguuaauuga..................  1  0  HJ1
......................................................................uggaccagugcaguuaauugaga................  2  0  HJ1

........................uugauuaaccacuguugu...................................................................  1  0  LA1
........................uugauuaaccacuguuguC..................................................................  1  1  LA1
........................uugauuaaccacugCuguu..................................................................  1  1  LA1
........................uugauuaaccacuguuguu..................................................................  1  0  LA1
........................uugauuaaccacuguuguuc.................................................................  3  0  LA1
........................uugauuaaccacuguuguuca................................................................  2  0  LA1
......................................................................uggaccagugcaguuaau.....................  1  0  LA1
......................................................................uggaccagugcaguuaauu....................  3  0  LA1
......................................................................uggaccagugcaguuaauuA...................  1  1  LA1
......................................................................uggaccagugcaguuaauug...................  8  0  LA1
......................................................................uggaccagugcaguuaauuga..................  12  0  LA1
......................................................................uggaccagugcaguuaauugag.................  23  0  LA1
......................................................................uggaccagugcaguuaauugaA.................  2  1  LA1
```

```
                        Mature                                      Star
aaacuaguaacauacacuagugccuugauuaaccacuguuguucaguuauccacaauuuuuugcuuuugcuggaccagugcaguuaauugagacaccagugcauauuca
......................................................................uggaccagugcaguuaauugagG................   2    1    LA1
......................................................................Cggaccagugcaguuaauugaga................   1    1    LA1
......................................................................uggaccagugcagCuaauugaga................   2    1    LA1
......................................................................uggaccagugcaguuaauugagU................   8    1    LA1
......................................................................uggaccagugcaguuaauugaga................   45   0    LA1
......................................................................uggaccagugcaguuaauugagaU...............   1    1    LA1

......................................................................uggaccagugcaguuaauug...................   3    0    CJ1
......................................................................uggaccagugcaguuaauuga..................   8    0    CJ1
......................................................................uggaccagugcaguuaauugag.................   12   0    CJ1
......................................................................ugCaccagugcaguuaauugaga................   1    1    CJ1
......................................................................ugUaccagugcaguuaauugaga................   2    1    CJ1
......................................................................uggaccagugcaguuaauugaga................   23   0    CJ1

........................uugauuaaccacuguuguu..................................................................   5    0    G01
........................uugauuaaccacuguuguuc.................................................................   8    0    G01
........................uugauuaaccacuguuguuca................................................................   8    0    G01
........................uugauuaaccacuguuguucagu..............................................................   23   0    G01
........................uugauuaaccacuguuguucaguu.............................................................   3    0    G01
.........................ugauuaaccacuguuguucaguu.............................................................   1    0    G01
......................................................................uggaccagugcaguuaauug...................   4    0    G01
......................................................................uggaccagugcaguuaauuga..................   1    0    G01
......................................................................uggaccagugcaguuaauugag.................   4    0    G01
......................................................................uggaccagugcaguuaauugagU................   1    1    G01
......................................................................uggaccagugcaguuaauugaga................   3    0    G01
......................................................................uggaccagugcaguuaauugagC................   1    1    G01

........................uugauuaaccacuguuguuc.................................................................   3    0    LJ1
........................uugauuaaccacuguuguucagu..............................................................   1    0    LJ1
......................................................................uggaccagugcaguuaauu....................   1    0    LJ1
......................................................................uggaccagugcaguuaauug...................   1    0    LJ1
......................................................................uggaccagugcaguuaauuga..................   5    0    LJ1
......................................................................ugUaccagugcaguuaauuga..................   1    1    LJ1
......................................................................uggaccagugcaguuaauugU..................   2    1    LJ1
......................................................................uggaccagugcaguuaauugaA.................   1    1    LJ1
......................................................................uggaccagugcaguuaauugag.................   8    0    LJ1
......................................................................uggaccagugcaguuaauugagG................   1    1    LJ1
......................................................................uggaccagugcaguuaauugaga................   26   0    LJ1

........................uugauuaaccacuguuguu..................................................................   3    0    E01
........................uugauuaaccacuguuguuc.................................................................   14   0    E01
........................uugauuaaccacuguuguuca................................................................   12   0    E01
........................uugauuaaccacuguuguucU................................................................   1    1    E01
........................uugauuaaccacuguuguucag...............................................................   1    0    E01
........................uugauuaaccacuguuguucagu..............................................................   46   0    E01
........................uugauuaaccacuguuguucaAu..............................................................   1    1    E01
.........................ugauuaaccacuguuguucagu..............................................................   3    0    E01
............................uuaaccacuguuguucagA..............................................................   1    1    E01
......................................................................uggaccagugcaguuaauu....................   1    0    E01
......................................................................uggaccagugcaguuaauug...................   9    0    E01
......................................................................Aggaccagugcaguuaauuga..................   1    1    E01
......................................................................uggaccagugcaguuaauuga..................   5    0    E01
......................................................................uggaccagugcaguuaauugag.................   4    0    E01
......................................................................uggaccagugcaguuaauugaga................   3    0    E01

........................uugauuaaccacuguuguu..................................................................   1    0    TA1
........................uugauuaaccacuguuguuc.................................................................   3    0    TA1
........................uugauuaaccacuguuguuca................................................................   1    0    TA1
........................uugauuaaccacuguuguucagu..............................................................   1    0    TA1
......................................................................uggaccagugcaguuaauC....................   1    1    TA1
......................................................................uggaccagugcaguuaauu....................   3    0    TA1
......................................................................uggaccagugcaguuaauug...................   4    0    TA1
......................................................................uggaccagugcaguuaauuga..................   4    0    TA1
......................................................................uggaccagugcaguuaauugag.................   9    0    TA1
......................................................................uggaccagugcaguuaauugaga................   14   0    TA1
......................................................................uggaccagugcaguuaauugagaA...............   1    1    TA1
.......................................................................Ugaccagugcaguuaauug...................   2    1    TA1

........................uugauuaaccacuguuguu..................................................................   2    0    2nd
........................uugauuaaccacuguuguuc.................................................................   2    0    2nd
```

Mature   Star

```
aaacuaguaacauacacuagugccuugauuaaccacuguuguucaguuauccacaauuuuuugcuuuugcuggaccagugcaguuaauugagacaccagugcauauuca
........................uugauuaaccacuguuguuca................................................................	3	0	2nd
........................uugauuaaccacuguuguucagu..............................................................	5	0	2nd
........................uugauuaaccacuguuguucaguuauc..........................................................	1	0	2nd
......................................................................uggaccagugcaguuaauu....................	3	0	2nd
......................................................................uggaccagugcaguuaauug...................	2	0	2nd
......................................................................uggaccagugcaguuaauuga..................	5	0	2nd
......................................................................uggaccagugcaguuaauugag.................	2	0	2nd
......................................................................uAgaccagugcaguuaauugag.................	1	1	2nd
......................................................................uggaccagugcaguuaauugaga................	6	0	2nd

........................uugauuaaccacuguuguu..................................................................	21	0	H01
........................uugauuaaccacuguuguuc.................................................................	31	0	H01
........................uugCuuaaccacuguuguuca................................................................	1	1	H01
........................uugauuaaccacuguuguuca................................................................	39	0	H01
........................uugauuaGccacuguuguuca................................................................	1	1	H01
........................uugauuaaccacuguuguucC................................................................	1	1	H01
........................uugauuaaccacuguuguucagu..............................................................	72	0	H01
........................uugauuaaccacuguuguucaguu.............................................................	1	0	H01
.........................ugauuaaccacuguuguuc.................................................................	1	0	H01
.........................ugauuaGccacuguuguucagu..............................................................	1	1	H01
.........................ugauuaaccacuguuguucagu..............................................................	8	0	H01
............................uuaaccacuguuguucagu..............................................................	1	0	H01
.....................................................................cuggaccagugcaguuaauug...................	1	0	H01
.....................................................................cuggaccagugcaguuaauugagU................	1	1	H01
......................................................................uggaccagugcaguuaauu....................	3	0	H01
......................................................................uggaccagugcaguuaauug...................	4	0	H01
......................................................................uggaccagugcaguuaauuga..................	15	0	H01
......................................................................uggaccagugcaguuaauugag.................	7	0	H01
......................................................................uggaccagugcaguuaauugagU................	4	1	H01
......................................................................uggaccagugcaguuaauugaga................	17	0	H01

........................uugauuaaccacuguuguu..................................................................	2	0	LJ5
........................uugauuaaccacuguuguuc.................................................................	1	0	LJ5
.....................................................................cuggaccagugcaguuaauugagU................	1	1	LJ5
......................................................................uggaccagugcaguuaau.....................	1	0	LJ5
......................................................................uggaccagugcaguuaauu....................	1	0	LJ5
......................................................................uggaccagugcaguuaauC....................	1	1	LJ5
......................................................................uggaccagugcaguuaaGu....................	1	1	LJ5
......................................................................uggaccagugcaguuaauug...................	10	0	LJ5
......................................................................uggaccagugcaguuaauuga..................	8	0	LJ5
......................................................................uggaccagugcaguuaauugaA.................	3	1	LJ5
......................................................................uggaccagugcaguuaauugag.................	18	0	LJ5
......................................................................ugAaccagugcaguuaauugaga................	1	1	LJ5
......................................................................uggaccagugcaguuaauugagU................	3	1	LJ5
......................................................................uggaccagugcaguuaauugaga................	31	0	LJ5
......................................................................uggaccagugcaguuaauugGga................	1	1	LJ5
......................................................................ugUaccagugcaguuaauugaga................	1	1	LJ5

........................uugauuaaccacuguugu...................................................................	1	0	LA5
........................uugauuaaccacuguuguu..................................................................	4	0	LA5
........................uugauuaaccacuguCguuc.................................................................	1	1	LA5
........................uugauuaaccacuguuguuc.................................................................	2	0	LA5
......................................................................uggaccagugcaguuaauug...................	9	0	LA5
......................................................................uggaccagugcaguuaauuA...................	1	1	LA5
......................................................................uggaccagugcaguuaauuga..................	14	0	LA5
......................................................................uggaccagugcaguuaauugaA.................	1	1	LA5
......................................................................uggaccagugcaguuaauugag.................	24	0	LA5
......................................................................uggaccagugcaguuaauugGg.................	1	1	LA5
......................................................................uggaccagugcaguuaGuugag.................	1	1	LA5
......................................................................uggaccagugcUguuaauugaga................	1	1	LA5
......................................................................uggGccagugcaguuaauugaga................	1	1	LA5
......................................................................uggaccagugcaguuaauugagU................	13	1	LA5
......................................................................uggaccagugcaguuaauugagG................	2	1	LA5
......................................................................uggaccagugcagCuaauugaga................	5	1	LA5
......................................................................uggaccagugUaguuaauugaga................	1	1	LA5
......................................................................uggaccagugcaguuaauCgaga................	1	1	LA5
......................................................................uggaccagugcaguuaauugaga................	41	0	LA5
......................................................................uggaccagugcaguuaauugagaU...............	3	1	LA5
```

Provisional ID	:	Scaffold_11093_3149
Score total	:	58060.6
Score for star read(s)	:	3.9
Score for read counts	:	58051.9
Score for mfe	:	0.2
Score for randfold	:	1.6
Score for cons. seed	:	3
Total read count	:	113878
Mature read count	:	113852
Loop read count	:	0
Star read count	:	26

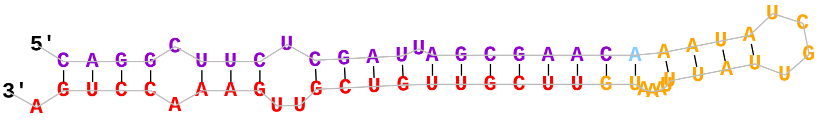

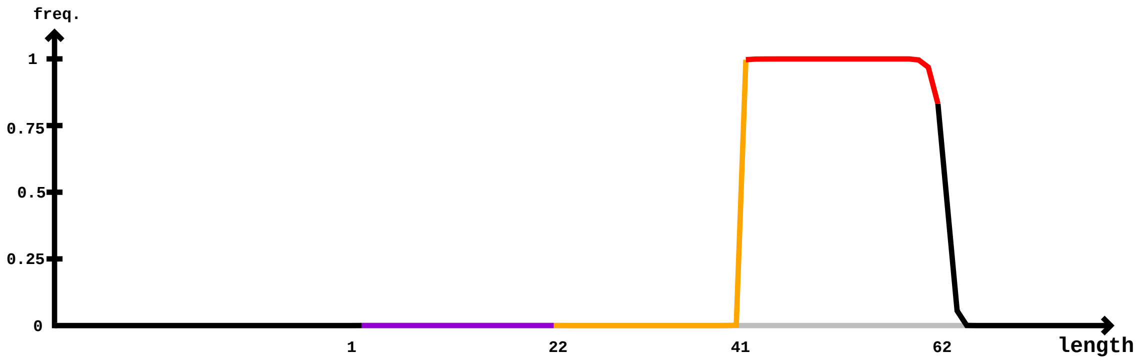

Star   Mature

```
5'- agcgguaggaaaucgagagaagauauuguacaggcuucucgauuagcgaacaaauaucguuauuuaaauguucguugucguugaaaccugaacgaaacuccucauucc   -3'    obs
    agcgguaggaaaucgagagaagauauuguacaggcuucucgauuagcgaacaaauaucguuauuuaaauguucguugucguugaaaccugaacgaaacuccucauucc          exp
    ....(.((((..((....)).....((((.((((.(((.((((.((((((((((((....))))....))))))))))))..))).)))).))))...))))).....   reads   mm   sample
    ............................acaggcuucucgauuagcgaac..........................................................   1       0    CJ1
    ..................................................................aauguucguugucguugaaaccC...................   1       1    CJ1
    .....................................................................guucguugucguugaaaccug..................   1       0    CJ1
    .....................................................................Cuucguugucguugaaaccug..................   3       1    CJ1
    .....................................................................Cuucguugucguugaaaccuga.................   1       1    CJ1
    ......................................................................uucguugucguugaaacU....................   12      1    CJ1
    ......................................................................uucguugucguugaaGcc....................   1       1    CJ1
    ......................................................................uucguugucguugaaacA....................   3       1    CJ1
    ......................................................................uucguugucguugaaacc....................   30      0    CJ1
    ......................................................................uucguugucguugaaaccG...................   1       1    CJ1
    ......................................................................uucguugucguugaGaccu...................   1       1    CJ1
    ......................................................................uucguugucguugaaaccu...................   58      0    CJ1
    ......................................................................uucguugucguugaaaccC...................   4       1    CJ1
    ......................................................................Cucguugucguugaaaccu...................   1       1    CJ1
    ......................................................................uucguugucguugaaaccA...................   7       1    CJ1
    ......................................................................uucguugucguugaaGccug..................   1       1    CJ1
    ......................................................................Gucguugucguugaaaccug..................   1       1    CJ1
    ......................................................................uucguugucguugaaaccuA..................   30      1    CJ1
    ......................................................................uucguugucguugaaaccuC..................   2       1    CJ1
    ......................................................................uuUguugucguugaaaccug..................   2       1    CJ1
    ......................................................................uucguuAucguugaaaccug..................   1       1    CJ1
    ......................................................................uucguugucguugaGaccug..................   2       1    CJ1
    ......................................................................uucguugucguugaaacUug..................   1       1    CJ1
    ......................................................................uucgCugucguugaaaccug..................   2       1    CJ1
    ......................................................................uucguugucguugaaaccGg..................   1       1    CJ1
    ......................................................................uucguugucguugGaaccug..................   6       1    CJ1
    ......................................................................uucguugucguugaaaccuU..................   7       1    CJ1
    ......................................................................uucguugucguugaaaccCg..................   2       1    CJ1
    ......................................................................uucguugucguugaaaccug..................   399     0    CJ1
    ......................................................................uucguugucguuCaaaccug..................   1       1    CJ1
    ......................................................................uuUguugucguugaaaccuga.................   2       1    CJ1
    ......................................................................uAcguugucguugaaaccuga.................   1       1    CJ1
    ......................................................................uuAguugucguugaaaccuga.................   1       1    CJ1
```

```
                                                Star                                     Mature
agcgguaggaaaucgagagaagauauuguacaggcuucucgauuagcgaacaaauaucguuauuuaaauguucguugucguugaaaccugaacgaaacuccucauucc
......................................................................uucguugucgCugaaaccuga.................   2      1    CJ1
......................................................................uucguGgucguugaaaccuga.................   1      1    CJ1
......................................................................uucgAugucguugaaaccuga.................   1      1    CJ1
......................................................................uucguugucguugaaaccugU.................   24     1    CJ1
......................................................................uucguugucguugaaGccuga.................   5      1    CJ1
......................................................................uucguuguUguugaaaccuga.................   1      1    CJ1
......................................................................uucguAgucguugaaaccuga.................   7      1    CJ1
......................................................................uucguuCucguugaaaccuga.................   2      1    CJ1
......................................................................uucguugucguugaaaccuga.................   1395   0    CJ1
......................................................................uucguugGcguugaaaccuga.................   2      1    CJ1
......................................................................uCcguugucguugaaaccuga.................   4      1    CJ1
......................................................................uucguugucguugUaaccuga.................   1      1    CJ1
......................................................................Gucguugucguugaaaccuga.................   2      1    CJ1
......................................................................uucgCugucguugaaaccuga.................   5      1    CJ1
......................................................................uucguugucAuugaaaccuga.................   2      1    CJ1
......................................................................uucguuAucguugaaaccuga.................   5      1    CJ1
......................................................................uucguugucguugGaaccuga.................   4      1    CJ1
......................................................................uucguugucguugaGaccuga.................   7      1    CJ1
......................................................................uucguugucguugaaaUcuga.................   1      1    CJ1
......................................................................uucguugucguugaaaccCga.................   7      1    CJ1
......................................................................uucguugCcguugaaaccuga.................   5      1    CJ1
......................................................................uucguugucgGugaaaccuga.................   1      1    CJ1
......................................................................uucguugucguugaaaccugC.................   5      1    CJ1
......................................................................uucguCgucguugaaaccuga.................   10     1    CJ1
......................................................................uucguugucguugaaCccuga.................   1      1    CJ1
......................................................................uucguugucguugaaaccuAa.................   1      1    CJ1
......................................................................uucguugucguugaaaccugG.................   23     1    CJ1
......................................................................uucAuugucguugaaaccuga.................   5      1    CJ1
......................................................................uucguugucguugaaacUuga.................   1      1    CJ1
......................................................................Cucguugucguugaaaccuga.................   5      1    CJ1
......................................................................uuGguugucguugaaaccuga.................   1      1    CJ1
......................................................................uucgGugucguugaaaccuga.................   1      1    CJ1
......................................................................uucguugucguugaaaccAga.................   1      1    CJ1
......................................................................uAcguugucguugaaaccugaa................   1      1    CJ1
......................................................................uucAuugucguugaaaccugaa................   1      1    CJ1
......................................................................uucguCgucguugaaaccugaa................   1      1    CJ1
......................................................................uucguugucgCugaaaccugaa................   1      1    CJ1
......................................................................uucgCugucguugaaaccugaa................   1      1    CJ1
......................................................................uucguugucguugaaaccugaU................   15     1    CJ1
......................................................................uucguugucguugaaaccugaa................   441    0    CJ1
......................................................................uucguugCcguugaaaccugaa................   1      1    CJ1
......................................................................uucguugucguugaaaccugGa................   1      1    CJ1
......................................................................uucguuAucguugaaaccugaa................   1      1    CJ1
......................................................................uucguuCucguugaaaccugaa................   1      1    CJ1
......................................................................uucguugucguugGaaccugaa................   3      1    CJ1
......................................................................uucguuguUguugaaaccugaa................   1      1    CJ1
......................................................................Aucguugucguugaaaccugaa................   2      1    CJ1
......................................................................uucguugucguugaGaccugaa................   1      1    CJ1
......................................................................uCcguugucguugaaaccugaa................   1      1    CJ1
......................................................................uucguugucguugaaaccugaC................   166    1    CJ1
......................................................................uuAguugucguugaaaccugaa................   1      1    CJ1
......................................................................uucguugucguugaaGccugaa................   3      1    CJ1
......................................................................uucguugucguugaaaccugaG................   5      1    CJ1
......................................................................uucguugucguugaaaccugaaA...............   25     1    CJ1
......................................................................uucguugucguugaaaccugaaU...............   27     1    CJ1
......................................................................uucguugucguugaaaccugaCc...............   59     1    CJ1
......................................................................uucguugucguugaaaccugaaG...............   2      1    CJ1
......................................................................uucguugucguugGaaccugaac...............   2      1    CJ1
......................................................................uucguugucguugaaaccugaac...............   11     0    CJ1
......................................................................uucguugucguugaaaccugaacU..............   1      1    CJ1
.......................................................................ucguugucguugaaaccug..................   1      0    CJ1
........................................................................cguugucguugaaaccuga.................   1      0    CJ1
.........................................................................guugucguugaaaccugaa................   1      0    CJ1

.....................................................................guucguugucguugaaaccug..................   1      0    G01
......................................................................uucguugucguugaaaccu...................   2      0    G01
......................................................................uucguugucguugaaaccug..................   16     0    G01
......................................................................uucguugucguugaaaccuga.................   9      0    G01
......................................................................uucguugucguugaaaccugaU................   1      1    G01
......................................................................uucguugucguugaaaccugaa................   7      0    G01
```

Star Mature

```
agcgguaggaaaucgagagaagauauuguacaggcuucucgauuagcgaacaaauaucguuauuuaaauguucguugucguugaaaccugaacgaaacuccucauucc
......................................................................uucguugucguugaaaccugaC................ 2 1 G01
......................................................................uucguugucguugaaaccugaCc............... 1 1 G01
......................................................................uucguugucguugaaaccugaaG............... 1 1 G01
......................................................................uucguugucguugaaaccugaacC.............. 1 1 G01
.......................................................................ucguugucguugaaaccug.................. 1 0 G01
........................................................................cguugucguugaaaccugaaG............... 1 1 G01

......................................................................uCcguugucguugaaacc.................... 1 1 HJ1
......................................................................uucguugucguugaaacc.................... 8 0 HJ1
......................................................................uucguugucguugaaaccG................... 1 1 HJ1
......................................................................uucguugucguugaaaccC................... 2 1 HJ1
......................................................................uucguugucguugaaaccu................... 10 0 HJ1
......................................................................uucguugucguugaaUccug.................. 1 1 HJ1
......................................................................uucguugucguugaaaUcug.................. 1 1 HJ1
......................................................................uucguugucgCugaaaccug.................. 1 1 HJ1
......................................................................uucguugucguugGaaccug.................. 1 1 HJ1
......................................................................uucguugucguugaaaccug.................. 62 0 HJ1
......................................................................uucgAugucguugaaaccug.................. 1 1 HJ1
......................................................................uucguuguUguugaaaccug.................. 2 1 HJ1
......................................................................uucguugucguugaaaccAg.................. 1 1 HJ1
......................................................................Aucguugucguugaaaccug.................. 1 1 HJ1
......................................................................uucguugucguugaaaccuA.................. 5 1 HJ1
......................................................................uCcguugucguugaaaccuga................. 3 1 HJ1
......................................................................uucguugucguugaaaccuga................. 682 0 HJ1
......................................................................uucguugucguugaaaccuCa................. 1 1 HJ1
......................................................................uucgAugucguugaaaccuga................. 5 1 HJ1
......................................................................uucguugucguugaaCccuga................. 2 1 HJ1
......................................................................uucguuguUguugaaaccuga................. 2 1 HJ1
......................................................................Aucguugucguugaaaccuga................. 1 1 HJ1
......................................................................uucguugCcguugaaaccuga................. 3 1 HJ1
......................................................................uucguCgucguugaaaccuga................. 5 1 HJ1
......................................................................uucguugucguugaaaccugG................. 10 1 HJ1
......................................................................uucguugucguAgaaaccuga................. 5 1 HJ1
......................................................................uucguugucguugaaaccCga................. 2 1 HJ1
......................................................................uucguugucguugaaaccuAa................. 1 1 HJ1
......................................................................uucguugucguugaaaccugU................. 23 1 HJ1
......................................................................uucguugucguugGaaccuga................. 6 1 HJ1
......................................................................uucgCugucguugaaaccuga................. 2 1 HJ1
......................................................................uucguuguAguugaaaccuga................. 1 1 HJ1
......................................................................uucguuAucguugaaaccuga................. 4 1 HJ1
......................................................................uAcguugucguugaaaccuga................. 2 1 HJ1
......................................................................uucguugucguuCaaaccuga................. 1 1 HJ1
......................................................................uucguugucguGgaaaccuga................. 2 1 HJ1
......................................................................uucguugucgCugaaaccuga................. 3 1 HJ1
......................................................................uucguAgucguugaaaccuga................. 3 1 HJ1
......................................................................uuUguugucguugaaaccuga................. 1 1 HJ1
......................................................................Cucguugucguugaaaccuga................. 6 1 HJ1
......................................................................uucguugucguugaaaccGga................. 6 1 HJ1
......................................................................uucguugucguugaGaccuga................. 2 1 HJ1
......................................................................uucguugucguugaaaccugaC................ 15 1 HJ1
......................................................................uucguugucguugaaaccugUa................ 1 1 HJ1
......................................................................Cucguugucguugaaaccugaa................ 1 1 HJ1
......................................................................uuUguugucguugaaaccugaa................ 1 1 HJ1
......................................................................uucgGugucguugaaaccugaa................ 1 1 HJ1
......................................................................uucguugucguugaaaccugaa................ 109 0 HJ1
......................................................................uucguugucguugaaaUcugaa................ 1 1 HJ1
......................................................................uucguugucguugaaaccugaU................ 3 1 HJ1
......................................................................uucguugucguugaaaccugGa................ 1 1 HJ1
......................................................................uucguugucguAgaaaccugaa................ 1 1 HJ1
......................................................................uucguCgucguugaaaccugaa................ 1 1 HJ1
......................................................................uucguugucguugCaaccugaa................ 1 1 HJ1
......................................................................uucguugucgCugaaaccugaa................ 1 1 HJ1
......................................................................Aucguugucguugaaaccugaa................ 1 1 HJ1
......................................................................uucguugucguugUaaccugaa................ 2 1 HJ1
......................................................................uucguugucguugaaaccugaG................ 3 1 HJ1
......................................................................uucguugucguugaaaccugaCc............... 5 1 HJ1
......................................................................uucguugucguugaaaccugaaU............... 12 1 HJ1
......................................................................uucguugucguAgaaaccugaac............... 1 1 HJ1
......................................................................uucguugucguugaaaccugaaA............... 4 1 HJ1
......................................................................uucguugucguugaaaccugaac............... 4 0 HJ1
```

Star **Mature**

```
agcgguaggaaaucgagagaagauauuguacaggcuucucgauuagcgaacaaauaucguuauuuaaauguucguugucguugaaaccugaacgaaacuccucauucc
......................................................................uucguugucguugaaaccugaacC..............   2     1   HJ1
......................................................................uucguugucguugaaaccugaacU..............   1     1   HJ1
......................................................................uucguugucguCgaaaccugaacg..............   6     1   HJ1

......................................................................uucguugucguugaaacc....................   2     0   LA1
......................................................................uucguugucguugaaacU....................   1     1   LA1
......................................................................uucguugucguugaaaccu...................   1     0   LA1
......................................................................uucguugucguugaaaccug..................   30    0   LA1
......................................................................uucguugucguugaaGccuga.................   1     1   LA1
......................................................................uucguugucguugaaacUuga.................   1     1   LA1
......................................................................uucguugucguugaaaccugG.................   5     1   LA1
......................................................................uucguugucguugaGaccuga.................   1     1   LA1
......................................................................uucguugucguugaaaccuga.................   183   0   LA1
......................................................................uucguugucguugaaaGcuga.................   1     1   LA1
......................................................................uucguugucgCugaaaccuga.................   1     1   LA1
......................................................................uucguCgucguugaaaccugaa................   1     1   LA1
......................................................................Cucguugucguugaaaccugaa................   2     1   LA1
......................................................................uucguuAucguugaaaccugaa................   1     1   LA1
......................................................................uucguugucAuugaaaccugaa................   2     1   LA1
......................................................................uuUguugucguugaaaccugaa................   2     1   LA1
......................................................................uCcguugucguugaaaccugaa................   1     1   LA1
......................................................................uucguugucguugaaaccugaC................   53    1   LA1
......................................................................uucguugucguugaaaccugGa................   1     1   LA1
......................................................................uucguugucguugGaaccugaa................   4     1   LA1
......................................................................uucguAgucguugaaaccugaa................   1     1   LA1
......................................................................uucAuugucguugaaaccugaa................   1     1   LA1
......................................................................uucguugucguugaGaccugaa................   1     1   LA1
......................................................................uucguugucguugaaaccugaa................   399   0   LA1
......................................................................uucguugucguugaaaccuUaa................   1     1   LA1
......................................................................uucguugucguugaaaccugaG................   2     1   LA1
......................................................................uucguugCcguugaaaccugaa................   2     1   LA1
......................................................................uucguugucguugaaaccugaU................   3     1   LA1
......................................................................uucguugucguugaaaccCgaa................   2     1   LA1
......................................................................uucguugucguugaaaccugaaU...............   2     1   LA1
......................................................................uucguugucguugaaaccugaCc...............   29    1   LA1
......................................................................uucguugucguugaaaccugaaA...............   11    1   LA1
......................................................................uucguugucguugaaaccugaac...............   1     0   LA1

......................................................................uucguugucguugaaaccuU..................   1     1   BA1
......................................................................uucguugucguugaaaccuga.................   1     0   BA1
......................................................................uucguugucguugaaaccugaa................   2     0   BA1

......................................................................uucguugucguugaaaccu...................   1     0   BRA
......................................................................uucguugucguugaaaccug..................   4     0   BRA
......................................................................uucguugucguugaaaccuA..................   2     1   BRA
......................................................................uucguugucguugaaGccuga.................   1     1   BRA
......................................................................uucguugucguugaaaccGga.................   1     1   BRA
......................................................................uucguugucguugaaaccuga.................   45    0   BRA
......................................................................uucguugucguuAaaaccuga.................   1     1   BRA
......................................................................uucguugucguugaaaccugaa................   36    0   BRA
......................................................................uucguugucguugaaaccugaC................   4     1   BRA
......................................................................uucguugucgCugaaaccugaa................   1     1   BRA
......................................................................uucgCugucguugaaaccugaa................   1     1   BRA
......................................................................uucguugucguugaaaccugaU................   3     1   BRA
......................................................................uucguugucguugaaacUugaa................   2     1   BRA
......................................................................uucguugucguugaaaccugaaA...............   2     1   BRA
......................................................................uucguugucguugaaaccugaCc...............   1     1   BRA
......................................................................uucguugucguugaaaccugaac...............   1     0   BRA
......................................................................uucguugucguugaaaccugaaG...............   1     1   BRA
......................................................................uucguugucguugaaaccugaaU...............   4     1   BRA

..............................caggcuucucgauuagcgaaca........................................................   1     0   3rd
..............................caggcuucucgauuagcgaacaU.......................................................   1     1   3rd
...............................aggcuucucgauuagcgaaca........................................................   1     0   3rd
...................................................................aGguucguugucguugaaaccugaa................   1     1   3rd
....................................................................uUuucguugucguugaaaccug..................   2     1   3rd
....................................................................uguucguugucguugaaaccug..................   1     0   3rd
....................................................................uUuucguugucguugaaaccuga.................   3     1   3rd
....................................................................uUuucguugucguugaaaccugaa................   1     1   3rd
....................................................................uguucguugucguugaaaccugaa................   1     0   3rd
```

Star **Mature**

agcgguaggaaaucgagagaagauauuguacaggcuucucgauuagcgaacaaauaucguuauuuaaauguucguugucguugaaaccugaacgaaacuccucauucc			
....guucguugucguugaaaccu...................	4	0	3rd
....guucguugucguugaaaccug..................	2	0	3rd
....Uuucguugucguugaaaccug..................	1	1	3rd
....guucguugucguugaaaccuga.................	7	0	3rd
....Uuucguugucguugaaaccuga.................	6	1	3rd
....guucguugucguugaaaccugaC................	1	1	3rd
....Uuucguugucguugaaaccugaa................	4	1	3rd
....Uuucguugucguugaaaccugaac...............	1	1	3rd
....uucguugucguugaaacc....................	70	0	3rd
....uucguugucguugaaaccA...................	4	1	3rd
....uucguugucguugUaaccu...................	1	1	3rd
....uucguuAucguugaaaccu...................	1	1	3rd
....uucgCugucguugaaaccu...................	1	1	3rd
....uucguGgucguugaaaccu...................	2	1	3rd
....uAcguugucguugaaaccu...................	1	1	3rd
....uucguugucguugaaaccC...................	10	1	3rd
....uucguugucguugaaaccu...................	693	0	3rd
....uucguAgucguugaaaccu...................	1	1	3rd
....uucguuUucguugaaaccu...................	1	1	3rd
....uucguugucgCugaaaccu...................	1	1	3rd
....uucguugucguugGaaccu...................	2	1	3rd
....uucguugCcguugaaaccu...................	1	1	3rd
....uucguuguUguugaaaccu...................	2	1	3rd
....Cucguugucguugaaaccug..................	2	1	3rd
....uucUuugucguugaaaccug..................	1	1	3rd
....uAcguugucguugaaaccug..................	5	1	3rd
....uucgAugucguugaaaccug..................	1	1	3rd
....uucguugucguugaaGccug..................	1	1	3rd
....uucguuAucguugaaaccug..................	1	1	3rd
....uucguugucguugaaaccGg..................	3	1	3rd
....uucguAgucguugaaaccug..................	3	1	3rd
....uucCuugucguugaaaccug..................	1	1	3rd
....uucguugCcguugaaaccug..................	3	1	3rd
....uucguugucguugaaacUug..................	1	1	3rd
....Aucguugucguugaaaccug..................	1	1	3rd
....uucguugucguugaaaUcug..................	1	1	3rd
....uucguugucguugaGaccug..................	1	1	3rd
....uucguugucguugaaaccuC..................	1	1	3rd
....uucguugucguugGaaccug..................	2	1	3rd
....uucguuguUguugaaaccug..................	1	1	3rd
....uucguugucguugaaacGug..................	2	1	3rd
....uucguugAcguugaaaccug..................	2	1	3rd
....uucguugucguugUaaccug..................	2	1	3rd
....uucguugGcguugaaaccug..................	1	1	3rd
....uucguugucguugaaaccAg..................	6	1	3rd
....uucgCugucguugaaaccug..................	2	1	3rd
....uucguugucguugaaaccuA..................	25	1	3rd
....uucguugucguugaaaccuU..................	4	1	3rd
....uucguugucguugaCaccug..................	1	1	3rd
....uuAguugucguugaaaccug..................	1	1	3rd
....uucguugucgCugaaaccug..................	1	1	3rd
....uGcguugucguugaaaccug..................	1	1	3rd
....uucguugucguugaaaccCg..................	9	1	3rd
....uucguCgucguugaaaccug..................	2	1	3rd
....uucguugucguugaaaccug..................	3689	0	3rd
....uucguugucguugCaaccug..................	1	1	3rd
....uucguGgucguugaaaccug..................	1	1	3rd
....uucguugucgGugaaaccug..................	1	1	3rd
....uucguCgucguugaaaccuga.................	6	1	3rd
....Aucguugucguugaaaccuga.................	1	1	3rd
....uucguugucguugaaaccugC.................	5	1	3rd
....uucguugucguuCaaaccuga.................	2	1	3rd
....uucgCugucguugaaaccuga.................	2	1	3rd
....uucguugucguugaaaccugU.................	40	1	3rd
....uucguugCcguugaaaccuga.................	3	1	3rd
....uucguugucgGugaaaccuga.................	2	1	3rd
....uucguugucCuugaaaccuga.................	4	1	3rd
....uucguugucguugaaaccugG.................	6	1	3rd
....uucguugucguugaaaccGga.................	1	1	3rd
....uucguuUucguugaaaccuga.................	7	1	3rd

```
                              Star                                    Mature
agcgguaggaaaucgagagaagauauuguacaggcuucucgauuagcgaacaaauaucguuauuuaaauguucguugucguugaaaccugaacgaaacuccucauucc
......................................................................uucguugucguugaaaccuCa.................    4      1    3rd
......................................................................uucguugucguugUaaccuga.................    6      1    3rd
......................................................................uucguugucguugaaCccuga.................    2      1    3rd
......................................................................uucguugucguugaaacAuga.................    1      1    3rd
......................................................................uucguugucguugaaacUuga.................    9      1    3rd
......................................................................uucguugucguugGaaccuga.................    9      1    3rd
......................................................................uucguugucguugaaGccuga.................    3      1    3rd
......................................................................uucguugGcguugaaaccuga.................    1      1    3rd
......................................................................uucguuguAguugaaaccuga.................    1      1    3rd
......................................................................uCcguugucguugaaaccuga.................    3      1    3rd
......................................................................uucgGugucguugaaaccuga.................    1      1    3rd
......................................................................uucguugucAuugaaaccuga.................    2      1    3rd
......................................................................uucguugucguugaaaccCga.................    7      1    3rd
......................................................................uucguugucguugaCaccuga.................    3      1    3rd
......................................................................uucgAugucguugaaaccuga.................    1      1    3rd
......................................................................Gucguugucguugaaaccuga.................    2      1    3rd
......................................................................uucguugucguuUaaaccuga.................    1      1    3rd
......................................................................uucguGgucguugaaaccuga.................    7      1    3rd
......................................................................uucguugucgCugaaaccuga.................    4      1    3rd
......................................................................uuAguugucguugaaaccuga.................    2      1    3rd
......................................................................uucguuCucguugaaaccuga.................    5      1    3rd
......................................................................uucguugucguugaaaccuAa.................    10     1    3rd
......................................................................uucguugucguugaGaccuga.................    3      1    3rd
......................................................................uucguugucUuugaaaccuga.................    1      1    3rd
......................................................................uAcguugucguugaaaccuga.................    8      1    3rd
......................................................................uucguuguUguugaaaccuga.................    4      1    3rd
......................................................................uucguugucguAgaaaccuga.................    2      1    3rd
......................................................................uucguugucguugCaaccuga.................    1      1    3rd
......................................................................uucguugucguugaaaGcuga.................    1      1    3rd
......................................................................uucguugucguugaaaUcuga.................    2      1    3rd
......................................................................uuUguugucguugaaaccuga.................    8      1    3rd
......................................................................uucguugucguugaUaccuga.................    1      1    3rd
......................................................................uucguAgucguugaaaccuga.................    13     1    3rd
......................................................................uuGguugucguugaaaccuga.................    1      1    3rd
......................................................................uucguugucguugaaaccuUa.................    5      1    3rd
......................................................................uucguugucguugaaaccuga.................    16072  0    3rd
......................................................................uucguugucguGgaaaccuga.................    1      1    3rd
......................................................................uucUuugucguugaaaccuga.................    3      1    3rd
......................................................................uucguugucguugaaacGuga.................    7      1    3rd
......................................................................uucguugucgAugaaaccuga.................    3      1    3rd
......................................................................uucguugucguugaaUccuga.................    2      1    3rd
......................................................................uucguugucguugaaaccAga.................    2      1    3rd
......................................................................uGcguugucguugaaaccuga.................    1      1    3rd
......................................................................uucguugucguugaCaccugaa................    3      1    3rd
......................................................................uucguugAcguugaaaccugaa................    2      1    3rd
......................................................................uucguugucUuugaaaccugaa................    1      1    3rd
......................................................................uucUuugucguugaaaccugaa................    1      1    3rd
......................................................................uucguugucguugaaacAugaa................    1      1    3rd
......................................................................uucguuCucguugaaaccugaa................    1      1    3rd
......................................................................uucguugucguugaaaUcugaa................    2      1    3rd
......................................................................uucguAgucguugaaaccugaa................    9      1    3rd
......................................................................uucguugucgAugaaaccugaa................    1      1    3rd
......................................................................uucguugucguugaaGccugaa................    1      1    3rd
......................................................................uucguugucguugaaaccugaa................    6494   0    3rd
......................................................................uucguugucguugaaaccugaG................    7      1    3rd
......................................................................uucguuUucguugaaaccugaa................    3      1    3rd
......................................................................uucguGgucguugaaaccugaa................    1      1    3rd
......................................................................uucguugCcguugaaaccugaa................    2      1    3rd
......................................................................uucguugucguugaaaccuCaa................    3      1    3rd
......................................................................uucguugucguugaaaccGgaa................    3      1    3rd
......................................................................uucguugucguugaaaccuUaa................    3      1    3rd
......................................................................uucguugucguugGaaccugaa................    4      1    3rd
......................................................................uucguugucguugCaaccugaa................    2      1    3rd
......................................................................uucguugucguugaaaccugCa................    2      1    3rd
......................................................................uucguugucguugaaaGcugaa................    2      1    3rd
......................................................................uucguugucguugUaaccugaa................    5      1    3rd
......................................................................uucguugGcguugaaaccugaa................    1      1    3rd
......................................................................uucguugucAuugaaaccugaa................    1      1    3rd
......................................................................uucguugucCuugaaaccugaa................    3      1    3rd
......................................................................uuAguugucguugaaaccugaa................    4      1    3rd
```

```
                              Star                          Mature
agcgguaggaaaucgagagaagauauuguacaggcuucucgauuagcgaacaaauaucguuauuuaaauguucguugucguugaaaccugaacgaaacuccucauucc
......................................................................uucguugucguugaUaccugaa................   1      1      3rd
......................................................................uucguugucguugaaCccugaa................   2      1      3rd
......................................................................uAcguugucguugaaaccugaa................   4      1      3rd
......................................................................uucguugucguugaaacGugaa................   3      1      3rd
......................................................................uucguugucguugaaaccugaU................   64     1      3rd
......................................................................uucguugucguugaaaccugaC................   1370   1      3rd
......................................................................uucguugucguugaaaccCgaa................   3      1      3rd
......................................................................uGcguugucguugaaaccugaa................   1      1      3rd
......................................................................uucguugucguuAaaaccugaa................   2      1      3rd
......................................................................Cucguugucguugaaaccugaa................   3      1      3rd
......................................................................uucguugucguugaGaccugaa................   1      1      3rd
......................................................................uucguugucguugaaacUugaa................   2      1      3rd
......................................................................uuUguugucguugaaaccugaa................   3      1      3rd
......................................................................uucguugucguugaaaccugUa................   2      1      3rd
......................................................................uucguCgucguugaaaccugaa................   3      1      3rd
......................................................................uucguugucguugaaaccugGa................   7      1      3rd
......................................................................uucguugucguugaaaccugaaA...............   979    1      3rd
......................................................................uucguugucguugaaaccugaCc...............   390    1      3rd
......................................................................uucguugucguugaaacGugaac...............   1      1      3rd
......................................................................uucguugucguugaaaccugaac...............   102    0      3rd
......................................................................uucguugucguugaaaccugaaG...............   34     1      3rd
......................................................................uucguugucguugaaaccugaaU...............   328    1      3rd
......................................................................uucguugucguugaaaccugaacU..............   3      1      3rd
......................................................................uucguugucguCgaaaccugaacg..............   2      1      3rd
.......................................................................ucguugucguugaaaccu...................   3      0      3rd
.......................................................................ucguugucguugaaaccug..................   8      0      3rd
.......................................................................ucguugucguugaaaccuga.................   41     0      3rd
.......................................................................ucguuguUguugaaaccuga.................   1      1      3rd
.......................................................................ucguugucguugaaaccugaC................   1      1      3rd
.......................................................................ucguugucguugaaaccugaa................   22     0      3rd
.......................................................................ucguugucguugaaaccugaU................   1      1      3rd
.......................................................................ucguugucguugaaaccugaaA...............   5      1      3rd
.......................................................................ucguugucguugaaaccugaaG...............   1      1      3rd
.......................................................................ucguugucguugaaaccugaac...............   2      0      3rd
.......................................................................ucguugucguugaaaccugaaU...............   7      1      3rd
.......................................................................ucguugucguugaaaccugaCc...............   5      1      3rd
........................................................................cguugucguugaaaccug..................   1      0      3rd
........................................................................cguugucguugaaaccuga.................   9      0      3rd
........................................................................cguugucguugaaaccugaa................   1      0      3rd
........................................................................cguugucguugaaaccugaU................   1      1      3rd
........................................................................cguugucguugaaaccugaaA...............   1      1      3rd
.........................................................................guugucguugaaaccuga.................   1      0      3rd
.........................................................................guugucguugaaaccugaa................   1      0      3rd

.....................................................................guucguugucguugaaaccug..................   2      0      CA1
.....................................................................Cuucguugucguugaaaccugaa................   1      1      CA1
......................................................................uucguugucguugaaacc....................   4      0      CA1
......................................................................uucguugucguugaaaccu...................   2      0      CA1
......................................................................uucguugucguugaaaccug..................   33     0      CA1
......................................................................uucguugucAuugaaaccug..................   1      1      CA1
......................................................................uucguugucguugaaGccug..................   1      1      CA1
......................................................................uucguugucguugaaaccGg..................   1      1      CA1
......................................................................uucguugucguugaaaccuA..................   3      1      CA1
......................................................................uucguugucguugaaaccCg..................   3      1      CA1
......................................................................uucguugucguugaaaccuU..................   1      1      CA1
......................................................................uucguugucguugGaaccuga.................   1      1      CA1
......................................................................uucguugucguugaaacUuga.................   1      1      CA1
......................................................................uucguugucguugaaaccuga.................   303    0      CA1
......................................................................Cucguugucguugaaaccuga.................   2      1      CA1
......................................................................uucguugucguugaaGccuga.................   3      1      CA1
......................................................................uucguugucguugaaaccCga.................   1      1      CA1
......................................................................uucguugucguugaaaccugU.................   2      1      CA1
......................................................................uucguugucgCugaaaccuga.................   1      1      CA1
......................................................................uucguugucguugaaaccugG.................   2      1      CA1
......................................................................uucguugucAuugaaaccuga.................   1      1      CA1
......................................................................uucguugucguugaaaccugaa................   269    0      CA1
......................................................................uucguugucguugaGaccugaa................   1      1      CA1
......................................................................uucguugucguugaaaccugaU................   5      1      CA1
......................................................................uucguugucguugaaGccugaa................   1      1      CA1
......................................................................Cucguugucguugaaaccugaa................   1      1      CA1
```

```
                              Star                                          Mature
agcgguaggaaaucgagagaagauauuguacaggcuucucgauuagcgaacaaauaucguuauuuaaauguucguugucguugaaaccugaacgaaacuccucauucc
......................................................................uCcguugucguugaaaccugaa................    1     1    CA1
......................................................................uucguugucguuAaaaccugaa................    1     1    CA1
......................................................................uucguugucguugaaaccugaC................    41    1    CA1
......................................................................uucguugCcguugaaaccugaa................    6     1    CA1
......................................................................uucguugucguugaaaccugGa................    3     1    CA1
......................................................................uucguugucguugaaaccugaCc...............    13    1    CA1
......................................................................uucguugucguugaaaccugaac...............    1     0    CA1
......................................................................uucguugucguugaaaccugaaU...............    4     1    CA1
......................................................................uucguugucguugaaaccugaaA...............    4     1    CA1

......................................................................uucguugucguugaaaccu...................    1     0    H01
......................................................................uucguugucguugaaaccug..................    2     0    H01
......................................................................uucguugucguugaaaccuga.................    2     0    H01
......................................................................uucguugucguugaaaccugaa................    4     0    H01

..............................caggcuucucgauuagcgaaca........................................................    1     0    LJ5
.....................................................................guucgCugucguugaaacc....................    2     1    LJ5
......................................................................uucguugucguugGaacc....................    1     1    LJ5
......................................................................uucguugucguugaaacU....................    6     1    LJ5
......................................................................uucguugucguugaaacc....................    13    0    LJ5
......................................................................uCcguugucguugaaacc....................    1     1    LJ5
......................................................................uucguugucguugaaacA....................    1     1    LJ5
......................................................................uucguugucguugaaaccC...................    7     1    LJ5
......................................................................uucgCugucguugaaaccu...................    1     1    LJ5
......................................................................uucguugucguugaaaccu...................    39    0    LJ5
......................................................................uucguugucguugaaaccA...................    4     1    LJ5
......................................................................uucguugucguugaaaccAg..................    1     1    LJ5
......................................................................uucguugucguugaaaccuC..................    2     1    LJ5
......................................................................uucguugucguugGaaccug..................    1     1    LJ5
......................................................................uucguugucguugaGaccug..................    1     1    LJ5
......................................................................uucguugucguugaaaccuA..................    15    1    LJ5
......................................................................uucguCgucguugaaaccug..................    2     1    LJ5
......................................................................uucguugucguugaaaccuU..................    1     1    LJ5
......................................................................uucguuAucguugaaaccug..................    1     1    LJ5
......................................................................uucguugucguugaaaccug..................    219   0    LJ5
......................................................................uucguugucguugaaaccGg..................    2     1    LJ5
......................................................................uucguugucguugaaaccCg..................    2     1    LJ5
......................................................................uucguugucguuAaaaccug..................    1     1    LJ5
......................................................................Cucguugucguugaaaccuga.................    2     1    LJ5
......................................................................uucguugCcguugaaaccuga.................    1     1    LJ5
......................................................................uucguugucgCugaaaccuga.................    10    1    LJ5
......................................................................uucAuugucguugaaaccuga.................    4     1    LJ5
......................................................................uucguugucguugaGaccuga.................    6     1    LJ5
......................................................................uuGguugucguugaaaccuga.................    1     1    LJ5
......................................................................uucguuguUguugaaaccuga.................    1     1    LJ5
......................................................................uucguugucguugaaaccCga.................    5     1    LJ5
......................................................................uucguCgucguugaaaccuga.................    1     1    LJ5
......................................................................uucguugucguugaaaccugC.................    1     1    LJ5
......................................................................uuAguugucguugaaaccuga.................    2     1    LJ5
......................................................................uucguugucguugaaaccuga.................    876   0    LJ5
......................................................................uucguugucguugGaaccuga.................    6     1    LJ5
......................................................................uucgCugucguugaaaccuga.................    4     1    LJ5
......................................................................uuUguugucguugaaaccuga.................    4     1    LJ5
......................................................................uucguugucguugaaaccugG.................    8     1    LJ5
......................................................................uucguuCucguugaaaccuga.................    2     1    LJ5
......................................................................uucguuAucguugaaaccuga.................    1     1    LJ5
......................................................................uCcguugucguugaaaccuga.................    1     1    LJ5
......................................................................uucguugucguugaaaccAga.................    1     1    LJ5
......................................................................uucguugucguugaaaccugU.................    9     1    LJ5
......................................................................Aucguugucguugaaaccuga.................    1     1    LJ5
......................................................................uucCuugucguugaaaccuga.................    1     1    LJ5
......................................................................uucguugucguugaaGccuga.................    6     1    LJ5
......................................................................uucguugucguugaaacUuga.................    1     1    LJ5
......................................................................uucguugucguugaGaccugaa................    1     1    LJ5
......................................................................uuUguugucguugaaaccugaa................    1     1    LJ5
......................................................................uucguugucguugaaaccugaU................    4     1    LJ5
......................................................................uucguugucguugaaaccugaC................    62    1    LJ5
......................................................................Cucguugucguugaaaccugaa................    2     1    LJ5
......................................................................uCcguugucguugaaaccugaa................    1     1    LJ5
......................................................................uucguuguGguugaaaccugaa................    2     1    LJ5
```

```
                             Star                          Mature
agcgguaggaaaucgagagaagauauuguacaggcuucucgauuagcgaacaaauaucguuauuuaaauguucguugucguugaaaccugaacgaaacuccucauucc
......................................................................uuAguugucguugaaaccugaa................   1     1   LJ5
......................................................................uucguuCucguugaaaccugaa................   1     1   LJ5
......................................................................uucguugucguugaaaccugaG................   2     1   LJ5
......................................................................uucguugucguugaaaccugaa................   175   0   LJ5
......................................................................uucguugucAuugaaaccugaa................   1     1   LJ5
......................................................................uucguugucguugaaaccugaaA...............   7     1   LJ5
......................................................................uucguugucguugaaGccugaac...............   1     1   LJ5
......................................................................uucguugucguugaaaccugaaG...............   2     1   LJ5
......................................................................uucguugucguugaaaccugaCc...............   39    1   LJ5
......................................................................uucguugucguugaaaccugaUc...............   1     1   LJ5
......................................................................uucguugucguugaaaccugaaU...............   14    1   LJ5
......................................................................uucguugucguugaaaccugaac...............   4     0   LJ5

..............................caggcuucucgauuagcgaac.........................................................   4     0   2nd
..............................caggcuucucgauuagcgaaca........................................................   3     0   2nd
...............................aggcuucucgauuagcgaac.........................................................   1     0   2nd
...............................aggcuucucgauuagcgaaca........................................................   1     0   2nd
..................................cuucucgauuagcgaaca........................................................   1     0   2nd
....................................................................uUucguugucguugaaaccuga..................   1     1   2nd
.....................................................................Uuucguugucguugaaaccu...................   1     1   2nd
.....................................................................guucguugucguugaaaccug..................   6     0   2nd
.....................................................................Uuucguugucguugaaaccug..................   1     1   2nd
.....................................................................guucguugucguugaaaccuga.................   5     0   2nd
.....................................................................Uuucguugucguugaaaccuga.................   8     1   2nd
.....................................................................Uuucguugucguugaaaccugaa................   8     1   2nd
.....................................................................guucguugucguugaaaccugaa................   1     0   2nd
......................................................................uucguugucguugaaacc....................   46    0   2nd
......................................................................uucguugucguugGaacc....................   1     1   2nd
......................................................................uucgGugucguugaaaccu...................   1     1   2nd
......................................................................uucguGgucguugaaaccu...................   2     1   2nd
......................................................................uucguugucguugaaacGu...................   1     1   2nd
......................................................................uucguugucguugaUaccu...................   1     1   2nd
......................................................................uucguugucguugaaaccA...................   6     1   2nd
......................................................................uucguugucguugaaaccu...................   589   0   2nd
......................................................................Cucguugucguugaaaccu...................   1     1   2nd
......................................................................uucguugucguugaaaccC...................   8     1   2nd
......................................................................uucguugucguugaGaccu...................   1     1   2nd
......................................................................uucguugucguugaaaccGg..................   2     1   2nd
......................................................................uucguugucguugaGaccug..................   5     1   2nd
......................................................................uucguugucguugaUaccug..................   3     1   2nd
......................................................................uucguugucUuugaaaccug..................   1     1   2nd
......................................................................uucguugucguugGaaccug..................   4     1   2nd
......................................................................uucguCgucguugaaaccug..................   4     1   2nd
......................................................................uucguAgucguugaaaccug..................   1     1   2nd
......................................................................uucguugucguugCaaccug..................   1     1   2nd
......................................................................uucguuguGguugaaaccug..................   1     1   2nd
......................................................................uuUguugucguugaaaccug..................   1     1   2nd
......................................................................uucguugGcguugaaaccug..................   1     1   2nd
......................................................................uAcguugucguugaaaccug..................   3     1   2nd
......................................................................uucguugucguugaaaccuU..................   6     1   2nd
......................................................................uucguuguUguugaaaccug..................   1     1   2nd
......................................................................uucguugucguugUaaccug..................   2     1   2nd
......................................................................uucguugucguugaaaccug..................   3310  0   2nd
......................................................................uucguugucguAgaaaccug..................   1     1   2nd
......................................................................uucguugucguugaaaccAg..................   3     1   2nd
......................................................................uucAuugucguugaaaccug..................   1     1   2nd
......................................................................uucguugucgCugaaaccug..................   1     1   2nd
......................................................................uucguugucguugaaaccuC..................   1     1   2nd
......................................................................uucguugucguugaaaccuA..................   22    1   2nd
......................................................................uucguugucguugaaacGug..................   1     1   2nd
......................................................................Gucguugucguugaaaccug..................   1     1   2nd
......................................................................uucguugucguugaaaccCg..................   26    1   2nd
......................................................................uucgGugucguugaaaccuga.................   1     1   2nd
......................................................................uucguugucguugaaUccuga.................   1     1   2nd
......................................................................uucguugucguugaaacUuga.................   6     1   2nd
......................................................................uucguugucguugaGaccuga.................   2     1   2nd
......................................................................uucguuUucguugaaaccuga.................   4     1   2nd
......................................................................uucguugucguugaaaccuUa.................   2     1   2nd
......................................................................uucguuCucguugaaaccuga.................   3     1   2nd
......................................................................uucguugucguugaaaccGga.................   5     1   2nd
```

Star Mature

agcgguaggaaaucgagagaagauauuguacaggcuucucgauuagcgaacaaauaucguuauuuaaauguucguugucguugaaaccugaacgaaacuccucauucc			
....uucguugucguugaUaccuga.................	2	1	2nd
....uucguuAucguugaaaccuga.................	5	1	2nd
....uucAuugucguugaaaccuga.................	1	1	2nd
....uucguugGcguugaaaccuga.................	1	1	2nd
....Gucguugucguugaaaccuga.................	3	1	2nd
....uucguugucCuugaaaccuga.................	1	1	2nd
....uucguugucgCugaaaccuga.................	3	1	2nd
....uucguugucguugGaaccuga.................	5	1	2nd
....uucguugucguugaCaccuga.................	3	1	2nd
....uucguugucguugaaaccAga.................	3	1	2nd
....uucguuguUguugaaaccuga.................	5	1	2nd
....uCcguugucguugaaaccuga.................	1	1	2nd
....uuAguugucguugaaaccuga.................	2	1	2nd
....uucguugucguugaaaccCga.................	7	1	2nd
....uucguugucguugaaaccuAa.................	5	1	2nd
....uucguugucguugaaacGuga.................	6	1	2nd
....uucguugucUuugaaaccuga.................	2	1	2nd
....uucguugucguugaaaccuga.................	11783	0	2nd
....uucguugucgAugaaaccuga.................	2	1	2nd
....uucgAugucguugaaaccuga.................	1	1	2nd
....uAcguugucguugaaaccuga.................	10	1	2nd
....uucguugAcguugaaaccuga.................	2	1	2nd
....uucguugucguuCaaaccuga.................	1	1	2nd
....uucCuugucguugaaaccuga.................	1	1	2nd
....uucguugucguugaaaAcuga.................	1	1	2nd
....Cucguugucguugaaaccuga.................	2	1	2nd
....uucguugucguugaaaUcuga.................	2	1	2nd
....Aucguugucguugaaaccuga.................	1	1	2nd
....uucguAgucguugaaaccuga.................	12	1	2nd
....uucgCugucguugaaaccuga.................	3	1	2nd
....uucguGgucguugaaaccuga.................	6	1	2nd
....uucguugucguugaaaccuCa.................	7	1	2nd
....uucguugucgGugaaaccuga.................	2	1	2nd
....uucguCgucguugaaaccuga.................	6	1	2nd
....uucguugucguugUaaccuga.................	6	1	2nd
....uucguugucguugaaaccugU.................	44	1	2nd
....uucguugucguugaaaccugC.................	4	1	2nd
....uucguugucguugaaGccuga.................	1	1	2nd
....uucguugucguugaaaccugG.................	9	1	2nd
....uuUguugucguugaaaccuga.................	2	1	2nd
....uucguugucguAgaaaccuga.................	1	1	2nd
....uucguugucguugaUaccugaa................	1	1	2nd
....uucguugucgCugaaaccugaa................	1	1	2nd
....Aucguugucguugaaaccugaa................	1	1	2nd
....uucgGugucguugaaaccugaa................	1	1	2nd
....uucguuAucguugaaaccugaa................	1	1	2nd
....uucguuCucguugaaaccugaa................	3	1	2nd
....uucguugucguugaaaccugCa................	1	1	2nd
....uuUguugucguugaaaccugaa................	1	1	2nd
....uucguugucguugaaaccugaG................	5	1	2nd
....uucguugucguugaaacGugaa................	2	1	2nd
....uucguugucguugaaaccugUa................	4	1	2nd
....uucguugAcguugaaaccugaa................	1	1	2nd
....uucguugucguugaaaccugaU................	77	1	2nd
....uucguugucguugaaaccugaC................	1006	1	2nd
....uucguugGcguugaaaccugaa................	1	1	2nd
....uucguugucguugaaaccCgaa................	3	1	2nd
....uucguugucguugGaaccugaa................	2	1	2nd
....uucguCgucguugaaaccugaa................	4	1	2nd
....uucguugucguugaaaccuUaa................	3	1	2nd
....uucguugucguugaaaccugaa................	4680	0	2nd
....uucguuUucguugaaaccugaa................	2	1	2nd
....uucguugucguugaaaccugGa................	2	1	2nd
....uucguuguUguugaaaccugaa................	1	1	2nd
....uucguugucguugaaCccugaa................	2	1	2nd
....uucguAgucguugaaaccugaa................	3	1	2nd
....uucguugucguugaaacUugaa................	3	1	2nd
....uucguugucguugaaaccuCaa................	1	1	2nd
....uucguGgucguugaaaccugaa................	4	1	2nd
....uAcguugucguugaaaccugaa................	5	1	2nd

```
                              Star                                  Mature
agcgguaggaaaucgagagaagauauuguacaggcuucucgauuagcgaacaaauaucguuauuuaaauguucguugucguugaaaccugaacgaaacuccucauucc
......................................................................uucguugucguuAaaaccugaa................	1	1	2nd
......................................................................uucgCugucguugaaaccugaa................	1	1	2nd
......................................................................uucguugucCuugaaaccugaa................	1	1	2nd
......................................................................uucguugucguugaaaUcugaa................	2	1	2nd
......................................................................uucguugucguugUaaccugaa................	1	1	2nd
......................................................................uucguugucguugaGaccugaa................	1	1	2nd
......................................................................uucguugucguugaCaccugaa................	2	1	2nd
......................................................................uucguugucguugaaaccugaCc...............	475	1	2nd
......................................................................uucguugucguugaaaccugaaU...............	280	1	2nd
......................................................................uucguugucguugaaaccugaaG...............	21	1	2nd
......................................................................uucguugucguugaUaccugaac...............	1	1	2nd
......................................................................uucguugucguugaaaccugaac...............	78	0	2nd
......................................................................uucguugucguugaaaccugaaA...............	538	1	2nd
......................................................................uucguugucguugCaaccugaac...............	1	1	2nd
......................................................................uucguugucguugaaaccugaacA..............	2	1	2nd
......................................................................uucguugucguugaaaccugaacU..............	1	1	2nd
......................................................................uucguugucguugaaaccugaacC..............	1	1	2nd
.......................................................................ucguugucguugaaaccug..................	7	0	2nd
.......................................................................ucguugucguugaaaccuga.................	32	0	2nd
.......................................................................ucguugucguugaaaccugU.................	1	1	2nd
.......................................................................ucguugucguugaaaccugaa................	8	0	2nd
.......................................................................ucguugucguugaaaccugaC................	5	1	2nd
.......................................................................ucguugucguugaaaccugaaA...............	2	1	2nd
.......................................................................ucguugucguugaaaccugaaU...............	2	1	2nd
........................................................................cguugucguugaaaccug..................	2	0	2nd
........................................................................cguugucguugaaaccuga.................	4	0	2nd
........................................................................cguugucguugaaaccugaa................	4	0	2nd
........................................................................cguugucguugaaaccugaaA...............	1	1	2nd
.........................................................................guugucguugaaaccuga.................	2	0	2nd
.........................................................................guugucguugaaaccugaa................	1	0	2nd
.........................................................................guugucguugaaaccugaCc...............	1	1	2nd

......................................................................uucguugucguugaaaccug..................	5	0	LA5
......................................................................uucguugucguugaaaccuA..................	2	1	LA5
......................................................................uucguugucguugaaaccuga.................	19	0	LA5
......................................................................uucguugucguugaaaccugaC................	4	1	LA5
......................................................................uucguugucguugaaaccugaa................	26	0	LA5
......................................................................uucguugucguugaaaccugaCc...............	2	1	LA5
......................................................................uucguugucguugaaaccugaaA...............	2	1	LA5

..............................caggcuucucgauuagcgaac.........................................................	3	0	E01
..............................caggcuucucgauuagcgaaca........................................................	3	0	E01
..................................................................aauguucguugucguugaaaccu...................	1	0	E01
...................................................................aGguucguugucguugaaaccuga.................	1	1	E01
......................................................................uucguugucguugaaacc....................	7	0	E01
......................................................................uucguugucguugGaaccu...................	1	1	E01
......................................................................uucguugucguugaaaccu...................	35	0	E01
......................................................................uucguugucguugUaaccu...................	2	1	E01
......................................................................uucguugAcguugaaaccug..................	1	1	E01
......................................................................uucguugucguugaaaccug..................	531	0	E01
......................................................................uucguugucguugaaaccuA..................	1	1	E01
......................................................................uucguugucguugaaacUug..................	1	1	E01
......................................................................uucguugucguugaaaccCg..................	1	1	E01
......................................................................uucguugucguugaGaccuga.................	1	1	E01
......................................................................uucguugucguugaUaccuga.................	1	1	E01
......................................................................uucguugucguugaaaccugG.................	1	1	E01
......................................................................uucguCgucguugaaaccuga.................	1	1	E01
......................................................................uucguugucguugaaacUuga.................	3	1	E01
......................................................................uucguugucCuugaaaccuga.................	1	1	E01
......................................................................uucguugucguugUaaccuga.................	2	1	E01
......................................................................uucguugucguugaaaccuga.................	1223	0	E01
......................................................................uucguGgucguugaaaccuga.................	1	1	E01
......................................................................uucguugucguugaaaccugU.................	2	1	E01
......................................................................uucguugucguugCaaccuga.................	1	1	E01
......................................................................uucguugucguugGaaccugaa................	1	1	E01
......................................................................uucguugucguugaaaccugaG................	1	1	E01
......................................................................uucguugucguugaaaccugGa................	1	1	E01
......................................................................uucguugucguugCaaccugaa................	1	1	E01
......................................................................uucguugucguugaaaccugaC................	217	1	E01
......................................................................uuUguugucguugaaaccugaa................	1	1	E01
```

```
                              Star                         Mature
agcgguaggaaaucgagagaagauauuguacaggcuucucgauuagcgaacaaauaucguuauuuaaauguucguugucguugaaaccugaacgaaacuccucauucc
......................................................................uucguugucguugaaaccCgaa................	1	1	E01
......................................................................uucguugucguugaaaccugaU................	16	1	E01
......................................................................uucguugucguugUaaccugaa................	1	1	E01
......................................................................uucguugucguugaaaccugaa................	670	0	E01
......................................................................uucguugucguugaaaccugaac...............	27	0	E01
......................................................................uucguugucguugaaaccugaaA...............	102	1	E01
......................................................................uucguugucguugaaaccugaaU...............	32	1	E01
......................................................................uucguugucguugaaaccugaaG...............	6	1	E01
......................................................................uucguugucguugaaaccugaCc...............	79	1	E01
......................................................................uucguugucguugaaaccugaacU..............	3	1	E01
.......................................................................ucguugucguugaaaccug..................	1	0	E01
.......................................................................ucguugucguugaaaccuga.................	3	0	E01
.........................................................................guugucguugaaaccuga.................	1	0	E01
.........................................................................guugucguugaaaccugaa................	1	0	E01

.....................................................................Cuucguugucguugaaaccug..................	2	1	TA1
.....................................................................Cuucguugucguugaaaccugaa................	2	1	TA1
......................................................................uucguugucguugaaacc....................	12	0	TA1
......................................................................uucguugucguugaaacU....................	5	1	TA1
......................................................................uucguugucguugaaacA....................	1	1	TA1
......................................................................uucguugucguugaaaccu...................	14	0	TA1
......................................................................uucguugucguugaaaccA...................	1	1	TA1
......................................................................uucguugucguugaaaccG...................	1	1	TA1
......................................................................uucguugucguugaaaccC...................	1	1	TA1
......................................................................uuUguugucguugaaaccug..................	1	1	TA1
......................................................................uucguuguUguugaaaccug..................	1	1	TA1
......................................................................uucguugucguugaaaccug..................	252	0	TA1
......................................................................uucguugucguugGaaccug..................	1	1	TA1
......................................................................uCcguugucguugaaaccug..................	1	1	TA1
......................................................................uucguugucgCugaaaccug..................	2	1	TA1
......................................................................uucguugucguugaaaccuU..................	3	1	TA1
......................................................................uucguugucguugaaaccCg..................	1	1	TA1
......................................................................uucguugucguugaGaccug..................	1	1	TA1
......................................................................uucguugucguugaaaccuA..................	18	1	TA1
......................................................................uucguugucguugaGaccuga.................	7	1	TA1
......................................................................uucguugucguugaaacUuga.................	1	1	TA1
......................................................................uucguugucguugaaaccCga.................	4	1	TA1
......................................................................uucguugucguugaaGccuga.................	9	1	TA1
......................................................................uucguugucguugaaaccuCa.................	1	1	TA1
......................................................................uucguugCcguugaaaccuga.................	2	1	TA1
......................................................................uucgCugucguugaaaccuga.................	5	1	TA1
......................................................................uucguugucguugaaaccugU.................	8	1	TA1
......................................................................uuUguugucguugaaaccuga.................	2	1	TA1
......................................................................uucguugucguugaaUccuga.................	1	1	TA1
......................................................................uucguuCucguugaaaccuga.................	2	1	TA1
......................................................................uucguAgucguugaaaccuga.................	3	1	TA1
......................................................................uucguuAucguugaaaccuga.................	1	1	TA1
......................................................................uucguugucguugaaaccugG.................	23	1	TA1
......................................................................uucguCgucguugaaaccuga.................	8	1	TA1
......................................................................uuAguugucguugaaaccuga.................	1	1	TA1
......................................................................uucguugucguugaaaccuga.................	1431	0	TA1
......................................................................uucguugucAuugaaaccuga.................	5	1	TA1
......................................................................uCcguugucguugaaaccuga.................	5	1	TA1
......................................................................uucguuguGguugaaaccuga.................	1	1	TA1
......................................................................uucguugucgGugaaaccuga.................	1	1	TA1
......................................................................Cucguugucguugaaaccuga.................	5	1	TA1
......................................................................uucguugucguugaaaccugC.................	1	1	TA1
......................................................................Aucguugucguugaaaccuga.................	2	1	TA1
......................................................................uucguugucguugaaaccuUa.................	1	1	TA1
......................................................................uucUuugucguugaaaccuga.................	1	1	TA1
......................................................................uucguugucgCugaaaccuga.................	2	1	TA1
......................................................................uucguugucguugGaaccuga.................	3	1	TA1
......................................................................uucguugucguugaaaUcuga.................	3	1	TA1
......................................................................uucguugucguugaUaccuga.................	1	1	TA1
......................................................................uAcguugucguugaaaccuga.................	1	1	TA1
......................................................................uucgAugucguugaaaccuga.................	6	1	TA1
......................................................................uucguugCcguugaaaccugaa................	2	1	TA1
......................................................................uucguugucguugaaaccugaC................	342	1	TA1
......................................................................uucguugucguugaaaccugaa................	1741	0	TA1
......................................................................uucguugucgAugaaaccugaa................	2	1	TA1
```

```
                              Star                                        Mature
agcgguaggaaaucgagagaagauauuguacaggcuucucgauuagcgaacaaauaucguuauuuaaauguucguugucguugaaaccugaacgaaacuccucauucc
......................................................................Aucguugucguugaaaccugaa................    2     1     TA1
......................................................................uAcguugucguugaaaccugaa................    4     1     TA1
......................................................................uucguugucgCugaaaccugaa................    5     1     TA1
......................................................................uucguugucguugaaaccugGa................    4     1     TA1
......................................................................uucguugucguugaaaccugUa................    6     1     TA1
......................................................................uucgCugucguugaaaccugaa................    6     1     TA1
......................................................................uucguugucguugaUaccugaa................    1     1     TA1
......................................................................uucguugucguugUaaccugaa................    3     1     TA1
......................................................................uucguuguUguugaaaccugaa................    1     1     TA1
......................................................................uucguugucguugaaaccugaU................    45    1     TA1
......................................................................uucguugucAuugaaaccugaa................    5     1     TA1
......................................................................uucguCgucguugaaaccugaa................    11    1     TA1
......................................................................uucAuugucguugaaaccugaa................    1     1     TA1
......................................................................uuAguugucguugaaaccugaa................    2     1     TA1
......................................................................uuUguugucguugaaaccugaa................    4     1     TA1
......................................................................Cucguugucguugaaaccugaa................    5     1     TA1
......................................................................uucguugucguugaGaccugaa................    5     1     TA1
......................................................................uucguugucguugaaaccAgaa................    2     1     TA1
......................................................................uCcguugucguugaaaccugaa................    4     1     TA1
......................................................................uucguugucguugaaaccugaG................    25    1     TA1
......................................................................uucguugucguugaaaccCgaa................    4     1     TA1
......................................................................uucguugucguugGaaccugaa................    6     1     TA1
......................................................................uucguugucguugaaUccugaa................    3     1     TA1
......................................................................uucguugucguugaaGccugaa................    6     1     TA1
......................................................................uucguugucguuAaaaccugaa................    1     1     TA1
......................................................................uucguugucguugaaaccugaaU...............    41    1     TA1
......................................................................uucguugucguugaaGccugaac...............    1     1     TA1
......................................................................uucguugucguugaaaccugaaA...............    64    1     TA1
......................................................................uucguugucguugaaaccugaaG...............    2     1     TA1
......................................................................uucguugucguugaaaccugaac...............    9     0     TA1
......................................................................uucguugucguugaaaccugaCc...............    104   1     TA1
......................................................................uucguugucguugaaaccugaacU..............    1     1     TA1
......................................................................uucguugucguugaaaccugaacA..............    2     1     TA1
......................................................................uucguugucguugaaaccugaacg..............    1     0     TA1
.......................................................................ucguugucguugaaaccugaa................    2     0     TA1
.......................................................................ucguugucguugaaaccugaCc...............    1     1     TA1
........................................................................cguugucguugaaaccugaa................    1     0     TA1

..............................caggcuucucgauuagcga...........................................................    1     0     LJ1
.....................................................................guucguugucguugaaaccuga.................    2     0     LJ1
......................................................................uucguugucguugaaacA....................    7     1     LJ1
......................................................................uucguugucguugaaacU....................    4     1     LJ1
......................................................................uucguugucguugaaCcc....................    1     1     LJ1
......................................................................uucguugucguugaaacc....................    18    0     LJ1
......................................................................uucguugucguugaaGcc....................    1     1     LJ1
......................................................................uucguugucguugaaaccA...................    9     1     LJ1
......................................................................uucguugucguugaaaccG...................    1     1     LJ1
......................................................................uucguugucguugaaaccu...................    52    0     LJ1
......................................................................uucguugucguugaaaccC...................    13    1     LJ1
......................................................................uucguugucguGgaaaccu...................    1     1     LJ1
......................................................................uucguugucguugaGaccu...................    1     1     LJ1
......................................................................uuAguugucguugaaaccu...................    1     1     LJ1
......................................................................uucguugucAuugaaaccug..................    1     1     LJ1
......................................................................uucguugucguugaGaccug..................    1     1     LJ1
......................................................................Cucguugucguugaaaccug..................    1     1     LJ1
......................................................................uucguugucguugaaaccug..................    268   0     LJ1
......................................................................uucguCgucguugaaaccug..................    2     1     LJ1
......................................................................uucguugucguugaaCccug..................    1     1     LJ1
......................................................................uuUguugucguugaaaccug..................    1     1     LJ1
......................................................................uucguugucguugaaaccCg..................    4     1     LJ1
......................................................................uucgCugucguugaaaccug..................    1     1     LJ1
......................................................................uucAuugucguugaaaccug..................    1     1     LJ1
......................................................................uucguuAucguugaaaccug..................    1     1     LJ1
......................................................................uucguugucguugaaaccuA..................    35    1     LJ1
......................................................................uucguAgucguugaaaccug..................    2     1     LJ1
......................................................................uucguugucguugaaGccug..................    1     1     LJ1
......................................................................uucguugCcguugaaaccug..................    1     1     LJ1
......................................................................uucguugucguugaaaccuU..................    12    1     LJ1
......................................................................uucguugucguugaaaUcuga.................    3     1     LJ1
......................................................................Cucguugucguugaaaccuga.................    4     1     LJ1
```

```
                              Star                                    Mature
agcgguaggaaaucgagagaagauauuguacaggcuucucgauuagcgaacaaauaucguuauuuaaauguucguugucguugaaaccugaacgaaacuccucauucc
......................................................................uucguuAucguugaaaccuga.................   5      1     LJ1
......................................................................uucguugucAuugaaaccuga.................   2      1     LJ1
......................................................................uucguugucguugaaaccugG.................   19     1     LJ1
......................................................................uucguugucguuUaaaccuga.................   2      1     LJ1
......................................................................uucguugucguugaaGccuga.................   6      1     LJ1
......................................................................uucguCgucguugaaaccuga.................   4      1     LJ1
......................................................................uuUguugucguugaaaccuga.................   2      1     LJ1
......................................................................uuAguugucguugaaaccuga.................   5      1     LJ1
......................................................................uucguuCucguugaaaccuga.................   2      1     LJ1
......................................................................uucguugucguugaaaccugU.................   24     1     LJ1
......................................................................uuGguugucguugaaaccuga.................   1      1     LJ1
......................................................................uCcguugucguugaaaccuga.................   5      1     LJ1
......................................................................uucguugCcguugaaaccuga.................   2      1     LJ1
......................................................................uucguugucguugaaacUuga.................   1      1     LJ1
......................................................................uucgGugucguugaaaccuga.................   2      1     LJ1
......................................................................uucguugucguugaaaccGga.................   2      1     LJ1
......................................................................uucguAgucguugaaaccuga.................   2      1     LJ1
......................................................................uucguugucguugaUaccuga.................   1      1     LJ1
......................................................................uucgCugucguugaaaccuga.................   4      1     LJ1
......................................................................uucguuUucguugaaaccuga.................   2      1     LJ1
......................................................................uucguugucguugaGaccuga.................   8      1     LJ1
......................................................................uucguugucguugaaaccuga.................   1315   0     LJ1
......................................................................uucguuguUguugaaaccuga.................   1      1     LJ1
......................................................................uucUuugucguugaaaccuga.................   2      1     LJ1
......................................................................uucguugucguugaaaccCga.................   2      1     LJ1
......................................................................uucgAugucguugaaaccuga.................   1      1     LJ1
......................................................................uucguugucgCugaaaccuga.................   1      1     LJ1
......................................................................uucguugucguuGaaccuga.................   11     1     LJ1
......................................................................Aucguugucguugaaaccuga.................   1      1     LJ1
......................................................................uucguugucguugaaaccugC.................   1      1     LJ1
......................................................................uucguugucguugaaaccugUa................   3      1     LJ1
......................................................................uucgCugucguugaaaccugaa................   1      1     LJ1
......................................................................uucguuAucguugaaaccugaa................   1      1     LJ1
......................................................................uucguugucguugaaaccugaC................   86     1     LJ1
......................................................................uucguCgucguugaaaccugaa................   3      1     LJ1
......................................................................uucguugucguuGaaccugaa................   1      1     LJ1
......................................................................uucguugucgCugaaaccugaa................   1      1     LJ1
......................................................................uucguugucguugaaaccugGa................   1      1     LJ1
......................................................................uucguugucguugaGaccugaa................   1      1     LJ1
......................................................................uucguugucguugaaaccugaa................   258    0     LJ1
......................................................................uucguugucguugaaGccugaa................   3      1     LJ1
......................................................................uucguugucguugaaaccugaU................   6      1     LJ1
......................................................................uucguugucguuGUaaccugaa................   1      1     LJ1
......................................................................uuUguugucguugaaaccugaa................   2      1     LJ1
......................................................................uucguugucguugaaaccugaG................   2      1     LJ1
......................................................................uucguAgucguugaaaccugaa................   2      1     LJ1
......................................................................uucguugucguugaaaccugaac...............   5      0     LJ1
......................................................................uucguugucguugaaaccugaaU...............   16     1     LJ1
......................................................................uucguugucguugaaaccugaCc...............   57     1     LJ1
......................................................................uucguugucguugaaaccugaaA...............   24     1     LJ1
......................................................................uucguugucguugaaaccugaaG...............   2      1     LJ1
......................................................................uucguugucguugaaaccugaUc...............   1      1     LJ1
......................................................................uCcguugucguugaaaccugaac...............   1      1     LJ1
.......................................................................ucguugucguugaaaccuga.................   1      0     LJ1
........................................................................cguugucguugaaaccuga.................   1      0     LJ1

......................................................................uucguugucguugaaaccug..................   7      0     BJ1
......................................................................uucguugucguugaaaccugG.................   1      1     BJ1
......................................................................uucguugucguugaaaccuga.................   22     0     BJ1
......................................................................uucguugucguugaaaccugaC................   5      1     BJ1
......................................................................uucguugucguugaaaccugaa................   3      0     BJ1
......................................................................uucguugucguugaaaccugGa................   1      1     BJ1
......................................................................uucguugucguugaaaccugaaU...............   1      1     BJ1

......................................................................uucguugucguugaaacc....................   2      0     HA1
......................................................................uucguugucguugaaaccu...................   1      0     HA1
......................................................................uucguugucguugaaaccA...................   6      1     HA1
......................................................................uucguugucguugaGaccug..................   1      1     HA1
......................................................................uucguugucguugaaaccug..................   44     0     HA1
......................................................................uucguugucguugaaaccuA..................   6      1     HA1
```

Star Mature

```
agcgguaggaaaucgagagaagauauuguacaggcuucucgauuagcgaacaaauaucguuauuuaaauguucguugucguugaaaccugaacgaaacuccucauucc
......................................................................Cucguugucguugaaaccug..................    1      1    HA1
......................................................................uucguugucguugaaaccugG.................    6      1    HA1
......................................................................uucguugucguugaaaccugU.................    1      1    HA1
......................................................................uucguugucguugaaaccuga.................    152    0    HA1
......................................................................uucguugucAuugaaaccuga.................    1      1    HA1
......................................................................uucguCgucguugaaaccuga.................    1      1    HA1
......................................................................uucAuugucguugaaaccuga.................    2      1    HA1
......................................................................uCcguugucguugaaaccuga.................    1      1    HA1
......................................................................uucgAugucguugaaaccuga.................    1      1    HA1
......................................................................uucguugucguugaaaccGga.................    1      1    HA1
......................................................................uucguugucguugaaacUuga.................    2      1    HA1
......................................................................uucguugucguugaaaccugaG................    1      1    HA1
......................................................................uucguugucgCugaaaccugaa................    1      1    HA1
......................................................................uucguugucguugaaaccugaC................    5      1    HA1
......................................................................uucguugucguugaaaccugGa................    1      1    HA1
......................................................................uucguugucguugGaaccugaa................    1      1    HA1
......................................................................uCcguugucguugaaaccugaa................    1      1    HA1
......................................................................uucguugucguugaaaccugaa................    75     0    HA1
......................................................................uucguugucguugaaaccugGac...............    1      1    HA1
......................................................................uucguugucguugaaaccugaCc...............    3      1    HA1
......................................................................uucguugucguugaaaccugaac...............    1      0    HA1
......................................................................uucguugucguugaaaccugaaU...............    4      1    HA1
......................................................................uucgAugucguugaaaccugaac...............    1      1    HA1
......................................................................uucguugucguCgaaaccugaacg..............    1      1    HA1

....................................................................uUuucguugucguugaaaccuga.................    1      1    EGG
.....................................................................guucguugucguugaaaccugaa................    4      0    EGG
.....................................................................Uuucguugucguugaaaccugaa................    1      1    EGG
......................................................................uucguugucguugaaacc....................    6      0    EGG
......................................................................uucguugucguugaaaccu...................    31     0    EGG
......................................................................uucguugucguugaaaccug..................    267    0    EGG
......................................................................uucguugucguGgaaaccug..................    1      1    EGG
......................................................................uucguugucAuugaaaccug..................    1      1    EGG
......................................................................uucguugucguugaaaccCg..................    2      1    EGG
......................................................................uucguugucguugaaaccugU.................    2      1    EGG
......................................................................uGcguugucguugaaaccuga.................    1      1    EGG
......................................................................uucguugucguugaaaccugG.................    1      1    EGG
......................................................................uucguGgucguugaaaccuga.................    1      1    EGG
......................................................................uucguugucguugaGaccuga.................    1      1    EGG
......................................................................uuUguugucguugaaaccuga.................    1      1    EGG
......................................................................uucguugucguugaaaUcuga.................    1      1    EGG
......................................................................uucguugucguugCaaccuga.................    1      1    EGG
......................................................................uucguugucguugaaaccuga.................    1627   0    EGG
......................................................................uucguugCcguugaaaccuga.................    1      1    EGG
......................................................................uucguugucUuugaaaccuga.................    1      1    EGG
......................................................................uucguugucguugaaaccuAa.................    4      1    EGG
......................................................................uucguugucguugaaacUuga.................    1      1    EGG
......................................................................uucguugucguugaCaccuga.................    1      1    EGG
......................................................................uucguugucguugaaaccugaa................    1370   0    EGG
......................................................................uucguugucguugaaaccugaG................    1      1    EGG
......................................................................uucguugucguugUaaccugaa................    3      1    EGG
......................................................................uucguugucguugaaaccugaU................    14     1    EGG
......................................................................uucguugucguugaaaccuAaa................    1      1    EGG
......................................................................uucguugucguugaaaccugGa................    4      1    EGG
......................................................................uucguugucguugaGaccugaa................    2      1    EGG
......................................................................uucguugucguuCaaaccugaa................    1      1    EGG
......................................................................uucguugucUuugaaaccugaa................    1      1    EGG
......................................................................uucguCgucguugaaaccugaa................    1      1    EGG
......................................................................uucguugucguugaaaccugaC................    285    1    EGG
......................................................................uucguugucguugaCaccugaa................    1      1    EGG
......................................................................uucguugucguugaaacUugaa................    1      1    EGG
......................................................................uucguugucguugaaacAugaa................    1      1    EGG
......................................................................uucguugucguugaaaccugaaA...............    189    1    EGG
......................................................................uucguugucguugaaaccugaaG...............    9      1    EGG
......................................................................uucguugucguugaaaccugaaU...............    56     1    EGG
......................................................................uucguugucguugaaaccugaCc...............    106    1    EGG
......................................................................uucguugucguugaaaccugaGc...............    1      1    EGG
......................................................................uucguugucguugCaaccugaac...............    1      1    EGG
......................................................................uucguugucguugaaaccugaac...............    29     0    EGG
......................................................................uucguugucguugaaaccugaacU..............    1      1    EGG
```

```
                              Star                                      Mature
agcgguaggaaaucgagagaagauauuguacaggcuucucgauuagcgaacaaauaucguuauuuaaauguucguugucguugaaaccugaacgaaacuccucauucc
.......................................................................ucguugucguugaaaccug..................  1     0  EGG
.......................................................................ucguugucguugaaaccuga.................  4     0  EGG
.......................................................................ucguugucguugaaaccugaa................  5     0  EGG
.......................................................................ucguugucguugaaaccugaaG...............  1     1  EGG
.......................................................................ucguugucguugaaaccugaCc...............  1     1  EGG
.......................................................................ucguugucguugaaaccugaac...............  1     0  EGG
........................................................................cguugucguugaaaccugaa................  1     0  EGG
........................................................................cguugucguugaaaccugaCc...............  1     1  EGG

..............................caggcuucucgauuagcgaac.........................................................  1     0  TJ1
...............................aggcuucucgauuagcgaac.........................................................  1     0  TJ1
.....................................................................Cuucguugucguugaaaccug..................  1     1  TJ1
.....................................................................Cuucguugucguugaaaccuga.................  3     1  TJ1
.....................................................................guucguugucguugaaaccuga.................  1     0  TJ1
.....................................................................Cuucguugucguugaaaccugaa................  2     1  TJ1
......................................................................uucguugucguugaaacU....................  18    1  TJ1
......................................................................uucguuAucguugaaacc....................  1     1  TJ1
......................................................................uucguugucguugaaacc....................  90    0  TJ1
......................................................................uuAguugucguugaaacc....................  1     1  TJ1
......................................................................uucguugucAuugaaacc....................  1     1  TJ1
......................................................................uuUguugucguugaaacc....................  1     1  TJ1
......................................................................uucguugucguugaaCcc....................  1     1  TJ1
......................................................................Aucguugucguugaaacc....................  1     1  TJ1
......................................................................uucguugucguugaaacA....................  8     1  TJ1
......................................................................uucguugucguugaaaccA...................  18    1  TJ1
......................................................................uucguugucguugaaaccG...................  2     1  TJ1
......................................................................uucguuAucguugaaaccu...................  2     1  TJ1
......................................................................uucguCgucguugaaaccu...................  2     1  TJ1
......................................................................uucguugucguugaGaccu...................  1     1  TJ1
......................................................................uucgCugucguugaaaccu...................  1     1  TJ1
......................................................................uucguugucguugGaaccu...................  1     1  TJ1
......................................................................uucguugucguugaaaccC...................  19    1  TJ1
......................................................................uucguugucguugaaaUcu...................  1     1  TJ1
......................................................................uucguugucguugaaaccu...................  155   0  TJ1
......................................................................uucguugucguugaaCccu...................  1     1  TJ1
......................................................................uucguCgucguugaaaccug..................  1     1  TJ1
......................................................................uucguugucgCugaaaccug..................  4     1  TJ1
......................................................................uucguugucguugaaaccug..................  1120  0  TJ1
......................................................................uCcguugucguugaaaccug..................  5     1  TJ1
......................................................................uucguugucguugaaaccGg..................  6     1  TJ1
......................................................................uuGguugucguugaaaccug..................  1     1  TJ1
......................................................................uucguugucguuUaaaccug..................  1     1  TJ1
......................................................................uucguugCcguugaaaccug..................  3     1  TJ1
......................................................................uucguugucguugaaaccAg..................  2     1  TJ1
......................................................................uucguugucguugGaaccug..................  8     1  TJ1
......................................................................uucguugucguugaGaccug..................  4     1  TJ1
......................................................................uucguugucguugaaUccug..................  1     1  TJ1
......................................................................Cucguugucguugaaaccug..................  3     1  TJ1
......................................................................uucguugucguugaaacUug..................  3     1  TJ1
......................................................................uucguuCucguugaaaccug..................  1     1  TJ1
......................................................................uuUguugucguugaaaccug..................  2     1  TJ1
......................................................................uucguuAucguugaaaccug..................  5     1  TJ1
......................................................................uucguugucguugaaaccuA..................  119   1  TJ1
......................................................................uucguugucguuAaaaccug..................  1     1  TJ1
......................................................................uucgCugucguugaaaccug..................  4     1  TJ1
......................................................................uucUuugucguugaaaccug..................  2     1  TJ1
......................................................................uucguAgucguugaaaccug..................  3     1  TJ1
......................................................................uucAuugucguugaaaccug..................  4     1  TJ1
......................................................................uucguGgucguugaaaccug..................  1     1  TJ1
......................................................................uuAguugucguugaaaccug..................  3     1  TJ1
......................................................................uucguugucguugaaaccuU..................  21    1  TJ1
......................................................................uucguugucguugaaaccuC..................  5     1  TJ1
......................................................................uucguugucguugaaaccCg..................  6     1  TJ1
......................................................................uucguugucguugaaGccug..................  4     1  TJ1
......................................................................uucguugucguugaaacAug..................  2     1  TJ1
......................................................................Aucguugucguugaaaccug..................  1     1  TJ1
......................................................................uucguugucguugaaaccugU.................  106   1  TJ1
......................................................................uucUuugucguugaaaccuga.................  2     1  TJ1
......................................................................uucgCugucguugaaaccuga.................  20    1  TJ1
......................................................................uucguugAcguugaaaccuga.................  1     1  TJ1
```

Star Mature

agcgguaggaaaucgagagaagauauuguacaggcuucucgauuagcgaacaaauaucguuauuuaaauguucguugucguugaaaccugaacgaaacuccucauucc			
....uucguugucguugaaaccuUa.................	6	1	TJ1
....uCcguugucguugaaaccuga.................	14	1	TJ1
....uAcguugucguugaaaccuga.................	1	1	TJ1
....uucguugucguugaaaccCga.................	11	1	TJ1
....uucguugucguuAaaaccuga.................	3	1	TJ1
....uucguugucguugUaaccuga.................	3	1	TJ1
....Cucguugucguugaaaccuga.................	19	1	TJ1
....uucguugucguuCaaaccuga.................	1	1	TJ1
....uucguGgucguugaaaccuga.................	1	1	TJ1
....Gucguugucguugaaaccuga.................	1	1	TJ1
....uucguuguUguugaaaccuga.................	5	1	TJ1
....uucguuAucguugaaaccuga.................	21	1	TJ1
....uucguugucguugCaaccuga.................	1	1	TJ1
....uucguuCucguugaaaccuga.................	2	1	TJ1
....uucgAugucguugaaaccuga.................	3	1	TJ1
....uucguugucguugaaacAuga.................	1	1	TJ1
....uucguugucguugaaaccuCa.................	2	1	TJ1
....uucguCgucguugaaaccuga.................	36	1	TJ1
....uucguugucguugaaaUcuga.................	5	1	TJ1
....uucguuguAguugaaaccuga.................	2	1	TJ1
....uucguugucguugaCaccuga.................	3	1	TJ1
....uucCuugucguugaaaccuga.................	1	1	TJ1
....uucguugucguugaaGccuga.................	27	1	TJ1
....uuUguugucguugaaaccuga.................	9	1	TJ1
....uucguugucgCugaaaccuga.................	18	1	TJ1
....uucguugucguugaaUccuga.................	2	1	TJ1
....uuGguugucguugaaaccuga.................	3	1	TJ1
....uucguugucguugaaaccAga.................	14	1	TJ1
....uucguugucguugaaaccugG.................	119	1	TJ1
....uucAuugucguugaaaccuga.................	9	1	TJ1
....uuAguugucguugaaaccuga.................	20	1	TJ1
....uucguugucguugaaaccuAa.................	5	1	TJ1
....uucguugucguugaGaccuga.................	28	1	TJ1
....uucguugCcguugaaaccuga.................	13	1	TJ1
....uucguugucguugaaacUuga.................	12	1	TJ1
....Aucguugucguugaaaccuga.................	3	1	TJ1
....uucguugucAuugaaaccuga.................	20	1	TJ1
....uucguugucguugaaCccuga.................	2	1	TJ1
....uucgGugucguugaaaccuga.................	1	1	TJ1
....uucguugucguugGaaccuga.................	19	1	TJ1
....uucguugucguugaaaccugC.................	12	1	TJ1
....uucguugucguAgaaaccuga.................	1	1	TJ1
....uucguugucguugaaaGcuga.................	1	1	TJ1
....uucguAgucguugaaaccuga.................	16	1	TJ1
....uucguugucguugaaaccGga.................	16	1	TJ1
....uucguugucUuugaaaccuga.................	4	1	TJ1
....uucguugucguugaaaccuga.................	6530	0	TJ1
....uucUuugucguugaaaccugaa................	2	1	TJ1
....uucguugucguugaaaccuUaa................	1	1	TJ1
....uAcguugucguugaaaccugaa................	1	1	TJ1
....uCcguugucguugaaaccugaa................	4	1	TJ1
....uucguugucguugaaaccugCa................	2	1	TJ1
....uucguuUucguugaaaccugaa................	1	1	TJ1
....uuUguugucguugaaaccugaa................	3	1	TJ1
....uucguugucguugaaaccugUa................	7	1	TJ1
....uucAuugucguugaaaccugaa................	4	1	TJ1
....uucguugucguugaaaccugGa................	8	1	TJ1
....Gucguugucguugaaaccugaa................	1	1	TJ1
....uuAguugucguugaaaccugaa................	5	1	TJ1
....uucguugucguugaaaccAgaa................	2	1	TJ1
....uucguugucguugaaGccugaa................	5	1	TJ1
....uucguugucguugaaCccugaa................	1	1	TJ1
....uucguugucguugaaaUcugaa................	1	1	TJ1
....uucguugCcguugaaaccugaa................	4	1	TJ1
....Cucguugucguugaaaccugaa................	5	1	TJ1
....uucguugucguugaaaccugaG................	24	1	TJ1
....uucguugucguugGaaccugaa................	6	1	TJ1
....uucguugucguugaaacUugaa................	2	1	TJ1
....uucguugucguugaaaccCgaa................	7	1	TJ1
....uucguugucguugaaaccugaa................	1245	0	TJ1

Star **Mature**

```
agcgguaggaaaucgagagaagauauuguacaggcuucucgauuagcgaacaaauaucguuauuuaaauguucguugucguugaaaccugaacgaaacuccucauucc
......................................................................uucgCugucguugaaaccugaa................  1      1  TJ1
......................................................................uucguugucguugaaaccugaC................  344    1  TJ1
......................................................................uucguGgucguugaaaccugaa................  2      1  TJ1
......................................................................uucguuCucguugaaaccugaa................  3      1  TJ1
......................................................................uucguugucguugaaaccugaU................  54     1  TJ1
......................................................................uucguugucgCugaaaccugaa................  5      1  TJ1
......................................................................uucguAgucguugaaaccugaa................  1      1  TJ1
......................................................................uucguugucguuAaaaccugaa................  1      1  TJ1
......................................................................uucguugucguugaGaccugaa................  2      1  TJ1
......................................................................uucguugucguugUaaccugaa................  1      1  TJ1
......................................................................uucguuAucguugaaaccugaa................  3      1  TJ1
......................................................................uucguCgucguugaaaccugaa................  13     1  TJ1
......................................................................uuGguugucguugaaaccugaa................  1      1  TJ1
......................................................................uucguugucAuugaaaccugaa................  4      1  TJ1
......................................................................uucguuguUguugaaaccugaa................  2      1  TJ1
......................................................................uucguugucguugaaaccugaaA...............  79     1  TJ1
......................................................................uucguuAucguugaaaccugaac...............  1      1  TJ1
......................................................................uucguugucguugaaaccugaac...............  32     0  TJ1
......................................................................uucguugucguugaaaccugaaU...............  83     1  TJ1
......................................................................uucguugucguugaaaccugaCc...............  259    1  TJ1
......................................................................uucguugucguugaaaccugaaG...............  7      1  TJ1
......................................................................uucguugucguugaaaccugaUc...............  3      1  TJ1
......................................................................uucguugucguugaaaccugaacC..............  1      1  TJ1
......................................................................uucguugucguugaaaccugaacA..............  3      1  TJ1
......................................................................uucguugucguugaaaccugaacg..............  2      0  TJ1
......................................................................uucguugucguugaaaccugaaGga.............  1      1  TJ1
.......................................................................ucguugucguugaaaccug..................  1      0  TJ1
.......................................................................ucguugucgAugaaaccug..................  1      1  TJ1
.......................................................................ucguugucguugaaaccuA..................  1      1  TJ1
.......................................................................ucguugucguugaaaccuga.................  6      0  TJ1
.......................................................................ucguugucguugaaaccugaCc...............  2      1  TJ1
........................................................................cguugucguugaaaccuA..................  1      1  TJ1
........................................................................cguugucguugaaaccug..................  1      0  TJ1
.........................................................................guugucguugaaaccuga.................  2      0  TJ1
..........................................................................uugucguugaaaccugaa................  1      0  TJ1

............................uacaggcuucucgauuagcga...........................................................  1      0  1st
..............................caggcuucucgauuagcgaac.........................................................  2      0  1st
....................................................................uCuucguugucguugaaaccuga.................  1      1  1st
....................................................................uUuucguugucguugaaaccuga.................  2      1  1st
....................................................................uUuucguugucguugaaaccugaa................  2      1  1st
.....................................................................guucguugucguugaaaccu...................  1      0  1st
.....................................................................Uuucguugucguugaaaccu...................  2      1  1st
.....................................................................guucguugucguugaaaccug..................  8      0  1st
.....................................................................Uuucguugucguugaaaccuga.................  3      1  1st
.....................................................................guucguugucguugaaaccuga.................  4      0  1st
.....................................................................Uuucguugucguugaaaccugaa................  5      1  1st
......................................................................uucguugucguugaaacc....................  54     0  1st
......................................................................uucguAgucguugaaaccu...................  1      1  1st
......................................................................uucguuguUguugaaaccu...................  1      1  1st
......................................................................uucguugucguugaaaccu...................  1227   0  1st
......................................................................uucguugucguugaaaccA...................  7      1  1st
......................................................................uucguugucguugaaaccG...................  2      1  1st
......................................................................uucguugucguugaUaccu...................  1      1  1st
......................................................................uucguugucguugaaaUcu...................  1      1  1st
......................................................................uucguugucguugaaaccC...................  5      1  1st
......................................................................uucguuUucguugaaaccu...................  1      1  1st
......................................................................uucguuCucguugaaaccug..................  1      1  1st
......................................................................uucguuguUguugaaaccug..................  1      1  1st
......................................................................uucguugucguugaaaccuU..................  17     1  1st
......................................................................uucguugucguugaaaccGg..................  13     1  1st
......................................................................uucguugucguugaCaccug..................  3      1  1st
......................................................................uucguugucguugaaaccCg..................  17     1  1st
......................................................................Cucguugucguugaaaccug..................  1      1  1st
......................................................................uucguugucguugUaaccug..................  3      1  1st
......................................................................uucguugucguugaaaccug..................  4573   0  1st
......................................................................uucguugucguugaaaUcug..................  1      1  1st
......................................................................uucguugucguugaGaccug..................  2      1  1st
......................................................................uucguugucguugCaaccug..................  1      1  1st
......................................................................uucgGugucguugaaaccug..................  2      1  1st
```

```
                                    Star                                        Mature
agcgguaggaaaucgagagaagauauuguacaggcuucucgauuagcgaacaaauaucguuauuuaaauguucguugucguugaaaccugaacgaaacuccucauucc
......................................................................uucguugCcguugaaaccug..................   1       1       1st
......................................................................uucguugucgCugaaaccug..................   2       1       1st
......................................................................uucguuUucguugaaaccug..................   1       1       1st
......................................................................uAcguugucguugaaaccug..................   2       1       1st
......................................................................uucguugucguugaaaccuA..................   20      1       1st
......................................................................uucguugucUuugaaaccug..................   1       1       1st
......................................................................uucguugucguugaaacUug..................   2       1       1st
......................................................................uucguugucgAugaaaccug..................   1       1       1st
......................................................................uuAguugucguugaaaccug..................   2       1       1st
......................................................................uucguugucguugaaaccAg..................   2       1       1st
......................................................................uucguugGcguugaaaccug..................   1       1       1st
......................................................................uucguugucguuAaaaccuga.................   1       1       1st
......................................................................uucguugucguugaCaccuga.................   6       1       1st
......................................................................uucguugucguugaGaccuga.................   9       1       1st
......................................................................uuAguugucguugaaaccuga.................   4       1       1st
......................................................................uuUguugucguugaaaccuga.................   2       1       1st
......................................................................uucguugucguuCaaaccuga.................   2       1       1st
......................................................................uucguugucguugaaaccugG.................   10      1       1st
......................................................................uucguugucguugaaaccAga.................   1       1       1st
......................................................................uucCuugucguugaaaccuga.................   2       1       1st
......................................................................uucguuAucguugaaaccuga.................   2       1       1st
......................................................................uucguugucCuugaaaccuga.................   2       1       1st
......................................................................uucguugucAuugaaaccuga.................   1       1       1st
......................................................................uucguugucguugaaaccuAa.................   3       1       1st
......................................................................uucguugucguugaaacGuga.................   2       1       1st
......................................................................uuGguugucguugaaaccuga.................   1       1       1st
......................................................................uucguugucguugCaaccuga.................   6       1       1st
......................................................................uucguuguUguugaaaccuga.................   3       1       1st
......................................................................Gucguugucguugaaaccuga.................   4       1       1st
......................................................................uucguugucgCugaaaccuga.................   3       1       1st
......................................................................uucguugucguugaaaccugU.................   75      1       1st
......................................................................uucguugucguugaaaccuCa.................   3       1       1st
......................................................................uucguugucguugaaGccuga.................   1       1       1st
......................................................................uucUuugucguugaaaccuga.................   4       1       1st
......................................................................uucguugucguugaaacAuga.................   1       1       1st
......................................................................uucguugucgAugaaaccuga.................   4       1       1st
......................................................................uGcguugucguugaaaccuga.................   1       1       1st
......................................................................uucguuCucguugaaaccuga.................   2       1       1st
......................................................................uucguugCcguugaaaccuga.................   1       1       1st
......................................................................uucguugGcguugaaaccuga.................   4       1       1st
......................................................................uucguugucguugaaaccuga.................   14082   0       1st
......................................................................uucguugucguugaaaccGga.................   3       1       1st
......................................................................uucguugucguugUaaccuga.................   7       1       1st
......................................................................uucguugucguAgaaaccuga.................   2       1       1st
......................................................................uucguugucguugaaaccuUa.................   6       1       1st
......................................................................Aucguugucguugaaaccuga.................   3       1       1st
......................................................................uucguugucguGgaaaccuga.................   1       1       1st
......................................................................uucguugucguugaUaccuga.................   4       1       1st
......................................................................uucguugucguugaaaccugC.................   1       1       1st
......................................................................uucguugucUuugaaaccuga.................   2       1       1st
......................................................................uucguugucguugaaaUcuga.................   5       1       1st
......................................................................uAcguugucguugaaaccuga.................   13      1       1st
......................................................................uucguugAcguugaaaccuga.................   1       1       1st
......................................................................uucgCugucguugaaaccuga.................   1       1       1st
......................................................................uucguugucguugaaaccCga.................   2       1       1st
......................................................................uucguugucguugaaUccuga.................   1       1       1st
......................................................................uucguugucguugaaacUuga.................   4       1       1st
......................................................................Cucguugucguugaaaccuga.................   3       1       1st
......................................................................uucguuUucguugaaaccuga.................   2       1       1st
......................................................................uucguugucguugGaaccuga.................   8       1       1st
......................................................................uucguugucguugGaaccugaa................   2       1       1st
......................................................................uucguugGcguugaaaccugaa................   1       1       1st
......................................................................uucguugucguugCaaccugaa................   1       1       1st
......................................................................uucguugucguugaaaccugaU................   155     1       1st
......................................................................uucguugucguuAaaaccugaa................   2       1       1st
......................................................................uucguugucguugUaaccugaa................   1       1       1st
......................................................................uucguugucguugaaaccugaC................   1069    1       1st
......................................................................uucguCgucguugaaaccugaa................   1       1       1st
......................................................................uucguugucguugaCaccugaa................   1       1       1st
......................................................................uucguugucguugaaaGcugaa................   1       1       1st
```

Star   Mature

```
agcgguaggaaaucgagagaagauauuguacaggcuucucgauuagcgaacaaauaucguuauuuaaauguucguugucguugaaaccugaacgaaacuccucauucc
.....................................................................uucguugucguAgaaaccugaa.................    2     1    1st
.....................................................................uucguugucguugaaaccAgaa.................    3     1    1st
.....................................................................uucguugucguugaaaccugaa.................    4137  0    1st
.....................................................................uucguugucguugaaaccugUa.................    2     1    1st
.....................................................................uucguugucguugaaaUcugaa.................    1     1    1st
.....................................................................uucUuugucguugaaaccugaa.................    1     1    1st
.....................................................................uucguugucguugaaaccugGa.................    9     1    1st
.....................................................................uucguugucgAugaaaccugaa.................    1     1    1st
.....................................................................uucguuCucguugaaaccugaa.................    1     1    1st
.....................................................................uucguugucguuCaaaccugaa.................    1     1    1st
.....................................................................uAcguugucguugaaaccugaa.................    4     1    1st
.....................................................................uucguugucguugaaaccuUaa.................    4     1    1st
.....................................................................Aucguugucguugaaaccugaa.................    1     1    1st
.....................................................................uuAguugucguugaaaccugaa.................    1     1    1st
.....................................................................uucguugucguugaaaccuCaa.................    2     1    1st
.....................................................................uucguugucguugaaaccCgaa.................    2     1    1st
.....................................................................uucguugucguugaaaccugaG.................    8     1    1st
.....................................................................uucguugucguugaaaccugaac................    61    0    1st
.....................................................................uucguugucguugaaaccugaCc................    445   1    1st
.....................................................................uucguugucguugaaaccugaaG................    29    1    1st
.....................................................................uucguugucguugaaaccugaaU................    406   1    1st
.....................................................................uucguugucguugaaaccugaaA................    332   1    1st
.....................................................................uucguugucguugaaaccugaaUg...............    1     1    1st
.....................................................................uucguugucguugaaaccugaacU...............    4     1    1st
.....................................................................uucguugucguCgaaaccugaacg...............    1     1    1st
......................................................................ucguugucguugaaaccug...................    12    0    1st
......................................................................uUguugucguugaaaccuga..................    1     1    1st
......................................................................ucguugucguugaaaccAga..................    1     1    1st
......................................................................ucguugucguugaaaccuga..................    43    0    1st
......................................................................ucguugucguugaaaccugaU.................    2     1    1st
......................................................................ucguugucguugaaaccugaa.................    17    0    1st
......................................................................ucguugucguugaaaccugaC.................    4     1    1st
......................................................................ucguugucguugaaaccugaaU................    5     1    1st
......................................................................ucguugucguugaaaccugaCc................    1     1    1st
......................................................................ucguugucguugaaaccugaaA................    1     1    1st
.......................................................................cguugucguugaaaccug...................    1     0    1st
.......................................................................Uguugucguugaaaccuga..................    1     1    1st
.......................................................................cguugucguugaaaccuga..................    1     0    1st
.......................................................................cguugucguugaaaccugaa.................    3     0    1st
........................................................................guugucguugaaaccugaa.................    1     0    1st
.........................................................................uugucguugaaaccugaCc................    1     1    1st
```

Provisional ID	:	Scaffold_11093_3586
Score total	:	366498.3
Score for star read(s)	:	3.9
Score for read counts	:	366491.5
Score for mfe	:	1.9
Score for randfold	:	1.6
Score for cons. seed	:	-0.6
Total read count	:	718869
Mature read count	:	597794
Loop read count	:	1
Star read count	:	121074

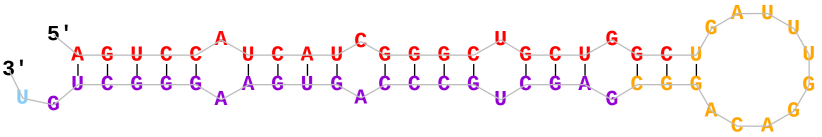

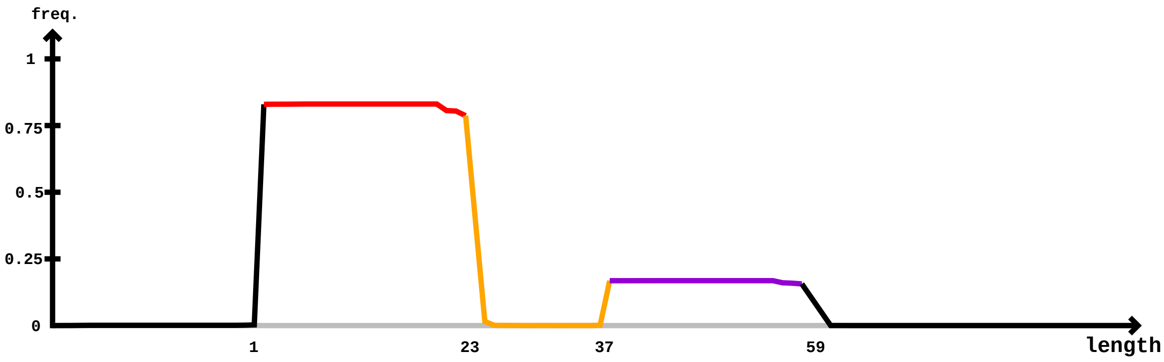

Mature Star

```
5'- aucuauggcucuuucaguauaguccaucaucgggcugcuggcugauuuggacaggcgagcugcccagugaagggcuguucuguuagggaagcaucagcagcuaagacucag -3'   obs
    aucuauggcucuuucaguauaguccaucaucgggcugcuggcugauuuggacaggcgagcugcccagugaagggcuguucuguuagggaagcaucagcagcuaagacucag        exp
    .(((.((((.((..(((.(((((((.((((.((((.(((.(((..........))).))).)))).)))).))))))).)))..))....((....)).)))))))..... reads  mm  sample
    ...................Caguccaucaucgggcugcuggc..................................................................... 2      1   CA1
    ...................Caguccaucaucgggcugcuggcu.................................................................... 1      1   CA1
    ....................aguccaucaucgggcugcu........................................................................ 175    0   CA1
    ....................aguccGucaucgggcugcu........................................................................ 2      1   CA1
    ....................aguccaucaucgggcugcA........................................................................ 11     1   CA1
    ....................agCccaucaucgggcugcu........................................................................ 1      1   CA1
    ....................aguccaucaucgggcCgcu........................................................................ 2      1   CA1
    ....................aguccaucaAcgggcugcu........................................................................ 1      1   CA1
    ....................aguccaucaucgggcugcG........................................................................ 2      1   CA1
    ....................aguccaucaucgggcAgcu........................................................................ 1      1   CA1
    ....................aguccaucaucgggcugcC........................................................................ 15     1   CA1
    ....................aguccaucaucgggcugcug....................................................................... 13     0   CA1
    ....................aguccaucaucgggcugcugC...................................................................... 2      1   CA1
    ....................aguccaucaucgggcugcugA...................................................................... 6      1   CA1
    ....................aguccaucaucgggcugcugg...................................................................... 105    0   CA1
    ....................aguccaucaucgggcuAcugg...................................................................... 1      1   CA1
    ....................aguccaucaucgUgcugcugg...................................................................... 1      1   CA1
    ....................aAuccaucaucgggcugcugg...................................................................... 1      1   CA1
    ....................aguccaucauAgggcugcugg...................................................................... 1      1   CA1
    ....................aguccaucaucgggcugcugU...................................................................... 1      1   CA1
    ....................aguccaucaucgggcugcGggc..................................................................... 9      1   CA1
    ....................aguccaucaucgggcCgcuggc..................................................................... 7      1   CA1
    ....................aguccaucaucgggcuAcuggc..................................................................... 6      1   CA1
    ....................aguccaucaucgggcugAuggc..................................................................... 1      1   CA1
    ....................aguccaucaucgggcugcuggU..................................................................... 556    1   CA1
    ....................aguccauUaucgggcugcuggc..................................................................... 3      1   CA1
    ....................agucAaucaucgggcugcuggc..................................................................... 2      1   CA1
    ....................aguccUucaucgggcugcuggc..................................................................... 1      1   CA1
    ....................aguccaucaucgggUugcuggc..................................................................... 5      1   CA1
    ....................agucUaucaucgggcugcuggc..................................................................... 1      1   CA1
    ....................aguccaucaucgggcugcuggA..................................................................... 170    1   CA1
    ....................Gguccaucaucgggcugcuggc..................................................................... 20     1   CA1
    ....................aAuccaucaucgggcugcuggc..................................................................... 2      1   CA1
```

```
                    Mature                             Star
aucuauggcucuuucaguauaguccaucaucgggcugcuggcugauuuggacaggcgagcugcccagugaagggcuguucuguuagggaagcaucagcagcuaagacucag
....................aguccaucaucgggcugcAggc.....................................................................  3     1   CA1
....................aguccaucaucgggcugcuggc.....................................................................  3582  0   CA1
....................aUuccaucaucgggcugcuggc.....................................................................  1     1   CA1
....................Uguccaucaucgggcugcuggc.....................................................................  18    1   CA1
....................aguccaucaucgggcugcuAgc.....................................................................  4     1   CA1
....................aguccaucaucgAgcugcuggc.....................................................................  9     1   CA1
....................aguccaucauUgggcugcuggc.....................................................................  5     1   CA1
....................aguccaucaucggAcugcuggc.....................................................................  5     1   CA1
....................aguccaucaucgggcugcugCc.....................................................................  3     1   CA1
....................Cguccaucaucgggcugcuggc.....................................................................  1     1   CA1
....................aguccaucaucgggcugcCggc.....................................................................  11    1   CA1
....................aguccaAcaucgggcugcuggc.....................................................................  2     1   CA1
....................aguccaucaucAggcugcuggc.....................................................................  3     1   CA1
....................agCccaucaucgggcugcuggc.....................................................................  14    1   CA1
....................aguccaucaCcgggcugcuggc.....................................................................  17    1   CA1
....................aguccaucaucgggcugUuggc.....................................................................  6     1   CA1
....................aguccaCcaucgggcugcuggc.....................................................................  13    1   CA1
....................aguccaucaucgggcugcugAc.....................................................................  5     1   CA1
....................aguccGucaucgggcugcuggc.....................................................................  13    1   CA1
....................aguccaucaucgggcugcuggG.....................................................................  7     1   CA1
....................aguccaucGucgggcugcuggc.....................................................................  6     1   CA1
....................aguccaucaucgggcAgcuggc.....................................................................  1     1   CA1
....................aguccaucaucgggcugcuggcu....................................................................  1798  0   CA1
....................aguccaucaucgggcugcugAcu....................................................................  2     1   CA1
....................aguccaucaucgggUugcuggcu....................................................................  1     1   CA1
....................aguccaucaucgggcAgcuggcu....................................................................  1     1   CA1
....................agCccaucaucgggcugcuggcu....................................................................  6     1   CA1
....................aguUcaucaucgggcugcuggcu....................................................................  1     1   CA1
....................aguccaucauUgggcugcuggcu....................................................................  3     1   CA1
....................agAccaucaucgggcugcuggcu....................................................................  1     1   CA1
....................aguccaucaucgggcugcuggAu....................................................................  8     1   CA1
....................aguccaucaucgggcugcuggcC....................................................................  236   1   CA1
....................aguccaucaucgggcugcuggUu....................................................................  6     1   CA1
....................Gguccaucaucgggcugcuggcu....................................................................  6     1   CA1
....................aguccaucaCcgggcugcuggcu....................................................................  11    1   CA1
....................aguccCucaucgggcugcuggcu....................................................................  2     1   CA1
....................aguccaucGucgggcugcuggcu....................................................................  5     1   CA1
....................aguccaucaucgggcugAuggcu....................................................................  2     1   CA1
....................aguAcaucaucgggcugcuggcu....................................................................  1     1   CA1
....................aguccaucaucgggcugcuggcA....................................................................  199   1   CA1
....................aguccaucaucgUgcugcuggcu....................................................................  1     1   CA1
....................aguccGucaucgggcugcuggcu....................................................................  2     1   CA1
....................aguccauAaucgggcugcuggcu....................................................................  1     1   CA1
....................Uguccaucaucgggcugcuggcu....................................................................  1     1   CA1
....................aguccUucaucgggcugcuggcu....................................................................  1     1   CA1
....................aguccaucaucgggcugUuggcu....................................................................  4     1   CA1
....................aguccaucaucggAcugcuggcu....................................................................  2     1   CA1
....................aAuccaucaucgggcugcuggcu....................................................................  2     1   CA1
....................aguccaucaucgggcCgcuggcu....................................................................  4     1   CA1
....................aguccaucaucgggcugcAggcu....................................................................  1     1   CA1
....................aguccaCcaucgggcugcuggcu....................................................................  10    1   CA1
....................aguccaucaucgggcugcuggGu....................................................................  2     1   CA1
....................aguccaucaucgggcugcCggcu....................................................................  5     1   CA1
....................aguccaucaucgAgcugcuggcu....................................................................  2     1   CA1
....................aguccaucaucgggcuAcuggcu....................................................................  2     1   CA1
....................aguccaucaucAggcugcuggcu....................................................................  1     1   CA1
....................agucUaucaucgggcugcuggcu....................................................................  1     1   CA1
....................aguccaucaucgggcugcuggcG....................................................................  38    1   CA1
....................aguGcaucaucgggcugcuggcu....................................................................  1     1   CA1
....................aguccaucaucgggcugcuAgcu....................................................................  2     1   CA1
....................aUuccaucaucgggcugcuggcu....................................................................  3     1   CA1
....................aguccaucaucgggcugcuggcug...................................................................  3     0   CA1
....................aguccaucaucgggcugcuggcuA...................................................................  11    1   CA1
....................aguccaucaucgggcugcuggcuU...................................................................  140   1   CA1
....................aguccaucaucgggcugcuggcuC...................................................................  6     1   CA1
....................aguccaucaucgggcugcuggcuAa..................................................................  1     1   CA1
....................aguccaucaucgggcugcuggcuAau.................................................................  2     1   CA1
....................aguccaucaucgggcugcuggcuUau.................................................................  1     1   CA1
.....................guccaucaucgggcugcuggc.....................................................................  3     0   CA1
.......................................................cgagcugcccagugaagggcugu.................................  2     0   CA1
```

```
                    Mature                            Star
aucuauggcucuuucaguauaguccaucaucgggcugcuggcugauuuggacaggcgagcugcccagugaagggcuguucuguuagggaagcaucagcagcuaagacucag
........................................................gagcugcccagugaaggg.....................................   5     0   CA1
........................................................gagcugcccagugaagggc....................................   1     0   CA1
........................................................gagcugcccagugaagggcug..................................   179   0   CA1
........................................................gagcugcccagugaagggcuA..................................   12    1   CA1
........................................................gagAugcccagugaagggcug..................................   3     1   CA1
........................................................gagcCgcccagugaagggcug..................................   1     1   CA1
........................................................gagcugcccUgugaagggcug..................................   2     1   CA1
........................................................gagcugcccagugaagggcuU..................................   8     1   CA1
........................................................gGgcugcccagugaagggcug..................................   1     1   CA1
........................................................gagcugcccagCgaagggcug..................................   2     1   CA1
........................................................gagcugcccGgugaagggcug..................................   1     1   CA1
........................................................gagcugcccaAugaagggcugu.................................   1     1   CA1
........................................................gagcugcccagugaagggcugA.................................   2     1   CA1
........................................................gagcugcccagugaagggcugC.................................   7     1   CA1
........................................................gagcugccUagugaagggcugu.................................   5     1   CA1
........................................................gagcugUccagugaagggcugu.................................   2     1   CA1
........................................................gagcugcccagugaagggcugG.................................   1     1   CA1
........................................................gagcugcccagugaagggcugu.................................   96    0   CA1
........................................................gGgcugcccagugaagggcugu.................................   1     1   CA1
........................................................gagcugcccagCgaagggcugu.................................   1     1   CA1
........................................................gagcugcccagugaagggcuguA................................   32    1   CA1
........................................................gagcugcGcagugaagggcuguu................................   1     1   CA1
........................................................gagcugcccagugaagggcuguu................................   5     0   CA1

aucuauggcucuuucaguau...........................................................................................   2     0   3rd
.ucuauggcucuuucaguau...........................................................................................   4     0   3rd
..................Uaguccaucaucgggcugcuggc......................................................................   4     1   3rd
...................uaguccaucaucgggcugcug.......................................................................   1     0   3rd
...................uaguccaucaucggAcugcuggc.....................................................................   1     1   3rd
...................uagCccaucaucgggcugcuggc.....................................................................   1     1   3rd
...................uaguccaucaucgggcugcuggc.....................................................................   5     0   3rd
...................uaguccaucaucgggcugcuggcu....................................................................   3     0   3rd
....................aguccaucaucgggcugc.........................................................................   1     0   3rd
....................aguccaucaucgggcugcu........................................................................   2     0   3rd
....................aguccaucaucgggcugcug.......................................................................   15    0   3rd
....................aguccaucaucgggcugcAgg......................................................................   1     1   3rd
....................aguccaucaucgggcugcugg......................................................................   108   0   3rd
....................aguccaucaucgggcAgcuggc.....................................................................   1     1   3rd
....................aguUcaucaucgggcugcuggc.....................................................................   1     1   3rd
....................agAccaucaucgggcugcuggc.....................................................................   1     1   3rd
....................aguccaucaucgggcuCcuggc.....................................................................   1     1   3rd
....................aguccaucaucgggcugUuggc.....................................................................   2     1   3rd
....................aguccauAaucgggcugcuggc.....................................................................   1     1   3rd
....................aguccGucaucgggcugcuggc.....................................................................   1     1   3rd
....................aguccaucaucgggcugcuggc.....................................................................   2522  0   3rd
....................aguccaucaucgggcugcuggA.....................................................................   9     1   3rd
....................aguccaucaucgggcugcuggU.....................................................................   16    1   3rd
....................aguccaucaucgggAugcuggc.....................................................................   1     1   3rd
....................aguccaucaucgggcugcugCc.....................................................................   3     1   3rd
....................aguccaucaucgggcugcugAc.....................................................................   5     1   3rd
....................aguccaucaucgggUugcuggc.....................................................................   1     1   3rd
....................aguccaucaucgggcuUcuggc.....................................................................   1     1   3rd
....................agCccaucaucgggcugcuggc.....................................................................   2     1   3rd
....................aguccaAcaucgggcugcuggc.....................................................................   1     1   3rd
....................aguccaCcaucgggcugcuggc.....................................................................   2     1   3rd
....................aguccaucauUgggcugcuggc.....................................................................   2     1   3rd
....................Gguccaucaucgggcugcuggc.....................................................................   1     1   3rd
....................Uguccaucaucgggcugcuggc.....................................................................   1     1   3rd
....................aguccaucaucgggcugAuggc.....................................................................   1     1   3rd
....................aguccaucaucgggcugcugUc.....................................................................   1     1   3rd
....................aguccaucaucggAcugcuggc.....................................................................   2     1   3rd
....................aguccaucaucAggcugcuggc.....................................................................   2     1   3rd
....................agucUaucaucgggcugcuggc.....................................................................   1     1   3rd
....................aguccCucaucgggcugcuggcu....................................................................   1     1   3rd
....................aguccaucaucgggcugcuggcC....................................................................   11    1   3rd
....................aguccaucUucgggcugcuggcu....................................................................   1     1   3rd
....................aguccaucaucgggcuUcuggcu....................................................................   1     1   3rd
....................aguccaucaucgggUugcuggcu....................................................................   1     1   3rd
....................aguccaucaucgggcugcuggcG....................................................................   2     1   3rd
....................aguccaucauUgggcugcuggcu....................................................................   1     1   3rd
```

Mature **Star**

aucuauggcucuuucaguauaguccaucaucgggcugcuggcugauuuggacaggcgagcugcccagugaagggcuguucuguuagggaagcaucagcagcuaagacucag			
....................aguccaucaucgggcugcuggUu..	1	1	3rd
....................aguccaucaucgggcugcugUcu..	1	1	3rd
....................aguccaucaucgggcugcuggcA..	84	1	3rd
....................aguccaucaucgggcugcuggcu..	317	0	3rd
....................aguccaucaucgggcugcGggcu..	1	1	3rd
....................aguccaucaucggAcugcuggcu..	1	1	3rd
....................aguccaucaucgggcugcuggAu..	2	1	3rd
....................aguccaucaucgggcugcuggcuA...	7	1	3rd
....................aguccaucaucgggcugcuggcuC...	1	1	3rd
....................aguccaucaucgggcugcuggcuU...	89	1	3rd
....................aguccaucaucgggcugcuggcuAa..	1	1	3rd
.....................guccaucaucgggcugcuggc...	3	0	3rd
.....................guccaucaucgggcugcuggcu..	1	0	3rd
......................uUcaucaucgggcugcuggc...	1	1	3rd
......................uccaucaucgggcugcuggc...	2	0	3rd
........................caucaucgggcugcuggc...	6	0	3rd
....aggUgagcugcccagugaagggcu...................................	1	1	3rd
....Gggcgagcugcccagugaagggcugu.................................	1	1	3rd
....ggcgagcugcccagugaagggcug..................................	1	0	3rd
....Ugagcugcccagugaagggc....................................	1	1	3rd
....cgagcugcccagugaagggcu...................................	4	0	3rd
....Ugagcugcccagugaagggcu...................................	1	1	3rd
....cgagcugcccagugaagggcA...................................	1	1	3rd
....cgagcugcccagugaagggcuU..................................	1	1	3rd
....Ugagcugcccagugaagggcug..................................	26	1	3rd
....cgagcugcccagugaagggcug..................................	6	0	3rd
....cgagcugcccagugaagggcugu.................................	24	0	3rd
....Ugagcugcccagugaagggcugu.................................	49	1	3rd
....cgagcugcccagugaagggcuguuA...............................	1	1	3rd
....gagcugcccagugaaggA.....................................	10	1	3rd
....gagcugcccagugaaggg.....................................	46	0	3rd
....gagcuAcccagugaagggc....................................	1	1	3rd
....gagcugcccagugaagggA....................................	4	1	3rd
....gagcugcccagugaagggc....................................	77	0	3rd
....gagcugcccagugaagggU....................................	2	1	3rd
....gagcuAcccagugaagggcu...................................	2	1	3rd
....gagUugcccagugaagggcu...................................	2	1	3rd
....gGgcugcccagugaagggcu...................................	4	1	3rd
....gagcugcccagugaUgggcu...................................	1	1	3rd
....gagcugcccagugaagggcu...................................	628	0	3rd
....gagcugcccagugaGgggcu...................................	3	1	3rd
....gagcugcccagugUagggcu...................................	1	1	3rd
....gagcugAccagugaagggcu...................................	1	1	3rd
....gUgcugcccagugaagggcu...................................	1	1	3rd
....gagcugccUagugaagggcu...................................	1	1	3rd
....gagcugcccagugaaggAcu...................................	2	1	3rd
....gagcugcccagugaagggcG...................................	1	1	3rd
....gagcugcccagugaagggcC...................................	1	1	3rd
....gagcugcccagugaaggCcu...................................	1	1	3rd
....gagcugcccaUugaagggcu...................................	1	1	3rd
....Uagcugcccagugaagggcug..................................	3	1	3rd
....gagcugcccagugaagggcuA..................................	30	1	3rd
....gagUugcccagugaagggcug..................................	8	1	3rd
....gCgcugcccagugaagggcug..................................	10	1	3rd
....gagcGgcccagugaagggcug..................................	1	1	3rd
....gaCcugcccagugaagggcug..................................	1	1	3rd
....gagcugcccagAgaagggcug..................................	1	1	3rd
....gagcugccUagugaagggcug..................................	3	1	3rd
....gagcugcccagugaagggcuU..................................	25	1	3rd
....gagcuCcccagugaagggcug..................................	5	1	3rd
....gagcugcccagCgaagggcug..................................	2	1	3rd
....Cagcugcccagugaagggcug..................................	2	1	3rd
....gagcuUcccagugaagggcug..................................	1	1	3rd
....gGgcugcccagugaagggcug..................................	38	1	3rd
....gagcugcccagugUagggcug..................................	3	1	3rd
....gaAcugcccagugaagggcug..................................	2	1	3rd
....gagcugcccaCugaagggcug..................................	3	1	3rd
....gagcugcccagugaaggCcug..................................	3	1	3rd
....gagcugcccagugaaggAcug..................................	9	1	3rd
....gagcugcccagugaagggcGg..................................	1	1	3rd

```
                    Mature                                Star
aucuauggcucuuucaguauaguccaucaucgggcugcuggcugauuuggacaggcgagcugcccagugaagggcuguucuguuagggaagcaucagcagcuaagacucag
........................................................gagcugAccagugaagggcug..................................  1     1  3rd
........................................................gagcugcccagugGagggcug..................................  5     1  3rd
........................................................gagcugcccUgugaagggcug..................................  1     1  3rd
........................................................gagcugcccaguCaagggcug..................................  3     1  3rd
........................................................gagcugGccagugaagggcug..................................  2     1  3rd
........................................................gaUcugcccagugaagggcug..................................  2     1  3rd
........................................................gagcugcccaAugaagggcug..................................  4     1  3rd
........................................................gagcugcccagugaUgggcug..................................  3     1  3rd
........................................................gagcugcccagugaagggUug..................................  12    1  3rd
........................................................gagcAgcccagugaagggcug..................................  1     1  3rd
........................................................gagcugUccagugaagggcug..................................  12    1  3rd
........................................................gagcugcccagugaaAggcug..................................  1     1  3rd
........................................................gagcugcccagugaagggcug..................................  9901  0  3rd
........................................................gUgcugcccagugaagggcug..................................  7     1  3rd
........................................................gagcugcUcagugaagggcug..................................  2     1  3rd
........................................................gagcugcccagugaagggcAg..................................  1     1  3rd
........................................................gagcugcccagugaagUgcug..................................  1     1  3rd
........................................................gagcugcccagugaaCggcug..................................  2     1  3rd
........................................................gagcugcccagugaagggcCg..................................  9     1  3rd
........................................................gagcugcccagugaagCgcug..................................  5     1  3rd
........................................................gagcugcccagugaaUggcug..................................  4     1  3rd
........................................................gagcugcccaguUaagggcug..................................  3     1  3rd
........................................................gagcugcccagugaGgggcug..................................  5     1  3rd
........................................................gagcuAcccagugaagggcug..................................  19    1  3rd
........................................................gagcugcccagugaaggUcug..................................  3     1  3rd
........................................................gagcugcccagugaaAggcugu.................................  2     1  3rd
........................................................gagcugcccagugaagAgcugu.................................  2     1  3rd
........................................................gagcugcccagugUagggcugu.................................  3     1  3rd
........................................................gagcugcccagugaagggcAgu.................................  1     1  3rd
........................................................gagcugcAcagugaagggcugu.................................  2     1  3rd
........................................................gaUcugcccagugaagggcugu.................................  4     1  3rd
........................................................gagcugcccagugaUgggcugu.................................  7     1  3rd
........................................................gagcugccUagugaagggcugu.................................  2     1  3rd
........................................................gagcAgcccagugaagggcugu.................................  2     1  3rd
........................................................gagcugcccagugaagggcuAu.................................  1     1  3rd
........................................................gagcugcccagugaagggcugA.................................  41    1  3rd
........................................................gagcuUcccagugaagggcugu.................................  3     1  3rd
........................................................gagcugcccagugaagggcuUu.................................  1     1  3rd
........................................................gagcugGccagugaagggcugu.................................  3     1  3rd
........................................................gagcugcccaUugaagggcugu.................................  2     1  3rd
........................................................gagcugAccagugaagggcugu.................................  2     1  3rd
........................................................gagcugcccaAugaagggcugu.................................  1     1  3rd
........................................................gagcugcccagugaCgggcugu.................................  2     1  3rd
........................................................gagcugcUcagugaagggcugu.................................  11    1  3rd
........................................................gagcugcccagugaagggcugC.................................  38    1  3rd
........................................................gagcugccGagugaagggcugu.................................  1     1  3rd
........................................................gagcugcccagugaagCgcugu.................................  8     1  3rd
........................................................gUgcugcccagugaagggcugu.................................  26    1  3rd
........................................................gagcCgcccagugaagggcugu.................................  2     1  3rd
........................................................gaAcugcccagugaagggcugu.................................  2     1  3rd
........................................................gaCcugcccagugaagggcugu.................................  4     1  3rd
........................................................gagcugcccCgugaagggcugu.................................  1     1  3rd
........................................................gagcugcccagugaagggUugu.................................  18    1  3rd
........................................................gagcugcccagugaagggcCgu.................................  5     1  3rd
........................................................gagcugcccagugaagggcuCu.................................  3     1  3rd
........................................................gagcugcccagugaagggcGgu.................................  6     1  3rd
........................................................gagcuCcccagugaagggcugu.................................  7     1  3rd
........................................................gagcugcccaguUaagggcugu.................................  3     1  3rd
........................................................gagcugcccagugaaggAcugu.................................  9     1  3rd
........................................................gagcugcccaguCaagggcugu.................................  3     1  3rd
........................................................Uagcugcccagugaagggcugu.................................  2     1  3rd
........................................................gagcugcccagAgaagggcugu.................................  1     1  3rd
........................................................gagcugUccagugaagggcugu.................................  19    1  3rd
........................................................gagcugcccagugCagggcugu.................................  3     1  3rd
........................................................gagcugcccagugaaCggcugu.................................  4     1  3rd
........................................................gagcugcccagugaaggCcugu.................................  2     1  3rd
........................................................gagcugcccagugGagggcugu.................................  1     1  3rd
........................................................gagcugcccagugaagggAugu.................................  1     1  3rd
........................................................gagcugcccUgugaagggcugu.................................  2     1  3rd
........................................................gagcugcccGgugaagggcugu.................................  2     1  3rd
```

Mature Star

```
aucuauggcucuuucaguauaguccaucaucgggcugcuggcugauuuggacaggcgagcugcccagugaagggcuguucuguuagggaagcaucagcagcuaagacucag
........................................................gagUugcccagugaagggcugu.................................   10      1   3rd
........................................................gagcugcccaCugaagggcugu.................................   3       1   3rd
........................................................gagcugcccagugaGgggcugu.................................   12      1   3rd
........................................................Cagcugcccagugaagggcugu.................................   2       1   3rd
........................................................gGgcugcccagugaagggcugu.................................   50      1   3rd
........................................................gagcugcccagugaaggUcugu.................................   7       1   3rd
........................................................gagcugcccagugaagggcugG.................................   2       1   3rd
........................................................gagcugcccagugaagggcugu.................................   14080   0   3rd
........................................................gagcuAcccagugaagggcugu.................................   23      1   3rd
........................................................gagcugcccagugaagUgcugu.................................   2       1   3rd
........................................................gagcugcccagugaaUggcugu.................................   2       1   3rd
........................................................gCgcugcccagugaagggcugu.................................   14      1   3rd
........................................................gagcugUccagugaagggcuguu................................   2       1   3rd
........................................................gagcugcccagugaagggcugAu................................   1       1   3rd
........................................................gagcuAcccagugaagggcuguu................................   1       1   3rd
........................................................gagcugcccagugaagUgcuguu................................   1       1   3rd
........................................................gagcugcccagugaagggcuguA................................   439     1   3rd
........................................................gagcugcccagugaagggUuguu................................   2       1   3rd
........................................................gagUugcccagugaagggcuguu................................   2       1   3rd
........................................................gagcuCcccagugaagggcuguu................................   1       1   3rd
........................................................gagcugcccagugaagggcuguu................................   418     0   3rd
........................................................gagcugcccagugaCgggcuguu................................   1       1   3rd
........................................................gagcugcccagugUagggcuguu................................   1       1   3rd
........................................................gagcugcccagCgaagggcuguu................................   1       1   3rd
........................................................gagcugcccagugaagggcuguuA...............................   1       1   3rd
........................................................gagcugcccagugaagggcuguuU...............................   3       1   3rd
........................................................gagcugcccagugaagggcuguuGu..............................   2       1   3rd
........................................................gagcugcccagugaagggcuguuUu..............................   9       1   3rd
.........................................................agcugcccagugaagggcug..................................   2       0   3rd
.........................................................agcugcccagugaagggcugu.................................   1       0   3rd
.........................................................Cgcugcccagugaagggcugu.................................   1       1   3rd
..........................................................gcugcccagugaagggcuguu................................   1       0   3rd
...........................................................cugcccagugaagggcugu.................................   5       0   3rd
...........................................................cugcccagugaagggcuguA................................   1       1   3rd
...........................................................cugcccagugaagggcuguu................................   1       0   3rd
............................................................ugcccagugaagggcugu.................................   4       0   3rd

aucuauggcucuuucaguau...........................................................................................   10      0   BRA
aucuauggcucuuucaguaA...........................................................................................   1       1   BRA
.ucuauggcucuuucaguau...........................................................................................   5       0   BRA
..cuauggcucuuucaguau...........................................................................................   2       0   BRA
...................Caguccaucaucgggcugcuggc.....................................................................   6       1   BRA
...................Caguccaucaucgggcugcuggcu....................................................................   9       1   BRA
....................aguccaucaucgggcugc.........................................................................   4       0   BRA
....................aguccaCcaucgggcugcu........................................................................   3       1   BRA
....................aguccaucaCcgggcugcu........................................................................   1       1   BRA
....................aguccaucaucgggcuAcu........................................................................   1       1   BRA
....................aguccaucGucgggcugcu........................................................................   2       1   BRA
....................aguccaucaucgggcugcG........................................................................   15      1   BRA
....................Cguccaucaucgggcugcu........................................................................   1       1   BRA
....................aguccaucaucgggcugcu........................................................................   532     0   BRA
....................agCccaucaucgggcugcu........................................................................   2       1   BRA
....................aguUcaucaucgggcugcu........................................................................   1       1   BRA
....................agucUaucaucgggcugcu........................................................................   2       1   BRA
....................aguccaucaucgggcugUu........................................................................   1       1   BRA
....................aguccaucaucgggcugAu........................................................................   1       1   BRA
....................aguccaucaucgggcugcA........................................................................   51      1   BRA
....................aguccaucaucgggcugcC........................................................................   46      1   BRA
....................aguccaucaucgggcugcuA.......................................................................   2       1   BRA
....................aguccaucaucgggAugcug.......................................................................   1       1   BRA
....................aguccaucaucggAcugcug.......................................................................   1       1   BRA
....................aguccaucauAgggcugcug.......................................................................   1       1   BRA
....................aguccaucaucgggcugcug.......................................................................   37      0   BRA
....................aguccaucaucgggcugcCgg......................................................................   1       1   BRA
....................aguccaucaucgggcuAcugg......................................................................   1       1   BRA
....................aguccaucaucgggcugcugg......................................................................   280     0   BRA
....................aguccaucaucgggcugcugA......................................................................   19      1   BRA
....................aguccaucaucgggcugcugC......................................................................   5       1   BRA
....................aguccaucaucgggUugcugg......................................................................   3       1   BRA
....................aguccaucaucAggcugcugg......................................................................   2       1   BRA
```

Mature Star

```
aucuauggcucuuucaguauaguccaucaucgggcugcuggcugauuuggacaggcgagcugcccagugaagggcuguucuguuagggaagcaucagcagcuaagacucag
....................aguccaCcaucgggcugcugg......................................................................   5      1   BRA
....................aguccaucaucgAgcugcugg......................................................................   1      1   BRA
....................aguccaucaucggUcugcugg......................................................................   1      1   BRA
....................aguccaucaucgggcugcugU......................................................................   5      1   BRA
....................aguccaucaucUggcugcugg......................................................................   1      1   BRA
....................aguccaucaCcgggcugcugg......................................................................   1      1   BRA
....................aguccaucaucgggcugcugCc.....................................................................   11     1   BRA
....................aguccaucaucUggcugcuggc.....................................................................   2      1   BRA
....................aguccaucaucgggcugcuggG.....................................................................   47     1   BRA
....................aguccaucaucCggcugcuggc.....................................................................   2      1   BRA
....................aguccaucauUgggcugcuggc.....................................................................   12     1   BRA
....................aguccGucaucgggcugcuggc.....................................................................   43     1   BRA
....................aguccaucaucgUgcugcuggc.....................................................................   2      1   BRA
....................aguccaucaucgggcuCcuggc.....................................................................   1      1   BRA
....................aguccaucaucgAgcugcuggc.....................................................................   37     1   BRA
....................aCuccaucaucgggcugcuggc.....................................................................   1      1   BRA
....................aguccCucaucgggcugcuggc.....................................................................   4      1   BRA
....................aguccaucGucgggcugcuggc.....................................................................   29     1   BRA
....................aguAcaucaucgggcugcuggc.....................................................................   2      1   BRA
....................aguccaucauAgggcugcuggc.....................................................................   3      1   BRA
....................aguccaucaucgggcugcCggc.....................................................................   53     1   BRA
....................aguccaucaucgggcGgcuggc.....................................................................   1      1   BRA
....................aguccaucaucgggAugcuggc.....................................................................   1      1   BRA
....................Uguccaucaucgggcugcuggc.....................................................................   15     1   BRA
....................aguccaucaucgggcugAuggc.....................................................................   5      1   BRA
....................agCccaucaucgggcugcuggc.....................................................................   45     1   BRA
....................aguccauUaucgggcugcuggc.....................................................................   12     1   BRA
....................aguccaucaucgggGugcuggc.....................................................................   1      1   BRA
....................aguccaucaucgggcugcugUc.....................................................................   6      1   BRA
....................agucAaucaucgggcugcuggc.....................................................................   8      1   BRA
....................agAccaucaucgggcugcuggc.....................................................................   3      1   BRA
....................agucUaucaucgggcugcuggc.....................................................................   16     1   BRA
....................aguccaucaucgggcugcAggc.....................................................................   12     1   BRA
....................aguccaucaucgggUugcuggc.....................................................................   17     1   BRA
....................aguccaucaAcgggcugcuggc.....................................................................   4      1   BRA
....................aguccaucaucgggcuAcuggc.....................................................................   22     1   BRA
....................aguccaucaucgggcugcuCgc.....................................................................   1      1   BRA
....................aguccaCcaucgggcugcuggc.....................................................................   43     1   BRA
....................aguccaucaucgggcAgcuggc.....................................................................   7      1   BRA
....................aguccaucaucgggcugcugAc.....................................................................   22     1   BRA
....................aguccaucaucgggcuUcuggc.....................................................................   1      1   BRA
....................aUuccaucaucgggcugcuggc.....................................................................   1      1   BRA
....................aguccaucaucgggcCgcuggc.....................................................................   50     1   BRA
....................aguccaucaucggUcugcuggc.....................................................................   2      1   BRA
....................aguccaucaucggAcugcuggc.....................................................................   17     1   BRA
....................aguccaucUucgggcugcuggc.....................................................................   4      1   BRA
....................aguccaucaucgggcugUuggc.....................................................................   23     1   BRA
....................aguccaucaucgggcugcuAgc.....................................................................   15     1   BRA
....................aguccaAcaucgggcugcuggc.....................................................................   9      1   BRA
....................aguccaucaucAggcugcuggc.....................................................................   19     1   BRA
....................aguccaucCucgggcugcuggc.....................................................................   6      1   BRA
....................aguccaucaucgggcugcuggc.....................................................................   14361  0   BRA
....................aAuccaucaucgggcugcuggc.....................................................................   10     1   BRA
....................Cguccaucaucgggcugcuggc.....................................................................   6      1   BRA
....................aguccaucaucgggcugcGggc.....................................................................   10     1   BRA
....................aguccaucaucgggcugcuggU.....................................................................   3116   1   BRA
....................aguccauAaucgggcugcuggc.....................................................................   1      1   BRA
....................aguGcaucaucgggcugcuggc.....................................................................   1      1   BRA
....................aguUcaucaucgggcugcuggc.....................................................................   8      1   BRA
....................aguccaucaCcgggcugcuggc.....................................................................   33     1   BRA
....................Gguccaucaucgggcugcuggc.....................................................................   36     1   BRA
....................aguccUucaucgggcugcuggc.....................................................................   6      1   BRA
....................aguccaucaucgggcugcuggA.....................................................................   926    1   BRA
....................agGccaucaucgggcugcuggc.....................................................................   2      1   BRA
....................aguccaucaucgggcugUuggcu....................................................................   5      1   BRA
....................aguccaucaCcgggcugcuggcu....................................................................   24     1   BRA
....................aguccaGcaucgggcugcuggcu....................................................................   2      1   BRA
....................aguccaucaucggUcugcuggcu....................................................................   4      1   BRA
....................aguccaucaucgggcugcCggcu....................................................................   28     1   BRA
....................aguccaucaucgggcugcuggGu....................................................................   3      1   BRA
```

Mature Star

```
aucuauggcucuuucaguauaguccaucaucgggcugcuggcugauuuggacaggcgagcugcccagugaagggcuguucuguuagggaagcaucagcagcuaagacucag
....................aguccaucaucgggcGgcuggcu....................................................................  1     1  BRA
....................aguccaucaucgggcugGuggcu....................................................................  1     1  BRA
....................aguccaucaucgggcugcuggcA....................................................................  1070  1  BRA
....................aguccaucaAcgggcugcuggcu....................................................................  4     1  BRA
....................aguccaucaucgggcugcuggcG....................................................................  339   1  BRA
....................aguccaucaucgggcuAcuggcu....................................................................  15    1  BRA
....................agAccaucaucgggcugcuggcu....................................................................  6     1  BRA
....................aguccaucaucUggcugcuggcu....................................................................  2     1  BRA
....................aguccaucaucgggcugcAggcu....................................................................  4     1  BRA
....................aguccaucaucgggcugcuAgcu....................................................................  11    1  BRA
....................agCccaucaucgggcugcuggcu....................................................................  25    1  BRA
....................agGccaucaucgggcugcuggcu....................................................................  2     1  BRA
....................aguccaucaucAggcugcuggcu....................................................................  16    1  BRA
....................aguccaucaucgUgcugcuggcu....................................................................  1     1  BRA
....................aguccaucaucgggUugcuggcu....................................................................  9     1  BRA
....................aguccaucaucgAgcugcuggcu....................................................................  17    1  BRA
....................aguccaucaucgggcugcugAcu....................................................................  18    1  BRA
....................aguccaucaucgggcugcuggcC....................................................................  1077  1  BRA
....................aguccaucauUgggcugcuggcu....................................................................  8     1  BRA
....................agucAaucaucgggcugcuggcu....................................................................  4     1  BRA
....................aguccaucaucgggcCgcuggcu....................................................................  19    1  BRA
....................Uguccaucaucgggcugcuggcu....................................................................  10    1  BRA
....................aguccaAcaucgggcugcuggcu....................................................................  3     1  BRA
....................Gguccaucaucgggcugcuggcu....................................................................  33    1  BRA
....................aguccaucaucggCcugcuggcu....................................................................  2     1  BRA
....................aAuccaucaucgggcugcuggcu....................................................................  5     1  BRA
....................aguccaucauAgggcugcuggcu....................................................................  2     1  BRA
....................agucUaucaucgggcugcuggcu....................................................................  19    1  BRA
....................aguccUucaucgggcugcuggcu....................................................................  4     1  BRA
....................aguccaucaucgggcAgcuggcu....................................................................  5     1  BRA
....................aguUcaucaucgggcugcuggcu....................................................................  6     1  BRA
....................aguccaucaucgggcugAuggcu....................................................................  1     1  BRA
....................aguccauAaucgggcugcuggcu....................................................................  2     1  BRA
....................aguccGucaucgggcugcuggcu....................................................................  32    1  BRA
....................aguccaucaucgggcuUcuggcu....................................................................  2     1  BRA
....................aguccaCcaucgggcugcuggcu....................................................................  34    1  BRA
....................aguccaucGucgggcugcuggcu....................................................................  21    1  BRA
....................aguccauUaucgggcugcuggcu....................................................................  8     1  BRA
....................Cguccaucaucgggcugcuggcu....................................................................  5     1  BRA
....................aguccaucaucgggcugcuggcu....................................................................  9236  0  BRA
....................aguccaucaucgggcugcuggAu....................................................................  51    1  BRA
....................aguccaucaucgggAugcuggcu....................................................................  1     1  BRA
....................aguccaucUucgggcugcuggcu....................................................................  3     1  BRA
....................aguAcaucaucgggcugcuggcu....................................................................  3     1  BRA
....................aguccaucaucgggcugcugCcu....................................................................  1     1  BRA
....................aguccaucaucgggcugcuggUu....................................................................  58    1  BRA
....................aUuccaucaucgggcugcuggcu....................................................................  1     1  BRA
....................aguccaucaucggAcugcuggcu....................................................................  7     1  BRA
....................aguccaucaucgggcugcuggcuA...................................................................  23    1  BRA
....................aguccaucaucgggcugcuggcuU...................................................................  443   1  BRA
....................aguccaucaucgggcugcuggcuC...................................................................  33    1  BRA
....................aguccaucaucgggcugcuggcGg...................................................................  1     1  BRA
....................aguccaucaucgggcugcuggcAg...................................................................  1     1  BRA
....................aguccaucaucgggcugcuggcug...................................................................  13    0  BRA
....................aguccaucaucgggcugcuggcuUa..................................................................  5     1  BRA
....................aguccaucaucgggcugcuggcuAa..................................................................  6     1  BRA
....................aguccaucaucgggcugcuggcuga..................................................................  1     0  BRA
....................aguccaucaucgggcugcuggcugaC.................................................................  1     1  BRA
....................aguccaucaucgggcugcuggcuAau.................................................................  2     1  BRA
....................aguccaucaucgggcugcuggcugau.................................................................  10    0  BRA
....................aguccaucaucgggcugcuggcuUau.................................................................  1     1  BRA
....................aguccaucaucgggcugcuggcugauC................................................................  2     1  BRA
....................aguccaucaucgggcugcuggcugauu................................................................  8     0  BRA
....................aguccaucaucgggcugcuggcuUauu................................................................  1     1  BRA
.....................guccaucaucgggcugcuggc.....................................................................  5     0  BRA
.....................guccaucaucgggcugcuggcuU...................................................................  1     1  BRA
......................uccaucaucgggcugcuggc.....................................................................  1     0  BRA
......................uccaucaucgggcugcuggU.....................................................................  1     1  BRA
......................uccaucaucgggcugcuggcugau.................................................................  4     0  BRA
........................caucaucgggcugcuggc.....................................................................  1     0  BRA
```

Mature Star

aucuauggcucuuucaguauaguccaucaucgggcugcuggcugauuuggacaggcgagcugcccagugaagggcuguucuguuagggaagcaucagcagcuaagacucag			
....cgagcugcccagugaagg......................................	1	0	BRA
....cgagcugcccagugaagggcugu.................................	1	0	BRA
....gagcugcccagugGaggg.....................................	1	1	BRA
....gagcugcccagugaaggg.....................................	32	0	BRA
....gagcugcccagugaaggU.....................................	1	1	BRA
....gagcugcccagugaagggc....................................	4	0	BRA
....gagcugcccagugaagggcu...................................	1	0	BRA
....gagcugcccagugaagggcAg..................................	1	1	BRA
....gagcugUccagugaagggcug..................................	1	1	BRA
....gagcugcccagugaagggcuA..................................	29	1	BRA
....gagcugcccagugaagggcCg..................................	8	1	BRA
....gagcugcccagugaagggcug..................................	306	0	BRA
....gagcugcccagugaagggcuC..................................	1	1	BRA
....gagcugcccagugaagggcuU..................................	3	1	BRA
....gagcugcUcagugaagggcug..................................	3	1	BRA
....gagcugcccagugaGgggcug..................................	1	1	BRA
....gagcugcccagugaagggUug..................................	2	1	BRA
....gagcugcccaguAaagggcug..................................	1	1	BRA
....gGgcugcccagugaagggcug..................................	3	1	BRA
....gagcugcccaAugaagggcug..................................	1	1	BRA
....gagcugcccagCgaagggcug..................................	2	1	BRA
....gagcugcccUgugaagggcug..................................	1	1	BRA
....gagcCgcccagugaagggcug..................................	2	1	BRA
....gagcugcccagugaaAggcugu.................................	3	1	BRA
....gagcugcccagugaGgggcugu.................................	2	1	BRA
....gagcugccUagugaagggcugu.................................	3	1	BRA
....gagcugcccagugaagggcugG.................................	2	1	BRA
....gagcugcccagugaagggcugC.................................	32	1	BRA
....gagcugcccagugaagggcugA.................................	9	1	BRA
....gagUugcccagugaagggcugu.................................	1	1	BRA
....gagcugUccagugaagggcugu.................................	1	1	BRA
....gagcugcccGgugaagggcugu.................................	2	1	BRA
....gaUcugcccagugaagggcugu.................................	2	1	BRA
....gagcugccGagugaagggcugu.................................	1	1	BRA
....gagcugcccagugaagggcugu.................................	331	0	BRA
....gagcugcccagugaagggcuCu.................................	1	1	BRA
....gagcugcUcagugaagggcugu.................................	7	1	BRA
....gGgcugcccagugaagggcugu.................................	4	1	BRA
....gagcugcccagugaGgggcuguu................................	1	1	BRA
....gagcugcUcagugaagggcuguu................................	3	1	BRA
....gagcugcccagugaagggcuguA................................	159	1	BRA
....gagcugcAcagugaagggcuguu................................	1	1	BRA
....gagcugcccagugaagggcuguu................................	31	0	BRA
....gagcugccUagugaagggcuguu................................	1	1	BRA
....gagcugUccagugaagggcuguu................................	1	1	BRA
....gagcugcccagugaagggcuguuUu..............................	1	1	BRA
....agcugcccagugaagggcugu.................................	1	0	BRA
....ucuguuagggaagcaucagcag...........	1	0	BRA
aucuauggcucuuucaguaA...	1	1	BA1
aucuaugAcucuuucaguau...	1	1	BA1
aucuauggcucuuucaguaC...	14	1	BA1
aucuauggcucuuucaguau...	60	0	BA1
aucuauggcCcuuucaguau...	1	1	BA1
.ucuauggcucuuucaguaC...	8	1	BA1
.ucuauggcucuuucaguaA...	3	1	BA1
.ucuauggcucuuucaguau...	58	0	BA1
.ucuauggcucuuCcaguau...	1	1	BA1
.ucuauggcucuCucaguau...	1	1	BA1
..cuauggcucuuucaguaG...	3	1	BA1
..cuauggcucuuucaguau...	2	0	BA1
..cuauggcucuuucaguaC...	1	1	BA1
.................uauaguccaucaucgggcugcu..	1	0	BA1
...................Caguccaucaucgggcugcu..	1	1	BA1
...................uaguccaucaucgggcugcugg..	1	0	BA1
...................Caguccaucaucgggcugcugg..	4	1	BA1
...................Gaguccaucaucgggcugcuggc...	1	1	BA1
...................uaguccaucaucgggcugcuggc...	1	0	BA1
...................Caguccaucaucgggcugcuggc...	80	1	BA1
...................uaguccaucaucgggcugcuggcC..	1	1	BA1

Mature Star

aucuauggcucuuucaguauaguccaucaucgggcugcuggcugauuuggacaggcgagcugcccagugaagggcuguucuguuagggaagcaucagcagcuaagacucag			
...................Caguccaucaucgggcugcuggcu..	51	1	BA1
...................Aaguccaucaucgggcugcuggcu..	1	1	BA1
...................uaguccaucaucgggcugcuggcu..	1	0	BA1
....................aguccaucaucgggcugA...	1	1	BA1
....................aguccaucaucgggcugc...	23	0	BA1
....................aguccaucaucggAcugc...	1	1	BA1
....................aguccaucaucgggcugU...	5	1	BA1
....................agAccaucaucgggcugcu..	10	1	BA1
....................aguccaucaAcgggcugcu..	6	1	BA1
....................aguccaucaucgggcuAcu..	15	1	BA1
....................agGccaucaucgggcugcu..	1	1	BA1
....................agucUaucaucgggcugcu..	11	1	BA1
....................aguccaAcaucgggcugcu..	4	1	BA1
....................aguccaucaCcgggcugcu..	24	1	BA1
....................aguccaCcaucgggcugcu..	29	1	BA1
....................aguccaucaGcgggcugcu..	1	1	BA1
....................aguccaucaucgggcugGu..	1	1	BA1
....................aguccaucaucgggcuUcu..	1	1	BA1
....................aguccaucaucgggcGgcu..	3	1	BA1
....................aguccaucaucggAcugcu..	15	1	BA1
....................aguccaucaucgggcugcu..	8805	0	BA1
....................aguccaucaucgUgcugcu..	8	1	BA1
....................aguccUucaucgggcugcu..	3	1	BA1
....................aguccaucaucgggcugAu..	3	1	BA1
....................aguccaucaucgggcugcA..	908	1	BA1
....................aguccaucaucCggcugcu..	2	1	BA1
....................aguccaucaucggCcugcu..	2	1	BA1
....................aguccaucaucgggcugUu..	5	1	BA1
....................aAuccaucaucgggcugcu..	2	1	BA1
....................aguccaucaucggUcugcu..	2	1	BA1
....................aguccaucaucgggcugcC..	1375	1	BA1
....................aguccaucaucUggcugcu..	1	1	BA1
....................aguccGucaucgggcugcu..	26	1	BA1
....................aguccaucaucgggAugcu..	3	1	BA1
....................Cguccaucaucgggcugcu..	1	1	BA1
....................aguccaucaucAggcugcu..	15	1	BA1
....................aguccaucaucgggcCgcu..	21	1	BA1
....................aguUcaucaucgggcugcu..	12	1	BA1
....................Gguccaucaucgggcugcu..	32	1	BA1
....................aguccaucaucgggcAgcu..	6	1	BA1
....................agCccaucaucgggcugcu..	22	1	BA1
....................aguccaucCucgggcugcu..	3	1	BA1
....................aguccauUaucgggcugcu..	8	1	BA1
....................aguccaucauUgggcugcu..	14	1	BA1
....................agucGaucaucgggcugcu..	1	1	BA1
....................aguccaucaucgAgcugcu..	8	1	BA1
....................aguccaucUucgggcugcu..	2	1	BA1
....................Uguccaucaucgggcugcu..	9	1	BA1
....................aguGcaucaucgggcugcu..	4	1	BA1
....................aguAcaucaucgggcugcu..	6	1	BA1
....................aguccaucauAgggcugcu..	1	1	BA1
....................aguccaucaucgggcugcG..	241	1	BA1
....................aguccaucaucgggUugcu..	16	1	BA1
....................aguccaucGucgggcugcu..	16	1	BA1
....................aguccaucaucgggcugcuC...	1	1	BA1
....................aguccaucaucgggcugcCg...	2	1	BA1
....................aguccaucaucUggcugcug...	1	1	BA1
....................aguccaucaucgggcugcug...	439	0	BA1
....................aguccaucaucgggcugcAg...	1	1	BA1
....................aguccaCcaucgggcugcug...	1	1	BA1
....................aguccaucauAgggcugcug...	1	1	BA1
....................aguccaucGucgggcugcug...	1	1	BA1
....................aguccaucaucgggcugcuA...	47	1	BA1
....................aguccaucUucgggcugcug...	1	1	BA1
....................aguccaucaucgggcAgcug...	1	1	BA1
....................Cguccaucaucgggcugcug...	1	1	BA1
....................aguccaucaucgggUugcug...	1	1	BA1
....................aguccGucaucgggcugcug...	2	1	BA1
....................aguccauUaucgggcugcugg..	3	1	BA1
....................aguccaCcaucgggcugcugg..	9	1	BA1

```
                    Mature                                   Star
aucuauggcucuuucaguauaguccaucaucgggcugcuggcugauuuggacaggcgagcugcccagugaagggcuguucuguuagggaagcaucagcagcuaagacucag
....................aguccaucaucgggcugcGgg......................................................................  3       1  BA1
....................aguccaucauUgggcugcugg......................................................................  1       1  BA1
....................aguccUucaucgggcugcugg......................................................................  1       1  BA1
....................aguccaucaucgggcuAcugg......................................................................  2       1  BA1
....................aguccaucaucgggcugcugC......................................................................  42      1  BA1
....................aguccaucCucgggcugcugg......................................................................  1       1  BA1
....................aguccaucaucgggcugcuAg......................................................................  4       1  BA1
....................aUuccaucaucgggcugcugg......................................................................  1       1  BA1
....................aguccaucaAcgggcugcugg......................................................................  3       1  BA1
....................Cguccaucaucgggcugcugg......................................................................  1       1  BA1
....................aguccaucaucgggcugUugg......................................................................  6       1  BA1
....................aguccaucaucggAcugcugg......................................................................  3       1  BA1
....................aguccaucaucgggcugcCgg......................................................................  8       1  BA1
....................aguccaucaucgggcugcugg......................................................................  3243    0  BA1
....................aguccaucauAgggcugcugg......................................................................  1       1  BA1
....................aguccaucaucgggcugcugU......................................................................  50      1  BA1
....................aAuccaucaucgggcugcugg......................................................................  2       1  BA1
....................aguccaucUucgggcugcugg......................................................................  1       1  BA1
....................Uguccaucaucgggcugcugg......................................................................  2       1  BA1
....................aguccaucaucAggcugcugg......................................................................  5       1  BA1
....................aguccaucaucgggcugcAgg......................................................................  3       1  BA1
....................aguccaucGucgggcugcugg......................................................................  7       1  BA1
....................aguccaucaucgggcugcugA......................................................................  325     1  BA1
....................aguccaucaucgggUugcugg......................................................................  9       1  BA1
....................aguccaucaucCggcugcugg......................................................................  1       1  BA1
....................agAccaucaucgggcugcugg......................................................................  4       1  BA1
....................Gguccaucaucgggcugcugg......................................................................  13      1  BA1
....................aguccaucaucgggcAgcugg......................................................................  7       1  BA1
....................aguccaucaucgggcuUcugg......................................................................  1       1  BA1
....................aguUcaucaucgggcugcugg......................................................................  4       1  BA1
....................aguccaucaucgggcugcuCg......................................................................  1       1  BA1
....................agucAaucaucgggcugcugg......................................................................  1       1  BA1
....................aguccaucaucgggcCgcugg......................................................................  5       1  BA1
....................aguccaucaCcgggcugcugg......................................................................  5       1  BA1
....................aguccGucaucgggcugcugg......................................................................  10      1  BA1
....................agCccaucaucgggcugcugg......................................................................  7       1  BA1
....................aguccaucaucgAgcugcugg......................................................................  3       1  BA1
....................agucUaucaucgggcugcugg......................................................................  7       1  BA1
....................aguccaucaucgggcugGuggc.....................................................................  4       1  BA1
....................aguccaucaucCggcugcuggc.....................................................................  13      1  BA1
....................aguccaucaucgggcugcAggc.....................................................................  77      1  BA1
....................aAuccaucaucgggcugcuggc.....................................................................  84      1  BA1
....................aguccaucauGgggcugcuggc.....................................................................  13      1  BA1
....................aguccaucaucgggcugUuggc.....................................................................  112     1  BA1
....................aguccaucaGcgggcugcuggc.....................................................................  12      1  BA1
....................agucAaucaucgggcugcuggc.....................................................................  20      1  BA1
....................aguccaucaucgggcugAuggc.....................................................................  25      1  BA1
....................aguccaucaucgggcugcuggG.....................................................................  417     1  BA1
....................aguccaucaucggAcugcuggc.....................................................................  100     1  BA1
....................aguccaucaucgggcAgcuggc.....................................................................  53      1  BA1
....................aguccaucaucgggcuAcuggc.....................................................................  169     1  BA1
....................aguccaucauAgggcugcuggc.....................................................................  29      1  BA1
....................aguccauAaucgggcugcuggc.....................................................................  14      1  BA1
....................aguccaucaucgggcCgcuggc.....................................................................  310     1  BA1
....................aguccaucaucgAgcugcuggc.....................................................................  248     1  BA1
....................aguccaucaucgggcugcugAc.....................................................................  216     1  BA1
....................aguccaucaucgggcGgcuggc.....................................................................  15      1  BA1
....................agucUaucaucgggcugcuggc.....................................................................  131     1  BA1
....................aguccaucaucgggcugcugUc.....................................................................  32      1  BA1
....................aguccaucaucgggAugcuggc.....................................................................  29      1  BA1
....................aguccaucCucgggcugcuggc.....................................................................  26      1  BA1
....................aguccaGcaucgggcugcuggc.....................................................................  10      1  BA1
....................aguccCucaucgggcugcuggc.....................................................................  24      1  BA1
....................aguccaAcaucgggcugcuggc.....................................................................  57      1  BA1
....................aguccaucaucgggcugcuggc.....................................................................  127865  0  BA1
....................aguccaucaucggUcugcuggc.....................................................................  17      1  BA1
....................aguccaucaucgggcugcuCgc.....................................................................  7       1  BA1
....................aguccaCcaucgggcugcuggc.....................................................................  429     1  BA1
....................aguccUucaucgggcugcuggc.....................................................................  64      1  BA1
....................aguccaucUucgggcugcuggc.....................................................................  38      1  BA1
```

```
                    Mature              Star
aucuauggcucuuucaguauaguccaucaucgggcugcuggcugauuuggacaggcgagcugcccagugaagggcuguucuguuagggaagcaucagcagcuaagacucag
....................aguccaucaucgggUugcuggc.....................................................................  162    1  BA1
....................aguccGucaucgggcugcuggc.....................................................................  319    1  BA1
....................aguGcaucaucgggcugcuggc.....................................................................  5      1  BA1
....................aguccaucaucgUgcugcuggc.....................................................................  14     1  BA1
....................aguccaucaucgCgcugcuggc.....................................................................  4      1  BA1
....................agCccaucaucgggcugcuggc.....................................................................  422    1  BA1
....................aguccaucauUgggcugcuggc.....................................................................  147    1  BA1
....................aguccaucaucUggcugcuggc.....................................................................  40     1  BA1
....................aguUcaucaucgggcugcuggc.....................................................................  97     1  BA1
....................Gguccaucaucgggcugcuggc.....................................................................  420    1  BA1
....................aCuccaucaucgggcugcuggc.....................................................................  2      1  BA1
....................aguccaucaucgggcuUcuggc.....................................................................  18     1  BA1
....................aguccaucaAcgggcugcuggc.....................................................................  30     1  BA1
....................aguAcaucaucgggcugcuggc.....................................................................  23     1  BA1
....................Uguccaucaucgggcugcuggc.....................................................................  116    1  BA1
....................aguccaucaCcgggcugcuggc.....................................................................  341    1  BA1
....................Cguccaucaucgggcugcuggc.....................................................................  34     1  BA1
....................aguccaucaucgggcugcuAgc.....................................................................  148    1  BA1
....................aguccauUaucgggcugcuggc.....................................................................  80     1  BA1
....................agGccaucaucgggcugcuggc.....................................................................  13     1  BA1
....................agucGaucaucgggcugcuggc.....................................................................  8      1  BA1
....................aguccaucaucgggcugcugCc.....................................................................  36     1  BA1
....................aguccaucaucgggcugcGggc.....................................................................  119    1  BA1
....................aguccaucaucgggcugcuggA.....................................................................  9242   1  BA1
....................aguccaucaucgggcugcuggU.....................................................................  33172  1  BA1
....................aguccaucaucgggcuCcuggc.....................................................................  3      1  BA1
....................aUuccaucaucgggcugcuggc.....................................................................  17     1  BA1
....................aguccaucaucggCcugcuggc.....................................................................  12     1  BA1
....................aguccaucaucgggcugcCggc.....................................................................  326    1  BA1
....................aguccaucGucgggcugcuggc.....................................................................  272    1  BA1
....................agAccaucaucgggcugcuggc.....................................................................  39     1  BA1
....................aguccaucaucAggcugcuggc.....................................................................  140    1  BA1
....................aguccaucGucgggcugcuggcu....................................................................  240    1  BA1
....................agAccaucaucgggcugcuggcu....................................................................  42     1  BA1
....................aguccaucaucgggUugcuggcu....................................................................  81     1  BA1
....................aguccaucaucgggcugcGggcu....................................................................  32     1  BA1
....................aguccaucaAcgggcugcuggcu....................................................................  19     1  BA1
....................aguccaucaucgggcuUcuggcu....................................................................  7      1  BA1
....................aguccaucaucgggcugAuggcu....................................................................  15     1  BA1
....................aguccaucaucgggcAgcuggcu....................................................................  53     1  BA1
....................aguccaucaucgggcugcugCcu....................................................................  7      1  BA1
....................agucAaucaucgggcugcuggcu....................................................................  20     1  BA1
....................aguccaucaucgggcCgcuggcu....................................................................  261    1  BA1
....................aAuccaucaucgggcugcuggcu....................................................................  73     1  BA1
....................aguccaucauAgggcugcuggcu....................................................................  24     1  BA1
....................Cguccaucaucgggcugcuggcu....................................................................  42     1  BA1
....................aguccaucCucgggcugcuggcu....................................................................  16     1  BA1
....................aguccaucauUgggcugcuggcu....................................................................  111    1  BA1
....................aguccaucaucgggcugcuggcA....................................................................  12181  1  BA1
....................agCccaucaucgggcugcuggcu....................................................................  281    1  BA1
....................aCuccaucaucgggcugcuggcu....................................................................  6      1  BA1
....................aguccaucaucgggcugcuggGu....................................................................  25     1  BA1
....................aguccaucaucgggcugUuggcu....................................................................  78     1  BA1
....................aguccaucaucgggcugGuggcu....................................................................  1      1  BA1
....................aguccauAaucgggcugcuggcu....................................................................  18     1  BA1
....................aguccaCcaucgggcugcuggcu....................................................................  315    1  BA1
....................aguccaucaucUggcugcuggcu....................................................................  25     1  BA1
....................aguAcaucaucgggcugcuggcu....................................................................  17     1  BA1
....................aguccaucaucgggcugcuCgcu....................................................................  1      1  BA1
....................aguccaucaucggAcugcuggcu....................................................................  72     1  BA1
....................aguccaucaucgggcugcAggcu....................................................................  91     1  BA1
....................aguccaucaucgAgcugcuggcu....................................................................  173    1  BA1
....................aguccaucaucgggcugcuggcG....................................................................  2995   1  BA1
....................Uguccaucaucgggcugcuggcu....................................................................  108    1  BA1
....................aguccaucUucgggcugcuggcu....................................................................  34     1  BA1
....................aguccaucaCcgggcugcuggcu....................................................................  237    1  BA1
....................aguccCucaucgggcugcuggcu....................................................................  23     1  BA1
....................aguccaucaucgggcugcuggUu....................................................................  440    1  BA1
....................aguccaucaucgUgcugcuggcu....................................................................  19     1  BA1
....................aguccauUaucgggcugcuggcu....................................................................  58     1  BA1
```

Mature Star

```
aucuauggcucuuucaguauaguccaucaucgggcugcuggcugauuuggacaggcgagcugcccagugaagggcuguucuguuagggaagcaucagcagcuaagacucag
....................aguccaAcaucgggcugcuggcu....................................................................  43     1  BA1
....................aguccUucaucgggcugcuggcu....................................................................  37     1  BA1
....................agGccaucaucgggcugcuggcu....................................................................  5      1  BA1
....................aguccaucaucgggcugcuggcu....................................................................  95471  0  BA1
....................aguccaGcaucgggcugcuggcu....................................................................  13     1  BA1
....................aguccaucauGgggcugcuggcu....................................................................  5      1  BA1
....................aguccaucaucAggcugcuggcu....................................................................  115    1  BA1
....................aguccaucaucggCcugcuggcu....................................................................  12     1  BA1
....................aguccaucaucgggcGgcuggcu....................................................................  13     1  BA1
....................aUuccaucaucgggcugcuggcu....................................................................  18     1  BA1
....................agucGaucaucgggcugcuggcu....................................................................  3      1  BA1
....................aguccaucaucgggcugcuAgcu....................................................................  84     1  BA1
....................aguccaucaucgggGugcuggcu....................................................................  1      1  BA1
....................aguccaucaucgggcuAcuggcu....................................................................  114    1  BA1
....................agucUaucaucgggcugcuggcu....................................................................  120    1  BA1
....................aguccaucaucCggcugcuggcu....................................................................  4      1  BA1
....................aguccaucaucgggcugcugAcu....................................................................  110    1  BA1
....................aguccaucaucgCgcugcuggcu....................................................................  4      1  BA1
....................aguUcaucaucgggcugcuggcu....................................................................  63     1  BA1
....................aguccaucaucgggcugcuggAu....................................................................  344    1  BA1
....................aguccaucaucgggAugcuggcu....................................................................  22     1  BA1
....................aguccaucaucgggcugcuggcC....................................................................  14087  1  BA1
....................aguccaucaucggUcugcuggcu....................................................................  1      1  BA1
....................aguccGucaucgggcugcuggcu....................................................................  270    1  BA1
....................aguccaucaucgggcugcCggcu....................................................................  252    1  BA1
....................Gguccaucaucgggcugcuggcu....................................................................  364    1  BA1
....................aguccaucaGcgggcugcuggcu....................................................................  4      1  BA1
....................aguGcaucaucgggcugcuggcu....................................................................  5      1  BA1
....................aguccaucaucgggcugcugUcu....................................................................  9      1  BA1
....................aguUcaucaucgggcugcuggcug...................................................................  1      1  BA1
....................aguccaucaucgggcugcuggcuC...................................................................  620    1  BA1
....................aguccaucaucgggcugcuggcuU...................................................................  4330   1  BA1
....................aguccaucaucgggcugcuggcGg...................................................................  4      1  BA1
....................aguccaucaucgggcugcuggcAg...................................................................  11     1  BA1
....................aguccaucaucgggcugcuggcug...................................................................  81     0  BA1
....................aguccaucaucgggcugcuggcuA...................................................................  302    1  BA1
....................aguccaucaucgggcugcuggAug...................................................................  1      1  BA1
....................aguccaucaucAggcugcuggcug...................................................................  2      1  BA1
....................aguccaucaucgggcugcuggcuCa..................................................................  2      1  BA1
....................aguccaucaucgggcugcuggcuAa..................................................................  87     1  BA1
....................aguccaucaucgggcugcuggcuga..................................................................  5      0  BA1
....................aguccaucaucgggcugcuggcuUa..................................................................  14     1  BA1
....................aguccaucaucgggcugcuggcugU..................................................................  4      1  BA1
....................aguccaucaucgggcugcuggcuUau.................................................................  2      1  BA1
....................aguccaucaucgggcugcuggcugaA.................................................................  1      1  BA1
....................aguccaucaucgggcugcuggcugau.................................................................  11     0  BA1
....................aguccaucaucgggcugcuggcugCu.................................................................  1      1  BA1
....................aguccaucaucgggcugcuggcuAau.................................................................  3      1  BA1
....................aguccaucaucgggcugcuggcugauu................................................................  36     0  BA1
....................aguccaucaucgggcugcuggcuUauu................................................................  2      1  BA1
....................aguccaucaucgggcugcuggcugauG................................................................  1      1  BA1
....................aguccaucaucgggcugcuggcugauC................................................................  3      1  BA1
.....................guccaucaucgggcugcu........................................................................  1      0  BA1
.....................guccaucaucgggcugcuggA.....................................................................  2      1  BA1
.....................guccaucaucgggcugUuggc.....................................................................  3      1  BA1
.....................Auccaucaucgggcugcuggc.....................................................................  2      1  BA1
.....................guccaucaucgggcugcuggc.....................................................................  22     0  BA1
.....................guccaucaucgggcugcuggU.....................................................................  6      1  BA1
.....................guccaucaucgggcugcuggcC....................................................................  2      1  BA1
.....................guccaucaucgggcugcuggcu....................................................................  13     0  BA1
.....................guccaucaucgggcugcuggcA....................................................................  3      1  BA1
.....................guccaucaucgggcugcuggcug...................................................................  1      0  BA1
......................uccaucaucgggcugcugg......................................................................  1      0  BA1
......................uccaucaucgggcugcuggc.....................................................................  3      0  BA1
......................uccaucaucgggcugcuggU.....................................................................  1      1  BA1
......................uccaucaucgggcCgcuggc.....................................................................  2      1  BA1
......................uccaucaucgggcugcuggA.....................................................................  1      1  BA1
......................uccaucaucgggcugcuggcA....................................................................  3      1  BA1
......................uccaucaucgggcugcuAgcu....................................................................  1      1  BA1
......................uccaucaucgggcugcuggcu....................................................................  2      0  BA1
```

Mature **Star**

aucuauggcucuuucaguauaguccaucaucgggcugcuggcugauuuggacaggcgagcugcccagugaagggcuguucuguuagggaagcaucagcagcuaagacucag			
......................uccaucaucgggcugcuggcU..	1	1	BA1
......................uccaucaucgggcugcuggcugau...	4	0	BA1
.......................ccaucaucgggcugcuggcC..	1	1	BA1
........................caucaucgggcugcuggA...	4	1	BA1
........................caucaucgggcugcuggU...	4	1	BA1
........................caucaucgggcugcuggc...	23	0	BA1
........................caucaucgggcugcuggcC..	2	1	BA1
........................caucaucgggcuUcuggcu..	1	1	BA1
........................caucaucgggcugcuggcu..	4	0	BA1
........................caucaucgggcugcuggAu..	1	1	BA1
........................caucaucgggcugcuggcuU...	4	1	BA1
.............................ucgggcugcuggcugauuuggacaggU...	1	1	BA1
...............................gggcugcuggcugauuuggac...	1	0	BA1
......................................uggcugauCuggacaggc...	1	1	BA1
....aggcgagcugcccagugaagggcugu.................................	1	0	BA1
....cgagcugcccagugaagA......................................	2	1	BA1
....cgagcugcccagugaagg......................................	4	0	BA1
....cgagcugcccagugaaggg.....................................	1	0	BA1
....cgagcugcccagugaaggA.....................................	2	1	BA1
....cgagcugcccagugaagggcug..................................	7	0	BA1
....cgagcugcccagugaagggcuA..................................	1	1	BA1
....cgagcugcccagugaagggcugu.................................	2	0	BA1
....gagcugcccGgugaaggg.....................................	2	1	BA1
....gagUugcccagugaaggg.....................................	1	1	BA1
....gagcugcccagugUaggg.....................................	1	1	BA1
....gagcugcccagugaUggg.....................................	1	1	BA1
....gGgcugcccagugaaggg.....................................	2	1	BA1
....gagcugcccagugaaggC.....................................	5	1	BA1
....gagcugcccagugaaggg.....................................	230	0	BA1
....gagcugcccagugaaggU.....................................	8	1	BA1
....gagcugcccagCgaaggg.....................................	2	1	BA1
....gagcugcccagugaaggA.....................................	32	1	BA1
....gagcugcccagugaagggc....................................	50	0	BA1
....gagcugUccagugaagggc....................................	2	1	BA1
....gagcugcccagugaaggAc....................................	3	1	BA1
....gagcugcccagugaagggG....................................	1	1	BA1
....gagcugcccagugaagggA....................................	3	1	BA1
....gagcugcccagugaagggU....................................	12	1	BA1
....gagcugccUagugaagggcu...................................	1	1	BA1
....gagcugcccagugaagggcu...................................	27	0	BA1
....gagcugcUcagugaagggcug..................................	18	1	BA1
....gagcugcccagugaagggcAg..................................	3	1	BA1
....gagcugcccaAugaagggcug..................................	3	1	BA1
....gagcugcccagugaagggcuA..................................	345	1	BA1
....gagcugcccagugaagggcug..................................	2682	0	BA1
....gagcugcccagugGagggcug..................................	15	1	BA1
....Uagcugcccagugaagggcug..................................	1	1	BA1
....gagcugcccagugaagggcuC..................................	8	1	BA1
....gagcugcccagAgaagggcug..................................	2	1	BA1
....gagcugcccagugaagggcCg..................................	27	1	BA1
....gagcugcAcagugaagggcug..................................	1	1	BA1
....gagcugcccagugaGgggcug..................................	13	1	BA1
....gagcGgcccagugaagggcug..................................	1	1	BA1
....gagcugcccagugUagggcug..................................	8	1	BA1
....gagAugcccagugaagggcug..................................	1	1	BA1
....gagcugcccagGgaagggcug..................................	1	1	BA1
....gagcugUccagugaagggcug..................................	4	1	BA1
....gaUcugcccagugaagggcug..................................	1	1	BA1
....gagcugcGcagugaagggcug..................................	1	1	BA1
....gagcuAcccagugaagggcug..................................	2	1	BA1
....gagcugccUagugaagggcug..................................	18	1	BA1
....gagcugcccGgugaagggcug..................................	8	1	BA1
....gagcugcccagugaCgggcug..................................	1	1	BA1
....gagUugcccagugaagggcug..................................	6	1	BA1
....gagcugcccUgugaagggcug..................................	3	1	BA1
....gagcugccGagugaagggcug..................................	1	1	BA1
....gagcugcccagugaagggcGg..................................	5	1	BA1
....gagcugcccagugaagggUug..................................	10	1	BA1
....gagcCgcccagugaagggcug..................................	4	1	BA1
....gagcugcccaguAaagggcug..................................	1	1	BA1

Mature **Star**

```
aucuauggcucuuucaguauaguccaucaucgggcugcuggcugauuuggacaggcgagcugcccagugaagggcuguucuguuagggaagcaucagcagcuaagacucag
........................................................gagcugcccagugaaggAcug..................................  4     1  BA1
........................................................gagcugcccagugaagAgcug..................................  3     1  BA1
........................................................gagcugAccagugaagggcug..................................  2     1  BA1
........................................................gaAcugcccagugaagggcug..................................  4     1  BA1
........................................................gGgcugcccagugaagggcug..................................  9     1  BA1
........................................................gagcugcccagugaaAggcug..................................  3     1  BA1
........................................................gagcugcccagCgaagggcug..................................  6     1  BA1
........................................................gagcAgcccagugaagggcug..................................  2     1  BA1
........................................................gagcugcccagugaUgggcug..................................  1     1  BA1
........................................................gagcugcccagugaagggcuU..................................  61    1  BA1
........................................................gagcugcccagugaagggcugA.................................  85    1  BA1
........................................................gagcugcccagugaagggcugG.................................  26    1  BA1
........................................................gagcugcUcagugaagggcugu.................................  34    1  BA1
........................................................gagcugcccagugaaggAcugu.................................  14    1  BA1
........................................................gagcugcGcagugaagggcugu.................................  3     1  BA1
........................................................gaAcugcccagugaagggcugu.................................  1     1  BA1
........................................................gagcugcccagugaagggcuCu.................................  1     1  BA1
........................................................gagcugcccagugaagAgcugu.................................  3     1  BA1
........................................................gagcugcccagugaagggcGgu.................................  1     1  BA1
........................................................gagcuUcccagugaagggcugu.................................  1     1  BA1
........................................................gagcugcccGgugaagggcugu.................................  2     1  BA1
........................................................gagcugcccagugGagggcugu.................................  12    1  BA1
........................................................gagcugcccagAgaagggcugu.................................  2     1  BA1
........................................................gagUugcccagugaagggcugu.................................  1     1  BA1
........................................................gagcugcccagugaagggUugu.................................  4     1  BA1
........................................................gagcugcccUgugaagggcugu.................................  2     1  BA1
........................................................gagcugcccaguAaagggcugu.................................  1     1  BA1
........................................................gagcugcAcagugaagggcugu.................................  7     1  BA1
........................................................gagcugcccagugaUgggcugu.................................  2     1  BA1
........................................................gGgcugcccagugaagggcugu.................................  13    1  BA1
........................................................gagcugcccagugaagggcuUu.................................  2     1  BA1
........................................................gagcugcccagugaagggcuAu.................................  5     1  BA1
........................................................gagcugcccagugaaggCcugu.................................  1     1  BA1
........................................................gagcugcccagCgaagggcugu.................................  6     1  BA1
........................................................gagcugcccagugaGgggcugu.................................  10    1  BA1
........................................................gagcugcccagugaagggcCgu.................................  9     1  BA1
........................................................gagcugcccagugaagggcAgu.................................  1     1  BA1
........................................................gagcugcccaAugaagggcugu.................................  3     1  BA1
........................................................gagcugAccagugaagggcugu.................................  4     1  BA1
........................................................gagcCgcccagugaagggcugu.................................  9     1  BA1
........................................................gagcugcccagugaagggcugu.................................  2638  0  BA1
........................................................gagcuCcccagugaagggcugu.................................  2     1  BA1
........................................................gagcugGccagugaagggcugu.................................  1     1  BA1
........................................................gagcuAcccagugaagggcugu.................................  6     1  BA1
........................................................gUgcugcccagugaagggcugu.................................  2     1  BA1
........................................................gagcugccUagugaagggcugu.................................  41    1  BA1
........................................................gagcugcccagugaagggcugC.................................  371   1  BA1
........................................................gagcugcccagugaaAggcugu.................................  3     1  BA1
........................................................gagcugUccagugaagggcugu.................................  14    1  BA1
........................................................gagcugcccagugaagggcuguG................................  19    1  BA1
........................................................gGgcugcccagugaagggcuguu................................  1     1  BA1
........................................................gaAcugcccagugaagggcuguu................................  1     1  BA1
........................................................gagcugcccagugaagggcuguu................................  244   0  BA1
........................................................gagcCgcccagugaagggcuguu................................  1     1  BA1
........................................................gagcugcUcagugaagggcuguu................................  9     1  BA1
........................................................gagcugcccagugCagggcuguu................................  1     1  BA1
........................................................gagcugcccagCgaagggcuguu................................  1     1  BA1
........................................................gagcugcccagugaagggcuUuu................................  1     1  BA1
........................................................gagcugccUagugaagggcuguu................................  5     1  BA1
........................................................gagcugcAcagugaagggcuguu................................  3     1  BA1
........................................................gagcugcccagugaagggcuguA................................  963   1  BA1
........................................................gagcugcccagugaagggcuguuU...............................  1     1  BA1
........................................................gagcugcccagugaagggcuguuUu..............................  3     1  BA1
........................................................gagcugcccagugaagggcuguAcu..............................  3     1  BA1
.........................................................agcugcccagugaagggc....................................  1     0  BA1
............................................................ugcccagugaagggcugu.................................  1     0  BA1
............................................................ugGccagugaagggcuguu................................  1     1  BA1
..............................................................cccagugaagggcuguuU...............................  1     1  BA1
..............................................................................ucuguuagggaagcaucagU.............  1     1  BA1
..............................................................................ucuguuagggaagcaucagc.............  1     0  BA1
```

Mature **Star**

```
aucuauggcucuuucaguauaguccaucaucgggcugcuggcugauuuggacaggcgagcugcccagugaagggcuguucuguuagggaagcaucagcagcuaagacucag
.......................................................................................gaagcaucagcagcuaagacuca.   1     0   BA1

.ucuauggcucuuucaguau...........................................................................................   1     0   G01
..cuauggcucuuucaguau...........................................................................................   6     0   G01
..................Uuaguccaucaucgggcugc.........................................................................   1     1   G01
..................Uuaguccaucaucgggcugcuggc.....................................................................   5     1   G01
..................Uuaguccaucaucgggcugcuggcu....................................................................   2     1   G01
...................uaguccaucaucgggcugcugg......................................................................   1     0   G01
...................uaguccaucaucgggcugcuggc.....................................................................   9     0   G01
...................uaguccaucaucgggcugcuggcu....................................................................   2     0   G01
....................aguccaucaucgggcugc.........................................................................   1     0   G01
....................aguccaucaucgggcugcu........................................................................   3     0   G01
....................aguccaucaucgggcugcug.......................................................................   1     0   G01
....................aguccaucaucgggcugcugg......................................................................   41    0   G01
....................aguccaucaucgggUugcugg......................................................................   1     1   G01
....................aguccaucaucgggcuUcugg......................................................................   1     1   G01
....................aguccaucaucgggcugcuAgc.....................................................................   1     1   G01
....................Uguccaucaucgggcugcuggc.....................................................................   1     1   G01
....................aguccaucaucgggcugcuggc.....................................................................   1676  0   G01
....................agucUaucaucgggcugcuggc.....................................................................   1     1   G01
....................aguccaucauGgggcugcuggc.....................................................................   1     1   G01
....................aguccaucaucgggcGgcuggc.....................................................................   1     1   G01
....................aguccaucaucgggUugcuggc.....................................................................   1     1   G01
....................aguccaucaucgggcugcugAc.....................................................................   6     1   G01
....................aguccaucaucgggcuUcuggc.....................................................................   2     1   G01
....................aguccaucaucggAcugcuggc.....................................................................   3     1   G01
....................aguccaucaucgggcugcuggU.....................................................................   9     1   G01
....................aguccaucaucgggcugcugCc.....................................................................   6     1   G01
....................aguccaucaucgggGugcuggc.....................................................................   2     1   G01
....................aguccaucaucgggcugcugUc.....................................................................   1     1   G01
....................aguccaucaucgggcugcCggc.....................................................................   2     1   G01
....................aguccaucaucgggcugUuggc.....................................................................   2     1   G01
....................aguccaucaucgggcuAcuggc.....................................................................   1     1   G01
....................aguccaucaGcgggcugcuggc.....................................................................   1     1   G01
....................aguccaucaucgCgcugcuggc.....................................................................   1     1   G01
....................aguccaucaucgggcugcuggcG....................................................................   1     1   G01
....................aguccaucaucgggcuUcuggcu....................................................................   2     1   G01
....................aguccaucaucgggcugcuggUu....................................................................   1     1   G01
....................aguccaucaucgggcugcuggcu....................................................................   198   0   G01
....................aguccaucaucgggcugcuggcA....................................................................   24    1   G01
....................aguccaucaucgggcugcuggcC....................................................................   11    1   G01
....................aguccaucaucgggcugcuggcuU...................................................................   20    1   G01
....................aguccaucaucgggcugcuggcuA...................................................................   2     1   G01
....................aguccaucaucgggcugcuggcuAau.................................................................   1     1   G01
....................aguccaucaucgggcugcuggcugauu................................................................   6     0   G01
.....................guccaucaucgggcugcuggc.....................................................................   1     0   G01
.....................guccaucaucgggcugcuggcu....................................................................   1     0   G01
......................uccaucaucgggcugcuggc.....................................................................   1     0   G01
.......................ccaucaucgggcugcuggc.....................................................................   5     0   G01
........................caucaucgggcugcuggc.....................................................................   3     0   G01
........................................gcugauuuggacaggcgag....................................................   1     0   G01
......................................................gUgagcugcccagugaagggcugu.................................   1     1   G01
.......................................................cgagcugcccagugaagggc....................................   1     0   G01
.......................................................cgagcugcccagugaagggcu...................................   1     0   G01
.......................................................Ugagcugcccagugaagggcu...................................   2     1   G01
.......................................................Ugagcugcccagugaagggcug..................................   24    1   G01
.......................................................cgagcugcccagugaagggCg..................................   1     1   G01
.......................................................cgagcugcccagugaagggcug..................................   16    0   G01
.......................................................Agagcugcccagugaagggcug..................................   1     1   G01
.......................................................Agagcugcccagugaagggcugu.................................   1     1   G01
.......................................................cgagcugcccagugaagggcugu.................................   6     0   G01
.......................................................Ugagcugcccagugaagggcugu.................................   16    1   G01
.......................................................cgagcugcccagugaagggcuguu................................   4     0   G01
.......................................................Ugagcugcccagugaagggcuguu................................   3     1   G01
........................................................gagcugcccagugaaggg.....................................   16    0   G01
........................................................gagcugcccagugaaggU.....................................   1     1   G01
........................................................gagcugccUagugaaggg.....................................   1     1   G01
........................................................gagcugcccagugaaggA.....................................   36    1   G01
........................................................gagcugcccagugGagggc....................................   2     1   G01
........................................................gagcugcccagugaagggc....................................   69    0   G01
```

Mature / Star

```
aucuauggcucuuucaguauaguccaucaucgggcugcuggcugauuuggacaggcgagcugcccagugaagggcuguucuguuagggaagcaucagcagcuaagacucag
........................................................gagcugcccagugaagggA....................................	6	1	G01
........................................................gagcugcUcagugaagggcu...................................	1	1	G01
........................................................gagcuAcccagugaagggcu...................................	1	1	G01
........................................................gagcugcccUgugaagggcu...................................	1	1	G01
........................................................gagcugcccagugaagggUu...................................	1	1	G01
........................................................gagcugcccagugGagggcu...................................	1	1	G01
........................................................gagcugcccagugaagggcC...................................	1	1	G01
........................................................gagcugcccagugaagggcu...................................	124	0	G01
........................................................gagcugcccagugaagggcA...................................	1	1	G01
........................................................gagcugcccagugaagggcGg..................................	1	1	G01
........................................................gUgcugcccagugaagggcug..................................	8	1	G01
........................................................gCgcugcccagugaagggcug..................................	5	1	G01
........................................................gagcugcccagugUagggcug..................................	3	1	G01
........................................................gagcugcccagugCagggcug..................................	1	1	G01
........................................................gagcugcccaUugaagggcug..................................	1	1	G01
........................................................gaCcugcccagugaagggcug..................................	1	1	G01
........................................................gagcugccUagugaagggcug..................................	4	1	G01
........................................................gagUugcccagugaagggcug..................................	3	1	G01
........................................................gagcugcccagugaagggcuA..................................	12	1	G01
........................................................gagcugcccagugaCgggcug..................................	1	1	G01
........................................................gagcAgcccagugaagggcug..................................	1	1	G01
........................................................gagcugAccagugaagggcug..................................	1	1	G01
........................................................gagcuAcccagugaagggcug..................................	7	1	G01
........................................................Uagcugcccagugaagggcug..................................	1	1	G01
........................................................gagcugcccagugGagggcug..................................	2	1	G01
........................................................gagcugcccaguCaagggcug..................................	1	1	G01
........................................................gagcugcccagugaaCggcug..................................	1	1	G01
........................................................gagcugcccUgugaagggcug..................................	3	1	G01
........................................................gagcugGccagugaagggcug..................................	1	1	G01
........................................................gagcugcccagugaGgggcug..................................	11	1	G01
........................................................gagcugcccaguUaagggcug..................................	1	1	G01
........................................................gGgcugcccagugaagggcug..................................	12	1	G01
........................................................gagcugcccagugaagggcCg..................................	2	1	G01
........................................................gagcugcccagugaagggcug..................................	6115	0	G01
........................................................gagcugcccagugaaUggcug..................................	1	1	G01
........................................................gagcugcccagugaagggcuC..................................	5	1	G01
........................................................gagcugUccagugaagggcug..................................	3	1	G01
........................................................gagcugcccagugaagggcuU..................................	17	1	G01
........................................................gagcugcccCgugaagggcug..................................	1	1	G01
........................................................gagcugcccagugaagggcAg..................................	1	1	G01
........................................................gagcugcccagugaaggAcug..................................	8	1	G01
........................................................gagcugcccagugaaggCcug..................................	2	1	G01
........................................................gagcugcccagugaagggUug..................................	6	1	G01
........................................................gagcugcccaUugaagggcugu.................................	1	1	G01
........................................................Uagcugcccagugaagggcugu.................................	1	1	G01
........................................................gagcugcccagugaGgggcugu.................................	2	1	G01
........................................................gagcugUccagugaagggcugu.................................	4	1	G01
........................................................gagcugcccagugaagggcugC.................................	12	1	G01
........................................................gagcugcccaguUaagggcugu.................................	1	1	G01
........................................................gagcugcccagugaaggAcugu.................................	3	1	G01
........................................................gaCcugcccagugaagggcugu.................................	1	1	G01
........................................................gagcugcccagugaagggcuAu.................................	2	1	G01
........................................................gagcugcccagugaagUgcugu.................................	1	1	G01
........................................................gagcugcccagugaagggcugA.................................	14	1	G01
........................................................gagcugcUcagugaagggcugu.................................	2	1	G01
........................................................gagcugcccagugaagggUugu.................................	3	1	G01
........................................................gagcugcccagugaagggcuCu.................................	2	1	G01
........................................................gUgcugcccagugaagggcugu.................................	3	1	G01
........................................................gGgcugcccagugaagggcugu.................................	7	1	G01
........................................................gagcuUcccagugaagggcugu.................................	1	1	G01
........................................................gagcugcccagugCagggcugu.................................	2	1	G01
........................................................gagcugcccagugaUgggcugu.................................	2	1	G01
........................................................gagcugcccagugUagggcugu.................................	5	1	G01
........................................................gagcugcccagugaagggcugu.................................	3251	0	G01
........................................................gagcuAcccagugaagggcugu.................................	1	1	G01
........................................................gagcugcccagugaagggcuUu.................................	1	1	G01
........................................................gagcugcccagugaaUggcugu.................................	1	1	G01
........................................................gagcugcccagugaagggcugG.................................	3	1	G01
........................................................gagcugcccCgugaagggcugu.................................	1	1	G01
........................................................gagcugcccagugaagCgcugu.................................	1	1	G01
```

Mature **Star**

```
aucuauggcucuuucaguauaguccaucaucgggcugcuggcugauuuggacaggcgagcugcccagugaagggcuguucuguuagggaagcaucagcagcuaagacucag
........................................................gagUugcccagugaagggcugu.................................	1	1	G01
........................................................gagcugcccagugaGggcuguu................................	2	1	G01
........................................................gagcugcccagugaagggcuguu................................	392	0	G01
........................................................gagcugcccagugaagggcuguA................................	137	1	G01
........................................................gagcugUccagugaagggcuguu................................	1	1	G01
........................................................gagUugcccagugaagggcuguu................................	1	1	G01
........................................................gGgcugcccagugaagggcuguu................................	2	1	G01
........................................................gagcugcccagugaagggcAguu................................	1	1	G01
........................................................gagcugcccagugaagggcuguuA...............................	1	1	G01
........................................................gagcugcccagugaagggcuguuU...............................	1	1	G01
.........................................................agUugcccagugaagggcug..................................	1	1	G01
.........................................................agcugcccagugaagggcug..................................	1	0	G01
.........................................................agcugcccagugaagggcugu.................................	1	0	G01
..........................................................gcugcccagugaagggcugu.................................	2	0	G01
..........................................................gcugcccagugaagggcuguA................................	1	1	G01
...........................................................cugcccagugaagggcugu.................................	1	0	G01
...........................................................cugcccagugaagggcuguu................................	1	0	G01
............................................................ugcccagugaagggcugu.................................	4	0	G01
............................................................ugcccagugaagggcuguu................................	2	0	G01
............................................................ugcccagugaagggcuguA................................	2	1	G01
.............................................................gcccagugaagggcuguu................................	5	0	G01
................................................................................uguuagggaagcaucagcagcuaag......	1	0	G01

aucuauggcucuuucaguau...........................................................................................	1	0	CJ1
...................Caguccaucaucgggcugcuggc.....................................................................	4	1	CJ1
...................Caguccaucaucgggcugcuggcu....................................................................	1	1	CJ1
....................aguccaucaucgggcugc.........................................................................	1	0	CJ1
....................agAccaucaucgggcugcu........................................................................	1	1	CJ1
....................aguccaucaucgggcugcG........................................................................	8	1	CJ1
....................agucAaucaucgggcugcu........................................................................	1	1	CJ1
....................aguccaAcaucgggcugcu........................................................................	1	1	CJ1
....................aguccaucaCcgggcugcu........................................................................	1	1	CJ1
....................aguccaucaucgggcugcA........................................................................	24	1	CJ1
....................aguccGucaucgggcugcu........................................................................	2	1	CJ1
....................aguccaucaucgggcugcu........................................................................	175	0	CJ1
....................Uguccaucaucgggcugcu........................................................................	1	1	CJ1
....................aguccaucaucgggcugcC........................................................................	24	1	CJ1
....................aguccaucaucgggcuAcug.......................................................................	1	1	CJ1
....................aguccaucaucgggcugcuA.......................................................................	6	1	CJ1
....................aguccaucaucgggcugcug.......................................................................	27	0	CJ1
....................agucUaucaucgggcugcugg......................................................................	2	1	CJ1
....................aguccaucaucgAgcugcugg......................................................................	3	1	CJ1
....................aguccaucaucgggcAgcugg......................................................................	2	1	CJ1
....................aguccaucaucgggcugcugA......................................................................	35	1	CJ1
....................aguccaucaucgggUugcugg......................................................................	1	1	CJ1
....................aguccaucaucgggcCgcugg......................................................................	4	1	CJ1
....................Gguccaucaucgggcugcugg......................................................................	3	1	CJ1
....................aguccaucaucggUcugcugg......................................................................	1	1	CJ1
....................aUuccaucaucgggcugcugg......................................................................	1	1	CJ1
....................aguccaucaucgggcugcCgg......................................................................	1	1	CJ1
....................aguccaucaCcgggcugcugg......................................................................	1	1	CJ1
....................agCccaucaucgggcugcugg......................................................................	2	1	CJ1
....................aguccaucaucgggcugcugU......................................................................	16	1	CJ1
....................aguUcaucaucgggcugcugg......................................................................	1	1	CJ1
....................aguccaucaucgggcugcugg......................................................................	305	0	CJ1
....................aguccUucaucgggcugcugg......................................................................	1	1	CJ1
....................aguccaucaucgggcugcAgg......................................................................	2	1	CJ1
....................aguccaucaucgggcugcugCc.....................................................................	3	1	CJ1
....................aguccaucGucgggcugcuggc.....................................................................	11	1	CJ1
....................aguccaucaCcgggcugcuggc.....................................................................	12	1	CJ1
....................aguccGucaucgggcugcuggc.....................................................................	18	1	CJ1
....................aguccaucaucgggcuAcuggc.....................................................................	4	1	CJ1
....................aguccauGaucgggcugcuggc.....................................................................	2	1	CJ1
....................aguccaucaucgggcAgcuggc.....................................................................	3	1	CJ1
....................aguccaucaucgggcugcuggc.....................................................................	4144	0	CJ1
....................aguccaucaucgggcugcAggc.....................................................................	2	1	CJ1
....................aguccaucaucgUgcugcuggc.....................................................................	1	1	CJ1
....................aguccaucaucgggcugcuggG.....................................................................	8	1	CJ1
....................agGccaucaucgggcugcuggc.....................................................................	6	1	CJ1
....................aguccaucaucgggcugcCggc.....................................................................	10	1	CJ1
```

Mature **Star**

aucuauggcucuuucaguauaguccaucaucgggcugcuggcugauuuggacaggcgagcugcccagugaagggcuguucuguuagggaagcaucagcagcuaagacucag			
....................aguccaucaucgggcugUuggc...	1	1	CJ1
....................Gguccaucaucgggcugcuggc...	13	1	CJ1
....................aguccaucaucAggcugcuggc...	6	1	CJ1
....................aguccaucauAgggcugcuggc...	1	1	CJ1
....................aguccaucaucUggcugcuggc...	1	1	CJ1
....................aguccaucaucgggcuUcuggc...	1	1	CJ1
....................aguccaucaucgggcugAuggc...	1	1	CJ1
....................aguAcaucaucgggcugcuggc...	1	1	CJ1
....................aguUcaucaucgggcugcuggc...	1	1	CJ1
....................aguccaucaucgggUugcuggc...	3	1	CJ1
....................agAccaucaucgggcugcuggc...	15	1	CJ1
....................aguccaucaGcgggcugcuggc...	1	1	CJ1
....................aguccaucaucgggcugcuAgc...	7	1	CJ1
....................aguccaucCucgggcugcuggc...	3	1	CJ1
....................aguccaucaucgggcCgcuggc...	16	1	CJ1
....................aguccaucaucgggcugcuggA...	266	1	CJ1
....................aguccCucaucgggcugcuggc...	1	1	CJ1
....................aguccaucauUgggcugcuggc...	5	1	CJ1
....................agucUaucaucgggcugcuggc...	4	1	CJ1
....................aguccaucaucCggcugcuggc...	1	1	CJ1
....................agCccaucaucgggcugcuggc...	28	1	CJ1
....................aguccaucaucgggcugcGggc...	3	1	CJ1
....................aguccaucaucgCgcugcuggc...	1	1	CJ1
....................aAuccaucaucgggcugcuggc...	5	1	CJ1
....................aguccaCcaucgggcugcuggc...	25	1	CJ1
....................Cguccaucaucgggcugcuggc...	1	1	CJ1
....................aguccaucaucgAgcugcuggc...	17	1	CJ1
....................Uguccaucaucgggcugcuggc...	1	1	CJ1
....................aguccaucaucggAcugcuggc...	10	1	CJ1
....................aguccaucaucgggcugcugAc...	3	1	CJ1
....................aguccaucaucgggcugcuggU...	906	1	CJ1
....................aguccaAcaucgggcugcuggc...	9	1	CJ1
....................aguccauUaucgggcugcuggc...	3	1	CJ1
....................aAuccaucaucgggcugcuggcu..	1	1	CJ1
....................aguccaucaucgggcugcuggcC..	219	1	CJ1
....................aguccUucaucgggcugcuggcu..	2	1	CJ1
....................aguUcaucaucgggcugcuggcu..	1	1	CJ1
....................aguccaucaucgggcAgcuggcu..	1	1	CJ1
....................aguccaucaucgggcugcuggUu..	18	1	CJ1
....................agAccaucaucgggcugcuggcu..	12	1	CJ1
....................agucUaucaucgggcugcuggcu..	2	1	CJ1
....................agucAaucaucgggcugcuggcu..	1	1	CJ1
....................aguccaucaucgggcugcCggcu..	5	1	CJ1
....................aguccaucGucgggcugcuggcu..	1	1	CJ1
....................aguccaucUucgggcugcuggcu..	1	1	CJ1
....................aguccaucaucgggcCgcuggcu..	5	1	CJ1
....................aguccaucaucgggUugcuggcu..	3	1	CJ1
....................aguccaucaucgggcugcugAcu..	1	1	CJ1
....................aguccaucaucgggcugcuggcG..	50	1	CJ1
....................aguccaCcaucgggcugcuggcu..	8	1	CJ1
....................aguAcaucaucgggcugcuggcu..	1	1	CJ1
....................aguccauGaucgggcugcuggcu..	1	1	CJ1
....................aguccaucauUgggcugcuggcu..	4	1	CJ1
....................agCccaucaucgggcugcuggcu..	6	1	CJ1
....................aguccaucaucgggcugUuggcu..	1	1	CJ1
....................Gguccaucaucgggcugcuggcu..	5	1	CJ1
....................aguccaucaucgggcugcuggcA..	211	1	CJ1
....................aguccaucaucUggcugcuggcu..	1	1	CJ1
....................aguccaucaucgAgcugcuggcu..	6	1	CJ1
....................aguccaucaucgggcugcuggGu..	1	1	CJ1
....................aguccauUaucgggcugcuggcu..	2	1	CJ1
....................Uguccaucaucgggcugcuggcu..	1	1	CJ1
....................aUuccaucaucgggcugcuggcu..	1	1	CJ1
....................aguccaucaucgggcugcuAgcu..	1	1	CJ1
....................aguccGucaucgggcugcuggcu..	3	1	CJ1
....................aguccaucaucgggcugcuggcu..	1530	0	CJ1
....................aguccaucaCcgggcugcuggcu..	5	1	CJ1
....................aguccaAcaucgggcugcuggcu..	3	1	CJ1
....................aguccaucaucgggcugcuggAu..	4	1	CJ1
....................aguccCucaucgggcugcuggcu..	2	1	CJ1

```
                    Mature                               Star
aucuauggcucuuucaguauaguccaucaucgggcugcuggcugauuuggacaggcgagcugcccagugaagggcuguucuguuagggaagcaucagcagcuaagacucag
....................aguccaucaucAggcugcuggcu....................................................................   1    1   CJ1
....................aguccaucaucgggcugcuggcuU...................................................................   57   1   CJ1
....................aguccaucaucgggcugcuggcuC...................................................................   10   1   CJ1
....................aguccaucaucgggcugcuggcug...................................................................   3    0   CJ1
....................aguccaucaucgggcugcuggcuA...................................................................   4    1   CJ1
....................aguccaucaucgggcugcuggcuAa..................................................................   1    1   CJ1
....................aguccaucaucgggcugcuggcuga..................................................................   1    0   CJ1
....................aguccaucaucgggcugcuggcugaC.................................................................   1    1   CJ1
....................aguccaucaucgggcugcuggcugau.................................................................   3    0   CJ1
....................aguccaucaucgggcugcuggcCgau.................................................................   1    1   CJ1
....................aguccaucaucgggcugcuggcugauu................................................................   3    0   CJ1
.....................guccaucaucgggcugcuggU.....................................................................   2    1   CJ1
.....................guccaucaucgggcugcuggcu....................................................................   1    0   CJ1
........................caucaucgggcugcuggU.....................................................................   1    1   CJ1
........................................................gagcugcccagugaaggg.....................................   7    0   CJ1
........................................................gagcugcccagugaaggA.....................................   1    1   CJ1
........................................................gagcugcccagugaagggc....................................   5    0   CJ1
........................................................gagcugcccagugaaggAc....................................   1    1   CJ1
........................................................gagcugcccagugaagggA....................................   2    1   CJ1
........................................................gagcugcccagugaagggcu...................................   2    0   CJ1
........................................................gagcugcccaguCaagggcug..................................   1    1   CJ1
........................................................gaUcugcccagugaagggcug..................................   3    1   CJ1
........................................................gagcugcccagugaaggAcug..................................   1    1   CJ1
........................................................gagUugcccagugaagggcug..................................   1    1   CJ1
........................................................gagcCgcccagugaagggcug..................................   1    1   CJ1
........................................................gagcugcccUgugaagggcug..................................   1    1   CJ1
........................................................gGgcugcccagugaagggcug..................................   1    1   CJ1
........................................................gagcugcccGgugaagggcug..................................   1    1   CJ1
........................................................gagcugcccagugaUgggcug..................................   1    1   CJ1
........................................................gaAcugcccagugaagggcug..................................   2    1   CJ1
........................................................gagcugcccagugaGgggcug..................................   1    1   CJ1
........................................................gagcugcccagugaagggcuA..................................   29   1   CJ1
........................................................gagcuAcccagugaagggcug..................................   2    1   CJ1
........................................................gagcugcccagugaagggcuU..................................   16   1   CJ1
........................................................gagcugcUcagugaagggcug..................................   1    1   CJ1
........................................................gagcugcccagugaagggcCg..................................   1    1   CJ1
........................................................gagcugcccagugaagggUug..................................   2    1   CJ1
........................................................gUgcugcccagugaagggcug..................................   1    1   CJ1
........................................................gagcugcccagugaagggcug..................................   360  0   CJ1
........................................................gagcugcccagugGagggcug..................................   1    1   CJ1
........................................................gagcugccUagugaagggcug..................................   3    1   CJ1
........................................................gagcugcccagugaagggcugC.................................   8    1   CJ1
........................................................gUgcugcccagugaagggcugu.................................   1    1   CJ1
........................................................gagcugcccagugaagggcugu.................................   65   0   CJ1
........................................................gagcugcccaAugaagggcugu.................................   1    1   CJ1
........................................................gaUcugcccagugaagggcugu.................................   1    1   CJ1
........................................................gagcugccUagugaagggcugu.................................   1    1   CJ1
........................................................gagcugcccagugaagggcugG.................................   1    1   CJ1
........................................................gagcugcccagugaagggcugA.................................   1    1   CJ1
........................................................gagcugcAcagugaagggcugu.................................   1    1   CJ1
........................................................gagcugcccagugaagggUugu.................................   1    1   CJ1
........................................................gagcugUccagugaagggcugu.................................   1    1   CJ1
........................................................gagcugcccagugCagggcugu.................................   1    1   CJ1
........................................................gagcugcUcagugaagggcugu.................................   2    1   CJ1
........................................................gagcugcccagugaagggcuguA................................   17   1   CJ1
........................................................gagcugcccagugaagggcuguu................................   13   0   CJ1

...................Caguccaucaucgggcugcuggc.....................................................................   2    1   HJ1
....................aguccaucaucgggcCgcu........................................................................   2    1   HJ1
....................agucAaucaucgggcugcu........................................................................   1    1   HJ1
....................aguccaucaucgggcugcu........................................................................   96   0   HJ1
....................aguccaucaucgggcugcA........................................................................   11   1   HJ1
....................aguccGucaucgggcugcu........................................................................   3    1   HJ1
....................aguccaucaucggAcugcu........................................................................   2    1   HJ1
....................aguccaucaucgggcugUu........................................................................   1    1   HJ1
....................aguccaucaucgggcugcC........................................................................   12   1   HJ1
....................aguccaucaucgggcuAcu........................................................................   1    1   HJ1
....................agucAaucaucgggcugcug.......................................................................   1    1   HJ1
....................aguccaucaCcgggcugcug.......................................................................   1    1   HJ1
....................aguccaucaucgggcugcug.......................................................................   34   0   HJ1
```

Mature **Star**

```
aucuauggcucuuucaguauaguccaucaucgggcugcuggcugauuuggacaggcgagcugcccagugaagggcuguucuguuagggaagcaucagcagcuaagacucag
....................aguccaucaucgggcugcuC.......................................................................	1	1	HJ1
....................agCccaucaucgggcugcug.......................................................................	1	1	HJ1
....................aguccaucaucgggcugcuA.......................................................................	3	1	HJ1
....................aguccaucGucgggcugcugg......................................................................	1	1	HJ1
....................aguccGucaucgggcugcugg......................................................................	3	1	HJ1
....................Gguccaucaucgggcugcugg......................................................................	4	1	HJ1
....................aguccaucaucgggcuCcugg......................................................................	2	1	HJ1
....................aguccaCcaucgggcugcugg......................................................................	2	1	HJ1
....................aguccaucaucggAcugcugg......................................................................	2	1	HJ1
....................agucUaucaucgggcugcugg......................................................................	4	1	HJ1
....................aguccaucaCcgggcugcugg......................................................................	1	1	HJ1
....................aguccaucaucgggcuAcugg......................................................................	2	1	HJ1
....................aguccaucauAgggcugcugg......................................................................	1	1	HJ1
....................aguccUucaucgggcugcugg......................................................................	1	1	HJ1
....................aguccaucaucgggcugcuAg......................................................................	3	1	HJ1
....................aguccaucaucgggcugcugU......................................................................	57	1	HJ1
....................aguccaucaucgggcAgcugg......................................................................	2	1	HJ1
....................aguccaucaucgAgcugcugg......................................................................	7	1	HJ1
....................agucAaucaucgggcugcugg......................................................................	6	1	HJ1
....................Uguccaucaucgggcugcugg......................................................................	2	1	HJ1
....................aguUcaucaucgggcugcugg......................................................................	1	1	HJ1
....................aguccaucaucgggcugcGgg......................................................................	2	1	HJ1
....................aguccaucaucgggcugcugA......................................................................	157	1	HJ1
....................agCccaucaucgggcugcugg......................................................................	4	1	HJ1
....................aguccaucaucgggcCgcugg......................................................................	2	1	HJ1
....................aguccaucaucgggcugcCgg......................................................................	2	1	HJ1
....................Cguccaucaucgggcugcugg......................................................................	1	1	HJ1
....................aguccaucaucgggcugcugg......................................................................	1185	0	HJ1
....................aguccaucaucAggcugcugg......................................................................	1	1	HJ1
....................aguccaucaucgggcugcugC......................................................................	14	1	HJ1
....................aUuccaucaucgggcugcugg......................................................................	2	1	HJ1
....................aguccaucaucgggUugcugg......................................................................	1	1	HJ1
....................agucGaucaucgggcugcugg......................................................................	1	1	HJ1
....................aguccaucaucgggcugcAgg......................................................................	2	1	HJ1
....................aguccaucaucggUcugcuggc.....................................................................	6	1	HJ1
....................aguccaucaucgggcugcuggG.....................................................................	29	1	HJ1
....................aguccaucaGcgggcugcuggc.....................................................................	2	1	HJ1
....................aguccaucaucUggcugcuggc.....................................................................	1	1	HJ1
....................agucGaucaucgggcugcuggc.....................................................................	7	1	HJ1
....................aguccaucaucAggcugcuggc.....................................................................	2	1	HJ1
....................aguccaucaucgggcugcuggc.....................................................................	3267	0	HJ1
....................aguccaucaucgggcAgcuggc.....................................................................	5	1	HJ1
....................aguccaucaucgAgcugcuggc.....................................................................	8	1	HJ1
....................aguccaucCucgggcugcuggc.....................................................................	2	1	HJ1
....................aguccaucaucgggAugcuggc.....................................................................	1	1	HJ1
....................aguccaCcaucgggcugcuggc.....................................................................	16	1	HJ1
....................aguccaucGucgggcugcuggc.....................................................................	7	1	HJ1
....................aguccaucauAgggcugcuggc.....................................................................	2	1	HJ1
....................Gguccaucaucgggcugcuggc.....................................................................	9	1	HJ1
....................aguccaucaucggAcugcuggc.....................................................................	10	1	HJ1
....................aguccauAaucgggcugcuggc.....................................................................	1	1	HJ1
....................aguccaucauUgggcugcuggc.....................................................................	5	1	HJ1
....................aUuccaucaucgggcugcuggc.....................................................................	3	1	HJ1
....................aguccaucaucgggcugcugUc.....................................................................	5	1	HJ1
....................aguccaucaucgggcugcugAc.....................................................................	17	1	HJ1
....................aguccaucaucgggUugcuggc.....................................................................	1	1	HJ1
....................agucUaucaucgggcugcuggc.....................................................................	1	1	HJ1
....................Uguccaucaucgggcugcuggc.....................................................................	7	1	HJ1
....................aguccGucaucgggcugcuggc.....................................................................	10	1	HJ1
....................aguccaucaucgggcugcuggU.....................................................................	805	1	HJ1
....................agAccaucaucgggcugcuggc.....................................................................	2	1	HJ1
....................aguccaucaucgggcugcugCc.....................................................................	15	1	HJ1
....................aguccaucaucgggcugcCggc.....................................................................	8	1	HJ1
....................aguccaucaucgggcugUuggc.....................................................................	4	1	HJ1
....................aguccaucaucgggcugcuCgc.....................................................................	9	1	HJ1
....................Cguccaucaucgggcugcuggc.....................................................................	1	1	HJ1
....................agucAaucaucgggcugcuggc.....................................................................	8	1	HJ1
....................aguccauUaucgggcugcuggc.....................................................................	1	1	HJ1
....................aguccaucaucgggcugcGggc.....................................................................	10	1	HJ1
....................aguccaucaAcgggcugcuggc.....................................................................	2	1	HJ1
```

Mature **Star**

```
aucuauggcucuuucaguauaguccaucaucgggcugcuggcugauuuggacaggcgagcugcccagugaagggcuguucuguuagggaagcaucagcagcuaagacucag
....................aguccaucaucgggcGgcuggc.....................................................................	1	1	HJ1
....................aguccaucaucgggcugcuAgc.....................................................................	15	1	HJ1
....................aguccaucaucgggcuAcuggc.....................................................................	2	1	HJ1
....................aguccCucaucgggcugcuggc.....................................................................	1	1	HJ1
....................aguccaucaucgggcugcAggc.....................................................................	2	1	HJ1
....................aguGcaucaucgggcugcuggc.....................................................................	2	1	HJ1
....................aguccaucUucgggcugcuggc.....................................................................	2	1	HJ1
....................aguccaucaCcgggcugcuggc.....................................................................	6	1	HJ1
....................agCccaucaucgggcugcuggc.....................................................................	5	1	HJ1
....................aguccaucaucgggcugcuggA.....................................................................	448	1	HJ1
....................aAuccaucaucgggcugcuggc.....................................................................	7	1	HJ1
....................aguUcaucaucgggcugcuggc.....................................................................	1	1	HJ1
....................aguccaucaucgggcCgcuggc.....................................................................	5	1	HJ1
....................aguccaucaucgggcugcGggcu....................................................................	1	1	HJ1
....................agucGaucaucgggcugcuggcu....................................................................	1	1	HJ1
....................aguccUucaucgggcugcuggcu....................................................................	1	1	HJ1
....................aguccaucaucgggcuAcuggcu....................................................................	3	1	HJ1
....................aguccaucaucAggcugcuggcu....................................................................	1	1	HJ1
....................aguccaucaucgggcugcugCcu....................................................................	1	1	HJ1
....................aguccaucaucgggcugcCggcu....................................................................	3	1	HJ1
....................aguccaucaucgggcugcuggAu....................................................................	25	1	HJ1
....................Gguccaucaucgggcugcuggcu....................................................................	3	1	HJ1
....................aguccaCcaucgggcugcuggcu....................................................................	3	1	HJ1
....................aguccaucaucgggcugUuggcu....................................................................	2	1	HJ1
....................aguccGucaucgggcugcuggcu....................................................................	2	1	HJ1
```
[truncated: 788,504 more chars]
