# Supplementary material for: Horseshoe crab genomes reveal the evolution of genes and microRNAs after three rounds of whole genome duplication
Source: Commun Biol. 2021 Jan 19;4:83. doi: 10.1038/s42003-020-01637-2 (PMC7815833; doi:10.1038/s42003-020-01637-2)
Supplement: Supplementary file 7 — Supplementary Data 4 [file 42003_2020_1637_MOESM7_ESM.pdf]

Provisional ID	:	Sc28yqQ_3523_191
Score total	:	128.5
Score for star read(s)	:	3.9
Score for read counts	:	118.4
Score for mfe	:	1.5
Score for randfold	:	1.6
Score for cons. seed	:	3
Total read count	:	244
Mature read count	:	238
Loop read count	:	0
Star read count	:	6

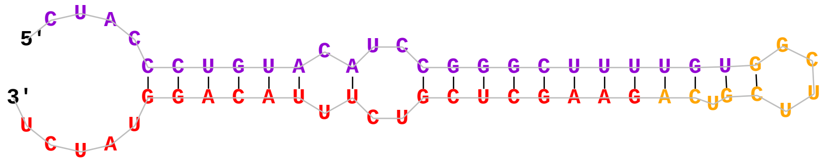

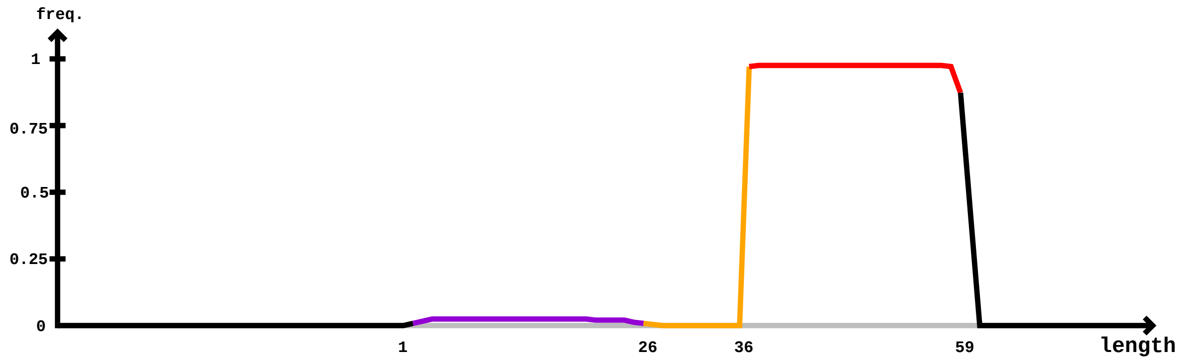


```
                                   Star                              Mature
5'- uccauggaaacgaaaacacccaagccauucguaaucuacccuguacauccgggcuuuuguggcuucgucagaagcucgucuuuacagguaucuugcgaauacggcugauaau  -3'  obs
    uccauggaaacgaaaacacccaagccauucguaaucuacccuguacauccgggcuuuuguggcuucgucagaagcucgucuuuacagguaucuugcgaauacggcugauaau       exp
    ....((....))..........((((((((((((..((((.((((.(..((((((((((((....)).))))))))))..).))))))))..))))))))..))))......   reads  mm  sample
    ....................................uaAccuguacauccgggcuuuugu....................................................   1      1   Le5
    ......................................................................gaagcucgucuuuacagguauc....................   11     0   Le5
    ......................................................................Aaagcucgucuuuacagguaucu...................   2      1   Le5
    ......................................................................gaagcucgucuuuacagguaucA...................   2      1   Le5
    ......................................................................gaagcucgucuuuacagguaAcu...................   1      1   Le5
    ......................................................................gaagGucgucuuuacagguaucu...................   1      1   Le5
    ......................................................................gaagcucgucuuuacagguaucu...................   62     0   Le5
    ......................................................................gaCgcucgucuuuacagguaucu...................   1      1   Le5
    ......................................................................gaagcucgucuCuacagguaucu...................   3      1   Le5
    ......................................................................gaagUucgucuuuacagguaucu...................   1      1   Le5
    ......................................................................gaagcucgucuuuacaggCaucu...................   1      1   Le5
    ......................................................................gaagcucgucuuuCcagguaucu...................   1      1   Le5
    ......................................................................gaagcucgucuuuacagguaucC...................   8      1   Le5

    ...................................cuacccuguaGauccgggc..........................................................   1      1   Le1
    ....................................uacccuguaGauccgggcuuuu......................................................   1      1   Le1
    .....................................acccuguaGauccgggcuuuu......................................................   1      1   Le1
    ......................................................................gaagcucgucuuuacagguauc....................   1      0   Le1
    ......................................................................gaagcucgucuCuacagguaucu...................   1      1   Le1
    ......................................................................gaagGucgucuuuacagguaucu...................   1      1   Le1
    ......................................................................gaagcucgucuuuacagguaucC...................   2      1   Le1
    ......................................................................gaagcucgucuuuacagguaucu...................   11     0   Le1

    .....................................acccuguaGauccgggcuuuug.....................................................   1      1   Br1
    ......................................................................gaagcucgucuuuacagguau.....................   1      0   Br1
    ......................................................................gaagUucgucuuuacagguauc....................   1      1   Br1
    ......................................................................gaagcucgucuuuacagguauc....................   11     0   Br1
    ......................................................................Uaagcucgucuuuacagguaucu...................   1      1   Br1
    ......................................................................gaagcucgucuuuacagguaucA...................   1      1   Br1
    ......................................................................gaagcucgucuuuacagguaucC...................   4      1   Br1
    ......................................................................gaagcucgucuuuacagguaucu...................   29     0   Br1
    ......................................................................gaagcucgucuCuacagguaucu...................   1      1   Br1
    ......................................................................gaagcucgAcuuuacagguaucu...................   1      1   Br1
```

```
                                                                   Star                                 Mature
uccauggaaacgaaaacacccaagccauucguaaucuacccuguacauccgggcuuuuguggcuucgucagaagcucgucuuuacagguaucuugcgaauacggcugauaau
......................................................................gaagcucgucuuuacagguaucuu..................    1    0    Br1
.......................................................................aagcucgucuuuacagguaucu...................    1    0    Br1

......................................................................gaagcucgucuuuacagguaucu...................    2    0    Bl1
......................................................................gaagcucgucuCuacagguaucuu..................    1    1    Bl1

......................................................................gaagcucgucuuuacagguaucC...................    1    1    Ch1
......................................................................gaagcucgucuuuacagguaucA...................    2    1    Ch1
......................................................................gaagcucgucuuuacagguaucu...................    12   0    Ch1
......................................................................AaagcucgucuuuacagguaucU...................    1    1    Ch1
......................................................................gaagcucgucuCuacagguaucu...................    3    1    Ch1
......................................................................gaagGucgucuuuacagguaucu...................    1    1    Ch1
......................................................................gaagcucgucuCuacagguaucuu..................    1    1    Ch1

...................................cuacccuguaGauccgggcuuuugu....................................................    1    1    Ta1
......................................................................gaagcucgucuuuacagguaucu...................    50   0    Ta1
......................................................................gaagUucgucuuuacagguaucu...................    1    1    Ta1
......................................................................gaagGucgucuuuacagguaucu...................    1    1    Ta1
```

Provisional ID	:	Sc28yqQ_3523_342
Score total	:	2221
Score for star read(s)	:	3.9
Score for read counts	:	2210.7
Score for mfe	:	1.7
Score for randfold	:	1.6
Score for cons. seed	:	3
Total read count	:	4348
Mature read count	:	4181
Loop read count	:	0
Star read count	:	167

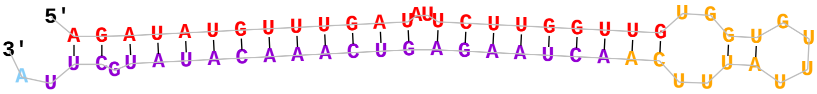

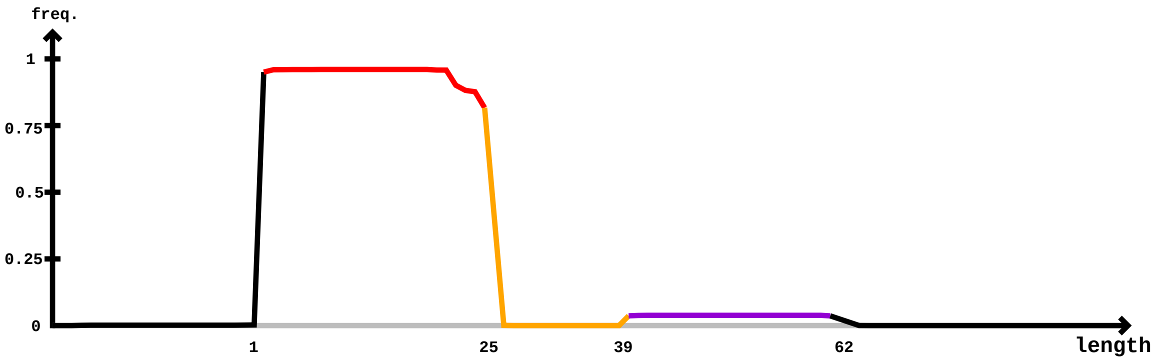


```
                          Mature                            Star
5'- gaaagaaucccuaagcugugagauauguuugauauucuugguuguugguguuuauuucaacuaagagucaaacauaugcuuauagagggggaaaauccugucaagaaaaa  -3'   obs
    gaaagaaucccuaagcugugagauauguuugauauucuugguuguugguguuuauuucaacuaagagucaaacauaugcuuauagagggggaaaauccugucaagaaaaa        exp
    .......(((((...((((((((((((((((..((((((((..((....))..))))))))))))))))))))).)))))))...)))))................  reads   mm   sample
    ....................agauauguuugauauucuug.....................................................................  5       0    Ta1
    ....................agauauguuugauauucuugg....................................................................  4       0    Ta1
    ....................agauauguuugauauucuuggu...................................................................  3       0    Ta1
    ....................agauGuguuugauauucuugguu..................................................................  1       1    Ta1
    ....................Cgauauguuugauauucuugguu..................................................................  1       1    Ta1
    ....................agauauguuugauauucuugguu..................................................................  8       0    Ta1
    ....................agauauguuugauauGcuugguug.................................................................  1       1    Ta1
    ....................Cgauauguuugauauucuugguug.................................................................  8       1    Ta1
    ....................agauauguuugauauucuugguuU.................................................................  9       1    Ta1
    ....................agauauguuugauauucuugguuA.................................................................  2       1    Ta1
    ....................agauGuguuugauauucuugguug.................................................................  6       1    Ta1
    ....................agauauguuugauauucuugguug.................................................................  316     0    Ta1
    ....................agCuauguuugauauucuugguug.................................................................  1       1    Ta1
    ....................Ggauauguuugauauucuugguug.................................................................  1       1    Ta1
    ....................Ugauauguuugauauucuugguug.................................................................  3       1    Ta1
    ....................agaGauguuugauauucuugguug.................................................................  2       1    Ta1
    ....................agauUuguuugauauucuugguug.................................................................  5       1    Ta1
    ....................agauauguuugauauucuugguugu................................................................  10      0    Ta1
    ....................agauauguuugauauucuugguugA................................................................  7       1    Ta1
    .....................gauauguuugCuauucuugguu..................................................................  1       1    Ta1
    .....................gauauguuugauauucuugguu..................................................................  1       0    Ta1
    .....................gauaGguuugauauucuugguug.................................................................  1       1    Ta1
    ..........................................................acuaagagucaaacauaugcuu.............................  11      0    Ta1
    ...........................................................cuaagagucaaacauaugcuuUu...........................  1       1    Ta1

    ..aagaaucccuaagcugug.........................................................................................  1       0    Ch1
    ....................agauauguuugauauucu.......................................................................  1       0    Ch1
    ....................agauauguuugauauucuug.....................................................................  12      0    Ch1
    ....................agauGuguuugauauucuug.....................................................................  1       1    Ch1
    ....................agauauguuugGuauucuug.....................................................................  1       1    Ch1
    ....................agauaGguuugauauucuug.....................................................................  1       1    Ch1
    ....................agauauguuugauauucuugg....................................................................  5       0    Ch1
    ....................Cgauauguuugauauucuugg....................................................................  1       1    Ch1
```

```
                    Mature                                Star
gaaagaaucccuaagcugugagauauguuugauauucuugguugugguguuuauuucaacuaagagucaaacauaugcuuauagagggggaaaauccugucaagaaaaa
....................agauauguuugauauucuuggu...................................................................   6     0   Ch1
....................agauauguuugauauucuugguC..................................................................   2     1   Ch1
....................Ugauauguuugauauucuugguu..................................................................   1     1   Ch1
....................agauauguuugauauucuugguu..................................................................   43    0   Ch1
....................Cgauauguuugauauucuugguu..................................................................   1     1   Ch1
....................agauauguuugauauucuugguug.................................................................   628   0   Ch1
....................agauaugAuugauauucuugguug.................................................................   1     1   Ch1
....................agauauguAugauauucuugguug.................................................................   1     1   Ch1
....................agaAauguuugauauucuugguug.................................................................   2     1   Ch1
....................agauauguuugauauucuuUguug.................................................................   1     1   Ch1
....................agCuauguuugauauucuugguug.................................................................   3     1   Ch1
....................agaGauguuugauauucuugguug.................................................................   6     1   Ch1
....................agauauguuugauaCucuugguug.................................................................   1     1   Ch1
....................agauCuguuugauauucuugguug.................................................................   1     1   Ch1
....................agauGuguuugauauucuugguug.................................................................   13    1   Ch1
....................agauUuguuugauauucuugguug.................................................................   10    1   Ch1
....................Cgauauguuugauauucuugguug.................................................................   7     1   Ch1
....................agauauguuugauauucuugguuC.................................................................   1     1   Ch1
....................agauauguuugauauucuugguuU.................................................................   5     1   Ch1
....................agauauguuugauUuucuugguug.................................................................   1     1   Ch1
....................agauauguuugauauucuugguuA.................................................................   1     1   Ch1
....................Ugauauguuugauauucuugguug.................................................................   7     1   Ch1
....................agauauguuugauauucuugguugA................................................................   24    1   Ch1
....................agauauguuugauauucuugguugu................................................................   23    0   Ch1
....................agauauguuugauauucuugguuUu................................................................   2     1   Ch1
.....................gauauguuugauauucuugguug.................................................................   7     0   Ch1
.....................gauauguuugCuauucuugguug.................................................................   2     1   Ch1
.....................gauauguuAgauauucuugguug.................................................................   1     1   Ch1
..........................................................acuaUgagucaaacauaugcuu.............................   1     1   Ch1
..........................................................acuaagagucaaacauaugcuu.............................   24    0   Ch1
..........................................................acuaagagucaaacauaugcuuU............................   2     1   Ch1
..........................................................acuaagagucaaacauaugcuuaA...........................   2     1   Ch1
..........................................................acuaagagucaaacauaugcuuUu...........................   1     1   Ch1
...........................................................cuaagagucaaacauaugcuu.............................   1     0   Ch1

.aaaUaaucccuaagcugug.........................................................................................   1     1   Bl1
....................agauauguuugauauucu.......................................................................   2     0   Bl1
....................agauauguuugauauucuug.....................................................................   12    0   Bl1
....................agauauguuugauauucuugg....................................................................   7     0   Bl1
....................agauauguuugauauucuuggu...................................................................   1     0   Bl1
....................agauauguuugauauucuugguu..................................................................   30    0   Bl1
....................Cgauauguuugauauucuugguu..................................................................   2     1   Bl1
....................agauauguuugauauucuugguug.................................................................   238   0   Bl1
....................agaCauguuugauauucuugguug.................................................................   2     1   Bl1
....................agauaugAuugauauucuugguug.................................................................   1     1   Bl1
....................agaGauguuugauauucuugguug.................................................................   2     1   Bl1
....................agauaugCuugauauucuugguug.................................................................   1     1   Bl1
....................Cgauauguuugauauucuugguug.................................................................   3     1   Bl1
....................agauauguAugauauucuugguug.................................................................   1     1   Bl1
....................Ngauauguuugauauucuugguug.................................................................   1     1   Bl1
....................Ggauauguuugauauucuugguug.................................................................   1     1   Bl1
....................agauUuguuugauauucuugguug.................................................................   5     1   Bl1
....................agauGuguuugauauucuugguug.................................................................   3     1   Bl1
....................agauauguuugauauucuugguuU.................................................................   2     1   Bl1
....................agauauguuugauUuucuugguug.................................................................   1     1   Bl1
....................agauauguuugGuauucuugguugu................................................................   1     1   Bl1
....................agauauguAugauauucuugguugu................................................................   1     1   Bl1
....................Cgauauguuugauauucuugguugu................................................................   1     1   Bl1
....................agauauguuugauauucuugguugu................................................................   35    0   Bl1
....................agauauguuugauauucuugguugA................................................................   7     1   Bl1
....................agauauguuugauauucuugguugC................................................................   1     1   Bl1
.....................gauauguuugauauucuugguug.................................................................   1     0   Bl1
..........................................................acuaagagucaaacauaugcuu.............................   10    0   Bl1
..........................................................acuaagagucaaacauaugcuC.............................   1     1   Bl1
..........................................................acuaagagucaaacauaugcuua............................   2     0   Bl1

.aaagaaucccuaagcugug.........................................................................................   2     0   He1
..aagaaucccuaagcugug.........................................................................................   1     0   He1
..................Ngagauauguuugauauucuugg....................................................................   1     1   He1
...................Cagauauguuugauauucuugguug.................................................................   2     1   He1
```

```
                    Mature                                Star
gaaagaaucccuaagcugugagauauguuugauauucuugguugugguguuuauuucaacuaagagucaaacauaugcuuauagaggggggaaaauccugucaagaaaaa
....................agauauguuugauauucu........................................................................   3     0   He1
....................agauGuguuugauauucuug......................................................................   1     1   He1
....................Ugauauguuugauauucuug......................................................................   1     1   He1
....................agauUuguuugauauucuug......................................................................   1     1   He1
....................agauauguuugauauucuug......................................................................   106   0   He1
....................agaGauguuugauauucuug......................................................................   1     1   He1
....................agauauguCugauauucuug......................................................................   1     1   He1
....................agauauguuugauCuucuug......................................................................   1     1   He1
....................agauauguuugauauucuugg.....................................................................   24    0   He1
....................agauGuguuugauauucuugg.....................................................................   1     1   He1
....................agauaGguuugauauucuugg.....................................................................   1     1   He1
....................Cgauauguuugauauucuugg.....................................................................   1     1   He1
....................agauauguuugauauucuuggu....................................................................   5     0   He1
....................agauUuguuugauauucuugguu...................................................................   2     1   He1
....................agauauguuugauauucuugguA...................................................................   1     1   He1
....................agauauguuugauauucuugguu...................................................................   54    0   He1
....................agauGuguuugauauucuugguu...................................................................   1     1   He1
....................agauauguuugauauucuugguC...................................................................   1     1   He1
....................agauauguuugauauucuugguG...................................................................   1     1   He1
....................agCuauguuugauauucuugguug..................................................................   4     1   He1
....................agauauguuuAauauucuugguug..................................................................   1     1   He1
....................agauauguuugUuauucuugguug..................................................................   2     1   He1
....................agaGauguuugauauucuugguug..................................................................   6     1   He1
....................agaCauguuugauauucuugguug..................................................................   1     1   He1
....................agauauguuugauauuUuugguug..................................................................   2     1   He1
....................agauauguuugauauucuGgguug..................................................................   1     1   He1
....................agauGuguuugauauucuugguug..................................................................   9     1   He1
....................agauauguuugauauucuugguuC..................................................................   1     1   He1
....................agauauguuugauCuucuugguug..................................................................   2     1   He1
....................agauUuguuugauauucuugguug..................................................................   13    1   He1
....................Ugauauguuugauauucuugguug..................................................................   3     1   He1
....................agauauguuugauauucuuUguug..................................................................   1     1   He1
....................agUuauguuugauauucuugguug..................................................................   1     1   He1
....................agauauguAugauauucuugguug..................................................................   1     1   He1
....................agauauguuugauauucuugguuU..................................................................   1     1   He1
....................Ngauauguuugauauucuugguug..................................................................   3     1   He1
....................agauauguuugauUuucuugguug..................................................................   2     1   He1
....................Cgauauguuugauauucuugguug..................................................................   8     1   He1
....................agauauguuugauauucuugAuug..................................................................   1     1   He1
....................agauauguuugauauucuugguuA..................................................................   5     1   He1
....................agauauguuugauauucuugguug..................................................................   651   0   He1
....................agCuauguuugauauucuugguugu.................................................................   1     1   He1
....................agauauguuugauauucuugguugC.................................................................   6     1   He1
....................agauauguuugauauucuugguugu.................................................................   19    0   He1
....................agauauguuugauauucuugguugA.................................................................   33    1   He1
.....................gauauguuugauauucuug......................................................................   1     0   He1
.....................gauauguuugauauucuugguu...................................................................   1     0   He1
.....................gauUuguuugauauucuugguug..................................................................   1     1   He1
.....................gCuauguuugauauucuugguug..................................................................   1     1   He1
.....................gauaGguuugauauucuugguug..................................................................   1     1   He1
.....................gauauguuugauauucuugguug..................................................................   7     0   He1
.......................uauguuugauauucuugguuU..................................................................   1     1   He1
..........................................................acuaagagucaaacauaugcu...............................   6     0   He1
..........................................................Ccuaagagucaaacauaugcuu..............................   2     1   He1
..........................................................acuaagagucaaacauaugcuC..............................   1     1   He1
..........................................................acuaagagCcaaacauaugcuu..............................   1     1   He1
..........................................................Ucuaagagucaaacauaugcuu..............................   1     1   He1
..........................................................acuaagagucaaacauaugcuu..............................   31    0   He1
..........................................................acuaagagucaaacauaugcuuU.............................   1     1   He1
..........................................................acuaagagucaaacauaugcuua.............................   2     0   He1
..........................................................acuaagagucaaacauaugcuuau............................   1     0   He1
..........................................................acuaagagucaaacauaugcuuaA............................   1     1   He1
..........................................................acuaagagucaaacauaugcuuUu............................   1     1   He1
...........................................................cuaagagucaaacauaugcC...............................   1     1   He1
...........................................................cuaagagucaaacauaugcuu..............................   2     0   He1
............................................................uaagagucaaacauaugcuuaAa...........................   1     1   He1

....................agauauguuuAauauucuug......................................................................   1     1   Br1
....................agaGauguuugauauucuug......................................................................   3     1   Br1
....................agauGuguuugauauucuug......................................................................   1     1   Br1
```

Mature **Star**

```
gaaagaaucccuaagcugugagauauguuugauauucuugguugugguguuuauuucaacuaagagucaaacauaugcuuauagaggggggaaaauccugucaagaaaaa
....................agauauguuCgauauucuug......................................................................   1     1    Br1
....................agauauguuugauauucuug......................................................................   38    0    Br1
....................Ugauauguuugauauucuug......................................................................   3     1    Br1
....................agauauguAugauauucuugg.....................................................................   1     1    Br1
....................agauauguuugauauucuugA.....................................................................   4     1    Br1
....................agauauguuugauauucuugg.....................................................................   13    0    Br1
....................agaGauguuugauauucuuggu....................................................................   1     1    Br1
....................agaGauguuugauauucuugguu...................................................................   1     1    Br1
....................agCuauguuugauauucuugguu...................................................................   1     1    Br1
....................agauauguuugauauucuuggAu...................................................................   1     1    Br1
....................agauauguuugauauucuugguA...................................................................   1     1    Br1
....................agauauguuugauauucuugguu...................................................................   34    0    Br1
....................agauauguuugauauucuugguC...................................................................   1     1    Br1
....................agauauguuugauauucAugguu...................................................................   1     1    Br1
....................agaAauguuugauauucuugguug..................................................................   1     1    Br1
....................Cgauauguuugauauucuugguug..................................................................   2     1    Br1
....................agaGauguuugauauucuugguug..................................................................   1     1    Br1
....................Ugauauguuugauauucuugguug..................................................................   2     1    Br1
....................agauGuguuugauauucuugguug..................................................................   3     1    Br1
....................agauUuguuugauauucuugguug..................................................................   4     1    Br1
....................agCuauguuugauauucuugguug..................................................................   1     1    Br1
....................Ngauauguuugauauucuugguug..................................................................   1     1    Br1
....................agauauguuugauauucuugguug..................................................................   120   0    Br1
....................agauauguuugauauucuugguugC.................................................................   1     1    Br1
....................agauauguuugauauucuugguugu.................................................................   3     0    Br1
....................agauauguuugauauucuugguugA.................................................................   15    1    Br1
.....................gauauguuugauauucuugguug..................................................................   2     0    Br1
.....................gCuauguuugauauucuugguug..................................................................   1     1    Br1
......................aGauguuugauauucuugguug..................................................................   1     1    Br1
..........................................................acuaagagucaaacauaugcuC..............................   2     1    Br1
..........................................................acuaagagucaaCcauaugcuu..............................   1     1    Br1
..........................................................acuaagagucaaacauaugcuu..............................   11    0    Br1
..........................................................acuaagagucaaacauaugcuua.............................   1     0    Br1
..........................................................acuaagagucaaacauaugcuuU.............................   1     1    Br1
..........................................................acuaagagucaaacauaugcuuaA............................   1     1    Br1
...........................................................cuaagagucaaacauaugcuu..............................   1     0    Br1
............................................................uaagagucaaacauaugcuu..............................   1     0    Br1

..................ugagauauguuugauauucu........................................................................   1     0    Le1
....................agauauguuugauauucuu.......................................................................   1     0    Le1
....................agauauguuugauauucuGg......................................................................   1     1    Le1
....................agauauguuugauauucuug......................................................................   20    0    Le1
....................agauUuguuugauauucuug......................................................................   3     1    Le1
....................agauauguuugauauucuugg.....................................................................   5     0    Le1
....................agauauguuugauauucuuggA....................................................................   1     1    Le1
....................agauUuguuugauauucuugguu...................................................................   1     1    Le1
....................agauauguuugauauucuugguu...................................................................   16    0    Le1
....................Ngauauguuugauauucuugguug..................................................................   1     1    Le1
....................agauGuguuugauauucuugguug..................................................................   5     1    Le1
....................agauUuguuugauauucuugguug..................................................................   5     1    Le1
....................agauauguuugauauucuugguuU..................................................................   1     1    Le1
....................Ugauauguuugauauucuugguug..................................................................   2     1    Le1
....................agauauguuugauauucuCgguug..................................................................   1     1    Le1
....................agauauguuugauauucuugguuA..................................................................   1     1    Le1
....................Ggauauguuugauauucuugguug..................................................................   1     1    Le1
....................Cgauauguuugauauucuugguug..................................................................   3     1    Le1
....................agaGauguuugauauucuugguug..................................................................   4     1    Le1
....................agauauguuugauauucuugguug..................................................................   254   0    Le1
....................agauauguuugauauucuugguugC.................................................................   1     1    Le1
....................agauGuguuugauauucuugguugu.................................................................   1     1    Le1
....................agauauguuugauauucuugguugA.................................................................   11    1    Le1
....................agauauguuugauauucuugguugu.................................................................   7     0    Le1
.....................gauauguuugauauucuugguug..................................................................   3     0    Le1
.....................gCuauguuugauauucuugguug..................................................................   1     1    Le1
..........................guuugauauucuugguug..................................................................   1     0    Le1
..........................................................acuaagagucaaacauaugcu...............................   1     0    Le1
..........................................................acuaagagucaaacauaugcuC..............................   2     1    Le1
..........................................................acuaagagucaaacauaugcuu..............................   4     0    Le1
..........................................................acuaagagucaaacauaugcuua.............................   1     0    Le1

.aaagaaucccuaagcugug..........................................................................................   1     0    Le5
```

Mature **Star**

```
gaaagaaucccuaagcugugagauauguuugauauucuugguugugguguuuauuucaacuaagagucaaacauaugcuuauagaggggggaaaauccugucaagaaaaa
....................agauauguuugauauucu........................................................................  2    0  Le5
....................agaGauguuugauauucuug......................................................................  1    1  Le5
....................agauauguuugauauucuug......................................................................  30   0  Le5
....................Ugauauguuugauauucuug......................................................................  1    1  Le5
....................agauUuguuugauauucuugg.....................................................................  1    1  Le5
....................agauauguuugauauucuugg.....................................................................  13   0  Le5
....................agauauguuugauauucuuggu....................................................................  5    0  Le5
....................agauauguuugauauucuugguA...................................................................  1    1  Le5
....................agauauguuugauauucuugguC...................................................................  2    1  Le5
....................agaGauguuugauauucuugguu...................................................................  1    1  Le5
....................agauauguAugauauucuugguu...................................................................  1    1  Le5
....................agauauguuugauauucuugguu...................................................................  44   0  Le5
....................Ugauauguuugauauucuugguu...................................................................  1    1  Le5
....................agauUuguuugauauucuugguu...................................................................  1    1  Le5
....................Cgauauguuugauauucuugguu...................................................................  1    1  Le5
....................agCuauguuugauauucuugguug..................................................................  4    1  Le5
....................agauaugCuugauauucuugguug..................................................................  1    1  Le5
....................agauauguGugauauucuugguug..................................................................  1    1  Le5
....................agauauguAugauauucuugguug..................................................................  1    1  Le5
....................agauauguuugauauucuuUguug..................................................................  2    1  Le5
....................agauauguuugauauucuugguCg..................................................................  1    1  Le5
....................agauGuguuugauauucuugguug..................................................................  7    1  Le5
....................agauauguCugauauucuugguug..................................................................  1    1  Le5
....................Ugauauguuugauauucuugguug..................................................................  2    1  Le5
....................agauauguuugauauucuugguuA..................................................................  1    1  Le5
....................agauauUuuugauauucuugguug..................................................................  1    1  Le5
....................agauauguuugauauucuugguug..................................................................  731  0  Le5
....................agauauguuugauCuucuugguug..................................................................  2    1  Le5
....................agaCauguuugauauucuugguug..................................................................  1    1  Le5
....................agaGauguuugauauucuugguug..................................................................  12   1  Le5
....................agauauguuugauauucuugguuU..................................................................  7    1  Le5
....................agauUuguuugauauucuugguug..................................................................  16   1  Le5
....................aCauauguuugauauucuugguug..................................................................  1    1  Le5
....................Cgauauguuugauauucuugguug..................................................................  13   1  Le5
....................aAauauguuugauauucuugguug..................................................................  1    1  Le5
....................agauauguuugauauucuugguugG.................................................................  1    1  Le5
....................agauauguuugauauucuugguugA.................................................................  23   1  Le5
....................agauauguuugauauucuugguugu.................................................................  35   0  Le5
....................agauauguuugauauucuugguugC.................................................................  1    1  Le5
....................agauauguuugauaAucuugguugu.................................................................  1    1  Le5
....................Ugauauguuugauauucuugguugu.................................................................  1    1  Le5
....................agauauguuugauauucuugguuguA................................................................  2    1  Le5
....................agauauguuugauauucuugguuguC................................................................  1    1  Le5
.....................gauauguuugauauucuugg.....................................................................  1    0  Le5
.....................gauauguuugauauucuuggu....................................................................  1    0  Le5
.....................gauaGguuugauauucuugguug..................................................................  1    1  Le5
.....................gauauguuugauauucuugguug..................................................................  2    0  Le5
.....................gauaGguuugauauucuugguugu.................................................................  1    1  Le5
.......................uauguuugauauucuugguug..................................................................  1    0  Le5
..........................................................acuaagagucaaacauaugcu...............................  1    0  Le5
..........................................................acuaagagucaaacaCaugcuu..............................  1    1  Le5
..........................................................acuaGgagucaaacauaugcuu..............................  1    1  Le5
..........................................................acuaUgagucaaacauaugcuu..............................  1    1  Le5
..........................................................acuaagagucaaacauaugcuu..............................  25   0  Le5
..........................................................acuaagagucaaacauaugcuuU.............................  2    1  Le5
..........................................................acuaagagucaaacauaugcuuUu............................  1    1  Le5
```

Provisional ID	:	Sc28yqQ_3523_735
Score total	:	1587.2
Score for star read(s)	:	3.9
Score for read counts	:	1577
Score for mfe	:	1.7
Score for randfold	:	1.6
Score for cons. seed	:	3
Total read count	:	3105
Mature read count	:	2928
Loop read count	:	0
Star read count	:	177

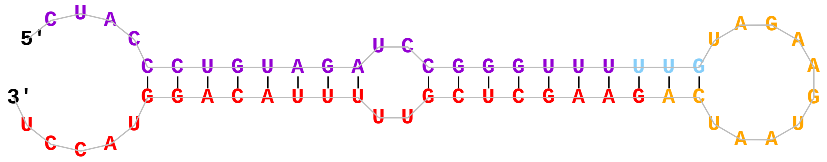

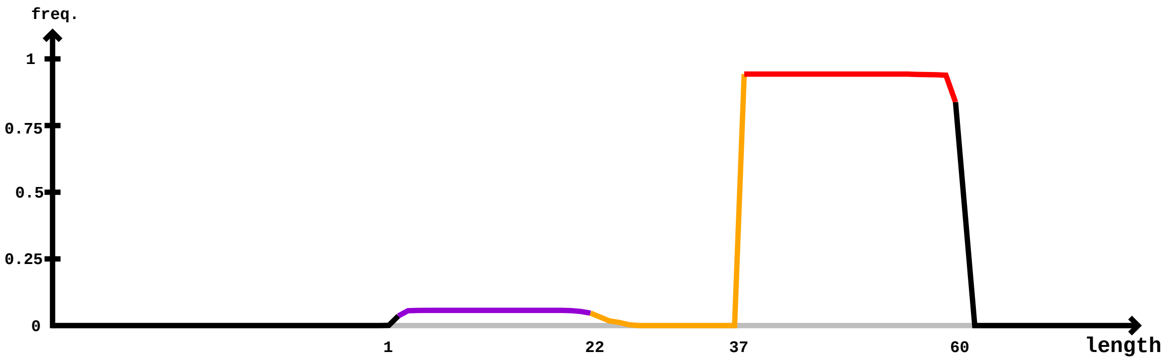

Star **Mature**

```
5'- aucgaaacggagacguaaccgagucauucguaaucuacccuguagauccggguuuuuguagaaguaaucagaagcucguuuuuacagguaccuugcgaauacggcugauug  -3'  obs
    aucgaaacggagacguaaccgagucauucguaaucuacccuguagauccggguuuuuguagaaguaaucagaagcucguuuuuacagguaccuugcgaauacggcugauug       exp
    ......(((....))).....(((((((((((..((((.((((((..((((((((((.........))))))))))..))))))))))..))))))))..)))).....      reads  mm  sample
    .................................ucuacccuguagauccggguu.........................................................  1     0   Le5
    .................................ucuacccuguagauccggguuu........................................................  1     0   Le5
    ..................................cuacccuguagauccggg...........................................................  3     0   Le5
    ..................................cuacccuguagauccgggu..........................................................  4     0   Le5
    ..................................cuacccuguagauccggguu.........................................................  8     0   Le5
    ..................................cuacccuguagauccggguuu........................................................  12    0   Le5
    ..................................Auacccuguagauccggguuuu.......................................................  1     1   Le5
    ..................................cuacccuguagaucGggguuuu.......................................................  1     1   Le5
    ..................................cuacccuguagauccggguuuu.......................................................  10    0   Le5
    ..................................cuacccuguCgauccggguuuu.......................................................  1     1   Le5
    ..................................cuacccuguagauccggguuuuu......................................................  2     0   Le5
    ..................................cuacUcuguagauccggguuuuu......................................................  1     1   Le5
    ..................................cuacccuguagauccggguuuuug.....................................................  6     0   Le5
    ...................................uacccuguagauccgggA..........................................................  1     1   Le5
    ...................................uacccuguagauccggguuu........................................................  7     0   Le5
    ...................................uaUccuguagauccggguuuu.......................................................  1     1   Le5
    ...................................uacccuguagauccggguuuu.......................................................  5     0   Le5
    ...................................uacccuguagauccggguuuuu......................................................  5     0   Le5
    ...................................uaccGuguagauccggguuuuug.....................................................  1     1   Le5
    ...................................uacccuguagauccggguuuuugC....................................................  1     1   Le5
    ...................................uacccuguagauccggguuuuugu....................................................  2     0   Le5
    ......................................................................gaagcucguuuuuacagg.......................  2     0   Le5
    ......................................................................gaagcucguuuuuacagguac....................  4     0   Le5
    ......................................................................gaagcucguuuuuacaAguacc...................  1     1   Le5
    ......................................................................gaaUcucguuuuuacagguacc...................  2     1   Le5
    ......................................................................gaUgcucguuuuuacagguacc...................  1     1   Le5
    ......................................................................gaCgcucguuuuuacagguacc...................  1     1   Le5
    ......................................................................gaagcucguuuuuacagguacA...................  1     1   Le5
    ......................................................................gaagcucguuuuuacagguacU...................  1     1   Le5
    ......................................................................gaagUucguuuuuacagguacc...................  1     1   Le5
    ......................................................................gaagGucguuuuuacagguacc...................  1     1   Le5
    ......................................................................gaagcucguuuuuacagguacc...................  138   0   Le5
    ......................................................................gaagcucguuuuuCcagguacc...................  2     1   Le5
```

Star Mature

```
aucgaaacggagacguaaccgagucauucguaaucuacccuguagauccggguuuuuguagaaguaaaucagaagcucguuuuuacagguaccuugcgaauacggcugauug
.......................................................................gaagcucUuuuuuacagguaccu..................  1    1  Le5
.......................................................................gaagcucguuuuuacagguaccG..................  10   1  Le5
.......................................................................gaCgcucguuuuuacagguaccu..................  2    1  Le5
.......................................................................gaagcucguuAuuacagguaccu..................  2    1  Le5
.......................................................................gaagcucguuuuuacagguacUu..................  1    1  Le5
.......................................................................gaagcucguuuuuacagguaccA..................  283  1  Le5
.......................................................................gaagUucguuuuuacagguaccu..................  7    1  Le5
.......................................................................gaagcucguuuuuCcagguaccu..................  4    1  Le5
.......................................................................gCagcucguuuuuacagguaccu..................  3    1  Le5
.......................................................................gaagcucguuCuuacagguaccu..................  1    1  Le5
.......................................................................gaagcucguuuuuacagguaccu..................  766  0  Le5
.......................................................................gaagcucguuuuuacagguaccC..................  296  1  Le5
.......................................................................gaagcucguuuuuacUgguaccu..................  1    1  Le5
.......................................................................gaagcucgAuuuuacagguaccu..................  1    1  Le5
.......................................................................gaaUcucguuuuuacagguaccu..................  1    1  Le5
.......................................................................Aaagcucguuuuuacagguaccu..................  3    1  Le5
.......................................................................gaagGucguuuuuacagguaccu..................  6    1  Le5
.......................................................................gaagcGcguuuuuacagguaccu..................  1    1  Le5
.......................................................................gUagcucguuuuuacagguaccu..................  1    1  Le5
.......................................................................gaagcucguuuGuacagguaccu..................  1    1  Le5
.......................................................................gGagcucguuuuuacagguaccu..................  1    1  Le5
.......................................................................gaagcucguuuuuacagguaccuC.................  1    1  Le5
.......................................................................gaagcucguuuuuacagguaccuu.................  5    0  Le5

..................................cuacccuguagauccggguuu.........................................................  4    0  Br1
..................................Auacccuguagauccggguuuu........................................................  1    1  Br1
..................................cuacccuguagauccggguuuu........................................................  2    0  Br1
..................................cuacccuguagauccggguuuuug......................................................  1    0  Br1
..................................cuacUcuguagauccggguuuuugu.....................................................  1    1  Br1
...................................uacccuguagauccggguuC.........................................................  1    1  Br1
...................................Nacccuguagauccggguu..........................................................  1    1  Br1
...................................uacccuguagauccggguu..........................................................  2    0  Br1
...................................uacccuguagauccggguuu.........................................................  1    0  Br1
...................................uacccuguagauccggguuuu........................................................  2    0  Br1
...................................uacccuguagauccggguuuuug......................................................  1    0  Br1
....................................acccuguagauccgggCuuuug......................................................  1    1  Br1
.....................................cccuguagauccggguu..........................................................  1    0  Br1
.......................................................................gaCgcucguuuuuacagg.......................  1    1  Br1
.......................................................................gaagcucguuuuuacaggua.....................  1    0  Br1
.......................................................................gaagcucguuuuuacagguac....................  1    0  Br1
.......................................................................gaagcucguuuuuacagguacA...................  1    1  Br1
.......................................................................gaagcucguuuuuacagguacU...................  1    1  Br1
.......................................................................gaagcucguuuuuacagguacc...................  41   0  Br1
.......................................................................gaagcAcguuuuuacagguacc...................  1    1  Br1
.......................................................................gaagcucguuuuuaUagguacc...................  1    1  Br1
.......................................................................gaagcucguuuuuCcagguaccu..................  2    1  Br1
.......................................................................gaagcucguuuuuacagguaccu..................  116  0  Br1
.......................................................................gaagcucguuuuuacagguaccC..................  63   1  Br1
.......................................................................gaagUucguuuuuacagguaccu..................  1    1  Br1
.......................................................................gaagcucguuuuuacagguaccA..................  55   1  Br1
.......................................................................gaagcucguuuuuacagguaccG..................  1    1  Br1
.......................................................................gaagcucguuuuuacagguaccuu.................  5    0  Br1

..................................cuacccuguagauccgggu...........................................................  2    0  Le1
..................................cuacccuguagauccgggC...........................................................  1    1  Le1
..................................cuacccuguagauccggguu..........................................................  2    0  Le1
..................................Uuacccuguagauccggguuu.........................................................  1    1  Le1
..................................cuacccuguagauccggguuu.........................................................  8    0  Le1
..................................Auacccuguagauccggguuuu........................................................  1    1  Le1
..................................cuacccuguagauccggguuuu........................................................  3    0  Le1
..................................cuacccuguagauccggguuuuu.......................................................  1    0  Le1
..................................cuacccuguagauccggguuuuug......................................................  1    0  Le1
...................................uacccuguagauccggguuu.........................................................  2    0  Le1
...................................uacccuguagauccgggCuuuu.......................................................  1    1  Le1
...................................uacccuguagauccggguuuuug......................................................  6    0  Le1
...................................uacccuguagauccggguuuuugA.....................................................  2    1  Le1
...................................uacccuguagauccggguuuuugG.....................................................  1    1  Le1
....................................acccuguagauccgggCuuuu.......................................................  1    1  Le1
.......................................................................gaagcucguuuuuacagg.......................  1    0  Le1
.......................................................................gaagcucguuuuuacaggu......................  2    0  Le1
```

```
                                  Star                                Mature
aucgaaacggagacguaaccgagucauucguaaucuacccuguagauccggguuuuuguagaaguaaucagaagcucguuuuuacagguaccuugcgaauacggcugauug
......................................................................gaagcucguuuuuacaggua.....................	1	0	Le1
......................................................................gaagcucguuuuuacUgguacc...................	1	1	Le1
......................................................................gaCgcucguuuuuacagguacc...................	1	1	Le1
......................................................................gaagcucguuuuuacagguacc...................	75	0	Le1
......................................................................gaagcucguuuuuacagguacA...................	2	1	Le1
......................................................................gaagcucgAuuuuacagguaccu..................	1	1	Le1
......................................................................gaagUucguuuuuacagguaccu..................	4	1	Le1
......................................................................gaagcucguuuuuUcagguaccu..................	1	1	Le1
......................................................................gaagcucguuuuuacagguaccC..................	118	1	Le1
......................................................................gaagcucguuuuuaUagguaccu..................	3	1	Le1
......................................................................gaCgcucguuuuuacagguaccu..................	3	1	Le1
......................................................................Aaagcucguuuuuacagguaccu..................	3	1	Le1
......................................................................gaagcucguuuuuacagguaccG..................	9	1	Le1
......................................................................gaagcucguuuuuacagguaccu..................	349	0	Le1
......................................................................gaagcGcguuuuuacagguaccu..................	2	1	Le1
......................................................................gaagcucguuuuuacagguacUu..................	1	1	Le1
......................................................................gaagGucguuuuuacagguaccu..................	4	1	Le1
......................................................................gaagcucguuuuuCcagguaccu..................	5	1	Le1
......................................................................gaagcucguuuuuacagguaccA..................	182	1	Le1
......................................................................gCagcucguuuuuacagguaccu..................	2	1	Le1
......................................................................gaagcucguuuGuacagguaccu..................	1	1	Le1
......................................................................gaaUcucguuuuuacagguaccu..................	1	1	Le1
......................................................................gaagcucguuuuuacagguaccuu.................	7	0	Le1
......................................................................gCagcucguuuuuacagguaccuu.................	1	1	Le1
......................................................................gaagcucguuuuuacagguaccuC.................	1	1	Le1
......................................................................gaagcucguuuuuacagguaccuA.................	2	1	Le1

......................................................................gaagcucguuuuuacagguacc...................	5	0	Ch1
......................................................................gaagcucguuuuuacagguaccA..................	4	1	Ch1
......................................................................gaagcucguuuuuacagguaccu..................	26	0	Ch1
......................................................................gaagcucguuuuuacagguaccC..................	4	1	Ch1

..................................cuacccuguagauccgggu..........................................................	1	0	He1
..................................cuacccuguagauccgggA..........................................................	1	1	He1
..................................cuacccuguagauccggguu.........................................................	2	0	He1
..................................cuacccuguagauccggguuu........................................................	10	0	He1
..................................cuacccuguagauccggguuuu.......................................................	6	0	He1
..................................cuacccuguagauccggguuuuu......................................................	1	0	He1
..................................cuacccuguagauccggguuuuug.....................................................	4	0	He1
..................................cuacccuguagauccggguuuuCg.....................................................	1	1	He1
..................................cuacccuguagauccggguuuuugu....................................................	1	0	He1
...................................uacccuguagauccgggu..........................................................	1	0	He1
...................................uacccuguagauccggguu.........................................................	5	0	He1
...................................uacccuguagauccggguuu........................................................	4	0	He1
...................................uacccuguagauccggguuuu.......................................................	3	0	He1
...................................uacccuguagauccggguuuuug.....................................................	4	0	He1
...................................uacccuguagauccggguuuuugA....................................................	1	1	He1
....................................acccuguagauccggAuu.........................................................	1	1	He1
......................................................................gaagcucguuuuuacagguacc...................	28	0	He1
......................................................................gaCgcucguuuuuacagguacc...................	1	1	He1
......................................................................gaagUucguuuuuacagguacc...................	1	1	He1
......................................................................gaagcucguuuuuacaAguacc...................	1	1	He1
......................................................................gaagGucguuuuuacagguacc...................	1	1	He1
......................................................................gaagcucguuuuuacagguaccC..................	59	1	He1
......................................................................gaaUcucguuuuuacagguaccu..................	1	1	He1
......................................................................Uaagcucguuuuuacagguaccu..................	1	1	He1
......................................................................gaagGucguuuuuacagguaccu..................	1	1	He1
......................................................................gaagcucguuuuuacagguaccu..................	110	0	He1
......................................................................gaagcucguuuuuacagguaccA..................	23	1	He1
......................................................................gaagcucguuuuuacagguaccG..................	5	1	He1
......................................................................gaagcucguuuuuCcagguaccu..................	1	1	He1
......................................................................gaagUucguuuuuacagguaccu..................	1	1	He1

......................................................................gaagcucguuuuuCcagguacc...................	1	1	Bl1
......................................................................gaagcucguuuuuacagguacc...................	1	0	Bl1
......................................................................gaagcucguuuuuacagguaccA..................	2	1	Bl1
......................................................................gaagcucguuuuuacagguaccC..................	1	1	Bl1
......................................................................gaagcucguuuuuacagguaccu..................	7	0	Bl1

..................................cuacccuguagauccggguu.........................................................	3	0	Ta1
```

```
                                  Star                                 Mature
aucgaaacggagacguaaccgagucauucguaaucuacccuguagauccggguuuuuguagaaguaaucagaagcucguuuuuacagguaccuugcgaauacggcugauug
..................................Auacccuguagauccggguu.........................................................   1    1    Ta1
..................................cuacccuguagauccgggCuuuugu....................................................   1    1    Ta1
......................................................................gaagcucguuuuuacagguacc...................   2    0    Ta1
......................................................................gaagcucguuuuuacagguaccu..................   14   0    Ta1
```

Provisional ID	:	Sc28yqQ_3523_740
Score total	:	4.9
Score for star read(s)	:	-1.3
Score for read counts	:	0
Score for mfe	:	1.6
Score for randfold	:	1.6
Score for cons. seed	:	3
Total read count	:	401
Mature read count	:	401
Loop read count	:	0
Star read count	:	0

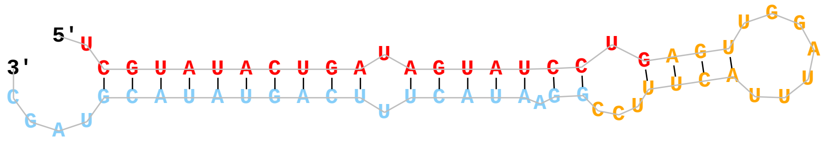

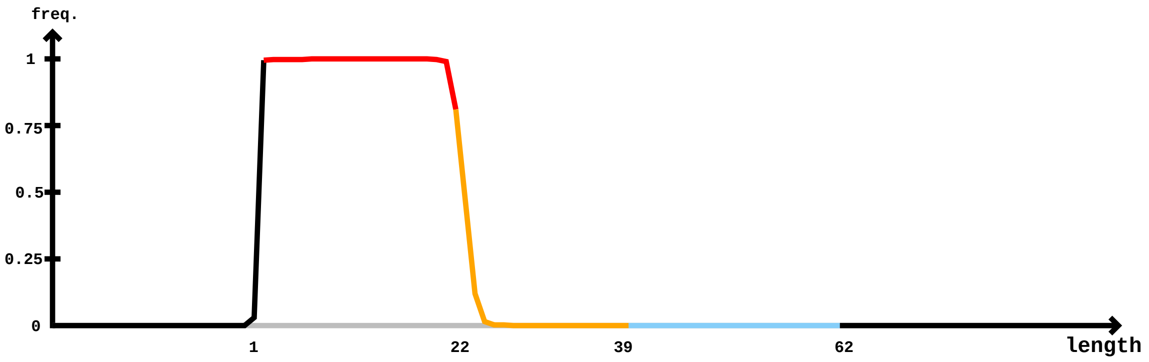

Mature Star

```
5'- guugugucagagacuccugcucguauacugauaguauccugaguuggauuuacuuuccggaauacuuucaguauacguagcagacaacucuguuucucgucgauguuuu   -3'    exp
    ((((((.(((((....((((((((((((((.(((((((.((((.......))))...)).))))).))))))))))).)))))....))))).....)).)))).....   reads      mm        sample
    ...................cucguauacugauaguauccu.....................................................................   1          0         Le5
    ....................ucguauacugauaguauc.......................................................................   1          0         Le5
    ....................ucguauacugauaguauccu.....................................................................   3          0         Le5
    ....................ucguauacugauaguauccug....................................................................   8          0         Le5
    ....................ucCuauacugauaguauccug....................................................................   1          1         Le5
    ....................ucguauacugauaguauccuga...................................................................   7          0         Le5
    ....................ucguauacugaGaguauccuga...................................................................   1          1         Le5

    ....................ucguauacugauaguaucccC....................................................................   1          1         Br1
    ....................ucguauacugauaguauccu.....................................................................   1          0         Br1
    ....................ucguauacugauaguauccug....................................................................   9          0         Br1
    ....................ucguauacugauaguauccuga...................................................................   4          0         Br1
    ....................ucguauacugauaguCuccuga...................................................................   1          1         Br1
    ....................ucguauacugauaguauccugaA..................................................................   2          1         Br1

    ....................ucguauacugauaguaucc......................................................................   1          0         Le1
    ....................ucguauacugauaguauccu.....................................................................   3          0         Le1
    ....................ucguauacugauaguauccug....................................................................   5          0         Le1

    ....................ucguauacugauaguauccu.....................................................................   1          0         Ch1
    ....................ucguauacugauaguauccug....................................................................   4          0         Ch1
    ....................ucguauacugauaguauccugaC..................................................................   1          1         Ch1
    ....................ucguauacugauaguauccugaU..................................................................   1          1         Ch1

    ....................ucguauaUugauaguauccu.....................................................................   1          1         He1
    ....................ucguauacugauaguauccu.....................................................................   4          0         He1
    ....................ucguauacugauaguaucccG....................................................................   2          1         He1
    ....................ucguauacugauaguauccug....................................................................   11         0         He1
    ....................ucguauacugauaguaucccCg...................................................................   1          1         He1
    ....................ucguauacugauagCauccuga...................................................................   2          1         He1
    ....................ucguauacugauaguauccuga...................................................................   4          0         He1
    ....................ucguauacugauaguauccugU...................................................................   1          1         He1
    ....................ucguauacugauaguauccugaC..................................................................   1          1         He1
    ....................ucguauacugauaguauccugagA.................................................................   1          1         He1
```

Mature **Star**

guugugucagagacuccugcucguauacugauaguauccugaguuggauuuacuuuccggaauacuuucaguauacguagcagacaacucuguuucucgucgauguuuu

...................cucguauacugauaguauccu...	2	0	Bl1
...................cucguauacugauaguauccug..	1	0	Bl1
....................ucguauacugauaguauccu...	1	0	Bl1
....................ucguauacugauaguauccA...	1	1	Bl1
....................ucguauacugauagGauccug..	1	1	Bl1
....................ucguauacugauaguauccug..	16	0	Bl1
....................Acguauacugauaguauccug..	1	1	Bl1
....................ucguauacugauaguauccuga...	3	0	Bl1
....................ucguauacugauaguauccugaA..	1	1	Bl1
....................ucguauacugauaguauccugag..	3	0	Bl1
...................cucguauacugauaguauccu...	8	0	Ta1
....................ucguauacugauaguaucc..	2	0	Ta1
....................ucguauacugauaguauccu...	40	0	Ta1
....................ucguGuacugauaguauccu...	2	1	Ta1
....................ucguGuacugauaguauccug..	3	1	Ta1
....................ucgAauacugauaguauccug..	1	1	Ta1
....................ucguUuacugauaguauccug..	2	1	Ta1
....................ucguauacugCuaguauccug..	1	1	Ta1
....................ucguauaUugauaguauccug..	1	1	Ta1
....................ucguauacugauaguauccug..	147	0	Ta1
....................ucguauacugUuaguauccug..	1	1	Ta1
....................ucguauacugauaguauccuga...	39	0	Ta1
....................ucguUuacugauaguauccuga...	1	1	Ta1
....................ucguGuacugauaguauccugag..	1	1	Ta1
....................ucguauacugauaguauccugaA..	5	1	Ta1
....................ucguauacugauaguauccugag..	23	0	Ta1
....................ucguauacugauaguauccugaC..	4	1	Ta1
....................ucguauacugauaguauccugagA...	4	1	Ta1
.....................cguauacugauaguauccu...	1	0	Ta1
.........................uacugauaguauccugaguug...	1	0	Ta1

Provisional ID : Sc28yqQ_3523_818
Score total : 2184.5
Score for star read(s) : 3.9
Score for read counts : 2174
Score for mfe : 1.9
Score for randfold : 1.6
Score for cons. seed : 3
Total read count : 4276
Mature read count : 4179
Loop read count : 0
Star read count : 97

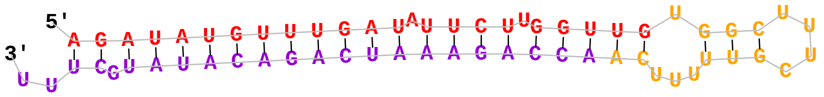

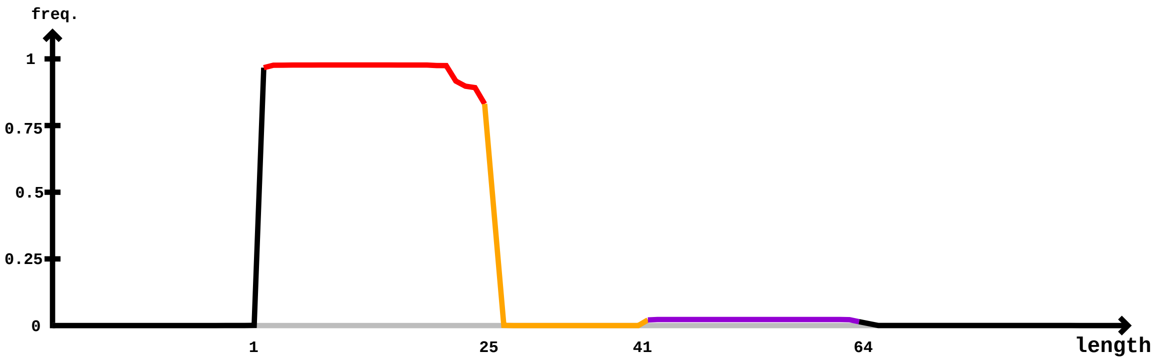


```
                         Mature                                  Star
5'- agauagaauccuuugcugaaagauauguuugauauucuugguuguggcuuucguuuuucaaccagaaaucagacauaugcuuuuagaggaggaguguaucuacugucaga  -3'   obs
    agauagaauccuuugcugaaagauauguuugauauucuugguuguggcuuucguuuuucaaccagaaaucagacauaugcuuuuagaggaggaguguaucuacugucaga         exp
    (((((.(.(((((..((((((((((((((((((.((((.(((((.(((....)))...))))))))))))))))))))).)))))))..))))).).)))))......... reads  mm  sample
    ....................agauauguuugauauucuu....................................................................... 1      0   Le1
    ....................agauauguuugauauucuGg...................................................................... 1      1   Le1
    ....................agauUuguuugauauucuug...................................................................... 3      1   Le1
    ....................agauauguuugauauucuug...................................................................... 20     0   Le1
    ....................agauauguuugauauucuugg..................................................................... 5      0   Le1
    ....................agauauguuugauauucuuggA.................................................................... 1      1   Le1
    ....................agauUuguuugauauucuugguu................................................................... 1      1   Le1
    ....................agauauguuugauauucuugguu................................................................... 16     0   Le1
    ....................agauGuguuugauauucuugguug.................................................................. 5      1   Le1
    ....................agauauguuugauauucuugguug.................................................................. 254    0   Le1
    ....................agaGauguuugauauucuugguug.................................................................. 4      1   Le1
    ....................Cgauauguuugauauucuugguug.................................................................. 3      1   Le1
    ....................agauauguuugauauucuCgguug.................................................................. 1      1   Le1
    ....................agauUuguuugauauucuugguug.................................................................. 5      1   Le1
    ....................Ggauauguuugauauucuugguug.................................................................. 1      1   Le1
    ....................agauauguuugauauucuugguuA.................................................................. 1      1   Le1
    ....................Ngauauguuugauauucuugguug.................................................................. 1      1   Le1
    ....................agauauguuugauauucuugguuU.................................................................. 1      1   Le1
    ....................Ugauauguuugauauucuugguug.................................................................. 2      1   Le1
    ....................agauauguuugauauucuugguugA................................................................. 11     1   Le1
    ....................agauauguuugauauucuugguugC................................................................. 1      1   Le1
    ....................agauGuguuugauauucuugguugu................................................................. 1      1   Le1
    ....................agauauguuugauauucuugguugu................................................................. 7      0   Le1
    .....................gauauguuugauauucuugguug.................................................................. 3      0   Le1
    .....................gCuauguuugauauucuugguug.................................................................. 1      1   Le1
    ..........................guuugauauucuugguug.................................................................. 1      0   Le1
    ............................................................accagaaaucagacauaugcuu............................ 3      0   Le1
    ............................................................accagaaaucagacauaugcuuu........................... 11     0   Le1
    .............................................................ccagaaaucagacauaugcuuu........................... 1      0   Le1

    ....................agauauguuuAauauucuug...................................................................... 1      1   Br1
    ....................agauauguuugauauucuug...................................................................... 38     0   Br1
    ....................agauGuguuugauauucuug...................................................................... 1      1   Br1
```

Mature Star

```
agauagaauccuuugcugaaagauauguuugauauucuugguuguggcuuucguuuuucaaccagaaaucagacauaugcuuuuagaggaggaguguaucuacugucaga
....................Ugauauguuugauauucuug......................................................................   3     1    Br1
....................agauauguuCgauauucuug......................................................................   1     1    Br1
....................agaGauguuugauauucuug......................................................................   3     1    Br1
....................agauauguAugauauucuugg.....................................................................   1     1    Br1
....................agauauguuugauauucuugg.....................................................................   13    0    Br1
....................agauauguuugauauucuugA.....................................................................   4     1    Br1
....................agaGauguuugauauucuuggu....................................................................   1     1    Br1
....................agauauguuugauauucuugguA...................................................................   1     1    Br1
....................agauauguuugauauucuugguC...................................................................   1     1    Br1
....................agCuauguuugauauucuugguu...................................................................   1     1    Br1
....................agaGauguuugauauucuugguu...................................................................   1     1    Br1
....................agauauguuugauauucuuggAu...................................................................   1     1    Br1
....................agauauguuugauauucuugguu...................................................................   34    0    Br1
....................agauauguuugauauucAugguu...................................................................   1     1    Br1
....................Ugauauguuugauauucuugguug..................................................................   2     1    Br1
....................Ngauauguuugauauucuugguug..................................................................   1     1    Br1
....................agCuauguuugauauucuugguug..................................................................   1     1    Br1
....................agauUuguuugauauucuugguug..................................................................   4     1    Br1
....................agauauguuugauauucuugguug..................................................................   120   0    Br1
....................agauGuguuugauauucuugguug..................................................................   3     1    Br1
....................Cgauauguuugauauucuugguug..................................................................   2     1    Br1
....................agaAauguuugauauucuugguug..................................................................   1     1    Br1
....................agaGauguuugauauucuugguug..................................................................   1     1    Br1
....................agauauguuugauauucuugguugu.................................................................   3     0    Br1
....................agauauguuugauauucuugguugC.................................................................   1     1    Br1
....................agauauguuugauauucuugguugA.................................................................   15    1    Br1
.....................gauauguuugauauucuugguug..................................................................   2     0    Br1
.....................gCuauguuugauauucuugguug..................................................................   1     1    Br1
......................aGauguuugauauucuugguug..................................................................   1     1    Br1
............................................................accagaaaucagacauaugcC.............................   1     1    Br1
............................................................accagaaaucagacauaugcuuu...........................   7     0    Br1
.............................................................ccagaaaucagacauaugcuuuu..........................   1     0    Br1

................ugaaagauauguGugaua............................................................................   1     1    Le5
....................agauauguuugauauucu........................................................................   2     0    Le5
....................agauauguuugauauucuug......................................................................   30    0    Le5
....................agaGauguuugauauucuug......................................................................   1     1    Le5
....................Ugauauguuugauauucuug......................................................................   1     1    Le5
....................agauauguuugauauucuugg.....................................................................   13    0    Le5
....................agauUuguuugauauucuugg.....................................................................   1     1    Le5
....................agauauguuugauauucuuggu....................................................................   5     0    Le5
....................agauauguAugauauucuugguu...................................................................   1     1    Le5
....................agaGauguuugauauucuugguu...................................................................   1     1    Le5
....................agauauguuugauauucuugguC...................................................................   2     1    Le5
....................agauUuguuugauauucuugguu...................................................................   1     1    Le5
....................agauauguuugauauucuugguA...................................................................   1     1    Le5
....................Cgauauguuugauauucuugguu...................................................................   1     1    Le5
....................Ugauauguuugauauucuugguu...................................................................   1     1    Le5
....................agauauguuugauauucuugguu...................................................................   44    0    Le5
....................agauauguuugauauucuugguuA..................................................................   1     1    Le5
....................agCuauguuugauauucuugguug..................................................................   4     1    Le5
....................Ugauauguuugauauucuugguug..................................................................   2     1    Le5
....................aAauauguuugauauucuugguug..................................................................   1     1    Le5
....................aCauauguuugauauucuugguug..................................................................   1     1    Le5
....................agauauguuugauauucuugguCg..................................................................   1     1    Le5
....................agauGuguuugauauucuugguug..................................................................   7     1    Le5
....................agauauguCugauauucuugguug..................................................................   1     1    Le5
....................agauauguuugauauucuugguug..................................................................   731   0    Le5
....................agauauguGugauauucuugguug..................................................................   1     1    Le5
....................agauUuguuugauauucuugguug..................................................................   16    1    Le5
....................agaGauguuugauauucuugguug..................................................................   12    1    Le5
....................agauauguuugauCuucuugguug..................................................................   2     1    Le5
....................Cgauauguuugauauucuugguug..................................................................   13    1    Le5
....................agauauguuugauauucuugguuU..................................................................   7     1    Le5
....................agauauguuugauauucuuUguug..................................................................   2     1    Le5
....................agauauUuuugauauucuugguug..................................................................   1     1    Le5
....................agauaugCuugauauucuugguug..................................................................   1     1    Le5
....................agaCauguuugauauucuugguug..................................................................   1     1    Le5
....................agauauguAugauauucuugguug..................................................................   1     1    Le5
....................agauauguuugauaAucuugguugu.................................................................   1     1    Le5
```

```
                    Mature                                  Star
agauagaauccuuugcugaaagauauguuugauauucuugguuguggcuuucguuuuucaaccagaaaucagacauaugcuuuuagaggaggaguguaucuacugucaga
....................agauauguuugauauucuugguugu.................................................................    35   0   Le5
....................agauauguuugauauucuugguugG.................................................................    1    1   Le5
....................Ugauauguuugauauucuugguugu.................................................................    1    1   Le5
....................agauauguuugauauucuugguugC.................................................................    1    1   Le5
....................agauauguuugauauucuugguugA.................................................................    23   1   Le5
....................agauauguuugauauucuugguuguC................................................................    1    1   Le5
....................agauauguuugauauucuugguuguA................................................................    2    1   Le5
.....................gauauguuugauauucuugg.....................................................................    1    0   Le5
.....................gauauguuugauauucuuggu....................................................................    1    0   Le5
.....................gauaGguuugauauucuugguug..................................................................    1    1   Le5
.....................gauauguuugauauucuugguug..................................................................    2    0   Le5
.....................gauaGguuugauauucuugguugu.................................................................    1    1   Le5
.......................uauguuugauauucuugguug..................................................................    1    0   Le5
............................................................accagaaaucagacauaugcA.............................    1    1   Le5
............................................................accagaaaucagacauaugcuu............................    11   0   Le5
............................................................accagaaaucagacauaugcuC............................    1    1   Le5
............................................................accaUaaaucagacauaugcuu............................    2    1   Le5
............................................................accagaaaucagacauaugcuuu...........................    10   0   Le5
.............................................................ccagaaaucagacauaugcuu............................    1    0   Le5
.............................................................ccagaaaucagacauaugcuuu...........................    1    0   Le5
.............................................................ccagUaaucagacauaugcuuu...........................    1    1   Le5

....................agauauguuugauauucuug......................................................................    5    0   Ta1
....................agauauguuugauauucuugg.....................................................................    4    0   Ta1
....................agauauguuugauauucuuggu....................................................................    3    0   Ta1
....................agauauguuugauauucuugguu...................................................................    8    0   Ta1
....................Cgauauguuugauauucuugguu...................................................................    1    1   Ta1
....................agauGuguuugauauucuugguu...................................................................    1    1   Ta1
....................agauGuguuugauauucuugguug..................................................................    6    1   Ta1
....................agauauguuugauauucuugguuA..................................................................    2    1   Ta1
....................Ugauauguuugauauucuugguug..................................................................    3    1   Ta1
....................agauauguuugauauucuugguug..................................................................    316  0   Ta1
....................Cgauauguuugauauucuugguug..................................................................    8    1   Ta1
....................agCuauguuugauauucuugguug..................................................................    1    1   Ta1
....................agauauguuugauauucuugguuU..................................................................    9    1   Ta1
....................Ggauauguuugauauucuugguug..................................................................    1    1   Ta1
....................agauauguuugauauGcuugguug..................................................................    1    1   Ta1
....................agauUuguuugauauucuugguug..................................................................    5    1   Ta1
....................agaGauguuugauauucuugguug..................................................................    2    1   Ta1
....................agauauguuugauauucuugguugA.................................................................    7    1   Ta1
....................agauauguuugauauucuugguugu.................................................................    10   0   Ta1
.....................gauauguuugCuauucuugguu...................................................................    1    1   Ta1
.....................gauauguuugauauucuugguu...................................................................    1    0   Ta1
.....................gauaGguuugauauucuugguug..................................................................    1    1   Ta1

...................Cagauauguuugauauucuugguug..................................................................    2    1   He1
....................agauauguuugauauucu........................................................................    3    0   He1
....................agauauguCugauauucuug......................................................................    1    1   He1
....................agauGuguuugauauucuug......................................................................    1    1   He1
....................agaGauguuugauauucuug......................................................................    1    1   He1
....................Ugauauguuugauauucuug......................................................................    1    1   He1
....................agauauguuugauauucuug......................................................................    106  0   He1
....................agauauguuugauCuucuug......................................................................    1    1   He1
....................agauUuguuugauauucuug......................................................................    1    1   He1
....................agauGuguuugauauucuugg.....................................................................    1    1   He1
....................agauaGguuugauauucuugg.....................................................................    1    1   He1
....................agauauguuugauauucuugg.....................................................................    24   0   He1
....................Cgauauguuugauauucuugg.....................................................................    1    1   He1
....................agauauguuugauauucuuggu....................................................................    5    0   He1
....................agauauguuugauauucuugguG...................................................................    1    1   He1
....................agauUuguuugauauucuugguu...................................................................    2    1   He1
....................agauauguuugauauucuugguu...................................................................    54   0   He1
....................agauGuguuugauauucuugguu...................................................................    1    1   He1
....................agauauguuugauauucuugguC...................................................................    1    1   He1
....................agauauguuugauauucuugguA...................................................................    1    1   He1
....................Ugauauguuugauauucuugguug..................................................................    3    1   He1
....................agauauguuugauauucuugguuC..................................................................    1    1   He1
....................Cgauauguuugauauucuugguug..................................................................    8    1   He1
....................agauauguuugauauucuugguuU..................................................................    1    1   He1
....................agauauguuugauUuucuugguug..................................................................    2    1   He1
```

Mature Star

```
agauagaauccuuugcugaaagauauguuugauauucuugguuguggcuuucguuuuucaaccagaaaucagacauaugcuuuuagaggaggaguguaucuacugucaga
....................agauauguuuAauauucuugguug..................................................................    1     1     He1
....................agauauguuugauauucuuUguug..................................................................    1     1     He1
....................agCuauguuugauauucuugguug..................................................................    4     1     He1
....................agauauguAugauauucuugguug..................................................................    1     1     He1
....................agauauguuugauauucuugguug..................................................................    651   0     He1
....................agauauguuugauauucuugAuug..................................................................    1     1     He1
....................agauGuguuugauauucuugguug..................................................................    9     1     He1
....................agauauguuugauauucuugguuA..................................................................    5     1     He1
....................agaGauguuugauauucuugguug..................................................................    6     1     He1
....................agauauguuugauauucuGgguug..................................................................    1     1     He1
....................agaCauguuugauauucuugguug..................................................................    1     1     He1
....................Ngauauguuugauauucuugguug..................................................................    3     1     He1
....................agauauguuugauauuUuugguug..................................................................    2     1     He1
....................agauUuguuugauauucuugguug..................................................................    13    1     He1
....................agauauguuugauCuucuugguug..................................................................    2     1     He1
....................agUuauguuugauauucuugguug..................................................................    1     1     He1
....................agauauguuugUuauucuugguug..................................................................    2     1     He1
....................agCuauguuugauauucuugguugu.................................................................    1     1     He1
....................agauauguuugauauucuugguugC.................................................................    6     1     He1
....................agauauguuugauauucuugguugu.................................................................    19    0     He1
....................agauauguuugauauucuugguugA.................................................................    33    1     He1
.....................gauauguuugauauucuug......................................................................    1     0     He1
.....................gauauguuugauauucuugguu...................................................................    1     0     He1
.....................gCuauguuugauauucuugguug..................................................................    1     1     He1
.....................gauauguuugauauucuugguug..................................................................    7     0     He1
.....................gauUuguuugauauucuugguug..................................................................    1     1     He1
.....................gauaGguuugauauucuugguug..................................................................    1     1     He1
.......................uauguuugauauucuugguuU..................................................................    1     1     He1
............................................................accaUaaaucagacauaugcuu............................    1     1     He1
............................................................accagaaaucagacauaugcuC............................    1     1     He1
............................................................accagaaaucagacauaugcuu............................    4     0     He1
............................................................accagaaaucagacauaugcuuu...........................    10    0     He1
............................................................Nccagaaaucagacauaugcuuu...........................    1     1     He1
............................................................accagaaaucagacauaugcuuuA..........................    2     1     He1
.............................................................ccagaaaucagacauaugcuu............................    1     0     He1

....................agauauguuugauauucu........................................................................    2     0     Bl1
....................agauauguuugauauucuug......................................................................    12    0     Bl1
....................agauauguuugauauucuugg.....................................................................    7     0     Bl1
....................agauauguuugauauucuuggu....................................................................    1     0     Bl1
....................Cgauauguuugauauucuugguu...................................................................    2     1     Bl1
....................agauauguuugauauucuugguu...................................................................    30    0     Bl1
....................agauauguuugauUuucuugguug..................................................................    1     1     Bl1
....................agauGuguuugauauucuugguug..................................................................    3     1     Bl1
....................agauaugAuugauauucuugguug..................................................................    1     1     Bl1
....................agauaugCuugauauucuugguug..................................................................    1     1     Bl1
....................agaGauguuugauauucuugguug..................................................................    2     1     Bl1
....................agauUuguuugauauucuugguug..................................................................    5     1     Bl1
....................agauauguuugauauucuugguug..................................................................    238   0     Bl1
....................Cgauauguuugauauucuugguug..................................................................    3     1     Bl1
....................agauauguuugauauucuugguuU..................................................................    2     1     Bl1
....................Ggauauguuugauauucuugguug..................................................................    1     1     Bl1
....................agauauguAugauauucuugguug..................................................................    1     1     Bl1
....................agaCauguuugauauucuugguug..................................................................    2     1     Bl1
....................Ngauauguuugauauucuugguug..................................................................    1     1     Bl1
....................agauauguAugauauucuugguugu.................................................................    1     1     Bl1
....................Cgauauguuugauauucuugguugu.................................................................    1     1     Bl1
....................agauauguuugauauucuugguugC.................................................................    1     1     Bl1
....................agauauguuugauauucuugguugA.................................................................    7     1     Bl1
....................agauauguuugauauucuugguugu.................................................................    35    0     Bl1
....................agauauguuugGuauucuugguugu.................................................................    1     1     Bl1
.....................gauauguuugauauucuugguug..................................................................    1     0     Bl1
............................................................accagaaaucagacauaugcC.............................    1     1     Bl1
............................................................accagaaaucagacauaugcuuu...........................    5     0     Bl1
.............................................................ccagaaaucagacauaugcuuu...........................    1     0     Bl1

....................agauauguuugauauucu........................................................................    1     0     Ch1
....................agauauguuugauauucuug......................................................................    12    0     Ch1
....................agauauguuugGuauucuug......................................................................    1     1     Ch1
....................agauGuguuugauauucuug......................................................................    1     1     Ch1
```

```
                    Mature                                      Star
agauagaauccuuugcugaaagauauguuugauauucuugguuguggcuuucguuuuucaaccagaaaucagacauaugcuuuuagaggaggaguguaucuacugucaga
....................agauaGguuugauauucuug......................................................................  1    1  Ch1
....................agauauguuugauauucuugg.....................................................................  5    0  Ch1
....................Cgauauguuugauauucuugg.....................................................................  1    1  Ch1
....................agauauguuugauauucuuggu....................................................................  6    0  Ch1
....................Cgauauguuugauauucuugguu...................................................................  1    1  Ch1
....................agauauguuugauauucuugguu...................................................................  43   0  Ch1
....................agauauguuugauauucuugguC...................................................................  2    1  Ch1
....................Ugauauguuugauauucuugguu...................................................................  1    1  Ch1
....................agauauguuugauauucuugguuU..................................................................  5    1  Ch1
....................agauCuguuugauauucuugguug..................................................................  1    1  Ch1
....................agauauguuugauauucuugguuA..................................................................  1    1  Ch1
....................agauauguuugauUuucuugguug..................................................................  1    1  Ch1
....................agauauguuugauauucuuUguug..................................................................  1    1  Ch1
....................agaGauguuugauauucuugguug..................................................................  6    1  Ch1
....................agauauguuugauauucuugguug..................................................................  628  0  Ch1
....................agauUuguuugauauucuugguug..................................................................  10   1  Ch1
....................agauGuguuugauauucuugguug..................................................................  13   1  Ch1
....................Ugauauguuugauauucuugguug..................................................................  7    1  Ch1
....................agauauguAugauauucuugguug..................................................................  1    1  Ch1
....................Cgauauguuugauauucuugguug..................................................................  7    1  Ch1
....................agauauguuugauaCucuugguug..................................................................  1    1  Ch1
....................agauaugAuugauauucuugguug..................................................................  1    1  Ch1
....................agaAauguuugauauucuugguug..................................................................  2    1  Ch1
....................agCuauguuugauauucuugguug..................................................................  3    1  Ch1
....................agauauguuugauauucuugguuC..................................................................  1    1  Ch1
....................agauauguuugauauucuugguuUu.................................................................  2    1  Ch1
....................agauauguuugauauucuugguugu.................................................................  23   0  Ch1
....................agauauguuugauauucuugguugA.................................................................  24   1  Ch1
.....................gauauguuAgauauucuugguug..................................................................  1    1  Ch1
.....................gauauguuugCuauucuugguug..................................................................  2    1  Ch1
.....................gauauguuugauauucuugguug..................................................................  7    0  Ch1
............................................................accagaaaucagacauaugcuu............................  7    0  Ch1
............................................................accagaaaucagacauaugcuuu...........................  11   0  Ch1
...................................................................................uagaggaggaguguaucuacug.....  1    0  Ch1
```

Provisional ID	:	Sc28yqQ_3523_820
Score total	:	2184.5
Score for star read(s)	:	3.9
Score for read counts	:	2174
Score for mfe	:	1.9
Score for randfold	:	1.6
Score for cons. seed	:	3
Total read count	:	4276
Mature read count	:	4179
Loop read count	:	0
Star read count	:	97

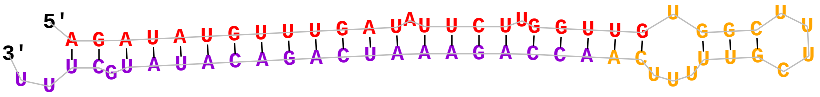

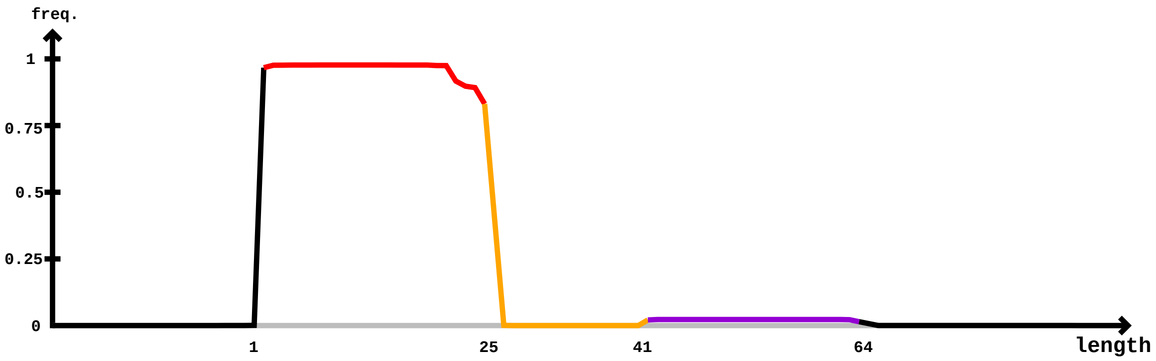

Mature **Star**

```
5'- agauagaauccuuugcugaaagauauguuugauauucuugguuguggcuuucguuuuucaaccagaaaucagacauaugcuuuuagaggaggaguguaucuacugucaga   -3'   obs
    agauagaauccuuugcugaaagauauguuugauauucuugguuguggcuuucguuuuucaaccagaaaucagacauaugcuuuuagaggaggaguguaucuacugucaga         exp
    (((((.(.(((((..((((((((((((((((((.((((.(((((.(((....)))...)))))))))))))))))))).))))))).))))).).))))).........    reads   mm   sample
    ....................agauauguuugauauucuug......................................................................    5       0    Ta1
    ....................agauauguuugauauucuugg.....................................................................    4       0    Ta1
    ....................agauauguuugauauucuuggu....................................................................    3       0    Ta1
    ....................agauauguuugauauucuugguu...................................................................    8       0    Ta1
    ....................Cgauauguuugauauucuugguu...................................................................    1       1    Ta1
    ....................agauGuguuugauauucuugguu...................................................................    1       1    Ta1
    ....................agaGauguuugauauucuugguug..................................................................    2       1    Ta1
    ....................agauUguuugauauucuugguug..................................................................    5       1    Ta1
    ....................Ugauauguuugauauucuugguug..................................................................    3       1    Ta1
    ....................Ggauauguuugauauucuugguug..................................................................    1       1    Ta1
    ....................agCuauguuugauauucuugguug..................................................................    1       1    Ta1
    ....................Cgauauguuugauauucuugguug..................................................................    8       1    Ta1
    ....................agauauguuugauauucuugguuU..................................................................    9       1    Ta1
    ....................agauauguuugauauGcuugguug..................................................................    1       1    Ta1
    ....................agauauguuugauauucuugguug..................................................................    316     0    Ta1
    ....................agauauguuugauauucuugguuA..................................................................    2       1    Ta1
    ....................agauGuguuugauauucuugguug..................................................................    6       1    Ta1
    ....................agauauguuugauauucuugguugA.................................................................    7       1    Ta1
    ....................agauauguuugauauucuugguugu.................................................................    10      0    Ta1
    .....................gauauguuugCuauucuugguu...................................................................    1       1    Ta1
    .....................gauauguuugauauucuugguu...................................................................    1       0    Ta1
    .....................gauaGguuugauauucuugguug..................................................................    1       1    Ta1

    ...................Cagauauguuugauauucuugguug..................................................................    2       1    He1
    ....................agauauguuugauauucu........................................................................    3       0    He1
    ....................agauUguuugauauucuug.......................................................................    1       1    He1
    ....................agauGuguuugauauucuug......................................................................    1       1    He1
    ....................agaGauguuugauauucuug......................................................................    1       1    He1
    ....................agauauguuugauauucuug......................................................................    106     0    He1
    ....................agauauguCugauauucuug......................................................................    1       1    He1
    ....................Ugauauguuugauauucuug......................................................................    1       1    He1
    ....................agauauguuugauCuucuug......................................................................    1       1    He1
    ....................agauaGguuugauauucuugg.....................................................................    1       1    He1
```

```
                    Mature                                      Star
agauagaauccuuugcugaaagauauguuugauauucuugguuguggcuuucguuuuucaaccagaaaucagacauaugcuuuuagaggaggaguguaucuacugucaga
....................agauGuguuugauauucuugg.....................................................................   1     1   He1
....................agauauguuugauauucuugg.....................................................................   24    0   He1
....................Cgauauguuugauauucuugg.....................................................................   1     1   He1
....................agauauguuugauauucuuggu....................................................................   5     0   He1
....................agauauguuugauauucuugguA...................................................................   1     1   He1
....................agauUuguuugauauucuugguu...................................................................   2     1   He1
....................agauauguuugauauucuugguu...................................................................   54    0   He1
....................agauauguuugauauucuugguC...................................................................   1     1   He1
....................agauauguuugauauucuugguG...................................................................   1     1   He1
....................agauGuguuugauauucuugguu...................................................................   1     1   He1
....................Ugauauguuugauauucuugguug..................................................................   3     1   He1
....................agauGuguuugauauucuugguug..................................................................   9     1   He1
....................agaGauguuugauauucuugguug..................................................................   6     1   He1
....................agauauguuugauauucuuUguug..................................................................   1     1   He1
....................agaCauguuugauauucuugguug..................................................................   1     1   He1
....................agauauguuugauauucuGgguug..................................................................   1     1   He1
....................agauauguuugauauucuugguug..................................................................   651   0   He1
....................agauauguuugauauucuugguuU..................................................................   1     1   He1
....................Cgauauguuugauauucuugguug..................................................................   8     1   He1
....................Ngauauguuugauauucuugguug..................................................................   3     1   He1
....................agauauguuugauauucuugAuug..................................................................   1     1   He1
....................agauauguuugauauucuugguuA..................................................................   5     1   He1
....................agauUuguuugauauucuugguug..................................................................   13    1   He1
....................agCuauguuugauauucuugguug..................................................................   4     1   He1
....................agauauguuuAauauucuugguug..................................................................   1     1   He1
....................agauauguuugauauucuugguuC..................................................................   1     1   He1
....................agauauguAugauauucuugguug..................................................................   1     1   He1
....................agauauguuugauUuucuugguug..................................................................   2     1   He1
....................agauauguuugUuauucuugguug..................................................................   2     1   He1
....................agUuauguuugauauucuugguug..................................................................   1     1   He1
....................agauauguuugauauuUuugguug..................................................................   2     1   He1
....................agauauguuugauCuucuugguug..................................................................   2     1   He1
....................agauauguuugauauucuugguugu.................................................................   19    0   He1
....................agCuauguuugauauucuugguugu.................................................................   1     1   He1
....................agauauguuugauauucuugguugC.................................................................   6     1   He1
....................agauauguuugauauucuugguugA.................................................................   33    1   He1
.....................gauauguuugauauucuug......................................................................   1     0   He1
.....................gauauguuugauauucuugguu...................................................................   1     0   He1
.....................gCuauguuugauauucuugguug..................................................................   1     1   He1
.....................gauauguuugauauucuugguug..................................................................   7     0   He1
.....................gauUuguuugauauucuugguug..................................................................   1     1   He1
.....................gauaGguuugauauucuugguug..................................................................   1     1   He1
.......................uauguuugauauucuugguuU..................................................................   1     1   He1
............................................................accagaaaucagacauaugcuu............................   4     0   He1
............................................................accagaaaucagacauaugcuC............................   1     1   He1
............................................................accaUaaaucagacauaugcuu............................   1     1   He1
............................................................accagaaaucagacauaugcuuu...........................   10    0   He1
............................................................Nccagaaaucagacauaugcuuu...........................   1     1   He1
............................................................accagaaaucagacauaugcuuuA..........................   2     1   He1
.............................................................ccagaaaucagacauaugcuu............................   1     0   He1

....................agauauguuugauauucu........................................................................   2     0   Bl1
....................agauauguuugauauucuug......................................................................   12    0   Bl1
....................agauauguuugauauucuugg.....................................................................   7     0   Bl1
....................agauauguuugauauucuuggu....................................................................   1     0   Bl1
....................agauauguuugauauucuugguu...................................................................   30    0   Bl1
....................Cgauauguuugauauucuugguu...................................................................   2     1   Bl1
....................agauauguuugauauucuugguuU..................................................................   2     1   Bl1
....................agauaugAuugauauucuugguug..................................................................   1     1   Bl1
....................agauUuguuugauauucuugguug..................................................................   5     1   Bl1
....................agauauguuugauUuucuugguug..................................................................   1     1   Bl1
....................agaGauguuugauauucuugguug..................................................................   2     1   Bl1
....................agauauguuugauauucuugguug..................................................................   238   0   Bl1
....................agauauguAugauauucuugguug..................................................................   1     1   Bl1
....................Ggauauguuugauauucuugguug..................................................................   1     1   Bl1
....................agaCauguuugauauucuugguug..................................................................   2     1   Bl1
....................agauaugCuugauauucuugguug..................................................................   1     1   Bl1
....................agauGuguuugauauucuugguug..................................................................   3     1   Bl1
....................Ngauauguuugauauucuugguug..................................................................   1     1   Bl1
....................Cgauauguuugauauucuugguug..................................................................   3     1   Bl1
```

```
                   Mature                                      Star
agauagaauccuuugcugaaagauauguuugauauucuugguuguggcuuucguuuuucaaccagaaaucagacauaugcuuuuagaggaggaguguaucuacugucaga
....................agauauguuugauauucuugguugA.................................................................    7     1     Bl1
....................agauauguuugauauucuugguugu.................................................................    35    0     Bl1
....................Cgauauguuugauauucuugguugu.................................................................    1     1     Bl1
....................agauauguuugGuauucuugguugu.................................................................    1     1     Bl1
....................agauauguuugauauucuugguugC.................................................................    1     1     Bl1
....................agauauguAugauauucuugguugu.................................................................    1     1     Bl1
.....................gauauguuugauauucuugguug..................................................................    1     0     Bl1
............................................................accagaaaucagacauaugcC.............................    1     1     Bl1
............................................................accagaaaucagacauaugcuuu...........................    5     0     Bl1
.............................................................ccagaaaucagacauaugcuuu...........................    1     0     Bl1

....................agauauguuugauauucu........................................................................    1     0     Ch1
....................agauauguuugauauucuug......................................................................    12    0     Ch1
....................agauauguuugGuauucuug......................................................................    1     1     Ch1
....................agauaGguuugauauucuug......................................................................    1     1     Ch1
....................agauGuguuugauauucuug......................................................................    1     1     Ch1
....................Cgauauguuugauauucuugg.....................................................................    1     1     Ch1
....................agauauguuugauauucuugg.....................................................................    5     0     Ch1
....................agauauguuugauauucuuggu....................................................................    6     0     Ch1
....................agauauguuugauauucuugguC...................................................................    2     1     Ch1
....................Ugauauguuugauauucuugguu...................................................................    1     1     Ch1
....................Cgauauguuugauauucuugguu...................................................................    1     1     Ch1
....................agauauguuugauauucuugguu...................................................................    43    0     Ch1
....................agauCuguuugauauucuugguug..................................................................    1     1     Ch1
....................agauauguuugauauucuugguug..................................................................    628   0     Ch1
....................agauauguuugauauucuugguuA..................................................................    1     1     Ch1
....................agauauguuugauauucuugguuC..................................................................    1     1     Ch1
....................Ugauauguuugauauucuugguug..................................................................    7     1     Ch1
....................agauauguuugauUuucuugguug..................................................................    1     1     Ch1
....................agauauguAugauauucuugguug..................................................................    1     1     Ch1
....................agauauguuugauauucuugguuU..................................................................    5     1     Ch1
....................agaAauguuugauauucuugguug..................................................................    2     1     Ch1
....................agaGauguuugauauucuugguug..................................................................    6     1     Ch1
....................agauauguuugauauucuuUguug..................................................................    1     1     Ch1
....................agCuauguuugauauucuugguug..................................................................    3     1     Ch1
....................Cgauauguuugauauucuugguug..................................................................    7     1     Ch1
....................agauUuguuugauauucuugguug..................................................................    10    1     Ch1
....................agauaugAuugauauucuugguug..................................................................    1     1     Ch1
....................agauauguuugauaCucuugguug..................................................................    1     1     Ch1
....................agauGuguuugauauucuugguug..................................................................    13    1     Ch1
....................agauauguuugauauucuugguuUu.................................................................    2     1     Ch1
....................agauauguuugauauucuugguugu.................................................................    23    0     Ch1
....................agauauguuugauauucuugguugA.................................................................    24    1     Ch1
.....................gauauguuugCuauucuugguug..................................................................    2     1     Ch1
.....................gauauguuugauauucuugguug..................................................................    7     0     Ch1
.....................gauauguuAgauauucuugguug..................................................................    1     1     Ch1
............................................................accagaaaucagacauaugcuu............................    7     0     Ch1
............................................................accagaaaucagacauaugcuuu...........................    11    0     Ch1
...................................................................................uagaggaggaguguaucuacug.....    1     0     Ch1

....................agauauguuugauauucuu.......................................................................    1     0     Le1
....................agauUuguuugauauucuug......................................................................    3     1     Le1
....................agauauguuugauauucuug......................................................................    20    0     Le1
....................agauauguuugauauucuGg......................................................................    1     1     Le1
....................agauauguuugauauucuugg.....................................................................    5     0     Le1
....................agauauguuugauauucuuggA....................................................................    1     1     Le1
....................agauauguuugauauucuugguu...................................................................    16    0     Le1
....................agauUuguuugauauucuugguu...................................................................    1     1     Le1
....................Ggauauguuugauauucuugguug..................................................................    1     1     Le1
....................agaGauguuugauauucuugguug..................................................................    4     1     Le1
....................Ngauauguuugauauucuugguug..................................................................    1     1     Le1
....................Ugauauguuugauauucuugguug..................................................................    2     1     Le1
....................agauUuguuugauauucuugguug..................................................................    5     1     Le1
....................agauGuguuugauauucuugguug..................................................................    5     1     Le1
....................agauauguuugauauucuugguug..................................................................    254   0     Le1
....................agauauguuugauauucuugguuA..................................................................    1     1     Le1
....................agauauguuugauauucuugguuU..................................................................    1     1     Le1
....................agauauguuugauauucuCgguug..................................................................    1     1     Le1
....................Cgauauguuugauauucuugguug..................................................................    3     1     Le1
....................agauauguuugauauucuugguugA.................................................................    11    1     Le1
```

Mature **Star**

```
agauagaauccuuugcugaaagauauguuugauauucuugguuguggcuuucguuuuucaaccagaaaucagacauaugcuuuuagaggaggaguguaucuacugucaga
....................agauauguuugauauucuugguugu.................................................................   7    0   Le1
....................agauauguuugauauucuugguugC.................................................................   1    1   Le1
....................agauGuguuugauauucuugguugu.................................................................   1    1   Le1
.....................gauauguuugauauucuugguug..................................................................   3    0   Le1
.....................gCuauguuugauauucuugguug..................................................................   1    1   Le1
..........................guuugauauucuugguug..................................................................   1    0   Le1
............................................................accagaaaucagacauaugcuu............................   3    0   Le1
............................................................accagaaaucagacauaugcuuu...........................   11   0   Le1
.............................................................ccagaaaucagacauaugcuuu...........................   1    0   Le1

....................agauauguuugauauucuug......................................................................   38   0   Br1
....................agauGuguuugauauucuug......................................................................   1    1   Br1
....................agauauguuuAauauucuug......................................................................   1    1   Br1
....................Ugauauguuugauauucuug......................................................................   3    1   Br1
....................agaGauguuugauauucuug......................................................................   3    1   Br1
....................agauauguuCgauauucuug......................................................................   1    1   Br1
....................agauauguuugauauucuugA.....................................................................   4    1   Br1
....................agauauguuugauauucuugg.....................................................................   13   0   Br1
....................agauauguAugauauucuugg.....................................................................   1    1   Br1
....................agaGauguuugauauucuuggu....................................................................   1    1   Br1
....................agauauguuugauauucuugguC...................................................................   1    1   Br1
....................agauauguuugauauucAugguu...................................................................   1    1   Br1
....................agauauguuugauauucuuggAu...................................................................   1    1   Br1
....................agCuauguuugauauucuugguu...................................................................   1    1   Br1
....................agauauguuugauauucuugguu...................................................................   34   0   Br1
....................agaGauguuugauauucuugguu...................................................................   1    1   Br1
....................agauauguuugauauucuugguA...................................................................   1    1   Br1
....................Ngauauguuugauauucuugguug..................................................................   1    1   Br1
....................agaAauguuugauauucuugguug..................................................................   1    1   Br1
....................agauauguuugauauucuugguug..................................................................   120  0   Br1
....................agauUuguuugauauucuugguug..................................................................   4    1   Br1
....................agCuauguuugauauucuugguug..................................................................   1    1   Br1
....................Ugauauguuugauauucuugguug..................................................................   2    1   Br1
....................agaGauguuugauauucuugguug..................................................................   1    1   Br1
....................Cgauauguuugauauucuugguug..................................................................   2    1   Br1
....................agauGuguuugauauucuugguug..................................................................   3    1   Br1
....................agauauguuugauauucuugguugC.................................................................   1    1   Br1
....................agauauguuugauauucuugguugu.................................................................   3    0   Br1
....................agauauguuugauauucuugguugA.................................................................   15   1   Br1
.....................gauauguuugauauucuugguug..................................................................   2    0   Br1
.....................gCuauguuugauauucuugguug..................................................................   1    1   Br1
......................aGauguuugauauucuugguug..................................................................   1    1   Br1
............................................................accagaaaucagacauaugcC.............................   1    1   Br1
............................................................accagaaaucagacauaugcuuu...........................   7    0   Br1
.............................................................ccagaaaucagacauaugcuuuu..........................   1    0   Br1

................ugaaagauauguGugaua............................................................................   1    1   Le5
....................agauauguuugauauucu........................................................................   2    0   Le5
....................Ugauauguuugauauucuug......................................................................   1    1   Le5
....................agauauguuugauauucuug......................................................................   30   0   Le5
....................agaGauguuugauauucuug......................................................................   1    1   Le5
....................agauauguuugauauucuugg.....................................................................   13   0   Le5
....................agauUuguuugauauucuugg.....................................................................   1    1   Le5
....................agauauguuugauauucuuggu....................................................................   5    0   Le5
....................agauauguuugauauucuugguA...................................................................   1    1   Le5
....................Cgauauguuugauauucuugguu...................................................................   1    1   Le5
....................agauauguuugauauucuugguu...................................................................   44   0   Le5
....................agauUuguuugauauucuugguu...................................................................   1    1   Le5
....................agaGauguuugauauucuugguu...................................................................   1    1   Le5
....................agauauguuugauauucuugguC...................................................................   2    1   Le5
....................Ugauauguuugauauucuugguu...................................................................   1    1   Le5
....................agauauguAugauauucuugguu...................................................................   1    1   Le5
....................agauauguuugauauucuugguuA..................................................................   1    1   Le5
....................Cgauauguuugauauucuugguug..................................................................   13   1   Le5
....................agauauguuugauauucuugguug..................................................................   731  0   Le5
....................agauauguGugauauucuugguug..................................................................   1    1   Le5
....................agauauguCugauauucuugguug..................................................................   1    1   Le5
....................agauauguuugauauucuuUguug..................................................................   2    1   Le5
....................agauauguuugauauucuugguCg..................................................................   1    1   Le5
....................agauauguAugauauucuugguug..................................................................   1    1   Le5
```

```
                    Mature                                  Star
agauagaauccuuugcugaaagauauguuugauauucuugguuguggcuuucguuuuucaaccagaaaucagacauaugcuuuuagaggaggaguguaucuacugucaga
....................aCauauguuugauauucuugguug..................................................................   1    1    Le5
....................agaGauguuugauauucuugguug..................................................................   12   1    Le5
....................agauUuguuugauauucuugguug..................................................................   16   1    Le5
....................aAauauguuugauauucuugguug..................................................................   1    1    Le5
....................agauauguuugauauucuugguuU..................................................................   7    1    Le5
....................agCuauguuugauauucuugguug..................................................................   4    1    Le5
....................agauaugCuugauauucuugguug..................................................................   1    1    Le5
....................agauGuguuugauauucuugguug..................................................................   7    1    Le5
....................agauauUuuugauauucuugguug..................................................................   1    1    Le5
....................agauauguuugauCuucuugguug..................................................................   2    1    Le5
....................Ugauauguuugauauucuugguug..................................................................   2    1    Le5
....................agaCauguuugauauucuugguug..................................................................   1    1    Le5
....................agauauguuugauauucuugguugC.................................................................   1    1    Le5
....................agauauguuugauaAucuugguugu.................................................................   1    1    Le5
....................agauauguuugauauucuugguugG.................................................................   1    1    Le5
....................Ugauauguuugauauucuugguugu.................................................................   1    1    Le5
....................agauauguuugauauucuugguugA.................................................................   23   1    Le5
....................agauauguuugauauucuugguugu.................................................................   35   0    Le5
....................agauauguuugauauucuugguuguA................................................................   2    1    Le5
....................agauauguuugauauucuugguuguC................................................................   1    1    Le5
.....................gauauguuugauauucuugg.....................................................................   1    0    Le5
.....................gauauguuugauauucuuggu....................................................................   1    0    Le5
.....................gauaGguuugauauucuugguug..................................................................   1    1    Le5
.....................gauauguuugauauucuugguug..................................................................   2    0    Le5
.....................gauaGguuugauauucuugguugu.................................................................   1    1    Le5
.......................uauguuugauauucuugguug..................................................................   1    0    Le5
............................................................accagaaaucagacauaugcA.............................   1    1    Le5
............................................................accaUaaaucagacauaugcuu............................   2    1    Le5
............................................................accagaaaucagacauaugcuC............................   1    1    Le5
............................................................accagaaaucagacauaugcuu............................   11   0    Le5
............................................................accagaaaucagacauaugcuuu...........................   10   0    Le5
.............................................................ccagaaaucagacauaugcuu............................   1    0    Le5
.............................................................ccagUaaucagacauaugcuuu...........................   1    1    Le5
.............................................................ccagaaaucagacauaugcuuu...........................   1    0    Le5
```

Provisional ID	:	Sc28yqQ_3523_1027
Score total	:	40.9
Score for star read(s)	:	3.9
Score for read counts	:	31.2
Score for mfe	:	1.1
Score for randfold	:	1.6
Score for cons. seed	:	3
Total read count	:	73
Mature read count	:	33
Loop read count	:	0
Star read count	:	40

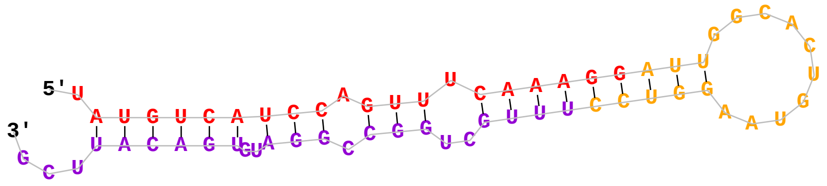

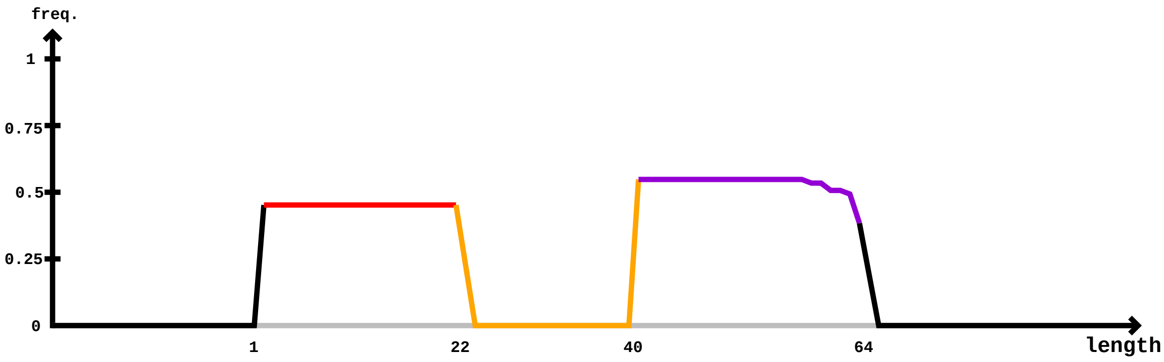

Mature **Star**

```
5'- gagaggcuguuacaguugugwuaugucauccaguuucaaaggauuggcacuguaagguccuuugcuggccggaugugacauucguuacauguaacauuucaccagugcugcg   -3'   obs
    gagaggcuguuacaguuguguaugucauccaguuucaaaggauuggcacuguaagguccuuugcuggccggaugugacauucguuacauguaacauuucaccagugcugcg         exp
    ..((((.(((((((((..((.(((((((((.(((.(((((((((..........)))))))))..))).)))..)))))).))..)).))))))))))).............   reads   mm   sample
    ....................uaugGcauccaguuucaaagg......................................................................   2       1    Le5
    ....................uaugucauccaguuucaaagg......................................................................   8       0    Le5
    ...........................................................uuugcuggccggauguga..................................   1       0    Le5
    ...........................................................uuugcuggccggaugugaca................................   1       0    Le5
    ...........................................................uuugcuggccggaugugacauucg............................   3       0    Le5

    ...........................................................uuugcuggccggaugugacauu..............................   1       0    Le1
    ...........................................................uuugcuggccggaugugacauuc.............................   2       0    Le1
    ...........................................................uuugGuggccggaugugacauucg............................   1       1    Le1
    ...........................................................uuugcuggccggaugugacauucg............................   7       0    Le1

    ....................uaugucauccaguuucaaagg......................................................................   1       0    Br1
    ...........................................................uuugcuggccggaugugacauuc.............................   1       0    Br1
    ...........................................................uuugUuggccggaugugacauuc.............................   1       1    Br1
    ...........................................................uuugcuggccggaugugacauucg............................   1       0    Br1

    ....................uaugucauccaguuucaaagg......................................................................   8       0    He1
    ...........................................................uuugcuggccggaugugacauuc.............................   3       0    He1
    ...........................................................uuugcuggccggaugugacauucg............................   3       0    He1
    ...........................................................uuugcuggccggaugugacauucgA...........................   1       1    He1
    ...........................................................uuugcuggccggaugugacauucgu...........................   1       0    He1

    ....................uaugucauccaguuucaaagg......................................................................   3       0    Bl1
    ...........................................................uuugcuggccggaugugaca................................   1       0    Bl1
    ...........................................................uuugcuggccggaugugacauuc.............................   1       0    Bl1
    ...........................................................uuugcuggccggaugugacauucU............................   1       1    Bl1
    ...........................................................uuugcuggccggaugugacauucg............................   1       0    Bl1

    ....................uaugucauccaUuuucaaagg......................................................................   1       1    Ch1
    ....................uaugucauccaguuucaaagg......................................................................   5       0    Ch1
    ...........................................................uuugcuggccggaugugacauucg............................   4       0    Ch1

    ....................uaugucauccaguuucaaagg......................................................................   5       0    Ta1
```

```
                    Mature                                      Star
gagaggcuguuacaguugugUAUGUCAUCCAGUUUCAAAGGauuggcacuguaagguccUUUGCUGGCCGGAUGUGACAUUCGuuacauguaacauuucaccagugcugcg
...........................................................uuugcuggccggaugugacauucg............................   5   0   Ta1
```

Provisional ID	:	Sc28yqQ_3523_1063
Score total	:	359390.6
Score for star read(s)	:	3.9
Score for read counts	:	359380.4
Score for mfe	:	1.7
Score for randfold	:	1.6
Score for cons. seed	:	3
Total read count	:	704921
Mature read count	:	703091
Loop read count	:	0
Star read count	:	1830

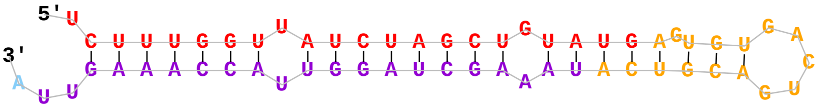

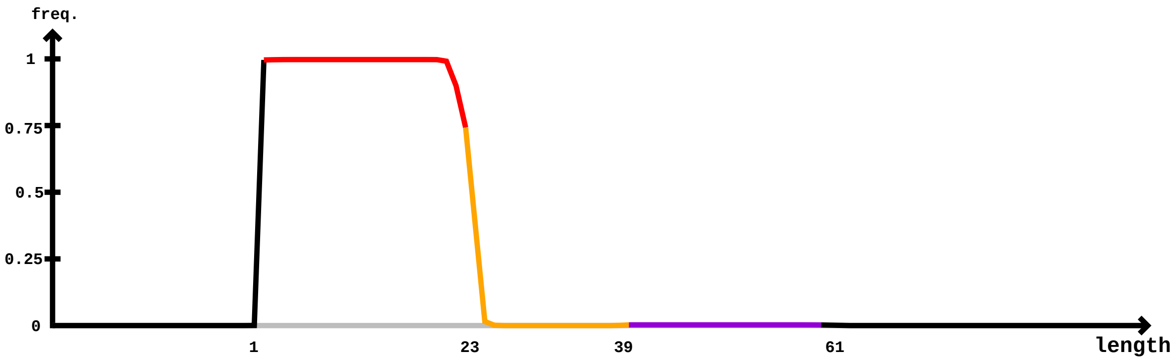

Mature Star

```
5'- gucggcaggcauugauguuuucuuugguuaucuagcuguaugagugugacugacgucauaaagcuagguuaccaaaguuaaaaacaaucgccagaaaucagcuggaugacag  -3'   obs
    gucggcaggcauugauguuuucuuugguuaucuagcuguaugagugugacugacgucauaaagcuagguuaccaaaguuaaaaacaaucgccagaaaucagcuggaugacag        exp
    .((((.(((((((...((..(((((((.((((((((.(((((.(((.....)))))))).)))))))).)))))))..))...)))).))).(....).)))))....... reads  mm  sample
    ...................Cucuuugguuaucuagcugu.........................................................................  1  1  Le1
    ...................Aucuuugguuaucuagcuguau.......................................................................  1  1  Le1
    ...................Cucuuugguuaucuagcuguau.......................................................................  4  1  Le1
    ...................Aucuuugguuaucuagcuguaug......................................................................  2  1  Le1
    ...................Cucuuugguuaucuagcuguaug......................................................................  21  1  Le1
    ...................Cucuuugguuaucuagcuguauga.....................................................................  4  1  Le1
    ....................ucuuugguuaucuagcuA..........................................................................  1  1  Le1
    ....................ucuGugguuaucuagcug..........................................................................  1  1  Le1
    ....................ucuuugguuaucuagcug..........................................................................  25  0  Le1
    ....................ucuuugguuaucuagUug..........................................................................  1  1  Le1
    ....................ucuuGgguuaucuagcug..........................................................................  1  1  Le1
    ....................ucuuCgguuaucuagcugu.........................................................................  4  1  Le1
    ....................ucuuugUuuaucuagcugu.........................................................................  2  1  Le1
    ....................ucuuugCuuaucuagcugu.........................................................................  1  1  Le1
    ....................ucuuugguuaucuagcugA.........................................................................  2  1  Le1
    ....................ucuuugguuaucuagcuUu.........................................................................  2  1  Le1
    ....................Ccuuugguuaucuagcugu.........................................................................  3  1  Le1
    ....................ucuuAgguuaucuagcugu.........................................................................  6  1  Le1
    ....................ucuGugguuaucuagcugu.........................................................................  9  1  Le1
    ....................ucuuuUguuaucuagcugu.........................................................................  2  1  Le1
    ....................ucuuugguuaucuaUcugu.........................................................................  3  1  Le1
    ....................ucuuugguuaucuagcugC.........................................................................  90  1  Le1
    ....................Acuuugguuaucuagcugu.........................................................................  3  1  Le1
    ....................ucuuugguuaucuagUugu.........................................................................  1  1  Le1
    ....................ucuuuggAuaucuagcugu.........................................................................  1  1  Le1
    ....................ucuuugguuCucuagcugu.........................................................................  2  1  Le1
    ....................ucuuugguAaucuagcugu.........................................................................  2  1  Le1
    ....................ucuuugguCaucuagcugu.........................................................................  1  1  Le1
    ....................ucuuGgguuaucuagcugu.........................................................................  36  1  Le1
    ....................ucCuugguuaucuagcugu.........................................................................  3  1  Le1
    ....................ucuCugguuaucuagcugu.........................................................................  1  1  Le1
    ....................uAuuugguuaucuagcugu.........................................................................  1  1  Le1
    ....................ucuuugguuaucuagcugu.........................................................................  977  0  Le1
```

Mature **Star**

```
gucggcaggcauugauguuuucuuugguuaucuagcuguaugagugugacugacgucauaaagcuagguuaccaaaguuaaaaacaaucgccagaaaucagcuggaugacag
....................ucuuugguCaucuagcugua........................................................................ 3 1 Le1
....................ucuuugguuaucuCgcugua........................................................................ 16 1 Le1
....................Ccuuugguuaucuagcugua........................................................................ 16 1 Le1
....................Ncuuugguuaucuagcugua........................................................................ 11 1 Le1
....................ucuuuggGuaucuagcugua........................................................................ 6 1 Le1
....................ucuCugguuaucuagcugua........................................................................ 17 1 Le1
....................ucCuugguuaucuagcugua........................................................................ 24 1 Le1
....................ucuuGgguuaucuagcugua........................................................................ 497 1 Le1
....................ucuuugguuaucuagcugCa........................................................................ 29 1 Le1
....................ucuuugguuaucuGgcugua........................................................................ 5 1 Le1
....................ucuuuCguuaucuagcugua........................................................................ 1 1 Le1
....................ucuuugUuuaucuagcugua........................................................................ 7 1 Le1
....................ucAuugguuaucuagcugua........................................................................ 1 1 Le1
....................uAuuugguuaucuagcugua........................................................................ 17 1 Le1
....................ucuuugguuaucuagcuguG........................................................................ 11 1 Le1
....................ucuuugguuauAuagcugua........................................................................ 1 1 Le1
....................ucuuugguuaucuagcAgua........................................................................ 1 1 Le1
....................Gcuuugguuaucuagcugua........................................................................ 5 1 Le1
....................ucuGugguuaucuagcugua........................................................................ 82 1 Le1
....................ucuuugguuaucuagcuAua........................................................................ 2 1 Le1
....................ucuuugguuaucCagcugua........................................................................ 4 1 Le1
....................ucuuugguuaucuagcuguU........................................................................ 12 1 Le1
....................ucGuugguuaucuagcugua........................................................................ 2 1 Le1
....................ucuuugCuuaucuagcugua........................................................................ 1 1 Le1
....................ucuuugguuauUuagcugua........................................................................ 1 1 Le1
....................ucuuugguuaucuagcugGa........................................................................ 4 1 Le1
....................ucuuugguuGucuagcugua........................................................................ 3 1 Le1
....................ucuuugguuaGcuagcugua........................................................................ 1 1 Le1
....................ucuuugguuaucuagcuguC........................................................................ 3 1 Le1
....................ucuuugguuaucuagcugua........................................................................ 11624 0 Le1
....................ucuuugguuaCcuagcugua........................................................................ 1 1 Le1
....................ucuuugguuaucuagUugua........................................................................ 11 1 Le1
....................ucuuugguuaucuaUcugua........................................................................ 29 1 Le1
....................ucuuugguuaucuaCcugua........................................................................ 3 1 Le1
....................ucuuuggCuaucuagcugua........................................................................ 6 1 Le1
....................ucuuugguAaucuagcugua........................................................................ 10 1 Le1
....................ucuuugguuaucuaAcugua........................................................................ 1 1 Le1
....................ucuuCgguuaucuagcugua........................................................................ 75 1 Le1
....................ucuuugguuaucuagcuUua........................................................................ 10 1 Le1
....................ucuuugguuaAcuagcugua........................................................................ 2 1 Le1
....................ucuuugguuaucAagcugua........................................................................ 2 1 Le1
....................ucuuugAuuaucuagcugua........................................................................ 1 1 Le1
....................ucuuugguuaucuUgcugua........................................................................ 4 1 Le1
....................ucuuugguuaucuagcGgua........................................................................ 1 1 Le1
....................ucuuAgguuaucuagcugua........................................................................ 87 1 Le1
....................uGuuugguuaucuagcugua........................................................................ 1 1 Le1
....................ucuAugguuaucuagcugua........................................................................ 21 1 Le1
....................Acuuugguuaucuagcugua........................................................................ 17 1 Le1
....................ucuuuUguuaucuagcugua........................................................................ 8 1 Le1
....................ucuuugguGaucuagcugua........................................................................ 1 1 Le1
....................ucuuugguuCucuagcugua........................................................................ 23 1 Le1
....................ucuuugguuaucuagcCgua........................................................................ 3 1 Le1
....................ucuuuggAuaucuagcugua........................................................................ 2 1 Le1
....................ucuuugguuaucuCgcuguau....................................................................... 19 1 Le1
....................ucuuugguCaucuagcuguau....................................................................... 4 1 Le1
....................ucuuuggGuaucuagcuguau....................................................................... 2 1 Le1
....................ucuuugguuaCcuagcuguau....................................................................... 2 1 Le1
....................ucuuugguuaucuaUcuguau....................................................................... 48 1 Le1
....................ucuuugguuaucuagcuguaG....................................................................... 63 1 Le1
....................ucuuugguuaucuagcugCau....................................................................... 5 1 Le1
....................ucuuugguuaucuagcCguau....................................................................... 4 1 Le1
....................ucuuugguuaucuagcuguaA....................................................................... 563 1 Le1
....................ucuuugguuaucuagcuCuau....................................................................... 2 1 Le1
....................ucuuugguuaucuGgcuguau....................................................................... 4 1 Le1
....................ucuuugguuaucuagcuUuau....................................................................... 7 1 Le1
....................ucuuuAguuaucuagcuguau....................................................................... 1 1 Le1
....................ucuuugguuaucuagcugGau....................................................................... 15 1 Le1
....................ucuuCgguuaucuagcuguau....................................................................... 114 1 Le1
....................ucuuugguuCucuagcuguau....................................................................... 62 1 Le1
....................ucuuAgguuaucuagcuguau....................................................................... 141 1 Le1
```

Mature Star

```
gucggcaggcauugauguuuucuuugguuaucuagcuguaugagugugacugacgucauaaagcuagguuaccaaaguuaaaaacaaucgccagaaaucagcuggaugacag
....................ucuuugguuaAcuagcuguau.......................................................................   8      1   Le1
....................Gcuuugguuaucuagcuguau.......................................................................   2      1   Le1
....................ucuuuUguuaucuagcuguau.......................................................................   3      1   Le1
....................Ccuuugguuaucuagcuguau.......................................................................   25     1   Le1
....................ucuuuggCuaucuagcuguau.......................................................................   2      1   Le1
....................ucuuugguuaucuagcugAau.......................................................................   4      1   Le1
....................ucuuugguuauGuagcuguau.......................................................................   3      1   Le1
....................Acuuugguuaucuagcuguau.......................................................................   37     1   Le1
....................ucuuugguuaucuagcuguUu.......................................................................   11     1   Le1
....................ucuuugguuaucuUgcuguau.......................................................................   4      1   Le1
....................ucuuugguuaGcuagcuguau.......................................................................   1      1   Le1
....................ucuuugguAaucuagcuguau.......................................................................   14     1   Le1
....................ucuAugguuaucuagcuguau.......................................................................   23     1   Le1
....................ucuuugguuGucuagcuguau.......................................................................   3      1   Le1
....................ucuuugAuuaucuagcuguau.......................................................................   1      1   Le1
....................ucuuugguuaucGagcuguau.......................................................................   1      1   Le1
....................ucuuugguuaucuaCcuguau.......................................................................   1      1   Le1
....................Ncuuugguuaucuagcuguau.......................................................................   16     1   Le1
....................ucuuugguGaucuagcuguau.......................................................................   2      1   Le1
....................ucuCugguuaucuagcuguau.......................................................................   25     1   Le1
....................ucuuugguuaucAagcuguau.......................................................................   2      1   Le1
....................ucuuugguuaucuagcAguau.......................................................................   3      1   Le1
....................ucuuugguuaucCagcuguau.......................................................................   4      1   Le1
....................ucuuGgguuaucuagcuguau.......................................................................   863    1   Le1
....................ucuuugguuaucuagcuAuau.......................................................................   4      1   Le1
....................ucAuugguuaucuagcuguau.......................................................................   2      1   Le1
....................ucuuugguuaucuagcGguau.......................................................................   1      1   Le1
....................ucuuugguuaucuagAuguau.......................................................................   1      1   Le1
....................ucuuugguuaucuagUuguau.......................................................................   14     1   Le1
....................ucuuugguuaucuagcuguGu.......................................................................   16     1   Le1
....................ucuuugguuaucuaAcuguau.......................................................................   7      1   Le1
....................ucuuugguuaucuagcuguau.......................................................................   19174  0   Le1
....................uGuuugguuaucuagcuguau.......................................................................   2      1   Le1
....................ucuGugguuaucuagcuguau.......................................................................   148    1   Le1
....................ucuuugguuauAuagcuguau.......................................................................   1      1   Le1
....................ucuuugUuuaucuagcuguau.......................................................................   30     1   Le1
....................ucuuugguuaucuagcuguaC.......................................................................   275    1   Le1
....................ucGuugguuaucuagcuguau.......................................................................   1      1   Le1
....................ucuuuCguuaucuagcuguau.......................................................................   2      1   Le1
....................ucCuugguuaucuagcuguau.......................................................................   56     1   Le1
....................ucuuugCuuaucuagcuguau.......................................................................   2      1   Le1
....................ucuuugguuauUuagcuguau.......................................................................   4      1   Le1
....................uAuuugguuaucuagcuguau.......................................................................   22     1   Le1
....................ucuuugguuaucuagcuguUug......................................................................   28     1   Le1
....................ucuuugguuaucuagcuguGug......................................................................   25     1   Le1
....................ucuuAgguuaucuagcuguaug......................................................................   450    1   Le1
....................ucuuCgguuaucuagcuguaug......................................................................   382    1   Le1
....................ucuuugguuaucuagcuguCug......................................................................   2      1   Le1
....................ucuuugguuauGuagcuguaug......................................................................   3      1   Le1
....................ucuuugguuaucCagcuguaug......................................................................   15     1   Le1
....................ucuuugguGaucuagcuguaug......................................................................   6      1   Le1
....................ucuuugguuaucuagcCguaug......................................................................   13     1   Le1
....................ucuuugguuaucuagcuCuaug......................................................................   2      1   Le1
....................Acuuugguuaucuagcuguaug......................................................................   99     1   Le1
....................ucuuuAguuaucuagcuguaug......................................................................   9      1   Le1
....................ucuuugguuaucuagcuguauA......................................................................   466    1   Le1
....................ucuuugguuCucuagcuguaug......................................................................   181    1   Le1
....................ucuuugguuaucuaCcuguaug......................................................................   4      1   Le1
....................ucuuugguCaucuagcuguaug......................................................................   20     1   Le1
....................ucuuugguuaucAagcuguaug......................................................................   5      1   Le1
....................Ccuuugguuaucuagcuguaug......................................................................   76     1   Le1
....................ucuuugguuaucuaAcuguaug......................................................................   12     1   Le1
....................ucuuuggGuaucuagcuguaug......................................................................   5      1   Le1
....................ucuuugguuaGcuagcuguaug......................................................................   2      1   Le1
....................Gcuuugguuaucuagcuguaug......................................................................   16     1   Le1
....................ucuuugguuaucuGgcuguaug......................................................................   9      1   Le1
....................ucuuugguuauUuagcuguaug......................................................................   17     1   Le1
....................ucuCugguuaucuagcuguaug......................................................................   69     1   Le1
....................ucuuugguuaucGagcuguaug......................................................................   4      1   Le1
....................ucuuuggAuaucuagcuguaug......................................................................   4      1   Le1
```

```
                    Mature                                      Star
gucggcaggcauugauguuuucuuugguuaucuagcuguaugagugugacugacgucauaaagcuagguuaccaaaguuaaaaacaaucgccagaaaucagcuggaugacag
....................ucuuugguuaucuUgcuguaug......................................................................   35      1   Le1
....................ucuGugguuaucuagcuguaug......................................................................   473     1   Le1
....................ucuuugguuaCcuagcuguaug......................................................................   8       1   Le1
....................ucuuugguuGucuagcuguaug......................................................................   9       1   Le1
....................ucuuuggCuaucuagcuguaug......................................................................   7       1   Le1
....................ucuuugguuaucuagcuguauC......................................................................   9       1   Le1
....................ucuuugguuaucuagcGguaug......................................................................   3       1   Le1
....................ucuAugguuaucuagcuguaug......................................................................   73      1   Le1
....................ucCuugguuaucuagcuguaug......................................................................   148     1   Le1
....................ucuuugguuaAcuagcuguaug......................................................................   14      1   Le1
....................ucAuugguuaucuagcuguaug......................................................................   3       1   Le1
....................ucuuugguuaucuagcugAaug......................................................................   9       1   Le1
....................ucuuugguuaucuagcugCaug......................................................................   10      1   Le1
....................ucuuugguuaucuagcuUuaug......................................................................   38      1   Le1
....................ucuuugguuaucuCgcuguaug......................................................................   52      1   Le1
....................ucuuugguuaucuaUcuguaug......................................................................   134     1   Le1
....................ucuuugguuaucuagUuguaug......................................................................   67      1   Le1
....................uAuuugguuaucuagcuguaug......................................................................   84      1   Le1
....................ucuuugguuaucuagAuguaug......................................................................   8       1   Le1
....................ucuuugguuaucuagcuguaCg......................................................................   23      1   Le1
....................ucuuugguuaucuagcuguaGg......................................................................   1       1   Le1
....................ucGuugguuaucuagcuguaug......................................................................   1       1   Le1
....................ucuuugguuaucuagGuguaug......................................................................   2       1   Le1
....................ucuuugguuaucuagcuAuaug......................................................................   6       1   Le1
....................ucuuugguAaucuagcuguaug......................................................................   46      1   Le1
....................ucuuGgguuaucuagcuguaug......................................................................   2544    1   Le1
....................ucuuugguuaucuagcuguaAg......................................................................   7       1   Le1
....................uGuuugguuaucuagcuguaug......................................................................   9       1   Le1
....................ucuuugguuaucuagcugGaug......................................................................   17      1   Le1
....................ucuuuCguuaucuagcuguaug......................................................................   1       1   Le1
....................ucuuugguuaucuagcAguaug......................................................................   8       1   Le1
....................ucuuugguuaucuagcuguaug......................................................................   60395   0   Le1
....................ucuuuUguuaucuagcuguaug......................................................................   35      1   Le1
....................ucuuugCuuaucuagcuguaug......................................................................   3       1   Le1
....................ucuuugUuuaucuagcuguaug......................................................................   64      1   Le1
....................Ncuuugguuaucuagcuguaug......................................................................   37      1   Le1
....................ucuuugguuaucuagcuguauU......................................................................   307     1   Le1
....................uUuuugguuaucuagcuguaug......................................................................   34      1   Le1
....................ucuuugAuuaucuagcuguaug......................................................................   8       1   Le1
....................ucuuugguuauAuagcuguaug......................................................................   2       1   Le1
....................ucuuugguuaucuaCcuguauga.....................................................................   3       1   Le1
....................ucuuugUuuaucuagcuguauga.....................................................................   14      1   Le1
....................ucuuugguuaucuagcugGauga.....................................................................   2       1   Le1
....................ucuuGgguuaucuagcuguauga.....................................................................   434     1   Le1
....................ucuuugguuaucuGgcuguauga.....................................................................   2       1   Le1
....................ucuuCgguuaucuagcuguauga.....................................................................   66      1   Le1
....................ucuuugguuaucuagcuguaGga.....................................................................   1       1   Le1
....................ucuuugCuuaucuagcuguauga.....................................................................   1       1   Le1
....................ucuGugguuaucuagcuguauga.....................................................................   82      1   Le1
....................ucuuugguuauUuagcuguauga.....................................................................   3       1   Le1
....................ucuuugguuauGuagcuguauga.....................................................................   1       1   Le1
....................Ccuuugguuaucuagcuguauga.....................................................................   12      1   Le1
....................ucuuugAuuaucuagcuguauga.....................................................................   1       1   Le1
....................ucuuuggCuaucuagcuguauga.....................................................................   6       1   Le1
....................ucuuuggAuaucuagcuguauga.....................................................................   5       1   Le1
....................ucuuugguuaucuagcuguUuga.....................................................................   1       1   Le1
....................uUuuugguuaucuagcuguauga.....................................................................   5       1   Le1
....................ucuuAgguuaucuagcuguauga.....................................................................   81      1   Le1
....................ucuuugguuaucuaAcuguauga.....................................................................   4       1   Le1
....................ucuuugguuaucuagcuguGuga.....................................................................   5       1   Le1
....................ucuuugguuaucCagcuguauga.....................................................................   4       1   Le1
....................uAuuugguuaucuagcuguauga.....................................................................   14      1   Le1
....................ucuuugguuaucuagcuguauCa.....................................................................   1       1   Le1
....................ucuuugguuaucuagcuguauUa.....................................................................   14      1   Le1
....................ucuuuCguuaucuagcuguauga.....................................................................   1       1   Le1
....................ucuuugguuaucuagUuguauga.....................................................................   7       1   Le1
....................ucuuugguuaucuagcugCauga.....................................................................   3       1   Le1
....................ucuuugguuaucuagcuguaAga.....................................................................   3       1   Le1
....................ucuuugguuaucuUgcuguauga.....................................................................   4       1   Le1
....................ucuuugguuaucuagcuguaCga.....................................................................   2       1   Le1
```

Mature Star

```
gucggcaggcauugauguuuucuuugguuaucuagcuguaugagugugacugacgucauaaagcuagguuaccaaaguuaaaaacaaucgccagaaaucagcuggaugacag
....................Gcuuugguuaucuagcuguauga.....................................................................  4      1   Le1
....................ucuuuUguuaucuagcuguauga.....................................................................  11     1   Le1
....................ucGuugguuaucuagcuguauga.....................................................................  1      1   Le1
....................ucuuuggGuaucuagcuguauga.....................................................................  2      1   Le1
....................ucuuugguuaucuagcGguauga.....................................................................  1      1   Le1
....................ucuuugguuCucuagcuguauga.....................................................................  27     1   Le1
....................ucuuugguuaucuagcuguaugG.....................................................................  32     1   Le1
....................ucuuugguAaucuagcuguauga.....................................................................  7      1   Le1
....................ucCuugguuaucuagcuguauga.....................................................................  26     1   Le1
....................ucuuugguuaucuagGuguauga.....................................................................  2      1   Le1
....................ucuuugguuaucuCgcuguauga.....................................................................  14     1   Le1
....................ucuuugguuaucuagcuguaugU.....................................................................  2404   1   Le1
....................ucuuugguuaucAagcuguauga.....................................................................  3      1   Le1
....................ucuuugguuaucuagcuguaugC.....................................................................  229    1   Le1
....................ucuuugguuaucuaUcuguauga.....................................................................  27     1   Le1
....................ucuCugguuaucuagcuguauga.....................................................................  10     1   Le1
....................Acuuugguuaucuagcuguauga.....................................................................  21     1   Le1
....................ucuuuAguuaucuagcuguauga.....................................................................  3      1   Le1
....................ucuuugguuaucuagcuUuauga.....................................................................  14     1   Le1
....................ucuuugguuaucuagcuguauAa.....................................................................  66     1   Le1
....................ucuuugguuaucuagcCguauga.....................................................................  6      1   Le1
....................ucuuugguuaCcuagcuguauga.....................................................................  10     1   Le1
....................ucuuuggCaucuagcuguauga.....................................................................  2      1   Le1
....................ucuuugguuaucuagcuguauga.....................................................................  10841  0   Le1
....................ucuuugguuaucuagcugAauga.....................................................................  1      1   Le1
....................ucuuugguuaucGagcuguauga.....................................................................  1      1   Le1
....................Ncuuugguuaucuagcuguauga.....................................................................  5      1   Le1
....................ucuuugguuaucuagcAguauga.....................................................................  1      1   Le1
....................ucuuugguuaAcuagcuguauga.....................................................................  1      1   Le1
....................ucuuugguuaucuagcuAuauga.....................................................................  3      1   Le1
....................ucuAugguuaucuagcuguauga.....................................................................  10     1   Le1
....................ucuuugguuaucuagcuguaugGg....................................................................  1      1   Le1
....................ucuuugguuaucuagcuguaugaC....................................................................  26     1   Le1
....................ucuuugguuaucuagcuguaugag....................................................................  23     0   Le1
....................ucuuugguuaucuagcuguaugaU....................................................................  847    1   Le1
....................ucuuugguuaucuagcuguauAag....................................................................  1      1   Le1
....................ucuuugguuaucuagcuguaugUg....................................................................  21     1   Le1
....................ucuuugguuaucuagcuguaugaA....................................................................  678    1   Le1
....................ucuuugguuaucuagcuguaugaAu...................................................................  43     1   Le1
....................ucuuugguuaucuagcuguaugagA...................................................................  2      1   Le1
....................ucuuugguuaucuagcuguaugaUu...................................................................  49     1   Le1
....................ucuuugguuCucuagcuguaugagu...................................................................  1      1   Le1
....................ucuuugguuaucuagcuguaugaCu...................................................................  6      1   Le1
....................ucuuugguuaucuagcuguaugagu...................................................................  3      0   Le1
....................ucuuugguuaucuagcuguaugUgu...................................................................  2      1   Le1
....................ucuuugguuaucuagcuguaugaguU..................................................................  1      1   Le1
.....................cuuuUguuaucuagcugu.........................................................................  1      1   Le1
.....................cuuuUguuaucuagcugua........................................................................  1      1   Le1
.....................cuuugguuaucuagcugua........................................................................  7      0   Le1
.....................cuuugguuaucuagAuguau.......................................................................  1      1   Le1
.....................cuuugguuaucuagcuguau.......................................................................  4      0   Le1
.....................cuuAgguuaucuagcuguau.......................................................................  1      1   Le1
.....................cuuugguuaucuagcuguaA.......................................................................  2      1   Le1
.....................cuuugguuaucuagcuguaC.......................................................................  1      1   Le1
.....................cuuGgguuaucuagcuguau.......................................................................  2      1   Le1
.....................cuuugguuaucuagcuguauA......................................................................  2      1   Le1
.....................cuuugguuaucuagcugGaug......................................................................  1      1   Le1
.....................cuuugguuCucuagcuguaug......................................................................  1      1   Le1
.....................cuuugguuaucuagGuguaug......................................................................  1      1   Le1
.....................cAuugguuaucuagcuguaug......................................................................  1      1   Le1
.....................cuuuUguuaucuagcuguaug......................................................................  4      1   Le1
.....................cuuugguuaucuagcuguaug......................................................................  32     0   Le1
.....................cuuugguuaucuagcuguUug......................................................................  1      1   Le1
.....................cuuGgguuaucuagcuguaug......................................................................  4      1   Le1
.....................cuuGgguuaucuagcuguauga.....................................................................  1      1   Le1
.....................cuuugguuaucuagcuguauga.....................................................................  20     0   Le1
.....................cuuugguuaucuagcuguaugU.....................................................................  3      1   Le1
.....................cuuuUguuaucuagcuguauga.....................................................................  2      1   Le1
.....................cuuugguuaucuagcuCuauga.....................................................................  1      1   Le1
.....................cuuugguuauAuagcuguauga.....................................................................  1      1   Le1
```

```
                    Mature                                  Star
gucggcaggcauugauguuuucuuugguuaucuagcuguaugagugugacugacgucauaaagcuagguuaccaaaguuaaaaacaaucgccagaaaucagcuggaugacag
.....................cuuugguuaucuagcuguaGga.....................................................................    1    1    Le1
.....................cuuugguuaucuagcuguaugaA....................................................................    2    1    Le1
.....................cuuugguuaucuagcuguaugaAu...................................................................    1    1    Le1
......................uuugguuaucuagcugua........................................................................    2    0    Le1
......................uuugguuaucuagcuguau.......................................................................    2    0    Le1
......................uCugguuaucuagcuguau.......................................................................    1    1    Le1
......................uuugUuuaucuagcuguaug......................................................................    3    1    Le1
......................uCugguuaucuagcuguaug......................................................................    4    1    Le1
......................uuugguuaucuagcuguaug......................................................................    31   0    Le1
......................uuugguuaucuagcuguaugU.....................................................................    1    1    Le1
......................uuugCuuaucuagcuguauga.....................................................................    1    1    Le1
......................uuugguuaucuagcuguauga.....................................................................    22   0    Le1
......................uuugUuuaucuagcuguauga.....................................................................    2    1    Le1
.......................uuggGuaucuagcuguau.......................................................................    1    1    Le1
.......................uugguuaucuagcuguaug......................................................................    6    0    Le1
.......................uugguuaucuagcuguauga.....................................................................    3    0    Le1
.......................uugguuaucuagcuguaugC.....................................................................    1    1    Le1
.......................uugguuaucuagcuguaugagu...................................................................    1    0    Le1
........................ugguuaucuagcuguaug......................................................................    8    0    Le1
........................ugguuaucuagcuguauga.....................................................................    6    0    Le1
.........................................................auaaagcuagguuaccaa.....................................    1    0    Le1
.........................................................auaaagcuagguuaccaaa....................................    1    0    Le1
.........................................................auaaagcuagguuaccaaag...................................    2    0    Le1
.........................................................auaaGgcuagguuaccaaagu..................................    1    1    Le1
.........................................................auaaagcuagguuaccaaagu..................................    8    0    Le1
.........................................................auaaagcuagguuaUcaaaguu.................................    1    1    Le1
.........................................................auaaagcuagguuaccaaaguC.................................    1    1    Le1
.........................................................auaaagcuagguuaccaaaguu.................................    38   0    Le1
.........................................................aCaaagcuagguuaccaaaguua................................    1    1    Le1
.........................................................Uuaaagcuagguuaccaaaguua................................    1    1    Le1
.........................................................auaaagcuagguuaccaaaguua................................    13   0    Le1
..........................................................uaaagcuagguuaccaaag...................................    3    0    Le1
..........................................................uaaagcuagguuaccaaagu..................................    16   0    Le1
..........................................................uaaaCcuagguuaccaaaguu.................................    1    1    Le1
..........................................................uaaagcuagguuacUaaaguu.................................    1    1    Le1
..........................................................uaaagcuagguuaccaaaguC.................................    1    1    Le1
..........................................................uaaagcuagguuaccaaaguu.................................    110  0    Le1
..........................................................Caaagcuagguuaccaaaguu.................................    1    1    Le1
..........................................................uaaagcuaAguuaccaaaguu.................................    1    1    Le1
..........................................................uaaaUcuagguuaccaaaguu.................................    4    1    Le1
..........................................................uaaaCcuagguuaccaaaguua................................    1    1    Le1
..........................................................uaaagcuagguuaccaaaguua................................    39   0    Le1
..........................................................uaaaUcuagguuaccaaaguua................................    1    1    Le1
..........................................................uCaagcuagguuaccaaaguua................................    1    1    Le1
..........................................................uaaagcuagguuaccaaaguuaU...............................    1    1    Le1
..........................................................uaaagcuagguuaccaaaguuaa...............................    1    0    Le1
...........................................................aaagcuagguuaccaaaguu.................................    2    0    Le1
...........................................................aaagcuagguuaccaaaguua................................    1    0    Le1

..................Cuucuuugguuaucuagcuguau.......................................................................    1    1    Br1
...................Aucuuugguuaucuagcuguaug......................................................................    1    1    Br1
....................ucuuugguuaucuagcug..........................................................................    2    0    Br1
....................ucuuugguuaucuGgcugu.........................................................................    1    1    Br1
....................ucuuugguuaucuCgcugu.........................................................................    1    1    Br1
....................ucuuugguuaucuagcugu.........................................................................    8    0    Br1
....................ucuuugguuaucuaUcugua........................................................................    1    1    Br1
....................ucuuugguuaucuagcuAua........................................................................    1    1    Br1
....................ucuuugguuaAcuagcugua........................................................................    1    1    Br1
....................ucCuugguuaucuagcugua........................................................................    2    1    Br1
....................ucuuugguuaucuagcugua........................................................................    141  0    Br1
....................ucuuugguuaucuagcugCa........................................................................    1    1    Br1
....................ucuuugguuCucuagcugua........................................................................    1    1    Br1
....................ucuCugguuaucuagcugua........................................................................    1    1    Br1
....................Acuuugguuaucuagcugua........................................................................    1    1    Br1
....................ucuuGgguuaucuagcugua........................................................................    5    1    Br1
....................ucuuugguuaucuagcuAuau.......................................................................    1    1    Br1
....................ucuuugguuaucuagcuguaC.......................................................................    5    1    Br1
....................ucuuuAguuaucuagcuguau.......................................................................    1    1    Br1
....................Acuuugguuaucuagcuguau.......................................................................    2    1    Br1
....................ucuuAgguuaucuagcuguau.......................................................................    3    1    Br1
```

Mature **Star**

```
gucggcaggcauugauguuuucuuugguuaucuagcuguaugagugugacugacgucauaaagcuagguuaccaaaguuaaaaacaaucgccagaaaucagcuggaugacag
....................ucuuuUguuaucuagcuguau....................................................................... 3 1 Br1
....................ucuuCgguuaucuagcuguau....................................................................... 3 1 Br1
....................ucuuugCuuaucuagcuguau....................................................................... 1 1 Br1
....................ucuuugguuaucuagcuguaA....................................................................... 11 1 Br1
....................ucuuugguuaucuagUuguau....................................................................... 1 1 Br1
....................ucuuugguuaucuagcuguau....................................................................... 327 0 Br1
....................ucuCugguuaucuagcuguau....................................................................... 2 1 Br1
....................uAuuugguuaucuagcuguau....................................................................... 1 1 Br1
....................ucuuugguuaucuagcuguUu....................................................................... 1 1 Br1
....................ucuuGgguuaucuagcuguau....................................................................... 16 1 Br1
....................ucuuugguuaucuaCcuguau....................................................................... 2 1 Br1
....................ucuGugguuaucuagcuguau....................................................................... 4 1 Br1
....................ucuuugguuaucuagcuguaGg...................................................................... 1 1 Br1
....................ucGuugguuaucuagcuguaug...................................................................... 1 1 Br1
....................ucuuugguuaucuagcuguauA...................................................................... 15 1 Br1
....................ucuuugguuaucuagcuguauU...................................................................... 5 1 Br1
....................ucuuugguuauGuagcuguaug...................................................................... 1 1 Br1
....................ucuuugguAaucuagcuguaug...................................................................... 4 1 Br1
....................ucuuugguuaucCagcuguaug...................................................................... 1 1 Br1
....................ucuuugguuaucuUgcuguaug...................................................................... 2 1 Br1
....................ucuuugAuuaucuagcuguaug...................................................................... 2 1 Br1
....................ucuuuggGuaucuagcuguaug...................................................................... 1 1 Br1
....................ucuuugguuaucGagcuguaug...................................................................... 1 1 Br1
....................ucuuAgguuaucuagcuguaug...................................................................... 15 1 Br1
....................ucuGugguuaucuagcuguaug...................................................................... 13 1 Br1
....................ucuuCgguuaucuagcuguaug...................................................................... 10 1 Br1
....................uAuuugguuaucuagcuguaug...................................................................... 4 1 Br1
....................ucuAugguuaucuagcuguaug...................................................................... 1 1 Br1
....................ucuuugguuaucuagcuUuaug...................................................................... 2 1 Br1
....................ucuuugguuaucuagcuguUug...................................................................... 1 1 Br1
....................ucuuGgguuaucuagcuguaug...................................................................... 63 1 Br1
....................ucCuugguuaucuagcuguaug...................................................................... 6 1 Br1
....................ucuuugguuaucuCgcuguaug...................................................................... 4 1 Br1
....................ucuuuUguuaucuagcuguaug...................................................................... 4 1 Br1
....................ucuuugguuCucuagcuguaug...................................................................... 4 1 Br1
....................ucuuugCuuaucuagcuguaug...................................................................... 1 1 Br1
....................ucuuugguuaucuagUuguaug...................................................................... 3 1 Br1
....................ucuuugguuaucuaUcuguaug...................................................................... 5 1 Br1
....................ucuuugguuaucuagcuAuaug...................................................................... 2 1 Br1
....................ucuuugguuaucuagAuguaug...................................................................... 2 1 Br1
....................ucuuugUuuaucuagcuguaug...................................................................... 5 1 Br1
....................Ccuuugguuaucuagcuguaug...................................................................... 2 1 Br1
....................ucuuugguuaucuagcuguaug...................................................................... 1552 0 Br1
....................ucuCugguuaucuagcuguaug...................................................................... 2 1 Br1
....................Acuuugguuaucuagcuguaug...................................................................... 12 1 Br1
....................ucuuugguuaucuagcuguauAa..................................................................... 1 1 Br1
....................ucCuugguuaucuagcuguauga..................................................................... 4 1 Br1
....................ucuuugguuaucuagcAguauga..................................................................... 1 1 Br1
....................ucuuugguuCucuagcuguauga..................................................................... 3 1 Br1
....................ucuuugguuaucuagcuguaugG..................................................................... 1 1 Br1
....................ucuuugguuaucuagcugGauga..................................................................... 1 1 Br1
....................ucuuugguuaucuagUuguauga..................................................................... 1 1 Br1
....................ucuuugguuaucuagcuguaugU..................................................................... 50 1 Br1
....................ucuuugguuaucuagcuguaugC..................................................................... 11 1 Br1
....................ucuuugguuaucuGgcuguauga..................................................................... 1 1 Br1
....................ucuuAgguuaucuagcuguauga..................................................................... 3 1 Br1
....................uAuuugguuaucuagcuguauga..................................................................... 2 1 Br1
....................ucuuugguuaucuagcuguauga..................................................................... 618 0 Br1
....................ucuuugguuaucuagAuguauga..................................................................... 1 1 Br1
....................ucuuugguAaucuagcuguauga..................................................................... 1 1 Br1
....................ucuuugguuaucuagcuguauCa..................................................................... 1 1 Br1
....................ucuuGgguuaucuagcuguauga..................................................................... 28 1 Br1
....................Gcuuugguuaucuagcuguauga..................................................................... 2 1 Br1
....................ucuuugguuaucuaUcuguauga..................................................................... 1 1 Br1
....................ucuuCgguuaucuagcuguauga..................................................................... 2 1 Br1
....................uUuuugguuaucuagcuguauga..................................................................... 1 1 Br1
....................ucuAugguuaucuagcuguauga..................................................................... 1 1 Br1
....................ucuGugguuaucuagcuguauga..................................................................... 1 1 Br1
....................ucuuuUguuaucuagcuguauga..................................................................... 2 1 Br1
....................Acuuugguuaucuagcuguauga..................................................................... 5 1 Br1
```

Mature Star

```
gucggcaggcauugauguuuucuuugguuaucuagcuguaugagugugacugacgucauaaagcuagguuaccaaaguuaaaaacaaucgccagaaaucagcuggaugacag
....................ucuuugguuaucuagcuguauUa.....................................................................    1      1    Br1
....................ucuuugguuaucuagcuguaugaC....................................................................    2      1    Br1
....................ucuuugguuaucuagcuguaugaU....................................................................    60     1    Br1
....................ucuuugguuaucuagcuguaugaA....................................................................    55     1    Br1
....................uAuuugguuaucuagcuguaugag....................................................................    1      1    Br1
....................ucuuugguuaucuagcuguaugagA...................................................................    1      1    Br1
....................ucuuugguuaucuagcuguaugaUu...................................................................    6      1    Br1
....................ucuuugguuaucuagcuguaugaCu...................................................................    1      1    Br1
....................ucuuugguuaucuagcuguaugaAu...................................................................    1      1    Br1
....................ucuuGgguuaucuagcuguaugagu...................................................................    1      1    Br1
....................ucuuugguuaucuagcuguaugagu...................................................................    1      0    Br1
....................ucuuugguuaucuagcuguaugaguA..................................................................    1      1    Br1
......................uugguuaucuagcuguaug.......................................................................    1      0    Br1
......................uugguuaucuagcuguauga......................................................................    2      0    Br1
.......................ugguuaucuagcuguaug.......................................................................    2      0    Br1
.........................................................auaaagcuagguuaccaaagu..................................    1      0    Br1
.........................................................auaaagcuagguuaccaaaguu.................................    4      0    Br1
.........................................................auaaagcuagguuaccaaaguua................................    1      0    Br1
..........................................................uaaagcuagguuaccaaaguu.................................    4      0    Br1
..........................................................uaaagcuagguuaccaaaguA.................................    1      1    Br1
..........................................................uaaagcuagguuaccaaaguua................................    2      0    Br1
..........................................................uaaaUcuagguuaccaaaguua................................    1      1    Br1

..................uuucuuugguuaucuagcugua........................................................................    1      0    Le5
...................Aucuuugguuaucuagcugu.........................................................................    1      1    Le5
...................Gucuuugguuaucuagcugu.........................................................................    1      1    Le5
...................Aucuuugguuaucuagcugua........................................................................    5      1    Le5
...................Cucuuugguuaucuagcugua........................................................................    5      1    Le5
...................uucuuugguuaucuagcugua........................................................................    1      0    Le5
...................Aucuuugguuaucuagcuguau.......................................................................    3      1    Le5
...................Cucuuugguuaucuagcuguau.......................................................................    8      1    Le5
...................Aucuuugguuaucuagcuguaug......................................................................    6      1    Le5
...................Cucuuugguuaucuagcuguaug......................................................................    33     1    Le5
...................Cucuuugguuaucuagcuguauga.....................................................................    2      1    Le5
....................ucuuGgguuaucuagcug..........................................................................    1      1    Le5
....................ucuuugguuaucuagcug..........................................................................    8      0    Le5
....................ucuuCgguuaucuagcugu.........................................................................    4      1    Le5
....................Ncuuugguuaucuagcugu.........................................................................    2      1    Le5
....................ucuuGgguuaucuagcugu.........................................................................    51     1    Le5
....................ucCuugguuaucuagcugu.........................................................................    3      1    Le5
....................ucuuugguuCucuagcugu.........................................................................    5      1    Le5
....................ucuuugguuaucuagcugC.........................................................................    70     1    Le5
....................ucuuugguuaucCagcugu.........................................................................    1      1    Le5
....................ucuuugUuuaucuagcugu.........................................................................    2      1    Le5
....................ucuuugguuaucuagcugu.........................................................................    1008   0    Le5
....................Acuuugguuaucuagcugu.........................................................................    3      1    Le5
....................ucuuugguuauUuagcugu.........................................................................    2      1    Le5
....................ucuuugguuaucuagcugA.........................................................................    1      1    Le5
....................ucuuAgguuaucuagcugu.........................................................................    5      1    Le5
....................ucuuugguuaAcuagcugu.........................................................................    1      1    Le5
....................ucuAugguuaucuagcugu.........................................................................    2      1    Le5
....................uAuuugguuaucuagcugu.........................................................................    4      1    Le5
....................Ccuuugguuaucuagcugu.........................................................................    2      1    Le5
....................ucuGugguuaucuagcugu.........................................................................    14     1    Le5
....................ucuuugguuaucuCgcugu.........................................................................    1      1    Le5
....................ucuuugguuaucuagcAgua........................................................................    3      1    Le5
....................ucuuugCuuaucuagcugua........................................................................    2      1    Le5
....................ucuuugguuaucuagcugua........................................................................    17362  0    Le5
....................ucuuugguGaucuagcugua........................................................................    1      1    Le5
....................ucuCugguuaucuagcugua........................................................................    11     1    Le5
....................ucuuugguuaAcuagcugua........................................................................    3      1    Le5
....................ucuuugguuaucuagcugGa........................................................................    3      1    Le5
....................ucuGugguuaucuagcugua........................................................................    146    1    Le5
....................ucuuuggCuaucuagcugua........................................................................    3      1    Le5
....................ucuuugAuuaucuagcugua........................................................................    3      1    Le5
....................ucuuugguuaucuagUugua........................................................................    10     1    Le5
....................ucuuugguuaucuCgcugua........................................................................    31     1    Le5
....................ucuuugguuaCcuagcugua........................................................................    2      1    Le5
....................Acuuugguuaucuagcugua........................................................................    36     1    Le5
....................ucuuugguuaucuagcugAa........................................................................    1      1    Le5
```

Mature Star

```
gucggcaggcauugauguuuucuuugguuaucuagcuguaugagugugacugacgucauaaagcuagguuaccaaaguuaaaaacaaucgccagaaaucagcuggaugacag
....................ucuuugguuaucuagcuAua........................................................................ 5 1 Le5
....................ucuuugguuauUuagcugua........................................................................ 8 1 Le5
....................ucCuugguuaucuagcugua........................................................................ 45 1 Le5
....................ucuuugguuaucuagcugCa........................................................................ 24 1 Le5
....................ucuuuggGuaucuagcugua........................................................................ 4 1 Le5
....................ucuuCgguuaucuagcugua........................................................................ 114 1 Le5
....................ucuuugguuCucuagcugua........................................................................ 55 1 Le5
....................ucuuugguuaucuaAcugua........................................................................ 3 1 Le5
....................uGuuugguuaucuagcugua........................................................................ 2 1 Le5
....................ucuuugguuaucuagcuUua........................................................................ 10 1 Le5
....................ucAuugguuaucuagcugua........................................................................ 2 1 Le5
....................ucGuugguuaucuagcugua........................................................................ 1 1 Le5
....................ucuuugguuaucuagcCgua........................................................................ 4 1 Le5
....................ucuuugguuaucuaUcugua........................................................................ 36 1 Le5
....................ucuuGgguuaucuagcugua........................................................................ 732 1 Le5
....................ucuuugguuaucuagcGgua........................................................................ 1 1 Le5
....................ucuuugguuaucuagcuguU........................................................................ 3 1 Le5
....................ucuuugguuaucCagcugua........................................................................ 5 1 Le5
....................ucuuugguuauGuagcugua........................................................................ 1 1 Le5
....................ucuuugguuaucuUgcugua........................................................................ 8 1 Le5
....................ucuuugguuaucuagcuguC........................................................................ 3 1 Le5
....................ucuuuggAuaucuagcugua........................................................................ 5 1 Le5
....................ucuuugguuaucuGgcugua........................................................................ 3 1 Le5
....................Ccuuugguuaucuagcugua........................................................................ 15 1 Le5
....................Ncuuugguuaucuagcugua........................................................................ 9 1 Le5
....................ucuuugguuaucuaCcugua........................................................................ 2 1 Le5
....................ucuuugguuGucuagcugua........................................................................ 6 1 Le5
....................uAuuugguuaucuagcugua........................................................................ 28 1 Le5
....................ucuuuCguuaucuagcugua........................................................................ 1 1 Le5
....................ucuAugguuaucuagcugua........................................................................ 20 1 Le5
....................ucuuugguAaucuagcugua........................................................................ 13 1 Le5
....................ucuuugUuuaucuagcugua........................................................................ 20 1 Le5
....................ucuuAgguuaucuagcugua........................................................................ 122 1 Le5
....................ucuuugguCaucuagcugua........................................................................ 9 1 Le5
....................ucuuuUguuaucuagcugua........................................................................ 5 1 Le5
....................Gcuuugguuaucuagcugua........................................................................ 6 1 Le5
....................ucuuugguuaucuagcuguG........................................................................ 18 1 Le5
....................ucuuCgguuaucuagcuguau....................................................................... 172 1 Le5
....................ucuuugguuaucuaUcuguau....................................................................... 86 1 Le5
....................ucuuugguuaGcuagcuguau....................................................................... 1 1 Le5
....................ucuuugguuauAuagcuguau....................................................................... 1 1 Le5
....................ucuuugguuCucuagcuguau....................................................................... 75 1 Le5
....................ucuuuggGuaucuagcuguau....................................................................... 3 1 Le5
....................ucuuugguuaucuagcuguGu....................................................................... 9 1 Le5
....................uGuuugguuaucuagcuguau....................................................................... 4 1 Le5
....................ucuuugguuauUuagcuguau....................................................................... 11 1 Le5
....................ucuuugguuaucuagcuguaC....................................................................... 437 1 Le5
....................ucuuugguuaucuagcuAuau....................................................................... 1 1 Le5
....................ucuuugguuaucuaCcuguau....................................................................... 1 1 Le5
....................Ncuuugguuaucuagcuguau....................................................................... 14 1 Le5
....................ucuuugguuaucuUgcuguau....................................................................... 22 1 Le5
....................ucuuugguuaucuaAcuguau....................................................................... 4 1 Le5
....................ucuuugguuaucuagcuguUu....................................................................... 10 1 Le5
....................ucuuugguuaucuagcuCuau....................................................................... 1 1 Le5
....................ucuuugguuaucAagcuguau....................................................................... 2 1 Le5
....................ucuuugguuaucuagcuUuau....................................................................... 15 1 Le5
....................ucuugCuuaucuagcuguau....................................................................... 6 1 Le5
....................ucuuugguuaCcuagcuguau....................................................................... 7 1 Le5
....................ucuuggCuaucuagcuguau....................................................................... 9 1 Le5
....................ucuuugguuaucuagcuguaG....................................................................... 73 1 Le5
....................ucuuugguuaucuagcuguaA....................................................................... 710 1 Le5
....................ucuuugguuaucCagcuguau....................................................................... 4 1 Le5
....................ucuuugUuuaucuagcuguau....................................................................... 51 1 Le5
....................Acuuugguuaucuagcuguau....................................................................... 47 1 Le5
....................ucuuugguuaucuagcGguau....................................................................... 3 1 Le5
....................ucuuugguuaucuagcuguau....................................................................... 27617 0 Le5
....................ucuCugguuaucuagcuguau....................................................................... 23 1 Le5
....................ucuuugguuaucuCgcuguau....................................................................... 34 1 Le5
....................ucuAugguuaucuagcuguau....................................................................... 31 1 Le5
....................ucGuugguuaucuagcuguau....................................................................... 2 1 Le5
```

```
                    Mature                         Star
gucggcaggcauugauguuuucuuugguuaucuagcuguaugagugugacugacgucauaaagcuagguuaccaaaguuaaaaacaaucgccagaaaucagcuggaugacag
....................ucuuuggAuaucuagcuguau.......................................................................   1      1    Le5
....................ucuuGgguuaucuagcuguau.......................................................................   1165   1    Le5
....................ucuuugguuGucuagcuguau.......................................................................   4      1    Le5
....................ucuGugguuaucuagcuguau.......................................................................   211    1    Le5
....................ucuuugAuuaucuagcuguau.......................................................................   6      1    Le5
....................Ccuuugguuaucuagcuguau.......................................................................   27     1    Le5
....................ucuuugguuaucuGgcuguau.......................................................................   9      1    Le5
....................ucuuugguuaucuagcuguCu.......................................................................   5      1    Le5
....................ucAuugguuaucuagcuguau.......................................................................   2      1    Le5
....................ucuuugguAaucuagcuguau.......................................................................   21     1    Le5
....................ucuuugguuauGuagcuguau.......................................................................   2      1    Le5
....................ucuuugguuaucuagcCguau.......................................................................   5      1    Le5
....................Gcuuugguuaucuagcuguau.......................................................................   8      1    Le5
....................ucuuuAguuaucuagcuguau.......................................................................   1      1    Le5
....................ucuuuCguuaucuagcuguau.......................................................................   1      1    Le5
....................ucuuugguuaucuagcAguau.......................................................................   1      1    Le5
....................ucuuugguuaucuagUuguau.......................................................................   32     1    Le5
....................uAuuugguuaucuagcuguau.......................................................................   39     1    Le5
....................ucuuAgguuaucuagcuguau.......................................................................   200    1    Le5
....................ucuuugguuaucuagAuguau.......................................................................   3      1    Le5
....................ucuuugguuaAcuagcuguau.......................................................................   5      1    Le5
....................ucuuugguuaucuagGuguau.......................................................................   2      1    Le5
....................ucuuugguuaucuagcugGau.......................................................................   11     1    Le5
....................ucuuugguuaucuagcugAau.......................................................................   3      1    Le5
....................ucuuugguuaucuagcugCau.......................................................................   2      1    Le5
....................ucuuugguCaucuagcuguau.......................................................................   12     1    Le5
....................ucCuugguuaucuagcuguau.......................................................................   93     1    Le5
....................ucuuuUguuaucuagcuguau.......................................................................   13     1    Le5
....................ucuuugguuaucuagcuguaCg......................................................................   28     1    Le5
....................ucuuuggAuaucuagcuguaug......................................................................   9      1    Le5
....................uAuuugguuaucuagcuguaug......................................................................   132    1    Le5
....................ucuuugguuaucuagcuguauA......................................................................   693    1    Le5
....................ucuuugguuGucuagcuguaug......................................................................   15     1    Le5
....................ucuuugguuaucuaCcuguaug......................................................................   7      1    Le5
....................ucuuugguuaucuagcugCaug......................................................................   17     1    Le5
....................ucuCugguuaucuagcuguaug......................................................................   93     1    Le5
....................ucuuuggCuaucuagcuguaug......................................................................   15     1    Le5
....................ucuuCgguuaucuagcuguaug......................................................................   688    1    Le5
....................ucuuuggGuaucuagcuguaug......................................................................   16     1    Le5
....................ucCuugguuaucuagcuguaug......................................................................   268    1    Le5
....................ucuuugguuaCcuagcuguaug......................................................................   15     1    Le5
....................ucuuAgguuaucuagcuguaug......................................................................   817    1    Le5
....................ucuuugguuauGuagcuguaug......................................................................   9      1    Le5
....................ucuuugUuuaucuagcuguaug......................................................................   124    1    Le5
....................ucGuugguuaucuagcuguaug......................................................................   3      1    Le5
....................ucuuugguuaucuagcuUuaug......................................................................   61     1    Le5
....................ucuuuAguuaucuagcuguaug......................................................................   7      1    Le5
....................ucuuugguuaucuagcuguCug......................................................................   11     1    Le5
....................ucuuugguuaAcuagcuguaug......................................................................   18     1    Le5
....................ucuuugguuauAuagcuguaug......................................................................   3      1    Le5
....................ucuuugguuaucuagcugGaug......................................................................   38     1    Le5
....................ucuuugguuaucuagcCguaug......................................................................   24     1    Le5
....................ucuuugguuaucCagcuguaug......................................................................   11     1    Le5
....................ucuuugguuaucAagcuguaug......................................................................   13     1    Le5
....................ucuuugCuuaucuagcuguaug......................................................................   7      1    Le5
....................ucuuugguuaucuagUuguaug......................................................................   89     1    Le5
....................ucuuugguuaucuagcuguUug......................................................................   46     1    Le5
....................ucuuugguuaucuCgcuguaug......................................................................   111    1    Le5
....................Acuuugguuaucuagcuguaug......................................................................   174    1    Le5
....................ucuuugguuCucuagcuguaug......................................................................   285    1    Le5
....................ucuuugAuuaucuagcuguaug......................................................................   18     1    Le5
....................ucuuGgguuaucuagcuguaug......................................................................   4425   1    Le5
....................ucuuugguuaucuagcuAuaug......................................................................   16     1    Le5
....................ucuuugguuauUuagcuguaug......................................................................   35     1    Le5
....................ucuuugguuaucuUgcuguaug......................................................................   37     1    Le5
....................ucuuugguGaucuagcuguaug......................................................................   4      1    Le5
....................ucuAugguuaucuagcuguaug......................................................................   118    1    Le5
....................ucuuugguuaucuagAuguaug......................................................................   7      1    Le5
....................Ncuuugguuaucuagcuguaug......................................................................   72     1    Le5
....................ucuuugguuaucuagcuguGug......................................................................   41     1    Le5
```

Mature Star

```
gucggcaggcauugauguuuucuuugguuaucuagcuguaugagugugacugacgucauaaagcuagguuaccaaaguuaaaaacaaucgccagaaaucagcuggaugacag
....................ucuuugguuaucuagcuguauU...................................................................... 500 1 Le5
....................ucuGgguuaucuagcuguaug...................................................................... 767 1 Le5
....................ucuuugguuaGcuagcuguaug...................................................................... 1 1 Le5
....................ucuuuCguuaucuagcuguaug...................................................................... 4 1 Le5
....................ucuuugguuaucuagcuCuaug...................................................................... 1 1 Le5
....................ucuuugguuaucGagcuguaug...................................................................... 6 1 Le5
....................uUuuugguuaucuagcuguaug...................................................................... 55 1 Le5
....................ucuuuUguuaucuagcuguaug...................................................................... 65 1 Le5
....................uGuuugguuaucuagcuguaug...................................................................... 5 1 Le5
....................ucuuugguuaucuagcuguaGg...................................................................... 7 1 Le5
....................ucuuugguuaucuaAcuguaug...................................................................... 15 1 Le5
....................Ccuuugguuaucuagcuguaug...................................................................... 134 1 Le5
....................ucuuugguuaucuagcuguaug...................................................................... 107994 0 Le5
....................ucuuugguuaucuagcuguaAg...................................................................... 14 1 Le5
....................ucuuugguuaucuaUcuguaug...................................................................... 225 1 Le5
....................ucuuugguCaucuagcuguaug...................................................................... 40 1 Le5
....................Gcuuugguuaucuagcuguaug...................................................................... 24 1 Le5
....................ucuuugguuaucuagcugAaug...................................................................... 4 1 Le5
....................ucuuugguAaucuagcuguaug...................................................................... 84 1 Le5
....................ucuuugguuaucuagcAguaug...................................................................... 8 1 Le5
....................ucuuugguuaucuGgcuguaug...................................................................... 21 1 Le5
....................ucAuugguuaucuagcuguaug...................................................................... 2 1 Le5
....................ucuuugguuaucuagcGguaug...................................................................... 2 1 Le5
....................ucuuugguuaucuagGuguaug...................................................................... 2 1 Le5
....................ucuuugguuaucuagcuguauC...................................................................... 15 1 Le5
....................uAuuugguuaucuagcuguauga..................................................................... 34 1 Le5
....................ucuuugguuaucAagcuguauga..................................................................... 1 1 Le5
....................ucuuugguuaucuagcuguUuga..................................................................... 5 1 Le5
....................ucuuugguuaucuagUuguauga..................................................................... 17 1 Le5
....................ucGuugguuaucuagcuguauga..................................................................... 1 1 Le5
....................ucuuuAguuaucuagcuguauga..................................................................... 2 1 Le5
....................ucuuuUguuaucuagcuguauga..................................................................... 11 1 Le5
....................ucuuugguuaucuagAuguauga..................................................................... 1 1 Le5
....................ucuuugguuaucuagcuguaCga..................................................................... 3 1 Le5
....................ucuuugguuaucuagcuguaugC..................................................................... 403 1 Le5
....................ucuuugguuaucuagcugAauga..................................................................... 5 1 Le5
....................ucuuugguuaGcuagcuguauga..................................................................... 3 1 Le5
....................ucuuugguuGucuagcuguauga..................................................................... 4 1 Le5
....................ucuuugguuaucuagcuguauga..................................................................... 20656 0 Le5
....................ucuuugAuuaucuagcuguauga..................................................................... 3 1 Le5
....................ucuCgguuaucuagcuguauga..................................................................... 14 1 Le5
....................ucuuugguuaAcuagcuguauga..................................................................... 4 1 Le5
....................ucuAgguuaucuagcuguauga..................................................................... 23 1 Le5
....................ucuuugguuaucuagcuUuauga..................................................................... 11 1 Le5
....................ucuuugguuauGuagcuguauga..................................................................... 1 1 Le5
....................uUuuugguuaucuagcuguauga..................................................................... 14 1 Le5
....................ucuuugguuaucuagcuguaugU..................................................................... 4379 1 Le5
....................ucuuugguuauUuagcuguauga..................................................................... 2 1 Le5
....................ucuuCgguuaucuagcuguauga..................................................................... 140 1 Le5
....................ucCuugguuaucuagcuguauga..................................................................... 56 1 Le5
....................ucuuugguuaucuagcGguauga..................................................................... 1 1 Le5
....................ucuuugguuaucuagcuguaAga..................................................................... 1 1 Le5
....................ucAuugguuaucuagcuguauga..................................................................... 1 1 Le5
....................ucuuugguuaucuagGuguauga..................................................................... 1 1 Le5
....................ucuuugguuaucuagcuguCuga..................................................................... 1 1 Le5
....................ucuuugguuaucuCgcuguauga..................................................................... 24 1 Le5
....................ucuuugCuuaucuagcuguauga..................................................................... 1 1 Le5
....................ucuuugguuaucuaCcuguauga..................................................................... 2 1 Le5
....................ucuuugguuCucuagcuguauga..................................................................... 60 1 Le5
....................ucuuugguuaucuagcuguaGga..................................................................... 1 1 Le5
....................ucuGgguuaucuagcuguauga..................................................................... 167 1 Le5
....................ucuuugguuaucuaAcuguauga..................................................................... 1 1 Le5
....................ucuuuggCuaucuagcuguauga..................................................................... 5 1 Le5
....................ucuuugguuaucuUgcuguauga..................................................................... 8 1 Le5
....................ucuuugguuaucuagcAguauga..................................................................... 2 1 Le5
....................ucuuugguuaucuagcCguauga..................................................................... 4 1 Le5
....................Gcuuugguuaucuagcuguauga..................................................................... 7 1 Le5
....................ucuuugguCaucuagcuguauga..................................................................... 15 1 Le5
....................ucuuugguAaucuagcuguauga..................................................................... 23 1 Le5
....................ucuuugguuaucuagcugCauga..................................................................... 4 1 Le5
```

```
                    Mature                Star
gucggcaggcauugauguuuucuuugguuaucuagcuguaugagugugacugacgucauaaagcuagguuaccaaaguuaaaaacaaucgccagaaaucagcuggaugacag
....................ucuuGgguuaucuagcuguauga.....................................................................   867    1   Le5
....................Acuuugguuaucuagcuguauga.....................................................................   34     1   Le5
....................ucuuugguuaucuaUcuguauga.....................................................................   59     1   Le5
....................ucuuugguuaucuagcuguaugG.....................................................................   73     1   Le5
....................ucuuuggGuaucuagcuguauga.....................................................................   3      1   Le5
....................ucuuugguuaucCagcuguauga.....................................................................   3      1   Le5
....................Ncuuugguuaucuagcuguauga.....................................................................   21     1   Le5
....................ucuuugguuaucuagcuguauAa.....................................................................   125    1   Le5
....................ucuuugguuaucuagcuAuauga.....................................................................   3      1   Le5
....................ucuuugguuaucuGgcuguauga.....................................................................   4      1   Le5
....................uGuuugguuaucuagcuguauga.....................................................................   1      1   Le5
....................Ccuuugguuaucuagcuguauga.....................................................................   27     1   Le5
....................ucuuugguuaCcuagcuguauga.....................................................................   1      1   Le5
....................ucuuugguGaucuagcuguauga.....................................................................   5      1   Le5
....................ucuuuggAuaucuagcuguauga.....................................................................   1      1   Le5
....................ucuuugguuaucuagcugGauga.....................................................................   2      1   Le5
....................ucuuuCguuaucuagcuguauga.....................................................................   2      1   Le5
....................ucuuugguuaucuagcuguGuga.....................................................................   12     1   Le5
....................ucuuugguuaucuagcuguauUa.....................................................................   25     1   Le5
....................ucuuAgguuaucuagcuguauga.....................................................................   148    1   Le5
....................ucuuugUuuaucuagcuguauga.....................................................................   34     1   Le5
....................ucuuugguuaucuagcuguaugag....................................................................   47     0   Le5
....................ucuuGgguuaucuagcuguaugag....................................................................   1      1   Le5
....................ucuuugguuaucuagcuguaugaA....................................................................   1124   1   Le5
....................ucuuugguuaucuagcuguCugag....................................................................   1      1   Le5
....................ucuuugguuaucuagcuguaugUg....................................................................   26     1   Le5
....................ucuuugguuaucuagcuguaugaU....................................................................   1865   1   Le5
....................ucuuugguuaucuagcuguaugCg....................................................................   3      1   Le5
....................Acuuugguuaucuagcuguaugag....................................................................   1      1   Le5
....................ucuuugguuaucuagcuguaugaC....................................................................   72     1   Le5
....................ucuuAgguuaucuagcuguaugag....................................................................   1      1   Le5
....................ucuuugguuaucuagcuguaugaUu...................................................................   131    1   Le5
....................ucuuugguuaucuagcuguaugagA...................................................................   4      1   Le5
....................ucuuugguuaucuagcuguaugaCu...................................................................   18     1   Le5
....................ucuuugguuaucuagcuguaugUgu...................................................................   3      1   Le5
....................ucuuugguuaucuagcuguaugaAu...................................................................   86     1   Le5
....................ucuuugguuaucuagcuguaugagu...................................................................   5      0   Le5
....................ucuuugguuaucuagcuguauAagu...................................................................   1      1   Le5
....................ucuuGgguuaucuagcuguaugagu...................................................................   2      1   Le5
....................ucuuugguuaucuagcuguaugagug..................................................................   1      0   Le5
.....................cuuugUuuaucuagcugu.........................................................................   1      1   Le5
.....................cuuGgguuaucuagcugu.........................................................................   1      1   Le5
.....................cuuugguuaucuagcugua........................................................................   6      0   Le5
.....................cuuuUguuaucuagcugua........................................................................   1      1   Le5
.....................cuuugguuaucuagcuguau.......................................................................   4      0   Le5
.....................cuuugguuCucuagcuguau.......................................................................   1      1   Le5
.....................cuuugguuauUuagcuguau.......................................................................   1      1   Le5
.....................cuuugguuaAcuagcuguaug......................................................................   1      1   Le5
.....................cuuugguuaucuagcuguUug......................................................................   3      1   Le5
.....................cuuGgguuaucuagcuguaug......................................................................   2      1   Le5
.....................cuuugguuCucuagcuguaug......................................................................   5      1   Le5
.....................Uuuugguuaucuagcuguaug......................................................................   2      1   Le5
.....................cAuugguuaucuagcuguaug......................................................................   1      1   Le5
.....................cuuugguuaucuCgcuguaug......................................................................   1      1   Le5
.....................Auuugguuaucuagcuguaug......................................................................   1      1   Le5
.....................cuuugguuaucuagcugCaug......................................................................   1      1   Le5
.....................cuuugguuaucuagcuAuaug......................................................................   1      1   Le5
.....................cuuugguuaucuagcuguaug......................................................................   60     0   Le5
.....................cuuugguuauAuagcuguaug......................................................................   1      1   Le5
.....................cuuuUguuaucuagcuguaug......................................................................   12     1   Le5
.....................cuuugguuaucuagGuguaug......................................................................   1      1   Le5
.....................cuuugguuaucuagcugGaug......................................................................   2      1   Le5
.....................cuuugguuaucuUgcuguaug......................................................................   2      1   Le5
.....................cuuugguuaucuaUcuguaug......................................................................   2      1   Le5
.....................Auuugguuaucuagcuguauga.....................................................................   1      1   Le5
.....................Uuuugguuaucuagcuguauga.....................................................................   1      1   Le5
.....................cuuugguuCucuagcuguauga.....................................................................   1      1   Le5
.....................cuuugguuaucuagcuguaugU.....................................................................   3      1   Le5
.....................cuuugguuauAuagcuguauga.....................................................................   1      1   Le5
.....................cuuGgguuaucuagcuguauga.....................................................................   1      1   Le5
```

Mature   Star

```
gucggcaggcauugauguuuucuuugguuaucuagcuguaugagugugacugacgucauaaagcuagguuaccaaaguuaaaaacaaucgccagaaaucagcuggaugacag
.....................cuuuAguuaucuagcuguauga.....................................................................     1     1     Le5
.....................cuuuUguuaucuagcuguauga.....................................................................     3     1     Le5
.....................cuuugguuaucuagcuguauga.....................................................................     17    0     Le5
.....................cuCugguuaucuagcuguauga.....................................................................     1     1     Le5
.....................cuuugguuaucuagGuguauga.....................................................................     1     1     Le5
.....................cuuugguuaucuagcuguaugaC....................................................................     1     1     Le5
.....................cuuugguuaucuagcuguaugaA....................................................................     3     1     Le5
.....................cuuugguuaucuagcuguaugaU....................................................................     1     1     Le5
......................uuugguuaucuagcugua........................................................................     1     0     Le5
......................uuugguuaucuagAuguau.......................................................................     1     1     Le5
......................uuugguuaucuagcuguau.......................................................................     1     0     Le5
......................uuugUuuaucuagcuguaug......................................................................     1     1     Le5
......................uuuUguuaucuagcuguaug......................................................................     1     1     Le5
......................uuugguuaucuagcuguaug......................................................................     41    0     Le5
......................Nuugguuaucuagcuguauga.....................................................................     1     1     Le5
......................uuugguuaucuagcuguaugC.....................................................................     1     1     Le5
......................uuugguuaucuagcuguaugU.....................................................................     1     1     Le5
......................uuugAuuaucuagcuguauga.....................................................................     1     1     Le5
......................uuugguuaucuagcuguauga.....................................................................     39    0     Le5
......................uuugUuuaucuagcuguauga.....................................................................     2     1     Le5
......................uuugguuaucuagcuguaugaA....................................................................     1     1     Le5
......................uuugguuaucuagcuguaugaAu...................................................................     1     1     Le5
.......................uuUguuaucuagcuguau.......................................................................     1     1     Le5
.......................uugguuaucuagcuguaug......................................................................     5     0     Le5
.......................uuUguuaucuagcuguaug......................................................................     1     1     Le5
.......................uugguuaucuagcuguauga.....................................................................     3     0     Le5
.......................uugguuaucuagcuguaugU.....................................................................     2     1     Le5
.......................uugguuaucuagcuguaugaU....................................................................     2     1     Le5
........................ugguuaucuagcuguaug......................................................................     4     0     Le5
........................ugguuaucuagcuguaugaU....................................................................     1     1     Le5
.........................gguuaucuagcuguauga.....................................................................     1     0     Le5
.........................................................auaaagcuagguuaccaaag...................................     1     0     Le5
.........................................................auaaagcuagguuaccaaagu..................................     9     0     Le5
.........................................................auaaagcuagguuaccaaaguu.................................     45    0     Le5
.........................................................Cuaaagcuagguuaccaaaguu.................................     1     1     Le5
.........................................................Uuaaagcuagguuaccaaaguu.................................     1     1     Le5
.........................................................auaaagcuagguuaAcaaaguu.................................     1     1     Le5
.........................................................auaaUgcuagguuaccaaaguu.................................     1     1     Le5
.........................................................auaaagcuagguuaccaaaguua................................     26    0     Le5
.........................................................auaaagcGagguuaccaaaguua................................     1     1     Le5
.........................................................auaaagcuagguuaccaaaguuU................................     1     1     Le5
.........................................................Uuaaagcuagguuaccaaaguua................................     1     1     Le5
.........................................................auaaagcuagguuaccaaaguuaU...............................     1     1     Le5
.........................................................auaaagcuagguuaccaaaguuaa...............................     2     0     Le5
..........................................................uaaagcuagguuaccaaag...................................     1     0     Le5
..........................................................uaaagcuagguuaccaaagu..................................     25    0     Le5
..........................................................uaaagcuagguuaccaaaguu.................................     148   0     Le5
..........................................................uaaagcuagguuaccaaaguA.................................     2     1     Le5
..........................................................uaaaAcuagguuaccaaaguu.................................     2     1     Le5
..........................................................uaaagcuagguuaccaaaguC.................................     3     1     Le5
..........................................................uaaagcuaAguuaccaaaguu.................................     1     1     Le5
..........................................................uaaaUcuagguuaccaaaguu.................................     4     1     Le5
..........................................................uaaagcuagguuaccaaaUuu.................................     1     1     Le5
..........................................................uaCagcuagguuaccaaaguu.................................     1     1     Le5
..........................................................uaaagcuagguuaccaaUguu.................................     1     1     Le5
..........................................................uaaaUcuagguuaccaaaguua................................     2     1     Le5
..........................................................uaaagcuagguuaccaaaguua................................     56    0     Le5
..........................................................uaaagcuagguuaccaaaguuaa...............................     1     0     Le5
...........................................................aaagcuagguuaccaaagu..................................     1     0     Le5
...........................................................Caagcuagguuaccaaaguu.................................     1     1     Le5
...........................................................aaagcuagguuaccaaaguu.................................     3     0     Le5
...........................................................aaagcuagCuuaccaaaguua................................     1     1     Le5
...........................................................aaagcuagguuaccaaaguua................................     2     0     Le5

...................Cucuuugguuaucuagcug..........................................................................     2     1     Ta1
...................Cucuuugguuaucuagcugua........................................................................     3     1     Ta1
...................Cucuuugguuaucuagcuguau.......................................................................     9     1     Ta1
...................Aucuuugguuaucuagcuguau.......................................................................     1     1     Ta1
...................Cucuuugguuaucuagcuguaug......................................................................     3     1     Ta1
...................Cucuuugguuaucuagcuguauga.....................................................................     3     1     Ta1
```

Mature **Star**

gucggcaggcauugauguuuucuuugguuaucuagcuguaugagugugacugacgucauaaagcuagguuaccaaaguuaaaaacaaucgccagaaaucagcuggaugacag			
....................ucuuugguuaucuGgcug..	1	1	Ta1
....................ucuuugguuaucuagcug..	12	0	Ta1
....................ucuuGgguuaucuagcug..	1	1	Ta1
....................ucuuugguuauGuagcugu...	1	1	Ta1
....................ucuGugguuaucuagcugu...	1	1	Ta1
....................ucuuugguuaucuaUcugu...	2	1	Ta1
....................ucuuugguuaucuagcugu...	213	0	Ta1
....................ucuuuUguuaucuagcugu...	1	1	Ta1
....................ucuuugguuaucuagcuUu...	1	1	Ta1
....................uAuuugguuaucuagcugu...	1	1	Ta1
....................ucCuugguuaucuagcugu...	2	1	Ta1
....................ucuuugguuaucuagcugC...	3	1	Ta1
....................ucuuugguuaucuagUugu...	1	1	Ta1
....................ucuuGgguuaucuagcugu...	14	1	Ta1
....................ucuuCgguuaucuagcugu...	2	1	Ta1
....................ucuuAgguuaucuagcugu...	1	1	Ta1
....................ucuuugguuaucuagcuguG..	5	1	Ta1
....................ucuuuggGuaucuagcugua..	1	1	Ta1
....................ucuuugguuaucuagcuguU..	4	1	Ta1
....................ucuuugguuaucuaUcugua..	6	1	Ta1
....................ucuCugguuaucuagcugua..	12	1	Ta1
....................ucuuugguuaucuagcugCa..	1	1	Ta1
....................ucuuugguuaucuCgcugua..	3	1	Ta1
....................ucuuugCuuaucuagcugua..	2	1	Ta1
....................ucuuCgguuaucuagcugua..	24	1	Ta1
....................ucuuugguuauUuagcugua..	1	1	Ta1
....................Gcuuugguuaucuagcugua..	2	1	Ta1
....................ucuuuggCuaucuagcugua..	1	1	Ta1
....................ucuuugguuaucuaCcugua..	1	1	Ta1
....................ucuuugguuaucuaAcugua..	1	1	Ta1
....................ucuuugguuaAcuagcugua..	1	1	Ta1
....................ucuGugguuaucuagcugua..	35	1	Ta1
....................Ccuuugguuaucuagcugua..	5	1	Ta1
....................ucuuugguuaucuagcugua..	3709	0	Ta1
....................uAuuugguuaucuagcugua..	3	1	Ta1
....................ucuuuAguuaucuagcugua..	1	1	Ta1
....................ucuuugguuaucuagcuUua..	5	1	Ta1
....................ucuuugguCaucuagcugua..	1	1	Ta1
....................Ncuuugguuaucuagcugua..	3	1	Ta1
....................ucuuGgguuaucuagcugua..	150	1	Ta1
....................ucuuugUuuaucuagcugua..	5	1	Ta1
....................ucAuugguuaucuagcugua..	1	1	Ta1
....................ucuuugguuCucuagcugua..	9	1	Ta1
....................ucuuugguuaucuagcCgua..	2	1	Ta1
....................ucuuugguuaucuagcuguC..	1	1	Ta1
....................ucuuugguAaucuagcugua..	2	1	Ta1
....................ucuuugguuaucCagcugua..	1	1	Ta1
....................ucuuugguuGucuagcugua..	1	1	Ta1
....................ucuuugguuaucuagcuAua..	2	1	Ta1
....................ucuuuggAuaucuagcugua..	1	1	Ta1
....................ucuuugguuaucuagUugua..	2	1	Ta1
....................ucuuugguuaucuUgcugua..	3	1	Ta1
....................ucuuAgguuaucuagcugua..	31	1	Ta1
....................ucCuugguuaucuagcugua..	5	1	Ta1
....................ucuuuUguuaucuagcugua..	1	1	Ta1
....................ucuAugguuaucuagcugua..	9	1	Ta1
....................ucuuugguuaucuagcAgua..	2	1	Ta1
....................Acuuugguuaucuagcugua..	4	1	Ta1
....................ucuuugguuaucuagcuguCu...	1	1	Ta1
....................ucuuugguuaucuagUuguau...	4	1	Ta1
....................ucuuugguuaucuagcugGau...	5	1	Ta1
....................ucuuugguuaucuGgcuguau...	1	1	Ta1
....................ucuuugguAaucuagcuguau...	11	1	Ta1
....................ucuCugguuaucuagcuguau...	8	1	Ta1
....................ucuGugguuaucuagcuguau...	71	1	Ta1
....................ucuuugguuaAcuagcuguau...	4	1	Ta1
....................ucuuugguuauUuagcuguau...	5	1	Ta1
....................ucuuugguuaucuagcuguaC...	12	1	Ta1
....................ucuuugguuaucAagcuguau...	1	1	Ta1
....................ucuuugguuaucuagcuCuau...	2	1	Ta1

```
                    Mature                Star
gucggcaggcauugauguuuucuuugguuaucuagcuguaugagugugacugacgucauaaagcuagguuaccaaaguuaaaaacaaucgccagaaaucagcuggaugacag
....................Ccuuugguuaucuagcuguau.......................................................................   14     1   Ta1
....................ucuuuggCuaucuagcuguau.......................................................................   2      1   Ta1
....................Ncuuugguuaucuagcuguau.......................................................................   6      1   Ta1
....................ucuuugguuGucuagcuguau.......................................................................   4      1   Ta1
....................ucuuugguuauAuagcuguau.......................................................................   1      1   Ta1
....................ucuuAgguuaucuagcuguau.......................................................................   69     1   Ta1
....................ucCuugguuaucuagcuguau.......................................................................   19     1   Ta1
....................ucuuugguuaucuagcuguaG.......................................................................   27     1   Ta1
....................ucuuugguuaucuagcuguGu.......................................................................   2      1   Ta1
....................ucuuugguCaucuagcuguau.......................................................................   6      1   Ta1
....................Gcuuugguuaucuagcuguau.......................................................................   2      1   Ta1
....................ucuuugguuaucuagcuguau.......................................................................   8547   0   Ta1
....................ucuuugguuCucuagcuguau.......................................................................   27     1   Ta1
....................ucuuuUguuaucuagcuguau.......................................................................   3      1   Ta1
....................ucuuugguuaucuagcuguUu.......................................................................   1      1   Ta1
....................uAuuugguuaucuagcuguau.......................................................................   12     1   Ta1
....................ucuuugguuaucuUgcuguau.......................................................................   6      1   Ta1
....................ucuuugguuaucCagcuguau.......................................................................   1      1   Ta1
....................ucuuugUuuaucuagcuguau.......................................................................   18     1   Ta1
....................ucuuCgguuaucuagcuguau.......................................................................   57     1   Ta1
....................ucuuugguuaucuagcCguau.......................................................................   2      1   Ta1
....................ucuuugguuauGuagcuguau.......................................................................   3      1   Ta1
....................Acuuugguuaucuagcuguau.......................................................................   21     1   Ta1
....................ucuuugguuaucuaAcuguau.......................................................................   1      1   Ta1
....................ucuuugguuaucuagAuguau.......................................................................   1      1   Ta1
....................ucuuuAguuaucuagcuguau.......................................................................   1      1   Ta1
....................ucuuugCuuaucuagcuguau.......................................................................   1      1   Ta1
....................ucuAugguuaucuagcuguau.......................................................................   5      1   Ta1
....................ucuuGgguuaucuagcuguau.......................................................................   352    1   Ta1
....................ucuuugguuaucuCgcuguau.......................................................................   13     1   Ta1
....................ucuuugguuaucuagcuUuau.......................................................................   3      1   Ta1
....................ucuuugguuaCcuagcuguau.......................................................................   4      1   Ta1
....................ucuuugguGaucuagcuguau.......................................................................   1      1   Ta1
....................uGuuugguuaucuagcuguau.......................................................................   2      1   Ta1
....................ucuuugguuaucuaUcuguau.......................................................................   28     1   Ta1
....................ucuuugguuaucuagcuguaA.......................................................................   230    1   Ta1
....................uUuuugguuaucuagcuguaug......................................................................   7      1   Ta1
....................ucuuugguuaucuagcuUuaug......................................................................   11     1   Ta1
....................ucuuugguCaucuagcuguaug......................................................................   6      1   Ta1
....................ucuuugguuaGcuagcuguaug......................................................................   2      1   Ta1
....................Ncuuugguuaucuagcuguaug......................................................................   20     1   Ta1
....................Acuuugguuaucuagcuguaug......................................................................   63     1   Ta1
....................ucuuugguuaucuagUuguaug......................................................................   32     1   Ta1
....................ucuuugguuaucuUgcuguaug......................................................................   23     1   Ta1
....................ucuuuggGuaucuagcuguaug......................................................................   4      1   Ta1
....................uAuuugguuaucuagcuguaug......................................................................   34     1   Ta1
....................ucuuugguuaCcuagcuguaug......................................................................   6      1   Ta1
....................ucuCugguuaucuagcuguaug......................................................................   33     1   Ta1
....................ucuuugguuaucuagcuCuaug......................................................................   3      1   Ta1
....................ucuuugguuaucuagcCguaug......................................................................   9      1   Ta1
....................Ccuuugguuaucuagcuguaug......................................................................   26     1   Ta1
....................ucuuuggAuaucuagcuguaug......................................................................   2      1   Ta1
....................ucuuugguuaucuagcugCaug......................................................................   4      1   Ta1
....................ucuuugguuaucCagcuguaug......................................................................   5      1   Ta1
....................ucuuugguuauGuagcuguaug......................................................................   4      1   Ta1
....................ucuuugguuaucuGgcuguaug......................................................................   6      1   Ta1
....................ucuuugguuaucuagcuguUug......................................................................   6      1   Ta1
....................ucCuugguuaucuagcuguaug......................................................................   63     1   Ta1
....................ucuuugguuauAuagcuguaug......................................................................   3      1   Ta1
....................ucuuugguuaucuCgcuguaug......................................................................   33     1   Ta1
....................ucuuugguuaucuagcuguauC......................................................................   1      1   Ta1
....................ucuuuAguuaucuagcuguaug......................................................................   2      1   Ta1
....................ucuGugguuaucuagcuguaug......................................................................   196    1   Ta1
....................ucuuugguAaucuagcuguaug......................................................................   30     1   Ta1
....................ucuuugguuaucuagcuguaCg......................................................................   6      1   Ta1
....................ucuuugguuaucuagcuguauU......................................................................   176    1   Ta1
....................ucuuugUuuaucuagcuguaug......................................................................   40     1   Ta1
....................ucuuAgguuaucuagcuguaug......................................................................   196    1   Ta1
....................ucuuuUguuaucuagcuguaug......................................................................   13     1   Ta1
....................ucuuugguuaAcuagcuguaug......................................................................   16     1   Ta1
```

Mature Star

```
gucggcaggcauugauguuuucuuugguuaucuagcuguaugagugugacugacgucauaaagcuagguuaccaaaguuaaaaacaaucgccagaaaucagcuggaugacag
....................ucuuugguuaucuagcuguGug......................................................................  9      1  Ta1
....................ucuuugguuaucuagcuguaGg......................................................................  4      1  Ta1
....................ucuuugguuaucuagcuguaug......................................................................  28616  0  Ta1
....................ucuuugguuaucuagcuguaAg......................................................................  5      1  Ta1
....................ucuuugguuaucuagcuAuaug......................................................................  3      1  Ta1
....................ucuuugguuaucuaAcuguaug......................................................................  2      1  Ta1
....................ucuuugguuauUuagcuguaug......................................................................  7      1  Ta1
....................ucuuugguuaucuaUcuguaug......................................................................  70     1  Ta1
....................ucuuugguuGucuagcuguaug......................................................................  3      1  Ta1
....................ucuuugCuuaucuagcuguaug......................................................................  2      1  Ta1
....................ucuuugguuaucAagcuguaug......................................................................  6      1  Ta1
....................ucuuugguuaucuagcuguCug......................................................................  2      1  Ta1
....................ucuuugguuCucuagcuguaug......................................................................  80     1  Ta1
....................ucuuuggCuaucuagcuguaug......................................................................  4      1  Ta1
....................ucuuugguuaucuagcugGaug......................................................................  16     1  Ta1
....................Gcuuugguuaucuagcuguaug......................................................................  6      1  Ta1
....................ucuAugguuaucuagcuguaug......................................................................  45     1  Ta1
....................ucuuugguuaucuagcAguaug......................................................................  4      1  Ta1
....................ucuuugAuuaucuagcuguaug......................................................................  7      1  Ta1
....................ucuuGgguuaucuagcuguaug......................................................................  1189   1  Ta1
....................ucuuCgguuaucuagcuguaug......................................................................  167    1  Ta1
....................ucAuugguuaucuagcuguaug......................................................................  1      1  Ta1
....................ucuuugguuaucuagcuguauA......................................................................  193    1  Ta1
....................uGuuugguuaucuagcuguaug......................................................................  2      1  Ta1
....................ucuuugguuaucuagAuguaug......................................................................  2      1  Ta1
....................ucuuugguuaGcuagcuguauga.....................................................................  1      1  Ta1
....................ucuuugguuaucuagcAguauga.....................................................................  1      1  Ta1
....................ucuuugguuauGuagcuguauga.....................................................................  1      1  Ta1
....................ucuuugguuaucuagcuguauUa.....................................................................  3      1  Ta1
....................ucuuugguuaucAagcuguauga.....................................................................  1      1  Ta1
....................ucuCugguuaucuagcuguauga.....................................................................  6      1  Ta1
....................ucuuugguuaucuagcuguauga.....................................................................  4961   0  Ta1
....................ucuuugguuaucuagUuguauga.....................................................................  4      1  Ta1
....................ucuuugguuaucuGgcuguauga.....................................................................  1      1  Ta1
....................ucuuugguuaCcuagcuguauga.....................................................................  30     1  Ta1
....................ucuuugguuaucuCgcuguauga.....................................................................  5      1  Ta1
....................ucuuugguuaucCagcuguauga.....................................................................  3      1  Ta1
....................ucuuuUguuaucuagcuguauga.....................................................................  7      1  Ta1
....................ucuuugguuaucuagcuguauAa.....................................................................  41     1  Ta1
....................ucuuugguuGucuagcuguauga.....................................................................  1      1  Ta1
....................ucuuCgguuaucuagcuguauga.....................................................................  27     1  Ta1
....................ucuuuggGuaucuagcuguauga.....................................................................  2      1  Ta1
....................Gcuuugguuaucuagcuguauga.....................................................................  1      1  Ta1
....................Acuuugguuaucuagcuguauga.....................................................................  14     1  Ta1
....................ucuuugguuaucuagcuguaugG.....................................................................  14     1  Ta1
....................ucuuugguuaucuagcuUuauga.....................................................................  4      1  Ta1
....................ucuuugguuaucuagcuguauCa.....................................................................  1      1  Ta1
....................ucuGugguuaucuagcuguauga.....................................................................  37     1  Ta1
....................ucuuugguuaucuaAcuguauga.....................................................................  2      1  Ta1
....................ucuuugguuaucuagAuguauga.....................................................................  1      1  Ta1
....................ucuuugguuaucuagcuguUuga.....................................................................  1      1  Ta1
....................ucCuugguuaucuagcuguauga.....................................................................  8      1  Ta1
....................ucuuugguuaucuagcuguaugC.....................................................................  20     1  Ta1
....................ucuuugguuauUuagcuguauga.....................................................................  1      1  Ta1
....................ucuuugguuaucuagcCguauga.....................................................................  2      1  Ta1
....................ucuuugguuaucuaUcuguauga.....................................................................  15     1  Ta1
....................ucuuugguuauAuagcuguauga.....................................................................  1      1  Ta1
....................ucuuugguCaucuagcuguauga.....................................................................  1      1  Ta1
....................ucuuAgguuaucuagcuguauga.....................................................................  41     1  Ta1
....................uUuuugguuaucuagcuguauga.....................................................................  3      1  Ta1
....................ucuuugguuaucuUgcuguauga.....................................................................  1      1  Ta1
....................ucuAugguuaucuagcuguauga.....................................................................  3      1  Ta1
....................ucuuugguuCucuagcuguauga.....................................................................  14     1  Ta1
....................ucuuugUuuaucuagcuguauga.....................................................................  9      1  Ta1
....................uAuuugguuaucuagcuguauga.....................................................................  1      1  Ta1
....................ucuuuggAuaucuagcuguauga.....................................................................  2      1  Ta1
....................ucuuugguAaucuagcuguauga.....................................................................  8      1  Ta1
....................ucuuugguuaucGagcuguauga.....................................................................  1      1  Ta1
....................ucuuugguuaucuagcuguaAga.....................................................................  1      1  Ta1
....................ucuuugguuaucuagcuguaugU.....................................................................  1194   1  Ta1
```

```
                    Mature                                Star
gucggcaggcauugauguuuucuuugguuaucuagcuguaugagugugacugacgucauaaagcuagguuaccaaaguuaaaaacaaucgccagaaaucagcuggaugacag
....................ucuuugguuaucuagcugGauga.....................................................................    1     1    Ta1
....................ucuuGgguuaucuagcuguauga.....................................................................    206   1    Ta1
....................Ccuuugguuaucuagcuguauga.....................................................................    9     1    Ta1
....................Ncuuugguuaucuagcuguauga.....................................................................    2     1    Ta1
....................ucuuugguuaucuagcuguaugag....................................................................    2     0    Ta1
....................ucuuugguuaucuagcuguaugaU....................................................................    563   1    Ta1
....................ucuuugguuaucuagcuguaugaA....................................................................    245   1    Ta1
....................ucuuugguuaucuagcuguaugaC....................................................................    2     1    Ta1
....................ucuuugguuaucuagcuguaugUg....................................................................    18    1    Ta1
....................ucuuugguuaucuagcuguaugaUu...................................................................    19    1    Ta1
....................ucuuugguuaucuagcuguaugaAu...................................................................    22    1    Ta1
.....................cuuugguuCucuagcugua........................................................................    1     1    Ta1
.....................cuuugguuaucuagcuguau.......................................................................    1     0    Ta1
.....................cuuugUuuaucuagcuguau.......................................................................    1     1    Ta1
.....................cuuuUguuaucuagcuguaug......................................................................    2     1    Ta1
.....................cuuugguuaucuagcugAaug......................................................................    1     1    Ta1
.....................cuuugguuaucuagcuguUug......................................................................    1     1    Ta1
.....................cuuugguuaucuagcuguaug......................................................................    11    0    Ta1
.....................cuuugguuaucuagcugGaug......................................................................    1     1    Ta1
.....................cuuuUguuaucuagcuguauga.....................................................................    1     1    Ta1
.....................cuuugUuuaucuagcuguauga.....................................................................    1     1    Ta1
.....................cuuugguuaucuGgcuguauga.....................................................................    1     1    Ta1
.....................cuuugguuaucuagcCguauga.....................................................................    1     1    Ta1
.....................cuCugguuaucuagcuguauga.....................................................................    1     1    Ta1
.....................cuuugguuaucuagcuguauga.....................................................................    14    0    Ta1
......................uuugguuaucuagcuguau.......................................................................    3     0    Ta1
......................uuugguuaucuagcuguaug......................................................................    13    0    Ta1
......................uuugAuuaucuagcuguaug......................................................................    1     1    Ta1
......................uuugguuaucuagcuguauga.....................................................................    6     0    Ta1
.......................uugguuaucuagcuguaug......................................................................    2     0    Ta1
.......................uugguuaucuagcuguauga.....................................................................    5     0    Ta1
........................ugguuaucuagcuguaug......................................................................    7     0    Ta1
....................................uguaCgagugugacugacgu........................................................    2     1    Ta1
.........................................................auaaagcuagguuaccaaagu..................................    11    0    Ta1
.........................................................auaaagcuCgguuaccaaaguu.................................    1     1    Ta1
.........................................................Cuaaagcuagguuaccaaaguu.................................    1     1    Ta1
.........................................................auaaagcuagUuuaccaaaguu.................................    1     1    Ta1
.........................................................auaaagcuagguuaccaaaguu.................................    43    0    Ta1
.........................................................auaaagcuagguuaccaaaguua................................    4     0    Ta1
..........................................................uaaagcuagguuaccaaagu..................................    14    0    Ta1
..........................................................uaaaUcuagguuaccaaagu..................................    1     1    Ta1
..........................................................uaaaCcuagguuaccaaaguu.................................    1     1    Ta1
..........................................................uaaaAcuagguuaccaaaguu.................................    1     1    Ta1
..........................................................uaaagcuaAguuaccaaaguu.................................    1     1    Ta1
..........................................................uaaagcuagguuaccaaaguC.................................    1     1    Ta1
..........................................................Aaaagcuagguuaccaaaguu.................................    1     1    Ta1
..........................................................uaaagcuagguuaccaaaguu.................................    124   0    Ta1
..........................................................uaaaUcuagguuaccaaaguu.................................    8     1    Ta1
..........................................................uaaagUuagguuaccaaaguu.................................    1     1    Ta1
..........................................................uaaaUcuagguuaccaaaguua................................    1     1    Ta1
..........................................................uaaagcuagguuaccaaaguua................................    24    0    Ta1
..........................................................uaaagUuagguuaccaaaguua................................    1     1    Ta1
...........................................................aaagcuagguuaccaaaguu.................................    4     0    Ta1

...................Cucuuugguuaucuagcuguau.......................................................................    3     1    He1
...................Aucuuugguuaucuagcuguau.......................................................................    1     1    He1
...................Cucuuugguuaucuagcuguaug......................................................................    13    1    He1
...................Cucuuugguuaucuagcuguauga.....................................................................    1     1    He1
....................ucuuugguuaucuagcuA..........................................................................    1     1    He1
....................ucuuugguuaucuagcug..........................................................................    8     0    He1
....................ucuuugguuauUuagcugu.........................................................................    1     1    He1
....................ucuuugguuaucuagcugu.........................................................................    214   0    He1
....................ucuuugguuaucuagcugC.........................................................................    17    1    He1
....................ucuuuUguuaucuagcugu.........................................................................    1     1    He1
....................ucuuGgguuaucuagcugu.........................................................................    7     1    He1
....................ucuuCgguuaucuagcugu.........................................................................    1     1    He1
....................Gcuuugguuaucuagcugu.........................................................................    1     1    He1
....................ucuGugguuaucuagcugu.........................................................................    4     1    He1
....................ucuuAgguuaucuagcugu.........................................................................    2     1    He1
....................ucuAugguuaucuagcugu.........................................................................    1     1    He1
```

Mature **Star**

```
gucggcaggcauugauguuuucuuugguuaucuagcuguaugagugugacugacgucauaaagcuagguuaccaaaguuaaaaacaaucgccagaaaucagcuggaugacag
....................ucuuugguuaucuaUcugu.........................................................................   1      1   He1
....................ucuuugguAaucuagcugu.........................................................................   1      1   He1
....................ucuAugguuaucuagcugua........................................................................   5      1   He1
....................ucuuugguuaucuUgcugua........................................................................   1      1   He1
....................ucuuugguuaucuCgcugua........................................................................   2      1   He1
....................ucuGugguuaucuagcugua........................................................................   17     1   He1
....................ucuuugCuuaucuagcugua........................................................................   1      1   He1
....................ucuuugguuaCcuagcugua........................................................................   2      1   He1
....................ucuCugguuaucuagcugua........................................................................   1      1   He1
....................ucuuugguuaucuagcugua........................................................................   2850   0   He1
....................ucCuugguuaucuagcugua........................................................................   3      1   He1
....................ucuuugUuuaucuagcugua........................................................................   6      1   He1
....................ucuuugguuaucuagcuguU........................................................................   3      1   He1
....................ucuuugguuaucuagUugua........................................................................   1      1   He1
....................ucuuugguuaucuagcuguG........................................................................   1      1   He1
....................ucuuugguuaucGagcugua........................................................................   1      1   He1
....................ucuuuUguuaucuagcugua........................................................................   1      1   He1
....................Gcuuugguuaucuagcugua........................................................................   2      1   He1
....................ucuuuggAuaucuagcugua........................................................................   1      1   He1
....................Acuuugguuaucuagcugua........................................................................   3      1   He1
....................ucuuGgguuaucuagcugua........................................................................   112    1   He1
....................ucuuugguuaucuagcugCa........................................................................   11     1   He1
....................uAuuugguuaucuagcugua........................................................................   3      1   He1
....................ucuuugAuuaucuagcugua........................................................................   1      1   He1
....................ucuuugguuaAcuagcugua........................................................................   1      1   He1
....................Ccuuugguuaucuagcugua........................................................................   3      1   He1
....................ucuuugguuGucuagcugua........................................................................   1      1   He1
....................ucuuugguuaucuagcuguC........................................................................   1      1   He1
....................ucuuCgguuaucuagcugua........................................................................   12     1   He1
....................ucuuugguuCucuagcugua........................................................................   7      1   He1
....................ucuuugguuaucuagcuUua........................................................................   3      1   He1
....................ucuuugguAaucuagcugua........................................................................   1      1   He1
....................ucuuugguuaucuaUcugua........................................................................   5      1   He1
....................ucuuugguuaucuagcAgua........................................................................   1      1   He1
....................ucuuAgguuaucuagcugua........................................................................   21     1   He1
....................ucuCugguuaucuagcuguau.......................................................................   1      1   He1
....................ucuuugguuaucuagcuguUu.......................................................................   2      1   He1
....................ucuuugguCaucuagcuguau.......................................................................   2      1   He1
....................ucuuugguuaucuGgcuguau.......................................................................   3      1   He1
....................ucuuugguAaucuagcuguau.......................................................................   5      1   He1
....................ucuuugguuaucuagcuguaC.......................................................................   98     1   He1
....................ucuuugguuaucuaUcuguau.......................................................................   18     1   He1
....................ucuAugguuaucuagcuguau.......................................................................   3      1   He1
....................ucuuAgguuaucuagcuguau.......................................................................   44     1   He1
....................ucuGugguuaucuagcuguau.......................................................................   39     1   He1
....................ucuuugguuaucuaCcuguau.......................................................................   1      1   He1
....................ucuuugguuaucuagcuUuau.......................................................................   2      1   He1
....................ucuuugguuaucuagAuguau.......................................................................   1      1   He1
....................uAuuugguuaucuagcuguau.......................................................................   10     1   He1
....................ucuuugguuaucuagcGguau.......................................................................   2      1   He1
....................ucuuugguuaucuagcuAuau.......................................................................   1      1   He1
....................Ncuuugguuaucuagcuguau.......................................................................   5      1   He1
....................ucuuuggCuaucuagcuguau.......................................................................   3      1   He1
....................ucuuugguuaucuagcuguaG.......................................................................   8      1   He1
....................ucuuugguuGucuagcuguau.......................................................................   1      1   He1
....................ucuuuUguuaucuagcuguau.......................................................................   3      1   He1
....................Gcuuugguuaucuagcuguau.......................................................................   4      1   He1
....................ucuuCgguuaucuagcuguau.......................................................................   28     1   He1
....................ucGuugguuaucuagcuguau.......................................................................   1      1   He1
....................ucuuugguuaucuagcuguGu.......................................................................   1      1   He1
....................ucuuugguuaucuagcCguau.......................................................................   3      1   He1
....................ucuuugguuaucuagUuguau.......................................................................   1      1   He1
....................ucuuugUuuaucuagcuguau.......................................................................   10     1   He1
....................Acuuugguuaucuagcuguau.......................................................................   11     1   He1
....................ucuuugguuaucuagcugGau.......................................................................   3      1   He1
....................ucuuugguuaucuagcuguau.......................................................................   5348   0   He1
....................ucuuugAuuaucuagcuguau.......................................................................   1      1   He1
....................ucuuGgguuaucuagcuguau.......................................................................   230    1   He1
....................ucuuugguuCucuagcuguau.......................................................................   14     1   He1
....................ucuuugguuauUuagcuguau.......................................................................   2      1   He1
```

Mature Star

gucggcaggcauugauguuuucuuugguuaucuagcuguaugagugugacugacgucauaaagcuagguuaccaaaguuaaaaacaaucgccagaaaucagcuggaugacag

....................ucuuugguuaCcuagcuguau...	3	1	He1
....................ucuuuAguuaucuagcuguau...	1	1	He1
....................ucuuugguuaucuagcuguCu...	1	1	He1
....................ucuuugguuaucuagcuguaA...	94	1	He1
....................Ccuuugguuaucuagcuguau...	7	1	He1
....................ucCuugguuaucuagcuguau...	12	1	He1
....................ucuuugguuaAcuagcuguau...	1	1	He1
....................ucuuugguuaucuCgcuguau...	5	1	He1
....................ucuuugguuaucuUgcuguau...	5	1	He1
....................ucuuuAguuaucuagcuguaug..	1	1	He1
....................ucuAugguuaucuagcuguaug..	35	1	He1
....................ucuuugguuauGuagcuguaug..	5	1	He1
....................ucuuugguuaucuagcuguauC..	4	1	He1
....................ucuuugguuaucuagcugCaug..	3	1	He1
....................ucuuugguuaucuagcuAuaug..	2	1	He1
....................ucuuugguuaucuagcuUuaug..	16	1	He1
....................ucuuugguuaucuagcuguaAg..	7	1	He1
....................ucuuugguuaucuagUuguaug..	22	1	He1
....................ucuuugguCaucuagcuguaug..	8	1	He1
....................ucuuugguuaGcuagcuguaug..	2	1	He1
....................ucuuugguAaucuagcuguaug..	20	1	He1
....................ucuuugguuaucAagcuguaug..	1	1	He1
....................ucuuugguuaucuagcuguaGg..	1	1	He1
....................ucuuCgguuaucuagcuguaug..	137	1	He1
....................ucuuugguuaucuagcugGaug..	12	1	He1
....................ucuuuggGuaucuagcuguaug..	2	1	He1
....................ucuuuCguuaucuagcuguaug..	1	1	He1
....................ucuuugguuaucuagcuguCug..	4	1	He1
....................uUuuugguuaucuagcuguaug..	9	1	He1
....................ucuuugguuCucuagcuguaug..	84	1	He1
....................ucuuugguGaucuagcuguaug..	1	1	He1
....................ucuuugUuuaucuagcuguaug..	32	1	He1
....................ucuuugguuaucuagcuguUug..	9	1	He1
....................ucuuugguuaucuaAcuguaug..	3	1	He1
....................Acuuugguuaucuagcuguaug..	50	1	He1
....................Ccuuugguuaucuagcuguaug..	43	1	He1
....................ucuuugguuaucuagcAguaug..	5	1	He1
....................ucuGgguuaucuagcuguaug..	217	1	He1
....................ucuuuUguuaucuagcuguaug..	20	1	He1
....................ucuuugguuauUuagcuguaug..	11	1	He1
....................ucuuugguuaucuCgcuguaug..	30	1	He1
....................uGuuugguuaucuagcuguaug..	3	1	He1
....................ucuuugguuaucuagcuguauU..	81	1	He1
....................ucCuugguuaucuagcuguaug..	67	1	He1
....................ucuuugguuaucuGgcuguaug..	4	1	He1
....................ucuCugguuaucuagcuguaug..	32	1	He1
....................ucuuugguuaucuagcuguauA..	153	1	He1
....................ucuuugguuaucuagcuguaug..	26976	0	He1
....................ucuuAgguuaucuagcuguaug..	188	1	He1
....................ucuuGgguuaucuagcuguaug..	1099	1	He1
....................ucuuuggAuaucuagcuguaug..	2	1	He1
....................ucuuugguuauAuagcuguaug..	2	1	He1
....................ucuuugguuaucuagcugAaug..	2	1	He1
....................ucuuugguuaucuagcuCuaug..	1	1	He1
....................ucuuugAuuaucuagcuguaug..	6	1	He1
....................ucuuugguuaAcuagcuguaug..	9	1	He1
....................ucuuugguuaucuUgcuguaug..	18	1	He1
....................ucuuugguuaucuagcuguGug..	10	1	He1
....................uAuuugguuaucuagcuguaug..	29	1	He1
....................Ncuuugguuaucuagcuguaug..	16	1	He1
....................ucuuugguuaucuagAuguaug..	2	1	He1
....................ucuuugguuaCcuagcuguaug..	5	1	He1
....................ucuuugguuaucuaUcuguaug..	50	1	He1
....................ucuuuggCuaucuagcuguaug..	6	1	He1
....................ucuuugguuaucuagcuguaCg..	31	1	He1
....................Gcuuugguuaucuagcuguaug..	4	1	He1
....................ucGuugguuaucuagcuguaug..	2	1	He1
....................ucuuugCuuaucuagcuguaug..	1	1	He1
....................ucuuugguuGucuagcuguaug..	5	1	He1
....................ucuuugguuaucuagcCguaug..	5	1	He1

Mature Star

gucggcaggcauugauguuuucuuugguuaucuagcuguaugagugugacugacgucauaaagcuagguuaccaaaguuaaaaacaaucgccagaaaucagcuggaugacag			
....................ucuuugguuaucuagcGguaug..	3	1	He1
....................ucuuugguuaucCagcuguaug..	6	1	He1
....................ucuuugguuaucuagcuCuauga...	1	1	He1
....................ucuuugguuaucuagcuguaugU...	675	1	He1
....................ucuuCgguuaucuagcuguauga...	28	1	He1
....................ucuuugguuaucuagcuguCuga...	1	1	He1
....................ucuuugguuaucuaUcuguauga...	8	1	He1
....................ucuuugguuaucuaAcuguauga...	1	1	He1
....................ucuuugguuaAcuagcuguauga...	1	1	He1
....................ucuuugguuaucuGgcuguauga...	2	1	He1
....................ucuuugguAaucuagcuguauga...	5	1	He1
....................ucuuugguuaucuagcuguaugC...	77	1	He1
....................Ncuuugguuaucuagcuguauga...	4	1	He1
....................ucuuugguuaucuagUuguauga...	4	1	He1
....................ucuuugguuaucCagcuguauga...	4	1	He1
....................uUuuugguuaucuagcuguauga...	3	1	He1
....................uAuuugguuaucuagcuguauga...	5	1	He1
....................ucuuugguuaucAagcuguauga...	1	1	He1
....................ucuCugguuaucuagcuguauga...	5	1	He1
....................ucuuugguuaucuagcuguauUa...	6	1	He1
....................ucuuugguuaucuagcugGauga...	1	1	He1
....................ucuGugguuaucuagcuguauga...	36	1	He1
....................ucuuugguuaucuagcugAauga...	1	1	He1
....................ucuuugguuaucuagcuguauAa...	25	1	He1
....................ucuuugAuuaucuagcuguauga...	2	1	He1
....................Acuuugguuaucuagcuguauga...	7	1	He1
....................ucuuuggAuaucuagcuguauga...	1	1	He1
....................ucuuuggGuaucuagcuguauga...	1	1	He1
....................ucuuugguCaucuagcuguauga...	2	1	He1
....................ucuuugguuaucuagcuUuauga...	3	1	He1
....................ucuuugguuaucuagcuguauga...	4064	0	He1
....................Gcuuugguuaucuagcuguauga...	1	1	He1
....................ucuuugguuaucuCgcuguauga...	6	1	He1
....................ucuuugguuauUuagcuguauga...	1	1	He1
....................ucuuuUguuaucuagcuguauga...	2	1	He1
....................ucuuugguuaucuagAuguauga...	1	1	He1
....................ucuuugguuaucuagcuguaugG...	16	1	He1
....................ucuuugguuaucuagcCguauga...	1	1	He1
....................ucuAugguuaucuagcuguauga...	4	1	He1
....................ucuuuAguuaucuagcuguauga...	2	1	He1
....................ucuuGgguuaucuagcuguauga...	190	1	He1
....................ucCuugguuaucuagcuguauga...	7	1	He1
....................ucuuugUuuaucuagcuguauga...	11	1	He1
....................ucuuugguuaucuagcAguauga...	1	1	He1
....................ucuuAgguuaucuagcuguauga...	25	1	He1
....................Ccuuugguuaucuagcuguauga...	4	1	He1
....................ucuuugguuaucuagcugCauga...	1	1	He1
....................ucuuugguuaucuagcuguGuga...	1	1	He1
....................ucuuugguuaucuagcuguUuga...	1	1	He1
....................ucuuugguuCucuagcuguauga...	13	1	He1
....................ucuuugguuaucuagcuguaugCg..	1	1	He1
....................ucuuugguuaucuagcuguaugaA..	256	1	He1
....................ucuuugguuaucuagcuguaugaC..	14	1	He1
....................ucuuugguuaucuagcuguaugUg..	4	1	He1
....................ucuuugguuaucuagcuguaugaU..	290	1	He1
....................ucuuuUguuaucuagcuguaugag..	1	1	He1
....................ucuuugguuaucuagcuguaugag..	5	0	He1
....................ucuuGgguuaucuagcuguaugag..	1	1	He1
....................ucuuugguuaucuagcuguaugagu...	1	0	He1
....................ucuuugguuaucuagcuguaugaCu...	3	1	He1
....................ucuuugguuaucuagcuguaugaAu...	17	1	He1
....................ucuuugguuaucuagcuguaugagA...	2	1	He1
....................ucuuugguuaucuagcuguaugaUu...	12	1	He1
.....................cuuugguuaucuagcugua..	3	0	He1
.....................cuuugguuaucuagcuguau...	1	0	He1
.....................cuuugguuaucuagGuguaug..	1	1	He1
.....................cuuugguuaucuCgcuguaug..	2	1	He1
.....................cuuugguuaucuagcuguauU..	1	1	He1
.....................cuuugguuCucuagcuguaug..	1	1	He1
.....................cuuuUguuaucuagcuguaug..	1	1	He1

Mature **Star**

gucggcaggcauugauguuuucuuugguuaucuagcuguaugagugugacugacgucauaaagcuagguuaccaaaguuaaaaacaaucgccagaaaucagcuggaugacag			
.....................cuuugguuaucuagcuguaug..	19	0	He1
.....................cuuuUguuaucuagcuguauga...	2	1	He1
.....................cuuugguuaucuagcuguauCa...	1	1	He1
.....................cuuugguuaucuagcuguauga...	7	0	He1
.....................cuuugguuaucuagcuguaugaA..	1	1	He1
......................uuugguuaucuagcugua..	1	0	He1
......................uuugguuaucuagcuguau...	2	0	He1
......................uuugUuuaucuagcuguaug..	1	1	He1
......................uuugguuaucuagcuguaug..	16	0	He1
......................uuugguuaucuagcuguauga...	11	0	He1
.......................uugguuaucuagcuguau...	1	0	He1
.......................uugguuaucuagcuguaug..	3	0	He1
.......................uugguuaucuagcuguauga...	1	0	He1
........................ugguuaucuagcuguauga...	2	0	He1
....auaaagcuagguuaccaaagu..................................	6	0	He1
....auaaagcuUgguuaccaaaguu.................................	1	1	He1
....auaaagcuagguuaccaaaguu.................................	13	0	He1
....auaaagcuagguuaccaCaguua................................	1	1	He1
....auaaUgcuagguuaccaaaguua................................	1	1	He1
....auaaagcuagguuaccaaaguua................................	7	0	He1
....auaaagcuagguuaccaaaguuaa...............................	2	0	He1
....uaaagcuagguuaccaaag...................................	1	0	He1
....uaaagcuagguuaccaaagu..................................	6	0	He1
....uaaagcuagguuaccaaaguu.................................	46	0	He1
....uaaagcuagguuaccaCaguu.................................	1	1	He1
....uaaUgcuagguuaccaaaguu.................................	1	1	He1
....Caaagcuagguuaccaaaguu.................................	1	1	He1
....uaaaUcuagguuaccaaaguu.................................	3	1	He1
....uaaagcuagguuaccaaaguua................................	23	0	He1
....uaaaUcuagguuaccaaaguua................................	1	1	He1
...................Cucuuugguuaucuagcuguau...	1	1	Bl1
....................ucuuugguuaucuagcug..	21	0	Bl1
....................ucuuugguuaucuagUug..	1	1	Bl1
....................ucuuugguuaucuagcugu...	71	0	Bl1
....................ucuuugguuaucuagcugC...	1	1	Bl1
....................ucuuugguuCucuagcugu...	1	1	Bl1
....................ucuuAgguuaucuagcugu...	1	1	Bl1
....................ucuuugguuaucuagcugGa..	1	1	Bl1
....................ucuuAgguuaucuagcugua..	15	1	Bl1
....................ucuuugguuaucuGgcugua..	1	1	Bl1
....................ucuuugguuaucuagcugua..	2040	0	Bl1
....................ucuuugguuaucCagcugua..	1	1	Bl1
....................ucuuCgguuaucuagcugua..	10	1	Bl1
....................uAuuugguuaucuagcugua..	5	1	Bl1
....................uGuuugguuaucuagcugua..	1	1	Bl1
....................ucuuugguuaucuUgcugua..	2	1	Bl1
....................ucuCugguuaucuagcugua..	1	1	Bl1
....................ucuuugguuaucuaUcugua..	6	1	Bl1
....................ucuuuAguuaucuagcugua..	2	1	Bl1
....................ucuuugguuCucuagcugua..	9	1	Bl1
....................Acuuugguuaucuagcugua..	5	1	Bl1
....................ucuuugguAaucuagcugua..	1	1	Bl1
....................ucuuugguuaucuagUugua..	3	1	Bl1
....................ucuuugguuaAcuagcugua..	1	1	Bl1
....................ucuuGgguuaucuagcugua..	71	1	Bl1
....................Ccuuugguuaucuagcugua..	2	1	Bl1
....................ucuuugguuaucuagcuUua..	1	1	Bl1
....................ucuuugUuuaucuagcugua..	2	1	Bl1
....................ucuuugguuaucuaAcugua..	1	1	Bl1
....................ucCuugguuaucuagcugua..	3	1	Bl1
....................ucuuugguuaucuCgcugua..	3	1	Bl1
....................ucuuugguuaucuagcuguG..	2	1	Bl1
....................ucuAugguuaucuagcugua..	4	1	Bl1
....................ucuuugguuaucuagcugCa..	5	1	Bl1
....................ucuuuUguuaucuagcugua..	2	1	Bl1
....................ucuuugguuauUuagcugua..	2	1	Bl1
....................ucuGugguuaucuagcugua..	12	1	Bl1
....................ucuCugguuaucuagcuguau...	4	1	Bl1
....................ucuuugUuuaucuagcuguau...	2	1	Bl1

```
                    Mature                                  Star
gucggcaggcauugauguuuucuuugguuaucuagcuguaugagugugacugacgucauaaagcuagguuaccaaaguuaaaaacaaucgccagaaaucagcuggaugacag
....................ucuuugguuaucuagcuguau.......................................................................   2086   0   Bl1
....................ucuuugguuaAcuagcuguau.......................................................................   2      1   Bl1
....................ucuuugguuaucuagcuguaG.......................................................................   1      1   Bl1
....................ucuuugguuaucuCgcuguau.......................................................................   4      1   Bl1
....................ucCuugguuaucuagcuguau.......................................................................   5      1   Bl1
....................Ccuuugguuaucuagcuguau.......................................................................   4      1   Bl1
....................ucuuugguuauUuagcuguau.......................................................................   2      1   Bl1
....................ucuuAgguuaucuagcuguau.......................................................................   22     1   Bl1
....................ucuuugguuaucuagcuUuau.......................................................................   2      1   Bl1
....................ucuuugguuaucuagcuguaA.......................................................................   30     1   Bl1
....................ucuuCgguuaucuagcuguau.......................................................................   8      1   Bl1
....................ucuuuUguuaucuagcuguau.......................................................................   2      1   Bl1
....................uAuuugguuaucuagcuguau.......................................................................   4      1   Bl1
....................ucuuugguCaucuagcuguau.......................................................................   1      1   Bl1
....................ucuuugguuauAuagcuguau.......................................................................   1      1   Bl1
....................ucuuGgguuaucuagcuguau.......................................................................   104    1   Bl1
....................ucuuugguuaucAagcuguau.......................................................................   1      1   Bl1
....................ucuAugguuaucuagcuguau.......................................................................   3      1   Bl1
....................ucuuugguAaucuagcuguau.......................................................................   2      1   Bl1
....................ucuuugguuaucuagcuguGu.......................................................................   1      1   Bl1
....................ucuuugguuaucuagcugAau.......................................................................   1      1   Bl1
....................Acuuugguuaucuagcuguau.......................................................................   4      1   Bl1
....................ucuuugguuaucuagUuguau.......................................................................   6      1   Bl1
....................ucuuugguuaucuUgcuguau.......................................................................   5      1   Bl1
....................ucuuuggCuaucuagcuguau.......................................................................   1      1   Bl1
....................ucuuugguuCucuagcuguau.......................................................................   1      1   Bl1
....................ucuuugguuaucuagcuguaC.......................................................................   12     1   Bl1
....................ucuuugguuaucuagcugGau.......................................................................   1      1   Bl1
....................ucuuugguuaucuaCcuguau.......................................................................   1      1   Bl1
....................ucuuugCuuaucuagcuguau.......................................................................   1      1   Bl1
....................ucuuugguuaucuaUcuguau.......................................................................   7      1   Bl1
....................ucuuugguuaucuaAcuguau.......................................................................   2      1   Bl1
....................ucuuugguuaucCagcuguau.......................................................................   1      1   Bl1
....................ucuuugguuaucuagcAguau.......................................................................   1      1   Bl1
....................ucuuugguuaucuagcCguau.......................................................................   1      1   Bl1
....................ucuGugguuaucuagcuguau.......................................................................   16     1   Bl1
....................Ncuuugguuaucuagcuguau.......................................................................   1      1   Bl1
....................ucuuugguuCucuagcuguaug......................................................................   2      1   Bl1
....................ucuuugguuaucuagcGguaug......................................................................   1      1   Bl1
....................ucuuugguuaucuagcuguauA......................................................................   7      1   Bl1
....................ucuuugguuaucuagUuguaug......................................................................   1      1   Bl1
....................ucuuugguAaucuagcuguaug......................................................................   2      1   Bl1
....................ucCuugguuaucuagcuguaug......................................................................   3      1   Bl1
....................ucuuCgguuaucuagcuguaug......................................................................   13     1   Bl1
....................ucuuuggAuaucuagcuguaug......................................................................   3      1   Bl1
....................Acuuugguuaucuagcuguaug......................................................................   9      1   Bl1
....................ucuGugguuaucuagcuguaug......................................................................   15     1   Bl1
....................ucuuugguuaucuagcuUuaug......................................................................   1      1   Bl1
....................uUuuugguuaucuagcuguaug......................................................................   1      1   Bl1
....................ucuuugguuaucuagcuguaug......................................................................   1831   0   Bl1
....................ucuCugguuaucuagcuguaug......................................................................   2      1   Bl1
....................ucuuugguuaucuagcuguauU......................................................................   6      1   Bl1
....................ucuAugguuaucuagcuguaug......................................................................   3      1   Bl1
....................ucuuAgguuaucuagcuguaug......................................................................   9      1   Bl1
....................ucuuuUguuaucuagcuguaug......................................................................   1      1   Bl1
....................ucuuugguuauUuagcuguaug......................................................................   1      1   Bl1
....................Gcuuugguuaucuagcuguaug......................................................................   1      1   Bl1
....................ucuuugguuaucuaUcuguaug......................................................................   3      1   Bl1
....................ucuuugguuaucuagcuguaCg......................................................................   1      1   Bl1
....................ucuuugguuaucuUgcuguaug......................................................................   2      1   Bl1
....................ucuuugUuuaucuagcuguaug......................................................................   5      1   Bl1
....................ucuuugguuaucuCgcuguaug......................................................................   3      1   Bl1
....................ucuuGgguuaucuagcuguaug......................................................................   74     1   Bl1
....................uAuuugguuaucuagcuguaug......................................................................   2      1   Bl1
....................ucuuugUuuaucuagcuguauga.....................................................................   1      1   Bl1
....................ucuuugguAaucuagcuguauga.....................................................................   1      1   Bl1
....................ucuuugguuaucuagcuguaugC.....................................................................   3      1   Bl1
....................ucuuGgguuaucuagcuguauga.....................................................................   20     1   Bl1
....................ucuuugguuaucuagcuguauga.....................................................................   446    0   Bl1
....................ucCuugguuaucuagcuguauga.....................................................................   1      1   Bl1
```

```
                    Mature                                  Star
gucggcaggcauugauguuuucuuugguuaucuagcuguaugagugugacugacgucauaaagcuagguuaccaaaguuaaaaacaaucgccagaaaucagcuggaugacag
....................ucuuugguuGucuagcuguauga.....................................................................    1     1     Bl1
....................Ncuuugguuaucuagcuguauga.....................................................................    1     1     Bl1
....................Acuuugguuaucuagcuguauga.....................................................................    1     1     Bl1
....................ucuuugguuaucuaCcuguauga.....................................................................    1     1     Bl1
....................ucuuCgguuaucuagcuguauga.....................................................................    3     1     Bl1
....................ucuuugguuaucuagcuCuauga.....................................................................    1     1     Bl1
....................ucuuugguuaucuaUcuguauga.....................................................................    1     1     Bl1
....................uGuuugguuaucuagcuguauga.....................................................................    1     1     Bl1
....................uUuuugguuaucuagcuguauga.....................................................................    1     1     Bl1
....................ucuuugguuaucuagcuguauUa.....................................................................    1     1     Bl1
....................ucuGugguuaucuagcuguauga.....................................................................    5     1     Bl1
....................Ccuuugguuaucuagcuguauga.....................................................................    1     1     Bl1
....................ucuuugguuaucuagcuguaugU.....................................................................    109   1     Bl1
....................uAuuugguuaucuagcuguauga.....................................................................    1     1     Bl1
....................ucuuugguuaucuaAcuguauga.....................................................................    1     1     Bl1
....................ucuuAgguuaucuagcuguauga.....................................................................    5     1     Bl1
....................ucuuugguuaucuagcuguaugG.....................................................................    1     1     Bl1
....................ucuAugguuaucuagcuguauga.....................................................................    1     1     Bl1
....................ucuuugguuCucuagcuguauga.....................................................................    2     1     Bl1
....................ucuuugguuaucuagcuguaugag....................................................................    2     0     Bl1
....................ucuuugguuaucuagcuguaugaU....................................................................    39    1     Bl1
....................ucuuugguuaucuagcuguaugaC....................................................................    3     1     Bl1
....................ucuuugguuaucuagcuguaugaA....................................................................    49    1     Bl1
....................ucuuugguuaucuagcuguaugaAu...................................................................    2     1     Bl1
.....................cuuugguuaucuagcuguaug......................................................................    2     0     Bl1
.....................cuuugguuaucuagcuguauga.....................................................................    3     0     Bl1
......................uuugguuaucuagcuguaug......................................................................    1     0     Bl1
......................uuugguuaucuagcuguaugU.....................................................................    1     1     Bl1
.......................uugguuaucuagcuguauga.....................................................................    2     0     Bl1
.........................................................auaaagcuagguuaccaa.....................................    1     0     Bl1
.........................................................auaaagcuagguuaccaaa....................................    3     0     Bl1
.........................................................auaaagcuagguuaccaaag...................................    6     0     Bl1
.........................................................Cuaaagcuagguuaccaaagu..................................    3     1     Bl1
.........................................................auaaagcuagguuaccaaagu..................................    11    0     Bl1
.........................................................auaaagcuCgguuaccaaaguu.................................    2     1     Bl1
.........................................................auaaagcuagguuaccaaaguu.................................    28    0     Bl1
.........................................................auaaGgcuagguuaccaaaguu.................................    1     1     Bl1
.........................................................auaaagcuagguuacAaaaguua................................    1     1     Bl1
.........................................................auaaagcuagguuaccaaaguua................................    9     0     Bl1
..........................................................uaaagcuagguuaccaaa....................................    6     0     Bl1
..........................................................uaaagcuagguuaccaaag...................................    8     0     Bl1
..........................................................uaaagcuagguuaccaaagu..................................    21    0     Bl1
..........................................................uaaagcuagguuaccaaaguu.................................    38    0     Bl1
..........................................................uaaaUcuagguuaccaaaguu.................................    1     1     Bl1
..........................................................uaUagcuagguuaccaaaguu.................................    1     1     Bl1
..........................................................uaaagcuagguuaccaaaUuu.................................    1     1     Bl1
..........................................................uaaagcuagguuaccaaUguu.................................    1     1     Bl1
..........................................................uaaagcuagguuaccaaaguua................................    9     0     Bl1
..........................................................uaaaUcuagguuaccaaaguua................................    3     1     Bl1
...........................................................aaagcuagguuaccaaagC..................................    1     1     Bl1
...........................................................aaagcuagguuaccaaCguu.................................    1     1     Bl1

..................uCucuuugguuaucuagcuguau.......................................................................    1     1     Ch1
...................Aucuuugguuaucuagcugua........................................................................    2     1     Ch1
...................Cucuuugguuaucuagcugua........................................................................    4     1     Ch1
...................Cucuuugguuaucuagcuguau.......................................................................    12    1     Ch1
...................uucuuugguuaucuagcuguau.......................................................................    1     0     Ch1
...................Aucuuugguuaucuagcuguau.......................................................................    7     1     Ch1
...................uucGuugguuaucuagcuguaug......................................................................    1     1     Ch1
...................Cucuuugguuaucuagcuguaug......................................................................    41    1     Ch1
...................uucuuugguuaucuagcuguaug......................................................................    1     0     Ch1
...................Aucuuugguuaucuagcuguaug......................................................................    8     1     Ch1
...................Gucuuugguuaucuagcuguaug......................................................................    2     1     Ch1
...................Cucuuugguuaucuagcuguauga.....................................................................    8     1     Ch1
...................Aucuuugguuaucuagcuguauga.....................................................................    3     1     Ch1
....................ucuuAgguuaucuagcug..........................................................................    1     1     Ch1
....................ucuuugguuaucuagcuA..........................................................................    2     1     Ch1
....................ucuuugguuaucuagcug..........................................................................    22    0     Ch1
....................ucuuugguuaucuagcCg..........................................................................    1     1     Ch1
....................ucuuGgguuaucuagcugu.........................................................................    47    1     Ch1
```

```
                   Mature                              Star
gucggcaggcauugauguuuucuuugguuaucuagcuguaugagugugacugacgucauaaagcuagguuaccaaaguuaaaaacaaucgccagaaaucagcuggaugacag
....................Ccuuugguuaucuagcugu.........................................................................   2      1   Ch1
....................ucuuugguuaucuagcugu.........................................................................   1075   0   Ch1
....................uAuuugguuaucuagcugu.........................................................................   2      1   Ch1
....................ucuuAgguuaucuagcugu.........................................................................   6      1   Ch1
....................ucuuugguuaucuagcAgu.........................................................................   1      1   Ch1
....................ucuGugguuaucuagcugu.........................................................................   10     1   Ch1
....................ucuuCgguuaucuagcugu.........................................................................   5      1   Ch1
....................ucuuugguuaucuagcuAu.........................................................................   1      1   Ch1
....................Acuuugguuaucuagcugu.........................................................................   3      1   Ch1
....................ucuuugguuaGcuagcugu.........................................................................   1      1   Ch1
....................ucuuugguuaucuagcugG.........................................................................   1      1   Ch1
....................ucuuugguuaucuCgcugu.........................................................................   3      1   Ch1
....................ucuuugguuaucuaUcugu.........................................................................   5      1   Ch1
....................ucuuugguuCucuagcugu.........................................................................   3      1   Ch1
....................ucuuugguuauUuagcugu.........................................................................   1      1   Ch1
....................ucuuugguuaucuUgcugu.........................................................................   1      1   Ch1
....................ucuuugguuaucuagcugC.........................................................................   84     1   Ch1
....................ucCuugguuaucuagcugu.........................................................................   3      1   Ch1
....................ucuuugguuaucuagcugA.........................................................................   2      1   Ch1
....................ucuuugguuaucuagcCgu.........................................................................   1      1   Ch1
....................ucuuugguuaucuagcuguU........................................................................   19     1   Ch1
....................ucuuugguuaucAagcugua........................................................................   4      1   Ch1
....................ucuuugCuuaucuagcugua........................................................................   4      1   Ch1
....................ucuuugguuaucuagcuAua........................................................................   2      1   Ch1
....................ucuuugguuaucuagcuguC........................................................................   7      1   Ch1
....................ucuuugguuaucuaCcugua........................................................................   2      1   Ch1
....................ucuuugguuaucuagcugua........................................................................   21246  0   Ch1
....................ucuuugguGaucuagcugua........................................................................   1      1   Ch1
....................ucuuugguuCucuagcugua........................................................................   70     1   Ch1
....................ucuuugguuaucuaUcugua........................................................................   40     1   Ch1
....................ucuuugguuaucuagUugua........................................................................   14     1   Ch1
....................ucuuugguuaucuagcugAa........................................................................   1      1   Ch1
....................ucuuuggCuaucuagcugua........................................................................   4      1   Ch1
....................ucuuCgguuaucuagcugua........................................................................   126    1   Ch1
....................ucuuugguuaucuagcugGa........................................................................   11     1   Ch1
....................Acuuugguuaucuagcugua........................................................................   40     1   Ch1
....................ucuuugguuaucuagcGgua........................................................................   2      1   Ch1
....................ucuuugguuaucuagcugCa........................................................................   45     1   Ch1
....................ucuuAgguuaucuagcugua........................................................................   179    1   Ch1
....................ucuuuggAuaucuagcugua........................................................................   5      1   Ch1
....................ucuuugguAaucuagcugua........................................................................   32     1   Ch1
....................ucuuugguuaAcuagcugua........................................................................   7      1   Ch1
....................uGuuugguuaucuagcugua........................................................................   2      1   Ch1
....................ucuuugguuauGuagcugua........................................................................   2      1   Ch1
....................ucuuugguuaucCagcugua........................................................................   2      1   Ch1
....................ucuuGgguuaucuagcugua........................................................................   892    1   Ch1
....................ucuuugguuaucuagcuguG........................................................................   23     1   Ch1
....................ucuuugguuaucuGgcugua........................................................................   4      1   Ch1
....................uAuuugguuaucuagcugua........................................................................   31     1   Ch1
....................ucuuugguuaucuagcuCua........................................................................   1      1   Ch1
....................Ncuuugguuaucuagcugua........................................................................   18     1   Ch1
....................ucuuugguuauAuagcugua........................................................................   1      1   Ch1
....................ucuAugguuaucuagcugua........................................................................   27     1   Ch1
....................ucuuugguuaucuagcuUua........................................................................   14     1   Ch1
....................ucuuuUguuaucuagcugua........................................................................   14     1   Ch1
....................ucuuugAuuaucuagcugua........................................................................   8      1   Ch1
....................ucuuugguuauUuagcugua........................................................................   6      1   Ch1
....................Gcuuugguuaucuagcugua........................................................................   6      1   Ch1
....................ucuuugguuaCcuagcugua........................................................................   3      1   Ch1
....................Ccuuugguuaucuagcugua........................................................................   21     1   Ch1
....................ucuuugguuaucuCgcugua........................................................................   24     1   Ch1
....................ucuuugguuaucuagAugua........................................................................   2      1   Ch1
....................ucuCugguuaucuagcugua........................................................................   30     1   Ch1
....................ucuuugguuaucuagcCgua........................................................................   6      1   Ch1
....................ucuuugguuaucuUgcugua........................................................................   6      1   Ch1
....................ucuGugguuaucuagcugua........................................................................   154    1   Ch1
....................ucuuuAguuaucuagcugua........................................................................   1      1   Ch1
....................ucuuugguCaucuagcugua........................................................................   9      1   Ch1
....................ucuuugguuaucuagcAgua........................................................................   2      1   Ch1
....................ucuuugUuuaucuagcugua........................................................................   23     1   Ch1
```

Mature **Star**

```
gucggcaggcauugauguuuucuuugguuaucuagcuguaugagugugacugacgucauaaagcuagguuaccaaaguuaaaaacaaucgccagaaaucagcuggaugacag
....................ucGuugguuaucuagcugua........................................................................ 1 1 Ch1
....................ucuuuggGuaucuagcugua........................................................................ 2 1 Ch1
....................ucuuugguuGucuagcugua........................................................................ 4 1 Ch1
....................ucuuugguuaucuaAcugua........................................................................ 5 1 Ch1
....................ucCuugguuaucuagcugua........................................................................ 52 1 Ch1
....................ucuuugguGaucuagcuguau....................................................................... 1 1 Ch1
....................ucuuugAuuaucuagcuguau....................................................................... 4 1 Ch1
....................ucuuugguCaucuagcuguau....................................................................... 6 1 Ch1
....................ucuuugguuaucuagcAguau....................................................................... 3 1 Ch1
....................ucuuugguuauAuagcuguau....................................................................... 3 1 Ch1
....................ucuCugguuaucuagcuguau....................................................................... 31 1 Ch1
....................ucuuugguAaucuagcuguau....................................................................... 23 1 Ch1
....................ucuuugguuaucuaUcuguau....................................................................... 74 1 Ch1
....................ucuuCgguuaucuagcuguau....................................................................... 202 1 Ch1
....................Gcuuugguuaucuagcuguau....................................................................... 14 1 Ch1
....................uAuuugguuaucuagcuguau....................................................................... 48 1 Ch1
....................ucuuugguuaucuGgcuguau....................................................................... 7 1 Ch1
....................ucuuugguuaucuagcuAuau....................................................................... 2 1 Ch1
....................ucuuuAguuaucuagcuguau....................................................................... 4 1 Ch1
....................ucuuugguuaAcuagcuguau....................................................................... 6 1 Ch1
....................ucuuugguuGucuagcuguau....................................................................... 6 1 Ch1
....................ucuuugguuaucuagcuUuau....................................................................... 19 1 Ch1
....................ucuuugUuuaucuagcuguau....................................................................... 39 1 Ch1
....................ucuuugguuaucuagcuguGu....................................................................... 13 1 Ch1
....................uGuuugguuaucuagcuguau....................................................................... 4 1 Ch1
....................ucuuugguuaucAagcuguau....................................................................... 4 1 Ch1
....................ucuuugguuaucuagAuguau....................................................................... 3 1 Ch1
....................Ccuuugguuaucuagcuguau....................................................................... 44 1 Ch1
....................ucuuuggAuaucuagcuguau....................................................................... 3 1 Ch1
....................ucuuugguuaucuagcCguau....................................................................... 7 1 Ch1
....................ucuuUgguuaucuagcuguau....................................................................... 27 1 Ch1
....................ucuuugguuaucuagcugCau....................................................................... 6 1 Ch1
....................ucuuugguuaCcuagcuguau....................................................................... 5 1 Ch1
....................ucCuugguuaucuagcuguau....................................................................... 88 1 Ch1
....................ucuuugguuaucuagcuguaG....................................................................... 80 1 Ch1
....................ucuuugguuaucuaAcuguau....................................................................... 9 1 Ch1
....................ucuuugCuuaucuagcuguau....................................................................... 1 1 Ch1
....................ucuuugguuaucuUgcuguau....................................................................... 12 1 Ch1
....................ucuuugguuaucuagcuguaC....................................................................... 448 1 Ch1
....................ucuuugguuaucuCgcuguau....................................................................... 53 1 Ch1
....................ucuuugguuCucuagcuguau....................................................................... 86 1 Ch1
....................ucuuugguuaucuagcuguau....................................................................... 34558 0 Ch1
....................ucuuugguuaucuaCcuguau....................................................................... 4 1 Ch1
....................ucuAugguuaucuagcuguau....................................................................... 37 1 Ch1
....................ucuuGgguuaucuagcuguau....................................................................... 1468 1 Ch1
....................ucuuugguuauUuagcuguau....................................................................... 14 1 Ch1
....................ucuuuCguuaucuagcuguau....................................................................... 3 1 Ch1
....................ucuuuggCuaucuagcuguau....................................................................... 12 1 Ch1
....................ucuuugguuaucuagUuguau....................................................................... 22 1 Ch1
....................ucuGugguuaucuagcuguau....................................................................... 274 1 Ch1
....................ucGuugguuaucuagcuguau....................................................................... 1 1 Ch1
....................ucuuugguuaucuagcuguaA....................................................................... 814 1 Ch1
....................ucuuugguuaucuagcugAau....................................................................... 5 1 Ch1
....................ucuuugguuaucuagcuguUu....................................................................... 11 1 Ch1
....................ucAuugguuaucuagcuguau....................................................................... 1 1 Ch1
....................ucuuAgguuaucuagcuguau....................................................................... 246 1 Ch1
....................ucuuugguuaucuagcuguCu....................................................................... 1 1 Ch1
....................ucuuugguuaucuagcugGau....................................................................... 18 1 Ch1
....................ucuuugguuauGuagcuguau....................................................................... 10 1 Ch1
....................Acuuugguuaucuagcuguau....................................................................... 81 1 Ch1
....................ucuuuggGuaucuagcuguau....................................................................... 3 1 Ch1
....................Ncuuugguuaucuagcuguau....................................................................... 29 1 Ch1
....................ucuuugguuaucCagcuguau....................................................................... 7 1 Ch1
....................ucuuugguuaucuaUcuguaug...................................................................... 298 1 Ch1
....................ucuuugAuuaucuagcuguaug...................................................................... 16 1 Ch1
....................ucuuugguuaucuagcGguaug...................................................................... 12 1 Ch1
....................ucuuugguuaucuagcAguaug...................................................................... 30 1 Ch1
....................uGuuugguuaucuagcuguaug...................................................................... 10 1 Ch1
....................ucuuugguuaucuagcuAuaug...................................................................... 21 1 Ch1
....................ucuuugguuaucuagcuguGug...................................................................... 57 1 Ch1
```

```
                    Mature                              Star
gucggcaggcauugauguuuucuuugguuaucuagcuguaugagugugacugacgucauaaagcuagguuaccaaaguuaaaaacaaucgccagaaaucagcuggaugacag
....................ucuuugguuaucuagcuguaCg......................................................................    45        1    Ch1
....................ucuuugguuaucuGgcuguaug......................................................................    39        1    Ch1
....................ucuuugguuaucuagGuguaug......................................................................    2         1    Ch1
....................ucuuugguuaucuagcuguauA......................................................................    901       1    Ch1
....................ucuuugguuaAcuagcuguaug......................................................................    37        1    Ch1
....................ucuuugguuauUuagcuguaug......................................................................    50        1    Ch1
....................ucuuugguuaucuagcuguCug......................................................................    10        1    Ch1
....................uUuuugguuaucuagcuguaug......................................................................    86        1    Ch1
....................ucGuugguuaucuagcuguaug......................................................................    6         1    Ch1
....................ucuuugguGaucuagcuguaug......................................................................    1         1    Ch1
....................ucuuugguuaucuagcCguaug......................................................................    20        1    Ch1
....................ucuuugguuaucuCgcuguaug......................................................................    175       1    Ch1
....................ucuuAgguuaucuagcuguaug......................................................................    1098      1    Ch1
....................ucuuugguuauGuagcuguaug......................................................................    19        1    Ch1
....................ucuuuggGuaucuagcuguaug......................................................................    16        1    Ch1
....................ucuuugguAaucuagcuguaug......................................................................    124       1    Ch1
....................ucuuugguuGucuagcuguaug......................................................................    16        1    Ch1
....................ucuuugguuaucuagcuguaAg......................................................................    29        1    Ch1
....................uAuuugguuaucuagcuguaug......................................................................    187       1    Ch1
....................ucuuugguuaucuagcuguaGg......................................................................    8         1    Ch1
....................ucuuugguuaucuagcuguUug......................................................................    45        1    Ch1
....................ucuuGgguuaucuagcuguaug......................................................................    6388      1    Ch1
....................ucuuugguuaucuUgcuguaug......................................................................    102       1    Ch1
....................Ccuuugguuaucuagcuguaug......................................................................    191       1    Ch1
....................ucuuugguuaucuagcuguaug......................................................................    151785    0    Ch1
....................ucuuugCuuaucuagcuguaug......................................................................    15        1    Ch1
....................ucuuugguCaucuagcuguaug......................................................................    51        1    Ch1
....................ucuuugguuauAuagcuguaug......................................................................    7         1    Ch1
....................ucuuugguuaucuagcuguauU......................................................................    606       1    Ch1
....................ucuuugguuaucAagcuguaug......................................................................    26        1    Ch1
....................ucuuugguuaucuagcuguauC......................................................................    15        1    Ch1
....................ucuuugguuaucuaAcuguaug......................................................................    20        1    Ch1
....................Acuuugguuaucuagcuguaug......................................................................    298       1    Ch1
....................ucuuugguuaucuagcuCuaug......................................................................    6         1    Ch1
....................ucuuugguuaucuagcuUuaug......................................................................    69        1    Ch1
....................ucuuUgguuaucuagcuguaug......................................................................    97        1    Ch1
....................ucuuugguuaCcuagcuguaug......................................................................    29        1    Ch1
....................Gcuuugguuaucuagcuguaug......................................................................    31        1    Ch1
....................ucuuugguuCucuagcuguaug......................................................................    416       1    Ch1
....................ucuuugguuaucuagcugCaug......................................................................    39        1    Ch1
....................ucuuugguuaucGagcuguaug......................................................................    6         1    Ch1
....................ucuuugguuaucuagAuguaug......................................................................    10        1    Ch1
....................ucuuugUuuaucuagcuguaug......................................................................    152       1    Ch1
....................ucuAugguuaucuagcuguaug......................................................................    178       1    Ch1
....................ucuuugguuaucuaCcuguaug......................................................................    6         1    Ch1
....................ucuGugguuaucuagcuguaug......................................................................    1106      1    Ch1
....................Ncuuugguuaucuagcuguaug......................................................................    109       1    Ch1
....................ucuCugguuaucuagcuguaug......................................................................    182       1    Ch1
....................ucAuugguuaucuagcuguaug......................................................................    16        1    Ch1
....................ucuuugguuaGcuagcuguaug......................................................................    2         1    Ch1
....................ucuuugguuaucCagcuguaug......................................................................    21        1    Ch1
....................ucuuuggCuaucuagcuguaug......................................................................    28        1    Ch1
....................ucuuuAguuaucuagcuguaug......................................................................    15        1    Ch1
....................ucuuugguuaucuagUuguaug......................................................................    116       1    Ch1
....................ucuuugguuaucuagcugAaug......................................................................    8         1    Ch1
....................ucuuuggAuaucuagcuguaug......................................................................    23        1    Ch1
....................ucuuugguuaucuagcugGaug......................................................................    65        1    Ch1
....................ucCuugguuaucuagcuguaug......................................................................    438       1    Ch1
....................ucuuCgguuaucuagcuguaug......................................................................    886       1    Ch1
....................ucuuuCguuaucuagcuguaug......................................................................    8         1    Ch1
....................ucuuugguNaucuagcuguauga.....................................................................    1         1    Ch1
....................ucuuuAguuaucuagcuguauga.....................................................................    4         1    Ch1
....................ucuuugguuaucuagcugGauga.....................................................................    13        1    Ch1
....................ucuuugUuuaucuagcuguauga.....................................................................    35        1    Ch1
....................ucuuugguuCucuagcuguauga.....................................................................    95        1    Ch1
....................ucuuugguuaucuagcuguCuga.....................................................................    3         1    Ch1
....................uUuuugguuaucuagcuguauga.....................................................................    24        1    Ch1
....................Gcuuugguuaucuagcuguauga.....................................................................    7         1    Ch1
....................ucuuugguuaAcuagcuguauga.....................................................................    4         1    Ch1
....................ucAuugguuaucuagcuguauga.....................................................................    4         1    Ch1
```

```
                    Mature                                Star
gucggcaggcauugauguuuucuuugguuaucuagcuguaugagugugacugacgucauaaagcuagguuaccaaaguuaaaaacaaucgccagaaaucagcuggaugacag
....................Acuuugguuaucuagcuguauga.....................................................................    55      1    Ch1
....................ucuuugguuaucuagcuCuauga.....................................................................    1       1    Ch1
....................ucuuuggGuaucuagcuguauga.....................................................................    6       1    Ch1
....................uAuuugguuaucuagcuguauga.....................................................................    39      1    Ch1
....................ucuuugguuaucuagcuguauUa.....................................................................    26      1    Ch1
....................ucuCugguuaucuagcuguauga.....................................................................    36      1    Ch1
....................ucuuugguuaucuaAcuguauga.....................................................................    9       1    Ch1
....................ucuuugguuaucuagcuguaugG.....................................................................    93      1    Ch1
....................ucuGugguuaucuagcuguauga.....................................................................    218     1    Ch1
....................ucuuugguuaucuagAuguauga.....................................................................    4       1    Ch1
....................ucuuugguuaucuagcuguaCga.....................................................................    5       1    Ch1
....................ucuuugguuaucuagGuguauga.....................................................................    1       1    Ch1
....................ucuuugguuaucuagcuguauCa.....................................................................    4       1    Ch1
....................ucuuugguuaucuagcuguaAga.....................................................................    3       1    Ch1
....................ucuuuUguuaucuagcuguauga.....................................................................    20      1    Ch1
....................ucuuugguuaGcuagcuguauga.....................................................................    3       1    Ch1
....................ucuuugguuaucuUgcuguauga.....................................................................    19      1    Ch1
....................ucGuugguuaucuagcuguauga.....................................................................    3       1    Ch1
....................ucuuugguuaucuagcuguauga.....................................................................    31050   0    Ch1
....................ucuuugguuauUuagcuguauga.....................................................................    9       1    Ch1
....................ucuuuggCuaucuagcuguauga.....................................................................    5       1    Ch1
....................ucuuugguuaucuagcCguauga.....................................................................    3       1    Ch1
....................ucuuugguuaucGagcuguauga.....................................................................    2       1    Ch1
....................ucuuugguuaucCagcuguauga.....................................................................    3       1    Ch1
....................ucuuugguuaCcuagcuguauga.....................................................................    2       1    Ch1
....................ucuuugguuaucuagcuguaugU.....................................................................    4745    1    Ch1
....................ucuuugguuaucuagcugAauga.....................................................................    3       1    Ch1
....................ucuuugguuaucuagUuguauga.....................................................................    19      1    Ch1
....................ucuuugguuaucuagcugCauga.....................................................................    8       1    Ch1
....................ucuuAgguuaucuagcuguauga.....................................................................    216     1    Ch1
....................ucuuugguuaucuagcuUuauga.....................................................................    18      1    Ch1
....................ucuuGgguuaucuagcuguauga.....................................................................    1297    1    Ch1
....................ucuuuggAuaucuagcuguauga.....................................................................    5       1    Ch1
....................ucuuuggUAaucuagcuguauga.....................................................................    33      1    Ch1
....................ucuuugguuaucuaUcuguauga.....................................................................    71      1    Ch1
....................ucuuugguCaucuagcuguauga.....................................................................    9       1    Ch1
....................ucuAugguuaucuagcuguauga.....................................................................    23      1    Ch1
....................ucuuugguuaucuGgcuguauga.....................................................................    12      1    Ch1
....................ucuuugguuaucAagcuguauga.....................................................................    4       1    Ch1
....................ucuugAuuaucuagcuguauga.....................................................................    1       1    Ch1
....................ucCuugguuaucuagcuguauga.....................................................................    96      1    Ch1
....................uGuuugguuaucuagcuguauga.....................................................................    1       1    Ch1
....................ucuuugguuaucuCgcuguauga.....................................................................    25      1    Ch1
....................ucuuugguuauAuagcuguauga.....................................................................    2       1    Ch1
....................ucuuugguuaucuagcuguauAa.....................................................................    171     1    Ch1
....................ucuuugguuaucuagcAguauga.....................................................................    6       1    Ch1
....................Ccuuugguuaucuagcuguauga.....................................................................    38      1    Ch1
....................ucuuugguuaucuaCcuguauga.....................................................................    2       1    Ch1
....................Ncuuugguuaucuagcuguauga.....................................................................    21      1    Ch1
....................ucuuCgguuaucuagcuguauga.....................................................................    184     1    Ch1
....................ucuuugguuGucuagcuguauga.....................................................................    9       1    Ch1
....................ucuuuCguuaucuagcuguauga.....................................................................    4       1    Ch1
....................ucuuugguuaucuagcuguaugC.....................................................................    393     1    Ch1
....................ucuuugguuaucuagcuguGuga.....................................................................    8       1    Ch1
....................ucuuugguuaucuagcuguUuga.....................................................................    10      1    Ch1
....................ucuuugguuaucuagcuAuauga.....................................................................    9       1    Ch1
....................ucuuugguuaucuagcuguaugag....................................................................    75      0    Ch1
....................ucuuugguuaucuCgcuguaugag....................................................................    1       1    Ch1
....................ucuuugguuCucuagcuguaugag....................................................................    1       1    Ch1
....................ucuuugguuaucuagcuguaugaA....................................................................    1392    1    Ch1
....................ucuuugguuaCcuagcuguaugag....................................................................    1       1    Ch1
....................ucuuugguuaucuagcuguaugaC....................................................................    65      1    Ch1
....................ucuuugguuaucuagcuguCugag....................................................................    4       1    Ch1
....................ucuuugguuaucuagcuguaugaU....................................................................    2072    1    Ch1
....................ucuuGgguuaucuagcuguaugag....................................................................    1       1    Ch1
....................ucuuugguuaucuagcuguaugUg....................................................................    34      1    Ch1
....................ucuuugguuaucuagcuguaugCg....................................................................    1       1    Ch1
....................ucuuugguuaucuagcuguaugagA...................................................................    8       1    Ch1
....................ucuuugguuaucuagcuguaugUgu...................................................................    7       1    Ch1
....................ucuuugguuaucuagcuguaugagu...................................................................    8       0    Ch1
```

Mature Star

```
gucggcaggcauugauguuuucuuugguuaucuagcuguaugagugugacugacgucauaaagcuagguuaccaaaguuaaaaacaaucgccagaaaucagcuggaugacag
....................ucuuugguuaucuagcuguaugaUu...................................................................   123   1   Ch1
....................ucuuugguuaucuagcuguaugagC...................................................................   1     1   Ch1
....................ucuuugguuaucuagcuguaugaAu...................................................................   86    1   Ch1
....................ucuuugguuaucuagcuguauAagu...................................................................   1     1   Ch1
....................ucuuugguuaucuagcuguaugaCu...................................................................   22    1   Ch1
....................ucuuugguuaucuagcuguaugaguA..................................................................   1     1   Ch1
.....................cuuugguuaucuagcugu.........................................................................   1     0   Ch1
.....................cuuugguuCucuagcugua........................................................................   1     1   Ch1
.....................cuuGgguuaucuagcugua........................................................................   1     1   Ch1
.....................cuuugguuaucuagcuUua........................................................................   1     1   Ch1
.....................cuuugguuaucuagcugua........................................................................   8     0   Ch1
.....................cuuugguuaucuagcuguau.......................................................................   10    0   Ch1
.....................cAuugguuaucuagcuguau.......................................................................   1     1   Ch1
.....................cuuugguuaucuagcuUuaug......................................................................   1     1   Ch1
.....................Uuuugguuaucuagcuguaug......................................................................   1     1   Ch1
.....................cuuugguuaucuagcGguaug......................................................................   1     1   Ch1
.....................cuuugguuaucuUgcuguaug......................................................................   1     1   Ch1
.....................cuuugguuaucuagcuguaug......................................................................   72    0   Ch1
.....................cuuugguuCucuagcuguaug......................................................................   6     1   Ch1
.....................cuuugguuaucuagcugGaug......................................................................   1     1   Ch1
.....................cuuugguuaucuagcugAaug......................................................................   2     1   Ch1
.....................cuuugguuauAuagcuguaug......................................................................   1     1   Ch1
.....................cuuuggAuaucuagcuguaug......................................................................   1     1   Ch1
.....................cuuGgguuaucuagcuguaug......................................................................   6     1   Ch1
.....................Auuugguuaucuagcuguaug......................................................................   2     1   Ch1
.....................cuuuUguuaucuagcuguaug......................................................................   14    1   Ch1
.....................cuuugguuaucuGgcuguaug......................................................................   1     1   Ch1
.....................cuuAgguuaucuagcuguaug......................................................................   3     1   Ch1
.....................cuuugguuaucuagcuguauA......................................................................   1     1   Ch1
.....................cuuugguuaucuagGuguaug......................................................................   1     1   Ch1
.....................cuuugguuaucuagcuguauga.....................................................................   28    0   Ch1
.....................cuuugguuaucuagcuguaugU.....................................................................   2     1   Ch1
.....................cuuuAguuaucuagcuguauga.....................................................................   1     1   Ch1
.....................cuuugguuaucuagcuguaugG.....................................................................   1     1   Ch1
.....................cuuugguuaucuagcuguauCa.....................................................................   1     1   Ch1
.....................cuCugguuaucuagcuguauga.....................................................................   1     1   Ch1
.....................cuuGgguuaucuagcuguauga.....................................................................   1     1   Ch1
.....................cuuuUguuaucuagcuguauga.....................................................................   6     1   Ch1
.....................cuuugguuauAuagcuguauga.....................................................................   1     1   Ch1
.....................cuuugguuCucuagcuguauga.....................................................................   1     1   Ch1
.....................cuuugguuaucuagcuguaugaU....................................................................   1     1   Ch1
.....................cuuugguuaucuagcuguaugaA....................................................................   4     1   Ch1
.....................cuuugguuaucuagcuguaugag....................................................................   1     0   Ch1
......................uuugguuaucuagcugua........................................................................   1     0   Ch1
......................uuugguuaucuagcuguau.......................................................................   7     0   Ch1
......................uCugguuaucuagcuguaug......................................................................   1     1   Ch1
......................uuuUguuaucuagcuguaug......................................................................   1     1   Ch1
......................uuugAuuaucuagcuguaug......................................................................   1     1   Ch1
......................uuugUuuaucuagcuguaug......................................................................   2     1   Ch1
......................uuugguuaucuagcuguaug......................................................................   59    0   Ch1
......................uuugguuaucuagcuguCuga.....................................................................   1     1   Ch1
......................uuugguuaucuagcuguaugC.....................................................................   1     1   Ch1
......................uuugguuaAcuagcuguauga.....................................................................   1     1   Ch1
......................uuugguuaucuagcuguauga.....................................................................   61    0   Ch1
......................uuugUuuaucuagcuguauga.....................................................................   1     1   Ch1
......................uuugguuaucuagcuguaugU.....................................................................   2     1   Ch1
......................uuugguuaucuagcuguaugaA....................................................................   2     1   Ch1
......................uuugguuaucuagcuguaugaU....................................................................   1     1   Ch1
......................uuugguuaucuagcuguaugaAu...................................................................   1     1   Ch1
.......................uugguuaucuaUcuguaug......................................................................   1     1   Ch1
.......................uugguuaucuagcuguaug......................................................................   6     0   Ch1
.......................uugguuaucuagcuguauga.....................................................................   1     0   Ch1
.......................uugguuaucuagcuguaugaA....................................................................   1     1   Ch1
........................ugguuaucuagcuguaug......................................................................   2     0   Ch1
........................ugguuaucuCgcuguaug......................................................................   1     1   Ch1
........................uUguuaucuagcuguaug......................................................................   1     1   Ch1
.........................gguuaucuagcuguauga.....................................................................   1     0   Ch1
.........................................................auaaagcuagguuaccaaa....................................   2     0   Ch1
.........................................................auCaagcuagguuaccaaag...................................   1     1   Ch1
.........................................................auaaagcuagguuaccaaag...................................   1     0   Ch1
```

```
                    Mature                              Star
gucggcaggcauugauguuuucuuugguuaucuagcuguaugagugugacugacgucauaaagcuagguuaccaaaguuaaaaacaaucgccagaaaucagcuggaugacag
.........................................................auaaagcuagguuaccaaagu..................................    22    0    Ch1
.........................................................auaaagcuagguuaccaaaguu.................................    95    0    Ch1
.........................................................Cuaaagcuagguuaccaaaguu.................................    2     1    Ch1
.........................................................auaaGgcuagguuaccaaaguu.................................    1     1    Ch1
.........................................................auaaagcuagguuaccaaaguA.................................    1     1    Ch1
.........................................................auaaagcuagCuuaccaaaguu.................................    1     1    Ch1
.........................................................auaaagcuagguuUccaaaguu.................................    2     1    Ch1
.........................................................Uuaaagcuagguuaccaaaguu.................................    1     1    Ch1
.........................................................Nuaaagcuagguuaccaaaguua................................    1     1    Ch1
.........................................................auaaagcuagguuaccaaaguua................................    48    0    Ch1
.........................................................auaaGgcuagguuaccaaaguua................................    1     1    Ch1
.........................................................Cuaaagcuagguuaccaaaguua................................    2     1    Ch1
.........................................................auaaagcuagguuaccaaaguuU................................    1     1    Ch1
.........................................................auaaUgcuagguuaccaaaguua................................    1     1    Ch1
.........................................................auaaagcuagguuaccaaaguuaa...............................    1     0    Ch1
..........................................................uaaagcuagguuaccaaag...................................    2     0    Ch1
..........................................................uaaaUcuagguuaccaaag...................................    1     1    Ch1
..........................................................uaaaUcuagguuaccaaagu..................................    3     1    Ch1
..........................................................uCaagcuagguuaccaaagu..................................    1     1    Ch1
..........................................................uaaagcuagguuaccaaagC..................................    1     1    Ch1
..........................................................uaaaAcuagguuaccaaagu..................................    1     1    Ch1
..........................................................Caaagcuagguuaccaaagu..................................    1     1    Ch1
..........................................................uaaagcuagguuaccaaagu..................................    35    0    Ch1
..........................................................uaaagcuagguuaccUaagu..................................    1     1    Ch1
..........................................................uaaaUcuagguuaccaaaguu.................................    15    1    Ch1
..........................................................uaaagcuagguuaccaaaguA.................................    1     1    Ch1
..........................................................uaCagcuagguuaccaaaguu.................................    1     1    Ch1
..........................................................uaaaCcuagguuaccaaaguu.................................    5     1    Ch1
..........................................................uaaagUuagguuaccaaaguu.................................    1     1    Ch1
..........................................................uaaagcuagguGaccaaaguu.................................    1     1    Ch1
..........................................................uaaagcuagUuuaccaaaguu.................................    1     1    Ch1
..........................................................uaaUgcuagguuaccaaaguu.................................    1     1    Ch1
..........................................................uaaagcuagguuaccaaaguC.................................    1     1    Ch1
..........................................................uaaaAcuagguuaccaaaguu.................................    2     1    Ch1
..........................................................uGaagcuagguuaccaaaguu.................................    1     1    Ch1
..........................................................uaaagcCagguuaccaaaguu.................................    1     1    Ch1
..........................................................uaaagcuagguuaccaaaguu.................................    312   0    Ch1
..........................................................uaaagcuagguuaccaaaUuu.................................    1     1    Ch1
..........................................................Aaaagcuagguuaccaaaguu.................................    1     1    Ch1
..........................................................uaaaUcuagguuaccaaaguua................................    5     1    Ch1
..........................................................uaaagcuagguuaccaCaguua................................    1     1    Ch1
..........................................................Naaagcuagguuaccaaaguua................................    1     1    Ch1
..........................................................uaaagcuagguuaccaaaguua................................    111   0    Ch1
..........................................................uaaagcuagguuaccaaaguuaa...............................    4     0    Ch1
...........................................................aaagcuagguuaccaaaguu.................................    3     0    Ch1
...........................................................aaagcuagguuaccaaaguua................................    2     0    Ch1
```

Provisional ID	:	Sc28yqQ_3523_1390
Score total	:	112237.6
Score for star read(s)	:	3.9
Score for read counts	:	112227.4
Score for mfe	:	1.6
Score for randfold	:	1.6
Score for cons. seed	:	3
Total read count	:	220141
Mature read count	:	177841
Loop read count	:	0
Star read count	:	42300

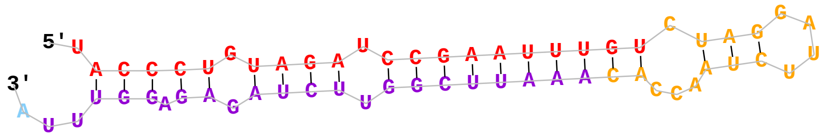

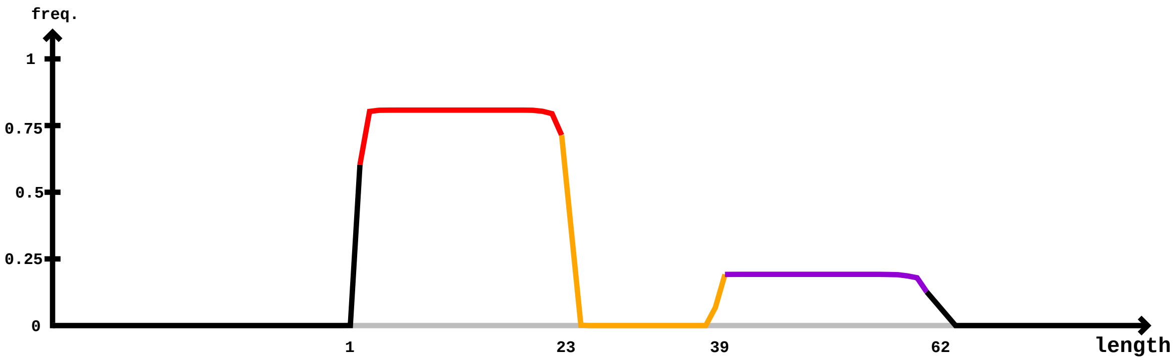


```
                                  Mature                        Star
5'- ggcacgcgcuuuucuuaggugcuuuacaucuacccuguagauccgaauuugucuaggauucuaaccacaaauucgguucuagagagguuuaugugaugcauacgaaacgacc   -3'   obs
    ggcacgcgcuuuucuuaggugcuuuacaucuacccuguagauccgaauuugucuaggauucuaaccacaaauucgguucuagagagguuuaugugaugcauacgaaacgacc         exp
    .((..((((((.....)))))).((((((..(((((.((((.((((((((((.(((....)))...)))))))))).)))).)).)))..)))))).)).............   reads   mm   sample
    .............................cuacccuguagauccgaauuug.............................................................   1       0    He1
    .............................cuacccuguagauccgaauuugA............................................................   1       1    He1
    ..............................uacccuguagauccgaau................................................................   10      0    He1
    ..............................uacUcuguagauccgaauu...............................................................   1       1    He1
    ..............................uacccuguagCuccgaauu...............................................................   1       1    He1
    ..............................uacccuguagauccgaauA...............................................................   1       1    He1
    ..............................uaccUuguagauccgaauu...............................................................   2       1    He1
    ..............................uGcccuguagauccgaauu...............................................................   1       1    He1
    ..............................uacccuguagauccgaauu...............................................................   52      0    He1
    ..............................uaccUuguagauccgaauuu..............................................................   1       1    He1
    ..............................uaccGuguagauccgaauuu..............................................................   2       1    He1
    ..............................uacUcuguagauccgaauuu..............................................................   1       1    He1
    ..............................uacccuguagauccgaauuA..............................................................   1       1    He1
    ..............................uacccuguCgauccgaauuu..............................................................   1       1    He1
    ..............................uacccuguagaucAgaauuu..............................................................   1       1    He1
    ..............................uacccuguaAauccgaauuu..............................................................   1       1    He1
    ..............................uacccuguagauccgUauuu..............................................................   1       1    He1
    ..............................uaGccuguagauccgaauuu..............................................................   1       1    He1
    ..............................uacccuguagauccgaauuu..............................................................   134     0    He1
    ..............................uaAccuguagauccgaauuu..............................................................   3       1    He1
    ..............................uacccuguagaucAgaauuug.............................................................   1       1    He1
    ..............................uacccuguagauccgaauuug.............................................................   1552    0    He1
    ..............................uacccuguagauccgaUuuug.............................................................   1       1    He1
    ..............................uacccuguagauccgaauuuC.............................................................   1       1    He1
    ..............................uacccuguCgauccgaauuug.............................................................   9       1    He1
    ..............................Cacccuguagauccgaauuug.............................................................   1       1    He1
    ..............................uacccuguagauAcgaauuug.............................................................   4       1    He1
    ..............................uacAcuguagauccgaauuug.............................................................   1       1    He1
    ..............................uaccUuguagauccgaauuug.............................................................   20      1    He1
    ..............................uCcccuguagauccgaauuug.............................................................   3       1    He1
    ..............................uacccuguagCuccgaauuug.............................................................   8       1    He1
    ..............................uacccuguagauccgUauuug.............................................................   2       1    He1
    ..............................uacUcuguagauccgaauuug.............................................................   5       1    He1
```

Mature **Star**

ggcacgcgcuuuucuuaggugcuuuacaucuacccuguagauccgaauuugucuaggauucuaaccacaaaucgguucuagagagguuuaugugaugcauacgaaacgacc			
..............................uacccuguagauccUaauuug..	3	1	He1
..............................uacccuguagUuccgaauuug..	1	1	He1
..............................uaGccuguagauccgaauuug..	2	1	He1
..............................uacccuguagGuccgaauuug..	1	1	He1
..............................uacccuguagauccgaauuCg..	1	1	He1
..............................uacccuguagauccgCauuug..	1	1	He1
..............................Nacccuguagauccgaauuug..	2	1	He1
..............................uacccuguaAauccgaauuug..	3	1	He1
..............................Aacccuguagauccgaauuug..	3	1	He1
..............................uaUccuguagauccgaauuug..	5	1	He1
..............................uacccuUuagauccgaauuug..	2	1	He1
..............................uacccGguagauccgaauuug..	1	1	He1
..............................uacccuguagauUcgaauuug..	4	1	He1
..............................uacccuguagauccgaCuuug..	2	1	He1
..............................uacccuguagauccgaauuuA..	7	1	He1
..............................Gacccuguagauccgaauuug..	1	1	He1
..............................uacccuguagaucUgaauuug..	2	1	He1
..............................uacccuguaUauccgaauuug..	1	1	He1
..............................uaAccuguagauccgaauuug..	12	1	He1
..............................uacccuguagauccgaauGug..	1	1	He1
..............................uacccuguagauccgaauuuU..	1	1	He1
..............................uaccGuguagauccgaauuug..	12	1	He1
..............................uacccAguagauccgaauuugu...	1	1	He1
..............................uacccuguagauccgaCuuugu...	14	1	He1
..............................Gacccuguagauccgaauuugu...	1	1	He1
..............................uaUccuguagauccgaauuugu...	11	1	He1
..............................uacccuguagauccgaauuugC...	289	1	He1
..............................uacccuguagaCccgaauuugu...	1	1	He1
..............................uacccuguagaAccgaauuugu...	1	1	He1
..............................uacccuguaAauccgaauuugu...	1	1	He1
..............................uaccAuguagauccgaauuugu...	1	1	He1
..............................uacccuguagauccgaUuuugu...	3	1	He1
..............................uacccuguagauccAaauuugu...	1	1	He1
..............................uacccuguagauccUaauuugu...	11	1	He1
..............................uacccuguagauccgaaAuugu...	4	1	He1
..............................uacccuguagauccgaauuAgu...	1	1	He1
..............................uGcccuguagauccgaauuugu...	2	1	He1
..............................uaccGuguagauccgaauuugu...	61	1	He1
..............................uacccuguGgauccgaauuugu...	1	1	He1
..............................uacccuguagauccgaauuCgu...	6	1	He1
..............................uUcccuguagauccgaauuugu...	3	1	He1
..............................uaccUuguagauccgaauuugu...	100	1	He1
..............................uacccuguagaucUgaauuugu...	1	1	He1
..............................Aacccuguagauccgaauuugu...	11	1	He1
..............................uacccuguagUuccgaauuugu...	3	1	He1
..............................uacGcuguagauccgaauuugu...	1	1	He1
..............................uacccuguagauAcgaauuugu...	4	1	He1
..............................uacccuguagauccgaauuugA...	78	1	He1
..............................Nacccuguagauccgaauuugu...	4	1	He1
..............................uacccugCagauccgaauuugu...	2	1	He1
..............................uacccuguagauccgGauuugu...	1	1	He1
..............................uacccuguagauccgCauuugu...	2	1	He1
..............................uacccuguagauccgUauuugu...	7	1	He1
..............................uacccCguagauccgaauuugu...	4	1	He1
..............................uacccuguagauccgaauGugu...	7	1	He1
..............................uacccuguagauccCaauuugu...	1	1	He1
..............................uacccuguagauccgaaCuugu...	1	1	He1
..............................uCcccuguagauccgaauuugu...	14	1	He1
..............................uacccuguUgauccgaauuugu...	3	1	He1
..............................uacccuUuagauccgaauuugu...	6	1	He1
..............................uaAccuguagauccgaauuugu...	69	1	He1
..............................uacccuguagCuccgaauuugu...	26	1	He1
..............................uacccuguagauccgaauCugu...	2	1	He1
..............................uacccuguagauccgaauuugG...	10	1	He1
..............................uacccuguagaucAgaauuugu...	2	1	He1
..............................uacccuguagauccgaGuuugu...	2	1	He1
..............................uacccugGagauccgaauuugu...	1	1	He1
..............................uacccuguCgauccgaauuugu...	54	1	He1
..............................uacccGguagauccgaauuugu...	3	1	He1
..............................uacccuguagauccgaauuuAu...	2	1	He1

Mature **Star**

ggcacgcgcuuuucuuaggugcuuuacaucuacccuguagauccgaauuugucuaggauucuaaccacaaauucgguucuagagagguuuaugugaugcauacgaaacgacc			
..............................uacccuguaUauccgaauuugu..	1	1	He1
..............................uacccuguagaucGgaauuugu..	1	1	He1
..............................uaGccuguagauccgaauuugu..	10	1	He1
..............................uacccuguagauUcgaauuugu..	6	1	He1
..............................uacccuguagauccgaauuGgu..	6	1	He1
..............................Cacccuguagauccgaauuugu..	7	1	He1
..............................uacccuguagauccgaauuuUu..	2	1	He1
..............................uacUcuguagauccgaauuugu..	29	1	He1
..............................uacccuguagauccgaauuuCu..	3	1	He1
..............................uacccuguagauccgaauuugu..	8635	0	He1
..............................uacccuAuagauccgaauuugu..	3	1	He1
..............................uacccuguagauccgaauuuguA...	367	1	He1
..............................uacccuguagauccgaauuugAc...	1	1	He1
..............................uaccUuguagauccgaauuuguc...	1	1	He1
..............................uacccuguagauccgaauuuguG...	40	1	He1
..............................uacccuguagauccgaauuuguc...	2	0	He1
..............................uacccuguagauccgaauuuguAu..	2	1	He1
..............................Cacccuguagauccgaauuugucu..	1	1	He1
..............................uacccuguagauccgaauuuguGu..	1	1	He1
...............................acccuguagauccgGauu...	1	1	He1
...............................acccGguagauccgaauu...	1	1	He1
...............................acccuguagauccgaauu...	23	0	He1
...............................acccuguagCuccgaauuu..	1	1	He1
...............................acccCguagauccgaauuu..	1	1	He1
...............................acccuguagauccgaauuu..	42	0	He1
...............................acccuguaCauccgaauuu..	1	1	He1
...............................Gcccuguagauccgaauuug...	1	1	He1
...............................Ccccuguagauccgaauuug...	8	1	He1
...............................acccGguagauccgaauuug...	24	1	He1
...............................acAcuguagauccgaauuug...	4	1	He1
...............................accUuguagauccgaauuug...	2	1	He1
...............................aAccuguagauccgaauuug...	1	1	He1
...............................Ucccuguagauccgaauuug...	3	1	He1
...............................acccuguagauccgaauuuA...	1	1	He1
...............................acccuguagauccgaauuug...	518	0	He1
...............................acccuguagauccgaauuCg...	1	1	He1
...............................acccAguagauccgaauuug...	4	1	He1
...............................acccuguagauUcgaauuug...	3	1	He1
...............................acccuguaAauccgaauuug...	1	1	He1
...............................acccuguagaucUgaauuug...	2	1	He1
...............................acccCguagauccgaauuug...	5	1	He1
...............................acccuguagauccgaauuuC...	1	1	He1
...............................acccuguagauccgaauuuU...	1	1	He1
...............................acccuguaUauccgaauuug...	1	1	He1
...............................acccuguagCuccgaauuug...	2	1	He1
...............................acccuguagauccgaauCugu..	1	1	He1
...............................acccuguagGuccgaauuugu..	1	1	He1
...............................acccuguagauccgaGuuugu..	1	1	He1
...............................Ucccuguagauccgaauuugu..	8	1	He1
...............................acccuguagauccgaauuuUu..	12	1	He1
...............................acccuguagauGcgaauuugu..	1	1	He1
...............................acccuguagauccgaauAugu..	1	1	He1
...............................acccCguagauccgaauuugu..	20	1	He1
...............................acccuguagCuccgaauuugu..	16	1	He1
...............................acccuguagauccgaUuuugu..	4	1	He1
...............................Gcccuguagauccgaauuugu..	2	1	He1
...............................acccuguagauccgaauGugu..	1	1	He1
...............................aAccuguagauccgaauuugu..	7	1	He1
...............................Ccccuguagauccgaauuugu..	44	1	He1
...............................acccuguagauccgaCuuugu..	1	1	He1
...............................acccuguagauccgaauuugC..	166	1	He1
...............................accUuguagauccgaauuugu..	12	1	He1
...............................acccuguagaAccgaauuugu..	3	1	He1
...............................acccuguaUauccgaauuugu..	1	1	He1
...............................acccuguagauccgaauuuCu..	2	1	He1
...............................acccuguagauccgCauuugu..	1	1	He1
...............................acAcuguagauccgaauuugu..	13	1	He1
...............................acccuguagauUcgaauuugu..	4	1	He1
...............................acccuguaCauccgaauuugu..	2	1	He1
...............................aUccuguagauccgaauuugu..	2	1	He1

Mature **Star**

ggcacgcgcuuuucuuaggugcuuuacaucuacccuguagauccgaauuugucuaggauucuaaccacaaauucgguucuagagagguuuaugugaugcauacgaaacgacc			
...............................acccuguagauccgaauuugG..	2	1	He1
...............................acccuguaAauccgaauuugu...	1	1	He1
...............................acccuguagaucUgaauuugu...	2	1	He1
...............................acccuguagauccgaauuuAu...	1	1	He1
...............................acccuguUgauccgaauuugu...	1	1	He1
...............................acUcuguagauccgaauuugu...	8	1	He1
...............................acccuguagauccgaauuugu...	3328	0	He1
...............................acccuUuagauccgaauuugu...	2	1	He1
...............................acccuguagaucAgaauuugu...	1	1	He1
...............................acccuguagauccgaauuGgu...	1	1	He1
...............................acccGguagauccgaauuugu...	136	1	He1
...............................acccAguagauccgaauuugu...	20	1	He1
...............................acccuguagauccgaauuugA...	31	1	He1
...............................acGcuguagauccgaauuugu...	3	1	He1
...............................acccuguagauccgaauuuguA..	74	1	He1
...............................acccuguagauccgaauuugCc..	5	1	He1
...............................acccuguagauGcgaauuuguc..	1	1	He1
...............................acccuguagaAccgaauuuguc..	1	1	He1
...............................acccAguagauccgaauuuguc..	1	1	He1
...............................acccuguagauccgaauuGguc..	1	1	He1
...............................acUcuguagauccgaauuuguc..	1	1	He1
...............................acccugGagauccgaauuuguc..	1	1	He1
...............................acccuguagauccgaauuuguG..	12	1	He1
...............................acccGguagauccgaauuuguc..	17	1	He1
...............................accUuguagauccgaauuuguc..	1	1	He1
...............................acccuAuagauccgaauuuguc..	1	1	He1
...............................acccuguagauccgaauuuguc..	334	0	He1
...............................acccuguagauccAaauuuguc..	1	1	He1
...............................acccCguagauccgaauuuguc..	1	1	He1
...............................Ccccuguagauccgaauuuguc..	2	1	He1
...............................acAcuguagauccgaauuuguc..	1	1	He1
...............................Ucccuguagauccgaauuuguc..	1	1	He1
...............................acccuguagauccgaauuugucG...	5	1	He1
...............................acccuguagauccgaauuugucu...	8	0	He1
...............................acccuguagauccgaauuugucC...	1	1	He1
................................cccuCuagauccgaauuu..	1	1	He1
................................cccuguagauccgaauuug...	8	0	He1
................................cccuguagUuccgaauuug...	1	1	He1
................................cccuUuagauccgaauuug...	1	1	He1
................................cccuAuagauccgaauuug...	1	1	He1
................................cccGguagauccgaauuugu..	2	1	He1
................................cccuUuagauccgaauuugu..	9	1	He1
................................ccUuguagauccgaauuugu..	1	1	He1
................................cccuguagauccgaauuugu..	81	0	He1
................................cccuguagauccgaUuuugu..	1	1	He1
................................cccuguagUuccgaauuugu..	2	1	He1
................................cccuguagauccAaauuugu..	1	1	He1
................................cccuguagauccgaauuugC..	3	1	He1
................................cccuguagauccgaauuugA..	1	1	He1
................................cccuguagauccgaauuuUu..	4	1	He1
................................cccuguagauccgaauuGguc...	1	1	He1
................................cccuguagauccgaauuuguc...	9	0	He1
................................cccuUuagauccgaauuuguc...	1	1	He1
................................cccuguagauccgaauuuguA...	3	1	He1
.................................ccuguagaucUgaauuugu..	1	1	He1
.................................ccuguagauccgaauuugu..	5	0	He1
.................................ccuguagauccgaauuuguc...	1	0	He1
..................................cuguagauccgaauuugu..	3	0	He1
....Ucaaauucgguucuagagagguu.......................	3	1	He1
....Ccaaauucgguucuagagagguuu......................	1	1	He1
....caaaCucgguucuagaga...........................	1	1	He1
....caaauucgguucuagaga...........................	14	0	He1
....caaaGucgguucuagagag..........................	1	1	He1
....caaauucgguucuagagag..........................	7	0	He1
....caaauucgguucuagagaA..........................	11	1	He1
....Uaaauucgguucuagagagg.........................	1	1	He1
....caaauucgguucuagagagA.........................	2	1	He1
....caaaCucgguucuagagagg.........................	1	1	He1
....caaauucgguucuagagagg.........................	15	0	He1
....caaauucgguucuagagaggu........................	18	0	He1

```
                              Mature                       Star
ggcacgcgcuuuucuuaggugcuuuacaucuacccuguagauccgaauuugucuaggauucuaaccacaaauucgguucuagagagguuuaugugaugcauacgaaacgacc
...................................................................caaaAucgguucuagagaggu........................   1     1   He1
...................................................................caaauucgguucuagagaggC........................   5     1   He1
...................................................................caaaGucgguucuagagaggu........................   1     1   He1
...................................................................caaauucgguucuagagaggA........................   3     1   He1
...................................................................caaaAucgguucuagagagguu.......................   1     1   He1
...................................................................caaUuucgguucuagagagguu.......................   1     1   He1
...................................................................caaauucgAuucuagagagguu.......................   1     1   He1
...................................................................caaauucgguucuagagaUguu.......................   1     1   He1
...................................................................Aaaauucgguucuagagagguu.......................   2     1   He1
...................................................................caaauucgguucuagagagguA.......................   1     1   He1
...................................................................caaauucgguucuagagagguu.......................   198   0   He1
...................................................................caaauucgguucuagagaggCu.......................   1     1   He1
...................................................................caaauucgguucuaUagagguu.......................   1     1   He1
...................................................................caaauucgguucuagagagguG.......................   1     1   He1
...................................................................caaauucgguucuagUgagguu.......................   2     1   He1
...................................................................caaaGucgguucuagagagguu.......................   7     1   He1
...................................................................caaauucgguucuagagagguC.......................   4     1   He1
...................................................................caaaCucgguucuagagagguu.......................   1     1   He1
...................................................................cCaauucgguucuagagagguu.......................   1     1   He1
...................................................................caaauucgguucuagagaUguuu......................   3     1   He1
...................................................................caaauucgguuAuagagagguuu......................   2     1   He1
...................................................................caaauucgguucuagagagguuC......................   29    1   He1
...................................................................cGaauucgguucuagagagguuu......................   1     1   He1
...................................................................Uaaauucgguucuagagagguuu......................   5     1   He1
...................................................................caaaCucgguucuagagagguuu......................   10    1   He1
...................................................................caaauucgguuUuagagagguuu......................   1     1   He1
...................................................................caaauucgguucuUgagagguuu......................   1     1   He1
...................................................................caCauucgguucuagagagguuu......................   5     1   He1
...................................................................Gaaauucgguucuagagagguuu......................   2     1   He1
...................................................................caaaGucgguucuagagagguuu......................   72    1   He1
...................................................................caGauucgguucuagagagguuu......................   2     1   He1
...................................................................caaauucUguucuagagagguuu......................   1     1   He1
...................................................................caaaAucgguucuagagagguuu......................   12    1   He1
...................................................................caaauucgAuucuagagagguuu......................   2     1   He1
...................................................................Aaaauucgguucuagagagguuu......................   12    1   He1
...................................................................caaauucgguucuagagaggAuu......................   1     1   He1
...................................................................cCaauucgguucuagagagguuu......................   7     1   He1
...................................................................caaauuUgguucuagagagguuu......................   2     1   He1
...................................................................caaauucgguucuagUgagguuu......................   2     1   He1
...................................................................caaauucgguucuagagagguuu......................   1725  0   He1
...................................................................caaauucgguucuagagagAuuu......................   1     1   He1
...................................................................caaauucgguucuagGgagguuu......................   1     1   He1
...................................................................caaauucgguucuagagagUuuu......................   4     1   He1
...................................................................caaUuucgguucuagagagguuu......................   8     1   He1
...................................................................caaauucgguucuagagaggGuu......................   1     1   He1
...................................................................caaauucgguucuaUagagguuu......................   4     1   He1
...................................................................caaauucgguucuagagUgguuu......................   2     1   He1
...................................................................caaauucgguucuCgagagguuu......................   3     1   He1
...................................................................caaauucgUuucuagagagguuu......................   1     1   He1
...................................................................cCaauucgguucuagagagguuua.....................   1     1   He1
...................................................................caaauucgguucuagagagguuua.....................   15    0   He1
...................................................................caaauucgguucuagagagguuuU.....................   23    1   He1
...................................................................caaauucgguucuagagagguuuaA....................   1     1   He1
....................................................................aaauGcgguucuagagag..........................   2     1   He1
....................................................................aaauucgguucuagagag..........................   26    0   He1
....................................................................aaauucgguucuagagaC..........................   1     1   He1
....................................................................aaaCucgguucuagagag..........................   1     1   He1
....................................................................Caauucgguucuagagag..........................   1     1   He1
....................................................................aaauucAguucuagagagg.........................   1     1   He1
....................................................................Caauucgguucuagagagg.........................   2     1   He1
....................................................................aaauucgguucuagagagg.........................   65    0   He1
....................................................................aaauAcgguucuagagagg.........................   1     1   He1
....................................................................aaUuucgguucuagagagg.........................   1     1   He1
....................................................................aaauGcgguucuagagagg.........................   1     1   He1
....................................................................aaauucgguucuagagagA.........................   11    1   He1
....................................................................aaCuucgguucuagagaggu........................   1     1   He1
....................................................................aaauucgguucuagagaggu........................   133   0   He1
....................................................................aaauucgAuucuagagaggu........................   1     1   He1
....................................................................Uaauucgguucuagagaggu........................   1     1   He1
....................................................................aaauucgguucuagagUggu........................   1     1   He1
```

```
                              Mature                  Star
ggcacgcgcuuuucuuaggugcuuuacaucuacccuguagauccgaauuugucuaggauucuaaccacaaauucgguucuagagagguuuaugugaugcauacgaaacgacc
....................................................................aaauucgguucuagagaggA........................    5     1     He1
....................................................................aaauGcgguucuagagaggu........................    6     1     He1
....................................................................aaauucgguucuagagaggG........................    2     1     He1
....................................................................aaauucgguucuagagaggC........................    20    1     He1
....................................................................aaauGcgguucuagagagguu.......................    55    1     He1
....................................................................Uaauucgguucuagagagguu.......................    6     1     He1
....................................................................aaauucgguucuagagaUguu.......................    1     1     He1
....................................................................aaauucgguucuagagCgguu.......................    2     1     He1
....................................................................aaaAucgguucuagagagguu.......................    3     1     He1
....................................................................aaauucgguucuagagagguA.......................    20    1     He1
....................................................................aGauucgguucuagagagguu.......................    1     1     He1
....................................................................aaGuucgguucuagagagguu.......................    1     1     He1
....................................................................aaauucgguucuagaUagguu.......................    1     1     He1
....................................................................aaauucggAucuagagagguu.......................    1     1     He1
....................................................................aaauucgguucuagagagguG.......................    4     1     He1
....................................................................Caauucgguucuagagagguu.......................    15    1     He1
....................................................................aaauucgguucuUgagagguu.......................    1     1     He1
....................................................................aaauucgguuAuagagagguu.......................    1     1     He1
....................................................................aaauucgguuUuagagagguu.......................    1     1     He1
....................................................................aaauucgguucuagagagguu.......................    1407  0     He1
....................................................................aaauAcgguucuagagagguu.......................    6     1     He1
....................................................................Gaauucgguucuagagagguu.......................    1     1     He1
....................................................................aaauCcgguucuagagagguu.......................    7     1     He1
....................................................................aaauucgguucuagagagguC.......................    93    1     He1
....................................................................aaauucgguucuCgagagguu.......................    3     1     He1
....................................................................aCauucgguucuagagagguu.......................    2     1     He1
....................................................................aaaCucgguucuagagagguu.......................    1     1     He1
....................................................................aaauucgguucuaAagagguu.......................    1     1     He1
....................................................................aaaGucgguucuagagagguu.......................    13    1     He1
....................................................................aaauucgguucuGgagagguu.......................    1     1     He1
....................................................................aaauucUguucuagagagguu.......................    1     1     He1
....................................................................aaCuucgguucuagagagguu.......................    4     1     He1
....................................................................aaauucgguucuagagaggCu.......................    2     1     He1
....................................................................aaauucgguucuagUgagguu.......................    2     1     He1
....................................................................aaauucgguuGuagagagguu.......................    1     1     He1
....................................................................aaauuAgguucuagagagguu.......................    1     1     He1
....................................................................aaauuUgguucuagagagguu.......................    3     1     He1
....................................................................aaauucgguucuCgagagguuu......................    2     1     He1
....................................................................aCauucgguucuagagagguuu......................    4     1     He1
....................................................................aaauucggAucuagagagguuu......................    4     1     He1
....................................................................Uaauucgguucuagagagguuu......................    10    1     He1
....................................................................aaauucgguCcuagagagguuu......................    1     1     He1
....................................................................aaauucggCucuagagagguuu......................    2     1     He1
....................................................................aaauucgguucuagagagguuG......................    2     1     He1
....................................................................aaauucgguucuagagaggGuu......................    3     1     He1
....................................................................Caauucgguucuagagagguuu......................    28    1     He1
....................................................................aaauucgguucuagagaUguuu......................    1     1     He1
....................................................................aaauucgguucuagagagguuu......................    1805  0     He1
....................................................................aaaAucgguucuagagagguuu......................    1     1     He1
....................................................................aaauucgguucuagagaggCuu......................    1     1     He1
....................................................................aaauucgguucuagagUgguuu......................    3     1     He1
....................................................................aaauucgguucuaAagagguuu......................    1     1     He1
....................................................................Naauucgguucuagagagguuu......................    1     1     He1
....................................................................aaaGucgguucuagagagguuu......................    11    1     He1
....................................................................aaauucgguucuagagagguuC......................    37    1     He1
....................................................................aaauucgguucuagGgagguuu......................    5     1     He1
....................................................................aaauucgguucGagagagguuu......................    1     1     He1
....................................................................aaauucgguucuagCgagguuu......................    2     1     He1
....................................................................aaauucgguucuagagaggAuu......................    1     1     He1
....................................................................aaauucUguucuagagagguuu......................    2     1     He1
....................................................................aaauucgguucuagagGgguuu......................    1     1     He1
....................................................................aaauucgguucuagagCgguuu......................    3     1     He1
....................................................................aaauGcgguucuagagagguuu......................    64    1     He1
....................................................................Gaauucgguucuagagagguuu......................    2     1     He1
....................................................................aaauCcgguucuagagagguuu......................    17    1     He1
....................................................................aaauuUgguucuagagagguuu......................    2     1     He1
....................................................................aaCuucgguucuagagagguuu......................    5     1     He1
....................................................................aaauAcgguucuagagagguuu......................    8     1     He1
....................................................................aaauucgguuUuagagagguuu......................    1     1     He1
....................................................................aaauucgguucuagagagUuuu......................    3     1     He1
```

Mature **Star**

ggcacgcgcuuuucuuaggugcuuuacaucuacccuguagauccgaauuugucuaggauucuaaccacaaauucgguucuagagagguuuaugugaugcauacgaaacgacc			
....aaauucgguucuagagagguuuU.....................	27	1	He1
....aaauucgguucuagagagguua......................	13	0	He1
....aaauCcgguucuagagagguua......................	1	1	He1
....aaauucgguucuagagagguuuaA....................	3	1	He1
....aauucgguucuagagaggu........................	1	0	He1
....aauucgguucuagagagguu.......................	1	0	He1
....aauucgguAcuagagagguu.......................	1	1	He1
....Cauucgguucuagagagguu.......................	1	1	He1
....aauucgguucuagagagguuu......................	3	0	He1
....uucgguucuagagagguu.......................	1	0	He1
....ucgguucuagagagguuu......................	1	0	He1
.............................cuacccuguagauccgaauuugu..	3	0	Bl1
.............................cuacUcuguagauccgaauuugu..	1	1	Bl1
.............................cuacccuguagauccgaaGuugu..	1	1	Bl1
..............................uacccuguagauccgaaA..	2	1	Bl1
..............................uacccuguCgauccgaau..	1	1	Bl1
..............................uaAccuguagauccgaau..	1	1	Bl1
..............................uaccGuguagauccgaau..	1	1	Bl1
..............................uacccuguagauccgaau..	48	0	Bl1
..............................uacccuguagauccUaau..	1	1	Bl1
..............................uacccCguagauccgaau..	2	1	Bl1
..............................uacccuguagauccgaauA...	11	1	Bl1
..............................uacccuguUgauccgaauu...	1	1	Bl1
..............................uaccUuguagauccgaauu...	1	1	Bl1
..............................uacccuguCgauccgaauu...	2	1	Bl1
..............................uacccuguagauccgaauC...	1	1	Bl1
..............................Aacccuguagauccgaauu...	1	1	Bl1
..............................uacUcuguagauccgaauu...	2	1	Bl1
..............................uacccuguagCuccgaauu...	1	1	Bl1
..............................uacccuguagauccgaauu...	208	0	Bl1
..............................uacccuguagauccgUauu...	1	1	Bl1
..............................uacccuguagauAcgaauuu..	1	1	Bl1
..............................uaccUuguagauccgaauuu..	4	1	Bl1
..............................uacUcuguagauccgaauuu..	2	1	Bl1
..............................uacccuguagCuccgaauuu..	1	1	Bl1
..............................uacccuguagauccgaauuu..	321	0	Bl1
..............................uacccuguagaGccgaauuu..	1	1	Bl1
..............................uacccuguagauccgaauuG..	1	1	Bl1
..............................uacccuUuagauccgaauuu..	1	1	Bl1
..............................uacccuguCgauccgaauuu..	5	1	Bl1
..............................Aacccuguagauccgaauuu..	1	1	Bl1
..............................uaGccuguagauccgaauuu..	1	1	Bl1
..............................uacccugCagauccgaauuug...	1	1	Bl1
..............................uGcccuguagauccgaauuug...	1	1	Bl1
..............................uacccuguagauccgaCuuug...	2	1	Bl1
..............................uacccuguagauccgCauuug...	1	1	Bl1
..............................uaccAuguagauccgaauuug...	2	1	Bl1
..............................uacccuUuagauccgaauuug...	4	1	Bl1
..............................uacccuguagauccgaauuuU...	1	1	Bl1
..............................uaGccuguagauccgaauuug...	2	1	Bl1
..............................Aacccuguagauccgaauuug...	2	1	Bl1
..............................uacccuguagCuccgaauuug...	8	1	Bl1
..............................uacccuguagaucUgaauuug...	1	1	Bl1
..............................uacccuguagauccAaauuug...	1	1	Bl1
..............................uacccugGagauccgaauuug...	1	1	Bl1
..............................uaUccuguagauccgaauuug...	1	1	Bl1
..............................uacccuAuagauccgaauuug...	1	1	Bl1
..............................uacccuguagauccgUauuug...	3	1	Bl1
..............................uacccuguagauccgaauuug...	1604	0	Bl1
..............................uCcccuguagauccgaauuug...	3	1	Bl1
..............................uacUcuguagauccgaauuug...	6	1	Bl1
..............................uaccGuguagauccgaauuug...	14	1	Bl1
..............................uacccuguagauccgaauAug...	1	1	Bl1
..............................uacccuguCgauccgaauuug...	8	1	Bl1
..............................uacccuguagauccgaauuGg...	1	1	Bl1
..............................uacccuguagaGccgaauuug...	1	1	Bl1
..............................uaAccuguagauccgaauuug...	17	1	Bl1
..............................uacccuguagaucAgaauuug...	1	1	Bl1
..............................uacccuguagauccgaUuuug...	1	1	Bl1

```
                              Mature                                    Star
ggcacgcgcuuuucuuaggugcuuuacaucuacccuguagauccgaauuugucuaggauucuaaccacaaauucgguucuagagagguuuaugugaugcauacgaaacgacc
..............................uaccUuguagauccgaauuug.............................................................  26     1  Bl1
..............................uacccuguagauccgaauuuA.............................................................  5      1  Bl1
..............................Cacccuguagauccgaauuug.............................................................  3      1  Bl1
..............................uacccuguagauccUaauuug.............................................................  2      1  Bl1
..............................uacccuguagauAcgaauuugu............................................................  7      1  Bl1
..............................uaccAuguagauccgaauuugu............................................................  3      1  Bl1
..............................uacccuguagauccgaauuCgu............................................................  2      1  Bl1
..............................uacccuguaUauccgaauuugu............................................................  2      1  Bl1
..............................uacccuguagauccgaUuuugu............................................................  2      1  Bl1
..............................uacccuguagauccgaauuuCu............................................................  1      1  Bl1
..............................uUcccuguagauccgaauuugu............................................................  3      1  Bl1
..............................uacccuguagauccgaauuugu............................................................  14772  0  Bl1
..............................uacccCguagauccgaauuugu............................................................  4      1  Bl1
..............................uacccuguagCuccgaauuugu............................................................  45     1  Bl1
..............................uacccuguagauccgaauuGgu............................................................  7      1  Bl1
..............................uacccuguagUuccgaauuugu............................................................  7      1  Bl1
..............................uaccGuguagauccgaauuugu............................................................  104    1  Bl1
..............................uacccuguagauccgaauCugu............................................................  2      1  Bl1
..............................uaUccuguagauccgaauuugu............................................................  20     1  Bl1
..............................uacccuguagaucUgaauuugu............................................................  9      1  Bl1
..............................uacccuguagauccgaauuuAu............................................................  1      1  Bl1
..............................uaAccuguagauccgaauuugu............................................................  132    1  Bl1
..............................uacccugGagauccgaauuugu............................................................  1      1  Bl1
..............................uacccuCuagauccgaauuugu............................................................  1      1  Bl1
..............................uacccGguagauccgaauuugu............................................................  6      1  Bl1
..............................uacccuguagauccgaCuuugu............................................................  18     1  Bl1
..............................Cacccuguagauccgaauuugu............................................................  24     1  Bl1
..............................uacccugCagauccgaauuugu............................................................  5      1  Bl1
..............................uacccuguagauccgaauuugC............................................................  156    1  Bl1
..............................uacAcuguagauccgaauuugu............................................................  1      1  Bl1
..............................uacccuguagauccgaauAugu............................................................  1      1  Bl1
..............................uacccuguagauccgaaAuugu............................................................  5      1  Bl1
..............................uacccuguagauUcgaauuugu............................................................  8      1  Bl1
..............................uacccuAuagauccgaauuugu............................................................  2      1  Bl1
..............................Aacccuguagauccgaauuugu............................................................  24     1  Bl1
..............................uacccuguagauccgaauuuUu............................................................  7      1  Bl1
..............................uacccuguUgauccgaauuugu............................................................  7      1  Bl1
..............................uacccuguagaGccgaauuugu............................................................  1      1  Bl1
..............................uacccAguagauccgaauuugu............................................................  2      1  Bl1
..............................Nacccuguagauccgaauuugu............................................................  7      1  Bl1
..............................uacccuguagauccgaauuugA............................................................  80     1  Bl1
..............................uacccuguagaCccgaauuugu............................................................  2      1  Bl1
..............................uacccugAagauccgaauuugu............................................................  3      1  Bl1
..............................uacccuguagauccCaauuugu............................................................  1      1  Bl1
..............................uaccUuguagauccgaauuugu............................................................  151    1  Bl1
..............................uCcccuguagauccgaauuugu............................................................  32     1  Bl1
..............................uacccuguagauccAaauuugu............................................................  2      1  Bl1
..............................uacccuguagauccgaauuAgu............................................................  2      1  Bl1
..............................uGcccuguagauccgaauuugu............................................................  2      1  Bl1
..............................uacccuguCgauccgaauuugu............................................................  116    1  Bl1
..............................uacccuguagaucGgaauuugu............................................................  1      1  Bl1
..............................uacccuguagauccgaGuuugu............................................................  2      1  Bl1
..............................uacccuguagauccgaauuugG............................................................  1      1  Bl1
..............................uacccuguagauccUaauuugu............................................................  21     1  Bl1
..............................uacccuguaAauccgaauuugu............................................................  3      1  Bl1
..............................uacccuguagaucAgaauuugu............................................................  10     1  Bl1
..............................uacccuguagGuccgaauuugu............................................................  1      1  Bl1
..............................uacccuguagauccgCauuugu............................................................  1      1  Bl1
..............................uaGccuguagauccgaauuugu............................................................  26     1  Bl1
..............................uacccuguagauGcgaauuugu............................................................  1      1  Bl1
..............................uacGcuguagauccgaauuugu............................................................  1      1  Bl1
..............................Gacccuguagauccgaauuugu............................................................  1      1  Bl1
..............................uacccuUuagauccgaauuugu............................................................  17     1  Bl1
..............................uacccuguagauccgaauGugu............................................................  16     1  Bl1
..............................uacccuguagauccgGauuugu............................................................  1      1  Bl1
..............................uacccuguagaAccgaauuugu............................................................  4      1  Bl1
..............................uacccuguaCauccgaauuugu............................................................  1      1  Bl1
..............................uacccuguagauccgUauuugu............................................................  18     1  Bl1
..............................uacUcuguagauccgaauuugu............................................................  53     1  Bl1
..............................uacccuguGgauccgaauuugu............................................................  2      1  Bl1
```

```
                              Mature                      Star
ggcacgcgcuuuucuuaggugcuuuacaucuacccuguagauccgaauuuguCuaggauucuaaccacaaauucgguucuagagagguuuaugugaugcauacgaaacgacc
..............................uacccuguagauccgaauuuguA...........................................................  623   1  Bl1
..............................uacccuguagauccgaauuuguG...........................................................  31    1  Bl1
..............................uacccuguagauccgaauuuguc...........................................................  1     0  Bl1
..............................uacccuguagauccgaauuuguAu..........................................................  2     1  Bl1
...............................Ncccuguagauccgaauu...............................................................  1     1  Bl1
...............................acccuguagauccgaauu...............................................................  43    0  Bl1
...............................acccuguagCuccgaauu...............................................................  1     1  Bl1
...............................acccGguagauccgaauu...............................................................  2     1  Bl1
...............................acccuguagauccgaauuA..............................................................  1     1  Bl1
...............................acccuguagauccgaauuu..............................................................  84    0  Bl1
...............................acccuguagauccgaauuG..............................................................  1     1  Bl1
...............................acccCguagauccgaauuu..............................................................  1     1  Bl1
...............................Ccccuguagauccgaauuu..............................................................  2     1  Bl1
...............................acccGguagauccgaauuu..............................................................  3     1  Bl1
...............................acccGguagauccgaauuug.............................................................  19    1  Bl1
...............................acccuguaAauccgaauuug.............................................................  1     1  Bl1
...............................acccuguagauccgaCuuug.............................................................  1     1  Bl1
...............................acAcuguagauccgaauuug.............................................................  2     1  Bl1
...............................acccuguagauccgaaAuug.............................................................  1     1  Bl1
...............................acccuguagauccgaUuuug.............................................................  1     1  Bl1
...............................acUcuguagauccgaauuug.............................................................  1     1  Bl1
...............................acccuguagauAcgaauuug.............................................................  1     1  Bl1
...............................Ccccuguagauccgaauuug.............................................................  3     1  Bl1
...............................Ucccuguagauccgaauuug.............................................................  1     1  Bl1
...............................aAccuguagauccgaauuug.............................................................  1     1  Bl1
...............................aUccuguagauccgaauuug.............................................................  1     1  Bl1
...............................acccuguagauccgaauuug.............................................................  388   0  Bl1
...............................acccuguaCauccgaauuug.............................................................  1     1  Bl1
...............................acccuguagCuccgaauuug.............................................................  4     1  Bl1
...............................acccuguagUuccgaauuug.............................................................  1     1  Bl1
...............................acccuguCgauccgaauuug.............................................................  1     1  Bl1
...............................acccAguagauccgaauuug.............................................................  6     1  Bl1
...............................acccugCagauccgaauuug.............................................................  1     1  Bl1
...............................acccCguagauccgaauuug.............................................................  3     1  Bl1
...............................acccuguagauccgaauuuU.............................................................  1     1  Bl1
...............................acccuUuagauccgaauuugu............................................................  3     1  Bl1
...............................acccugGagauccgaauuugu............................................................  2     1  Bl1
...............................acccuguagauccgaauuuCu............................................................  1     1  Bl1
...............................acccuguagauccgaauuAgu............................................................  1     1  Bl1
...............................acccuCuagauccgaauuugu............................................................  1     1  Bl1
...............................acccuguagauccgaauuugC............................................................  50    1  Bl1
...............................acUcuguagauccgaauuugu............................................................  2     1  Bl1
...............................Ccccuguagauccgaauuugu............................................................  45    1  Bl1
...............................aAccuguagauccgaauuugu............................................................  2     1  Bl1
...............................acccuguagauccgaUuuugu............................................................  4     1  Bl1
...............................accAuguagauccgaauuugu............................................................  2     1  Bl1
...............................acccuguagUuccgaauuugu............................................................  4     1  Bl1
...............................acccuguagaCccgaauuugu............................................................  1     1  Bl1
...............................acccCguagauccgaauuugu............................................................  17    1  Bl1
...............................acGcuguagauccgaauuugu............................................................  6     1  Bl1
...............................acccuguaAauccgaauuugu............................................................  4     1  Bl1
...............................acccuguUgauccgaauuugu............................................................  3     1  Bl1
...............................acccuguagauGcgaauuugu............................................................  1     1  Bl1
...............................acccGguagauccgaauuugu............................................................  153   1  Bl1
...............................Ucccuguagauccgaauuugu............................................................  3     1  Bl1
...............................acccuguGgauccgaauuugu............................................................  1     1  Bl1
...............................acccuguagauccgaauuugu............................................................  3119  0  Bl1
...............................acccuguagauccgaGuuugu............................................................  1     1  Bl1
...............................acccuguagauUcgaauuugu............................................................  4     1  Bl1
...............................acccuguagaucAgaauuugu............................................................  2     1  Bl1
...............................acccuguagauccgaCuuugu............................................................  3     1  Bl1
...............................acccuguagauccAaauuugu............................................................  2     1  Bl1
...............................acccuguagCuccgaauuugu............................................................  18    1  Bl1
...............................acccuguagGuccgaauuugu............................................................  2     1  Bl1
...............................accUuguagauccgaauuugu............................................................  14    1  Bl1
...............................Gcccuguagauccgaauuugu............................................................  1     1  Bl1
...............................acccuguagauccgaauGugu............................................................  1     1  Bl1
...............................acccuguagauccgaauuGgu............................................................  3     1  Bl1
...............................acccuguaCauccgaauuugu............................................................  3     1  Bl1
...............................acccuguagauccgaauuuUu............................................................  16    1  Bl1
```

Mature **Star**

```
ggcacgcgcuuuucuuaggugcuuuacaucuacccuguagauccgaauuugucuaggauucuaaccacaaauucgguucuagagagguuuaugugaugcauacgaaacgacc
...............................acccuguagauccgaauuCgu............................................................ 2 1 Bl1
...............................acccugCagauccgaauuugu............................................................ 2 1 Bl1
...............................acccuguagauccgUauuugu............................................................ 2 1 Bl1
...............................acccuguagaAccgaauuugu............................................................ 1 1 Bl1
...............................acccuguagauccgGauuugu............................................................ 1 1 Bl1
...............................acAcuguagauccgaauuugu............................................................ 20 1 Bl1
...............................acccAguagauccgaauuugu............................................................ 26 1 Bl1
...............................acccuguagaucUgaauuugu............................................................ 3 1 Bl1
...............................acccuguagauccgaauuugA............................................................ 21 1 Bl1
...............................acccuAuagauccgaauuugu............................................................ 1 1 Bl1
...............................acccuguagauccgaauCugu............................................................ 1 1 Bl1
...............................acccGguagauccgaauuuguc........................................................... 1 1 Bl1
...............................acccuguagauccgaauuuguA........................................................... 87 1 Bl1
...............................acccCguagauccgaauuuguc........................................................... 1 1 Bl1
...............................acccuguagCuccgaauuuguc........................................................... 1 1 Bl1
...............................acccuguagauccgaauuuguc........................................................... 51 0 Bl1
...............................acccuguagauccgaauuuguG........................................................... 14 1 Bl1
...............................acccuguagauccgaauuuguAu.......................................................... 1 1 Bl1
................................cccuguagauccgaauuu.............................................................. 1 0 Bl1
................................cccuUuagauccgaauuug............................................................. 1 1 Bl1
................................ccUuguagauccgaauuug............................................................. 1 1 Bl1
................................cccuguagauccgaauuug............................................................. 10 0 Bl1
................................cccGguagauccgaauuugu............................................................ 1 1 Bl1
................................cccuUuagauccgaauuugu............................................................ 5 1 Bl1
................................cccuguagauccgaauuuUu............................................................ 3 1 Bl1
................................ccUuguagauccgaauuugu............................................................ 1 1 Bl1
................................cccuguagauccgaauuGgu............................................................ 2 1 Bl1
................................cccuguagauccgaauuugu............................................................ 58 0 Bl1
................................cccuguagauccgaUuuugu............................................................ 3 1 Bl1
................................cccuguagUuccgaauuugu............................................................ 1 1 Bl1
................................cccugAagauccgaauuugu............................................................ 2 1 Bl1
................................cccuguagauccgaauuuguc........................................................... 1 0 Bl1
................................cccuguagauccgaauuuguA........................................................... 3 1 Bl1
.................................ccugAagauccgaauuug............................................................. 1 1 Bl1
.................................ccugGagauccgaauuug............................................................. 1 1 Bl1
.................................ccuguagauccgaauuugu............................................................ 5 0 Bl1
.................................ccugGagauccgaauuugu............................................................ 1 1 Bl1
..................................cuguagauccgaauuugu............................................................ 1 0 Bl1
..................................cuguagauccgaauuugC............................................................ 1 1 Bl1
...................................................................caaaGucgguucuagaga........................... 1 1 Bl1
...................................................................caaauucgguucuagaga........................... 23 0 Bl1
...................................................................caaauucgguucuagagaA.......................... 1 1 Bl1
...................................................................caaauucgguucuagagag.......................... 6 0 Bl1
...................................................................Naaauucgguucuagagagg......................... 1 1 Bl1
...................................................................caaauucgguucuagagagg......................... 37 0 Bl1
...................................................................caaaGucgguucuagagagg......................... 2 1 Bl1
...................................................................caaauucgAuucuagagaggu........................ 1 1 Bl1
...................................................................Uaaauucgguucuagagaggu........................ 1 1 Bl1
...................................................................caaauucgguucuagagaggC........................ 1 1 Bl1
...................................................................caaauucgguucuagagaggu........................ 70 0 Bl1
...................................................................caaaGucgguucuagagaggu........................ 6 1 Bl1
...................................................................Uaaauucgguucuagagagguu....................... 2 1 Bl1
...................................................................caaauucgguucuagagagguu....................... 239 0 Bl1
...................................................................Gaaauucgguucuagagagguu....................... 1 1 Bl1
...................................................................caaaAucgguucuagagagguu....................... 1 1 Bl1
...................................................................caaauucgguucuagagaggCu....................... 1 1 Bl1
...................................................................caaaGucgguucuagagagguu....................... 16 1 Bl1
...................................................................cCaauucgguucuagagagguu....................... 3 1 Bl1
...................................................................caaauucgguucuagagagUuu....................... 1 1 Bl1
...................................................................Aaaauucgguucuagagagguu....................... 3 1 Bl1
...................................................................caaauucgguucuagagagguA....................... 2 1 Bl1
...................................................................caaauucgAuucuagagagguu....................... 1 1 Bl1
...................................................................caaauucgguucGagagagguu....................... 1 1 Bl1
...................................................................caaauucgguucuagagagguC....................... 8 1 Bl1
...................................................................cCaauucgguucuagagagguuu...................... 2 1 Bl1
...................................................................caaaCucgguucuagagagguuu...................... 5 1 Bl1
...................................................................caaaAucgguucuagagagguuu...................... 6 1 Bl1
...................................................................caaauucgguucuagagaUguuu...................... 1 1 Bl1
...................................................................cGaauucgguucuagagagguuu...................... 1 1 Bl1
...................................................................caaauucgguucuagagagguuu...................... 744 0 Bl1
```

Mature Star

ggcacgcgcuuuucuuaggugcuuuacaucuacccuguagauccgaauuugucuaggauucuaaccacaaauucgguucuagagagguuuaugugaugcauacgaaacgacc			
....caaauucgguuAuagagagguuu......................	1	1	Bl1
....caaauucgguucuagagagAuuu......................	1	1	Bl1
....caaaGucgguucuagagagguuu......................	24	1	Bl1
....caaauucgguucuagagaggGuu......................	1	1	Bl1
....caaauucgguucuagagagguCu......................	1	1	Bl1
....Uaaauucgguucuagagagguuu......................	5	1	Bl1
....caaauucgguucCagagagguuu......................	1	1	Bl1
....Aaaauucgguucuagagagguuu......................	12	1	Bl1
....caaauucgguucuagagUgguuu......................	1	1	Bl1
....caaauucgguucuagagagguuC......................	11	1	Bl1
....Naaauucgguucuagagagguuu......................	1	1	Bl1
....caaauucgguucuagagagUuuu......................	1	1	Bl1
....caaUuucgguucuagagagguuu......................	1	1	Bl1
....caaauucgAuucuagagagguuua.....................	1	1	Bl1
....caaaGucgguucuagagagguuua.....................	1	1	Bl1
....caaauucgguucuagagagguuuU.....................	10	1	Bl1
....caaauucgguucuagagagguuuUu....................	1	1	Bl1
....Caauucgguucuagagag..........................	1	1	Bl1
....aaauucgguucGagagag..........................	1	1	Bl1
....aaauucgguucuagagag..........................	39	0	Bl1
....aaauGcgguucuagagag..........................	1	1	Bl1
....aaauCcgguucuagagagg.........................	7	1	Bl1
....aaauucgguucuagagUgg.........................	1	1	Bl1
....aaauucgguucuagagagg.........................	243	0	Bl1
....aaauucgguucuagagagC.........................	1	1	Bl1
....aaauuUgguucuagagagg.........................	1	1	Bl1
....aaCuucgguucuagagagg.........................	2	1	Bl1
....aaauAcgguucuagagagg.........................	2	1	Bl1
....aaauGcgguucuagagagg.........................	8	1	Bl1
....aaauucgguucuagagagU.........................	3	1	Bl1
....aaaCucgguucuagagagg.........................	1	1	Bl1
....Caauucgguucuagagagg.........................	6	1	Bl1
....aaCuucgguucuagagaggu........................	1	1	Bl1
....aaauGcgguucuagagaggu........................	16	1	Bl1
....aaauucgguucuagagaggC........................	24	1	Bl1
....aCauucgguucuagagaggu........................	1	1	Bl1
....aaauucgguuAuagagaggu........................	1	1	Bl1
....aaauucgguucuagagagUu........................	3	1	Bl1
....aaauucgguucuagagaggA........................	1	1	Bl1
....aaauucUguucuagagaggu........................	3	1	Bl1
....aaauucgguucuagagUggu........................	1	1	Bl1
....aaauucgguucuagagaggu........................	347	0	Bl1
....Caauucgguucuagagaggu........................	1	1	Bl1
....aaauucgguucuagaUaggu........................	1	1	Bl1
....Gaauucgguucuagagaggu........................	1	1	Bl1
....aaaAucgguucuagagaggu........................	1	1	Bl1
....aaauCcgguucuagagaggu........................	1	1	Bl1
....Naauucgguucuagagagguu.......................	1	1	Bl1
....aCauucgguucuagagagguu.......................	2	1	Bl1
....Uaauucgguucuagagagguu.......................	6	1	Bl1
....aaauucgguucuagGgagguu.......................	1	1	Bl1
....aaauuUgguucuagagagguu.......................	1	1	Bl1
....aaauucgguucuUgagagguu.......................	1	1	Bl1
....aaauucgguucuagagagguA.......................	14	1	Bl1
....aaauGcgguucuagagagguu.......................	43	1	Bl1
....aaauucgguAcuagagagguu.......................	1	1	Bl1
....aaauucgguucuagagagguu.......................	1127	0	Bl1
....aaauucUguucuagagagguu.......................	1	1	Bl1
....aaauucgguucuagagagguC.......................	19	1	Bl1
....Gaauucgguucuagagagguu.......................	2	1	Bl1
....aaauucggCucuagagagguu.......................	1	1	Bl1
....aaauucgguucuagagGgguu.......................	1	1	Bl1
....aaauucgguucuagagagUuu.......................	1	1	Bl1
....aaauCcgguucuagagagguu.......................	7	1	Bl1
....aaauucgguucuagagCgguu.......................	2	1	Bl1
....aaauAcgguucuagagagguu.......................	4	1	Bl1
....aaauucgguuAuagagagguu.......................	1	1	Bl1
....aaCuucgguucuagagagguu.......................	5	1	Bl1
....aaauucgguucuagagagguG.......................	2	1	Bl1
....Caauucgguucuagagagguu.......................	11	1	Bl1

```
                              Mature                                Star
ggcacgcgcuuuucuuaggugcuuuacaucuacccuguagauccgaauuugucuaggauucuaaccacaaauucgguucuagagagguuuaugugaugcauacgaaacgacc
....................................................................aaaGucgguucuagagagguu.......................   9     1    Bl1
....................................................................aaauucgguucGagagagguu.......................   1     1    Bl1
....................................................................aGauucgguucuagagagguu.......................   1     1    Bl1
....................................................................aaauucgguucCagagagguuu......................   1     1    Bl1
....................................................................aaauucggAucuagagagguuu......................   1     1    Bl1
....................................................................aaauucgguuAuagagagguuu......................   1     1    Bl1
....................................................................aaauucgguucuagagaUguuu......................   1     1    Bl1
....................................................................aaCuucgguucuagagagguuu......................   3     1    Bl1
....................................................................Caauucgguucuagagagguuu......................   5     1    Bl1
....................................................................aaauucgguucuagagCgguuu......................   1     1    Bl1
....................................................................aaauucgguucuagagUgguuu......................   1     1    Bl1
....................................................................aaauucgguucuUgagagguuu......................   1     1    Bl1
....................................................................aaauuAgguucuagagagguuu......................   1     1    Bl1
....................................................................Uaauucgguucuagagagguuu......................   5     1    Bl1
....................................................................aaauucgguucuagCgagguuu......................   1     1    Bl1
....................................................................aaauCcgguucuagagagguuu......................   6     1    Bl1
....................................................................aaauAcgguucuagagagguuu......................   5     1    Bl1
....................................................................aaauucgguucuagagagguuu......................   582   0    Bl1
....................................................................aaaGucgguucuagagagguuu......................   4     1    Bl1
....................................................................aaauGcgguucuagagagguuu......................   13    1    Bl1
....................................................................aaauucgguucuagagagguuC......................   5     1    Bl1
....................................................................aaauucgguucuagagagguuG......................   4     1    Bl1
....................................................................aaauucgguucuagagagUuuu......................   2     1    Bl1
....................................................................aaauucgguucuagagagguuua.....................   4     0    Bl1
....................................................................aaauucgguucuagagagguuuU.....................   14    1    Bl1
....................................................................aaauucgguucuagagagguuCau....................   1     1    Bl1
.....................................................................aauucgguucuagagagguu.......................   2     0    Bl1
.....................................................................Cauucgguucuagagagguu.......................   1     1    Bl1
.....................................................................aauucgUuucuagagagguu.......................   1     1    Bl1
.....................................................................aauucgguucuagagagguuu......................   1     0    Bl1
......................................................................auucgguucuagagagguuu......................   1     0    Bl1

.............................cuacGcuguagauccgaauuugu............................................................   1     1    Ch1
..............................uacccCguagauccgaau................................................................   1     1    Ch1
..............................uacccuguagauccgaau................................................................   1     0    Ch1
..............................uacccuguagauccgaauA...............................................................   2     1    Ch1
..............................uacccuguagauccgaauu...............................................................   22    0    Ch1
..............................uacccuguagauccgaauuu..............................................................   66    0    Ch1
..............................uacccuguCgauccgaauuu..............................................................   2     1    Ch1
..............................uaccGuguagauccgaauuu..............................................................   1     1    Ch1
..............................uacccuguagauccgaauuG..............................................................   1     1    Ch1
..............................uaccUuguagauccgaauuu..............................................................   2     1    Ch1
..............................uacccuguagauccgaauuug.............................................................   523   0    Ch1
..............................uacAcuguagauccgaauuug.............................................................   1     1    Ch1
..............................uacccuguagauccgaauuuA.............................................................   3     1    Ch1
..............................uaccUuguagauccgaauuug.............................................................   3     1    Ch1
..............................uacccuguagaucUgaauuug.............................................................   1     1    Ch1
..............................uacccuguagauccgaauuuU.............................................................   1     1    Ch1
..............................uacUcuguagauccgaauuug.............................................................   1     1    Ch1
..............................uacccuguagCuccgaauuug.............................................................   2     1    Ch1
..............................Cacccuguagauccgaauuug.............................................................   1     1    Ch1
..............................uacccuguagauccgUauuug.............................................................   1     1    Ch1
..............................uacccuguagauUcgaauuug.............................................................   1     1    Ch1
..............................uaUccuguagauccgaauuug.............................................................   1     1    Ch1
..............................uaAccuguagauccgaauuug.............................................................   3     1    Ch1
..............................uacccuguagauAcgaauuug.............................................................   1     1    Ch1
..............................uaccGuguagauccgaauuug.............................................................   2     1    Ch1
..............................uaGccuguagauccgaauuug.............................................................   3     1    Ch1
..............................uacccuguCgauccgaauuug.............................................................   4     1    Ch1
..............................uacccuguagauccgaauuGg.............................................................   1     1    Ch1
..............................uCcccuguagauccgaauuug.............................................................   3     1    Ch1
..............................uacccuUuagauccgaauuug.............................................................   1     1    Ch1
..............................Aacccuguagauccgaauuug.............................................................   3     1    Ch1
..............................uacccuguagauccgGauuug.............................................................   1     1    Ch1
..............................uacccuguagauccgaauuGgu............................................................   3     1    Ch1
..............................uacccGguagauccgaauuugu............................................................   2     1    Ch1
..............................uacccuguaAauccgaauuugu............................................................   1     1    Ch1
..............................uacccuguagauccgCauuugu............................................................   1     1    Ch1
..............................uacccuguUgauccgaauuugu............................................................   1     1    Ch1
..............................uacccuguagauccgaaAuugu............................................................   2     1    Ch1
```

Mature **Star**

```
ggcacgcgcuuuucuuaggugcuuuacaucuacccuguagauccgaauuugucuaggauucuaaccacaaauucgguucuagagagguuuaugugaugcauacgaaacgacc
..............................uacccuguagUuccgaauuugu............................................................  1     1  Ch1
..............................uUcccuguagauccgaauuugu............................................................  2     1  Ch1
..............................uacccuguagauccgUauuugu............................................................  2     1  Ch1
..............................uacccuguagaGccgaauuugu............................................................  3     1  Ch1
..............................uacccuguagauccgaauuugu............................................................  4116  0  Ch1
..............................uacccuguagauccgaauGugu............................................................  3     1  Ch1
..............................uacccuguagaucAgaauuugu............................................................  3     1  Ch1
..............................uacAcuguagauccgaauuugu............................................................  3     1  Ch1
..............................uacccuguagauccgaauuuCu............................................................  1     1  Ch1
..............................uacccuguagauccgaauuuAu............................................................  1     1  Ch1
..............................uaccUuguagauccgaauuugu............................................................  31    1  Ch1
..............................uacccuguagauccCaauuugu............................................................  1     1  Ch1
..............................uaccGuguagauccgaauuugu............................................................  39    1  Ch1
..............................uacccuguagauccUaauuugu............................................................  8     1  Ch1
..............................uaGccuguagauccgaauuugu............................................................  8     1  Ch1
..............................uacUcuguagauccgaauuugu............................................................  16    1  Ch1
..............................uacccuguagauccgaCuuugu............................................................  6     1  Ch1
..............................Gacccuguagauccgaauuugu............................................................  2     1  Ch1
..............................uacccuAuagauccgaauuugu............................................................  2     1  Ch1
..............................uacccuguagauccgaaCuugu............................................................  3     1  Ch1
..............................uacccuguagauccgaauCugu............................................................  1     1  Ch1
..............................uacccuguagauccgaauuugA............................................................  39    1  Ch1
..............................uacccuguagauccgaauuugC............................................................  96    1  Ch1
..............................uacccuguagGuccgaauuugu............................................................  1     1  Ch1
..............................uacccugAagauccgaauuugu............................................................  1     1  Ch1
..............................uacccuguGgauccgaauuugu............................................................  1     1  Ch1
..............................uacccuguCgauccgaauuugu............................................................  32    1  Ch1
..............................Aacccuguagauccgaauuugu............................................................  4     1  Ch1
..............................uacccuguagCuccgaauuugu............................................................  12    1  Ch1
..............................uacccuguagaCccgaauuugu............................................................  1     1  Ch1
..............................uacccuguagauccAaauuugu............................................................  1     1  Ch1
..............................uacccuguagauccgaauuuUu............................................................  2     1  Ch1
..............................uacccuguagaucUgaauuugu............................................................  4     1  Ch1
..............................uaAccuguagauccgaauuugu............................................................  27    1  Ch1
..............................uaUccuguagauccgaauuugu............................................................  2     1  Ch1
..............................uacccCguagauccgaauuugu............................................................  2     1  Ch1
..............................uacccuguagauUcgaauuugu............................................................  6     1  Ch1
..............................uacccuguagaAccgaauuugu............................................................  1     1  Ch1
..............................uCcccuguagauccgaauuugu............................................................  7     1  Ch1
..............................uacccuUuagauccgaauuugu............................................................  1     1  Ch1
..............................uacccuguagauAcgaauuugu............................................................  3     1  Ch1
..............................Nacccuguagauccgaauuugu............................................................  3     1  Ch1
..............................Cacccuguagauccgaauuugu............................................................  7     1  Ch1
..............................uacccuguagauccgaauuuguG...........................................................  34    1  Ch1
..............................uacccuguagauccgaauuuguA...........................................................  141   1  Ch1
..............................uacccuguagauccgaauuuguc...........................................................  6     0  Ch1
..............................uacccuguagauccgaauuuguGu..........................................................  14    1  Ch1
..............................uacccuguagauccgaauuuguAu..........................................................  1     1  Ch1
...............................acccuguagauccgaauu...............................................................  6     0  Ch1
...............................acccGguagauccgaauuu..............................................................  1     1  Ch1
...............................acccuguagauccgaauuu..............................................................  17    0  Ch1
...............................acccGguagauccgaauuug.............................................................  11    1  Ch1
...............................acccuguagauccgaauuug.............................................................  182   0  Ch1
...............................accAuguagauccgaauuug.............................................................  1     1  Ch1
...............................acccuguagauccgaauuuA.............................................................  2     1  Ch1
...............................accUuguagauccgaauuug.............................................................  1     1  Ch1
...............................acccAguagauccgaauuug.............................................................  2     1  Ch1
...............................acccuguagauccgCauuug.............................................................  1     1  Ch1
...............................Ucccuguagauccgaauuug.............................................................  1     1  Ch1
...............................acccuguagauccgaauuuU.............................................................  1     1  Ch1
...............................acccuguagCuccgaauuug.............................................................  1     1  Ch1
...............................Ccccuguagauccgaauuug.............................................................  3     1  Ch1
...............................acccuguagaAccgaauuug.............................................................  1     1  Ch1
...............................Ucccuguagauccgaauuugu............................................................  6     1  Ch1
...............................acccuguagCuccgaauuugu............................................................  11    1  Ch1
...............................acccuUuagauccgaauuugu............................................................  2     1  Ch1
...............................acUcuguagauccgaauuugu............................................................  1     1  Ch1
...............................acccuguagauccgaauuugA............................................................  3     1  Ch1
...............................acccuguagauccgGauuugu............................................................  2     1  Ch1
...............................acccuguagauccgaauuuAu............................................................  1     1  Ch1
```

Mature **Star**

ggcacgcgcuuuucuuaggugcuuuacaucuacccuguagauccgaauuugucuaggauucuaaccacaaauucgguucuagagagguuuaugugaugcauacgaaacgacc

...............................acccuguagauccgaauuugC..	66	1	Ch1
...............................acccAguagauccgaauuugu..	11	1	Ch1
...............................accGuguagauccgaauuugu..	1	1	Ch1
...............................acccuguagauccgUauuugu..	1	1	Ch1
...............................acccuguaAauccgaauuugu..	5	1	Ch1
...............................Ccccuguagauccgaauuugu..	21	1	Ch1
...............................acccuguaUauccgaauuugu..	2	1	Ch1
...............................acccuguagauccgaauAugu..	1	1	Ch1
...............................acccuguagauccgaauuuUu..	1	1	Ch1
...............................acccuguagauccgaauuugu..	1779	0	Ch1
...............................acccugGagauccgaauuugu..	1	1	Ch1
...............................acccuguagauccgaauuGgu..	1	1	Ch1
...............................Ncccuguagauccgaauuugu..	1	1	Ch1
...............................acccuguagauccgaauGugu..	2	1	Ch1
...............................acccuguagauUcgaauuugu..	1	1	Ch1
...............................acccuguagauccgaauuCgu..	1	1	Ch1
...............................acGcuguagauccgaauuugu..	1	1	Ch1
...............................acccuguagauccAaauuugu..	1	1	Ch1
...............................aUccuguagauccgaauuugu..	1	1	Ch1
...............................acccuguagauccUaauuugu..	1	1	Ch1
...............................acccuguagaAccgaauuugu..	3	1	Ch1
...............................aGccuguagauccgaauuugu..	1	1	Ch1
...............................accAuguagauccgaauuugu..	1	1	Ch1
...............................accUuguagauccgaauuugu..	7	1	Ch1
...............................acccGguagauccgaauuugu..	72	1	Ch1
...............................aAccuguagauccgaauuugu..	2	1	Ch1
...............................Gcccuguagauccgaauuugu..	1	1	Ch1
...............................acccCguagauccgaauuugu..	10	1	Ch1
...............................acAcuguagauccgaauuugu..	5	1	Ch1
...............................acccuguagaCccgaauuugu..	1	1	Ch1
...............................acccuguagGuccgaauuugu..	1	1	Ch1
...............................acccuguagauccgaUuuugu..	3	1	Ch1
...............................acccuguagauccgaauCugu..	1	1	Ch1
...............................acccuguagauccgaauuuguA...	41	1	Ch1
...............................acccuguagauccgaauuuguc...	54	0	Ch1
...............................acccCguagauccgaauuuguc...	1	1	Ch1
...............................acccuguagauccgaauuuguG...	24	1	Ch1
...............................accUuguagauccgaauuuguc...	1	1	Ch1
...............................Ccccuguagauccgaauuuguc...	1	1	Ch1
...............................acccuguagauccgaauuuguGu..	4	1	Ch1
...............................acccuguagauccgaauuugucu..	2	0	Ch1
...............................acccuguagauccgaauuugucG..	3	1	Ch1
...............................acccuguagauccgaauuugucAa...	2	1	Ch1
...............................acccuguagauccgaauuuguUua...	1	1	Ch1
................................cccuguagauccgaauuug...	3	0	Ch1
................................cccuguagauccgaauuugA..	1	1	Ch1
................................cccGguagauccgaauuugu..	1	1	Ch1
................................cccuguagaGccgaauuugu..	1	1	Ch1
................................cccuguagauccgaauuugu..	23	0	Ch1
................................cccuguagauccgaauuuUu..	1	1	Ch1
................................cccuguagUuccgaauuugu..	1	1	Ch1
................................cccuUuagauccgaauuugu..	4	1	Ch1
................................cccuguagauccgaauuugC..	2	1	Ch1
................................cccuguagauccgaauuuguA...	1	1	Ch1
................................cccuguagauccgaauuuguGu..	1	1	Ch1
.................................ccuguagauccgaauuugu..	2	0	Ch1
.................................ccuguagauccgaauuuguc...	1	0	Ch1
....caaauucgguucuagaga...........................	1	0	Ch1
....caaauucgguucuagagagg.........................	4	0	Ch1
....caaauucgguucuagagaggu........................	2	0	Ch1
....caaaGucgguucuagagaggu........................	1	1	Ch1
....caaaGucgguucuagagagguu.......................	2	1	Ch1
....caaauucgguucuagagagguu.......................	28	0	Ch1
....caaauucgguucuagagagAuuu......................	1	1	Ch1
....caaauucgguucuagagagguuu......................	46	0	Ch1
....caCauucgguucuagagagguuu......................	1	1	Ch1
....caaaGucgguucuagagagguuu......................	2	1	Ch1
....Uaaauucgguucuagagagguuu......................	1	1	Ch1
....caaauucgguucuagagaUguuu......................	1	1	Ch1
....caaauucgguucuagagaggAuu......................	1	1	Ch1

Mature **Star**

```
ggcacgcgcuuuucuuaggugcuuuacaucuacccuguagauccgaauuugucuaggauucuaaccacaaauucgguucuagagagguuuaugugaugcauacgaaacgacc
...................................................................caaauucgguucuagagagguuuU.....................   2    1   Ch1
....................................................................aaauucgguucuagagag..........................   3    0   Ch1
....................................................................aaauucgguucuagagagA.........................   3    1   Ch1
....................................................................aaaGucgguucuagagagg.........................   1    1   Ch1
....................................................................Uaauucgguucuagagagg.........................   1    1   Ch1
....................................................................aaauucgguucuagagagg.........................   35   0   Ch1
....................................................................aaauucgguucuagagaggA........................   4    1   Ch1
....................................................................aaauucgguucuagagagUu........................   1    1   Ch1
....................................................................aaauCcgguucuagagaggu........................   1    1   Ch1
....................................................................aaauGcgguucuagagaggu........................   3    1   Ch1
....................................................................aaauucgguucuagagaggC........................   7    1   Ch1
....................................................................aaauAcgguucuagagaggu........................   1    1   Ch1
....................................................................aaaGucgguucuagagaggu........................   2    1   Ch1
....................................................................aaauucgguucuagagaggu........................   71   0   Ch1
....................................................................Uaauucgguucuagagaggu........................   1    1   Ch1
....................................................................aaauucgguucuagagaggG........................   1    1   Ch1
....................................................................aaauucgguucuCgagaggu........................   1    1   Ch1
....................................................................aaaAucgguucuagagagguu.......................   2    1   Ch1
....................................................................aaauucgguucuagagagguC.......................   32   1   Ch1
....................................................................aaauAcgguucuagagagguu.......................   3    1   Ch1
....................................................................aaauucgguucuagagagguG.......................   1    1   Ch1
....................................................................aaauucgguucuCgagagguu.......................   1    1   Ch1
....................................................................aaauucgguucuagagaUguu.......................   1    1   Ch1
....................................................................aaauucgguucuagagagguA.......................   13   1   Ch1
....................................................................aaCuucgguucuagagagguu.......................   2    1   Ch1
....................................................................aaauCcgguucuagagagguu.......................   3    1   Ch1
....................................................................aaauucgguucuagagCgguu.......................   3    1   Ch1
....................................................................aaauGcgguucuagagagguu.......................   18   1   Ch1
....................................................................Caauucgguucuagagagguu.......................   8    1   Ch1
....................................................................Gaauucgguucuagagagguu.......................   2    1   Ch1
....................................................................aaauucgguucuagagagguu.......................   686  0   Ch1
....................................................................aaauucgguucuagCgagguu.......................   1    1   Ch1
....................................................................aaauucggCucuagagagguu.......................   1    1   Ch1
....................................................................Uaauucgguucuagagagguu.......................   2    1   Ch1
....................................................................aaaGucgguucuagagagguu.......................   6    1   Ch1
....................................................................aaauucgguucuagagagUuu.......................   1    1   Ch1
....................................................................aaauGcgguucuagagagguuu......................   4    1   Ch1
....................................................................aaauucgguucuagagagguuu......................   101  0   Ch1
....................................................................aaauucgguucuagagagguuG......................   2    1   Ch1
....................................................................aaauCcgguucuagagagguuu......................   2    1   Ch1
....................................................................aUauucgguucuagagagguuu......................   1    1   Ch1
....................................................................Caauucgguucuagagagguuu......................   3    1   Ch1
....................................................................aaauucgguucuagagagguuC......................   3    1   Ch1
....................................................................aaauucUguucuagagagguuu......................   1    1   Ch1
.....................................................................aauucgguucuagagaggu........................   1    0   Ch1
.....................................................................aauucgguucuagagaggG........................   1    1   Ch1
.....................................................................aauucgguucuagagagguu.......................   1    0   Ch1
......................................................................auucgguucuagagagguuu......................   1    0   Ch1

.............................cuacGcuguagauccgaauuugu............................................................   2    1   Ta1
.............................cuacccuguagauccgaauuuUu............................................................   1    1   Ta1
.............................cuacccuguagauccgaauuugu............................................................   6    0   Ta1
.............................Auacccuguagauccgaauuugu............................................................   1    1   Ta1
..............................uacccuguagauccgaau................................................................   7    0   Ta1
..............................uacccuUuagauccgaauu...............................................................   1    1   Ta1
..............................uacccuguagauccgaauA...............................................................   8    1   Ta1
..............................uacccuguagauccgUauu...............................................................   1    1   Ta1
..............................uaccGuguagauccgaauu...............................................................   2    1   Ta1
..............................uacccuguagauccgaauu...............................................................   80   0   Ta1
..............................uacccuguagaucAgaauu...............................................................   1    1   Ta1
..............................uaccUuguagauccgaauu...............................................................   1    1   Ta1
..............................uaAccuguagauccgaauu...............................................................   3    1   Ta1
..............................uacccuguagauccgaCuuu..............................................................   1    1   Ta1
..............................uacccuguagCuccgaauuu..............................................................   1    1   Ta1
..............................uacccuguagauccgaauuu..............................................................   318  0   Ta1
..............................uacccuguagauccgaaCuu..............................................................   1    1   Ta1
..............................uaAccuguagauccgaauuu..............................................................   2    1   Ta1
..............................uaccGuguagauccgaauuu..............................................................   2    1   Ta1
..............................uacccuguCgauccgaauuu..............................................................   2    1   Ta1
..............................uaccUuguagauccgaauuu..............................................................   3    1   Ta1
```

```
                              Mature                                Star
ggcacgcgcuuuucuuaggugcuuuacaucuacccuguagauccgaauuugucuaggauucuaaccacaaauucgguucuagagagguuuaugugaugcauacgaaacgacc
..............................uacccuguagauccgaauGu..............................................................   1     1   Ta1
..............................uacccuguagauAcgaauuu..............................................................   1     1   Ta1
..............................uacccuguagauUcgaauuug.............................................................   2     1   Ta1
..............................uacccuguagauAcgaauuug.............................................................   1     1   Ta1
..............................uCcccuguagauccgaauuug.............................................................   8     1   Ta1
..............................uaccGuguagauccgaauuug.............................................................   18    1   Ta1
..............................Nacccuguagauccgaauuug.............................................................   2     1   Ta1
..............................uacccuguagauccUaauuug.............................................................   3     1   Ta1
..............................uacccuguagaCccgaauuug.............................................................   1     1   Ta1
..............................uacccuguGgauccgaauuug.............................................................   2     1   Ta1
..............................uacccuguagauccgaaGuug.............................................................   1     1   Ta1
..............................uaccUuguagauccgaauuug.............................................................   43    1   Ta1
..............................uacUcuguagauccgaauuug.............................................................   11    1   Ta1
..............................uacccuguaUauccgaauuug.............................................................   1     1   Ta1
..............................uGcccuguagauccgaauuug.............................................................   1     1   Ta1
..............................uaAccuguagauccgaauuug.............................................................   22    1   Ta1
..............................uacccuguCgauccgaauuug.............................................................   24    1   Ta1
..............................uacccuguagauccgaauuuA.............................................................   7     1   Ta1
..............................uacccuguagauccgaauuug.............................................................   2708  0   Ta1
..............................uacccuguagauccgaauGug.............................................................   4     1   Ta1
..............................uacccuguagauccgaauAug.............................................................   1     1   Ta1
..............................uacccuguagCuccgaauuug.............................................................   8     1   Ta1
..............................uacccuguagaucAgaauuug.............................................................   2     1   Ta1
..............................uacccuguagaucUgaauuug.............................................................   1     1   Ta1
..............................Cacccuguagauccgaauuug.............................................................   2     1   Ta1
..............................Aacccuguagauccgaauuug.............................................................   9     1   Ta1
..............................uacccuguagauccgCauuug.............................................................   1     1   Ta1
..............................uacccuguagauccAaauuug.............................................................   1     1   Ta1
..............................uacccuguagUuccgaauuug.............................................................   1     1   Ta1
..............................uacccugGagauccgaauuug.............................................................   2     1   Ta1
..............................uacccugCagauccgaauuug.............................................................   1     1   Ta1
..............................uacccuUuagauccgaauuug.............................................................   3     1   Ta1
..............................uacccuguagauccgaCuuug.............................................................   4     1   Ta1
..............................uacccuguagauccgaauCug.............................................................   1     1   Ta1
..............................uacccuguUgauccgaauuug.............................................................   2     1   Ta1
..............................uaUccuguagauccgaauuug.............................................................   3     1   Ta1
..............................uacccuguagauccgUauuug.............................................................   4     1   Ta1
..............................uaUccuguagauccgaauuugu............................................................   24    1   Ta1
..............................uacccuguagaCccgaauuugu............................................................   2     1   Ta1
..............................uacccuguagauccgaauuGgu............................................................   17    1   Ta1
..............................uacccuguagauccgaCuuugu............................................................   26    1   Ta1
..............................uacccuguagauccgaauuuCu............................................................   2     1   Ta1
..............................Aacccuguagauccgaauuugu............................................................   25    1   Ta1
..............................uacccuguagauccgaauuugC............................................................   40    1   Ta1
..............................Nacccuguagauccgaauuugu............................................................   16    1   Ta1
..............................uacccuguagauccgaUuuugu............................................................   4     1   Ta1
..............................uGcccuguagauccgaauuugu............................................................   3     1   Ta1
..............................uaccGuguagauccgaauuugu............................................................   125   1   Ta1
..............................uacccuguagaucGgaauuugu............................................................   1     1   Ta1
..............................uacccuguCgauccgaauuugu............................................................   113   1   Ta1
..............................uacccuguagauccCaauuugu............................................................   1     1   Ta1
..............................uacccuguagauccgaauAugu............................................................   1     1   Ta1
..............................uacccuguagauccgaauuuUu............................................................   6     1   Ta1
..............................uacccuguagauccgaaAuugu............................................................   16    1   Ta1
..............................uacGcuguagauccgaauuugu............................................................   1     1   Ta1
..............................uacccuguagauccgaauuAgu............................................................   1     1   Ta1
..............................uacccuguagauccgaGuuugu............................................................   7     1   Ta1
..............................uacccuguGgauccgaauuugu............................................................   8     1   Ta1
..............................uacccuguagauGcgaauuugu............................................................   1     1   Ta1
..............................uCcccuguagauccgaauuugu............................................................   49    1   Ta1
..............................uacccuguagauUcgaauuugu............................................................   5     1   Ta1
..............................uacccuguUgauccgaauuugu............................................................   7     1   Ta1
..............................Cacccuguagauccgaauuugu............................................................   24    1   Ta1
..............................uacccuguagaucAgaauuugu............................................................   12    1   Ta1
..............................uacccCguagauccgaauuugu............................................................   4     1   Ta1
..............................uacccuguagauccgCauuugu............................................................   4     1   Ta1
..............................uacccuguagauccgaauGugu............................................................   5     1   Ta1
..............................uacAcuguagauccgaauuugu............................................................   4     1   Ta1
..............................uacUcuguagauccgaauuugu............................................................   55    1   Ta1
..............................uacccuguagauccgaauuugA............................................................   201   1   Ta1
```

```
                              Mature                    Star
ggcacgcgcuuuucuuaggugcuuuacaucuacccuguagauccgaauuugucuaggauucuaaccacaaauucgguucuagagagguuuaugugaugcauacgaaacgacc
..............................uacccuAuagauccgaauuugu............................................................  1      1  Ta1
..............................uacccuguagaucUgaauuugu............................................................  11     1  Ta1
..............................uUcccuguagauccgaauuugu............................................................  3      1  Ta1
..............................uaAccuguagauccgaauuugu............................................................  120    1  Ta1
..............................uaGccuguagauccgaauuugu............................................................  22     1  Ta1
..............................uaccAuguagauccgaauuugu............................................................  3      1  Ta1
..............................uacccAguagauccgaauuugu............................................................  2      1  Ta1
..............................Gacccuguagauccgaauuugu............................................................  2      1  Ta1
..............................uacccuguaAauccgaauuugu............................................................  10     1  Ta1
..............................uacccuguagauccgaaGuugu............................................................  3      1  Ta1
..............................uacccuguagCuccgaauuugu............................................................  47     1  Ta1
..............................uacccuguagauccgUauuugu............................................................  14     1  Ta1
..............................uacccuUuagauccgaauuugu............................................................  11     1  Ta1
..............................uacccuguagauccgaaCuugu............................................................  6      1  Ta1
..............................uacccuguagaGccgaauuugu............................................................  10     1  Ta1
..............................uacccuguaCauccgaauuugu............................................................  1      1  Ta1
..............................uacccuguagauAcgaauuugu............................................................  17     1  Ta1
..............................uacccuguagGuccgaauuugu............................................................  3      1  Ta1
..............................uacccuguagauccgaauuugu............................................................  16632  0  Ta1
..............................uacccuguaUauccgaauuugu............................................................  4      1  Ta1
..............................uacccGguagauccgaauuugu............................................................  5      1  Ta1
..............................uacccuguagauccgaauuCgu............................................................  4      1  Ta1
..............................uacccuguagauccAaauuugu............................................................  4      1  Ta1
..............................uacccuguagauccgaauuuAu............................................................  3      1  Ta1
..............................uacccuguagauccUaauuugu............................................................  22     1  Ta1
..............................uaccUuguagauccgaauuugu............................................................  204    1  Ta1
..............................uacccuguagUuccgaauuugu............................................................  5      1  Ta1
..............................uacccuguagaAccgaauuugu............................................................  2      1  Ta1
..............................uacccuguagauccgaauuuguA...........................................................  285    1  Ta1
..............................uacccuguagCuccgaauuuguc...........................................................  1      1  Ta1
..............................uacccuguagauccgaauuuguG...........................................................  36     1  Ta1
..............................uacccuguagauccgaauuuguGu..........................................................  12     1  Ta1
..............................uacccuUuagauccgaauuugucu..........................................................  1      1  Ta1
..............................uacccuguagauccgaauuugucu..........................................................  5      0  Ta1
...............................Ccccuguagauccgaauu...............................................................  1      1  Ta1
...............................acccGguagauccgaauu...............................................................  2      1  Ta1
...............................acccuguagauccgaauu...............................................................  10     0  Ta1
...............................acccuguagauccgaauuG..............................................................  1      1  Ta1
...............................acccGguagauccgaauuu..............................................................  4      1  Ta1
...............................Ccccuguagauccgaauuu..............................................................  3      1  Ta1
...............................acccuguagauccgaauuu..............................................................  52     0  Ta1
...............................Gcccuguagauccgaauuug.............................................................  1      1  Ta1
...............................acccCguagauccgaauuug.............................................................  4      1  Ta1
...............................acccuguagauccgaauuug.............................................................  443    0  Ta1
...............................acAcuguagauccgaauuug.............................................................  4      1  Ta1
...............................Ucccuguagauccgaauuug.............................................................  2      1  Ta1
...............................acccuguaUauccgaauuug.............................................................  1      1  Ta1
...............................acccuguagCuccgaauuug.............................................................  3      1  Ta1
...............................accUuguagauccgaauuug.............................................................  3      1  Ta1
...............................Ccccuguagauccgaauuug.............................................................  8      1  Ta1
...............................acccuguagauccgaauuuU.............................................................  2      1  Ta1
...............................acccAguagauccgaauuug.............................................................  6      1  Ta1
...............................acccGguagauccgaauuug.............................................................  21     1  Ta1
...............................acccuguagauccgUauuugu............................................................  2      1  Ta1
...............................acccuguagauccgaUuuugu............................................................  2      1  Ta1
...............................acccugCagauccgaauuugu............................................................  1      1  Ta1
...............................acccuguagauccgaaGuugu............................................................  1      1  Ta1
...............................acccuguagauccgaauGugu............................................................  1      1  Ta1
...............................acccAguagauccgaauuugu............................................................  25     1  Ta1
...............................acccuguagCuccgaauuugu............................................................  23     1  Ta1
...............................acccuguagauccgaauuuAu............................................................  1      1  Ta1
...............................acccuguagauccgaauuugC............................................................  15     1  Ta1
...............................Ncccuguagauccgaauuugu............................................................  3      1  Ta1
...............................acccuguagauccgaauuuUu............................................................  11     1  Ta1
...............................acccuguagauccgaCuuugu............................................................  1      1  Ta1
...............................acccuguUgauccgaauuugu............................................................  1      1  Ta1
...............................acccuguagauccgaauAugu............................................................  4      1  Ta1
...............................acccuguCgauccgaauuugu............................................................  1      1  Ta1
...............................acccuCuagauccgaauuugu............................................................  1      1  Ta1
...............................acccugGagauccgaauuugu............................................................  1      1  Ta1
```

```
                               Mature                Star
ggcacgcgcuuuucuuaggugcuuuacaucuacccuguagauccgaauuugucuaggauucuaaccacaaauucgguucuagagagguuuaugugaugcauacgaaacgacc
...............................Ccccuguagauccgaauuugu............................................................ 51     1     Ta1
...............................acccuguagauccAaauuugu............................................................ 1      1     Ta1
...............................accUuguagauccgaauuugu............................................................ 18     1     Ta1
...............................acUcuguagauccgaauuugu............................................................ 10     1     Ta1
...............................accAuguagauccgaauuugu............................................................ 2      1     Ta1
...............................acccuguaAauccgaauuugu............................................................ 4      1     Ta1
...............................acccuguagaAccgaauuugu............................................................ 6      1     Ta1
...............................acccuguaCauccgaauuugu............................................................ 5      1     Ta1
...............................Ucccuguagauccgaauuugu............................................................ 7      1     Ta1
...............................acccuUuagauccgaauuugu............................................................ 3      1     Ta1
...............................acccuguagauUcgaauuugu............................................................ 3      1     Ta1
...............................acccuguagauccUaauuugu............................................................ 1      1     Ta1
...............................aUccuguagauccgaauuugu............................................................ 2      1     Ta1
...............................acccCguagauccgaauuugu............................................................ 35     1     Ta1
...............................acccuguagauccgCauuugu............................................................ 2      1     Ta1
...............................acAcuguagauccgaauuugu............................................................ 19     1     Ta1
...............................acccuguagaucAgaauuugu............................................................ 2      1     Ta1
...............................Gcccuguagauccgaauuugu............................................................ 3      1     Ta1
...............................acGcuguagauccgaauuugu............................................................ 5      1     Ta1
...............................acccGguagauccgaauuugu............................................................ 174    1     Ta1
...............................aAccuguagauccgaauuugu............................................................ 6      1     Ta1
...............................acccuguagUuccgaauuugu............................................................ 3      1     Ta1
...............................acccuguagauccgaauuugA............................................................ 40     1     Ta1
...............................acccuguagauccgaauuugu............................................................ 3891   0     Ta1
...............................acccuguagaucUgaauuugu............................................................ 2      1     Ta1
...............................acccuguaUauccgaauuugu............................................................ 2      1     Ta1
...............................acccuguagauccgaauuGgu............................................................ 1      1     Ta1
...............................acccCguagauccgaauuuguc........................................................... 3      1     Ta1
...............................acccuguagauccgaUuuuguc........................................................... 1      1     Ta1
...............................acccuguagauccgaauuuguG........................................................... 29     1     Ta1
...............................acccuguagauccgaauuuguc........................................................... 232    0     Ta1
...............................Ccccuguagauccgaauuuguc........................................................... 2      1     Ta1
...............................acccuguUgauccgaauuuguc........................................................... 1      1     Ta1
...............................acccuguagCuccgaauuuguc........................................................... 1      1     Ta1
...............................acGcuguagauccgaauuuguc........................................................... 1      1     Ta1
...............................acccGguagauccgaauuuguc........................................................... 7      1     Ta1
...............................acccuguagauccgaauuuguA........................................................... 16     1     Ta1
...............................acAcuguagauccgaauuuguc........................................................... 1      1     Ta1
...............................aAccuguagauccgaauuuguc........................................................... 1      1     Ta1
...............................acccuguaCauccgaauuuguc........................................................... 1      1     Ta1
...............................acccuguagGuccgaauuuguc........................................................... 1      1     Ta1
...............................acccuUuagauccgaauuuguc........................................................... 1      1     Ta1
...............................acccGguagauccgaauuugucu.......................................................... 1      1     Ta1
...............................acccuguagauccgaauuugucu.......................................................... 9      0     Ta1
...............................acccuguagauccgaauuuguGu.......................................................... 5      1     Ta1
...............................acccuguagauccgaauuugucG.......................................................... 24     1     Ta1
................................cccuguagauccgaauu............................................................... 2      0     Ta1
................................cccuguagauccgaauug.............................................................. 9      0     Ta1
................................cccuUuagauccgaauuugu............................................................ 7      1     Ta1
................................cccuguagauccgaauuuUu............................................................ 6      1     Ta1
................................cccuguagCuccgaauuugu............................................................ 1      1     Ta1
................................cccuguagauccgaUuuugu............................................................ 1      1     Ta1
................................cccugAagauccgaauuugu............................................................ 1      1     Ta1
................................cccuguagauccgaauuugu............................................................ 70     0     Ta1
................................cccuguagauccgaauuGgu............................................................ 1      1     Ta1
................................cccGguagauccgaauuuguc........................................................... 1      1     Ta1
................................cccuguagauccgaauuuguc........................................................... 11     0     Ta1
................................cccuguagauccgaauuuUuc........................................................... 2      1     Ta1
................................cccuUuagauccgaauuuguc........................................................... 1      1     Ta1
.................................ccuguagauccgaauuugu............................................................ 14     0     Ta1
.................................ccugGagauccgaauuugu............................................................ 1      1     Ta1
.................................ccuguagauccgaauuuguc........................................................... 2      0     Ta1
..................................................................Ccaaauucgguucuagagagg......................... 1      1     Ta1
...................................................................caaauucgguucuagaga........................... 10     0     Ta1
...................................................................caCauucgguucuagaga........................... 1      1     Ta1
...................................................................caaauucgguucuagagag.......................... 3      0     Ta1
...................................................................caaauucgguucuagagaA.......................... 3      1     Ta1
...................................................................caaaGucgguucuagagag.......................... 1      1     Ta1
...................................................................caaauucgguucuagagagg......................... 21     0     Ta1
...................................................................Aaaauucgguucuagagagg......................... 1      1     Ta1
```

```
                                 Mature                                    Star
ggcacgcgcuuuucuuaggugcuuuacaucuacccuguagauccgaauuugucuaggauucuaaccacaaauucgguucuagagagguuuaugugaugcauacgaaacgacc
...................................................................cUaauucgguucuagagagg.........................  1     1  Ta1
...................................................................caaaGucgguucuagagagg.........................  1     1  Ta1
...................................................................Uaaauucgguucuagagaggu........................  1     1  Ta1
...................................................................caaauucgguucuagagaggu........................  10    0  Ta1
...................................................................caaaAucgguucuagagaggu........................  1     1  Ta1
...................................................................caaaGucgguucuagagaggu........................  1     1  Ta1
...................................................................Aaaauucgguucuagagagguu.......................  1     1  Ta1
...................................................................caaauucgguucuagagagguu.......................  90    0  Ta1
...................................................................Gaaauucgguucuagagagguu.......................  1     1  Ta1
...................................................................caUauucgguucuagagagguu.......................  1     1  Ta1
...................................................................caaaAucgguucuagagagguu.......................  1     1  Ta1
...................................................................caaUuucgguucuagagagguu.......................  1     1  Ta1
...................................................................Uaaauucgguucuagagagguu.......................  6     1  Ta1
...................................................................Naaauucgguucuagagagguu.......................  1     1  Ta1
...................................................................caaauucgguucuagagaggAuu......................  1     1  Ta1
...................................................................caaaAucgguucuagagagguuu......................  14    1  Ta1
...................................................................caaauucgguucCagagagguuu......................  1     1  Ta1
...................................................................caaUuucgguucuagagagguuu......................  9     1  Ta1
...................................................................caaauuUgguucuagagagguuu......................  2     1  Ta1
...................................................................Uaaauucgguucuagagagguuu......................  3     1  Ta1
...................................................................caaauucgguucuaUagagguuu......................  2     1  Ta1
...................................................................caaauucgguucuagagagguuu......................  1114  0  Ta1
...................................................................caaauucgguucuagagaUguuu......................  1     1  Ta1
...................................................................caaaGucgguucuagagagguuu......................  47    1  Ta1
...................................................................cCaauucgguucuagagagguuu......................  3     1  Ta1
...................................................................caaaCucgguucuagagagguuu......................  5     1  Ta1
...................................................................caaauucgguucuagUgagguuu......................  1     1  Ta1
...................................................................caaauucgguucuCgagagguuu......................  3     1  Ta1
...................................................................caaauucgguucuagGgagguuu......................  1     1  Ta1
...................................................................caCauucgguucuagagagguuu......................  5     1  Ta1
...................................................................caaauucgguAcuagagagguuu......................  1     1  Ta1
...................................................................Aaaauucgguucuagagagguuu......................  9     1  Ta1
...................................................................caaauucgguucuagagaggCuu......................  1     1  Ta1
...................................................................caaauucgguucuagCgagguuu......................  1     1  Ta1
...................................................................caaauucgguucuaAagagguuu......................  1     1  Ta1
...................................................................caaauucgguuGuagagagguuu......................  1     1  Ta1
...................................................................caaauucgguucuagagagguuC......................  1     1  Ta1
...................................................................caaauucgguucGagagagguuu......................  1     1  Ta1
...................................................................caaauucgAuucuagagagguuu......................  4     1  Ta1
...................................................................caaauucgguucuagagagguuuU.....................  9     1  Ta1
....................................................................aaauucgguucuagagag..........................  5     0  Ta1
....................................................................aaauucgguucuagagCgg.........................  1     1  Ta1
....................................................................Caauucgguucuagagagg.........................  1     1  Ta1
....................................................................aaauucgguucuagagagA.........................  4     1  Ta1
....................................................................aaauCcgguucuagagagg.........................  1     1  Ta1
....................................................................aaauGcgguucuagagagg.........................  2     1  Ta1
....................................................................aaauucgguucuagagagg.........................  80    0  Ta1
....................................................................aaauAcgguucuagagagg.........................  1     1  Ta1
....................................................................aCauucgguucuagagaggu........................  1     1  Ta1
....................................................................Uaauucgguucuagagaggu........................  1     1  Ta1
....................................................................Caauucgguucuagagaggu........................  1     1  Ta1
....................................................................aaauGcgguucuagagaggu........................  2     1  Ta1
....................................................................aaaGucgguucuagagaggu........................  1     1  Ta1
....................................................................aaauucgguucuagagaggu........................  126   0  Ta1
....................................................................aaauucAguucuagagaggu........................  1     1  Ta1
....................................................................aaCuucgguucuagagaggu........................  2     1  Ta1
....................................................................Gaauucgguucuagagagguu.......................  1     1  Ta1
....................................................................aaauucgguucuagagagguA.......................  4     1  Ta1
....................................................................aaauucgguucuagagagguu.......................  1003  0  Ta1
....................................................................aaauCcgguucuagagagguu.......................  6     1  Ta1
....................................................................aaauucgguucuagagagguG.......................  5     1  Ta1
....................................................................aaauucUguucuagagagguu.......................  2     1  Ta1
....................................................................Uaauucgguucuagagagguu.......................  5     1  Ta1
....................................................................aaauucgguucuagagagUuu.......................  1     1  Ta1
....................................................................aaauucgguucuagagCgguu.......................  1     1  Ta1
....................................................................aaauucgguucCagagagguu.......................  1     1  Ta1
....................................................................aaaGucgguucuagagagguu.......................  9     1  Ta1
....................................................................aUauucgguucuagagagguu.......................  1     1  Ta1
....................................................................aaauucgguucuagagUgguu.......................  3     1  Ta1
....................................................................aaaAucgguucuagagagguu.......................  1     1  Ta1
```

```
                              Mature                                Star
ggcacgcgcuuuucuuaggugcuuuacaucuacccuguagauccgaauuugucuaggauucuaaccacaaauucgguucuagagagguuuaugugaugcauacgaaacgacc
....................................................................aaauucgguuUuagagagguu.......................  1     1  Ta1
....................................................................aaauGcgguucuagagagguu.......................  33    1  Ta1
....................................................................aaauucgguucuagagagguC.......................  14    1  Ta1
....................................................................aaauucgguuAuagagagguu.......................  1     1  Ta1
....................................................................Caauucgguucuagagagguu.......................  14    1  Ta1
....................................................................aaauucgUuucuagagagguu.......................  1     1  Ta1
....................................................................aaauuGgguucuagagagguu.......................  1     1  Ta1
....................................................................aaauAcgguucuagagagguu.......................  6     1  Ta1
....................................................................aaauuUgguucuagagagguu.......................  1     1  Ta1
....................................................................aaCuucgguucuagagagguu.......................  2     1  Ta1
....................................................................aaauucgguucuagaUagguu.......................  1     1  Ta1
....................................................................aaauucgguucuagUgagguu.......................  1     1  Ta1
....................................................................aaauCcgguucuagagagguuu......................  7     1  Ta1
....................................................................Caauucgguucuagagagguuu......................  18    1  Ta1
....................................................................aaauucgguucuagagagguuG......................  1     1  Ta1
....................................................................aaaCucgguucuagagagguuu......................  1     1  Ta1
....................................................................aaauucggCucuagagagguuu......................  1     1  Ta1
....................................................................aaauucgguucuUgagagguuu......................  1     1  Ta1
....................................................................aaauucgguucuagagagguuu......................  1158  0  Ta1
....................................................................aaauucgguucuagUgagguuu......................  1     1  Ta1
....................................................................aCauucgguucuagagagguuu......................  1     1  Ta1
....................................................................aaauucgguuAuagagagguuu......................  2     1  Ta1
....................................................................aaaGucgguucuagagagguuu......................  8     1  Ta1
....................................................................aaauAcgguucuagagagguuu......................  4     1  Ta1
....................................................................aaauucgguucuagagagUuuu......................  2     1  Ta1
....................................................................Uaauucgguucuagagagguuu......................  3     1  Ta1
....................................................................Naauucgguucuagagagguuu......................  1     1  Ta1
....................................................................aaauucgguucGagagagguuu......................  1     1  Ta1
....................................................................aaauucgguucuGgagagguuu......................  1     1  Ta1
....................................................................aaauucAguucuagagagguuu......................  1     1  Ta1
....................................................................aaauucgguucuagagaggGuu......................  5     1  Ta1
....................................................................aaauucgguucuagagagguuC......................  6     1  Ta1
....................................................................aaauucgguucuaAagagguuu......................  1     1  Ta1
....................................................................aaaAucgguucuagagagguuu......................  2     1  Ta1
....................................................................aaauucgguucuagagaggCuu......................  2     1  Ta1
....................................................................aaCuucgguucuagagagguuu......................  8     1  Ta1
....................................................................aaauucggAucuagagagguuu......................  1     1  Ta1
....................................................................aaauGcgguucuagagagguuu......................  45    1  Ta1
....................................................................aaauucgguucuagagagguuuU.....................  12    1  Ta1
....................................................................aaauucgguucuagagagguuua.....................  5     0  Ta1
....................................................................aaauucgguucuagagagguuuaA....................  6     1  Ta1
.....................................................................aauucgguucuagagaggu........................  1     0  Ta1
.....................................................................aauucgguucuagagagguu.......................  1     0  Ta1
.....................................................................aauucgguucuagagagguuu......................  2     0  Ta1
......................................................................auucgguucuagagagguu.......................  4     0  Ta1
......................................................................auucgguucuagagagguuu......................  4     0  Ta1

.............................cuacccuguagauccgaauuug.............................................................  1     0  Le5
.............................cuacccuguagauccUaauuugu............................................................  1     1  Le5
.............................cuacGcuguagauccgaauuugu............................................................  2     1  Le5
.............................cuacccuguagauccgaauuugu............................................................  18    0  Le5
.............................cuacUcuguagauccgaauuugu............................................................  1     1  Le5
.............................cuacccuguGgauccgaauuugu............................................................  1     1  Le5
.............................Auacccuguagauccgaauuugu............................................................  2     1  Le5
.............................cuacccuguagauccgaauuugC............................................................  1     1  Le5
.............................cuacccuguagauccgaaGuugu............................................................  1     1  Le5
.............................cuacccuguCgauccgaauuugu............................................................  1     1  Le5
.............................cuaccUuguagauccgaauuugu............................................................  1     1  Le5
.............................cuacccuguagauccgaauuuUu............................................................  2     1  Le5
..............................uaccUuguagauccgaau................................................................  1     1  Le5
..............................uacccuguagCuccgaau................................................................  1     1  Le5
..............................uaccGuguagauccgaau................................................................  1     1  Le5
..............................uacccuguagauccgaau................................................................  24    0  Le5
..............................uacccuguagauccgaCu................................................................  1     1  Le5
..............................uacccuguagauccgaaC................................................................  1     1  Le5
..............................uacccuguagaCccgaau................................................................  1     1  Le5
..............................uacccuguagauccgaaA................................................................  1     1  Le5
..............................uaccUuguagauccgaauu...............................................................  2     1  Le5
..............................uacccuguagauccgaauA...............................................................  12    1  Le5
..............................uacccuguagauccgaauu...............................................................  173   0  Le5
```

```
                              Mature                          Star
ggcacgcgcuuuucuuaggugcuuuacaucuacccuguagauccgaauuugucuaggauucuaaccacaaauucgguucuagagagguuuaugugaugcauacgaaacgacc
..............................uacccuguagauccgaCuu...............................................................   1     1   Le5
..............................Nacccuguagauccgaauu...............................................................   1     1   Le5
..............................uaAccuguagauccgaauu...............................................................   3     1   Le5
..............................uacccuguCgauccgaauu...............................................................   1     1   Le5
..............................uacccuguagCuccgaauu...............................................................   1     1   Le5
..............................uacccuguagauccgaauG...............................................................   1     1   Le5
..............................uacUcuguagauccgaauu...............................................................   1     1   Le5
..............................uacccuguagauccgUauu...............................................................   1     1   Le5
..............................uacccuguagauccUaauu...............................................................   1     1   Le5
..............................uacccugCagauccgaauu...............................................................   1     1   Le5
..............................Aacccuguagauccgaauu...............................................................   1     1   Le5
..............................uacccuguagauccgaUuuu..............................................................   1     1   Le5
..............................uacccGguagauccgaauuu..............................................................   1     1   Le5
..............................uaccUuguagauccgaauuu..............................................................   6     1   Le5
..............................uacUcuguagauccgaauuu..............................................................   1     1   Le5
..............................uacccuguagauccgUauuu..............................................................   1     1   Le5
..............................uacccuguagaucAgaauuu..............................................................   1     1   Le5
..............................uacccuguCgauccgaauuu..............................................................   4     1   Le5
..............................uGcccuguagauccgaauuu..............................................................   1     1   Le5
..............................uaccGuguagauccgaauuu..............................................................   1     1   Le5
..............................Aacccuguagauccgaauuu..............................................................   3     1   Le5
..............................uacccuguagCuccgaauuu..............................................................   1     1   Le5
..............................uacccuguagauccgaauuA..............................................................   1     1   Le5
..............................Cacccuguagauccgaauuu..............................................................   1     1   Le5
..............................uacccuguagauccgaauuu..............................................................   553   0   Le5
..............................Gacccuguagauccgaauuu..............................................................   1     1   Le5
..............................uCcccuguagauccgaauuu..............................................................   1     1   Le5
..............................uaAccuguagauccgaauuu..............................................................   4     1   Le5
..............................uacccuguagaucUgaauuug.............................................................   7     1   Le5
..............................uaccAuguagauccgaauuug.............................................................   1     1   Le5
..............................uacccuguagauccgaauGug.............................................................   2     1   Le5
..............................uacUcuguagauccgaauuug.............................................................   12    1   Le5
..............................uacccuguagauccgUauuug.............................................................   7     1   Le5
..............................uUcccuguagauccgaauuug.............................................................   3     1   Le5
..............................uacccuguagauccgaauuCg.............................................................   1     1   Le5
..............................uacccuguagaucAgaauuug.............................................................   1     1   Le5
..............................uacccuguagauccAaauuug.............................................................   2     1   Le5
..............................uacccuguagauccgaaGuug.............................................................   1     1   Le5
..............................uacccuguagauccgaauuuA.............................................................   24    1   Le5
..............................uacccuguagauAcgaauuug.............................................................   6     1   Le5
..............................uacccuguagauccgaauuuU.............................................................   5     1   Le5
..............................uacccuguagauccgaauCug.............................................................   1     1   Le5
..............................uacccuguagauccUaauuug.............................................................   7     1   Le5
..............................uacccugCagauccgaauuug.............................................................   2     1   Le5
..............................uacccuguGgauccgaauuug.............................................................   1     1   Le5
..............................uacccuguagCuccgaauuug.............................................................   16    1   Le5
..............................uacccuguCgauccgaauuug.............................................................   39    1   Le5
..............................uacccuguagUuccgaauuug.............................................................   3     1   Le5
..............................uacccuguagaGccgaauuug.............................................................   1     1   Le5
..............................uacccCguagauccgaauuug.............................................................   1     1   Le5
..............................uacccuguagauccgaauuGg.............................................................   7     1   Le5
..............................uaUccuguagauccgaauuug.............................................................   5     1   Le5
..............................uacccuguagauccgaGuuug.............................................................   2     1   Le5
..............................uacccuguagaAccgaauuug.............................................................   1     1   Le5
..............................uacccuguagauccgaaCuug.............................................................   1     1   Le5
..............................Cacccuguagauccgaauuug.............................................................   5     1   Le5
..............................uacccuguagauccgaauuug.............................................................   5459  0   Le5
..............................uacccuguagauUcgaauuug.............................................................   5     1   Le5
..............................uaccUuguagauccgaauuug.............................................................   62    1   Le5
..............................uacccuguagaCccgaauuug.............................................................   3     1   Le5
..............................uaAccuguagauccgaauuug.............................................................   55    1   Le5
..............................uaGccuguagauccgaauuug.............................................................   9     1   Le5
..............................Nacccuguagauccgaauuug.............................................................   6     1   Le5
..............................uacccuguagauccgaCuuug.............................................................   8     1   Le5
..............................uacccuguagauccgaauuuC.............................................................   1     1   Le5
..............................uacccuUuagauccgaauuug.............................................................   6     1   Le5
..............................uacccuguagauccgGauuug.............................................................   1     1   Le5
..............................uaccGuguagauccgaauuug.............................................................   37    1   Le5
..............................uacccuguagauccgaUuuug.............................................................   1     1   Le5
..............................uCcccuguagauccgaauuug.............................................................   10    1   Le5
```

```
                              Mature                                  Star
ggcacgcgcuuuucuuaggugcuuuacaucuacccuguagauccgaauuugucuaggauucuaaccacaaauucgguucuagagagguuuaugugaugcauacgaaacgacc
..............................uacccuguagGuccgaauuug.............................................................  2      1  Le5
..............................uacccGguagauccgaauuug.............................................................  1      1  Le5
..............................Aacccuguagauccgaauuug.............................................................  12     1  Le5
..............................uacccuguagauccCaauuug.............................................................  1      1  Le5
..............................uacccuguUgauccgaauuug.............................................................  1      1  Le5
..............................uGcccuguagauccgaauuug.............................................................  1      1  Le5
..............................uacccugAagauccgaauuug.............................................................  1      1  Le5
..............................uacccuguagCuccgaauuugu............................................................  130    1  Le5
..............................uacccGguagauccgaauuugu............................................................  8      1  Le5
..............................uacccugCagauccgaauuugu............................................................  7      1  Le5
..............................uaccAuguagauccgaauuugu............................................................  7      1  Le5
..............................uacccuguagauccgaauAugu............................................................  3      1  Le5
..............................uacccuguagauccgaCuuugu............................................................  53     1  Le5
..............................uacccuguagauccgaauCugu............................................................  7      1  Le5
..............................Cacccuguagauccgaauuugu............................................................  50     1  Le5
..............................uacccuguagauGcgaauuugu............................................................  2      1  Le5
..............................uacccuguagauccgaauuCgu............................................................  5      1  Le5
..............................uaccUuguagauccgaauuugu............................................................  527    1  Le5
..............................uacccuguagauccgaaCuugu............................................................  7      1  Le5
..............................uacccCguagauccgaauuugu............................................................  10     1  Le5
..............................uacccuguagauccgaauuuCu............................................................  10     1  Le5
..............................uacccuguagauccgaauuuUu............................................................  10     1  Le5
..............................uacccuguagaAccgaauuugu............................................................  4      1  Le5
..............................uacccuguagaucGgaauuugu............................................................  3      1  Le5
..............................uCcccuguagauccgaauuugu............................................................  107    1  Le5
..............................uacAcuguagauccgaauuugu............................................................  13     1  Le5
..............................uacccuguagauccgaauuugC............................................................  1117   1  Le5
..............................uacccuCuagauccgaauuugu............................................................  3      1  Le5
..............................uacccuguaCauccgaauuugu............................................................  1      1  Le5
..............................uacccuguagaucAgaauuugu............................................................  26     1  Le5
..............................uacccuAuagauccgaauuugu............................................................  3      1  Le5
..............................uacccuguagauccgaauuAgu............................................................  1      1  Le5
..............................uacccugGagauccgaauuugu............................................................  8      1  Le5
..............................uacccuguaAauccgaauuugu............................................................  10     1  Le5
..............................uacccuguagauccgGauuugu............................................................  9      1  Le5
..............................uacccuguagauccgaauuGgu............................................................  29     1  Le5
..............................uacccuguagauccgCauuugu............................................................  6      1  Le5
..............................uacccuguagauccUaauuugu............................................................  60     1  Le5
..............................uaccGuguagauccgaauuugu............................................................  367    1  Le5
..............................uacccuguaUauccgaauuugu............................................................  6      1  Le5
..............................uacccuguagauccgUauuugu............................................................  55     1  Le5
..............................uacccuguagauccCaauuugu............................................................  4      1  Le5
..............................uacUcuguagauccgaauuugu............................................................  165    1  Le5
..............................uacccuguagauccgaauuugG............................................................  25     1  Le5
..............................uGcccuguagauccgaauuugu............................................................  14     1  Le5
..............................uacccuguagauccgaauuugu............................................................  45731  0  Le5
..............................uacccuguagauUcgaauuugu............................................................  22     1  Le5
..............................uacccuguagauAcgaauuugu............................................................  21     1  Le5
..............................uacccuguagauccgaaAuugu............................................................  8      1  Le5
..............................uacccuguagauccgaauuugA............................................................  397    1  Le5
..............................uacccuguagauccgaaGuugu............................................................  2      1  Le5
..............................uacccuguUgauccgaauuugu............................................................  27     1  Le5
..............................uacccuguagUuccgaauuugu............................................................  13     1  Le5
..............................uacccuguagauccgaauGugu............................................................  43     1  Le5
..............................uacccuguagauccgaUuuugu............................................................  15     1  Le5
..............................Gacccuguagauccgaauuugu............................................................  10     1  Le5
..............................uUcccuguagauccgaauuugu............................................................  13     1  Le5
..............................uaGccuguagauccgaauuugu............................................................  61     1  Le5
..............................uacccuguagGuccgaauuugu............................................................  4      1  Le5
..............................uacccugAagauccgaauuugu............................................................  6      1  Le5
..............................uacccuguCgauccgaauuugu............................................................  318    1  Le5
..............................uacccAguagauccgaauuugu............................................................  4      1  Le5
..............................uaUccuguagauccgaauuugu............................................................  55     1  Le5
..............................uacccuguagauccgaauuuAu............................................................  9      1  Le5
..............................Nacccuguagauccgaauuugu............................................................  24     1  Le5
..............................uacccuguagauccgaGuuugu............................................................  7      1  Le5
..............................uacccuUuagauccgaauuugu............................................................  28     1  Le5
..............................Aacccuguagauccgaauuugu............................................................  63     1  Le5
..............................uacccuguagaGccgaauuugu............................................................  11     1  Le5
..............................uacccuguagaCccgaauuugu............................................................  4      1  Le5
```

```
                              Mature                                  Star
ggcacgcgcuuuucuuaggugcuuuacaucuacccuguagauccgaauuugucuaggauucuaaccacaaauucgguucuagagagguuuaugugaugcauacgaaacgacc
..............................uaAccuguagauccgaauuugu............................................................ 382   1 Le5
..............................uacccuguGgauccgaauuugu............................................................ 4     1 Le5
..............................uacccuguagaucUgaauuugu............................................................ 28    1 Le5
..............................uacccuguagauccAaauuugu............................................................ 16    1 Le5
..............................uacccuguagauccgaauuuguA........................................................... 1432  1 Le5
..............................Aacccuguagauccgaauuuguc........................................................... 2     1 Le5
..............................uacccuguagauccgaauuCguc........................................................... 1     1 Le5
..............................Cacccuguagauccgaauuuguc........................................................... 2     1 Le5
..............................uacccuguagauccgaauuuguc........................................................... 38    0 Le5
..............................uacccuguagauccgaauuuguG........................................................... 146   1 Le5
..............................uacccuguagauccgaauuugucG.......................................................... 1     1 Le5
..............................uacccuguagauccgaauuuguAu.......................................................... 9     1 Le5
..............................uacccuguagauccgaauuugucC.......................................................... 1     1 Le5
..............................uacccuguagauccgaauuuguGu.......................................................... 2     1 Le5
...............................acccGguagauccgaauu............................................................... 2     1 Le5
...............................acccuguagauccgaaCu............................................................... 1     1 Le5
...............................acccuguagauccgaauu............................................................... 30    0 Le5
...............................acccCguagauccgaauu............................................................... 1     1 Le5
...............................Ccccuguagauccgaauu............................................................... 1     1 Le5
...............................acccuguagCuccgaauu............................................................... 1     1 Le5
...............................acccuguagauccgaauA............................................................... 3     1 Le5
...............................acccGguagauccgaauuu.............................................................. 4     1 Le5
...............................acccuguagauccgaauuu.............................................................. 99    0 Le5
...............................acUcuguagauccgaauuu.............................................................. 1     1 Le5
...............................Ccccuguagauccgaauuu.............................................................. 3     1 Le5
...............................acccuguagauccgaauuA.............................................................. 2     1 Le5
...............................acccAguagauccgaauuu.............................................................. 2     1 Le5
...............................acccCguagauccgaauuug............................................................. 15    1 Le5
...............................acccuguagauccgaUuuug............................................................. 2     1 Le5
...............................acAcuguagauccgaauuug............................................................. 4     1 Le5
...............................acccuUuagauccgaauuug............................................................. 2     1 Le5
...............................acccuguagauccgaauuuA............................................................. 3     1 Le5
...............................Ucccuguagauccgaauuug............................................................. 4     1 Le5
...............................acccuguagauccCaauuug............................................................. 1     1 Le5
...............................Ccccuguagauccgaauuug............................................................. 9     1 Le5
...............................acccuguUgauccgaauuug............................................................. 1     1 Le5
...............................acccGguagauccgaauuug............................................................. 48    1 Le5
...............................acccuguaCauccgaauuug............................................................. 3     1 Le5
...............................acccuguagGuccgaauuug............................................................. 1     1 Le5
...............................acccuguagauGcgaauuug............................................................. 1     1 Le5
...............................Ncccuguagauccgaauuug............................................................. 1     1 Le5
...............................acccuAuagauccgaauuug............................................................. 1     1 Le5
...............................acccuguagauccgaauAug............................................................. 1     1 Le5
...............................aAccuguagauccgaauuug............................................................. 3     1 Le5
...............................acccuguagCuccgaauuug............................................................. 12    1 Le5
...............................acccuguagauccgaauuuU............................................................. 1     1 Le5
...............................acccuguagauccgaauuug............................................................. 1108  0 Le5
...............................acccuguagauccgaauuCg............................................................. 1     1 Le5
...............................acccuguagaucUgaauuug............................................................. 1     1 Le5
...............................acccuguagauUcgaauuug............................................................. 1     1 Le5
...............................accUuguagauccgaauuug............................................................. 3     1 Le5
...............................acccAguagauccgaauuug............................................................. 13    1 Le5
...............................Gcccuguagauccgaauuug............................................................. 1     1 Le5
...............................acccuguagauccgaauuAgu............................................................ 1     1 Le5
...............................acccuguagauccgUauuugu............................................................ 4     1 Le5
...............................acccuUuagauccgaauuugu............................................................ 8     1 Le5
...............................acGcuguagauccgaauuugu............................................................ 6     1 Le5
...............................accAuguagauccgaauuugu............................................................ 2     1 Le5
...............................acccuguagUuccgaauuugu............................................................ 9     1 Le5
...............................acUcuguagauccgaauuugu............................................................ 13    1 Le5
...............................acccuguagaucUgaauuugu............................................................ 9     1 Le5
...............................Ucccuguagauccgaauuugu............................................................ 37    1 Le5
...............................acccuguagauccCaauuugu............................................................ 1     1 Le5
...............................acccGguagauccgaauuugu............................................................ 591   1 Le5
...............................Gcccuguagauccgaauuugu............................................................ 10    1 Le5
...............................acccuguagauccgaauAugu............................................................ 3     1 Le5
...............................acccugGagauccgaauuugu............................................................ 5     1 Le5
...............................acccuguagauccgaaCuugu............................................................ 3     1 Le5
...............................acccugAagauccgaauuugu............................................................ 2     1 Le5
...............................acccuguaUauccgaauuugu............................................................ 8     1 Le5
```

Mature **Star**

ggcacgcgcuuuucuuaggugcuuuacaucuacccuguagauccgaauuuguc uaggauucuaaccacaaauucgguucuagagagguuuaugugaugcauacgaaacgacc

acccuguagauccgaauGugu	4	1	Le5
acccuguagauccgaauuugA	85	1	Le5
acccuguagCuccgaauuugu	80	1	Le5
accUuguagauccgaauuugu	48	1	Le5
acccuguaAauccgaauuugu	20	1	Le5
Ncccuguagauccgaauuugu	5	1	Le5
acccuguagauccgaauuugG	1	1	Le5
acccuguagauccgaauuGgu	5	1	Le5
acccuguagauccgaauuuCu	9	1	Le5
acccuguagauccgaUuuugu	10	1	Le5
acccuguagaucAgaauuugu	8	1	Le5
aGccuguagauccgaauuugu	2	1	Le5
acccuguagGuccgaauuugu	2	1	Le5
acccuguagauccUaauuugu	1	1	Le5
acccAguagauccgaauuugu	84	1	Le5
Ccccuguagauccgaauuugu	187	1	Le5
acAcuguagauccgaauuugu	68	1	Le5
acccuguagauccgaauCugu	9	1	Le5
aUccuguagauccgaauuugu	3	1	Le5
acccuguagauccgaauuugu	13093	0	Le5
acccuguagaGccgaauuugu	1	1	Le5
acccuguagauccAaauuugu	5	1	Le5
acccuguagauccgaauuuAu	2	1	Le5
acccuguagauccgaauuugC	489	1	Le5
accGuguagauccgaauuugu	1	1	Le5
acccuguagaucGgaauuugu	1	1	Le5
acccuguagauccgaCuuugu	6	1	Le5
acccuguagauccgaaAuugu	5	1	Le5
acccuguagauccgCauuugu	9	1	Le5
acccCguagauccgaauuugu	130	1	Le5
acccuguagauccgaauuuUu	30	1	Le5
acccuguagauUcgaauuugu	19	1	Le5
acccuguaCauccgaauuugu	5	1	Le5
acccuguUgauccgaauuugu	1	1	Le5
acccuguCgauccgaauuugu	2	1	Le5
acccugCagauccgaauuugu	2	1	Le5
acccuguagauccgaGuuugu	5	1	Le5
aAccuguagauccgaauuugu	19	1	Le5
acccuguagaAccgaauuugu	12	1	Le5
acccuguagauccgGauuugu	6	1	Le5
acccuAuagauccgaauuugu	3	1	Le5
acccuCuagauccgaauuugu	1	1	Le5
acccCguagauccgaauuuguc	25	1	Le5
acccugAagauccgaauuuguc	1	1	Le5
acAcuguagauccgaauuuguc	10	1	Le5
acccuguagauccgaauuuguG	27	1	Le5
Gcccuguagauccgaauuuguc	3	1	Le5
acccuguagUuccgaauuuguc	1	1	Le5
acccuguagaCccgaauuuguc	1	1	Le5
acccuguagauccgaauuuguA	260	1	Le5
acccuUuagauccgaauuuguc	1	1	Le5
acccuguagauccgaauuuguc	2989	0	Le5
acccuguagauccAaauuuguc	4	1	Le5
acccuguagauccgaauuuUuc	2	1	Le5
acccuguagaucUgaauuuguc	10	1	Le5
acccugCagauccgaauuuguc	4	1	Le5
acccAguagauccgaauuuguc	23	1	Le5
acccuguagauccgaauuugCc	9	1	Le5
acccuguagauccgaauuugAc	1	1	Le5
acccuguagCuccgaauuuguc	16	1	Le5
Ucccuguagauccgaauuuguc	9	1	Le5
acccuguCgauccgaauuuguc	1	1	Le5
accUuguagauccgaauuuguc	7	1	Le5
acccuguaAauccgaauuuguc	2	1	Le5
acccugGagauccgaauuuguc	4	1	Le5
aAccuguagauccgaauuuguc	3	1	Le5
acccuguagauccgaauGuguc	1	1	Le5
acccuCuagauccgaauuuguc	1	1	Le5
Ccccuguagauccgaauuuguc	28	1	Le5
acccuguagauccgaCuuuguc	1	1	Le5

```
                              Mature                              Star
ggcacgcgcuuuucuuaggugcuuuacaucuacccuguagauccgaauuugucuaggauucuaaccacaaauucgguucuagagagguuuaugugaugcauacgaaacgacc
...............................acccuAuagauccgaauuuguc...........................................................   1     1   Le5
...............................acccGguagauccgaauuuguc...........................................................   124   1   Le5
...............................acGcuguagauccgaauuuguc...........................................................   3     1   Le5
...............................acccuguagauccgaauuAguc...........................................................   1     1   Le5
...............................acUcuguagauccgaauuuguc...........................................................   4     1   Le5
...............................acccuguagauccgaaCuuguc...........................................................   1     1   Le5
...............................acccuguagauUcgaauuuguc...........................................................   10    1   Le5
...............................acccuguUgauccgaauuuguc...........................................................   1     1   Le5
...............................aUccuguagauccgaauuuguc...........................................................   3     1   Le5
...............................acccuguagaAccgaauuuguc...........................................................   2     1   Le5
...............................acccuguagauccgaauuuguGu..........................................................   3     1   Le5
...............................acccGguagauccgaauuugucu..........................................................   4     1   Le5
...............................accUuguagauccgaauuugucu..........................................................   1     1   Le5
...............................acccuguagauccgaauuugucC..........................................................   7     1   Le5
...............................acccuguagauccgaauuugucG..........................................................   24    1   Le5
...............................Ccccuguagauccgaauuugucu..........................................................   1     1   Le5
...............................acccCguagauccgaauuugucu..........................................................   1     1   Le5
...............................acccuguagauccgaauuuguAu..........................................................   1     1   Le5
...............................acccuguagauccgaauuCgucu..........................................................   1     1   Le5
...............................acccuguagauccgaauuugucu..........................................................   66    0   Le5
...............................acccuguagauccgaauuugucAa.........................................................   2     1   Le5
...............................acccuguagauccgaauuugucuaU........................................................   1     1   Le5
................................cccuguagauccgaauuu..............................................................   1     0   Le5
................................cccuguagauccgaUuuu..............................................................   1     1   Le5
................................cccuguagauccgaauuug.............................................................   28    0   Le5
................................cccuguagCuccgaauuug.............................................................   1     1   Le5
................................cccuUuagauccgaauuug.............................................................   1     1   Le5
................................cccuguagauccgaUuuug.............................................................   1     1   Le5
................................cccuguagauccgaauuGg.............................................................   1     1   Le5
................................cccugAagauccgaauuug.............................................................   1     1   Le5
................................cccGguagauccgaauuug.............................................................   1     1   Le5
................................cccuguagauccgaauuugu............................................................   241   0   Le5
................................cccuguagauccgaauuuUu............................................................   14    1   Le5
................................cccuguagauccAaauuugu............................................................   1     1   Le5
................................cccuguagGuccgaauuugu............................................................   1     1   Le5
................................ccAuguagauccgaauuugu............................................................   1     1   Le5
................................cccuguagauccgaauuGgu............................................................   4     1   Le5
................................ccUuguagauccgaauuugu............................................................   3     1   Le5
................................cccCguagauccgaauuugu............................................................   1     1   Le5
................................cccGguagauccgaauuugu............................................................   9     1   Le5
................................cccuguagauccgaUuuugu............................................................   2     1   Le5
................................cccuCuagauccgaauuugu............................................................   1     1   Le5
................................cccuguagauccgaauuugC............................................................   12    1   Le5
................................cccuguagUuccgaauuugu............................................................   8     1   Le5
................................cccuguagaucGgaauuugu............................................................   1     1   Le5
................................cccugAagauccgaauuugu............................................................   1     1   Le5
................................cccuUuagauccgaauuugu............................................................   28    1   Le5
................................Accuguagauccgaauuugu............................................................   1     1   Le5
................................cccuguagCuccgaauuugu............................................................   2     1   Le5
................................cccGguagauccgaauuuguc...........................................................   1     1   Le5
................................ccUuguagauccgaauuuguc...........................................................   2     1   Le5
................................cccuguagauccgaauuuguc...........................................................   68    0   Le5
................................cccuguagauccgaauuuUuc...........................................................   2     1   Le5
................................cccuguagauccgaauuGguc...........................................................   1     1   Le5
................................cccuguagUuccgaauuuguc...........................................................   1     1   Le5
................................cccuUuagauccgaauuuguc...........................................................   4     1   Le5
................................cccuguagauccgaUuuuguc...........................................................   1     1   Le5
................................cccuguagauccgaauuuguA...........................................................   5     1   Le5
................................cccuguagauccgaauuugucu..........................................................   1     0   Le5
................................cccuguagauccgaauuugucAa.........................................................   1     1   Le5
.................................ccuguagauccgaauuug.............................................................   4     0   Le5
.................................ccugGagauccgaauuugu............................................................   1     1   Le5
.................................ccuguagauccgaauuugu............................................................   22    0   Le5
.................................ccuguagauccgaauuuguc...........................................................   6     0   Le5
.................................ccuguagauccgaauuuguA...........................................................   1     1   Le5
.................................ccuguagauAcgaauuuguc...........................................................   1     1   Le5
..................................cuguagauccgaauuugu............................................................   3     0   Le5
..................................cuCuagauccgaauuugu............................................................   1     1   Le5
..................................cuUuagauccgaauuugu............................................................   1     1   Le5
..................................................................Ccaaauucgguucuagagagguuu......................   2     1   Le5
```

```
                              Mature                                      Star
ggcacgcgcuuuucuuaggugcuuuacaucuacccuguagauccgaauuugucuaggauucuaaccaccaaauucgguucuagagagguuuaugugaugcauacgaaacgacc
..................................................................acaaauucgguucuagagagguuu......................   2     0   Le5
...................................................................cGaauucgguucuagaga...........................   1     1   Le5
...................................................................caaaGucgguucuagaga...........................   4     1   Le5
...................................................................caaauucgguucuagaga...........................   71    0   Le5
...................................................................caaauucgguucuagUga...........................   1     1   Le5
...................................................................caaauucgguucuagagaA..........................   18    1   Le5
...................................................................caaauucgguucuagagag..........................   10    0   Le5
...................................................................caaauucgguucuagagagA.........................   6     1   Le5
...................................................................caaaGucgguucuagagagg.........................   2     1   Le5
...................................................................caaauucgguucuagagagg.........................   65    0   Le5
...................................................................caaauucgguucuagagUgg.........................   2     1   Le5
...................................................................caaauucgAuucuagagaggu........................   1     1   Le5
...................................................................caaauucgguucuagagaggC........................   9     1   Le5
...................................................................caaaGucgguucuagagaggu........................   3     1   Le5
...................................................................caaauucgguucuagagaggA........................   1     1   Le5
...................................................................caaauucgguuUuagagaggu........................   1     1   Le5
...................................................................caaauucgguucuagagaggu........................   50    0   Le5
...................................................................caaauucgguucuagagagguA.......................   4     1   Le5
...................................................................caaauucgguucuagagagguC.......................   29    1   Le5
...................................................................caaauucgguucuagagagguu.......................   504   0   Le5
...................................................................caaauucgguucuagagagAuu.......................   1     1   Le5
...................................................................Aaaauucgguucuagagagguu.......................   4     1   Le5
...................................................................caaaCucgguucuagagagguu.......................   1     1   Le5
...................................................................caaauucgguucuagUgagguu.......................   3     1   Le5
...................................................................caaauucgguucuagagagguG.......................   1     1   Le5
...................................................................cCaauucgguucuagagagguu.......................   3     1   Le5
...................................................................caaauucgguucuCgagagguu.......................   1     1   Le5
...................................................................caaaAucgguucuagagagguu.......................   7     1   Le5
...................................................................caaUuucgguucuagagagguu.......................   1     1   Le5
...................................................................caaauucgguucuaAagagguu.......................   1     1   Le5
...................................................................caaaGucgguucuagagagguu.......................   29    1   Le5
...................................................................caaauucgguAcuagagagguuu......................   2     1   Le5
...................................................................caaauucgguucuCgagagguuu......................   21    1   Le5
...................................................................caaauucgguucuagagagCuuu......................   2     1   Le5
...................................................................caaauucgguGcuagagagguuu......................   1     1   Le5
...................................................................caaauucgguucuagagaUguuu......................   7     1   Le5
...................................................................caaauucgguucuagCgagguuu......................   2     1   Le5
...................................................................caaauucgAuucuagagagguuu......................   7     1   Le5
...................................................................caaauucgguucuagagaggGuu......................   1     1   Le5
...................................................................Aaaauucgguucuagagagguuu......................   70    1   Le5
...................................................................caaauucgguucuaUagagguuu......................   12    1   Le5
...................................................................caaauucgguucuagagGgguuu......................   1     1   Le5
...................................................................caaauucgguucAagagagguuu......................   1     1   Le5
...................................................................caaUuucgguucuagagagguuu......................   24    1   Le5
...................................................................caaauucgguucuaAagagguuu......................   1     1   Le5
...................................................................caaauucggCucuagagagguuu......................   1     1   Le5
...................................................................caaauucgguucuagagaggCuu......................   4     1   Le5
...................................................................caaauucgguucuagagagAuuu......................   1     1   Le5
...................................................................caaauucAguucuagagagguuu......................   1     1   Le5
...................................................................caaauucgguucuagaAagguuu......................   1     1   Le5
...................................................................cCaauucgguucuagagagguuu......................   20    1   Le5
...................................................................caUauucgguucuagagagguuu......................   1     1   Le5
...................................................................caaauuUgguucuagagagguuu......................   4     1   Le5
...................................................................Uaaauucgguucuagagagguuu......................   25    1   Le5
...................................................................caGauucgguucuagagagguuu......................   1     1   Le5
...................................................................caaauucgguucuGgagagguuu......................   2     1   Le5
...................................................................caaauucgguucuagagagUuuu......................   16    1   Le5
...................................................................caaaAucgguucuagagagguuu......................   49    1   Le5
...................................................................Gaaauucgguucuagagagguuu......................   3     1   Le5
...................................................................caaauucgguucuagagagguuC......................   117   1   Le5
...................................................................caaauucUguucuagagagguuu......................   1     1   Le5
...................................................................caaauAcgguucuagagagguuu......................   2     1   Le5
...................................................................caaauucgguucCagagagguuu......................   1     1   Le5
...................................................................caaauucgguucuagUgagguuu......................   6     1   Le5
...................................................................caaauucgguuAuagagagguuu......................   1     1   Le5
...................................................................caaauucggAucuagagagguuu......................   3     1   Le5
...................................................................caaauuGgguucuagagagguuu......................   1     1   Le5
...................................................................caaauucgguucuagagCgguuu......................   3     1   Le5
...................................................................caaauucgguucuaCagagguuu......................   1     1   Le5
...................................................................caaauucgguucuagagaggAuu......................   3     1   Le5
```

```
                              Mature                                Star
ggcacgcgcuuuucuuaggugcuuuacaucuacccuguagauccgaauuugucuaggauucuaaccacaaauucgguucuagagagguuuaugugaugcauacgaaacgacc
...................................................................caaauucgguucuagagagguCu......................   4      1    Le5
...................................................................caaauucgguucuagagUgguuu......................   11     1    Le5
...................................................................caaauucgguCcuagagagguuu......................   2      1    Le5
...................................................................caaaGucgguucuagagagguuu......................   276    1    Le5
...................................................................caCauucgguucuagagagguuu......................   16     1    Le5
...................................................................caaGuucgguucuagagagguuu......................   1      1    Le5
...................................................................caaauucgCuucuagagagguuu......................   4      1    Le5
...................................................................caaauucgguuUuagagagguuu......................   3      1    Le5
...................................................................Naaauucgguucuagagagguuu......................   5      1    Le5
...................................................................caaaCucgguucuagagagguuu......................   42     1    Le5
...................................................................caaauucgguucuagagagguGu......................   2      1    Le5
...................................................................caaauucgguucuagagagguuu......................   7144   0    Le5
...................................................................caaauCcgguucuagagagguuu......................   3      1    Le5
...................................................................caaauucgguucuagagagguuuU.....................   104    1    Le5
...................................................................caaauucgguucuagaAagguuua.....................   1      1    Le5
...................................................................caaUuucgguucuagagagguuua.....................   1      1    Le5
...................................................................caaauucgguucuagagagguuua.....................   27     0    Le5
...................................................................caaauucgguucuagagagguuuaA....................   3      1    Le5
...................................................................caaauucgguucuagagagguuuau....................   1      0    Le5
...................................................................caaauucgguucuagagagguuuUu....................   3      1    Le5
....................................................................aaauucgguucuagagag..........................   28     0    Le5
....................................................................aaauucAguucuagagagg.........................   1      1    Le5
....................................................................aaauucggCucuagagagg.........................   2      1    Le5
....................................................................aaauuUgguucuagagagg.........................   1      1    Le5
....................................................................aaauucgguucuagagagg.........................   183    0    Le5
....................................................................aaauucgguucuagagagA.........................   20     1    Le5
....................................................................aaauGcgguucuagagagg.........................   8      1    Le5
....................................................................aaauCcgguucuagagagg.........................   2      1    Le5
....................................................................aaauucgguucuagagagU.........................   2      1    Le5
....................................................................aCauucgguucuagagagg.........................   1      1    Le5
....................................................................aUauucgguucuagagagg.........................   1      1    Le5
....................................................................aaCuucgguucuagagagg.........................   1      1    Le5
....................................................................Caauucgguucuagagagg.........................   2      1    Le5
....................................................................aaauucgguuAuagagaggu........................   1      1    Le5
....................................................................Gaauucgguucuagagaggu........................   1      1    Le5
....................................................................aaauAcgguucuagagaggu........................   3      1    Le5
....................................................................aaauucgguucuagagaggC........................   48     1    Le5
....................................................................aaauucgguucGagagaggu........................   1      1    Le5
....................................................................Caauucgguucuagagaggu........................   6      1    Le5
....................................................................aaauuUgguucuagagaggu........................   1      1    Le5
....................................................................aaauucgguucuagagaggA........................   6      1    Le5
....................................................................Uaauucgguucuagagaggu........................   1      1    Le5
....................................................................aaauCcgguucuagagaggu........................   3      1    Le5
....................................................................aaauucgguucuagaUaggu........................   1      1    Le5
....................................................................aaCuucgguucuagagaggu........................   2      1    Le5
....................................................................aaaGucgguucuagagaggu........................   1      1    Le5
....................................................................aaauucUguucuagagaggu........................   1      1    Le5
....................................................................aaauGcgguucuagagaggu........................   6      1    Le5
....................................................................aaauucgguucuagagaggu........................   322    0    Le5
....................................................................aaauucgguucuagCgagguu.......................   4      1    Le5
....................................................................aaauucgguucuagagagUuu.......................   5      1    Le5
....................................................................aaauucUguucuagagagguu.......................   3      1    Le5
....................................................................aaauucgguucuagagaggCu.......................   3      1    Le5
....................................................................Caauucgguucuagagagguu.......................   31     1    Le5
....................................................................aaauuGgguucuagagagguu.......................   1      1    Le5
....................................................................aaauucgguucuagUgagguu.......................   1      1    Le5
....................................................................aaauuUgguucuagagagguu.......................   7      1    Le5
....................................................................aaauAcgguucuagagagguu.......................   15     1    Le5
....................................................................aaauucgguucuagagaggGu.......................   3      1    Le5
....................................................................aaauucgguuAuagagagguu.......................   3      1    Le5
....................................................................aaauucgguucuagaUagguu.......................   5      1    Le5
....................................................................aaauGcgguucuagagagguu.......................   126    1    Le5
....................................................................aaauCcgguucuagagagguu.......................   31     1    Le5
....................................................................aaauucggCucuagagagguu.......................   5      1    Le5
....................................................................aGauucgguucuagagagguu.......................   1      1    Le5
....................................................................aaaGucgguucuagagagguu.......................   35     1    Le5
....................................................................aCauucgguucuagagagguu.......................   5      1    Le5
....................................................................aaaAucgguucuagagagguu.......................   2      1    Le5
....................................................................aaaCucgguucuagagagguu.......................   1      1    Le5
....................................................................aUauucgguucuagagagguu.......................   1      1    Le5
```

Mature **Star**

```
ggcacgcgcuuuucuuaggugcuuuacaucuacccuguagauccgaauuugucuaggauucuaaccacaaauucgguucuagagagguuuaugugaugcauacgaaacgacc
....................................................................aaauucgguucuagagagguG.......................  5     1  Le5
....................................................................aaauucgguucuagaAagguu.......................  1     1  Le5
....................................................................aaauucgguucuagagagguC.......................  176   1  Le5
....................................................................aaauucgguucuGgagagguu.......................  1     1  Le5
....................................................................aaauucgguucuCgagagguu.......................  4     1  Le5
....................................................................aaauucggAucuagagagguu.......................  4     1  Le5
....................................................................aaauucgUuucuagagagguu.......................  2     1  Le5
....................................................................aaauucgguucuagagagguA.......................  49    1  Le5
....................................................................aaauucgguucuagagagguu.......................  3472  0  Le5
....................................................................aaauucgguucuagagUgguu.......................  2     1  Le5
....................................................................Naauucgguucuagagagguu.......................  1     1  Le5
....................................................................aaauucgguucuagagCgguu.......................  7     1  Le5
....................................................................aaauucgguCcuagagagguu.......................  1     1  Le5
....................................................................aaauucgguucuagGgagguu.......................  1     1  Le5
....................................................................aaauucgguucuagagGgguu.......................  1     1  Le5
....................................................................aaauucgguAcuagagagguu.......................  1     1  Le5
....................................................................aaauuAgguucuagagagguu.......................  1     1  Le5
....................................................................aaCuucgguucuagagagguu.......................  10    1  Le5
....................................................................Gaauucgguucuagagagguu.......................  3     1  Le5
....................................................................aaauucgguucuUgagagguu.......................  1     1  Le5
....................................................................aaauucgguucuagagaggAu.......................  1     1  Le5
....................................................................Uaauucgguucuagagagguu.......................  10    1  Le5
....................................................................aaauucAguucuagagagguu.......................  1     1  Le5
....................................................................aaauucgAuucuagagagguuu......................  1     1  Le5
....................................................................aaauuAgguucuagagagguuu......................  1     1  Le5
....................................................................Caauucgguucuagagagguuu......................  117   1  Le5
....................................................................aaauucUguucuagagagguuu......................  7     1  Le5
....................................................................aaaGucgguucuagagagguuu......................  47    1  Le5
....................................................................aaauucgguucuagagCgguuu......................  9     1  Le5
....................................................................aaauucAguucuagagagguuu......................  4     1  Le5
....................................................................aaauucgguucuagagaCguuu......................  1     1  Le5
....................................................................aaGuucgguucuagagagguuu......................  2     1  Le5
....................................................................aaauucgguucuCgagagguuu......................  9     1  Le5
....................................................................aaaCucgguucuagagagguuu......................  7     1  Le5
....................................................................aaauucgguucuagagagAuuu......................  2     1  Le5
....................................................................Uaauucgguucuagagagguuu......................  39    1  Le5
....................................................................aaauucgguuUuagagagguuu......................  7     1  Le5
....................................................................aaauucgguucuagagagguuC......................  123   1  Le5
....................................................................Naauucgguucuagagagguuu......................  5     1  Le5
....................................................................aaauucggCucuagagagguuu......................  1     1  Le5
....................................................................aaCuucgguucuagagagguuu......................  18    1  Le5
....................................................................aaauucgguucuagGgagguuu......................  4     1  Le5
....................................................................aaauucgUuucuagagagguuu......................  3     1  Le5
....................................................................aaauucgguucuagaUagguuu......................  1     1  Le5
....................................................................aaauucgguucuagagagUuuu......................  14    1  Le5
....................................................................aaaAucgguucuagagagguuu......................  10    1  Le5
....................................................................aaauucgguucuaAagagguuu......................  1     1  Le5
....................................................................aaauucgguucuUgagagguuu......................  5     1  Le5
....................................................................aaauucggAucuagagagguuu......................  7     1  Le5
....................................................................aaauucCguucuagagagguuu......................  1     1  Le5
....................................................................aaauuUgguucuagagagguuu......................  8     1  Le5
....................................................................aaauucgguucuagagaggGuu......................  10    1  Le5
....................................................................aaauucgguuAuagagagguuu......................  10    1  Le5
....................................................................aaauGcgguucuagagagguuu......................  284   1  Le5
....................................................................aaauucgguucGagagagguuu......................  1     1  Le5
....................................................................aaauucgguucuagCgagguuu......................  1     1  Le5
....................................................................aaauucgguucuagagagguuG......................  6     1  Le5
....................................................................aaauucgguCcuagagagguuu......................  1     1  Le5
....................................................................aaauucgguucCagagagguuu......................  4     1  Le5
....................................................................aaauucgguucuagagagguGu......................  1     1  Le5
....................................................................aaauucgguucuagagaggCuu......................  1     1  Le5
....................................................................aaauucgguucuagagUgguuu......................  1     1  Le5
....................................................................aaauucgguucuagagagCuuu......................  2     1  Le5
....................................................................aaauucgguucuagagagguCu......................  4     1  Le5
....................................................................aaauucgguucuagagGgguuu......................  4     1  Le5
....................................................................aaauCcgguucuagagagguuu......................  79    1  Le5
....................................................................aaauucgguucuGgagagguuu......................  3     1  Le5
....................................................................aGauucgguucuagagagguuu......................  1     1  Le5
....................................................................aaauAcgguucuagagagguuu......................  33    1  Le5
....................................................................aaauucgguucuagagagguAu......................  3     1  Le5
```

```
                              Mature                                Star
ggcacgcgcuuuucuuaggugcuuuacaucuacccuguagauccgaauuugucuaggauucuaaccacaaauucgguucuagagagguuuaugugaugcauacgaaacgacc
....................................................................aaauucgguucuagagaUguuu......................   6      1   Le5
....................................................................aaauucgguucuagagagguuu......................   8479   0   Le5
....................................................................aUauucgguucuagagagguuu......................   1      1   Le5
....................................................................aCauucgguucuagagagguuu......................   16     1   Le5
....................................................................Gaauucgguucuagagagguuu......................   7      1   Le5
....................................................................aaauucgguucuagUgagguuu......................   2      1   Le5
....................................................................aaaGucgguucuagagagguuua.....................   1      1   Le5
....................................................................aaauucgguucuagagagguuuU.....................   103    1   Le5
....................................................................aaauCcgguucuagagagguuua.....................   1      1   Le5
....................................................................aaauGcgguucuagagagguuua.....................   2      1   Le5
....................................................................aaauucgguucuagagagguuua.....................   39     0   Le5
....................................................................aaauucgguucuagagagguuuau....................   2      0   Le5
....................................................................aaauucgguucuagagagguuuaA....................   2      1   Le5
....................................................................aaauucgguucuagagagguuuUu....................   3      1   Le5
.....................................................................aauCcgguucuagagaggu........................   1      1   Le5
.....................................................................aauucgguucuagagGggu........................   1      1   Le5
.....................................................................aauucgguucuagagaggu........................   5      0   Le5
.....................................................................aauuGgguucuagagagguuu......................   1      1   Le5
.....................................................................aauGcgguucuagagagguuu......................   2      1   Le5
.....................................................................aauucgUuucuagagagguuu......................   1      1   Le5
.....................................................................aauucgguucuagagagguuu......................   15     0   Le5
.....................................................................aauucgguAcuagagagguuu......................   1      1   Le5
.....................................................................Cauucgguucuagagagguuu......................   1      1   Le5
.....................................................................aCuucgguucuagagagguuu......................   1      1   Le5
.....................................................................aauucgguucuagCgagguuu......................   1      1   Le5
.....................................................................aauucUguucuagagagguuu......................   1      1   Le5
......................................................................auucgguucuagagaggu........................   1      0   Le5
......................................................................auucgguucuagagagguuu......................   4      0   Le5
......................................................................auucUguucuagagagguuu......................   1      1   Le5
.......................................................................uucgguucuagagagguuu......................   3      0   Le5
.......................................................................uucgguucuagagagguuuUu....................   1      1   Le5

..............................uacccuguagauccgaau................................................................   8      0   Le1
..............................uacccuguagCuccgaau................................................................   1      1   Le1
..............................uacccuguagauccgaauu...............................................................   24     0   Le1
..............................uaAccuguagauccgaauu...............................................................   1      1   Le1
..............................uacccuUuagauccgaauu...............................................................   1      1   Le1
..............................uacccuguagauccgaauA...............................................................   1      1   Le1
..............................uacccuUuagauccgaauuu..............................................................   1      1   Le1
..............................uaAccuguagauccgaauuu..............................................................   1      1   Le1
..............................uacccuguagauccgaauuu..............................................................   62     0   Le1
..............................uacccuguagauccgaauuA..............................................................   2      1   Le1
..............................uacccuguagauccgaUuuug.............................................................   1      1   Le1
..............................uaccGuguagauccgaauuug.............................................................   3      1   Le1
..............................uacccuguagauccgaauuGg.............................................................   1      1   Le1
..............................uacccuguagauccgaauuuU.............................................................   1      1   Le1
..............................uaGccuguagauccgaauuug.............................................................   2      1   Le1
..............................uacccuguCgauccgaauuug.............................................................   1      1   Le1
..............................uacccuguagauccUaauuug.............................................................   1      1   Le1
..............................uacccuguagauccgUauuug.............................................................   1      1   Le1
..............................uacccuguagaucAgaauuug.............................................................   1      1   Le1
..............................uaAccuguagauccgaauuug.............................................................   5      1   Le1
..............................uacUcuguagauccgaauuug.............................................................   2      1   Le1
..............................uacccuguagauccAaauuug.............................................................   1      1   Le1
..............................uacccuguagauccgaauuug.............................................................   557    0   Le1
..............................uacccuguagaucUgaauuug.............................................................   1      1   Le1
..............................uacccuguagauccgaaAuug.............................................................   1      1   Le1
..............................uCcccuguagauccgaauuug.............................................................   1      1   Le1
..............................uaccUuguagauccgaauuug.............................................................   6      1   Le1
..............................Cacccuguagauccgaauuug.............................................................   1      1   Le1
..............................uacccugAagauccgaauuug.............................................................   1      1   Le1
..............................uacccuguagauccgaauuuA.............................................................   2      1   Le1
..............................uacccuguagauUcgaauuug.............................................................   3      1   Le1
..............................uacccuguagCuccgaauuug.............................................................   2      1   Le1
..............................uacccuguagauAcgaauuug.............................................................   1      1   Le1
..............................uacccuguaCauccgaauuugu............................................................   1      1   Le1
..............................uacccuguagauccgUauuugu............................................................   9      1   Le1
..............................uacccuguCgauccgaauuugu............................................................   32     1   Le1
..............................uaUccuguagauccgaauuugu............................................................   6      1   Le1
..............................uacccuguagauccgaaAuugu............................................................   4      1   Le1
```

```
                              Mature                              Star
ggcacgcgcuuuucuuaggugcuuuacaucuacccuguagauccgaauuugucuaggauucuaaccacaaauucgguucuagagagguuuaugugaugcauacgaaacgacc
..............................uacccuAuagauccgaauuugu............................................................ 1     1  Le1
..............................uacccuguUgauccgaauuugu............................................................ 3     1  Le1
..............................uacAcuguagauccgaauuugu............................................................ 1     1  Le1
..............................uacccuguagauccgaauuugG............................................................ 3     1  Le1
..............................uCcccuguagauccgaauuugu............................................................ 11    1  Le1
..............................uacccuguagauccgaauuugC............................................................ 119   1  Le1
..............................uaAccuguagauccgaauuugu............................................................ 32    1  Le1
..............................Aacccuguagauccgaauuugu............................................................ 8     1  Le1
..............................uaGccuguagauccgaauuugu............................................................ 5     1  Le1
..............................uacccuguGgauccgaauuugu............................................................ 1     1  Le1
..............................Cacccuguagauccgaauuugu............................................................ 3     1  Le1
..............................uacccuguagauccgaauuuCu............................................................ 2     1  Le1
..............................uacccuguagaGccgaauuugu............................................................ 1     1  Le1
..............................uacccuguagauAcgaauuugu............................................................ 1     1  Le1
..............................uacccuguagauccUaauuugu............................................................ 6     1  Le1
..............................uacccuguagCuccgaauuugu............................................................ 19    1  Le1
..............................uacccGguagauccgaauuugu............................................................ 1     1  Le1
..............................uacccuguaUauccgaauuugu............................................................ 3     1  Le1
..............................Nacccuguagauccgaauuugu............................................................ 3     1  Le1
..............................uacccuguagaucUgaauuugu............................................................ 8     1  Le1
..............................uacUcuguagauccgaauuugu............................................................ 21    1  Le1
..............................uacccuguagUuccgaauuugu............................................................ 1     1  Le1
..............................uacccugCagauccgaauuugu............................................................ 1     1  Le1
..............................uacccuguagauccgCauuugu............................................................ 2     1  Le1
..............................uacccuguagauccgaauuugu............................................................ 4096  0  Le1
..............................uacccuguagaucAgaauuugu............................................................ 3     1  Le1
..............................uacccuguagauUcgaauuugu............................................................ 1     1  Le1
..............................uacccuguagauccgaaCuugu............................................................ 1     1  Le1
..............................uacccuguagauccgaauuuUu............................................................ 1     1  Le1
..............................uacccuUuagauccgaauuugu............................................................ 3     1  Le1
..............................uacccuguagauccgaauAugu............................................................ 1     1  Le1
..............................uacccuguagauccAaauuugu............................................................ 1     1  Le1
..............................uacccuguagauccgaauuugA............................................................ 26    1  Le1
..............................uaccUuguagauccgaauuugu............................................................ 52    1  Le1
..............................uGcccuguagauccgaauuugu............................................................ 2     1  Le1
..............................uaccGuguagauccgaauuugu............................................................ 33    1  Le1
..............................uacccuguagauccgaauuCgu............................................................ 1     1  Le1
..............................uacccuguagauccgaauGugu............................................................ 1     1  Le1
..............................uacccuguagauccgaauuGgu............................................................ 2     1  Le1
..............................uacccuguagauccgGauuugu............................................................ 1     1  Le1
..............................uacccCguagauccgaauuugu............................................................ 2     1  Le1
..............................uacccuguagauccgaUuuugu............................................................ 4     1  Le1
..............................uacccuguagaAccgaauuugu............................................................ 1     1  Le1
..............................uaccAuguagauccgaauuugu............................................................ 3     1  Le1
..............................uacccuguagauccgaCuuugu............................................................ 4     1  Le1
..............................uacccuguagauccgaauuuguA........................................................... 146   1  Le1
..............................uacccuguagauccgaauuuguG........................................................... 16    1  Le1
..............................uacccuguagauccgaauuuguc........................................................... 2     0  Le1
..............................uacccuguagauccgaauuugucu.......................................................... 1     0  Le1
..............................uacccuguagauccgaauuuguAu.......................................................... 1     1  Le1
...............................acccuguagauccgaauA............................................................... 1     1  Le1
...............................acccuguagauccgaauu............................................................... 16    0  Le1
...............................acccGguagauccgaauuu.............................................................. 2     1  Le1
...............................Ucccuguagauccgaauuu.............................................................. 1     1  Le1
...............................acccuguagauccgaauuu.............................................................. 25    0  Le1
...............................acccuguagauccgaauuA.............................................................. 1     1  Le1
...............................acccuguagaucAgaauuu.............................................................. 1     1  Le1
...............................acccuUuagauccgaauuu.............................................................. 1     1  Le1
...............................Ucccuguagauccgaauuug............................................................. 2     1  Le1
...............................acccuguagauccgaauuug............................................................. 247   0  Le1
...............................acccAguagauccgaauuug............................................................. 3     1  Le1
...............................acGcuguagauccgaauuug............................................................. 1     1  Le1
...............................accUuguagauccgaauuug............................................................. 1     1  Le1
...............................acccuguagauccgaauuuA............................................................. 2     1  Le1
...............................acUcuguagauccgaauuug............................................................. 1     1  Le1
...............................Ccccuguagauccgaauuug............................................................. 3     1  Le1
...............................acccGguagauccgaauuug............................................................. 10    1  Le1
...............................acccCguagauccgaauuug............................................................. 2     1  Le1
...............................acccuguagauccgGauuug............................................................. 1     1  Le1
...............................acccuguagauccgaauGugu............................................................ 2     1  Le1
```

```
                              Mature                        Star
ggcacgcgcuuuucuuaggugcuuuacaucuacccuguagauccgaauuugucuaggauucuaaccacaaauucgguucuagagagguuuaugugaugcauacgaaacgacc
...............................acccGguagauccgaauuugu............................................................  105   1  Le1
...............................acccuguagaCccgaauuugu............................................................  1     1  Le1
...............................acccuguagauccgaauAugu............................................................  1     1  Le1
...............................acccuguagauccgaCuuugu............................................................  1     1  Le1
...............................Ucccuguagauccgaauuugu............................................................  7     1  Le1
...............................acccuguagGuccgaauuugu............................................................  1     1  Le1
...............................acUcuguagauccgaauuugu............................................................  2     1  Le1
...............................acccuguagauccgaauuugC............................................................  77    1  Le1
...............................acccuguagauccgaauuugA............................................................  13    1  Le1
...............................accUuguagauccgaauuugu............................................................  8     1  Le1
...............................accAuguagauccgaauuugu............................................................  1     1  Le1
...............................acccuguagauccgCauuugu............................................................  1     1  Le1
...............................acccuguaAauccgaauuugu............................................................  3     1  Le1
...............................acGcuguagauccgaauuugu............................................................  2     1  Le1
...............................acccuguagauccgaauuuAu............................................................  1     1  Le1
...............................acccAguagauccgaauuugu............................................................  20    1  Le1
...............................acccuguagaucAgaauuugu............................................................  3     1  Le1
...............................aAccuguagauccgaauuugu............................................................  4     1  Le1
...............................acccuguagauUcgaauuugu............................................................  2     1  Le1
...............................acccugGagauccgaauuugu............................................................  2     1  Le1
...............................acccCguagauccgaauuugu............................................................  13    1  Le1
...............................Ccccuguagauccgaauuugu............................................................  21    1  Le1
...............................acccuguaCauccgaauuugu............................................................  4     1  Le1
...............................Gcccuguagauccgaauuugu............................................................  2     1  Le1
...............................acccuguagauccgaUuuugu............................................................  5     1  Le1
...............................aUccuguagauccgaauuugu............................................................  1     1  Le1
...............................acccuguagCuccgaauuugu............................................................  15    1  Le1
...............................acccuguagaucUgaauuugu............................................................  1     1  Le1
...............................acccuguagauccgaauuuCu............................................................  2     1  Le1
...............................acccuUuagauccgaauuugu............................................................  1     1  Le1
...............................acccuguagauccgaaCuugu............................................................  1     1  Le1
...............................acccuguagauccgaauuugu............................................................  2139  0  Le1
...............................acccuguagauccgaauuuUu............................................................  5     1  Le1
...............................acccuguagUuccgaauuugu............................................................  3     1  Le1
...............................aGccuguagauccgaauuugu............................................................  1     1  Le1
...............................acAcuguagauccgaauuugu............................................................  6     1  Le1
...............................Ccccuguagauccgaauuuguc...........................................................  2     1  Le1
...............................acccuguagCuccgaauuuguc...........................................................  1     1  Le1
...............................acccuguagaucUgaauuuguc...........................................................  1     1  Le1
...............................acccuguagauccgaauuuguc...........................................................  82    0  Le1
...............................acccGguagauccgaauuuguc...........................................................  3     1  Le1
...............................acccuguagauccgaauuuguG...........................................................  4     1  Le1
...............................acccAguagauccgaauuuguc...........................................................  1     1  Le1
...............................acccuguagauccgaauuuguA...........................................................  51    1  Le1
...............................acGcuguagauccgaauuuguc...........................................................  1     1  Le1
...............................acAcuguagauccgaauuuguc...........................................................  1     1  Le1
...............................acccCguagauccgaauuuguc...........................................................  1     1  Le1
...............................acccuguagauccgaauuuCuc...........................................................  1     1  Le1
...............................acccuguagauccgaauuugucu..........................................................  1     0  Le1
................................cccuguagauccgaauuug.............................................................  3     0  Le1
................................cccuguagUuccgaauuug.............................................................  1     1  Le1
................................cccGguagauccgaauuugu............................................................  1     1  Le1
................................cccugAagauccgaauuugu............................................................  2     1  Le1
................................ccUuguagauccgaauuugu............................................................  1     1  Le1
................................cccuguagUuccgaauuugu............................................................  2     1  Le1
................................cccuUuagauccgaauuugu............................................................  3     1  Le1
................................cccuguagauccgaauuugu............................................................  39    0  Le1
................................cccuguagauccgaauuuUu............................................................  1     1  Le1
................................cccuguagauccgaauuugC............................................................  3     1  Le1
................................cccuguagauccAaauuugu............................................................  1     1  Le1
................................cccuguagauccgaauuGguc...........................................................  1     1  Le1
................................cccuguagauccgaauuuguA...........................................................  1     1  Le1
.................................ccuguagauccgaauuugu............................................................  4     0  Le1
..................................cuguagauccgaauuugu............................................................  2     0  Le1
...................................................................caaauucgguucuagUga...........................  1     1  Le1
...................................................................caaauucgguucuagagag..........................  2     0  Le1
...................................................................caaauucgguucuaUagagg.........................  1     1  Le1
...................................................................caaaAucgguucuagagagg.........................  1     1  Le1
...................................................................caaauucgguucuagagagg.........................  6     0  Le1
...................................................................caaauucgguucuagagaggu........................  4     0  Le1
```

```
                              Mature                 Star
ggcacgcgcuuuucuuaggugcuuuacaucuacccuguagauccgaauuugucuaggauucuaaccacaaauucgguucuagagagguuuaugugaugcauacgaaacgacc
...................................................................caaauucgguucuagagagguu.......................   24   0   Le1
...................................................................caaauucgguucuagagagguA.......................   1    1   Le1
...................................................................caaaGucgguucuagagagguu.......................   1    1   Le1
...................................................................cCaauucgguucuagagagguu.......................   1    1   Le1
...................................................................caaaAucgguucuagagagguu.......................   2    1   Le1
...................................................................caaauucgguucuagagagguuu......................   30   0   Le1
...................................................................caaUuucgguucuagagagguuu......................   1    1   Le1
...................................................................caaauucgguucuagagagguuC......................   2    1   Le1
...................................................................caaaGucgguucuagagagguuu......................   3    1   Le1
...................................................................caaauucgguucuagagagguuuaA....................   1    1   Le1
....................................................................aaauucgguucuagagag..........................   4    0   Le1
....................................................................aaaGucgguucuagagagg.........................   1    1   Le1
....................................................................aaauGcgguucuagagagg.........................   1    1   Le1
....................................................................aaauucgguucuagagagA.........................   6    1   Le1
....................................................................aaauucgguucuagagagU.........................   1    1   Le1
....................................................................aaCuucgguucuagagagg.........................   1    1   Le1
....................................................................Caauucgguucuagagagg.........................   1    1   Le1
....................................................................aaauucgguuUuagagagg.........................   1    1   Le1
....................................................................aaauucgguucuagagagg.........................   53   0   Le1
....................................................................aaauGcgguucuagagaggu........................   2    1   Le1
....................................................................aaauucgguucGagagaggu........................   1    1   Le1
....................................................................aaauucgguucuagagaggA........................   2    1   Le1
....................................................................Caauucgguucuagagaggu........................   1    1   Le1
....................................................................Uaauucgguucuagagaggu........................   1    1   Le1
....................................................................aaauCcgguucuagagaggu........................   1    1   Le1
....................................................................aaauucgguucuagagaggC........................   8    1   Le1
....................................................................aaauucgguucuagagaggu........................   62   0   Le1
....................................................................aaauucgguucuagagCgguu.......................   1    1   Le1
....................................................................aaauucgguCcuagagagguu.......................   1    1   Le1
....................................................................aaCuucgguucuagagagguu.......................   3    1   Le1
....................................................................aGauucgguucuagagagguu.......................   1    1   Le1
....................................................................Caauucgguucuagagagguu.......................   11   1   Le1
....................................................................Naauucgguucuagagagguu.......................   1    1   Le1
....................................................................aaauucgguucuagagagguu.......................   682  0   Le1
....................................................................aUauucgguucuagagagguu.......................   1    1   Le1
....................................................................aaauucgguucuagagagguC.......................   33   1   Le1
....................................................................aaauucgguucuGgagagguu.......................   1    1   Le1
....................................................................aaauucgguucuagCgagguu.......................   1    1   Le1
....................................................................aaauAcgguucuagagagguu.......................   1    1   Le1
....................................................................Uaauucgguucuagagagguu.......................   1    1   Le1
....................................................................aaauucgguucuagagaggGu.......................   1    1   Le1
....................................................................aaauucgguucuCgagagguu.......................   1    1   Le1
....................................................................aaauGcgguucuagagagguu.......................   19   1   Le1
....................................................................Gaauucgguucuagagagguu.......................   1    1   Le1
....................................................................aaaGucgguucuagagagguu.......................   5    1   Le1
....................................................................aaauucgguucuagagagguG.......................   1    1   Le1
....................................................................aaauucgguucuagagagguA.......................   10   1   Le1
....................................................................aaauucgguucuagGgagguu.......................   1    1   Le1
....................................................................aaauCcgguucuagagagguu.......................   7    1   Le1
....................................................................aaauucggAucuagagagguu.......................   1    1   Le1
....................................................................aaauucgguucuagagagguuu......................   99   0   Le1
....................................................................aaauucgguucuagagGgguuu......................   1    1   Le1
....................................................................aaauucgguuAuagagagguuu......................   1    1   Le1
....................................................................aaauucAguucuagagagguuu......................   2    1   Le1
....................................................................aaauucgguucuagagagguAu......................   1    1   Le1
....................................................................Naauucgguucuagagagguuu......................   1    1   Le1
....................................................................aaaGucgguucuagagagguuu......................   2    1   Le1
....................................................................aaauCcgguucuagagagguuu......................   2    1   Le1
....................................................................aaauucgguucuCgagagguuu......................   1    1   Le1
....................................................................aaauucgguucuagagagguuC......................   4    1   Le1
....................................................................aaauucUguucuagagagguuu......................   1    1   Le1
....................................................................Caauucgguucuagagagguuu......................   4    1   Le1
....................................................................aaauucgguucuagagagguuua.....................   1    0   Le1
....................................................................aaauucgguucuagagagguuuaA....................   1    1   Le1
.....................................................................aauucgguucuagagagguu.......................   3    0   Le1
......................................................................auucgguucuagagaggu........................   1    0   Le1

.............................cuacccuguagauccgaauuug.............................................................   1    0   Br1
.............................Auacccuguagauccgaauuugu............................................................   1    1   Br1
.............................cuacccuguagauccgaauuugu............................................................   2    0   Br1
```

```
                              Mature                                  Star
ggcacgcgcuuuucuuaggugcuuuacaucuacccuguagauccgaauuugucuaggauucuaaccacaaauucgguucuagagagguuuaugugaugcauacgaaacgacc
..............................uacccuguagauccgaau................................................................	10	0	Br1
..............................uacccuguagauccgaauA...............................................................	1	1	Br1
..............................uacccuguagauccgUauu...............................................................	1	1	Br1
..............................uacccuguagauccgaauu...............................................................	20	0	Br1
..............................uaUccuguagauccgaauu...............................................................	1	1	Br1
..............................Aacccuguagauccgaauu...............................................................	1	1	Br1
..............................uacccuguagauccgaauuu..............................................................	66	0	Br1
..............................uacUcuguagauccgaauuu..............................................................	1	1	Br1
..............................uaAccuguagauccgaauuu..............................................................	2	1	Br1
..............................uacccuguagauccgaauuug.............................................................	840	0	Br1
..............................uacccuguagauccgaauuuU.............................................................	1	1	Br1
..............................uaccGuguagauccgaauuug.............................................................	6	1	Br1
..............................uacccuguCgauccgaauuug.............................................................	3	1	Br1
..............................uacccuguagCuccgaauuug.............................................................	3	1	Br1
..............................uacccuguagauccgaauuuA.............................................................	3	1	Br1
..............................uacccuguagauccgaGuuug.............................................................	1	1	Br1
..............................Aacccuguagauccgaauuug.............................................................	1	1	Br1
..............................Nacccuguagauccgaauuug.............................................................	1	1	Br1
..............................uacccuguagauAcgaauuug.............................................................	1	1	Br1
..............................uaccUuguagauccgaauuug.............................................................	8	1	Br1
..............................uacUcuguagauccgaauuug.............................................................	2	1	Br1
..............................uacccuguagauccgUauuug.............................................................	2	1	Br1
..............................uacccuguagauccgaaCuug.............................................................	1	1	Br1
..............................uaAccuguagauccgaauuug.............................................................	10	1	Br1
..............................uacccuguaUauccgaauuug.............................................................	1	1	Br1
..............................uaUccuguagauccgaauuug.............................................................	1	1	Br1
..............................uacccuguagauccgaauuCg.............................................................	2	1	Br1
..............................uCcccuguagauccgaauuug.............................................................	4	1	Br1
..............................uacccuguagauccgaCuuug.............................................................	1	1	Br1
..............................uacccuguagaucUgaauuug.............................................................	1	1	Br1
..............................uacccuguagauccUaauuug.............................................................	1	1	Br1
..............................uacccuguagauccgaauuugu............................................................	8658	0	Br1
..............................uaccAuguagauccgaauuugu............................................................	2	1	Br1
..............................uacccuguagauccgaauuuCu............................................................	1	1	Br1
..............................uacccugGagauccgaauuugu............................................................	3	1	Br1
..............................uaAccuguagauccgaauuugu............................................................	69	1	Br1
..............................uacccuguagauccgaGuuugu............................................................	2	1	Br1
..............................uacccuguagauccgaauuuAu............................................................	3	1	Br1
..............................uacccCguagauccgaauuugu............................................................	3	1	Br1
..............................uacccuguagaucAgaauuugu............................................................	4	1	Br1
..............................uaccUuguagauccgaauuugu............................................................	96	1	Br1
..............................uCcccuguagauccgaauuugu............................................................	24	1	Br1
..............................uacccugCagauccgaauuugu............................................................	1	1	Br1
..............................uacccuguagauccgaUuuugu............................................................	4	1	Br1
..............................uacccuUuagauccgaauuugu............................................................	6	1	Br1
..............................uacccuguagauccgaauuugC............................................................	314	1	Br1
..............................uacccuguagauccgaCuuugu............................................................	14	1	Br1
..............................Nacccuguagauccgaauuugu............................................................	3	1	Br1
..............................uacccuguaCauccgaauuugu............................................................	1	1	Br1
..............................uacccuAuagauccgaauuugu............................................................	1	1	Br1
..............................Gacccuguagauccgaauuugu............................................................	1	1	Br1
..............................uacccuguagauccgaauuCgu............................................................	2	1	Br1
..............................Cacccuguagauccgaauuugu............................................................	12	1	Br1
..............................uacccuguaAauccgaauuugu............................................................	2	1	Br1
..............................uGcccuguagauccgaauuugu............................................................	3	1	Br1
..............................uacccuguagauccgaaCuugu............................................................	1	1	Br1
..............................uacccuguagauGcgaauuugu............................................................	1	1	Br1
..............................uacccuguagauUcgaauuugu............................................................	2	1	Br1
..............................uacccuguagCuccgaauuugu............................................................	29	1	Br1
..............................uacccuguagauccgaaAuugu............................................................	4	1	Br1
..............................uaUccuguagauccgaauuugu............................................................	10	1	Br1
..............................uacccuguagaucUgaauuugu............................................................	4	1	Br1
..............................uacccuguagauccgaauCugu............................................................	2	1	Br1
..............................uaGccuguagauccgaauuugu............................................................	14	1	Br1
..............................uacccuguGgauccgaauuugu............................................................	2	1	Br1
..............................uacccuguagauccgUauuugu............................................................	13	1	Br1
..............................uacccuguagauccgaauuugA............................................................	44	1	Br1
..............................uUcccuguagauccgaauuugu............................................................	3	1	Br1
..............................uacccuguUgauccgaauuugu............................................................	8	1	Br1
..............................uacccAguagauccgaauuugu............................................................	2	1	Br1
```

```
                              Mature                                            Star
ggcacgcgcuuuucuuaggugcuuuacaucuacccuguagauccgaauuugucuaggauucuaaccacaaauucgguucuagagagguuuaugugaugcauacgaaacgacc
..............................uacccuguaUauccgaauuugu............................................................   1     1   Br1
..............................uacccuguCgauccgaauuugu............................................................   68    1   Br1
..............................uacccuguagaGccgaauuugu............................................................   2     1   Br1
..............................uacccuguagauccgaauuGgu............................................................   12    1   Br1
..............................Aacccuguagauccgaauuugu............................................................   21    1   Br1
..............................uacccuguagauccgaauAugu............................................................   1     1   Br1
..............................uacGcuguagauccgaauuugu............................................................   1     1   Br1
..............................uacccuguagauccgGauuugu............................................................   3     1   Br1
..............................uacccuguagUuccgaauuugu............................................................   3     1   Br1
..............................uaccGuguagauccgaauuugu............................................................   70    1   Br1
..............................uacccugAagauccgaauuugu............................................................   1     1   Br1
..............................uacccuguagauccgaauuugG............................................................   3     1   Br1
..............................uacccuguagauccUaauuugu............................................................   8     1   Br1
..............................uacccuguagauccgaauGugu............................................................   4     1   Br1
..............................uacccuguagauccgCauuugu............................................................   4     1   Br1
..............................uacccuguagauAcgaauuugu............................................................   3     1   Br1
..............................uacccGguagauccgaauuugu............................................................   4     1   Br1
..............................uacccuguagauccgaauuuUu............................................................   3     1   Br1
..............................uacccuCuagauccgaauuugu............................................................   1     1   Br1
..............................uacUcuguagauccgaauuugu............................................................   34    1   Br1
..............................uacccuguagauccAaauuugu............................................................   2     1   Br1
..............................uacccuguagauccgaauuuguG...........................................................   17    1   Br1
..............................uacccuguagauccgaauuuguA...........................................................   318   1   Br1
..............................uacccuguagauccgaauuuguc...........................................................   2     0   Br1
..............................uacccuguagauccgaauuuguAu..........................................................   3     1   Br1
...............................acccuguagauccgaauu...............................................................   3     0   Br1
...............................acccGguagauccgaauu...............................................................   1     1   Br1
...............................Gcccuguagauccgaauu...............................................................   1     1   Br1
...............................acccCguagauccgaauuu..............................................................   1     1   Br1
...............................acccGguagauccgaauuu..............................................................   1     1   Br1
...............................acccuguagauccgaauuu..............................................................   17    0   Br1
...............................Ucccuguagauccgaauuug.............................................................   1     1   Br1
...............................Ncccuguagauccgaauuug.............................................................   1     1   Br1
...............................acccGguagauccgaauuug.............................................................   10    1   Br1
...............................acccuguagauccgaauuug.............................................................   231   0   Br1
...............................acccuguagauccgaauCug.............................................................   1     1   Br1
...............................acccuguagCuccgaauuug.............................................................   1     1   Br1
...............................acccAguagauccgaauuug.............................................................   3     1   Br1
...............................acccuguagUuccgaauuug.............................................................   1     1   Br1
...............................acAcuguagauccgaauuug.............................................................   2     1   Br1
...............................acccCguagauccgaauuug.............................................................   1     1   Br1
...............................acccuguagauccgaauuuA.............................................................   3     1   Br1
...............................Ccccuguagauccgaauuug.............................................................   4     1   Br1
...............................acccuguagUuccgaauuugu............................................................   1     1   Br1
...............................accUuguagauccgaauuugu............................................................   12    1   Br1
...............................acccuguagauccAaauuugu............................................................   1     1   Br1
...............................acccuguagauccgCauuugu............................................................   2     1   Br1
...............................acccCguagauccgaauuugu............................................................   24    1   Br1
...............................acccuguagauccUaauuugu............................................................   1     1   Br1
...............................acccAguagauccgaauuugu............................................................   25    1   Br1
...............................acccuguagauccgaauuuCu............................................................   1     1   Br1
...............................Ncccuguagauccgaauuugu............................................................   2     1   Br1
...............................acGcuguagauccgaauuugu............................................................   3     1   Br1
...............................acccuguUgauccgaauuugu............................................................   3     1   Br1
...............................aGccuguagauccgaauuugu............................................................   1     1   Br1
...............................acccuguagCuccgaauuugu............................................................   21    1   Br1
...............................acccuguaCauccgaauuugu............................................................   4     1   Br1
...............................acccuguagauccgaauuugC............................................................   154   1   Br1
...............................acccuguagauccgaaCuugu............................................................   2     1   Br1
...............................acccuguagauccgaaAuugu............................................................   1     1   Br1
...............................acccuguagaAccgaauuugu............................................................   2     1   Br1
...............................acccuguagauccgaCuuugu............................................................   3     1   Br1
...............................acccuguagauccgaauuugA............................................................   11    1   Br1
...............................acccuguagauUcgaauuugu............................................................   2     1   Br1
...............................acccuguagaucUgaauuugu............................................................   1     1   Br1
...............................acccuguagaucAgaauuugu............................................................   1     1   Br1
...............................Ccccuguagauccgaauuugu............................................................   38    1   Br1
...............................acUcuguagauccgaauuugu............................................................   2     1   Br1
...............................acccGguagauccgaauuugu............................................................   129   1   Br1
...............................acccuguGgauccgaauuugu............................................................   1     1   Br1
```

Mature Star

```
ggcacgcgcuuuucuuaggugcuuuacaucuacccuguagauccgaauuuguCuaggauucuaaccacaaauucgguucuagagagguuuaugugaugcauacgaaacgacc
...............................acccuguagauccgaauuCgu............................................................ 1 1 Br1
...............................acccuguagauccgaauuuUu............................................................ 5 1 Br1
...............................acccuguagauccgaauuGgu............................................................ 2 1 Br1
...............................acccuguaAauccgaauuugu............................................................ 3 1 Br1
...............................aAccuguagauccgaauuugu............................................................ 4 1 Br1
...............................acccuUuagauccgaauuugu............................................................ 1 1 Br1
...............................acccuguagauccgGauuugu............................................................ 2 1 Br1
...............................acAcuguagauccgaauuugu............................................................ 14 1 Br1
...............................acccuguagauccgaauAugu............................................................ 2 1 Br1
...............................Ucccuguagauccgaauuugu............................................................ 10 1 Br1
...............................Gcccuguagauccgaauuugu............................................................ 1 1 Br1
...............................acccugAagauccgaauuugu............................................................ 1 1 Br1
...............................acccuguagauccgaauuugu............................................................ 2663 0 Br1
...............................acccuguagaCccgaauuugu............................................................ 2 1 Br1
...............................aUccuguagauccgaauuugu............................................................ 1 1 Br1
...............................acccuguagauccgaUuuugu............................................................ 2 1 Br1
...............................accUuguagauccgaauuuguc........................................................... 1 1 Br1
...............................Ccccuguagauccgaauuuguc........................................................... 1 1 Br1
...............................acccCguagauccgaauuuguc........................................................... 2 1 Br1
...............................acccAguagauccgaauuuguc........................................................... 3 1 Br1
...............................aAccuguagauccgaauuuguc........................................................... 1 1 Br1
...............................acccuguagauccgaauuugCc........................................................... 5 1 Br1
...............................acccuguagauccgaauuuguG........................................................... 4 1 Br1
...............................acccGguagauccgaauuuguc........................................................... 4 1 Br1
...............................acccuguagauccgaauuuguc........................................................... 229 0 Br1
...............................acccuguagauccgaauuuguA........................................................... 62 1 Br1
...............................acccuguagauccgaauCuguc........................................................... 1 1 Br1
...............................acccuguagCuccgaauuuguc........................................................... 1 1 Br1
...............................acAcuguagauccgaauuuguc........................................................... 3 1 Br1
...............................acccuguagauccgaauuugucu.......................................................... 6 0 Br1
...............................acccuguagauccgaauuugucG.......................................................... 3 1 Br1
...............................acccugGagauccgaauuugucu.......................................................... 1 1 Br1
...............................acccuguagauccgaauuugucuaU........................................................ 1 1 Br1
................................cccuguagauccgaauuu.............................................................. 1 0 Br1
................................cccuguagauccAaauuug............................................................. 1 1 Br1
................................cccuUuagauccgaauuug............................................................. 1 1 Br1
................................cccuguagauccgaauuug............................................................. 8 0 Br1
................................cccuguagauccgaauuugC............................................................ 3 1 Br1
................................cccuguagCuccgaauuugu............................................................ 1 1 Br1
................................cccuUuagauccgaauuugu............................................................ 3 1 Br1
................................cccuguagauccgaauuuUu............................................................ 2 1 Br1
................................cccGguagauccgaauuugu............................................................ 1 1 Br1
................................ccUuguagauccgaauuugu............................................................ 1 1 Br1
................................cccAguagauccgaauuugu............................................................ 1 1 Br1
................................cccuguagauccgaUuuugu............................................................ 3 1 Br1
................................cccuguagauccgaauuugu............................................................ 57 0 Br1
................................cccuguagauccgaauuuguc........................................................... 8 0 Br1
................................cccuguagauccgaauuuguA........................................................... 4 1 Br1
.................................ccuguagauccgaauuugu............................................................ 6 0 Br1
..................................cuguagauccgaauuugu............................................................ 1 0 Br1
..................................cugGagauccgaauuugu............................................................ 1 1 Br1
..................................................................aAaaauucgguucuagagagguu....................... 1 1 Br1
...................................................................caaauucgguucuagaga........................... 10 0 Br1
...................................................................caaauucgguucuagagag.......................... 3 0 Br1
...................................................................caaauucgguucuagagaA.......................... 1 1 Br1
...................................................................caaauucgguucuagagagg......................... 13 0 Br1
...................................................................caaauucgguucuagagagA......................... 1 1 Br1
...................................................................caaauucgguucuagagagC......................... 1 1 Br1
...................................................................Aaaauucgguucuagagaggu........................ 1 1 Br1
...................................................................caaauucgguucuagagaggu........................ 2 0 Br1
...................................................................caaauucgguucuCgagaggu........................ 1 1 Br1
...................................................................caaauucgguucuagagagguC....................... 3 1 Br1
...................................................................caaAucgguucuagagagguu....................... 1 1 Br1
...................................................................caaaGucgguucuagagagguu....................... 1 1 Br1
...................................................................Naaauucgguucuagagagguu....................... 1 1 Br1
...................................................................caaauucgguucuagagagguu....................... 52 0 Br1
...................................................................caaUuucgguucuagagagguu....................... 1 1 Br1
...................................................................caaauucgguucuagagagguG....................... 1 1 Br1
...................................................................caaauucgguucuagagaUguuu...................... 1 1 Br1
...................................................................caaauucgguucuGgagagguuu...................... 2 1 Br1
```

Mature **Star**

ggcacgcgcuuuucuuaggugcuuuacaucuacccuguagauccgaauuugucuaggauucuaaccacaaauucgguucuagagagguuuaugugaugcauacgaaacgacc

....Uaaauucgguucuagagagguuu......................	2	1	Br1
....caCauucgguucuagagagguuu......................	1	1	Br1
....caaauucgguucuagagUgguuu......................	2	1	Br1
....caaauucgAuucuagagagguuu......................	1	1	Br1
....Aaaauucgguucuagagagguuu......................	6	1	Br1
....caaaGucgguucuagagagguuu......................	28	1	Br1
....caaauucgguucuagagagguuC......................	20	1	Br1
....caaaCucgguucuagagagguuu......................	5	1	Br1
....caaauucgguucuaUagagguuu......................	1	1	Br1
....caaauucgguucuagagagguuu......................	697	0	Br1
....caGauucgguucuagagagguuu......................	2	1	Br1
....caaUuucgguucuagagagguuu......................	5	1	Br1
....Naaauucgguucuagagagguuu......................	1	1	Br1
....caaauucgguucuagUgagguuu......................	3	1	Br1
....caaauucgguuUuagagagguuu......................	1	1	Br1
....caaauGcgguucuagagagguuu......................	1	1	Br1
....caaaAucgguucuagagagguuu......................	5	1	Br1
....caaauucgguucuagagagUuuu......................	1	1	Br1
....caaauucgguucuCgagagguuu......................	1	1	Br1
....caaauucgguucuagagagguuuU.....................	7	1	Br1
....caaauucgguucuagagagguuua.....................	6	0	Br1
....cCaauucgguucuagagagguuua.....................	1	1	Br1
....caaauucgguucuagagagguuuUu....................	1	1	Br1
....caaauucgguucuagagagguuuaA....................	1	1	Br1
....aaauucgguucuagag............................	3	0	Br1
....Caauucgguucuagagagg.........................	1	1	Br1
....aaauucgguucuagagA...........................	2	1	Br1
....aaaCucgguucuagagagg.........................	1	1	Br1
....aaauucgguucuagagagg.........................	13	0	Br1
....aaauGcgguucuagagagg.........................	1	1	Br1
....Caauucgguucuagagaggu........................	2	1	Br1
....aaauucgguucuagagaggu........................	41	0	Br1
....aaauGcgguucuagagaggu........................	1	1	Br1
....aaauucgguucuagagaggC........................	8	1	Br1
....aaauCcgguucuagagagguu.......................	5	1	Br1
....Naauucgguucuagagagguu.......................	1	1	Br1
....aaauucgguucuagagUgguu.......................	1	1	Br1
....aaauucgguucuagagagguA.......................	10	1	Br1
....aaaAucgguucuagagagguu.......................	2	1	Br1
....aaCuucgguucuagagagguu.......................	3	1	Br1
....aaauucggAucuagagagguu.......................	1	1	Br1
....aaaGucgguucuagagagguu.......................	3	1	Br1
....aaauucgguucuagagaUguu.......................	1	1	Br1
....aaauucgguucuagagagguC.......................	37	1	Br1
....aaauucgguucuagGgagguu.......................	1	1	Br1
....aaauGcgguucuagagagguu.......................	20	1	Br1
....aaauucgguucuagagagUuu.......................	1	1	Br1
....aaauAcgguucuagagagguu.......................	2	1	Br1
....aaauucUguucuagagagguu.......................	1	1	Br1
....Gaauucgguucuagagagguu.......................	1	1	Br1
....aaauucgguucuagagagguu.......................	555	0	Br1
....aaauucgguuAuagagagguu.......................	1	1	Br1
....Caauucgguucuagagagguu.......................	5	1	Br1
....aaauucgguucuagagGgguu.......................	3	1	Br1
....aCauucgguucuagagagguuu......................	2	1	Br1
....aaauucgguucuagagaUguuu......................	1	1	Br1
....aaUuucgguucuagagagguuu......................	1	1	Br1
....aaauucgguucuagagagguuu......................	785	0	Br1
....aaauucgguucuagagagguCu......................	1	1	Br1
....aaauucgguucuagagagguuC......................	13	1	Br1
....aaauucggAucuagagagguuu......................	1	1	Br1
....Uaauucgguucuagagagguuu......................	4	1	Br1
....aUauucgguucuagagagguuu......................	1	1	Br1
....aaauGcgguucuagagagguuu......................	25	1	Br1
....aaauucUguucuagagagguuu......................	1	1	Br1
....aaCuucgguucuagagagguuu......................	3	1	Br1
....aaauAcgguucuagagagguuu......................	5	1	Br1
....aaauucgguAcuagagagguuu......................	1	1	Br1
....aaauucgguucuagagUgguuu......................	2	1	Br1
....aaauCcgguucuagagagguuu......................	6	1	Br1

```
                              Mature                                Star
ggcacgcgcuuuucuuaggugcuuuacaucuacccuguagauccgaauuugucuaggauucuaaccacaaauucgguucuagagagguuuaugugaugcauacgaaacgacc
....................................................................aaauuUgguucuagagagguuu......................    1     1     Br1
....................................................................aaaGucgguucuagagagguuu......................    10    1     Br1
....................................................................Caauucgguucuagagagguuu......................    9     1     Br1
....................................................................aaGuucgguucuagagagguuu......................    1     1     Br1
....................................................................aaauucgguucuagagCgguuu......................    4     1     Br1
....................................................................aaauucgguucuagagagUuuu......................    2     1     Br1
....................................................................aaauucgguucuagagagguuuU.....................    15    1     Br1
....................................................................aaauucgguucuagagagguuua.....................    5     0     Br1
.....................................................................aauucgguucuagagagg.........................    1     0     Br1
.....................................................................aauucgguucuagagagguu.......................    1     0     Br1
.....................................................................Cauucgguucuagagagguuu......................    1     1     Br1
......................................................................auucgguucuagagagguuu......................    1     0     Br1
```

Provisional ID	:	Sc28yqQ_3523_1393
Score total	:	59.9
Score for star read(s)	:	3.9
Score for read counts	:	49.6
Score for mfe	:	1.8
Score for randfold	:	1.6
Score for cons. seed	:	3
Total read count	:	109
Mature read count	:	100
Loop read count	:	0
Star read count	:	9

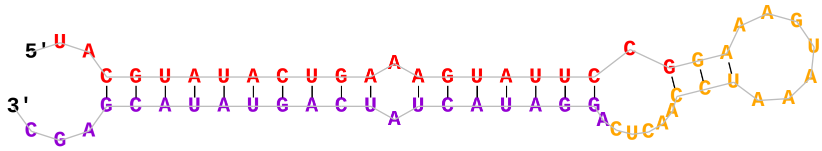

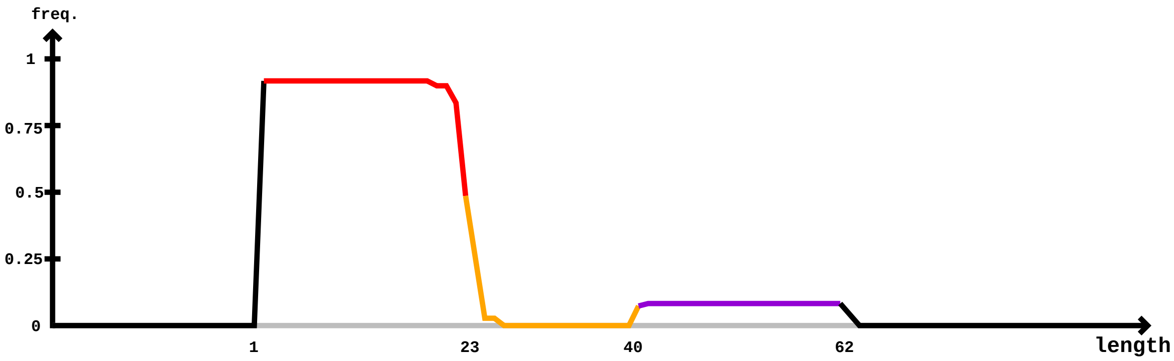

Mature Star

```
5'- cgagaaacagaguugucugcuacguauacugaaaguauuccggaaaguaaaauccaacucaggauacuaucaguauacgagcaggagucucugacacaaccacggaguuucac   -3'   obs
    cgagaaacagaguugucugcuacguauacugaaaguauuccggaaaguaaaauccaacucaggauacuaucaguauacgagcaggagucucugacacaaccacggaguuucac         exp
    .((((..(((((...((((((.((((((((((.(((((((.(((.......)))......))))))).))))))))))))))))...)))))......(....)..))))..      reads   mm   sample
    ....................uacguauacugaaaguauuc.........................................................................      1       0    Le5
    ....................uacgGauacugaaaguauuccg.......................................................................      3       1    Le5
    ....................uacguauacugaaaguauuccg.......................................................................      1       0    Le5

    ....................uacguauacugaaaguauucc........................................................................      1       0    Br1
    ............................................................aggauacAaucaguauacgagc...............................      1       1    Br1

    ....................uacguauCcugaaaguauucc........................................................................      1       1    He1
    ....................uacguauacugaaaguauucc........................................................................      2       0    He1
    ....................uacguauacugaaaguauuccg.......................................................................      1       0    He1

    ....................uacguauacugaaGguau...........................................................................      2       1    Ta1
    ....................uacguauacugaaaguauuc.........................................................................      6       0    Ta1
    ....................uacguaGacugaaaguauucc........................................................................      5       1    Ta1
    ....................uacguauacugaaaguauucc........................................................................      27      0    Ta1
    ....................uacgGauacugaaaguauucc........................................................................      1       1    Ta1
    ....................uacgCauacugaaaguauucc........................................................................      1       1    Ta1
    ....................uacgAauacugaaaguauuccg.......................................................................      1       1    Ta1
    ....................uacgGauacugaaaguauuccg.......................................................................      1       1    Ta1
    ....................uacguauacugaaaguauuccg.......................................................................      34      0    Ta1
    ....................uacguauacugaaaguauuccgg......................................................................      9       0    Ta1
    ....................uacguauacugaaaguauuccggaC....................................................................      3       1    Ta1
    ............................................................aggauacuaucaguauacgagc...............................      7       0    Ta1
    .............................................................ggauacAaucaguauacgagc...............................      1       1    Ta1
```

Provisional ID	:	Sc28yqQ_4222_1654
Score total	:	4.7
Score for star read(s)	:	-1.3
Score for read counts	:	0
Score for mfe	:	1.4
Score for randfold	:	1.6
Score for cons. seed	:	3
Total read count	:	114
Mature read count	:	114
Loop read count	:	0
Star read count	:	0

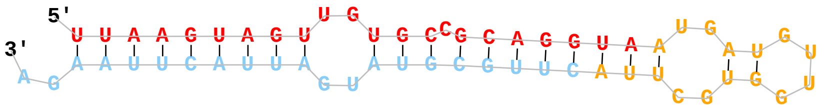

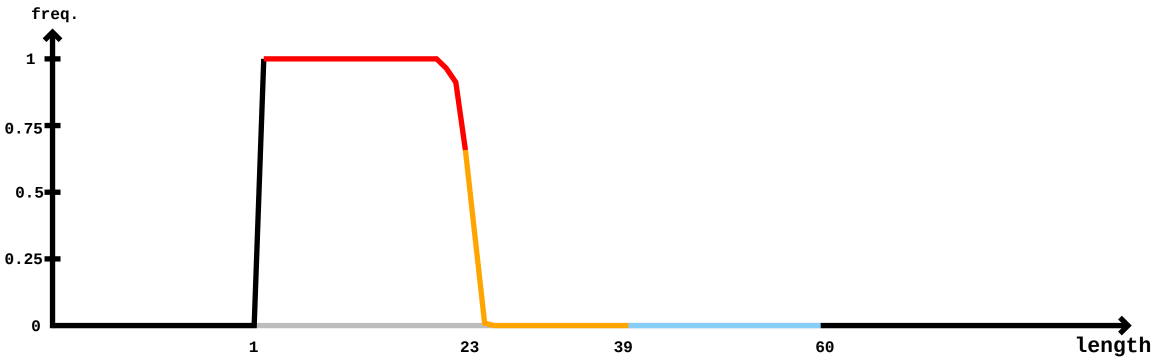

Mature   Star

```
5'- uuuuauugacguuuugccuauuaaguaguugugccgcaggua augauguuggugcuuacuugcguaugauuacuuaagaggccaaacgaaucaccaucucuacaguacua  -3'   exp
    .........(((((.((((.(((((((((..(((.((((((((..((....))..)))))))))))..))))))))).)))).))))).....................     reads   mm   sample
    ....................uuaaUuaguugugccgcagg......................................................................     1       1    Ch1
    ....................uuaaguaguugugccgcagg......................................................................     4       0    Ch1
    ....................uuaaguaguugugccgcaggA.....................................................................     1       1    Ch1
    ....................uuaaguaguugugccgcaggC.....................................................................     1       1    Ch1
    ....................uuaaguaguugugccgcaggu.....................................................................     9       0    Ch1
    ....................uuGaguaguugugccgcaggu.....................................................................     1       1    Ch1
    ....................uuaaguaguGgugccgcaggu.....................................................................     1       1    Ch1
    ....................uuaaUuaguugugccgcaggu.....................................................................     3       1    Ch1
    ....................uuaaguaguugugccgcagguU....................................................................     1       1    Ch1
    ....................uuaUguaguugugccgcaggua....................................................................     1       1    Ch1
    ....................uuaaguaguAgugccgcaggua....................................................................     1       1    Ch1
    ....................uuaaUuaguugugccgcaggua....................................................................     2       1    Ch1
    ....................uuaaguaguugugccgcaggua....................................................................     20      0    Ch1
    ....................uuaaguaguGgugccgcaggua....................................................................     1       1    Ch1
    ....................uuaaguaguugugccgcagguaU...................................................................     2       1    Ch1
    ....................uuaaguaguugugccgcagguaa...................................................................     6       0    Ch1

    ....................uuaaguaguugugccgcag.......................................................................     1       0    Bl1
    ....................uuaaguaguugugccgcagg......................................................................     1       0    Bl1
    ....................uuaaguaguugugccgcaggu.....................................................................     1       0    Bl1
    ....................uuaaguaguugugccgcaggC.....................................................................     1       1    Bl1
    ....................uuaaguaguugugccgcaggua....................................................................     2       0    Bl1
    ....................uuaaguaguugugccgcagguaa...................................................................     2       0    Bl1
    ....................uuaaguaguugugccgcagguaaA..................................................................     1       1    Bl1

    ....................uuaaguaguugugccgcag.......................................................................     2       0    Ta1
    ....................uuaaguaguugugccgcaggua....................................................................     4       0    Ta1
    ....................uuaaguaguugugccgcagguaa...................................................................     1       0    Ta1

    ....................uuaaguaguugugccgcag.......................................................................     1       0    Le5
    ....................uuaaguaguugugccgcaggA.....................................................................     1       1    Le5
    ....................uuaaguaguugugccgcaggu.....................................................................     6       0    Le5
    ....................uuaaguaguGgugccgcaggu.....................................................................     1       1    Le5
    ....................uuaaguaguugugccgcaggua....................................................................     17      0    Le5
```

```
                    Mature                                  Star
uuuuauugacguuuugccuauuaaguaguugugccgcagguaaugauguuggugcuuacuugcguaugauuacuuaagaggccaaacgaaucaccaucucuacaguacua
....................uuaaUuaguugugccgcaggua....................................................................    1    1    Le5
....................uuaaAuaguugugccgcaggua....................................................................    1    1    Le5
....................uuaaguaguugugccgcagUua....................................................................    1    1    Le5
....................uuaaguaguugugccgcagguaa...................................................................    2    0    Le5
....................uuaaguaguugugccgcagguaU...................................................................    1    1    Le5

....................uuaaguaguAgugccgcaggu.....................................................................    1    1    Le1
....................uuaaguaguugugccgcaggA.....................................................................    2    1    Le1
....................uuaaguaguugugccgcaggua....................................................................    2    0    Le1
....................uuaaguaguGgugccgcaggua....................................................................    2    1    Le1
....................uuaaguaguugugccgcagguaU...................................................................    1    1    Le1
....................uuaaguaguugugccgcagguaa...................................................................    3    0    Le1
```

Provisional ID	:	Sc28yqQ_4222_1736
Score total	:	4703.8
Score for star read(s)	:	3.9
Score for read counts	:	4693.6
Score for mfe	:	1.8
Score for randfold	:	1.6
Score for cons. seed	:	3
Total read count	:	9218
Mature read count	:	8900
Loop read count	:	0
Star read count	:	318

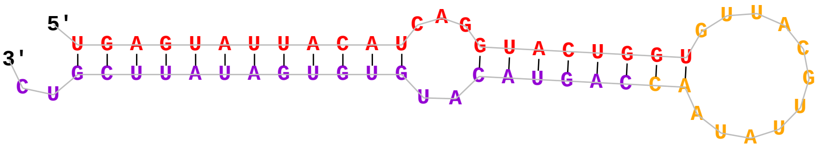

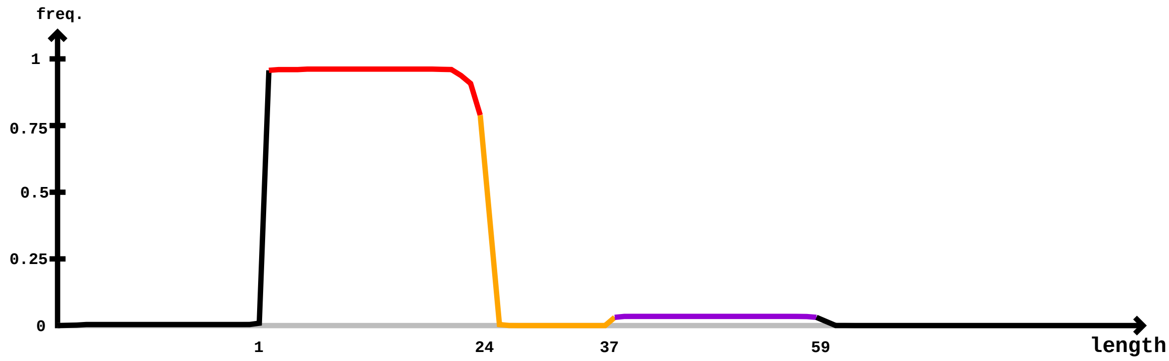


```
                        Mature                               Star
5'- aagggauagcauucagugaaugaguauuacaucagguacugguguuacguuauaaccaguacaugugugauauucgucuacuguacgcuacaugcuucgguugguaucuug   -3'   obs
    aagggauagcauucagugaaugaguauuacaucagguacugguguuacguuauaaccaguacaugugugauauucgucuacuguacgcuacaugcuucgguugguaucuug         exp
    ..((((((.((..(((((.(((((((((((((...((((((((...........))))))))..))))))))))))).)))))...((.....))......)).)))))).   reads   mm   sample
    ....................ugaguauuacaucagguacu.......................................................................   9       0    Ta1
    ....................ugagGauuacaucagguacu.......................................................................   1       1    Ta1
    ....................ugagAauuacaucagguacugg.....................................................................   1       1    Ta1
    ....................ugaguauuacaucagguacugg.....................................................................   10      0    Ta1
    ....................ugagGauuacaucagguacugg.....................................................................   1       1    Ta1
    ....................ugagGauuacaucagguacuggu....................................................................   6       1    Ta1
    ....................Agaguauuacaucagguacuggu....................................................................   1       1    Ta1
    ....................ugaguauuacaucagguacuggC....................................................................   3       1    Ta1
    ....................ugagAauuacaucagguacuggu....................................................................   2       1    Ta1
    ....................ugaguauuCcaucagguacuggu....................................................................   1       1    Ta1
    ....................ugaguauuacaucagguacuggu....................................................................   79      0    Ta1
    ....................ugaguauuacaucagguacugguU...................................................................   15      1    Ta1
    ........................................................caguUcaugugugauauucguc.................................   1       1    Ta1
    ........................................................caguacaugugugauauucguc.................................   10      0    Ta1

    .agggauagcauucagugaa...........................................................................................   1       0    Ch1
    ..................aaugaguauuacaucagguacuggu....................................................................   1       0    Ch1
    ...................augaguauuacauUagguacu.......................................................................   1       1    Ch1
    ...................augaguauuacaucagguacugg.....................................................................   2       0    Ch1
    ...................augaguauuacaucagguacuggu....................................................................   1       0    Ch1
    ....................ugaguauuacaucagguacu.......................................................................   15      0    Ch1
    ....................ugaguauuacaucagguacug......................................................................   12      0    Ch1
    ....................ugaguauuCcaucagguacugg.....................................................................   1       1    Ch1
    ....................ugagAauuacaucagguacugg.....................................................................   1       1    Ch1
    ....................ugaguauCacaucagguacugg.....................................................................   1       1    Ch1
    ....................Agaguauuacaucagguacugg.....................................................................   1       1    Ch1
    ....................ugagGauuacaucagguacugg.....................................................................   6       1    Ch1
    ....................ugaguauuacaucagguacugU.....................................................................   3       1    Ch1
    ....................ugaguauuacaucagguacugg.....................................................................   81      0    Ch1
    ....................ugagAauuacaucagguacuggu....................................................................   3       1    Ch1
    ....................ugCguauuacaucagguacuggu....................................................................   1       1    Ch1
    ....................ugaguauuacaucagguacuggC....................................................................   55      1    Ch1
    ....................ugaguauuacaucaUguacuggu....................................................................   1       1    Ch1
```

Mature **Star**

```
aagggauagcauucagugaaugaguauuacaucagguacugguguuacguuauaaccaguacaugugugauauucgucuacuguacgcuacaugcuucgguugguaucuug
....................ugaUuauuacaucagguacuggu....................................................................  1    1   Ch1
....................Agaguauuacaucagguacuggu....................................................................  1    1   Ch1
....................ugaguauuacaucagguacuggA....................................................................  9    1   Ch1
....................ugaguauuCcaucagguacuggu....................................................................  2    1   Ch1
....................ugaguauuacaucagguacuggG....................................................................  2    1   Ch1
....................ugaguauuacaucagguacuggu....................................................................  332  0   Ch1
....................ugaguauuacaucagguacuCgu....................................................................  1    1   Ch1
....................ugagCauuacaucagguacuggu....................................................................  1    1   Ch1
....................ugagGauuacaucagguacuggu....................................................................  14   1   Ch1
....................ugaguauuacaucaggCacuggu....................................................................  1    1   Ch1
....................ugaguauuacaucagguUcuggu....................................................................  1    1   Ch1
....................ugaguauuacaucagguacugguU...................................................................  12   1   Ch1
....................ugaguauuacaucagguacuggAg...................................................................  1    1   Ch1
....................ugaguauuacaucagguacugguUu..................................................................  2    1   Ch1
....................ugaguauuacaucagguacugguUuu.................................................................  1    1   Ch1
.....................gaguUuuacaucagguacugg.....................................................................  1    1   Ch1
.....................gaguGuuacaucagguacuggu....................................................................  1    1   Ch1
.....................gaguauuacaucagguacuggu....................................................................  1    0   Ch1
........................uauuacaucagguacuggu....................................................................  1    0   Ch1
........................................................caguacaugugugauauucgu..................................  4    0   Ch1
........................................................caguacaugugugauauucguc.................................  14   0   Ch1
........................................................caguacaugugugCuauucguc.................................  1    1   Ch1
........................................................caguacaugugugauauucguU.................................  1    1   Ch1
........................................................caguacaugugugauauucgucu................................  1    0   Ch1
.........................................................aguacaugugugauauucguc.................................  1    0   Ch1
.........................................................aguacaugAgugauauucguc.................................  1    1   Ch1

aagggauagcauucagugaa...........................................................................................  1    0   Bl1
.agggauagcauucagugaa...........................................................................................  3    0   Bl1
...................augaUuauuacaucagguacugg.....................................................................  1    1   Bl1
...................Cugaguauuacaucagguacuggu....................................................................  1    1   Bl1
....................ugaguauuacaucagguacu.......................................................................  1    0   Bl1
....................ugaguauuacaucagguacug......................................................................  2    0   Bl1
....................ugaguauuacaucagguacugU.....................................................................  1    1   Bl1
....................ugaguauuacaucagguacugC.....................................................................  1    1   Bl1
....................ugaguauuacaucagguacugg.....................................................................  13   0   Bl1
....................ugaguauuacaucagguacuAgu....................................................................  1    1   Bl1
....................ugaguauuacaucagguUcuggu....................................................................  2    1   Bl1
....................ugaguauuacCucagguacuggu....................................................................  6    1   Bl1
....................ugaguauuUcaucagguacuggu....................................................................  2    1   Bl1
....................ugagAauuacaucagguacuggu....................................................................  6    1   Bl1
....................ugagGauuacaucagguacuggu....................................................................  29   1   Bl1
....................Cgaguauuacaucagguacuggu....................................................................  2    1   Bl1
....................ugaguauuacaucagguacGggu....................................................................  2    1   Bl1
....................ugaguauuCcaucagguacuggu....................................................................  6    1   Bl1
....................ugCguauuacaucagguacuggu....................................................................  3    1   Bl1
....................ugaguauuacaucagguacuggA....................................................................  8    1   Bl1
....................ugaguauuacaucagguaUuggu....................................................................  1    1   Bl1
....................ugaguauuacaucagguacuggu....................................................................  744  0   Bl1
....................uCaguauuacaucagguacuggu....................................................................  2    1   Bl1
....................Ngaguauuacaucagguacuggu....................................................................  1    1   Bl1
....................ugaguauuacaucagguacugUu....................................................................  2    1   Bl1
....................ugaguauuaAaucagguacuggu....................................................................  1    1   Bl1
....................ugagCauuacaucagguacuggu....................................................................  4    1   Bl1
....................ugaguauuacaucagguacuggC....................................................................  107  1   Bl1
....................ugaUuauuacaucagguacuggu....................................................................  1    1   Bl1
....................ugaguUuuacaucagguacuggu....................................................................  2    1   Bl1
....................ugaguauuacaucCgguacuggu....................................................................  1    1   Bl1
....................Agaguauuacaucagguacuggu....................................................................  2    1   Bl1
....................ugaguauuacaucGgguacuggu....................................................................  1    1   Bl1
....................ugaguauuacaucagguacugguC...................................................................  1    1   Bl1
....................ugaguauuacaucagguacugguU...................................................................  26   1   Bl1
....................ugaguauuacaucagguacugguUu..................................................................  2    1   Bl1
.....................gaguauuacaucagguacuggu....................................................................  4    0   Bl1
.....................gaguauuacaucagguacuggC....................................................................  1    1   Bl1
........................uauuacaucagguacuggC....................................................................  2    1   Bl1
........................uauuacaucagguacuggu....................................................................  2    0   Bl1
........................uauuacaucagguacugguU...................................................................  1    1   Bl1
........................................................cagCacaugugugauauucguc.................................  1    1   Bl1
........................................................Gaguacaugugugauauucguc.................................  1    1   Bl1
```

Mature **Star**

```
aagggauagcauucagugaaugaguauuacaucagguacugguguuacguuauaaccaguacaugugugauauucgucuacuguacgcuacaugcuucgguugguaucuug
........................................................caguGcaugugugauauucguc.................................  1    1  Bl1
........................................................caguacaugugugauauucgCc.................................  1    1  Bl1
........................................................Uaguacaugugugauauucguc.................................  1    1  Bl1
........................................................caguacaugugugauauucguU.................................  1    1  Bl1
........................................................caguacaugugugauauucguc.................................  69   0  Bl1
........................................................caguacaugAgugauauucguc.................................  1    1  Bl1
........................................................caguacaugugugauauucgucC................................  3    1  Bl1
........................................................caguacaugugugauauucgucu................................  3    0  Bl1
.........................................................aguacaugugugauauucguc.................................  6    0  Bl1
.........................................................aguaGaugugugauauucguc.................................  1    1  Bl1

aagggauaAcauucagugaa...........................................................................................  1    1  He1
aagggauagcauucagugaa...........................................................................................  13   0  He1
.agggauagcauucagugaa...........................................................................................  15   0  He1
.agggUuagcauucagugaa...........................................................................................  1    1  He1
...................augaUuauuacaucagguac........................................................................  1    1  He1
...................augaguauuacaucagguac........................................................................  1    0  He1
...................augaguauuacaucagguacG.......................................................................  2    1  He1
...................augaguauuacaucagguacu.......................................................................  3    0  He1
...................augaguauuacaucagguacug......................................................................  3    0  He1
...................augaguauuacaucagguacugU.....................................................................  1    1  He1
...................augaguauuacaucagguacugg.....................................................................  14   0  He1
...................Cugaguauuacaucagguacuggu....................................................................  1    1  He1
...................augaguauuacaucagguacuggC....................................................................  1    1  He1
...................augaguauuacaucagguacuggu....................................................................  8    0  He1
...................augaUuauuacaucagguacuggu....................................................................  1    1  He1
...................augaguauuacaucagguacuggA....................................................................  1    1  He1
....................ugagGauuacaucaggua.........................................................................  1    1  He1
....................ugaguauuacaucaggua.........................................................................  9    0  He1
....................ugagCauuacaucagguac........................................................................  1    1  He1
....................ugaguauuacaucagguac........................................................................  2    0  He1
....................ugagAauuacaucagguacu.......................................................................  2    1  He1
....................ugaguaGuacaucagguacu.......................................................................  1    1  He1
....................ugaguauuacaucagguacC.......................................................................  7    1  He1
....................ugaguauuacaucaUguacu.......................................................................  1    1  He1
....................ugaguauuacaucagguaGu.......................................................................  1    1  He1
....................ugagCauuacaucagguacu.......................................................................  1    1  He1
....................ugaUuauuacaucagguacu.......................................................................  1    1  He1
....................ugaguauuacaucagguacA.......................................................................  7    1  He1
....................ugagGauuacaucagguacu.......................................................................  11   1  He1
....................ugaguauuacCucagguacu.......................................................................  1    1  He1
....................ugaguauuacaucagguacu.......................................................................  131  0  He1
....................ugaguauuCcaucagguacu.......................................................................  3    1  He1
....................ugaguauuacaucaUguacug......................................................................  1    1  He1
....................ugagCauuacaucagguacug......................................................................  2    1  He1
....................ugaguauuacaucUgguacug......................................................................  1    1  He1
....................ugaguauuCcaucagguacug......................................................................  1    1  He1
....................ugaguauuacaucagguacug......................................................................  223  0  He1
....................ugagAauuacaucagguacug......................................................................  3    1  He1
....................ugaguauuacaucagguacCg......................................................................  2    1  He1
....................ugaguauuaAaucagguacug......................................................................  1    1  He1
....................ugaguauuacaucagguacuA......................................................................  1    1  He1
....................ugaguauuacaucagguacAg......................................................................  1    1  He1
....................ugagGauuacaucagguacug......................................................................  11   1  He1
....................ugaguauuacaucagguUcug......................................................................  1    1  He1
....................ugaguauuacaucGgguacug......................................................................  1    1  He1
....................Ngaguauuacaucagguacugg.....................................................................  1    1  He1
....................ugaguauuacaucagguacugU.....................................................................  55   1  He1
....................ugagGauuacaucagguacugg.....................................................................  30   1  He1
....................ugaguauCacaucagguacugg.....................................................................  1    1  He1
....................ugaUuauuacaucagguacugg.....................................................................  4    1  He1
....................ugaguauuacaCcagguacugg.....................................................................  2    1  He1
....................ugagAauuacaucagguacugg.....................................................................  6    1  He1
....................ugaguauuacaucagguacugC.....................................................................  24   1  He1
....................ugaguauuacaucagguacugg.....................................................................  714  0  He1
....................ugaguauuacaucagguacugA.....................................................................  13   1  He1
....................Agaguauuacaucagguacugg.....................................................................  1    1  He1
....................ugagCauuacaucagguacugg.....................................................................  6    1  He1
....................ugUguauuacaucagguacugg.....................................................................  2    1  He1
....................ugaguauuacaucaUguacugg.....................................................................  3    1  He1
```

```
                    Mature                          Star
aagggauagcauucagugaaugaguauuacaucagguacugguguuacguuauaaccaguacaugugugauauucgucuacuguacgcuacaugcuucgguugguaucuug
....................ugCguauuacaucagguacugg....................................................................    2      1     He1
....................ugaguauuCcaucagguacugg....................................................................    5      1     He1
....................ugaguauuacaucCgguacugg....................................................................    1      1     He1
....................ugaguUuuacaucagguacugg....................................................................    1      1     He1
....................ugaguauuacauAagguacugg....................................................................    1      1     He1
....................ugaguauuacCucagguacugg....................................................................    4      1     He1
....................Cgaguauuacaucagguacugg....................................................................    1      1     He1
....................ugaguauuacaucagguacugUu...................................................................    5      1     He1
....................uAaguauuacaucagguacuggu...................................................................    1      1     He1
....................ugaguauuCcaucagguacuggu...................................................................    23     1     He1
....................ugaguauuacaucUgguacuggu...................................................................    3      1     He1
....................ugaguauuaAaucagguacuggu...................................................................    4      1     He1
....................ugaguaAuacaucagguacuggu...................................................................    1      1     He1
....................ugaCuauuacaucagguacuggu...................................................................    1      1     He1
....................ugUguauuacaucagguacuggu...................................................................    1      1     He1
....................ugaUuauuacaucagguacuggu...................................................................    9      1     He1
....................ugaguauuacauAagguacuggu...................................................................    3      1     He1
....................ugaguauuacaucagguCcuggu...................................................................    2      1     He1
....................ugaguauuacauUagguacuggu...................................................................    2      1     He1
....................ugaguauuacaucCgguacuggu...................................................................    3      1     He1
....................ugaguauuacaucagguacugCu...................................................................    4      1     He1
....................ugaguauuacaucagguacuggu...................................................................    3436   0     He1
....................ugaguauuacaucagUuacuggu...................................................................    1      1     He1
....................ugaguauuacaucaUguacuggu...................................................................    3      1     He1
....................ugaguauuacaucagguaUuggu...................................................................    3      1     He1
....................ugaguaCuacaucagguacuggu...................................................................    1      1     He1
....................ugagCauuacaucagguacuggu...................................................................    22     1     He1
....................Agaguauuacaucagguacuggu...................................................................    6      1     He1
....................ugCguauuacaucagguacuggu...................................................................    16     1     He1
....................ugaguauuacaucaggAacuggu...................................................................    1      1     He1
....................ugaguauuaUaucagguacuggu...................................................................    2      1     He1
....................ugaguauuacaucagguacCggu...................................................................    1      1     He1
....................ugaguauuacaucagguacGggu...................................................................    1      1     He1
....................ugaguauuacaucagguacuggG...................................................................    15     1     He1
....................ugaguaGuacaucagguacuggu...................................................................    4      1     He1
....................ugaguUuuacaucagguacuggu...................................................................    2      1     He1
....................ugagAauuacaucagguacuggu...................................................................    32     1     He1
....................ugaguauuacaucagguacAggu...................................................................    1      1     He1
....................ugaguauuacaucGgguacuggu...................................................................    1      1     He1
....................ugaguauuacaucaggCacuggu...................................................................    2      1     He1
....................ugaguauuacaucagguacuggC...................................................................    1237   1     He1
....................ugagGauuacaucagguacuggu...................................................................    188    1     He1
....................ugaguauuacaucagguacuggA...................................................................    120    1     He1
....................ugaguauuacaucagCuacuggu...................................................................    1      1     He1
....................Cgaguauuacaucagguacuggu...................................................................    3      1     He1
....................ugaguauuacaucagguaGuggu...................................................................    3      1     He1
....................ugaguCuuacaucagguacuggu...................................................................    1      1     He1
....................ugaguauuacaucagguacuCgu...................................................................    1      1     He1
....................uCaguauuacaucagguacuggu...................................................................    2      1     He1
....................ugaguauuacCucagguacuggu...................................................................    8      1     He1
....................ugaguauuacaucagguacuAgu...................................................................    1      1     He1
....................ugaguauuacaucagguacugAu...................................................................    1      1     He1
....................ugaguauuacaucagguacugguC..................................................................    3      1     He1
....................ugaguauuacUucagguacuggug..................................................................    5      1     He1
....................ugaguauuacaucagguacugguU..................................................................    131    1     He1
....................ugaguauuacaucagguacuggug..................................................................    6      0     He1
....................ugaguauuacaucagguacuggAg..................................................................    10     1     He1
....................ugaguauuacaucagguacuggugA.................................................................    3      1     He1
....................ugaguauuacaucagguacuggugu.................................................................    2      0     He1
....................ugaguauuacaucagguacugguUu.................................................................    19     1     He1
....................ugaguauuacaucagguacugguCu.................................................................    2      1     He1
....................ugaguauuacaucagguacuggugAu................................................................    1      1     He1
.....................gaguauuacaucagguacu......................................................................    1      0     He1
.....................gaguauuacaucagguacugg....................................................................    1      0     He1
.....................gagGauuacaucagguacuggu...................................................................    1      1     He1
.....................gaCuauuacaucagguacuggu...................................................................    1      1     He1
.....................gaguauuacaucagguacuggu...................................................................    7      0     He1
.....................gaguauuacaucagguacuggC...................................................................    3      1     He1
........................uauuacaucagguacugg....................................................................    1      0     He1
........................uauuacaucagguacuggC...................................................................    4      1     He1
```

Mature Star

```
aagggauagcauucagugaaugaguauuacaucagguacugguguuacguuauaaccaguacaugugugauauucgucuacuguacgcuacaugcuucgguugguaucuug
........................uauuacaucagguacugCu....................................................................    1    1    He1
........................uauuacaucaggAacuggu....................................................................    1    1    He1
........................uauuacaucagguacuggu....................................................................    5    0    He1
........................................................caguacaugugugauauuc....................................    1    0    He1
........................................................caguacaugugugauauucg...................................    3    0    He1
........................................................caguacaugugugauauucgu..................................    16   0    He1
........................................................caguacaugugugauauucgC..................................    3    1    He1
........................................................Gaguacaugugugauauucgu..................................    1    1    He1
........................................................caguUcaugugugauauucguc.................................    1    1    He1
........................................................caguacaugugugUuauucguc.................................    1    1    He1
........................................................cagGacaugugugauauucguc.................................    1    1    He1
........................................................caguacaugAgugauauucguc.................................    8    1    He1
........................................................caguacaugugugauauucgCc.................................    10   1    He1
........................................................caguGcaugugugauauucguc.................................    3    1    He1
........................................................caguacaugugugauauucgGc.................................    1    1    He1
........................................................Uaguacaugugugauauucguc.................................    1    1    He1
........................................................caguacauguAugauauucguc.................................    1    1    He1
........................................................caguacaugugugauauucguc.................................    96   0    He1
........................................................caguacaugugugauauucgucu................................    6    0    He1
.........................................................aguUcaugugugauauucg...................................    1    1    He1
.........................................................aguacaugugugauauucgu..................................    1    0    He1
.........................................................aguacaugAgugauauucguc.................................    1    1    He1
.........................................................Cguacaugugugauauucguc.................................    1    1    He1
.........................................................aguacaugugugauauucguc.................................    5    0    He1
.........................................................aguacaugugugauauucgucC................................    1    1    He1
.........................................................aguacaugugugauauucgucu................................    3    0    He1
.........................................................aguacaugugugauauucgucA................................    1    1    He1
.........................................................aguacaugAgugauauucgucu................................    6    1    He1
.........................................................aguacaugugugauauucgucuaA..............................    2    1    He1
..........................................................guacaugugugauauucgucu................................    1    0    He1

....................ugaguauuacaucagguacu.......................................................................    1    0    Br1
....................ugaguauuacaucagguacugU.....................................................................    1    1    Br1
....................ugaguauuacaucagguacugg.....................................................................    2    0    Br1
....................ugaguauuacaucagguacuggC....................................................................    9    1    Br1
....................ugaguauuacaucagguacuggu....................................................................    21   0    Br1
....................ugagGauuacaucagguacuggu....................................................................    1    1    Br1
....................ugaguauuacaucagguacuggA....................................................................    1    1    Br1
....................ugaguauuacaucagguacugguU...................................................................    1    1    Br1
.....................gaguGuuacaucagguacuggu....................................................................    1    1    Br1
........................................................caguacaugugugauauucguc.................................    1    0    Br1

....................ugaguauuacaucagguac........................................................................    1    0    Le1
....................ugaguauuacaucagguacu.......................................................................    2    0    Le1
....................ugaguauuacaucagguacug......................................................................    4    0    Le1
....................Agaguauuacaucagguacugg.....................................................................    1    1    Le1
....................ugaguauuacaucCgguacugg.....................................................................    1    1    Le1
....................ugaguauuacaucagguacugg.....................................................................    17   0    Le1
....................ugaguauuacaucagguaUuggu....................................................................    1    1    Le1
....................Agaguauuacaucagguacuggu....................................................................    1    1    Le1
....................Cgaguauuacaucagguacuggu....................................................................    1    1    Le1
....................ugaguauuacaucagguacuggG....................................................................    1    1    Le1
....................ugaguauuaAaucagguacuggu....................................................................    1    1    Le1
....................ugaguauuacaucagguacuggC....................................................................    22   1    Le1
....................ugaguauuacaucagguacuggu....................................................................    95   0    Le1
....................ugagCauuacaucagguacuggu....................................................................    3    1    Le1
....................ugagGauuacaucagguacuggu....................................................................    7    1    Le1
....................ugaguauuacaucGgguacuggu....................................................................    1    1    Le1
....................ugaguauuacaucagguacuggug...................................................................    1    0    Le1
....................ugaguauuacaucagguacugguU...................................................................    2    1    Le1
.....................gaguauuacaucagguacuggC....................................................................    1    1    Le1
........................................................caguacaugugugauauucgu..................................    1    0    Le1
........................................................caguacaugugugauauucguc.................................    3    0    Le1
.........................................................aguacaugugugauauucguc.................................    1    0    Le1

aagggauagcauucagugaa...........................................................................................    1    0    Le5
...................augaguauuacaucagguacug......................................................................    1    0    Le5
...................augaguauuacaucagguacugg.....................................................................    1    0    Le5
....................ugaguauuacaucagguacC.......................................................................    1    1    Le5
....................ugaguauuacaucagguacu.......................................................................    3    0    Le5
```

```
                    Mature                            Star
aagggauagcauucagugaaugaguauuacaucagguacugguguuacguuauaaccaguacaugugugauauucgucuacuguacgcuacaugcuucgguugguaucuug
....................ugaguauuacaucagguacug......................................................................   6     0     Le5
....................ugagGauuacaucagguacugg.....................................................................   3     1     Le5
....................ugaguauuacaucagguacugC.....................................................................   1     1     Le5
....................ugaguauuacaucagguacugg.....................................................................   47    0     Le5
....................Ggaguauuacaucagguacugg.....................................................................   1     1     Le5
....................ugagCauuacaucagguacugg.....................................................................   1     1     Le5
....................ugaguauuacaucagguacugU.....................................................................   3     1     Le5
....................uAaguauuacaucagguacugg.....................................................................   1     1     Le5
....................ugaguauuacaucagguacuggu....................................................................   148   0     Le5
....................ugaguauuCcaucagguacuggu....................................................................   1     1     Le5
....................ugaguauuacaucagguacuggA....................................................................   2     1     Le5
....................ugaguauuacaucagguacCggu....................................................................   1     1     Le5
....................ugagGauuacaucagguacuggu....................................................................   9     1     Le5
....................ugagAauuacaucagguacuggu....................................................................   1     1     Le5
....................Ngaguauuacaucagguacuggu....................................................................   1     1     Le5
....................ugaguauuacaucagguacuggC....................................................................   28    1     Le5
....................Agaguauuacaucagguacuggu....................................................................   1     1     Le5
....................ugCguauuacaucagguacuggu....................................................................   2     1     Le5
....................ugagCauuacaucagguacuggu....................................................................   1     1     Le5
....................ugaguauuacaucagguacugguU...................................................................   5     1     Le5
....................ugaguauuacaucagguacuggug...................................................................   1     0     Le5
....................ugaguauuacaucagguacuggAg...................................................................   1     1     Le5
....................ugaguauuacaucagguacugguCu..................................................................   1     1     Le5
....................ugaguauuacaucagguacuggugA..................................................................   1     1     Le5
....................ugaguauuacaucagguacugguUu..................................................................   1     1     Le5
.....................gaguauuacaucagguacugg.....................................................................   1     0     Le5
........................................................caguacaugugugauauucgC..................................   1     1     Le5
........................................................caguacaugugugauauucgu..................................   1     0     Le5
........................................................caguacaugAgugauauucguc.................................   2     1     Le5
........................................................caguacaugugugauauucguc.................................   8     0     Le5
........................................................caguacaugugugauauucguUu................................   1     1     Le5
```

Provisional ID	:	Sc28yqQ_4222_2506
Score total	:	4.7
Score for star read(s)	:	-1.3
Score for read counts	:	0
Score for mfe	:	1.4
Score for randfold	:	1.6
Score for cons. seed	:	3
Total read count	:	13396
Mature read count	:	13396
Loop read count	:	0
Star read count	:	0

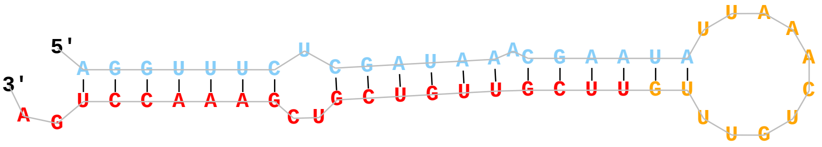

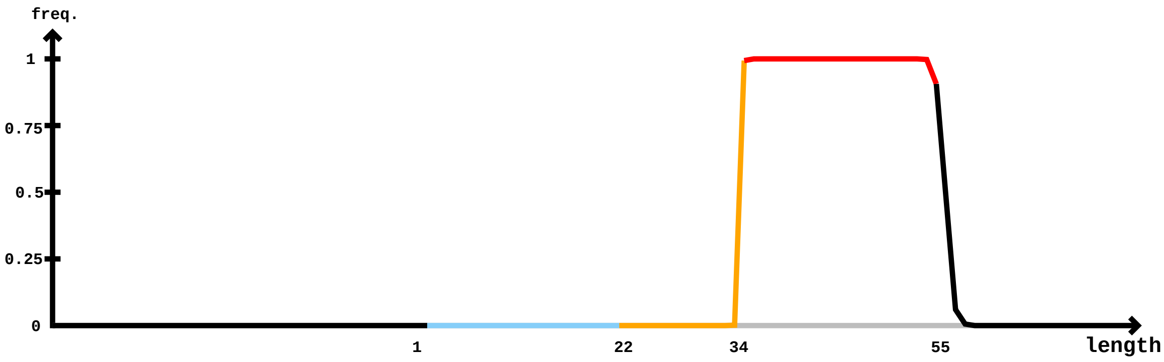


```
                                      Star                       Mature
5'- caucucuucacguuaauaauagagggagaaguugcccagguuucucgauaaacgaauauuaaacuguuuguucguugucgucgaaaccugaacaaaacucccuggacccuc  -3'   exp
    ......................((((((...(((..((((((((.((((((.((((((..........))))))))))))..))))))))..)))..))))))........   reads   mm   sample
    .....................................................................guucguugucgucgaaaccug.....................   4       0    Br1
    .....................................................................Cuucguugucgucgaaaccuga....................   2       1    Br1
    .....................................................................guucguugucgucgaaaccuga....................   7       0    Br1
    .....................................................................Cuucguugucgucgaaaccugaa...................   1       1    Br1
    .....................................................................guucguugucgucgaaaccugaa...................   1       0    Br1
    ......................................................................uucguugucgucgaaaccC......................   4       1    Br1
    ......................................................................uucguugucgucgaaaccA......................   4       1    Br1
    ......................................................................uucguugucgucgaaaccu......................   15      0    Br1
    ......................................................................uucUuugucgucgaaaccug.....................   3       1    Br1
    ......................................................................uucguugucgucgaaaccuU.....................   2       1    Br1
    ......................................................................uucguugucgCcgaaaccug.....................   1       1    Br1
    ......................................................................uucguugucgucgaaaccAg.....................   4       1    Br1
    ......................................................................uucguugucguGgaaaccug.....................   1       1    Br1
    ......................................................................uucguuguAgucgaaaccug.....................   1       1    Br1
    ......................................................................uuUguugucgucgaaaccug.....................   2       1    Br1
    ......................................................................uucguugucAucgaaaccug.....................   2       1    Br1
    ......................................................................uucguugucgucgaaaccCg.....................   20      1    Br1
    ......................................................................Cucguugucgucgaaaccug.....................   2       1    Br1
    ......................................................................uucguugucgucgCaaccug.....................   3       1    Br1
    ......................................................................Aucguugucgucgaaaccug.....................   4       1    Br1
    ......................................................................uAcguugucgucgaaaccug.....................   1       1    Br1
    ......................................................................uucguugucgucgaaaccug.....................   748     0    Br1
    ......................................................................uucgGugucgucgaaaccug.....................   30      1    Br1
    ......................................................................uucguugucgucgaUaccug.....................   1       1    Br1
    ......................................................................uucguugucgucgaaaAcug.....................   1       1    Br1
    ......................................................................uucguugucgucgaaaccGg.....................   1       1    Br1
    ......................................................................uucguugucgucgaaaccuA.....................   12      1    Br1
    ......................................................................uucguuAucgucgaaaccug.....................   1       1    Br1
    ......................................................................uucguuguUgucgaaaccug.....................   2       1    Br1
    ......................................................................uucgCugucgucgaaaccug.....................   2       1    Br1
    ......................................................................uuAguugucgucgaaaccug.....................   6       1    Br1
    ......................................................................Nucguugucgucgaaaccuga....................   5       1    Br1
    ......................................................................Gucguugucgucgaaaccuga....................   1       1    Br1
    ......................................................................uucgGugucgucgaaaccuga....................   208     1    Br1
```

Star Mature

```
caucucuucacguuaauaauagagggagaaguugcccagguuucucgauaaacgaauauuaaacuguuuguucguugucgucgaaaccugaacaaaacucccuggacccuc
......................................................................uucguugucgucgUaaccuga....................	1	1	Br1
......................................................................uucUuugucgucgaaaccuga....................	13	1	Br1
......................................................................uucguugucAucgaaaccuga....................	3	1	Br1
......................................................................uCcguugucgucgaaaccuga....................	1	1	Br1
......................................................................uucguugucgucgaaaccuga....................	4850	0	Br1
......................................................................uucguugucUucgaaaccuga....................	1	1	Br1
......................................................................uucguuUucgucgaaaccuga....................	10	1	Br1
......................................................................uucguugucgucgaaacAuga....................	1	1	Br1
......................................................................uucguugucgucgaaaccugU....................	14	1	Br1
......................................................................uucguugGcgucgaaaccuga....................	3	1	Br1
......................................................................uucguugucgucgaaaccAga....................	1	1	Br1
......................................................................uucguugucgucgaUaccuga....................	3	1	Br1
......................................................................uucguugucgucgCaaccuga....................	17	1	Br1
......................................................................Aucguugucgucgaaaccuga....................	10	1	Br1
......................................................................uucguugucgucUaaaccuga....................	2	1	Br1
......................................................................uucAuugucgucgaaaccuga....................	3	1	Br1
......................................................................uucguCgucgucgaaaccuga....................	1	1	Br1
......................................................................uucguugucgucgaaaccGga....................	5	1	Br1
......................................................................uucguugucgAcgaaaccuga....................	3	1	Br1
......................................................................uucguugucgucgaaaAcuga....................	3	1	Br1
......................................................................uucguuguAgucgaaaccuga....................	17	1	Br1
......................................................................uucguugucgucgaaUccuga....................	1	1	Br1
......................................................................uucguugucgucgaaaccugG....................	1	1	Br1
......................................................................uucguugucgucgaGaccuga....................	1	1	Br1
......................................................................Cucguugucgucgaaaccuga....................	11	1	Br1
......................................................................uuGguugucgucgaaaccuga....................	4	1	Br1
......................................................................uucgAugucgucgaaaccuga....................	45	1	Br1
......................................................................uucguugucgucAaaaccuga....................	2	1	Br1
......................................................................uucguugucgucgaaacUuga....................	5	1	Br1
......................................................................uucguugucguAgaaaccuga....................	2	1	Br1
......................................................................uuAguugucgucgaaaccuga....................	33	1	Br1
......................................................................uucguugucgucgaaaccuUa....................	11	1	Br1
......................................................................uucguugucgucgaaaccugC....................	6	1	Br1
......................................................................uucguugucgucgaaaccCga....................	3	1	Br1
......................................................................uucgCugucgucgaaaccuga....................	36	1	Br1
......................................................................uuUguugucgucgaaaccuga....................	13	1	Br1
......................................................................uucguuguUgucgaaaccuga....................	4	1	Br1
......................................................................uucguugucguGgaaaccuga....................	1	1	Br1
......................................................................uucguuCucgucgaaaccuga....................	2	1	Br1
......................................................................uucguugCcgucgaaaccuga....................	1	1	Br1
......................................................................uAcguugucgucgaaaccuga....................	1	1	Br1
......................................................................uucguugucgucgaaaccuCa....................	1	1	Br1
......................................................................uucguuguUgucgaaaccugaa...................	4	1	Br1
......................................................................uucguugucgucgaaaccugaU...................	52	1	Br1
......................................................................uucguugucgucgCaaccugaa...................	9	1	Br1
......................................................................uucgAugucgucgaaaccugaa...................	38	1	Br1
......................................................................uucguugucgucgaaacAugaa...................	1	1	Br1
......................................................................Cucguugucgucgaaaccugaa...................	1	1	Br1
......................................................................uucguugucgucgaaaccugaG...................	3	1	Br1
......................................................................uucguugucgucgaaaccGgaa...................	1	1	Br1
......................................................................Gucguugucgucgaaaccugaa...................	1	1	Br1
......................................................................uuAguugucgucgaaaccugaa...................	18	1	Br1
......................................................................uucguugucgucgUaaccugaa...................	2	1	Br1
......................................................................uCcguugucgucgaaaccugaa...................	1	1	Br1
......................................................................uucUuugucgucgaaaccugaa...................	8	1	Br1
......................................................................uucguugucgucgaaaAcugaa...................	2	1	Br1
......................................................................uucAuugucgucgaaaccugaa...................	1	1	Br1
......................................................................Aucguugucgucgaaaccugaa...................	5	1	Br1
......................................................................uucgGugucgucgaaaccugaa...................	126	1	Br1
......................................................................uucguugucgAcgaaaccugaa...................	2	1	Br1
......................................................................uucguuguGgucgaaaccugaa...................	1	1	Br1
......................................................................uucguugGcgucgaaaccugaa...................	2	1	Br1
......................................................................uucguugucgucgaaacUugaa...................	4	1	Br1
......................................................................uucguugucgucgaaaccugCa...................	1	1	Br1
......................................................................uucguugucgucgaaaccugaC...................	523	1	Br1
......................................................................Nucguugucgucgaaaccugaa...................	3	1	Br1
......................................................................uuUguugucgucgaaaccugaa...................	3	1	Br1
......................................................................uucguuAucgucgaaaccugaa...................	2	1	Br1
......................................................................uuGguugucgucgaaaccugaa...................	4	1	Br1
......................................................................uucguugucgucgaaaccuUaa...................	4	1	Br1
```

```
                                                     Star                           Mature
caucucuucacguuaauaauagagggagaaguugcccagguuucucgauaaacgaauauuaaacuguuuguucguugucgucgaaaccugaacaaaacucccuggacccuc
......................................................................uucguugucguAgaaaccugaa...................   2     1   Br1
......................................................................uucguugucgucgaaaccugaa...................   2862  0   Br1
......................................................................uucguugucgucgaaUccugaa...................   3     1   Br1
......................................................................uucguuUucgucgaaaccugaa...................   5     1   Br1
......................................................................uucguuguAgucgaaaccugaa...................   10    1   Br1
......................................................................uucgCugucgucgaaaccugaa...................   10    1   Br1
......................................................................uAcguugucgucgaaaccugaa...................   2     1   Br1
......................................................................uucguugucgucgaaaccugaCc..................   38    1   Br1
......................................................................uucguugucgucgaaaccugaaG..................   10    1   Br1
......................................................................uucgCugucgucgaaaccugaac..................   1     1   Br1
......................................................................uucgAugucgucgaaaccugaac..................   1     1   Br1
......................................................................uucguugucgucgaaaccugaaU..................   94    1   Br1
......................................................................uucguuguAgucgaaaccugaac..................   1     1   Br1
......................................................................uucgGugucgucgaaaccugaac..................   5     1   Br1
......................................................................uucguugucgucgaaaccugaac..................   49    0   Br1
......................................................................uuAguugucgucgaaaccugaac..................   1     1   Br1
......................................................................uucguugucgucgaaaccugaaA..................   376   1   Br1
......................................................................uucguugucguUgaaaccugaaca.................   7     1   Br1
......................................................................uucguuguAgucgaaaccugaaca.................   1     1   Br1
......................................................................uucguugucgucgaaaccugaaca.................   20    0   Br1
......................................................................uucguugucgucgaaaccugaacU.................   1     1   Br1
......................................................................uucguugucgucgaaaccugaaUa.................   2     1   Br1
......................................................................uucguugucgucgaaaccugaaAa.................   7     1   Br1
.......................................................................ucgGugucgucgaaaccug.....................   1     1   Br1
.......................................................................ucguugucgucgaaaccug.....................   2     0   Br1
.......................................................................ucguugucAucgaaaccuga....................   1     1   Br1
.......................................................................ucguugucgucgaaaccuga....................   26    0   Br1
.......................................................................ucgGugucgucgaaaccuga....................   2     1   Br1
.......................................................................ucguuAucgucgaaaccuga....................   1     1   Br1
.......................................................................uAguugucgucgaaaccuga....................   2     1   Br1
.......................................................................ucCuugucgucgaaaccuga....................   2     1   Br1
.......................................................................ucguCgucgucgaaaccuga....................   1     1   Br1
.......................................................................ucguGgucgucgaaaccuga....................   7     1   Br1
.......................................................................ucguugucgucUaaaccuga....................   1     1   Br1
.......................................................................uAguugucgucgaaaccugaa...................   1     1   Br1
.......................................................................ucguugucgucgaaaccugaC...................   1     1   Br1
.......................................................................ucgGugucgucgaaaccugaa...................   1     1   Br1
.......................................................................ucguCgucgucgaaaccugaa...................   2     1   Br1
.......................................................................ucguugucgucgaaaccugaa...................   9     0   Br1
.......................................................................ucguugucgucgaaaUcugaa...................   1     1   Br1
.......................................................................ucguugucgucgaaaccugaaU..................   1     1   Br1
........................................................................cguugucgucgaaaccuga....................   1     0   Br1
........................................................................cguugucgucgaaaccugaaA..................   1     1   Br1

......................................................................uucguugucgucgaaaccu......................   3     0   Le1
......................................................................uucguuAucgucgaaaccu......................   1     1   Le1
......................................................................uucUuugucgucgaaaccug.....................   2     1   Le1
......................................................................uucguugucgucgaaaccug.....................   94    0   Le1
......................................................................uucguugucUucgaaaccug.....................   1     1   Le1
......................................................................Aucguugucgucgaaaccug.....................   1     1   Le1
......................................................................uucguugucgucgaaaccuA.....................   2     1   Le1
......................................................................uuGguugucgucgaaaccug.....................   1     1   Le1
......................................................................uucguugucgucgaaaccCg.....................   1     1   Le1
......................................................................uuAguugucgucgaaaccug.....................   3     1   Le1
......................................................................uucguugucgucgaaacUug.....................   1     1   Le1
......................................................................uucguugucgucgaaaccuC.....................   1     1   Le1
......................................................................uucgGugucgucgaaaccug.....................   5     1   Le1
......................................................................uucguugucgucgaaaUcuga....................   2     1   Le1
......................................................................uuAguugucgucgaaaccuga....................   1     1   Le1
......................................................................uucgAugucgucgaaaccuga....................   2     1   Le1
......................................................................Nucguugucgucgaaaccuga....................   1     1   Le1
......................................................................uucgCugucgucgaaaccuga....................   2     1   Le1
......................................................................uucgGugucgucgaaaccuga....................   15    1   Le1
......................................................................uucguuguAgucgaaaccuga....................   2     1   Le1
......................................................................uuUguugucgucgaaaccuga....................   1     1   Le1
......................................................................uucguugucgucgaaaccuUa....................   1     1   Le1
......................................................................uucguuUucgucgaaaccuga....................   2     1   Le1
......................................................................Cucguugucgucgaaaccuga....................   1     1   Le1
......................................................................uucUuugucgucgaaaccuga....................   3     1   Le1
......................................................................uucguugucgCcgaaaccuga....................   1     1   Le1
```

Star Mature

```
caucucuucacguuaauaauagagggagaaguugcccagguuucucgauaaacgaauauuaaacuguuuguucguugucgucgaaaccugaacaaaacucccuggacccuc
......................................................................uucguugucgucgaaaccuga....................   282   0   Le1
......................................................................uucguugucgucgaaaccugU....................   2     1   Le1
......................................................................uucguugucgucgCaaccuga....................   3     1   Le1
......................................................................uucguugucgucgaaaccuUaa...................   1     1   Le1
......................................................................uucguugucgucgaaaccugaC...................   32    1   Le1
......................................................................uucguugucgucgaaaccugaa...................   113   0   Le1
......................................................................uucgAugucgucgaaaccugaa...................   2     1   Le1
......................................................................uucgGugucgucgaaaccugaa...................   5     1   Le1
......................................................................uucguuUucgucgaaaccugaa...................   1     1   Le1
......................................................................uucguugucgucgaaaccugaG...................   1     1   Le1
......................................................................uucguugucgucgaaaccugaCc..................   4     1   Le1
......................................................................uucgGugucgucgaaaccugaac..................   1     1   Le1
......................................................................uucguugucgucgaaaccugaaA..................   5     1   Le1
......................................................................uucguugucgucgaaaccugaac..................   3     0   Le1
......................................................................uucguugucgucgaaaccugaaU..................   3     1   Le1
......................................................................uucgAugucgucgaaaccugaaca.................   1     1   Le1
.......................................................................ucguugucgucgaaaccuga....................   2     0   Le1

.....................................................................Cuucguugucgucgaaaccuga....................   1     1   Le5
......................................................................uucguugucgucgaaaccG......................   1     1   Le5
......................................................................uucguugucgucgaaaccC......................   1     1   Le5
......................................................................uucguugucgucgaaaccug.....................   87    0   Le5
......................................................................uuGguugucgucgaaaccug.....................   1     1   Le5
......................................................................uucgGugucgucgaaaccug.....................   5     1   Le5
......................................................................uucguugucgucgaaCccug.....................   1     1   Le5
......................................................................uucgAugucgucgaaaccug.....................   2     1   Le5
......................................................................uuUguugucgucgaaaccug.....................   1     1   Le5
......................................................................uucguugucgucgaaaccAg.....................   1     1   Le5
......................................................................Aucguugucgucgaaaccug.....................   1     1   Le5
......................................................................uucguugucgucgaaaccuU.....................   1     1   Le5
......................................................................uucgCugucgucgaaaccug.....................   1     1   Le5
......................................................................uuAguugucgucgaaaccuga....................   4     1   Le5
......................................................................uucgGugucgucgaaaccuga....................   13    1   Le5
......................................................................uucgCugucgucgaaaccuga....................   3     1   Le5
......................................................................uucguugucUucgaaaccuga....................   1     1   Le5
......................................................................uucguugucgucgaaUccuga....................   1     1   Le5
......................................................................uucguuUucgucgaaaccuga....................   1     1   Le5
......................................................................uucguugucgucgCaaccuga....................   1     1   Le5
......................................................................uucguugucgucgaaaccuga....................   240   0   Le5
......................................................................uuUguugucgucgaaaccuga....................   1     1   Le5
......................................................................uucguugucgucgaaaccugaU...................   2     1   Le5
......................................................................uucguugucgucgaaaccugaC...................   27    1   Le5
......................................................................uuGguugucgucgaaaccugaa...................   1     1   Le5
......................................................................uucguugucgucgaaaccugaG...................   1     1   Le5
......................................................................uuAguugucgucgaaaccugaa...................   2     1   Le5
......................................................................uucgAugucgucgaaaccugaa...................   2     1   Le5
......................................................................uucgGugucgucgaaaccugaa...................   10    1   Le5
......................................................................uucguugucgucgaaaccugaa...................   126   0   Le5
......................................................................uucgCugucgucgaaaccugaa...................   2     1   Le5
......................................................................uucguugucgucgaaUccugaa...................   1     1   Le5
......................................................................uucguugucgucgaaaccugaac..................   1     0   Le5
......................................................................uucguugucgucgaaaccugaCc..................   2     1   Le5
......................................................................uucguugucgucgaaaccugaaA..................   23    1   Le5
......................................................................uucgGugucgucgaaaccugaac..................   1     1   Le5
......................................................................uucguugucgucgaaaccugaaU..................   2     1   Le5
......................................................................uucguugucgucgaaaccugaaca.................   1     0   Le5
......................................................................uucguugucgucgaaaccugaacC.................   1     1   Le5
......................................................................uucguugucguUgaaaccugaaca.................   4     1   Le5
......................................................................uucguugucgucgaaaccugaCca.................   1     1   Le5
......................................................................uucguugucgucgaaaccugaaUa.................   1     1   Le5
.......................................................................ucguugucCucgaaaccug.....................   1     1   Le5
.......................................................................ucguugucgucgaaaccuga....................   6     0   Le5

......................................................................uucguugucgucgaaaccug.....................   14    0   Ta1
......................................................................uucgGugucgucgaaaccug.....................   1     1   Ta1
......................................................................uucgGugucgucgaaaccuga....................   2     1   Ta1
......................................................................uucgAugucgucgaaaccuga....................   1     1   Ta1
......................................................................uucgCugucgucgaaaccuga....................   1     1   Ta1
......................................................................uuUguugucgucgaaaccuga....................   1     1   Ta1
......................................................................uucguugucgucgaaaccuga....................   43    0   Ta1
```

```
                                     Star                            Mature
caucucuucacguuaauaauagagggagaaguugcccagguuucucgauaaacgaauauuaaacuguuuguucguugucgucgaaaccugaacaaaacucccuggacccuc
.....................................................................uucguugucgucgaaaccugaC...................   9    1   Ta1
.....................................................................uucguugucgucgaaaccugaa...................   9    0   Ta1
.....................................................................uucgGugucgucgaaaccugaa...................   1    1   Ta1
.....................................................................uucguugucgucgaaaccugaaA..................   8    1   Ta1
.....................................................................uucguugucgucgaaaccugaaG..................   6    1   Ta1
.....................................................................uucguugucgucgaaaccugaCc..................   5    1   Ta1
.....................................................................uucguugucgucgaaaccugaaca.................   4    0   Ta1

.....................................................................uuAguugucgucgaaaccug.....................   2    1   Ch1
.....................................................................uucgAugucgucgaaaccug.....................   1    1   Ch1
.....................................................................uucgGugucgucgaaaccug.....................   2    1   Ch1
.....................................................................uucguugucguGgaaaccug.....................   1    1   Ch1
.....................................................................uucUuugucgucgaaaccug.....................   1    1   Ch1
.....................................................................uucguugucgucgaaaccug.....................   63   0   Ch1
.....................................................................uucgAugucgucgaaaccuga....................   1    1   Ch1
.....................................................................uucgGugucgucgaaaccuga....................   11   1   Ch1
.....................................................................Aucguugucgucgaaaccuga....................   1    1   Ch1
.....................................................................uuAguugucgucgaaaccuga....................   2    1   Ch1
.....................................................................uCcguugucgucgaaaccuga....................   1    1   Ch1
.....................................................................uucguCgucgucgaaaccuga....................   1    1   Ch1
.....................................................................uucgCugucgucgaaaccuga....................   1    1   Ch1
.....................................................................uucguugucgucgaaaccuga....................   312  0   Ch1
.....................................................................uucguuguUgucgaaaccuga....................   1    1   Ch1
.....................................................................uucguugGcgucgaaaccuga....................   1    1   Ch1
.....................................................................uucguugucguGgaaaccuga....................   1    1   Ch1
.....................................................................Gucguugucgucgaaaccuga....................   1    1   Ch1
.....................................................................uucguugCcgucgaaaccuga....................   1    1   Ch1
.....................................................................uucguugucgucgaaaccugaU...................   6    1   Ch1
.....................................................................uuAguugucgucgaaaccugaa...................   1    1   Ch1
.....................................................................uucguugucgucgaaUccugaa...................   1    1   Ch1
.....................................................................uucguugucgucgaaaccugaC...................   50   1   Ch1
.....................................................................uucgCugucgucgaaaccugaa...................   1    1   Ch1
.....................................................................uucgAugucgucgaaaccugaa...................   2    1   Ch1
.....................................................................uucguuUucgucgaaaccugaa...................   1    1   Ch1
.....................................................................uucgGugucgucgaaaccugaa...................   9    1   Ch1
.....................................................................Cucguugucgucgaaaccugaa...................   1    1   Ch1
.....................................................................uucguugucguAgaaaccugaa...................   1    1   Ch1
.....................................................................uucguugucgucgaaaccugaa...................   193  0   Ch1
.....................................................................uucguuguAgucgaaaccugaa...................   2    1   Ch1
.....................................................................uucguugucgucgaaaccugaac..................   1    0   Ch1
.....................................................................uucguugucgucgaaaccugaaA..................   36   1   Ch1
.....................................................................uucguugucgucgaaaccugaaU..................   8    1   Ch1
.....................................................................uucguugucguUgaaaccugaaca.................   5    1   Ch1
.....................................................................uucguugucgucgaaaccugaacC.................   1    1   Ch1
.....................................................................uucgGugucgucgaaaccugaaca.................   1    1   Ch1
.....................................................................uucguugucgucgaaaccugaaca.................   11   0   Ch1
......................................................................ucguugucgucgaaaccug.....................   1    0   Ch1
......................................................................ucguugucgucgaaaccuga....................   2    0   Ch1
......................................................................ucguugucgucgaaaccugaa...................   1    0   Ch1
......................................................................ucguugucgucgaaaccugaC...................   1    1   Ch1
......................................................................ucguugucgucgaaaccCgaa...................   1    1   Ch1
......................................................................ucguugucgucgaaaccuCaa...................   1    1   Ch1

....................................................................guucguugucgucgaaaccuga....................   2    0   He1
....................................................................guucguugucgucgaaaccugaa...................   1    0   He1
.....................................................................uucguugucgucgaaaccC......................   1    1   He1
.....................................................................uucguugucgucgaaaccu......................   2    0   He1
.....................................................................uucgGugucgucgaaaccug.....................   3    1   He1
.....................................................................uucguugucgucgaaaUcug.....................   2    1   He1
.....................................................................uucguugucgucgaaaccCg.....................   2    1   He1
.....................................................................uucgCugucgucgaaaccug.....................   1    1   He1
.....................................................................uuAguugucgucgaaaccug.....................   1    1   He1
.....................................................................uucguuUucgucgaaaccug.....................   2    1   He1
.....................................................................uucguugucgucgaaaccug.....................   52   0   He1
.....................................................................uuAguugucgucgaaaccuga....................   1    1   He1
.....................................................................uucguugucgucgaaacUuga....................   1    1   He1
.....................................................................uucguuAucgucgaaaccuga....................   1    1   He1
.....................................................................uucguugucgucgaaaccuga....................   293  0   He1
.....................................................................uucgAugucgucgaaaccuga....................   1    1   He1
.....................................................................uuGguugucgucgaaaccuga....................   3    1   He1
```

```
                                                         Star                                         Mature
caucucuucacguuaauaauagagggagaaguugcccagguuucucgauaaacgaauauuaaacuguuuguucguugucgucgaaaccugaacaaaacucccuggacccuc
......................................................................uucgGugucgucgaaaccuga....................    17    1    He1
......................................................................Gucguugucgucgaaaccuga....................    1     1    He1
......................................................................uucguugGcgucgaaaccuga....................    1     1    He1
......................................................................uucUuugucgucgaaaccuga....................    1     1    He1
......................................................................uucguugucgucgaaaccugC....................    1     1    He1
......................................................................Aucguugucgucgaaaccugaa...................    2     1    He1
......................................................................uuGguugucgucgaaaccugaa...................    1     1    He1
......................................................................uucgAugucgucgaaaccugaa...................    3     1    He1
......................................................................uucguugucgucgaaaccugaU...................    6     1    He1
......................................................................uucguugucgucgaaaccuUaa...................    1     1    He1
......................................................................uucgGugucgucgaaaccugaa...................    9     1    He1
......................................................................uucguugucgucgaaaccugaa...................    191   0    He1
......................................................................uucguugucgucgaaaccugaC...................    27    1    He1
......................................................................uucguugucgucgCaaccugaa...................    1     1    He1
......................................................................uucguugucgucgaaaccugUa...................    1     1    He1
......................................................................uucguuAucgucgaaaccugaa...................    1     1    He1
......................................................................uucguuUucgucgaaaccugaa...................    1     1    He1
......................................................................uuAguugucgucgaaaccugaa...................    1     1    He1
......................................................................uucguugucgucgaaaccAgaa...................    1     1    He1
......................................................................uucguugucgucgaaaccugaCc..................    3     1    He1
......................................................................uucguugucgucgaaaccugaac..................    2     0    He1
......................................................................uucguugucgucgaaaccugaaU..................    5     1    He1
......................................................................uucgAugucgucgaaaccugaac..................    1     1    He1
......................................................................uucguugucgucgaaaccugaaA..................    22    1    He1
......................................................................uucguugucgucgaaaccugaaG..................    4     1    He1
......................................................................uucguugucgucgaaaccugaaUa.................    2     1    He1
......................................................................uucguugucgucgaaaccugaaca.................    4     0    He1
......................................................................uucguugucgucgaaaccugaaAa.................    1     1    He1
.......................................................................ucgGugucgucgaaaccuga....................    1     1    He1
.......................................................................ucguugucgucgaaaccuga....................    3     0    He1
.......................................................................ucgAugucgucgaaaccuga....................    1     1    He1
.......................................................................ucguugucgucgaaaccugaa...................    1     0    He1

......................................................................uucguugucgucgaaaccug.....................    4     0    Bl1
......................................................................uucguugucgucgaaaccuga....................    6     0    Bl1
......................................................................uucguugucgucgaaaccugGa...................    1     1    Bl1
......................................................................uucguugucgucgCaaccugaa...................    1     1    Bl1
......................................................................uucguugucgucgaaaccugaa...................    8     0    Bl1
......................................................................uucguugucgucgaaaccugaaA..................    1     1    Bl1
......................................................................uucguugucgucgaaaccugaaAa.................    1     1    Bl1
```

Provisional ID	:	Sc28yqQ_4222_2508
Score total	:	4.7
Score for star read(s)	:	-1.3
Score for read counts	:	0
Score for mfe	:	1.4
Score for randfold	:	1.6
Score for cons. seed	:	3
Total read count	:	13396
Mature read count	:	13396
Loop read count	:	0
Star read count	:	0

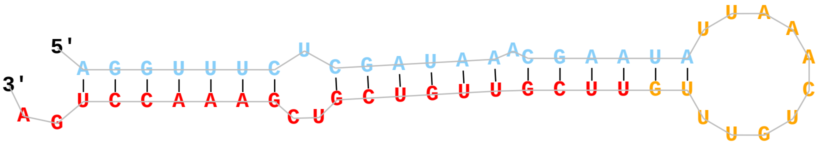

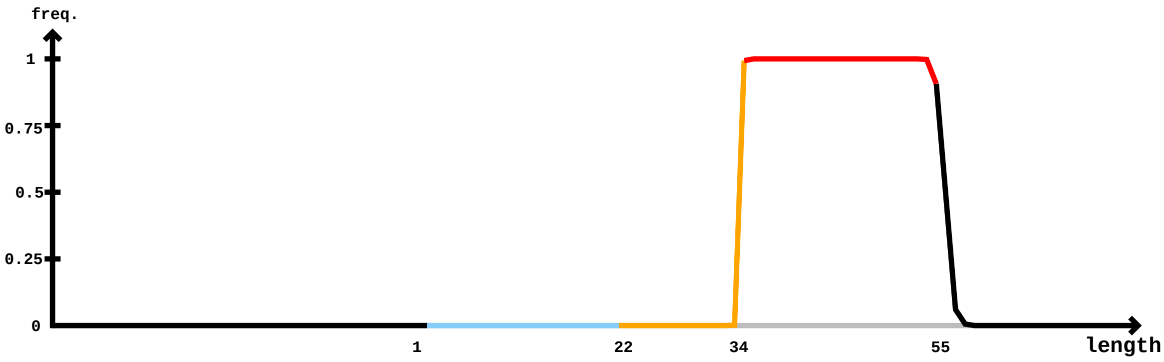


```
                                     Star                  Mature
5'- caucucuucacguuaauaauagagggagaaguugcccagguuucucgauaaacgaauauuaaacuguuuguucguugucgucgaaaccugaacaaaacucccuggacccuc  -3'   exp
    ......................((((((...(((..((((((((.((((((.((((((..........))))))))))))..))))))))..)))..))))))........  reads  mm  sample
    .....................................uucgAugucgucgaaaccug......................................................  1      1   Ch1
    .....................................uucguugucguGgaaaccug......................................................  1      1   Ch1
    .....................................uucUuugucgucgaaaccug......................................................  1      1   Ch1
    .....................................uucgGugucgucgaaaccug......................................................  2      1   Ch1
    .....................................uuAguugucgucgaaaccug......................................................  2      1   Ch1
    .....................................uucguugucgucgaaaccug......................................................  63     0   Ch1
    .....................................uucgGugucgucgaaaccuga.....................................................  11     1   Ch1
    .....................................uucguugGcgucgaaaccuga.....................................................  1      1   Ch1
    .....................................uCcguugucgucgaaaccuga.....................................................  1      1   Ch1
    .....................................uucguugucguGgaaaccuga.....................................................  1      1   Ch1
    .....................................uucguugCcgucgaaaccuga.....................................................  1      1   Ch1
    .....................................uucguCgucgucgaaaccuga.....................................................  1      1   Ch1
    .....................................Gucguugucgucgaaaccuga.....................................................  1      1   Ch1
    .....................................uuAguugucgucgaaaccuga.....................................................  2      1   Ch1
    .....................................uucguugucgucgaaaccuga.....................................................  312    0   Ch1
    .....................................uucguuguUgucgaaaccuga.....................................................  1      1   Ch1
    .....................................uucgAugucgucgaaaccuga.....................................................  1      1   Ch1
    .....................................uucgCugucgucgaaaccuga.....................................................  1      1   Ch1
    .....................................Aucguugucgucgaaaccuga.....................................................  1      1   Ch1
    .....................................uucgAugucgucgaaaccugaa....................................................  2      1   Ch1
    .....................................uucguugucguAgaaaccugaa....................................................  1      1   Ch1
    .....................................uucguugucgucgaaaccugaC....................................................  50     1   Ch1
    .....................................uuAguugucgucgaaaccugaa....................................................  1      1   Ch1
    .....................................uucguuUucgucgaaaccugaa....................................................  1      1   Ch1
    .....................................uucguugucgucgaaaccugaU....................................................  6      1   Ch1
    .....................................uucguugucgucgaaUccugaa....................................................  1      1   Ch1
    .....................................uucgCugucgucgaaaccugaa....................................................  1      1   Ch1
    .....................................uucgGugucgucgaaaccugaa....................................................  9      1   Ch1
    .....................................Cucguugucgucgaaaccugaa....................................................  1      1   Ch1
    .....................................uucguugucgucgaaaccugaa....................................................  193    0   Ch1
    .....................................uucguuguAgucgaaaccugaa....................................................  2      1   Ch1
    .....................................uucguugucgucgaaaccugaaU...................................................  8      1   Ch1
    .....................................uucguugucgucgaaaccugaaA...................................................  36     1   Ch1
    .....................................uucguugucgucgaaaccugaac...................................................  1      0   Ch1
```

Star Mature

caucucuucacguuaauaauagagggagaaguugcccagguuucucgauaaacgaauauuaaacuguuuguucguugucgucgaaaccugaacaaaacucccuggacccuc			
....uucguugucgucgaaaccugaaca.................	11	0	Ch1
....uucguugucguUgaaaccugaaca.................	5	1	Ch1
....uucguugucgucgaaaccugaacC.................	1	1	Ch1
....uucgGugucgucgaaaccugaaca.................	1	1	Ch1
....ucguugucgucgaaaccug.....................	1	0	Ch1
....ucguugucgucgaaaccuga....................	2	0	Ch1
....ucguugucgucgaaaccugaa...................	1	0	Ch1
....ucguugucgucgaaaccuCaa...................	1	1	Ch1
....ucguugucgucgaaaccugaC...................	1	1	Ch1
....ucguugucgucgaaaccCgaa...................	1	1	Ch1
....uucguugucgucgaaaccug.....................	4	0	Bl1
....uucguugucgucgaaaccuga....................	6	0	Bl1
....uucguugucgucgCaaccugaa...................	1	1	Bl1
....uucguugucgucgaaaccugGa...................	1	1	Bl1
....uucguugucgucgaaaccugaa...................	8	0	Bl1
....uucguugucgucgaaaccugaaA..................	1	1	Bl1
....uucguugucgucgaaaccugaaAa.................	1	1	Bl1
....guucguugucgucgaaaccuga....................	2	0	He1
....guucguugucgucgaaaccugaa...................	1	0	He1
....uucguugucgucgaaaccC......................	1	1	He1
....uucguugucgucgaaaccu......................	2	0	He1
....uucguugucgucgaaaUcug.....................	2	1	He1
....uucgGugucgucgaaaccug.....................	3	1	He1
....uucguugucgucgaaaccCg.....................	2	1	He1
....uucguugucgucgaaaccug.....................	52	0	He1
....uuAguugucgucgaaaccug.....................	1	1	He1
....uucguuUucgucgaaaccug.....................	2	1	He1
....uucgCugucgucgaaaccug.....................	1	1	He1
....uucguugucgucgaaacUuga....................	1	1	He1
....uucgGugucgucgaaaccuga....................	17	1	He1
....Gucguugucgucgaaaccuga....................	1	1	He1
....uuGguugucgucgaaaccuga....................	3	1	He1
....uucguuAucgucgaaaccuga....................	1	1	He1
....uucguugGcgucgaaaccuga....................	1	1	He1
....uuAguugucgucgaaaccuga....................	1	1	He1
....uucUuugucgucgaaaccuga....................	1	1	He1
....uucguugucgucgaaaccugC....................	1	1	He1
....uucguugucgucgaaaccuga....................	293	0	He1
....uucgAugucgucgaaaccuga....................	1	1	He1
....uucgAugucgucgaaaccugaa...................	3	1	He1
....uucguugucgucgaaaccugaC...................	27	1	He1
....uucguugucgucgaaaccuUaa...................	1	1	He1
....uucguugucgucgaaaccAgaa...................	1	1	He1
....Aucguugucgucgaaaccugaa...................	2	1	He1
....uucguugucgucgaaaccugaa...................	191	0	He1
....uucgGugucgucgaaaccugaa...................	9	1	He1
....uucguuUucgucgaaaccugaa...................	1	1	He1
....uucguugucgucgCaaccugaa...................	1	1	He1
....uucguuAucgucgaaaccugaa...................	1	1	He1
....uuAguugucgucgaaaccugaa...................	1	1	He1
....uucguugucgucgaaaccugUa...................	1	1	He1
....uuGguugucgucgaaaccugaa...................	1	1	He1
....uucguugucgucgaaaccugaU...................	6	1	He1
....uucguugucgucgaaaccugaaG..................	4	1	He1
....uucguugucgucgaaaccugaac..................	2	0	He1
....uucgAugucgucgaaaccugaac..................	1	1	He1
....uucguugucgucgaaaccugaaA..................	22	1	He1
....uucguugucgucgaaaccugaaU..................	5	1	He1
....uucguugucgucgaaaccugaCc..................	3	1	He1
....uucguugucgucgaaaccugaaAa.................	1	1	He1
....uucguugucgucgaaaccugaaUa.................	2	1	He1
....uucguugucgucgaaaccugaaca.................	4	0	He1
....ucgAugucgucgaaaccuga....................	1	1	He1
....ucguugucgucgaaaccuga....................	3	0	He1
....ucgGugucgucgaaaccuga....................	1	1	He1
....ucguugucgucgaaaccugaa...................	1	0	He1
....uucguugucgucgaaaccug.....................	14	0	Ta1

Star Mature

```
caucucuucacguuaauaauagagggagaaguugcccagguuucucgauaaacgaauauuaaacuguuuguucguugucgucgaaaccugaacaaaacucccuggacccuc
......................................................................uucgGugucgucgaaaccug.....................   1   1   Ta1
......................................................................uucgAugucgucgaaaccuga....................   1   1   Ta1
......................................................................uucgCugucgucgaaaccuga....................   1   1   Ta1
......................................................................uucgGugucgucgaaaccuga....................   2   1   Ta1
......................................................................uuUguugucgucgaaaccuga....................   1   1   Ta1
......................................................................uucguugucgucgaaaccuga....................   43  0   Ta1
......................................................................uucgGugucgucgaaaccugaa...................   1   1   Ta1
......................................................................uucguugucgucgaaaccugaa...................   9   0   Ta1
......................................................................uucguugucgucgaaaccugaC...................   9   1   Ta1
......................................................................uucguugucgucgaaaccugaaG..................   6   1   Ta1
......................................................................uucguugucgucgaaaccugaaA..................   8   1   Ta1
......................................................................uucguugucgucgaaaccugaCc..................   5   1   Ta1
......................................................................uucguugucgucgaaaccugaaca.................   4   0   Ta1

.....................................................................Cuucguugucgucgaaaccuga....................   1   1   Le5
......................................................................uucguugucgucgaaaccC......................   1   1   Le5
......................................................................uucguugucgucgaaaccG......................   1   1   Le5
......................................................................uuGguugucgucgaaaccug.....................   1   1   Le5
......................................................................uucgGugucgucgaaaccug.....................   5   1   Le5
......................................................................uucgCugucgucgaaaccug.....................   1   1   Le5
......................................................................uucguugucgucgaaaccuU.....................   1   1   Le5
......................................................................uucguugucgucgaaaccAg.....................   1   1   Le5
......................................................................uucguugucgucgaaaccug.....................   87  0   Le5
......................................................................uuUguugucgucgaaaccug.....................   1   1   Le5
......................................................................Aucguugucgucgaaaccug.....................   1   1   Le5
......................................................................uucguugucgucgaaCccug.....................   1   1   Le5
......................................................................uucgAugucgucgaaaccug.....................   2   1   Le5
......................................................................uucguuUucgucgaaaccuga....................   1   1   Le5
......................................................................uucguugucgucgaaUccuga....................   1   1   Le5
......................................................................uuAguugucgucgaaaccuga....................   4   1   Le5
......................................................................uucguugucUucgaaaccuga....................   1   1   Le5
......................................................................uucguugucgucgaaaccuga....................   240 0   Le5
......................................................................uucgCugucgucgaaaccuga....................   3   1   Le5
......................................................................uucguugucgucgCaaccuga....................   1   1   Le5
......................................................................uuUguugucgucgaaaccuga....................   1   1   Le5
......................................................................uucgGugucgucgaaaccuga....................   13  1   Le5
......................................................................uucgAugucgucgaaaccugaa...................   2   1   Le5
......................................................................uucguugucgucgaaaccugaa...................   126 0   Le5
......................................................................uucguugucgucgaaaccugaG...................   1   1   Le5
......................................................................uucguugucgucgaaaccugaC...................   27  1   Le5
......................................................................uucgGugucgucgaaaccugaa...................   10  1   Le5
......................................................................uuAguugucgucgaaaccugaa...................   2   1   Le5
......................................................................uuGguugucgucgaaaccugaa...................   1   1   Le5
......................................................................uucgCugucgucgaaaccugaa...................   2   1   Le5
......................................................................uucguugucgucgaaUccugaa...................   1   1   Le5
......................................................................uucguugucgucgaaaccugaU...................   2   1   Le5
......................................................................uucguugucgucgaaaccugaaU..................   2   1   Le5
......................................................................uucguugucgucgaaaccugaCc..................   2   1   Le5
......................................................................uucguugucgucgaaaccugaaA..................   23  1   Le5
......................................................................uucgGugucgucgaaaccugaac..................   1   1   Le5
......................................................................uucguugucgucgaaaccugaac..................   1   0   Le5
......................................................................uucguugucgucgaaaccugaacC.................   1   1   Le5
......................................................................uucguugucgucgaaaccugaaUa.................   1   1   Le5
......................................................................uucguugucguUgaaaccugaaca.................   4   1   Le5
......................................................................uucguugucgucgaaaccugaaca.................   1   0   Le5
......................................................................uucguugucgucgaaaccugaCca.................   1   1   Le5
.......................................................................ucguugucCucgaaaccug.....................   1   1   Le5
.......................................................................ucguugucgucgaaaccuga....................   6   0   Le5

.....................................................................guucguugucgucgaaaccug.....................   4   0   Br1
.....................................................................guucguugucgucgaaaccuga....................   7   0   Br1
.....................................................................Cuucguugucgucgaaaccuga....................   2   1   Br1
.....................................................................guucguugucgucgaaaccugaa...................   1   0   Br1
.....................................................................Cuucguugucgucgaaaccugaa...................   1   1   Br1
......................................................................uucguugucgucgaaaccA......................   4   1   Br1
......................................................................uucguugucgucgaaaccu......................   15  0   Br1
......................................................................uucguugucgucgaaaccC......................   4   1   Br1
......................................................................uucguugucgucgaaaccAg.....................   4   1   Br1
......................................................................uAcguugucgucgaaaccug.....................   1   1   Br1
......................................................................Aucguugucgucgaaaccug.....................   4   1   Br1
```

Star Mature

```
caucucuucacguuaauaauagagggagaaguugcccagguuucucgauaaacgaauauuaaacuguuuguucguugucgucgaaaccugaacaaaacucccuggacccuc
......................................................................uucguuguAgucgaaaccug.....................	1	1	Br1
......................................................................uucguugucgucgaaaccGg.....................	1	1	Br1
......................................................................uuUguugucgucgaaaccug.....................	2	1	Br1
......................................................................uucguugucgucgaaaccCg.....................	20	1	Br1
......................................................................uucguugucAucgaaaccug.....................	2	1	Br1
......................................................................uucguugucguGgaaaccug.....................	1	1	Br1
......................................................................uucguugucgucgaaaccuU.....................	2	1	Br1
......................................................................uucguugucgucgaaaccug.....................	748	0	Br1
......................................................................uucguugucgucgaaaccuA.....................	12	1	Br1
......................................................................uucguugucgucgaaaAcug.....................	1	1	Br1
......................................................................uucguuguUgucgaaaccug.....................	2	1	Br1
......................................................................uucguugucgCcgaaaccug.....................	1	1	Br1
......................................................................uucgCugucgucgaaaccug.....................	2	1	Br1
......................................................................uucUuugucgucgaaaccug.....................	3	1	Br1
......................................................................uucguugucgucgCaaccug.....................	3	1	Br1
......................................................................uucguuAucgucgaaaccug.....................	1	1	Br1
......................................................................uucgGugucgucgaaaccug.....................	30	1	Br1
......................................................................Cucguugucgucgaaaccug.....................	2	1	Br1
......................................................................uuAguugucgucgaaaccug.....................	6	1	Br1
......................................................................uucguugucgucgaUaccug.....................	1	1	Br1
......................................................................uCcguugucgucgaaaccuga....................	1	1	Br1
......................................................................uucguugucgucgaaaccugC....................	6	1	Br1
......................................................................uucguugucgucgaaacUuga....................	5	1	Br1
......................................................................uucguugucgucgaaaccugG....................	1	1	Br1
......................................................................uucguugucguAgaaaccuga....................	2	1	Br1
......................................................................uucgCugucgucgaaaccuga....................	36	1	Br1
......................................................................uucguugucgucgCaaccuga....................	17	1	Br1
......................................................................uucgAugucgucgaaaccuga....................	45	1	Br1
......................................................................Aucguugucgucgaaaccuga....................	10	1	Br1
......................................................................uucguugucgucgaaaccCga....................	3	1	Br1
......................................................................Nucguugucgucgaaaccuga....................	5	1	Br1
......................................................................Gucguugucgucgaaaccuga....................	1	1	Br1
......................................................................uucguugucgucgaaaccGga....................	5	1	Br1
......................................................................uuUguugucgucgaaaccuga....................	13	1	Br1
......................................................................uucguuguAgucgaaaccuga....................	17	1	Br1
......................................................................uucguugucgucUaaaccuga....................	2	1	Br1
......................................................................uucguugucgAcgaaaccuga....................	3	1	Br1
......................................................................uucguugucgucgaaaAcuga....................	3	1	Br1
......................................................................uucguugucgucgaaacAuga....................	1	1	Br1
......................................................................uucguugucgucgaGaccuga....................	1	1	Br1
......................................................................uucgGugucgucgaaaccuga....................	208	1	Br1
......................................................................uucguuguUgucgaaaccuga....................	4	1	Br1
......................................................................uucguugucgucgaaaccuCa....................	1	1	Br1
......................................................................uucguugucgucgaaaccuUa....................	11	1	Br1
......................................................................uAcguugucgucgaaaccuga....................	1	1	Br1
......................................................................uucguugucUucgaaaccuga....................	1	1	Br1
......................................................................Cucguugucgucgaaaccuga....................	11	1	Br1
......................................................................uucguugCcgucgaaaccuga....................	1	1	Br1
......................................................................uucguugucguGgaaaccuga....................	1	1	Br1
......................................................................uucguugGcgucgaaaccuga....................	3	1	Br1
......................................................................uucUuugucgucgaaaccuga....................	13	1	Br1
......................................................................uucguuCucgucgaaaccuga....................	2	1	Br1
......................................................................uucguugucAucgaaaccuga....................	3	1	Br1
......................................................................uucguugucgucgaaaccugU....................	14	1	Br1
......................................................................uucguugucgucgaaaccuga....................	4850	0	Br1
......................................................................uucguCgucgucgaaaccuga....................	1	1	Br1
......................................................................uucguugucgucgaaUccuga....................	1	1	Br1
......................................................................uucguugucgucgaaaccAga....................	1	1	Br1
......................................................................uucguuUucgucgaaaccuga....................	10	1	Br1
......................................................................uucAuugucgucgaaaccuga....................	3	1	Br1
......................................................................uuGguugucgucgaaaccuga....................	4	1	Br1
......................................................................uucguugucgucgaUaccuga....................	3	1	Br1
......................................................................uuAguugucgucgaaaccuga....................	33	1	Br1
......................................................................uucguugucgucAaaaccuga....................	2	1	Br1
......................................................................uucguugucgucgUaaccuga....................	1	1	Br1
......................................................................uuUguugucgucgaaaccugaa...................	3	1	Br1
......................................................................uucguugucgucgaaaAcugaa...................	2	1	Br1
......................................................................uucguugucgucgaaUccugaa...................	3	1	Br1
......................................................................uucgAugucgucgaaaccugaa...................	38	1	Br1
......................................................................uucguugucgucgaaacUugaa...................	4	1	Br1
```

```
                                     Star                            Mature
caucucuucacguuaauaauagagggagaaguugcccagguuucucgauaaacgaauauuaaacuguuuguucguugucgucgaaaccugaacaaaacucccuggacccuc
......................................................................uucguuguGgucgaaaccugaa...................   1      1    Br1
......................................................................uucgGugucgucgaaaccugaa...................   126    1    Br1
......................................................................uucguuAucgucgaaaccugaa...................   2      1    Br1
......................................................................uuGguugucgucgaaaccugaa...................   4      1    Br1
......................................................................Cucguugucgucgaaaccugaa...................   1      1    Br1
......................................................................uucUuugucgucgaaaccugaa...................   8      1    Br1
......................................................................uucguugucgucgaaaccuUaa...................   4      1    Br1
......................................................................uucguugucgAcgaaaccugaa...................   2      1    Br1
......................................................................uucguugucgucgaaaccugaC...................   523    1    Br1
......................................................................uucguugucgucgaaaccugaa...................   2862   0    Br1
......................................................................uucguugucgucgaaaccGgaa...................   1      1    Br1
......................................................................Gucguugucgucgaaaccugaa...................   1      1    Br1
......................................................................uucguugucgucgaaaccugaG...................   3      1    Br1
......................................................................uucguuguAgucgaaaccugaa...................   10     1    Br1
......................................................................uucguugucgucgaaaccugCa...................   1      1    Br1
......................................................................Nucguugucgucgaaaccugaa...................   3      1    Br1
......................................................................uuAguugucgucgaaaccugaa...................   18     1    Br1
......................................................................uucguuguUgucgaaaccugaa...................   4      1    Br1
......................................................................uucguuUucgucgaaaccugaa...................   5      1    Br1
......................................................................uAcguugucgucgaaaccugaa...................   2      1    Br1
......................................................................uucguugucguAgaaaccugaa...................   2      1    Br1
......................................................................uucguugucgucgaaacAugaa...................   1      1    Br1
......................................................................uucgCugucgucgaaaccugaa...................   10     1    Br1
......................................................................Aucguugucgucgaaaccugaa...................   5      1    Br1
......................................................................uucguugucgucgUaaccugaa...................   2      1    Br1
......................................................................uucguugucgucgCaaccugaa...................   9      1    Br1
......................................................................uucguugGcgucgaaaccugaa...................   2      1    Br1
......................................................................uucAuugucgucgaaaccugaa...................   1      1    Br1
......................................................................uucguugucgucgaaaccugaU...................   52     1    Br1
......................................................................uCcguugucgucgaaaccugaa...................   1      1    Br1
......................................................................uucgGugucgucgaaaccugaac..................   5      1    Br1
......................................................................uucguugucgucgaaaccugaCc..................   38     1    Br1
......................................................................uucguuguAgucgaaaccugaac..................   1      1    Br1
......................................................................uucguugucgucgaaaccugaaA..................   376    1    Br1
......................................................................uuAguugucgucgaaaccugaac..................   1      1    Br1
......................................................................uucguugucgucgaaaccugaaG..................   10     1    Br1
......................................................................uucgCugucgucgaaaccugaac..................   1      1    Br1
......................................................................uucgAugucgucgaaaccugaac..................   1      1    Br1
......................................................................uucguugucgucgaaaccugaac..................   49     0    Br1
......................................................................uucguugucgucgaaaccugaaU..................   94     1    Br1
......................................................................uucguuguAgucgaaaccugaaca.................   1      1    Br1
......................................................................uucguugucgucgaaaccugaacU.................   1      1    Br1
......................................................................uucguugucgucgaaaccugaaAa.................   7      1    Br1
......................................................................uucguugucgucgaaaccugaaUa.................   2      1    Br1
......................................................................uucguugucguUgaaaccugaaca.................   7      1    Br1
......................................................................uucguugucgucgaaaccugaaca.................   20     0    Br1
.......................................................................ucguugucgucgaaaccug.....................   2      0    Br1
.......................................................................ucgGugucgucgaaaccug.....................   1      1    Br1
.......................................................................ucCuugucgucgaaaccuga....................   2      1    Br1
.......................................................................ucguGgucgucgaaaccuga....................   7      1    Br1
.......................................................................ucguugucAucgaaaccuga....................   1      1    Br1
.......................................................................ucguuAucgucgaaaccuga....................   1      1    Br1
.......................................................................ucgGugucgucgaaaccuga....................   2      1    Br1
.......................................................................ucguCgucgucgaaaccuga....................   1      1    Br1
.......................................................................ucguugucgucUaaaccuga....................   1      1    Br1
.......................................................................ucguugucgucgaaaccuga....................   26     0    Br1
.......................................................................uAguugucgucgaaaccuga....................   2      1    Br1
.......................................................................ucgGugucgucgaaaccugaa...................   1      1    Br1
.......................................................................uAguugucgucgaaaccugaa...................   1      1    Br1
.......................................................................ucguugucgucgaaaccugaa...................   9      0    Br1
.......................................................................ucguCgucgucgaaaccugaa...................   2      1    Br1
.......................................................................ucguugucgucgaaaUcugaa...................   1      1    Br1
.......................................................................ucguugucgucgaaaccugaC...................   1      1    Br1
.......................................................................ucguugucgucgaaaccugaaU..................   1      1    Br1
........................................................................cguugucgucgaaaccuga....................   1      0    Br1
........................................................................cguugucgucgaaaccugaaA..................   1      1    Br1

......................................................................uucguugucgucgaaaccu......................   3      0    Le1
......................................................................uucguuAucgucgaaaccu......................   1      1    Le1
......................................................................uuAguugucgucgaaaccug.....................   3      1    Le1
```

```
                                                     Star                             Mature
caucucuucacguuaauaauagagggagaaguugcccagguuucucgauaaacgaauauuaaacuguuuguucguugucgucgaaaccugaacaaaacucccuggacccuc
.....................................................................uucguugucgucgaaaccuA......................	2	1	Le1
.....................................................................uuGguugucgucgaaaccug......................	1	1	Le1
.....................................................................uucUuugucgucgaaaccug......................	2	1	Le1
.....................................................................uucguugucgucgaaacUug......................	1	1	Le1
.....................................................................uucguugucgucgaaaccuC......................	1	1	Le1
.....................................................................uucguugucgucgaaaccug......................	94	0	Le1
.....................................................................Aucguugucgucgaaaccug......................	1	1	Le1
.....................................................................uucguugucUucgaaaccug......................	1	1	Le1
.....................................................................uucgGugucgucgaaaccug......................	5	1	Le1
.....................................................................uucguugucgucgaaaccCg......................	1	1	Le1
.....................................................................uucguuUucgucgaaaccuga....................	2	1	Le1
.....................................................................uucgCugucgucgaaaccuga....................	2	1	Le1
.....................................................................uucgAugucgucgaaaccuga....................	2	1	Le1
.....................................................................Cucguugucgucgaaaccuga....................	1	1	Le1
.....................................................................uucguugucgucgCaaccuga....................	3	1	Le1
.....................................................................uucguuguAgucgaaaccuga....................	2	1	Le1
.....................................................................uuUguugucgucgaaaccuga....................	1	1	Le1
.....................................................................Nucguugucgucgaaaccuga....................	1	1	Le1
.....................................................................uucguugucgucgaaaUcuga....................	2	1	Le1
.....................................................................uucUuugucgucgaaaccuga....................	3	1	Le1
.....................................................................uucguugucgucgaaaccugU....................	2	1	Le1
.....................................................................uucguugucgCcgaaaccuga....................	1	1	Le1
.....................................................................uuAguugucgucgaaaccuga....................	1	1	Le1
.....................................................................uucgGugucgucgaaaccuga....................	15	1	Le1
.....................................................................uucguugucgucgaaaccuUa....................	1	1	Le1
.....................................................................uucguugucgucgaaaccuga....................	282	0	Le1
.....................................................................uucguugucgucgaaaccuUaa...................	1	1	Le1
.....................................................................uucgAugucgucgaaaccugaa...................	2	1	Le1
.....................................................................uucguugucgucgaaaccugaG...................	1	1	Le1
.....................................................................uucgGugucgucgaaaccugaa...................	5	1	Le1
.....................................................................uucguugucgucgaaaccugaC...................	32	1	Le1
.....................................................................uucguuUucgucgaaaccugaa...................	1	1	Le1
.....................................................................uucguugucgucgaaaccugaa...................	113	0	Le1
.....................................................................uucguugucgucgaaaccugaCc..................	4	1	Le1
.....................................................................uucguugucgucgaaaccugaac..................	3	0	Le1
.....................................................................uucguugucgucgaaaccugaaU..................	3	1	Le1
.....................................................................uucgGugucgucgaaaccugaac..................	1	1	Le1
.....................................................................uucguugucgucgaaaccugaaA..................	5	1	Le1
.....................................................................uucgAugucgucgaaaccugaaca.................	1	1	Le1
......................................................................ucguugucgucgaaaccuga.....................	2	0	Le1
```

Provisional ID	:	Sc28yqQ_4222_2657
Score total	:	958.8
Score for star read(s)	:	3.9
Score for read counts	:	948.4
Score for mfe	:	1.8
Score for randfold	:	1.6
Score for cons. seed	:	3
Total read count	:	1872
Mature read count	:	1859
Loop read count	:	0
Star read count	:	13

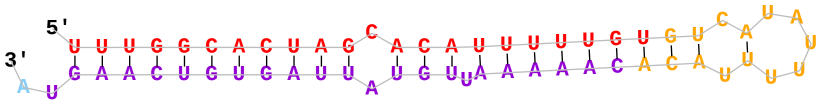

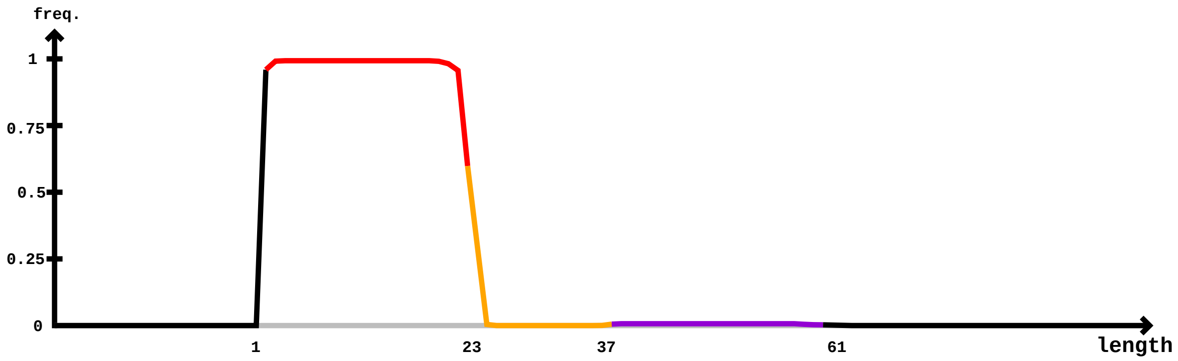

Mature Star

```
5'- cuuuuugcacucucccauuuuuuggcacuagcacauuuuuguguucauauuuuuacacaaaaauuguauuagugucaaguaauguggggaggcgaacgacaaaauaauucauc   -3'   obs
    cuuuuugcacucucccauuuuuuggcacuagcacauuuuugugucauauuuuuacacaaaaauuguauuagugucaaguaauguggggaggcgaacgacaaaauaauucauc         exp
    ...(((((.((((((((((.(((((((((((.((((((((((((.(.....).))))))))).))).))))))))))).)))).)))))))))))................. reads   mm   sample
    ....................uuugUcacuagcacauuuu.........................................................................  1     1    Ch1
    ....................uuuggcacuagcacauuuu.........................................................................  2     0    Ch1
    ....................uuuggcacuagcacauuuuu........................................................................  14    0    Ch1
    ....................uuuggcacuagcacauuuuA........................................................................  1     1    Ch1
    ....................uuugUcacuagcacauuuuu........................................................................  2     1    Ch1
    ....................uuuggcacuCgcacauuuuu........................................................................  1     1    Ch1
    ....................uuuggcacuaAcacauuuuug.......................................................................  1     1    Ch1
    ....................uuuggcaUuagcacauuuuug.......................................................................  1     1    Ch1
    ....................uuuUgcacuagcacauuuuug.......................................................................  1     1    Ch1
    ....................uuuggcacuagcacauuuuCg.......................................................................  1     1    Ch1
    ....................uuugUcacuagcacauuuuug.......................................................................  13    1    Ch1
    ....................uuuggcacuagcacauuuuuA.......................................................................  1     1    Ch1
    ....................uuuggcacuUgcacauuuuug.......................................................................  1     1    Ch1
    ....................uuugCcacuagcacauuuuug.......................................................................  2     1    Ch1
    ....................uuuggcacuagcacauuuuug.......................................................................  217   0    Ch1
    ....................uuuggcacuagcacauuuuugC......................................................................  8     1    Ch1
    ....................uuuUgcacuagcacauuuuugu......................................................................  2     1    Ch1
    ....................Auuggcacuagcacauuuuugu......................................................................  2     1    Ch1
    ....................uuugCcacuagcacauuuuugu......................................................................  5     1    Ch1
    ....................Cuuggcacuagcacauuuuugu......................................................................  1     1    Ch1
    ....................uuugAcacuagcacauuuuugu......................................................................  1     1    Ch1
    ....................uuuggcacuagcacauuuuugu......................................................................  308   0    Ch1
    ....................uuCggcacuagcacauuuuugu......................................................................  1     1    Ch1
    ....................uuuggcacuCgcacauuuuugu......................................................................  2     1    Ch1
    ....................uuuggcaUuagcacauuuuugu......................................................................  1     1    Ch1
    ....................uuuggcacCagcacauuuuugu......................................................................  1     1    Ch1
    ....................uuuggcacuagGacauuuuugu......................................................................  1     1    Ch1
    ....................uuuggcacuagcacauuuuugA......................................................................  40    1    Ch1
    ....................uuuggcacuagcCcauuuuugu......................................................................  2     1    Ch1
    ....................uuugUcacuagcacauuuuugu......................................................................  15    1    Ch1
    ....................uuuggcacuagcacauuuuugug.....................................................................  24    0    Ch1
    ....................uuuggcacuagcacauuuuuguU.....................................................................  16    1    Ch1
    ....................uuuggcacuagcacauuuuGgug.....................................................................  1     1    Ch1
```

```
                    Mature                            Star
cuuuuugcacucucccauuuuuuggcacuagcacauuuuugugucauauuuuuacacaaaaauuguauuagugucaaguaauguggggaggcgaacgacaaaauaauucauc
....................uuuggcacuCgcacauuuuugug.....................................................................    1    1    Ch1
....................uuuggcacuagcacauuuuugugA....................................................................    1    1    Ch1
.....................uuCgcacuagcacauuuuu........................................................................    1    1    Ch1
.....................uuggcacuCgcacauuuuug.......................................................................    1    1    Ch1
.....................uuggcacuagcacauuuuug.......................................................................    4    0    Ch1
.....................uuggcacuagcacauuuuugu......................................................................    11   0    Ch1
.....................uuggcacuagcacauuuuugA......................................................................    3    1    Ch1
.....................Cuggcacuagcacauuuuugu......................................................................    1    1    Ch1
.....................uuggcacuagcacUuuuuugu......................................................................    1    1    Ch1
.....................uuggcacuagcacauuuuuguU.....................................................................    1    1    Ch1
.....................uuggcacuagcacauuuuugugu....................................................................    2    0    Ch1
......................uggcacuagcacauuuuugug.....................................................................    2    0    Ch1
........................................................caaaaauuguauuaguguca....................................    1    0    Ch1
........................................................caaaaauuguauuagugucaagu.................................    2    0    Ch1
.........................................................aaaaauuguauuaguguca....................................    1    0    Ch1
.........................................................aaaaauuguauuagugucaa...................................    1    0    Ch1

....................uuugAcacuagcacauuu..........................................................................    1    1    Bl1
....................uuuggcacuagcacauuu..........................................................................    1    0    Bl1
....................uuuggcacuagcacauuuu.........................................................................    2    0    Bl1
....................uuuggcacuagcacauuuuug.......................................................................    16   0    Bl1
....................uuugUcacuagcacauuuuugu......................................................................    2    1    Bl1
....................uuuggcacuagcacauuuuugu......................................................................    16   0    Bl1
....................uuuggcacuagcacauuuuugA......................................................................    2    1    Bl1
....................uuuggcacuagcacauuuuugug.....................................................................    1    0    Bl1
.....................uuggcacuagcacauuuuug.......................................................................    3    0    Bl1
.....................uuggcacuagcacauuuuugu......................................................................    2    0    Bl1
........................................................caaaaauuguauuagugucaag..................................    1    0    Bl1

....................uuuggcacuagcacauuu..........................................................................    1    0    He1
....................uuugUcacuagcacauuu..........................................................................    1    1    He1
....................uuuggcacuagcacauuuu.........................................................................    3    0    He1
....................uuuggcacuagcacauuuuu........................................................................    3    0    He1
....................uuuggcacuagcCcauuuuug.......................................................................    1    1    He1
....................uuuggcacuagcacauuuuug.......................................................................    35   0    He1
....................uuugCcacuagcacauuuuug.......................................................................    2    1    He1
....................uuugUcacuagcacauuuuug.......................................................................    2    1    He1
....................uuugCcacuagcacauuuuugu......................................................................    1    1    He1
....................uuugAcacuagcacauuuuugu......................................................................    1    1    He1
....................uuuggcacuagcacauuuuugG......................................................................    1    1    He1
....................uuuggcacuagcacauuuuugu......................................................................    44   0    He1
....................uuuggcacuagcacauuuuugC......................................................................    1    1    He1
....................uuugUcacuagcacauuuuugu......................................................................    3    1    He1
....................uuuggcacuagcacauuuuugA......................................................................    5    1    He1
....................uuuggcacuagcacauuuuuguU.....................................................................    2    1    He1
.....................uuggcacuagcacauuuuug.......................................................................    2    0    He1
.....................uuggcacuagcacauuuuugu......................................................................    2    0    He1
........................................................caaaaauuguauuagugucaagu.................................    2    0    He1

....................uuuggcacuCgcacauuuuug.......................................................................    1    1    Ta1
....................uuuggcacuagcacauuuuug.......................................................................    28   0    Ta1
....................uuugUcacuagcacauuuuug.......................................................................    3    1    Ta1
....................uuuggcaUuagcacauuuuug.......................................................................    1    1    Ta1
....................uuuggcacuagcaUauuuuugu......................................................................    1    1    Ta1
....................uuugUcacuagcacauuuuugu......................................................................    1    1    Ta1
....................uuuggcacuagcacauuuuugu......................................................................    29   0    Ta1
....................uuuggcacuagcacauuuuugA......................................................................    2    1    Ta1
....................uuugAcacuagcacauuuuugu......................................................................    1    1    Ta1
....................uuuUgcacuagcacauuuuugu......................................................................    1    1    Ta1
....................uuuggcacuagcacauuuuuguU.....................................................................    8    1    Ta1
....................uuuggcacuagcacauuuuugug.....................................................................    2    0    Ta1

....................uuuggcacuagcacauuuu.........................................................................    3    0    Le5
....................uuugAcacuagcacauuuuu........................................................................    1    1    Le5
....................uuuggcacuagcacauuuuu........................................................................    7    0    Le5
....................uuugAcacuagcacauuuuug.......................................................................    1    1    Le5
....................uuugCcacuagcacauuuuug.......................................................................    2    1    Le5
....................uuuggcacuagcacauuuuug.......................................................................    69   0    Le5
....................uuuggcacuaAcacauuuuug.......................................................................    1    1    Le5
....................uuugUcacuagcacauuuuug.......................................................................    3    1    Le5
```

Mature **Star**

cuuuuugcacucucccauuuuuuggcacuagcacauuuuugugucauauuuuuacacaaaaauuguauuagugucaaguaauguggggaggcgaacgacaaaauaauucauc

....................uuuggcaUuagcacauuuuug...	1	1	Le5
....................uuugUcacuagcacauuuuugu..	6	1	Le5
....................uuuggcacuagcacauuuuugA..	12	1	Le5
....................uuugAcacuagcacauuuuugu..	1	1	Le5
....................uuugCcacuagcacauuuuugu..	2	1	Le5
....................uuuggcacuagcacauuuuugu..	77	0	Le5
....................uuuggcacuagcacauuuuugC..	2	1	Le5
....................uuuggcacuCgcacauuuuugu..	1	1	Le5
....................uuuggcacuagcacauuuuugug...	1	0	Le5
....................uuuggcacuagcacauuuuuguU...	10	1	Le5
....................uuugUcacuagcacauuuuugug...	1	1	Le5
....................uuuggcacuagcacauuuuugugu..	1	0	Le5
.....................uuggcacuagcacauuuuug...	3	0	Le5
.....................uuggcacuaAcacauuuuugu..	1	1	Le5
.....................uuggcacuagcacauuuuugA..	1	1	Le5
.....................uuggcacuagcacauuuuugu..	5	0	Le5
.....................uuggcacuagcacauuuuugug...	1	0	Le5
.....................uuggcacuagcacauuuuugugu..	1	0	Le5
......................uggcacuagcacauuuuugu..	1	0	Le5
....acaaaaauuguauuaguguca....................................	1	0	Le5
....caaaaauuguauuaguguca....................................	1	0	Le5
....caaaaauuguauuagugucaa...................................	1	0	Le5
....caaaaauuguauuagugucaagu.................................	1	0	Le5
....................uuuggcacuagcacauuuu...	3	0	Br1
....................uuugUcacuagcacauuuu...	1	1	Br1
....................uuuggcacuagcacauuuuu..	10	0	Br1
....................uuugUcacuagcacauuuuu..	1	1	Br1
....................uuugCcacuagcacauuuuug...	2	1	Br1
....................uuuggcacuaAcacauuuuug...	1	1	Br1
....................uuugAcacuagcacauuuuug...	1	1	Br1
....................uuuggcacuagcacauuuuug...	135	0	Br1
....................uuuggUacuagcacauuuuug...	1	1	Br1
....................uuuggcacuagcCcauuuuug...	1	1	Br1
....................uuugUcacuagcacauuuuug...	12	1	Br1
....................uuuggcaUuagcacauuuuug...	1	1	Br1
....................uuuggcacuagcacauuuuugG..	2	1	Br1
....................uuuggcacuaAcacauuuuugu..	1	1	Br1
....................uuuggcacuagcacauuuuugu..	202	0	Br1
....................uuuggcacuagcacauuuuugA..	47	1	Br1
....................uuuggcacuagcacauuuuugC..	6	1	Br1
....................uuuggcacuCgcacauuuuugu..	1	1	Br1
....................uuugUcacuagcacauuuuugu..	7	1	Br1
....................uuuggcacuagcacauuuuuguU...	2	1	Br1
....................uuuggcacuagcacauuuuugug...	2	0	Br1
.....................uuggcacuagcacauuuuu..	1	0	Br1
.....................uuggcacuagcacauuuuug...	2	0	Br1
.....................uuggcacuagcacauuuuugu..	3	0	Br1
.....................uuggcacuCgcacauuuuugu..	1	1	Br1
....aaaaauuguauuagugucaa...................................	1	0	Br1
....................uuuggcacuagcacauuuu...	2	0	Le1
....................uuuggcacuagcacauuuuu..	5	0	Le1
....................uuuggcacuagcacauuuuug...	83	0	Le1
....................uuCggcacuagcacauuuuug...	1	1	Le1
....................uuAggcacuagcacauuuuug...	1	1	Le1
....................uuugUcacuagcacauuuuug...	8	1	Le1
....................uuuggcaUuagcacauuuuug...	1	1	Le1
....................uuugAcacuagcacauuuuug...	1	1	Le1
....................uuuggcacuagcacauuuuugA..	18	1	Le1
....................uuuggcacuagcacauuuuugu..	98	0	Le1
....................uuuggcacuagcacauuuuugC..	2	1	Le1
....................uuugUcacuagcacauuuuugu..	7	1	Le1
....................uuugUcacuagcacauuuuugug...	3	1	Le1
....................uuuggcacuagcacauuuuugug...	2	0	Le1
....................uuuggcacuagcacauuuuuguU...	4	1	Le1
....................uuuggcacuagcacauuuuugugA..	1	1	Le1
....................uuuggcacuagcacauuuuugugu..	1	0	Le1
.....................uCggcacuagcacauuuuug...	1	1	Le1
.....................uuggcacuagcacauuuuug...	2	0	Le1

```
                    Mature                             Star
cuuuuugcacucucccauuuuuuuggcacuagcacauuuuugugucauauuuuuacacaaaaauuguauuagugucaaguaauguggggaggcgaacgacaaaauaauucauc
......................uuggcacuagcacauuuuugu......................................................................	1	0	Le1
......................uuggcacuagcacauuuuugug.....................................................................	1	0	Le1
......................uuggcacuagcacauuuuuguU.....................................................................	1	1	Le1
......................uuggcacuagcacauuuuugugu....................................................................	1	0	Le1
```

Provisional ID	:	Sc28yqQ_4222_2661
Score total	:	962.4
Score for star read(s)	:	3.9
Score for read counts	:	952
Score for mfe	:	1.9
Score for randfold	:	1.6
Score for cons. seed	:	3
Total read count	:	1879
Mature read count	:	1866
Loop read count	:	0
Star read count	:	13

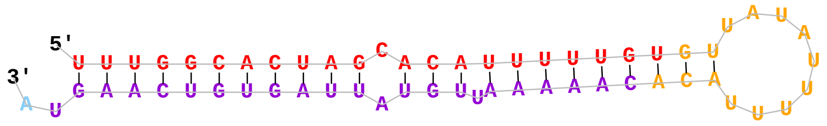

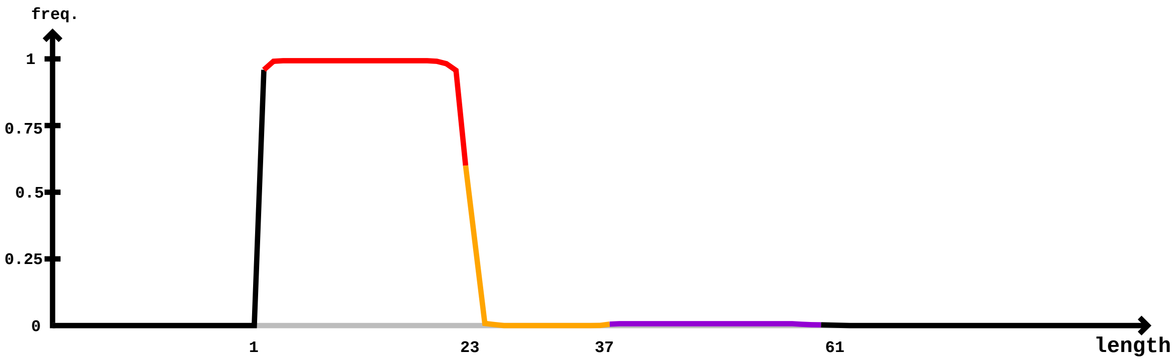

Mature **Star**

```
5'- cuuuuugcacucucccauuuuuuggcacuagcacauuuuuguguuauauuuuuacacaaaaauuguauuagugucaaguaauguggggaggcaaacgacaaaauaauucauc   -3'   obs
    cuuuuugcacucucccauuuuuuggcacuagcacauuuuuguguuauauuuuuacacaaaaauuguauuagugucaaguaauguggggaggcaaacgacaaaauaauucauc         exp
    ...(((((.((((((((((.((((((((((.(((((((((((((.........))))))))).))).))))))))))).)))).))))))))))).................   reads   mm   sample
    ....................uuuggcacuagcacauuuu.........................................................................   3   0   Le5
    ....................uuugAcacuagcacauuuuu........................................................................   1   1   Le5
    ....................uuuggcacuagcacauuuuu........................................................................   7   0   Le5
    ....................uuuggcacuagcacauuuuug.......................................................................   69   0   Le5
    ....................uuugAcacuagcacauuuuug.......................................................................   1   1   Le5
    ....................uuugCcacuagcacauuuuug.......................................................................   2   1   Le5
    ....................uuugUcacuagcacauuuuug.......................................................................   3   1   Le5
    ....................uuuggcacuaAcacauuuuug.......................................................................   1   1   Le5
    ....................uuuggcaUuagcacauuuuug.......................................................................   1   1   Le5
    ....................uuuggcacuCgcacauuuuugu......................................................................   1   1   Le5
    ....................uuugCcacuagcacauuuuugu......................................................................   2   1   Le5
    ....................uuugUcacuagcacauuuuugu......................................................................   6   1   Le5
    ....................uuuggcacuagcacauuuuugA......................................................................   12   1   Le5
    ....................uuuggcacuagcacauuuuugC......................................................................   2   1   Le5
    ....................uuuggcacuagcacauuuuugu......................................................................   77   0   Le5
    ....................uuugAcacuagcacauuuuugu......................................................................   1   1   Le5
    ....................uuugUcacuagcacauuuuugug.....................................................................   1   1   Le5
    ....................uuuggcacuagcacauuuuugug.....................................................................   1   0   Le5
    ....................uuuggcacuagcacauuuuuguU.....................................................................   10   1   Le5
    ....................uuuggcacuagcacauuuuugugu....................................................................   1   0   Le5
    .....................uuggcacuagcacauuuuug.......................................................................   3   0   Le5
    .....................uuggcacuagcacauuuuugA......................................................................   1   1   Le5
    .....................uuggcacuaAcacauuuuugu......................................................................   1   1   Le5
    .....................uuggcacuagcacauuuuugu......................................................................   5   0   Le5
    .....................uuggcacuagcacauuuuugug.....................................................................   1   0   Le5
    .....................uuggcacuagcacauuuuugugu....................................................................   1   0   Le5
    ......................uggcacuagcacauuuuugu......................................................................   1   0   Le5
    .......................................................acaaaaauuguauuaguguca....................................   1   0   Le5
    ........................................................caaaaauuguauuaguguca....................................   1   0   Le5
    ........................................................caaaaauuguauuagugucaa...................................   1   0   Le5
    ........................................................caaaaauuguauuagugucaagu.................................   1   0   Le5

    ....................uuuggcacuagcacauuuu.........................................................................   3   0   Br1
```

Mature Star

```
cuuuuugcacucucccauuuuuuggcacuagcacauuuuuguguuauauuuuuacacaaaaauuguauuagugucaaguaauguggggaggcaaacgacaaaauaauucauc
....................uuugUcacuagcacauuuu.........................................................................   1     1   Br1
....................uuuggcacuagcacauuuuu........................................................................   10    0   Br1
....................uuugUcacuagcacauuuuu........................................................................   1     1   Br1
....................uuuggcacuaAcacauuuuug.......................................................................   1     1   Br1
....................uuuggcacuagcacauuuuug.......................................................................   135   0   Br1
....................uuuggcaUuagcacauuuuug.......................................................................   1     1   Br1
....................uuuggUacuagcacauuuuug.......................................................................   1     1   Br1
....................uuugUcacuagcacauuuuug.......................................................................   12    1   Br1
....................uuugCcacuagcacauuuuug.......................................................................   2     1   Br1
....................uuugAcacuagcacauuuuug.......................................................................   1     1   Br1
....................uuuggcacuagcCcauuuuug.......................................................................   1     1   Br1
....................uuuggcacuagcacauuuuugu......................................................................   202   0   Br1
....................uuuggcacuaAcacauuuuugu......................................................................   1     1   Br1
....................uuuggcacuagcacauuuuugC......................................................................   6     1   Br1
....................uuuggcacuagcacauuuuugA......................................................................   47    1   Br1
....................uuuggcacuCgcacauuuuugu......................................................................   1     1   Br1
....................uuuggcacuagcacauuuuugG......................................................................   2     1   Br1
....................uuugUcacuagcacauuuuugu......................................................................   7     1   Br1
....................uuuggcacuagcacauuuuugug.....................................................................   2     0   Br1
....................uuuggcacuagcacauuuuuguU.....................................................................   2     1   Br1
.....................uuggcacuagcacauuuuu........................................................................   1     0   Br1
.....................uuggcacuagcacauuuuug.......................................................................   2     0   Br1
.....................uuggcacuCgcacauuuuugu......................................................................   1     1   Br1
.....................uuggcacuagcacauuuuugu......................................................................   3     0   Br1
.........................................................aaaaauuguauuagugucaa...................................   1     0   Br1

....................uuuggcacuagcacauuuu.........................................................................   2     0   Le1
....................uuuggcacuagcacauuuuu........................................................................   5     0   Le1
....................uuugAcacuagcacauuuuug.......................................................................   1     1   Le1
....................uuugUcacuagcacauuuuug.......................................................................   8     1   Le1
....................uuAggcacuagcacauuuuug.......................................................................   1     1   Le1
....................uuuggcaUuagcacauuuuug.......................................................................   1     1   Le1
....................uuCggcacuagcacauuuuug.......................................................................   1     1   Le1
....................uuuggcacuagcacauuuuug.......................................................................   83    0   Le1
....................uuuggcacuagcacauuuuugu......................................................................   98    0   Le1
....................uuugUcacuagcacauuuuugu......................................................................   7     1   Le1
....................uuuggcacuagcacauuuuugA......................................................................   18    1   Le1
....................uuuggcacuagcacauuuuugC......................................................................   2     1   Le1
....................uuuggcacuagcacauuuuuguU.....................................................................   4     1   Le1
....................uuuggcacuagcacauuuuugug.....................................................................   2     0   Le1
....................uuugUcacuagcacauuuuugug.....................................................................   3     1   Le1
....................uuuggcacuagcacauuuuugugu....................................................................   1     0   Le1
....................uuuggcacuagcacauuuuugugA....................................................................   1     1   Le1
.....................uuggcacuagcacauuuuug.......................................................................   2     0   Le1
.....................uCggcacuagcacauuuuug.......................................................................   1     1   Le1
.....................uuggcacuagcacauuuuugu......................................................................   1     0   Le1
.....................uuggcacuagcacauuuuugug.....................................................................   1     0   Le1
.....................uuggcacuagcacauuuuuguU.....................................................................   1     1   Le1
.....................uuggcacuagcacauuuuugugu....................................................................   1     0   Le1

....................uuugUcacuagcacauuuu.........................................................................   1     1   Ch1
....................uuuggcacuagcacauuuu.........................................................................   2     0   Ch1
....................uuuggcacuCgcacauuuuu........................................................................   1     1   Ch1
....................uuuggcacuagcacauuuuA........................................................................   1     1   Ch1
....................uuuggcacuagcacauuuuu........................................................................   14    0   Ch1
....................uuugUcacuagcacauuuuu........................................................................   2     1   Ch1
....................uuuggcacuagcacauuuuuA.......................................................................   1     1   Ch1
....................uuuggcacuaAcacauuuuug.......................................................................   1     1   Ch1
....................uuugUcacuagcacauuuuug.......................................................................   13    1   Ch1
....................uuuggcacuUgcacauuuuug.......................................................................   1     1   Ch1
....................uuugCcacuagcacauuuuug.......................................................................   2     1   Ch1
....................uuuUgcacuagcacauuuuug.......................................................................   1     1   Ch1
....................uuuggcacuagcacauuuuCg.......................................................................   1     1   Ch1
....................uuuggcacuagcacauuuuug.......................................................................   217   0   Ch1
....................uuuggcaUuagcacauuuuug.......................................................................   1     1   Ch1
....................uuuUgcacuagcacauuuuugu......................................................................   2     1   Ch1
....................uuCggcacuagcacauuuuugu......................................................................   1     1   Ch1
....................Auuggcacuagcacauuuuugu......................................................................   2     1   Ch1
....................Cuuggcacuagcacauuuuugu......................................................................   1     1   Ch1
....................uuugCcacuagcacauuuuugu......................................................................   5     1   Ch1
```

Mature **Star**

cuuuuugcacucucccauuuuuuggcacuagcacauuuuuguguuauauuuuuacacaaaaauuguauuagugucaaguaaugugggaggcaaacgacaaaauaauucauc			
....................uuugUcacuagcacauuuuugu...	15	1	Ch1
....................uuuggcacuagcacauuuuugA...	40	1	Ch1
....................uuuggcacuagcacauuuuugC...	8	1	Ch1
....................uuuggcacuCgcacauuuuugu...	2	1	Ch1
....................uuuggcacuagcacauuuuugu...	308	0	Ch1
....................uuuggcaUuagcacauuuuugu...	1	1	Ch1
....................uuuggcacCagcacauuuuugu...	1	1	Ch1
....................uuuggcacuagGacauuuuugu...	1	1	Ch1
....................uuuggcacuagcCcauuuuugu...	2	1	Ch1
....................uuugAcacuagcacauuuuugu...	1	1	Ch1
....................uuuggcacuagcacauuuuuguU..	16	1	Ch1
....................uuuggcacuCgcacauuuuugug..	1	1	Ch1
....................uuuggcacuagcacauuuuugug..	24	0	Ch1
....................uuuggcacuagcacauuuuGgug..	1	1	Ch1
....................uuuggcacuagcacauuuuugugA...	1	1	Ch1
.....................uuCgcacuagcacauuuuu...	1	1	Ch1
.....................uuggcacuagcacauuuuug..	4	0	Ch1
.....................uuggcacuCgcacauuuuug..	1	1	Ch1
.....................Cuggcacuagcacauuuuugu...	1	1	Ch1
.....................uuggcacuagcacauuuuugu...	11	0	Ch1
.....................uuggcacuagcacUuuuugu...	1	1	Ch1
.....................uuggcacuagcacauuuuugA...	3	1	Ch1
.....................uuggcacuagcacauuuuuguU..	1	1	Ch1
.....................uuggcacuagcacauuuuugugu...	2	0	Ch1
......................uggcacuagcacauuuuugug..	2	0	Ch1
......................uggcacuagcacauuuuuguguu..	1	0	Ch1
....caaaaauuguauuaguguca...................................	1	0	Ch1
....caaaaauuguauuagugucaagu................................	2	0	Ch1
....aaaaauuguauuaguguca...................................	1	0	Ch1
....aaaaauuguauuagugucaa..................................	1	0	Ch1
....................uuuggcacuagcacauuu...	1	0	Bl1
....................uuugAcacuagcacauuu...	1	1	Bl1
....................uuuggcacuagcacauuuu..	2	0	Bl1
....................uuuggcacuagcacauuuuug..	16	0	Bl1
....................uuuggcacuagcacauuuuugu...	16	0	Bl1
....................uuuggcacuagcacauuuuugA...	2	1	Bl1
....................uuugUcacuagcacauuuuugu...	2	1	Bl1
....................uuuggcacuagcacauuuuugug..	1	0	Bl1
.....................uuggcacuagcacauuuuug..	3	0	Bl1
.....................uuggcacuagcacauuuuugu...	2	0	Bl1
....caaaaauuguauuagugucaag.................................	1	0	Bl1
....................uuuggcacuagcacauuu...	1	0	He1
....................uuugUcacuagcacauuu...	1	1	He1
....................uuuggcacuagcacauuuu..	3	0	He1
....................uuuggcacuagcacauuuuu...	3	0	He1
....................uuugCcacuagcacauuuuug..	2	1	He1
....................uuugUcacuagcacauuuuug..	2	1	He1
....................uuuggcacuagcacauuuuug..	35	0	He1
....................uuuggcacuagcCcauuuuug..	1	1	He1
....................uuuggcacuagcacauuuuugG...	1	1	He1
....................uuugCcacuagcacauuuuugu...	1	1	He1
....................uuuggcacuagcacauuuuugA...	5	1	He1
....................uuugAcacuagcacauuuuugu...	1	1	He1
....................uuugUcacuagcacauuuuugu...	3	1	He1
....................uuuggcacuagcacauuuuugC...	1	1	He1
....................uuuggcacuagcacauuuuugu...	44	0	He1
....................uuuggcacuagcacauuuuuguU..	2	1	He1
.....................uuggcacuagcacauuuuug..	2	0	He1
.....................uuggcacuagcacauuuuugu...	2	0	He1
....caaaaauuguauuagugucaagu................................	2	0	He1
....................uuuggcacuagcacauuuuug..	28	0	Ta1
....................uuuggcacuCgcacauuuuug..	1	1	Ta1
....................uuuggcaUuagcacauuuuug..	1	1	Ta1
....................uuugUcacuagcacauuuuug..	3	1	Ta1
....................uuuggcacuagcacauuuuugu...	29	0	Ta1
....................uuugUcacuagcacauuuuugu...	1	1	Ta1
....................uuuggcacuagcaUauuuuugu...	1	1	Ta1

```
                    Mature                                  Star
cuuuuugcacucucccauuuuuuggcacuagcacauuuuuguguuauauuuuuacacaaaaauuguauuagugucaaguaauguggggaggcaaacgacaaaauaauucauc
....................uuuggcacuagcacauuuuugA......................................................................    2    1    Ta1
....................uuugAcacuagcacauuuuugu......................................................................    1    1    Ta1
....................uuuUgcacuagcacauuuuugu......................................................................    1    1    Ta1
....................uuuggcacuagcacauuuuuguU.....................................................................    8    1    Ta1
....................uuuggcacuagcacauuuuugug.....................................................................    2    0    Ta1
....................uuuggcacuagcacauuuuuguguu...................................................................    6    0    Ta1
```

Provisional ID	:	Sc28yqQ_4222_2665
Score total	:	6455.3
Score for star read(s)	:	3.9
Score for read counts	:	6444.8
Score for mfe	:	2
Score for randfold	:	1.6
Score for cons. seed	:	3
Total read count	:	12653
Mature read count	:	12650
Loop read count	:	0
Star read count	:	3

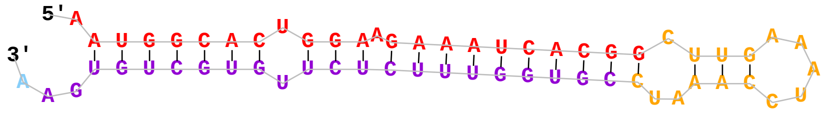

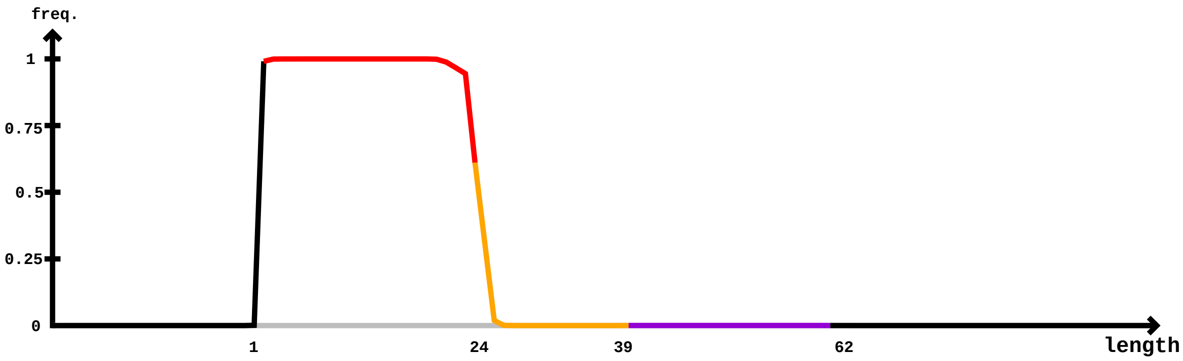

Mature **Star**

```
5'- gugccuuacuuaccgcucuuaauggcacuggaagaaaucacggcuugaaauccaaaucgugguuucucuugugcugugaagagcgguuggacaucucuacauannnnnnnnn  -3'   obs
    gugccuuacuuaccgcucuuaauggcacuggaagaaaucacggcuugaaauccaaauccgugguuucucuugugcugugaagagcgguuggacaucucuacauannnnnnnnn         exp
    ........((.(((((((((.(((((((.(((.((((((((((.(((.....)))..))))))))))))).))))))).))))))))).))......................   reads   mm   sample
    ....................aauggcacuggaagaaau...........................................................................   2   0   Ta1
    ....................aauggcacuggaagaaauc..........................................................................   3   0   Ta1
    ....................aaugUcacuggaagaaauc..........................................................................   2   1   Ta1
    ....................aaugAcacuggaagaaauca.........................................................................   2   1   Ta1
    ....................Cauggcacuggaagaaauca.........................................................................   1   1   Ta1
    ....................aaugUcacuggaagaaauca.........................................................................   1   1   Ta1
    ....................aauggcacuggaagaaauca.........................................................................   50  0   Ta1
    ....................aauggcacuggaagaaaucac........................................................................   9   0   Ta1
    ....................Gauggcacuggaagaaaucacg.......................................................................   2   1   Ta1
    ....................aauAgcacuggaagaaaucacg.......................................................................   1   1   Ta1
    ....................aauggUacuggaagaaaucacg.......................................................................   1   1   Ta1
    ....................aauggcacuAgaagaaaucacg.......................................................................   1   1   Ta1
    ....................aauggcaUuggaagaaaucacg.......................................................................   1   1   Ta1
    ....................aaCggcacuggaagaaaucacg.......................................................................   3   1   Ta1
    ....................aauggcacuggaagaaaucacg.......................................................................   618 0   Ta1
    ....................aaugAcacuggaagaaaucacg.......................................................................   3   1   Ta1
    ....................aauggcacuggGagaaaucacg.......................................................................   1   1   Ta1
    ....................Nauggcacuggaagaaaucacg.......................................................................   2   1   Ta1
    ....................Uauggcacuggaagaaaucacg.......................................................................   1   1   Ta1
    ....................aCuggcacuggaagaaaucacg.......................................................................   1   1   Ta1
    ....................aauggcacuggaagaaCucacg.......................................................................   4   1   Ta1
    ....................aaugUcacuggaagaaaucacg.......................................................................   20  1   Ta1
    ....................aauggcacuggaagaaaucacU.......................................................................   2   1   Ta1
    ....................aaugCcacuggaagaaaucacg.......................................................................   3   1   Ta1
    ....................aauggcacugUaagaaaucacg.......................................................................   1   1   Ta1
    ....................aauUgcacuggaagaaaucacg.......................................................................   1   1   Ta1
    ....................Cauggcacuggaagaaaucacg.......................................................................   17  1   Ta1
    ....................aauggcacuggaagaaCucacgg......................................................................   2   1   Ta1
    ....................aauggcacuggUagaaaucacgg......................................................................   1   1   Ta1
    ....................aCuggcacuggaagaaaucacgg......................................................................   2   1   Ta1
    ....................aauggcacuggaagaaaucacgA......................................................................   5   1   Ta1
    ....................aaGggcacuggaagaaaucacgg......................................................................   1   1   Ta1
    ....................aaugCcacuggaagaaaucacgg......................................................................   1   1   Ta1
```

```
                    Mature                 Star
gugccuuacuuaccgcucuuaauggcacuggaagaaaucacggcuugaaauccaaauccgugguuucucuugugcugugaagagcgguuggacaucucuacauannnnnnnnn
....................aaCggcacuggaagaaaucacgg......................................................................  4     1  Ta1
....................aauggcacuggaagaaaucacgU......................................................................  12    1  Ta1
....................aauggcUcuggaagaaaucacgg......................................................................  1     1  Ta1
....................aauUgcacuggaagaaaucacgg......................................................................  3     1  Ta1
....................Gauggcacuggaagaaaucacgg......................................................................  1     1  Ta1
....................Cauggcacuggaagaaaucacgg......................................................................  9     1  Ta1
....................aUuggcacuggaagaaaucacgg......................................................................  1     1  Ta1
....................aaugUcacuggaagaaaucacgg......................................................................  16    1  Ta1
....................aaugAcacuggaagaaaucacgg......................................................................  4     1  Ta1
....................aauggcacuggaagaaaucacAg......................................................................  1     1  Ta1
....................aauggcCcuggaagaaaucacgg......................................................................  1     1  Ta1
....................aauggcacuggaagaaaucacgg......................................................................  552   0  Ta1
....................Cauggcacuggaagaaaucacggc.....................................................................  1     1  Ta1
....................aauggcacuggaagaaaucacggU.....................................................................  35    1  Ta1
....................aauggcaUuggaagaaaucacggc.....................................................................  1     1  Ta1
....................aCuggcacuggaagaaaucacggc.....................................................................  1     1  Ta1
....................Uauggcacuggaagaaaucacggc.....................................................................  1     1  Ta1
....................aauggcacuggaagaaaucacggc.....................................................................  105   0  Ta1
....................aauggcacuggaagaaaucacggA.....................................................................  24    1  Ta1
....................aaugUcacuggaagaaaucacggc.....................................................................  5     1  Ta1
....................Uauggcacuggaagaaaucacggcu....................................................................  1     1  Ta1
....................aauggcacuggaagaaaucacggcu....................................................................  31    0  Ta1
....................Cauggcacuggaagaaaucacggcu....................................................................  1     1  Ta1
....................aaugUcacuggaagaaaucacggcu....................................................................  1     1  Ta1
....................aauggcacuggaagaaaucacgUcu....................................................................  1     1  Ta1
....................aauggcacugAaagaaaucacggcu....................................................................  1     1  Ta1
....................aauggcacuggaagaaaucacggcuA...................................................................  5     1  Ta1
.....................auggcacuggaagaaaucacg.......................................................................  4     0  Ta1
.....................auggGacuggaagaaaucacg.......................................................................  1     1  Ta1
.....................auggcacuggaagaaaucacgg......................................................................  1     0  Ta1
.....................auCgcacuggaagaaaucacggc.....................................................................  1     1  Ta1

...................Caauggcacuggaagaaaucacg.......................................................................  2     1  Ch1
...................Caauggcacuggaagaaaucacgg......................................................................  3     1  Ch1
...................uaauggcacuggaagaaaucacgg......................................................................  2     0  Ch1
....................aauggcacuggaagaaau...........................................................................  1     0  Ch1
....................Cauggcacuggaagaaauc..........................................................................  1     1  Ch1
....................aauggcacuggaagaaauc..........................................................................  17    0  Ch1
....................aauggcacuggaagaaaCc..........................................................................  1     1  Ch1
....................aaugUcacuggaagaaauc..........................................................................  1     1  Ch1
....................aauggcacuggaagaaauca.........................................................................  37    0  Ch1
....................aaugUcacuggaagaaauca.........................................................................  1     1  Ch1
....................aauggcacAggaagaaauca.........................................................................  1     1  Ch1
....................aaugAcacuggaagaaaucac........................................................................  2     1  Ch1
....................aaugUcacuggaagaaaucac........................................................................  1     1  Ch1
....................aauggcacuggaagaaaucac........................................................................  78    0  Ch1
....................Uauggcacuggaagaaaucac........................................................................  1     1  Ch1
....................Cauggcacuggaagaaaucac........................................................................  1     1  Ch1
....................aauggcacuggaagaaaucGcg.......................................................................  1     1  Ch1
....................aaugUcacuggaagaaaucacg.......................................................................  63    1  Ch1
....................aauggcacuggaagaaGucacg.......................................................................  3     1  Ch1
....................aauggcacuggaagaaaGcacg.......................................................................  1     1  Ch1
....................aauggcacuggaagaaaucacU.......................................................................  4     1  Ch1
....................Cauggcacuggaagaaaucacg.......................................................................  30    1  Ch1
....................Nauggcacuggaagaaaucacg.......................................................................  1     1  Ch1
....................aauggcacuggaagaaaucacC.......................................................................  1     1  Ch1
....................aauggcacuggaCgaaaucacg.......................................................................  1     1  Ch1
....................aauggcGcuggaagaaaucacg.......................................................................  1     1  Ch1
....................aauggcacuggaagaaaucacg.......................................................................  1625  0  Ch1
....................aauggcacuggaagaUaucacg.......................................................................  1     1  Ch1
....................aauggcUcuggaagaaaucacg.......................................................................  1     1  Ch1
....................aauggcacuAgaagaaaucacg.......................................................................  1     1  Ch1
....................aauggcacuggaagaaauUacg.......................................................................  1     1  Ch1
....................Gauggcacuggaagaaaucacg.......................................................................  1     1  Ch1
....................aauggcacuggaaAaaaucacg.......................................................................  1     1  Ch1
....................aauUgcacuggaagaaaucacg.......................................................................  5     1  Ch1
....................aUuggcacuggaagaaaucacg.......................................................................  1     1  Ch1
....................aauggcacuUgaagaaaucacg.......................................................................  1     1  Ch1
....................aauggcacuggaagaaCucacg.......................................................................  2     1  Ch1
....................aauggcacuggUagaaaucacg.......................................................................  2     1  Ch1
```

Mature Star

```
gugccuuacuuaccgcucuuaauggcacuggaagaaaucacggcuugaaaauccaaauccgugguuucucuugugcugugaagagcgguuggacaucucuacauannnnnnnnnn
....................aauggcacuggaagaaaucaUg.........................................................................   3     1   Ch1
....................aaCggcacuggaagaaaucacg.........................................................................   4     1   Ch1
....................aauggcacuCgaagaaaucacg.........................................................................   1     1   Ch1
....................aauggcacuggaagaaaucacA.........................................................................   8     1   Ch1
....................Uauggcacuggaagaaaucacg.........................................................................   9     1   Ch1
....................aaugCcacuggaagaaaucacg.........................................................................   11    1   Ch1
....................aaAggcacuggaagaaaucacg.........................................................................   1     1   Ch1
....................aaugAcacuggaagaaaucacg.........................................................................   10    1   Ch1
....................aauggUacuggaagaaaucacg.........................................................................   2     1   Ch1
....................aauggcacAggaagaaaucacg.........................................................................   2     1   Ch1
....................aauggcacuggaCgaaaucacgg........................................................................   2     1   Ch1
....................aaugCcacuggaagaaaucacgg........................................................................   10    1   Ch1
....................Cauggcacuggaagaaaucacgg........................................................................   48    1   Ch1
....................aauggcacCggaagaaaucacgg........................................................................   1     1   Ch1
....................aGuggcacuggaagaaaucacgg........................................................................   2     1   Ch1
....................aaAggcacuggaagaaaucacgg........................................................................   1     1   Ch1
....................aauggcacuggaagaaaucacgA........................................................................   20    1   Ch1
....................aauggcacuggaagUaaucacgg........................................................................   1     1   Ch1
....................aauggcacugUaagaaaucacgg........................................................................   1     1   Ch1
....................aauggUacuggaagaaaucacgg........................................................................   3     1   Ch1
....................aaugAcacuggaagaaaucacgg........................................................................   18    1   Ch1
....................aaCggcacuggaagaaaucacgg........................................................................   5     1   Ch1
....................aauggcacuggaagaaaucacgC........................................................................   6     1   Ch1
....................aauggcacuggaagaaaGcacgg........................................................................   1     1   Ch1
....................aauggcacuggaagaaauAacgg........................................................................   1     1   Ch1
....................aauggcacuggGagaaaucacgg........................................................................   2     1   Ch1
....................aauggcacuggaagaUaucacgg........................................................................   4     1   Ch1
....................aauggcacuggUagaaaucacgg........................................................................   2     1   Ch1
....................aauggcacuggaagaaaucaAgg........................................................................   1     1   Ch1
....................aUuggcacuggaagaaaucacgg........................................................................   1     1   Ch1
....................aauggcacAggaagaaaucacgg........................................................................   2     1   Ch1
....................aauggcacuggaUgaaaucacgg........................................................................   1     1   Ch1
....................Nauggcacuggaagaaaucacgg........................................................................   2     1   Ch1
....................aauggcacuggaagaaaucacgg........................................................................   2535  0   Ch1
....................Uauggcacuggaagaaaucacgg........................................................................   8     1   Ch1
....................aauggcacuggaagaaaCcacgg........................................................................   1     1   Ch1
....................aauggcacuggaagaaaucaUgg........................................................................   3     1   Ch1
....................aauggcaUuggaagaaaucacgg........................................................................   2     1   Ch1
....................aauggcacuggaagaGaucacgg........................................................................   1     1   Ch1
....................aauggcacuggaGgaaaucacgg........................................................................   1     1   Ch1
....................aaugUcacuggaagaaaucacgg........................................................................   100   1   Ch1
....................aauggcacuggaagaaaucacgU........................................................................   14    1   Ch1
....................aauggcacuggaagaaaucacUg........................................................................   2     1   Ch1
....................aauggcacuggaagaaaAcacgg........................................................................   1     1   Ch1
....................aauggcacuggaagaaGucacgg........................................................................   1     1   Ch1
....................aauUgcacuggaagaaaucacgg........................................................................   6     1   Ch1
....................aauggcacuggaagaaCucacgg........................................................................   10    1   Ch1
....................aCuggcacuggaagaaaucacgg........................................................................   7     1   Ch1
....................aauggcacuggaagaaaucacAg........................................................................   3     1   Ch1
....................Gauggcacuggaagaaaucacgg........................................................................   5     1   Ch1
....................aauggcacuggaagaaaucacgAc.......................................................................   3     1   Ch1
....................aauggcacuggaagaaaucacgCc.......................................................................   2     1   Ch1
....................aauggcacuggaagaaaucacggA.......................................................................   21    1   Ch1
....................aauggUacuggaagaaaucacggc.......................................................................   3     1   Ch1
....................aaugCcacuggaagaaaucacggc.......................................................................   3     1   Ch1
....................aauggcacuggaagaaaucacggc.......................................................................   558   0   Ch1
....................aaugAcacuggaagaaaucacggc.......................................................................   5     1   Ch1
....................aauggcacuggaCgaaaucacggc.......................................................................   1     1   Ch1
....................aauggcacuggaagaaaucacggU.......................................................................   116   1   Ch1
....................aaugUcacuggaagaaaucacggc.......................................................................   23    1   Ch1
....................Cauggcacuggaagaaaucacggc.......................................................................   13    1   Ch1
....................aauCgcacuggaagaaaucacggc.......................................................................   1     1   Ch1
....................aauUgcacuggaagaaaucacggc.......................................................................   1     1   Ch1
....................aGuggcacuggaagaaaucacggc.......................................................................   1     1   Ch1
....................aauggcacuggaagaaCucacggc.......................................................................   3     1   Ch1
....................aauggcaUuggaagaaaucacggc.......................................................................   1     1   Ch1
....................aauggcacugUaagaaaucacggc.......................................................................   1     1   Ch1
....................aaugUcacuggaagaaaucacggcu......................................................................   4     1   Ch1
....................aauggcacuggaagaaaucacggGu......................................................................   1     1   Ch1
....................aauggcacuggaagaaaucacggUu......................................................................   2     1   Ch1
```

Mature   Star

```
gugccuuacuuaccgcucuuaauggcacuggaagaaaucacggcuugaaauccaaauccgugguuucucuugugcugugaagagcgguuggacaucucuacauannnnnnnnn
....................aauggcacuggaagaaaucacggcA....................................................................  31   1   Ch1
....................aauggUacuggaagaaaucacggcu....................................................................  1    1   Ch1
....................aaugAcacuggaagaaaucacggcu....................................................................  1    1   Ch1
....................aauggcacuggaagaaaucacggAu....................................................................  1    1   Ch1
....................Cauggcacuggaagaaaucacggcu....................................................................  2    1   Ch1
....................aauggcacuggaagaaaucacggcu....................................................................  65   0   Ch1
....................aauggcacuggaagaaaucacggcC....................................................................  9    1   Ch1
....................aauggcacuggaagaaaucacggcG....................................................................  1    1   Ch1
....................aauggcacuggaagaaaucacggcuu...................................................................  1    0   Ch1
.....................auggcacuggaagCaauca.........................................................................  1    1   Ch1
.....................auggcacuggaagaaauca.........................................................................  1    0   Ch1
.....................auggcacuAgaagaaaucacg.......................................................................  1    1   Ch1
.....................Cuggcacuggaagaaaucacg.......................................................................  1    1   Ch1
.....................auggGacuggaagaaaucacg.......................................................................  1    1   Ch1
.....................auggcacuggaagaaaucacg.......................................................................  9    0   Ch1
.....................auggUacuggaagaaaucacg.......................................................................  1    1   Ch1
.....................auggcacuggaagaaaucacgg......................................................................  19   0   Ch1
.....................auggUacuggaagaaaucacgg......................................................................  1    1   Ch1
.....................auggcacuggaagCaaucacgg......................................................................  1    1   Ch1
.....................augUcacuggaagaaaucacgg......................................................................  1    1   Ch1
.....................Cuggcacuggaagaaaucacgg......................................................................  2    1   Ch1
.....................auCgcacuggaagaaaucacgg......................................................................  1    1   Ch1
.....................auggGacuggaagaaaucacgg......................................................................  1    1   Ch1
.....................auggcacuggaagaaCucacgg......................................................................  1    1   Ch1
.....................auggcacuggaagaaaucacggU.....................................................................  2    1   Ch1
.....................auCgcacuggaagaaaucacggc.....................................................................  1    1   Ch1
.....................auggcacuggaagaaaucacggc.....................................................................  6    0   Ch1
.....................auggcacuggaagaaaucacggcu....................................................................  1    0   Ch1
......................uggcacuggaagaaGucacggcu....................................................................  1    1   Ch1
........................gcacuggaagaaaucacggU.....................................................................  1    1   Ch1
..........................................................cgugguuucucuugugcugug..................................  1    0   Ch1
...........................................................gugguuucucuugugcugug..................................  1    0   Ch1

....................aauggcacuggaagaaauc..........................................................................  3    0   He1
....................aauggcacuggaagaaauca.........................................................................  1    0   He1
....................aaugUcacuggaagaaaucac........................................................................  1    1   He1
....................Cauggcacuggaagaaaucac........................................................................  1    1   He1
....................aauggcacuggaagaaaucac........................................................................  1    0   He1
....................aauUgcacuggaagaaaucacg.......................................................................  1    1   He1
....................aauggcacuggaagaaaucaAg.......................................................................  1    1   He1
....................aauggcacuggaagaaaucacg.......................................................................  57   0   He1
....................aaugUcacuggaagaaaucacg.......................................................................  5    1   He1
....................Cauggcacuggaagaaaucacgg......................................................................  2    1   He1
....................aauggcacuggaagaaaucacgU......................................................................  2    1   He1
....................aaugUcacuggaagaaaucacgg......................................................................  2    1   He1
....................aauUgcacuggaagaaaucacgg......................................................................  1    1   He1
....................aauggcacuggaagaaauUacgg......................................................................  1    1   He1
....................aauggcacuggaagaaaucacgg......................................................................  100  0   He1
....................aauggcacuggaaAaaaucacgg......................................................................  1    1   He1
....................aaugAcacuggaagaaaucacgg......................................................................  3    1   He1
....................aaugCcacuggaagaaaucacgg......................................................................  1    1   He1
....................aauggcacuggaagaaaucacggc.....................................................................  10   0   He1
....................aauggcaUuggaagaaaucacggc.....................................................................  1    1   He1
....................aauggcacuggaagaaaucacggU.....................................................................  3    1   He1
....................aaugCcacuggaagaaaucacggcu....................................................................  1    1   He1
....................aauggcacuggaagaaaucacggcC....................................................................  1    1   He1
....................aauggcacuggaagaaaucacggcu....................................................................  1    0   He1
.....................auggcacuggaagaaaucacgg......................................................................  1    0   He1
.....................auggcacuggaagaaaucacggc.....................................................................  1    0   He1

...................Caauggcacuggaagaaauca.........................................................................  1    1   Bl1
....................Cauggcacuggaagaaau...........................................................................  1    1   Bl1
....................aauggcacuggaagaaau...........................................................................  8    0   Bl1
....................aauggUacuggaagaaau...........................................................................  1    1   Bl1
....................aaugUcacuggaagaaauc..........................................................................  1    1   Bl1
....................aauggcacuggaagaaauc..........................................................................  82   0   Bl1
....................Uauggcacuggaagaaauc..........................................................................  1    1   Bl1
....................Cauggcacuggaagaaauc..........................................................................  1    1   Bl1
....................aaugAcacuggaagaaauc..........................................................................  1    1   Bl1
....................aauggcacuggaagaaCuc..........................................................................  1    1   Bl1
```

```
                    Mature                                Star
gugccuuacuuaccgcucuuaauggcacuggaagaaaucacggcuugaaauccaaauccgugguuucucuugugcugugaagagcgguuggacaucucuacauannnnnnnnn
....................aauggcacuggaagaaauU..........................................................................   1     1     Bl1
....................aauggcGcuggaagaaauca.........................................................................   1     1     Bl1
....................aauggcacCggaagaaauca.........................................................................   1     1     Bl1
....................Cauggcacuggaagaaauca.........................................................................   1     1     Bl1
....................aaugUcacuggaagaaauca.........................................................................   7     1     Bl1
....................aauggcacuggaagaaauca.........................................................................   99    0     Bl1
....................aaugCcacuggaagaaauca.........................................................................   1     1     Bl1
....................aauggcacuggaagaaaucC.........................................................................   1     1     Bl1
....................aaugAcacuggaagaaaucac........................................................................   1     1     Bl1
....................aaCggcacuggaagaaaucac........................................................................   2     1     Bl1
....................Cauggcacuggaagaaaucac........................................................................   2     1     Bl1
....................aaugUcacuggaagaaaucac........................................................................   6     1     Bl1
....................Uauggcacuggaagaaaucac........................................................................   1     1     Bl1
....................aauggcacuggaagaaaucac........................................................................   109   0     Bl1
....................aauggUacuggaagaaaucac........................................................................   1     1     Bl1
....................aaugCcacuggaagaaaucac........................................................................   1     1     Bl1
....................aaugUcacuggaagaaaucacg.......................................................................   9     1     Bl1
....................aauggcacuggaagaaauUacg.......................................................................   2     1     Bl1
....................aaugAcacuggaagaaaucacg.......................................................................   1     1     Bl1
....................aaugCcacuggaagaaaucacg.......................................................................   2     1     Bl1
....................aauggcacuggaagaaaucacA.......................................................................   6     1     Bl1
....................aauggcacuggaagaaCucacg.......................................................................   2     1     Bl1
....................Cauggcacuggaagaaaucacg.......................................................................   4     1     Bl1
....................aauggcacAggaagaaaucacg.......................................................................   1     1     Bl1
....................aauggcacuggaCgaaaucacg.......................................................................   1     1     Bl1
....................Gauggcacuggaagaaaucacg.......................................................................   2     1     Bl1
....................aauggcacCggaagaaaucacg.......................................................................   1     1     Bl1
....................aauggcacuggaagGaaucacg.......................................................................   1     1     Bl1
....................aauggcacuggaagaaaucacg.......................................................................   250   0     Bl1
....................aauggcacuggUagaaaucacg.......................................................................   1     1     Bl1
....................aauggUacuggaagaaaucacgg......................................................................   1     1     Bl1
....................aauggcacuggaagaaaucacgg......................................................................   122   0     Bl1
....................aaugAcacuggaagaaaucacgg......................................................................   1     1     Bl1
....................aauggcacuggaagaaCucacgg......................................................................   1     1     Bl1
....................aaugUcacuggaagaaaucacgg......................................................................   5     1     Bl1
....................aauggcacuggaagaaaucacggc.....................................................................   12    0     Bl1
....................aauggcacuggaagaaaucacggU.....................................................................   4     1     Bl1
....................aaugUcacuggaagaaaucacggc.....................................................................   1     1     Bl1
....................aauggcacuggaagaaaucacUgcu....................................................................   1     1     Bl1
....................aauggcacuggaagaaaucacggcu....................................................................   1     0     Bl1
.....................auggcacuggUagaaauc..........................................................................   1     1     Bl1
.....................auggcacuggaagaaauca.........................................................................   1     0     Bl1
.....................auggcacuggaagaaaucac........................................................................   2     0     Bl1
.....................auggcacuggaagaaaucacg.......................................................................   4     0     Bl1
.....................auggcacuggaagaaaucacgg......................................................................   1     0     Bl1
......................uggcacuggaagaaGuca.........................................................................   1     1     Bl1

....................aauggcacuggaagaaauc..........................................................................   1     0     Br1
....................aauggcacuggaagaaauca.........................................................................   2     0     Br1
....................aauggcacuggaagaaaucac........................................................................   1     0     Br1
....................aauggcacuggaagaaaucacg.......................................................................   40    0     Br1
....................aauggcCcuggaagaaaucacg.......................................................................   1     1     Br1
....................aauggcacuggaagaaaucacA.......................................................................   1     1     Br1
....................aauUgcacuggaagaaaucacg.......................................................................   1     1     Br1
....................aaugUcacuggaagaaaucacgg......................................................................   4     1     Br1
....................aauggcacuggaagaaaucacgg......................................................................   96    0     Br1
....................aauggcacuggaagaaaucacgU......................................................................   1     1     Br1
....................Nauggcacuggaagaaaucacgg......................................................................   1     1     Br1
....................aCuggcacuggaagaaaucacgg......................................................................   1     1     Br1
....................Cauggcacuggaagaaaucacgg......................................................................   2     1     Br1
....................aauggcacuggaagaaaucacgA......................................................................   2     1     Br1
....................aauggUacuggaagaaaucacggc.....................................................................   1     1     Br1
....................aauggcacuggaagaaaucacgCc.....................................................................   1     1     Br1
....................aauggcacuggaagaaCucacggc.....................................................................   1     1     Br1
....................aauggcacuggaagaaaucacggc.....................................................................   58    0     Br1
....................Uauggcacuggaagaaaucacggc.....................................................................   1     1     Br1
....................aaugCcacuggaagaaaucacggc.....................................................................   2     1     Br1
....................aauggcacuggaagaaaucacggU.....................................................................   2     1     Br1
....................aaCggcacuggaagaaaucacggc.....................................................................   1     1     Br1
....................aauggcacuggaagaaaucacggcu....................................................................   2     0     Br1
```

Mature **Star**

```
gugccuuacuuaccgcucuuaauggcacuggaagaaaucacggcuugaaauccaaauccgugguuucucuugugcugugaagagcgguuggacaucucuacauannnnnnnnn
....................aauggcacuggaagaaaucacggcA....................................................................   1     1    Br1
.....................auggcacuggaagaaaucac........................................................................   1     0    Br1
.....................auggcacuggaagaaaucacgg......................................................................   1     0    Br1
.....................auggcacuggaagaaaucacggc.....................................................................   1     0    Br1

....................aauggcacuggaagaaau...........................................................................   1     0    Le1
....................aauggcacuggaagaaauc..........................................................................   10    0    Le1
....................aaugAcacuggaagaaauca.........................................................................   1     1    Le1
....................aauggcacuUgaagaaauca.........................................................................   1     1    Le1
....................aauggcacuggaagaaauca.........................................................................   22    0    Le1
....................aauggcacuggaagaaaucac........................................................................   25    0    Le1
....................aauggcacGggaagaaaucacg.......................................................................   1     1    Le1
....................aauggcacuggaagaaaucacA.......................................................................   8     1    Le1
....................aauggcacuggaCgaaaucacg.......................................................................   1     1    Le1
....................aauggcacuggaagaaaucacU.......................................................................   1     1    Le1
....................aauUgcacuggaagaaaucacg.......................................................................   3     1    Le1
....................aauggcacuggaagaaaucacg.......................................................................   413   0    Le1
....................aauggcacugAaagaaaucacg.......................................................................   1     1    Le1
....................aauggcaUuggaagaaaucacg.......................................................................   1     1    Le1
....................Gauggcacuggaagaaaucacg.......................................................................   1     1    Le1
....................aauggcacuggaagaaaucacC.......................................................................   1     1    Le1
....................aauggcacuggaagaaCucacg.......................................................................   2     1    Le1
....................aauggcacAggaagaaaucacg.......................................................................   1     1    Le1
....................aaugUcacuggaagaaaucacg.......................................................................   17    1    Le1
....................Cauggcacuggaagaaaucacg.......................................................................   11    1    Le1
....................aCuggcacuggaagaaaucacg.......................................................................   1     1    Le1
....................aauggcacuggaagaaaucacAg......................................................................   1     1    Le1
....................aauggcacAggaagaaaucacgg......................................................................   1     1    Le1
....................aUuggcacuggaagaaaucacgg......................................................................   1     1    Le1
....................aauUgcacuggaagaaaucacgg......................................................................   5     1    Le1
....................aaugCcacuggaagaaaucacgg......................................................................   3     1    Le1
....................aaugUcacuggaagaaaucacgg......................................................................   29    1    Le1
....................aaugAcacuggaagaaaucacgg......................................................................   3     1    Le1
....................aauggcacuggaagaaCucacgg......................................................................   3     1    Le1
....................aauAgcacuggaagaaaucacgg......................................................................   1     1    Le1
....................aauggcacuggaagaaauUacgg......................................................................   1     1    Le1
....................aauggcacuggaagaaaucacUg......................................................................   1     1    Le1
....................aauggcacuggaagUaaucacgg......................................................................   1     1    Le1
....................aauggcacuggaUgaaaucacgg......................................................................   1     1    Le1
....................aauggcGcuggaagaaaucacgg......................................................................   1     1    Le1
....................aauggcacuggaCgaaaucacgg......................................................................   1     1    Le1
....................aauggcacuggaagaaaucacgA......................................................................   9     1    Le1
....................aauggcacuggaagaaaucacgU......................................................................   2     1    Le1
....................aauggcacuggaagaaaucaUgg......................................................................   1     1    Le1
....................Uauggcacuggaagaaaucacgg......................................................................   4     1    Le1
....................aauggcaUuggaagaaaucacgg......................................................................   1     1    Le1
....................aaCggcacuggaagaaaucacgg......................................................................   1     1    Le1
....................aauggcacuggCagaaaucacgg......................................................................   1     1    Le1
....................aauggcacuggaagaaaucacgg......................................................................   724   0    Le1
....................Cauggcacuggaagaaaucacgg......................................................................   11    1    Le1
....................aCuggcacuggaagaaaucacggc.....................................................................   1     1    Le1
....................aauggcacuggaagaaaucacggU.....................................................................   36    1    Le1
....................aauggcacuggaagaaaucacggc.....................................................................   105   0    Le1
....................aaugAcacuggaagaaaucacggc.....................................................................   1     1    Le1
....................aauggcacuggaagaaaucacgAc.....................................................................   1     1    Le1
....................aaugUcacuggaagaaaucacggc.....................................................................   7     1    Le1
....................aauggcacuggCagaaaucacggc.....................................................................   1     1    Le1
....................aauggcacuggaagaaaucacggA.....................................................................   11    1    Le1
....................aauggcacAggaagaaaucacggc.....................................................................   1     1    Le1
....................Cauggcacuggaagaaaucacggc.....................................................................   3     1    Le1
....................aaugCcacuggaagaaaucacggc.....................................................................   2     1    Le1
....................aauggcacuggaagaaGucacggc.....................................................................   1     1    Le1
....................Uauggcacuggaagaaaucacggc.....................................................................   1     1    Le1
....................aauggcacuggaagaaaucacggcC....................................................................   5     1    Le1
....................aauggcacuggaagaaaucacggcA....................................................................   5     1    Le1
....................aauggcacuggaagaaaucacggUu....................................................................   1     1    Le1
....................aauggcacuAgaagaaaucacggcu....................................................................   1     1    Le1
....................aaugUcacuggaagaaaucacggcu....................................................................   1     1    Le1
....................aauggcacuggaagaaaucacggcu....................................................................   14    0    Le1
....................aauggcacuggaagaaaucacggcuuU..................................................................   1     1    Le1
```

```
                    Mature                                Star
gugccuuacuuaccgcucuuaauggcacuggaagaaaucacggcuugaaauccaaauccgugguuucucuugugcugugaagagcgguuggacaucucuacauannnnnnnnn
.....................auggUacuggaagaaaucacg.......................................................................   1     1    Le1
.....................auggcacuggaagaaaucacg.......................................................................   2     0    Le1
.....................auCgcacuggaagaaaucacgg......................................................................   1     1    Le1
.....................auggcacuggaagaaaucacgg......................................................................   4     0    Le1
.....................auggcacuggaagaaaucacggc.....................................................................   2     0    Le1
.....................auggcacuggaagaaaucacggcG....................................................................   1     1    Le1
......................uggcacuggaagaaGucacggcu....................................................................   1     1    Le1

...................Caauggcacuggaagaaaucacg.......................................................................   1     1    Le5
...................Caauggcacuggaagaaaucacgg......................................................................   2     1    Le5
....................aauggcacuggaagaaau...........................................................................   1     0    Le5
....................aauggcacuggaagaaauc..........................................................................   6     0    Le5
....................aaugUcacuggaagaaauca.........................................................................   1     1    Le5
....................aauggcGcuggaagaaauca.........................................................................   1     1    Le5
....................aauggcacuggaagaaauca.........................................................................   32    0    Le5
....................aauggcacuggaagaaaucac........................................................................   30    0    Le5
....................aauggUacuggaagaaaucac........................................................................   1     1    Le5
....................Uauggcacuggaagaaaucac........................................................................   1     1    Le5
....................Cauggcacuggaagaaaucacg.......................................................................   9     1    Le5
....................aauggcacuggaagaaaucacg.......................................................................   776   0    Le5
....................aaCggcacuggaagaaaucacg.......................................................................   2     1    Le5
....................aUuggcacuggaagaaaucacg.......................................................................   1     1    Le5
....................Uauggcacuggaagaaaucacg.......................................................................   3     1    Le5
....................aauggcacuggaagaaaucacA.......................................................................   15    1    Le5
....................Nauggcacuggaagaaaucacg.......................................................................   1     1    Le5
....................aauggUacuggaagaaaucacg.......................................................................   1     1    Le5
....................aaugUcacuggaagaaaucacg.......................................................................   24    1    Le5
....................aauggcacuggaagaaCucacg.......................................................................   1     1    Le5
....................aauggcacuggaagaaaucacC.......................................................................   1     1    Le5
....................aCuggcacuggaagaaaucacg.......................................................................   1     1    Le5
....................aauggcacuggaagaaaucacU.......................................................................   4     1    Le5
....................aauggcacuggaagaaaucGcg.......................................................................   1     1    Le5
....................aauCgcacuggaagaaaucacg.......................................................................   1     1    Le5
....................aauUgcacuggaagaaaucacg.......................................................................   1     1    Le5
....................aaugAcacuggaagaaaucacg.......................................................................   6     1    Le5
....................aaugCcacuggaagaaaucacg.......................................................................   4     1    Le5
....................aauggcacAggaagaaaucacg.......................................................................   1     1    Le5
....................aauggcacuggaUgaaaucacg.......................................................................   1     1    Le5
....................aCuggcacuggaagaaaucacgg......................................................................   5     1    Le5
....................Gauggcacuggaagaaaucacgg......................................................................   1     1    Le5
....................aauggcacuggaagaaaucacgC......................................................................   2     1    Le5
....................aauggcacuggUagaaaucacgg......................................................................   1     1    Le5
....................aauggcacuggaagaaaucGcgg......................................................................   2     1    Le5
....................Nauggcacuggaagaaaucacgg......................................................................   2     1    Le5
....................aauggcacuggaagaaaucacgU......................................................................   11    1    Le5
....................aauggcacuggaagaaaucacAg......................................................................   1     1    Le5
....................Uauggcacuggaagaaaucacgg......................................................................   9     1    Le5
....................aauggcacuggaagGaaucacgg......................................................................   2     1    Le5
....................Cauggcacuggaagaaaucacgg......................................................................   21    1    Le5
....................aauggcacuggCagaaaucacgg......................................................................   1     1    Le5
....................aauggcacuggaCgaaaucacgg......................................................................   6     1    Le5
....................aUuggcacuggaagaaaucacgg......................................................................   1     1    Le5
....................aauggcacCggaagaaaucacgg......................................................................   2     1    Le5
....................aauggcacuggaaUaaaucacgg......................................................................   1     1    Le5
....................aaCggcacuggaagaaaucacgg......................................................................   4     1    Le5
....................aaugCcacuggaagaaaucacgg......................................................................   4     1    Le5
....................aauggcacuggaagaaaucacUg......................................................................   1     1    Le5
....................aauggcacuggaagaaaucacgA......................................................................   18    1    Le5
....................aauggcacuggaagaaaucacgg......................................................................   1182  0    Le5
....................aauggcacuggaagaaCucacgg......................................................................   3     1    Le5
....................aauggcacuggaagaaauUacgg......................................................................   1     1    Le5
....................aauggcacAggaagaaaucacgg......................................................................   1     1    Le5
....................aaugAcacuggaagaaaucacgg......................................................................   5     1    Le5
....................aauggUacuggaagaaaucacgg......................................................................   2     1    Le5
....................aaugUcacuggaagaaaucacgg......................................................................   49    1    Le5
....................aauggcacuggGagaaaucacgg......................................................................   3     1    Le5
....................aaugUcacuggaagaaaucacggc.....................................................................   13    1    Le5
....................Uauggcacuggaagaaaucacggc.....................................................................   1     1    Le5
....................Cauggcacuggaagaaaucacggc.....................................................................   5     1    Le5
....................aauggcacuggaagaaaucacggA.....................................................................   15    1    Le5
```

```
                    Mature                              Star
gugccuuacuuaccgcucuuaauggcacuggaagaaaucacggcuugaaauccaaauccgugguuucucuugugcugugaagagcgguuggacaucucuacauannnnnnnnn
....................aauggcacuggaagaaaucacggc.....................................................................    160    0    Le5
....................aaugAcacuggaagaaaucacggc.....................................................................    1      1    Le5
....................aauggcacuggaCgaaaucacggc.....................................................................    1      1    Le5
....................aauggcacuggaagaaaucacggU.....................................................................    65     1    Le5
....................aauggcacuggaagaaaucacggG.....................................................................    1      1    Le5
....................aauggcacuggaagaaaucacggcC....................................................................    6      1    Le5
....................aauggcacuggaagaaaucacggcG....................................................................    2      1    Le5
....................aauggcacuggaagaaaucacggcA....................................................................    7      1    Le5
....................aauggcacuggaagaaaucacggcu....................................................................    22     0    Le5
....................aaugUcacuggaagaaaucacggcu....................................................................    2      1    Le5
....................aauggcacuggaagaaaucacggUu....................................................................    2      1    Le5
....................aauggcacuggaagaaaucacggcuu...................................................................    2      0    Le5
.....................auggcacuggaagaaaucacg.......................................................................    7      0    Le5
.....................Cuggcacuggaagaaaucacg.......................................................................    1      1    Le5
.....................auggcacuggaagaaaucaGgg......................................................................    1      1    Le5
.....................auggcacuggaagaaaucacgg......................................................................    7      0    Le5
.....................auggUacuggaagaaaucacggc.....................................................................    1      1    Le5
.....................auggcacuggaagaaaucacggU.....................................................................    1      1    Le5
.....................auCgcacuggaagaaaucacggc.....................................................................    1      1    Le5
.....................auggcacuggaagaaaucacggc.....................................................................    3      0    Le5
......................uggcacuggaagaaaucacgg......................................................................    1      0    Le5
......................Nggcacuggaagaaaucacggc.....................................................................    1      1    Le5
..........................................................cgugguuucucuAgugcuguga.................................    1      1    Le5
```

Provisional ID	: Sc28yqQ_4222_2910
Score total	: 954096
Score for star read(s)	: 3.9
Score for read counts	: 954085.8
Score for mfe	: 1.7
Score for randfold	: 1.6
Score for cons. seed	: 3
Total read count	: 1871410
Mature read count	: 1871251
Loop read count	: 0
Star read count	: 159

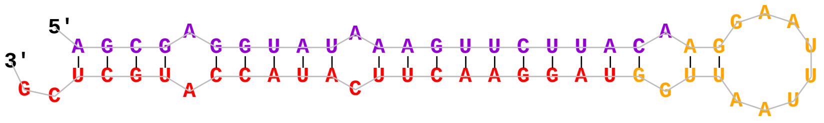

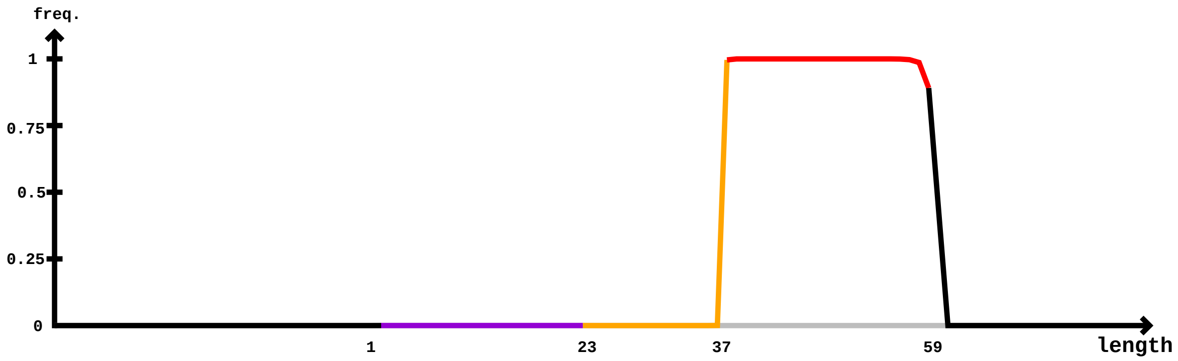

Star **Mature**

```
5'- agugaagaaaggacaacagcugggguucucgagagcgagguauaaaguucuuacaaggaauuuaauugguaggaacuucauaccaugcucgcgaugauucaaaguccacaaca   -3'    obs
    agugaagaaaggacaacagcugggguucucgagagcgagguauaaaguucuuacaaggaauuuaauugguaggaacuucauaccaugcucgcgaugauucaaaguccacaaca          exp
    ..........((((......((((((.(((.(((((.(((((.((((((((((.((........)).)))))))))).))))).))))).))).))))))..)))).......   reads   mm   sample
    .................................agcgagguCuaaaguucuu.............................................................   1       1    Le5
    .................................agcgagguauaaaguucuu.............................................................   3       0    Le5
    .................................agcgGgguauaaaguucuua............................................................   1       1    Le5
    .................................agcgagguauaaaguucuua............................................................   2       0    Le5
    .................................agcgagguauaaaguucuuaca..........................................................   13      0    Le5
    .................................Cgcgagguauaaaguucuuaca..........................................................   1       1    Le5
    ..................................gcgagguauaGaguucuuaca..........................................................   1       1    Le5
    ..................................gcgUgguauaaaguucuuacaa.........................................................   1       1    Le5
    ................................................................Uuugguaggaacuucauaccaugcucg......................   1       1    Le5
    ....................................................................Cuaggaacuucauaccaugcuc.......................   10      1    Le5
    ....................................................................Uuaggaacuucauaccaugcuc.......................   2       1    Le5
    ....................................................................guaggaacuucauaccaugcuc.......................   1       0    Le5
    ....................................................................Auaggaacuucauaccaugcucg......................   8       1    Le5
    ....................................................................Cuaggaacuucauaccaugcucg......................   65      1    Le5
    .....................................................................uaggaacuucauaccauU..........................   3       1    Le5
    .....................................................................uaggUacuucauaccaug..........................   1       1    Le5
    .....................................................................uaggaacuucauaccaug..........................   95      0    Le5
    .....................................................................uaggaacuuAauaccaug..........................   1       1    Le5
    .....................................................................uaggaacuCcauaccaugc.........................   1       1    Le5
    .....................................................................uaggaacuucauCccaugc.........................   1       1    Le5
    .....................................................................uaggaacuucauaccauUc.........................   1       1    Le5
    .....................................................................uagUaacuucauaccaugc.........................   2       1    Le5
    .....................................................................uaggaacuucauaccaugc.........................   380     0    Le5
    .....................................................................Naggaacuucauaccaugc.........................   1       1    Le5
    .....................................................................uaggUacuucauaccaugc.........................   7       1    Le5
    .....................................................................uaggaacuucauaccaugA.........................   284     1    Le5
    .....................................................................uaggaacuucauacUaugc.........................   2       1    Le5
    .....................................................................uaggaacuucCuaccaugc.........................   2       1    Le5
    .....................................................................uaggaacuucauaccaugU.........................   1       1    Le5
    .....................................................................uaggaacuucauaccaugG.........................   2       1    Le5
    .....................................................................uaggGacuucauaccaugc.........................   3       1    Le5
    .....................................................................uaggaacuucauaUcaugc.........................   2       1    Le5
    .....................................................................uaCgaacuucauaccaugc.........................   2       1    Le5
```

Star Mature

agugaagaaaggacaacagcuggguucucgagagcgagguauaaaguucuuacaaggaauuuaauugguaggaacuucauaccaugcucgcgaugauucaaaguccacaaca			
....Caggaacuucauaccaugcu........................	3	1	Le5
....uaggUacuucauaccaugcu........................	45	1	Le5
....uagUaacuucauaccaugcu........................	2	1	Le5
....uaggaacuucauaccaugcG........................	9	1	Le5
....uaggaacuucCuaccaugcu........................	18	1	Le5
....uaggaacuAcauaccaugcu........................	2	1	Le5
....Aaggaacuucauaccaugcu........................	4	1	Le5
....uaCgaacuucauaccaugcu........................	7	1	Le5
....uaggaacuucauaccaugcC........................	431	1	Le5
....uaggaacuucauaccaugcA........................	253	1	Le5
....uaggaacuucauUccaugcu........................	1	1	Le5
....uaggaacuucauCccaugcu........................	4	1	Le5
....uaggaacuucauaccaugcu........................	2286	0	Le5
....uaggaacuucauaccaCgcu........................	1	1	Le5
....uaggaacuucauaccaugGu........................	1	1	Le5
....uaggaUcuucauaccaugcu........................	1	1	Le5
....uaggaacuucauaUcaugcu........................	3	1	Le5
....uCggaacuucauaccaugcu........................	9	1	Le5
....uaggaacuCcauaccaugcu........................	1	1	Le5
....uaggaacuuUauaccaugcu........................	1	1	Le5
....uGggaacuucauaccaugcu........................	2	1	Le5
....uaggGacuucauaccaugcu........................	22	1	Le5
....uaggaacuucauacUaugcu........................	1	1	Le5
....uaggaacuucauaccauUcu........................	1	1	Le5
....uaggaacGucauaccaugcu........................	1	1	Le5
....uaggCacuucauaccaugcu........................	1	1	Le5
....uaggaacuucauaAcaugcu........................	2	1	Le5
....uaggaacuucauaccUugcu........................	1	1	Le5
....uUggaacuucauaccaugcu........................	2	1	Le5
....uaggaacuucauGccaugcu........................	1	1	Le5
....uaggaacuucauaccGugcu........................	2	1	Le5
....uaggaacAucauaccaugcuc.......................	5	1	Le5
....uaggaacuucauaccGugcuc.......................	3	1	Le5
....uaggaacuucauaccaugcAc.......................	5	1	Le5
....uaggUacuucauaccaugcuc.......................	355	1	Le5
....uaggaacuucauCccaugcuc.......................	28	1	Le5
....uaggaacuucauGccaugcuc.......................	2	1	Le5
....uaggaacuCcauaccaugcuc.......................	20	1	Le5
....Naggaacuucauaccaugcuc.......................	25	1	Le5
....uaggaacuucauaUcaugcuc.......................	21	1	Le5
....uagAaacuucauaccaugcuc.......................	6	1	Le5
....uaggaacuucauUccaugcuc.......................	1	1	Le5
....uaggaacuucauaccaugcCc.......................	68	1	Le5
....uaggaacuucauaccUugcuc.......................	18	1	Le5
....uaggaacuucCuaccaugcuc.......................	153	1	Le5
....uUggaacuucauaccaugcuc.......................	12	1	Le5
....uaggaacGucauaccaugcuc.......................	13	1	Le5
....uaggaacuucauaccauUcuc.......................	20	1	Le5
....Caggaacuucauaccaugcuc.......................	26	1	Le5
....uaggaacuucauaAcaugcuc.......................	9	1	Le5
....Gaggaacuucauaccaugcuc.......................	3	1	Le5
....uaggaacuuAauaccaugcuc.......................	17	1	Le5
....uaggaacuucauaccaAgcuc.......................	3	1	Le5
....uCggaacuucauaccaugcuc.......................	96	1	Le5
....uagUaacuucauaccaugcuc.......................	65	1	Le5
....uaggaacuucaAaccaugcuc.......................	1	1	Le5
....uaggaacuucauacGaugcuc.......................	6	1	Le5
....uaggaacuucaCaccaugcuc.......................	2	1	Le5
....uaggaacuucauaccaugcuA.......................	189	1	Le5
....uaggaacuGcauaccaugcuc.......................	1	1	Le5
....uaggaacuuUauaccaugcuc.......................	84	1	Le5
....uaggaacuuGauaccaugcuc.......................	2	1	Le5
....uaggaacuucauaccaugGuc.......................	3	1	Le5
....uaggCacuucauaccaugcuc.......................	4	1	Le5
....uagCaacuucauaccaugcuc.......................	2	1	Le5
....uaggaaAuucauaccaugcuc.......................	2	1	Le5
....uaggaacuucauacUaugcuc.......................	21	1	Le5
....uaggaGcuucauaccaugcuc.......................	5	1	Le5
....uaggaacuucauaccaCgcuc.......................	3	1	Le5
....uaggaaUuucauaccaugcuc.......................	3	1	Le5

```
                                 Star                                     Mature
agugaagaaaggacaacagcugggguucucgagagcgagguauaaaguucuuacaaggaauuuaauugguaggaacuucauaccaugcucgcgaugauucaaaguccacaaca
.....................................................................uaCgaacuucauaccaugcuc.......................  114     1  Le5
.....................................................................uaUgaacuucauaccaugcuc.......................  7       1  Le5
.....................................................................uaggaacuucauaccauAcuc.......................  9       1  Le5
.....................................................................uaggaUcuucauaccaugcuc.......................  21      1  Le5
.....................................................................uaAgaacuucauaccaugcuc.......................  2       1  Le5
.....................................................................uaggaacuucauaccaugcGc.......................  16      1  Le5
.....................................................................uaggaacuucauaccCugcuc.......................  3       1  Le5
.....................................................................uaggaacuAcauaccaugcuc.......................  31      1  Le5
.....................................................................uGggaacuucauaccaugcuc.......................  11      1  Le5
.....................................................................uaggaacuucauaccaugUuc.......................  7       1  Le5
.....................................................................uaggaacCucauaccaugcuc.......................  5       1  Le5
.....................................................................Aaggaacuucauaccaugcuc.......................  41      1  Le5
.....................................................................uaggaacuucauaccaugcuG.......................  6       1  Le5
.....................................................................uaggaacuucaGaccaugcuc.......................  9       1  Le5
.....................................................................uaggGacuucauaccaugcuc.......................  238     1  Le5
.....................................................................uaggaacuucauaccaugAuc.......................  3       1  Le5
.....................................................................uaggaacuucauaccaugcuc.......................  25075   0  Le5
.....................................................................uaggaacuucauaccaugcGcg......................  108     1  Le5
.....................................................................uaggaacuucauaccauUcucg......................  140     1  Le5
.....................................................................uaggaacuucauaccaugcucA......................  2294    1  Le5
.....................................................................uaggGacuucauaccaugcucg......................  2000    1  Le5
.....................................................................uagUaacuucauaccaugcucg......................  481     1  Le5
.....................................................................uaggaacuucauacAaugcucg......................  26      1  Le5
.....................................................................uaggaaGuucauaccaugcucg......................  12      1  Le5
.....................................................................uaggaacuucauUccaugcucg......................  26      1  Le5
.....................................................................uaggaacuucauGccaugcucg......................  21      1  Le5
.....................................................................uaggaacuucauaccGugcucg......................  33      1  Le5
.....................................................................uaggaCcuucauaccaugcucg......................  6       1  Le5
.....................................................................Gaggaacuucauaccaugcucg......................  50      1  Le5
.....................................................................uaggaacuCcauaccaugcucg......................  120     1  Le5
.....................................................................uagNaacuucauaccaugcucg......................  1       1  Le5
.....................................................................uaggaaUuucauaccaugcucg......................  21      1  Le5
.....................................................................uaggaacuucauaGcaugcucg......................  8       1  Le5
.....................................................................uaggaacuucauaUcaugcucg......................  85      1  Le5
.....................................................................uaggaacuucCuaccaugcucg......................  1034    1  Le5
.....................................................................uaggaacuucauaAcaugcucg......................  57      1  Le5
.....................................................................uaAgaacuucauaccaugcucg......................  34      1  Le5
.....................................................................uaggaacuucaGaccaugcucg......................  31      1  Le5
.....................................................................uUggaacuucauaccaugcucg......................  74      1  Le5
.....................................................................uaggaacuAcauaccaugcucg......................  254     1  Le5
.....................................................................uaCgaacuucauaccaugcucg......................  817     1  Le5
.....................................................................Naggaacuucauaccaugcucg......................  140     1  Le5
.....................................................................uaggaacuucauaccaCgcucg......................  17      1  Le5
.....................................................................uagCaacuucauaccaugcucg......................  6       1  Le5
.....................................................................uaggaGcuucauaccaugcucg......................  15      1  Le5
.....................................................................Aaggaacuucauaccaugcucg......................  324     1  Le5
.....................................................................uaggaacuNcauaccaugcucg......................  4       1  Le5
.....................................................................uaggaacCucauaccaugcucg......................  41      1  Le5
.....................................................................uaggUacuucauaccaugcucg......................  2827    1  Le5
.....................................................................uaggaacuGcauaccaugcucg......................  6       1  Le5
.....................................................................uaggaacuucauaccauAcucg......................  213     1  Le5
.....................................................................uagAaacuucauaccaugcucg......................  16      1  Le5
.....................................................................uaggaacuuAauaccaugcucg......................  139     1  Le5
.....................................................................uaggaacAucauaccaugcucg......................  34      1  Le5
.....................................................................uaggaacuucauaccaugcucC......................  186     1  Le5
.....................................................................uaggCacuucauaccaugcucg......................  24      1  Le5
.....................................................................uaggaUcuucauaccaugcucg......................  153     1  Le5
.....................................................................uaggaacuucauaccCugcucg......................  16      1  Le5
.....................................................................uaggaacuucauCccaugcucg......................  210     1  Le5
.....................................................................uaggaacuucauaccaugAucg......................  12      1  Le5
.....................................................................uaggaacuucauaccaugcucg......................  197594  0  Le5
.....................................................................uGggaacuucauaccaugcucg......................  147     1  Le5
.....................................................................uaggaacuucauaccaAgcucg......................  20      1  Le5
.....................................................................uaggaacuucauacGaugcucg......................  24      1  Le5
.....................................................................uaggaacGucauaccaugcucg......................  64      1  Le5
.....................................................................uaggaacuucauaccaGgcucg......................  7       1  Le5
.....................................................................uaggaacuucauaccaugcucU......................  2272    1  Le5
.....................................................................uaggaacuucauaccaugcAcg......................  22      1  Le5
.....................................................................uaggaaAuucauaccaugcucg......................  4       1  Le5
.....................................................................uaggaacuucauaccauCcucg......................  12      1  Le5
```

Star **Mature**

```
agugaagaaaggacaacagcuggguucucgagagcgagguauaaaguucuuacaaggaauuuaauugguaggaacuucauaccaugcucgcgaugauucaaaguccacaaca
....................................................................uCggaacuucauaccaugcucg......................	643	1	Le5
....................................................................uaggaacuucaCaccaugcucg......................	42	1	Le5
....................................................................uaggaacuucauaccaugGucg......................	18	1	Le5
....................................................................uaggaacuucauaccaugcuGg......................	7	1	Le5
....................................................................uaggaacuucauaccUugcucg......................	147	1	Le5
....................................................................uaggaacuucGuaccaugcucg......................	29	1	Le5
....................................................................uaggaacuuGauaccaugcucg......................	4	1	Le5
....................................................................uaggaacuucauaccaugcCcg......................	48	1	Le5
....................................................................uaggaacuucaAaccaugcucg......................	12	1	Le5
....................................................................uaggaacuucauacUaugcucg......................	202	1	Le5
....................................................................CaggaacuucauaccaugcucG......................	200	1	Le5
....................................................................uaggaacuucauaccaugcuAg......................	98	1	Le5
....................................................................uaggaacuuUauaccaugcucg......................	284	1	Le5
....................................................................uaggaacuucauaccaugUucg......................	92	1	Le5
....................................................................uaUgaacuucauaccaugcucg......................	45	1	Le5
....................................................................uaggGacuucauaccaugcucgc.....................	1	1	Le5
....................................................................uaCgaacuucauaccaugcucgc.....................	1	1	Le5
....................................................................uaggaacuucauaccaugcucgA.....................	4233	1	Le5
....................................................................uaggaacuucauaccaugcucUc.....................	1	1	Le5
....................................................................uaggaacuucCuaccaugcucgc.....................	1	1	Le5
....................................................................uaggUacuucauaccaugcucgc.....................	3	1	Le5
....................................................................uaggaacuucauaccaugcucgc.....................	133	0	Le5
....................................................................uaggaacuucauaccaugcucgG.....................	6	1	Le5
....................................................................uaggaacuucauaccaugcucgcC....................	1	1	Le5
.....................................................................aggaacuucauaccaugc.........................	1	0	Le5
.....................................................................aggaacuucauaccaugA.........................	2	1	Le5
.....................................................................aggaGcuucauaccaugcu........................	1	1	Le5
.....................................................................aggaacuucauaccaugcu........................	4	0	Le5
.....................................................................aggaacuucauaccaugcC........................	1	1	Le5
.....................................................................aggaUcuucauaccaugcuc.......................	5	1	Le5
.....................................................................aggaacuucauaccaugcuA.......................	1	1	Le5
.....................................................................aggaacuucauaccaugcuc.......................	60	0	Le5
.....................................................................aggaacuuAauaccaugcuc.......................	3	1	Le5
.....................................................................aggaacuCcauaccaugcuc.......................	1	1	Le5
.....................................................................agCaacuucauaccaugcuc.......................	2	1	Le5
.....................................................................Cggaacuucauaccaugcuc.......................	2	1	Le5
.....................................................................aggaacuGcauaccaugcuc.......................	1	1	Le5
.....................................................................aggaacuucauaccCugcuc.......................	1	1	Le5
.....................................................................aggaacuucauaccauAcuc.......................	2	1	Le5
.....................................................................aggUacuucauaccaugcuc.......................	3	1	Le5
.....................................................................aggaacuucCuaccaugcuc.......................	2	1	Le5
.....................................................................Gggaacuucauaccaugcuc.......................	1	1	Le5
.....................................................................aggaGcuucauaccaugcuc.......................	4	1	Le5
.....................................................................aggaacuucauaccaugcucg......................	567	0	Le5
.....................................................................agCaacuucauaccaugcucg......................	23	1	Le5
.....................................................................aggaacuucauaccaugcCcg......................	2	1	Le5
.....................................................................aggaacuucauaAcaugcucg......................	4	1	Le5
.....................................................................aggaacuucauaccaAgcucg......................	1	1	Le5
.....................................................................aggaaUuucauaccaugcucg......................	3	1	Le5
.....................................................................aggaacuucauaccaugGucg......................	1	1	Le5
.....................................................................aggaacuucauaccaugcAcg......................	1	1	Le5
.....................................................................Uggaacuucauaccaugcucg......................	4	1	Le5
.....................................................................aggaacGucauaccaugcucg......................	1	1	Le5
.....................................................................aggaGcuucauaccaugcucg......................	21	1	Le5
.....................................................................aggaacuucauaccaugcucA......................	4	1	Le5
.....................................................................aggaUcuucauaccaugcucg......................	30	1	Le5
.....................................................................aggaacuucauaccGugcucg......................	4	1	Le5
.....................................................................aCgaacuucauaccaugcucg......................	5	1	Le5
.....................................................................aggaacuucauaccaugcucU......................	4	1	Le5
.....................................................................aggaacuucauaccCugcucg......................	1	1	Le5
.....................................................................aggaacuucauacGaugcucg......................	1	1	Le5
.....................................................................aggaacuucauaccaugUucg......................	3	1	Le5
.....................................................................aggaacuucauaccaugcGcg......................	1	1	Le5
.....................................................................aggaacuucauaccaGgcucg......................	1	1	Le5
.....................................................................agAaacuucauaccaugcucg......................	1	1	Le5
.....................................................................aAgaacuucauaccaugcucg......................	4	1	Le5
.....................................................................aggaacuucauacAaugcucg......................	1	1	Le5
.....................................................................aggaacuAcauaccaugcucg......................	2	1	Le5
.....................................................................aggaacuucauGccaugcucg......................	1	1	Le5
.....................................................................aggaacuucauaccUugcucg......................	2	1	Le5
```

```
                                Star                                  Mature
agugaagaaaggacaacagcuggguucucgagagcgagguauaaaguucuuacaaggaauuuaauugguaggaacuucauaccaugcucgcgaugauucaaaguccacaaca
.....................................................................aggaacuucCuaccaugcucg......................  20   1   Le5
.....................................................................aggaacuucaAaccaugcucg......................  2    1   Le5
.....................................................................aggaacuCcauaccaugcucg......................  6    1   Le5
.....................................................................aggaacuucauaccaugcucC......................  2    1   Le5
.....................................................................aggaacuucauaccauCcucg......................  3    1   Le5
.....................................................................Cggaacuucauaccaugcucg......................  24   1   Le5
.....................................................................aggaacuuAauaccaugcucg......................  11   1   Le5
.....................................................................aggaacuGcauaccaugcucg......................  2    1   Le5
.....................................................................aggaacuucGuaccaugcucg......................  1    1   Le5
.....................................................................aggaacuuUauaccaugcucg......................  3    1   Le5
.....................................................................aggUacuucauaccaugcucg......................  29   1   Le5
.....................................................................aggaacuucauaccaugcucgc.....................  1    0   Le5
.....................................................................aggaacuucauaccaugcucgA.....................  14   1   Le5
......................................................................ggaacuucauaccaugcC........................  1    1   Le5
......................................................................Ugaacuucauaccaugcu........................  1    1   Le5
......................................................................ggaacuucauaccaugcuc.......................  3    0   Le5
......................................................................ggaaGuucauaccaugcuc.......................  1    1   Le5
......................................................................ggaacuucauaccaugcucg......................  22   0   Le5
......................................................................ggaacuucauaccaugcucC......................  1    1   Le5
......................................................................ggaacuucauaccauUcucg......................  1    1   Le5
......................................................................gAaacuucauaccaugcucg......................  1    1   Le5
......................................................................ggaacuucCuaccaugcucg......................  2    1   Le5
......................................................................ggaaGuucauaccaugcucg......................  2    1   Le5
......................................................................Ugaacuucauaccaugcucg......................  3    1   Le5
......................................................................ggaacuucauaccCugcucg......................  1    1   Le5
........................................................................aacuucauaccaugcucg......................  2    0   Le5

.............caacagcuggguucucgag................................................................................  2    0   Br1
................................agcgagguauaaaguucuu.............................................................  2    0   Br1
................................agcgagguauaUaguucuu.............................................................  1    1   Br1
................................agcgUgguauaaaguucuua............................................................  1    1   Br1
................................agcgagguauaaaguucuuac...........................................................  12   0   Br1
................................agcgagguauaGaguucuuaca..........................................................  2    1   Br1
................................agcgGgguauaaaguucuuaca..........................................................  2    1   Br1
................................agAgagguauaaaguucuuaca..........................................................  1    1   Br1
................................agcgagguCuaaaguucuuaca..........................................................  1    1   Br1
................................agcgUgguauaaaguucuuaca..........................................................  3    1   Br1
................................Cgcgagguauaaaguucuuaca..........................................................  1    1   Br1
................................agcgagguauaaaguucuuaca..........................................................  65   0   Br1
................................agcgagguauaaaguucuuacaa.........................................................  2    0   Br1
.................................gcgagguauaaaguucuuaca..........................................................  3    0   Br1
.................................gcgaUguauaaaguucuuaca..........................................................  1    1   Br1
.................................gcgagguCuaaaguucuuaca..........................................................  1    1   Br1
.................................gcgagguauaaaguucuuacaa.........................................................  1    0   Br1
.................................................................Cgguaggaacuucauaccaugcucg......................  1    1   Br1
..................................................................gUuaggaacuucauaccaugcucg......................  1    1   Br1
...................................................................Cuaggaacuucauaccaugc.........................  1    1   Br1
...................................................................Cuaggaacuucauaccaugcu........................  2    1   Br1
...................................................................Auaggaacuucauaccaugcuc.......................  3    1   Br1
...................................................................Uuaggaacuucauaccaugcuc.......................  1    1   Br1
...................................................................guaggaacuucauaccaugcuc.......................  4    0   Br1
...................................................................Cuaggaacuucauaccaugcuc.......................  28   1   Br1
...................................................................Auaggaacuucauaccaugcucg......................  35   1   Br1
...................................................................guaggaacuucauaccaugcucU......................  1    1   Br1
...................................................................Cuaggaacuucauaccaugcucg......................  282  1   Br1
...................................................................guCggaacuucauaccaugcucg......................  1    1   Br1
...................................................................guaggaacuucauaccaugcucg......................  6    0   Br1
...................................................................Uuaggaacuucauaccaugcucg......................  10   1   Br1
....................................................................uaggaacuucauaccUug..........................  1    1   Br1
....................................................................uaggaacuucCuaccaug..........................  3    1   Br1
....................................................................uaggaacuucauaccaug..........................  283  0   Br1
....................................................................uaggGacuucauaccaug..........................  1    1   Br1
....................................................................uaggaacuucauaccaCg..........................  2    1   Br1
....................................................................uaggaacuCcauaccaug..........................  1    1   Br1
....................................................................uagUaacuucauaccaug..........................  1    1   Br1
....................................................................uaggUacuucauaccaug..........................  5    1   Br1
....................................................................uaCgaacuucauaccaug..........................  3    1   Br1
....................................................................uaggGacuucauaccaugc.........................  5    1   Br1
....................................................................uagUaacuucauaccaugc.........................  3    1   Br1
....................................................................uaggaacuucCuaccaugc.........................  6    1   Br1
```

Star **Mature**

agugaagaaaggacaacagcuggguucucgagagcgagguauaaaguucuuacaaggaauuuaauugguaggaacuucauaccaugcucgcgaugauucaaaguccacaaca			
....Aaggaacuucauaccaugc.........................	2	1	Br1
....uaggaacuucauaccaugc.........................	712	0	Br1
....Gaggaacuucauaccaugc.........................	1	1	Br1
....Naggaacuucauaccaugc.........................	1	1	Br1
....uaggaacuAcauaccaugc.........................	2	1	Br1
....uaggaacuucauaccaCgc.........................	2	1	Br1
....uaggaacuucauaccauAc.........................	1	1	Br1
....uaggUacuucauaccaugc.........................	7	1	Br1
....uaggaacGucauaccaugc.........................	1	1	Br1
....Caggaacuucauaccaugc.........................	1	1	Br1
....uaggaacuuAauaccaugc.........................	3	1	Br1
....uaggaacuucGuaccaugc.........................	1	1	Br1
....uaggaacuucauaAcaugc.........................	1	1	Br1
....uagAaacuucauaccaugc.........................	1	1	Br1
....uaggaacuuUauaccaugc.........................	1	1	Br1
....uaggaacuucauaccaugU.........................	6	1	Br1
....uaCgaacuucauaccaugc.........................	2	1	Br1
....uaggaacuCcauaccaugc.........................	1	1	Br1
....uaggaacuucauaccaugG.........................	2	1	Br1
....uaggaacuucauaccUugc.........................	2	1	Br1
....uCggaacuucauaccaugc.........................	7	1	Br1
....uaggaacuucauaccaugA.........................	413	1	Br1
....uaggaacuucauaccauCcu........................	1	1	Br1
....uaggaacuuUauaccaugcu........................	11	1	Br1
....uaggGacuucauaccaugcu........................	56	1	Br1
....uaggaacuucauaccaugcG........................	26	1	Br1
....Gaggaacuucauaccaugcu........................	2	1	Br1
....uaAgaacuucauaccaugcu........................	1	1	Br1
....uaggaacuucauacAaugcu........................	1	1	Br1
....uaggaacuCcauaccaugcu........................	4	1	Br1
....uaggUacuucauaccaugcu........................	66	1	Br1
....uaggaacGucauaccaugcu........................	1	1	Br1
....uaggaacuucaCaccaugcu........................	1	1	Br1
....uaggaacCucauaccaugcu........................	2	1	Br1
....uaggaacuucauaccaugUu........................	7	1	Br1
....uaggaUcuucauaccaugcu........................	5	1	Br1
....uaggaCcuucauaccaugcu........................	2	1	Br1
....uagUaacuucauaccaugcu........................	10	1	Br1
....uaggaacuucauaAcaugcu........................	2	1	Br1
....uaggaacuucCuaccaugcu........................	20	1	Br1
....uaggaacuAcauaccaugcu........................	6	1	Br1
....uaggaacuucauaccaCgcu........................	4	1	Br1
....uaUgaacuucauaccaugcu........................	1	1	Br1
....uaggaacuucauacUaugcu........................	5	1	Br1
....uaggaacuucauaccaAgcu........................	1	1	Br1
....uaggCacuucauaccaugcu........................	2	1	Br1
....uUggaacuucauaccaugcu........................	1	1	Br1
....uaggaacuucauaccaugcC........................	1465	1	Br1
....uaCgaacuucauaccaugcu........................	22	1	Br1
....uGggaacuucauaccaugcu........................	1	1	Br1
....uCggaacuucauaccaugcu........................	21	1	Br1
....uaggaacuucauaccauAcu........................	2	1	Br1
....uaggaacuuAauaccaugcu........................	5	1	Br1
....uaggaacuucauaccauUcu........................	2	1	Br1
....Aaggaacuucauaccaugcu........................	9	1	Br1
....uaggaacuucauacGaugcu........................	2	1	Br1
....uaggaacuucGuaccaugcu........................	4	1	Br1
....uagCaacuucauaccaugcu........................	1	1	Br1
....uaggaacuucauaccaugcu........................	5260	0	Br1
....uaggaacAucauaccaugcu........................	1	1	Br1
....uaggaacuucauaccUugcu........................	5	1	Br1
....uaggaacuucauaccaugcA........................	593	1	Br1
....uaggaacuucauaccaugGu........................	1	1	Br1
....Naggaacuucauaccaugcu........................	7	1	Br1
....uaggaacuucauaUcaugcu........................	7	1	Br1
....uaggaacuucauCccaugcu........................	10	1	Br1
....Caggaacuucauaccaugcu........................	2	1	Br1
....uaggaacuucauaUcaugcuc.......................	19	1	Br1
....uaggaUcuucauaccaugcuc.......................	53	1	Br1
....uagAaacuucauaccaugcuc.......................	10	1	Br1

```
                                Star                                Mature
agugaagaaaggacaacagcuggguucucgagagcgagguauaaaguucuuacaaggaauuuaauugguaggaacuucauaccaugcucgcgaugauucaaaguccacaaca
...................................................................uaggaacuuAauaccaugcuc.......................   53      1   Br1
...................................................................uaUgaacuucauaccaugcuc.......................   11      1   Br1
...................................................................uaggaacuucauCccaugcuc.......................   86      1   Br1
...................................................................uaggaacGucauaccaugcuc.......................   40      1   Br1
...................................................................uaggaCcuucauaccaugcuc.......................   3       1   Br1
...................................................................uaggaacuucauaccaCgcuc.......................   5       1   Br1
...................................................................uaggaacuucauacAaugcuc.......................   6       1   Br1
...................................................................uaggaacuucauaccaAgcuc.......................   10      1   Br1
...................................................................uaggaacuucauUccaugcuc.......................   12      1   Br1
...................................................................uaggaacuucauaccCugcuc.......................   5       1   Br1
...................................................................uaggaGcuucauaccaugcuc.......................   10      1   Br1
...................................................................uaggaacuuUauaccaugcuc.......................   169     1   Br1
...................................................................uaggaacuucauaccauUcuc.......................   51      1   Br1
...................................................................uaggUacuucauaccaugcuc.......................   1070    1   Br1
...................................................................uUggaacuucauaccaugcuc.......................   21      1   Br1
...................................................................uaggaacuucauaccaugAuc.......................   3       1   Br1
...................................................................uaggaacuGcauaccaugcuc.......................   2       1   Br1
...................................................................uaggaacuCcauaccaugcuc.......................   47      1   Br1
...................................................................uGggaacuucauaccaugcuc.......................   41      1   Br1
...................................................................uCggaacuucauaccaugcuc.......................   255     1   Br1
...................................................................uagUaacuucauaccaugcuc.......................   155     1   Br1
...................................................................uaggaacuucaGaccaugcuc.......................   15      1   Br1
...................................................................Caggaacuucauaccaugcuc.......................   65      1   Br1
...................................................................uaggaacuucauaccaugcAc.......................   57      1   Br1
...................................................................uaggaacuucauaccaugGuc.......................   10      1   Br1
...................................................................uaggaacuNcauaccaugcuc.......................   2       1   Br1
...................................................................uaAgaacuucauaccaugcuc.......................   11      1   Br1
...................................................................uaggaacuucauacGaugcuc.......................   11      1   Br1
...................................................................uaggGacuucauaccaugcuc.......................   799     1   Br1
...................................................................uaggaacuucauaGcaugcuc.......................   2       1   Br1
...................................................................uaggaacAucauaccaugcuc.......................   11      1   Br1
...................................................................uaggaaUuucauaccaugcuc.......................   10      1   Br1
...................................................................Gaggaacuucauaccaugcuc.......................   18      1   Br1
...................................................................uaggaaAuucauaccaugcuc.......................   2       1   Br1
...................................................................uaggaacCucauaccaugcuc.......................   12      1   Br1
...................................................................uaggaacuucauaccauCcuc.......................   6       1   Br1
...................................................................uaCgaacuucauaccaugcuc.......................   248     1   Br1
...................................................................uaggaacuucauGccaugcuc.......................   13      1   Br1
...................................................................uaggaacuucauaccaugcCc.......................   835     1   Br1
...................................................................uaggaacuucauaccGugcuc.......................   11      1   Br1
...................................................................uaggaacuuCuaccaugcuc.......................   451     1   Br1
...................................................................uaggaacuucauaccaugcuG.......................   45      1   Br1
...................................................................uagCaacuucauaccaugcuc.......................   4       1   Br1
...................................................................uaggaacuucauaccaugcuA.......................   1141    1   Br1
...................................................................uaggaacuucauaccaugUuc.......................   30      1   Br1
...................................................................uaggaacuucauaccauAcuc.......................   43      1   Br1
...................................................................uaggaacuucauaccaugcGc.......................   63      1   Br1
...................................................................uaggaacuucauaccUugcuc.......................   37      1   Br1
...................................................................uaggaacuucaCaccaugcuc.......................   12      1   Br1
...................................................................uaggCacuucauaccaugcuc.......................   10      1   Br1
...................................................................uaggaacuucaAaccaugcuc.......................   4       1   Br1
...................................................................uaggaacuucauacUaugcuc.......................   45      1   Br1
...................................................................uaggaacuucauaAcaugcuc.......................   22      1   Br1
...................................................................uaggaacuucauaccaugcuc.......................   68562   0   Br1
...................................................................uaggaaGuucauaccaugcuc.......................   5       1   Br1
...................................................................uaggaacuucGuaccaugcuc.......................   7       1   Br1
...................................................................AaggaacuucauaccaugcucA......................   90      1   Br1
...................................................................uaggaacuAcauaccaugcuc.......................   92      1   Br1
...................................................................Naggaacuucauaccaugcuc.......................   58      1   Br1
...................................................................uaggaacuucauaccaugcuGg......................   40      1   Br1
...................................................................uaggaacuucauaccaugcucA......................   12846   1   Br1
...................................................................uUggaacuucauaccaugcucg......................   281     1   Br1
...................................................................uaggaacuucauaccaugcucU......................   12526   1   Br1
...................................................................uaggaacuucauaAcaugcucg......................   213     1   Br1
...................................................................uaggaaGuucauaccaugcucg......................   28      1   Br1
...................................................................Gaggaacuucauaccaugcucg......................   143     1   Br1
...................................................................uaggaaAuucauaccaugcucg......................   19      1   Br1
...................................................................uaggaacuucauCccaugcucg......................   821     1   Br1
...................................................................uaggaacuAcauaccaugcucg......................   944     1   Br1
...................................................................uaggaacuucauUccaugcucg......................   129     1   Br1
```

Star Mature

```
agugaagaaaggacaacagcuggguucucgagagcgagguauaaaguucuuacaaggaauuuaauugguaggaacuucauaccaugcucgcgaugauucaaaguccacaaca
....................................................................Aaggaacuucauaccaugcucg......................   1347     1   Br1
....................................................................uaggaCcuucauaccaugcucg......................   36       1   Br1
....................................................................uaggaaUuucauaccaugcucg......................   113      1   Br1
....................................................................uaggaacuucauaccauCcucg......................   49       1   Br1
....................................................................uagCaacuucauaccaugcucg......................   27       1   Br1
....................................................................uaggaacuucauaccaugcuAg......................   464      1   Br1
....................................................................uaggaacuucauaccaugcAcg......................   176      1   Br1
....................................................................uaggaacuucauaccaugcucg......................   793144   0   Br1
....................................................................uaggaacuCcauaccaugcucg......................   479      1   Br1
....................................................................uaggaacuucauacAaugcucg......................   62       1   Br1
....................................................................uaggaacuucauaccauAcucg......................   708      1   Br1
....................................................................uaggaacuucaGaccaugcucg......................   150      1   Br1
....................................................................uaggaacuucauaccCugcucg......................   34       1   Br1
....................................................................uaggaacAucauaccaugcucg......................   84       1   Br1
....................................................................uaggaacuucauaccauUcucg......................   546      1   Br1
....................................................................uaggaacCucauaccaugcucg......................   148      1   Br1
....................................................................uaggaacuucauaccaugcGcg......................   428      1   Br1
....................................................................uaggaacuucauacUaugcucg......................   473      1   Br1
....................................................................uaAgaacuucauaccaugcucg......................   91       1   Br1
....................................................................uaggaacGucauaccaugcucg......................   306      1   Br1
....................................................................uaggGacuucauaccaugcucg......................   9076     1   Br1
....................................................................Caggaacuucauaccaugcucg......................   883      1   Br1
....................................................................uaggaacuucauaccGugcucg......................   257      1   Br1
....................................................................uaggaacuucauacGaugcucg......................   135      1   Br1
....................................................................uaggaacuucauaccaCgcucg......................   103      1   Br1
....................................................................uaggaGcuucauaccaugcucg......................   94       1   Br1
....................................................................uaggaacuGcauaccaugcucg......................   34       1   Br1
....................................................................uaggaacuucaCaccaugcucg......................   127      1   Br1
....................................................................uaggaacuucaAaccaugcucg......................   50       1   Br1
....................................................................Naggaacuucauaccaugcucg......................   603      1   Br1
....................................................................uaggaacuNcauaccaugcucg......................   8        1   Br1
....................................................................uaggCacuucauaccaugcucg......................   135      1   Br1
....................................................................uaggaacuucCuaccaugcucg......................   3940     1   Br1
....................................................................uaggaacuuGauaccaugcucg......................   27       1   Br1
....................................................................uaggaacuucauaccUugcucg......................   537      1   Br1
....................................................................uaggaacuucGuaccaugcucg......................   121      1   Br1
....................................................................uaggaacuucauaccaugAucg......................   37       1   Br1
....................................................................uaggaacuucauaUcaugcucg......................   311      1   Br1
....................................................................uaggaUcuucauaccaugcucg......................   627      1   Br1
....................................................................uaggaacuucauaccaugGucg......................   72       1   Br1
....................................................................uaUgaacuucauaccaugcucg......................   134      1   Br1
....................................................................uaggaacuuAauaccaugcucg......................   551      1   Br1
....................................................................uaCgaacuucauaccaugcucg......................   2804     1   Br1
....................................................................uagNaacuucauaccaugcucg......................   3        1   Br1
....................................................................uaggaacuucauaccaugcucC......................   1400     1   Br1
....................................................................uaggaacuucauaGcaugcucg......................   18       1   Br1
....................................................................uaggaacuucauaccaAgcucg......................   69       1   Br1
....................................................................uaggUacuucauaccaugcucg......................   12416    1   Br1
....................................................................uaggaacuucauaccaugcCcg......................   705      1   Br1
....................................................................uaggaacuucauaccaugUucg......................   433      1   Br1
....................................................................uagUaacuucauaccaugcucg......................   1766     1   Br1
....................................................................uCggaacuucauaccaugcucg......................   2652     1   Br1
....................................................................uaggaacuuUauaccaugcucg......................   897      1   Br1
....................................................................uGggaacuucauaccaugcucg......................   444      1   Br1
....................................................................uagAaacuucauaccaugcucg......................   83       1   Br1
....................................................................uaggaacuucauaccaGgcucg......................   11       1   Br1
....................................................................uaggaacuucauGccaugcucg......................   94       1   Br1
....................................................................uaggaacuucauaccaCgcucgc.....................   1        1   Br1
....................................................................uaggaacuAcauaccaugcucgc.....................   1        1   Br1
....................................................................uaggaacuucauacUaugcucgc.....................   1        1   Br1
....................................................................uaggGacuucauaccaugcucgc.....................   7        1   Br1
....................................................................uaggaacuucauaccaugcucgc.....................   733      0   Br1
....................................................................uaggaacAucauaccaugcucgc.....................   1        1   Br1
....................................................................uUggaacuucauaccaugcucgc.....................   1        1   Br1
....................................................................uagUaacuucauaccaugcucgc.....................   2        1   Br1
....................................................................uCggaacuucauaccaugcucgc.....................   7        1   Br1
....................................................................uaggaUcuucauaccaugcucgc.....................   1        1   Br1
....................................................................uaggaacuucauaAcaugcucgc.....................   2        1   Br1
....................................................................uaggaacuucauaccaugcucUc.....................   2        1   Br1
....................................................................uaggaacuuAauaccaugcucgc.....................   1        1   Br1
```

Star   Mature

```
agugaagaaaggacaacagcuggguucucgagagcgagguauaaaguucuuacaaggaauuuaauugguaggaacuucauaccaugcucgcgaugauucaaaguccacaaca
....................................................................uaggaacuucauaccaugcucgA.....................	19559	1	Br1
....................................................................uaggaacuucauaccaugcucCc.....................	1	1	Br1
....................................................................uaggaacuucCuaccaugcucgc.....................	3	1	Br1
....................................................................uaggaacuucaCaccaugcucgc.....................	1	1	Br1
....................................................................uaggaacuucauaccaugcucgG.....................	42	1	Br1
....................................................................Aaggaacuucauaccaugcucgc.....................	3	1	Br1
....................................................................uaCgaacuucauaccaugcucgc.....................	3	1	Br1
....................................................................uaggaacuCcauaccaugcucgc.....................	2	1	Br1
....................................................................uaggaacuucauaccaugcuAgc.....................	1	1	Br1
....................................................................uaggUacuucauaccaugcucgc.....................	10	1	Br1
....................................................................Caggaacuucauaccaugcucgc.....................	2	1	Br1
....................................................................uaggaacuucauCccaugcucgc.....................	1	1	Br1
....................................................................uaggaacuucauaccaugUucgc.....................	1	1	Br1
....................................................................uaggaacuucauaccaugcucgcA....................	9	1	Br1
....................................................................uaggaacuucauaccaugcucgAg....................	3	1	Br1
....................................................................uaggaacuucauaccaugcucgcC....................	16	1	Br1
....................................................................uaggaacuucauaccaugcucgcg....................	1	0	Br1
....................................................................uaggaacuucauaccaugcucgGg....................	2	1	Br1
....................................................................uaggaacuucauaccaugcucgcU....................	6	1	Br1
....................................................................uaggaacuucauaccaugcucgcgC...................	1	1	Br1
....................................................................uaggaacuucauaccaugcucgcCa...................	8	1	Br1
....................................................................uaggaacuucauaccaugcucgAga...................	1	1	Br1
....................................................................uaggaacuucauaccaugcucgGga...................	1	1	Br1
....................................................................uaggaacuucauaccaugcucgcAa...................	1	1	Br1
.....................................................................aggaacuucauaccaugc.........................	1	0	Br1
.....................................................................aggaacuucauaccaugA.........................	3	1	Br1
.....................................................................aggaacuucauaccaugcA........................	2	1	Br1
.....................................................................aggaacuucauaccauAcu........................	1	1	Br1
.....................................................................aggaacuucauaccaugcu........................	21	0	Br1
.....................................................................aggaacuucauaccaugcC........................	2	1	Br1
.....................................................................agCaacuucauaccaugcu........................	1	1	Br1
.....................................................................aggaUcuucauaccaugcu........................	1	1	Br1
.....................................................................aggaGcuucauaccaugcu........................	2	1	Br1
.....................................................................aggUacuucauaccaugcu........................	1	1	Br1
.....................................................................aggaacuuAauaccaugcu........................	1	1	Br1
.....................................................................aggaacuucCuaccaugcu........................	1	1	Br1
.....................................................................aggaacuucauaccaugcCc.......................	2	1	Br1
.....................................................................aggaacuucauaccGugcuc.......................	1	1	Br1
.....................................................................aggaacuucauaccaugUuc.......................	1	1	Br1
.....................................................................aggaacuuUauaccaugcuc.......................	1	1	Br1
.....................................................................agUaacuucauaccaugcuc.......................	1	1	Br1
.....................................................................aggaGcuucauaccaugcuc.......................	7	1	Br1
.....................................................................Nggaacuucauaccaugcuc.......................	1	1	Br1
.....................................................................Cggaacuucauaccaugcuc.......................	6	1	Br1
.....................................................................aggaacuucauaAcaugcuc.......................	3	1	Br1
.....................................................................aggaacuucauaccUugcuc.......................	2	1	Br1
.....................................................................aAgaacuucauaccaugcuc.......................	1	1	Br1
.....................................................................aggaacuucCuaccaugcuc.......................	2	1	Br1
.....................................................................aggaacuucauaccaugcuc.......................	173	0	Br1
.....................................................................aggaacuucauacAaugcuc.......................	1	1	Br1
.....................................................................aggaacuucauaccaugcAc.......................	1	1	Br1
.....................................................................aggaUcuucauaccaugcuc.......................	9	1	Br1
.....................................................................agCaacuucauaccaugcuc.......................	9	1	Br1
.....................................................................aggUacuucauaccaugcuc.......................	8	1	Br1
.....................................................................aggaacuGcauaccaugcuc.......................	3	1	Br1
.....................................................................aCgaacuucauaccaugcuc.......................	1	1	Br1
.....................................................................aggaacuuAauaccaugcuc.......................	3	1	Br1
.....................................................................aggaacuucauaccauAcuc.......................	1	1	Br1
.....................................................................aggaacuucauaccaugcuA.......................	3	1	Br1
.....................................................................aggaacuucauaccaugcuG.......................	1	1	Br1
.....................................................................Gggaacuucauaccaugcucg......................	1	1	Br1
.....................................................................aggaacGucauaccaugcucg......................	3	1	Br1
.....................................................................aggaacuuGauaccaugcucg......................	1	1	Br1
.....................................................................aggaacuucauaccaugcCcg......................	6	1	Br1
.....................................................................aggaacuucaCaccaugcucg......................	4	1	Br1
.....................................................................agAaacuucauaccaugcucg......................	1	1	Br1
.....................................................................aggaacuucauaccaAgcucg......................	1	1	Br1
.....................................................................aggaacuuUauaccaugcucg......................	7	1	Br1
.....................................................................aggaacuucauaccaugcuGg......................	2	1	Br1
.....................................................................aggaacuucCuaccaugcucg......................	82	1	Br1
```

```
                                Star                                Mature
agugaagaaaggacaacagcuggguucucgagagcgagguauaaaguucuuacaaggaauuuaauugguaggaacuucauaccaugcucgcgaugauucaaaguccacaaca
.....................................................................aggaacCucauaccaugcucg......................    1          1          Br1
.....................................................................aggaacuucauaccaugcucC......................    13         1          Br1
.....................................................................aggCacuucauaccaugcucg......................    2          1          Br1
.....................................................................aggaacuucauaccGugcucg......................    7          1          Br1
.....................................................................aggaacuucauaccaugcucA......................    60         1          Br1
.....................................................................aggaacuucauaccaugUucg......................    10         1          Br1
.....................................................................aggaacuucauacUaugcucg......................    1          1          Br1
.....................................................................aggaacuucauacAaugcucg......................    7          1          Br1
.....................................................................aggaacuucauaccaugcucU......................    44         1          Br1
.....................................................................aggaacuucauaAcaugcucg......................    17         1          Br1
.....................................................................aggaacuucauaccaugcGcg......................    4          1          Br1
.....................................................................agUaacuucauaccaugcucg......................    1          1          Br1
.....................................................................aCgaacuucauaccaugcucg......................    11         1          Br1
.....................................................................aggaacuucauaccCugcucg......................    8          1          Br1
.....................................................................aUgaacuucauaccaugcucg......................    2          1          Br1
.....................................................................aggaacuucauaccaugcAcg......................    1          1          Br1
.....................................................................aggaUcuucauaccaugcucg......................    142        1          Br1
.....................................................................aggaacuucauaccaugAucg......................    1          1          Br1
.....................................................................aggaacuuAauaccaugcucg......................    46         1          Br1
.....................................................................aggaacuucauaccauCcucg......................    8          1          Br1
.....................................................................aggaacuucauaccaugcucg......................    2252       0          Br1
.....................................................................Nggaacuucauaccaugcucg......................    1          1          Br1
.....................................................................aAgaacuucauaccaugcucg......................    10         1          Br1
.....................................................................aggaaAuucauaccaugcucg......................    1          1          Br1
.....................................................................aggaacuucGuaccaugcucg......................    5          1          Br1
.....................................................................aggaacuAcauaccaugcucg......................    5          1          Br1
.....................................................................aggaacuucauaccauAcucg......................    10         1          Br1
.....................................................................aggaacuucauaccaGgcucg......................    14         1          Br1
.....................................................................aggaGcuucauaccaugcucg......................    91         1          Br1
.....................................................................Uggaacuucauaccaugcucg......................    9          1          Br1
.....................................................................aggaaUuucauaccaugcucg......................    5          1          Br1
.....................................................................aggaacuucauaccaCgcucg......................    2          1          Br1
.....................................................................aggaacuucaAaccaugcucg......................    13         1          Br1
.....................................................................agCaacuucauaccaugcucg......................    74         1          Br1
.....................................................................aggaacuGcauaccaugcucg......................    16         1          Br1
.....................................................................aggUacuucauaccaugcucg......................    96         1          Br1
.....................................................................aggaacuucauaUcaugcucg......................    2          1          Br1
.....................................................................aggaacuCcauaccaugcucg......................    15         1          Br1
.....................................................................aggaacuucauaccUugcucg......................    5          1          Br1
.....................................................................aggaacuucauaccaugGucg......................    1          1          Br1
.....................................................................Cggaacuucauaccaugcucg......................    114        1          Br1
.....................................................................aggaacuucauaccauUcucg......................    8          1          Br1
.....................................................................aggaacuucauaccaugcucgG.....................    1          1          Br1
.....................................................................aggaacuucauaccaugcucgc.....................    5          0          Br1
.....................................................................aggaacuucauaccaugcucgA.....................    60         1          Br1
......................................................................ggaaUuucauaccaugcuc.......................    1          1          Br1
......................................................................ggaacuucauaccCugcuc.......................    1          1          Br1
......................................................................Agaacuucauaccaugcuc.......................    1          1          Br1
......................................................................ggaacuucauaccaugcuc.......................    7          0          Br1
......................................................................Ugaacuucauaccaugcuc.......................    1          1          Br1
......................................................................ggaacuucauaccaugcucg......................    78         0          Br1
......................................................................Agaacuucauaccaugcucg......................    1          1          Br1
......................................................................ggaacuuUauaccaugcucg......................    1          1          Br1
......................................................................ggaaUuucauaccaugcucg......................    4          1          Br1
......................................................................Ugaacuucauaccaugcucg......................    12         1          Br1
......................................................................ggaUcuucauaccaugcucg......................    6          1          Br1
......................................................................ggaacuucauaccaGgcucg......................    1          1          Br1
......................................................................ggaacuucauaccaugcuGg......................    1          1          Br1
......................................................................Cgaacuucauaccaugcucg......................    1          1          Br1
......................................................................gAaacuucauaccaugcucg......................    1          1          Br1
......................................................................ggaacuucauacAaugcucg......................    1          1          Br1
......................................................................ggaacuucauaccaugcucU......................    1          1          Br1
......................................................................ggaacuucCuaccaugcucg......................    4          1          Br1
......................................................................ggaaGuucauaccaugcucg......................    8          1          Br1
......................................................................ggaacuucauaccaugcucgA.....................    3          1          Br1
.......................................................................Uaacuucauaccaugcucg......................    1          1          Br1
........................................................................aacuucauaccaugcucC......................    1          1          Br1
........................................................................aacuucauaccaugcucg......................    2          0          Br1

................................agcgagguauaaaguucuu.............................................................    2          0          Le1
```

Star Mature

```
agugaagaaaggacaacagcuggguucucgagagcgagguauaaaguucuuacaaggaauuuaauugguaggaacuucauaccaugcucgcgaugauucaaaguccacaaca
................................agcgagguauaaaguucuua............................................................ 4 0 Le1
................................agcgagguauaGaguucuuaca.......................................................... 1 1 Le1
................................agcgagguauaaaguucuuaca.......................................................... 3 0 Le1
................................Cgcgagguauaaaguucuuaca.......................................................... 1 1 Le1
................................agcgagguauaaaguucuuacaaA........................................................ 1 1 Le1
..................................cgagguauaaaguucuuaca.......................................................... 1 0 Le1
...................................................................Auaggaacuucauaccaug.......................... 1 1 Le1
...................................................................Auaggaacuucauaccaugc......................... 1 1 Le1
...................................................................Cuaggaacuucauaccaugcu........................ 1 1 Le1
...................................................................guaggaacuucauaccaugcuc....................... 1 0 Le1
...................................................................Auaggaacuucauaccaugcuc....................... 1 1 Le1
...................................................................Cuaggaacuucauaccaugcuc....................... 9 1 Le1
...................................................................Cuaggaacuucauaccaugcucg...................... 41 1 Le1
....................................................................Aaggaacuucauaccaug.......................... 1 1 Le1
....................................................................uaggaacuucauaccaCg.......................... 2 1 Le1
....................................................................uaggaacuuUauaccaug.......................... 1 1 Le1
....................................................................uaggUacuucauaccaug.......................... 1 1 Le1
....................................................................uaggaacuucauaccauU.......................... 1 1 Le1
....................................................................uaggGacuucauaccaug.......................... 3 1 Le1
....................................................................uaggaacuucauaccaug.......................... 104 0 Le1
....................................................................uaggaacuucauaccUug.......................... 1 1 Le1
....................................................................uaggaacuucCuaccaug.......................... 2 1 Le1
....................................................................uaggaacuucauacUaug.......................... 1 1 Le1
....................................................................uaggaacuuAauaccaugc......................... 1 1 Le1
....................................................................uGggaacuucauaccaugc......................... 1 1 Le1
....................................................................uaggaacuucauaccaugc......................... 371 0 Le1
....................................................................uCggaacuucauaccaugc......................... 3 1 Le1
....................................................................uaggaacuucauaccaugU......................... 4 1 Le1
....................................................................Aaggaacuucauaccaugc......................... 1 1 Le1
....................................................................uaggGacuucauaccaugc......................... 3 1 Le1
....................................................................uaCgaacuucauaccaugc......................... 2 1 Le1
....................................................................uaggaacuucauaccaCgc......................... 1 1 Le1
....................................................................uaggaacuucauaccaugA......................... 216 1 Le1
....................................................................uaggaacuCcauaccaugc......................... 2 1 Le1
....................................................................uaggaacGucauaccaugc......................... 2 1 Le1
....................................................................uaggUacuucauaccaugc......................... 4 1 Le1
....................................................................uaggaacuucauaccauAc......................... 1 1 Le1
....................................................................uaggaacuucauaUcaugc......................... 1 1 Le1
....................................................................uaggaacuucCuaccaugc......................... 1 1 Le1
....................................................................uCggaacuucauaccaugcu........................ 5 1 Le1
....................................................................uaggaacuucauaccaugcA........................ 245 1 Le1
....................................................................uaggaacuucauaccaugcG........................ 7 1 Le1
....................................................................uaggaacuuUauaccaugcu........................ 7 1 Le1
....................................................................uaggaacuCcauaccaugcu........................ 1 1 Le1
....................................................................Naggaacuucauaccaugcu........................ 2 1 Le1
....................................................................uaggaacuAcauaccaugcu........................ 1 1 Le1
....................................................................uaCgaacuucauaccaugcu........................ 4 1 Le1
....................................................................uagUaacuucauaccaugcu........................ 3 1 Le1
....................................................................uGggaacuucauaccaugcu........................ 1 1 Le1
....................................................................uaggUacuucauaccaugcu........................ 20 1 Le1
....................................................................uaggaacuucauaUcaugcu........................ 2 1 Le1
....................................................................uaggaacuucauaccaCgcu........................ 1 1 Le1
....................................................................Aaggaacuucauaccaugcu........................ 1 1 Le1
....................................................................uaggaacuucauCccaugcu........................ 3 1 Le1
....................................................................uaggaacuucauacAaugcu........................ 1 1 Le1
....................................................................uaggGacuucauaccaugcu........................ 16 1 Le1
....................................................................uUggaacuucauaccaugcu........................ 2 1 Le1
....................................................................uaggaacuucauaccaugcC........................ 321 1 Le1
....................................................................uaggaacuucauaccauAcu........................ 2 1 Le1
....................................................................uaggaacuucauaccaugcu........................ 1738 0 Le1
....................................................................uaggaUcuucauaccaugcu........................ 2 1 Le1
....................................................................uaggaacuucCuaccaugcu........................ 10 1 Le1
....................................................................Caggaacuucauaccaugcu........................ 1 1 Le1
....................................................................uaggCacuucauaccaugcu........................ 1 1 Le1
....................................................................uaggaacuucauacUaugcu........................ 2 1 Le1
....................................................................uaggaacuucaCaccaugcu........................ 2 1 Le1
....................................................................uaAgaacuucauaccaugcu........................ 1 1 Le1
....................................................................uaggaacuucauaccaugAu........................ 1 1 Le1
....................................................................uaggaacuuAauaccaugcu........................ 3 1 Le1
....................................................................uagAaacuucauaccaugcuc....................... 4 1 Le1
```

Star **Mature**

agugaagaaaggacaacagcuggguucucgagagcgagguauaaaguucuuacaaggaauuuaauugguaggaacuucauaccaugcucgcgaugauucaaaguccacaaca			
....uaggaacuucauaccaAgcuc.......................	3	1	Le1
....uaggaacuucauaccauCcuc.......................	1	1	Le1
....uaggaacuucauaccaugcCc.......................	87	1	Le1
....uaggaacuucauacAaugcuc.......................	1	1	Le1
....uaggaacuucauaccaCgcuc.......................	2	1	Le1
....uaggaUcuucauaccaugcuc.......................	11	1	Le1
....uaggaacuucauaccauAcuc.......................	5	1	Le1
....uaggaacuucauUccaugcuc.......................	2	1	Le1
....uaCgaacuucauaccaugcuc.......................	64	1	Le1
....uaggaacuNcauaccaugcuc.......................	1	1	Le1
....Aaggaacuucauaccaugcuc.......................	18	1	Le1
....uaUgaacuucauaccaugcuc.......................	5	1	Le1
....uaggaacuucauaccaugcuG.......................	16	1	Le1
....uaggaacuCcauaccaugcuc.......................	12	1	Le1
....uCggaacuucauaccaugcuc.......................	55	1	Le1
....uaggaacuAcauaccaugcuc.......................	18	1	Le1
....uaggaacuucauaccaugGuc.......................	2	1	Le1
....uagUaacuucauaccaugcuc.......................	41	1	Le1
....uaggaacuucauaUcaugcuc.......................	5	1	Le1
....uaggaacuucaCaccaugcuc.......................	5	1	Le1
....uaggaacuucauaccaugAuc.......................	3	1	Le1
....uaggaacuucauaccaugcuA.......................	155	1	Le1
....uaggaacuucauGccaugcuc.......................	2	1	Le1
....uaggaacuucauaccUugcuc.......................	14	1	Le1
....uaggaacuucauaccGugcuc.......................	4	1	Le1
....Naggaacuucauaccaugcuc.......................	12	1	Le1
....uaggaacuucauacGaugcuc.......................	6	1	Le1
....uaggaacGucauaccaugcuc.......................	11	1	Le1
....uaggaacuucauaccauUcuc.......................	19	1	Le1
....uaggaacuucGuaccaugcuc.......................	6	1	Le1
....uaggaacuucauaccaugcGc.......................	17	1	Le1
....uaggaacuucauaccaugcAc.......................	9	1	Le1
....uaggaacuucaGaccaugcuc.......................	2	1	Le1
....uaggaacuuAauaccaugcuc.......................	20	1	Le1
....uaggaaUuucauaccaugcuc.......................	4	1	Le1
....uaggaGcuucauaccaugcuc.......................	2	1	Le1
....uaggaacuucaAaccaugcuc.......................	1	1	Le1
....uaAgaacuucauaccaugcuc.......................	5	1	Le1
....uaggaacuucCuaccaugcuc.......................	103	1	Le1
....uaggaacuucauaccaugcuc.......................	16370	0	Le1
....uaggaacuucauaccaugUuc.......................	10	1	Le1
....Gaggaacuucauaccaugcuc.......................	1	1	Le1
....uaggaacuuUauaccaugcuc.......................	47	1	Le1
....uGggaacuucauaccaugcuc.......................	8	1	Le1
....uUggaacuucauaccaugcuc.......................	8	1	Le1
....uaggaacuucauacUaugcuc.......................	16	1	Le1
....uaggaacuucauaAcaugcuc.......................	6	1	Le1
....uaggaacuucauCccaugcuc.......................	17	1	Le1
....uaggaacuucauaccCugcuc.......................	3	1	Le1
....uaggCacuucauaccaugcuc.......................	2	1	Le1
....uaggaacCucauaccaugcuc.......................	1	1	Le1
....uaggGacuucauaccaugcuc.......................	145	1	Le1
....uaggaaGuucauaccaugcuc.......................	1	1	Le1
....Caggaacuucauaccaugcuc.......................	17	1	Le1
....uaggaCcuucauaccaugcuc.......................	1	1	Le1
....uaggUacuucauaccaugcuc.......................	210	1	Le1
....uaggaacuucauGccaugcucg......................	14	1	Le1
....uUggaacuucauaccaugcucg......................	35	1	Le1
....uaggaacuucauaccauUcucg......................	92	1	Le1
....uaggaacuucauaccUugcucg......................	90	1	Le1
....uaggaGcuucauaccaugcucg......................	17	1	Le1
....uaggaacuuUauaccaugcucg......................	145	1	Le1
....uaggaacuucaCaccaugcucg......................	9	1	Le1
....uaggaacuucauaccaugUucg......................	42	1	Le1
....uaggaacuucauaccauAcucg......................	87	1	Le1
....uaggaacuucaGaccaugcucg......................	20	1	Le1
....uaggaacuucauaccaugcCcg......................	44	1	Le1
....Naggaacuucauaccaugcucg......................	107	1	Le1
....uaggaacuucauaAcaugcucg......................	27	1	Le1
....uaggaacuucauacAaugcucg......................	14	1	Le1

Star Mature

```
agugaagaaaggacaacagcuggguucucgagagcgagguauaaaguucuuacaaggaauuuaauugguaggaacuucauaccaugcucgcgaugauucaaaguccacaaca
....................................................................uaggaacuucauacGaugcucg......................  22      1  Le1
....................................................................uaggGacuucauaccaugcucg......................  1298    1  Le1
....................................................................uaAgaacuucauaccaugcucg......................  15      1  Le1
....................................................................uaggaacuucauaccaugcAcg......................  28      1  Le1
....................................................................uagCaacuucauaccaugcucg......................  1       1  Le1
....................................................................uaggaacuucauaccaugcucC......................  142     1  Le1
....................................................................uaUgaacuucauaccaugcucg......................  29      1  Le1
....................................................................uaggaacuucauaccaugcucA......................  1750    1  Le1
....................................................................Aaggaacuucauaccaugcucg......................  192     1  Le1
....................................................................uaggUacuucauaccaugcucg......................  1715    1  Le1
....................................................................uaggaacuuGauaccaugcucg......................  3       1  Le1
....................................................................Caggaacuucauaccaugcucg......................  120     1  Le1
....................................................................uaggaacuucauaGcaugcucg......................  4       1  Le1
....................................................................uaCgaacuucauaccaugcucg......................  449     1  Le1
....................................................................uaggaacuucauaccaugcucU......................  1407    1  Le1
....................................................................uaggaacuucGuaccaugcucg......................  18      1  Le1
....................................................................uaggaacuucauaccaugcucg......................  120356  0  Le1
....................................................................uaggaacuucauaccaugGucg......................  6       1  Le1
....................................................................uaggaacuucauaccGugcucg......................  36      1  Le1
....................................................................uaggaacuucauaccauCcucg......................  6       1  Le1
....................................................................uaggaacuucauCccaugcucg......................  131     1  Le1
....................................................................uagAaacuucauaccaugcucg......................  18      1  Le1
....................................................................uaggaacAucauaccaugcucg......................  9       1  Le1
....................................................................uaggaacuucaAaccaugcucg......................  4       1  Le1
....................................................................uaggaacCucauaccaugcucg......................  24      1  Le1
....................................................................uagUaacuucauaccaugcucg......................  265     1  Le1
....................................................................uaggaacuNcauaccaugcucg......................  3       1  Le1
....................................................................uCggaacuucauaccaugcucg......................  407     1  Le1
....................................................................uaggaacuucauaccaAgcucg......................  13      1  Le1
....................................................................uaggaacuucauaccaugcGcg......................  81      1  Le1
....................................................................uaggaUcuucauaccaugcucg......................  88      1  Le1
....................................................................uaggaacuucCuaccaugcucg......................  564     1  Le1
....................................................................uaggaaUuucauaccaugcucg......................  8       1  Le1
....................................................................uaggaacuucauacUaugcucg......................  106     1  Le1
....................................................................uaggaCcuucauaccaugcucg......................  6       1  Le1
....................................................................uaggaacuucauUccaugcucg......................  23      1  Le1
....................................................................uaggaacGucauaccaugcucg......................  33      1  Le1
....................................................................uaggaacuucauaccaugcuAg......................  75      1  Le1
....................................................................uaggaaAuucauaccaugcucg......................  2       1  Le1
....................................................................uaggaacuucauaccaugAucg......................  6       1  Le1
....................................................................uaggaacuGcauaccaugcucg......................  10      1  Le1
....................................................................uaggaacuucauaccaugcuGg......................  5       1  Le1
....................................................................uGggaacuucauaccaugcucg......................  70      1  Le1
....................................................................uaggaaGuucauaccaugcucg......................  7       1  Le1
....................................................................uaggaacuucauaccaCgcucg......................  15      1  Le1
....................................................................uaggaacuAcauaccaugcucg......................  122     1  Le1
....................................................................uaggaacuucauaccCugcucg......................  7       1  Le1
....................................................................uaggCacuucauaccaugcucg......................  14      1  Le1
....................................................................uaggaacuCcauaccaugcucg......................  56      1  Le1
....................................................................uaggaacuuAauaccaugcucg......................  73      1  Le1
....................................................................Gaggaacuucauaccaugcucg......................  28      1  Le1
....................................................................uaggaacuucauaUcaugcucg......................  62      1  Le1
....................................................................uaggaacuucauaccaGgcucg......................  5       1  Le1
....................................................................uagUaacuucauaccaugcucgc.....................  1       1  Le1
....................................................................uaggaacuucauaccaugcucgA.....................  2336    1  Le1
....................................................................uaggaacuucauaccaugcucUc.....................  1       1  Le1
....................................................................uaggaacuucauaccaugcucgc.....................  90      0  Le1
....................................................................uaggaacuucauaccaugcucgG.....................  5       1  Le1
....................................................................uGggaacuucauaccaugcucgc.....................  1       1  Le1
....................................................................uaggaacuucauaccaugcucgGg....................  2       1  Le1
....................................................................uaggaacuucauaccaugcucgcC....................  1       1  Le1
.....................................................................aggaacuucauaccaugc.........................  2       0  Le1
.....................................................................aggaUcuucauaccaugcu........................  1       1  Le1
.....................................................................aggUacuucauaccaugcu........................  2       1  Le1
.....................................................................Cggaacuucauaccaugcu........................  1       1  Le1
.....................................................................aCgaacuucauaccaugcu........................  1       1  Le1
.....................................................................aggaacuucauaccaugcA........................  1       1  Le1
.....................................................................aggaacuucauaccaugcC........................  2       1  Le1
.....................................................................aggaacuucauaccaugcu........................  3       0  Le1
.....................................................................aggaGcuucauaccaugcuc.......................  2       1  Le1
```

Star Mature

agugaagaaaggacaacagcuggguucucgagagcgagguauaaaguucuuacaaggaauuuaauugguaggaacuucauaccaugcucgcgaugauucaaaguccacaaca			
....aggaacuucauacAaugcuc.......................	1	1	Le1
....aggaacuucauaccaugcuc.......................	52	0	Le1
....aggaacuucauUccaugcuc.......................	1	1	Le1
....aggUacuucauaccaugcuc.......................	1	1	Le1
....agCaacuucauaccaugcuc.......................	2	1	Le1
....aggaUcuucauaccaugcuc.......................	4	1	Le1
....aggaacuucauaccGugcuc.......................	1	1	Le1
....Cggaacuucauaccaugcuc.......................	6	1	Le1
....aggaacuuAauaccaugcuc.......................	1	1	Le1
....aggaacuuAauaccaugcucg......................	10	1	Le1
....aggaUcuucauaccaugcucg......................	18	1	Le1
....aAgaacuucauaccaugcucg......................	1	1	Le1
....aggaacuucauaccaugUucg......................	1	1	Le1
....aggUacuucauaccaugcucg......................	23	1	Le1
....aggaacuuUauaccaugcucg......................	2	1	Le1
....aggaacuGcauaccaugcucg......................	2	1	Le1
....aggaacuucauaccCugcucg......................	2	1	Le1
....aggaacCucauaccaugcucg......................	1	1	Le1
....aggaacuucaAaccaugcucg......................	1	1	Le1
....aCgaacuucauaccaugcucg......................	1	1	Le1
....aggaacuucauaccaugcucC......................	2	1	Le1
....agCaacuucauaccaugcucg......................	9	1	Le1
....aggaacuucauaccGugcucg......................	1	1	Le1
....aggaGcuucauaccaugcucg......................	12	1	Le1
....aggaacuucauaccaugcucg......................	376	0	Le1
....aggaaUuucauaccaugcucg......................	1	1	Le1
....aggaacuucauacUaugcucg......................	3	1	Le1
....aggaacuucaCaccaugcucg......................	1	1	Le1
....aggaacuucauaccaugcucU......................	4	1	Le1
....Cggaacuucauaccaugcucg......................	12	1	Le1
....aggaacuucauUccaugcucg......................	1	1	Le1
....aggaacuucauaccaugcucA......................	9	1	Le1
....aggaacuucauacAaugcucg......................	1	1	Le1
....aggaacuAcauaccaugcucg......................	2	1	Le1
....aggaacuucCuaccaugcucg......................	13	1	Le1
....aggaacuucauaccaugcGcg......................	2	1	Le1
....Uggaacuucauaccaugcucg......................	3	1	Le1
....aggaacuucauaccaugcucgc.....................	1	0	Le1
....aggaacuucauaccaugcucgA.....................	10	1	Le1
....ggaacuucauaccaugcu........................	2	0	Le1
....ggaacuucauaccaugcuc.......................	2	0	Le1
....ggaacuucauaccaugcucg......................	21	0	Le1
....ggaacuucCuaccaugcucg......................	2	1	Le1
....ggaacuucauaccauAcucg......................	1	1	Le1
....ggaacuucauaccaugcucgA.....................	2	1	Le1

................................agcgagguauaaaguucuu...	2	0	Ch1
................................agcgagguauaaaguucuua..	1	0	Ch1
................................agcgagguauaGaguucuuac...	1	1	Ch1
................................agcgagguauaaaguucuuaU...	1	1	Ch1
................................agcgagguauaaaguucuuaca..	4	0	Ch1
................................agcgagguauaaaguucuuacU..	1	1	Ch1
................................agcgagguauaaaguucuuacaa...	2	0	Ch1
.................................gcgagguauaaaguucuuaca..	1	0	Ch1
.................................gcgagguauaaaguucuuacaa...	1	0	Ch1
....Cuaggaacuucauaccaug..........................	1	1	Ch1
....guaggaacuucauaccaugcuc.......................	2	0	Ch1
....Cuaggaacuucauaccaugcuc.......................	6	1	Ch1
....Auaggaacuucauaccaugcucg......................	4	1	Ch1
....Cuaggaacuucauaccaugcucg......................	52	1	Ch1
....Uuaggaacuucauaccaugcucg......................	1	1	Ch1
....guaggaacuucauaccaugcucgA.....................	1	1	Ch1
....uaggaacuucauaccaug..........................	75	0	Ch1
....uaggUacuucauaccaug..........................	1	1	Ch1
....uaggaacuucauaAcaug..........................	1	1	Ch1
....uaggaacuucCuaccaug..........................	1	1	Ch1
....uaggUacuucauaccaugc.........................	3	1	Ch1
....Caggaacuucauaccaugc.........................	1	1	Ch1
....uaggaacuucauaccUugc.........................	1	1	Ch1
....uaggGacuucauaccaugc.........................	2	1	Ch1

Star **Mature**

```
agugaagaaaggacaacagcuggguucucgagagcgagguauaaaguucuuacaaggaauuuaauugguaggaacuucauaccaugcucgcgaugauucaaaguccacaaca
....................................................................uaggaacuucauaccaugc.........................	292	0	Ch1
....................................................................uaggaacuucauacUaugc.........................	1	1	Ch1
....................................................................uaggaacuucCuaccaugc.........................	1	1	Ch1
....................................................................uaggaacuucauaccaugA.........................	151	1	Ch1
....................................................................uCggaacuucauaccaugc.........................	1	1	Ch1
....................................................................uaggaacuuUauaccaugc.........................	1	1	Ch1
....................................................................uaggaacuucauaUcaugc.........................	1	1	Ch1
....................................................................uaggaacuucauaAcaugcu........................	1	1	Ch1
....................................................................uaggaacuucauaccaugcu........................	2007	0	Ch1
....................................................................uaggaacuuUauaccaugcu........................	5	1	Ch1
....................................................................uagUaacuucauaccaugcu........................	7	1	Ch1
....................................................................uaggaacuucauaccaugcG........................	5	1	Ch1
....................................................................uaggaacuucauaccauUcu........................	1	1	Ch1
....................................................................uaggaacGucauaccaugcu........................	1	1	Ch1
....................................................................uaggaCcuucauaccaugcu........................	2	1	Ch1
....................................................................uaggaaUuucauaccaugcu........................	1	1	Ch1
....................................................................uaggaacuucauacGaugcu........................	3	1	Ch1
....................................................................uaggaacuucauacUaugcu........................	5	1	Ch1
....................................................................uaggaacuCcauaccaugcu........................	2	1	Ch1
....................................................................Gaggaacuucauaccaugcu........................	2	1	Ch1
....................................................................uaCgaacuucauaccaugcu........................	6	1	Ch1
....................................................................uaggaacuucaCaccaugcu........................	1	1	Ch1
....................................................................uaggaacuucauaccaAgcu........................	1	1	Ch1
....................................................................uaggaUcuucauaccaugcu........................	1	1	Ch1
....................................................................uaggaacuucauaccaugcC........................	361	1	Ch1
....................................................................uaggGacuucauaccaugcu........................	20	1	Ch1
....................................................................uaggaacuucauaUcaugcu........................	1	1	Ch1
....................................................................uGggaacuucauaccaugcu........................	3	1	Ch1
....................................................................uaggaacuucauaccaugUu........................	1	1	Ch1
....................................................................uaggUacuucauaccaugcu........................	39	1	Ch1
....................................................................uaggaacuucauaccaugcA........................	149	1	Ch1
....................................................................uaUgaacuucauaccaugcu........................	1	1	Ch1
....................................................................Caggaacuucauaccaugcu........................	2	1	Ch1
....................................................................uaggaacuAcauaccaugcu........................	3	1	Ch1
....................................................................uaggaacuucauCccaugcu........................	1	1	Ch1
....................................................................uaggaacuucCuaccaugcu........................	15	1	Ch1
....................................................................uCggaacuucauaccaugcu........................	4	1	Ch1
....................................................................uaggaacuuAauaccaugcu........................	4	1	Ch1
....................................................................uaggaacuucauaccaGgcuc.......................	1	1	Ch1
....................................................................uaggaaUuucauaccaugcuc.......................	4	1	Ch1
....................................................................uaggaacuucauaccaugcuG.......................	12	1	Ch1
....................................................................uaggaacuucaGaccaugcuc.......................	1	1	Ch1
....................................................................uaggaacuucauacUaugcuc.......................	13	1	Ch1
....................................................................uaggaacuucauaccaugcuA.......................	162	1	Ch1
....................................................................uaggaacuCcauaccaugcuc.......................	18	1	Ch1
....................................................................uagAaacuucauaccaugcuc.......................	6	1	Ch1
....................................................................uaUgaacuucauaccaugcuc.......................	4	1	Ch1
....................................................................uaggaacuucauaccaugcGc.......................	16	1	Ch1
....................................................................uaggaacCucauaccaugcuc.......................	6	1	Ch1
....................................................................uaggaacuucaAaccaugcuc.......................	1	1	Ch1
....................................................................uaggaacuGcauaccaugcuc.......................	1	1	Ch1
....................................................................uaggaacuuGauaccaugcuc.......................	1	1	Ch1
....................................................................uaggaacuucauaccaugUuc.......................	10	1	Ch1
....................................................................uaggaacuucauaccaugcCc.......................	85	1	Ch1
....................................................................Gaggaacuucauaccaugcuc.......................	2	1	Ch1
....................................................................uaggaacGucauaccaugcuc.......................	13	1	Ch1
....................................................................uaggaacuucauUccaugcuc.......................	7	1	Ch1
....................................................................uaggUacuucauaccaugcuc.......................	267	1	Ch1
....................................................................uaggaacuucauaAcaugcuc.......................	6	1	Ch1
....................................................................uaggaacuucauaccauAcuc.......................	15	1	Ch1
....................................................................uaggaacuucauaUcaugcuc.......................	5	1	Ch1
....................................................................uaggGacuucauaccaugcuc.......................	197	1	Ch1
....................................................................Caggaacuucauaccaugcuc.......................	25	1	Ch1
....................................................................uaggaacuucauaccaugcuc.......................	22290	0	Ch1
....................................................................uaggaUcuucauaccaugcuc.......................	19	1	Ch1
....................................................................uaggaacuuAauaccaugcuc.......................	22	1	Ch1
....................................................................uaggaacuucauaccCugcuc.......................	1	1	Ch1
....................................................................uaggaacuuUauaccaugcuc.......................	43	1	Ch1
....................................................................uaggaCcuucauaccaugcuc.......................	1	1	Ch1
....................................................................uCggaacuucauaccaugcuc.......................	91	1	Ch1
```

```
                               Star                              Mature
agugaagaaaggacaacagcuggguucucgagagcgagguauaaaguucuuacaaggaauuuaauugguaggaacuucauaccaugcucgcgaugauucaaaguccacaaca
....................................................................uaggaacuucCuaccaugcuc.......................   132     1   Ch1
....................................................................uaAgaacuucauaccaugcuc.......................   4       1   Ch1
....................................................................uUggaacuucauaccaugcuc.......................   8       1   Ch1
....................................................................uaggaacuucauaccauUcuc.......................   10      1   Ch1
....................................................................uaggaacuAcauaccaugcuc.......................   25      1   Ch1
....................................................................uaggaacuucauaccaAgcuc.......................   5       1   Ch1
....................................................................Naggaacuucauaccaugcuc.......................   21      1   Ch1
....................................................................uaggCacuucauaccaugcuc.......................   2       1   Ch1
....................................................................Aaggaacuucauaccaugcuc.......................   39      1   Ch1
....................................................................uaggaacuucauGccaugcuc.......................   2       1   Ch1
....................................................................uagUaacuucauaccaugcuc.......................   35      1   Ch1
....................................................................uaggaacuucauacGaugcuc.......................   4       1   Ch1
....................................................................uaggaacuucauaccUugcuc.......................   13      1   Ch1
....................................................................uaggaacuucauCccaugcuc.......................   22      1   Ch1
....................................................................uaggaacuucGuaccaugcuc.......................   4       1   Ch1
....................................................................uaCgaacuucauaccaugcuc.......................   85      1   Ch1
....................................................................uaggaGcuucauaccaugcuc.......................   2       1   Ch1
....................................................................uaggaacuucauaccGugcuc.......................   3       1   Ch1
....................................................................uaggaacuNcauaccaugcuc.......................   1       1   Ch1
....................................................................uGggaacuucauaccaugcuc.......................   11      1   Ch1
....................................................................uaggaacAucauaccaugcuc.......................   3       1   Ch1
....................................................................uaggaaGuucauaccaugcuc.......................   1       1   Ch1
....................................................................uaggaacuucauaccaugGuc.......................   1       1   Ch1
....................................................................uaggaacuucaCaccaugcuc.......................   4       1   Ch1
....................................................................uaggaacuucauacAaugcuc.......................   4       1   Ch1
....................................................................uaggaacuucauaccaugcAc.......................   4       1   Ch1
....................................................................uaggaacuucauaccaCgcuc.......................   4       1   Ch1
....................................................................uaggaacuucauaccaugAuc.......................   3       1   Ch1
....................................................................uaggaacuucauaccaugcAcg......................   22      1   Ch1
....................................................................uaggaacuucauaccaugcCcg......................   55      1   Ch1
....................................................................uaggaaAuucauaccaugcucg......................   6       1   Ch1
....................................................................uaggaacuucauacUaugcucg......................   142     1   Ch1
....................................................................uaggaaGuucauaccaugcucg......................   8       1   Ch1
....................................................................uaggaacuucauaccaugcGcg......................   98      1   Ch1
....................................................................uagCaacuucauaccaugcucg......................   3       1   Ch1
....................................................................uaggaCcuucauaccaugcucg......................   11      1   Ch1
....................................................................uaggaacuNcauaccaugcucg......................   2       1   Ch1
....................................................................uGggaacuucauaccaugcucg......................   80      1   Ch1
....................................................................uaggaacuucauaccaugcucC......................   166     1   Ch1
....................................................................uaggaacuucauaccaAgcucg......................   16      1   Ch1
....................................................................uaggaacuucauGccaugcucg......................   16      1   Ch1
....................................................................uaggaacAucauaccaugcucg......................   17      1   Ch1
....................................................................uaggaacuucauaccaugcuGg......................   8       1   Ch1
....................................................................Gaggaacuucauaccaugcucg......................   35      1   Ch1
....................................................................uaggaacuucauaUcaugcucg......................   74      1   Ch1
....................................................................uagUaacuucauaccaugcucg......................   395     1   Ch1
....................................................................uagAaacuucauaccaugcucg......................   21      1   Ch1
....................................................................uaggGacuucauaccaugcucg......................   1555    1   Ch1
....................................................................uaCgaacuucauaccaugcucg......................   631     1   Ch1
....................................................................uaggaacuucauaAcaugcucg......................   60      1   Ch1
....................................................................uaggaacuucauaccauCcucg......................   14      1   Ch1
....................................................................uaggaacuucauaccaugcuAg......................   104     1   Ch1
....................................................................uaAgaacuucauaccaugcucg......................   15      1   Ch1
....................................................................uaggaacuucauaccaugcucg......................   162702  0   Ch1
....................................................................uaggaacuucauaccaugcucA......................   1706    1   Ch1
....................................................................uaggaacuucGuaccaugcucg......................   24      1   Ch1
....................................................................Caggaacuucauaccaugcucg......................   179     1   Ch1
....................................................................uaggaacuuUauaccaugcucg......................   188     1   Ch1
....................................................................uaggaacuuGauaccaugcucg......................   4       1   Ch1
....................................................................uaggaUcuucauaccaugcucg......................   129     1   Ch1
....................................................................uaggaacCucauaccaugcucg......................   41      1   Ch1
....................................................................uaggaacuucauUccaugcucg......................   17      1   Ch1
....................................................................uaggaacuucauaccGugcucg......................   46      1   Ch1
....................................................................uaggaacuucauaccCugcucg......................   15      1   Ch1
....................................................................uaggaacuucauaccaugcucU......................   2212    1   Ch1
....................................................................uaggaacuucauaccaugGucg......................   20      1   Ch1
....................................................................uUggaacuucauaccaugcucg......................   66      1   Ch1
....................................................................uaggaacuuAauaccaugcucg......................   160     1   Ch1
....................................................................uaggaacuucauaccaugAucg......................   8       1   Ch1
....................................................................uaggaacuucauaccaugUucg......................   91      1   Ch1
```

Star Mature

```
agugaagaaaggacaacagcuggguucucgagagcgagguauaaaguucuuacaaggaauuuaauugguaggaacuucauaccaugcucgcgaugauucaaaguccacaaca
....................................................................uaUgaacuucauaccaugcucg......................   29     1   Ch1
....................................................................uaggaacuCcauaccaugcucg......................   104    1   Ch1
....................................................................uaggaacuucaGaccaugcucg......................   24     1   Ch1
....................................................................uaggaacuucaCaccaugcucg......................   22     1   Ch1
....................................................................uaggaacuucauaccauAcucg......................   150    1   Ch1
....................................................................uaggaacuucauaccaGgcucg......................   4      1   Ch1
....................................................................uaggaacGucauaccaugcucg......................   61     1   Ch1
....................................................................uaggaacuucauacAaugcucg......................   18     1   Ch1
....................................................................uaggaacuucauaccauUcucg......................   152    1   Ch1
....................................................................uaggaacuucauaccaCgcucg......................   22     1   Ch1
....................................................................uaggaacuGcauaccaugcucg......................   5      1   Ch1
....................................................................uCggaacuucauaccaugcucg......................   560    1   Ch1
....................................................................Aaggaacuucauaccaugcucg......................   276    1   Ch1
....................................................................uaggCacuucauaccaugcucg......................   11     1   Ch1
....................................................................Naggaacuucauaccaugcucg......................   112    1   Ch1
....................................................................uaggaacuucauaccUugcucg......................   190    1   Ch1
....................................................................uaggaacuucauacGaugcucg......................   37     1   Ch1
....................................................................uaggaGcuucauaccaugcucg......................   30     1   Ch1
....................................................................uaggUacuucauaccaugcucg......................   2043   1   Ch1
....................................................................uaggaacuAcauaccaugcucg......................   219    1   Ch1
....................................................................uaggaaUuucauaccaugcucg......................   19     1   Ch1
....................................................................uaggaacuucCuaccaugcucg......................   779    1   Ch1
....................................................................uaggaacuucauCccaugcucg......................   191    1   Ch1
....................................................................uaggaacuucauaGcaugcucg......................   4      1   Ch1
....................................................................uaggaacuucaAaccaugcucg......................   2      1   Ch1
....................................................................uaggaacuucauCccaugcucgc.....................   1      1   Ch1
....................................................................uaggaacuucauaAcaugcucgc.....................   1      1   Ch1
....................................................................uaggaacuucauaccaugcucgA.....................   2877   1   Ch1
....................................................................uaggUacuucauaccaugcucgc.....................   1      1   Ch1
....................................................................uCggaacuucauaccaugcucgc.....................   2      1   Ch1
....................................................................uaggaacuucCuaccaugcucgc.....................   2      1   Ch1
....................................................................uaggaacuucauaccaugcucgG.....................   10     1   Ch1
....................................................................uaggaacuucauaccaugcucgc.....................   83     0   Ch1
....................................................................Caggaacuucauaccaugcucgc.....................   1      1   Ch1
....................................................................uaggaacuucauaccauCcucgc.....................   1      1   Ch1
....................................................................uaggaacuucauaccaugcucgcC....................   4      1   Ch1
....................................................................uaggaacuucauaccaugcucgcA....................   1      1   Ch1
.....................................................................aggaacuucauaccaugcu........................   6      0   Ch1
.....................................................................agAaacuucauaccaugcu........................   1      1   Ch1
.....................................................................agCaacuucauaccaugcu........................   1      1   Ch1
.....................................................................aggUacuucauaccaugcu........................   1      1   Ch1
.....................................................................aAgaacuucauaccaugcuc.......................   1      1   Ch1
.....................................................................aggaacuucauUccaugcuc.......................   1      1   Ch1
.....................................................................aggaGcuucauaccaugcuc.......................   5      1   Ch1
.....................................................................aggaacuuAauaccaugcuc.......................   1      1   Ch1
.....................................................................aggaacuCcauaccaugcuc.......................   1      1   Ch1
.....................................................................aggaacuucauaccaugcuA.......................   1      1   Ch1
.....................................................................aggaacuucauaccauUcuc.......................   2      1   Ch1
.....................................................................aggUacuucauaccaugcuc.......................   4      1   Ch1
.....................................................................aggaacuucauaAcaugcuc.......................   2      1   Ch1
.....................................................................aggaacuucCuaccaugcuc.......................   4      1   Ch1
.....................................................................aggaacuucauaccaugcCc.......................   1      1   Ch1
.....................................................................aggaacuucauaccGugcuc.......................   1      1   Ch1
.....................................................................Cggaacuucauaccaugcuc.......................   6      1   Ch1
.....................................................................aggaacuucauaccaugcuc.......................   63     0   Ch1
.....................................................................aggaacuucauaccaugcucA......................   6      1   Ch1
.....................................................................Gggaacuucauaccaugcucg......................   1      1   Ch1
.....................................................................aggaacuuAauaccaugcucg......................   5      1   Ch1
.....................................................................aggaacuucauaccaAgcucg......................   1      1   Ch1
.....................................................................aggaacuCcauaccaugcucg......................   3      1   Ch1
.....................................................................aggaacuucauaccaugcucU......................   4      1   Ch1
.....................................................................aggaUcuucauaccaugcucg......................   20     1   Ch1
.....................................................................Cggaacuucauaccaugcucg......................   24     1   Ch1
.....................................................................aggaacuucauaccCugcucg......................   3      1   Ch1
.....................................................................aggaacuucauaccUugcucg......................   2      1   Ch1
.....................................................................aggaacuucaAaccaugcucg......................   5      1   Ch1
.....................................................................aggaacuuUauaccaugcucg......................   1      1   Ch1
.....................................................................aggaacuucauacAaugcucg......................   1      1   Ch1
.....................................................................Uggaacuucauaccaugcucg......................   3      1   Ch1
.....................................................................aCgaacuucauaccaugcucg......................   2      1   Ch1
```

Star   Mature

```
agugaagaaaggacaacagcuggguucucgagagcgagguauaaaguucuuacaaggaauuuaauugguaggaacuucauaccaugcucgcgaugauucaaaguccacaaca
.....................................................................aggUacuucauaccaugcucg......................   17     1   Ch1
.....................................................................aggaacuucauUccaugcucg......................   1      1   Ch1
.....................................................................aggaacuucauaAcaugcucg......................   1      1   Ch1
.....................................................................aAgaacuucauaccaugcucg......................   1      1   Ch1
.....................................................................aggaacuucauaccauCcucg......................   1      1   Ch1
.....................................................................aggaacuucaCaccaugcucg......................   2      1   Ch1
.....................................................................aggaGcuucauaccaugcucg......................   14     1   Ch1
.....................................................................aggaacuucCuaccaugcucg......................   22     1   Ch1
.....................................................................aggaacuGcauaccaugcucg......................   3      1   Ch1
.....................................................................aggaacuucGuaccaugcucg......................   1      1   Ch1
.....................................................................aggaacuucauaccaugcucg......................   472    0   Ch1
.....................................................................aggaacuucauaccauAcucg......................   2      1   Ch1
.....................................................................aggaacuucauaccauUcucg......................   2      1   Ch1
.....................................................................aggaacuucauaccGugcucg......................   2      1   Ch1
.....................................................................aggaaUuucauaccaugcucg......................   2      1   Ch1
.....................................................................agCaacuucauaccaugcucg......................   16     1   Ch1
.....................................................................agUaacuucauaccaugcucg......................   1      1   Ch1
.....................................................................aggaacuucauaccaugUucg......................   1      1   Ch1
.....................................................................aggaacuucauaccaugcucC......................   6      1   Ch1
.....................................................................aggaacuucauaccaugcucgc.....................   3      0   Ch1
.....................................................................aggaacuucauaccaugcucgA.....................   10     1   Ch1
......................................................................ggaacuucCuaccaugcuc.......................   1      1   Ch1
......................................................................ggaacuucauaccaugcuc.......................   1      0   Ch1
......................................................................ggaacuucauaccaugcucg......................   11     0   Ch1
......................................................................Ugaacuucauaccaugcucg......................   2      1   Ch1
......................................................................Agaacuucauaccaugcucg......................   1      1   Ch1
......................................................................ggaacuucCuaccaugcucg......................   1      1   Ch1
......................................................................ggCacuucauaccaugcucg......................   1      1   Ch1
......................................................................ggaacuucauaccaugcucgA.....................   2      1   Ch1
.......................................................................Uaacuucauaccaugcucg......................   1      1   Ch1

................................agcgagguauaGaguucuuaca..........................................................   3      1   Bl1
...................................................................Auaggaacuucauaccaugcuc.......................   1      1   Bl1
...................................................................Cuaggaacuucauaccaugcuc.......................   5      1   Bl1
...................................................................Auaggaacuucauaccaugcucg......................   2      1   Bl1
...................................................................Cuaggaacuucauaccaugcucg......................   20     1   Bl1
....................................................................uaggaacGucauaccaug..........................   1      1   Bl1
....................................................................uaggUacuucauaccaug..........................   3      1   Bl1
....................................................................uagUaacuucauaccaug..........................   1      1   Bl1
....................................................................uaggGacuucauaccaug..........................   3      1   Bl1
....................................................................uaCgaacuucauaccaug..........................   1      1   Bl1
....................................................................uaggaacuucauaccaug..........................   167    0   Bl1
....................................................................uaggaacuucCuaccaug..........................   1      1   Bl1
....................................................................Naggaacuucauaccaug..........................   1      1   Bl1
....................................................................uUggaacuucauaccaug..........................   1      1   Bl1
....................................................................Caggaacuucauaccaug..........................   1      1   Bl1
....................................................................uaggaacuucauGccaug..........................   1      1   Bl1
....................................................................uaggaacuucauaccaugc.........................   1024   0   Bl1
....................................................................uaggUacuucauaccaugc.........................   27     1   Bl1
....................................................................Aaggaacuucauaccaugc.........................   2      1   Bl1
....................................................................uGggaacuucauaccaugc.........................   2      1   Bl1
....................................................................uaggaacuucauaccaAgc.........................   1      1   Bl1
....................................................................Caggaacuucauaccaugc.........................   1      1   Bl1
....................................................................uaggaacuucauaUcaugc.........................   1      1   Bl1
....................................................................uaggaacuucauaccUugc.........................   1      1   Bl1
....................................................................uaggaacuuUauaccaugc.........................   2      1   Bl1
....................................................................uaggaacuucauaccaugU.........................   2      1   Bl1
....................................................................uaggaacuCcauaccaugc.........................   1      1   Bl1
....................................................................uaggCacuucauaccaugc.........................   1      1   Bl1
....................................................................uaggaacuAcauaccaugc.........................   2      1   Bl1
....................................................................uaggaacuucCuaccaugc.........................   7      1   Bl1
....................................................................uCggaacuucauaccaugc.........................   3      1   Bl1
....................................................................uagUaacuucauaccaugc.........................   3      1   Bl1
....................................................................uaggGacuucauaccaugc.........................   13     1   Bl1
....................................................................uaggaacuucauaccaugA.........................   25     1   Bl1
....................................................................uaggaacuucauacUaugc.........................   4      1   Bl1
....................................................................uaggaUcuucauaccaugc.........................   1      1   Bl1
....................................................................uaggaacuucaAaccaugc.........................   1      1   Bl1
....................................................................uaCgaacuucauaccaugc.........................   5      1   Bl1
....................................................................uaggaacuucauCccaugc.........................   2      1   Bl1
```

```
                                Star                                 Mature
agugaagaaaggacaacagcuggguucucgagagcgagguauaaaguucuuacaaggaauuuaauugguaggaacuucauaccaugcucgcgaugauucaaaguccacaaca
....................................................................uaggaacuucauaccauAc.........................    1      1    Bl1
....................................................................uaggaacuAcauaccaugcu........................    2      1    Bl1
....................................................................uaggUacuucauaccaugcu........................    22     1    Bl1
....................................................................uGggaacuucauaccaugcu........................    3      1    Bl1
....................................................................uaggaacuucauaccaugcC........................    101    1    Bl1
....................................................................uaggaacuucauCccaugcu........................    2      1    Bl1
....................................................................uaggGacuucauaccaugcu........................    10     1    Bl1
....................................................................uaggaacuucauacUaugcu........................    4      1    Bl1
....................................................................uaggaacuucauaccaugcA........................    138    1    Bl1
....................................................................uaggaacuucauaccaugcu........................    1097   0    Bl1
....................................................................Aaggaacuucauaccaugcu........................    2      1    Bl1
....................................................................uaggaacuCcauaccaugcu........................    1      1    Bl1
....................................................................Caggaacuucauaccaugcu........................    2      1    Bl1
....................................................................uaggaacuuUauaccaugcu........................    1      1    Bl1
....................................................................uaCgaacuucauaccaugcu........................    11     1    Bl1
....................................................................uCggaacuucauaccaugcu........................    2      1    Bl1
....................................................................uaggaacuucauaccaugcG........................    2      1    Bl1
....................................................................uaggaacuucauaccUugcu........................    1      1    Bl1
....................................................................uaggaacuucCuaccaugcu........................    8      1    Bl1
....................................................................uagUaacuucauaccaugcu........................    2      1    Bl1
....................................................................uaggaaGuucauaccaugcuc.......................    1      1    Bl1
....................................................................uaAgaacuucauaccaugcuc.......................    1      1    Bl1
....................................................................uaggGacuucauaccaugcuc.......................    135    1    Bl1
....................................................................uaggaacuucauaccaugcAc.......................    8      1    Bl1
....................................................................uaggaacuucauaccaugcuG.......................    2      1    Bl1
....................................................................uaggaacuucauaGcaugcuc.......................    1      1    Bl1
....................................................................uaggaacuucauacUaugcuc.......................    11     1    Bl1
....................................................................uUggaacuucauaccaugcuc.......................    6      1    Bl1
....................................................................uaggaacuucauGccaugcuc.......................    1      1    Bl1
....................................................................uaggaacuucauaccaugUuc.......................    6      1    Bl1
....................................................................uaggaacuuAauaccaugcuc.......................    5      1    Bl1
....................................................................uaggaacuucauaccUugcuc.......................    7      1    Bl1
....................................................................Caggaacuucauaccaugcuc.......................    11     1    Bl1
....................................................................uaggaacuucauaccaugGuc.......................    2      1    Bl1
....................................................................uaggaacuucauaccauCcuc.......................    1      1    Bl1
....................................................................uaggaacuuUauaccaugcuc.......................    18     1    Bl1
....................................................................Aaggaacuucauaccaugcuc.......................    18     1    Bl1
....................................................................uaggaacuucCuaccaugcuc.......................    74     1    Bl1
....................................................................uaggaacAucauaccaugcuc.......................    1      1    Bl1
....................................................................uaggCacuucauaccaugcuc.......................    3      1    Bl1
....................................................................uaggaacuAcauaccaugcuc.......................    15     1    Bl1
....................................................................uaggaacuucGuaccaugcuc.......................    2      1    Bl1
....................................................................uaggaacuucauaccaugcuc.......................    12047  0    Bl1
....................................................................uaggaacuucauacGaugcuc.......................    1      1    Bl1
....................................................................Naggaacuucauaccaugcuc.......................    14     1    Bl1
....................................................................uaggaacuucauaUcaugcuc.......................    5      1    Bl1
....................................................................uaggaaAuucauaccaugcuc.......................    1      1    Bl1
....................................................................uaggaacuucauaccauUcuc.......................    13     1    Bl1
....................................................................uaggaacuucauaccaugAuc.......................    3      1    Bl1
....................................................................uaggaacuucauCccaugcuc.......................    13     1    Bl1
....................................................................uaggaacuucauaccaugcCc.......................    17     1    Bl1
....................................................................uaggaUcuucauaccaugcuc.......................    8      1    Bl1
....................................................................uaggaacuucauaccaGgcuc.......................    1      1    Bl1
....................................................................uaggaacuCcauaccaugcuc.......................    7      1    Bl1
....................................................................uaUgaacuucauaccaugcuc.......................    2      1    Bl1
....................................................................uaCgaacuucauaccaugcuc.......................    40     1    Bl1
....................................................................uaggaacuucauaccaAgcuc.......................    2      1    Bl1
....................................................................uaggaacuucauaccaugcuA.......................    64     1    Bl1
....................................................................uGggaacuucauaccaugcuc.......................    5      1    Bl1
....................................................................Gaggaacuucauaccaugcuc.......................    3      1    Bl1
....................................................................uaggaacCucauaccaugcuc.......................    2      1    Bl1
....................................................................uaggaacuucauaAcaugcuc.......................    6      1    Bl1
....................................................................uaggaGcuucauaccaugcuc.......................    1      1    Bl1
....................................................................uagUaacuucauaccaugcuc.......................    30     1    Bl1
....................................................................uaggaacuucauaccauAcuc.......................    2      1    Bl1
....................................................................uaggaaUuucauaccaugcuc.......................    4      1    Bl1
....................................................................uaggaacuucaAaccaugcuc.......................    1      1    Bl1
....................................................................uaggaacGucauaccaugcuc.......................    5      1    Bl1
....................................................................uaggaacuucauaccaCgcuc.......................    1      1    Bl1
....................................................................uCggaacuucauaccaugcuc.......................    43     1    Bl1
```

Star Mature

agugaagaaaggacaacagcuggguucucgagagcgagguauaaaguucuuacaaggaauuuaauugguaggaacuucauaccaugcucgcgaugauucaaaguccacaaca			
....uaggUacuucauaccaugcuc.......................	151	1	Bl1
....uaggaacuucauGccaugcucg......................	3	1	Bl1
....uaggaacuucauaGcaugcucg......................	1	1	Bl1
....uaCgaacuucauaccaugcucg......................	188	1	Bl1
....uGggaacuucauaccaugcucg......................	26	1	Bl1
....uaggCacuucauaccaugcucg......................	6	1	Bl1
....uaggaacuucauUccaugcucg......................	9	1	Bl1
....uaggaacuucauaccauAcucg......................	46	1	Bl1
....uaggaacuucauaccGugcucg......................	14	1	Bl1
....uaggaCcuucauaccaugcucg......................	1	1	Bl1
....Naggaacuucauaccaugcucg......................	44	1	Bl1
....uaggaacuucauCccaugcucg......................	35	1	Bl1
....uagAaacuucauaccaugcucg......................	6	1	Bl1
....uaggaacuuUauaccaugcucg......................	39	1	Bl1
....uaggaacuucaAaccaugcucg......................	4	1	Bl1
....uaggaacuuCuaccaugcucg.......................	247	1	Bl1
....uaggaacuuAauaccaugcucg......................	37	1	Bl1
....uaggaGcuucauaccaugcucg......................	5	1	Bl1
....Gaggaacuucauaccaugcucg......................	13	1	Bl1
....Caggaacuucauaccaugcucg......................	42	1	Bl1
....uaggaacuucauaccaGgcucg......................	1	1	Bl1
....uaggaacuucauaccaugcGcg......................	20	1	Bl1
....uaggaacuucauaccaugcuGg......................	1	1	Bl1
....uaggaacuucauaccCugcucg......................	6	1	Bl1
....uaggUacuucauaccaugcucg......................	698	1	Bl1
....uaggaacuucauaccaugcCcg......................	10	1	Bl1
....uaggaacuucauaccaugUucg......................	31	1	Bl1
....uaggaacuAcauaccaugcucg......................	57	1	Bl1
....uaggaaAuucauaccaugcucg......................	1	1	Bl1
....uaggaacuucauaccauCcucg......................	3	1	Bl1
....uaggaacuucauacGaugcucg......................	8	1	Bl1
....uaggaaUuucauaccaugcucg......................	3	1	Bl1
....uagUaacuucauaccaugcucg......................	108	1	Bl1
....uaggaacuucauaccauUcucg......................	42	1	Bl1
....uaggaacuucauaccaugcuAg......................	26	1	Bl1
....uaggaacuucauaccaugcucA......................	1042	1	Bl1
....uaggaacGucauaccaugcucg......................	14	1	Bl1
....uaAgaacuucauaccaugcucg......................	11	1	Bl1
....uaggaacuGcauaccaugcucg......................	5	1	Bl1
....uaggaacuucauacUaugcucg......................	42	1	Bl1
....uaggaacuucauacAaugcucg......................	2	1	Bl1
....uaggaacAucauaccaugcucg......................	4	1	Bl1
....uUggaacuucauaccaugcucg......................	21	1	Bl1
....uaUgaacuucauaccaugcucg......................	16	1	Bl1
....uaggaacuucauaccaCgcucg......................	7	1	Bl1
....uaggaacuucauaccUugcucg......................	48	1	Bl1
....uaggaacCucauaccaugcucg......................	10	1	Bl1
....uaggaUcuucauaccaugcucg......................	40	1	Bl1
....uaggaacuucauaUcaugcucg......................	29	1	Bl1
....uaggaacuucauaccaugGucg......................	5	1	Bl1
....uaggaacuucauaccaugcucC......................	87	1	Bl1
....uaggaacuucauaccaugcucU......................	2317	1	Bl1
....uaggaacuucaGaccaugcucg......................	13	1	Bl1
....uaggaacuucGuaccaugcucg......................	5	1	Bl1
....uaggaacuucauaccaugAucg......................	1	1	Bl1
....uaggaacuucauaccaugcucg......................	50179	0	Bl1
....uaggGacuucauaccaugcucg......................	524	1	Bl1
....uCggaacuucauaccaugcucg......................	181	1	Bl1
....uaggaacuucaCaccaugcucg......................	11	1	Bl1
....uaggaacuCcauaccaugcucg......................	38	1	Bl1
....uaggaacuuGauaccaugcucg......................	1	1	Bl1
....Aaggaacuucauaccaugcucg......................	78	1	Bl1
....uaggaacuucauaccaugcAcg......................	9	1	Bl1
....uaggaacuucauaccaAgcucg......................	3	1	Bl1
....uaggaacuucauaAcaugcucg......................	22	1	Bl1
....uagCaacuucauaccaugcucg......................	5	1	Bl1
....uaggaacuucauaccaugcucgc.....................	44	0	Bl1
....uaggaacuucauaccaugcucgG.....................	5	1	Bl1
....uaggaacuucauaccaugcucgA.....................	942	1	Bl1
....uCggaacuucauaccaugcucgc.....................	1	1	Bl1

Star **Mature**

```
agugaagaaaggacaacagcuggguucucgagagcgagguauaaaguucuuacaaggaauuuaauugguaggaacuucauaccaugcucgcgaugauucaaaguccacaaca
....................................................................uaggaacuucauaccaugcucgcA....................    1     1     Bl1
....................................................................uaggaacuucauaccaugcucgcU....................    2     1     Bl1
....................................................................uaggaacuucauaccaugcucgGga...................    2     1     Bl1
.....................................................................aggaacuucauaccaugc.........................    2     0     Bl1
.....................................................................aggaUcuucauaccaugc.........................    1     1     Bl1
.....................................................................aggaacuucauaccaugcu........................    2     0     Bl1
.....................................................................aggaacuucauaccaugcC........................    2     1     Bl1
.....................................................................aggUacuucauaccaugcu........................    1     1     Bl1
.....................................................................aggaacuuAauaccaugcuc.......................    1     1     Bl1
.....................................................................aggaacuucauaccaugcuA.......................    1     1     Bl1
.....................................................................aggaacuucCuaccaugcuc.......................    2     1     Bl1
.....................................................................aggaacuucauaccaugcuc.......................    26    0     Bl1
.....................................................................aggaGcuucauaccaugcuc.......................    3     1     Bl1
.....................................................................Cggaacuucauaccaugcuc.......................    2     1     Bl1
.....................................................................aggaGcuucauaccaugcucg......................    4     1     Bl1
.....................................................................aggaacuucauaccaugcucA......................    6     1     Bl1
.....................................................................agCaacuucauaccaugcucg......................    5     1     Bl1
.....................................................................aggaacuuUauaccaugcucg......................    1     1     Bl1
.....................................................................Cggaacuucauaccaugcucg......................    6     1     Bl1
.....................................................................aggaacuucauaccaugcucC......................    1     1     Bl1
.....................................................................aggaacuucGuaccaugcucg......................    1     1     Bl1
.....................................................................aggaacuucauaccaugcucg......................    133   0     Bl1
.....................................................................aggaacuucauCccaugcucg......................    1     1     Bl1
.....................................................................aggaacuucauaccUugcucg......................    1     1     Bl1
.....................................................................aggaacuucauaccauCcucg......................    1     1     Bl1
.....................................................................aggaUcuucauaccaugcucg......................    3     1     Bl1
.....................................................................aggaacuuAauaccaugcucg......................    2     1     Bl1
.....................................................................aggaacuucCuaccaugcucg......................    4     1     Bl1
.....................................................................aggaacuucauaAcaugcucg......................    2     1     Bl1
.....................................................................aggaacuucauaccauUcucg......................    1     1     Bl1
.....................................................................aggUacuucauaccaugcucg......................    8     1     Bl1
.....................................................................Gggaacuucauaccaugcucg......................    1     1     Bl1
.....................................................................aggaacGucauaccaugcucg......................    1     1     Bl1
.....................................................................aggaacuucauaccaugcucU......................    4     1     Bl1
.....................................................................aggaacuucauaccaugcucgc.....................    1     0     Bl1
.....................................................................aggaacuucauaccaugcucgA.....................    3     1     Bl1
......................................................................Agaacuucauaccaugcu........................    1     1     Bl1
......................................................................ggaacuucauaccaugcuc.......................    1     0     Bl1
......................................................................ggaacuucauaccaugcucg......................    6     0     Bl1
......................................................................ggaacuucauaccaugcucU......................    1     1     Bl1
......................................................................ggaaUuucauaccaugcucg......................    1     1     Bl1
......................................................................Ugaacuucauaccaugcucg......................    1     1     Bl1
......................................................................Agaacuucauaccaugcucg......................    1     1     Bl1

................................agcgagguauaaaguucuuac...........................................................    3     0     He1
................................agcgagguauaaaguucuuaca..........................................................    3     0     He1
.................................gcgagguauaaaguucuuaca..........................................................    1     0     He1
..................................................................gAuaggaacuucauaccaugcucg......................    1     1     He1
...................................................................Cuaggaacuucauaccaugcuc.......................    8     1     He1
...................................................................guaggaacuucauaccaugcuc.......................    1     0     He1
...................................................................Cuaggaacuucauaccaugcucg......................    60    1     He1
...................................................................Uuaggaacuucauaccaugcucg......................    1     1     He1
....................................................................uaggGacuucauaccaug..........................    1     1     He1
....................................................................uaggaacuucauaccaCg..........................    2     1     He1
....................................................................uCggaacuucauaccaug..........................    1     1     He1
....................................................................uaggUacuucauaccaug..........................    1     1     He1
....................................................................uaCgaacuucauaccaug..........................    1     1     He1
....................................................................uaggaacuAcauaccaug..........................    1     1     He1
....................................................................uaggaacuucauacUaug..........................    1     1     He1
....................................................................uaggaacuucauaccaug..........................    85    0     He1
....................................................................uaggaacuucCuaccaug..........................    3     1     He1
....................................................................uaggGacuucauaccaugc.........................    3     1     He1
....................................................................Aaggaacuucauaccaugc.........................    2     1     He1
....................................................................uaggaacuucauGccaugc.........................    1     1     He1
....................................................................uaggaacuucauaccaugc.........................    236   0     He1
....................................................................uaggaacuucauaccaugU.........................    3     1     He1
....................................................................uaggaacuucCuaccaugc.........................    2     1     He1
....................................................................uaggaacuucauaccaugG.........................    1     1     He1
....................................................................uagUaacuucauaccaugc.........................    2     1     He1
....................................................................uaggaacuucauaccaugA.........................    66    1     He1
```

Star Mature

```
agugaagaaaggacaacagcuggguucucgagagcgagguauaaaguucuuacaaggaauuuaauugguaggaacuucauaccaugcucgcgaugauucaaaguccacaaca
....................................................................uaggUacuucauaccaugc.........................   2      1   He1
....................................................................uaggaacuuAauaccaugcu........................   1      1   He1
....................................................................Naggaacuucauaccaugcu........................   1      1   He1
....................................................................uaggaacuucauaccaugUu........................   3      1   He1
....................................................................uaggCacuucauaccaugcu........................   1      1   He1
....................................................................uaggaacuucaAaccaugcu........................   1      1   He1
....................................................................uaggaacuucauaccauUcu........................   3      1   He1
....................................................................uaggUacuucauaccaugcu........................   17     1   He1
....................................................................uaggaacuucauaccaugcG........................   9      1   He1
....................................................................uaggaacuucauCccaugcu........................   5      1   He1
....................................................................uaggaacuAcauaccaugcu........................   3      1   He1
....................................................................uaggGacuucauaccaugcu........................   21     1   He1
....................................................................uaggaacuucauaccaugcA........................   128    1   He1
....................................................................uaCgaacuucauaccaugcu........................   6      1   He1
....................................................................uagUaacuucauaccaugcu........................   2      1   He1
....................................................................Gaggaacuucauaccaugcu........................   1      1   He1
....................................................................uaggaacuucauaccaugcC........................   389    1   He1
....................................................................uaggaacuucauacUaugcu........................   2      1   He1
....................................................................uaggaacuucauaccaugcu........................   1466   0   He1
....................................................................uaggaacuucauaccUugcu........................   2      1   He1
....................................................................uaggaacuucauacGaugcu........................   1      1   He1
....................................................................uaggaacuucauaccauAcu........................   1      1   He1
....................................................................Aaggaacuucauaccaugcu........................   1      1   He1
....................................................................uaggaacuucCuaccaugcu........................   9      1   He1
....................................................................uaggaacuucauaccaAgcu........................   1      1   He1
....................................................................uCggaacuucauaccaugcu........................   7      1   He1
....................................................................uaggaacuucauaccaugGuc.......................   2      1   He1
....................................................................uaggaacuucauacUaugcuc.......................   10     1   He1
....................................................................uaggaacuucauaccaugcuG.......................   9      1   He1
....................................................................uaggaacuucCuaccaugcuc.......................   99     1   He1
....................................................................uaggaacuucauaAcaugcuc.......................   8      1   He1
....................................................................uaggaacuucauaccUugcuc.......................   14     1   He1
....................................................................uaggaacuucauaccaugcCc.......................   330    1   He1
....................................................................uaggaaUuucauaccaugcuc.......................   5      1   He1
....................................................................uaUgaacuucauaccaugcuc.......................   2      1   He1
....................................................................uaggaacuucauaccCugcuc.......................   2      1   He1
....................................................................uaAgaacuucauaccaugcuc.......................   5      1   He1
....................................................................uUggaacuucauaccaugcuc.......................   7      1   He1
....................................................................uaggaUcuucauaccaugcuc.......................   12     1   He1
....................................................................uaggaacuucauaccGugcuc.......................   4      1   He1
....................................................................uaggaacuuUauaccaugcuc.......................   31     1   He1
....................................................................uaggaacuucaGaccaugcuc.......................   3      1   He1
....................................................................uaggaacuucauaccauUcuc.......................   11     1   He1
....................................................................uaggaacuucauUccaugcuc.......................   3      1   He1
....................................................................uaggGacuucauaccaugcuc.......................   155    1   He1
....................................................................uaCgaacuucauaccaugcuc.......................   68     1   He1
....................................................................uaggaaGuucauaccaugcuc.......................   2      1   He1
....................................................................uaggaacuucauacAaugcuc.......................   2      1   He1
....................................................................uaggaacuucauaccaugcuc.......................   16169  0   He1
....................................................................uaggaacuucauaccauCcuc.......................   1      1   He1
....................................................................uaggaacuucauaUcaugcuc.......................   3      1   He1
....................................................................uGggaacuucauaccaugcuc.......................   5      1   He1
....................................................................uaggaCcuucauaccaugcuc.......................   1      1   He1
....................................................................Gaggaacuucauaccaugcuc.......................   7      1   He1
....................................................................uaggaacuucauaccaugcAc.......................   19     1   He1
....................................................................uaggaaAuucauaccaugcuc.......................   1      1   He1
....................................................................uaggaacuucauaccaugcuA.......................   144    1   He1
....................................................................uaggaacuAcauaccaugcuc.......................   26     1   He1
....................................................................Caggaacuucauaccaugcuc.......................   15     1   He1
....................................................................uaggaacuucaCaccaugcuc.......................   3      1   He1
....................................................................uaggaacuuAauaccaugcuc.......................   11     1   He1
....................................................................uaggUacuucauaccaugcuc.......................   202    1   He1
....................................................................uaggaacuucGuaccaugcuc.......................   4      1   He1
....................................................................uaggaacuucauaccaugcGc.......................   25     1   He1
....................................................................uaggaacuucauaccaugUuc.......................   8      1   He1
....................................................................uaggaacuCcauaccaugcuc.......................   16     1   He1
....................................................................uCggaacuucauaccaugcuc.......................   65     1   He1
....................................................................uaggaacuucauGccaugcuc.......................   5      1   He1
....................................................................uaggaacGucauaccaugcuc.......................   8      1   He1
....................................................................uagCaacuucauaccaugcuc.......................   1      1   He1
```

```
                                Star                              Mature
agugaagaaaggacaacagcuggguucucgagagcgagguauaaaguucuuacaaggaauuuaauugguaggaacuucauaccaugcucgcgaugauucaaaguccacaaca
....................................................................Naggaacuucauaccaugcuc.......................    14      1    He1
....................................................................Aaggaacuucauaccaugcuc.......................    36      1    He1
....................................................................uaggaacCucauaccaugcuc.......................    4       1    He1
....................................................................uaggaacuucauCccaugcuc.......................    27      1    He1
....................................................................uagAaacuucauaccaugcuc.......................    2       1    He1
....................................................................uaggaacAucauaccaugcuc.......................    1       1    He1
....................................................................uaggaGcuucauaccaugcuc.......................    1       1    He1
....................................................................uaggaacuucauaccaCgcuc.......................    2       1    He1
....................................................................uagUaacuucauaccaugcuc.......................    37      1    He1
....................................................................uaggaacuucauaccaugAuc.......................    1       1    He1
....................................................................uaggaacuucauaccauAcuc.......................    8       1    He1
....................................................................uaggGacuucauaccaugcucg......................    1436    1    He1
....................................................................uaggaacuucauaccaugAucg......................    7       1    He1
....................................................................uaggaacuucauacUaugcucg......................    83      1    He1
....................................................................uCggaacuucauaccaugcucg......................    476     1    He1
....................................................................uaggaacAucauaccaugcucg......................    17      1    He1
....................................................................uaggaacuucaCaccaugcucg......................    30      1    He1
....................................................................Caggaacuucauaccaugcucg......................    129     1    He1
....................................................................uaggaaAuucauaccaugcucg......................    3       1    He1
....................................................................Aaggaacuucauaccaugcucg......................    212     1    He1
....................................................................uaggaacuucauGccaugcucg......................    11      1    He1
....................................................................uaggaacuucauaccaugUucg......................    82      1    He1
....................................................................uaggaacuucauaccauUcucg......................    119     1    He1
....................................................................uaUgaacuucauaccaugcucg......................    29      1    He1
....................................................................uaggaacuNcauaccaugcucg......................    1       1    He1
....................................................................uaggaacuuGauaccaugcucg......................    3       1    He1
....................................................................uUggaacuucauaccaugcucg......................    44      1    He1
....................................................................uaggaacuucauaccUugcucg......................    119     1    He1
....................................................................uaggaacuucauaccauCcucg......................    7       1    He1
....................................................................uaggaacuGcauaccaugcucg......................    6       1    He1
....................................................................uaggaacuucauacGaugcucg......................    33      1    He1
....................................................................uaggaacuucaAaccaugcucg......................    7       1    He1
....................................................................uagCaacuucauaccaugcucg......................    6       1    He1
....................................................................uaggaacuucauaccaAgcucg......................    15      1    He1
....................................................................uaggUacuucauaccaugcucg......................    1963    1    He1
....................................................................uagUaacuucauaccaugcucg......................    325     1    He1
....................................................................uaggaacuucauacAaugcucg......................    14      1    He1
....................................................................uaggaacCucauaccaugcucg......................    27      1    He1
....................................................................uaAgaacuucauaccaugcucg......................    21      1    He1
....................................................................uaggaacuucauaccaugcucU......................    1095    1    He1
....................................................................uaggaacuucauaAcaugcucg......................    32      1    He1
....................................................................uaggaacuucGuaccaugcucg......................    20      1    He1
....................................................................uaCgaacuucauaccaugcucg......................    577     1    He1
....................................................................uaggaaUuucauaccaugcucg......................    11      1    He1
....................................................................uaggaacuucauaccaGgcucg......................    2       1    He1
....................................................................uaggaacuCcauaccaugcucg......................    76      1    He1
....................................................................uaggaacuuUauaccaugcucg......................    90      1    He1
....................................................................uaggaacuucauaGcaugcucg......................    2       1    He1
....................................................................uaggaGcuucauaccaugcucg......................    21      1    He1
....................................................................uaggaacGucauaccaugcucg......................    42      1    He1
....................................................................uaggaacuucauaccaugcucg......................    138746  0    He1
....................................................................uaggaCcuucauaccaugcucg......................    6       1    He1
....................................................................uaggaacuucauaccaugcAcg......................    42      1    He1
....................................................................uaggaacuucauaccaugcCcg......................    296     1    He1
....................................................................uaggaacuucauUccaugcucg......................    15      1    He1
....................................................................uaggaacuAcauaccaugcucg......................    170     1    He1
....................................................................uaggaacuucauaUcaugcucg......................    48      1    He1
....................................................................uaggaaGuucauaccaugcucg......................    6       1    He1
....................................................................uaggaacuucCuaccaugcucg......................    683     1    He1
....................................................................uaggaUcuucauaccaugcucg......................    132     1    He1
....................................................................uaggaacuucauaccGugcucg......................    35      1    He1
....................................................................uaggaacuucauaccaugcuAg......................    91      1    He1
....................................................................Naggaacuucauaccaugcucg......................    105     1    He1
....................................................................uaggaacuucauaccauAcucg......................    121     1    He1
....................................................................uagAaacuucauaccaugcucg......................    17      1    He1
....................................................................uaggaacuucauaccaugcucA......................    1431    1    He1
....................................................................uaggaacuucauaccaugcGcg......................    94      1    He1
....................................................................uaggaacuucauaccaugcucC......................    159     1    He1
....................................................................Gaggaacuucauaccaugcucg......................    28      1    He1
....................................................................uaggaacuucauCccaugcucg......................    154     1    He1
```

```
                                Star                                  Mature
agugaagaaaggacaacagcugggguucucgagagcgagguauaaaguucuuacaaggaauuuaauugguaggaacuucauaccaugcucgcgaugauucaaaguccacaaca
.....................................................................uaggaacuucauaccCugcucg......................   17    1   He1
.....................................................................uaggaacuuAauaccaugcucg......................   107   1   He1
.....................................................................uaggaacuucauaccaugcuGg......................   7     1   He1
.....................................................................uGggaacuucauaccaugcucg......................   68    1   He1
.....................................................................uaggaacuucauaccaCgcucg......................   21    1   He1
.....................................................................uaggaacuucaGaccaugcucg......................   23    1   He1
.....................................................................uaggCacuucauaccaugcucg......................   11    1   He1
.....................................................................uaggaacuucauaccaugGucg......................   17    1   He1
.....................................................................uaggaacuucauaccaugcucgG.....................   9     1   He1
.....................................................................uaggaacuuAauaccaugcucgc.....................   1     1   He1
.....................................................................uaggaacuucauaccaugcucgA.....................   1115  1   He1
.....................................................................uaggaacuucauaccaugcucgc.....................   49    0   He1
.....................................................................uaggaacuucauaccaugcucgcC....................   2     1   He1
.....................................................................uaggaacuucauaccaugcucgcCa...................   2     1   He1
......................................................................Cggaacuucauaccaugcu........................   1     1   He1
......................................................................aggaacuucauaccaugcu........................   2     0   He1
......................................................................aggaacuuAauaccaugcu........................   1     1   He1
......................................................................aggaacuucauaccaGgcuc.......................   1     1   He1
......................................................................aggUacuucauaccaugcuc.......................   3     1   He1
......................................................................aAgaacuucauaccaugcuc.......................   1     1   He1
......................................................................aggaacuucCuaccaugcuc.......................   1     1   He1
......................................................................aggaacuuUauaccaugcuc.......................   1     1   He1
......................................................................aggaacuucauGccaugcuc.......................   1     1   He1
......................................................................aggaGcuucauaccaugcuc.......................   1     1   He1
......................................................................agCaacuucauaccaugcuc.......................   3     1   He1
......................................................................aggaUcuucauaccaugcuc.......................   3     1   He1
......................................................................aggaacuucauaccaAgcuc.......................   1     1   He1
......................................................................aggaacuucauaccaugcAc.......................   1     1   He1
......................................................................aggaacuucauUccaugcuc.......................   1     1   He1
......................................................................Cggaacuucauaccaugcuc.......................   3     1   He1
......................................................................aggaacuucauaccaugcuc.......................   50    0   He1
......................................................................aggaacuucauaAcaugcuc.......................   1     1   He1
......................................................................aggaacuuAauaccaugcucg......................   22    1   He1
......................................................................aggaacuucauacUaugcucg......................   1     1   He1
......................................................................agCaacuucauaccaugcucg......................   18    1   He1
......................................................................aggaacuucauaccaCgcucg......................   1     1   He1
......................................................................aggaacuucauaccUugcucg......................   3     1   He1
......................................................................aggaacuucaAaccaugcucg......................   2     1   He1
......................................................................aggaacuucauaccaugAucg......................   1     1   He1
......................................................................aggaCcuucauaccaugcucg......................   1     1   He1
......................................................................aggaacuAcauaccaugcucg......................   3     1   He1
......................................................................aggaacuucauaUcaugcucg......................   1     1   He1
......................................................................aggaacuucauaccaugcucC......................   1     1   He1
......................................................................aggaacuucauaAcaugcucg......................   2     1   He1
......................................................................aggaacuucCuaccaugcucg......................   8     1   He1
......................................................................aggaacuuUauaccaugcucg......................   1     1   He1
......................................................................aggaacuucauaccaugUucg......................   3     1   He1
......................................................................aggaacuucauaccaugcGcg......................   3     1   He1
......................................................................aggaacuucauaccaugcucg......................   421   0   He1
......................................................................aAgaacuucauaccaugcucg......................   2     1   He1
......................................................................Uggaacuucauaccaugcucg......................   1     1   He1
......................................................................aggaUcuucauaccaugcucg......................   22    1   He1
......................................................................aggaacuucauaccauCcucg......................   3     1   He1
......................................................................aggaacuucauaccGugcucg......................   3     1   He1
......................................................................aggUacuucauaccaugcucg......................   20    1   He1
......................................................................agUaacuucauaccaugcucg......................   1     1   He1
......................................................................aggaacuucauacAaugcucg......................   1     1   He1
......................................................................aggaacuucauaccaGgcucg......................   3     1   He1
......................................................................aggaacuucauaccaugcucU......................   1     1   He1
......................................................................aggaacuCcauaccaugcucg......................   5     1   He1
......................................................................aggaGcuucauaccaugcucg......................   15    1   He1
......................................................................aCgaacuucauaccaugcucg......................   4     1   He1
......................................................................aggaacCucauaccaugcucg......................   1     1   He1
......................................................................Cggaacuucauaccaugcucg......................   21    1   He1
......................................................................aggaacuucauaccaugcucA......................   5     1   He1
......................................................................aggaacuucauaccCugcucg......................   1     1   He1
......................................................................aggaacuucauUccaugcucg......................   2     1   He1
......................................................................aggaacuucauaccaugcCcg......................   4     1   He1
......................................................................aggaacuucauaccaugcucgA.....................   3     1   He1
.......................................................................ggaacuucauaccaugcu........................   1     0   He1
```

Star Mature

```
agugaagaaaggacaacagcuggguucucgagagcgagguauaaaguucuuacaaggaauuuaauugguaggaacuucauaccaugcucgcgaugauucaaaguccacaaca
......................................................................ggaacuucauaccaugcuc.......................  1     0  He1
......................................................................ggaacuucauaccaugAuc.......................  1     1  He1
......................................................................ggaacuucauCccaugcuc.......................  1     1  He1
......................................................................Ugaacuucauaccaugcucg......................  6     1  He1
......................................................................ggaaUuucauaccaugcucg......................  1     1  He1
......................................................................ggaacuucauaccaugcucg......................  10    0  He1

...................................................................Uuaggaacuucauaccaugcucg......................  1     1  Ta1
...................................................................Cuaggaacuucauaccaugcucg......................  12    1  Ta1
....................................................................uaggaacuucauaccaug..........................  22    0  Ta1
....................................................................uaCgaacuucauaccaugc.........................  1     1  Ta1
....................................................................uaggaUcuucauaccaugc.........................  1     1  Ta1
....................................................................uaggGacuucauaccaugc.........................  1     1  Ta1
....................................................................uaggaacuCcauaccaugc.........................  1     1  Ta1
....................................................................uaggaacuucauaccaugc.........................  84    0  Ta1
....................................................................uaggaacuucauaccaugA.........................  23    1  Ta1
....................................................................uCggaacuucauaccaugc.........................  1     1  Ta1
....................................................................uaggUacuucauaccaugc.........................  1     1  Ta1
....................................................................uaggaacuucauacUaugcu........................  2     1  Ta1
....................................................................uaggUacuucauaccaugcu........................  5     1  Ta1
....................................................................uaggCacuucauaccaugcu........................  1     1  Ta1
....................................................................Gaggaacuucauaccaugcu........................  1     1  Ta1
....................................................................uaCgaacuucauaccaugcu........................  1     1  Ta1
....................................................................Caggaacuucauaccaugcu........................  1     1  Ta1
....................................................................uGggaacuucauaccaugcu........................  1     1  Ta1
....................................................................uaggaacuucauaccaugcA........................  12    1  Ta1
....................................................................uagUaacuucauaccaugcu........................  4     1  Ta1
....................................................................uaggaacuucauaccaugcC........................  9     1  Ta1
....................................................................uaggaacuCcauaccaugcu........................  1     1  Ta1
....................................................................uaggaacuucauacGaugcu........................  1     1  Ta1
....................................................................uaggaacuAcauaccaugcu........................  2     1  Ta1
....................................................................uaggaacuucCuaccaugcu........................  2     1  Ta1
....................................................................uCggaacuucauaccaugcu........................  3     1  Ta1
....................................................................uaggaacuucauaccaugcu........................  466   0  Ta1
....................................................................uaggGacuucauaccaugcu........................  4     1  Ta1
....................................................................uaggaacuucauaccaugUuc.......................  3     1  Ta1
....................................................................uaggaacuucCuaccaugcuc.......................  32    1  Ta1
....................................................................uaggaacGucauaccaugcuc.......................  2     1  Ta1
....................................................................Gaggaacuucauaccaugcuc.......................  2     1  Ta1
....................................................................uaggaaUuucauaccaugcuc.......................  1     1  Ta1
....................................................................uaggCacuucauaccaugcuc.......................  2     1  Ta1
....................................................................uaggaacuucauaAcaugcuc.......................  2     1  Ta1
....................................................................uaggaacuucauaccaugcuA.......................  11    1  Ta1
....................................................................uaggaacuucauaccaugcuc.......................  4218  0  Ta1
....................................................................uaggaacuucauaUcaugcuc.......................  1     1  Ta1
....................................................................uaggaacuucauaccUugcuc.......................  4     1  Ta1
....................................................................uaggaacuuAauaccaugcuc.......................  4     1  Ta1
....................................................................uaggaacuucauaccauUcuc.......................  5     1  Ta1
....................................................................uaggaacuucauUccaugcuc.......................  1     1  Ta1
....................................................................Aaggaacuucauaccaugcuc.......................  6     1  Ta1
....................................................................uCggaacuucauaccaugcuc.......................  14    1  Ta1
....................................................................Naggaacuucauaccaugcuc.......................  4     1  Ta1
....................................................................uaggGacuucauaccaugcuc.......................  48    1  Ta1
....................................................................uaggaacuucauaccaugcGc.......................  2     1  Ta1
....................................................................uaggaacuuUauaccaugcuc.......................  11    1  Ta1
....................................................................uaggaacuucauaccauCcuc.......................  1     1  Ta1
....................................................................uaggaacuucauacGaugcuc.......................  2     1  Ta1
....................................................................uaggaacuucauaccauAcuc.......................  1     1  Ta1
....................................................................uaAgaacuucauaccaugcuc.......................  1     1  Ta1
....................................................................uaggaacuucauacUaugcuc.......................  6     1  Ta1
....................................................................uaUgaacuucauaccaugcuc.......................  1     1  Ta1
....................................................................uUggaacuucauaccaugcuc.......................  1     1  Ta1
....................................................................uaggaacuucauCccaugcuc.......................  2     1  Ta1
....................................................................uaggaacAucauaccaugcuc.......................  2     1  Ta1
....................................................................uagUaacuucauaccaugcuc.......................  7     1  Ta1
....................................................................uaggaacuucauaccGugcuc.......................  1     1  Ta1
....................................................................Caggaacuucauaccaugcuc.......................  7     1  Ta1
....................................................................uaggUacuucauaccaugcuc.......................  51    1  Ta1
....................................................................uaggaUcuucauaccaugcuc.......................  3     1  Ta1
....................................................................uaggaacuuGauaccaugcuc.......................  1     1  Ta1
```

Star **Mature**

```
agugaagaaaggacaacagcuggguucucgagagcgagguauaaaguucuuacaaggaauuuaauugguaggaacuucauaccaugcucgcgaugauucaaaguccacaaca
....................................................................uaCgaacuucauaccaugcuc.......................    24      1    Ta1
....................................................................uaggaacCucauaccaugcuc.......................    1       1    Ta1
....................................................................uaggaGcuucauaccaugcuc.......................    1       1    Ta1
....................................................................uaggaacuAcauaccaugcuc.......................    2       1    Ta1
....................................................................uaggaacuucauaccaCgcucg......................    1       1    Ta1
....................................................................uaggaacuucCuaccaugcucg......................    138     1    Ta1
....................................................................uaggaacuucauaccauUcucg......................    30      1    Ta1
....................................................................uaggaacuCcauaccaugcucg......................    13      1    Ta1
....................................................................uaggaacuucauacGaugcucg......................    4       1    Ta1
....................................................................uaggaacuucauaccaAgcucg......................    2       1    Ta1
....................................................................uaggaacuucauaccaugcCcg......................    4       1    Ta1
....................................................................uaggaacAucauaccaugcucg......................    6       1    Ta1
....................................................................uaggaacGucauaccaugcucg......................    12      1    Ta1
....................................................................uaggaacuucauaccaugcucC......................    7       1    Ta1
....................................................................uUggaacuucauaccaugcucg......................    13      1    Ta1
....................................................................uaggaacuucGuaccaugcucg......................    6       1    Ta1
....................................................................uaggaUcuucauaccaugcucg......................    20      1    Ta1
....................................................................uCggaacuucauaccaugcucg......................    94      1    Ta1
....................................................................uaggaacuucauaccaugcuAg......................    10      1    Ta1
....................................................................uaggGacuucauaccaugcucg......................    321     1    Ta1
....................................................................uagCaacuucauaccaugcucg......................    2       1    Ta1
....................................................................uaggaacuucauaccUugcucg......................    19      1    Ta1
....................................................................uaggaacuAcauaccaugcucg......................    31      1    Ta1
....................................................................uaggaacuucauacAaugcucg......................    3       1    Ta1
....................................................................uaggaacuuUauaccaugcucg......................    20      1    Ta1
....................................................................uaggaacuucaGaccaugcucg......................    3       1    Ta1
....................................................................uaggaacuucauacUaugcucg......................    15      1    Ta1
....................................................................uaggaacuucauaccaugAucg......................    2       1    Ta1
....................................................................uaggaGcuucauaccaugcucg......................    6       1    Ta1
....................................................................uaggaacuucauaccaugcGcg......................    14      1    Ta1
....................................................................uaggaacuucauGccaugcucg......................    3       1    Ta1
....................................................................uaggaacuucauaccaugcAcg......................    1       1    Ta1
....................................................................uaggaacuucauaccauCcucg......................    2       1    Ta1
....................................................................uaggaacuucaAaccaugcucg......................    2       1    Ta1
....................................................................uaggaacuucauaccaugcucA......................    48      1    Ta1
....................................................................uaggaCcuucauaccaugcucg......................    1       1    Ta1
....................................................................uaggaacuucauUccaugcucg......................    5       1    Ta1
....................................................................uaggaacuucauaUcaugcucg......................    11      1    Ta1
....................................................................uaggaacuucauaccauAcucg......................    38      1    Ta1
....................................................................uaggaacuucauaccaugcucU......................    530     1    Ta1
....................................................................uaggaacCucauaccaugcucg......................    2       1    Ta1
....................................................................uaUgaacuucauaccaugcucg......................    1       1    Ta1
....................................................................uGggaacuucauaccaugcucg......................    8       1    Ta1
....................................................................Aaggaacuucauaccaugcucg......................    45      1    Ta1
....................................................................uaggaaGuucauaccaugcucg......................    2       1    Ta1
....................................................................uaggaacuucauaccGugcucg......................    7       1    Ta1
....................................................................uaggaacuucauaAcaugcucg......................    7       1    Ta1
....................................................................uaAgaacuucauaccaugcucg......................    5       1    Ta1
....................................................................Naggaacuucauaccaugcucg......................    27      1    Ta1
....................................................................uaggUacuucauaccaugcucg......................    402     1    Ta1
....................................................................uaggaacuucauaccaugcuGg......................    3       1    Ta1
....................................................................uaggaacuucauaccaugUucg......................    16      1    Ta1
....................................................................uaggaaUuucauaccaugcucg......................    6       1    Ta1
....................................................................uaggaacuucauCccaugcucg......................    32      1    Ta1
....................................................................uaCgaacuucauaccaugcucg......................    120     1    Ta1
....................................................................uaggaacuucauaccCugcucg......................    3       1    Ta1
....................................................................Caggaacuucauaccaugcucg......................    36      1    Ta1
....................................................................uaggaacuucauaccaugcucg......................    28702   0    Ta1
....................................................................uaggaacuucauaccaugGucg......................    5       1    Ta1
....................................................................uaggaaAuucauaccaugcucg......................    1       1    Ta1
....................................................................uagUaacuucauaccaugcucg......................    71      1    Ta1
....................................................................uaggCacuucauaccaugcucg......................    5       1    Ta1
....................................................................uaggaacuucauaGcaugcucg......................    1       1    Ta1
....................................................................uaggaacuuAauaccaugcucg......................    21      1    Ta1
....................................................................uaggaacuNcauaccaugcucg......................    1       1    Ta1
....................................................................uaggaacuuGauaccaugcucg......................    2       1    Ta1
....................................................................uagAaacuucauaccaugcucg......................    4       1    Ta1
....................................................................Gaggaacuucauaccaugcucg......................    8       1    Ta1
....................................................................uaggaacuucaCaccaugcucg......................    1       1    Ta1
....................................................................uaggaacuucauaccaugcucgA.....................    460     1    Ta1
```

```
                                 Star                                Mature
agugaagaaaggacaacagcugggguucucgagagcgagguauaaaguucuuacaaggaauuuaauugguaggaacuucauaccaugcucgcgaugauucaaaguccacaaca
.....................................................................uaggaacuucauaccaugcucgc.....................    2    0    Ta1
......................................................................aggaacuucauaccaugcu........................    3    0    Ta1
......................................................................aggaacuucauaccaugcuc.......................    9    0    Ta1
......................................................................aggaacuuUauaccaugcuc.......................    1    1    Ta1
......................................................................aggaaUuucauaccaugcuc.......................    1    1    Ta1
......................................................................aggaUcuucauaccaugcuc.......................    1    1    Ta1
......................................................................Cggaacuucauaccaugcuc.......................    1    1    Ta1
......................................................................aCgaacuucauaccaugcucg......................    1    1    Ta1
......................................................................aggaacuuAauaccaugcucg......................    2    1    Ta1
......................................................................aggaacuucauaAcaugcucg......................    1    1    Ta1
......................................................................aggaacuucauaccaugcucg......................    99   0    Ta1
......................................................................aggaaUuucauaccaugcucg......................    1    1    Ta1
......................................................................aggaacuucauaccaugUucg......................    1    1    Ta1
......................................................................aggaacuucCuaccaugcucg......................    3    1    Ta1
......................................................................aggaacuucauaccaugcucU......................    3    1    Ta1
......................................................................aggaacuucauacUaugcucg......................    1    1    Ta1
......................................................................Cggaacuucauaccaugcucg......................    2    1    Ta1
......................................................................agAaacuucauaccaugcucg......................    1    1    Ta1
......................................................................aggaacuGcauaccaugcucg......................    2    1    Ta1
......................................................................aggaacGucauaccaugcucg......................    1    1    Ta1
......................................................................aggaGcuucauaccaugcucg......................    4    1    Ta1
......................................................................aAgaacuucauaccaugcucg......................    2    1    Ta1
......................................................................aggaUcuucauaccaugcucg......................    5    1    Ta1
......................................................................agCaacuucauaccaugcucg......................    2    1    Ta1
......................................................................aggaacuucauaccaugcGcg......................    1    1    Ta1
......................................................................aggaacuucauaccauUcucg......................    2    1    Ta1
......................................................................aggUacuucauaccaugcucg......................    5    1    Ta1
......................................................................aggaacuucauaccaugcucgA.....................    4    1    Ta1
.......................................................................ggaacuucauaccaugcu........................    1    0    Ta1
.......................................................................ggaacuucauaccaugcucg......................    4    0    Ta1
.......................................................................gAaacuucauaccaugcucg......................    1    1    Ta1
```

Provisional ID	:	Sc28yqQ_4222_2911
Score total	:	4276.5
Score for star read(s)	:	3.9
Score for read counts	:	4266.3
Score for mfe	:	1.6
Score for randfold	:	1.6
Score for cons. seed	:	3
Total read count	:	8380
Mature read count	:	8212
Loop read count	:	0
Star read count	:	168

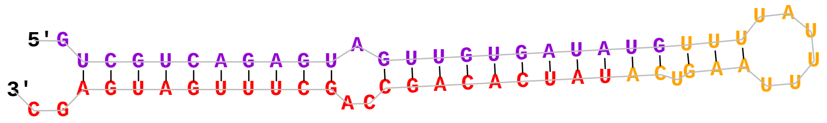

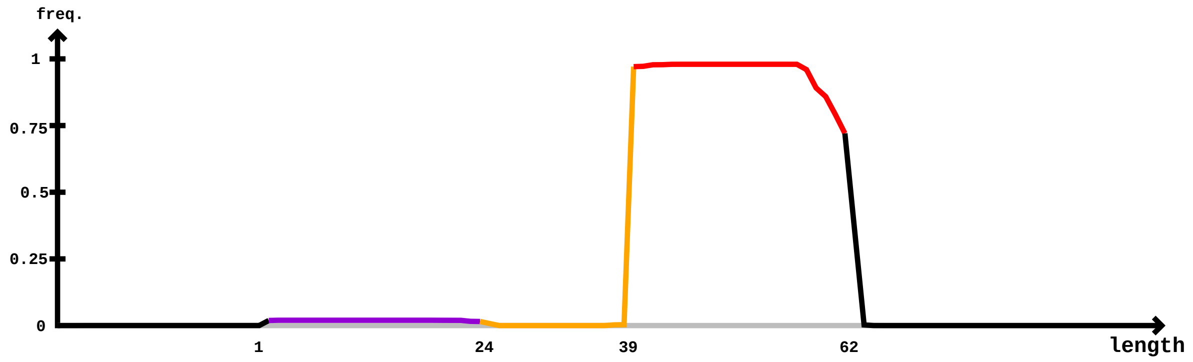

Star Mature

5'- auauuaaucccacgucuucggucgucagaguaguugugauauguuuuauuuuaagucauaucacagccagcuuugaugagcagagaugcagguuucaagcacauuuucuuaau -3'	obs		
auauuaaucccacgucuucgggucgucagaguaguugugauauguuuuauuuuaagucauaucacagccagcuuugaugagcagagaugcagguuucaagcacauuuucuuaau	exp		
........((..(((((..(.((((((((((.((((((((((((((......))).)))))))))))..)))))))))).)..)))))..))....................	reads	mm	sample
....................gucgucagaguaguugugaua..	6	0	Br1
....................gucgucagaguaguugugauau...	1	0	Br1
....................gucgucagaguaguugugauaug..	19	0	Br1
....................gucgAcagaguaguugugauaug..	1	1	Br1
....................gucgucagCguaguugugauaug..	2	1	Br1
....................gucgGcagaguaguugugauaug..	1	1	Br1
....................gucgucagaguaguugugauaugC...	1	1	Br1
.....................ucgucagaguaguugugauaug..	1	0	Br1
....cauaucacagccagcuuugau.....................................	1	0	Br1
....cauaucacagccagcuuugaug....................................	1	0	Br1
....Aauaucacagccagcuuugaugagc.................................	1	1	Br1
....auaucacagccagcuuugaugagc.................................	2	0	Br1
....uaucacagccCgcuuuga......................................	1	1	Br1
....uaucacagccagcuuuga......................................	6	0	Br1
....Caucacagccagcuuugau.....................................	1	1	Br1
....uaucUcagccagcuuugau.....................................	2	1	Br1
....uaucacagccCgcuuugau.....................................	1	1	Br1
....uaucacagccagcuuugau.....................................	44	0	Br1
....uaucacagccagcuuugaC.....................................	2	1	Br1
....uaCcacagccagcuuugau.....................................	1	1	Br1
....uaucacagccagcuuugaA.....................................	4	1	Br1
....uaucGcagccagcuuugau.....................................	1	1	Br1
....Aaucacagccagcuuugau.....................................	1	1	Br1
....uaucacagccagcuuugauA....................................	4	1	Br1
....uaucacagccagcuuugaug....................................	38	0	Br1
....uaucacagccagcuuugauU....................................	2	1	Br1
....uaucacagccagAuuugaug....................................	1	1	Br1
....uaucacagccagcuuugauga...................................	108	0	Br1
....Naucacagccagcuuugauga...................................	1	1	Br1
....uaucacagUcagcuuugauga...................................	1	1	Br1
....uauAacagccagcuuugauga...................................	1	1	Br1
....uaucacagccagcCuugauga...................................	1	1	Br1
....uaucacagccagcuuugaugaA..................................	5	1	Br1

Star **Mature**

```
auauuaaucccacgucuucggucgucagaguaguugugauauguuuuauuuuaagucauaucacagccagcuuugaugagcagagaugcagguuucaagcacauuuucuuaau
..........................................................uaucacagccagcuuCgaugag.................................   1     1     Br1
..........................................................uaucacagAcagcuuugaugag.................................   1     1     Br1
..........................................................uaucacagccagcuuugaugCg.................................   1     1     Br1
..........................................................uCucacagccagcuuugaugag.................................   1     1     Br1
..........................................................uaucacagccUgcuuugaugag.................................   1     1     Br1
..........................................................uUucacagccagcuuugaugag.................................   1     1     Br1
..........................................................uaCcacagccagcuuugaugag.................................   1     1     Br1
..........................................................uaucacagccagcuuugaugag.................................   97    0     Br1
..........................................................uaucUcagccagcuuugaugag.................................   1     1     Br1
..........................................................uaucacagAcagcuuugaugagc................................   5     1     Br1
..........................................................uaucacagccagcuuugaugaUc................................   1     1     Br1
..........................................................Aaucacagccagcuuugaugagc................................   1     1     Br1
..........................................................uaucacagccCgcuuugaugagc................................   4     1     Br1
..........................................................uaucacagccagAuuugaugagc................................   1     1     Br1
..........................................................uaCcacagccagcuuugaugagc................................   4     1     Br1
..........................................................uaucacagccagcuuugUugagc................................   1     1     Br1
..........................................................uaucacagccagUuuugaugagc................................   1     1     Br1
..........................................................uaucacagccagcCuugaugagc................................   2     1     Br1
..........................................................uaucGcagccagcuuugaugagc................................   3     1     Br1
..........................................................uaucacagccagcuuugaugagA................................   26    1     Br1
..........................................................uaucacagccagcuuugaugagc................................   961   0     Br1
..........................................................uaucUcagccagcuuugaugagc................................   14    1     Br1
..........................................................uaucacagUcagcuuugaugagc................................   1     1     Br1
..........................................................uaucacagccUgcuuugaugagc................................   1     1     Br1
..........................................................uaucacagccagcuGugaugagc................................   1     1     Br1
..........................................................uaucacagccagcuuugaugaCc................................   6     1     Br1
..........................................................uaucacagcUagcuuugaugagc................................   1     1     Br1
..........................................................uauUacagccagcuuugaugagc................................   3     1     Br1
..........................................................Caucacagccagcuuugaugagc................................   2     1     Br1
..........................................................uaucacaUccagcuuugaugagc................................   1     1     Br1
..........................................................uaucacagccagcuuGgaugagc................................   2     1     Br1
..........................................................uaucacagccagcuuugauCagc................................   1     1     Br1
..........................................................uaucaUagccagcuuugaugagc................................   1     1     Br1
..........................................................uaucacaAccagcuuugaugagc................................   1     1     Br1
..........................................................uaucacagccagcuuugauUagc................................   3     1     Br1
..........................................................uaucUcagccagcuuugaugagca...............................   1     1     Br1
..........................................................uaucacagccagcuuugaugagcU...............................   9     1     Br1
..........................................................uaucacagccaUcuuugaugagca...............................   17    1     Br1
..........................................................uaucacagccaCcuuugaugagca...............................   12    1     Br1
..........................................................uaucacagAcagcuuugaugagca...............................   1     1     Br1
..........................................................uaucacagccagcuuugaugagGa...............................   4     1     Br1
..........................................................uaucacagccagcuuugaugagca...............................   153   0     Br1
..........................................................uaucacagccaAcuuugaugagca...............................   6     1     Br1
..........................................................uaucacagccagcuuugaugaAca...............................   1     1     Br1
..........................................................uaucacagccagcuuugaugagcC...............................   5     1     Br1
..........................................................Gaucacagccagcuuugaugagca...............................   1     1     Br1
..........................................................uaucacagccagcuuugaugagcaC..............................   1     1     Br1
..........................................................uaucacagccagcuuugaugagcaA..............................   3     1     Br1
...........................................................aucacagccagcuuugaugagc................................   1     0     Br1
............................................................ucacagccagcuuugauga..................................   1     0     Br1
............................................................ucacagccagcuuugaugagc................................   2     0     Br1
............................................................ucacagccagcuuugaugagca...............................   2     0     Br1
.............................................................cacagccagcuuugaugagc................................   1     0     Br1
..............................................................acagccagcuuugaugag.................................   1     0     Br1
..............................................................acagccagcuuugaugagc................................   2     0     Br1

....................gucgucagaguaguuguga..........................................................................   1     0     Le1
....................gucgucagaguaguugugaua........................................................................   3     0     Le1
.....................ucgucagaguaguugugauaug......................................................................   1     0     Le1
........................................................cauaucacagccagcuuugau....................................   1     0     Le1
........................................................cauaucacagccagcuuugaugagAa...............................   1     1     Le1
.........................................................auaucacagccagcuuug......................................   1     0     Le1
..........................................................uaucacagccagcuuuga.....................................   22    0     Le1
..........................................................uauUacagccagcuuuga.....................................   1     1     Le1
..........................................................uaucGcagccagcuuuga.....................................   1     1     Le1
..........................................................uaucacagccagcuuuU......................................   1     1     Le1
..........................................................uaucGcagccagcuuugau....................................   1     1     Le1
..........................................................uaucacagccagcuuugau....................................   52    0     Le1
..........................................................uaucacagccagcuuugaG....................................   1     1     Le1
..........................................................uaucacagccagcuuugaA....................................   4     1     Le1
```

```
                    Star                                   Mature
auauuaaucccacgucuucggucgucagaguaguugugauauguuuuauuuuaaggucauaucacagccagcuuugaugagcagagaugcagguuucaagcacauuuucuuaau
...........................................................uaucUcagccagcuuugau....................................   1     1   Le1
...........................................................uaucacagccagcuuugaug...................................   13    0   Le1
...........................................................uaucacagccagcuuugauA...................................   2     1   Le1
...........................................................uaucUcagccagcuuugaug...................................   1     1   Le1
...........................................................uaucacagccagcuuugauga..................................   26    0   Le1
...........................................................uaucacagccagcuuugaugaA.................................   1     1   Le1
...........................................................uaucacagccagcuuugaugag.................................   36    0   Le1
...........................................................uaucaUagccagcuuugaugag.................................   1     1   Le1
...........................................................uaucUcagccagcuuugaugagc................................   2     1   Le1
...........................................................Caucacagccagcuuugaugagc................................   2     1   Le1
...........................................................uaucacagccCgcuuugaugagc................................   1     1   Le1
...........................................................uCucacagccagcuuugaugagc................................   3     1   Le1
...........................................................uaucacagccagcuuugaugagc................................   218   0   Le1
...........................................................Naucacagccagcuuugaugagc................................   1     1   Le1
...........................................................uaucacagUcagcuuugaugagc................................   2     1   Le1
...........................................................uaucacagccagcuuugaugaCc................................   1     1   Le1
...........................................................uaucacagAcagcuuugaugagc................................   2     1   Le1
...........................................................uaucacagccagcuuugaugCgc................................   1     1   Le1
...........................................................uaucGcagccagcuuugaugagc................................   1     1   Le1
...........................................................uauUacagccagcuuugaugagc................................   1     1   Le1
...........................................................uaucacagccagcuuugaugagA................................   4     1   Le1
...........................................................uaucacagccagcuuugaugagcC...............................   2     1   Le1
...........................................................uaucacagccCgcuuugaugagca...............................   2     1   Le1
...........................................................uaucacagccagcuuugaugagca...............................   28    0   Le1
...........................................................uaucacagccagcuuugaugagGa...............................   2     1   Le1
...........................................................uaucacagccaCcuuugaugagca...............................   14    1   Le1
...........................................................uaucacagccagcuGugaugagca...............................   1     1   Le1
...........................................................uaucGcagccagcuuugaugagca...............................   1     1   Le1
...........................................................uaucacagccaUcuuugaugagca...............................   6     1   Le1
...........................................................uaucacagccagcuuugaugagcU...............................   6     1   Le1
...........................................................uaucacagccagcuuugaugagcaU..............................   2     1   Le1
...........................................................uaucacagccagcuuugaugagcaA..............................   1     1   Le1
............................................................aucacagccagcuuugaugagc................................   1     0   Le1
............................................................aucaUagccagcuuugaugagca...............................   1     1   Le1
.............................................................ucacagccagcuuugaugagc................................   6     0   Le1
.............................................................ucacagccCgcuuugaugagc................................   1     1   Le1
.............................................................ucacagccagcuuugaugagca...............................   2     0   Le1
...............................................................acagccagcuuugaugagca...............................   1     0   Le1

.uauuaaucccacgucuucg..............................................................................................   1     0   Le5
....................gucgucagaguaguugugaua.........................................................................   7     0   Le5
....................gucgAcagaguaguugugauaug.......................................................................   1     1   Le5
....................gucgucagaguaguugugauaug.......................................................................   6     0   Le5
....................gucgucagaguaguugugauaugu......................................................................   1     0   Le5
.....................ucgucagaguaguugugauau........................................................................   1     0   Le5
.....................ucguGagaguaguugugauaug.......................................................................   1     1   Le5
.........................................................cauaucacagccagcuuugau....................................   1     0   Le5
...........................................................uaucUcagccagcuuuga.....................................   1     1   Le5
...........................................................uaucacagccagcuuuga.....................................   14    0   Le5
...........................................................uaucacagccagcuuugaA....................................   6     1   Le5
...........................................................uaucacagccagcuuCgau....................................   1     1   Le5
...........................................................uaucacagccagcuuugau....................................   67    0   Le5
...........................................................Caucacagccagcuuugau....................................   1     1   Le5
...........................................................uaucacagccagcuuugaC....................................   4     1   Le5
...........................................................uaucacagccagcuuugauA...................................   3     1   Le5
...........................................................uaucacagccagcuuugaug...................................   22    0   Le5
...........................................................uaucacagAcagcuuugauga..................................   1     1   Le5
...........................................................uaucacagccagcuuugauga..................................   44    0   Le5
...........................................................uaucGcagccagcuuugauga..................................   1     1   Le5
...........................................................uaucacaAccagcuuugauga..................................   1     1   Le5
...........................................................uaucacagccagcuuugaugU..................................   1     1   Le5
...........................................................uaucacagccagcuuugaugUg.................................   1     1   Le5
...........................................................uaucacagccagcuuugaugag.................................   46    0   Le5
...........................................................uaucacagUcagcuuugaugag.................................   2     1   Le5
...........................................................uaucacagccagcuuugaugaA.................................   2     1   Le5
...........................................................uaucacagccagcuuugauUagc................................   1     1   Le5
...........................................................uCucacagccagcuuugaugagc................................   4     1   Le5
...........................................................uaucacagAcagcuuugaugagc................................   1     1   Le5
...........................................................uaucacagccagcCuugaugagc................................   1     1   Le5
...........................................................uaCcacagccagcuuugaugagc................................   3     1   Le5
```

Star **Mature**

auauuaaaucccacgucuucggucgucagaguaguugugauauguuuuauuuuaagucauaucacagccagcuuugaugagcagagaugcagguuucaagcacauuuucuuaau			
....uaucacagccagcuuugaugagc................................	507	0	Le5
....uaucUcagccagcuuugaugagc................................	2	1	Le5
....uaucacagccagcuuugaugCgc................................	1	1	Le5
....uaucGcagccagcuuugaugagc................................	6	1	Le5
....uaucacagccCgcuuugaugagc................................	2	1	Le5
....uaucacagccagcuuugaugagA................................	5	1	Le5
....uaucacagccagcuuGgaugagc................................	1	1	Le5
....Aaucacagccagcuuugaugagc................................	2	1	Le5
....uaucacagUcagcuuugaugagc................................	2	1	Le5
....uaucaUagccagcuuugaugagc................................	1	1	Le5
....uauUacagccagcuuugaugagc................................	3	1	Le5
....uaucacagccaCcuuugaugagca...............................	14	1	Le5
....uCucacagccagcuuugaugagca...............................	1	1	Le5
....uaucacagccagcuuugaugagcU...............................	1	1	Le5
....uaucacagccaUcuuugaugagca...............................	3	1	Le5
....uaucacagccagcuuugaugagca...............................	61	0	Le5
....uaucacagccagcuuugaugagcC...............................	1	1	Le5
....uaucacagccagcuuugaugagGa...............................	6	1	Le5
....uaucacagccaAcuuugaugagca...............................	1	1	Le5
....uaucacagccagcuuugaugagcaA..............................	3	1	Le5
....aucacagccagcuuugaugagca...............................	1	0	Le5
....ucacagccagcuuugaugagc................................	3	0	Le5
....ucacagccagcuuugaugagca...............................	2	0	Le5
....ucacagccagcuuugaugagcaC..............................	1	1	Le5
.....................gucgucagaguaguugugauaug..	2	0	Ta1
....uaucacagccagcuuuga.....................................	1	0	Ta1
....uaucUcagccagcuuugau....................................	2	1	Ta1
....uaucacagccagcuuugau....................................	8	0	Ta1
....uaucacagccagcuuugaug...................................	6	0	Ta1
....uauUacagccagcuuugauga..................................	6	1	Ta1
....uaucacagccagcuuugauga..................................	7	0	Ta1
....uaucacagccCgcuuugaugag.................................	1	1	Ta1
....uaucacagccagcuuugaugag.................................	16	0	Ta1
....uaucUcagccagcuuugaugagc................................	1	1	Ta1
....uaucacagcAagcuuugaugagc................................	1	1	Ta1
....Caucacagccagcuuugaugagc................................	1	1	Ta1
....uaucacagccagcuuugaugagA................................	5	1	Ta1
....uCucacagccagcuuugaugagc................................	1	1	Ta1
....uaucacaUccagcuuugaugagc................................	1	1	Ta1
....uauUacagccagcuuugaugagc................................	1	1	Ta1
....uaucacagccagAuuugaugagc................................	1	1	Ta1
....uaucacagccagcuuugaugagc................................	106	0	Ta1
....uaucGcagccagcuuugaugagc................................	4	1	Ta1
....Aaucacagccagcuuugaugagc................................	1	1	Ta1
....uaucacagccagcuuugaugagca...............................	14	0	Ta1
....ucacagccagcuuugaugagc................................	4	0	Ta1
.....................gucgucagaguaguuguU...	1	1	Ch1
.....................gucgucagaguaguugugaua..	1	0	Ch1
.....................gucgucagaguaguugugauaug..	2	0	Ch1
......................ucgucagaguaguugugaua..	3	0	Ch1
....cauaucacagccagcuuuga.....................................	2	0	Ch1
....cauaucacagccagcuuugau....................................	1	0	Ch1
....uaucUcagccagcuuuga.....................................	1	1	Ch1
....uaucacagccagcuuugU.....................................	1	1	Ch1
....uaucacagccagcuuuga.....................................	26	0	Ch1
....uaucacagccCgcuuugau....................................	1	1	Ch1
....uaucacagccagcuuugaC....................................	1	1	Ch1
....uaucacagcAagcuuugau....................................	1	1	Ch1
....uaucGcagccagcuuugau....................................	1	1	Ch1
....uaucacagccagcuuugau....................................	30	0	Ch1
....uaucacagcAagcuuugaug...................................	1	1	Ch1
....uaucacagccagcuuugauU...................................	2	1	Ch1
....uaCcacagccagcuuugaug...................................	1	1	Ch1
....uaucacagccagcuuugaug...................................	23	0	Ch1
....uaucacagccagcuuugauA...................................	1	1	Ch1
....uaucacagccagcuuugauga..................................	32	0	Ch1
....uaucacagcAagcuuugauga..................................	1	1	Ch1
....uaucacagccagcuuugaugaA.................................	1	1	Ch1

```
                    Star                                    Mature
auauuaaucccacgucuucggucgucagaguaguugugauauguuuuauuuuaagucauaucacagccagcuuugaugagcagagaugcagguuucaagcacauuuucuuaau
..........................................................uaucGcagccagcuuugaugag.................................    1      1      Ch1
..........................................................uaucacagccagcuuugaugag.................................    45     0      Ch1
..........................................................uaucacagccagcuuugaugaC.................................    1      1      Ch1
..........................................................uaucacagccagcuuugaugUg.................................    1      1      Ch1
..........................................................uaucacagccagcuuugaugCgc................................    2      1      Ch1
..........................................................uaucacagccCgcuuugaugagc................................    4      1      Ch1
..........................................................uaucUcagccagcuuugaugagc................................    7      1      Ch1
..........................................................uaucacagccagcuuugaugagc................................    534    0      Ch1
..........................................................uaucGcagccagcuuugaugagc................................    4      1      Ch1
..........................................................uaucacagccagcuuugaugagA................................    9      1      Ch1
..........................................................uaucacagccagcuuuUaugagc................................    1      1      Ch1
..........................................................uaucacagccagcuuugaugaCc................................    1      1      Ch1
..........................................................uaucacagccagcuGugaugagc................................    1      1      Ch1
..........................................................uaucacagccagcuuugUugagc................................    1      1      Ch1
..........................................................uaucacagccagcuuugaugaUc................................    1      1      Ch1
..........................................................uaucacagUcagcuuugaugagc................................    3      1      Ch1
..........................................................uaCcacagccagcuuugaugagc................................    3      1      Ch1
..........................................................uCucacagccagcuuugaugagc................................    2      1      Ch1
..........................................................uaucacagccagcuuugaCgagc................................    1      1      Ch1
..........................................................Caucacagccagcuuugaugagc................................    1      1      Ch1
..........................................................uaucacagccagcuuugaugaAc................................    1      1      Ch1
..........................................................uCucacagccagcuuugaugagca...............................    1      1      Ch1
..........................................................Naucacagccagcuuugaugagca...............................    1      1      Ch1
..........................................................uaucacagccCgcuuugaugagca...............................    1      1      Ch1
..........................................................uaucacagccagcuuugaugagAa...............................    1      1      Ch1
..........................................................uaucacagccaAcuuugaugagca...............................    7      1      Ch1
..........................................................uaucacaAccagcuuugaugagca...............................    1      1      Ch1
..........................................................uaucacagccaCcuuugaugagca...............................    7      1      Ch1
..........................................................uaucacagccagcuuugaugagcU...............................    4      1      Ch1
..........................................................uaucGcagccagcuuugaugagca...............................    1      1      Ch1
..........................................................uaucacagccagcuuugaugagGa...............................    3      1      Ch1
..........................................................uaucacagccagcuuugaugagca...............................    71     0      Ch1
..........................................................uaucacagccagcuuugaugagcaA..............................    1      1      Ch1
...........................................................aucacagccagcuuugaugag.................................    1      0      Ch1
...........................................................aucacagccagcuuugaugagc................................    1      0      Ch1
............................................................ucacagccagcuuugaugagc................................    10     0      Ch1
............................................................ucacagccagcuuugaugagca...............................    3      0      Ch1

....................gucgucagaguaguugugaua........................................................................    1      0      Bl1
....................gucgucagaguaguugugauau.......................................................................    1      0      Bl1
....................gucgucagaguaguugugauaug......................................................................    7      0      Bl1
........................................................cauaucacagccagcuuugauga..................................    1      0      Bl1
..........................................................uaucacagccagcuuuga.....................................    14     0      Bl1
..........................................................uaucacagccCgcuuugau....................................    1      1      Bl1
..........................................................uaucacagccagcuuCgau....................................    1      1      Bl1
..........................................................uaucacagccagcuuugau....................................    35     0      Bl1
..........................................................uaucacagccagcuuugaA....................................    9      1      Bl1
..........................................................uaucacagccagcuuugauA...................................    3      1      Bl1
..........................................................uaucacagccCgcuuugaug...................................    1      1      Bl1
..........................................................uaucacagccagcuuugaug...................................    17     0      Bl1
..........................................................uaucacagccagcuuugauU...................................    1      1      Bl1
..........................................................uaucUcagccagcuuugauga..................................    1      1      Bl1
..........................................................uaCcacagccagcuuugauga..................................    1      1      Bl1
..........................................................uaucacagccagcuuugUuga..................................    1      1      Bl1
..........................................................uaucacagccCgcuuugauga..................................    1      1      Bl1
..........................................................uaucGcagccagcuuugauga..................................    1      1      Bl1
..........................................................uaucacagccagcuuuAauga..................................    1      1      Bl1
..........................................................uaucacagccagcuuugauga..................................    78     0      Bl1
..........................................................uaucGcagccagcuuugaugag.................................    1      1      Bl1
..........................................................uaucacagccagcuuugaugag.................................    34     0      Bl1
..........................................................uaucUcagccagcuuugaugag.................................    1      1      Bl1
..........................................................uaucacagccagcuuugaugaA.................................    4      1      Bl1
..........................................................uaucacagccCgcuuugaugagc................................    3      1      Bl1
..........................................................Caucacagccagcuuugaugagc................................    1      1      Bl1
..........................................................uaucacaAccagcuuugaugagc................................    1      1      Bl1
..........................................................uaucacagUcagcuuugaugagc................................    1      1      Bl1
..........................................................uaucacagccagcuuugaugaAc................................    1      1      Bl1
..........................................................uaucacagccagcuGugaugagc................................    1      1      Bl1
..........................................................uaucacagccagcuuugaugagc................................    150    0      Bl1
..........................................................uaucGcagccagcuuugaugagc................................    2      1      Bl1
```

```
                    Star                                   Mature
auauuaaucccacgucuucggucgucagaguaguugugauauguuuuauuuuaagucauaucacagccagcuuugaugagcagagaugcagguuucaagcacauuuucuuaau
..........................................................uaucacagcAagcuuugaugagc................................   1     1     Bl1
..........................................................uaucacagccagcuuugaugagA................................   16    1     Bl1
..........................................................uaucacagccaUcuuugaugagca...............................   1     1     Bl1
..........................................................uaucacagccaCcuuugaugagca...............................   13    1     Bl1
..........................................................uaucUcagccagcuuugaugagca...............................   1     1     Bl1
..........................................................uaucacagccagcuuugaugagGa...............................   3     1     Bl1
..........................................................uaucacagccaAcuuugaugagca...............................   12    1     Bl1
..........................................................uaucacagccagcuuugaugagca...............................   74    0     Bl1
..........................................................uaucacagccagcuuugaugagcU...............................   1     1     Bl1
..........................................................uaucacagccagcuuugaugagcaA..............................   1     1     Bl1
..........................................................uaucacagccagcuuugaugagcaC..............................   1     1     Bl1
...........................................................aucacagccagcuuugaugagc................................   1     0     Bl1
............................................................ucacagccagcuuugauga..................................   1     0     Bl1

....................gucgucagaguaguugug...........................................................................   1     0     He1
....................gucgucagaguaguugugaua........................................................................   10    0     He1
....................gucgGcagaguaguugugauau.......................................................................   1     1     He1
....................gucgCcagaguaguugugauau.......................................................................   1     1     He1
....................gucgucagaguaguugugauaug......................................................................   63    0     He1
....................gucgucagCguaguugugauaug......................................................................   3     1     He1
....................Nucgucagaguaguugugauaug......................................................................   1     1     He1
....................gucgAcagaguaguugugauaug......................................................................   1     1     He1
....................guAgucagaguaguugugauaug......................................................................   1     1     He1
....................Uucgucagaguaguugugauaug......................................................................   1     1     He1
....................gucgucagaguaguugugauCug......................................................................   1     1     He1
....................Aucgucagaguaguugugauaug......................................................................   1     1     He1
....................gucgGcagaguaguugugauaug......................................................................   3     1     He1
....................gucgucagaguaguugugauaugu.....................................................................   7     0     He1
.....................ucgucagaguaguugugau.........................................................................   1     0     He1
.......................................................ucauaucacagccagcuuugau....................................   1     0     He1
........................................................cauaucacagccagcuuuga.....................................   1     0     He1
........................................................cauaucacagccagcuuugau....................................   7     0     He1
........................................................cauaucacagccagcuuugaugag.................................   3     0     He1
.........................................................auaucacagccaCcuuugaugagca...............................   1     1     He1
..........................................................uaucUcagccagcuuuga.....................................   1     1     He1
..........................................................uaucacagccagcuuugU.....................................   2     1     He1
..........................................................uaucacagccagcuuuga.....................................   71    0     He1
..........................................................uaucacagAcagcuuugau....................................   1     1     He1
..........................................................uaucacagccagcuuugau....................................   249   0     He1
..........................................................uaucacagccagcuuugaA....................................   15    1     He1
..........................................................uaucacagccCgcuuugau....................................   2     1     He1
..........................................................uauUacagccagcuuugau....................................   2     1     He1
..........................................................uaucGcagccagcuuugau....................................   1     1     He1
..........................................................uCucacagccagcuuugau....................................   1     1     He1
..........................................................uaucacagccagAuuugau....................................   1     1     He1
..........................................................Caucacagccagcuuugau....................................   1     1     He1
..........................................................uaucUcagccagcuuugau....................................   1     1     He1
..........................................................uaucacagccagcuuugaC....................................   4     1     He1
..........................................................uaucacaUccagcuuugau....................................   1     1     He1
..........................................................uaucacagccagcuuugaug...................................   110   0     He1
..........................................................uaucacaAccagcuuugaug...................................   2     1     He1
..........................................................uaucUcagccagcuuugaug...................................   1     1     He1
..........................................................uaucacagccagcuuugUug...................................   1     1     He1
..........................................................uaucacagcAagcuuugaug...................................   1     1     He1
..........................................................uaucacagccagcuuugauA...................................   6     1     He1
..........................................................uaucacagccagcuuugaCg...................................   2     1     He1
..........................................................uaucacagAcagcuuugaug...................................   1     1     He1
..........................................................uaucacaUccagcuuugaug...................................   1     1     He1
..........................................................uaucacagccagcuuugauU...................................   2     1     He1
..........................................................uaucacagccCgcuuugaug...................................   1     1     He1
..........................................................uaucacagccagcuuugUuga..................................   1     1     He1
..........................................................uaucacagccagcuuugaGga..................................   1     1     He1
..........................................................uCucacagccagcuuugauga..................................   1     1     He1
..........................................................Caucacagccagcuuugauga..................................   1     1     He1
..........................................................uaucacagccagcuuugauga..................................   225   0     He1
..........................................................uaCcacagccagcuuugauga..................................   1     1     He1
..........................................................uaucGcagccagcuuugauga..................................   1     1     He1
..........................................................uaucacaUccagcuuugauga..................................   1     1     He1
..........................................................uaucUcagccagcuuugauga..................................   3     1     He1
..........................................................uaucacagccagcuuugGuga..................................   1     1     He1
```

Star **Mature**

```
auauuaaucccacgucuucggucgucagaguaguugugauauguuuuauuuuaagucauaucacagccagcuuugaugagcagagaugcagguuucaagcacauuuucuuaau
..........................................................uaucacagccagcuuugaugC..................................  1     1  He1
..........................................................uaucacagccCgcuuugauga..................................  2     1  He1
..........................................................uaucacagccagcuuugaugU..................................  2     1  He1
..........................................................uaucacagccagcuuugUugag.................................  2     1  He1
..........................................................uaucacagccagcuuGgaugag.................................  1     1  He1
..........................................................uaucacagccCgcuuugaugag.................................  3     1  He1
..........................................................uaucacagccagcuGugaugag.................................  1     1  He1
..........................................................Naucacagccagcuuugaugag.................................  1     1  He1
..........................................................uaucacagccagcuuugaugag.................................  260   0  He1
..........................................................Caucacagccagcuuugaugag.................................  1     1  He1
..........................................................uaucacagccagcuuugaugGg.................................  1     1  He1
..........................................................uaucacagccagcuuugaugaC.................................  3     1  He1
..........................................................uaucacagAcagcuuugaugag.................................  1     1  He1
..........................................................uaucacagccagcuuugauUag.................................  1     1  He1
..........................................................uaucacagccagcuuugaugaA.................................  3     1  He1
..........................................................uaucUcagccagcuuugaugag.................................  1     1  He1
..........................................................uauUacagccagcuuugaugag.................................  1     1  He1
..........................................................uaucGcagccagcuuugaugag.................................  2     1  He1
..........................................................uaucacagccagcuuugaugaU.................................  1     1  He1
..........................................................uaucacagccUgcuuugaugag.................................  1     1  He1
..........................................................uaCcacagccagcuuugaugagc................................  4     1  He1
..........................................................uaucaAagccagcuuugaugagc................................  1     1  He1
..........................................................uaucacagccagcuuugaGgagc................................  1     1  He1
..........................................................uaucacaAccagcuuugaugagc................................  3     1  He1
..........................................................uaucacagUcagcuuugaugagc................................  4     1  He1
..........................................................uaucacagccagcuuugaugaUc................................  2     1  He1
..........................................................uaucGcagccagcuuugaugagc................................  10    1  He1
..........................................................uaucacagccagcuuugaugaCc................................  10    1  He1
..........................................................uaucacagccagcAuugaugagc................................  1     1  He1
..........................................................uaucacagcUagcuuugaugagc................................  3     1  He1
..........................................................uaucacagccagcuuugUugagc................................  1     1  He1
..........................................................Naucacagccagcuuugaugagc................................  1     1  He1
..........................................................uaucaUagccagcuuugaugagc................................  2     1  He1
..........................................................uaucUcagccagcuuugaugagc................................  22    1  He1
..........................................................uCucacagccagcuuugaugagc................................  4     1  He1
..........................................................uauUacagccagcuuugaugagc................................  4     1  He1
..........................................................uaucacagccagcuGugaugagc................................  1     1  He1
..........................................................uaucacagccagcuuugaugaAc................................  6     1  He1
..........................................................uaucacagccagcuuugaugagc................................  2015  0  He1
..........................................................uaucacagccagcuuugGugagc................................  1     1  He1
..........................................................Aaucacagccagcuuugaugagc................................  4     1  He1
..........................................................uaucacagcAagcuuugaugagc................................  2     1  He1
..........................................................uaucacagccagUuuugaugagc................................  3     1  He1
..........................................................uaucacagccagcuuugaugUgc................................  2     1  He1
..........................................................uaucacagccagcuuCgaugagc................................  1     1  He1
..........................................................uaucacagccagcuuugaugagA................................  42    1  He1
..........................................................uaucacagccagcuuugaugCgc................................  4     1  He1
..........................................................uGucacagccagcuuugaugagc................................  1     1  He1
..........................................................uaucacagccagcuuugauUagc................................  1     1  He1
..........................................................uaucacagccagcuuugauAagc................................  3     1  He1
..........................................................uaucacagccagcuCugaugagc................................  1     1  He1
..........................................................uaucacaUccagcuuugaugagc................................  2     1  He1
..........................................................uaucacagccCgcuuugaugagc................................  15    1  He1
..........................................................uaucacagccGgcuuugaugagc................................  1     1  He1
..........................................................Caucacagccagcuuugaugagc................................  2     1  He1
..........................................................uaucacagAcagcuuugaugagc................................  6     1  He1
..........................................................uaucacagccaCcuuugaugagca...............................  63    1  He1
..........................................................uaucacagccaAcuuugaugagca...............................  21    1  He1
..........................................................uaucacagccagcuuugaugagca...............................  281   0  He1
..........................................................uaucacagcUagcuuugaugagca...............................  1     1  He1
..........................................................uaucacagccaUcuuugaugagca...............................  34    1  He1
..........................................................uaucacagccagcuuugaugagcU...............................  18    1  He1
..........................................................uaucacagccCgcuuugaugagca...............................  2     1  He1
..........................................................uaucacaUccagcuuugaugagca...............................  1     1  He1
..........................................................uaucUcagccagcuuugaugagca...............................  8     1  He1
..........................................................uaucacagccagcuuugaugagcC...............................  19    1  He1
..........................................................uaucacagccagcuuugaugagGa...............................  27    1  He1
..........................................................uaucacagAcagcuuugaugagca...............................  1     1  He1
..........................................................uaucGcagccagcuuugaugagca...............................  5     1  He1
..........................................................uaucacagccagcuuugaugagcaU..............................  1     1  He1
```

Star **Mature**

```
auauuaaucccacgucuucggucgucagaguaguugugauauguuuuauuuuaagucauaucacagccagcuuugaugagcagagaugcagguuucaagcacauuuucuuaau
..........................................................uaucacagccagcuuugaugagcaA..............................   3   1   He1
..........................................................uaucacagccagcuuugaugagcaC..............................   1   1   He1
..........................................................uaucacagccagcuuugaugagcaga.............................   1   0   He1
...........................................................aucacagccagcuuugaugag.................................   1   0   He1
...........................................................aucacagccagcuuugaugagc................................   1   0   He1
...........................................................aucacagccaAcuuugaugagca...............................   1   1   He1
............................................................ucacagccagcuuugaugagc................................   7   0   He1
............................................................ucacagccCgcuuugaugagc................................   1   1   He1
............................................................ucacagccagcuuugaugaCc................................   1   1   He1
............................................................ucacagccagcuuugaugagca...............................   2   0   He1
.............................................................cacagccagcuuugaugagca...............................   1   0   He1
..............................................................acagccagcuuugaugag.................................   3   0   He1
..............................................................acagccagcuuugaugagA................................   3   1   He1
..............................................................acagccagcuuugaugagc................................   3   0   He1
...............................................................cagccagcuuugaugagc................................   1   0   He1
```

Provisional ID : Sc28yqQ_4222_2914
Score total : 9630.4
Score for star read(s) : 3.9
Score for read counts : 9620
Score for mfe : 1.9
Score for randfold : 1.6
Score for cons. seed : 3
Total read count : 18881
Mature read count : 18555
Loop read count : 0
Star read count : 326

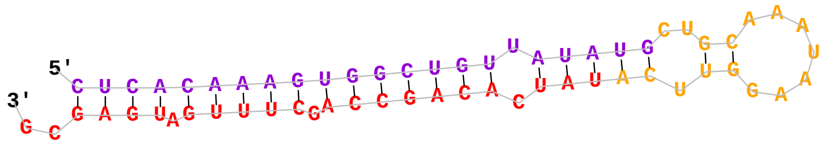

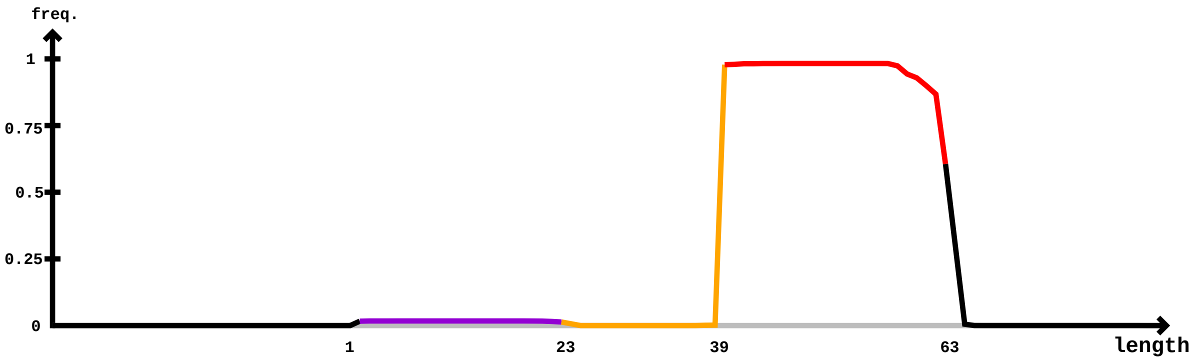

Star Mature

```
5'- cacaagagauguaaagucaguuccccaucacucacaaaguggcuguuauaugcugcaaauaagguucauaucacagccagcuuugaugagcgaagaggauacuguuggcaucaa  -3'  obs
    cacaagagauguaaagucaguuccccaucacucacaaaguggcuguuauaugcugcaaauaagguucauaucacagccagcuuugaugagcgaagaggauacuguuggcaucaa       exp
    .......(((((.....(((((((.(.((.((((((((((((((((.(((((..((.......)).))))).))))))).))))).)))).)).).))).))))...)))))..  reads  mm  sample
    ..............................cucacaaaguggcuguuauau...............................................................  7      0   Ta1
    ..............................cucacaaaguggcuguuauaug..............................................................  25     0   Ta1
    ..............................cuAacaaaguggcuguuauaug..............................................................  1      1   Ta1
    ..............................cucacaaaguggcuUuuauaug..............................................................  1      1   Ta1
    ....................................................................uaucacagccagcuuuga............................  1      0   Ta1
    ....................................................................uaucUcagccagcuuugau...........................  2      1   Ta1
    ....................................................................uaucacagccagcuuugau...........................  8      0   Ta1
    ....................................................................uaucacagccagcuuugaug..........................  6      0   Ta1
    ....................................................................uaucacagccagcuuugauga.........................  7      0   Ta1
    ....................................................................uauUacagccagcuuugauga.........................  6      1   Ta1
    ....................................................................uaucacagccagcuuugaugag........................  16     0   Ta1
    ....................................................................uaucacagccCgcuuugaugag........................  1      1   Ta1
    ....................................................................uaucacagcAagcuuugaugagc.......................  1      1   Ta1
    ....................................................................uaucacagccagAuuugaugagc.......................  1      1   Ta1
    ....................................................................Aaucacagccagcuuugaugagc.......................  1      1   Ta1
    ....................................................................uaucGcagccagcuuugaugagc.......................  4      1   Ta1
    ....................................................................uCucacagccagcuuugaugagc.......................  1      1   Ta1
    ....................................................................uauUacagccagcuuugaugagc.......................  1      1   Ta1
    ....................................................................uaucacagccagcuuugaugagc.......................  106    0   Ta1
    ....................................................................Caucacagccagcuuugaugagc.......................  1      1   Ta1
    ....................................................................uaucacaUccagcuuugaugagc.......................  1      1   Ta1
    ....................................................................uaucUcagccagcuuugaugagc.......................  1      1   Ta1
    ....................................................................uaucacagccagcuuugaugagA.......................  5      1   Ta1
    ....................................................................uaucacagccagcuuugaugagcg......................  238    0   Ta1
    ....................................................................Aaucacagccagcuuugaugagcg......................  1      1   Ta1
    ....................................................................uaucacagccCgcuuugaugagcg......................  3      1   Ta1
    ....................................................................uauUacagccagcuuugaugagcg......................  1      1   Ta1
    ....................................................................uaucacaAccagcuuugaugagcg......................  1      1   Ta1
    ....................................................................uaucGcagccagcuuugaugagcg......................  4      1   Ta1
    ....................................................................uaucacagccagcuuGgaugagcg......................  1      1   Ta1
    ....................................................................uCucacagccagcuuugaugagcg......................  1      1   Ta1
    ....................................................................uaucacagccagcuuugaugaUcg......................  1      1   Ta1
    ....................................................................uaucacagccagcuuugaugagGga.....................  11     1   Ta1
```

Star **Mature**

```
cacaagagauguaaagucaguuccccaucacucacaaaguggcuguuauaugcugcaaauaagguucauaucacagccagcuuugaugagcgaagaggauacuguuggcaucaa
....................................................................uaucacagccagcuuugaugagcgU.....................	120	1	Ta1
....................................................................uaucacagccagcuuugaugagGgaa....................	7	1	Ta1
......................................................................ucacagccagcuuugaugagc.......................	4	0	Ta1
............................................................................................aagaggauacuguuggcauc..	1	0	Ta1

..............................cucacaaaguggcuguua..................................................................	1	0	He1
..............................cucacaaaguggcuguuau.................................................................	2	0	He1
..............................cucacaaaguggcuguuaua................................................................	8	0	He1
..............................cucacaaaAuggcuguuaua................................................................	1	1	He1
..............................cucGcaaaguggcuguuaua................................................................	1	1	He1
..............................cucacaaaguggcuguuauau...............................................................	1	0	He1
..............................cuUacaaaguggcuguuauaug..............................................................	1	1	He1
..............................Aucacaaaguggcuguuauaug..............................................................	1	1	He1
..............................cuAacaaaguggcuguuauaug..............................................................	1	1	He1
..............................cucUcaaaguggcuguuauaug..............................................................	1	1	He1
..............................cucacaaaguggcuguuauaug..............................................................	50	0	He1
..............................cucacaaaguggcuUuuauaug..............................................................	1	1	He1
..............................cucacaaaguggcuguuauaugc.............................................................	1	0	He1
...............................ucacaaaguggcuguuauaug..............................................................	3	0	He1
...............................ucacaaaguggcuguuauaugA.............................................................	1	1	He1
.................................................................ucauaucacagccagcuuugau...........................	1	0	He1
..................................................................cauaucacagccagcuuuga............................	1	0	He1
..................................................................cauaucacagccagcuuugau...........................	7	0	He1
..................................................................cauaucacagccagcuuugaugag........................	3	0	He1
...................................................................Cuaucacagccagcuuugaugagcg......................	3	1	He1
...................................................................auauUacagccagcuuugaugagcg......................	1	1	He1
....................................................................uaucUcagccagcuuuga............................	1	1	He1
....................................................................uaucacagccagcuuugU............................	2	1	He1
....................................................................uaucacagccagcuuuga............................	71	0	He1
....................................................................uaucGcagccagcuuugau...........................	1	1	He1
....................................................................uCucacagccagcuuugau...........................	1	1	He1
....................................................................uaucacagccagcuuugaC...........................	4	1	He1
....................................................................uauUacagccagcuuugau...........................	2	1	He1
....................................................................uaucacagccagAuuugau...........................	1	1	He1
....................................................................uaucUcagccagcuuugau...........................	1	1	He1
....................................................................uaucacagccCgcuuugau...........................	2	1	He1
....................................................................uaucacagAcagcuuugau...........................	1	1	He1
....................................................................Caucacagccagcuuugau...........................	1	1	He1
....................................................................uaucacagccagcuuugau...........................	249	0	He1
....................................................................uaucacagccagcuuugaA...........................	15	1	He1
....................................................................uaucacaUccagcuuugau...........................	1	1	He1
....................................................................uaucacagAcagcuuugaug..........................	1	1	He1
....................................................................uaucacagccagcuuugaug..........................	110	0	He1
....................................................................uaucacagccCgcuuugaug..........................	1	1	He1
....................................................................uaucacagcAagcuuugaug..........................	1	1	He1
....................................................................uaucacagccagcuuugUug..........................	1	1	He1
....................................................................uaucacaAccagcuuugaug..........................	2	1	He1
....................................................................uaucacagccagcuuugauA..........................	6	1	He1
....................................................................uaucacagccagcuuugaCg..........................	2	1	He1
....................................................................uaucUcagccagcuuugaug..........................	1	1	He1
....................................................................uaucacaUccagcuuugaug..........................	1	1	He1
....................................................................uaucacagccagcuuugauU..........................	2	1	He1
....................................................................uCucacagccagcuuugauga.........................	1	1	He1
....................................................................uaucacaUccagcuuugauga.........................	1	1	He1
....................................................................uaucacagccagcuuugauga.........................	225	0	He1
....................................................................uaucacagccCgcuuugauga.........................	2	1	He1
....................................................................uaCcacagccagcuuugauga.........................	1	1	He1
....................................................................uaucacagccagcuuugaugU.........................	2	1	He1
....................................................................uaucGcagccagcuuugauga.........................	1	1	He1
....................................................................uaucacagccagcuuugaGga.........................	1	1	He1
....................................................................uaucUcagccagcuuugauga.........................	3	1	He1
....................................................................uaucacagccagcuuugaugC.........................	1	1	He1
....................................................................Caucacagccagcuuugauga.........................	1	1	He1
....................................................................uaucacagccagcuuugUuga.........................	1	1	He1
....................................................................uaucacagccagcuuugGuga.........................	1	1	He1
....................................................................uaucGcagccagcuuugaugag........................	2	1	He1
....................................................................uauUacagccagcuuugaugag........................	1	1	He1
....................................................................uaucacagAcagcuuugaugag........................	1	1	He1
....................................................................uaucacagccagcuuugaugaC........................	3	1	He1
....................................................................uaucacagccUgcuuugaugag........................	1	1	He1
```

Star Mature

```
cacaagagauguaaagucaguuccccaucacucacaaagguggcuguuauaugcugcaaauaagguucauaucacagccagcuuugaugagcgaagaggauacuguuggcaucaa
.....................................................................uaucacagccCgcuuugaugag........................ 3 1 He1
.....................................................................uaucacagccagcuGugaugag........................ 1 1 He1
.....................................................................uaucacagccagcuuugaugGg........................ 1 1 He1
.....................................................................uaucacagccagcuuugauUag........................ 1 1 He1
.....................................................................uaucUcagccagcuuugaugag........................ 1 1 He1
.....................................................................uaucacagccagcuuugaugaA........................ 3 1 He1
.....................................................................uaucacagccagcuuugaugaU........................ 1 1 He1
.....................................................................uaucacagccagcuuGgaugag........................ 1 1 He1
.....................................................................Caucacagccagcuuugaugag........................ 1 1 He1
.....................................................................Naucacagccagcuuugaugag........................ 1 1 He1
.....................................................................uaucacagccagcuuugUugag........................ 2 1 He1
.....................................................................uaucacagccagcuuugaugag........................ 260 0 He1
.....................................................................uaucaUagccagcuuugaugagc....................... 2 1 He1
.....................................................................uaucacagccagcAuugaugagc....................... 1 1 He1
.....................................................................uaucacagccGgcuuugaugagc....................... 1 1 He1
.....................................................................uaucacagccagUuuugaugagc....................... 3 1 He1
.....................................................................uaucacagAcagcuuugaugagc....................... 6 1 He1
.....................................................................uaucacagccagcuuugauUagc....................... 1 1 He1
.....................................................................uaucacagccagcuuugUugagc....................... 1 1 He1
.....................................................................uaucacaUccagcuuugaugagc....................... 2 1 He1
.....................................................................uaucacagccCgcuuugaugagc....................... 15 1 He1
.....................................................................uaucacagccagcuuugaugUgc....................... 2 1 He1
.....................................................................uaucGcagccagcuuugaugagc....................... 10 1 He1
.....................................................................uaucacagccagcuuugaugagA....................... 42 1 He1
.....................................................................uauUacagccagcuuugaugagc....................... 4 1 He1
.....................................................................uaucacagcAagcuuugaugagc....................... 2 1 He1
.....................................................................uGucacagccagcuuugaugagc....................... 1 1 He1
.....................................................................uaucacagccagcuuugaugaCc....................... 10 1 He1
.....................................................................uaucacagUcagcuuugaugagc....................... 4 1 He1
.....................................................................uaucUcagccagcuuugaugagc....................... 22 1 He1
.....................................................................Caucacagccagcuuugaugagc....................... 2 1 He1
.....................................................................uaucacagccagcuuugaugCgc....................... 4 1 He1
.....................................................................uaucacagccagcuCugaugagc....................... 1 1 He1
.....................................................................uaucacagccagcuuugauAagc....................... 3 1 He1
.....................................................................uaucacagccagcuuugaugaUc....................... 2 1 He1
.....................................................................uCucacagccagcuuugaugagc....................... 4 1 He1
.....................................................................uaCcacagccagcuuugaugagc....................... 4 1 He1
.....................................................................uaucacagccagcuuCgaugagc....................... 1 1 He1
.....................................................................Aaucacagccagcuuugaugagc....................... 4 1 He1
.....................................................................uaucacagccagcuuugaGgagc....................... 1 1 He1
.....................................................................uaucacagcUagcuuugaugagc....................... 3 1 He1
.....................................................................Naucacagccagcuuugaugagc....................... 1 1 He1
.....................................................................uaucaAagccagcuuugaugagc....................... 1 1 He1
.....................................................................uaucacaAccagcuuugaugagc....................... 3 1 He1
.....................................................................uaucacagccagcuuugGugagc....................... 1 1 He1
.....................................................................uaucacagccagcuuugaugaAc....................... 6 1 He1
.....................................................................uaucacagccagcuuugaugagc....................... 2015 0 He1
.....................................................................uaucacagccagcuGugaugagc....................... 1 1 He1
.....................................................................uaucacagccagcuuugUugagcg...................... 3 1 He1
.....................................................................uaucGcagccagcuuugaugagcg...................... 17 1 He1
.....................................................................uaucacagccagcuuugaugagcg...................... 2569 0 He1
.....................................................................uaucacagccagcuuugaugagAg...................... 2 1 He1
.....................................................................uaucUcagccagcuuugaugagcg...................... 25 1 He1
.....................................................................uaucacagccagcuuugaugCgcg...................... 2 1 He1
.....................................................................uaucacagccagcuuugaugagcC...................... 19 1 He1
.....................................................................uaucacagccagAuuugaugagcg...................... 2 1 He1
.....................................................................uauUacagccagcuuugaugagcg...................... 9 1 He1
.....................................................................uaucaUagccagcuuugaugagcg...................... 1 1 He1
.....................................................................uaucacaAccagcuuugaugagcg...................... 2 1 He1
.....................................................................uaucacagccagcuuugaAgagcg...................... 1 1 He1
.....................................................................uaucacagccagUuuugaugagcg...................... 1 1 He1
.....................................................................uGucacagccagcuuugaugagcg...................... 2 1 He1
.....................................................................uaucacagccagcuuugaugaAcg...................... 3 1 He1
.....................................................................uaCcacagccagcuuugaugagcg...................... 6 1 He1
.....................................................................Aaucacagccagcuuugaugagcg...................... 5 1 He1
.....................................................................uaucacagccagcuuCgaugagcg...................... 1 1 He1
.....................................................................uaAcacagccagcuuugaugagcg...................... 1 1 He1
.....................................................................uaucacagUcagcuuugaugagcg...................... 4 1 He1
.....................................................................uaucacagccagcuuugaugGgcg...................... 1 1 He1
.....................................................................uaucacUgccagcuuugaugagcg...................... 1 1 He1
```

```
                              Star                                  Mature
cacaagagauguaaagucaguuccccaucacucacaaaguggcuguuauaugcugcaaauaagguucauaucacagccagcuuugaugagcgaagaggauacuguuggcaucaa
....................................................................uaucacagcAagcuuugaugagcg......................   1      1   He1
....................................................................uaucacagccagcuuugauUagcg......................   6      1   He1
....................................................................uaucacGgccagcuuugaugagcg......................   2      1   He1
....................................................................uCucacagccagcuuugaugagcg......................   8      1   He1
....................................................................uaucacagccagcuuugaugaUcg......................   1      1   He1
....................................................................uaucacagccagcuuugCugagcg......................   1      1   He1
....................................................................uaucacagAcagcuuugaugagcg......................   3      1   He1
....................................................................uaucacagccCgcuuugaugagcg......................   15     1   He1
....................................................................Naucacagccagcuuugaugagcg......................   3      1   He1
....................................................................uaucacaUccagcuuugaugagcg......................   1      1   He1
....................................................................uaucacagccagcuuugaugagcU......................   18     1   He1
....................................................................uaucacagccaCcuuugaugagcg......................   9      1   He1
....................................................................uaucacagccaUcuuugaugagcg......................   6      1   He1
....................................................................uaucacagccagcCuugaugagcg......................   1      1   He1
....................................................................uaucacagccagcuGugaugagcg......................   2      1   He1
....................................................................uaucacagccagcuuugaugaCcg......................   1      1   He1
....................................................................Caucacagccagcuuugaugagcg......................   2      1   He1
....................................................................uaucacagccagcuuugaugagcgU.....................   1326   1   He1
....................................................................uaucacagccagcCuugaugagcga.....................   2      1   He1
....................................................................uaucacagccaAcuuugaugagcga.....................   22     1   He1
....................................................................uauUacagccagcuuugaugagcga.....................   1      1   He1
....................................................................uaucacagccagcuuugaugagGga.....................   124    1   He1
....................................................................uaucacagccagcuuugaugagcgC.....................   247    1   He1
....................................................................uaucacagccagcuuugaugagcAa.....................   3      1   He1
....................................................................uaucacagccagcuuugGugagcga.....................   1      1   He1
....................................................................uaucacagccagcuuugaugagcga.....................   126    0   He1
....................................................................uaucacagccagcuuugaugagcgUa....................   16     1   He1
....................................................................uaucacagccagcuuugaugagGgaa....................   10     1   He1
....................................................................uaucacagccagcuuugaugagcgaU....................   2      1   He1
....................................................................uaucacagccagcuuugaugagcgaaU...................   1      1   He1
.....................................................................aucacagccagcuuugaugag........................   1      0   He1
.....................................................................aucacagccagcuuugaugagc.......................   1      0   He1
.....................................................................aucacagccagcuuugaugagcg......................   2      0   He1
.....................................................................aucacagccagcuuugaugagcgC.....................   1      1   He1
.....................................................................aucacagccaAcuuugaugagcga.....................   1      1   He1
.....................................................................aucacagccagcuuugaugagcgU.....................   1      1   He1
......................................................................ucacagccagcuuugaugaCc.......................   1      1   He1
......................................................................ucacagccCgcuuugaugagc.......................   1      1   He1
......................................................................ucacagccagcuuugaugagc.......................   7      0   He1
......................................................................ucacagccagcuuugaugagcgU.....................   3      1   He1
......................................................................ucacagccagcuuugaugagcgC.....................   1      1   He1
......................................................................ucacagccagcuuugaugagcga.....................   1      0   He1
........................................................................acagccagcuuugaugag........................   3      0   He1
........................................................................acagccagcuuugaugagA.......................   3      1   He1
........................................................................acagccagcuuugaugagc.......................   3      0   He1
.........................................................................cagccagcuuugaugagc.......................   1      0   He1

..............................cucacaaaguggcuguuaua................................................................   3      0   Bl1
..............................cucacaaagugAcuguuaua................................................................   1      1   Bl1
..............................cucacaaaguggcuguuauau...............................................................   4      0   Bl1
..............................cucacaaaguggcuguuauaug..............................................................   11     0   Bl1
..............................cucacaaaguggcuguuauaugA.............................................................   1      1   Bl1
..................................................................cauaucacagccagcuuugauga.........................   1      0   Bl1
...................................................................auaucacagccagcuuugaugagcg......................   1      0   Bl1
....................................................................uaucacagccagcuuuga............................   14     0   Bl1
....................................................................uaucacagccagcuuugaA...........................   9      1   Bl1
....................................................................uaucacagccagcuuCgau...........................   1      1   Bl1
....................................................................uaucacagccCgcuuugau...........................   1      1   Bl1
....................................................................uaucacagccagcuuugau...........................   35     0   Bl1
....................................................................uaucacagccagcuuugauA..........................   3      1   Bl1
....................................................................uaucacagccagcuuugaug..........................   17     0   Bl1
....................................................................uaucacagccCgcuuugaug..........................   1      1   Bl1
....................................................................uaucacagccagcuuugauU..........................   1      1   Bl1
....................................................................uaucacagccagcuuugUuga.........................   1      1   Bl1
....................................................................uaCcacagccagcuuugauga.........................   1      1   Bl1
....................................................................uaucacagccagcuuugauga.........................   78     0   Bl1
....................................................................uaucacagccagcuuuAauga.........................   1      1   Bl1
....................................................................uaucacagccCgcuuugauga.........................   1      1   Bl1
....................................................................uaucUcagccagcuuugauga.........................   1      1   Bl1
....................................................................uaucGcagccagcuuugauga.........................   1      1   Bl1
```

Star   Mature

```
cacaagagauguaaagucaguuccccaucacucacaaaguggcuguuauaugcugcaaauaagguucauaucacagccagcuuugaugagcgaagaggauacuguuggcaucaa
....................................................................uaucacagccagcuuugaugag........................ 34 0 Bl1
....................................................................uaucUcagccagcuuugaugag........................ 1 1 Bl1
....................................................................uaucGcagccagcuuugaugag........................ 1 1 Bl1
....................................................................uaucacagccagcuuugaugaA........................ 4 1 Bl1
....................................................................uaucacagccagcuuugaugagA....................... 16 1 Bl1
....................................................................uaucGcagccagcuuugaugagc....................... 2 1 Bl1
....................................................................uaucacagccagcuGugaugagc....................... 1 1 Bl1
....................................................................uaucacagccagcuuugaugaAc....................... 1 1 Bl1
....................................................................uaucacagccagcuuugaugagc....................... 150 0 Bl1
....................................................................uaucacagcAagcuuugaugagc....................... 1 1 Bl1
....................................................................uaucacagccCgcuuugaugagc....................... 3 1 Bl1
....................................................................uaucacagUcagcuuugaugagc....................... 1 1 Bl1
....................................................................Caucacagccagcuuugaugagc....................... 1 1 Bl1
....................................................................uaucacaAccagcuuugaugagc....................... 1 1 Bl1
....................................................................uaucUcagccagcuuugaugagcg...................... 2 1 Bl1
....................................................................uaucacagccCgcuuugaugagcg...................... 3 1 Bl1
....................................................................uaucacagccagcuuugUugagcg...................... 1 1 Bl1
....................................................................uaucacagcAagcuuugaugagcg...................... 1 1 Bl1
....................................................................uaucacagUcagcuuugaugagcg...................... 2 1 Bl1
....................................................................uaucacaUccagcuuugaugagcg...................... 1 1 Bl1
....................................................................Naucacagccagcuuugaugagcg...................... 1 1 Bl1
....................................................................uaucGcagccagcuuugaugagcg...................... 5 1 Bl1
....................................................................uaucacagccagcuuugaugCgcg...................... 1 1 Bl1
....................................................................uaucacagccagcuuugaugagcU...................... 1 1 Bl1
....................................................................uaucacagccagcuuugaugagcg...................... 450 0 Bl1
....................................................................uauUacagccagcuuugaugagcg...................... 2 1 Bl1
....................................................................uaucacagAcagcuuugaugagcg...................... 3 1 Bl1
....................................................................uaCcacagccagcuuugaugagcg...................... 1 1 Bl1
....................................................................uaucacagcUagcuuugaugagcg...................... 1 1 Bl1
....................................................................uaucacagccagcuuugauUagcg...................... 1 1 Bl1
....................................................................uaucacagccaCcuuugaugagcg...................... 2 1 Bl1
....................................................................uaucacagccagcuuGgaugagcg...................... 1 1 Bl1
....................................................................uaucacagccUgcuuugaugagcg...................... 2 1 Bl1
....................................................................uaucacagccagcuuuAaugagcg...................... 1 1 Bl1
....................................................................uaucacagccagcuuugaugagcga..................... 24 0 Bl1
....................................................................uaucacagccagcuuugaugagcgU..................... 757 1 Bl1
....................................................................uaucacagccagcuuugaugagGga..................... 29 1 Bl1
....................................................................uaucacagccagcuuugaugagcgC..................... 56 1 Bl1
....................................................................uaucacagccagcuuugaugagcAa..................... 1 1 Bl1
....................................................................uaucacagccaAcuuugaugagcga..................... 14 1 Bl1
....................................................................uaucacagccagcuuugaugagcgUa.................... 16 1 Bl1
....................................................................uaucacagccagcuuugaugagGgaa.................... 5 1 Bl1
.....................................................................aucacagccagcuuugaugagc....................... 1 0 Bl1
.....................................................................aucacagccagcuuugaugagcgU..................... 3 1 Bl1
......................................................................ucacagccagcuuugauga......................... 1 0 Bl1
......................................................................ucacagccagcuuugaugagcg...................... 1 0 Bl1

..............................cucacaaaguggcuguua.................................................................. 1 0 Ch1
..............................cucaUaaaguggcuguua.................................................................. 1 1 Ch1
..............................cucacaaaguggcuguuaua................................................................ 3 0 Ch1
..............................cucacaaaguggcuguuauau............................................................... 6 0 Ch1
..............................Aucacaaaguggcuguuauaug.............................................................. 1 1 Ch1
..............................cucacUaaguggcuguuauaug.............................................................. 1 1 Ch1
..............................cucacaaaguggcuguuauaug.............................................................. 52 0 Ch1
..............................cucacaaaguggcuguuauaugA............................................................. 1 1 Ch1
..................................................................cauaucacagccagcuuuga............................ 2 0 Ch1
..................................................................cauaucacagccagcuuugau........................... 1 0 Ch1
....................................................................uaucacagccagcuuugU............................ 1 1 Ch1
....................................................................uaucacagccagcuuuga............................ 26 0 Ch1
....................................................................uaucUcagccagcuuuga............................ 1 1 Ch1
....................................................................uaucacagccagcuuugaC........................... 1 1 Ch1
....................................................................uaucGcagccagcuuugau........................... 1 1 Ch1
....................................................................uaucacagcAagcuuugau........................... 1 1 Ch1
....................................................................uaucacagccagcuuugau........................... 30 0 Ch1
....................................................................uaucacagccCgcuuugau........................... 1 1 Ch1
....................................................................uaucacagccagcuuugauA.......................... 1 1 Ch1
....................................................................uaucacagccagcuuugaug.......................... 23 0 Ch1
....................................................................uaCcacagccagcuuugaug.......................... 1 1 Ch1
....................................................................uaucacagcAagcuuugaug.......................... 1 1 Ch1
....................................................................uaucacagccagcuuugauU.......................... 2 1 Ch1
```

```
                              Star                                  Mature
cacaagagauguaaagucaguuccccaucacucacaaaguggcuguuauaugcugcaaauaagguucauaucacagccagcuuugaugagcgaagaggauacuguuggcaucaa
....................................................................uaucacagccagcuuugauga.........................   32   0   Ch1
....................................................................uaucacagcAagcuuugauga.........................   1    1   Ch1
....................................................................uaucGcagccagcuuugaugag........................   1    1   Ch1
....................................................................uaucacagccagcuuugaugUg........................   1    1   Ch1
....................................................................uaucacagccagcuuugaugaC........................   1    1   Ch1
....................................................................uaucacagccagcuuugaugaA........................   1    1   Ch1
....................................................................uaucacagccagcuuugaugag........................   45   0   Ch1
....................................................................uaucacagccagcuuugUugagc.......................   1    1   Ch1
....................................................................uaucacagccagcuuugaugaAc.......................   1    1   Ch1
....................................................................uaucacagccagcuuugaCgagc.......................   1    1   Ch1
....................................................................uaucGcagccagcuuugaugagc.......................   4    1   Ch1
....................................................................uaucacagccagcuuugaugaCc.......................   1    1   Ch1
....................................................................uaucacagccagcuuuUaugagc.......................   1    1   Ch1
....................................................................uaucacagccagcuuugaugagA.......................   9    1   Ch1
....................................................................uaucacagccCgcuuugaugagc.......................   4    1   Ch1
....................................................................uaucacagccagcuuugaugaUc.......................   1    1   Ch1
....................................................................uaucacagUcagcuuugaugagc.......................   3    1   Ch1
....................................................................uaucacagccagcuuugaugagc.......................   534  0   Ch1
....................................................................Caucacagccagcuuugaugagc.......................   1    1   Ch1
....................................................................uaucUcagccagcuuugaugagc.......................   7    1   Ch1
....................................................................uaCcacagccagcuuugaugagc.......................   3    1   Ch1
....................................................................uCucacagccagcuuugaugagc.......................   2    1   Ch1
....................................................................uaucacagccagcuGugaugagc.......................   1    1   Ch1
....................................................................uaucacagccagcuuugaugCgc.......................   2    1   Ch1
....................................................................uaucacagccagcuuugaugaAcg......................   1    1   Ch1
....................................................................uauUacagccagcuuugaugagcg......................   1    1   Ch1
....................................................................uaucacagccCgcuuugaugagcg......................   5    1   Ch1
....................................................................uaucaUagccagcuuugaugagcg......................   1    1   Ch1
....................................................................uaCcacagccagcuuugaugagcg......................   1    1   Ch1
....................................................................uUucacagccagcuuugaugagcg......................   1    1   Ch1
....................................................................uaucUcagccagcuuugaugagcg......................   11   1   Ch1
....................................................................uaucacUgccagcuuugaugagcg......................   1    1   Ch1
....................................................................uaucacagccagcuGugaugagcg......................   1    1   Ch1
....................................................................Aaucacagccagcuuugaugagcg......................   2    1   Ch1
....................................................................uaucacagccagcuuugaugagcg......................   661  0   Ch1
....................................................................uGucacagccagcuuugaugagcg......................   1    1   Ch1
....................................................................uaucacagccagcuuugaugaUcg......................   1    1   Ch1
....................................................................uCucacagccagcuuugaugagcg......................   1    1   Ch1
....................................................................uaucGcagccagcuuugaugagcg......................   4    1   Ch1
....................................................................uaucacagccagcuuugauUagcg......................   1    1   Ch1
....................................................................uaucacagAcagcuuugaugagcg......................   4    1   Ch1
....................................................................uaucacagccagcuuugaugagcU......................   4    1   Ch1
....................................................................uaucacagccagcuuugaugagGga.....................   24   1   Ch1
....................................................................uaucacagccagcuuugaugagcgC.....................   45   1   Ch1
....................................................................uaucacagccagcuuugaugagcAa.....................   1    1   Ch1
....................................................................uaucacagccagcuuugaugagcga.....................   20   0   Ch1
....................................................................uaucacagccagcuuugaugagcgU.....................   372  1   Ch1
....................................................................uaucacagccaAcuuugaugagcga.....................   3    1   Ch1
....................................................................uaucacagccagcuuugaugagGgaa....................   5    1   Ch1
....................................................................uaucacagccagcuuugaugagcgUa....................   4    1   Ch1
.....................................................................aucacagccagcuuugaugag........................   1    0   Ch1
.....................................................................aucacagccagcuuugaugagc.......................   1    0   Ch1
......................................................................ucacagccagcuuugaugagc.......................   10   0   Ch1
......................................................................ucacagccagcuuCgaugagcg......................   1    1   Ch1
......................................................................ucacagccagcuuugaugagcg......................   2    0   Ch1
......................................................................ucacagccagcuuugaugagcgU.....................   1    1   Ch1
..........................................................................agccagcuuugaugagcg......................   1    0   Ch1

..............................cucacaaaguggcuguuaua................................................................   2    0   Le1
..............................cucacaaaguggcuguuauau...............................................................   2    0   Le1
..............................cucacaaaguggcuguuauaug..............................................................   18   0   Le1
..............................Nucacaaaguggcuguuauaug..............................................................   1    1   Le1
..............................Uucacaaaguggcuguuauaug..............................................................   1    1   Le1
...............................ucacaaaguggcuguuau.................................................................   2    0   Le1
...............................ucacaaaguggcuguuauaug..............................................................   2    0   Le1
.................................................................cauaucacagccagcuuugau............................   1    0   Le1
..................................................................auaucacagccagcuuug..............................   1    0   Le1
....................................................................uaucacagccagcuuuga............................   22   0   Le1
....................................................................uaucacagccagcuuugU............................   1    1   Le1
....................................................................uaucGcagccagcuuuga............................   1    1   Le1
```

```
                              Star                                      Mature
cacaagagauguaaagucaguuccccaucacucacaaaguggcuguuauaugcugcaaauaagguucauaucacagccagcuuugaugagcgaagaggauacuguuggcaucaa
....................................................................uauUacagccagcuuuga............................    1     1     Le1
....................................................................uaucacagccagcuuugaA...........................    4     1     Le1
....................................................................uaucacagccagcuuugaG...........................    1     1     Le1
....................................................................uaucUcagccagcuuugau...........................    1     1     Le1
....................................................................uaucacagccagcuuugau...........................    52    0     Le1
....................................................................uaucGcagccagcuuugau...........................    1     1     Le1
....................................................................uaucUcagccagcuuugaug..........................    1     1     Le1
....................................................................uaucacagccagcuuugaug..........................    13    0     Le1
....................................................................uaucacagccagcuuugauA..........................    2     1     Le1
....................................................................uaucacagccagcuuugauga.........................    26    0     Le1
....................................................................uaucacagccagcuuugaugag........................    36    0     Le1
....................................................................uaucaUagccagcuuugaugag........................    1     1     Le1
....................................................................uaucacagccagcuuugaugaA........................    1     1     Le1
....................................................................Naucacagccagcuuugaugagc.......................    1     1     Le1
....................................................................uaucacagccagcuuugaugagc.......................    218   0     Le1
....................................................................uaucUcagccagcuuugaugagc.......................    2     1     Le1
....................................................................uaucacagccagcuuugaugaCc.......................    1     1     Le1
....................................................................uaucGcagccagcuuugaugagc.......................    1     1     Le1
....................................................................uauUacagccagcuuugaugagc.......................    1     1     Le1
....................................................................uaucacagUcagcuuugaugagc.......................    2     1     Le1
....................................................................uCucacagccagcuuugaugagc.......................    3     1     Le1
....................................................................uaucacagccagcuuugaugagA.......................    4     1     Le1
....................................................................uaucacagAcagcuuugaugagc.......................    2     1     Le1
....................................................................uaucacagccCgcuuugaugagc.......................    1     1     Le1
....................................................................uaucacagccagcuuugaugCgc.......................    1     1     Le1
....................................................................Caucacagccagcuuugaugagc.......................    2     1     Le1
....................................................................uCucacagccagcuuugaugagcg......................    1     1     Le1
....................................................................uaucGcagccagcuuugaugagcg......................    2     1     Le1
....................................................................uaucacagccagcuuugaugagcU......................    6     1     Le1
....................................................................uaucacaAccagcuuugaugagcg......................    1     1     Le1
....................................................................uaucacagccagcCuugaugagcg......................    1     1     Le1
....................................................................uaCcacagccagcuuugaugagcg......................    1     1     Le1
....................................................................uaucacagccagcuuugaugagcg......................    339   0     Le1
....................................................................uaucacagccCgcuuugaugagcg......................    2     1     Le1
....................................................................uaucacagccagcuuugaugCgcg......................    1     1     Le1
....................................................................uaucUcagccagcuuugaugagcg......................    3     1     Le1
....................................................................Caucacagccagcuuugaugagcg......................    1     1     Le1
....................................................................uaucacagccagcuuugaugagcC......................    2     1     Le1
....................................................................uaucacagcAagcuuugaugagcg......................    1     1     Le1
....................................................................uaucacagccagcuuuAaugagcg......................    1     1     Le1
....................................................................uCucacagccagcuuugaugagcga.....................    1     1     Le1
....................................................................uaucacagccagcuuugaugagcga.....................    17    0     Le1
....................................................................uaucacagccagcuuugaugagGga.....................    15    1     Le1
....................................................................uaucacagccagcuuugaugagcgC.....................    29    1     Le1
....................................................................uaucacagccaAcuuugaugagcga.....................    1     1     Le1
....................................................................uaucacagccagcuuugaugagcgU.....................    218   1     Le1
....................................................................uaucacagccagcuuugaugagAga.....................    1     1     Le1
....................................................................uaucacagccagcuuugaugagcAa.....................    1     1     Le1
....................................................................uaucacagccagcuuugaugagcgUa....................    1     1     Le1
....................................................................uaucacagccagcuuugaugagGgaa....................    1     1     Le1
.....................................................................aucacagccagcuuugaugagc.......................    1     0     Le1
.....................................................................aucacagccagcuuugaugagcgU.....................    1     1     Le1
......................................................................ucacagccCgcuuugaugagc.......................    1     1     Le1
......................................................................ucacagccagcuuugaugagc.......................    6     0     Le1

..............................cucacaaaguggcuguuau.................................................................    1     0     Br1
..............................cucacaaaguggcuguuaua................................................................    5     0     Br1
..............................Aucacaaaguggcuguuaua................................................................    1     1     Br1
..............................cucacaaaguggcuguuauau...............................................................    8     0     Br1
..............................cucaUaaaguggcuguuauau...............................................................    1     1     Br1
..............................cuAacaaaguggcuguuauaug..............................................................    1     1     Br1
..............................cucacaaaguggcuguuauaug..............................................................    27    0     Br1
..............................cucacaaaguggcuguuauauAc.............................................................    1     1     Br1
..............................cucacaaaguggcuguuauaugA.............................................................    1     1     Br1
...............................ucacaaaguggcuguuauau...............................................................    2     0     Br1
...............................ucacaaaguggcuguuauaug..............................................................    1     0     Br1
...............................ucacaaaguggcuguuauaugA.............................................................    1     1     Br1
..................................caaaguggcuguuauaug..............................................................    1     0     Br1
..................................................................cauaucacagccagcuuugau...........................    1     0     Br1
..................................................................cauaucacagccagcuuugaug..........................    1     0     Br1
```

Star Mature

```
cacaagagauguaaagucaguuccccaucacucacaaaguggcuguuauaugcugcaaauaagguucauaucacagccagcuuugaugagcgaagaggauacuguuggcaucaa
..................................................................Aauaucacagccagcuuugaugagc.......................	1	1	Br1
...................................................................auaucacagccagcuuugaugagc.......................	2	0	Br1
...................................................................auaucacagccagcuuugaugagcg......................	1	0	Br1
....................................................................uaucacagccCgcuuuga............................	1	1	Br1
....................................................................uaucacagccagcuuuga............................	6	0	Br1
....................................................................uaucacagccagcuuugau...........................	44	0	Br1
....................................................................Aaucacagccagcuuugau...........................	1	1	Br1
....................................................................Caucacagccagcuuugau...........................	1	1	Br1
....................................................................uaucacagccCgcuuugau...........................	1	1	Br1
....................................................................uaucacagccagcuuugaA...........................	4	1	Br1
....................................................................uaCcacagccagcuuugau...........................	1	1	Br1
....................................................................uaucacagccagcuuugaC...........................	2	1	Br1
....................................................................uaucUcagccagcuuugau...........................	2	1	Br1
....................................................................uaucGcagccagcuuugau...........................	1	1	Br1
....................................................................uaucacagccagcuuugauU..........................	2	1	Br1
....................................................................uaucacagccagcuuugaug..........................	38	0	Br1
....................................................................uaucacagccagcuuugauA..........................	4	1	Br1
....................................................................uaucacagccagAuuugaug..........................	1	1	Br1
....................................................................uauAacagccagcuuugauga.........................	1	1	Br1
....................................................................uaucacagUcagcuuugauga.........................	1	1	Br1
....................................................................uaucacagccagcCuugauga.........................	1	1	Br1
....................................................................Naucacagccagcuuugauga.........................	1	1	Br1
....................................................................uaucacagccagcuuugauga.........................	108	0	Br1
....................................................................uaCcacagccagcuuugaugag........................	1	1	Br1
....................................................................uaucUcagccagcuuugaugag........................	1	1	Br1
....................................................................uUucacagccagcuuugaugag........................	1	1	Br1
....................................................................uaucacagccagcuuCgaugag........................	1	1	Br1
....................................................................uaucacagccagcuuugaugag........................	97	0	Br1
....................................................................uaucacagccagcuuugaugaA........................	5	1	Br1
....................................................................uCucacagccagcuuugaugag........................	1	1	Br1
....................................................................uaucacagccUgcuuugaugag........................	1	1	Br1
....................................................................uaucacagAcagcuuugaugag........................	1	1	Br1
....................................................................uaucacagccagcuuugaugCg........................	1	1	Br1
....................................................................uaucacagccagcuuGgaugagc.......................	2	1	Br1
....................................................................Caucacagccagcuuugaugagc.......................	2	1	Br1
....................................................................uaucacagccagcuuugauUagc.......................	3	1	Br1
....................................................................uauUacagccagcuuugaugagc.......................	3	1	Br1
....................................................................uaucacagccagcCuugaugagc.......................	2	1	Br1
....................................................................uaucaUagccagcuuugaugagc.......................	1	1	Br1
....................................................................uaucacagccagcuuugaugagc.......................	961	0	Br1
....................................................................uaucacagccagcuuugaugaCc.......................	6	1	Br1
....................................................................uaCcacagccagcuuugaugagc.......................	4	1	Br1
....................................................................uaucacagccCgcuuugaugagc.......................	4	1	Br1
....................................................................uaucacagccagcuuugaugagA.......................	26	1	Br1
....................................................................uaucacagcUagcuuugaugagc.......................	1	1	Br1
....................................................................uaucacagccagcuGugaugagc.......................	1	1	Br1
....................................................................uaucacagccagcuuugaugaUc.......................	1	1	Br1
....................................................................uaucacaUccagcuuugaugagc.......................	1	1	Br1
....................................................................uaucGcagccagcuuugaugagc.......................	3	1	Br1
....................................................................uaucacagccagUuuugaugagc.......................	1	1	Br1
....................................................................uaucacagccagcuuugauCagc.......................	1	1	Br1
....................................................................uaucacagccUgcuuugaugagc.......................	1	1	Br1
....................................................................uaucacagccagcuuugUugagc.......................	1	1	Br1
....................................................................Aaucacagccagcuuugaugagc.......................	1	1	Br1
....................................................................uaucUcagccagcuuugaugagc.......................	14	1	Br1
....................................................................uaucacagccagAuuugaugagc.......................	1	1	Br1
....................................................................uaucacagUcagcuuugaugagc.......................	1	1	Br1
....................................................................uaucacaAccagcuuugaugagc.......................	1	1	Br1
....................................................................uaucacagAcagcuuugaugagc.......................	5	1	Br1
....................................................................uaucacagccCgcuuugaugagcg......................	5	1	Br1
....................................................................uaucacagccagcuuugaugagcg......................	1068	0	Br1
....................................................................Caucacagccagcuuugaugagcg......................	5	1	Br1
....................................................................uaucaUagccagcuuugaugagcg......................	1	1	Br1
....................................................................uaucGcagccagcuuugaugagcg......................	8	1	Br1
....................................................................uaucacagccaCcuuugaugagcg......................	1	1	Br1
....................................................................uaucacagccagcuuugaugagcU......................	9	1	Br1
....................................................................Aaucacagccagcuuugaugagcg......................	2	1	Br1
....................................................................uGucacagccagcuuugaugagcg......................	1	1	Br1
....................................................................uaucacagccagcuuugaugCgcg......................	1	1	Br1
....................................................................uaucacaAccagcuuugaugagcg......................	1	1	Br1
```

```
                              Star                                   Mature
cacaagagauguaaagucaguuccccaucacucacaaaguggcuguuauaugcugcaaauaagguucauaucacagccagcuuugaugagcgaagaggauacuguuggcaucaa
....................................................................uaucacagcAagcuuugaugagcg......................   1     1     Br1
....................................................................uaucacagccagcuuugauUagcg......................   1     1     Br1
....................................................................uaucacagccagcCuugaugagcg......................   3     1     Br1
....................................................................Naucacagccagcuuugaugagcg......................   1     1     Br1
....................................................................uaucacagccagcuuugaugaUcg......................   1     1     Br1
....................................................................uaCcacagccagcuuugaugagcg......................   1     1     Br1
....................................................................uCucacagccagcuuugaugagcg......................   2     1     Br1
....................................................................uaucacagAcagcuuugaugagcg......................   1     1     Br1
....................................................................uaucacagccagcuuugUugagcg......................   3     1     Br1
....................................................................uaucacagccagAuuugaugagcg......................   1     1     Br1
....................................................................Gaucacagccagcuuugaugagcg......................   1     1     Br1
....................................................................uaucacagccagcuuugaugagcC......................   5     1     Br1
....................................................................uaucUcagccagcuuugaugagcg......................   8     1     Br1
....................................................................uauUacagccagcuuugaugagcg......................   4     1     Br1
....................................................................uUucacagccagcuuugaugagcg......................   1     1     Br1
....................................................................uauUacagccagcuuugaugagcga.....................   1     1     Br1
....................................................................uaucGcagccagcuuugaugagcga.....................   1     1     Br1
....................................................................uaucacagccCgcuuugaugagcga.....................   1     1     Br1
....................................................................uaucacagccagcuuugaugagcgC.....................   132   1     Br1
....................................................................uaucacagAcagcuuugaugagcga.....................   2     1     Br1
....................................................................uaucacagccagcuuugaugagcgU.....................   649   1     Br1
....................................................................uaucacagccaAcuuugaugagcga.....................   3     1     Br1
....................................................................uaucUcagccagcuuugaugagcga.....................   1     1     Br1
....................................................................uaucacagccagcuuugaugagGga.....................   34    1     Br1
....................................................................uaucacagccagcuuugaugagcga.....................   65    0     Br1
....................................................................uaucacagccagcuuugaugagAga.....................   1     1     Br1
....................................................................uaucacagccagcuuugaugagcAa.....................   3     1     Br1
....................................................................uaucacagccagcCuugaugagcga.....................   1     1     Br1
....................................................................uaucacagccagcuuugaugagGgaa....................   2     1     Br1
....................................................................uaucacagccagcuuugaugagcgUa....................   8     1     Br1
....................................................................uaucacagccagcuuugaugagcgaU....................   1     1     Br1
.....................................................................aucacagccagcuuugaugagc.......................   1     0     Br1
.....................................................................aucacagccagcuuugaugagcg......................   1     0     Br1
.....................................................................auUacagccagcuuugaugagcga.....................   1     1     Br1
.....................................................................aucacagccagcuuugaugagcgU.....................   1     1     Br1
......................................................................ucacagccagcuuugauga.........................   1     0     Br1
......................................................................ucacagccagcuuugaugagc.......................   2     0     Br1
......................................................................ucacagccagcuuugaugagcg......................   1     0     Br1
.......................................................................cacagccagcuuugaugagc.......................   1     0     Br1
........................................................................acagccagcuuugaugag........................   1     0     Br1
........................................................................acagccagcuuugaugagc.......................   2     0     Br1

..............................cucacaaaguggcuguuaua................................................................   2     0     Le5
..............................cucacaaaguggcuguuauau...............................................................   7     0     Le5
..............................cucacaaaguggcuguuauaug..............................................................   37    0     Le5
..............................cucacaaaguggcuguuauaugA.............................................................   3     1     Le5
...............................ucacaaaguggcuguuauaug..............................................................   4     0     Le5
..................................................................cauaucacagccagcuuugau...........................   1     0     Le5
....................................................................uaucUcagccagcuuuga............................   1     1     Le5
....................................................................uaucacagccagcuuuga............................   14    0     Le5
....................................................................uaucacagccagcuuugaA...........................   6     1     Le5
....................................................................uaucacagccagcuuCgau...........................   1     1     Le5
....................................................................uaucacagccagcuuugaC...........................   4     1     Le5
....................................................................uaucacagccagcuuugau...........................   67    0     Le5
....................................................................Caucacagccagcuuugau...........................   1     1     Le5
....................................................................uaucacagccagcuuugaug..........................   22    0     Le5
....................................................................uaucacagccagcuuugauA..........................   3     1     Le5
....................................................................uaucacaAccagcuuugauga.........................   1     1     Le5
....................................................................uaucacagccagcuuugaugU.........................   1     1     Le5
....................................................................uaucacagccagcuuugauga.........................   44    0     Le5
....................................................................uaucGcagccagcuuugauga.........................   1     1     Le5
....................................................................uaucacagAcagcuuugauga.........................   1     1     Le5
....................................................................uaucacagccagcuuugaugUg........................   1     1     Le5
....................................................................uaucacagccagcuuugaugaA........................   2     1     Le5
....................................................................uaucacagUcagcuuugaugag........................   2     1     Le5
....................................................................uaucacagccagcuuugaugag........................   46    0     Le5
....................................................................uaucUcagccagcuuugaugagc.......................   2     1     Le5
....................................................................uaucGcagccagcuuugaugagc.......................   6     1     Le5
....................................................................uaucacagccagcCuugaugagc.......................   1     1     Le5
....................................................................uaucacagccagcuuugaugagA.......................   5     1     Le5
```

Star Mature

```
cacaagagauguaaagucaguuccccaucacucacaaaguggcuguuauaugcugcaaauaagguucauaucacagccagcuuugaugagcgaagaggauacuguuggcaucaa
....................................................................uaucacagUcagcuuugaugagc.......................	2	1	Le5
....................................................................uaucacagccagcuuugauUagc.......................	1	1	Le5
....................................................................uaucacagccagcuuugaugagc.......................	507	0	Le5
....................................................................uCucacagccagcuuugaugagc.......................	4	1	Le5
....................................................................uaucacagccagcuuGgaugagc.......................	1	1	Le5
....................................................................uaucacagccCgcuuugaugagc.......................	2	1	Le5
....................................................................uaucacagAcagcuuugaugagc.......................	1	1	Le5
....................................................................uaucacagccagcuuugaugCgc.......................	1	1	Le5
....................................................................uaucaUagccagcuuugaugagc.......................	1	1	Le5
....................................................................Aaucacagccagcuuugaugagc.......................	2	1	Le5
....................................................................uaCcacagccagcuuugaugagc.......................	3	1	Le5
....................................................................uauUacagccagcuuugaugagc.......................	3	1	Le5
....................................................................uaucacagccagcuuugaugagcg......................	572	0	Le5
....................................................................uaucUcagccagcuuugaugagcg......................	11	1	Le5
....................................................................uaucacagUcagcuuugaugagcg......................	1	1	Le5
....................................................................Aaucacagccagcuuugaugagcg......................	1	1	Le5
....................................................................uaucacagccagcuuugaugagcC......................	1	1	Le5
....................................................................uauUacagccagcuuugaugagcg......................	2	1	Le5
....................................................................uaucacagccGgcuuugaugagcg......................	1	1	Le5
....................................................................uaucacagccaCcuuugaugagcg......................	1	1	Le5
....................................................................uaucacagccagcuuugaugGgcg......................	1	1	Le5
....................................................................Caucacagccagcuuugaugagcg......................	1	1	Le5
....................................................................Naucacagccagcuuugaugagcg......................	1	1	Le5
....................................................................uaucacagccagcuuugauUagcg......................	1	1	Le5
....................................................................uaucGcagccagcuuugaugagcg......................	4	1	Le5
....................................................................uUucacagccagcuuugaugagcg......................	1	1	Le5
....................................................................uCucacagccagcuuugaugagcg......................	5	1	Le5
....................................................................uaucacagccagcuuugUugagcg......................	2	1	Le5
....................................................................uaucacagccCgcuuugaugagcg......................	1	1	Le5
....................................................................uaucacagAcagcuuugaugagcg......................	3	1	Le5
....................................................................uaucacagccagcuuugaugagcU......................	1	1	Le5
....................................................................uaucacagccagcuuugaugaUcg......................	1	1	Le5
....................................................................uaucacaAccagcuuugaugagcg......................	1	1	Le5
....................................................................uaucacagccagcuuugaugagGga.....................	32	1	Le5
....................................................................uaucacagccagcuuugaugagcga.....................	28	0	Le5
....................................................................uaucacagccagcuuugaugagcgU.....................	396	1	Le5
....................................................................uaucacagccagcuuugaugagcAa.....................	3	1	Le5
....................................................................uaucacagccagcuuugaugagcgC.....................	45	1	Le5
....................................................................uaucacagccagcuuugaugagcgaU....................	3	1	Le5
....................................................................uaucacagccagcuuugaugagGgaa....................	4	1	Le5
....................................................................uaucacagccagcuuugaugagcgUa....................	7	1	Le5
....................................................................uaucacagccagcuuugaugagcgaaA...................	2	1	Le5
......................................................................ucacagccagcuuugaugagc.......................	3	0	Le5
......................................................................ucacagccagcuuugaugCgcg......................	1	1	Le5
```

Provisional ID	:	Sc28yqQ_4222_2916
Score total	:	1176.8
Score for star read(s)	:	3.9
Score for read counts	:	1166.6
Score for mfe	:	1.7
Score for randfold	:	1.6
Score for cons. seed	:	3
Total read count	:	2300
Mature read count	:	2231
Loop read count	:	2
Star read count	:	67

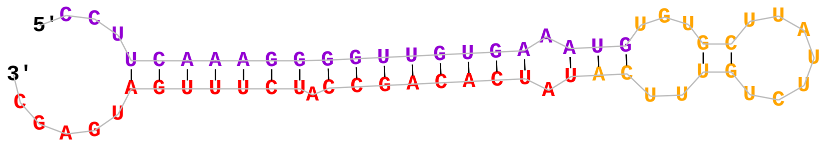

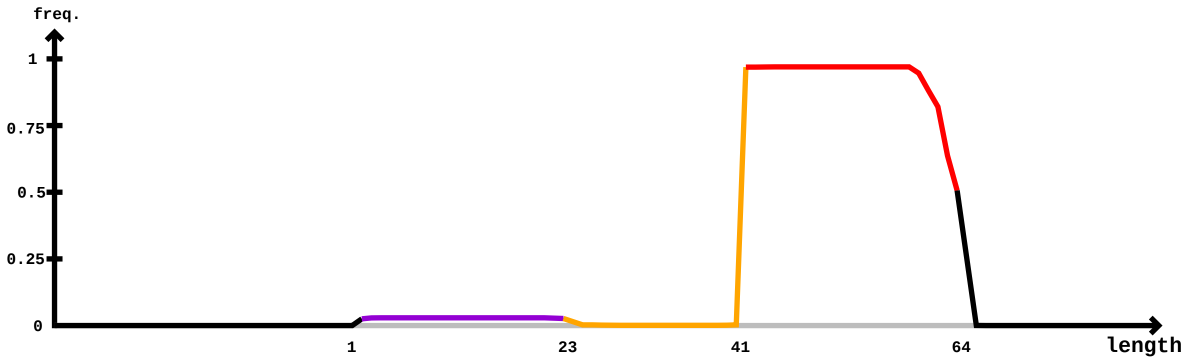

Star   Mature

```
5'- auuuucaaagguuaauuuccuguuuugcagccuucaaaggggguugugaaaugugugcuuauucuguuucauaucacagccaucuuugaugagcuguacagcaguuccagacug  -3'  obs
    auuuucaaagguuaauuuccuguuuugcagccuucaaaggggguugugaaaugugugcuuauucuguuucauaucacagccaucuuugaugagcuguacagcaguuccagacug       exp
    .........((((......(((((.(((((((.((((((((((((((.(((...((.......))..))).)))))))).))))))).).))))))).))))).....)))).  reads  mm  sample
    ..............................ccuucaaaggggguugugaaaugA............................................................  6  1  Ta1
    .......................................................................uaucacagccaucuuugau........................  5  0  Ta1
    .......................................................................uaucacagccaucuuugaug.......................  8  0  Ta1
    .......................................................................uaucacagccaucuuugauga......................  11  0  Ta1
    .......................................................................uaucacagccaucuuugaugag.....................  3  0  Ta1
    .......................................................................uaucacaAccaucuuugaugagc....................  1  1  Ta1
    .......................................................................uaucacagccaucuuugaugagc....................  24  0  Ta1
    .......................................................................uaucUcagccaucuuugaugagc....................  2  1  Ta1
    .......................................................................uaucacagccaucuuugaugagA....................  9  1  Ta1
    .......................................................................uaucGcagccaucuuugaugagc....................  1  1  Ta1

    ..............................ccuucaaaggggguugugaaau..............................................................  1  0  Ch1
    ..............................ccuucaaaggggguugugaaaug.............................................................  7  0  Ch1
    ..............................ccuGcaaaggggguugugaaaug.............................................................  1  1  Ch1
    ..............................ccuucaaaggggguugugaaaugu............................................................  1  0  Ch1
    ...............................cuucaaaggggguugugaaaug.............................................................  1  0  Ch1
    .......................................................................uaucacagccaucuuuga.........................  7  0  Ch1
    .......................................................................uaucacagccaucuuugau........................  13  0  Ch1
    .......................................................................uaucacagccaucuuugaA........................  3  1  Ch1
    .......................................................................uaucacagccaucuuugaug.......................  19  0  Ch1
    .......................................................................uaucacagccaucuuugauU.......................  2  1  Ch1
    .......................................................................uaucacagccaucuuugauga......................  32  0  Ch1
    .......................................................................uaucacagccaucAuugauga......................  1  1  Ch1
    .......................................................................uaucacagccaucuCugauga......................  1  1  Ch1
    .......................................................................uaucUcagccaucuuugauga......................  1  1  Ch1
    .......................................................................Aaucacagccaucuuugauga......................  1  1  Ch1
    .......................................................................uaucGcagccaucuuugauga......................  1  1  Ch1
    .......................................................................uaCcacagccaucuuugaugag.....................  1  1  Ch1
    .......................................................................uaucacagAcaucuuugaugag.....................  1  1  Ch1
    .......................................................................uaucacagccaucuuugaugaA.....................  2  1  Ch1
    .......................................................................uaucacagccaucuuugaugag.....................  26  0  Ch1
    .......................................................................uaucGcagccaucuuugaugagc....................  1  1  Ch1
    .......................................................................Aaucacagccaucuuugaugagc....................  1  1  Ch1
```

Star	Mature		
auuuucaaagguuaauuuccuguuuugcagccuucaaagggguugugaaaugugugcuuauucuguuucauaucacagccaucuuugaugagcuguacagcaguuccagacug			
....uaCcacagccaucuuugaugagc....................	1	1	Ch1
....uaucacagccaucuuugaugagc....................	112	0	Ch1
....uaucUcagccaucuuugaugagc....................	2	1	Ch1
....uaucacagccauAuuugaugagc....................	1	1	Ch1
....uaucacagccaucuuugaugagA....................	2	1	Ch1
....uCucacagccaucuuugaugagc....................	2	1	Ch1
....uaucacagccaucuuugaugagU....................	1	1	Ch1
....uaucacagccaucuuugaugagG....................	1	1	Ch1
....uaucacagUcaucuuugaugagc....................	1	1	Ch1
....uaucacagccaucuuugaugagcC...................	4	1	Ch1
....uaucacagccaGcuuugaugagcu...................	4	1	Ch1
....uaucacagccCucuuugaugagcu...................	2	1	Ch1
....uaucacagccaucuuugaugagcu...................	23	0	Ch1
..............................ccuucaaagggguugugaaa...	1	0	Bl1
..............................ccuucaaagggguugugaaaug...	1	0	Bl1
..............................ccuucaaagggguugugaaaugu..	2	0	Bl1
..............................ccuucaaagggguugugaaaugugu..	1	0	Bl1
....uaucacagccaucuuuga.........................	7	0	Bl1
....uaucacagccaucuuugau........................	16	0	Bl1
....uaucacagccaucuuugaA........................	3	1	Bl1
....uaucacagccaucuuugaug.......................	11	0	Bl1
....uaucacagccaucuGugaug.......................	1	1	Bl1
....uaucacagccCucuuugaug.......................	1	1	Bl1
....uaucacagUcaucuuugauga......................	1	1	Bl1
....uaucacagccaucuuugauga......................	48	0	Bl1
....uaucGcagccaucuuugauga......................	1	1	Bl1
....uaucGcagccaucuuugaugag.....................	1	1	Bl1
....uaucacagccaucuuugaugag.....................	18	0	Bl1
....uaucacagccaucuuugaugagc....................	5	0	Bl1
....uaucacagccaucuuugaugagU....................	5	1	Bl1
....uaucacagccaucuuugaugagA....................	9	1	Bl1
....uaucacagccaucuuugaugagcA...................	1	1	Bl1
....uaucacagccaGcuuugaugagcu...................	1	1	Bl1
....uaucacagccaucuuugaugagcC...................	1	1	Bl1
....uaucacagccaAcuuugaugagcu...................	1	1	Bl1
....uaucacagccUucuuugaugagcu...................	1	1	Bl1
....ucacagccaucuuugaug.......................	1	0	Bl1
..............................ccuucaaagggguugugaaau..	1	0	He1
..............................ccuucaaagggguugugaaaug...	10	0	He1
..............................ccuucaaagggguugugaaaugA..	1	1	He1
..............................ccuucaaagggguugugaaaugu..	1	0	He1
..............................ccuucaaagggguugugaaaugugug...	1	0	He1
..............................ccuucaaagggguugugaaaugugugc..	1	0	He1
...............................cuucaaagggguugugaaaug...	5	0	He1
...............................cuucaaagggguugugaaaugA..	2	1	He1
....ugugcuuauucuguuuca...	1	0	He1
....cauaucacagccaucuuugaugagc....................	1	0	He1
....Cuaucacagccaucuuugaugagc....................	2	1	He1
....uaucacagccaucuuuAa.........................	1	1	He1
....uaucacagccaucuuuga.........................	23	0	He1
....uaucGcagccaucuuuga.........................	1	1	He1
....uaucacagccaucuuugaA........................	3	1	He1
....uaucacagccCucuuugau........................	2	1	He1
....uauUacagccaucuuugau........................	2	1	He1
....Gaucacagccaucuuugau........................	1	1	He1
....uaucacagccaucuuugaC........................	1	1	He1
....uaucacagccaucuuugau........................	60	0	He1
....Caucacagccaucuuugau........................	1	1	He1
....Caucacagccaucuuugaug.......................	1	1	He1
....uaucacagccaucuuugauA.......................	1	1	He1
....uaucacagccaucuuugaug.......................	48	0	He1
....uauUacagccaucuuugauga......................	3	1	He1
....uaucUcagccaucuuugauga......................	3	1	He1
....uaucGcagccaucuuugauga......................	2	1	He1
....Caucacagccaucuuugauga......................	2	1	He1
....uaucacagccaucuuugauga......................	169	0	He1
....uaCcacagccaucuuugauga......................	1	1	He1
....uaucacaUccaucuuugauga......................	1	1	He1

```
                              Star                                  Mature
auuuucaaagguuaauuuccuguuuugcagccuucaaagggguugugaaaugugugcuuauucuguuucauaucacagccaucuuugaugagcuguacagcaguuccagacug
......................................................................uaucacagccCucuuugauga......................  2    1    He1
......................................................................uaucacagccaucuuugaugaA.....................  11   1    He1
......................................................................uaucacagccaucuuugaugag.....................  126  0    He1
......................................................................uaucGcagccaucuuugaugag.....................  2    1    He1
......................................................................uaucacagccCucuuugaugag.....................  4    1    He1
......................................................................uaucUcagccaucuuugaugag.....................  5    1    He1
......................................................................uaucacagccaucuuugaugaC.....................  1    1    He1
......................................................................uaucacagccaucCuugaugag.....................  1    1    He1
......................................................................uaucacagccaucuCugaugagc....................  1    1    He1
......................................................................uaucUcagccaucuuugaugagc....................  3    1    He1
......................................................................uaucacagccaucuuugaugagc....................  282  0    He1
......................................................................uaucacagccaucuuugaugagG....................  5    1    He1
......................................................................uCucacagccaucuuugaugagc....................  1    1    He1
......................................................................uaucaAagccaucuuugaugagc....................  1    1    He1
......................................................................uaucacUgccaucuuugaugagc....................  1    1    He1
......................................................................uaucacagccaucuuugaugagA....................  19   1    He1
......................................................................Aaucacagccaucuuugaugagc....................  2    1    He1
......................................................................uaucacagccaucuuugaugaUc....................  1    1    He1
......................................................................uaucacagccaucuuugaugagU....................  20   1    He1
......................................................................Caucacagccaucuuugaugagc....................  1    1    He1
......................................................................uaucacagccaucuuugaGgagc....................  1    1    He1
......................................................................uauUacagccaucuuugaugagc....................  1    1    He1
......................................................................uaucGcagccaucuuugaugagc....................  1    1    He1
......................................................................uaucacagccaAcuuugaugagcu...................  6    1    He1
......................................................................uaucacagccaucuuugaugagcA...................  34   1    He1
......................................................................uaucacagccaucuuugaugagcG...................  6    1    He1
......................................................................uaucacagccaucuuugaugagcC...................  30   1    He1
......................................................................uaucacagccaGcuuugaugagcu...................  18   1    He1
......................................................................uaucacagccaucuuugaugagcu...................  95   0    He1
......................................................................uaucacagccaucuuuGugagcu...................  1    1    He1
......................................................................uaucacaAccaucuuugaugagcu...................  1    1    He1
......................................................................uaucacagccCucuuugaugagcu...................  2    1    He1

..............................ccuucaaagggguugugaaau..............................................................  1    0    Br1
..............................ccuucaaagggguugugaaaug.............................................................  7    0    Br1
..............................ccuuUaaagggguugugaaaug.............................................................  1    1    Br1
..............................ccuucaaagggguugugaaauguAu..........................................................  1    1    Br1
.....................................................................auaucacagccaucuuugaug.......................  1    0    Br1
......................................................................uaucacagccaucuuuga.........................  4    0    Br1
......................................................................uaucacagccaucuuugaA........................  3    1    Br1
......................................................................uaucacagccaucuuugau........................  13   0    Br1
......................................................................uaucacagccaucuuugaug.......................  19   0    Br1
......................................................................uUucacagccaucuuugaug.......................  1    1    Br1
......................................................................uaucacagccauUuuugaug.......................  1    1    Br1
......................................................................uaucacagccaucuuugauU.......................  3    1    Br1
......................................................................uaucUcagccaucuuugauga......................  1    1    Br1
......................................................................uaucacagccaucuuugauga......................  84   0    Br1
......................................................................uaucacagccaucCuugauga......................  1    1    Br1
......................................................................uaucacagccaucuuugauUa......................  1    1    Br1
......................................................................uaucGcagccaucuuugauga......................  1    1    Br1
......................................................................uaucacagccauAuuugauga......................  1    1    Br1
......................................................................Caucacagccaucuuugauga......................  1    1    Br1
......................................................................uaucacagccauAuuugaugag.....................  1    1    Br1
......................................................................uaucacagccCucuuugaugag.....................  1    1    Br1
......................................................................uaucUcagccaucuuugaugag.....................  1    1    Br1
......................................................................uaucacagccaucuuugaugag.....................  52   0    Br1
......................................................................uaucacagccaucuuugaugaA.....................  5    1    Br1
......................................................................uauUacagccaucuuugaugagc....................  1    1    Br1
......................................................................uaucacagccaucuuugaugaCc....................  1    1    Br1
......................................................................uaucacagccaucuuugaugagc....................  91   0    Br1
......................................................................uaucacagccaucuuugaugagA....................  18   1    Br1
......................................................................uaucacagccaucuuugaugaAc....................  2    1    Br1
......................................................................uaucacagccaucuuugaugagU....................  4    1    Br1
......................................................................uaucacagccaucuuugaugagcA...................  17   1    Br1
......................................................................uaucacagccaAcuuugaugagcu...................  1    1    Br1
......................................................................uaucacagccaGcuuugaugagcu...................  9    1    Br1
......................................................................uaucacagccaucuuugaugagcC...................  13   1    Br1
......................................................................uaucacagccaucuuugaugagcu...................  38   0    Br1

..............................ccuucaaagggguugugaaa...............................................................  2    0    Le1
```

Star **Mature**

auuuucaaagguuaauuuccuguuuugcagccuucaaagggguugugaaaugugugcuuauucuguuucauaucacagccaucuuugaugagcuguacagcaguuccagacug

..............................ccuucaaagggguugugaaaug...	4	0	Le1
..............................ccuucaaagggguugugaaaugu..	1	0	Le1
..............................ccuucaaagggguugugaaaugC..	1	1	Le1
................................uucaaagggguugugaaaug...	1	0	Le1
....uaucacagccaucuuuAa.........................	1	1	Le1
....uaucacagccaucuuuga.........................	8	0	Le1
....uaucacagccaucuuugau........................	5	0	Le1
....uaucacagccaucuuugaug.......................	12	0	Le1
....uaucacagccaucuuugauga......................	18	0	Le1
....uaucacagccaucuuugaugaA.....................	1	1	Le1
....uaucacagccaucuuugaugag.....................	22	0	Le1
....uaucacagccaucuuugaugagA....................	3	1	Le1
....uaucUcagccaucuuugaugagc....................	2	1	Le1
....uaucacagUcaucuuugaugagc....................	1	1	Le1
....uaucacagccaucuuugaugagc....................	21	0	Le1
....uaucGcagccaucuuugaugagc....................	1	1	Le1
....uaucacagccaucuuugaugagcA...................	6	1	Le1
....uaucUcagccaucuuugaugagcu...................	1	1	Le1
....uaucacagccaGcuuugaugagcu...................	6	1	Le1
....uaucacagccaucuuugaugagcu...................	12	0	Le1
..............................ccuucaaagggguugugaaaug...	2	0	Le5
..............................ccuucaaagggguugugaaaugA..	1	1	Le5
..............................ccuucaaagggguugugaaaugu..	1	0	Le5
....ugugcuuauucuguuuca...	1	0	Le5
....uaucacagccaucuuuga.........................	2	0	Le5
....uaucacagccaucuuugau........................	16	0	Le5
....uaucacagccaucuuugaA........................	1	1	Le5
....uaucacagccaucuuugaug.......................	9	0	Le5
....uaucacagccaucuuugaAg.......................	1	1	Le5
....uauUacagccaucuuugaug.......................	2	1	Le5
....uaucUcagccaucuuugauga......................	1	1	Le5
....uaucGcagccaucuuugauga......................	1	1	Le5
....uaucacagccaucuuugauga......................	30	0	Le5
....uaCcacagccaucuuugauga......................	1	1	Le5
....uaucacagccaucuuugaugag.....................	15	0	Le5
....uaucUcagccaucuuugaugagc....................	1	1	Le5
....uaucacagcUaucuuugaugagc....................	1	1	Le5
....uaucacagccaucuuugaugagA....................	5	1	Le5
....uaucacagccaucuuugaugaCc....................	1	1	Le5
....uaucacagccaucuuugauUagc....................	1	1	Le5
....uaucacagccaucuuCgaugagc....................	1	1	Le5
....uCucacagccaucuuugaugagc....................	1	1	Le5
....uaucacagccCucuuugaugagc....................	1	1	Le5
....uaucacaUccaucuuugaugagc....................	2	1	Le5
....uaucacagccauAuuugaugagc....................	1	1	Le5
....uaucacagccaucuuugaugagU....................	3	1	Le5
....uaucacagccaucuuugaugagG....................	1	1	Le5
....uaucGcagccaucuuugaugagc....................	3	1	Le5
....Aaucacagccaucuuugaugagc....................	1	1	Le5
....uaCcacagccaucuuugaugagc....................	1	1	Le5
....uaucacagccaucuuugaugagc....................	91	0	Le5
....uaucacagccaucuuugaugagUu...................	2	1	Le5
....uaucacagccaucuuugaugagcA...................	3	1	Le5
....uaucacagccaAcuuugaugagcu...................	1	1	Le5
....uaucacagccaucuuugaugagcC...................	9	1	Le5
....uaucacagccaucuuugaugagcu...................	27	0	Le5
....uaucacagccaGcuuugaugagcu...................	1	1	Le5
....uaucacagAcaucuuugaugagcu...................	1	1	Le5
....uaucacagccCucuuugaugagcu...................	1	1	Le5
....uaucacagccaucuuugaugagcug..................	1	0	Le5
....cacagccaucuuugaugagc....................	1	0	Le5

Provisional ID	:	Sc28yqQ_4222_2917
Score total	:	124340.9
Score for star read(s)	:	3.9
Score for read counts	:	124330.7
Score for mfe	:	1.8
Score for randfold	:	1.6
Score for cons. seed	:	3
Total read count	:	243881
Mature read count	:	176173
Loop read count	:	0
Star read count	:	67708

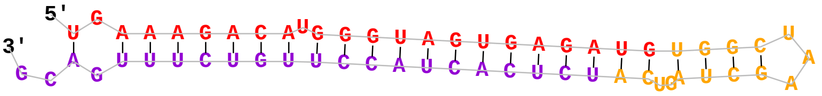

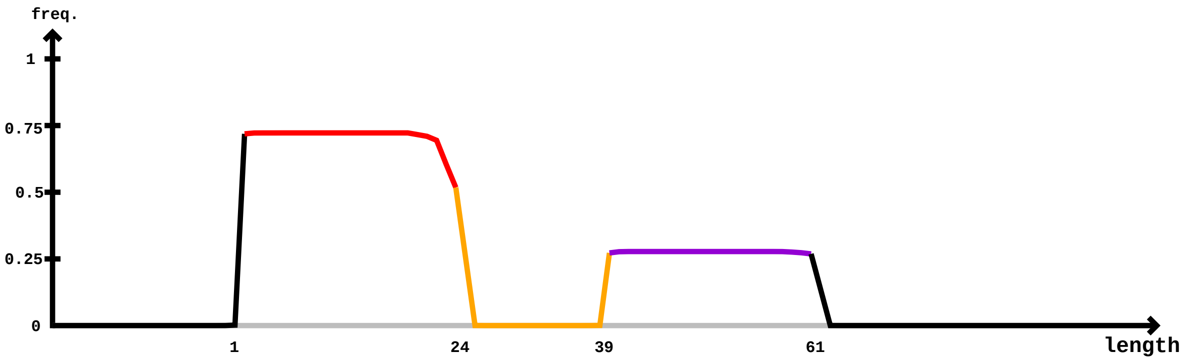

Mature **Star**

```
5'- aauaaacauuuuuuccca ugaaagacauggguagugagaug uggcuaagcuaguca ucucacuaccuugucuuugacg gaaagaauuguaaaagucaguacagaauggaaagu  -3'   obs
    aauaaacauuuuuucccaugaaagacauggguagugagauguggcuaagcuagucaucucacuaccuugucuuugacggaaagaauuguaaaagucaguacagaauggaaagu        exp
    .....((((((((((..(.(((((((.((((((((((((((((...))))..))))))))))))))))))).).))))))).))).............................   reads   mm   sample
    ...............ccCugaaagacauggguagugagaug........................................................................   1       1    He1
    ................Uaugaaagacauggguagugagaug........................................................................   1       1    He1
    ................Aaugaaagacauggguagugagaug........................................................................   1       1    He1
    .................augaaagacauggguagug.............................................................................   2       0    He1
    .................augaaagacauggguaguga............................................................................   1       0    He1
    .................augaaagacauggguagugag...........................................................................   1       0    He1
    .................Cugaaagacauggguagugag...........................................................................   2       1    He1
    .................augaaagacauggguagugaga..........................................................................   5       0    He1
    .................augaaagacauggguagugagaA.........................................................................   1       1    He1
    .................augaaagacauggguagugagau.........................................................................   12      0    He1
    .................Cugaaagacauggguagugagau.........................................................................   1       1    He1
    .................augaaagacauggguagugagauA........................................................................   1       1    He1
    .................augaUagacauggguagugagaug........................................................................   1       1    He1
    .................augaaagacauggguagugagauU........................................................................   1       1    He1
    .................augaaagacauggguagugagaAg........................................................................   1       1    He1
    .................augaaagacauggguagugagaug........................................................................   68      0    He1
    .................Cugaaagacauggguagugagaug........................................................................   11      1    He1
    .................augaaagacaugUguagugagaug........................................................................   1       1    He1
    .................augaaagacauggguagugagaugu.......................................................................   2       0    He1
    .................augaaagacauggguagugagauguA......................................................................   1       1    He1
    ..................ugaaagacauggguagCg.............................................................................   1       1    He1
    ..................ugaaGgacauggguagug.............................................................................   1       1    He1
    ..................ugaUagacauggguagug.............................................................................   2       1    He1
    ..................ugaaagacCuggguagug.............................................................................   6       1    He1
    ..................ugaaagacaugUguagug.............................................................................   1       1    He1
    ..................ugCaagacauggguagug.............................................................................   1       1    He1
    ..................ugaaagacauggguaguA.............................................................................   14      1    He1
    ..................uCaaagacauggguagug.............................................................................   1       1    He1
    ..................Cgaaagacauggguagug.............................................................................   1       1    He1
    ..................ugaaagacauggguagug.............................................................................   255     0    He1
    ..................Agaaagacauggguagug.............................................................................   1       1    He1
    ..................ugaaUgacauggguagug.............................................................................   3       1    He1
    ..................ugCaagacauggguaguga............................................................................   2       1    He1
```

Mature **Star**

aauaaacauuuuuucccaugaaagacauggguagugagauguggcuaagcuagucaucucacuaccuugucuuugacggaaagaauuguaaaagucaguacagaauggaaagu			
..................ugaaGgacauggguaguga..	3	1	He1
..................Agaaagacauggguaguga..	1	1	He1
..................Ngaaagacauggguaguga..	1	1	He1
..................ugaaUgacauggguaguga..	1	1	He1
..................uCaaagacauggguaguga..	1	1	He1
..................ugaUagacauggguaguga..	2	1	He1
..................ugaaagacauggguagugC..	3	1	He1
..................ugaaagacCuggguaguga..	3	1	He1
..................ugaaagacauggguaguga..	232	0	He1
..................ugaaagacauggAuaguga..	1	1	He1
..................ugaaagacauggguagugU..	11	1	He1
..................Ngaaagacauggguagugag...	3	1	He1
..................ugaUagacauggguagugag...	2	1	He1
..................ugaaagacauggguaguUag...	1	1	He1
..................ugaaagacauggguGgugag...	1	1	He1
..................ugCaagacauggguagugag...	4	1	He1
..................Agaaagacauggguagugag...	1	1	He1
..................ugaaagacauggUuagugag...	1	1	He1
..................ugaaagacCuggguagugag...	7	1	He1
..................Cgaaagacauggguagugag...	1	1	He1
..................ugaaagacaugggCagugag...	2	1	He1
..................ugaaagacauggguagCgag...	2	1	He1
..................ugaaagacauggguagugag...	874	0	He1
..................ugaaGgacauggguagugag...	8	1	He1
..................ugaaagaUauggguagugag...	3	1	He1
..................ugaaagacauggguagugaU...	4	1	He1
..................ugaaagacauggguagugaA...	34	1	He1
..................ugaaagacaugUguagugag...	2	1	He1
..................ugaaUgacauggguagugag...	10	1	He1
..................ugaaagacUuggguagugag...	1	1	He1
..................ugaaagacauAgguagugaga..	7	1	He1
..................ugaaaAacauggguagugaga..	1	1	He1
..................ugaaagacCuggguagugaga..	48	1	He1
..................ugaaaCacauggguagugaga..	1	1	He1
..................ugaaagacauggCuagugaga..	1	1	He1
..................Ngaaagacauggguagugaga..	8	1	He1
..................ugaaagacauggguagCgaga..	2	1	He1
..................ugaaagacauggguagugagU..	54	1	He1
..................ugaaagacauggAuagugaga..	1	1	He1
..................ugaaagacauggguagugagG..	12	1	He1
..................Cgaaagacauggguagugaga..	12	1	He1
..................ugaaagacaCgguagugaga..	1	1	He1
..................ugCaagacauggguagugaga..	9	1	He1
..................ugaaagacaAgguagugaga..	2	1	He1
..................ugaaagacauggguaUugaga..	2	1	He1
..................ugaUagacauggguagugaga..	10	1	He1
..................uCaaagacauggguagugaga..	2	1	He1
..................ugaaagacUuggguagugaga..	5	1	He1
..................ugaaagacauggguagugaCa..	9	1	He1
..................ugaaagacaugggCagugaga..	1	1	He1
..................ugaaagUcauggguagugaga..	5	1	He1
..................ugaaagaUauggguagugaga..	3	1	He1
..................ugaaagGcauggguagugaga..	2	1	He1
..................ugaaGgacauggguagugaga..	44	1	He1
..................ugaaagacauggguagugGga..	1	1	He1
..................Agaaagacauggguagugaga..	15	1	He1
..................ugaaagacaugUguagugaga..	6	1	He1
..................ugaaagacauggguUgugaga..	1	1	He1
..................uAaaagacauggguagugaga..	1	1	He1
..................ugaaaUacauggguagugaga..	2	1	He1
..................ugaaagacauggguagugaAa..	24	1	He1
..................ugaaUgacauggguagugaga..	54	1	He1
..................ugaaagacauggguagugaUa..	7	1	He1
..................ugaaagacauggguagugUga..	3	1	He1
..................ugaaagacauggguaguUaga..	1	1	He1
..................ugaaagacaugAguagugaga..	1	1	He1
..................ugaaagacauggguaguAaga..	1	1	He1
..................ugaaagacauggguagugaga..	4508	0	He1
..................ugGaagacauggguagugaga..	3	1	He1
..................ugaaagacauCgguagugaga..	1	1	He1

Mature **Star**

aauaaacauuuuuucccaugaaagacauggguagugagauguggcuaagcuagucaucucacuaccuugucuuugacggaaagaauuguaaaagucaguacagaauggaaagu			
..................ugaaagacauggguagugagC..	20	1	He1
..................ugaaagacauggguGgugagau...	7	1	He1
..................ugaaagacauggguagugagau...	4256	0	He1
..................ugaaagacauggguaAugagau...	1	1	He1
..................ugaUagacauggguagugagau...	10	1	He1
..................ugaaagacauggguagugagaA...	186	1	He1
..................ugaaagacauggguagugGgau...	3	1	He1
..................ugaaagacauggguagCgagau...	2	1	He1
..................ugaaaUacauggguagugagau...	1	1	He1
..................ugaaagacauggguagugagaC...	77	1	He1
..................Ngaaagacauggguagugagau...	6	1	He1
..................ugaaagUcauggguagugagau...	2	1	He1
..................Ggaaagacauggguagugagau...	1	1	He1
..................uUaaagacauggguagugagau...	1	1	He1
..................uAaaagacauggguagugagau...	2	1	He1
..................ugaaagacauAgguagugagau...	2	1	He1
..................ugaaagacaAggguagugagau...	1	1	He1
..................ugaaGgacauggguagugagau...	39	1	He1
..................ugaaagacauggguaUugagau...	1	1	He1
..................ugaaagacaugUguagugagau...	6	1	He1
..................ugaaagaAauggguagugagau...	1	1	He1
..................ugaaagacauggguagAgagau...	2	1	He1
..................ugaaagacauggguaguAagau...	1	1	He1
..................ugaaagacauggAuagugagau...	2	1	He1
..................uCaaagacauggguagugagau...	2	1	He1
..................ugaaagacauggguUgugagau...	1	1	He1
..................ugaaagacUuggguagugagau...	1	1	He1
..................ugaaagacauggguagugagGu...	3	1	He1
..................ugaaagacaugCguagugagau...	1	1	He1
..................ugaaagacauggguagugaUau...	8	1	He1
..................Agaaagacauggguagugagau...	4	1	He1
..................ugaaaCacauggguagugagau...	1	1	He1
..................ugaaagacauggguagugagUu...	5	1	He1
..................Cgaaagacauggguagugagau...	8	1	He1
..................ugaaagacauggguagugagCu...	8	1	He1
..................ugaaagacauUgguagugagau...	3	1	He1
..................ugaaagacaugggAagugagau...	2	1	He1
..................ugaaagacCuggguagugagau...	45	1	He1
..................ugGaagacauggguagugagau...	2	1	He1
..................ugaaUgacauggguagugagau...	44	1	He1
..................ugaaagacaCggguagugagau...	1	1	He1
..................ugCaagacauggguagugagau...	8	1	He1
..................ugaaagacauggguaguUagau...	1	1	He1
..................ugaaagacaugggCagugagau...	1	1	He1
..................ugaaagaUauggguagugagau...	4	1	He1
..................ugaaagacauggguagugagaug..	21792	0	He1
..................ugaaagacauggguagCgagaug..	3	1	He1
..................ugaaagacauggguagugCgaug..	1	1	He1
..................ugaaagacauggUuagugagaug..	5	1	He1
..................ugaaagacauggCuagugagaug..	3	1	He1
..................uCaaagacauggguagugagaug..	5	1	He1
..................uAaaagacauggguagugagaug..	7	1	He1
..................uUaaagacauggguagugagaug..	3	1	He1
..................ugaaagacauggguaUugagaug..	8	1	He1
..................ugaaagacauggguagugGgaug..	8	1	He1
..................ugaaagacaugggGagugagaug..	3	1	He1
..................ugaaagacaugggCagugagaug..	8	1	He1
..................ugaGagacauggguagugagaug..	5	1	He1
..................ugaaagacauggguagugaCaug..	2	1	He1
..................ugaaagacauggguagugaUaug..	39	1	He1
..................ugaaaAacauggguagugagaug..	2	1	He1
..................ugaaagacauggguaguCagaug..	2	1	He1
..................ugaaagUcauggguagugagaug..	6	1	He1
..................ugaaagacauggguagugagauU..	482	1	He1
..................ugaaagacauggguagugagauC..	20	1	He1
..................ugaaagacauUgguagugagaug..	5	1	He1
..................ugaaagacaugggAagugagaug..	7	1	He1
..................ugaaagacauggguagugagGug..	11	1	He1
..................ugaaagacaugCguagugagaug..	4	1	He1
..................ugaaagacauggguUgugagaug..	10	1	He1

```
                  Mature                               Star
aauaaacauuuuuuccca ugaaagacauggguagugagaug uggcuaagcuaguca ucucacuaccuugucuuugacg gaaagaauuguaaaagucaguacagaauggaaagu
..................Agaaagacauggguagugagaug........................................................................    35    1    He1
..................ugaaagacaugAguagugagaug........................................................................    2     1    He1
..................ugaaagacaCggguagugagaug........................................................................    2     1    He1
..................ugGaagacauggguagugagaug........................................................................    7     1    He1
..................ugUaagacauggguagugagaug........................................................................    2     1    He1
..................ugaaUgacauggguagugagaug........................................................................    238   1    He1
..................ugaaagacauggguagugaAaug........................................................................    1     1    He1
..................ugaaagacauggguaguUagaug........................................................................    10    1    He1
..................ugaaagacauggguagAgagaug........................................................................    6     1    He1
..................ugaaagacauggguaAugagaug........................................................................    1     1    He1
..................ugaaagacaAggguagugagaug........................................................................    4     1    He1
..................ugaaaUacauggguagugagaug........................................................................    6     1    He1
..................ugaaagacCuggguagugagaug........................................................................    234   1    He1
..................Ggaaagacauggguagugagaug........................................................................    6     1    He1
..................ugCaagacauggguagugagaug........................................................................    36    1    He1
..................ugaaagacauggguagugagCug........................................................................    11    1    He1
..................Ngaaagacauggguagugagaug........................................................................    23    1    He1
..................ugaaagGcauggguagugagaug........................................................................    4     1    He1
..................ugaaagacauAgguagugagaug........................................................................    3     1    He1
..................ugaUagacauggguagugagaug........................................................................    47    1    He1
..................ugaaagaAauggguagugagaug........................................................................    1     1    He1
..................ugaaagacauggguaguAagaug........................................................................    4     1    He1
..................ugaaagacaugUguagugagaug........................................................................    42    1    He1
..................ugaaagacauggguagugagaAg........................................................................    11    1    He1
..................Cgaaagacauggguagugagaug........................................................................    40    1    He1
..................ugaaagacauggguagugagauA........................................................................    249   1    He1
..................ugaaagacauCgguagugagaug........................................................................    1     1    He1
..................ugaaagacauggguGgugagaug........................................................................    15    1    He1
..................ugaaagacauggguagugagaCg........................................................................    99    1    He1
..................ugaaagacauggguagugUgaug........................................................................    7     1    He1
..................ugaaGgacauggguagugagaug........................................................................    156   1    He1
..................ugaaCgacauggguagugagaug........................................................................    1     1    He1
..................ugaaagacauggguagugagUug........................................................................    8     1    He1
..................ugaaagacUuggguagugagaug........................................................................    26    1    He1
..................ugaaagacauggAuagugagaug........................................................................    7     1    He1
..................ugaaagaUauggguagugagaug........................................................................    37    1    He1
..................ugaaagacauggguUgugagaugu.......................................................................    1     1    He1
..................ugaaGgacauggguagugagaugu.......................................................................    3     1    He1
..................ugaaagacaugUguagugagaugu.......................................................................    2     1    He1
..................ugaaagacauggguagugagauAu.......................................................................    3     1    He1
..................ugaaagacauggguagugagauUu.......................................................................    9     1    He1
..................ugaUagacauggguagugagaugu.......................................................................    1     1    He1
..................ugaaagacauggguagugagaugu.......................................................................    378   0    He1
..................ugaaagaUauggguagugagaugu.......................................................................    1     1    He1
..................ugaaagacauggguagugagaugG.......................................................................    6     1    He1
..................ugCaagacauggguagugagaugu.......................................................................    1     1    He1
..................ugaaagacauggguagugagauCu.......................................................................    1     1    He1
..................ugaaaUacauggguagugagaugu.......................................................................    1     1    He1
..................ugaaUgacauggguagugagaugu.......................................................................    3     1    He1
..................ugaaagacauggguagugagaugC.......................................................................    39    1    He1
..................ugaaagacCuggguagugagaugu.......................................................................    5     1    He1
..................ugaaagacauggguagugagaugug......................................................................    1     0    He1
..................ugaaagacauggguagugagaugAg......................................................................    2     1    He1
..................ugaaagacauggguagugagauguA......................................................................    7     1    He1
..................ugaaagacauggguagugagauguC......................................................................    2     1    He1
..................ugaaagacauggguagugagaugugC.....................................................................    1     1    He1
..................ugaaagacauggguagugagaugugg.....................................................................    1     0    He1
..................ugaaagacauggguagugagauguggU....................................................................    1     1    He1
..................ugaaagacauggguagugagauguggc....................................................................    1     0    He1
...................gaaagacauggguaguga............................................................................    1     0    He1
...................gaaaUacauggguagugag...........................................................................    1     1    He1
...................gaaagacauggguagugag...........................................................................    4     0    He1
...................gaaagacaCggguagugag...........................................................................    1     1    He1
...................gaCagacauggguagugag...........................................................................    1     1    He1
...................gaaUgacauggguagugaga..........................................................................    1     1    He1
...................gaaagacauggguagugaga..........................................................................    22    0    He1
...................gaaagUcauggguagugaga..........................................................................    1     1    He1
...................gaaagacaCggguagugaga..........................................................................    1     1    He1
...................gaaaUacauggguagugaga..........................................................................    3     1    He1
...................gaaagacUuggguagugaga..........................................................................    1     1    He1
```

```
                    Mature                                   Star
aauaaacauuuuuucccaugaaagacauggguagugagauguggcuaagcuagucaucucacuaccuugucuuugacggaaagaauuguaaaagucaguacagaauggaaagu
...................gaaUgacauggguagugagau.........................................................................   3    1    He1
...................gaaagacauggguagugagGu.........................................................................   1    1    He1
...................gaaaUacauggguagugagau.........................................................................   1    1    He1
...................gaaagacauggguaCugagau.........................................................................   1    1    He1
...................gaaagacauggguaguAagau.........................................................................   1    1    He1
...................gaaagacauggguagugagau.........................................................................   18   0    He1
...................gaaagacauggguUgugagau.........................................................................   1    1    He1
...................gaaagacauggguagugagauU........................................................................   3    1    He1
...................gaaagacaAggguagugagaug........................................................................   3    1    He1
...................gaaagacauggguagugUgaug........................................................................   1    1    He1
...................gaaaUacauggguagugagaug........................................................................   11   1    He1
...................gaaagacaCggguagugagaug........................................................................   1    1    He1
...................gaaagacauggUuagugagaug........................................................................   1    1    He1
...................gaaagacauggguagugagGug........................................................................   1    1    He1
...................gaaUgacauggguagugagaug........................................................................   5    1    He1
...................gaaagacauggguagugaAaug........................................................................   1    1    He1
...................gaaagacauggguUgugagaug........................................................................   1    1    He1
...................gaaagacauggguagugagauA........................................................................   2    1    He1
...................gaaagacauggguagugagaug........................................................................   82   0    He1
...................gaaagacauAgguagugagaug........................................................................   1    1    He1
...................gaaagacauggguCgugagaug........................................................................   1    1    He1
...................gaaagacauggguaUugagaug........................................................................   3    1    He1
...................Caaagacauggguagugagaug........................................................................   1    1    He1
...................gaaagacauggAuagugagaug........................................................................   2    1    He1
...................gaCagacauggguagugagaug........................................................................   1    1    He1
...................gaaagacauggguagugagaugu.......................................................................   1    0    He1
...................gaaagacauggguagugaUaugu.......................................................................   1    1    He1
....................aaagacauggguagugagaug........................................................................   3    0    He1
.....................aagacauggguagugagaug........................................................................   1    0    He1
......................agacauggguagugagaug........................................................................   3    0    He1
.......................gacauggguagugagaug........................................................................   1    0    He1
......................................................caucucacuaccuugucuuu.......................................   2    0    He1
......................................................caucucacuaccuugucuuug......................................   11   0    He1
......................................................Aaucucacuaccuugucuuugacg...................................   1    1    He1
.......................................................aucucacuaccuugucuuugac....................................   1    0    He1
.......................................................Cucucacuaccuugucuuugacg...................................   11   1    He1
.......................................................aucucacuaccuugucuuugacgU..................................   1    1    He1
........................................................ucucacuaccuugucuuu.......................................   19   0    He1
........................................................ucucacuaAcuugucuuu.......................................   2    1    He1
........................................................ucucUcuaccuugucuuug......................................   4    1    He1
........................................................ucucacuaAcuugucuuug......................................   4    1    He1
........................................................ucucacuaccuugucuuug......................................   131  0    He1
........................................................ucucGcuaccuugucuuug......................................   3    1    He1
........................................................ucucacuaccuugucuuuA......................................   1    1    He1
........................................................ucucacuacUuugucuuuga.....................................   1    1    He1
........................................................ucucacuaccuugucuuugU.....................................   2    1    He1
........................................................ucucacuaccuugucuuuga.....................................   184  0    He1
........................................................ucucacuUccuugucuuuga.....................................   1    1    He1
........................................................ucucacuaAcuugucuuuga.....................................   1    1    He1
........................................................ucucaUuaccuugucuuuga.....................................   1    1    He1
........................................................uAucacuaccuugucuuuga.....................................   1    1    He1
........................................................ucucacuaccuugucGuuga.....................................   1    1    He1
........................................................ucucGcuaccuugucuuuga.....................................   1    1    He1
........................................................uGucacuaccuugucuuuga.....................................   1    1    He1
........................................................ucucUcuaccuugucuuuga.....................................   3    1    He1
........................................................ucucacuaccuugucuuuUac....................................   2    1    He1
........................................................ucucUcuaccuugucuuugac....................................   2    1    He1
........................................................ucuUacuaccuugucuuugac....................................   2    1    He1
........................................................ucucacuaccuugucuuugGc....................................   1    1    He1
........................................................ucucacuaAcuugucuuugac....................................   1    1    He1
........................................................ucucacuaccuugucuuugaU....................................   4    1    He1
........................................................Acucacuaccuugucuuugac....................................   1    1    He1
........................................................ucucacuaccuugucGuugac....................................   1    1    He1
........................................................ucucGcuaccuugucuuugac....................................   2    1    He1
........................................................ucucacuaccuugucuuugac....................................   288  0    He1
........................................................uAucacuaccuugucuuugac....................................   1    1    He1
........................................................ucucacuaccuugucuuugUc....................................   1    1    He1
........................................................Ccucacuaccuugucuuugac....................................   1    1    He1
........................................................ucucacuUccuugucuuugac....................................   1    1    He1
........................................................ucucacuaccuuUucuuugac....................................   1    1    He1
```

Mature	Star		
aauaaacauuuuuucccaugaaagacauggguagugagauguggcuaagcuagucaucucacuaccuugucuuugacggaaagaauuguaaaagucaguacagaauggaaagu			
....ucucacuaccuugucCuugacg...................................	9	1	He1
....ucucacuaccuugucuuuUacg...................................	78	1	He1
....ucucacuaccuugCcuuugacg...................................	5	1	He1
....uAucacuaccuugucuuugacg...................................	40	1	He1
....ucucacuaccuugucuuugaUg...................................	15	1	He1
....ucucaUuaccuugucuuugacg...................................	13	1	He1
....ucucacuaccuugGcuuugacg...................................	1	1	He1
....ucucacuaccuGgucuuugacg...................................	4	1	He1
....ucucacuaccuugucuuugaGg...................................	2	1	He1
....ucucacuaccuugucGuugacg...................................	15	1	He1
....ucucacuaccuugucuuugUcg...................................	12	1	He1
....uGucacuaccuugucuuugacg...................................	8	1	He1
....ucucacuaccuugAcuuugacg...................................	4	1	He1
....ucucacuaccuugucuuGgacg...................................	2	1	He1
....ucucacuaccGugucuuugacg...................................	2	1	He1
....ucCcacuaccuugucuuugacg...................................	45	1	He1
....ucucacuaccuugucuuugacC...................................	53	1	He1
....ucucUcuaccuugucuuugacg...................................	225	1	He1
....ucucaAuaccuugucuuugacg...................................	1	1	He1
....ucucacuaccCugucuuugacg...................................	4	1	He1
....ucucacuacAuugucuuugacg...................................	13	1	He1
....ucucacuaccuugucuuugGcg...................................	10	1	He1
....ucucacuaAcuugucuuugacg...................................	75	1	He1
....ucucGcuaccuugucuuugacg...................................	167	1	He1
....ucucacuaccuugucuuugacg...................................	18250	0	He1
....Ncucacuaccuugucuuugacg...................................	19	1	He1
....ucucacuGccuugucuuugacg...................................	2	1	He1
....ucucacuaccuugucuuCgacg...................................	3	1	He1
....ucucacuUccuugucuuugacg...................................	26	1	He1
....ucucacuaGcuugucuuugacg...................................	2	1	He1
....Gcucacuaccuugucuuugacg...................................	4	1	He1
....Acucacuaccuugucuuugacg...................................	46	1	He1
....ucucacuaccuugucuuuAacg...................................	3	1	He1
....ucucacGaccuugucuuugacg...................................	2	1	He1
....ucucacuaccuugucuuuCacg...................................	1	1	He1
....ucucacuaccuugucuuugacU...................................	59	1	He1
....ucucacuaccuuguUuuugacg...................................	3	1	He1
....ucucacuaccAugucuuugacg...................................	4	1	He1
....ucucacCaccuugucuuugacg...................................	6	1	He1
....ucAcacuaccuugucuuugacg...................................	4	1	He1
....ucucacuaccuuguGuuugacg...................................	3	1	He1
....ucucacuacUuugucuuugacg...................................	10	1	He1
....Ccucacuaccuugucuuugacg...................................	21	1	He1
....ucucacuaccuugucuCugacg...................................	1	1	He1
....ucucacuaccuugucuAugacg...................................	2	1	He1
....ucuUacuaccuugucuuugacg...................................	61	1	He1
....ucuAacuaccuugucuuugacg...................................	1	1	He1
....ucucacuaccuugucAuugacg...................................	2	1	He1
....ucucacuaccuugucuuAgacg...................................	1	1	He1
....uUucacuaccuugucuuugacg...................................	9	1	He1
....ucucacuaccuugucuGugacg...................................	1	1	He1
....ucucacuaccuugucuuugCcg...................................	8	1	He1
....ucucacuaccuugucuuugacA...................................	723	1	He1
....ucucacuaccuuguAuuugacg...................................	2	1	He1
....ucucacuaccuCgucuuugacg...................................	3	1	He1
....ucucacuaccuuUucuuugacg...................................	1	1	He1
....ucucacuaccuuAucuuugacg...................................	3	1	He1
....ucucacuaccuugucuuugacgA..................................	62	1	He1
....ucucacuaccuugucuuugacgC..................................	28	1	He1
....ucucacuaccuugucuuugacgU..................................	521	1	He1
....ucucacuaccuugucuuugacgg..................................	6	0	He1
....ucucacuaccuugucuuugCcgg..................................	1	1	He1
....ucucGcuaccuugucuuugacgg..................................	1	1	He1
....ucucacuaccuugucuuugacgCa.................................	2	1	He1
....ucucacuaccuugucuuugacgAa.................................	3	1	He1
....ucucacuaccuugucuuugacgUa.................................	7	1	He1
....cucacuaccuugCcuuug......................................	1	1	He1
....cucacuacAuugucuuug......................................	1	1	He1
....cucacuaccuuUucuuug......................................	1	1	He1
....Aucacuaccuugucuuug......................................	1	1	He1

Mature Star

```
aauaaacauuuuuucccaugaaagacauggguagugagauguggcuaagcuagucaucucacuaccuugucuuugacggaaagaauuguaaaagucaguacagaauggaaagu
.........................................................cuAacuaccuugucuuuga.....................................   1   1   He1
.........................................................cucacuaccuugucuuuga.....................................   2   0   He1
.........................................................cucacuaccuugucuuugac....................................   4   0   He1
.........................................................cucacuaccuuUucuuugacg...................................   10  1   He1
.........................................................cucacuaccCugucuuugacg...................................   1   1   He1
.........................................................cucacuaccuugucuuugCcg...................................   1   1   He1
.........................................................cucacuaAcuugucuuugacg...................................   1   1   He1
.........................................................cucUcuaccuugucuuugacg...................................   2   1   He1
.........................................................cucaGuaccuugucuuugacg...................................   7   1   He1
.........................................................cucacuaccuugucuuugacg...................................   260 0   He1
.........................................................cucacuaccuugucuuugUcg...................................   1   1   He1
.........................................................cuAacuaccuugucuuugacg...................................   14  1   He1
.........................................................cucacuaccuugCcuuugacg...................................   1   1   He1
.........................................................cAcacuaccuugucuuugacg...................................   1   1   He1
.........................................................cucacuaccuugucuuugacU...................................   1   1   He1
.........................................................cucacuaccuuAucuuugacg...................................   1   1   He1
.........................................................cucacuaccuugucuuugacA...................................   11  1   He1
.........................................................cucacuaccuuguAuuugacg...................................   1   1   He1
.........................................................cucacuacGuugucuuugacg...................................   1   1   He1
.........................................................cucacuaccuugucuuugacC...................................   3   1   He1
.........................................................Aucacuaccuugucuuugacg...................................   9   1   He1
.........................................................cucacuacAuugucuuugacg...................................   5   1   He1
.........................................................cucacuGccuugucuuugacg...................................   1   1   He1
.........................................................cucaUuaccuugucuuugacg...................................   12  1   He1
.........................................................cucacuaccuugucuuugGcg...................................   1   1   He1
.........................................................cucacuaccuugucuuugacgU..................................   9   1   He1
.........................................................cucacuaccuugucuuugacgC..................................   1   1   He1
..........................................................ucacuaccuugucuuuga.....................................   1   0   He1
..........................................................ucacuaccuugucuuugac....................................   1   0   He1
..........................................................ucacuaccuugucuuugacg...................................   34  0   He1
..........................................................ucacuaccuugucCuugacg...................................   2   1   He1
..........................................................ucCcuaccuugucuuugacg...................................   1   1   He1
..........................................................ucacuaccuugucuuugacA...................................   1   1   He1
..........................................................uUacuaccuugucuuugacg...................................   1   1   He1
..........................................................ucacAaccuugucuuugacg...................................   1   1   He1
..........................................................ucacGaccuugucuuugacg...................................   3   1   He1
..........................................................ucacuaccuugucuuugacgU..................................   3   1   He1
...........................................................cacuaccuugucuuugacg...................................   6   0   He1
............................................................acuaccuugucCuugacg...................................   1   1   He1

................Aaugaaagacauggguagugaga..........................................................................   1   1   Bl1
................Aaugaaagacauggguagugagaug........................................................................   9   1   Bl1
.................augaaagacauggguagu..............................................................................   2   0   Bl1
.................augaaagacauggguagug.............................................................................   3   0   Bl1
.................augaaagacauggguaguga............................................................................   1   0   Bl1
.................auCaaagacauggguagugag...........................................................................   1   1   Bl1
.................Cugaaagacauggguagugaga..........................................................................   4   1   Bl1
.................augaaagacauggguagugaga..........................................................................   9   0   Bl1
.................Cugaaagacauggguagugagau.........................................................................   1   1   Bl1
.................augaaagacauggguagugagau.........................................................................   14  0   Bl1
.................augUaagacauggguagugagaug........................................................................   1   1   Bl1
.................auCaaagacauggguagugagaug........................................................................   1   1   Bl1
.................augaGagacauggguagugagaug........................................................................   1   1   Bl1
.................augaaagacauggguagugagaug........................................................................   51  0   Bl1
.................augaaagacauggguagugagauU........................................................................   1   1   Bl1
.................Cugaaagacauggguagugagaug........................................................................   35  1   Bl1
.................augaaagacauggguagugagaugu.......................................................................   1   0   Bl1
..................ugaaagacauggguGgug.............................................................................   1   1   Bl1
..................ugaaagacUuggguagug.............................................................................   3   1   Bl1
..................ugaaGgacauggguagug.............................................................................   1   1   Bl1
..................ugaaagacaugUguagug.............................................................................   1   1   Bl1
..................ugaaUgacauggguagug.............................................................................   5   1   Bl1
..................ugaaaUacauggguagug.............................................................................   1   1   Bl1
..................ugaaagacauggguaguA.............................................................................   8   1   Bl1
..................ugaaagacCuggguagug.............................................................................   1   1   Bl1
..................ugaaagacauggguagug.............................................................................   338 0   Bl1
..................ugaaagacauggguagugU............................................................................   3   1   Bl1
..................ugaaagacauggguaguUa............................................................................   1   1   Bl1
..................Agaaagacauggguaguga............................................................................   1   1   Bl1
..................ugaaGgacauggguaguga............................................................................   1   1   Bl1
```

```
                  Mature                  Star
aauaaacauuuuuucccaugaaagacauggguagugagauguggcuaagcuagucaucucacuaccuugucuuugacggaaagaauuguaaaagucaguacagaauggaaagu
..................ugaaUgacauggguaguga............................................................................    1     1   Bl1
..................Cgaaagacauggguaguga............................................................................    1     1   Bl1
..................ugaaagacauggguaguga............................................................................    269   0   Bl1
..................ugaaagacaAggguaguga............................................................................    1     1   Bl1
..................ugaaagacauggguaguAa............................................................................    3     1   Bl1
..................ugaUagacauggguaguga............................................................................    1     1   Bl1
..................ugaaagacaugUguaguga............................................................................    2     1   Bl1
..................ugaaagacCuggguaguga............................................................................    6     1   Bl1
..................ugaaagacauAgguaguga............................................................................    1     1   Bl1
..................ugaaagacauggguagugaC...........................................................................    2     1   Bl1
..................Agaaagacauggguagugag...........................................................................    1     1   Bl1
..................ugaaagacauggguagugaU...........................................................................    1     1   Bl1
..................ugCaagacauggguagugag...........................................................................    1     1   Bl1
..................ugaaUgacauggguagugag...........................................................................    5     1   Bl1
..................ugaaagacauggguagugag...........................................................................    575   0   Bl1
..................ugaaagacauggguaguAag...........................................................................    1     1   Bl1
..................ugaaagacUuggguagugag...........................................................................    2     1   Bl1
..................ugaaagacaugUguagugag...........................................................................    1     1   Bl1
..................ugaaagacauggguGgugag...........................................................................    1     1   Bl1
..................ugaaagacauggguagugaA...........................................................................    47    1   Bl1
..................ugaaagUcauggguagugag...........................................................................    2     1   Bl1
..................Ngaaagacauggguagugag...........................................................................    1     1   Bl1
..................ugaaGgacauggguagugag...........................................................................    5     1   Bl1
..................ugaaagacCuggguagugag...........................................................................    7     1   Bl1
..................ugaGagacauggguagugaga..........................................................................    1     1   Bl1
..................ugaaagacauggCuagugaga..........................................................................    5     1   Bl1
..................uCaaagacauggguagugaga..........................................................................    3     1   Bl1
..................ugaaaAacauggguagugaga..........................................................................    2     1   Bl1
..................ugaaagacauggguaguUaga..........................................................................    4     1   Bl1
..................ugaaagacauAgguagugaga..........................................................................    3     1   Bl1
..................ugaaagacaCggguagugaga..........................................................................    2     1   Bl1
..................ugaaagacauggguagAgaga..........................................................................    1     1   Bl1
..................uAaaagacauggguagugaga..........................................................................    2     1   Bl1
..................ugaaagacaAggguagugaga..........................................................................    2     1   Bl1
..................Cgaaagacauggguagugaga..........................................................................    10    1   Bl1
..................ugaaagGcauggguagugaga..........................................................................    1     1   Bl1
..................ugaaagacaugggAagugaga..........................................................................    1     1   Bl1
..................ugaaCgacauggguagugaga..........................................................................    1     1   Bl1
..................ugaaagacauggguagugaga..........................................................................    5760  0   Bl1
..................ugaaagacauggguagugagC..........................................................................    5     1   Bl1
..................ugaaUgacauggguagugaga..........................................................................    76    1   Bl1
..................ugaaagacaugggCagugaga..........................................................................    3     1   Bl1
..................ugaaagacUuggguagugaga..........................................................................    5     1   Bl1
..................ugaaagacauggguaUugaga..........................................................................    3     1   Bl1
..................ugaaagacauggguagugagG..........................................................................    3     1   Bl1
..................ugaaaUacauggguagugaga..........................................................................    3     1   Bl1
..................Ngaaagacauggguagugaga..........................................................................    6     1   Bl1
..................ugaaagacaGggguagugaga..........................................................................    1     1   Bl1
..................ugaaagacauggguagugagU..........................................................................    32    1   Bl1
..................ugaaagacauggguagugCga..........................................................................    1     1   Bl1
..................ugCaagacauggguagugaga..........................................................................    14    1   Bl1
..................uUaaagacauggguagugaga..........................................................................    1     1   Bl1
..................ugaaagacauggguagugaAa..........................................................................    55    1   Bl1
..................ugaUagacauggguagugaga..........................................................................    20    1   Bl1
..................ugaaagacCuggguagugaga..........................................................................    70    1   Bl1
..................ugaaagacaugUguagugaga..........................................................................    12    1   Bl1
..................ugaaagacauggguGgugaga..........................................................................    2     1   Bl1
..................ugaaGgacauggguagugaga..........................................................................    45    1   Bl1
..................Agaaagacauggguagugaga..........................................................................    13    1   Bl1
..................ugaaagacauggguagugaUa..........................................................................    11    1   Bl1
..................ugaaagacauggguagugUga..........................................................................    2     1   Bl1
..................ugaaagaUauggguagugaga..........................................................................    11    1   Bl1
..................ugaaagacauggguagugaCa..........................................................................    12    1   Bl1
..................Ggaaagacauggguagugaga..........................................................................    5     1   Bl1
..................ugaaagacauggguagugaUau.........................................................................    5     1   Bl1
..................ugCaagacauggguagugagau.........................................................................    10    1   Bl1
..................ugaaagacauggguagAgagau.........................................................................    1     1   Bl1
..................Cgaaagacauggguagugagau.........................................................................    6     1   Bl1
..................ugaaagacauggguaguUagau.........................................................................    2     1   Bl1
..................ugaaagaUauggguagugagau.........................................................................    8     1   Bl1
```

```
                  Mature                                       Star
aauaaacauuuuuucccaugaaagacauggguagugagauguggcuaagcuagucaucucacuaccuugucuuugacggaaagaauuguaaaagucaguacagaauggaaagu
..................ugaaagacauggAuagugagau.........................................................................   1      1   Bl1
..................ugaaagacauggguaguCagau.........................................................................   1      1   Bl1
..................ugaaagacauggguagugagaA.........................................................................   324    1   Bl1
..................ugGaagacauggguagugagau.........................................................................   1      1   Bl1
..................uCaaagacauggguagugagau.........................................................................   2      1   Bl1
..................ugaaagacauAgguagugagau.........................................................................   2      1   Bl1
..................ugaaagacauggguagugagCu.........................................................................   5      1   Bl1
..................ugaaagacauggguagugagaC.........................................................................   35     1   Bl1
..................ugaaaAacauggguagugagau.........................................................................   1      1   Bl1
..................ugaaagacaugggCagugagau.........................................................................   2      1   Bl1
..................uAaaagacauggguagugagau.........................................................................   1      1   Bl1
..................ugaaagacauggguagugagGu.........................................................................   1      1   Bl1
..................ugaaagacauggUuagugagau.........................................................................   1      1   Bl1
..................ugaaUgacauggguagugagau.........................................................................   54     1   Bl1
..................ugaaagacauggguagugagUu.........................................................................   2      1   Bl1
..................ugaGagacauggguagugagau.........................................................................   1      1   Bl1
..................ugaaagacUuggguagugagau.........................................................................   7      1   Bl1
..................Ggaaagacauggguagugagau.........................................................................   1      1   Bl1
..................ugaUagacauggguagugagau.........................................................................   17     1   Bl1
..................ugaaagacCuggguagugagau.........................................................................   70     1   Bl1
..................ugaaagacaugggu Cgugagau........................................................................   1      1   Bl1
..................ugaaagacaGgguagugagau..........................................................................   1      1   Bl1
..................Agaaagacauggguagugagau.........................................................................   6      1   Bl1
..................ugaaagacauggguaUugagau.........................................................................   1      1   Bl1
..................ugaaaUacauggguagugagau.........................................................................   1      1   Bl1
..................ugaaaCacauggguagugagau.........................................................................   1      1   Bl1
..................ugaaagacauggguagugGgau.........................................................................   2      1   Bl1
..................ugaaagacaAgguagugagau..........................................................................   1      1   Bl1
..................ugaaGgacauggguagugagau.........................................................................   40     1   Bl1
..................ugaaagacauggguaguAagau.........................................................................   1      1   Bl1
..................ugaaagacaugUguagugagau.........................................................................   15     1   Bl1
..................Ngaaagacauggguagugagau.........................................................................   7      1   Bl1
..................ugaaagacauggguagugagau.........................................................................   5142   0   Bl1
..................ugaaagacaugggAagugagau.........................................................................   1      1   Bl1
..................ugaaagacaugggu Ggugagau........................................................................   1      1   Bl1
..................ugaaagUcauggguagugagaug........................................................................   20     1   Bl1
..................ugaaagacauggUuagugagaug........................................................................   16     1   Bl1
..................ugaaagacauggguagugagaug........................................................................   61766  0   Bl1
..................ugCaagacauggguagugagaug........................................................................   102    1   Bl1
..................ugaaagacauggguaguCagaug........................................................................   2      1   Bl1
..................ugaaagacauggguagugagCug........................................................................   50     1   Bl1
..................ugaaagacauggguagugagaAg........................................................................   10     1   Bl1
..................ugaaUgacauggguagugagaug........................................................................   480    1   Bl1
..................ugaaagacaugUguagugagaug........................................................................   121    1   Bl1
..................ugaaagacaugggCagugagaug........................................................................   13     1   Bl1
..................ugaaaUacauggguagugagaug........................................................................   22     1   Bl1
..................ugaGagacauggguagugagaug........................................................................   8      1   Bl1
..................ugaaaCacauggguagugagaug........................................................................   3      1   Bl1
..................ugaaagacauggguagugaUaug........................................................................   119    1   Bl1
..................ugaaagacNuggguagugagaug........................................................................   1      1   Bl1
..................uCaaagacauggguagugagaug........................................................................   18     1   Bl1
..................ugaaagacaCgguagugagaug.........................................................................   6      1   Bl1
..................ugaaagacaGgguagugagaug.........................................................................   1      1   Bl1
..................ugaaagacauggguagugagGug........................................................................   10     1   Bl1
..................ugaaagacauggguaguAagaug........................................................................   3      1   Bl1
..................ugaaGgacauggguagugagaug........................................................................   352    1   Bl1
..................uAaaagacauggguagugagaug........................................................................   33     1   Bl1
..................ugaaCgacauggguagugagaug........................................................................   1      1   Bl1
..................ugaaagacaugggu Cgugagaug.......................................................................   3      1   Bl1
..................ugaaagacaugAguagugagaug........................................................................   7      1   Bl1
..................ugaaagacauggguagugagUug........................................................................   21     1   Bl1
..................ugaaagacaugggGagugagaug........................................................................   4      1   Bl1
..................Cgaaagacauggguagugagaug........................................................................   99     1   Bl1
..................ugaaagacauggguagugGgaug........................................................................   11     1   Bl1
..................ugaaagaAauggguagugagaug........................................................................   3      1   Bl1
..................ugaaagacauggguagAgagaug........................................................................   13     1   Bl1
..................ugaaagacauggguagugaAaug........................................................................   6      1   Bl1
..................ugaaagacauAgguagugagaug........................................................................   20     1   Bl1
..................ugUaagacauggguagugagaug........................................................................   8      1   Bl1
..................ugaaagGcauggguagugagaug........................................................................   9      1   Bl1
```

Mature **Star**

aauaaacauuuuuucccaugaaagacauggguagugagauguggcuaagcuagucaucucacuaccuugucuuugacggaaagaauuguaaaagucaguacagaauggaaagu			
..................ugaaagacauggguagugagauC..	39	1	Bl1
..................ugaaagacauUgguagugagaug..	11	1	Bl1
..................ugaaagacauggguaAugagaug..	3	1	Bl1
..................ugaaagacauggguaguUagaug..	37	1	Bl1
..................ugaaagacauggguagugagauA..	549	1	Bl1
..................ugaaagacauggguagugaCaug..	7	1	Bl1
..................ugaaagacCuggguagugagaug..	705	1	Bl1
..................ugaaagCcauggguagugagaug..	5	1	Bl1
..................ugaaagacauggguaCugagaug..	2	1	Bl1
..................uUaaagacauggguagugagaug..	13	1	Bl1
..................ugaaagacauggguagugagauU..	693	1	Bl1
..................ugaaagacauggguagugagaCg..	10	1	Bl1
..................ugaaagacUuggguagugagaug..	91	1	Bl1
..................ugaaagacauggguagugCgaug..	1	1	Bl1
..................ugaaagacaugggguGgugagaug...	31	1	Bl1
..................Ngaaagacauggguagugagaug..	43	1	Bl1
..................ugaaagacauCgguagugagaug..	6	1	Bl1
..................ugaaagacaAggguagugagaug..	8	1	Bl1
..................ugaaagacauggguaUugagaug..	21	1	Bl1
..................ugaaaAacauggguagugagaug..	4	1	Bl1
..................ugaaagacauggguagGgagaug..	1	1	Bl1
..................ugGaagacauggguagugagaug..	11	1	Bl1
..................ugaaagacaugggAagugagaug..	15	1	Bl1
..................ugaaagacauggguagCgagaug..	5	1	Bl1
..................ugaaagacaugggguUgugagaug...	18	1	Bl1
..................ugaaagacauggguagugUgaug..	13	1	Bl1
..................ugaUagacauggguagugagaug..	88	1	Bl1
..................ugaaagaUauggguagugagaug..	140	1	Bl1
..................ugaaagacaugCguagugagaug..	5	1	Bl1
..................Ggaaagacauggguagugagaug..	12	1	Bl1
..................Agaaagacauggguagugagaug..	117	1	Bl1
..................ugaaagacauggAuagugagaug..	7	1	Bl1
..................ugaaagacauggguagugagaugu...	526	0	Bl1
..................ugaaagacaugUguagugagaugu...	1	1	Bl1
..................ugaaagacauggguagugagaugG...	6	1	Bl1
..................ugaaaAacauggguagugagaugu...	1	1	Bl1
..................ugaaagacaCggguagugagaugu...	1	1	Bl1
..................ugaUagacauggguagugagaugu...	1	1	Bl1
..................ugaaagacauggguagugagauAu...	3	1	Bl1
..................ugaaGgacauggguagugagaugu...	1	1	Bl1
..................ugaaagacauggguagugagaugC...	16	1	Bl1
..................ugaaagacUuggguagugagaugu...	2	1	Bl1
..................ugaaagacauggguagugaUaugu...	2	1	Bl1
..................ugaaagacauggguagugagauCu...	1	1	Bl1
..................ugaaagacaugggguGgugagaugu..	1	1	Bl1
..................ugaaagCcauggguagugagaugu...	1	1	Bl1
..................Cgaaagacauggguagugagaugu...	1	1	Bl1
..................ugUaagacauggguagugagaugu...	1	1	Bl1
..................ugaaagacCuggguagugagaugu...	7	1	Bl1
..................ugaaagacauggguagugagauUu...	19	1	Bl1
..................ugaaUgacauggguagugagaugu...	4	1	Bl1
..................ugaaagacauggguagugagauguA..	22	1	Bl1
..................ugaaagacauggguagugagaugAg..	3	1	Bl1
..................ugaaagacauggguagugagaugug..	1	0	Bl1
...................gaaagacauggguaguga..	1	0	Bl1
...................gaaagacaAggguagugag...	1	1	Bl1
...................gCaagacauggguagugag...	1	1	Bl1
...................gaaaUacauggguagugag...	1	1	Bl1
...................gaaagacauggguagugag...	1	0	Bl1
...................gaaaUacauggguagugaga..	1	1	Bl1
...................gaCagacauggguagugaga..	1	1	Bl1
...................gaaUgacauggguagugaga..	1	1	Bl1
...................gaaagacauggguagugaCa..	1	1	Bl1
...................gaaagacauggguagugaga..	23	0	Bl1
...................Uaaagacauggguagugaga..	1	1	Bl1
...................gaaagacaugggguCgugaga...	1	1	Bl1
...................gCaagacauggguagugaga..	1	1	Bl1
...................gaaaUacauggguagugagau...	3	1	Bl1
...................gaaaCacauggguagugagau...	1	1	Bl1
...................gaaagacauggguagugagau...	19	0	Bl1

Mature **Star**

```
aauaaacauuuuuucccaugaaagacauggguagugagauguggcuaagcuagucaucucacuaccuugucuuugacggaaagaauuguaaaagucaguacagaauggaaagu
...................gaCagacauggguagugagau.........................................................................   1      1      Bl1
...................gaaagacauggguagugUgau.........................................................................   1      1      Bl1
...................gaaagacauggguagugaAau.........................................................................   1      1      Bl1
...................gaaagacauggguCgugagaug........................................................................   4      1      Bl1
...................gaaagacauggguagugagauU........................................................................   3      1      Bl1
...................gaaagacauggguagugaAaug........................................................................   1      1      Bl1
...................gaaagUcauggguagugagaug........................................................................   1      1      Bl1
...................gaaagacauggguagugagaug........................................................................   180    0      Bl1
...................Caaagacauggguagugagaug........................................................................   1      1      Bl1
...................gaaagacaCggguagugagaug........................................................................   1      1      Bl1
...................gaaagacauggguagugagGug........................................................................   1      1      Bl1
...................gaaUgacauggguagugagaug........................................................................   3      1      Bl1
...................gCaagacauggguagugagaug........................................................................   1      1      Bl1
...................gaaagacauggguaUugagaug........................................................................   1      1      Bl1
...................gaaagacauggguagugaCaug........................................................................   1      1      Bl1
...................Aaaagacauggguagugagaug........................................................................   1      1      Bl1
...................gaaagacUuggguagugagaug........................................................................   1      1      Bl1
...................gaaagacauggguagugaUaug........................................................................   5      1      Bl1
...................gaaagacaugggAagugagaug........................................................................   2      1      Bl1
...................gaaagacauggAuagugagaug........................................................................   1      1      Bl1
...................gaaaUacauggguagugagaug........................................................................   24     1      Bl1
...................gaaaCacauggguagugagaug........................................................................   1      1      Bl1
...................gaaagacaAggguagugagaug........................................................................   5      1      Bl1
...................gaCagacauggguagugagaug........................................................................   4      1      Bl1
...................gaaagacauggguagugagauA........................................................................   1      1      Bl1
...................gaaagacaGggguagugagaug........................................................................   1      1      Bl1
...................gCaagacauggguagugagaugu.......................................................................   1      1      Bl1
...................gaaaUacauggguagugagaugu.......................................................................   1      1      Bl1
...................gaaagacauggguagugagaugu.......................................................................   2      0      Bl1
...................gaaagacauggguagugagaugC.......................................................................   1      1      Bl1
....................aaagacauggguagugagau.........................................................................   1      0      Bl1
....................aaagacauggguagugagauA........................................................................   2      1      Bl1
....................aaagacauggguagugagaug........................................................................   6      0      Bl1
.....................aagacauggguagugagau.........................................................................   1      0      Bl1
.....................aagacauggguagugagauU........................................................................   2      1      Bl1
.....................aagacauggguagugagaug........................................................................   4      0      Bl1
......................agacauggguagugagauA........................................................................   1      1      Bl1
......................agacauggguagugagaug........................................................................   3      0      Bl1
......................................................caucucacuaccuugucuuuga.....................................   1      0      Bl1
......................................................caucucacuaccuugucuuugac....................................   1      0      Bl1
.......................................................aucuGacuaccuugucuuug......................................   1      1      Bl1
.......................................................aucucacuaccuugucuuuga.....................................   4      0      Bl1
.......................................................Cucucacuaccuugucuuugacg...................................   2      1      Bl1
........................................................ucucacuaccuugucuuu.......................................   3      0      Bl1
........................................................ucucGcuaccuugucuuug......................................   2      1      Bl1
........................................................ucucacuaccuugucuuug......................................   24     0      Bl1
........................................................ucucacuaccuugucAuug......................................   1      1      Bl1
........................................................ucucacuaccuGgucuuuga.....................................   1      1      Bl1
........................................................ucucacuaccuugucuCuga.....................................   1      1      Bl1
........................................................ucucUcuaccuugucuuuga.....................................   1      1      Bl1
........................................................ucucacuaccuugucuuuga.....................................   52     0      Bl1
........................................................uUucacuaccuugucuuugac....................................   1      1      Bl1
........................................................ucucacuaccuugucuuugac....................................   92     0      Bl1
........................................................ucucacuaccuugucuuugaU....................................   1      1      Bl1
........................................................ucucGcuaccuugucuuugac....................................   1      1      Bl1
........................................................ucucacuaAcuugucuuugac....................................   1      1      Bl1
........................................................ucuUacuaccuugucuuugac....................................   1      1      Bl1
........................................................ucucacuaccuugCcuuugac....................................   1      1      Bl1
........................................................uAucacuaccuugucuuugacg...................................   2      1      Bl1
........................................................Acucacuaccuugucuuugacg...................................   2      1      Bl1
........................................................uUucacuaccuugucuuugacg...................................   3      1      Bl1
........................................................ucucacuaccuuguGuuugacg...................................   2      1      Bl1
........................................................Ccucacuaccuugucuuugacg...................................   4      1      Bl1
........................................................ucucacuaccuugucuuugacC...................................   2      1      Bl1
........................................................ucucacuaccuugucuuuUacg...................................   7      1      Bl1
........................................................ucucaUuaccuugucuuugacg...................................   3      1      Bl1
........................................................ucucacuCccuugucuuugacg...................................   1      1      Bl1
........................................................ucucacuaccuugucuuugacg...................................   2089   0      Bl1
........................................................ucucacuaccuugucGuugacg...................................   2      1      Bl1
........................................................ucucacuaccuuguUuuugacg...................................   1      1      Bl1
```

```
                  Mature                              Star
aauaaacauuuuuucccaugaaagacauggguagugagauguggcuaagcuagucaucucacuaccuugucuuugacggaaagaauuguaaaagucaguacagaauggaaagu
........................................................ucucGcuaccuugucuuugacg...................................   19    1   Bl1
........................................................ucucacuaccuugucuuugacU...................................   13    1   Bl1
........................................................Ncucacuaccuugucuuugacg...................................   1     1   Bl1
........................................................ucucacuUccuugucuuugacg...................................   2     1   Bl1
........................................................ucucacuaccuugucuuugacA...................................   177   1   Bl1
........................................................ucucacuaccAugucuuugacg...................................   4     1   Bl1
........................................................ucucacuaccuugCcuuugacg...................................   1     1   Bl1
........................................................ucucacuaccCugucuuugacg...................................   1     1   Bl1
........................................................ucucacuaccuuUucuuugacg...................................   1     1   Bl1
........................................................ucCcacuaccuugucuuugacg...................................   6     1   Bl1
........................................................ucucacuaccuugucuuugaUg...................................   1     1   Bl1
........................................................ucucacuacAuugucuuugacg...................................   5     1   Bl1
........................................................uGucacuaccuugucuuugacg...................................   3     1   Bl1
........................................................ucucacuaAcuugucuuugacg...................................   13    1   Bl1
........................................................ucucacuacUuugucuuugacg...................................   1     1   Bl1
........................................................ucuUacuaccuugucuuugacg...................................   8     1   Bl1
........................................................ucucUcuaccuugucuuugacg...................................   16    1   Bl1
........................................................ucucacuGccuugucuuugacg...................................   1     1   Bl1
........................................................ucucacuaccuugucuuugacgU..................................   117   1   Bl1
........................................................ucucacuaccuugucuuugacgC..................................   4     1   Bl1
........................................................ucucacuaccuugucuuugacgA..................................   6     1   Bl1
........................................................ucucacuaccuugucuuugacgUa.................................   6     1   Bl1
........................................................ucucacuaccuugucuuugacgAa.................................   1     1   Bl1
.........................................................cucacuaccuugucuuuga.....................................   1     0   Bl1
.........................................................cucacuaccuugucuuuUac....................................   1     1   Bl1
.........................................................cucacuaccuuguUuuugacg...................................   1     1   Bl1
.........................................................cucacuaccuugucuuugacg...................................   35    0   Bl1
.........................................................cucUcuaccuugucuuugacg...................................   1     1   Bl1
.........................................................Aucacuaccuugucuuugacg...................................   2     1   Bl1
.........................................................cucacuaccuugucuuugacA...................................   2     1   Bl1
.........................................................cucacuaccuugucuuugacU...................................   1     1   Bl1
.........................................................cuAacuaccuugucuuugacg...................................   1     1   Bl1
.........................................................cucaUuaccuugucuuugacg...................................   1     1   Bl1
.........................................................cucacuaccuugucuuuUacg...................................   1     1   Bl1
.........................................................cucacuaccuugucuuugacgU..................................   4     1   Bl1
..........................................................ucacuaccuugucuuugacg...................................   5     0   Bl1
..........................................................ucacuaccuugucuuugacgga.................................   1     0   Bl1

................Aaugaaagacauggguagugagaug........................................................................   2     1   Ch1
.................augaaagacauggguagug.............................................................................   2     0   Ch1
.................augaaagacauggguaguga............................................................................   1     0   Ch1
.................augaaagacauggguagugag...........................................................................   1     0   Ch1
.................augaaagacauggguagugaga..........................................................................   2     0   Ch1
.................augaaagaAauggguagugaga..........................................................................   2     1   Ch1
.................augaUagacauggguagugaga..........................................................................   1     1   Ch1
.................Cugaaagacauggguagugaga..........................................................................   2     1   Ch1
.................augaUagacauggguagugagau.........................................................................   1     1   Ch1
.................augaaagacauggguagugagau.........................................................................   7     0   Ch1
.................augaaagacauggguagugagauA........................................................................   1     1   Ch1
.................Cugaaagacauggguagugagaug........................................................................   1     1   Ch1
.................augaGagacauggguagugagaug........................................................................   1     1   Ch1
.................augaaagacauggguagugagaug........................................................................   29    0   Ch1
.................augaUagacauggguagugagaug........................................................................   1     1   Ch1
..................ugaaagacCuggguagug.............................................................................   2     1   Ch1
..................ugaaGgacauggguagug.............................................................................   1     1   Ch1
..................ugaaagacauggguaguA.............................................................................   9     1   Ch1
..................Agaaagacauggguagug.............................................................................   2     1   Ch1
..................ugaaagacauggguagug.............................................................................   220   0   Ch1
..................uCaaagacauggguaguga............................................................................   1     1   Ch1
..................Cgaaagacauggguaguga............................................................................   1     1   Ch1
..................ugaaagacCuggguaguga............................................................................   3     1   Ch1
..................ugaaagacauggguagugU............................................................................   5     1   Ch1
..................ugCaagacauggguaguga............................................................................   1     1   Ch1
..................ugaaGgacauggguaguga............................................................................   1     1   Ch1
..................ugaaagacauggguaguga............................................................................   177   0   Ch1
..................ugaaagacauggguaguAa............................................................................   1     1   Ch1
..................ugaaUgacauggguaguga............................................................................   1     1   Ch1
..................ugaaagacauggguagugag...........................................................................   395   0   Ch1
..................ugaaaAacauggguagugag...........................................................................   1     1   Ch1
..................ugaaagacauggguagugaA...........................................................................   21    1   Ch1
```

```
                  Mature                        Star
aauaaacauuuuuucccaugaaagacauggguagugagauguggcuaagcuagucaucucacuaccuugucuuugacggaaagaauuguaaaagucaguacagaauggaaagu
..................ugaaagacCuggguagugag...........................................................................  6     1   Ch1
..................ugaaagacauggguagugaC...........................................................................  1     1   Ch1
..................Agaaagacauggguagugag...........................................................................  3     1   Ch1
..................ugaaUgacauggguagugag...........................................................................  7     1   Ch1
..................Ngaaagacauggguagugag...........................................................................  1     1   Ch1
..................ugaaagacaugCguagugag...........................................................................  1     1   Ch1
..................ugaaagacauggguagugaU...........................................................................  1     1   Ch1
..................ugCaagacauggguagugag...........................................................................  2     1   Ch1
..................ugaaGgacauggguagugag...........................................................................  4     1   Ch1
..................ugaaagaUauggguagugag...........................................................................  1     1   Ch1
..................ugaaagacaugUguagugag...........................................................................  2     1   Ch1
..................ugaaagacauggguGgugaga..........................................................................  3     1   Ch1
..................ugaaagacauggCuagugaga..........................................................................  1     1   Ch1
..................ugaaagacauggUuagugaga..........................................................................  1     1   Ch1
..................ugaaagacauggguagugagU..........................................................................  26    1   Ch1
..................ugaaagacauggguaUugaga..........................................................................  2     1   Ch1
..................ugaaagacauggguCgugaga..........................................................................  1     1   Ch1
..................ugaaagGcauggguagugaga..........................................................................  1     1   Ch1
..................ugaUagacauggguagugaga..........................................................................  7     1   Ch1
..................ugaaagacauggguagugagC..........................................................................  12    1   Ch1
..................ugaaagacauggguagugaCa..........................................................................  5     1   Ch1
..................ugaaagaUauggguagugaga..........................................................................  2     1   Ch1
..................Agaaagacauggguagugaga..........................................................................  6     1   Ch1
..................ugaaagacauggguagugagG..........................................................................  3     1   Ch1
..................ugaaagacauggguagugGga..........................................................................  1     1   Ch1
..................ugaaagacCuggguagugaga..........................................................................  33    1   Ch1
..................uCaaagacauggguagugaga..........................................................................  1     1   Ch1
..................ugaaagacaugAguagugaga..........................................................................  1     1   Ch1
..................ugaaUgacauggguagugaga..........................................................................  32    1   Ch1
..................ugaaagacaugggCagugaga..........................................................................  2     1   Ch1
..................ugUaagacauggguagugaga..........................................................................  1     1   Ch1
..................Cgaaagacauggguagugaga..........................................................................  6     1   Ch1
..................ugaaagacaugggAagugaga..........................................................................  1     1   Ch1
..................ugaaagacaugUguagugaga..........................................................................  6     1   Ch1
..................ugCaagacauggguagugaga..........................................................................  9     1   Ch1
..................ugaaGgacauggguagugaga..........................................................................  20    1   Ch1
..................uUaaagacauggguagugaga..........................................................................  2     1   Ch1
..................ugaaagacauggguagugaUa..........................................................................  3     1   Ch1
..................ugaaagacauggguagugaga..........................................................................  2486  0   Ch1
..................ugaaaUacauggguagugaga..........................................................................  2     1   Ch1
..................ugaaagacauggguagugaAa..........................................................................  15    1   Ch1
..................ugaaagacUuggguagugaga..........................................................................  4     1   Ch1
..................ugaaagacauggguaguUagau.........................................................................  1     1   Ch1
..................ugaaagacUuggguagugagau.........................................................................  2     1   Ch1
..................Cgaaagacauggguagugagau.........................................................................  1     1   Ch1
..................ugCaagacauggguagugagau.........................................................................  3     1   Ch1
..................ugaaGgacauggguagugagau.........................................................................  22    1   Ch1
..................ugaaagaUauggguagugagau.........................................................................  4     1   Ch1
..................ugaaagacauAgguagugagau.........................................................................  1     1   Ch1
..................ugaUagacauggguagugagau.........................................................................  6     1   Ch1
..................ugaaagacauCgguagugagau.........................................................................  6     1   Ch1
..................ugaaagacaugggAagugagau.........................................................................  3     1   Ch1
..................ugaaagacauggguagugagGu.........................................................................  1     1   Ch1
..................Agaaagacauggguagugagau.........................................................................  4     1   Ch1
..................ugaaagacaCggguagugagau.........................................................................  1     1   Ch1
..................ugaaagacauggguagugagaA.........................................................................  89    1   Ch1
..................ugaaagacCuggguagugagau.........................................................................  20    1   Ch1
..................ugaaagacauggguagugaUau.........................................................................  2     1   Ch1
..................ugaaagacauggguUgugagau.........................................................................  2     1   Ch1
..................ugaaagacauggguGgugagau.........................................................................  1     1   Ch1
..................ugaaagacauggguagugagaC.........................................................................  21    1   Ch1
..................ugaaagacaugUguagugagau.........................................................................  5     1   Ch1
..................ugaaagacauggguagugagau.........................................................................  2081  0   Ch1
..................ugaaUgacauggguagugagau.........................................................................  34    1   Ch1
..................ugaaagacauggAuagugagau.........................................................................  1     1   Ch1
..................ugaaagacauggguaguCagau.........................................................................  1     1   Ch1
..................ugaaaUacauggguagugagau.........................................................................  3     1   Ch1
..................ugaaagacauUgguagugagau.........................................................................  1     1   Ch1
..................ugaaagacauggguagugagCu.........................................................................  3     1   Ch1
..................Ngaaagacauggguagugagau.........................................................................  2     1   Ch1
```

```
                  Mature                Star
aauaaacauuuuuucccaugaaagacauggguagugagauguggcuaagcuagucaucucacuaccuugucuuugacggaaagaauuguaaaagucaguacagaauggaaagu
..................ugaaagacaugggu Cgugagau.........................................................................	1	1	Ch1
..................ugaaagacaugAguagugagau.........................................................................	3	1	Ch1
..................ugaaagacUuggguagugagaug........................................................................	11	1	Ch1
..................ugaaagacaugCguagugagaug........................................................................	5	1	Ch1
..................ugaaagaUauggguagugagaug........................................................................	28	1	Ch1
..................Agaaagacauggguagugagaug........................................................................	35	1	Ch1
..................ugaaagacauggguagugagGug........................................................................	3	1	Ch1
..................ugaaagacauggguagAgagaug........................................................................	3	1	Ch1
..................Cgaaagacauggguagugagaug........................................................................	11	1	Ch1
..................ugaaagacauggCuagugagaug........................................................................	4	1	Ch1
..................ugaaaAacauggguagugagaug........................................................................	2	1	Ch1
..................ugaaagacauggguagugagaCg........................................................................	7	1	Ch1
..................Ngaaagacauggguagugagaug........................................................................	12	1	Ch1
..................ugaaagacauUgguagugagaug........................................................................	3	1	Ch1
..................uCaaagacauggguagugagaug........................................................................	1	1	Ch1
..................ugaaagacauggguagugagauC........................................................................	9	1	Ch1
..................ugaaagaAauggguagugagaug........................................................................	1	1	Ch1
..................ugaaagacaCggguagugagaug........................................................................	2	1	Ch1
..................ugaaagacaugggAagugagaug........................................................................	3	1	Ch1
..................ugaaagacauAgguagugagaug........................................................................	2	1	Ch1
..................ugaaagacauggguagugagCug........................................................................	6	1	Ch1
..................ugaaagacauggguaguAagaug........................................................................	2	1	Ch1
..................ugaaagGcauggguagugagaug........................................................................	1	1	Ch1
..................ugaaagacaugggu Ggugagaug........................................................................	3	1	Ch1
..................ugaaUgacauggguagugagaug........................................................................	119	1	Ch1
..................ugaaagacauggguaguUagaug........................................................................	3	1	Ch1
..................ugaaagacauggguagGgagaug........................................................................	1	1	Ch1
..................ugCaagacauggguagugagaug........................................................................	21	1	Ch1
..................ugGaagacauggguagugagaug........................................................................	1	1	Ch1
..................ugaaagacauggguagugagauU........................................................................	193	1	Ch1
..................ugaUagacauggguagugagaug........................................................................	23	1	Ch1
..................ugaaagacaAggguagugagaug........................................................................	2	1	Ch1
..................ugaaagacaugggCagugagaug........................................................................	1	1	Ch1
..................ugaaagUcauggguagugagaug........................................................................	2	1	Ch1
..................ugaaaUacauggguagugagaug........................................................................	2	1	Ch1
..................uUaaagacauggguagugagaug........................................................................	1	1	Ch1
..................uAaaagacauggguagugagaug........................................................................	5	1	Ch1
..................ugaaagacauggguagugCgaug........................................................................	3	1	Ch1
..................ugaaagacCuggguagugagaug........................................................................	113	1	Ch1
..................ugaGagacauggguagugagaug........................................................................	1	1	Ch1
..................ugaaagacauggUuagugagaug........................................................................	3	1	Ch1
..................ugaaagacauggguagugGgaug........................................................................	2	1	Ch1
..................ugaaagacaugUguagugagaug........................................................................	27	1	Ch1
..................ugaaagacauggguaAugagaug........................................................................	1	1	Ch1
..................ugaaagacauggguagugaUaug........................................................................	14	1	Ch1
..................ugaaagacaugggu Ugugagaug........................................................................	2	1	Ch1
..................ugUaagacauggguagugagaug........................................................................	2	1	Ch1
..................ugaaGgacauggguagugagaug........................................................................	72	1	Ch1
..................ugaaagacauggAuagugagaug........................................................................	1	1	Ch1
..................ugaaagacauggguagugagUug........................................................................	1	1	Ch1
..................ugaaagacauggguagugagaug........................................................................	10453	0	Ch1
..................Ggaaagacauggguagugagaug........................................................................	3	1	Ch1
..................ugaaagacauCgguagugagaug........................................................................	3	1	Ch1
..................ugaaagacauggguagugagauA........................................................................	100	1	Ch1
..................ugaaagacNuggguagugagaug........................................................................	1	1	Ch1
..................ugaaagacauggguaUugagaug........................................................................	4	1	Ch1
..................ugaaagacauggguagugagaAg........................................................................	11	1	Ch1
..................ugaaagacCuggguagugagaugu.......................................................................	2	1	Ch1
..................ugCaagacauggguagugagaugu.......................................................................	1	1	Ch1
..................ugaaagacauggguagugagauCu.......................................................................	1	1	Ch1
..................ugaaagacauggguagugagauUu.......................................................................	2	1	Ch1
..................ugaaagacauggguagugagaugu.......................................................................	133	0	Ch1
..................ugaaUgacauggguagugagaugu.......................................................................	1	1	Ch1
..................ugaaagacauggguagugagCugu.......................................................................	1	1	Ch1
..................ugaaagacauggguagugagaugC.......................................................................	7	1	Ch1
..................ugaaagacauggguagugagauguC......................................................................	1	1	Ch1
..................ugaaagacauggguagugagauguA......................................................................	2	1	Ch1
..................ugaaagacauggguagugagaugug......................................................................	1	0	Ch1
..................ugaaagacauggguagugagauguggA....................................................................	1	1	Ch1
..................ugaaagacauggguagugagauguggc....................................................................	5	0	Ch1
```

Mature Star

aauaaacauuuuuucccaugaaagacauggguagugagauguggcuaagcuagucaucucacuaccuugucuuugacggaaagaauuguaaaagucaguacagaauggaaagu			
...................gaaagacauggguagugaga..	11	0	Ch1
...................gaaagacauggguagugagU..	1	1	Ch1
...................gaaagacaAggguagugagau...	1	1	Ch1
...................gaaagacauggguagugagau...	11	0	Ch1
...................gaaagacauggguagugUgaug..	1	1	Ch1
...................gaaagacauggguCgugagaug..	4	1	Ch1
...................gaaagacaAggguagugagaug..	1	1	Ch1
...................gaCagacauggguagugagaug..	3	1	Ch1
...................gaaagacauggguUgugagaug..	1	1	Ch1
...................gaaUgacauggguagugagaug..	2	1	Ch1
...................gaaagacauggguagugagaug..	37	0	Ch1
...................gaaaUacauggguagugagaug..	4	1	Ch1
....................aaagacauggguagugag...	1	0	Ch1
....................aaagacauggguagugagaug..	4	0	Ch1
.....................aagacauggguagugaga..	1	0	Ch1
.....................aagacauggguagugagau...	2	0	Ch1
.....................aagacauggguagugagaug..	5	0	Ch1
....caucucacuaccuugucuuug......................................	2	0	Ch1
....caucucacuaccuugucuuugac....................................	1	0	Ch1
....Aaucucacuaccuugucuuugacg...................................	2	1	Ch1
....aucucacuaccuugucuuug......................................	2	0	Ch1
....aucucacuaccuugucuuuga.....................................	2	0	Ch1
....aucucacuaccuugucuuugacg...................................	1	0	Ch1
....aucucacuaccuugucuuuCacg...................................	1	1	Ch1
....Cucucacuaccuugucuuugacg...................................	4	1	Ch1
....ucucacuaccuugucuuu.......................................	5	0	Ch1
....ucucacuaccuugucuuuA......................................	1	1	Ch1
....ucucacuaccuugucuuug......................................	49	0	Ch1
....ucCcacuaccuugucuuug......................................	1	1	Ch1
....uAucacuaccuugucuuuga.....................................	1	1	Ch1
....ucucacuaccuugucuuuga.....................................	65	0	Ch1
....ucucUcuaccuugucuuugac....................................	1	1	Ch1
....ucucacuaccuugucuuugac....................................	131	0	Ch1
....ucCcacuaccuugucuuugac....................................	2	1	Ch1
....ucucGcuaccuugucuuugac....................................	1	1	Ch1
....ucucacuaccuugucCuugacg...................................	4	1	Ch1
....ucucaUuaccuugucuuugacg...................................	6	1	Ch1
....ucucacuaccuugucuuugacU...................................	28	1	Ch1
....Gcucacuaccuugucuuugacg...................................	3	1	Ch1
....ucuUacuaccuugucuuugacg...................................	34	1	Ch1
....ucucacuaccuugucuuuUacg...................................	25	1	Ch1
....ucucacuGccuugucuuugacg...................................	2	1	Ch1
....ucucacuaccuCgucuuugacg...................................	2	1	Ch1
....ucucacuaccuugucuGugacg...................................	1	1	Ch1
....ucucacuaccAugucuuugacg...................................	6	1	Ch1
....ucucacuaccuugucuuCgacg...................................	4	1	Ch1
....ucucUcuaccuugucuuugacg...................................	94	1	Ch1
....Ncucacuaccuugucuuugacg...................................	11	1	Ch1
....ucucCcuaccuugucuuugacg...................................	1	1	Ch1
....ucucacuaccuuCucuuugacg...................................	3	1	Ch1
....ucucacuaccuugucuuugacC...................................	10	1	Ch1
....ucucacuaccuugucuuugacg...................................	8172	0	Ch1
....ucucacuaNcuugucuuugacg...................................	1	1	Ch1
....ucucacuaccuuguGuuugacg...................................	3	1	Ch1
....Ccucacuaccuugucuuugacg...................................	16	1	Ch1
....ucucacuaccuuAucuuugacg...................................	1	1	Ch1
....uGucacuaccuugucuuugacg...................................	4	1	Ch1
....ucucacuaccuGgucuuugacg...................................	2	1	Ch1
....ucucacuaccuugAcuuugacg...................................	1	1	Ch1
....ucucacuacAuugucuuugacg...................................	5	1	Ch1
....ucucacuaccuugucuuugaGg...................................	1	1	Ch1
....ucucacuacUuugucuuugacg...................................	6	1	Ch1
....ucucacuaGcuugucuuugacg...................................	1	1	Ch1
....ucucacuaccuugucuuGgacg...................................	3	1	Ch1
....ucucacuUccuugucuuugacg...................................	15	1	Ch1
....ucucacuaccuugucuuugGcg...................................	7	1	Ch1
....ucCcacuaccuugucuuugacg...................................	20	1	Ch1
....ucucacGaccuugucuuugacg...................................	2	1	Ch1
....ucucGcuaccuugucuuugacg...................................	55	1	Ch1
....ucucacuaccuugucuuugaUg...................................	11	1	Ch1

```
                 Mature                              Star
aauaaacauuuuuucccaugaaagacauggguagugagauguggcuaagcuagucaucucacuaccuugucuuugacggaaagaauuguaaaagucaguacagaauggaaagu
........................................................ucucacuaccuugucGuugacg...................................   8     1    Ch1
........................................................ucucacuaccuuguUuuugacg...................................   3     1    Ch1
........................................................ucucacuaccCugucuuugacg...................................   1     1    Ch1
........................................................ucucacuaccuugCcuuugacg...................................   1     1    Ch1
........................................................ucucacuaccuugucuuugCcg...................................   1     1    Ch1
........................................................uUucacuaccuugucuuugacg...................................   7     1    Ch1
........................................................ucucacuaccuugucuuugUcg...................................   3     1    Ch1
........................................................uAucacuaccuugucuuugacg...................................   21    1    Ch1
........................................................ucucacuaAcuugucuuugacg...................................   35    1    Ch1
........................................................ucucacuaccuugucuuugacA...................................   264   1    Ch1
........................................................ucucacuaccuuUucuuugacg...................................   3     1    Ch1
........................................................Acucacuaccuugucuuugacg...................................   13    1    Ch1
........................................................ucucaGuaccuugucuuugacg...................................   1     1    Ch1
........................................................ucucacuaccuugucuuugacgU..................................   172   1    Ch1
........................................................ucucacuaccuugucuuugacgg..................................   2     0    Ch1
........................................................ucucacuaccuugucuuugacgC..................................   10    1    Ch1
........................................................ucucacuaccuugucuuugacgA..................................   8     1    Ch1
.........................................................cucacuaccuugucuuuga.....................................   1     0    Ch1
.........................................................cucacuacAuugucuuugac....................................   1     1    Ch1
.........................................................cucacuaccuugucuuugac....................................   2     0    Ch1
.........................................................cucUcuaccuugucuuugacg...................................   1     1    Ch1
.........................................................cucaUuaccuugucuuugacg...................................   4     1    Ch1
.........................................................cucacuaccuuUucuuugacg...................................   5     1    Ch1
.........................................................cucacuacAuugucuuugacg...................................   1     1    Ch1
.........................................................cucacuaccuugucuuuAacg...................................   1     1    Ch1
.........................................................cuAacuaccuugucuuugacg...................................   6     1    Ch1
.........................................................cucacuaccuuAucuuugacg...................................   1     1    Ch1
.........................................................Aucacuaccuugucuuugacg...................................   1     1    Ch1
.........................................................cucaGuaccuugucuuugacg...................................   3     1    Ch1
.........................................................cucacuaccuugucuuugacA...................................   5     1    Ch1
.........................................................cucacuaccuugucuuuUacg...................................   1     1    Ch1
.........................................................Nucacuaccuugucuuugacg...................................   1     1    Ch1
.........................................................cucacuaccuugucuuugacg...................................   123   0    Ch1
.........................................................cucacuaccuugucuuugacgU..................................   2     1    Ch1
..........................................................ucacGaccuugucuuuga.....................................   1     1    Ch1
..........................................................ucacuaccuugucuuugacg...................................   24    0    Ch1
..........................................................ucacuaccuugucuuuUacg...................................   1     1    Ch1
..........................................................ucacuaccuugucuuugacA...................................   3     1    Ch1
..........................................................ucacuaccuugucuuugacgga.................................   1     0    Ch1
...........................................................cacuaccuuAucuuugacg...................................   1     1    Ch1
...........................................................cacuaccuugucuuugacg...................................   1     0    Ch1
...........................................................cacuaccuugucuuugacgg..................................   1     0    Ch1

.................augaaagacauggguagugaga..........................................................................   3     0    Ta1
.................augaaagacauggguagugagau.........................................................................   8     0    Ta1
.................augaaagacauggguagugagaug........................................................................   5     0    Ta1
.................augaaagacCuggguagugagaug........................................................................   1     1    Ta1
.................Cugaaagacauggguagugagaug........................................................................   11    1    Ta1
..................ugaaagacauggguagug.............................................................................   91    0    Ta1
..................ugaaUgacauggguagug.............................................................................   2     1    Ta1
..................ugaaaUacauggguagug.............................................................................   1     1    Ta1
..................ugaUagacauggguaguga............................................................................   1     1    Ta1
..................ugaaagacauggguaguga............................................................................   84    0    Ta1
..................ugaaGgacauggguaguga............................................................................   1     1    Ta1
..................ugaaagacauggguagugU............................................................................   10    1    Ta1
..................ugaaagacauggguaUuga............................................................................   1     1    Ta1
..................ugaaUgacauggguagugag...........................................................................   2     1    Ta1
..................ugaaagacauggguagugag...........................................................................   224   0    Ta1
..................ugaaagacauggguagugaU...........................................................................   2     1    Ta1
..................uCaaagacauggguagugag...........................................................................   1     1    Ta1
..................Agaaagacauggguagugag...........................................................................   1     1    Ta1
..................ugaaagacCuggguagugag...........................................................................   2     1    Ta1
..................ugaaagacauggguagugaA...........................................................................   10    1    Ta1
..................ugCaagacauggguagugag...........................................................................   1     1    Ta1
..................ugaaGgacauggguagugag...........................................................................   3     1    Ta1
..................ugaUagacauggguagugaga..........................................................................   3     1    Ta1
..................Ngaaagacauggguagugaga..........................................................................   1     1    Ta1
..................ugaaGgacauggguagugaga..........................................................................   16    1    Ta1
..................ugaaagacaugggguCgugaga.........................................................................   1     1    Ta1
..................Agaaagacauggguagugaga..........................................................................   1     1    Ta1
```

Mature Star

aauaaacauuuuuucccaugaaagacauggguagugagauguggcuaagcuagucaucucacuaccuugucuuugacggaaagaauuguaaaagucaguacagaauggaaagu			
..................ugaaagacUuggguagugaga..	1	1	Ta1
..................ugaaagacauggguaUugaga..	1	1	Ta1
..................ugaaagacauggguagugaUa..	2	1	Ta1
..................Cgaaagacauggguagugaga..	2	1	Ta1
..................ugaaUgacauggguagugaga..	18	1	Ta1
..................ugaaagacauggguagugagU..	8	1	Ta1
..................ugaaagacCuggguagugaga..	9	1	Ta1
..................ugaaagaUauggguagugaga..	1	1	Ta1
..................ugaaagCcauggguagugaga..	1	1	Ta1
..................ugaaagacauggguagugagC..	1	1	Ta1
..................ugaaagacauggguagugUga..	1	1	Ta1
..................ugaaagacauggguagugaga..	977	0	Ta1
..................ugCaagacauggguagugaga..	6	1	Ta1
..................ugaaagacaugUguagugaga..	1	1	Ta1
..................ugaaUgacauggguagugagau...	13	1	Ta1
..................ugaaagacCuggguagugagau...	14	1	Ta1
..................ugaaGgacauggguagugagau...	9	1	Ta1
..................uCaaagacauggguagugagau...	1	1	Ta1
..................Agaaagacauggguagugagau...	3	1	Ta1
..................ugaaagacauggguagugagau...	1238	0	Ta1
..................ugaaagacaugUguagugagau...	1	1	Ta1
..................ugaaagaUauggguagugagau...	4	1	Ta1
..................ugaaagacauggguagugaUau...	1	1	Ta1
..................ugaaagacauggguagugagaA...	60	1	Ta1
..................ugaaagacUuggguagugagau...	5	1	Ta1
..................Cgaaagacauggguagugagau...	3	1	Ta1
..................ugCaagacauggguagugagau...	5	1	Ta1
..................ugaUagacauggguagugagau...	1	1	Ta1
..................ugaaagacaugAguagugagau...	1	1	Ta1
..................ugaaagacauggguagugagCu...	1	1	Ta1
..................ugaaagacauggUuagugagau...	3	1	Ta1
..................ugaaagacauAgguagugagaug..	1	1	Ta1
..................ugaaagacauggguaguUagaug..	6	1	Ta1
..................ugaaagacauggguagugagaug..	6033	0	Ta1
..................ugaaagacauggguagugCgaug..	1	1	Ta1
..................ugaaagacauggguagugaUaug..	15	1	Ta1
..................ugaaagacaugUguagugagaug..	36	1	Ta1
..................ugaaagacauggguagugagaCg..	4	1	Ta1
..................ugaaagacauggguagugagCug..	4	1	Ta1
..................ugaaagacUuggguagugagaug..	6	1	Ta1
..................ugaaagCcauggguagugagaug..	2	1	Ta1
..................ugaaagacauggguaUugagaug..	1	1	Ta1
..................ugaaagacNuggguagugagaug..	1	1	Ta1
..................ugaaagacauggguGgugagaug..	1	1	Ta1
..................ugaaagacauggguagugUgaug..	2	1	Ta1
..................ugaaagacaugAguagugagaug..	1	1	Ta1
..................ugaaagacauggguaguAagaug..	1	1	Ta1
..................ugaaagacauUgguagugagaug..	1	1	Ta1
..................uUaaagacauggguagugagaug..	1	1	Ta1
..................ugCaagacauggguagugagaug..	12	1	Ta1
..................ugaaaUacauggguagugagaug..	1	1	Ta1
..................ugaaagaUauggguagugagaug..	9	1	Ta1
..................ugaaagacauggCuagugagaug..	4	1	Ta1
..................ugaaagacauggguagugagauA..	48	1	Ta1
..................ugaaagacauggguagCgagaug..	1	1	Ta1
..................ugaaagacauggguagugagauC..	16	1	Ta1
..................ugaaagacauggguagAgagaug..	2	1	Ta1
..................ugaaagacaugggCagugagaug..	1	1	Ta1
..................ugaaagacCuggguagugagaug..	64	1	Ta1
..................ugaaagacauggguagugaCaug..	1	1	Ta1
..................Cgaaagacauggguagugagaug..	9	1	Ta1
..................ugaaagacauggUuagugagaug..	1	1	Ta1
..................uAaaagacauggguagugagaug..	3	1	Ta1
..................ugaaagacauggguaguCagaug..	3	1	Ta1
..................ugaaagacauggguCgugagaug..	1	1	Ta1
..................ugaaagacauggAuagugagaug..	1	1	Ta1
..................ugaaGgacauggguagugagaug..	58	1	Ta1
..................Agaaagacauggguagugagaug..	11	1	Ta1
..................ugaaagacaCggguagugagaug..	3	1	Ta1
..................ugaUagacauggguagugagaug..	13	1	Ta1

```
                  Mature                              Star
aauaaacauuuuuucccaugaaagacauggguagugagauguggcuaagcuagucaucucacuaccuugucuuugacggaaagaauuguaaaagucaguacagaauggaaagu
..................ugaaUgacauggguagugagaug........................................................................  66    1  Ta1
..................ugaaagUcauggguagugagaug........................................................................  2     1  Ta1
..................ugaaagacauggguUgugagaug........................................................................  2     1  Ta1
..................Ngaaagacauggguagugagaug........................................................................  5     1  Ta1
..................ugaaagacauggguagugagUug........................................................................  1     1  Ta1
..................ugaaagacaugCguagugagaug........................................................................  5     1  Ta1
..................ugaaagacauggguagugagauU........................................................................  80    1  Ta1
..................ugaaagacaugggGagugagaug........................................................................  1     1  Ta1
..................ugaaagacaugggAagugagaug........................................................................  3     1  Ta1
..................ugaaagacauggguagugagCugu.......................................................................  1     1  Ta1
..................ugaaagaUauggguagugagaugu.......................................................................  1     1  Ta1
..................ugaaagacauggguagugagauAu.......................................................................  3     1  Ta1
..................ugaaagacCuggguagugagaugu.......................................................................  2     1  Ta1
..................ugaaagacauggguagugagauUu.......................................................................  5     1  Ta1
..................Agaaagacauggguagugagaugu.......................................................................  1     1  Ta1
..................ugaaGgacauggguagugagaugu.......................................................................  2     1  Ta1
..................ugaaagacauggguagugagaugu.......................................................................  75    0  Ta1
..................ugaaagacauggguagugagaugug......................................................................  6     0  Ta1
...................gaaagacauggguaguga............................................................................  1     0  Ta1
...................gaaagacauggguagugag...........................................................................  2     0  Ta1
...................gaaaUacauggguagugaga..........................................................................  2     1  Ta1
...................gaaagacauggguagugaga..........................................................................  6     0  Ta1
...................gaCagacauggguagugaga..........................................................................  1     1  Ta1
...................gaaagacauggguagugagaC.........................................................................  1     1  Ta1
...................gaaagacauggguagugagau.........................................................................  2     0  Ta1
...................gCaagacauggguagugagau.........................................................................  1     1  Ta1
...................gaaaAacauggguagugagaug........................................................................  3     1  Ta1
...................gaaagacaugggAagugagaug........................................................................  1     1  Ta1
...................gaaUgacauggguagugagaug........................................................................  2     1  Ta1
...................gaaagUcauggguagugagaug........................................................................  1     1  Ta1
...................gaaaUacauggguagugagaug........................................................................  3     1  Ta1
...................gaaaCacauggguagugagaug........................................................................  2     1  Ta1
...................Caaagacauggguagugagaug........................................................................  1     1  Ta1
...................gaaagacauggguagugagaug........................................................................  37    0  Ta1
....................aaagacauggguagugaga..........................................................................  1     0  Ta1
....................aUagacauggguagugaga..........................................................................  1     1  Ta1
......................................................caucucacuaccuugucuuugacU...................................  2     1  Ta1
.......................................................aucuGacuaccuugucuuug......................................  1     1  Ta1
.......................................................aucucacuaccuugucuuug......................................  7     0  Ta1
.......................................................Cucucacuaccuugucuuugacg...................................  4     1  Ta1
.......................................................aucucacuaccuugucuuugacg...................................  1     0  Ta1
........................................................ucuUacuaccuugucuuu.......................................  1     1  Ta1
........................................................ucucacuaccuugucuuu.......................................  1     0  Ta1
........................................................ucucacuaccuugucuuug......................................  10    0  Ta1
........................................................ucucacuaccuugucuuuga.....................................  31    0  Ta1
........................................................ucucUcuaccuugucuuugac....................................  1     1  Ta1
........................................................ucucacuaccuugucuuugac....................................  36    0  Ta1
........................................................ucucacuUccuugucuuugacg...................................  1     1  Ta1
........................................................ucucacuacUuugucuuugacg...................................  1     1  Ta1
........................................................ucucaUuaccuugucuuugacg...................................  3     1  Ta1
........................................................ucucacGaccuugucuuugacg...................................  1     1  Ta1
........................................................ucucacuaccuugucuuugacg...................................  3489  0  Ta1
........................................................ucucacuaccuugucuuugacU...................................  18    1  Ta1
........................................................ucucacuaccuugucGuugacg...................................  2     1  Ta1
........................................................Acucacuaccuugucuuugacg...................................  7     1  Ta1
........................................................Ncucacuaccuugucuuugacg...................................  5     1  Ta1
........................................................Ccucacuaccuugucuuugacg...................................  4     1  Ta1
........................................................ucucacuaccuugucuuuUacg...................................  11    1  Ta1
........................................................ucucacuaccAugucuuugacg...................................  2     1  Ta1
........................................................ucucacuaccuugucuuugaUg...................................  1     1  Ta1
........................................................ucucacuaccuugucuAugacg...................................  1     1  Ta1
........................................................ucucacuaccuugucuuugUcg...................................  1     1  Ta1
........................................................ucucCcuaccuugucuuugacg...................................  1     1  Ta1
........................................................ucGcacuaccuugucuuugacg...................................  1     1  Ta1
........................................................ucucacuaccuugucuuugGcg...................................  2     1  Ta1
........................................................ucuUacuaccuugucuuugacg...................................  19    1  Ta1
........................................................ucucacuacAuugucuuugacg...................................  4     1  Ta1
........................................................ucucacuaNcuugucuuugacg...................................  1     1  Ta1
........................................................uAucacuaccuugucuuugacg...................................  11    1  Ta1
........................................................ucucacuaccuugucuuugCcg...................................  1     1  Ta1
```

Mature **Star**

```
aauaaacauuuuuuccca ugaaagacauggguagugagaug uggcuaagcuaguca ucucacuaccuugucuuugacg gaaagaauuguaaaagucaguacagaauggaaagu
........................................................uGucacuaccuugucuuugacg...................................   1   1   Ta1
........................................................ucucacAaccuugucuuugacg...................................   1   1   Ta1
........................................................ucCcacuaccuugucuuugacg...................................   9   1   Ta1
........................................................ucucacuaccuugucuuugacA...................................   66  1   Ta1
........................................................ucucUcuaccuugucuuugacg...................................   43  1   Ta1
........................................................Gcucacuaccuugucuuugacg...................................   2   1   Ta1
........................................................uUucacuaccuugucuuugacg...................................   3   1   Ta1
........................................................ucucacuaccuuAucuuugacg...................................   1   1   Ta1
........................................................ucucGcuaccuugucuuugacg...................................   20  1   Ta1
........................................................ucucacuaAcuugucuuugacg...................................   29  1   Ta1
........................................................ucucacuaccuugucuuAgacg...................................   1   1   Ta1
........................................................ucucacuaccuugucuuugacgg..................................   3   0   Ta1
........................................................ucucacuaccuugucuuugacgU..................................   118 1   Ta1
.........................................................cucacuaccuugucuuug......................................   1   0   Ta1
.........................................................cucacuaccuugucuuuga.....................................   1   0   Ta1
.........................................................cucacuaccuugucuuugac....................................   1   0   Ta1
.........................................................cucaUuaccuugucuuugacg...................................   2   1   Ta1
.........................................................cucacuaccuugucuuugacg...................................   51  0   Ta1
.........................................................cucacAaccuugucuuugacg...................................   2   1   Ta1
.........................................................cucacCaccuugucuuugacg...................................   1   1   Ta1
.........................................................cucacuaccuugucuuugacA...................................   1   1   Ta1
.........................................................cucaGuaccuugucuuugacg...................................   2   1   Ta1
.........................................................cucacuaccuugucuuugGcg...................................   1   1   Ta1
.........................................................cucacuaAcuugucuuugacg...................................   1   1   Ta1
.........................................................cucacuaccuugucuuugaGg...................................   1   1   Ta1
.........................................................cucacuaccuuUucuuugacg...................................   1   1   Ta1
.........................................................cucUcuaccuugucuuugacg...................................   1   1   Ta1
.........................................................Aucacuaccuugucuuugacg...................................   3   1   Ta1
.........................................................cucCcuaccuugucuuugacg...................................   1   1   Ta1
.........................................................cuAacuaccuugucuuugacg...................................   4   1   Ta1
.........................................................cucacuaccuugucuuugacgU..................................   5   1   Ta1
..........................................................ucacuaccuugucuuugacA...................................   1   1   Ta1

................Aaugaaagacauggguagugaga..........................................................................   1   1   Le5
.................augaaagacauggguagug.............................................................................   1   0   Le5
.................augaaagacauggguaguga............................................................................   2   0   Le5
.................augaaagacauggguagugag...........................................................................   2   0   Le5
.................auCaaagacauggguagugaga..........................................................................   1   1   Le5
.................augaaagacauggguagugaga..........................................................................   10  0   Le5
.................augaaagacauggguagugagU..........................................................................   1   1   Le5
.................augaaagacauggguagugagau.........................................................................   6   0   Le5
.................auCaaagacauggguagugagau.........................................................................   1   1   Le5
.................Cugaaagacauggguagugagau.........................................................................   2   1   Le5
.................augaaagacauggguagugagaug........................................................................   12  0   Le5
.................augaaagUcauggguagugagaug........................................................................   1   1   Le5
.................Cugaaagacauggguagugagaug........................................................................   3   1   Le5
..................ugaaagacauggCuagug.............................................................................   1   1   Le5
..................ugaaagaUauggguagug.............................................................................   1   1   Le5
..................ugaaagacUuggguagug.............................................................................   1   1   Le5
..................ugaaagacauggguaguA.............................................................................   10  1   Le5
..................ugaaUgacauggguagug.............................................................................   2   1   Le5
..................ugaaGgacauggguagug.............................................................................   1   1   Le5
..................ugaaagacauggguagAg.............................................................................   1   1   Le5
..................ugaaagacCuggguagug.............................................................................   1   1   Le5
..................ugaaagacauggguagug.............................................................................   244 0   Le5
..................Cgaaagacauggguaguga............................................................................   1   1   Le5
..................ugaaagacaugUguaguga............................................................................   3   1   Le5
..................ugaaagacauggguagugU............................................................................   7   1   Le5
..................ugaaUgacauggguaguga............................................................................   1   1   Le5
..................ugaaGgacauggguaguga............................................................................   2   1   Le5
..................ugaaagacauggguagugC............................................................................   3   1   Le5
..................ugaaagacaugggCaguga............................................................................   1   1   Le5
..................ugaaagacauggguaguUa............................................................................   1   1   Le5
..................ugaaagacauggguagAga............................................................................   1   1   Le5
..................ugaaagacauggguaguga............................................................................   339 0   Le5
..................ugaaagacauggguaguAa............................................................................   7   1   Le5
..................ugaaagacaugggUguga.............................................................................   1   1   Le5
..................ugaaagacCuggguaguga............................................................................   6   1   Le5
..................ugaaagacauggguagugaA...........................................................................   62  1   Le5
..................ugaaagacCuggguagugag...........................................................................   7   1   Le5
```

```
                  Mature                                   Star
aauaaacauuuuuucccaugaaagacauggguagugagauguggcuaagcuagucaucucacuaccuugucuuugacggaaagaauuguaaaagucaguacagaauggaaagu
..................ugaaagacauggguagugag...........................................................................    623    0    Le5
..................ugaaagacauggguagGgag...........................................................................    1      1    Le5
..................ugaaagacauggguaguUag...........................................................................    1      1    Le5
..................Cgaaagacauggguagugag...........................................................................    3      1    Le5
..................ugaaagacauggguagugaC...........................................................................    1      1    Le5
..................ugaaagacaAggguagugag...........................................................................    1      1    Le5
..................Ngaaagacauggguagugag...........................................................................    1      1    Le5
..................ugaaagacauggguagAgag...........................................................................    1      1    Le5
..................ugaaagacaugUguagugag...........................................................................    3      1    Le5
..................ugaUagacauggguagugag...........................................................................    2      1    Le5
..................ugaaagacauggAuagugag...........................................................................    1      1    Le5
..................ugaaagacauggguagugUg...........................................................................    1      1    Le5
..................ugaaagacauUgguagugag...........................................................................    1      1    Le5
..................ugaaGgacauggguagugag...........................................................................    2      1    Le5
..................ugaaagacauggguagugaU...........................................................................    2      1    Le5
..................Agaaagacauggguagugag...........................................................................    3      1    Le5
..................ugaaUgacauggguagugag...........................................................................    7      1    Le5
..................ugaaagacauggguagugGg...........................................................................    1      1    Le5
..................ugaaagacauggguagugaAa..........................................................................    18     1    Le5
..................ugaaagacauggguagugagC..........................................................................    14     1    Le5
..................ugaaagacauggguagugagG..........................................................................    4      1    Le5
..................Agaaagacauggguagugaga..........................................................................    8      1    Le5
..................ugaaGgacauggguagugaga..........................................................................    35     1    Le5
..................ugCaagacauggguagugaga..........................................................................    6      1    Le5
..................ugaUagacauggguagugaga..........................................................................    10     1    Le5
..................ugaaagacUuggguagugaga..........................................................................    3      1    Le5
..................ugaaagacauUgguagugaga..........................................................................    2      1    Le5
..................ugaaagUcauggguagugaga..........................................................................    1      1    Le5
..................ugaaagacauggguagugaCa..........................................................................    6      1    Le5
..................uCaaagacauggguagugaga..........................................................................    2      1    Le5
..................uAaaagacauggguagugaga..........................................................................    3      1    Le5
..................ugaaagaUauggguagugaga..........................................................................    6      1    Le5
..................ugaaagacauggAuagugaga..........................................................................    1      1    Le5
..................ugaaagacauggguagugagU..........................................................................    17     1    Le5
..................ugaaagacaugUguagugaga..........................................................................    6      1    Le5
..................ugaaagacauggguagCgaga..........................................................................    2      1    Le5
..................ugaaagacauggguaguUaga..........................................................................    1      1    Le5
..................ugaaagacaCggguagugaga..........................................................................    1      1    Le5
..................ugaaUgacauggguagugaga..........................................................................    30     1    Le5
..................ugaaagacaugAguagugaga..........................................................................    1      1    Le5
..................ugaaagacauggguaCugaga..........................................................................    1      1    Le5
..................ugaaagacCuggguagugaga..........................................................................    21     1    Le5
..................Cgaaagacauggguagugaga..........................................................................    8      1    Le5
..................ugaaagacauggguagugaga..........................................................................    3395   0    Le5
..................ugaaagacauggguagugaUa..........................................................................    7      1    Le5
..................ugaaagacauggUuagugaga..........................................................................    1      1    Le5
..................ugaaaUacauggguagugaga..........................................................................    1      1    Le5
..................ugGaagacauggguagugaga..........................................................................    2      1    Le5
..................ugaaagacauggguagugaUau.........................................................................    7      1    Le5
..................ugaaagacauggguagugagGu.........................................................................    2      1    Le5
..................ugaaUgacauggguagugagau.........................................................................    34     1    Le5
..................ugaaagacauggguagugagUu.........................................................................    1      1    Le5
..................Agaaagacauggguagugagau.........................................................................    6      1    Le5
..................ugaGagacauggguagugagau.........................................................................    3      1    Le5
..................ugaaagaUauggguagugagau.........................................................................    6      1    Le5
..................Ngaaagacauggguagugagau.........................................................................    4      1    Le5
..................ugGaagacauggguagugagau.........................................................................    1      1    Le5
..................ugaaaUacauggguagugagau.........................................................................    2      1    Le5
..................ugaaagacauAgguagugagau.........................................................................    3      1    Le5
..................ugaaagacauggguUgugagau.........................................................................    2      1    Le5
..................ugaaagacaugAguagugagau.........................................................................    2      1    Le5
..................ugaaagacauggguagugagaA.........................................................................    162    1    Le5
..................ugaaagacaugUguagugagau.........................................................................    8      1    Le5
..................ugaaagacauggguagugagau.........................................................................    3476   0    Le5
..................ugaUagacauggguagugagau.........................................................................    6      1    Le5
..................ugaaagacauggguagugagaC.........................................................................    48     1    Le5
..................ugaaGgacauggguagugagau.........................................................................    20     1    Le5
..................ugaaagUcauggguagugagau.........................................................................    1      1    Le5
..................uAaaagacauggguagugagau.........................................................................    3      1    Le5
..................Cgaaagacauggguagugagau.........................................................................    8      1    Le5
```

Mature **Star**

```
aauaaacauuuuuuccca ugaaagacauggguagugagaug uggcuaagcuaguca ucucacuaccuugucuuugacg gaaagaauuguaaaagucaguacagaauggaaagu
```

```
aauaaacauuuuuucccaugaaagacauggguagugagauguggcuaagcuagucaucucacuaccuugucuuugacggaaagaauuguaaaagucaguacagaauggaaagu
..................uCaaagacauggguagugagau.........................................................................    1     1    Le5
..................ugaaagacauUgguagugagau.........................................................................    1     1    Le5
..................ugaaagacCuggguagugagau.........................................................................    50    1    Le5
..................ugaaagacauggguagugGgau.........................................................................    2     1    Le5
..................ugaaagacauggguagugagCu.........................................................................    5     1    Le5
..................ugaaagacauCgguagugagau.........................................................................    1     1    Le5
..................ugaaagacUuggguagugagau.........................................................................    4     1    Le5
..................ugaaagacaugggu Ggugagau.........................................................................    2     1    Le5
..................ugaaagacaugggAagugagau.........................................................................    2     1    Le5
..................ugaaagacauggUuagugagau.........................................................................    1     1    Le5
..................ugaaagacauggguagugUgau.........................................................................    1     1    Le5
..................ugCaagacauggguagugagau.........................................................................    6     1    Le5
..................ugaaagacauggCuagugagau.........................................................................    1     1    Le5
..................ugaaagacauggguaUugagau.........................................................................    3     1    Le5
..................ugaaagacauggguagugUgaug........................................................................    2     1    Le5
..................ugaaagacauggguagugagUug........................................................................    2     1    Le5
..................ugaaagacauggguagugagaCg........................................................................    2     1    Le5
..................Ggaaagacauggguagugagaug........................................................................    2     1    Le5
..................ugaaagacauAgguagugagaug........................................................................    6     1    Le5
..................ugaaagacauggguagugaAaug........................................................................    3     1    Le5
..................ugaaagacaugggCagugagaug........................................................................    3     1    Le5
..................ugaaagacaAggguagugagaug........................................................................    3     1    Le5
..................ugCaagacauggguagugagaug........................................................................    12    1    Le5
..................uUaaagacauggguagugagaug........................................................................    1     1    Le5
..................ugaaagacCuggguagugagaug........................................................................    70    1    Le5
..................ugaaagacauggguagugagCug........................................................................    5     1    Le5
..................ugaaGgacauggguagugagaug........................................................................    59    1    Le5
..................ugaaagacaugggu Ugugagaug........................................................................    5     1    Le5
..................ugaaagacauggguagugagaug........................................................................    6966  0    Le5
..................ugaaagacauggguagugagauC........................................................................    6     1    Le5
..................ugaaagacaugggGagugagaug........................................................................    2     1    Le5
..................ugaaagacauggguagugagGug........................................................................    1     1    Le5
..................uAaaagacauggguagugagaug........................................................................    12    1    Le5
..................ugaaagUcauggguagugagaug........................................................................    1     1    Le5
..................ugaaagacauggguaUugagaug........................................................................    5     1    Le5
..................ugaaagacauggguagugagauA........................................................................    125   1    Le5
..................Cgaaagacauggguagugagaug........................................................................    8     1    Le5
..................Ngaaagacauggguagugagaug........................................................................    3     1    Le5
..................ugaaagacaGgguagugagaug........................................................................    2     1    Le5
..................ugaaagaUauggguagugagaug........................................................................    21    1    Le5
..................ugaaagacauggguagugaCaug........................................................................    1     1    Le5
..................ugaUagacauggguagugagaug........................................................................    11    1    Le5
..................ugaaagacauggCuagugagaug........................................................................    1     1    Le5
..................ugaaagacaugUguagugagaug........................................................................    20    1    Le5
..................ugaaagacauggguagugagauU........................................................................    250   1    Le5
..................ugaaaUacauggguagugagaug........................................................................    3     1    Le5
..................ugaaagacUuggguagugagaug........................................................................    2     1    Le5
..................Agaaagacauggguagugagaug........................................................................    15    1    Le5
..................ugaaagacauggguagugagaAg........................................................................    1     1    Le5
..................ugaaagacauggguaguCagaug........................................................................    1     1    Le5
..................uCaaagacauggguagugagaug........................................................................    5     1    Le5
..................ugaaagacauggguagAgagaug........................................................................    1     1    Le5
..................ugaaagacauggAuagugagaug........................................................................    2     1    Le5
..................ugaaagacauggUuagugagaug........................................................................    1     1    Le5
..................ugaaUgacauggguagugagaug........................................................................    64    1    Le5
..................ugGaagacauggguagugagaug........................................................................    2     1    Le5
..................ugaaagacaugCguagugagaug........................................................................    2     1    Le5
..................ugaaagacaugggu Ggugagaug........................................................................    6     1    Le5
..................ugaaagacaugggAagugagaug........................................................................    3     1    Le5
..................ugaaagacauggguagugaUaug........................................................................    15    1    Le5
..................ugaaagacauggguaguUagaug........................................................................    4     1    Le5
..................ugaaagaGauggguagugagaug........................................................................    1     1    Le5
..................ugaaagacauggguagugagaugu.......................................................................    165   0    Le5
..................ugaaUgacauggguagugagaugu.......................................................................    1     1    Le5
..................ugaaagacauggguagugagaugG.......................................................................    1     1    Le5
..................Cgaaagacauggguagugagaugu.......................................................................    1     1    Le5
..................ugUaagacauggguagugagaugu.......................................................................    1     1    Le5
..................ugaaagacauggguagugagCugu.......................................................................    1     1    Le5
..................ugaaagacauggguagugagauUu.......................................................................    5     1    Le5
..................ugaaagacauggguagugagaugC.......................................................................    12    1    Le5
```

Mature **Star**

```
aauaaacauuuuuucccaugaaagacauggguagugagauguggcuaagcuagucaucucacuaccuugucuuugacggaaagaauuguaaaagucaguacagaauggaaagu
..................ugaaagacCuggguagugagaugu.......................................................................   1      1   Le5
..................ugaaagacauggguagugagaugug......................................................................   1      0   Le5
..................ugaaagacauggguagugagauguA......................................................................   3      1   Le5
..................ugaaagacauggguagugagauguggc....................................................................   1      0   Le5
...................gaaagacauggguaguga............................................................................   3      0   Le5
...................gGaagacauggguagugaga..........................................................................   1      1   Le5
...................gaaagacauggguagugaga..........................................................................   13     0   Le5
...................gaaaUacauggguagugaga..........................................................................   1      1   Le5
...................gaaaUacauggguagugagau.........................................................................   3      1   Le5
...................gaaUgacauggguagugagau.........................................................................   1      1   Le5
...................gGaagacauggguagugagau.........................................................................   1      1   Le5
...................gaaagacauggguagugagau.........................................................................   10     0   Le5
...................gaaagacauggguagugaUau.........................................................................   1      1   Le5
...................gaaagacauggguagugagauA........................................................................   2      1   Le5
...................gCaagacauggguagugagaug........................................................................   1      1   Le5
...................gaCagacauggguagugagaug........................................................................   1      1   Le5
...................gaaagacaAgguagugagaug.........................................................................   1      1   Le5
...................gaaUgacauggguagugagaug........................................................................   1      1   Le5
...................gaaagacauggUuagugagaug........................................................................   1      1   Le5
...................gaaagacauggguagugagaug........................................................................   37     0   Le5
...................gaaagacauggAuagugagaug........................................................................   1      1   Le5
...................gaaaUacauggguagugagaug........................................................................   3      1   Le5
...................gaaagacauggguagugagaugu.......................................................................   1      0   Le5
....................aaagacauggguagugagaug........................................................................   3      0   Le5
......................agacauggguagugagau.........................................................................   1      0   Le5
.....................................................ucaucucacuaccuugucuuug......................................   1      0   Le5
......................................................caucucacuaccuugucuuug......................................   4      0   Le5
.......................................................aucucacuaccuugucuuug......................................   2      0   Le5
.......................................................aucucacuaccuugucuuuga.....................................   3      0   Le5
.......................................................Cucucacuaccuugucuuugacg...................................   8      1   Le5
.......................................................aucucacuaccuugucuuugacg...................................   4      0   Le5
........................................................ucucacuaccuugucuuu.......................................   4      0   Le5
........................................................ucucacCaccuugucuuug......................................   1      1   Le5
........................................................ucucacuaccuugucuuuA......................................   3      1   Le5
........................................................ucucUcuaccuugucuuug......................................   1      1   Le5
........................................................ucucacuaccuuguUuuug......................................   1      1   Le5
........................................................ucucacuaccuugucuuug......................................   71     0   Le5
........................................................ucucacuaAcuugucuuuga.....................................   1      1   Le5
........................................................ucucacuacUuugucuuuga.....................................   2      1   Le5
........................................................ucucacuaccuugucuuuUa.....................................   1      1   Le5
........................................................ucucacuaccuugucCuuga.....................................   2      1   Le5
........................................................ucucacuaccuugucuuuga.....................................   93     0   Le5
........................................................ucucacuaccuugucuuugac....................................   179    0   Le5
........................................................ucuUacuaccuugucuuugac....................................   2      1   Le5
........................................................ucucUcuaccuugucuuugac....................................   2      1   Le5
........................................................ucucacuaccuugucuuugaU....................................   3      1   Le5
........................................................ucucacuUccuugucuuugac....................................   1      1   Le5
........................................................ucucacuaccuugucuuuAac....................................   1      1   Le5
........................................................ucuUacuaccuugucuuugacg...................................   27     1   Le5
........................................................Ncucacuaccuugucuuugacg...................................   8      1   Le5
........................................................ucucacuaccuugucAuugacg...................................   1      1   Le5
........................................................Ccucacuaccuugucuuugacg...................................   17     1   Le5
........................................................Gcucacuaccuugucuuugacg...................................   2      1   Le5
........................................................ucucacuaccuugucuuugGcg...................................   8      1   Le5
........................................................ucucacuaccuuguUuuugacg...................................   3      1   Le5
........................................................ucucGcuaccuugucuuugacg...................................   89     1   Le5
........................................................ucucacuaccuugucuuuUacg...................................   30     1   Le5
........................................................ucCcacuaccuugucuuugacg...................................   34     1   Le5
........................................................ucucacuaccuugucuuugUcg...................................   1      1   Le5
........................................................ucucacuaccuugucuuugacg...................................   11586  0   Le5
........................................................ucucacuaccGugucuuugacg...................................   1      1   Le5
........................................................ucucacuacGuugucuuugacg...................................   1      1   Le5
........................................................ucucacuacUuugucuuugacg...................................   11     1   Le5
........................................................ucucacuGccuugucuuugacg...................................   3      1   Le5
........................................................uUucacuaccuugucuuugacg...................................   3      1   Le5
........................................................ucucacuaccuGgucuuugacg...................................   1      1   Le5
........................................................ucucacuaccAugucuuugacg...................................   4      1   Le5
........................................................ucucacuaccuugucuCugacg...................................   2      1   Le5
........................................................ucucacuaccuugGcuuugacg...................................   1      1   Le5
........................................................ucucacuaccuAgucuuugacg...................................   1      1   Le5
```

```
                  Mature                        Star
aauaaacauuuuuucccaugaaagacaugguagugagaugguggcuaagcuagucaucucacuaccuugucuuugacggaaagaauuguaaaagucaguacagaauggaaagu
.......................................................ucucacCaccuugucuuugacg...................................	1	1	Le5
.......................................................ucucacuaccuugucGuugacg...................................	5	1	Le5
.......................................................ucucacAaccuugucuuugacg...................................	1	1	Le5
.......................................................ucucacuaccuugucuuugCcg...................................	1	1	Le5
.......................................................ucucacuaccuuguAuuugacg...................................	1	1	Le5
.......................................................ucucacuaccuCgucuuugacg...................................	3	1	Le5
.......................................................ucucaUuaccuugucuuugacg...................................	7	1	Le5
.......................................................ucucacuaccuugucCuugacg...................................	8	1	Le5
.......................................................ucucacuaccuugAcuuugacg...................................	2	1	Le5
.......................................................uAucacuaccuugucuuugacg...................................	28	1	Le5
.......................................................ucucUcuaccuugucuuugacg...................................	127	1	Le5
.......................................................ucucacGaccuugucuuugacg...................................	5	1	Le5
.......................................................ucucacuaccCugucuuugacg...................................	2	1	Le5
.......................................................ucucacuaccuuUucuuugacg...................................	5	1	Le5
.......................................................ucucacuUccuugucuuugacg...................................	22	1	Le5
.......................................................ucucacuaccuugucuuugaUg...................................	10	1	Le5
.......................................................ucucacuaccuugCcuuugacg...................................	5	1	Le5
.......................................................ucucaAuaccuugucuuugacg...................................	2	1	Le5
.......................................................ucucacuaccuugucuuAgacg...................................	1	1	Le5
.......................................................ucucacuaccuugucuuugacC...................................	12	1	Le5
.......................................................Acucacuaccuugucuuugacg...................................	25	1	Le5
.......................................................ucucacuaccuugucuuuAacg...................................	2	1	Le5
.......................................................ucucacuaccuugucuuCgacg...................................	3	1	Le5
.......................................................ucucacuaGcuugucuuugacg...................................	2	1	Le5
.......................................................ucuGacuaccuugucuuugacg...................................	1	1	Le5
.......................................................ucucacuacAuugucuuugacg...................................	5	1	Le5
.......................................................ucAcacuaccuugucuuugacg...................................	1	1	Le5
.......................................................ucucacuaAcuugucuuugacg...................................	42	1	Le5
.......................................................uGucacuaccuugucuuugacg...................................	2	1	Le5
.......................................................ucucacuaccuugucuuGgacg...................................	2	1	Le5
.......................................................ucuAacuaccuugucuuugacg...................................	1	1	Le5
.......................................................ucucacuaccuugucuuugacA...................................	377	1	Le5
.......................................................ucucacuaccuugucuuuCacg...................................	2	1	Le5
.......................................................ucucacuaccuuAucuuugacg...................................	9	1	Le5
.......................................................ucucacuaccuugucuuugacU...................................	34	1	Le5
.......................................................ucucacuaccuugucuuugacgU..................................	244	1	Le5
.......................................................ucucacuaccuugucuuugacgC..................................	7	1	Le5
.......................................................ucucacuaccuugucuuugacgA..................................	16	1	Le5
.......................................................ucucacuaccuugucuuugacgg..................................	3	0	Le5
.......................................................ucucacuaccuugucuuugacgUa.................................	4	1	Le5
........................................................cucacuaccuugucuuuga.....................................	2	0	Le5
........................................................cucacuaccuugucuuugac....................................	2	0	Le5
........................................................cucacuaccuAgucuuugac....................................	1	1	Le5
........................................................cucacuaccuugucuuugacA...................................	6	1	Le5
........................................................cucacuaccuugucuuugacU...................................	1	1	Le5
........................................................cucacuacAuugucuuugacg...................................	1	1	Le5
........................................................cucacuaccuugucuuugaGg...................................	2	1	Le5
........................................................cucacuaccuugucuuugUcg...................................	1	1	Le5
........................................................cucacuaccuugucuGugacg...................................	1	1	Le5
........................................................cucacuaccuugucuuugacg...................................	159	0	Le5
........................................................cucaUuaccuugucuuugacg...................................	3	1	Le5
........................................................Aucacuaccuugucuuugacg...................................	1	1	Le5
........................................................cucaGuaccuugucuuugacg...................................	3	1	Le5
........................................................cCcacuaccuugucuuugacg...................................	1	1	Le5
........................................................cucacuaAcuugucuuugacg...................................	1	1	Le5
........................................................cucacuaccuugucuuugGcg...................................	2	1	Le5
........................................................cucacuaccuuUucuuugacg...................................	8	1	Le5
........................................................cucacuaccuuguUuuugacg...................................	1	1	Le5
........................................................cuAacuaccuugucuuugacg...................................	4	1	Le5
........................................................cucCcuaccuugucuuugacg...................................	1	1	Le5
........................................................cucUcuaccuugucuuugacg...................................	7	1	Le5
........................................................cucacuaccuugucuuugacgU..................................	5	1	Le5
.........................................................ucacuaccuugucuuugacA...................................	2	1	Le5
.........................................................ucaUuaccuugucuuugacg...................................	1	1	Le5
.........................................................ucacuaccuugucuuugacC...................................	1	1	Le5
.........................................................ucacuaccuugucuuugacU...................................	1	1	Le5
.........................................................ucCcuaccuugucuuugacg...................................	1	1	Le5
.........................................................ucacuaccuugucuuugacg...................................	30	0	Le5
.........................................................ucacGaccuugucuuugacg...................................	2	1	Le5
.........................................................ucacuaccuugucuuugacgU..................................	1	1	Le5
```

```
                  Mature                              Star
aauaaacauuuuuucccaugaaagacauggguagugagauguggcuaagcuagucaucucacuaccuugucuuugacggaaagaauuguaaaagucaguacagaauggaaagu

................Aaugaaagacauggguagugagaug........................................................................    1      1      Le1
.................Uugaaagacauggguagug.............................................................................    1      1      Le1
.................augaaagacauggguagug.............................................................................    2      0      Le1
.................augaaagacauggguaguga............................................................................    1      0      Le1
.................augaaagacauggguagugag...........................................................................    1      0      Le1
.................Cugaaagacauggguagugaga..........................................................................    1      1      Le1
.................augaaagacauggguagugaga..........................................................................    6      0      Le1
.................Cugaaagacauggguagugagau.........................................................................    1      1      Le1
.................augaaagacauggguagugagau.........................................................................    4      0      Le1
.................augaaagacauggguagugagaug........................................................................    9      0      Le1
.................Cugaaagacauggguagugagaug........................................................................    1      1      Le1
..................ugaaagaUauggguagug.............................................................................    1      1      Le1
..................ugaaagacauggguaguA.............................................................................    7      1      Le1
..................ugaaagacauggCuagug.............................................................................    1      1      Le1
..................ugaaagacauUgguagug.............................................................................    1      1      Le1
..................Ngaaagacauggguagug.............................................................................    1      1      Le1
..................ugaaagacUuggguagug.............................................................................    1      1      Le1
..................ugaaUgacauggguagug.............................................................................    3      1      Le1
..................ugaaagacauggguagug.............................................................................    214    0      Le1
..................ugaaagacauggUuagug.............................................................................    2      1      Le1
..................ugaaagacaugUguagug.............................................................................    1      1      Le1
..................ugCaagacauggguagug.............................................................................    2      1      Le1
..................ugaaGgacauggguagug.............................................................................    1      1      Le1
..................ugaaagacCuggguagug.............................................................................    3      1      Le1
..................ugaaagacCuggguaguga............................................................................    2      1      Le1
..................ugaaagacauggguaguUa............................................................................    2      1      Le1
..................Ngaaagacauggguaguga............................................................................    1      1      Le1
..................ugaaGgacauggguaguga............................................................................    5      1      Le1
..................ugaaagacauggguaguga............................................................................    214    0      Le1
..................ugaaagacauggguagugU............................................................................    4      1      Le1
..................ugaaUgacauggguaguga............................................................................    4      1      Le1
..................ugaaagacauggguagugC............................................................................    1      1      Le1
..................ugaaagacauggguaUuga............................................................................    1      1      Le1
..................ugUaagacauggguaguga............................................................................    1      1      Le1
..................ugaaagacauggguaguAa............................................................................    1      1      Le1
..................ugaaagaGauggguagugag...........................................................................    1      1      Le1
..................ugaaagacauggguagugaA...........................................................................    33     1      Le1
..................ugaaUgacauggguagugag...........................................................................    2      1      Le1
..................ugaaagacCuggguagugag...........................................................................    3      1      Le1
..................Agaaagacauggguagugag...........................................................................    1      1      Le1
..................ugaUagacauggguagugag...........................................................................    1      1      Le1
..................ugaaagacaugUguagugag...........................................................................    3      1      Le1
..................ugCaagacauggguagugag...........................................................................    1      1      Le1
..................uCaaagacauggguagugag...........................................................................    1      1      Le1
..................ugaaGgacauggguagugag...........................................................................    6      1      Le1
..................ugaaagacauggguagugGg...........................................................................    1      1      Le1
..................ugaaagacauggguagugaU...........................................................................    4      1      Le1
..................Cgaaagacauggguagugag...........................................................................    1      1      Le1
..................ugaaagacaugggAagugag...........................................................................    1      1      Le1
..................ugaaagacauggguagugag...........................................................................    394    0      Le1
..................ugaaagacauggguGgugaga..........................................................................    2      1      Le1
..................ugaaagacauUgguagugaga..........................................................................    1      1      Le1
..................ugaaagacauggguagugagG..........................................................................    2      1      Le1
..................ugaaagacauggguagugaga..........................................................................    2106   0      Le1
..................ugCaagacauggguagugaga..........................................................................    6      1      Le1
..................ugaaagacauggguagugaUa..........................................................................    7      1      Le1
..................ugaaUgacauggguagugaga..........................................................................    25     1      Le1
..................ugUaagacauggguagugaga..........................................................................    1      1      Le1
..................ugaaagacauggguagugaCa..........................................................................    6      1      Le1
..................ugaaagaUauggguagugaga..........................................................................    6      1      Le1
..................ugaUagacauggguagugaga..........................................................................    4      1      Le1
..................ugaaagacaCggguagugaga..........................................................................    1      1      Le1
..................ugaaagacaugggCagugaga..........................................................................    1      1      Le1
..................Agaaagacauggguagugaga..........................................................................    7      1      Le1
..................Ngaaagacauggguagugaga..........................................................................    4      1      Le1
..................ugaaagacauggguagCgaga..........................................................................    2      1      Le1
..................ugaaagacUuggguagugaga..........................................................................    4      1      Le1
..................ugaaagacauggguagugaAa..........................................................................    10     1      Le1
..................ugaaagacauggguagugagC..........................................................................    6      1      Le1
```

Mature Star

aauaaacauuuuuucccaugaaagacauggguagugagauguggcuaagcuagucaucucacuaccuugucuuugacggaaagaauuguaaaagucaguacagaauggaaagu			
..................Cgaaagacauggguagugaga..	1	1	Le1
..................ugaaagacauggguagugagU..	13	1	Le1
..................uCaaagacauggguagugaga..	1	1	Le1
..................ugaaGgacauggguagugaga..	17	1	Le1
..................ugaaagacaugUguagugaga..	3	1	Le1
..................ugaaagacCuggguagugaga..	21	1	Le1
..................ugaaagacauAgguagugaga..	1	1	Le1
..................ugaaagacaugCguagugaga..	2	1	Le1
..................ugaaagacUuggguagugagau...	1	1	Le1
..................ugaaGgacauggguagugagau...	18	1	Le1
..................uCaaagacauggguagugagau...	1	1	Le1
..................ugaaagacauggguagugGgau...	1	1	Le1
..................ugaaagacCuggguagugagau...	16	1	Le1
..................Agaaagacauggguagugagau...	1	1	Le1
..................Ggaaagacauggguagugagau...	1	1	Le1
..................ugaaagacauggAuagugagau...	1	1	Le1
..................uUaaagacauggguagugagau...	1	1	Le1
..................ugaaagacauggguGgugagau...	3	1	Le1
..................ugaaagacaugggGagugagau...	1	1	Le1
..................Cgaaagacauggguagugagau...	2	1	Le1
..................ugCaagacauggguagugagau...	3	1	Le1
..................ugaaagacauggguagugaUau...	3	1	Le1
..................ugaaagacauggguagugagUu...	3	1	Le1
..................Ngaaagacauggguagugagau...	1	1	Le1
..................ugaaagacaugUguagugagau...	7	1	Le1
..................ugaaagacauggguagugagaC...	32	1	Le1
..................ugaaagacauggguagugagCu...	3	1	Le1
..................ugaaaUacauggguagugagau...	1	1	Le1
..................ugaaagaUauggguagugagau...	4	1	Le1
..................ugaaagacauggguagugagaA...	83	1	Le1
..................ugaaagGcauggguagugagau...	1	1	Le1
..................ugaaagacauggguagugagau...	1721	0	Le1
..................ugaUagacauggguagugagau...	2	1	Le1
..................ugaaUgacauggguagugagau...	13	1	Le1
..................ugaaagacauggguaCugagaug..	1	1	Le1
..................uCaaagacauggguagugagaug..	1	1	Le1
..................ugaaagacauggguCgugagaug..	1	1	Le1
..................ugaaagacauggguagugagaCg..	7	1	Le1
..................ugaaagacaAggguagugagaug..	1	1	Le1
..................Cgaaagacauggguagugagaug..	6	1	Le1
..................ugaaagaUauggguagugagaug..	8	1	Le1
..................ugaaagUcauggguagugagaug..	3	1	Le1
..................ugaaagacauggguagugagUug..	1	1	Le1
..................ugaaagacUuggguagugagaug..	6	1	Le1
..................ugaaUgacauggguagugagaug..	55	1	Le1
..................ugaaagacauggguaguAagaug..	1	1	Le1
..................ugUaagacauggguagugagaug..	1	1	Le1
..................ugaaagacauggguaguUagaug..	4	1	Le1
..................Ggaaagacauggguagugagaug..	3	1	Le1
..................ugCaagacauggguagugagaug..	8	1	Le1
..................ugaaagacauggguagugaUaug..	7	1	Le1
..................ugaaagacauggguagugagCug..	5	1	Le1
..................ugaaagacauggguUgugagaug..	2	1	Le1
..................ugaaagacauggguagugagaug..	4928	0	Le1
..................ugaUagacauggguagugagaug..	7	1	Le1
..................ugaaGgacauggguagugagaug..	42	1	Le1
..................uAaaagacauggguagugagaug..	2	1	Le1
..................ugaaagacauggUuagugagaug..	5	1	Le1
..................ugaaagacauUgguagugagaug..	1	1	Le1
..................Ngaaagacauggguagugagaug..	6	1	Le1
..................ugaaagacauggguagugUgaug..	2	1	Le1
..................ugaaagacauggguagAgagaug..	1	1	Le1
..................ugaaagacauggguaguCagaug..	1	1	Le1
..................ugaaagacauggguagCgagaug..	1	1	Le1
..................Agaaagacauggguagugagaug..	8	1	Le1
..................ugaaagacauggguagugagauC..	4	1	Le1
..................ugaaagacaCggguagugagaug..	1	1	Le1
..................ugaaagacaugUguagugagaug..	10	1	Le1
..................ugaaagacauggguagugagGug..	1	1	Le1
..................ugaaagacauggguagugagauA..	84	1	Le1

```
                  Mature                              Star
aauaaacauuuuuucccaugaaagacauggguagugagauguggcuaagcuagucaucucacuaccuugucuuugacggaaagaauuguaaaagucaguacagaauggaaagu
..................ugaaagacaugggAagugagaug........................................................................ 1     1  Le1
..................ugaaagacCuggguagugagaug........................................................................ 61    1  Le1
..................ugaaagacauCgguagugagaug........................................................................ 1     1  Le1
..................ugaaagacauggguGgugagaug........................................................................ 5     1  Le1
..................ugaaagacauggguagugagauU........................................................................ 137   1  Le1
..................ugaaagacaugCguagugagaug........................................................................ 3     1  Le1
..................ugaaagCcauggguagugagaug........................................................................ 1     1  Le1
..................ugaaagGcauggguagugagaug........................................................................ 2     1  Le1
..................ugaaCgacauggguagugagaug........................................................................ 1     1  Le1
..................ugaaagacaugggCagugagaug........................................................................ 3     1  Le1
..................ugGaagacauggguagugagaug........................................................................ 1     1  Le1
..................ugaaagacauggguagugagaugC....................................................................... 6     1  Le1
..................ugaaagacauggUuagugagaugu....................................................................... 1     1  Le1
..................ugaaUgacauggguagugagaugu....................................................................... 2     1  Le1
..................ugaaagacauggguagugagauAu....................................................................... 2     1  Le1
..................Agaaagacauggguagugagaugu....................................................................... 1     1  Le1
..................ugaaagacauggguagugagauUu....................................................................... 3     1  Le1
..................ugaaagacauggguagugagaugu....................................................................... 73    0  Le1
..................ugaaagacauggguagugagaugG....................................................................... 1     1  Le1
..................ugaaagacauggguagugagaugAg...................................................................... 1     1  Le1
..................ugaaagacauggguagugagauguA...................................................................... 2     1  Le1
..................ugaaagacauggguagugagaugug...................................................................... 2     0  Le1
...................gaaaUacauggguaguga............................................................................ 1     1  Le1
...................gaaagacauggguaUugag........................................................................... 1     1  Le1
...................gaaagacauggguagugag........................................................................... 1     0  Le1
...................gaaagacauggguagugaga.......................................................................... 4     0  Le1
...................gaaagacaCggguagugaga.......................................................................... 1     1  Le1
...................gaaaUacauggguagugaga.......................................................................... 2     1  Le1
...................gaaagacaugAguagugaga.......................................................................... 1     1  Le1
...................gaaaUacauggguagugagau......................................................................... 2     1  Le1
...................gaaagacauggguagugagau......................................................................... 6     0  Le1
...................gCaagacauggguagugagaug........................................................................ 2     1  Le1
...................gaaaUacauggguagugagaug........................................................................ 1     1  Le1
...................gUaagacauggguagugagaug........................................................................ 1     1  Le1
...................gaaagacauggguagugagauU........................................................................ 1     1  Le1
...................gaaagacauggguagugagaug........................................................................ 17    0  Le1
...................gaaUgacauggguagugagaug........................................................................ 1     1  Le1
...................gaaagacaAggguagugagaug........................................................................ 1     1  Le1
...................gaCagacauggguagugagaug........................................................................ 1     1  Le1
...................gaaagacauggguaUugagaug........................................................................ 1     1  Le1
...................gaaagacauggguCgugagaug........................................................................ 1     1  Le1
....................aaagacauggguagugaga.......................................................................... 1     0  Le1
....................aaagacauggguagugagau......................................................................... 1     0  Le1
....................aaagacauggguagugagaug........................................................................ 3     0  Le1
....................aaagUcauggguagugagaug........................................................................ 1     1  Le1
.....................aagacauggguagugaga.......................................................................... 1     0  Le1
.....................aagacauggguagugagaug........................................................................ 2     0  Le1
......................................................caucucacuaccuugucuuug...................................... 3     0  Le1
......................................................Aaucucacuaccuugucuuugacg................................... 2     1  Le1
.......................................................aucucacuaccuugucuuug...................................... 4     0  Le1
.......................................................aucucacuCccuugucuuug...................................... 1     1  Le1
.......................................................aucucacuaccuugucuuuga..................................... 2     0  Le1
.......................................................aucucacuaccuugucuuugacg................................... 1     0  Le1
.......................................................Cucucacuaccuugucuuugacg................................... 8     1  Le1
........................................................ucucacuaccuugucuuu....................................... 3     0  Le1
........................................................ucucacuaccuugucuuA....................................... 1     1  Le1
........................................................ucucacuaccuugucuuuA...................................... 3     1  Le1
........................................................ucucaUuaccuugucuuug...................................... 1     1  Le1
........................................................ucucacuUccuugucuuug...................................... 2     1  Le1
........................................................ucucacuaccuugucuuuU...................................... 1     1  Le1
........................................................Acucacuaccuugucuuug...................................... 1     1  Le1
........................................................ucucacuaccuugucuuug...................................... 52    0  Le1
........................................................ucucacuaccuugucuuuga..................................... 63    0  Le1
........................................................ucucGcuaccuugucuuuga..................................... 1     1  Le1
........................................................ucucGcuaccuugucuuugac.................................... 1     1  Le1
........................................................ucucacuacAuugucuuugac.................................... 1     1  Le1
........................................................ucucacuaccuugucuuugac.................................... 113   0  Le1
........................................................ucucacuaccuugucuuCgacg................................... 1     1  Le1
........................................................ucucacuaccuCgucuuugacg................................... 3     1  Le1
........................................................ucucacuaccuugucuuugacg................................... 6865  0  Le1
```

	Mature	Star	
aauaaacauuuuuucccaugaaagacauggguagugagauguggcuaagcuagucaucucacuaccuugucuuugacggaaagaauuguaaaagucaguacagaauggaaagu			
....ucucacuaccuugucuuuUacg...................................	25	1	Le1
....ucucacuaccuugCcuuugacg...................................	1	1	Le1
....ucucaUuaccuugucuuugacg...................................	2	1	Le1
....ucucacuaccuugucCuugacg...................................	4	1	Le1
....ucucacuaccuugucuuAgacg...................................	1	1	Le1
....ucucacuaccuugucuuugaUg...................................	6	1	Le1
....ucucacuCccuugucuuugacg...................................	1	1	Le1
....ucucacuaccuuguUuuugacg...................................	1	1	Le1
....ucucaAuaccuugucuuugacg...................................	2	1	Le1
....ucucacuacAuugucuuugacg...................................	7	1	Le1
....ucucGcuaccuugucuuugacg...................................	56	1	Le1
....Ccucacuaccuugucuuugacg...................................	4	1	Le1
....ucucacuaccuugucuuugGcg...................................	6	1	Le1
....Ncucacuaccuugucuuugacg...................................	7	1	Le1
....ucucacuaccuugucuuuAacg...................................	1	1	Le1
....ucucacAaccuugucuuugacg...................................	1	1	Le1
....Gcucacuaccuugucuuugacg...................................	1	1	Le1
....ucCcacuaccuugucuuugacg...................................	21	1	Le1
....ucucacuaccuugucuuugacA...................................	278	1	Le1
....uGucacuaccuugucuuugacg...................................	4	1	Le1
....ucuGacuaccuugucuuugacg...................................	1	1	Le1
....ucucacuaccuuguGuuugacg...................................	2	1	Le1
....ucucacuaccuugucGuugacg...................................	9	1	Le1
....ucuUacuaccuugucuuugacg...................................	19	1	Le1
....ucucacuaAcuugucuuugacg...................................	27	1	Le1
....ucucacuaccuugucuuugUcg...................................	5	1	Le1
....ucuAacuaccuugucuuugacg...................................	1	1	Le1
....uUucacuaccuugucuuugacg...................................	4	1	Le1
....ucucacuaccAugucuuugacg...................................	6	1	Le1
....ucucacuaccuuAucuuugacg...................................	1	1	Le1
....ucucacuaccuuguAuuugacg...................................	1	1	Le1
....ucuCcuaccuugucuuugacg....................................	1	1	Le1
....ucucacuaccuugucuuugacC...................................	15	1	Le1
....ucucacuaccuGgucuuugacg...................................	1	1	Le1
....ucucacuaccuugucuuugaGg...................................	2	1	Le1
....Acucacuaccuugucuuugacg...................................	9	1	Le1
....uAucacuaccuugucuuugacg...................................	14	1	Le1
....ucucacuGccuugucuuugacg...................................	1	1	Le1
....ucucUcuaccuugucuuugacg...................................	70	1	Le1
....ucucacuaccuugucuuugCcg...................................	2	1	Le1
....ucucacuacUuugucuuugacg...................................	10	1	Le1
....ucucacGaccuugucuuugacg...................................	3	1	Le1
....ucucacuUccuugucuuugacg...................................	10	1	Le1
....ucucacuaccuugucuuugacU...................................	24	1	Le1
....ucucacuaccuugucuAugacg...................................	1	1	Le1
....ucucacuaccuugucuuugacgA..................................	16	1	Le1
....ucucacuaccuugucuuugacgU..................................	120	1	Le1
....ucucacuaccuugucuuugacgg..................................	1	0	Le1
....ucucacuaccuugucuuugacgC..................................	10	1	Le1
....ucucacuaccuugucuuugacgUa.................................	1	1	Le1
....ucucacuaccuugucuuugacgUaa................................	1	1	Le1
....ucucacuaccuugucuuugacgUaaag..............................	1	1	Le1
....cucacuaccuugucuuug.......................................	1	0	Le1
....cucacuaccuugucuuugac.....................................	2	0	Le1
....cucacuacUuugucuuugacg...................................	1	1	Le1
....cucacuaccuugAcuuugacg...................................	1	1	Le1
....Aucacuaccuugucuuugacg...................................	3	1	Le1
....cucUcuaccuugucuuugacg...................................	1	1	Le1
....cucaGuaccuugucuuugacg...................................	2	1	Le1
....cucacuaccuugucuuuUacg...................................	2	1	Le1
....cucacuaccuugucuuugacg...................................	85	0	Le1
....cucaUuaccuugucuuugacg...................................	1	1	Le1
....cucCcuaccuugucuuugacg...................................	1	1	Le1
....cucacuaccuuUucuuugacg...................................	2	1	Le1
....cucacuaAcuugucuuugacg...................................	1	1	Le1
....cucacuaccuugucuuugacC...................................	1	1	Le1
....cucacuaccuugucuuugCcg...................................	1	1	Le1
....cucacuaccuugucuuugacA...................................	3	1	Le1
....cuAacuaccuugucuuugacg...................................	1	1	Le1
....cucacuaccuuguUuuugacg...................................	1	1	Le1

```
                 Mature                      Star
aauaaacauuuuuucccaugaaagacauggguagugagauguggcuaagcuagucaucucacuaccuugucuuugacggaaagaauuguaaaagucaguacagaauggaaagu
.........................................................cucacuaccuugucuuugacgU..................................    3    1    Le1
..........................................................ucacuaccuugucuuugac....................................    1    0    Le1
..........................................................ucacuaccuugucAuugacg...................................    1    1    Le1
..........................................................ucacCaccuugucuuugacg...................................    1    1    Le1
..........................................................ucacuaccuugucuuugacU...................................    2    1    Le1
..........................................................ucacuaccuugucuuugacg...................................    27   0    Le1

.................augaaagacauggguagug.............................................................................    1    0    Br1
.................augaaagacauggguaguga............................................................................    1    0    Br1
.................Cugaaagacauggguagugaga..........................................................................    1    1    Br1
.................augaaagacauggguagugaga..........................................................................    3    0    Br1
.................Cugaaagacauggguagugagau.........................................................................    3    1    Br1
.................augaaagacauggguagugagau.........................................................................    3    0    Br1
.................augaaagacauggguagugagaug........................................................................    9    0    Br1
.................augaaagaAauggguagugagaug........................................................................    1    1    Br1
..................ugaaagacCuggguagug.............................................................................    1    1    Br1
..................ugaaagacauggguagug.............................................................................    36   0    Br1
..................ugaaagacauggguaguA.............................................................................    5    1    Br1
..................ugaaagacauggguaguAa............................................................................    1    1    Br1
..................ugaaagacauggguagugU............................................................................    1    1    Br1
..................Ngaaagacauggguaguga............................................................................    1    1    Br1
..................ugaaGgacauggguaguga............................................................................    2    1    Br1
..................ugaaagacCuggguaguga............................................................................    2    1    Br1
..................ugaaagacauggguaguga............................................................................    107  0    Br1
..................ugaaUgacauggguaguga............................................................................    3    1    Br1
..................ugaaagacauggguagugC............................................................................    1    1    Br1
..................ugaaagacauggguagugag...........................................................................    134  0    Br1
..................ugaUagacauggguagugag...........................................................................    2    1    Br1
..................Cgaaagacauggguagugag...........................................................................    1    1    Br1
..................ugaaagacauggguagugaU...........................................................................    1    1    Br1
..................ugaaagacauggguagugaA...........................................................................    14   1    Br1
..................ugaaagacCuggguagugag...........................................................................    1    1    Br1
..................ugaaagUcauggguagugag...........................................................................    1    1    Br1
..................ugaaUgacauggguagugag...........................................................................    3    1    Br1
..................ugaaagacauggguagugagG..........................................................................    5    1    Br1
..................ugaaUgacauggguagugaga..........................................................................    15   1    Br1
..................ugaaagacauggguagugaUa..........................................................................    3    1    Br1
..................ugaaagacauggguagugagC..........................................................................    3    1    Br1
..................ugaGagacauggguagugaga..........................................................................    1    1    Br1
..................Cgaaagacauggguagugaga..........................................................................    4    1    Br1
..................ugaaagacauggguagugaAa..........................................................................    4    1    Br1
..................ugaaagacauggguagAgaga..........................................................................    1    1    Br1
..................ugaaGgacauggguagugaga..........................................................................    13   1    Br1
..................ugaaagacaugggguGgugaga..........................................................................    1    1    Br1
..................ugaaagacaugUguagugaga..........................................................................    6    1    Br1
..................ugaaagacauggguagugagU..........................................................................    10   1    Br1
..................ugaaagacaugggguUgugaga..........................................................................    1    1    Br1
..................ugaaagacauggguagugaCa..........................................................................    2    1    Br1
..................Agaaagacauggguagugaga..........................................................................    3    1    Br1
..................ugaaagacauggguaguUaga..........................................................................    2    1    Br1
..................ugaaagaUauggguagugaga..........................................................................    2    1    Br1
..................ugaaagacCuggguagugaga..........................................................................    13   1    Br1
..................ugaaagacaAgguagugaga..........................................................................    1    1    Br1
..................ugaaagGcauggguagugaga..........................................................................    1    1    Br1
..................ugaaagacauggguaUugaga..........................................................................    1    1    Br1
..................ugCaagacauggguagugaga..........................................................................    2    1    Br1
..................ugaUagacauggguagugaga..........................................................................    4    1    Br1
..................ugaaagacauggguagugaga..........................................................................    1018 0    Br1
..................ugaaGgacauggguagugagau.........................................................................    4    1    Br1
..................uAaaagacauggguagugagau.........................................................................    1    1    Br1
..................ugaaagaUauggguagugagau.........................................................................    3    1    Br1
..................uCaaagacauggguagugagau.........................................................................    1    1    Br1
..................Agaaagacauggguagugagau.........................................................................    2    1    Br1
..................ugaaagaAauggguagugagau.........................................................................    1    1    Br1
..................ugaaagacauggguagugaUau.........................................................................    3    1    Br1
..................ugCaagacauggguagugagau.........................................................................    3    1    Br1
..................ugaaagacCuggguagugagau.........................................................................    11   1    Br1
..................ugaaagacauggguagugagaC.........................................................................    19   1    Br1
..................ugaaagacaCggguagugagau.........................................................................    2    1    Br1
..................ugaaagacauggguagugagUu.........................................................................    1    1    Br1
```

```
                  Mature                                      Star
aauaaacauuuuuucccaugaaagacauggguagugagauguggcuaagcuagucaucucacuaccuugucuuugacggaaagaauuguaaaagucaguacagaauggaaagu
..................ugaUagacauggguagugagau.........................................................................    4     1   Br1
..................ugaaagacauggguagugagaA.........................................................................    43    1   Br1
..................ugaaUgacauggguagugagau.........................................................................    9     1   Br1
..................ugUaagacauggguagugagau.........................................................................    1     1   Br1
..................ugaaagacauggguagugagau.........................................................................    849   0   Br1
..................ugaaaUacauggguagugagau.........................................................................    1     1   Br1
..................ugaaagacauggguaguUagau.........................................................................    1     1   Br1
..................Ngaaagacauggguagugagau.........................................................................    1     1   Br1
..................ugaaaUacauggguagugagaug........................................................................    2     1   Br1
..................ugaaagacauggguagugagaug........................................................................    2342  0   Br1
..................ugaaagacaugggCagugagaug........................................................................    1     1   Br1
..................ugaaagacauggguGgugagaug........................................................................    2     1   Br1
..................ugaaagacUuggguagugagaug........................................................................    5     1   Br1
..................ugaaagacauggguagugagauC........................................................................    3     1   Br1
..................ugaaagacauggguagugagaCg........................................................................    5     1   Br1
..................ugaaagacauggguagugagGug........................................................................    1     1   Br1
..................Ggaaagacauggguagugagaug........................................................................    1     1   Br1
..................ugaaagUcauggguagugagaug........................................................................    2     1   Br1
..................uCaaagacauggguagugagaug........................................................................    1     1   Br1
..................ugaaagacauAgguagugagaug........................................................................    1     1   Br1
..................ugaaagacauggUuagugagaug........................................................................    1     1   Br1
..................ugaaagacaugAguagugagaug........................................................................    2     1   Br1
..................ugaaagacauggguagugaUaug........................................................................    3     1   Br1
..................ugaaagacauggguagugagauU........................................................................    107   1   Br1
..................ugaaagacaugUguagugagaug........................................................................    5     1   Br1
..................ugaaagaUauggguagugagaug........................................................................    6     1   Br1
..................ugaaagacaCggguagugagaug........................................................................    1     1   Br1
..................ugaaagacauggAuagugagaug........................................................................    1     1   Br1
..................ugaaagacauggguagugagauA........................................................................    66    1   Br1
..................ugaaagacauggguagugagUug........................................................................    2     1   Br1
..................ugaaagacauggguaguUagaug........................................................................    1     1   Br1
..................ugaaagacaAggguagugagaug........................................................................    1     1   Br1
..................Cgaaagacauggguagugagaug........................................................................    3     1   Br1
..................Ngaaagacauggguagugagaug........................................................................    2     1   Br1
..................ugaaagacauggguaUugagaug........................................................................    1     1   Br1
..................ugaaGgacauggguagugagaug........................................................................    21    1   Br1
..................ugaaagacauggguagugagaAg........................................................................    1     1   Br1
..................ugaaagacaugggAagugagaug........................................................................    2     1   Br1
..................uAaaagacauggguagugagaug........................................................................    2     1   Br1
..................ugaaUgacauggguagugagaug........................................................................    21    1   Br1
..................ugaaagacauggguUgugagaug........................................................................    1     1   Br1
..................ugaaagacauggguagugagCug........................................................................    2     1   Br1
..................ugaaagacauggguagugUgaug........................................................................    2     1   Br1
..................ugaaagacCuggguagugagaug........................................................................    42    1   Br1
..................ugaUagacauggguagugagaug........................................................................    5     1   Br1
..................ugCaagacauggguagugagaug........................................................................    5     1   Br1
..................ugaaGgacauggguagugagaugu.......................................................................    1     1   Br1
..................ugaaagacauggguagugagaugu.......................................................................    59    0   Br1
..................ugaaagacCuggguagugagaugu.......................................................................    2     1   Br1
..................ugaaagacauggguagugagauUu.......................................................................    1     1   Br1
..................ugaaagacauggguagugagaugC.......................................................................    2     1   Br1
..................ugaaagacauggguagugagauguA......................................................................    2     1   Br1
..................ugaaagacauggguagugagauguggc....................................................................    1     0   Br1
...................gaaagacauggguagugag...........................................................................    1     0   Br1
...................gaaagacauggguagugaga..........................................................................    7     0   Br1
...................gaaaUacauggguagugaga..........................................................................    2     1   Br1
...................gaaagacUuggguagugagau.........................................................................    1     1   Br1
...................gaaagacauggguCgugagau.........................................................................    1     1   Br1
...................gaaagacauggguagugagau.........................................................................    2     0   Br1
...................gaaaUacauggguagugagaug........................................................................    1     1   Br1
...................gaaUgacauggguagugagaug........................................................................    3     1   Br1
...................Aaaagacauggguagugagaug........................................................................    1     1   Br1
...................gaaagacauggguagugagaug........................................................................    8     0   Br1
...................gaaagacauggguCgugagaug........................................................................    1     1   Br1
...................gaaagacaAggguagugagaug........................................................................    1     1   Br1
.......................gacauggguagugagauA........................................................................    1     1   Br1
......................................................caucucacuaccuugucuuu.......................................    1     0   Br1
......................................................caucucacuaccuugucuuuga.....................................    1     0   Br1
.......................................................aucucacuaccuugucuuugacA...................................    1     1   Br1
.......................................................Cucucacuaccuugucuuugacg...................................    6     1   Br1
```

Mature Star

```
aauaaacauuuuuucccaugaaagacaugguagugagauguggcuaagcuagucaucucacuaccuugucuuugacggaaagaauuguaaaagucaguacagaauggaaagu
......................................................aucucacuaccuugucuuugacg...................................    2     0    Br1
.......................................................ucucacuaccuugucuuu.......................................    2     0    Br1
.......................................................ucucGcuaccuugucuuug......................................    1     1    Br1
.......................................................ucucacuaccuugucuuug......................................    32    0    Br1
.......................................................ucucacuaccuugucuuuU......................................    1     1    Br1
.......................................................ucucacuaccuuguUuuug......................................    1     1    Br1
.......................................................ucucUcuaccuugucuuuga.....................................    1     1    Br1
.......................................................ucucacuaccuugucuuuga.....................................    91    0    Br1
.......................................................ucucGcuaccuugucuuuga.....................................    2     1    Br1
.......................................................ucucacuaccuugucuuugUc....................................    1     1    Br1
.......................................................ucucacuaccuugucuuuUac....................................    2     1    Br1
.......................................................ucucacuaccuugucGuugac....................................    1     1    Br1
.......................................................ucucacuaccuugucuuugac....................................    130   0    Br1
.......................................................ucucacGaccuugucuuugac....................................    1     1    Br1
.......................................................ucucacuaccuugucuuugCcg...................................    1     1    Br1
.......................................................ucucacuaccuugucuuugaUg...................................    8     1    Br1
.......................................................ucucacuaccuugAcuuugacg...................................    3     1    Br1
.......................................................ucucacuaccuugucuuugacg...................................    6618  0    Br1
.......................................................ucucacuaccuugucuuugGcg...................................    1     1    Br1
.......................................................ucucacuaccuuguUuuugacg...................................    1     1    Br1
.......................................................ucucacuaccCugucuuugacg...................................    3     1    Br1
.......................................................uUucacuaccuugucuuugacg...................................    3     1    Br1
.......................................................ucucCcuaccuugucuuugacg...................................    1     1    Br1
.......................................................ucucacuaccuugucGuugacg...................................    5     1    Br1
.......................................................ucucacuaccuuguGuuugacg...................................    1     1    Br1
.......................................................ucucacuaccuugCcuuugacg...................................    1     1    Br1
.......................................................ucucacuaccuugucuuugUcg...................................    6     1    Br1
.......................................................Acucacuaccuugucuuugacg...................................    13    1    Br1
.......................................................Ncucacuaccuugucuuugacg...................................    4     1    Br1
.......................................................ucucacuaccuugucuuugacA...................................    310   1    Br1
.......................................................ucucacGaccuugucuuugacg...................................    5     1    Br1
.......................................................ucucacuaccuugucCuugacg...................................    10    1    Br1
.......................................................ucucacuaccuugucuuuUacg...................................    24    1    Br1
.......................................................ucucacuaccuugucuuugacU...................................    26    1    Br1
.......................................................ucucacCaccuugucuuugacg...................................    3     1    Br1
.......................................................ucucacuaccAugucuuugacg...................................    6     1    Br1
.......................................................ucucacuaccuCgucuuugacg...................................    2     1    Br1
.......................................................ucCcacuaccuugucuuugacg...................................    12    1    Br1
.......................................................ucucacuaccuugucuuugacC...................................    10    1    Br1
.......................................................ucucacuaccuuAucuuugacg...................................    4     1    Br1
.......................................................ucucaAuaccuugucuuugacg...................................    1     1    Br1
.......................................................ucucacuaccuugucuuCgacg...................................    2     1    Br1
.......................................................ucucacuUccuugucuuugacg...................................    15    1    Br1
.......................................................Ccucacuaccuugucuuugacg...................................    13    1    Br1
.......................................................ucucacuaAcuugucuuugacg...................................    14    1    Br1
.......................................................ucucacuaccuugucuCugacg...................................    1     1    Br1
.......................................................ucucUcuaccuugucuuugacg...................................    64    1    Br1
.......................................................ucucaUuaccuugucuuugacg...................................    5     1    Br1
.......................................................uGucacuaccuugucuuugacg...................................    1     1    Br1
.......................................................ucucacuacUuugucuuugacg...................................    7     1    Br1
.......................................................ucucacuaccuuUucuuugacg...................................    1     1    Br1
.......................................................ucucacuaccuugucuuGgacg...................................    2     1    Br1
.......................................................ucucacuaccuGgucuuugacg...................................    3     1    Br1
.......................................................ucucacuaGcuugucuuugacg...................................    2     1    Br1
.......................................................Gcucacuaccuugucuuugacg...................................    1     1    Br1
.......................................................uAucacuaccuugucuuugacg...................................    11    1    Br1
.......................................................ucucGcuaccuugucuuugacg...................................    49    1    Br1
.......................................................ucucacuacAuugucuuugacg...................................    3     1    Br1
.......................................................ucuUacuaccuugucuuugacg...................................    16    1    Br1
.......................................................ucucacuaccuugucuAugacg...................................    2     1    Br1
.......................................................ucucacuaccuugucuuugacAg..................................    1     1    Br1
.......................................................ucucacuaccuugucuuugacgA..................................    14    1    Br1
.......................................................ucucacuaccuugucuuugacgU..................................    122   1    Br1
.......................................................ucucacuaccuugucuuugacgg..................................    1     0    Br1
.......................................................ucucacuaccuugucuuugacgC..................................    5     1    Br1
.......................................................ucucacuaccuugucuuugacgUa.................................    5     1    Br1
.......................................................ucucacuaccuugucuuugacgga.................................    1     0    Br1
........................................................cucacuaccuugucuuuga.....................................    1     0    Br1
........................................................cucacuaccuugucuuugac....................................    1     0    Br1
........................................................cuAacuaccuugucuuugac....................................    1     1    Br1
```

```
                  Mature                                  Star
aauaaacauuuuuucccaugaaagacauggguagugagauguggcuaagcuagucaucucacuaccuugucuuugacggaaagaauuguaaaagucaguacagaauggaaagu
.........................................................cucaUuaccuugucuuugacg...................................    5    1    Br1
.........................................................cucaGuaccuugucuuugacg...................................    1    1    Br1
.........................................................cucacAaccuugucuuugacg...................................    1    1    Br1
.........................................................cucUcuaccuugucuuugacg...................................    1    1    Br1
.........................................................cucacuacUuugucuuugacg...................................    2    1    Br1
.........................................................cucacuaccuAgucuuugacg...................................    1    1    Br1
.........................................................cucacuaccuugucuuugacA...................................    2    1    Br1
.........................................................cucacuaccuugucuuugacg...................................    92   0    Br1
.........................................................cucacuaccuuUucuuugacg...................................    6    1    Br1
.........................................................cuAacuaccuugucuuugacg...................................    2    1    Br1
.........................................................cucacuaccAugucuuugacg...................................    1    1    Br1
.........................................................cucacuaccuugCcuuugacg...................................    1    1    Br1
.........................................................Aucacuaccuugucuuugacg...................................    2    1    Br1
.........................................................cucacuaccuugucuuugacgU..................................    1    1    Br1
..........................................................ucacGaccuugucuuugacg...................................    1    1    Br1
..........................................................ucacCaccuugucuuugacg...................................    1    1    Br1
..........................................................ucacuaccuugucuuugacg...................................    12   0    Br1
...........................................................cacuaccuugucuuugacg...................................    3    0    Br1
```

Provisional ID : Sc28yqQ_6159_3358
Score total : 5
Score for star read(s) : -1.3
Score for read counts : 0
Score for mfe : 1.7
Score for randfold : 1.6
Score for cons. seed : 3
Total read count : 981738
Mature read count : 981710
Loop read count : 0
Star read count : 28

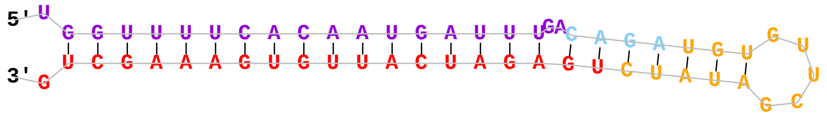

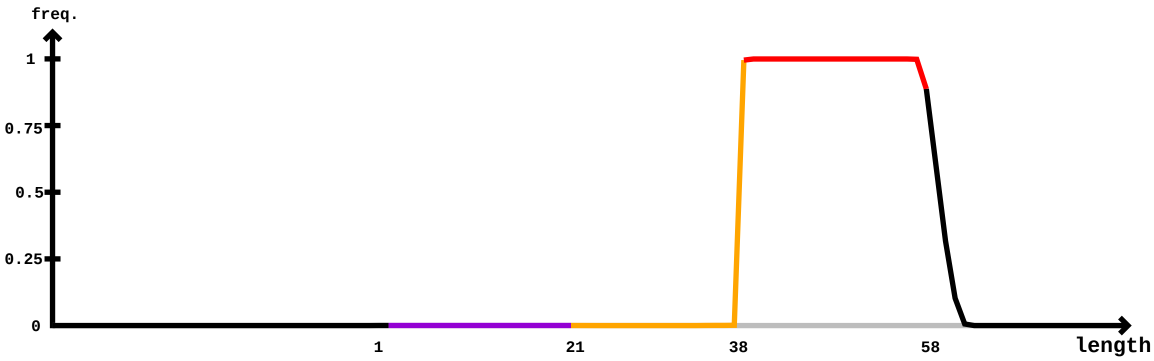

Star Mature

```
5'- ggaacucgaaagcgcccuagcuguagacaaaacugguuuucacaaugauuugacagauguguucgauaucugagaucauugugaaagcugauuuuguugucccaucaucu  -3'   obs
    ggaacucgaaagcgcccuagcuguagacaaaacugguuuucacaaugauuugacagauguguucgauaucugagaucauugugaaagcugauuuuguugucccaucaucu         exp
    (((.......(((......)))...(((((((..(((((((((((((((((..(((((((.....))))))))))))))))))))))))))..))))))).)))........    reads   mm   sample
    ................................cugguuuucacaaugauuuga.........................................................    4       0    Ta1
    ................................cugguuuucacaaugauuugac........................................................    4       0    Ta1
    ................................cugguuuucacaaugauuugaca.......................................................    3       0    Ta1
    ..................................................................uaucugagaucauugugaaag.......................    8       0    Ta1
    ..................................................................uCucugagaucauugugaaag.......................    1       1    Ta1
    ..................................................................uaucAgagaucauugugaaagc......................    1       1    Ta1
    ..................................................................uaucugagaucauugugaaagc......................    23      0    Ta1
    ..................................................................uaucGgagaucauugugaaagc......................    1       1    Ta1
    ..................................................................uaucugagCucauugugaaagcu.....................    1       1    Ta1
    ..................................................................uaucugagaucauugugaaagcA.....................    5       1    Ta1
    ..................................................................uaucugagaucauugugaaagcu.....................    15      0    Ta1
    ...................................................................aucugagaucauugugaaagcu.....................    9       0    Ta1
    ...................................................................aucuUagaucauugugaaagcu.....................    1       1    Ta1
    ...................................................................aucugagaucauugugaaagcC.....................    1       1    Ta1
    ...................................................................Gucugagaucauugugaaagcug....................    1       1    Ta1
    ...................................................................aucugagaucauugugaaagcug....................    2       0    Ta1
    ...................................................................aucugagaucauugugaaagcuU....................    7       1    Ta1
    ....................................................................ucugagaucauugugaaagcuU....................    4       1    Ta1
    .....................................................................cugagaucauugugaaagcu.....................    5       0    Ta1
    .....................................................................cugaUaucauugugaaagcug....................    1       1    Ta1
    .....................................................................cugagaucauugugaaagcug....................    13      0    Ta1
    .....................................................................cugagaucauugugaaagcuga...................    8       0    Ta1
    .....................................................................Augagaucauugugaaagcuga...................    1       1    Ta1
    .....................................................................cugagaucauugugaaagcugU...................    9       1    Ta1
    .....................................................................cugagaucauugugaaagcugau..................    4       0    Ta1
    .....................................................................cugaUaucauugugaaagcugau..................    1       1    Ta1
    ......................................................................ugagaucauugugaaagc......................    25      0    Ta1
    ......................................................................ugagaAcauugugaaagcu.....................    1       1    Ta1
    ......................................................................ugagGucauugugaaagcu.....................    116     1    Ta1
    ......................................................................ugagaucauugugCaagcu.....................    25      1    Ta1
    ......................................................................ugagaucauCgugaaagcu.....................    7       1    Ta1
    ......................................................................Ggagaucauugugaaagcu.....................    5       1    Ta1
    ......................................................................ugaUaucauugugaaagcu.....................    27      1    Ta1
```

Star Mature

```
ggaacucgaaagcgcccuagcuguagacaaaacugguuuucacaaugauuugacagauguguucgauaucugagaucauugugaaagcugauuuuguuguccccaucaucu
......................................................................ugagaucUuugugaaagcu...................... 26 1 Ta1
......................................................................ugCgaucauugugaaagcu...................... 18 1 Ta1
......................................................................ugagaucauugugaUagcu...................... 3 1 Ta1
......................................................................Cgagaucauugugaaagcu...................... 8 1 Ta1
......................................................................ugagaucauAgugaaagcu...................... 2 1 Ta1
......................................................................ugagaucauugugaaUgcu...................... 7 1 Ta1
......................................................................ugagaucauugugaCagcu...................... 1 1 Ta1
......................................................................ugGgaucauugugaaagcu...................... 1 1 Ta1
......................................................................ugagaucauugugaaagcG...................... 4 1 Ta1
......................................................................ugagaucGuugugaaagcu...................... 4 1 Ta1
......................................................................uCagaucauugugaaagcu...................... 4 1 Ta1
......................................................................ugagaucauugugaaaAcu...................... 2 1 Ta1
......................................................................ugagUucauugugaaagcu...................... 160 1 Ta1
......................................................................ugUgaucauugugaaagcu...................... 2 1 Ta1
......................................................................ugagaucauuguAaaagcu...................... 3 1 Ta1
......................................................................ugagaucauugGgaaagcu...................... 2 1 Ta1
......................................................................ugagaucauuAugaaagcu...................... 1 1 Ta1
......................................................................ugagaucauugugaaagcu...................... 9474 0 Ta1
......................................................................ugagaucauuUugaaagcu...................... 1 1 Ta1
......................................................................Agagaucauugugaaagcu...................... 15 1 Ta1
......................................................................uAagaucauugugaaagcu...................... 6 1 Ta1
......................................................................ugagaucauGgugaaagcu...................... 1 1 Ta1
......................................................................ugagaucauugCgaaagcu...................... 1 1 Ta1
......................................................................ugagaCcauugugaaagcu...................... 1 1 Ta1
......................................................................ugagaucauugugaaagcC...................... 111 1 Ta1
......................................................................ugagaucaAugugaaagcu...................... 24 1 Ta1
......................................................................ugagaGcauugugaaagcu...................... 3 1 Ta1
......................................................................ugagaucauugugaaagUu...................... 7 1 Ta1
......................................................................ugagaucauugugaaagcA...................... 55 1 Ta1
......................................................................ugagaucauugugaGagcu...................... 1 1 Ta1
......................................................................ugagauUauugugaaagcu...................... 3 1 Ta1
......................................................................Ngagaucauugugaaagcu...................... 4 1 Ta1
......................................................................ugagaucauugugaaGgcu...................... 3 1 Ta1
......................................................................ugagaucauugAgaaagcu...................... 1 1 Ta1
......................................................................ugaCaucauugugaaagcu...................... 1 1 Ta1
......................................................................ugagaucauuCugaaagcu...................... 1 1 Ta1
......................................................................ugagaucauugugGaagcu...................... 1 1 Ta1
......................................................................ugagauGauugugaaagcu...................... 2 1 Ta1
......................................................................uUagaucauugugaaagcu...................... 7 1 Ta1
......................................................................ugagaucauugugUaagcu...................... 2 1 Ta1
......................................................................ugagaucauuguAaaagcug..................... 5 1 Ta1
......................................................................ugagaucauugugaaagcug..................... 40148 0 Ta1
......................................................................ugagaucauugugaaGgcug..................... 11 1 Ta1
......................................................................ugagauAauugugaaagcug..................... 5 1 Ta1
......................................................................uUagaucauugugaaagcug..................... 9 1 Ta1
......................................................................ugagGucauugugaaagcug..................... 498 1 Ta1
......................................................................ugagaucauugugaaaUcug..................... 6 1 Ta1
......................................................................ugagUucauugugaaagcug..................... 685 1 Ta1
......................................................................ugagaucauugugUaagcug..................... 8 1 Ta1
......................................................................ugagCucauugugaaagcug..................... 10 1 Ta1
......................................................................ugagaucauugugaaagUug..................... 36 1 Ta1
......................................................................ugagaucaAugugaaagcug..................... 65 1 Ta1
......................................................................ugagaucauugGgaaagcug..................... 10 1 Ta1
......................................................................ugagaucauugugaCagcug..................... 2 1 Ta1
......................................................................uAagaucauugugaaagcug..................... 29 1 Ta1
......................................................................ugagaucauuUugaaagcug..................... 2 1 Ta1
......................................................................ugaUaucauugugaaagcug..................... 93 1 Ta1
......................................................................ugagaucauugugaaCgcug..................... 5 1 Ta1
......................................................................ugagaucauGgugaaagcug..................... 1 1 Ta1
......................................................................ugagaucauuguCaaagcug..................... 3 1 Ta1
......................................................................ugGgaucauugugaaagcug..................... 11 1 Ta1
......................................................................ugUgaucauugugaaagcug..................... 8 1 Ta1
......................................................................ugagaucauugugaGagcug..................... 6 1 Ta1
......................................................................ugagaAcauugugaaagcug..................... 6 1 Ta1
......................................................................ugagaucUuugugaaagcug..................... 107 1 Ta1
......................................................................ugagaGcauugugaaagcug..................... 15 1 Ta1
......................................................................ugagaucauugugaaagcGg..................... 18 1 Ta1
......................................................................ugagaucauugugaaagcAg..................... 2 1 Ta1
......................................................................ugagaucGuugugaaagcug..................... 3 1 Ta1
......................................................................ugagaucauugAgaaagcug..................... 3 1 Ta1
```

```
                                  Star                              Mature
ggaacucgaaagcgcccuagcuguagacaaaacugguuuucacaaugauuugacagauguguucgauaucugagaucauugugaaagcugauuuuguuguccccaucaucu
......................................................................ugagaucauugCgaaagcug.....................   11   1   Ta1
......................................................................ugagaucauugugaaagcCg.....................   15   1   Ta1
......................................................................Ngagaucauugugaaagcug.....................   37   1   Ta1
......................................................................ugCgaucauugugaaagcug.....................   106  1   Ta1
......................................................................ugagaucCuugugaaagcug.....................   4    1   Ta1
......................................................................Ggagaucauugugaaagcug.....................   7    1   Ta1
......................................................................ugagaucauugugaaUgcug.....................   41   1   Ta1
......................................................................ugagaucauugugaaaCcug.....................   1    1   Ta1
......................................................................ugagaucauugugGaagcug.....................   3    1   Ta1
......................................................................ugagauUauugugaaagcug.....................   9    1   Ta1
......................................................................ugagaucauugugaaagcuA.....................   51   1   Ta1
......................................................................ugagaucauuguUaaagcug.....................   6    1   Ta1
......................................................................ugagaucauuCugaaagcug.....................   1    1   Ta1
......................................................................ugagaucauuAugaaagcug.....................   16   1   Ta1
......................................................................ugagaucauugugaaagcuU.....................   110  1   Ta1
......................................................................Agagaucauugugaaagcug.....................   77   1   Ta1
......................................................................ugagaucaGugugaaagcug.....................   3    1   Ta1
......................................................................ugagaucauugugCaagcug.....................   160  1   Ta1
......................................................................ugagaucauCgugaaagcug.....................   3    1   Ta1
......................................................................Cgagaucauugugaaagcug.....................   75   1   Ta1
......................................................................ugagauGauugugaaagcug.....................   3    1   Ta1
......................................................................ugagaucauugugaUagcug.....................   5    1   Ta1
......................................................................ugagaucauugugaaaAcug.....................   5    1   Ta1
......................................................................ugagaCcauugugaaagcug.....................   11   1   Ta1
......................................................................ugagaucauugugaaagcuC.....................   4    1   Ta1
......................................................................ugagaucauAgugaaagcug.....................   3    1   Ta1
......................................................................ugaAaucauugugaaagcug.....................   2    1   Ta1
......................................................................uCagaucauugugaaagcug.....................   12   1   Ta1
......................................................................Ngagaucauugugaaagcuga....................   7    1   Ta1
......................................................................Agagaucauugugaaagcuga....................   16   1   Ta1
......................................................................ugagaucauugugaaagcAga....................   4    1   Ta1
......................................................................Ggagaucauugugaaagcuga....................   2    1   Ta1
......................................................................ugagaAcauugugaaagcuga....................   1    1   Ta1
......................................................................ugagaucauugugaUagcuga....................   2    1   Ta1
......................................................................uUagaucauugugaaagcuga....................   3    1   Ta1
......................................................................ugagaucauugugaaagUuga....................   2    1   Ta1
......................................................................ugagaucauugugCaagcuga....................   16   1   Ta1
......................................................................ugagUucauugugaaagcuga....................   119  1   Ta1
......................................................................Cgagaucauugugaaagcuga....................   7    1   Ta1
......................................................................ugGgaucauugugaaagcuga....................   3    1   Ta1
......................................................................ugagauGauugugaaagcuga....................   1    1   Ta1
......................................................................ugagaucCuugugaaagcuga....................   1    1   Ta1
......................................................................ugagaucaAugugaaagcuga....................   7    1   Ta1
......................................................................ugagaucauugugGaagcuga....................   1    1   Ta1
......................................................................ugaUaucauugugaaagcuga....................   15   1   Ta1
......................................................................ugagaucauugugaaaAcuga....................   1    1   Ta1
......................................................................ugagaucauugugaaagcugG....................   1    1   Ta1
......................................................................ugagaucauuguCaaagcuga....................   1    1   Ta1
......................................................................ugagaucUuugugaaagcuga....................   17   1   Ta1
......................................................................ugagaucauuUugaaagcuga....................   8    1   Ta1
......................................................................ugagaGcauugugaaagcuga....................   5    1   Ta1
......................................................................ugagaucauGgugaaagcuga....................   1    1   Ta1
......................................................................ugagCucauugugaaagcuga....................   1    1   Ta1
......................................................................ugagaucauugugUaagcuga....................   3    1   Ta1
......................................................................ugagGucauugugaaagcuga....................   92   1   Ta1
......................................................................ugagaucauugugaaagcugU....................   92   1   Ta1
......................................................................ugCgaucauugugaaagcuga....................   32   1   Ta1
......................................................................uAagaucauugugaaagcuga....................   5    1   Ta1
......................................................................ugagaucauugugaaagcugC....................   4    1   Ta1
......................................................................ugagaucauugugaGagcuga....................   3    1   Ta1
......................................................................uCagaucauugugaaagcuga....................   3    1   Ta1
......................................................................ugagaucauugugaaGgcuga....................   3    1   Ta1
......................................................................ugagaucauugugaaagcCga....................   2    1   Ta1
......................................................................ugagaucauuAugaaagcuga....................   3    1   Ta1
......................................................................ugagauAauugugaaagcuga....................   1    1   Ta1
......................................................................ugagaucauuCugaaagcuga....................   1    1   Ta1
......................................................................ugagaucGuugugaaagcuga....................   2    1   Ta1
......................................................................ugagaCcauugugaaagcuga....................   2    1   Ta1
......................................................................ugagaucauugugaaUgcuga....................   8    1   Ta1
......................................................................ugagaucauugCgaaagcuga....................   5    1   Ta1
```

```
                                  Star                                     Mature
ggaacucgaaagcgcccuagcuguagacaaaacugguuuucacaaugauuugacagauguguucgauaucugagaucauugugaaagcugauuuuguuguccccaucaucu
......................................................................ugagaucauugugaaagcuga....................    6615    0    Ta1
......................................................................ugagaucauugugaaagcuUa....................    9       1    Ta1
......................................................................ugagaucauugugaaagcGga....................    2       1    Ta1
......................................................................ugagaucauugGgaaagcugau...................    1       1    Ta1
......................................................................ugaCaucauugugaaagcugau...................    1       1    Ta1
......................................................................ugagaucauugugaaagcugau...................    10368   0    Ta1
......................................................................ugagaucauugugaaagUugau...................    6       1    Ta1
......................................................................ugagaucauugugaaagcugaA...................    153     1    Ta1
......................................................................ugagaucauAgugaaagcugau...................    1       1    Ta1
......................................................................Cgagaucauugugaaagcugau...................    23      1    Ta1
......................................................................ugagaucauCgugaaagcugau...................    2       1    Ta1
......................................................................Ggagaucauugugaaagcugau...................    3       1    Ta1
......................................................................ugagaucauugugaaagcCgau...................    7       1    Ta1
......................................................................ugagaucauugugaaagcuUau...................    19      1    Ta1
......................................................................ugagaucauugugaaaUcugau...................    1       1    Ta1
......................................................................ugagaucaAugugaaagcugau...................    12      1    Ta1
......................................................................ugagaucauugCgaaagcugau...................    2       1    Ta1
......................................................................ugagaucauuAugaaagcugau...................    2       1    Ta1
......................................................................ugagaAcauugugaaagcugau...................    1       1    Ta1
......................................................................ugagaucauugugaaUgcugau...................    9       1    Ta1
......................................................................ugagaucauugugaaagcugCu...................    4       1    Ta1
......................................................................ugagaucauugugaaagcugaG...................    9       1    Ta1
......................................................................ugagaucUuugugaaagcugau...................    26      1    Ta1
......................................................................ugGgaucauugugaaagcugau...................    1       1    Ta1
......................................................................ugagaucauugugaaagcuCau...................    1       1    Ta1
......................................................................uAagaucauugugaaagcugau...................    5       1    Ta1
......................................................................ugagUucauugugaaagcugau...................    178     1    Ta1
......................................................................ugagaucauuguUaaagcugau...................    4       1    Ta1
......................................................................Ngagaucauugugaaagcugau...................    8       1    Ta1
......................................................................ugagaucauugugCaagcugau...................    47      1    Ta1
......................................................................ugagaucauugugaGagcugau...................    2       1    Ta1
......................................................................Agagaucauugugaaagcugau...................    14      1    Ta1
......................................................................ugagauUauugugaaagcugau...................    5       1    Ta1
......................................................................ugagaucauuguCaaagcugau...................    1       1    Ta1
......................................................................ugagaucauugugaaagcugUu...................    15      1    Ta1
......................................................................ugagaucauugugaaagcugGu...................    3       1    Ta1
......................................................................ugagCucauugugaaagcugau...................    3       1    Ta1
......................................................................ugagaucauugugaaagcuAau...................    5       1    Ta1
......................................................................ugagaucauugugGaagcugau...................    1       1    Ta1
......................................................................uUagaucauugugaaagcugau...................    1       1    Ta1
......................................................................ugagaucauuCugaaagcugau...................    2       1    Ta1
......................................................................ugagaucauugugaaagAugau...................    1       1    Ta1
......................................................................ugagGucauugugaaagcugau...................    128     1    Ta1
......................................................................uCagaucauugugaaagcugau...................    4       1    Ta1
......................................................................ugaAaucauugugaaagcugau...................    1       1    Ta1
......................................................................ugagaucauugugaaagcugaC...................    24      1    Ta1
......................................................................ugagaucauugugaaagcGgau...................    7       1    Ta1
......................................................................ugagaucauuguAaaagcugau...................    3       1    Ta1
......................................................................ugagaucauugugaaagcAgau...................    1       1    Ta1
......................................................................ugagaucauugugaCagcugau...................    1       1    Ta1
......................................................................ugagaCcauugugaaagcugau...................    2       1    Ta1
......................................................................ugaUaucauugugaaagcugau...................    19      1    Ta1
......................................................................ugCgaucauugugaaagcugau...................    35      1    Ta1
......................................................................ugagaGcauugugaaagcugau...................    3       1    Ta1
......................................................................Ggagaucauugugaaagcugauu..................    2       1    Ta1
......................................................................ugagaucauugugaUagcugauu..................    1       1    Ta1
......................................................................ugagaucauugugaaagcAgauu..................    1       1    Ta1
......................................................................ugagaucauugugaaagUugauu..................    1       1    Ta1
......................................................................ugagaucauugugaaagcugauu..................    3658    0    Ta1
......................................................................ugaUaucauugugaaagcugauu..................    5       1    Ta1
......................................................................ugagaucauugugUaagcugauu..................    1       1    Ta1
......................................................................ugagauGauugugaaagcugauu..................    3       1    Ta1
......................................................................ugaAaucauugugaaagcugauu..................    1       1    Ta1
......................................................................ugagaucauuguCaaagcugauu..................    1       1    Ta1
......................................................................ugagaucUuugugaaagcugauu..................    4       1    Ta1
......................................................................ugagaucauugugaaUgcugauu..................    3       1    Ta1
......................................................................ugagaGcauugugaaagcugauu..................    1       1    Ta1
......................................................................ugagaucauugugaaagcugaCu..................    2       1    Ta1
......................................................................ugagaucauugugaaGgcugauu..................    3       1    Ta1
......................................................................ugagaucauugugaaagcugauC..................    3       1    Ta1
```

Star Mature

ggaacucgaaagcgcccuagcuguagacaaaacugguuuucacaaugauuugacagauguguucgauaucugagaucauugugaaagcugauuuuguugucccaucaucu			
....ugagaucauugugaaagcugauA.................	215	1	Ta1
....ugagaucauugugCaagcugauu.................	16	1	Ta1
....ugagaucauugugaaagcugauG.................	96	1	Ta1
....uAagaucauugugaaagcugauu.................	1	1	Ta1
....ugagaucGuugugaaagcugauu.................	1	1	Ta1
....ugagaucauuUugaaagcugauu.................	2	1	Ta1
....ugagaucaAugugaaagcugauu.................	2	1	Ta1
....ugGgaucauugugaaagcugauu.................	1	1	Ta1
....Agagaucauugugaaagcugauu.................	9	1	Ta1
....ugagaucauugugaaagcuUauu.................	9	1	Ta1
....Ngagaucauugugaaagcugauu.................	3	1	Ta1
....ugagaucauugugaaagcuAauu.................	1	1	Ta1
....ugagGucauugugaaagcugauu.................	40	1	Ta1
....ugagaucauugugaaaCcugauu.................	1	1	Ta1
....ugagaucauugugaaagcugUuu.................	1	1	Ta1
....uCagaucauugugaaagcugauu.................	5	1	Ta1
....ugagaucauugugaaagcugGuu.................	1	1	Ta1
....ugagaucauugugaaagcugCuu.................	3	1	Ta1
....ugagaCcauugugaaagcugauu.................	4	1	Ta1
....ugagaucauCgugaaagcugauu.................	4	1	Ta1
....ugagaucauuguAaaagcugauu.................	2	1	Ta1
....ugCgaucauugugaaagcugauu.................	14	1	Ta1
....ugagaucauugugaCagcugauu.................	1	1	Ta1
....ugagauUauugugaaagcugauu.................	8	1	Ta1
....Cgagaucauugugaaagcugauu.................	4	1	Ta1
....ugagaucCuugugaaagcugauu.................	1	1	Ta1
....ugagaucauugugaaagcuCauu.................	1	1	Ta1
....ugagUucauugugaaagcugauu.................	53	1	Ta1
....ugagaucauuAugaaagcugauu.................	1	1	Ta1
....ugagaucauugCgaaagcugauu.................	1	1	Ta1
....ugagaucauugugaaagcugauuu................	107	0	Ta1
....ugagaucauugugaaagcGgauuu................	1	1	Ta1
....ugaUaucauugugaaagcugauuu................	1	1	Ta1
....ugagaucauugugCaagcugauuu................	1	1	Ta1
....ugagaucaAugugaaagcugauuu................	1	1	Ta1
....ugagGucauugugaaagcugauuu................	2	1	Ta1
....ugagaucauugugaaagcugauuA................	71	1	Ta1
....Agagaucauugugaaagcugauuu................	1	1	Ta1
....ugagUucauugugaaagcugauuu................	1	1	Ta1
....gagUucauugugaaagcu.....................	1	1	Ta1
....gaCaucauugugaaagcu.....................	2	1	Ta1
....gagaucauugugaaagcu.....................	24	0	Ta1
....gagaAcauugugaaagcu.....................	2	1	Ta1
....gagaucauugugaaagcA.....................	1	1	Ta1
....gagaGcauugugaaagcu.....................	2	1	Ta1
....gagaucauuguUaaagcu.....................	1	1	Ta1
....gagaAcauugugaaagcug....................	2	1	Ta1
....gagaucauuguAaaagcug....................	1	1	Ta1
....gaCaucauugugaaagcug....................	3	1	Ta1
....gagaCcauugugaaagcug....................	3	1	Ta1
....gagaucCuugugaaagcug....................	1	1	Ta1
....gagaucauugugaUagcug....................	1	1	Ta1
....gagaucauugugaaagUug....................	1	1	Ta1
....gagauAauugugaaagcug....................	1	1	Ta1
....gagaucauuguUaaagcug....................	1	1	Ta1
....gagaucauugAgaaagcug....................	1	1	Ta1
....gagaucauAgugaaagcug....................	3	1	Ta1
....gagaucauugugaaagcug....................	85	0	Ta1
....gagaucauugugCaagcug....................	1	1	Ta1
....gCgaucauugugaaagcug....................	1	1	Ta1
....gagaucauugugaaagcGg....................	1	1	Ta1
....Aagaucauugugaaagcug....................	2	1	Ta1
....gagUucauugugaaagcug....................	6	1	Ta1
....gagaGcauugugaaagcug....................	4	1	Ta1
....gagaGcauugugaaagcuga...................	3	1	Ta1
....gagaucauCgugaaagcuga...................	1	1	Ta1
....gagUucauugugaaagcuga...................	1	1	Ta1
....Cagaucauugugaaagcuga...................	2	1	Ta1
....gagaucauugugaaagcuga...................	13	0	Ta1
....gaCaucauugugaaagcuga...................	1	1	Ta1

Star **Mature**

ggaacucgaaagcgcccuagcuguagacaaaacugguuuucacaaugauuugacagauguguucgauaucugagaucauugugaaagcugauuuuguugucccaucaucu			
....Uagaucauugugaaagcuga...................	1	1	Ta1
....gaAaucauugugaaagcugau..................	1	1	Ta1
....gagaucauAgugaaagcugau..................	1	1	Ta1
....gaCaucauugugaaagcugau..................	2	1	Ta1
....gagaCcauugugaaagcugau..................	1	1	Ta1
....gagauAauugugaaagcugau..................	2	1	Ta1
....gagaucauugugaaagAugau..................	1	1	Ta1
....gagaucaGugugaaagcugau..................	1	1	Ta1
....gagaucauugugaaagcugau..................	31	0	Ta1
....gagauUauugugaaagcugau..................	1	1	Ta1
....gagaGcauugugaaagcugau..................	4	1	Ta1
....gagUucauugugaaagcugau..................	1	1	Ta1
....gaUaucauugugaaagcugauu.................	1	1	Ta1
....gagaucauAgugaaagcugauu.................	1	1	Ta1
....gaCaucauugugaaagcugauu.................	1	1	Ta1
....gagaGcauugugaaagcugauu.................	3	1	Ta1
....Aagaucauugugaaagcugauu.................	1	1	Ta1
....gagaucauugugaaagcugauu.................	20	0	Ta1
....gagaucauugugaCagcugauu.................	1	1	Ta1
....agaucauugugaaagcug....................	3	0	Ta1
....agaucauugugaaagcuga...................	5	0	Ta1
....ucauuguUaaagcugauu.................	2	1	Ta1
................................cugguuuucacaaugauuuga...	7	0	Bl1
................................cugguuuucacaaugauuugac..	2	0	Bl1
....uaucugagaucauugugaaag.......................	3	0	Bl1
....uaucugagaucauugugaaagc......................	4	0	Bl1
....uaucugagaucauugugaaagcuga...................	3	0	Bl1
....aucugagaucauugugaaagcu.....................	1	0	Bl1
....Augagaucauugugaaag.......................	2	1	Bl1
....cugaUaucauugugaaagcug....................	1	1	Bl1
....cugagaucauugugaaagcug....................	7	0	Bl1
....cugagaucCuugugaaagcug....................	1	1	Bl1
....cugagaucauugugaaagcuga...................	7	0	Bl1
....cugagaucauugugaaagcugau..................	5	0	Bl1
....cugaAaucauugugaaagcugauu.................	1	1	Bl1
....Agagaucauugugaaagc......................	1	1	Bl1
....ugagaucUuugugaaagc......................	5	1	Bl1
....Cgagaucauugugaaagc......................	1	1	Bl1
....ugaUaucauugugaaagc......................	2	1	Bl1
....ugagGucauugugaaagc......................	3	1	Bl1
....ugagUucauugugaaagc......................	10	1	Bl1
....ugagaucauugugCaagc......................	2	1	Bl1
....ugCgaucauugugaaagc......................	1	1	Bl1
....ugagaucauugugaaagc......................	462	0	Bl1
....ugagaucauCgugaaagc......................	1	1	Bl1
....ugagaucaAugugaaagc......................	1	1	Bl1
....ugagaucauugugaaUgc......................	1	1	Bl1
....ugagaucauugugaaaCcu.....................	1	1	Bl1
....uCagaucauugugaaagcu.....................	2	1	Bl1
....ugagaucauugCgaaagcu.....................	2	1	Bl1
....Ngagaucauugugaaagcu.....................	4	1	Bl1
....ugGgaucauugugaaagcu.....................	2	1	Bl1
....ugagaucauugugUaagcu.....................	1	1	Bl1
....ugagaucauCgugaaagcu.....................	1	1	Bl1
....ugagauUauugugaaagcu.....................	1	1	Bl1
....Cgagaucauugugaaagcu.....................	10	1	Bl1
....ugagaucauugugCaagcu.....................	32	1	Bl1
....ugagaucauugugaGagcu.....................	2	1	Bl1
....ugaAaucauugugaaagcu.....................	1	1	Bl1
....ugagaucaAugugaaagcu.....................	15	1	Bl1
....ugagaucauAgugaaagcu.....................	2	1	Bl1
....ugagaucauuguAaaagcu.....................	1	1	Bl1
....ugaUaucauugugaaagcu.....................	12	1	Bl1
....ugagaucauugugaaagcu.....................	5523	0	Bl1
....ugagaucauugugaUagcu.....................	2	1	Bl1
....ugagCucauugugaaagcu.....................	1	1	Bl1
....ugagaucUuugugaaagcu.....................	14	1	Bl1
....ugagaucauuAugaaagcu.....................	4	1	Bl1
....ugagaucauugugaaagcA.....................	791	1	Bl1

Star Mature

```
ggaacucgaaagcgcccuagcuguagacaaaacugguuuucacaaugauuugacagauguguucgauaucugagaucauugugaaagcugauuuuguuguccccaucaucu
......................................................................ugagaucGuugugaaagcu......................	1	1	Bl1
......................................................................ugagaucauugugaaaUcu......................	1	1	Bl1
......................................................................ugagaAcauugugaaagcu......................	1	1	Bl1
......................................................................ugagaucauugugaaUgcu......................	6	1	Bl1
......................................................................ugCgaucauugugaaagcu......................	14	1	Bl1
......................................................................uAagaucauugugaaagcu......................	7	1	Bl1
......................................................................ugagUucauugugaaagcu......................	98	1	Bl1
......................................................................ugagaucauugugaaagcC......................	351	1	Bl1
......................................................................ugagaucaNugugaaagcu......................	1	1	Bl1
......................................................................ugagaGcauugugaaagcu......................	1	1	Bl1
......................................................................ugagaucauuguUaaagcu......................	2	1	Bl1
......................................................................ugagaucauugugaaGgcu......................	3	1	Bl1
......................................................................ugagaucauugugaaaAcu......................	5	1	Bl1
......................................................................ugagGucauugugaaagcu......................	71	1	Bl1
......................................................................ugagaucauugugaaagUu......................	1	1	Bl1
......................................................................ugagaucauugugaaagcG......................	4	1	Bl1
......................................................................uUagaucauugugaaagcu......................	1	1	Bl1
......................................................................ugagaCcauugugaaagcu......................	1	1	Bl1
......................................................................ugagauAauugugaaagcu......................	1	1	Bl1
......................................................................Agagaucauugugaaagcu......................	9	1	Bl1
......................................................................ugagaucauuCugaaagcu......................	1	1	Bl1
......................................................................ugagaucauugugaaGgcug.....................	7	1	Bl1
......................................................................ugagaucauugugaGagcug.....................	11	1	Bl1
......................................................................ugGgaucauugugaaagcug.....................	4	1	Bl1
......................................................................ugagaucUuugugaaagcug.....................	87	1	Bl1
......................................................................ugUgaucauugugaaagcug.....................	6	1	Bl1
......................................................................ugagaucaAugugaaagcug.....................	51	1	Bl1
......................................................................ugagaucauugugaaagcAg.....................	9	1	Bl1
......................................................................ugagaucauugugaaagcCg.....................	36	1	Bl1
......................................................................ugagaCcauugugaaagcug.....................	8	1	Bl1
......................................................................ugagaucauugugaaagcuA.....................	157	1	Bl1
......................................................................uCagaucauugugaaagcug.....................	10	1	Bl1
......................................................................ugagaucauugCgaaagcug.....................	7	1	Bl1
......................................................................ugagUucauugugaaagcug.....................	620	1	Bl1
......................................................................ugagaucauugugaaaAcug.....................	12	1	Bl1
......................................................................ugaUaucauugugaaagcug.....................	81	1	Bl1
......................................................................ugagauUauugugaaagcug.....................	10	1	Bl1
......................................................................ugagaucauugugUaagcug.....................	4	1	Bl1
......................................................................ugagaucaNugugaaagcug.....................	1	1	Bl1
......................................................................ugagaucauugugaaagcug.....................	33125	0	Bl1
......................................................................ugagauGauugugaaagcug.....................	8	1	Bl1
......................................................................uUagaucauugugaaagcug.....................	4	1	Bl1
......................................................................ugagaAcauugugaaagcug.....................	5	1	Bl1
......................................................................ugagaucauuAugaaagcug.....................	14	1	Bl1
......................................................................Ggagaucauugugaaagcug.....................	1	1	Bl1
......................................................................ugagauAauugugaaagcug.....................	1	1	Bl1
......................................................................ugagaucauuguAaaagcug.....................	5	1	Bl1
......................................................................ugagaucauugugGaagcug.....................	10	1	Bl1
......................................................................ugagaucauugugCaagcug.....................	131	1	Bl1
......................................................................Ngagaucauugugaaagcug.....................	25	1	Bl1
......................................................................ugagaucauugugaaagUug.....................	31	1	Bl1
......................................................................ugagaGcauugugaaagcug.....................	20	1	Bl1
......................................................................ugagaucauugugaaagcuU.....................	81	1	Bl1
......................................................................ugagaucauugugaaagcuC.....................	6	1	Bl1
......................................................................ugagCucauugugaaagcug.....................	9	1	Bl1
......................................................................ugagaucGuugugaaagcug.....................	6	1	Bl1
......................................................................ugagaucauugGgaaagcug.....................	9	1	Bl1
......................................................................ugagaucauCgugaaagcug.....................	6	1	Bl1
......................................................................ugagaucauugugaUagcug.....................	6	1	Bl1
......................................................................ugagaucauugugaaCgcug.....................	2	1	Bl1
......................................................................ugagaucauugugaCagcug.....................	1	1	Bl1
......................................................................ugagaucauuCugaaagcug.....................	1	1	Bl1
......................................................................uAagaucauugugaaagcug.....................	19	1	Bl1
......................................................................ugaCaucauugugaaagcug.....................	2	1	Bl1
......................................................................ugagaucauuguUaaagcug.....................	6	1	Bl1
......................................................................ugagaucaGugugaaagcug.....................	1	1	Bl1
......................................................................ugagaucauGgugaaagcug.....................	5	1	Bl1
......................................................................ugagaucauugAgaaagcug.....................	1	1	Bl1
......................................................................ugaAaucauugugaaagcug.....................	1	1	Bl1
......................................................................Cgagaucauugugaaagcug.....................	35	1	Bl1
```

```
                                 Star                              Mature
ggaacucgaaagcgcccuagcuguagacaaaacugguuuucacaaugauuugacagauguguucgauaucugagaucauugugaaagcugauuuuguuguccccaucaucu
......................................................................Agagaucauugugaaagcug.....................   71      1   Bl1
......................................................................ugagaucCuugugaaagcug.....................   2       1   Bl1
......................................................................ugagaucauugugaaagcGg.....................   20      1   Bl1
......................................................................ugagaucauugugaaUgcug.....................   29      1   Bl1
......................................................................ugCgaucauugugaaagcug.....................   98      1   Bl1
......................................................................ugagGucauugugaaagcug.....................   442     1   Bl1
......................................................................ugagaucauuUugaaagcug.....................   4       1   Bl1
......................................................................ugagaucauugugaaaUcug.....................   6       1   Bl1
......................................................................ugagaucauugugaaagAuga....................   1       1   Bl1
......................................................................Ggagaucauugugaaagcuga....................   4       1   Bl1
......................................................................ugagaucauCgugaaagcuga....................   4       1   Bl1
......................................................................ugagaucaAugugaaagcuga....................   25      1   Bl1
......................................................................uCagaucauugugaaagcuga....................   3       1   Bl1
......................................................................ugaUaucauugugaaagcuga....................   18      1   Bl1
......................................................................ugagaucauugugGaagcuga....................   2       1   Bl1
......................................................................ugagaucauugugaaagcugG....................   3       1   Bl1
......................................................................ugagaGcauugugaaagcuga....................   2       1   Bl1
......................................................................ugUgaucauugugaaagcuga....................   3       1   Bl1
......................................................................ugagaucauugugaaagUuga....................   6       1   Bl1
......................................................................ugCgaucauugugaaagcuga....................   61      1   Bl1
......................................................................ugagUucauugugaaagcuga....................   241     1   Bl1
......................................................................ugagaucauugugaaagcugU....................   87      1   Bl1
......................................................................ugagaucauuAugaaagcuga....................   2       1   Bl1
......................................................................ugagaucauugugaaagcGga....................   1       1   Bl1
......................................................................ugagaucauugugaaagcAga....................   1       1   Bl1
......................................................................ugagaucauugugaaUgcuga....................   7       1   Bl1
......................................................................ugagaucauugugaaagcuCa....................   1       1   Bl1
......................................................................ugagGucauugugaaagcuga....................   153     1   Bl1
......................................................................ugagaucauugugaaagcuUa....................   45      1   Bl1
......................................................................ugagaucauugugCaagcuga....................   36      1   Bl1
......................................................................ugagaucauuguUaaagcuga....................   3       1   Bl1
......................................................................ugagaucauAgugaaagcuga....................   1       1   Bl1
......................................................................ugagaucauugugaaagcuAa....................   4       1   Bl1
......................................................................ugagaucauuguAaaagcuga....................   3       1   Bl1
......................................................................ugagCucauugugaaagcuga....................   1       1   Bl1
......................................................................ugagaucauugugaaagcCga....................   3       1   Bl1
......................................................................ugagaucauugugaGagcuga....................   3       1   Bl1
......................................................................ugagaCcauugugaaagcuga....................   3       1   Bl1
......................................................................ugagaucauugCgaaagcuga....................   1       1   Bl1
......................................................................ugagaucauugGgaaagcuga....................   1       1   Bl1
......................................................................ugagaucauuguCaaagcuga....................   1       1   Bl1
......................................................................ugagaucauugugUaagcuga....................   1       1   Bl1
......................................................................ugagaucauugugaaaUcuga....................   3       1   Bl1
......................................................................ugagaucUuugugaaagcuga....................   25      1   Bl1
......................................................................ugagaAcauugugaaagcuga....................   4       1   Bl1
......................................................................ugagaucauugugaaagcugC....................   14      1   Bl1
......................................................................ugGgaucauugugaaagcuga....................   5       1   Bl1
......................................................................ugagaucauGgugaaagcuga....................   1       1   Bl1
......................................................................Agagaucauugugaaagcuga....................   23      1   Bl1
......................................................................ugagaucauugugaaagcuga....................   12538   0   Bl1
......................................................................uAagaucauugugaaagcuga....................   8       1   Bl1
......................................................................Cgagaucauugugaaagcuga....................   29      1   Bl1
......................................................................uUagaucauugugaaagcuga....................   2       1   Bl1
......................................................................ugagaucauugugaUagcuga....................   4       1   Bl1
......................................................................ugagauUauugugaaagcuga....................   1       1   Bl1
......................................................................ugagaucauugugaaaAcuga....................   3       1   Bl1
......................................................................ugagaucGuugugaaagcuga....................   1       1   Bl1
......................................................................Ngagaucauugugaaagcuga....................   9       1   Bl1
......................................................................ugagaucGuugugaaagcugau...................   3       1   Bl1
......................................................................ugagaucauCgugaaagcugau...................   3       1   Bl1
......................................................................ugUgaucauugugaaagcugau...................   1       1   Bl1
......................................................................ugagaucauugugaaagcGgau...................   3       1   Bl1
......................................................................Ggagaucauugugaaagcugau...................   2       1   Bl1
......................................................................ugagaucauugugaaaAcugau...................   1       1   Bl1
......................................................................ugagaucauugugaaagcugaG...................   4       1   Bl1
......................................................................ugagauUauugugaaagcugau...................   6       1   Bl1
......................................................................ugagaGcauugugaaagcugau...................   4       1   Bl1
......................................................................ugagaucauugugCaagcugau...................   27      1   Bl1
......................................................................uCagaucauugugaaagcugau...................   1       1   Bl1
......................................................................ugagaucauugugaGagcugau...................   4       1   Bl1
```

```
                                Star                              Mature
ggaacucgaaagcgcccuagcuguagacaaaacugguuuucacaaugauuugacagauguguucgauaucugagaucauugugaaagcugauuuuguuguccccaucaucu
......................................................................ugagaucauugugaaaCcugau...................  1     1  Bl1
......................................................................ugagaucauugugaaaUcugau...................  3     1  Bl1
......................................................................ugagaucUuugugaaagcugau...................  19    1  Bl1
......................................................................ugagaucauugugaaagcugau...................  8467  0  Bl1
......................................................................ugagaucauuguAaaagcugau...................  1     1  Bl1
......................................................................ugagaucaAugugaaagcugau...................  12    1  Bl1
......................................................................Ngagaucauugugaaagcugau...................  5     1  Bl1
......................................................................ugaAaucauugugaaagcugau...................  1     1  Bl1
......................................................................ugagCucauugugaaagcugau...................  4     1  Bl1
......................................................................ugagaucauugugaaagcCgau...................  3     1  Bl1
......................................................................ugagaucauugugaUagcugau...................  3     1  Bl1
......................................................................ugagaucauugugaaagcugGu...................  3     1  Bl1
......................................................................ugagaucauugugaaagcuAau...................  1     1  Bl1
......................................................................ugagGucauugugaaagcugau...................  96    1  Bl1
......................................................................ugagaucauugugaaagcugCu...................  4     1  Bl1
......................................................................ugagaucauugugaaagUugau...................  8     1  Bl1
......................................................................ugagaucauugugaaCgcugau...................  1     1  Bl1
......................................................................ugagaucauugAgaaagcugau...................  1     1  Bl1
......................................................................Cgagaucauugugaaagcugau...................  12    1  Bl1
......................................................................uAagaucauugugaaagcugau...................  4     1  Bl1
......................................................................ugagaucauugugaaagcuUau...................  23    1  Bl1
......................................................................ugagaucauugugaaUgcugau...................  8     1  Bl1
......................................................................ugagUucauugugaaagcugau...................  130   1  Bl1
......................................................................ugaUaucauugugaaagcugau...................  10    1  Bl1
......................................................................ugGgaucauugugaaagcugau...................  2     1  Bl1
......................................................................ugagauGauugugaaagcugau...................  1     1  Bl1
......................................................................ugCgaucauugugaaagcugau...................  23    1  Bl1
......................................................................ugagaucauugugaaagcugUu...................  150   1  Bl1
......................................................................uUagaucauugugaaagcugau...................  2     1  Bl1
......................................................................ugagaucauugugUaagcugau...................  1     1  Bl1
......................................................................ugagaucauugugaaagcAgau...................  1     1  Bl1
......................................................................ugagaucauuguCaaagcugau...................  1     1  Bl1
......................................................................ugagaucauugugaaagcuCau...................  2     1  Bl1
......................................................................ugagaucauugugaaagcugaC...................  88    1  Bl1
......................................................................ugagaucauugugaaagcugaA...................  83    1  Bl1
......................................................................ugagaCcauugugaaagcugau...................  5     1  Bl1
......................................................................ugagaucauuAugaaagcugau...................  3     1  Bl1
......................................................................ugagaAcauugugaaagcugau...................  1     1  Bl1
......................................................................Agagaucauugugaaagcugau...................  10    1  Bl1
......................................................................ugagaucauAgugaaagcugau...................  2     1  Bl1
......................................................................ugagaucauugugaaagcuAauu..................  1     1  Bl1
......................................................................ugagaucauugugaaagcugauG..................  13    1  Bl1
......................................................................ugagaGcauugugaaagcugauu..................  1     1  Bl1
......................................................................ugagaucauugugaaagcugCuu..................  1     1  Bl1
......................................................................ugagCucauugugaaagcugauu..................  1     1  Bl1
......................................................................ugagaucauugugaaCgcugauu..................  2     1  Bl1
......................................................................ugaUaucauugugaaagcugauu..................  2     1  Bl1
......................................................................ugagaucauugugCaagcugauu..................  2     1  Bl1
......................................................................ugagaucauugugaaagcugUuu..................  120   1  Bl1
......................................................................ugagaucauAgugaaagcugauu..................  1     1  Bl1
......................................................................Cgagaucauugugaaagcugauu..................  3     1  Bl1
......................................................................ugagaucauugugaaagcugauA..................  146   1  Bl1
......................................................................ugagaucauugugaaagUugauu..................  2     1  Bl1
......................................................................ugagGucauugugaaagcugauu..................  4     1  Bl1
......................................................................uUagaucauugugaaagcugauu..................  1     1  Bl1
......................................................................ugagaucauugugaaagcugGuu..................  1     1  Bl1
......................................................................ugagaucauugugaaagcugauu..................  504   0  Bl1
......................................................................ugagaucauugugaaagcugauC..................  1     1  Bl1
......................................................................ugCgaucauugugaaagcugauu..................  1     1  Bl1
......................................................................ugagUucauugugaaagcugauu..................  12    1  Bl1
......................................................................ugagaucauugugaaagcuUauu..................  2     1  Bl1
......................................................................ugagaucauugugaaagcGgauu..................  1     1  Bl1
......................................................................ugagaucauugugaaagcugUuuu.................  4     1  Bl1
......................................................................ugagaucauugugaaagcugauuu.................  12    0  Bl1
......................................................................ugagaucauugugaaagcugauuA.................  23    1  Bl1
......................................................................ugagaucauugugaaagcugUuuuu................  1     1  Bl1
.......................................................................gagaucauugugaaagcu......................  12    0  Bl1
.......................................................................gagaAcauugugaaagcu......................  1     1  Bl1
.......................................................................gagaGcauugugaaagcu......................  1     1  Bl1
.......................................................................gagaucauugugaaagcA......................  1     1  Bl1
```

```
                                 Star                                 Mature
ggaacucgaaagcgcccuagcuguagacaaaacugguuuucacaaugauuugacagauguguucgauaucugagaucauugugaaagcugauuuuguugucccaucaucu
.......................................................................gaCaucauugugaaagcu.....................    1    1    Bl1
.......................................................................gagaAcauugugaaagcug....................    1    1    Bl1
.......................................................................gaCaucauugugaaagcug....................    2    1    Bl1
.......................................................................gagaucauuguUaaagcug....................    1    1    Bl1
.......................................................................gagaucaAugugaaagcug....................    1    1    Bl1
.......................................................................gagaucauuCugaaagcug....................    1    1    Bl1
.......................................................................gagaucauugugaaagcCg....................    1    1    Bl1
.......................................................................gagUucauugugaaagcug....................    5    1    Bl1
.......................................................................gagaucauAgugaaagcug....................    1    1    Bl1
.......................................................................gagaucauugugaCagcug....................    1    1    Bl1
.......................................................................gagaucauugugaaagcug....................    106  0    Bl1
.......................................................................gCgaucauugugaaagcug....................    2    1    Bl1
.......................................................................gagaGcauugugaaagcug....................    12   1    Bl1
.......................................................................gagaucaGugugaaagcug....................    2    1    Bl1
.......................................................................gagaucauugugaaaUcug....................    1    1    Bl1
.......................................................................gagaucauugugaaUgcug....................    1    1    Bl1
.......................................................................gagaucauugugaaagcuA....................    1    1    Bl1
.......................................................................gagaucauugugaaagcuU....................    3    1    Bl1
.......................................................................gagaucauugugCaagcuga...................    1    1    Bl1
.......................................................................gagaucauuguAaaagcuga...................    1    1    Bl1
.......................................................................gagUucauugugaaagcuga...................    2    1    Bl1
.......................................................................gagaAcauugugaaagcuga...................    1    1    Bl1
.......................................................................gaCaucauugugaaagcuga...................    2    1    Bl1
.......................................................................gagaucaGugugaaagcuga...................    1    1    Bl1
.......................................................................gagaGcauugugaaagcuga...................    6    1    Bl1
.......................................................................gagaucauugugaCagcuga...................    1    1    Bl1
.......................................................................Uagaucauugugaaagcuga...................    1    1    Bl1
.......................................................................gagaucauAgugaaagcuga...................    1    1    Bl1
.......................................................................gagaucauugAgaaagcuga...................    1    1    Bl1
.......................................................................gagaucauugugaaagcuga...................    63   0    Bl1
.......................................................................gCgaucauugugaaagcugau..................    1    1    Bl1
.......................................................................gagaucauugugaaagAugau..................    1    1    Bl1
.......................................................................gagUucauugugaaagcugau..................    1    1    Bl1
.......................................................................gagaAcauugugaaagcugau..................    1    1    Bl1
.......................................................................gagaucauugugaaaUcugau..................    1    1    Bl1
.......................................................................gagaucauugugaaagcugau..................    46   0    Bl1
.......................................................................gagaucauugugaaagcugaA..................    1    1    Bl1
.......................................................................gagaucauugugCaagcugau..................    1    1    Bl1
.......................................................................gagaucaGugugaaagcugau..................    1    1    Bl1
.......................................................................gagaucauugCgaaagcugau..................    1    1    Bl1
.......................................................................gagaGcauugugaaagcugau..................    3    1    Bl1
.......................................................................gagaCcauugugaaagcugau..................    1    1    Bl1
.......................................................................gagaucauugugaaagcugauu.................    2    0    Bl1
.......................................................................gagaucauugugaaagcuUauu.................    1    1    Bl1
.......................................................................gagUucauugugaaagcugauu.................    1    1    Bl1

.............gcccuagcuguagacaaaa..............................................................................    1    0    He1
................................cugguuuucacaaugauuug..........................................................    3    0    He1
................................Uugguuuucacaaugauuuga.........................................................    1    1    He1
................................cuggCuuucacaaugauuuga.........................................................    1    1    He1
................................cugguuuucacaaugauuuga.........................................................    41   0    He1
................................cuggGuuucacaaugauuuga.........................................................    2    1    He1
................................Augguuuucacaaugauuuga.........................................................    1    1    He1
................................cuggCuuucacaaugauuugac........................................................    1    1    He1
................................cugguuuucacaaugauuugac........................................................    9    0    He1
................................cuggGuuucacaaugauuugac........................................................    1    1    He1
................................cugguuuucacaaugauuugaca.......................................................    9    0    He1
................................cugguuuucacaaugauuugacU.......................................................    1    1    He1
.................................ugguuuucacaaugauuuga.........................................................    7    0    He1
.................................ugguuuucacaaugauuugac........................................................    1    0    He1
.................................ugguuuucacaaugauuugaca.......................................................    1    0    He1
.................................ugguuuucacaaugauuugacaga.....................................................    1    0    He1
....................................uuuucacaaugauuugacagaugugC................................................    1    1    He1
..................................................................uaucugagaucauugugaa.........................    1    0    He1
..................................................................uaucugagaucauugugaaa........................    4    0    He1
..................................................................uaucGgagaucauugugaaa........................    1    1    He1
..................................................................uaucugagaucauugugaaag.......................    1    0    He1
..................................................................uaucugagaucauugugaaagA......................    1    1    He1
..................................................................uaucugagaucauugugaaagc......................    32   0    He1
..................................................................uaucCgagaucauugugaaagc......................    1    1    He1
```

Star Mature

ggaacucgaaagcgcccuagcuguagacaaaacugguuuucacaaugauuugacagauguguucgauaucugagaucauugugaaagcugauuuuguugucccaucaucu			
....uauUugagaucauugugaaagc......................	1	1	He1
....uaucGgagaucauugugaaagc......................	1	1	He1
....uaucugagaucauugugaaagcG.....................	1	1	He1
....uaucugUgaucauugugaaagcu.....................	1	1	He1
....uaucugagaucauugugaaagcu.....................	7	0	He1
....uaucGgagaucauugugaaagcu.....................	2	1	He1
....uaucugagaucauugugaaagcC.....................	1	1	He1
....uaucugagaucauugugaaagcuga...................	1	0	He1
....aucuUagaucauugugaa.........................	1	1	He1
....aucugagaucauugugaaagc......................	1	0	He1
....aucugagaucauugugaaagcC.....................	3	1	He1
....aucugagaucauugugaaagcu.....................	7	0	He1
....aucugagGucauugugaaagcu.....................	1	1	He1
....aucuUagaucauugugaaagcu.....................	1	1	He1
....aucugagaucauugugaaagcA.....................	3	1	He1
....aucugagaucauugugaaagcuU....................	1	1	He1
....aucugagaucauugugaaagcug....................	2	0	He1
....ucugagaucauugugaaagcC.....................	1	1	He1
....ucugagaucauugugaaagcu.....................	1	0	He1
....ucugagaucauugugaaagcug....................	5	0	He1
....ucugagaucauugugaaagcGg....................	1	1	He1
....ucugUgaucauugugaaagcug....................	1	1	He1
....ucugagaucauugugaaagcCg....................	1	1	He1
....ucugagaucauugugaaagcugau..................	2	0	He1
....cugagaucauugugaaagcu.....................	5	0	He1
....cugagaucauugugCaagcu.....................	1	1	He1
....Augagaucauugugaaagcug....................	1	1	He1
....cugaUaucauugugaaagcug....................	1	1	He1
....Uugagaucauugugaaagcug....................	1	1	He1
....cugagaucauugugaaagcCg....................	1	1	He1
....cugagaucauugugaaagcug....................	23	0	He1
....Uugagaucauugugaaagcuga...................	1	1	He1
....cugagaucauugugaaagcugU...................	2	1	He1
....cugagaucauuUugaaagcuga...................	1	1	He1
....cugagaucauugugaaagcuga...................	15	0	He1
....cugagaucauugugaaagcugau..................	41	0	He1
....cugagaucauugugaaagcugaC..................	3	1	He1
....cugagaucauugugaaagcugaA..................	3	1	He1
....Augagaucauugugaaagcugau..................	1	1	He1
....Uugagaucauugugaaagcugau..................	1	1	He1
....cugaUaucauugugaaagcugau..................	1	1	He1
....cugagaucCuugugaaagcugau..................	1	1	He1
....cugagaucauuUugaaagcugau..................	1	1	He1
....Augagaucauugugaaagcugauu.................	1	1	He1
....cugagaucauugugaaagcugauu.................	15	0	He1
....cugagaucauugugaaagcugauA.................	1	1	He1
....ugagaucauugGgaaagc......................	1	1	He1
....ugagGucauugugaaagc......................	2	1	He1
....ugagaucauugugaaAc.......................	1	1	He1
....ugagaCcauugugaaagc......................	1	1	He1
....ugagaucauugugaaagc......................	105	0	He1
....Ngagaucauugugaaagcu.....................	16	1	He1
....Agagaucauugugaaagcu.....................	23	1	He1
....ugagaucauugugaaUgcu.....................	16	1	He1
....ugGgaucauugugaaagcu.....................	3	1	He1
....ugagaucGuugugaaagcu.....................	3	1	He1
....ugCgaucauugugaaagcu.....................	39	1	He1
....ugagaucauugugaUagcu.....................	2	1	He1
....ugaUaucauugugaaagcu.....................	37	1	He1
....ugagGucauugugaaagcu.....................	237	1	He1
....Ggagaucauugugaaagcu.....................	1	1	He1
....ugagaucauugAgaaagcu.....................	2	1	He1
....ugagaucauGgugaaagcu.....................	2	1	He1
....ugagaucauuguAaaagcu.....................	2	1	He1
....Cgagaucauugugaaagcu.....................	19	1	He1
....ugagaucauuguUaaagcu.....................	3	1	He1
....ugagaucauugCgaaagcu.....................	5	1	He1
....ugagaucauugugaGagcu.....................	1	1	He1
....ugagCucauugugaaagcu.....................	6	1	He1
....ugagaucauugugaaagcG.....................	199	1	He1

Star **Mature**

ggaacucgaaagcgcccuagcuguagacaaaacugguuuucacaaugauuugacagauguguucgauaucugagaucauugugaaagcugauuuuguuguccccaucaucu

....ugagaucauugugaaagcA......................	1331	1	He1
....ugagaucauugugaaaAcu......................	2	1	He1
....ugagaucauugugaaaUcu......................	1	1	He1
....ugagaCcauugugaaagcu......................	8	1	He1
....ugagaucauugugaaagUu......................	10	1	He1
....ugagaucauugGgaaagcu......................	7	1	He1
....ugagaucauugugaaGgcu......................	2	1	He1
....uCagaucauugugaaagcu......................	2	1	He1
....ugagaucUuugugaaagcu......................	45	1	He1
....uUagaucauugugaaagcu......................	4	1	He1
....ugagaucauugugUaagcu......................	3	1	He1
....ugagUucauugugaaagcu......................	323	1	He1
....ugagaAcauugugaaagcu......................	8	1	He1
....ugUgaucauugugaaagcu......................	1	1	He1
....ugagaucauCgugaaagcu......................	3	1	He1
....ugagauUauugugaaagcu......................	6	1	He1
....ugagaucauuAugaaagcu......................	9	1	He1
....ugagaucauugugGaagcu......................	3	1	He1
....ugagaucaGugugaaagcu......................	2	1	He1
....ugagaucauugugCaagcu......................	73	1	He1
....ugagaucauAgugaaagcu......................	4	1	He1
....ugagaucauugugaaagcu......................	17319	0	He1
....ugaAaucauugugaaagcu......................	1	1	He1
....ugagaGcauugugaaagcu......................	9	1	He1
....ugagaucauuUugaaagcu......................	1	1	He1
....ugagaucauugugaaagcC......................	3337	1	He1
....ugagaucaAugugaaagcu......................	30	1	He1
....uAagaucauugugaaagcu......................	10	1	He1
....ugagaucauuCugaaagcu......................	1	1	He1
....ugagaucauugugaCagcu......................	1	1	He1
....ugagaucauugugaaaCcug.....................	4	1	He1
....ugagaucauugCgaaagcug.....................	8	1	He1
....ugagaucauugugaaagcug.....................	80625	0	He1
....ugagaucauugAgaaagcug.....................	5	1	He1
....ugagaucCuugugaaagcug.....................	1	1	He1
....ugagaucauuguUaaagcug.....................	10	1	He1
....ugagaucauugugaaCgcug.....................	10	1	He1
....Ngagaucauugugaaagcug.....................	57	1	He1
....ugagaucauuAugaaagcug.....................	31	1	He1
....ugagaucauugugaaaAcug.....................	16	1	He1
....ugagaucauugugaaGgcug.....................	15	1	He1
....ugagaucauugugaUagcug.....................	18	1	He1
....ugagaucauAgugaaagcug.....................	10	1	He1
....ugagaucauCgugaaagcug.....................	21	1	He1
....ugagaucauugugaaagcuA.....................	404	1	He1
....ugagGucauugugaaagcug.....................	1034	1	He1
....ugagaucauugugaaagcCg.....................	1349	1	He1
....ugagaCcauugugaaagcug.....................	14	1	He1
....ugCgaucauugugaaagcug.....................	236	1	He1
....ugagaucUuugugaaagcug.....................	214	1	He1
....ugagaAcauugugaaagcug.....................	13	1	He1
....ugagauAauugugaaagcug.....................	4	1	He1
....ugagaucauugugaCagcug.....................	5	1	He1
....ugagaucGuugugaaagcug.....................	15	1	He1
....ugagaucauugugaaaUcug.....................	13	1	He1
....ugagaucaGugugaaagcug.....................	2	1	He1
....uUagaucauugugaaagcug.....................	15	1	He1
....ugagCucauugugaaagcug.....................	22	1	He1
....ugagauUauugugaaagcug.....................	14	1	He1
....ugGgaucauugugaaagcug.....................	15	1	He1
....ugagaucauugugGaagcug.....................	10	1	He1
....ugagaucauugugCaagcug.....................	293	1	He1
....ugagauGauugugaaagcug.....................	6	1	He1
....ugagaucauugugaaUgcug.....................	80	1	He1
....ugagaucauugugaaagcuC.....................	33	1	He1
....ugaUaucauugugaaagcug.....................	150	1	He1
....ugagaucauugugaaagUug.....................	75	1	He1
....ugagaucaAugugaaagcug.....................	113	1	He1
....ugagaucauugugaaagcAg.....................	124	1	He1
....uAagaucauugugaaagcug.....................	37	1	He1

```
                                 Star                                  Mature
ggaacucgaaagcgcccuagcuguagacaaaacugguuuucacaaugauuugacagauguguucgauaucugagaucauugugaaagcugauuuuguuguccccaucaucu
......................................................................ugagUucauugugaaagcug.....................    1416    1    He1
......................................................................uCagaucauugugaaagcug.....................    34      1    He1
......................................................................ugagaucauugugaaagcGg.....................    176     1    He1
......................................................................ugagaucauuguCaaagcug.....................    5       1    He1
......................................................................ugagaGcauugugaaagcug.....................    26      1    He1
......................................................................ugagaucauuUugaaagcug.....................    7       1    He1
......................................................................ugUgaucauugugaaagcug.....................    12      1    He1
......................................................................ugaAaucauugugaaagcug.....................    11      1    He1
......................................................................ugagaucauGgugaaagcug.....................    2       1    He1
......................................................................ugagaucauuguAaaagcug.....................    16      1    He1
......................................................................Agagaucauugugaaagcug.....................    147     1    He1
......................................................................ugagaucauugGgaaagcug.....................    26      1    He1
......................................................................ugagaucauuCugaaagcug.....................    6       1    He1
......................................................................ugagaucauugugaaagcuU.....................    345     1    He1
......................................................................ugagaucauugugUaagcug.....................    15      1    He1
......................................................................Ggagaucauugugaaagcug.....................    9       1    He1
......................................................................ugagaucauugugaGagcug.....................    20      1    He1
......................................................................Cgagaucauugugaaagcug.....................    101     1    He1
......................................................................ugagaucUuugugaaagcuga....................    84      1    He1
......................................................................ugagaucCuugugaaagcuga....................    2       1    He1
......................................................................ugagauAauugugaaagcuga....................    2       1    He1
......................................................................Ggagaucauugugaaagcuga....................    7       1    He1
......................................................................ugagaucauugugUaagcuga....................    10      1    He1
......................................................................ugGgaucauugugaaagcuga....................    16      1    He1
......................................................................ugagaucauAgugaaagcuga....................    5       1    He1
......................................................................ugagauUauugugaaagcuga....................    11      1    He1
......................................................................Agagaucauugugaaagcuga....................    109     1    He1
......................................................................ugagauGauugugaaagcuga....................    5       1    He1
......................................................................ugagaucauugugGaagcuga....................    10      1    He1
......................................................................ugagaucauugugaaagAuga....................    2       1    He1
......................................................................ugaUaucauugugaaagcuga....................    78      1    He1
......................................................................ugagaucauugugaaagcuUa....................    104     1    He1
......................................................................ugagaucauuAugaaagcuga....................    21      1    He1
......................................................................ugagaCcauugugaaagcuga....................    11      1    He1
......................................................................uUagaucauugugaaagcuga....................    8       1    He1
......................................................................uCagaucauugugaaagcuga....................    17      1    He1
......................................................................ugagaucGuugugaaagcuga....................    4       1    He1
......................................................................ugagaucauugugaaagcGga....................    27      1    He1
......................................................................ugaCaucauugugaaagcuga....................    1       1    He1
......................................................................ugagaucauugugaaagcugG....................    16      1    He1
......................................................................ugagaucauugugaaagUuga....................    29      1    He1
......................................................................ugagaucauuUugaaagcuga....................    5       1    He1
......................................................................ugagGucauugugaaagcuga....................    485     1    He1
......................................................................ugagaucauugCgaaagcuga....................    7       1    He1
......................................................................ugagaucauugugaaCgcuga....................    3       1    He1
......................................................................ugagaucauuguCaaagcuga....................    2       1    He1
......................................................................ugagaucauugugaaagcugU....................    694     1    He1
......................................................................ugagaucauuguAaaagcuga....................    4       1    He1
......................................................................ugagaucauuCugaaagcuga....................    3       1    He1
......................................................................ugagaucaAugugaaagcuga....................    55      1    He1
......................................................................ugagUucauugugaaagcuga....................    684     1    He1
......................................................................ugagaGcauugugaaagcuga....................    14      1    He1
......................................................................Cgagaucauugugaaagcuga....................    88      1    He1
......................................................................ugagaucauugugaaGgcuga....................    2       1    He1
......................................................................Ngagaucauugugaaagcuga....................    35      1    He1
......................................................................ugagCucauugugaaagcuga....................    1       1    He1
......................................................................ugagaucauCgugaaagcuga....................    14      1    He1
......................................................................ugagaucauugugaaagcugC....................    144     1    He1
......................................................................ugagaucauugugaUagcuga....................    10      1    He1
......................................................................ugagaucauugugaaUgcuga....................    16      1    He1
......................................................................ugUgaucauugugaaagcuga....................    9       1    He1
......................................................................ugCgaucauugugaaagcuga....................    177     1    He1
......................................................................ugagaucauugugaaagcCga....................    26      1    He1
......................................................................ugagaucauugugaaaAcuga....................    8       1    He1
......................................................................ugagaucauugugaGagcuga....................    6       1    He1
......................................................................ugagaucauugugaaagcAga....................    1       1    He1
......................................................................ugagaucauGgugaaagcuga....................    3       1    He1
......................................................................ugagaucauugugaaagcuga....................    38842   0    He1
......................................................................ugagaucauugugaaagGuga....................    2       1    He1
......................................................................ugagaucauugugaCagcuga....................    2       1    He1
```

```
                                                Star                                Mature
ggaacucgaaagcgcccuagcuguagacaaaacugguuuucacaaugauuugacagauguguucgauaucugagaucauugugaaagcugauuuuguugucccaucaucu
......................................................................ugagaucauugugaaagcuAa...................    13     1     He1
......................................................................ugaAaucauugugaaagcuga...................    3      1     He1
......................................................................ugagaucaGugugaaagcuga...................    2      1     He1
......................................................................ugagaucauugugaaagcuCa...................    1      1     He1
......................................................................ugagaucauugAgaaagcuga...................    9      1     He1
......................................................................ugagaucauuguUaaagcuga...................    4      1     He1
......................................................................ugagaucauugugCaagcuga...................    124    1     He1
......................................................................uAagaucauugugaaagcuga...................    10     1     He1
......................................................................ugagaucauugugaaaUcuga...................    5      1     He1
......................................................................ugagaucauugGgaaagcuga...................    8      1     He1
......................................................................ugagaAcauugugaaagcuga...................    11     1     He1
......................................................................ugagaucauGgugaaagcugau..................    3      1     He1
......................................................................ugagaucauugugaaCgcugau..................    3      1     He1
......................................................................ugagaucauugugaaagcugGu..................    17     1     He1
......................................................................uCagaucauugugaaagcugau..................    31     1     He1
......................................................................ugagauGauugugaaagcugau..................    9      1     He1
......................................................................ugagaucauugugaaaCcugau..................    1      1     He1
......................................................................ugagaucauugugaUagcugau..................    18     1     He1
......................................................................Cgagaucauugugaaagcugau..................    90     1     He1
......................................................................ugaCaucauugugaaagcugau..................    2      1     He1
......................................................................ugagaucauAgugaaagcugau..................    12     1     He1
......................................................................ugagaAcauugugaaagcugau..................    12     1     He1
......................................................................ugagaucGuugugaaagcugau..................    10     1     He1
......................................................................ugagaucauugugaaaUcugau..................    13     1     He1
......................................................................ugagaucauugugaaaAcugau..................    14     1     He1
......................................................................ugagaucauugugaaagcugaG..................    46     1     He1
......................................................................ugagaucauugugaGagcugau..................    11     1     He1
......................................................................ugagaucauugugaaagcuCau..................    3      1     He1
......................................................................Ggagaucauugugaaagcugau..................    11     1     He1
......................................................................ugagaucauugugaaagcuUau..................    108    1     He1
......................................................................ugagaucauuCugaaagcugau..................    6      1     He1
......................................................................ugagaucauuguAaaagcugau..................    14     1     He1
......................................................................Agagaucauugugaaagcugau..................    131    1     He1
......................................................................ugagaucauugugUaagcugau..................    17     1     He1
......................................................................ugagaucauugugaaagcugaC..................    1888   1     He1
......................................................................ugagaucauugugaaagUugau..................    51     1     He1
......................................................................ugagaucauugugaaUgcugau..................    60     1     He1
......................................................................ugagaucauCgugaaagcugau..................    14     1     He1
......................................................................ugagaucauuUugaaagcugau..................    8      1     He1
......................................................................ugagaucaGugugaaagcugau..................    2      1     He1
......................................................................ugagaucauugugCaagcugau..................    238    1     He1
......................................................................ugaAaucauugugaaagcugau..................    7      1     He1
......................................................................ugaUaucauugugaaagcugau..................    140    1     He1
......................................................................ugCgaucauugugaaagcugau..................    208    1     He1
......................................................................ugagauAauugugaaagcugau..................    1      1     He1
......................................................................ugagaucauugugaaagcCgau..................    26     1     He1
......................................................................ugagauUauugugaaagcugau..................    32     1     He1
......................................................................ugagaucauugugaaagcugaA..................    678    1     He1
......................................................................ugagaucauugugaaagcugau..................    69231  0     He1
......................................................................uUagaucauugugaaagcugau..................    10     1     He1
......................................................................ugagaucauugugaaagcGgau..................    34     1     He1
......................................................................ugagCucauugugaaagcugau..................    11     1     He1
......................................................................ugagaucauuguCaaagcugau..................    4      1     He1
......................................................................ugagaucauugGgaaagcugau..................    16     1     He1
......................................................................uAagaucauugugaaagcugau..................    31     1     He1
......................................................................ugagaGcauugugaaagcugau..................    24     1     He1
......................................................................ugagaucauuguUaaagcugau..................    4      1     He1
......................................................................ugagaucauuAugaaagcugau..................    26     1     He1
......................................................................ugagaucauugugaaagcuAau..................    9      1     He1
......................................................................ugGgaucauugugaaagcugau..................    14     1     He1
......................................................................ugagUucauugugaaagcugau..................    1199   1     He1
......................................................................ugagGucauugugaaagcugau..................    892    1     He1
......................................................................ugagaucaAugugaaagcugau..................    95     1     He1
......................................................................ugagaucUuugugaaagcugau..................    200    1     He1
......................................................................ugagaucauugugaaagcugCu..................    61     1     He1
......................................................................ugagaucauugugaCagcugau..................    5      1     He1
......................................................................Ngagaucauugugaaagcugau..................    54     1     He1
......................................................................ugagaucauugAgaaagcugau..................    8      1     He1
......................................................................ugagaucauugugaaagAugau..................    5      1     He1
......................................................................ugagaucauugugaaGgcugau..................    12     1     He1
```

```
                                Star                                 Mature
ggaacucgaaagcgcccuagcuguagacaaaacugguuuucacaaugauuugacagauguguucgauaucugagaucauugugaaagcugauuuuguuguccccaucaucu
......................................................................ugagaCcauugugaaagcugau...................  9      1  He1
......................................................................ugagaucauugCgaaagcugau...................  6      1  He1
......................................................................ugUgaucauugugaaagcugau...................  14     1  He1
......................................................................ugagaucauugugaaagcAgau...................  3      1  He1
......................................................................ugagaucauugugaaagGugau...................  2      1  He1
......................................................................ugagaucauugugaaagcugUu...................  110    1  He1
......................................................................ugagaucauugugGaagcugau...................  8      1  He1
......................................................................ugagaucauuguUaaagcugauu..................  2      1  He1
......................................................................ugagaucauGgugaaagcugauu..................  1      1  He1
......................................................................ugaAaucauugugaaagcugauu..................  3      1  He1
......................................................................ugagaucauugugaaUgcugauu..................  40     1  He1
......................................................................ugagaucauugugaaagcugaAu..................  7      1  He1
......................................................................ugagaucauugugaaagcugauA..................  2145   1  He1
......................................................................ugagaucauugugaaCgcugauu..................  3      1  He1
......................................................................ugagaucauugugaaagcugauG..................  256    1  He1
......................................................................ugagaucauugugaaagcGgauu..................  9      1  He1
......................................................................ugagaucauAgugaaagcugauu..................  4      1  He1
......................................................................ugagaucaAugugaaagcugauu..................  45     1  He1
......................................................................ugCgaucauugugaaagcugauu..................  111    1  He1
......................................................................ugagaucauugugaaagcugCuu..................  32     1  He1
......................................................................ugagaucauugugaaagcugUuu..................  15     1  He1
......................................................................ugagaucauugugaaagAugauu..................  3      1  He1
......................................................................ugagaucauugugaaagcAgauu..................  2      1  He1
......................................................................ugagUucauugugaaagcugauu..................  745    1  He1
......................................................................ugagaAcauugugaaagcugauu..................  6      1  He1
......................................................................ugagaCcauugugaaagcugauu..................  13     1  He1
......................................................................ugagaucauugugaaaUcugauu..................  10     1  He1
......................................................................ugagGucauugugaaagcugauu..................  524    1  He1
......................................................................ugagaucauuguCaaagcugauu..................  1      1  He1
......................................................................ugagauGauugugaaagcugauu..................  5      1  He1
......................................................................ugGgaucauugugaaagcugauu..................  10     1  He1
......................................................................Agagaucauugugaaagcugauu..................  58     1  He1
......................................................................ugagaucaGugugaaagcugauu..................  2      1  He1
......................................................................ugagaucauuAugaaagcugauu..................  23     1  He1
......................................................................ugagaucauugugaUagcugauu..................  8      1  He1
......................................................................ugaCaucauugugaaagcugauu..................  1      1  He1
......................................................................ugagaucauuguAaaagcugauu..................  8      1  He1
......................................................................ugagaucauugCgaaagcugauu..................  6      1  He1
......................................................................ugagaucauugugaaagcugaGu..................  4      1  He1
......................................................................uCagaucauugugaaagcugauu..................  5      1  He1
......................................................................ugagaucauCgugaaagcugauu..................  9      1  He1
......................................................................ugagaucauugugaaagcCgauu..................  8      1  He1
......................................................................ugagaucauugugaaaAcugauu..................  8      1  He1
......................................................................ugagaucauugugaaaCcugauu..................  2      1  He1
......................................................................ugagaucauugugUaagcugauu..................  6      1  He1
......................................................................ugUgaucauugugaaagcugauu..................  6      1  He1
......................................................................ugagaucauugugaaagcuCauu..................  1      1  He1
......................................................................ugaUaucauugugaaagcugauu..................  93     1  He1
......................................................................ugagaucauugugaGagcugauu..................  14     1  He1
......................................................................ugagaucaNugugaaagcugauu..................  1      1  He1
......................................................................Ggagaucauugugaaagcugauu..................  13     1  He1
......................................................................uUagaucauugugaaagcugauu..................  8      1  He1
......................................................................ugagaucauugugaaGgcugauu..................  10     1  He1
......................................................................ugagaucauugugaaagcuUauu..................  73     1  He1
......................................................................Cgagaucauugugaaagcugauu..................  55     1  He1
......................................................................ugagauUauugugaaagcugauu..................  42     1  He1
......................................................................ugagaucauugAgaaagcugauu..................  2      1  He1
......................................................................ugagaucauugugGaagcugauu..................  9      1  He1
......................................................................ugagaucauugugaaagUugauu..................  25     1  He1
......................................................................ugagaucauuUugaaagcugauu..................  1      1  He1
......................................................................ugagaucauugugaaagcugauC..................  303    1  He1
......................................................................ugagaucUuugugaaagcugauu..................  114    1  He1
......................................................................ugagaucauugugaaagcuAauu..................  5      1  He1
......................................................................ugagaucauugugaaagcugGuu..................  3      1  He1
......................................................................ugagaucauugugaaagcugaCu..................  21     1  He1
......................................................................ugagaucauugugaaagcugauu..................  38414  0  He1
......................................................................ugagaucauugugCaagcugauu..................  113    1  He1
......................................................................ugagauAauugugaaagcugauu..................  3      1  He1
......................................................................ugagaucauuCugaaagcugauu..................  2      1  He1
......................................................................ugagaGcauugugaaagcugauu..................  12     1  He1
```

Star Mature

```
ggaacucgaaagcgcccuagcuguagacaaaacugguuuucacaaugauuugacagauguguucgauaucugagaucauugugaaagcugauuuuguuguccccaucaucu
......................................................................uAagaucauugugaaagcugau...................   16     1   He1
......................................................................ugagaucGuugugaaagcugau...................   6      1   He1
......................................................................ugagCucauugugaaagcugau...................   12     1   He1
......................................................................ugagaucauugGgaaagcugau...................   10     1   He1
......................................................................Ngagaucauugugaaagcugau...................   35     1   He1
......................................................................ugagaCcauugugaaagcugauuu.................   1      1   He1
......................................................................ugagaucauugugaaagcuUauuu.................   3      1   He1
......................................................................ugagaucauugugaaagcugaGuu.................   1      1   He1
......................................................................ugCgaucauugugaaagcugauuu.................   3      1   He1
......................................................................Agagaucauugugaaagcugauuu.................   2      1   He1
......................................................................ugagaucauugugaaagcuCauuu.................   1      1   He1
......................................................................ugagaucauugugaaagcugauuA.................   1060   1   He1
......................................................................ugagaucauugugaaCgcugauuu.................   1      1   He1
......................................................................ugagaucauugugaaagcGgauuu.................   1      1   He1
......................................................................ugagaucauugugaaagUugauuu.................   1      1   He1
......................................................................ugagaGcauugugaaagcugauuu.................   1      1   He1
......................................................................ugagaucauugugaaagcuAauuu.................   1      1   He1
......................................................................ugagaucauugugaaagcugaCuu.................   1      1   He1
......................................................................ugagaucauugugaaGgcugauuu.................   1      1   He1
......................................................................ugagaucauugugGaagcugauuu.................   2      1   He1
......................................................................ugGgaucauugugaaagcugauuu.................   1      1   He1
......................................................................ugagaucauugugaaagcugauuG.................   2      1   He1
......................................................................ugagUucauugugaaagcugauuu.................   28     1   He1
......................................................................ugagaucauugugaaagcugauuu.................   1400   0   He1
......................................................................ugagaucauugugaaagcugauAu.................   3      1   He1
......................................................................ugagaucaAugugaaagcugauuu.................   2      1   He1
......................................................................uUagaucauugugaaagcugauuu.................   1      1   He1
......................................................................ugagCucauugugaaagcugauuu.................   1      1   He1
......................................................................ugagGucauugugaaagcugauuu.................   20     1   He1
......................................................................ugagaucauugugaUagcugauuu.................   2      1   He1
......................................................................ugagaucUuugugaaagcugauuu.................   4      1   He1
......................................................................ugagaucauugugaaagcugauuC.................   12     1   He1
......................................................................ugagaucauugugCaagcugauuu.................   6      1   He1
......................................................................ugaUaucauugugaaagcugauuu.................   2      1   He1
......................................................................ugagaucauugugaaagcugauuuA................   2      1   He1
......................................................................ugagaucauugugaaagcugauuAu................   3      1   He1
......................................................................ugagaucauugugaaagcugauuuuU...............   10     1   He1
.......................................................................gaAaucauugugaaagcu......................   1      1   He1
.......................................................................gagaucauugugaaagcu......................   36     0   He1
.......................................................................gagUucauugugaaagcu......................   2      1   He1
.......................................................................gagaGcauugugaaagcu......................   2      1   He1
.......................................................................gUgaucauugugaaagcu......................   1      1   He1
.......................................................................gagaAcauugugaaagcu......................   1      1   He1
.......................................................................gagaucauugugaaUgcu......................   1      1   He1
.......................................................................gagaucauugugaaagcG......................   1      1   He1
.......................................................................gagaucauugugaaagcC......................   5      1   He1
.......................................................................Uagaucauugugaaagcu......................   1      1   He1
.......................................................................gCgaucauugugaaagcu......................   1      1   He1
.......................................................................gagaucauugugaaagcA......................   5      1   He1
.......................................................................gaCaucauugugaaagcu......................   1      1   He1
.......................................................................gagaCcauugugaaagcug.....................   5      1   He1
.......................................................................gagaucauugugaaagcuA.....................   1      1   He1
.......................................................................gagaucauuguAaaagcug.....................   2      1   He1
.......................................................................gagUucauugugaaagcug.....................   11     1   He1
.......................................................................gagaGcauugugaaagcug.....................   23     1   He1
.......................................................................gagaucauugugaUagcug.....................   1      1   He1
.......................................................................gagaucauuguUaaagcug.....................   3      1   He1
.......................................................................gagaucauugugaaagcuU.....................   6      1   He1
.......................................................................gagaucauugugCaagcug.....................   4      1   He1
.......................................................................gagaucauAgugaaagcug.....................   1      1   He1
.......................................................................gagaucaAugugaaagcug.....................   1      1   He1
.......................................................................gagaucauugugaaagcGg.....................   1      1   He1
.......................................................................Uagaucauugugaaagcug.....................   2      1   He1
.......................................................................gagaAcauugugaaagcug.....................   11     1   He1
.......................................................................gagaucaGugugaaagcug.....................   3      1   He1
.......................................................................Aagaucauugugaaagcug.....................   3      1   He1
.......................................................................gaCaucauugugaaagcug.....................   2      1   He1
.......................................................................Cagaucauugugaaagcug.....................   1      1   He1
.......................................................................gagauAauugugaaagcug.....................   1      1   He1
.......................................................................gCgaucauugugaaagcug.....................   5      1   He1
```

Star **Mature**

ggaacucgaaagcgcccuagcuguagacaaaacugguuuucacaaugauuugacagauguguucgauaucugagaucauugugaaagcugauuuuguuguccccaucaucu

....gagaucauugugaaagcCg.....................	3	1	He1
....gagaucauuAugaaagcug.....................	1	1	He1
....gagaucauCgugaaagcug.....................	2	1	He1
....gagaucauugugaaagcug.....................	212	0	He1
....gagaucauugugaaagcuUa....................	3	1	He1
....gagaucauugugaaagcugC....................	1	1	He1
....gaCaucauugugaaagcuga....................	5	1	He1
....Aagaucauugugaaagcuga....................	3	1	He1
....gagaucauAgugaaagcuga....................	2	1	He1
....gagUucauugugaaagcuga....................	7	1	He1
....gagaucauugugCaagcuga....................	1	1	He1
....gagaucauugugaaagcCga....................	1	1	He1
....gaUaucauugugaaagcuga....................	1	1	He1
....gagaucauugugaaagcuAa....................	1	1	He1
....Uagaucauugugaaagcuga....................	1	1	He1
....gagaGcauugugaaagcuga....................	17	1	He1
....gagaucauuguCaaagcuga....................	1	1	He1
....gagaucaGugugaaagcuga....................	3	1	He1
....gagaucaAugugaaagcuga....................	1	1	He1
....gagaucauugugaCagcuga....................	1	1	He1
....gagaucauCgugaaagcuga....................	2	1	He1
....gagaucauugugaaagcugU....................	2	1	He1
....gagaAcauugugaaagcuga....................	2	1	He1
....gagaucauugugaaagcuga....................	110	0	He1
....gagaGcauugugaaagcugau...................	19	1	He1
....gCgaucauugugaaagcugau...................	7	1	He1
....Cagaucauugugaaagcugau...................	1	1	He1
....gagaucauAgugaaagcugau...................	1	1	He1
....gagauUauugugaaagcugau...................	1	1	He1
....gUgaucauugugaaagcugau...................	1	1	He1
....gagaucauugugaaagcuUau...................	1	1	He1
....gaCaucauugugaaagcugau...................	9	1	He1
....gagaucauugugaCagcugau...................	1	1	He1
....gagaucauugugCaagcugau...................	1	1	He1
....gagaucCuugugaaagcugau...................	1	1	He1
....gagaucauugugaaaUcugau...................	1	1	He1
....gagUucauugugaaagcugau...................	17	1	He1
....gagaucauCgugaaagcugau...................	3	1	He1
....gagaucauugugaaagcugaC...................	8	1	He1
....gagaucauugugaaagAugau...................	3	1	He1
....gagaucauugAgaaagcugau...................	1	1	He1
....gagauAauugugaaagcugau...................	1	1	He1
....gagaucauugugaaagcugaA...................	1	1	He1
....gagaucauugugaaGgcugau...................	3	1	He1
....gaUaucauugugaaagcugau...................	1	1	He1
....gagaucauugugaaUgcugau...................	1	1	He1
....gagaAcauugugaaagcugau...................	2	1	He1
....gagaucauugugaaagcuAau...................	1	1	He1
....gGgaucauugugaaagcugau...................	1	1	He1
....gagaucauugugaaagcugau...................	271	0	He1
....gagaucauugCgaaagcugau...................	1	1	He1
....gagaGcauugugaaagcugauu..................	20	1	He1
....gagaucauuguUaaagcugauu..................	3	1	He1
....gaCaucauugugaaagcugauu..................	4	1	He1
....gagaCcauugugaaagcugauu..................	2	1	He1
....gagaucauugugaaagcugauC..................	2	1	He1
....gagaucauugugaaagcuUauu..................	3	1	He1
....gagaucauugugaaagcGgauu..................	1	1	He1
....gagaucauugugaaaUcugauu..................	1	1	He1
....gagUucauugugaaagcugauu..................	7	1	He1
....gagaucUuugugaaagcugauu..................	1	1	He1
....Aagaucauugugaaagcugauu..................	1	1	He1
....gagaAcauugugaaagcugauu..................	4	1	He1
....gagaucauuCugaaagcugauu..................	1	1	He1
....gagaucauAgugaaagcugauu..................	1	1	He1
....gagaucaAugugaaagcugauu..................	1	1	He1
....gagaucauugugaaagcugauA..................	6	1	He1
....gagaucauugugaUagcugauu..................	1	1	He1
....Uagaucauugugaaagcugauu..................	1	1	He1
....gagaucaGugugaaagcugauu..................	1	1	He1

Star **Mature**

```
ggaacucgaaagcgcccuagcuguagacaaaacugguuuucacaaugauuugacagauguguucgauaucugagaucauugugaaagcugauuuuguugucccaucaucu
.......................................................................gCgaucauugugaaagcugauu.................	4	1	He1
.......................................................................gagaucauugugaaagcugauG.................	3	1	He1
.......................................................................gagaucauugugaaagcugauu.................	191	0	He1
.......................................................................gagaucauugugaaagcugauuA................	8	1	He1
.......................................................................gagaucauugugaaagcugauuu................	1	0	He1
.......................................................................gagaGcauugugaaagcugauuu................	2	1	He1
........................................................................agaucauugugaaagcug....................	4	0	He1
........................................................................agaucauugugaaCgcuga...................	1	1	He1
........................................................................agaucauugugaaagcuga...................	4	0	He1
........................................................................Ugaucauugugaaagcugau..................	2	1	He1
........................................................................agaucauugugaaagcugau..................	2	0	He1
..........................................................................aucauugugaaagcugauuu................	1	0	He1
...........................................................................ucauGgugaaagcugauu.................	1	1	He1
...........................................................................ucauCgugaaagcugauu.................	1	1	He1
...........................................................................ucauugugaaagcugauu.................	32	0	He1
...........................................................................ucauugugaaagcugauuuuU..............	1	1	He1
............................................................................cauugugaaagcugauuu................	1	0	He1
............................................................................cauugugaaagcugauuA................	1	1	He1

................................cugguuuucacaauUauuuga.........................................................	1	1	Ch1
................................cuggCuuucacaaugauuuga.........................................................	1	1	Ch1
................................cuggAuuucacaaugauuuga.........................................................	1	1	Ch1
................................cuggGuuucacaaugauuuga.........................................................	1	1	Ch1
................................cugguuuucacaaugauuuga.........................................................	39	0	Ch1
................................cugguuuCcacaaugauuuga.........................................................	1	1	Ch1
................................cuggAuuucacaaugauuugac........................................................	1	1	Ch1
................................cuCguuuucacaaugauuugac........................................................	1	1	Ch1
................................cugguuuucacaaugauuugac........................................................	16	0	Ch1
................................cugguuuucCcaaugauuugac........................................................	1	1	Ch1
................................cugguuuucacaaugauuugaca.......................................................	15	0	Ch1
................................cugguuuucacaaugauuugacaga.....................................................	2	0	Ch1
.................................ugguuuucacaaugauuuga.........................................................	1	0	Ch1
.................................ugguuuucacaaugauuugac........................................................	4	0	Ch1
.................................ugguuuucacaaugauuugacagauU...................................................	1	1	Ch1
..................................................................uaucugagaucauugugaa.........................	1	0	Ch1
..................................................................uaucugagaucauugugaaa........................	4	0	Ch1
..................................................................uaucugagaucauugugaaag.......................	1	0	Ch1
..................................................................uaucugagaucauugugUaag.......................	1	1	Ch1
..................................................................uaucCgagaucauugugaaagc......................	2	1	Ch1
..................................................................uaucugagaucauugugaaagA......................	2	1	Ch1
..................................................................uaucugagaucauuAugaaagc......................	1	1	Ch1
..................................................................uaucugagaucauugugaaagc......................	32	0	Ch1
..................................................................uaucGgagaucauugugaaagc......................	1	1	Ch1
..................................................................uaucAgagaucauugugaaagc......................	1	1	Ch1
..................................................................uaucugagaucauugugUaagc......................	1	1	Ch1
..................................................................uaucugagaucauugugaaagU......................	1	1	Ch1
..................................................................uaucugagGucauugugaaagc......................	1	1	Ch1
..................................................................uaucugagaucauugugaaagcG.....................	1	1	Ch1
..................................................................uaucugagaucauugugaaagcC.....................	2	1	Ch1
..................................................................uaucugagaucauugugaaagcA.....................	5	1	Ch1
..................................................................uaucugagaucauugugaaagcu.....................	10	0	Ch1
..................................................................uaucugagaucauugugaaagcuU....................	1	1	Ch1
...................................................................aucugagaucauugugaaag.......................	1	0	Ch1
...................................................................aucugagaucauugugaaagc......................	4	0	Ch1
...................................................................aucugagaucauugugaaagcA.....................	1	1	Ch1
...................................................................aucugagaucauugugaaagcu.....................	20	0	Ch1
...................................................................aucugagaucauugugaaagcC.....................	4	1	Ch1
...................................................................aucuUagaucauugugaaagcu.....................	2	1	Ch1
...................................................................aucugagaucauugugaaagcug....................	2	0	Ch1
....................................................................ucugagaucauugugaaagcu.....................	2	0	Ch1
....................................................................ucugagaucauugugaaagcuU....................	4	1	Ch1
....................................................................ucugUgaucauugugaaagcug....................	1	1	Ch1
....................................................................ucugagaucauugugaaagcug....................	17	0	Ch1
....................................................................ucugagaucauugugaaagcuA....................	1	1	Ch1
....................................................................Acugagaucauugugaaagcuga...................	1	1	Ch1
.....................................................................cugagaucauugugaaagcA.....................	1	1	Ch1
.....................................................................cugagaucauugugaaagcu.....................	3	0	Ch1
.....................................................................cugagaucCuugugaaagcug....................	1	1	Ch1
.....................................................................cugagaucauugugaaagcug....................	20	0	Ch1
.....................................................................Augagaucauugugaaagcug....................	1	1	Ch1
```

Star Mature

ggaacucgaaagcgcccuagcuguagacaaaacugguuuucacaaugauuugacagauguguucgauaucugagaucauugugaaagcugauuuuguuguccccaucaucu			
....cugagaucauugugaaagcugU....................	1	1	Ch1
....cugagaucauugugaaagcuga....................	14	0	Ch1
....cugagaucauugugaaaAcuga....................	1	1	Ch1
....cugaUaucauugugaaagcugau...................	1	1	Ch1
....cugagaucauugugaaagcugaC...................	1	1	Ch1
....cugagaucauugugaaagcugau...................	20	0	Ch1
....cugagaucauugugaaagcugauu..................	5	0	Ch1
....ugagaucauugugaaagc.......................	73	0	Ch1
....ugagaucauugugaaagU.......................	1	1	Ch1
....Cgagaucauugugaaagc.......................	1	1	Ch1
....ugagUucauugugaaagc.......................	2	1	Ch1
....ugagaucauugugaaagUu......................	10	1	Ch1
....ugagaCcauugugaaagcu......................	4	1	Ch1
....ugagaAcauugugaaagcu......................	1	1	Ch1
....ugagaucGuugugaaagcu......................	2	1	Ch1
....ugagaucauAgugaaagcu......................	4	1	Ch1
....ugagaucauugugaaagcG......................	39	1	Ch1
....ugagaucauugugaaCgcu......................	1	1	Ch1
....ugagauUauugugaaagcu......................	4	1	Ch1
....Ngagaucauugugaaagcu......................	10	1	Ch1
....ugagaucauCgugaaagcu......................	2	1	Ch1
....ugagaucauugugUaagcu......................	1	1	Ch1
....ugagaucCuugugaaagcu......................	1	1	Ch1
....ugaUaucauugugaaagcu......................	39	1	Ch1
....ugagaucauuguUaaagcu......................	3	1	Ch1
....ugagaucauugugaaGgcu......................	4	1	Ch1
....ugCgaucauugugaaagcu......................	42	1	Ch1
....uUagaucauugugaaagcu......................	4	1	Ch1
....ugagaucauuguAaaagcu......................	3	1	Ch1
....ugagaucaGugugaaagcu......................	1	1	Ch1
....ugagaucauuUugaaagcu......................	1	1	Ch1
....uCagaucauugugaaagcu......................	8	1	Ch1
....ugagaucauugugaaaAcu......................	4	1	Ch1
....ugagaGcauugugaaagcu......................	9	1	Ch1
....Cgagaucauugugaaagcu......................	22	1	Ch1
....ugagaucauugugaaagcC......................	2126	1	Ch1
....ugUgaucauugugaaagcu......................	2	1	Ch1
....ugaAaucauugugaaagcu......................	2	1	Ch1
....Ggagaucauugugaaagcu......................	3	1	Ch1
....uAagaucauugugaaagcu......................	9	1	Ch1
....ugagaucauugugaaagcA......................	1243	1	Ch1
....ugagaucauuCugaaagcu......................	1	1	Ch1
....ugagCucauugugaaagcu......................	5	1	Ch1
....ugagaucauugugaUagcu......................	2	1	Ch1
....ugagaucauugugCaagcu......................	84	1	Ch1
....ugagaucauugugaaagcu......................	16570	0	Ch1
....ugagauGauugugaaagcu......................	3	1	Ch1
....ugagaucauugugaGagcu......................	4	1	Ch1
....ugagaucauuguCaaagcu......................	3	1	Ch1
....ugagaucauuAugaaagcu......................	7	1	Ch1
....ugagaucaAugugaaagcu......................	20	1	Ch1
....ugagaucauugCgaaagcu......................	5	1	Ch1
....ugagaucauugugaaUgcu......................	19	1	Ch1
....ugagaucauugGgaaagcu......................	5	1	Ch1
....ugagUucauugugaaagcu......................	331	1	Ch1
....ugGgaucauugugaaagcu......................	3	1	Ch1
....ugagGucauugugaaagcu......................	206	1	Ch1
....ugagaucauugugaaaUcu......................	1	1	Ch1
....ugagaucauugAgaaagcu......................	1	1	Ch1
....ugagaucauugugGaagcu......................	5	1	Ch1
....Agagaucauugugaaagcu......................	31	1	Ch1
....ugagaucUuugugaaagcu......................	50	1	Ch1
....ugagaucauugugaaaCcu......................	1	1	Ch1
....ugaUaucauugugaaagcug.....................	169	1	Ch1
....ugagaucauugugaaUgcug.....................	72	1	Ch1
....ugagaucaGugugaaagcug.....................	3	1	Ch1
....uUagaucauugugaaagcug.....................	15	1	Ch1
....ugagaucauCgugaaagcug.....................	14	1	Ch1
....ugagaucauugugCaagcug.....................	276	1	Ch1
....ugagaucauugugaaaAcug.....................	12	1	Ch1

Star **Mature**

ggaacucgaaagcgcccuagcuguagacaaaacugguuuucacaaugauuugacagauguguucgauaucugagaucauugugaaagcugauuuuguugucccaucaucu			
....Ggagaucauugugaaagcug....................	14	1	Ch1
....ugagUucauugugaaagcug....................	1424	1	Ch1
....ugagaucauugugaaaUcug....................	11	1	Ch1
....uCagaucauugugaaagcug....................	34	1	Ch1
....ugagaucauugugaaagcuA....................	286	1	Ch1
....ugagaucauuCugaaagcug....................	4	1	Ch1
....Ngagaucauugugaaagcug....................	63	1	Ch1
....ugagaucauGgugaaagcug....................	4	1	Ch1
....ugagaucauugugaaCgcug....................	5	1	Ch1
....ugagaucauugugaaagcAg....................	34	1	Ch1
....ugagaucauugugaCagcug....................	3	1	Ch1
....ugagaucauuguUaaagcug....................	14	1	Ch1
....ugaAaucauugugaaagcug....................	6	1	Ch1
....ugagaucaAugugaaagcug....................	101	1	Ch1
....ugagaucauugugaGagcug....................	20	1	Ch1
....ugagaucauuUugaaagcug....................	4	1	Ch1
....ugagGucauugugaaagcug....................	980	1	Ch1
....ugagaucauugugaaagUug....................	73	1	Ch1
....Cgagaucauugugaaagcug....................	134	1	Ch1
....ugCgaucauugugaaagcug....................	196	1	Ch1
....ugagaucauuguAaaagcug....................	12	1	Ch1
....ugagaucauuguCaaagcug....................	4	1	Ch1
....ugagaucauuAugaaagcug....................	27	1	Ch1
....Agagaucauugugaaagcug....................	177	1	Ch1
....ugagaGcauugugaaagcug....................	24	1	Ch1
....ugGgaucauugugaaagcug....................	11	1	Ch1
....ugagaucauugAgaaagcug....................	5	1	Ch1
....ugagaucauugugaaGgcug....................	20	1	Ch1
....ugagaAcauugugaaagcug....................	17	1	Ch1
....ugagauUauugugaaagcug....................	15	1	Ch1
....ugagauAauugugaaagcug....................	5	1	Ch1
....ugagaucauugugaUagcug....................	14	1	Ch1
....ugagaucCuugugaaagcug....................	2	1	Ch1
....ugagaucauugGgaaagcug....................	27	1	Ch1
....uAagaucauugugaaagcug....................	39	1	Ch1
....ugagaucauugCgaaagcug....................	14	1	Ch1
....ugagaucauugugaaagcCg....................	173	1	Ch1
....ugagaucauugugaaagcug....................	77259	0	Ch1
....ugagaucauugugaaaCcug....................	1	1	Ch1
....ugaCaucauugugaaagcug....................	4	1	Ch1
....ugagaucauAgugaaagcug....................	11	1	Ch1
....ugagaucauugugaaagcuC....................	22	1	Ch1
....ugagaucauugugUaagcug....................	22	1	Ch1
....ugagaucauugugaaagcuU....................	257	1	Ch1
....ugagaucGuugugaaagcug....................	21	1	Ch1
....ugagauGauugugaaagcug....................	4	1	Ch1
....ugagaucauugugaaagcGg....................	58	1	Ch1
....ugagaucUuugugaaagcug....................	197	1	Ch1
....ugagaucauugugGaagcug....................	17	1	Ch1
....ugagaCcauugugaaagcug....................	14	1	Ch1
....ugUgaucauugugaaagcug....................	12	1	Ch1
....ugagCucauugugaaagcug....................	12	1	Ch1
....ugagaucauuguAaaagcuga...................	2	1	Ch1
....ugagaucauugugaaagAuga...................	1	1	Ch1
....ugagaucauugugaaGgcuga...................	1	1	Ch1
....ugagaucauCgugaaagcuga...................	7	1	Ch1
....ugagaucauugugCaagcuga...................	68	1	Ch1
....Ggagaucauugugaaagcuga...................	6	1	Ch1
....ugaCaucauugugaaagcuga...................	2	1	Ch1
....ugaUaucauugugaaagcuga...................	46	1	Ch1
....ugUgaucauugugaaagcuga...................	5	1	Ch1
....ugagaucUuugugaaagcuga...................	39	1	Ch1
....ugGgaucauugugaaagcuga...................	8	1	Ch1
....ugagaucauugugaaaUcuga...................	3	1	Ch1
....uAagaucauugugaaagcuga...................	16	1	Ch1
....ugagaucauugugaaagcuga...................	21281	0	Ch1
....ugagaucauugugaaUgcuga...................	20	1	Ch1
....uCagaucauugugaaagcuga...................	5	1	Ch1
....ugagaucauuguCaaagcuga...................	2	1	Ch1
....ugagauUauugugaaagcuga...................	2	1	Ch1

```
                                Star                                 Mature
ggaacucgaaagcgcccuagcuguagacaaaacugguuuucacaaugauuugacagauguguucgauaucugagaucauugugaaagcugauuuuguuguccccaucaucu
......................................................................ugagaucauugAgaaagcuga...................   4     1   Ch1
......................................................................ugagaucauugugaaagcugG...................   5     1   Ch1
......................................................................ugagaAcauugugaaagcuga...................   5     1   Ch1
......................................................................uUagaucauugugaaagcuga...................   5     1   Ch1
......................................................................ugagaucauugugaGagcuga...................   3     1   Ch1
......................................................................Agagaucauugugaaagcuga...................   46    1   Ch1
......................................................................ugagaucauugugaaagcugU...................   342   1   Ch1
......................................................................ugagaucauugugaaagcuCa...................   1     1   Ch1
......................................................................ugagaucauugugaaagcugC...................   54    1   Ch1
......................................................................ugaAaucauugugaaagcuga...................   2     1   Ch1
......................................................................ugagaucaAugugaaagcuga...................   29    1   Ch1
......................................................................ugagaucauugugaaaAcuga...................   4     1   Ch1
......................................................................ugagaucauuguUaaagcuga...................   2     1   Ch1
......................................................................ugagaCcauugugaaagcuga...................   6     1   Ch1
......................................................................ugagaucauugugUaagcuga...................   7     1   Ch1
......................................................................ugagaGcauugugaaagcuga...................   8     1   Ch1
......................................................................ugagaucauuCugaaagcuga...................   4     1   Ch1
......................................................................ugagaucauugugaaagcCga...................   2     1   Ch1
......................................................................ugagUucauugugaaagcuga...................   394   1   Ch1
......................................................................ugagauGauugugaaagcuga...................   1     1   Ch1
......................................................................ugagaucaGugugaaagcuga...................   2     1   Ch1
......................................................................ugagaucauugugaaagcuUa...................   49    1   Ch1
......................................................................ugagaucauuAugaaagcuga...................   15    1   Ch1
......................................................................ugagaucauugugaaaCcuga...................   1     1   Ch1
......................................................................ugagGucauugugaaagcuga...................   299   1   Ch1
......................................................................ugagaucauugugaUagcuga...................   4     1   Ch1
......................................................................ugagaucauugugaaagcuAa...................   4     1   Ch1
......................................................................ugagaucauugCgaaagcuga...................   5     1   Ch1
......................................................................ugCgaucauugugaaagcuga...................   86    1   Ch1
......................................................................ugagaucauugugaaagcAga...................   1     1   Ch1
......................................................................ugagaucauugugaaagcGga...................   14    1   Ch1
......................................................................ugagaucauugGgaaagcuga...................   11    1   Ch1
......................................................................ugagaucauGgugaaagcuga...................   1     1   Ch1
......................................................................ugagCucauugugaaagcuga...................   4     1   Ch1
......................................................................Cgagaucauugugaaagcuga...................   31    1   Ch1
......................................................................Ngagaucauugugaaagcuga...................   20    1   Ch1
......................................................................ugagaucauAgugaaagcuga...................   3     1   Ch1
......................................................................ugagaucGuugugaaagcuga...................   6     1   Ch1
......................................................................ugagaucauugugaaagGuga...................   2     1   Ch1
......................................................................ugagaucauugugGaagcuga...................   8     1   Ch1
......................................................................ugagaucauugugaaagUuga...................   14    1   Ch1
......................................................................uCagaucauugugaaagcugau..................   19    1   Ch1
......................................................................ugagaucauugugaaUgcugau..................   27    1   Ch1
......................................................................ugagaucauugugaaagcuAau..................   9     1   Ch1
......................................................................Cgagaucauugugaaagcugau..................   52    1   Ch1
......................................................................ugagaucGuugugaaagcugau..................   3     1   Ch1
......................................................................ugagaucauugugaUagcugau..................   6     1   Ch1
......................................................................uUagaucauugugaaagcugau..................   7     1   Ch1
......................................................................ugagaucaGugugaaagcugau..................   1     1   Ch1
......................................................................ugUgaucauugugaaagcugau..................   10    1   Ch1
......................................................................ugagaucauugugaaagAugau..................   1     1   Ch1
......................................................................ugagaucauugugaaagcugaA..................   298   1   Ch1
......................................................................ugagaucauugugaaagcugUu..................   88    1   Ch1
......................................................................Ggagaucauugugaaagcugau..................   6     1   Ch1
......................................................................ugagaucCuugugaaagcugau..................   3     1   Ch1
......................................................................ugagaAcauugugaaagcugau..................   4     1   Ch1
......................................................................ugCgaucauugugaaagcugau..................   82    1   Ch1
......................................................................ugagaucauugCgaaagcugau..................   3     1   Ch1
......................................................................ugagaucauugugCaagcugau..................   129   1   Ch1
......................................................................ugagaucauugugGaagcugau..................   9     1   Ch1
......................................................................ugagaucauugugaaaUcugau..................   5     1   Ch1
......................................................................ugagaucauugugaaagUugau..................   28    1   Ch1
......................................................................ugagaGcauugugaaagcugau..................   8     1   Ch1
......................................................................ugagaucaAugugaaagcugau..................   51    1   Ch1
......................................................................ugagaucauugGgaaagcugau..................   7     1   Ch1
......................................................................ugagaucauugAgaaagcugau..................   2     1   Ch1
......................................................................ugagaucauuguCaaagcugau..................   1     1   Ch1
......................................................................ugagaucauugugaaagcugaC..................   706   1   Ch1
......................................................................ugagaucauuCugaaagcugau..................   2     1   Ch1
......................................................................ugagaucauugugaaagcugGu..................   8     1   Ch1
```

Star Mature

ggaacucgaaagcgcccuagcuguagacaaaacugguuuucacaaugauuugacagauguguucgauaucugagaucauugugaaagcugauuuuguugucccaucaucu			
....ugaAaucauugugaaagcugau..................	6	1	Ch1
....ugaNaucauugugaaagcugau..................	1	1	Ch1
....ugagaucauugugaaagcugau..................	33933	0	Ch1
....ugagaucauugugaaagcuCau..................	3	1	Ch1
....ugagaucauuguUaaagcugau..................	2	1	Ch1
....ugagaucauuUugaaagcugau..................	2	1	Ch1
....ugagauUauugugaaagcugau..................	13	1	Ch1
....ugagaucauugugaGagcugau..................	9	1	Ch1
....ugagGucauugugaaagcugau..................	428	1	Ch1
....ugagaucauugugaaagcCgau..................	10	1	Ch1
....ugagCucauugugaaagcugau..................	1	1	Ch1
....ugagaucauugugUaagcugau..................	5	1	Ch1
....ugaUaucauugugaaagcugau..................	64	1	Ch1
....Agagaucauugugaaagcugau..................	63	1	Ch1
....ugagUucauugugaaagcugau..................	606	1	Ch1
....ugagaCcauugugaaagcugau..................	8	1	Ch1
....ugagaucauAgugaaagcugau..................	2	1	Ch1
....ugagaucauugugaaagcugCu..................	23	1	Ch1
....uAagaucauugugaaagcugau..................	18	1	Ch1
....ugagaucauugugaCagcugau..................	1	1	Ch1
....ugagaucauugugaaGgcugau..................	5	1	Ch1
....ugagaucauugugaaagcuUau..................	67	1	Ch1
....ugagaucauugugaaagcugaG..................	15	1	Ch1
....ugagaucauuAugaaagcugau..................	15	1	Ch1
....ugagaucauuguAaaagcugau..................	10	1	Ch1
....ugagaucauugugaaagcGgau..................	9	1	Ch1
....ugagaucUuugugaaagcugau..................	82	1	Ch1
....ugagaucauugugaaagcAgau..................	1	1	Ch1
....Ngagaucauugugaaagcugau..................	30	1	Ch1
....ugagaucauGgugaaagcugau..................	1	1	Ch1
....ugagaucauugugaaaAcugau..................	5	1	Ch1
....ugagaucauugugaaCgcugau..................	4	1	Ch1
....ugagauAauugugaaagcugau..................	2	1	Ch1
....ugGgaucauugugaaagcugau..................	6	1	Ch1
....ugagaucauCgugaaagcugau..................	7	1	Ch1
....uAagaucauugugaaagcugauu.................	5	1	Ch1
....ugagUucauugugaaagcugauu.................	237	1	Ch1
....ugagaucauugugCaagcugauu.................	51	1	Ch1
....ugagaucauugugaaGgcugauu.................	2	1	Ch1
....ugagaucauugugaaagcugUuu.................	7	1	Ch1
....Agagaucauugugaaagcugauu.................	22	1	Ch1
....ugCgaucauugugaaagcugauu.................	29	1	Ch1
....ugagaucauugCgaaagcugauu.................	1	1	Ch1
....ugagaucauugugaaagcugauG.................	114	1	Ch1
....uCagaucauugugaaagcugauu.................	3	1	Ch1
....ugagauUauugugaaagcugauu.................	20	1	Ch1
....ugagaucauCgugaaagcugauu.................	3	1	Ch1
....ugagaucauugGgaaagcugauu.................	7	1	Ch1
....ugaAaucauugugaaagcugauu.................	3	1	Ch1
....ugagaGcauugugaaagcugauu.................	1	1	Ch1
....ugagaCcauugugaaagcugauu.................	3	1	Ch1
....ugagaucauugugaaagcugaCu.................	1	1	Ch1
....ugagaucauugugaaagcugCuu.................	9	1	Ch1
....ugagaucauugugaaCgcugauu.................	1	1	Ch1
....ugagaucauugugaGagcugauu.................	6	1	Ch1
....Ggagaucauugugaaagcugauu.................	4	1	Ch1
....ugagaucauugugaaagcugaAu.................	4	1	Ch1
....ugagaucauGgugaaagcugauu.................	1	1	Ch1
....ugagaucauuguUaaagcugauu.................	5	1	Ch1
....ugagaucauugugaaagcugauC.................	69	1	Ch1
....ugagaucauuCugaaagcugauu.................	1	1	Ch1
....ugagaucauugugaaagcuUauu.................	35	1	Ch1
....ugaUaucauugugaaagcugauu.................	23	1	Ch1
....ugagaucauugugaaUgcugauu.................	12	1	Ch1
....ugagaucauAgugaaagcugauu.................	3	1	Ch1
....Cgagaucauugugaaagcugauu.................	15	1	Ch1
....ugagCucauugugaaagcugauu.................	4	1	Ch1
....ugagaucauugugaaaUcugauu.................	2	1	Ch1
....ugagaucauugugaUagcugauu.................	4	1	Ch1
....ugagaucauugugaaagcCgauu.................	4	1	Ch1

Star   Mature

ggaacucgaaagcgcccuagcuguagacaaaacugguuuucacaaugauuugacagauguguucgauaucugagaucauugugaaagcugauuuuguugucccaucaucu			
....ugagaucauugugaaaAcugauu.................	2	1	Ch1
....ugagaucGuugugaaagcugauu.................	5	1	Ch1
....ugagaucaAugugaaagcugauu.................	23	1	Ch1
....ugagaucauugugaaagcugauu.................	12826	0	Ch1
....ugagaucauugugUaagcugauu.................	2	1	Ch1
....ugagGucauugugaaagcugauu.................	183	1	Ch1
....ugagaucauuAugaaagcugauu.................	8	1	Ch1
....ugagaucUuugugaaagcugauu.................	27	1	Ch1
....ugagaucauugugaaagcGgauu.................	3	1	Ch1
....ugagaucauugugaaagcugauA.................	711	1	Ch1
....ugagaucauugugaaagcugaGu.................	3	1	Ch1
....ugGgaucauugugaaagcugauu.................	5	1	Ch1
....ugagaucauuguAaaagcugauu.................	5	1	Ch1
....ugagaucauugugaaagUugauu.................	7	1	Ch1
....Ngagaucauugugaaagcugauu.................	5	1	Ch1
....ugagauAauugugaaagcugauu.................	1	1	Ch1
....ugagaucauugugaaagcugauAu................	2	1	Ch1
....ugagaucauugugaaagcugaCuu................	1	1	Ch1
....ugagaucauugugaaagcugauuA................	265	1	Ch1
....ugagauUauugugaaagcugauuu................	1	1	Ch1
....ugagaucauugugaaagcugauuu................	429	0	Ch1
....Agagaucauugugaaagcugauuu................	1	1	Ch1
....ugagGucauugugaaagcugauuu................	9	1	Ch1
....ugagaucauugugaaUgcugauuu................	1	1	Ch1
....ugagUucauugugaaagcugauuu................	4	1	Ch1
....ugCgaucauugugaaagcugauuu................	1	1	Ch1
....ugagaucauugugaaagcugauGu................	1	1	Ch1
....ugagaucauugugaaagcugauuC................	6	1	Ch1
....ugagaucauugugCaagcugauuu................	1	1	Ch1
....ugaUaucauugugaaagcugauuu................	1	1	Ch1
....ugagaucauugugaaagcugauuuuU..............	2	1	Ch1
....gaCaucauugugaaagcu.....................	3	1	Ch1
....gagaucauugugaaagcC.....................	8	1	Ch1
....Aagaucauugugaaagcu.....................	1	1	Ch1
....gagaucauuguUaaagcu.....................	1	1	Ch1
....gagaGcauugugaaagcu.....................	2	1	Ch1
....Cagaucauugugaaagcu.....................	1	1	Ch1
....gagUucauugugaaagcu.....................	3	1	Ch1
....gagaucauugugaaaAcu.....................	1	1	Ch1
....gagaucauugugaCagcu.....................	1	1	Ch1
....gagaucauugugaaagcA.....................	1	1	Ch1
....gagaucauugugCaagcu.....................	1	1	Ch1
....gagauUauugugaaagcu.....................	1	1	Ch1
....gagaAcauugugaaagcu.....................	1	1	Ch1
....gagaucauugugaaagcu.....................	32	0	Ch1
....gCgaucauugugaaagcug....................	7	1	Ch1
....gagaAcauugugaaagcug....................	5	1	Ch1
....gagaucauugugaaaUcug....................	2	1	Ch1
....gagaucauugugaaagcCg....................	1	1	Ch1
....gagaucauugugaaCgcug....................	1	1	Ch1
....gaAaucauugugaaagcug....................	1	1	Ch1
....gagaucauugugaCagcug....................	1	1	Ch1
....gagaucauugugCaagcug....................	2	1	Ch1
....gagaucauAgugaaagcug....................	2	1	Ch1
....Cagaucauugugaaagcug....................	2	1	Ch1
....gagaucauugugaaagUug....................	1	1	Ch1
....gagauAauugugaaagcug....................	1	1	Ch1
....gagUucauugugaaagcug....................	11	1	Ch1
....Uagaucauugugaaagcug....................	2	1	Ch1
....gagaCcauugugaaagcug....................	2	1	Ch1
....gagaucaGugugaaagcug....................	2	1	Ch1
....gagaGcauugugaaagcug....................	24	1	Ch1
....gagaucauugAgaaagcug....................	1	1	Ch1
....gGgaucauugugaaagcug....................	1	1	Ch1
....gagaucauugugaaagcAg....................	1	1	Ch1
....gagaucauuguUaaagcug....................	1	1	Ch1
....gaCaucauugugaaagcug....................	7	1	Ch1
....gagaucCuugugaaagcug....................	1	1	Ch1
....gagaucauugugaaagcug....................	209	0	Ch1
....gagaucaAugugaaagcug....................	3	1	Ch1

Star Mature

```
ggaacucgaaagcgcccuagcuguagacaaaacugguuuucacaaugauuugacagauguguucgauaucugagaucauugugaaagcugauuuuguuguccccaucaucu
.......................................................................gagaucauuguAaaagcug.....................  1    1  Ch1
.......................................................................gagaucauugugaaagcuU.....................  1    1  Ch1
.......................................................................gagaGcauugugaaagcuga....................  9    1  Ch1
.......................................................................gagaucauCgugaaagcuga....................  1    1  Ch1
.......................................................................gagaCcauugugaaagcuga....................  1    1  Ch1
.......................................................................gagaucauugugaaUgcuga....................  1    1  Ch1
.......................................................................gagaucauugugaaagAuga....................  2    1  Ch1
.......................................................................gagaucaAugugaaagcuga....................  1    1  Ch1
.......................................................................gagaucauAgugaaagcuga....................  1    1  Ch1
.......................................................................Uagaucauugugaaagcuga....................  1    1  Ch1
.......................................................................gagaucauugugaaagcuAa....................  1    1  Ch1
.......................................................................gagaucauugugaCagcuga....................  2    1  Ch1
.......................................................................gagaAcauugugaaagcuga....................  3    1  Ch1
.......................................................................gaCaucauugugaaagcuga....................  3    1  Ch1
.......................................................................gagUucauugugaaagcuga....................  4    1  Ch1
.......................................................................gagaucauugugaaagcuga....................  77   0  Ch1
.......................................................................gCgaucauugugaaagcuga....................  1    1  Ch1
.......................................................................gagaucauugugaaagcugU....................  1    1  Ch1
.......................................................................gagaAcauugugaaagcugau...................  2    1  Ch1
.......................................................................Cagaucauugugaaagcugau...................  1    1  Ch1
.......................................................................gagaucauugugCaagcugau...................  2    1  Ch1
.......................................................................gagaucauugugaaagcugaC...................  2    1  Ch1
.......................................................................gagaucauugugaaagcuCau...................  1    1  Ch1
.......................................................................gUgaucauugugaaagcugau...................  1    1  Ch1
.......................................................................gagaCcauugugaaagcugau...................  1    1  Ch1
.......................................................................gagaucauugugaaagcugaA...................  4    1  Ch1
.......................................................................gagaucauugugaaGgcugau...................  2    1  Ch1
.......................................................................gagaucaGugugaaagcugau...................  2    1  Ch1
.......................................................................Aagaucauugugaaagcugau...................  2    1  Ch1
.......................................................................gagaucauugugaaagcuUau...................  3    1  Ch1
.......................................................................gagaucauugugaCagcugau...................  3    1  Ch1
.......................................................................gagaucauugugaaagcugau...................  141  0  Ch1
.......................................................................gaCaucauugugaaagcugau...................  2    1  Ch1
.......................................................................gCgaucauugugaaagcugau...................  2    1  Ch1
.......................................................................gagUucauugugaaagcugau...................  4    1  Ch1
.......................................................................gagaGcauugugaaagcugau...................  13   1  Ch1
.......................................................................gagaucauugugaaUgcugau...................  1    1  Ch1
.......................................................................gagaucauuguAaaagcugau...................  2    1  Ch1
.......................................................................gagaucauugugaaCgcugau...................  1    1  Ch1
.......................................................................gagaucauugugaaagcugauG..................  1    1  Ch1
.......................................................................gagaucauAgugaaagcugauu..................  1    1  Ch1
.......................................................................gagaGcauugugaaagcugauu..................  10   1  Ch1
.......................................................................gagaucauugugaaagcugauu..................  66   0  Ch1
.......................................................................gagUucauugugaaagcugauu..................  2    1  Ch1
.......................................................................gagaucauugugaaagcugUuu..................  1    1  Ch1
.......................................................................gagaucauugugaaaUcugauu..................  2    1  Ch1
.......................................................................gagaucaAugugaaagcugauu..................  1    1  Ch1
.......................................................................gCgaucauugugaaagcugauu..................  1    1  Ch1
.......................................................................gagaAcauugugaaagcugauu..................  1    1  Ch1
.......................................................................gaCaucauugugaaagcugauu..................  2    1  Ch1
.......................................................................Cagaucauugugaaagcugauu..................  1    1  Ch1
.......................................................................gagaucauugugaaagcugauA..................  2    1  Ch1
.......................................................................gagaucauugugaaagcugauuA.................  4    1  Ch1
.......................................................................gagaucauugugaaagcugauuu.................  1    0  Ch1
........................................................................agaucauugugaaagcug.....................  1    0  Ch1
........................................................................agaucauugugaaagcugau...................  1    0  Ch1
........................................................................Ugaucauugugaaagcugau...................  1    1  Ch1
.........................................................................gaucauugugaaagcugU....................  1    1  Ch1
...........................................................................ucauugugaaagcugau...................  3    0  Ch1

................................cuggGuuucacaaugauuuga..........................................................  1    1  Le1
................................cugguuuucacaaugauuuga..........................................................  17   0  Le1
................................cugguuuucacaaugauuugac.........................................................  7    0  Le1
................................cugguuuucGcaaugauuugac.........................................................  1    1  Le1
................................cugguuuucacaaugauuugaca........................................................  3    0  Le1
................................cuggGuuucacaaugauuugaca........................................................  1    1  Le1
................................cugguuuucacaaugauuugacaga......................................................  1    0  Le1
.................................ugguuuucacaaugauuug...........................................................  1    0  Le1
..................................................................uaucugagaucauugugaa..........................  1    0  Le1
..................................................................uaucugagaucauugugaaa.........................  4    0  Le1
```

Star Mature			
ggaacucgaaagcgcccuagcuguagacaaaacugguuuucacaaugauuugacagauguguucgauaucugagaucauugugaaagcugauuuuguugucccaucaucu			
....uaucugagaucauugugaaag.......................	3	0	Le1
....uaucugagaucauuUugaaagc......................	1	1	Le1
....uaucCgagaucauugugaaagc......................	1	1	Le1
....uaucugagaucauugugaaagc......................	43	0	Le1
....uaucGgagaucauugugaaagc......................	1	1	Le1
....uaucugagaucauugugaaagcC.....................	2	1	Le1
....uaucugagaucauugugaaagcG.....................	1	1	Le1
....uaucugagaucauugugaaagcu.....................	9	0	Le1
....uaucugagaucauugugaaagcA.....................	3	1	Le1
....uaucugagaucauugugaaagcuU....................	3	1	Le1
....Gaucugagaucauugugaaagcugauu.................	1	1	Le1
....aucugagaucauugugaaagc......................	3	0	Le1
....aucugagaucauugugaaagcu.....................	8	0	Le1
....aucugagaucauugugaaagcC.....................	3	1	Le1
....aucuUagaucauugugaaagcu.....................	1	1	Le1
....ucugagaucauugugaaagcu.....................	1	0	Le1
....ucugagaucauugugaaagcuU....................	1	1	Le1
....ucugagaucauugugaaagcug....................	4	0	Le1
....ucugagaucauugugaaagcugau..................	1	0	Le1
....cugagaucauugugaaagc......................	1	0	Le1
....cugagaucauugugaaagcu.....................	4	0	Le1
....Uugagaucauugugaaagcug....................	1	1	Le1
....cugagaucauugugaaagcug....................	14	0	Le1
....cugagaucauugugaaagcugU...................	3	1	Le1
....cugaUaucauugugaaagcuga...................	1	1	Le1
....cugagaucauugugaaagcuga...................	13	0	Le1
....cugagaucauugugaaagcugau..................	6	0	Le1
....cugagaucauugugaaagcugauu.................	1	0	Le1
....ugagaucauugCgaaagc......................	1	1	Le1
....ugagaucauugugaaUgc......................	1	1	Le1
....ugagUucauugugaaagc......................	3	1	Le1
....ugagGucauugugaaagc......................	3	1	Le1
....ugagaucauugugaaagc......................	81	0	Le1
....ugagaucauugugCaagc......................	1	1	Le1
....ugagaucaAugugaaagcu.....................	17	1	Le1
....ugagaAcauugugaaagcu.....................	2	1	Le1
....ugagaucauugugaaagcG.....................	49	1	Le1
....ugagUucauugugaaagcu.....................	191	1	Le1
....ugagaucauuUugaaagcu.....................	2	1	Le1
....ugagaucauugCgaaagcu.....................	2	1	Le1
....ugagaucauugugaGagcu.....................	2	1	Le1
....ugagaucUuugugaaagcu.....................	43	1	Le1
....ugagaucauugugaaagcA.....................	1420	1	Le1
....ugagaucauugugUaagcu.....................	2	1	Le1
....ugagaucauuguUaaagcu.....................	3	1	Le1
....ugaUaucauugugaaagcu.....................	27	1	Le1
....ugagaucauugugaaagcu.....................	11559	0	Le1
....ugagaucauugugaaUgcu.....................	14	1	Le1
....ugagaucauuAugaaagcu.....................	5	1	Le1
....ugagaucauugugaaagUu.....................	10	1	Le1
....ugagaucauugugaUagcu.....................	2	1	Le1
....ugagauAauugugaaagcu.....................	2	1	Le1
....ugagGucauugugaaagcu.....................	131	1	Le1
....uAagaucauugugaaagcu.....................	11	1	Le1
....ugagaucauugugaaGgcu.....................	1	1	Le1
....ugagaucauugugaaaUcu.....................	1	1	Le1
....Ngagaucauugugaaagcu.....................	10	1	Le1
....ugagaucGuugugaaagcu.....................	5	1	Le1
....ugagaucauugugCaagcu.....................	52	1	Le1
....ugGgaucauugugaaagcu.....................	2	1	Le1
....ugagaucauAgugaaagcu.....................	3	1	Le1
....Ggagaucauugugaaagcu.....................	1	1	Le1
....ugaAaucauugugaaagcu.....................	2	1	Le1
....ugagaucauugugaaagcC.....................	1730	1	Le1
....ugagaucauugGgaaagcu.....................	2	1	Le1
....uUagaucauugugaaagcu.....................	2	1	Le1
....ugagaucauGgugaaagcu.....................	1	1	Le1
....uCagaucauugugaaagcu.....................	3	1	Le1
....Cgagaucauugugaaagcu.....................	20	1	Le1
....ugagaucauuCugaaagcu.....................	1	1	Le1

```
                                   Star                                     Mature
ggaacucgaaagcgcccuagcuguagacaaaacugguuuucacaaugauuugacagauguguucgauaucugagaucauugugaaagcugauuuuguugucccaucaucu
......................................................................Agagaucauugugaaagcu.....................  15     1  Le1
......................................................................ugagaGcauugugaaagcu.....................  6      1  Le1
......................................................................ugUgaucauugugaaagcu.....................  3      1  Le1
......................................................................ugagaucauugugaaCgcu.....................  4      1  Le1
......................................................................ugagaucauugugaaaAcu.....................  1      1  Le1
......................................................................ugagaucauugugaaagGu.....................  1      1  Le1
......................................................................ugagaCcauugugaaagcu.....................  2      1  Le1
......................................................................ugagauUauugugaaagcu.....................  5      1  Le1
......................................................................ugagCucauugugaaagcu.....................  3      1  Le1
......................................................................ugagaucauugAgaaagcu.....................  3      1  Le1
......................................................................ugCgaucauugugaaagcu.....................  24     1  Le1
......................................................................ugagaucauuUugaaagcug....................  1      1  Le1
......................................................................ugagaucaAugugaaagcug....................  70     1  Le1
......................................................................ugagaucauugugaGagcug....................  13     1  Le1
......................................................................ugagaucauugugaaagcug....................  45964  0  Le1
......................................................................uUagaucauugugaaagcug....................  11     1  Le1
......................................................................ugagaucaGugugaaagcug....................  3      1  Le1
......................................................................Agagaucauugugaaagcug....................  96     1  Le1
......................................................................Cgagaucauugugaaagcug....................  70     1  Le1
......................................................................ugagaucauugugaaaCcug....................  1      1  Le1
......................................................................ugagaucauugugaaCgcug....................  3      1  Le1
......................................................................ugagaucauugugaaaAcug....................  8      1  Le1
......................................................................ugagauAauugugaaagcug....................  4      1  Le1
......................................................................ugagaucauugugaUagcug....................  10     1  Le1
......................................................................ugagaucauuCugaaagcug....................  3      1  Le1
......................................................................ugagaucauugugaaagcuC....................  16     1  Le1
......................................................................ugagaucauugugaaagcCg....................  118    1  Le1
......................................................................ugagGucauugugaaagcug....................  591    1  Le1
......................................................................ugagaucauugugaaUgcug....................  34     1  Le1
......................................................................ugagaAcauugugaaagcug....................  6      1  Le1
......................................................................uCagaucauugugaaagcug....................  15     1  Le1
......................................................................ugGgaucauugugaaagcug....................  13     1  Le1
......................................................................ugagaucauugugaaagcGg....................  37     1  Le1
......................................................................ugagaucauugugCaagcug....................  162    1  Le1
......................................................................ugagaucauugugaaagcAg....................  29     1  Le1
......................................................................ugagaucauuAugaaagcug....................  15     1  Le1
......................................................................ugagaucauugugaaaUcug....................  9      1  Le1
......................................................................ugUgaucauugugaaagcug....................  9      1  Le1
......................................................................ugagaucGuugugaaagcug....................  10     1  Le1
......................................................................ugagaucauugugGaagcug....................  6      1  Le1
......................................................................ugagaucauuguUaaagcug....................  5      1  Le1
......................................................................ugagauUauugugaaagcug....................  19     1  Le1
......................................................................ugagaGcauugugaaagcug....................  15     1  Le1
......................................................................ugagaucauugugaaagcuA....................  202    1  Le1
......................................................................ugagCucauugugaaagcug....................  6      1  Le1
......................................................................ugCgaucauugugaaagcug....................  141    1  Le1
......................................................................ugaUaucauugugaaagcug....................  102    1  Le1
......................................................................ugagaucauugugaCagcug....................  2      1  Le1
......................................................................ugagaucauugugaaGgcug....................  15     1  Le1
......................................................................ugagaucauCgugaaagcug....................  10     1  Le1
......................................................................ugagaucauAgugaaagcug....................  9      1  Le1
......................................................................Ggagaucauugugaaagcug....................  11     1  Le1
......................................................................ugagaucauugAgaaagcug....................  3      1  Le1
......................................................................ugagaucauugugaaagUug....................  52     1  Le1
......................................................................ugagaucauGgugaaagcug....................  1      1  Le1
......................................................................ugaAaucauugugaaagcug....................  6      1  Le1
......................................................................ugagaucauugCgaaagcug....................  15     1  Le1
......................................................................uAagaucauugugaaagcug....................  18     1  Le1
......................................................................ugagaucUuugugaaagcug....................  106    1  Le1
......................................................................ugagaucauugGgaaagcug....................  7      1  Le1
......................................................................ugagUucauugugaaagcug....................  834    1  Le1
......................................................................ugagaCcauugugaaagcug....................  11     1  Le1
......................................................................ugagaucauuguCaaagcug....................  3      1  Le1
......................................................................ugagauGauugugaaagcug....................  1      1  Le1
......................................................................ugaCaucauugugaaagcug....................  1      1  Le1
......................................................................ugagaucauugugaaagcuU....................  136    1  Le1
......................................................................ugagaucauuguAaaagcug....................  9      1  Le1
......................................................................ugagaucauugugUaagcug....................  13     1  Le1
......................................................................ugagaucCuugugaaagcug....................  2      1  Le1
......................................................................Ngagaucauugugaaagcug....................  32     1  Le1
```

```
                                Star                                 Mature
ggaacucgaaagcgcccuagcuguagacaaaacugguuuucacaaugauuugacagauguguucgauaucugagaucauugugaaagcugauuuuguuguccccaucaucu
......................................................................ugagaucauugugaaagcAga....................   1      1    Le1
......................................................................ugagaucauugugUaagcuga....................   5      1    Le1
......................................................................ugagaucauugugaGagcuga....................   2      1    Le1
......................................................................uUagaucauugugaaagcuga....................   2      1    Le1
......................................................................uCagaucauugugaaagcuga....................   10     1    Le1
......................................................................ugaAaucauugugaaagcuga....................   1      1    Le1
......................................................................ugagaAcauugugaaagcuga....................   2      1    Le1
......................................................................ugagaucauugAgaaagcuga....................   3      1    Le1
......................................................................Ngagaucauugugaaagcuga....................   12     1    Le1
......................................................................ugagaucauugGgaaagcuga....................   5      1    Le1
......................................................................ugagaucauugugaaagUuga....................   17     1    Le1
......................................................................ugagaucGuugugaaagcuga....................   2      1    Le1
......................................................................ugagaucauugugaaaAcuga....................   2      1    Le1
......................................................................ugagaucauuguCaaagcuga....................   3      1    Le1
......................................................................Cgagaucauugugaaagcuga....................   34     1    Le1
......................................................................ugagauUauugugaaagcuga....................   7      1    Le1
......................................................................ugagaucauugugCaagcuga....................   66     1    Le1
......................................................................ugagaucauuCugaaagcuga....................   3      1    Le1
......................................................................ugaUaucauugugaaagcuga....................   30     1    Le1
......................................................................ugagaucauugugaaagcGga....................   4      1    Le1
......................................................................ugagaCcauugugaaagcuga....................   2      1    Le1
......................................................................ugagaucauugCgaaagcuga....................   6      1    Le1
......................................................................Agagaucauugugaaagcuga....................   43     1    Le1
......................................................................ugagaucauuguUaaagcuga....................   2      1    Le1
......................................................................ugagaucauugugaaaCcuga....................   1      1    Le1
......................................................................ugagCucauugugaaagcuga....................   2      1    Le1
......................................................................ugagaucauCgugaaagcuga....................   4      1    Le1
......................................................................ugUgaucauugugaaagcuga....................   3      1    Le1
......................................................................ugagaucaGugugaaagcuga....................   4      1    Le1
......................................................................ugagGucauugugaaagcuga....................   228    1    Le1
......................................................................uAagaucauugugaaagcuga....................   5      1    Le1
......................................................................ugagaucauuUugaaagcuga....................   4      1    Le1
......................................................................Ggagaucauugugaaagcuga....................   5      1    Le1
......................................................................ugagaucauGgugaaagcuga....................   4      1    Le1
......................................................................ugagaGcauugugaaagcuga....................   11     1    Le1
......................................................................ugagaucauugugaUagcuga....................   3      1    Le1
......................................................................ugagaucauugugaaagcuga....................   18251  0    Le1
......................................................................ugagaucauugugaaagcugU....................   290    1    Le1
......................................................................ugagaucauugugaaGgcuga....................   2      1    Le1
......................................................................ugagaucauugugaaagcuAa....................   4      1    Le1
......................................................................ugagaucauugugaaagcuUa....................   35     1    Le1
......................................................................ugagUucauugugaaagcuga....................   311    1    Le1
......................................................................ugagaucCuugugaaagcuga....................   2      1    Le1
......................................................................ugagauGauugugaaagcuga....................   1      1    Le1
......................................................................ugagaucauugugGaagcuga....................   5      1    Le1
......................................................................ugagaucaAugugaaagcuga....................   25     1    Le1
......................................................................ugagaucauAgugaaagcuga....................   2      1    Le1
......................................................................ugCgaucauugugaaagcuga....................   82     1    Le1
......................................................................ugagaucauugugaaagcugG....................   1      1    Le1
......................................................................ugagaucauuguAaaagcuga....................   4      1    Le1
......................................................................ugagaucauugugaCagcuga....................   1      1    Le1
......................................................................ugagaucauuAugaaagcuga....................   14     1    Le1
......................................................................ugGgaucauugugaaagcuga....................   6      1    Le1
......................................................................ugagaucauugugaaagcCga....................   7      1    Le1
......................................................................ugagaucauugugaaUgcuga....................   15     1    Le1
......................................................................ugagaucauugugaaagcugC....................   41     1    Le1
......................................................................ugagaucUuugugaaagcuga....................   38     1    Le1
......................................................................ugagaucauuUugaaagcugau...................   2      1    Le1
......................................................................ugagaucauugCgaaagcugau...................   3      1    Le1
......................................................................ugagaucauugugaaagcugaA...................   309    1    Le1
......................................................................ugagaucauugugaaagcAgau...................   2      1    Le1
......................................................................ugagaucauugGgaaagcugau...................   2      1    Le1
......................................................................ugagaucauugugaaagcugGu...................   8      1    Le1
......................................................................ugagaucauugugaCagcugau...................   3      1    Le1
......................................................................ugagGucauugugaaagcugau...................   214    1    Le1
......................................................................ugagaucauugugaaagcuUau...................   40     1    Le1
......................................................................ugagaucauuAugaaagcugau...................   6      1    Le1
......................................................................ugagaucauugugaaaUcugau...................   1      1    Le1
......................................................................ugagaucauugugUaagcugau...................   2      1    Le1
......................................................................ugagaucauuCugaaagcugau...................   1      1    Le1
```

```
                                Star                                  Mature
ggaacucgaaagcgcccuagcuguagacaaaacugguuuucacaaugauuugacagauguguucgauaucugagaucauugugaaagcugauuuguuguccccaucaucu
......................................................................ugagaucauugugaaagUugau..................  10     1  Le1
......................................................................ugagaucauugugaaagcugaC..................  455    1  Le1
......................................................................ugagaucauugugaaagcuCau..................  1      1  Le1
......................................................................ugagaucaGugugaaagcugau..................  1      1  Le1
......................................................................ugagaucauugugaaUgcugau..................  14     1  Le1
......................................................................ugGgaucauugugaaagcugau..................  5      1  Le1
......................................................................ugagauGauugugaaagcugau..................  1      1  Le1
......................................................................ugagaucauugugGaagcugau..................  4      1  Le1
......................................................................ugagaucauuguUaaagcugau..................  2      1  Le1
......................................................................ugagauAauugugaaagcugau..................  1      1  Le1
......................................................................ugagaucauugugaaCgcugau..................  3      1  Le1
......................................................................ugagaucauugugaaaAcugau..................  5      1  Le1
......................................................................ugagaucGuugugaaagcugau..................  4      1  Le1
......................................................................ugagaucauuguCaaagcugau..................  3      1  Le1
......................................................................ugagaucauugugaaagGugau..................  1      1  Le1
......................................................................Agagaucauugugaaagcugau..................  33     1  Le1
......................................................................ugagaucauuguAaaagcugau..................  11     1  Le1
......................................................................ugagaucUuugugaaagcugau..................  42     1  Le1
......................................................................ugagaucauCgugaaagcugau..................  3      1  Le1
......................................................................uCagaucauugugaaagcugau..................  4      1  Le1
......................................................................ugagaucauugugaUagcugau..................  3      1  Le1
......................................................................ugagaucauugugaaagcugau..................  18495  0  Le1
......................................................................ugagaucauugugaaagcGgau..................  5      1  Le1
......................................................................ugagUucauugugaaagcugau..................  313    1  Le1
......................................................................ugagaucauugugCaagcugau..................  59     1  Le1
......................................................................ugagaucCuugugaaagcugau..................  1      1  Le1
......................................................................ugaUaucauugugaaagcugau..................  33     1  Le1
......................................................................ugagaucauugugaaagcugCu..................  18     1  Le1
......................................................................Ngagaucauugugaaagcugau..................  16     1  Le1
......................................................................ugagaucauugugaaagcuAau..................  4      1  Le1
......................................................................ugagaucauugugaaGgcugau..................  2      1  Le1
......................................................................ugagaCcauugugaaagcugau..................  2      1  Le1
......................................................................ugagaucauugugaGagcugau..................  4      1  Le1
......................................................................ugUgaucauugugaaagcugau..................  5      1  Le1
......................................................................ugagCucauugugaaagcugau..................  4      1  Le1
......................................................................ugaAaucauugugaaagcugau..................  1      1  Le1
......................................................................ugagaucauugugaaagcugaG..................  12     1  Le1
......................................................................ugagaucauugugaaaCcugau..................  2      1  Le1
......................................................................ugagauUauugugaaagcugau..................  5      1  Le1
......................................................................uAagaucauugugaaagcugau..................  7      1  Le1
......................................................................ugagaucauugAgaaagcugau..................  4      1  Le1
......................................................................Ggagaucauugugaaagcugau..................  2      1  Le1
......................................................................ugagaucaAugugaaagcugau..................  24     1  Le1
......................................................................ugagaucauugugaaagcCgau..................  4      1  Le1
......................................................................uUagaucauugugaaagcugau..................  4      1  Le1
......................................................................ugagaucauugugaaagcugUu..................  96     1  Le1
......................................................................Cgagaucauugugaaagcugau..................  24     1  Le1
......................................................................ugCgaucauugugaaagcugau..................  54     1  Le1
......................................................................ugagaAcauugugaaagcugau..................  2      1  Le1
......................................................................ugagaGcauugugaaagcugau..................  4      1  Le1
......................................................................ugagaucauAgugaaagcugau..................  4      1  Le1
......................................................................ugagaucauugGgaaagcugauu.................  1      1  Le1
......................................................................ugagaucauugugaaagcugaGu.................  2      1  Le1
......................................................................uAagaucauugugaaagcugauu.................  3      1  Le1
......................................................................ugagaucauugugaaagcugauu.................  5911   0  Le1
......................................................................ugagaucauugugaaagcuUauu.................  16     1  Le1
......................................................................Agagaucauugugaaagcugauu.................  10     1  Le1
......................................................................ugagaucauugugaaagcuCauu.................  2      1  Le1
......................................................................ugagaucauugugaaagcugUuu.................  3      1  Le1
......................................................................ugagaGcauugugaaagcugauu.................  3      1  Le1
......................................................................ugagaucauugugaaagcugGuu.................  2      1  Le1
......................................................................ugagaucauugugCaagcugauu.................  23     1  Le1
......................................................................ugagaucUuugugaaagcugauu.................  25     1  Le1
......................................................................ugagaucauugugaGagcugauu.................  2      1  Le1
......................................................................ugagaAcauugugaaagcugauu.................  1      1  Le1
......................................................................ugagaucauugugaaagcugauG.................  65     1  Le1
......................................................................ugagaucauugugaaUgcugauu.................  5      1  Le1
......................................................................ugagaucauugugaaagcugCuu.................  7      1  Le1
......................................................................ugagaucGuugugaaagcugauu.................  3      1  Le1
......................................................................ugagauGauugugaaagcugauu.................  1      1  Le1
```

```
                                 Star                                Mature
ggaacucgaaagcgcccuagcuguagacaaaacugguuuucacaaugauuugacagauguguucgauaucugagaucauugugaaagcugauuuuguugucccaucaucu
......................................................................ugagaucauugugaaagcugauC.................    36    1    Le1
......................................................................ugagaucauugCgaaagcugauu.................    3     1    Le1
......................................................................ugUgaucauugugaaagcugauu.................    2     1    Le1
......................................................................ugCgaucauugugaaagcugauu.................    14    1    Le1
......................................................................Ngagaucauugugaaagcugauu.................    3     1    Le1
......................................................................ugagaucauugugaaagcCgauu.................    2     1    Le1
......................................................................ugaUaucauugugaaagcugauu.................    8     1    Le1
......................................................................ugagaucaAugugaaagcugauu.................    4     1    Le1
......................................................................ugagGucauugugaaagcugauu.................    85    1    Le1
......................................................................ugagaucauugugaCagcugauu.................    1     1    Le1
......................................................................ugaAaucauugugaaagcugauu.................    1     1    Le1
......................................................................Cgagaucauugugaaagcugauu.................    7     1    Le1
......................................................................uCagaucauugugaaagcugauu.................    1     1    Le1
......................................................................ugagauUauugugaaagcugauu.................    11    1    Le1
......................................................................ugagaucauugugaaagcugauA.................    448   1    Le1
......................................................................ugagaucauugugaaagcugaAu.................    9     1    Le1
......................................................................ugagaucCuugugaaagcugauu.................    2     1    Le1
......................................................................ugagUucauugugaaagcugauu.................    119   1    Le1
......................................................................ugagaucauAgugaaagcugauu.................    2     1    Le1
......................................................................ugagCucauugugaaagcugauu.................    2     1    Le1
......................................................................ugagauAauugugaaagcugauu.................    1     1    Le1
......................................................................ugagaucauugugaUagcugauu.................    1     1    Le1
......................................................................ugagaucauuguUaaagcugauu.................    3     1    Le1
......................................................................ugagaucauugugaaaAcugauu.................    1     1    Le1
......................................................................ugagaucauugugaaaUcugauu.................    2     1    Le1
......................................................................ugagaucauugAgaaagcugauu.................    1     1    Le1
......................................................................ugagaucauCgugaaagcugauu.................    3     1    Le1
......................................................................ugagaucauugugaaagUugauu.................    2     1    Le1
......................................................................ugagaucauugugaaagcGgauu.................    2     1    Le1
......................................................................ugagaucauuAugaaagcugauu.................    1     1    Le1
......................................................................ugagUucauugugaaagcugauuu................    6     1    Le1
......................................................................ugagaucauugugaaagcugauuu................    201   0    Le1
......................................................................ugCgaucauugugaaagcugauuu................    1     1    Le1
......................................................................ugagaucauugugaaagcuUauuu................    1     1    Le1
......................................................................ugagaucauugugaaagcugCuuu................    1     1    Le1
......................................................................ugaUaucauugugaaagcugauuu................    1     1    Le1
......................................................................ugagaucauugugaaagcugauuA................    184   1    Le1
......................................................................ugagGucauugugaaagcugauuu................    4     1    Le1
......................................................................ugaCaucauugugaaagcugauuu................    1     1    Le1
......................................................................ugagaucauugugaaagcugauuC................    2     1    Le1
......................................................................ugagaucauugugCaagcugauuu................    3     1    Le1
......................................................................ugagauUauugugaaagcugauuu................    1     1    Le1
......................................................................ugagaucauugugaaagcugauuGu...............    1     1    Le1
.......................................................................gagaucauugugaaagcC.....................    6     1    Le1
.......................................................................gagaucauugugaaagcu.....................    27    0    Le1
.......................................................................gagaCcauugugaaagcu.....................    1     1    Le1
.......................................................................gagUucauugugaaagcu.....................    1     1    Le1
.......................................................................gagaucauugugaaagcA.....................    6     1    Le1
.......................................................................gagaucauuguAaaagcu.....................    1     1    Le1
.......................................................................Aagaucauugugaaagcu.....................    1     1    Le1
.......................................................................gagaucauCgugaaagcug....................    3     1    Le1
.......................................................................gagaCcauugugaaagcug....................    2     1    Le1
.......................................................................gagaucauugugaaagcug....................    181   0    Le1
.......................................................................gagaGcauugugaaagcug....................    18    1    Le1
.......................................................................gagaucauugugCaagcug....................    1     1    Le1
.......................................................................gCgaucauugugaaagcug....................    2     1    Le1
.......................................................................gagaucauuguAaaagcug....................    1     1    Le1
.......................................................................gagUucauugugaaagcug....................    5     1    Le1
.......................................................................gagaucaGugugaaagcug....................    1     1    Le1
.......................................................................Uagaucauugugaaagcug....................    2     1    Le1
.......................................................................gagaucauugugaUagcug....................    1     1    Le1
.......................................................................gagaucauugugaCagcug....................    2     1    Le1
.......................................................................Cagaucauugugaaagcug....................    2     1    Le1
.......................................................................gagaucauugugaaagcCg....................    2     1    Le1
.......................................................................gagaucauAgugaaagcug....................    4     1    Le1
.......................................................................gaCaucauugugaaagcug....................    6     1    Le1
.......................................................................gagaAcauugugaaagcug....................    2     1    Le1
.......................................................................gagaucauugugaaagcuU....................    1     1    Le1
.......................................................................gagaucauugugaaagcuga...................    60    0    Le1
.......................................................................gagaAcauugugaaagcuga...................    1     1    Le1
```

Star **Mature**

ggaacucgaaagcgcccuagcuguagacaaaacugguuuucacaaugauuugacagauguguucgauaucugagaucauugugaaagcugauuuuguuguccccaucaucu			
....gagaucauugugaaagUuga....................	1	1	Le1
....gagaucauugugaaGgcuga....................	1	1	Le1
....gagaucauuguAaaagcuga....................	1	1	Le1
....gagaucaAugugaaagcuga....................	1	1	Le1
....gagaCcauugugaaagcuga....................	5	1	Le1
....gagaucauCgugaaagcuga....................	2	1	Le1
....gagaucauuguUaaagcuga....................	1	1	Le1
....gagaucaGugugaaagcuga....................	1	1	Le1
....gagUucauugugaaagcuga....................	4	1	Le1
....gagaGcauugugaaagcuga....................	8	1	Le1
....gagaucauugugaaagcuUa....................	1	1	Le1
....gaCaucauugugaaagcuga....................	1	1	Le1
....gagauUauugugaaagcugau...................	1	1	Le1
....gagaucauugugaaagAugau...................	1	1	Le1
....gagaucauuguUaaagcugau...................	1	1	Le1
....gagaucauugugaaagcugaC...................	1	1	Le1
....gagUucauugugaaagcugau...................	5	1	Le1
....gagaucauAgugaaagcugau...................	1	1	Le1
....gagaucauugAgaaagcugau...................	1	1	Le1
....Aagaucauugugaaagcugau...................	2	1	Le1
....gCgaucauugugaaagcugau...................	1	1	Le1
....gagaucauugugaaaUcugau...................	2	1	Le1
....gagaucauugugaaagcugaA...................	3	1	Le1
....gaCaucauugugaaagcugau...................	1	1	Le1
....gagaGcauugugaaagcugau...................	11	1	Le1
....gagaucauugugaaagcugau...................	77	0	Le1
....gagaAcauugugaaagcugau...................	4	1	Le1
....gagauUauugugaaagcugauu..................	1	1	Le1
....gaCaucauugugaaagcugauu..................	1	1	Le1
....gagaGcauugugaaagcugauu..................	1	1	Le1
....gagaucauugugaaagcugauu..................	30	0	Le1
....gagUucauugugaaagcugauu..................	1	1	Le1
....gagaucauuguCaaagcugauu..................	1	1	Le1
....gagaucauugugaaagcugauA..................	1	1	Le1
....gagaucauugugaaagcugauuA.................	1	1	Le1
....agaucauugugaaagcug.....................	1	0	Le1
....agCucauugugaaagcug.....................	1	1	Le1
....agaucauugugaaagcuga....................	5	0	Le1
....agaucauugugaaagcugau...................	2	0	Le1
....agaucauugugaaagcugaA...................	1	1	Le1
....ucauugugaaagcugauu..................	3	0	Le1
................................cugguuuucacaaugauuug...	1	0	Br1
................................cugguuuucacaaugauuuga..	10	0	Br1
................................Uugguuuucacaaugauuuga..	1	1	Br1
................................cuggGuuucacaaugauuuga..	1	1	Br1
................................cugguuuucacaaugauuugac...	3	0	Br1
................................cugguuuucacaaugauuugaca..	1	0	Br1
.................................ugguuuucacaaugauuuga..	1	0	Br1
....................................uuuucacaaugauuugacagaugugC...	2	1	Br1
....uaucAgagaucauuguga...........................	1	1	Br1
....uaucGgagaucauugugaaa.........................	1	1	Br1
....uaucugagaucauugugaaa.........................	1	0	Br1
....uaucugagaucauugugaaag........................	1	0	Br1
....uaucugagaucauugugaaagc.......................	13	0	Br1
....uaucugagaucauugugaaagcu......................	5	0	Br1
....uaucAgagaucauugugaaagcu......................	1	1	Br1
....uaucugagaucauugugaaagcA......................	3	1	Br1
....aucugagaucauugugaaagc.......................	1	0	Br1
....aucugagaucauugugaaagcu......................	3	0	Br1
....ucugagaucauugugaaagcuU.....................	1	1	Br1
....cugagaucauugugaaagcug.....................	2	0	Br1
....cugagaucauugugaaagcuga....................	2	0	Br1
....cugaUaucauugugaaagcugau...................	1	1	Br1
....cugagaucauugugaaagcugau...................	8	0	Br1
....cugagaucauugugaaagcugUuu..................	1	1	Br1
....cugagaucauugugaaagcugauu..................	3	0	Br1
....cugagaucauugugaaagcugauA..................	1	1	Br1
....ugagaucauugugaaagc.......................	16	0	Br1
....ugagUucauugugaaagcu......................	38	1	Br1

```
                                 Star                               Mature
ggaacucgaaagcgcccuagcuguagacaaaacugguuuucacaaugauuugacagauguguucgauaucugagaucauugugaaagcugauuuuguuguccccaucaucu
......................................................................ugagaGcauugugaaagcu......................  1     1  Br1
......................................................................ugagaucauugugCaagcu......................  12    1  Br1
......................................................................Agagaucauugugaaagcu......................  1     1  Br1
......................................................................ugagaucauugugaaagcA......................  168   1  Br1
......................................................................uAagaucauugugaaagcu......................  2     1  Br1
......................................................................ugagaucUuugugaaagcu......................  2     1  Br1
......................................................................ugagaucaAugugaaagcu......................  3     1  Br1
......................................................................ugCgaucauugugaaagcu......................  5     1  Br1
......................................................................ugagauUauugugaaagcu......................  1     1  Br1
......................................................................ugagaucauugugaaUgcu......................  2     1  Br1
......................................................................ugagGucauugugaaagcu......................  23    1  Br1
......................................................................ugagaucauugugaaGgcu......................  1     1  Br1
......................................................................uCagaucauugugaaagcu......................  2     1  Br1
......................................................................ugagaucauugugaaagcC......................  344   1  Br1
......................................................................ugagaucauugugaaagcG......................  11    1  Br1
......................................................................ugagaucauugugaaagcu......................  1567  0  Br1
......................................................................ugagaucauugCgaaagcu......................  1     1  Br1
......................................................................Ggagaucauugugaaagcu......................  1     1  Br1
......................................................................ugaUaucauugugaaagcu......................  6     1  Br1
......................................................................ugagaucauuAugaaagcu......................  1     1  Br1
......................................................................Cgagaucauugugaaagcu......................  1     1  Br1
......................................................................ugagCucauugugaaagcug.....................  1     1  Br1
......................................................................ugagaucauugugaaagcuC.....................  6     1  Br1
......................................................................ugagaucauuguUaaagcug.....................  1     1  Br1
......................................................................ugagaucauugugaaagcuU.....................  27    1  Br1
......................................................................ugagaucauugugaUagcug.....................  1     1  Br1
......................................................................ugCgaucauugugaaagcug.....................  19    1  Br1
......................................................................ugagaucauugCgaaagcug.....................  1     1  Br1
......................................................................ugagaucauugugaaagcGg.....................  6     1  Br1
......................................................................ugagaucauugugaaCgcug.....................  2     1  Br1
......................................................................uAagaucauugugaaagcug.....................  7     1  Br1
......................................................................ugagaucauugugaaagcuA.....................  40    1  Br1
......................................................................ugagaucauugugaaaUcug.....................  1     1  Br1
......................................................................ugagaucauugugaaaAcug.....................  3     1  Br1
......................................................................ugagaucGuugugaaagcug.....................  1     1  Br1
......................................................................ugagaucauugugaCagcug.....................  1     1  Br1
......................................................................ugagaCcauugugaaagcug.....................  2     1  Br1
......................................................................Cgagaucauugugaaagcug.....................  6     1  Br1
......................................................................ugagaucauugugGaagcug.....................  2     1  Br1
......................................................................ugagaucauugugaaUgcug.....................  6     1  Br1
......................................................................ugagaucaAugugaaagcug.....................  15    1  Br1
......................................................................ugagaucauAgugaaagcug.....................  2     1  Br1
......................................................................uCagaucauugugaaagcug.....................  2     1  Br1
......................................................................uUagaucauugugaaagcug.....................  3     1  Br1
......................................................................ugUgaucauugugaaagcug.....................  1     1  Br1
......................................................................ugagaGcauugugaaagcug.....................  1     1  Br1
......................................................................ugagauUauugugaaagcug.....................  3     1  Br1
......................................................................ugagaAcauugugaaagcug.....................  1     1  Br1
......................................................................Ngagaucauugugaaagcug.....................  7     1  Br1
......................................................................ugagaucauuUugaaagcug.....................  1     1  Br1
......................................................................ugagaucauuAugaaagcug.....................  3     1  Br1
......................................................................ugagaucauugugCaagcug.....................  30    1  Br1
......................................................................ugagaucauugugaaagcug.....................  7337  0  Br1
......................................................................ugagaucauugugaGagcug.....................  1     1  Br1
......................................................................ugaUaucauugugaaagcug.....................  22    1  Br1
......................................................................ugagaucauugugUaagcug.....................  1     1  Br1
......................................................................ugagaucauugGgaaagcug.....................  2     1  Br1
......................................................................ugagGucauugugaaagcug.....................  99    1  Br1
......................................................................ugagaucauCgugaaagcug.....................  1     1  Br1
......................................................................ugagaucauuguAaaagcug.....................  1     1  Br1
......................................................................ugagaucauugugaaGgcug.....................  3     1  Br1
......................................................................ugagaucauugugaaagcAg.....................  6     1  Br1
......................................................................ugagauGauugugaaagcug.....................  2     1  Br1
......................................................................ugGgaucauugugaaagcug.....................  1     1  Br1
......................................................................ugagUucauugugaaagcug.....................  131   1  Br1
......................................................................ugagaucauugugaaagUug.....................  5     1  Br1
......................................................................ugagaucUuugugaaagcug.....................  15    1  Br1
......................................................................ugagaucauugugaaagcCg.....................  38    1  Br1
......................................................................Agagaucauugugaaagcug.....................  10    1  Br1
......................................................................ugagaucauugugaGagcuga....................  2     1  Br1
```

```
                                 Star                                 Mature
ggaacucgaaagcgcccuagcuguagacaaaacugguuuucacaaugauuugacagauguguucgauaucugagaucauugugaaagcugauuuuguuguccccaucaucu
......................................................................ugagaucauugugaaagcuCa...................   3     1   Br1
......................................................................ugagaucauCgugaaagcuga...................   5     1   Br1
......................................................................Ggagaucauugugaaagcuga...................   2     1   Br1
......................................................................ugCgaucauugugaaagcuga...................   28    1   Br1
......................................................................ugagUucauugugaaagcuga...................   126   1   Br1
......................................................................ugagCucauugugaaagcuga...................   2     1   Br1
......................................................................ugagaucauugugaaagcuga...................   7028  0   Br1
......................................................................ugagaucauuguAaaagcuga...................   3     1   Br1
......................................................................ugagaucauugGgaaagcuga...................   1     1   Br1
......................................................................ugagaucauugugaaagcugC...................   30    1   Br1
......................................................................ugagaucauugCgaaagcuga...................   2     1   Br1
......................................................................Ngagaucauugugaaagcuga...................   2     1   Br1
......................................................................ugagaucauugugaaagcugG...................   4     1   Br1
......................................................................ugagaucCuugugaaagcuga...................   1     1   Br1
......................................................................ugagaucauugugaaagcAga...................   1     1   Br1
......................................................................ugagaucauuguCaaagcuga...................   1     1   Br1
......................................................................uCagaucauugugaaagcuga...................   5     1   Br1
......................................................................ugagaucauugugaaagcuUa...................   17    1   Br1
......................................................................ugagaucauAgugaaagcuga...................   2     1   Br1
......................................................................Agagaucauugugaaagcuga...................   14    1   Br1
......................................................................ugagaucauugugaaaUcuga...................   1     1   Br1
......................................................................ugagaucauugugaaGgcuga...................   1     1   Br1
......................................................................ugGgaucauugugaaagcuga...................   1     1   Br1
......................................................................ugagaucauugugCaagcuga...................   22    1   Br1
......................................................................ugagaucauuCugaaagcuga...................   1     1   Br1
......................................................................ugagaucaAugugaaagcuga...................   12    1   Br1
......................................................................ugagaucauugugaaagUuga...................   6     1   Br1
......................................................................ugagaucGuugugaaagcuga...................   1     1   Br1
......................................................................ugagaucUuugugaaagcuga...................   17    1   Br1
......................................................................ugagaCcauugugaaagcuga...................   2     1   Br1
......................................................................ugagaucauuguUaaagcuga...................   2     1   Br1
......................................................................ugagaucauugugaaagcCga...................   1     1   Br1
......................................................................ugagGucauugugaaagcuga...................   90    1   Br1
......................................................................ugagauGauugugaaagcuga...................   1     1   Br1
......................................................................ugagaGcauugugaaagcuga...................   6     1   Br1
......................................................................ugagaucauugugaaagcuAa...................   3     1   Br1
......................................................................Cgagaucauugugaaagcuga...................   13    1   Br1
......................................................................ugagaucauugugaaagcGga...................   5     1   Br1
......................................................................ugaUaucauugugaaagcuga...................   6     1   Br1
......................................................................ugaCaucauugugaaagcuga...................   1     1   Br1
......................................................................ugagaucauugugaaUgcuga...................   6     1   Br1
......................................................................ugagaucauugugaaagcugU...................   65    1   Br1
......................................................................ugagauUauugugaaagcuga...................   1     1   Br1
......................................................................ugagaucauugugUaagcuga...................   2     1   Br1
......................................................................ugagaAcauugugaaagcuga...................   1     1   Br1
......................................................................ugUgaucauugugaaagcuga...................   4     1   Br1
......................................................................ugagaucauugugaaaAcuga...................   5     1   Br1
......................................................................ugagaucauugugaaagUugau..................   9     1   Br1
......................................................................ugagaucauugugaUagcugau..................   4     1   Br1
......................................................................ugagaucauugugaaagcCgau..................   2     1   Br1
......................................................................ugCgaucauugugaaagcugau..................   36    1   Br1
......................................................................ugagaucCuugugaaagcugau..................   1     1   Br1
......................................................................ugagaucauAgugaaagcugau..................   3     1   Br1
......................................................................ugagaucauugugaaaUcugau..................   1     1   Br1
......................................................................ugagaucauCgugaaagcugau..................   2     1   Br1
......................................................................ugagaucUuugugaaagcugau..................   35    1   Br1
......................................................................ugagUucauugugaaagcugau..................   235   1   Br1
......................................................................uUagaucauugugaaagcugau..................   2     1   Br1
......................................................................ugagaucauugugaaCgcugau..................   1     1   Br1
......................................................................ugagaucauugugaaagcGgau..................   3     1   Br1
......................................................................ugagaucauugugUaagcugau..................   2     1   Br1
......................................................................ugagaucauugugaGagcugau..................   4     1   Br1
......................................................................ugagaucauugugaaaCcugau..................   1     1   Br1
......................................................................ugagaucaAugugaaagcugau..................   23    1   Br1
......................................................................ugagaucauugugaaaAcugau..................   8     1   Br1
......................................................................ugaUaucauugugaaagcugau..................   19    1   Br1
......................................................................Ngagaucauugugaaagcugau..................   8     1   Br1
......................................................................ugagaucauugugaaagcugaA..................   132   1   Br1
......................................................................ugagaucauugugaaagcugCu..................   9     1   Br1
......................................................................ugagaucauuguUaaagcugau..................   1     1   Br1
```

Star **Mature**

```
ggaacucgaaagcgcccuagcuguagacaaaacugguuuucacaaugauuugacagauguguucgauaucugagaucauugugaaagcugauuuuguuguccccaucaucu
......................................................................ugagaucauugugaaagcuUau...................  21     1  Br1
......................................................................ugGgaucauugugaaagcugau...................  2      1  Br1
......................................................................ugagauUauugugaaagcugau...................  4      1  Br1
......................................................................ugagaucauugugaaagcugaG...................  9      1  Br1
......................................................................ugagaucauuUugaaagcugau...................  1      1  Br1
......................................................................ugagauGauugugaaagcugau...................  3      1  Br1
......................................................................ugagaucauugugaaUgcugau...................  7      1  Br1
......................................................................ugagaucauugugaaagcugGu...................  1      1  Br1
......................................................................ugagCucauugugaaagcugau...................  1      1  Br1
......................................................................ugagaucauugugaaagcugaC...................  410    1  Br1
......................................................................uAagaucauugugaaagcugau...................  6      1  Br1
......................................................................ugUgaucauugugaaagcugau...................  1      1  Br1
......................................................................Cgagaucauugugaaagcugau...................  22     1  Br1
......................................................................ugagaucauugugaaagAugau...................  2      1  Br1
......................................................................ugagaucGuugugaaagcugau...................  1      1  Br1
......................................................................ugagaucauugugaaagcuAau...................  2      1  Br1
......................................................................ugagaucauuAugaaagcugau...................  5      1  Br1
......................................................................uCagaucauugugaaagcugau...................  4      1  Br1
......................................................................ugagauAauugugaaagcugau...................  1      1  Br1
......................................................................ugagaucauugugaaagcugUu...................  29     1  Br1
......................................................................ugagaucauugugCaagcugau...................  40     1  Br1
......................................................................ugagaGcauugugaaagcugau...................  1      1  Br1
......................................................................Agagaucauugugaaagcugau...................  25     1  Br1
......................................................................ugagaucauugugaaGgcugau...................  4      1  Br1
......................................................................ugagaucauugugaaagcugau...................  13123  0  Br1
......................................................................ugagaucauugAgaaagcugau...................  2      1  Br1
......................................................................ugagGucauugugaaagcugau...................  170    1  Br1
......................................................................ugagaucauugCgaaagcugau...................  2      1  Br1
......................................................................Ggagaucauugugaaagcugau...................  3      1  Br1
......................................................................ugagaucauugugaaagUugauu..................  8      1  Br1
......................................................................ugagaucauugugaaaUcugauu..................  2      1  Br1
......................................................................ugagUucauugugaaagcugauu..................  147    1  Br1
......................................................................ugagaucauugugaaaAcugauu..................  2      1  Br1
......................................................................Ggagaucauugugaaagcugauu..................  1      1  Br1
......................................................................ugagaucauugugaaGgcugauu..................  1      1  Br1
......................................................................Cgagaucauugugaaagcugauu..................  12     1  Br1
......................................................................ugagaGcauugugaaagcugauu..................  3      1  Br1
......................................................................ugagaucauugugUaagcugauu..................  2      1  Br1
......................................................................ugagaucauugugaaagcAgauu..................  1      1  Br1
......................................................................ugagaucCuugugaaagcugauu..................  1      1  Br1
......................................................................ugagaucauugugaaagcugaGu..................  1      1  Br1
......................................................................ugagaucauugugaaCgcugauu..................  1      1  Br1
......................................................................ugagaucauugugaaagcugauG..................  41     1  Br1
......................................................................ugagauUauugugaaagcugauu..................  6      1  Br1
......................................................................ugagaucauugGgaaagcugauu..................  2      1  Br1
......................................................................uAagaucauugugaaagcugauu..................  5      1  Br1
......................................................................ugagaucauugugaaUgcugauu..................  5      1  Br1
......................................................................ugagaucauugugaaagcugauu..................  8662   0  Br1
......................................................................ugCgaucauugugaaagcugauu..................  24     1  Br1
......................................................................ugagaucauugugaaagcugCuu..................  5      1  Br1
......................................................................Ngagaucauugugaaagcugauu..................  7      1  Br1
......................................................................ugaUaucauugugaaagcugauu..................  15     1  Br1
......................................................................ugagaucaAugugaaagcugauu..................  16     1  Br1
......................................................................ugagaucauuAugaaagcugauu..................  3      1  Br1
......................................................................Agagaucauugugaaagcugauu..................  20     1  Br1
......................................................................ugagaucauugugaaagcugauA..................  526    1  Br1
......................................................................ugagaucauugugaaagcugUuu..................  1      1  Br1
......................................................................ugagaucauugugGaagcugauu..................  3      1  Br1
......................................................................ugagaucauugugaGagcugauu..................  3      1  Br1
......................................................................ugagGucauugugaaagcugauu..................  118    1  Br1
......................................................................ugagaucauugAgaaagcugauu..................  1      1  Br1
......................................................................ugagaucUuugugaaagcugauu..................  24     1  Br1
......................................................................ugagaucauugCgaaagcugauu..................  1      1  Br1
......................................................................ugagaucauugugaaagcuAauu..................  1      1  Br1
......................................................................ugagaucauuguAaaagcugauu..................  2      1  Br1
......................................................................ugagaucauugugCaagcugauu..................  31     1  Br1
......................................................................ugagaucauugugaaaCcugauu..................  1      1  Br1
......................................................................ugagaucauugugaaagcugauC..................  62     1  Br1
......................................................................ugagaucauGgugaaagcugauu..................  1      1  Br1
......................................................................ugagaucauugugaaagcugaAu..................  4      1  Br1
```

```
                                Star                                    Mature
ggaacucgaaagcgcccuagcuguagacaaaacugguuuucacaaugauuugacagauguguucgauaucugagaucauugugaaagcugauuuuguugucccaucaucu
......................................................................ugagaucauugugaaagcuUauu.................  16   1   Br1
......................................................................ugagaucauCgugaaagcugauu.................  2    1   Br1
......................................................................ugagaucGuugugaaagcugauu.................  2    1   Br1
......................................................................ugagaCcauugugaaagcugauu.................  2    1   Br1
......................................................................uCagaucauugugaaagcugauu.................  5    1   Br1
......................................................................ugGgaucauugugaaagcugauu.................  5    1   Br1
......................................................................ugagaucauuguUaaagcugauu.................  2    1   Br1
......................................................................ugagaucauAgugaaagcugauu.................  4    1   Br1
......................................................................ugUgaucauugugaaagcugauu.................  2    1   Br1
......................................................................ugagaucauugugaaagcugaCu.................  3    1   Br1
......................................................................ugagauGauugugaaagcugauu.................  1    1   Br1
......................................................................ugagaucauugugaUagcugauu.................  1    1   Br1
......................................................................ugagaucUuugugaaagcugauuu................  5    1   Br1
......................................................................ugagaucauugugaaagcugauuG................  1    1   Br1
......................................................................ugagaucauugugaaagcugauuu................  503  0   Br1
......................................................................ugagaucauugugaaagcugauGu................  2    1   Br1
......................................................................ugagauUauugugaaagcugauuu................  1    1   Br1
......................................................................ugagaGcauugugaaagcugauuu................  1    1   Br1
......................................................................ugagaucauugugaaagcugCuuu................  2    1   Br1
......................................................................ugagaucauugugaaagcugauuA................  369  1   Br1
......................................................................ugagaucauugugaaagcugauuC................  7    1   Br1
......................................................................ugagaucauugugaaagcugauAu................  2    1   Br1
......................................................................ugCgaucauugugaaagcugauuu................  3    1   Br1
......................................................................ugagaucauugugaaagcuUauuu................  1    1   Br1
......................................................................ugaUaucauugugaaagcugauuu................  1    1   Br1
......................................................................ugagUucauugugaaagcugauuu................  6    1   Br1
......................................................................ugagaucauugugCaagcugauuu................  2    1   Br1
......................................................................ugagGucauugugaaagcugauuu................  5    1   Br1
......................................................................ugagaucauugugaaagcugauuAu...............  2    1   Br1
......................................................................ugagaucauugugaaagcugauuuA...............  1    1   Br1
......................................................................ugagaucauugugaaagcugauuuuU..............  5    1   Br1
.......................................................................gagaGcauugugaaagcu.....................  1    1   Br1
.......................................................................gagaCcauugugaaagcu.....................  1    1   Br1
.......................................................................gagaucauugugaaagcu.....................  2    0   Br1
.......................................................................gagaGcauugugaaagcug....................  1    1   Br1
.......................................................................gagaAcauugugaaagcug....................  2    1   Br1
.......................................................................gagaucauugugaaagcug....................  30   0   Br1
.......................................................................gagaucauAgugaaagcug....................  1    1   Br1
.......................................................................gagaucauuguUaaagcug....................  1    1   Br1
.......................................................................gagaucauugugaaagcCg....................  1    1   Br1
.......................................................................gCgaucauugugaaagcuga...................  2    1   Br1
.......................................................................gagaGcauugugaaagcuga...................  4    1   Br1
.......................................................................gagUucauugugaaagcuga...................  1    1   Br1
.......................................................................gagaucauugugaaagcuga...................  21   0   Br1
.......................................................................gagGucauugugaaagcuga...................  1    1   Br1
.......................................................................gaCaucauugugaaagcugau..................  5    1   Br1
.......................................................................gagaGcauugugaaagcugau..................  2    1   Br1
.......................................................................gagaucauugugaaagcugau..................  74   0   Br1
.......................................................................gagUucauugugaaagcugau..................  1    1   Br1
.......................................................................gagaucauugugaaagcugaA..................  1    1   Br1
.......................................................................gagaucaGugugaaagcugau..................  1    1   Br1
.......................................................................gCgaucauugugaaagcugau..................  3    1   Br1
.......................................................................gagaucauugugaaagcugGu..................  1    1   Br1
.......................................................................gagaucauugugaaagcugaC..................  3    1   Br1
.......................................................................gagaAcauugugaaagcugau..................  2    1   Br1
.......................................................................gagauUauugugaaagcugau..................  1    1   Br1
.......................................................................Aagaucauugugaaagcugau..................  1    1   Br1
.......................................................................gagaCcauugugaaagcugau..................  1    1   Br1
.......................................................................gagaucauugGgaaagcugau..................  1    1   Br1
.......................................................................gagUucauugugaaagcugauu.................  2    1   Br1
.......................................................................gCgaucauugugaaagcugauu.................  2    1   Br1
.......................................................................gagaAcauugugaaagcugauu.................  1    1   Br1
.......................................................................gagaucauAgugaaagcugauu.................  1    1   Br1
.......................................................................gagaCcauugugaaagcugauu.................  1    1   Br1
.......................................................................gaCaucauugugaaagcugauu.................  2    1   Br1
.......................................................................gagaucauugugaaagcugauA.................  2    1   Br1
.......................................................................gagaucauugugaaagcugauu.................  62   0   Br1
.......................................................................gagaGcauugugaaagcugauu.................  6    1   Br1
.......................................................................gagauUauugugaaagcugauu.................  1    1   Br1
.......................................................................gagaucauugugaaagcugauC.................  1    1   Br1
```

```
                           Star                                  Mature
ggaacucgaaagcgcccuagcuguagacaaaacugguuuucacaaugauuugacagauguguucgauaucugagaucauugugaaagcugauuuuguugucccaucaucu
.......................................................................gagaucauugugaaagcugauuA................   2   1   Br1
.......................................................................gagaucauugugaaagcugauuu................   1   0   Br1
.......................................................................gagaucauuguUaaagcugauuu................   1   1   Br1
.......................................................................gaCaucauugugaaagcugauuu................   1   1   Br1
.......................................................................gagaucauugugaaagcuUauuu................   1   1   Br1
........................................................................agaucauugugaaagcugau..................   1   0   Br1
.........................................................................gaucauugugaaagcugUu..................   1   1   Br1
...........................................................................ucauGgugaaagcugauu.................   1   1   Br1
...........................................................................ucauugugaaagcugauu.................   1   0   Br1
...........................................................................ucauugugaaagcugauuA................   1   1   Br1
............................................................................cauugugaaagcugauuu................   1   0   Br1

................................cugguuuucacaaugauuug..........................................................   2   0   Le5
................................cugguuuucUcaaugauuuga.........................................................   1   1   Le5
................................cugUuuuucacaaugauuuga.........................................................   1   1   Le5
................................cuggGuuucacaaugauuuga.........................................................   4   1   Le5
................................cugguuuucacaaugauuuga.........................................................   54  0   Le5
................................cuggAuuucacaaugauuuga.........................................................   3   1   Le5
................................Augguuuucacaaugauuuga.........................................................   1   1   Le5
................................cugguuuucacaaugauuugac........................................................   17  0   Le5
................................cugguuuucacaaugauuugaca.......................................................   13  0   Le5
................................cugguuuucacaaugauuugacU.......................................................   1   1   Le5
.................................ugguCuucacaaugauuuga.........................................................   1   1   Le5
.................................ugguuuucacaaugauuuga.........................................................   1   0   Le5
.................................ugguuuucacaaugauuugaca.......................................................   3   0   Le5
.................................ugguGuucacaaugauuugaca.......................................................   1   1   Le5
....................................uuuucacaaugauuugacagaugu..................................................   1   0   Le5
..................................................................uaucugagaucauugugaaa........................   6   0   Le5
..................................................................uaucugagCucauugugaaag.......................   1   1   Le5
..................................................................uaucugagaucauugugaaag.......................   7   0   Le5
..................................................................uaucGgagaucauugugaaagc......................   9   1   Le5
..................................................................uaucugagaucauugugaaagA......................   1   1   Le5
..................................................................uaucugagaucauugugaaagc......................   50  0   Le5
..................................................................uaucugagaucauugugaaagcA.....................   6   1   Le5
..................................................................uaucAgagaucauugugaaagcu.....................   1   1   Le5
..................................................................uaucugagaucauugugaaagcu.....................   19  0   Le5
..................................................................uaucugagCucauugugaaagcu.....................   1   1   Le5
..................................................................uaucugagaucauugugaaagcC.....................   5   1   Le5
..................................................................uaucugagaucauugugaaagcuU....................   2   1   Le5
..................................................................uaucugagaucauugugaaagcuga...................   1   0   Le5
...................................................................aucugaUaucauugugaaa........................   1   1   Le5
...................................................................aucugagaucauugugaaagc......................   6   0   Le5
...................................................................aucuUagaucauugugaaagcu.....................   2   1   Le5
...................................................................aucugagaucauugugaaagcA.....................   2   1   Le5
...................................................................aucugagaucauugugaaagcC.....................   2   1   Le5
...................................................................aucugagaucauugugaaagcu.....................   38  0   Le5
...................................................................aucugagaucauugugaaagcuC....................   1   1   Le5
...................................................................aucugagaucauugugaaagcug....................   4   0   Le5
....................................................................Ccugagaucauugugaaagcu.....................   1   1   Le5
....................................................................ucugagaucauugugaaagcu.....................   5   0   Le5
....................................................................ucugagaucauugugaaagcC.....................   3   1   Le5
....................................................................ucugagaucauugugaaagcA.....................   1   1   Le5
....................................................................ucugagaucauugugaaagcuA....................   1   1   Le5
....................................................................ucugagaucauugugaaagcug....................   9   0   Le5
....................................................................Ccugagaucauugugaaagcug....................   1   1   Le5
....................................................................ucugagaucauugugaaagcuU....................   4   1   Le5
.....................................................................cugagaucCuugugaaagcu.....................   1   1   Le5
.....................................................................cugagaucauugugaaagcC.....................   2   1   Le5
.....................................................................Augagaucauugugaaagcu.....................   1   1   Le5
.....................................................................Gugagaucauugugaaagcu.....................   1   1   Le5
.....................................................................cugagaucauugugaaagcu.....................   8   0   Le5
.....................................................................cugagaucauugugaaagcuU....................   1   1   Le5
.....................................................................cCgagaucauugugaaagcug....................   1   1   Le5
.....................................................................cugagaucauugugaaagcug....................   30  0   Le5
```
[truncated: 813,297 more chars]
